# Insights into Dynamic Polymicrobial Synergy Revealed by Time-Coursed RNA-Seq

**DOI:** 10.3389/fmicb.2017.00261

**Published:** 2017-02-28

**Authors:** Erik L. Hendrickson, David A. C. Beck, Daniel P. Miller, Qian Wang, Marvin Whiteley, Richard J. Lamont, Murray Hackett

**Affiliations:** ^1^Center for Microbial Proteomics and Chemical Engineering, University of WashingtonSeattle, WA, USA; ^2^eScience Institute, University of WashingtonSeattle, WA, USA; ^3^Department of Oral Immunology and Infectious Diseases, University of Louisville School of DentistryLouisville, KY, USA; ^4^Department of Molecular Biosciences, University of Texas at AustinAustin, TX, USA

**Keywords:** *Porphyromonas gingivalis*, *Streptococcus gordonii*, model community, RNA-Seq, time-course profiling

## Abstract

Many bacterial infections involve polymicrobial communities in which constituent organisms are synergistically pathogenic. Periodontitis, a commonly occurring chronic inflammatory disorder, is induced by multispecies bacterial communities. The periodontal keystone pathogen *Porphyromonas gingivalis* and the accessory pathogen *Streptococcus gordonii* exhibit polymicrobial synergy in animal models of disease. Mechanisms of co-adhesion and community formation by *P. gingivalis* and *S. gordonii* are well-established; however, little is known regarding the basis for increased pathogenicity. In this study we used time-coursed RNA-Seq to comprehensively and quantitatively examine the dynamic transcriptional landscape of *P. gingivalis* in a model consortium with *S. gordonii*. Genes encoding a number of potential virulence determinants had higher relative mRNA levels in the context of dual species model communities than *P. gingivalis* alone, including adhesins, the Type IX secretion apparatus, and tetratricopeptide repeat (TPR) motif proteins. In contrast, genes encoding conjugation systems and many of the stress responses showed lower levels of expression in *P. gingivalis*. A notable exception to reduced abundance of stress response transcripts was the genes encoding components of the oxidative stress-related OxyR regulon, indicating an adaptation of *P. gingivalis* to detoxify peroxide produced by the streptococcus. Collectively, the results are consistent with evolutionary adaptation of *P. gingivalis* to a polymicrobial oral environment, one outcome of which is increased pathogenic potential.

## Introduction

Microbiome studies have enhanced our awareness of the polymicrobial etiology of many infectious diseases. Organisms within polymicrobial communities often exhibit synergistic interactions and such polymicrobial synergy helps define the pathogenic potential, or nososymbiocity, of the entire community (Hajishengallis and Lamont, 2016; Stacy et al., 2016). Periodontal diseases, which are among the most common infectious diseases worldwide (Kassebaum et al., 2014), are an exemplar of polymicrobial infections in which disease is initiated by a complex microbial community residing in the subgingival compartment (Darveau, 2010; Hajishengallis and Lamont, 2012). Pathogenic outcomes in periodontal diseases depend on interbacterial interactions among microbial colonizers that engender a dysbiotic community and destructive inflammatory responses (Hajishengallis and Lamont, 2014; Lamont and Hajishengallis, 2015).

A series of well-defined developmental processes characterize periodontal microbial community formation. Early colonizers of the hard and soft tissues are primarily Gram-positive facultatives such as the oral streptococci, and these organisms adhere to mucosal and saliva-coated surfaces (Rosan and Lamont, 2000; Jenkinson and Lamont, 2005). Primary colonizers in turn provide an attachment substratum, along with physiological and nutritional support, for later colonizers such as the keystone pathogen *Porphyromonas gingivalis* (Pg). The interaction between Pg and *Streptococcus gordonii* (Sg) has been extensively studied, and the organisms are synergistically pathogenic in animal models of periodontal disease (Periasamy and Kolenbrander, 2009; Daep et al., 2011; Wright et al., 2013). Co-adhesion between Pg and Sg is mediated by the FimA and Mfa1 component fimbriae of Pg, which interact with streptococcal surface GAPDH and SspA/B proteins, respectively (Kuboniwa and Lamont, 2010; Wright et al., 2013). Subsequent accumulation of Pg is regulated by a protein tyrosine (de)phosphorylation signal transduction cascade that controls transcription factor activity (Maeda et al., 2008; Wright et al., 2014). The long term (18 h) association between Pg and Sg has been studied on the proteome level by our group and paints a picture consistent with a mutually physiologically supportive environment (Kuboniwa et al., 2009b, 2012; Hendrickson et al., 2012). However, little is known regarding the initial dynamic adaptation of Pg on a global scale to a dual species community and the potential mechanisms of synergistic pathogenicity.

RNA sequencing (RNA-Seq) is a sensitive method for the comprehensive analysis of gene expression, which is now commonly used for the study of microbial pathogenicity and environmental adaptations (Westermann et al., 2012; Creecy and Conway, 2015). Moreover, as computational methods and sequencing technology have improved, RNA-Seq can also be exploited to reveal changes in gene expression over a time course. Herein, we have used RNA-Seq to examine the dynamic transcriptional landscape of Pg in a model community with Sg. The results provide insights into the nature of the physiologic support provide by Sg to Pg, and the basis for pathogenic synergy exhibited by PgSg communities.

## Materials and methods

### Bacteria and culture conditions

*Porphyromonas gingivalis* ATCC 33277 (Pg) was grown anaerobically (85% N2, 10% H2, 5% CO2) at 37°C in trypticase soy broth supplemented with 1 mg/ml yeast extract, 1 μg/ml menadione and 5 μg/ml hemin. *S. gordonii* DL1 (Sg) was grown anaerobically at 37°C in Todd-Hewitt broth. Bacteria were cultured to mid-log phase, harvested by centrifugation and resuspended in pre-reduced PBS, pH 7.2. Model communities were generated by the method described by Merritt et al. (2005). 1 × 10^9^ cells of Pg and Sg were mixed in an equimolar ratio, pelleted and held anaerobically at 37°C for 5, 30, 120, 240, and 360 min. Pg cells alone, pelleted and held over the same time course were used for comparison with the PgSg condition. To obtain a baseline reading, Pg cells were pelleted and then immediately lysed for RNA extraction (see below), this represented the *T* = 1 condition.

### RNA sequencing

RNA was extracted from cells using the Ambion mirVana miRNA Isolation Kit AM1561 (ThermoFisher Scientific, Waltham, MA). Library construction was performed with the Illumina TruSeq Stranded mRNA library prep kit RS-122-2101 (Illumina, San Diego, CA). Fragmented RNA was converted to cDNA, and enriched and purified by PCR to create the library without rRNA depletion. High throughput sequencing was performed on a HiSeq2500-v4 sequencer (Illumina) at the High-Throughput Genomics Center (htSEQ) at the University of Washington Department of Genome Sciences.

### Validation

To corroborate the sequencing results, expression of a subset of genes was determined by qRT-PCR analysis. Total RNA samples were prepared from communities of PgSg and Pg alone at 5 and 360 min under the same conditions as those for RNASeq. RNA was isolated from three independent experiments and converted to cDNA with an iScript cDNA synthesis kit (Bio-Rad, Hercules, CA). qRT-PCR was performed by StepOne plus by the ΔΔCt method using 16S rRNA as an internal control. Three experimental replicates were analyzed for each biological sample. Although the magnitudes of fold differences were different between the two techniques as expected, expression profiles for the genes tested were concordant with respect to direction of change.

### Data processing

The genomes for Pg ATCC 33277 (Naito et al., 2008) and Sg DL1 were retrieved from Genbank (loci AP009380 and NC_009785). The Genbank files were combined and converted to a GFF file suitable for use with *htseq-count* tool from HTSeq (Anders et al., 2015) with BioPerl's (Stajich et al., 2002) *bp_genbank2gff3.pl* tool. The raw reads were aligned to the combined genomes at the same time using BWA (Li et al., 2009; Li and Durbin, 2010) version 0.7.4-r385 using the *aln/samse* mode under default options. The alignments were post-processed, sorted into BAM files, and indexed with SAMTools version 0.1.19-44428cd (Li et al., 2009). Reads per gene was computed from the alignments with *htseq-count* version 0.5.4p5 in the “intersection-nonempty” mode.

### Data analysis for differential expression

Normalized read counts and *p-*values for differential abundance were computed for three biological replicates using DESeq2 (Anders and Huber, 2010). The *p-*values were subsequently corrected for multiple hypothesis testing using the *q-*value method of Storey (Storey and Tibshirani, 2003a,b). Principal components (PCA) of the normalized read abundances were computed with R (R Core Team, 2015) using the top 500 genes with the most expression abundance variance across all samples. Transcripts were considered statistically different if they made the *q*-value cutoff of 0.001 and had an absolute log_2_ fold difference of greater than 0.5.

### Ontology analysis

An ontology analysis was conducted using the DAVID functional annotation clustering feature (Database for Annotation, Visualization and Integrated Discovery, Huang et al., 2007). Lists of transcripts with increased or decreased levels were compared to the list of overall detected transcripts as the background using Entrez gene identifiers to designate the genes. The databases used were left at the default settings. Potentially interesting clusters were then inspected manually.

### Gene expression data

All sequence data for this study have been deposited in GEO (Gene Expression Omnibus). The accession number for the dataset is GSE78126, see http://www.ncbi.nlm.nih.gov/geo/query/acc.cgi?acc=GSE78126.

## Results and discussion

Within the complex multispecies environment of oral microbial communities, *P. gingivalis* (Pg) interfaces with a multitude of bacterial species. Both antagonist and synergistic interactions have been documented (Jenkinson and Lamont, 2005), and elevated pathogenicity in animal models occurs with Pg and a number of partner species (Kesavalu et al., 2007; Metzger et al., 2009; Orth et al., 2011). *In vivo*, Pg can be found in association with streptococci including *S. gordonii* (Sg) (Valm et al., 2011; Griffen et al., 2012), and these two organisms in combination are synergistically pathogenic (Daep et al., 2011).

To investigate the responses of Pg to Sg in a heterotypic community over time, we employed a well-established experimentally tractable model of nascent community interaction (Merritt et al., 2005; Kuboniwa et al., 2009b; Hendrickson et al., 2012, 2014). Model Communities were constructed using Pg alone or an equal combination of Pg and Sg, and total RNA extracted after 1, 5, 30, 120, 240, or 360 min. This combination of time course and experimental model was chosen, based on our previous studies cited above, to cover the interval in which most early stage and (or) pre-programmed interactions take place, while minimizing confounding starvation or growth responses. Obvious starvation responses using Pg with this model, either alone or with Sg, have not been observed prior to 360 min. Transcriptomes were determined for each sample using high throughput RNA sequencing, and between 44 and 57 million mapped reads were obtained from each library. Prior work in our group with a variety of ribosomal RNA depletion schemes suggested that our sampling was deep enough with this model system that we could safely avoid the biases introduced by such depletion schemes, without compromising our ability to detect low abundance transcripts (data not shown). *P. gingivalis* alone (Pg) showed large changes in the transcriptome. Of the 2090 predicted genes in the Pg 33277 genome, 69% were differentially expressed by 360 min. In *P. gingivalis-S. gordonii* communities (PgSg), 48% of genes were significantly altered at the 5 min time point compared to Pg alone, and this increased to 63% by 360 min. The results for all annotated genes at all time points are summarized in Tables S1–S3, ordered by PGN number. Tables S1–S3 are a static PDF version of a comprehensive relational database that contains a number of convenient search functions and adjustable parameters. The file size requirements of the journal required breaking up the main supplement into three parts. Readers desiring access to the full database capability should contact the corresponding author. A key advantage of working with the full database is that the log-fold, *p*- and *q-*value cut-offs are treated as adjustable variables, allowing the user to visualize and mine the dataset under a variety of conditions, and to use alternative statistical models of their own choosing. Table S4 in the supplemental material covers a specific subset of genes discussed in the main text, those annotated to play a role in translation initiation, elongation and termination. Representative qRT-PCR validation data is illustrated in Figure S1.

### Principal components

**Figure 1:**
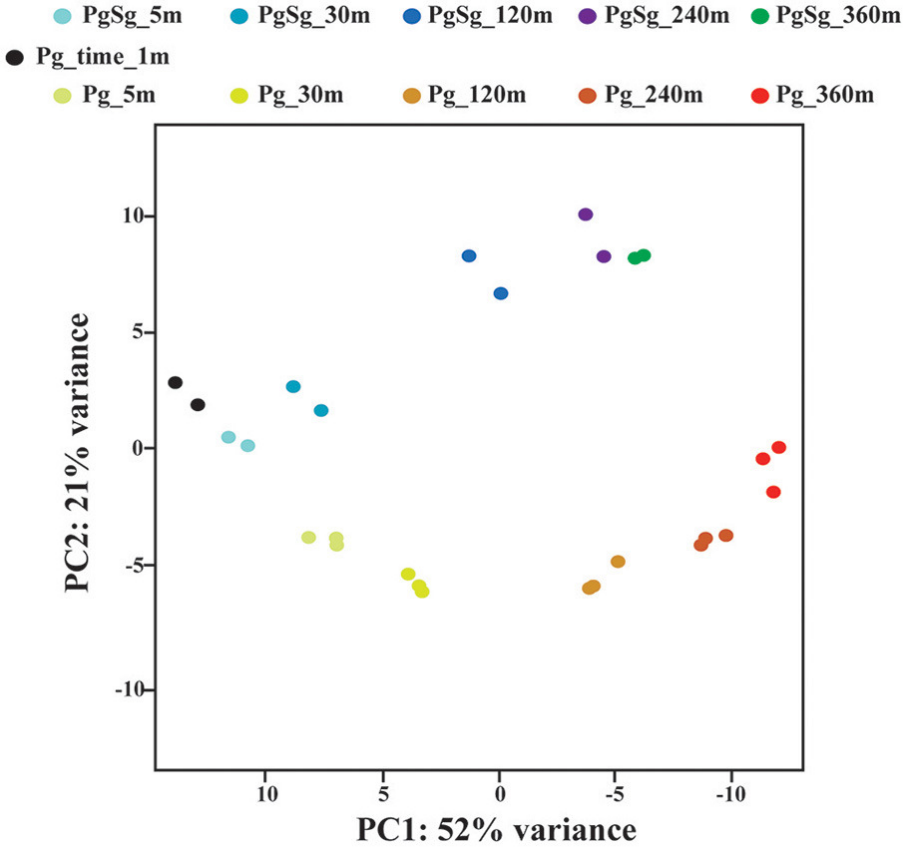
**Principal Component Analysis. The figure shows each biological replicate for each condition on the first two components of the principal component analysis**. The analysis was run using the 500 genes with the largest normalized count variance across all samples and time points (see Methods). The first two components account for 73% of the variance in the data. The first component primarily reflects the influence of time. The second component reflects the presence or absence of *S. gordonii*.

A principal component analysis was run using the 500 genes with the highest variance across the time course samples. Figure 1 shows a plot of the first and second components, which accounted for 73% of the variance. The first component generally followed the time course, consistent with the finding that a large percentage of the Pg transcriptome was differentially increased or decreased over time. The second component largely separated the PgSg samples from Pg alone. Consistent with this, the most significant genes for the second component generally showed increased or decreased transcript levels for PgSg compared to Pg across the time course.

### *P. gingivalis* transcriptional landscape over time in a monospecies community

**Figure 2:**
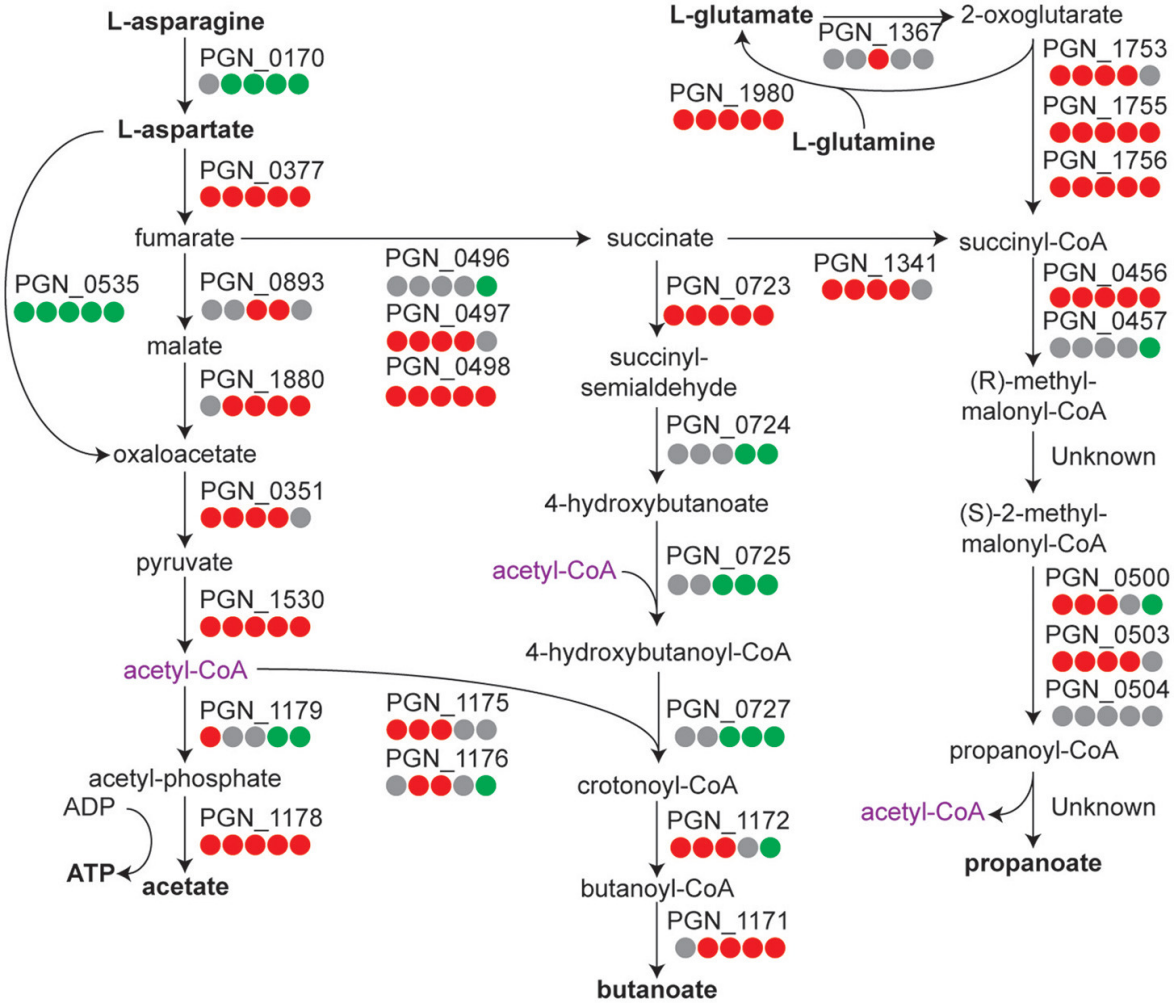
**Metabolic pathway diagram showing amino acid metabolism over the time course for *P. gingivalis* alone**. Each dot represents a comparison of a time point in the course to the *T* = 1 control, reading from left to right starting with 5 min. Red indicates higher levels, green lower levels, and gray no significant change. Full details for each Pg ORF can be found in Tables S1–S3.

In our control condition Pg is maintained as a high density monospecies community in the absence of exogenous nutrients. Pg is an asaccharolytic organism and primarily derives its energy from amino acids, with strain 33277 preferring dipeptides to peptides, and glutamine/glutamate and asparagine/aspartate as amino acids (Takahashi et al., 2000). Examination of the pathways related to metabolism of these amino acids (Figure 2) showed that most of the genes had increased transcript levels, compared to the *T* = 1 condition, albeit the increase tapered off at the 360 min time point. Differential gene expression levels indicated a trend toward increased propanoate and a possible shift of acetyl-CoA from acetate to butanoate. These results suggest that Pg has sufficient energy reserves to continue metabolic activity in buffer at least through 240 min. Similarly, ribosomal protein genes showed increased expression across the time course (see Tables S1–S3 for individual genes annotated as coding for ribosomal proteins). The largest number of genes with increased expression was seen at 120 min and the number was reduced at later time points. The genes for translation initiation, elongation, and termination followed a similar pattern (Table S4). Hence, Pg appears capable of maintaining protein synthesis up to at least 120 min following the loss of exogenous nutrients.

### Adhesin gene expression in PgSg communities

**Figure 3:**
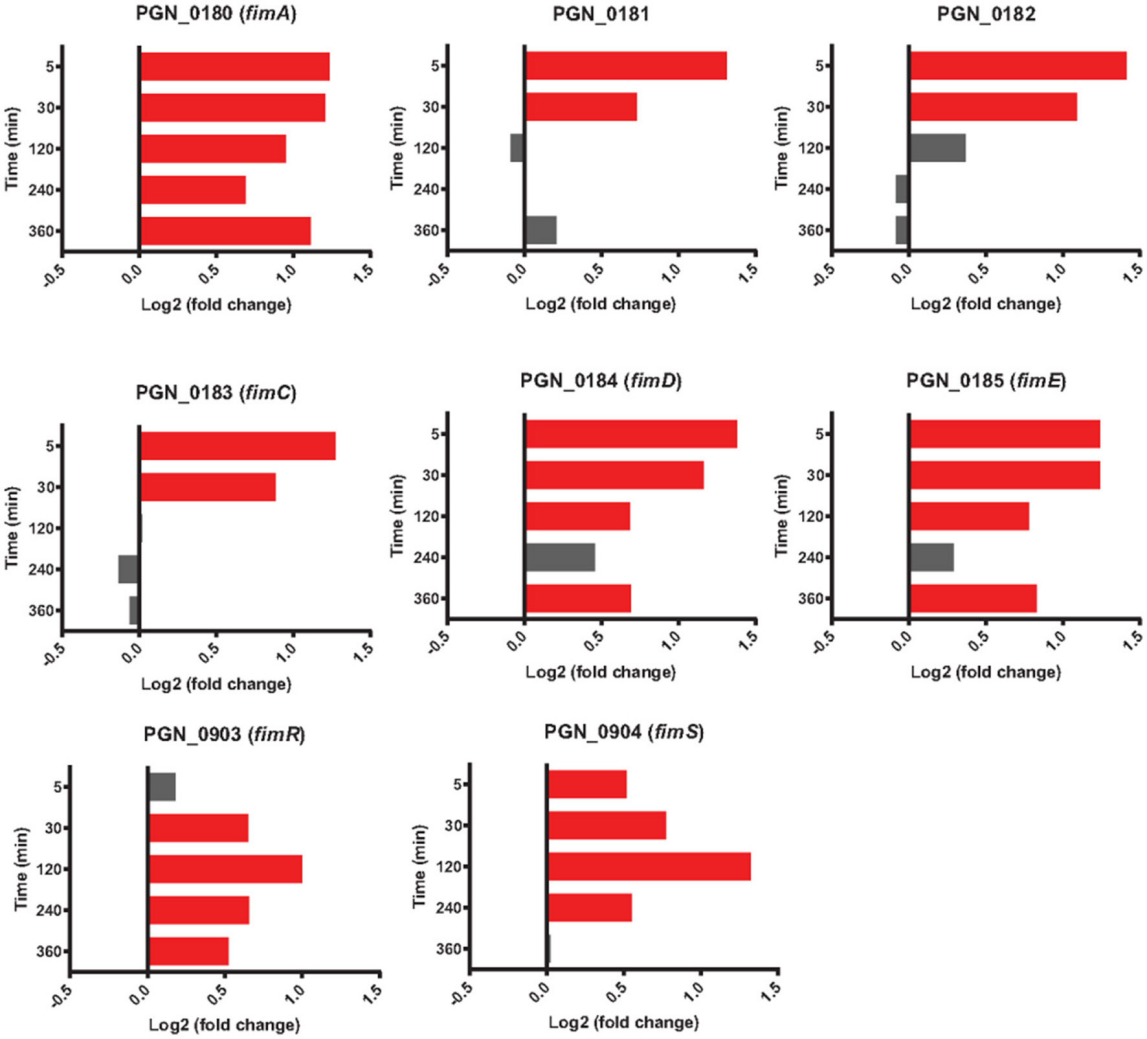
**Differential expression of *fimA* locus genes (PGN_0180-PGN_0185) along with *fimSR* (PGN_0903 and PGN_0904) in communities of PgSg**. Results are expressed as log_2_ fold change in PgSg compared to Pg alone at the times indicated. Higher mRNA levels are represented by red bars, see Methods for statistical thresholds.

Comparison of genes regulated in the PgSg condition with Pg alone revealed that adherence-associated genes comprised 8 of the top 15 (by degree of expression change) genes with higher relative RNA levels after 5 min (Table 1). Attachment of Pg to Sg involves two types of fimbriae: the longer fimbriae comprised of the FimA structural subunit and the shorter fimbriae, comprised of the Mfa1 structural subunit (Wright et al., 2013). Initial contact between Pg and Sg is mediated by engagement of the FimA protein with GAPDH on the streptococcal surface (Maeda et al., 2004). Expression of the *fimA* gene was increased in PgSg communities at all time points (Figure 3), indicating that Pg responds to the presence of Sg by increasing the availability of a major coadhesin. Similarly, Maeda et al. (2015) reported increased fimbrial expression, at both the protein and mRNA levels, in communities of Pg with the closely related streptococcus *S. oralis*. Pg genes for FimA accessory proteins PGN_0181 and PGN_0182 (the two fragments of *fimB* in strain 33277, Nagano et al., 2010), *fimC, fimD*, and *fimE* had higher mRNA levels compared to Pg alone, although more consistently at the 5 and 30 min time points. Alignment of the RNA-Seq reads with the genome (Figure S2), also showed different levels of expression of *fimA* compared to *fimB-E*, in accord with previous reports that the major fimbrial genes in Pg do not comprise an operon (Nishikawa et al., 2004). Interestingly, mRNA levels of *fimS* and *fimR*, encoding the two component system (TCS) which controls *fimA* transcription (Nishikawa et al., 2004), were highest compared to Pg alone at 120 min when *fimA* mRNA levels were beginning to decline (Figure 3) indicating a complex control system for the production of FimA. However, TCS require the appropriate signal for activity, and thus there is not a linear relation between level of protein (or mRNA) and information flow.

**Table 1:** **Top 25 genes with the highest mRNA levels in PgSg communities compared to Pg over time by RNA-Seq**.

**Time (min)**				
**5**	**30**	**120**	**240**	**360**
PGN_1401	PGN_1535	PGN_0798	PGN_0458	PGN_0458
PGN_0273	PGN_0273	PGN_0458	PGN_0798	PGN_0798
PGN_0182 *fimB*	PGN_1534	PGN_0357 *sufB*	PGN_1227 TPR motif	PGN_0357 *sufB*
PGN_1906 *hagC*	PGN_1639	PGN_0802	PGN_0357 *sufB*	PGN_0388
PGN_1904 *hagB*	PGN_0802	PGN_1238	PGN_1238	PGN_1413
PGN_0184 *fimD*	PGN_0798	PGN_0856	PGN_0856	PGN_1507
PGN_1535	PGN_1541	PGN_1227 TPR motif	PGN_0855	PGN_1204
PGN_0181 *fimB*	PGN_1401	PGN_1210	PGN_1413	PGN_0937
PGN_1639	PGN_1326	PGN_0273	PGN_0937	PGN_0927
PGN_0183 *fimC*	PGN_0931	PGN_1535	PGN_1507	PGN_1816
PGN_1534	PGN_0778 *porT*	PGN_0358 *sufC*	PGN_0043	PGN_1238
PGN_0185 *fimE*	PGN_0932	PGN_1817	PGN_1204	PGN_0043
PGN_0180 *fimA*	PGN_0458	PGN_0937	PGN_1903	PGN_0423
PGN_1460	PGN_1687	PGN_0982	PGN_1817	PGN_0752
PGN_0826	PGN_2009	PGN_0928	PGN_1210	PGN_1752
PGN_0778 *porT*	PGN_0856	PGN_1534	PGN_0928	PGN_1424
PGN_0549	PGN_1823	PGN_0778 *porT*	PGN_0927	PGN_0855
PGN_0412	PGN_1821	PGN_0043	PGN_0982	PGN_1705
PGN_1674	PGN_1673	PGN_0336	PGN_0358 *sufC*	PGN_0928
PGN_1505	PGN_1837	PGN_1211	PGN_1988	PGN_0380
PGN_1461	PGN_1906 *hagC*	PGN_1376	PGN_1480	PGN_0739
PGN_1402	PGN_1904 *hagB*	PGN_1340	PGN_0972 TPR motif	PGN_0569
PGN_2050	PGN_0185 *fimE*	PGN_1903	PGN_0172	PGN_1423
PGN_1673	PGN_0580	PGN_1413	PGN_0380	PGN_1145
PGN_1053	PGN_0180 *fimA*	PGN_1507	PGN_0388	PGN_0972 TPR motif

**Figure 4:**
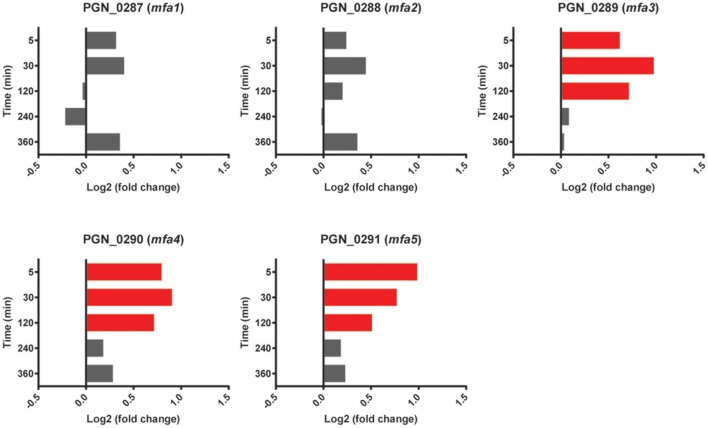
**Differential expression of *mfa1* locus genes (PGN_0287-PGN_0291) in communities of PgSg**. Results are expressed as log_2_ fold change in PgSg compared to Pg alone at the times indicated. Higher mRNA levels are represented by red bars.

Expression of Mfa1 in dual species PgSg communities is downregulated after 20 h, as the developing Pg microcolony no longer requires interspecies adhesion (Park et al., 2006). *mfa1*, along with genes encoding Mfa2, the anchor and filament length regulating protein, and Mfa3-5, which decorate the fimbrial structure have been shown to comprise an operon (Hasegawa et al., 2009, 2013; Ikai et al., 2015). Neither *mfa1* nor *mfa2* showed significant differences with Sg (Figure 4); however, *mfa3, mfa4* and *mfa5* had higher expression levels in PgSg compared to Pg at 5 min through 120 min. Examination of sequence reads in the intergenic *mfa2*-*mfa3* region did not reveal the presence of a potential transcriptional start site (tss) upstream of *mfa3*. Hence, Pg may utilize posttranscriptional mechanisms to fine tune production of Mfa3-5. Furthermore, alignment of the RNA-Seq reads with the genome (Figure S3), shows that *mfa1* is transcriptionally detached from the downstream genes in the *mfa* locus. Sequence read analysis did not indicate the presence of a tss upstream of *mfa2*, and thus the lower levels of *mfa2-5*, compared to *mfa1*, may result from the presence of transcriptional terminators, or be caused by differences in RNA stability. The specific roles of Mfa2-5 in PgSg community formation are under investigation in our laboratories.

In addition to contributing to the PgSg interaction, both the FimA and Mfa fimbriae display a number of properties consistent with a role in the periodontal disease process. FimA can mediate attachment to a number of oral substrates including epithelial cells, matrix proteins and a variety of bacteria (Lamont and Jenkinson, 1998; Enersen et al., 2013). FimA is strongly proinflammatory while concomitantly reducing TLR2 mediated responses, a strategy that has been proposed to benefit the organism through the production of proteinaceous inflammatory breakdown products while impeding clearance (Hajishengallis et al., 2008; Hajishengallis and Lambris, 2011). The Mfa fimbriae can also mediate adherence to host cells and are involved in auto-aggregation and monotypic biofilm formation (Umemoto and Hamada, 2003; Kuboniwa et al., 2009a). Additionally, Mfa1 selectively engages the dendritic cell (DC) C-type lectin DC-SIGN, which facilitates evasion of antibacterial autophagy and lysosome fusion, and enhances intracellular persistence in myeloid DCs (El-Awady et al., 2015).

**Figure 5:**
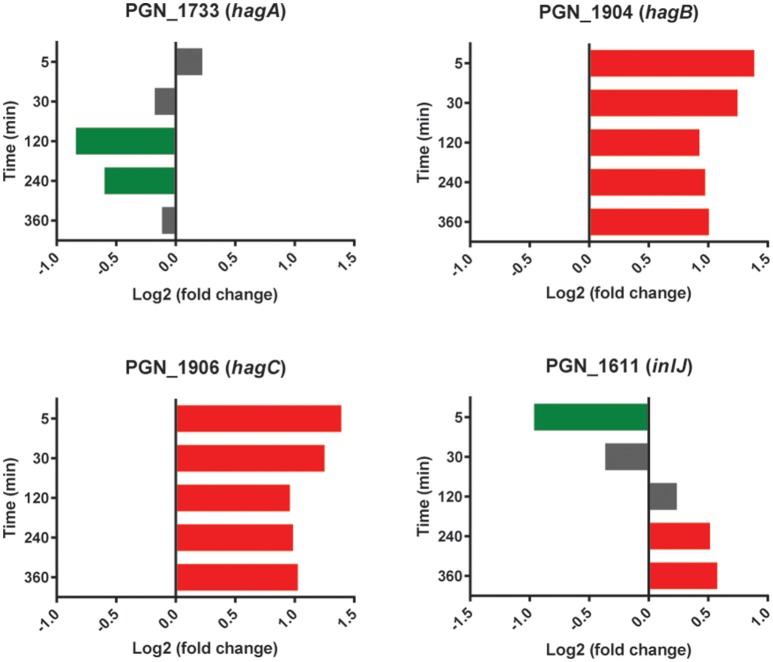
**Differential expression of hemagglutinin and *inlJ* genes in communities of PgSg**. Results are expressed as log_2_ fold change in PgSg compared to Pg alone at the times indicated. Higher mRNA levels are represented by red bars, lower mRNA levels are represented by green bars.

Transcripts for the highly conserved hemagglutinin (Hag) B and HagC showed higher levels in the presence of Sg at all time points (Figure 5). Although these adhesins have not been implicated in interbacterial binding (Lépine et al., 1996), they can mediate attachment to host cells (Song et al., 2005). Pg may thus use Sg as a cue for the presence of oral tissues and maintaining higher levels of the relevant adhesins. HagB is considered a major virulence factor of the organism (Pingel et al., 2008) and increased expression may thus contribute to the synergistic pathogenicity of Pg-Sg communities (Daep et al., 2011). In contrast, the gene encoding HagA, which is structurally and functionally distinct from HagB/C (Bélanger et al., 2012), showed lower levels of expression with Sg at 120 and 240 min, and was not significantly different between PgSg and Pg at other time points (Figure 5), suggestive of the existence of a selective process for the regulation of haemagglutinin adhesins in Pg prompted by the presence of Sg.

The leucine-rich repeat (LRR) domain Internalin (Inl) J of Pg is involved in adherence to abiotic surfaces (Capestany et al., 2006). An InlJ mutant, however, exhibits enhanced Pg-Sg community formation (Capestany et al., 2006). The expression profile of *inlJ* (Figure 5) was consistent with a role for InlJ in constraining Pg-Sg community development. Transcripts for inlJ were lower with Sg at the 5 min time point but higher at 120–360 min consistent with Pg facilitating community development early in the interaction and then implementing strategies to constrain heterotypic community accumulation (Chawla et al., 2010).

### Regulators of *P. gingivalis* community formation with *S. gordonii*

**Figure 6:**
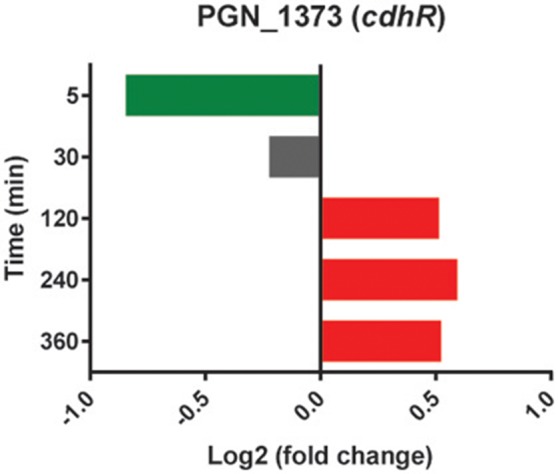
**Differential expression of the *cdhR* gene in communities of PgSg**. Results are expressed as log_2_ fold change in PgSg compared to Pg alone at the times indicated. Higher mRNA levels are represented by red bars, lower mRNA levels are represented by green bars.

In addition to co-adhesion between Pg and Sg, a phosphotyrosine dependent signaling network controls heterotypic community development (Maeda et al., 2008; Wright et al., 2014). Pg can sense metabolites produced by Sg and activates phosphoprotein signal transduction leading to the production of the Mfa1 adhesin and the LuxS enzyme responsible for the production of AI-2 (Kuboniwa et al., 2006; Chawla et al., 2010). However, in the current model system whereby immediate close contact is imposed upon the cells, and in the absence of the opportunity to first interact with Sg metabolites, PgSg did not show higher levels of *mfa1* or *luxS* transcription relative to Pg alone. Interestingly, the expression pattern of the gene encoding CdhR, a transcriptional regulator which suppresses *mfa1* and *luxS* (Chawla et al., 2010), showed lower levels at 5 min and higher levels at 120–360 min compared to Pg (Figure 6), consistent with its role in PgSg community development (Chawla et al., 2010), but indicating the existence of mulilevel control of *mfa1* and *luxS* expression. Dissection of these pathways is an area of active investigation in our laboratories.

### Stress responses

**Figure 7:**
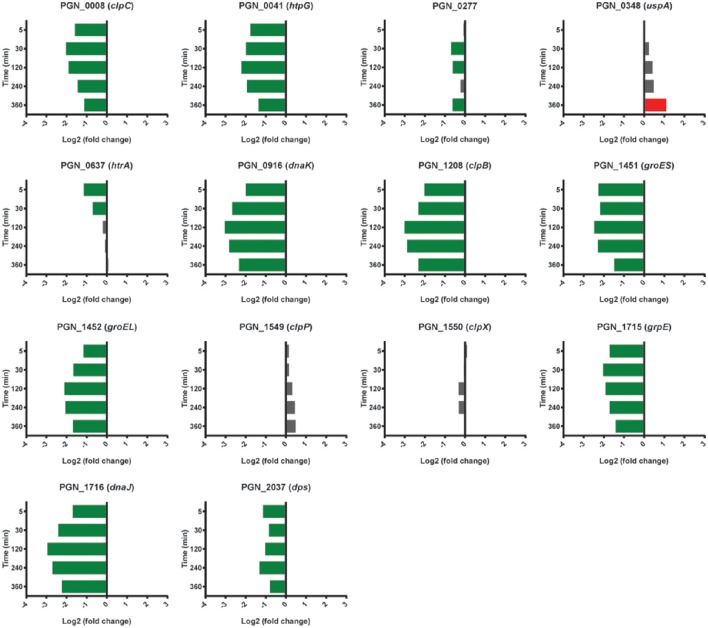
**Differential expression of stress response related genes in communities of PgSg**. Results are expressed as log_2_ fold change in PgSg compared to Pg alone at the times indicated. Higher mRNA levels are represented by red bars, lower mRNA levels are represented by green bars.

Models of the formation and development of heterotypic plaque communities hold that physiologically compatible organisms tend to cluster together (Stacy et al., 2016). In support of this notion, mRNA levels for Pg genes involved in stress responses were lower in PgSg communities compared to Pg alone over all time points. Stress-response related genes that were lower with Sg throughout the time course included *groES, groEL, dnaJ, dnaK, clpB, clpC, and htpG* (Figure 7). A notable exception to the overall reduced stress environment for Pg, was a number of oxidative stress related genes that were increased. These genes comprised predominantly the OxyR regulon (Diaz et al., 2006). The gene encoding OxyR itself showed higher mRNA levels at 5 min through 30 min, followed by higher levels in PgSg compared to Pg of 15 of 26 predicted regulon genes at 120 min, and by 360 min, 20 of the predicted regulon genes demonstrated elevated expression (Table 2). In addition, mRNA coding for the bacterioferritin co-migratory protein (Bcp, PGN_1058), which contributes to oxidative stress resistance in Pg (Johnson et al., 2011), was higher at 240 and 360 min compared to Pg alone. Sg produces H_2_O_2_ as an end product of carbohydrate fermentation, and levels of H_2_O_2_ can reach millimolar concentrations in heterotypic communities with another periodontal pathogen, *Aggregatibacter actinomycetemcomitans* (Liu et al., 2011). Indeed, the spatial organization within *A. actinomycetemcomitans*—Sg communities is such that *A. actinomycetemcomitans* is sufficiently distant from Sg to reduce H_2_O_2_ toxicity, while remaining in proximity to utilize streptococcal lactic acid as a nutritional substrate (Stacy et al., 2014). Thus, we propose that H_2_O_2_ produced by Sg within PgSg communities results in a detoxifying OxyR response by Pg to maintain a synergistic interaction.

**Table 2:** **Differential expression^a^ of genes associated with the OxyR regulon in communities of PgSg**.

**Gene**	**Gene name/function**	**Log_2_ (Fold change)**				
		**5 min**	**30 min**	**120 min**	**240 min**	**360 min**
PGN_0368	*oxyR*, redox-sensitive transcriptional activator	0.57	0.56	0.35	−0.01	−0.62
PGN_0035	*rplS*, 50S ribosomal protein L19	−1.05	−0.83	−0.93	−1.23	−0.70
PGN_0167	*rpsP*, 30S ribosomal protein S16	−1.62	−1.47	−1.49	−1.43	−0.66
PGN_0301	conserved hypothetical protein	−0.20	0.59	0.86	0.68	0.83
PGN_0357	ABC transporter membrane protein	−1.05	0.91	2.62	2.48	2.38
PGN_0373	putative thioredoxin	−1.39	0.23	1.66	1.35	1.67
PGN_0564	superoxide dismutase Fe-Mn	−1.13	−0.04	1.03	1.33	1.76
PGN_0567	*prtC*, collagenase	0.84	0.90	0.88	0.52	0.14
PGN_0569	S-adenosylmethionine: tRNA ribosyltransferase-isomerase	−0.58	−0.03	1.21	1.44	1.85
PGN_0604	ferritin	−0.60	−0.04	0.59	0.46	0.72
PGN_0638	*rpoD*, RNA polymerase sigma factor	−0.76	−0.21	0.55	0.54	0.56
PGN_0639	*rpsF*, 30S ribosomal protein S6	−0.57	−0.25	0.12	0.25	0.66
PGN_0660	*ahpC*, alkyl hydroperoxide reductase C subunit	−1.12	−0.54	−1.30	−1.75	−1.14
PGN_0661	*ahpF*, alkyl hydroperoxide reductase F subunit	−0.56	−0.17	−1.10	−1.33	−0.82
PGN_0722	conserved hypothetical protein	−0.74	0.35	1.46	1.50	1.56
PGN_0741	TonB-dependent receptor	−0.56	−0.52	0.50	0.80	1.00
PGN_1111	formate-tetrahydrofolate ligase	−0.56	0.01	0.79	0.65	0.70
PGN_1172	acyl-CoA dehydrogenase short-chain specific	0.95	0.89	0.64	0.63	0.64
PGN_1186	*rprY*, DNA-binding response regulator	−0.85	−0.37	0.10	0.12	0.71
PGN_1206	putative methylenetetrahydrofolate dehydrogenase	−0.07	−0.08	0.25	0.57	0.51
PGN_1221	probable ATP:corrinoid adenosyltransferase	−1.01	−0.34	0.52	0.39	0.54
PGN_1232	thioredoxin reductase	−0.72	−0.08	1.08	1.42	1.40
PGN_1526	conserved hypothetical protein	−0.90	0.65	1.57	1.15	0.71
PGN_1547	conserved hypothetical protein	−1.44	−0.29	0.65	1.05	1.13
PGN_1580	*rpsU*, putative 30S ribosomal protein S21	−1.11	−0.60	−0.01	0.49	1.01
PGN_1891	*rpmB*, 50S ribosomal protein L28	−0.18	0.22	0.44	0.33	0.92
PGN_2037	*dps*, DNA-binding protein from starved cells	−1.14	−0.84	−1.02	−1.31	−0.78

a *Results are expressed as log_2_ fold change in PgSg compared to Pg alone at the times indicated. Higher mRNA levels are represented by red font, lower mRNA levels are represented by green font*.

### Type IX secretion

**Figure 8:**
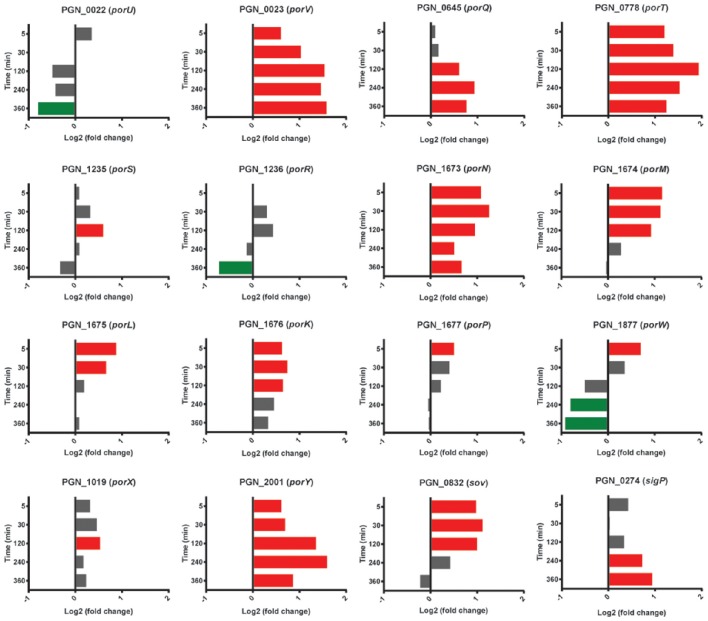
**Differential expression of genes for type IX secretion system components in communities of PgSg**. Results are expressed as log_2_ fold change in PgSg compared to Pg alone at the times indicated. Higher mRNA levels are represented by red bars, lower mRNA levels are represented by green bars.

Pg produces a type IX secretion system (T9SS) for the translocation of around 30 proteins, from the periplasm across the outer membrane (Nakayama, 2015). Target proteins possess a conserved C-terminal domain that is necessary for secretion. The machinery of the T9SS comprises over 10 proteins and is controlled by the PorX/Y TCS and the extracytoplasmic function (ECF) sigma factor SigP (Kadowaki et al., 2016). In the heterotypic community with Sg, Pg had higher mRNA levels for the gene for PorY at all time points, and *sigP* expression was higher at 240–360 min (Figure 8). mRNA of T9SS machinery components PorK, PorL, PorM, PorN (genes *porKLMN* are adjacent in the chromosome), PorQ, PorT, PorV, and Sov were also higher in the presence of Sg, predominantly over 5–120 min. Indeed *porT* was among the top 25 genes with the highest levels of mRNA for PgSg compared to Pg during this period (Table 1). Although not all of the T9SS-associated genes were consistently differentially expressed, at present little is known regarding the structural organization of the T9SS system and proteins with distinct functional roles may be optimally present in different amounts. A number of virulence-associated proteins, including the gingipain proteases and PAD, are secreted through the T9SS (Nakayama, 2015). Thus, higher levels of T9SS components could make a significant contribution to the increased pathogenicity of communities of PgSg communities compared to Pg alone, even in the absence of increased expression of the virulence factors themselves.

### Tetratricopeptide repeat proteins

**Figure 9:**
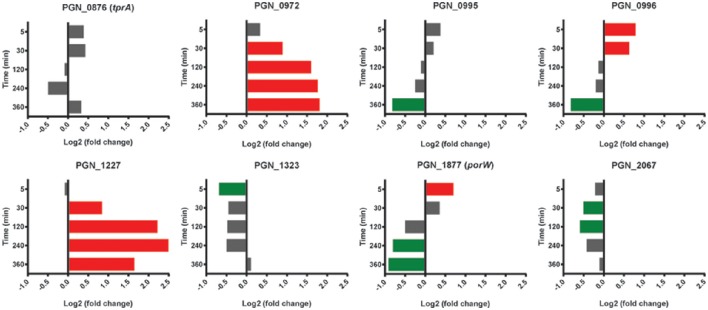
**Differential expression of genes encoding tetratricopeptide repeat (TPR) motif containing proteins in communities of PgSg**. Results are expressed as log_2_ fold change in PgSg compared to Pg alone at the times indicated. Higher mRNA levels are represented by red bars, lower mRNA levels are represented by green bars.

The tetratricopeptide repeat (TPR) motif is a protein-protein interaction module found in multiple copies in a number of functionally different proteins and which facilitates specific interactions with a partner protein. In Pg loss of the TPR protein TprA renders the organism less virulent in the murine subcutaneous model of infection (Kondo et al., 2008). TprA (PGN_0876) did not show differential relative abundance changes with Sg; however mRNA for 3 of 8 predicted TPR-domain proteins was higher compared to Pg at two or more time points (Figure 9). PGN_0972 and PGN_1227 were higher at 30 min through 360 min, with PGN_0972 among the top 25 most differentially expressed genes at 120 and 240 min, and PGN_0972 in the top 25 at 240 and 360 min (Table 1).

### Conjugation system

A number of Pg strains, including 33277, are capable of conjugal transfer of both chromosomal DNA and conjugative transposons via a homolog of the type IV secretion system, and based on homologs of the DNA transfer regions (*tra* genes) of the *Bacteroides* conjugative transposons cTnDot and cTn*341* (Tribble et al., 2007). Strain 33277 contains three clusters of *tra* genes with more than one ortholog of many of the components. As shown in Table 3, in the PGN_0056 to PGN_0076 cluster, mRNA levels all of the *tra* homologs were lower in PgSg than Pg over at least two time points, and 5 genes showed lower levels at all time points. A similar pattern of expression was observed in the PGN_1281-1285 region, whereas the PGN_0578-PGN_0599 region showed variable differences. In general, transfer of DNA is utilized by panmictic pathogens to facilitate adaptation as environmental conditions become challenging (Jolley et al., 2007; Tribble et al., 2007). Although the functionality of the Tra homologs in Pg has not been verified, these data are consistent with the stress response data and support the concept that Pg finds the community environment with Sg to be physiological supportive.

**Table 3:** **Differential expression^a^ of genes associated with conjugation in communities of PgSg**.

**Gene**	**Gene name/function**	**Log_2_ (Fold change)**				
		**5 min**	**30 min**	**120 min**	**240 min**	**360 min**
PGN_0056	probable conserved protein found in conjugate transposon	−0.74	−0.65	−0.41	−0.51	−0.08
PGN_0057	*traP*, probable conserved protein found in conjugate transposon	−0.49	−0.30	−0.42	−0.70	−0.50
PGN_0058	probable conserved protein found in conjugate transposon	−0.91	−1.11	−1.05	−1.07	−0.50
PGN_0059	*traN*, conserved protein found in conjugate transposon	−0.61	−0.62	−0.76	−0.71	−0.17
PGN_0060	*traM*, conserved protein found in conjugate transposon	−0.61	−0.67	−1.07	−1.03	−0.53
PGN_0061	hypothetical protein	−0.94	−0.71	−0.91	−0.42	0.01
PGN_0062	*traK*, putative conserved protein found in conjugate transposon	−0.971	−0.81	−1.20	−0.79	−0.57
PGN_0063	*traJ*, conserved transmembrane protein found in conjugate transposon	−0.71	−0.94	−1.11	−0.86	−0.59
PGN_0064	*traI*, putative conserved protein found in conjugate transposon	−0.53	−0.77	−0.97	−0.81	−0.26
PGN_0065	*traG*, conserved protein found in conjugate transposon	−0.80	−0.92	−1.11	−1.23	−0.96
PGN_0066	*traF*, conserved transmembrane protein found in conjugate transposon	−0.84	−0.96	−1.14	−0.98	−0.48
PGN_0067	*traE*, conserved transmembrane protein found in conjugate transposon	−0.59	−0.69	−1.01	−0.85	−0.42
PGN_0068	hypothetical protein	0.11	0.13	0.03	−0.11	0.29
PGN_0069	*traA*, probable conserved protein found in conjugate transposon	−0.16	−0.44	−0.62	−0.54	−0.49
PGN_0070	hypothetical protein	−0.13	−0.58	−0.53	−0.54	−0.65
PGN_0071	hypothetical protein	−0.25	−0.41	−0.46	−0.48	−0.50
PGN_0072	hypothetical protein	−0.31	−0.68	−0.51	−0.52	−0.63
PGN_0073	*traA*, putative conserved protein found in conjugate transposon	−0.50	−0.71	−0.70	−0.54	−0.82
PGN_0074	conserved hypothetical protein	−1.11	−0.89	−0.51	−0.81	−1.09
PGN_0075	conserved hypothetical protein	−0.70	−0.57	0.28	−0.22	−1.04
PGN_0076	putative mobilization protein TraG family	−0.35	−0.55	−0.59	−1.03	−1.41
PGN_0592	*traQ*, putative conserved protein found in conjugate transposon	0.40	0.65	0.31	0.68	−0.29
PGN_0593	*traO*, putative conserved protein found in conjugate transposon	0.25	0.57	0.54	0.32	−0.20
PGN_0594	*traN*, conserved protein found in conjugate transposon	0.06	0.11	0.39	0.27	−0.44
PGN_0595	*traM*, putative conserved protein found in conjugate transposon	−0.27	0.06	0.04	−0.45	−0.85
PGN_0596	conserved hypothetical protein found in conjugate transposon	−0.58	−0.31	0.41	0.46	0.23
PGN_0597	*traK*, putative conserved protein found in conjugate transposon	−0.60	−0.58	0.16	0.38	−0.71
PGN_0598	*traJ*, conserved transmembrane protein found in conjugate transposon	−0.76	−0.89	0.03	0.16	−1.62
PGN_0599	*traI*, putative conserved protein found in conjugate transposon	−0.39	−0.39	0.20	0.52	−0.66
PGN_1281	*traM*, putative conserved protein found in conjugate transposon	−0.54	−0.80	−1.06	−1.03	−0.53
PGN_1282	*traN*, conserved protein found in conjugate transposon	−0.64	−0.69	−0.79	−0.62	−0.15
PGN_1283	*traO*, conserved protein found in conjugate transposon	−0.84	−1.10	−1.17	−0.97	−0.57
PGN_1284	*traP*, putative DNA primase involved in conjugation	−0.36	−0.44	−0.44	−0.57	−0.58
PGN_1285	*traQ*, conserved protein found in conjugate transposon	−0.73	−0.50	−0.50	−0.58	−0.04

a *Results are expressed as log_2_ fold change in PgSg compared to Pg alone at the times indicated. Higher mRNA levels are represented by red font, lower mRNA levels are represented by green font*.

### Hemin acquisition

Pg has an obligate requirement for iron in the form of hemin (Lewis, 2010; Smalley and Olczak, 2017). As Pg adapted to attachment and accretion in our model, there was a general trend of lower mRNA of hemin uptake associated genes in the presence of Sg (Table 4). One notable exception was higher levels, over 120–360 min, of the genes encoding the HaeS/R TCS, which regulates components of hemin uptake systems including the *hmu*Y*RSTUV* operon along with a number of TonB-dependent receptors, transporters and ABC transporters (Scott et al., 2013). While, as noted above, the activity of a TCS will depend on the presence of appropriate stimulatory signals in addition to the abundance of the components, collectively these data suggest that Pg may streamline and optimize hemin uptake in the absence of growth and division. The increased mRNA levels of the gene for ferritin PGN_0604 at 120 and 360 min compared to Pg (with a non-statistically significant trend up at 240 min) would be consistent with Pg transitioning intracellular iron into storage. Interestingly, Scott et al. (2013) reported that strain 33277 was mutant in *haeS* (~2.5 kbp deletion) and found barely detectable transcription from the gene. In addition, this group was unsuccessful in creating mutants in *haeS/R*, consistent with their report that *haeS/R* mutants were not represented in a transposon mutagenesis library (Scott et al., 2013). In the current model system, transcription of *haeS* was abundant and we have also found *haeS/R* mutants in a transposon library of Pg (Hutcherson et al., 2015). Differences between these datasets are likely attributable to differing growth medium between laboratories and the levels of available hemin.

**Table 4:** **Differential expression^a^ of genes associated with the hemin/iron uptake in communities of PgSg**.

**Gene**	**Protein name/function**	**Log_2_ (Fold change)**				
		**5 min**	**30 min**	**120 min**	**240 min**	**360 min**
PGN_0553	*hmuV*, conserved hypothetical protein	−0.58	−0.79	−0.66	0.18	−1.25
PGN_0554	*hmuU*, conserved hypothetical protein	−0.43	−0.67	−0.08	−0.69	−1.45
PGN_0555	*hmuT*, conserved hypothetical protein	−0.32	−0.16	−0.04	−0.26	−0.73
PGN_0556	*hmuS*, putative cobalamin biosynthesis-related protein	−0.62	−0.76	−0.38	−0.43	−1.07
PGN_0557	*hmuR*, TonB-dependent receptor	−0.94	−0.70	−0.19	−0.03	−0.65
PGN_0558	*hmuY*, conserved hypothetical protein	−2.52	−2.04	−1.63	−1.69	−1.64
PGN_0604	ferritin	−0.60	−0.04	0.59	0.46	0.72
PGN_0704	*ihtA*, putative tonB-linked outer membrane receptor	−0.19	−0.04	−0.12	−0.28	−0.01
PGN_0705	*ihtB*, heme-binding protein FetB	0.16	0.41	0.05	−0.66	0.01
PGN_0706	*ihtC*, putative exported periplasmic protein	0.65	0.43	−0.65	−0.40	−0.62
PGN_0707	*ihtD*, putative iron compound ABC transporter	0.60	0.28	−0.70	−0.58	−0.76
PGN_0708	*ihtE*, putative iron compound ABC transporter	0.33	0.05	−0.91	−0.67	−0.67
PGN_0659	HBP35, 35 kDa hemin binding protein	−0.38	0.02	0.08	0.35	0.90
PGN_0683	*tlr*, TonB-linked receptor	0.32	0.21	−0.21	−0.50	−0.30
PGN_0684	*htrD*, conserved hypothetical protein	0.25	0.24	0.35	0.16	−0.10
PGN_0685	*htrC*, putative iron compound ABC transporter	0.17	0.10	0.12	−0.43	−0.35
PGN_0686	*htrB*, putative iron compound ABC transporter	−0.12	−0.57	−0.97	−0.77	−0.65
PGN_0687	*htrA*, putative iron compound ABC transporter	0.16	−0.35	−0.41	−0.51	−0.29
PGN_0752	*haeS*, hypothetical protein	−0.23	0.14	1.32	1.64	2.08
PGN_0753	*haeR*, probable two component system response regulator	−0.21	0.54	1.78	1.67	1.81
PGN_1335	conserved hypothetical protein	−0.57	−0.46	−0.29	−0.67	−1.45
PGN_1336	conserved hypothetical protein	−0.83	−0.47	−0.32	−0.54	−0.72
PGN_2091	*husA*, conserved hypothetical protein	−0.48	−0.94	−1.21	−1.27	−0.88

a *Results are expressed as log_2_ fold change in PgSg compared to Pg alone at the times indicated. Higher mRNA levels are represented by red font, lower mRNA levels are represented by green font*.

### Suf ABC complex

**Figure 10:**
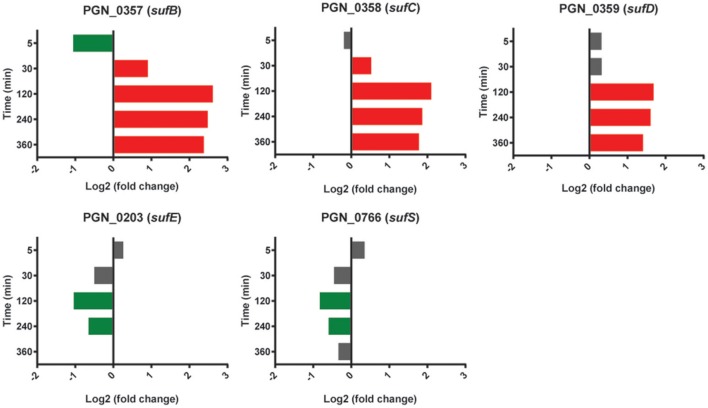
**Differential expression of genes encoding the SufABC complex in communities of PgSg**. Results are expressed as log_2_ fold change in PgSg compared to Pg alone at the times indicated. Higher mRNA levels are represented by red bars, lower mRNA levels are represented by green bars.

Expression of the *sufBCD* genes (PGN_0357-PGN_0359) was increased in PgSg compared to Pg over 120–360 min (Figure 10). *sufB* was one of the top five most significantly more highly expressed genes over this interval, and *sufC* was in the top 25 at 120 and 240 min (Table 1). SufBCD comprise an ABC protein complex. While ABC proteins are traditionally involved in transport across membranes, recent studies have established that they can also be structural maintenance of chromosome (SMC) proteins and participate in iron-sulfur (Fe-S) cluster biogenesis (Higgins, 1992; Hirano, 2005; Hirabayashi et al., 2015). The SufBCD complex is a constituent of the Suf machinery that is responsible for the *de novo* biogenesis of iron-sulfur (Fe-S) clusters which act as cofactors of Fe-S proteins (Takahashi and Tokumoto, 2002). The Suf machinery in *E. coli* includes six proteins encoded by the *sufABCDSE* operon. In Pg, *sufBCD* are adjacent in the chromosome whereas the *sufE* homolog is separate. The gene encoding SufE, the sulfur transport protein (Layer et al., 2007), was not more highly expressed in the PgSg condition compared to Pg and, moreover, was lower over 120–240 min. Expression of a potential homolog of *sufS*, encoding the cysteine desulfurase, PGN_0766, was also lower over 120–240 min. Pg does not appear to have a homolog of SufA, the F-S carrier protein and thus may co-opt another protein for this purpose. Collectively these results indicate that higher levels of *sufBCD* may be related to the need to conserve intracellular iron (see section above), rather than sulfur metabolism, although again with the caveat that functional verification of Suf proteins in Pg has yet to be established.

## Conclusions

This study presents a time–resolved comprehensive analysis of gene expression in an important periodontal pathogen as it adapts to a heterotypic community environment and pathogenic potential is elevated. While the presence of Sg resulted in more oxidative stress resistance mRNA, presumably in response to streptococcal peroxide, a community with Sg otherwise appeared to be a low stress environment for Pg, likely reflective of a long term evolutionary relationship. Higher relative levels of transcripts encoding adhesins, the type IX secretion apparatus, and tetratricopeptide repeat (TPR) motif proteins are consistent with a more virulent phenotype. Further studies are required to determine the concordance between mRNA levels and protein expression and metabolic activity, and the extent to which this *in vitro* model is reflective of the *in vivo* situation.

## Author contributions

EH, DB, DM, and QW analyzed data, prepared samples and prepared figures and tables; EH, MW, RL, and MH designed the experiments and wrote the manuscript.

## Funding

We thank the NIH NIDCR for support through DE014372 (MH), DE012505 (RL) and DE023193 (MW).

### Conflict of interest statement

The authors declare that the research was conducted in the absence of any commercial or financial relationships that could be construed as a potential conflict of interest.

## Supplementary material

Table S1 **This is the first part of three files containing the master list of differential expression data for annotated genes discussed in this study, ordered by PGN number**. Four different bar graphs showing the three possible differential expression comparisons are presented for each ORF, as an aid to visualizing the gene's behavior throughout the time course.

Table S2 **The second of three sequential files making up the master list**.

Table S3 **The third of three sequential files making up the master list**.

Table S4 **A table of differentially expressed genes annotated for the initiation, elongation or termination steps of protein translation**.

Figure S1 **Representative qRT-PCR data for *fimA, mfa1, clpB*, and *clpP***.

Figure S2 **RNA-Seq reads across the *fim* gene loci**.

Figure S3 **RNA-Seq reads across the *mfa* gene loci**.
