# Supplementary material for: Insights into Dynamic Polymicrobial Synergy Revealed by Time-Coursed RNA-Seq
Source: Front Microbiol. 2017 Feb 28;8:261. doi: 10.3389/fmicb.2017.00261 (PMC5329018; doi:10.3389/fmicb.2017.00261)

PgSg Time Course

log$_2$ (Fold Change)

Legend: P vs T=1 | PS vs T=1 | PS vs P

Locus		5m	30m	120m	240m	360m
**PGN_0001**	P vs T=1	-0.993	-1.280	-1.679	-1.982	-1.992
dnaA	PS vs T=1	-0.524	-1.110	-1.162	-0.863	-1.051
	PS vs P	0.468	0.155	0.475	1.031	0.888
	chromosomal replication initiator protein DnaA					
	DNA metabolism					
**PGN_0002**	P vs T=1	-1.196	-1.124	-1.125	-0.968	-0.738
	PS vs T=1	-0.560	-0.933	-0.657	-0.644	-0.786
	PS vs P	0.567	0.138	0.390	0.268	-0.055
	conserved hypothetical protein					
	unknown function					
**PGN_0003**	P vs T=1	-0.165	-0.328	-0.083	0.591	1.728
	PS vs T=1	0.203	0.178	0.489	1.107	1.034
	PS vs P	0.313	0.399	0.458	0.544	-0.556
	conserved hypothetical protein					
	hypothetical proteins-Conserved					
**PGN_0004**	P vs T=1	0.632	1.073	1.177	1.173	0.907
	PS vs T=1	0.252	0.567	1.036	1.393	1.388
	PS vs P	-0.392	-0.492	-0.134	0.222	0.470
	putative nicotinate mononucleotide:5,6-dimethylbenzimidazole phosphoribosyltransferase					
	energy metabolism					
**PGN_0005**	P vs T=1	0.130	0.117	0.268	0.711	1.174
	PS vs T=1	-0.751	-0.899	-0.244	0.250	0.636
	PS vs P	-0.857	-0.988	-0.500	-0.391	-0.477
	conserved hypothetical protein					
	hypothetical proteins-Conserved					
**PGN_0006**	P vs T=1	-0.799	-0.651	-0.088	0.549	1.262
	PS vs T=1	-1.438	-1.186	-0.722	-0.274	0.230
	PS vs P	-0.659	-0.548	-0.607	-0.700	-0.928
	putative Na+driven multidrug efflux pump					
	transport and binding proteins					
**PGN_0007**	P vs T=1	-0.315	0.017	0.273	-0.648	0.678
	PS vs T=1	-1.721	-2.463	-1.462	-1.145	-1.228
	PS vs P	-1.088	-1.674	-1.053	-0.505	-1.089
	hypothetical protein					
	hypothetical proteins					

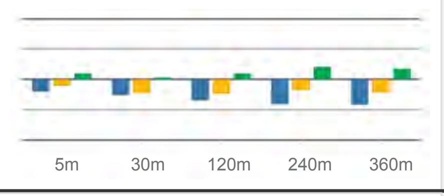

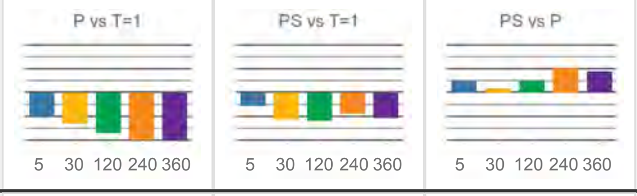

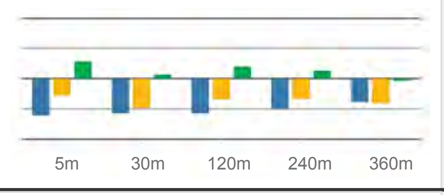

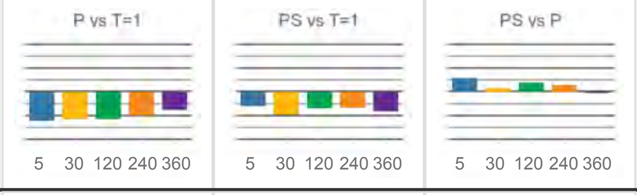

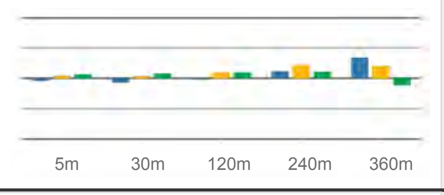

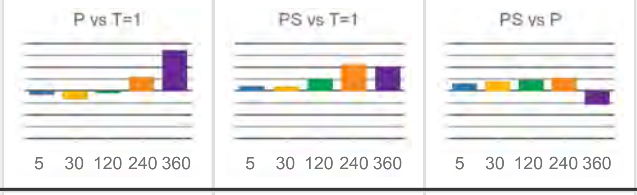

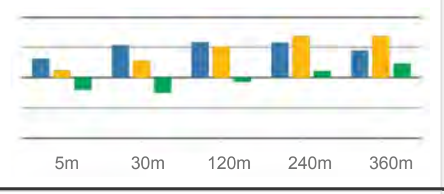

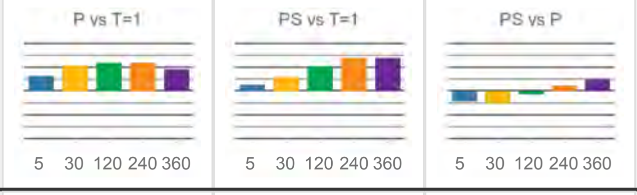

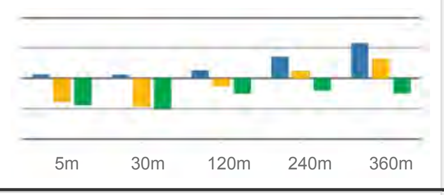

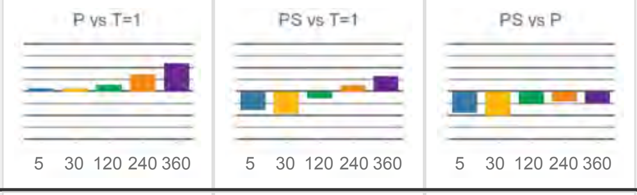

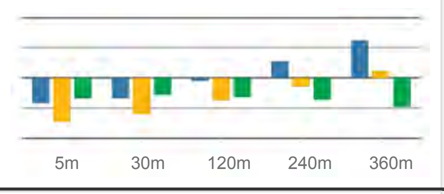

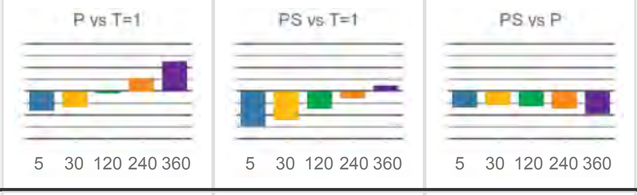

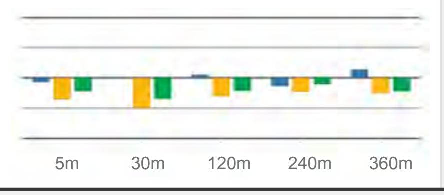

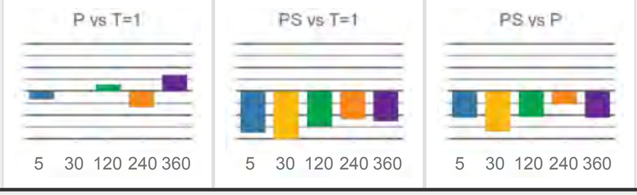

PgSg Time Course

Legend: P vs T=1 | PS vs T=1 | PS vs P

Locus		log$_2$ (Fold Change) 5m	30m	120m	240m	360m
**PGN_0008**	P vs T=1	1.417	2.180	2.781	2.919	2.498
clpC	PS vs T=1	-0.205	0.048	0.784	1.374	1.331
	PS vs P	-1.590	-2.030	-1.896	-1.445	-1.119
	ATP-dependent Clp protease ATP-binding subunit ClpC					
	protein fate					
**PGN_0009**	P vs T=1	-0.292	0.114	0.219	0.193	0.150
	PS vs T=1	-0.013	-0.119	-0.361	-0.127	-0.163
	PS vs P	0.258	-0.223	-0.566	-0.309	-0.308
	glycosyl hydrolase family 3					
	cell envelope					
**PGN_0010**	P vs T=1	-0.597	-0.502	-0.962	-1.122	-1.215
CobC	PS vs T=1	-0.204	-0.185	-0.947	-1.421	-1.569
	PS vs P	0.387	0.326	-0.005	-0.328	-0.378
	probable L-threonine-O-3-phosphate decarboxylase					
	biosynthesis of cofactors, prosthetic groups, and carriers					
**PGN_0011**	P vs T=1	-0.008	0.107	-0.411	-0.703	-0.737
	PS vs T=1	0.655	0.467	-0.297	-0.690	-0.841
	PS vs P	0.652	0.372	0.085	-0.066	-0.148
	conserved hypothetical protein					
	hypothetical proteins-Conserved					
**PGN_0012**	P vs T=1	-0.617	-0.682	-0.649	-0.475	-0.271
	PS vs T=1	-0.350	-0.614	-0.962	-0.540	-0.611
	PS vs P	0.255	0.052	-0.329	-0.065	-0.328
	two-component system response regulator					
	signal transduction					
**PGN_0013**	P vs T=1	-1.061	-1.348	-1.247	-0.655	0.456
	PS vs T=1	-0.956	-1.287	-1.417	-1.149	-0.687
	PS vs P	0.099	0.004	-0.244	-0.417	-0.994
	putative two-component system sensor histidine kinase					
	signal transduction					
**PGN_0014**	P vs T=1	-1.166	-1.659	-1.638	-0.888	0.217
	PS vs T=1	-0.776	-1.354	-1.900	-1.696	-1.376
	PS vs P	0.393	0.259	-0.326	-0.732	-1.490
	conserved hypothetical protein					
	hypothetical proteins-Conserved					

PGN_0008

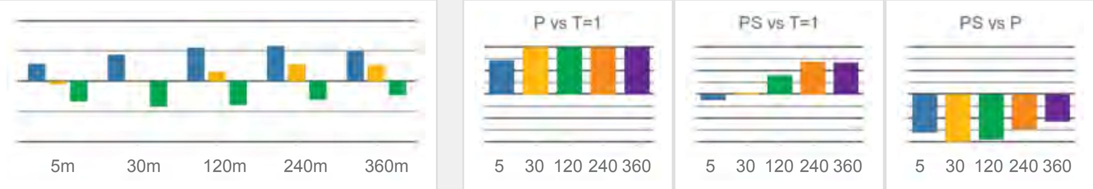

PGN_0009

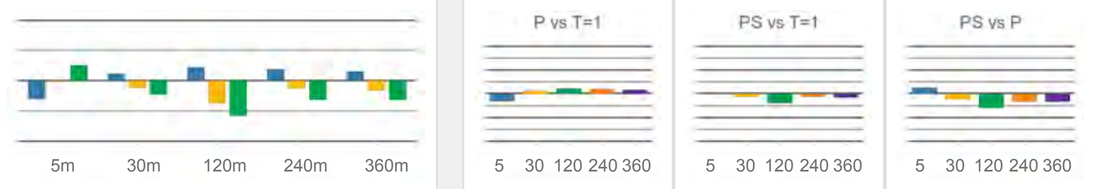

PGN_0010

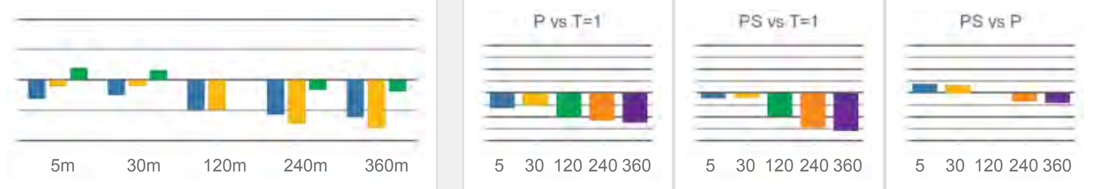

PGN_0011

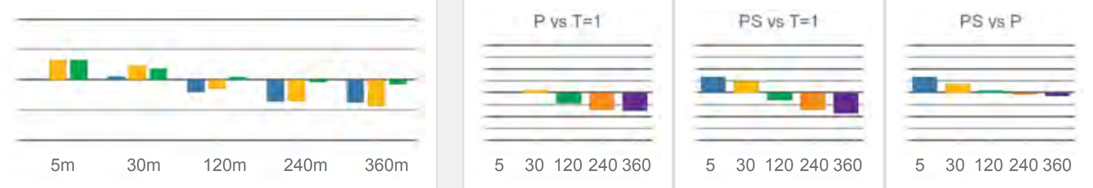

PGN_0012

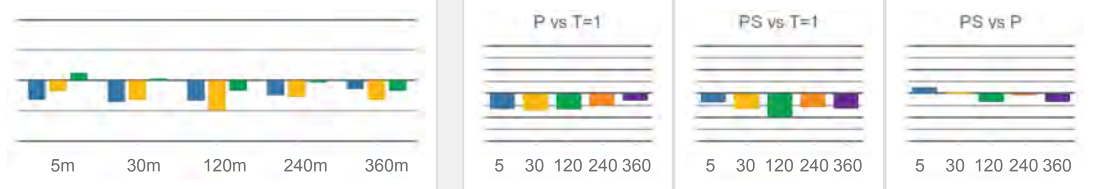

PGN_0013

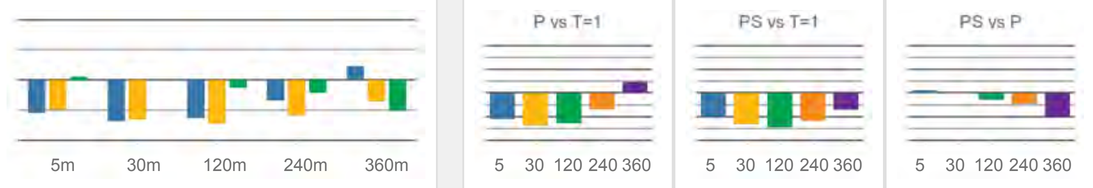

PGN_0014

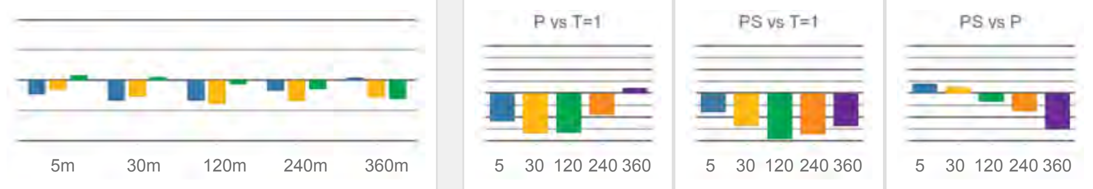

PgSg Time Course

log_2 (Fold Change)

Legend: P vs T=1 | PS vs T=1 | PS vs P

Locus		5m	30m	120m	240m	360m
**PGN_0015**	P vs T=1	-0.102	-0.741	-1.244	-1.297	-1.123
	PS vs T=1	-0.269	-0.785	-0.691	-0.354	-0.489
	PS vs P	-0.134	-0.059	0.481	0.845	0.593

probable transcriptional regulator

regulatory functions

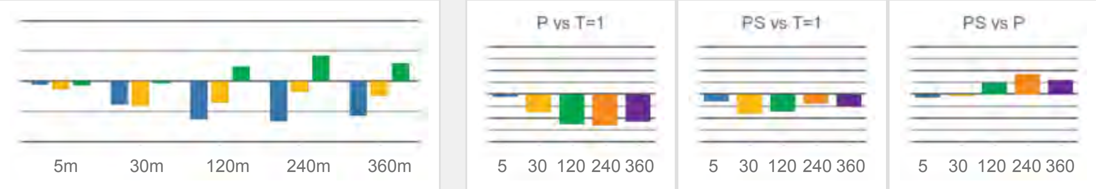

Locus		5m	30m	120m	240m	360m
**PGN_0016**	P vs T=1	-0.236	-0.628	-0.683	-0.260	0.574
	PS vs T=1	-0.641	-1.133	-0.681	-0.312	0.129
	PS vs P	-0.380	-0.526	-0.083	-0.056	-0.367

putative TIM-barrel protein

unknown function

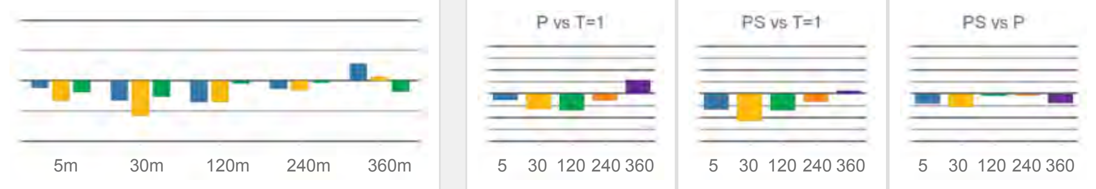

Locus		5m	30m	120m	240m	360m
**PGN_0017**	P vs T=1	0.223	0.110	0.180	0.625	1.281
	PS vs T=1	-0.079	-0.083	-0.042	0.460	0.386
	PS vs P	-0.293	-0.198	-0.239	-0.117	-0.820

sulfate transporter permease

transport and binding proteins

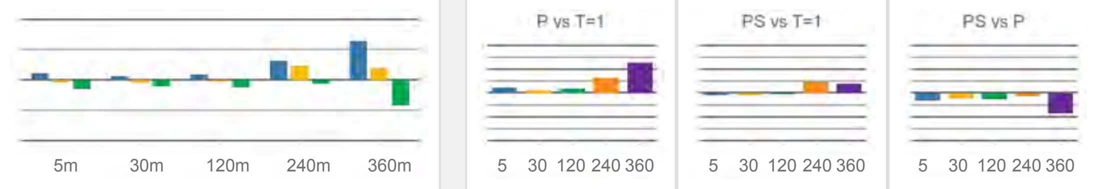

Locus		5m	30m	120m	240m	360m
**PGN_0018**	P vs T=1	0.024	0.832	1.489	1.336	2.831
	PS vs T=1	-0.207	-0.188	0.648	1.240	1.104
	PS vs P	-0.437	-0.570	-0.256	0.140	-0.485

hypothetical protein

hypothetical proteins

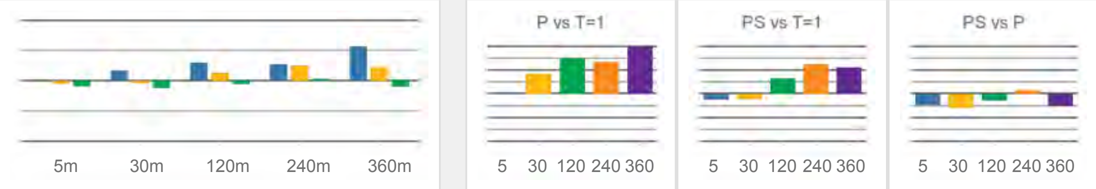

Locus		5m	30m	120m	240m	360m
**PGN_0019**	P vs T=1	0.165	0.238	1.396	2.102	3.247
	PS vs T=1	-0.387	0.262	1.618	1.838	1.976
	PS vs P	-0.665	-0.265	0.370	0.265	-0.257

hypothetical protein

hypothetical proteins

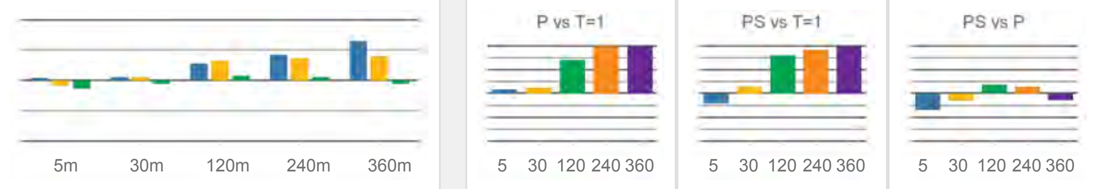

Locus		5m	30m	120m	240m	360m
**PGN_0020**	P vs T=1	-0.864	-0.542	-0.614	-0.641	-0.701
	PS vs T=1	-0.595	-0.338	-0.163	-0.051	-0.084
	PS vs P	0.178	0.185	0.407	0.531	0.565

probable DNA-binding protein

unknown function

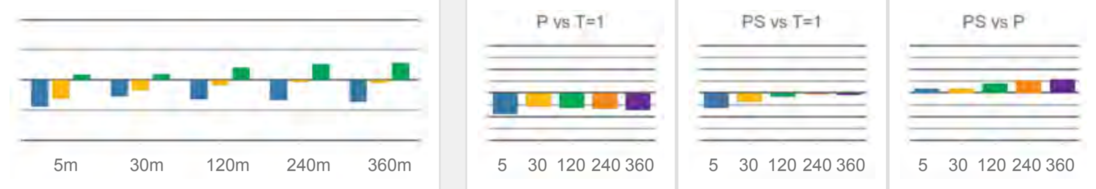

Locus		5m	30m	120m	240m	360m
**PGN_0021**	P vs T=1	-0.886	-0.571	-0.217	-0.280	-0.622
	PS vs T=1	-0.755	-0.586	-0.566	-0.210	-0.179
	PS vs P	0.099	-0.022	-0.329	0.079	0.422

probable hydrolase

energy metabolism

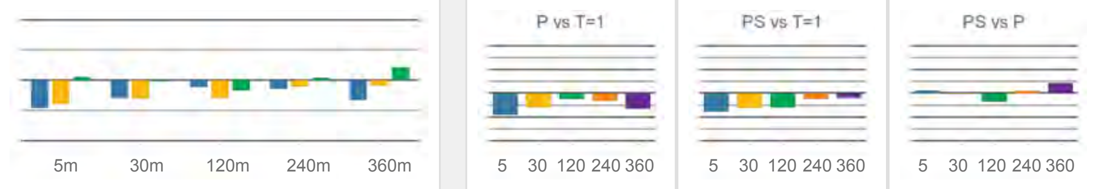

log$_2$ (Fold Change)

Locus		5m	30m	120m	240m	360m
**PGN_0022**	P vs T=1	-0.469	-0.349	-0.626	-0.604	-0.099
porU	PS vs T=1	-0.119	-0.344	-1.111	-1.026	-0.919
	PS vs P	0.347	0.013	-0.489	-0.423	-0.798

Por secretion system protein porU

hypothetical proteins

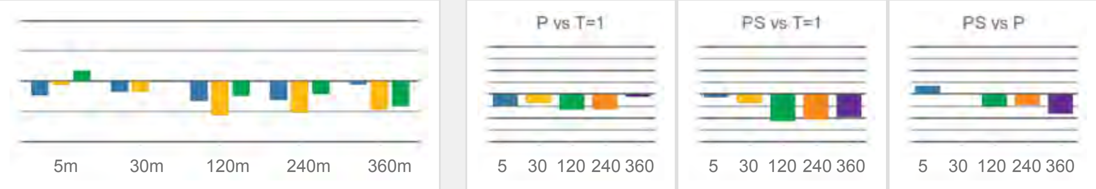

Locus		5m	30m	120m	240m	360m
**PGN_0023**	P vs T=1	1.319	1.642	1.137	0.437	0.046
porV	PS vs T=1	1.935	2.661	2.691	1.972	1.690
	PS vs P	0.597	1.024	1.534	1.454	1.576

Por secretion system protein porV (pg27, lptO)

hypothetical proteins

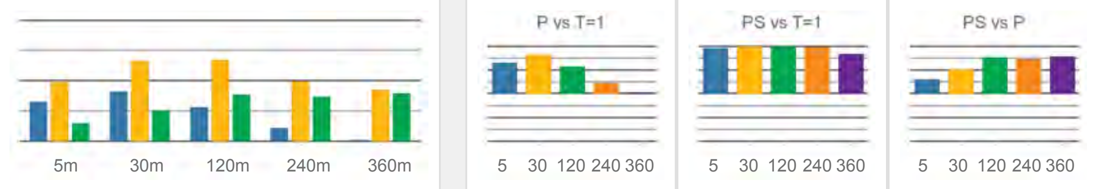

Locus		5m	30m	120m	240m	360m
**PGN_0024**	P vs T=1	-0.074	-0.562	-1.097	-1.082	-0.910
	PS vs T=1	0.145	-0.039	-0.007	-0.377	-0.350
	PS vs P	0.235	0.499	0.993	0.595	0.517

putative 2C-methyl-D-erythritol 2,4-cyclodiphosphate synthase

hypothetical proteins-Conserved

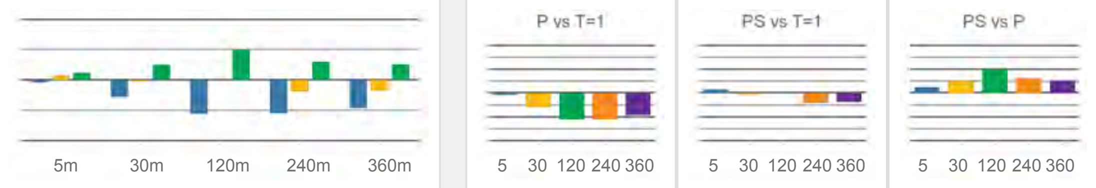

Locus		5m	30m	120m	240m	360m
**PGN_0025**	P vs T=1	-0.592	-1.772	-1.998	-1.689	-1.491
	PS vs T=1	-0.478	-0.957	-0.654	-0.693	-0.882
	PS vs P	0.157	0.716	1.204	0.908	0.571

probable SpoU rRNA methylase family protein

transcription

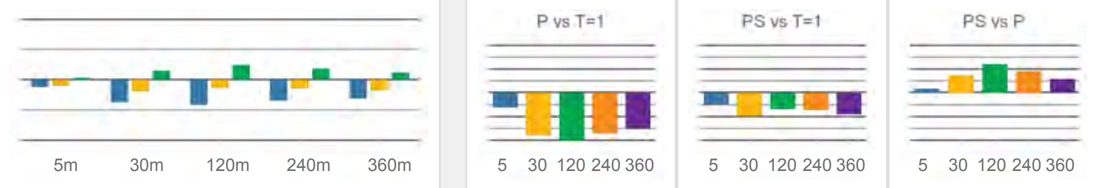

Locus		5m	30m	120m	240m	360m
**PGN_0026**	P vs T=1	0.866	1.001	0.916	0.881	1.076
	PS vs T=1	0.531	0.984	1.782	2.050	2.116
	PS vs P	-0.357	-0.032	0.790	1.041	0.990

putative cytidine deaminase

purines, pyrimidines, nucleosides and nucleotides

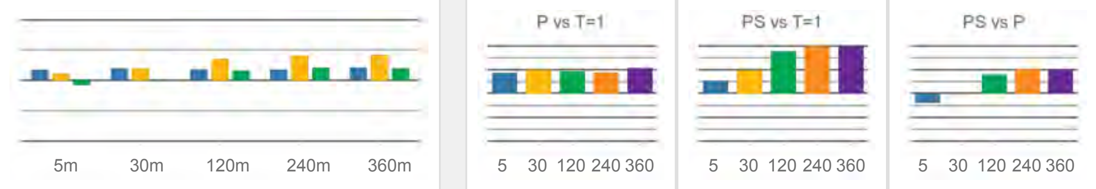

Locus		5m	30m	120m	240m	360m
**PGN_0027**	P vs T=1	0.299	0.265	0.357	0.490	0.133
	PS vs T=1	0.194	0.047	0.151	0.255	0.269
	PS vs P	-0.111	-0.228	-0.213	-0.229	0.112

transposase in ISPg1

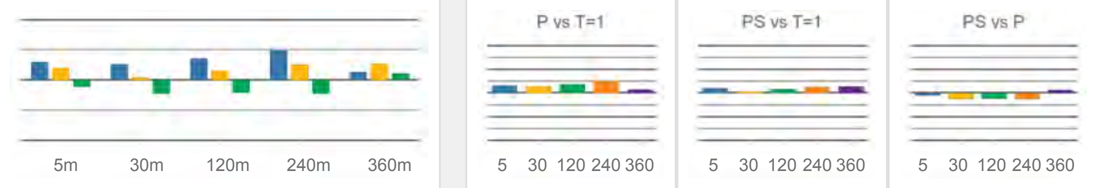

Locus		5m	30m	120m	240m	360m
**PGN_0028**	P vs T=1	0.496	0.349	0.855	0.681	0.837
	PS vs T=1	0.089	0.203	0.517	0.555	1.018
	PS vs P	-0.397	-0.193	-0.277	-0.154	0.190

hypothetical protein

hypothetical proteins

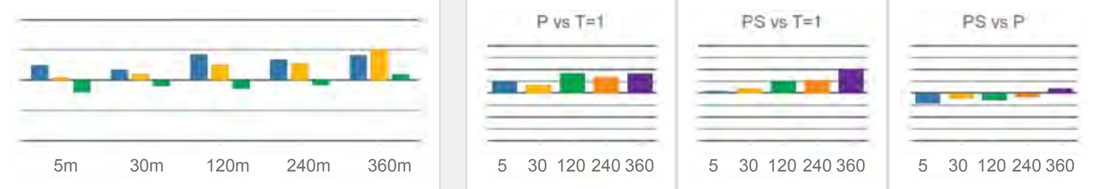

PgSg Time Course

log$_2$ (Fold Change)

P vs T=1 | PS vs T=1 | PS vs P

Locus		5m	30m	120m	240m	360m
**PGN_0029**	P vs T=1	-0.092	-0.189	-0.144	0.152	0.335
	PS vs T=1	-0.237	-0.306	-0.156	0.199	0.100
	PS vs P	-0.146	-0.132	-0.042	0.069	-0.206

conserved hypothetical protein

hypothetical proteins-Conserved

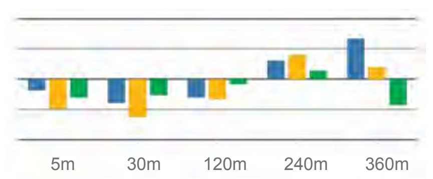

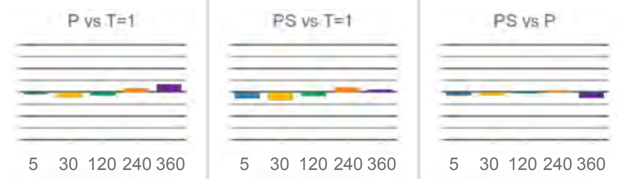

Locus		5m	30m	120m	240m	360m
**PGN_0030**	P vs T=1	-0.512	-0.345	-0.195	0.256	0.885
	PS vs T=1	-0.279	-0.647	-0.800	-0.157	0.046
	PS vs P	0.206	-0.307	-0.612	-0.367	-0.776

beta-mannosidase

cell envelope

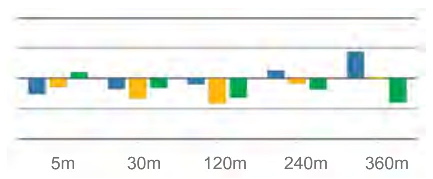

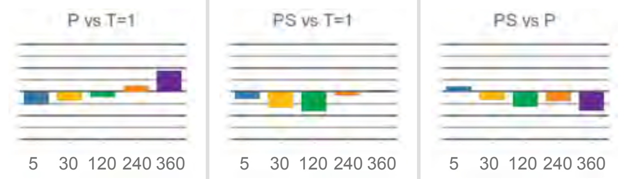

Locus		5m	30m	120m	240m	360m
**PGN_0031**	P vs T=1	0.158	0.015	-0.197	0.178	0.678
	PS vs T=1	-0.438	-0.280	0.187	0.697	0.501
	PS vs P	-0.576	-0.292	0.333	0.517	-0.140

conserved hypothetical protein with RmuC domain

hypothetical proteins-Conserved

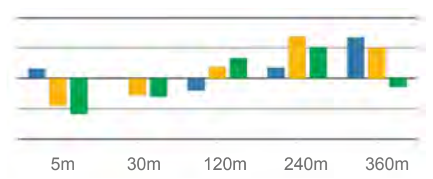

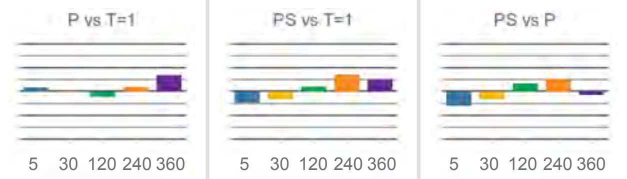

Locus		5m	30m	120m	240m	360m
**PGN_0032**	P vs T=1	0.307	0.204	0.848	1.256	2.379
	PS vs T=1	-0.626	0.117	0.212	0.332	1.069
	PS vs P	-0.799	-0.157	-0.404	-0.453	-0.591

conserved hypothetical protein

hypothetical proteins-Conserved

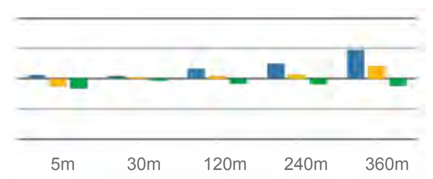

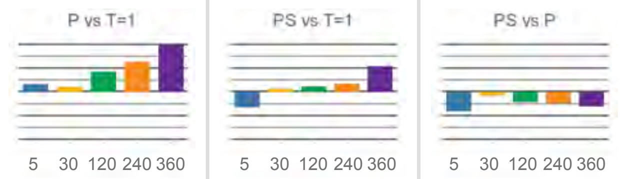

Locus		5m	30m	120m	240m	360m
**PGN_0033**	P vs T=1	-0.872	-0.437	-0.124	-0.135	-0.715
	PS vs T=1	-1.447	-0.220	0.467	-0.053	-0.353
	PS vs P	-0.609	0.217	0.611	0.104	0.338

thioredoxin

energy metabolism

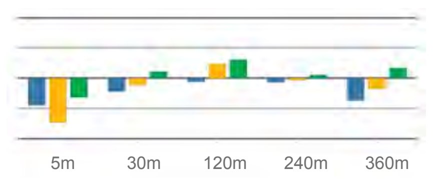

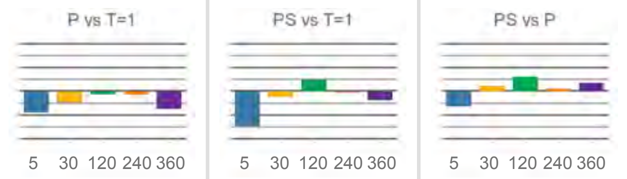

Locus		5m	30m	120m	240m	360m
**PGN_0034**	P vs T=1	0.333	0.011	-0.780	-0.839	-0.910
	PS vs T=1	0.672	0.283	-0.091	-0.179	-0.472
	PS vs P	0.352	0.275	0.659	0.630	0.418

DNA polymerase III alpha subunit

DNA metabolism

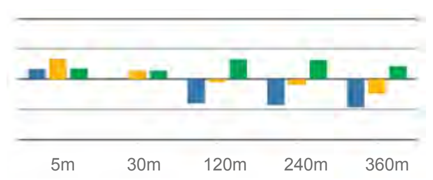

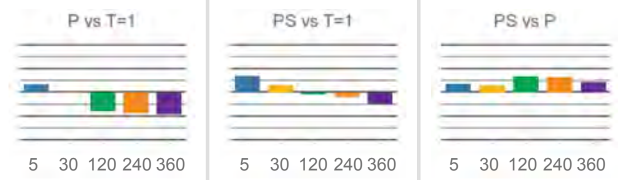

Locus		5m	30m	120m	240m	360m
**PGN_0035** *rplS*	P vs T=1	-0.004	0.184	0.753	0.832	0.213
	PS vs T=1	-1.083	-0.686	-0.242	-0.469	-0.509
	PS vs P	-1.049	-0.830	-0.926	-1.226	-0.702

50S ribosomal protein L19

protein synthesis

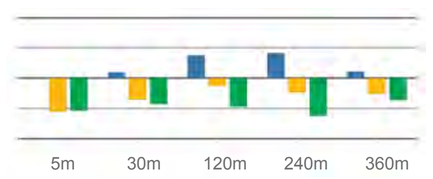

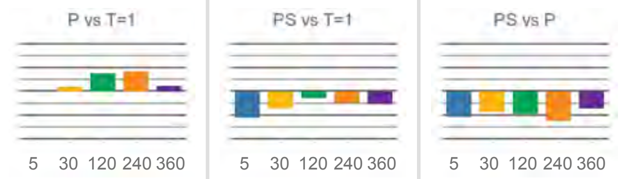

log$_2$ (Fold Change)

Legend: P vs T=1 · PS vs T=1 · PS vs P

Locus		5m	30m	120m	240m	360m
**PGN_0036**	P vs T=1	-0.935	-1.186	-1.069	-0.607	0.241
	PS vs T=1	-1.570	-2.122	-1.501	-0.980	-0.527
	PS vs P	-0.572	-0.897	-0.457	-0.215	-0.476

hypothetical protein

hypothetical proteins

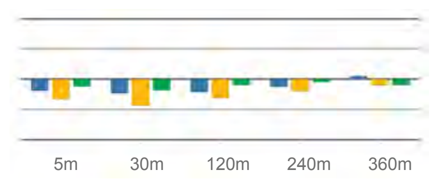

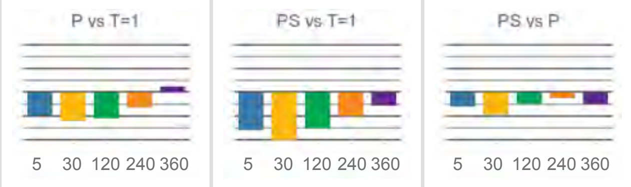

Locus		5m	30m	120m	240m	360m
**PGN_0037**	P vs T=1	-0.632	-1.113	-0.269	0.290	1.059
	PS vs T=1	-1.226	-1.344	-0.973	-0.117	0.298
	PS vs P	-0.525	-0.457	-0.517	-0.004	-0.206

conserved hypothetical protein

hypothetical proteins-Conserved

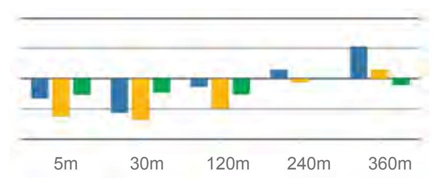

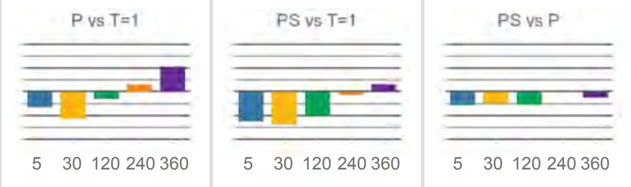

Locus		5m	30m	120m	240m	360m
**PGN_0038**	P vs T=1	0.120	0.546	1.253	1.419	0.851
	PS vs T=1	0.004	0.356	0.683	1.170	1.144
	PS vs P	-0.146	-0.189	-0.527	-0.201	0.293

serine hydroxymethyltransferase

central intermediary metabolism

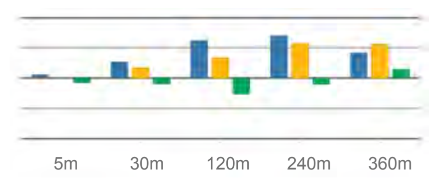

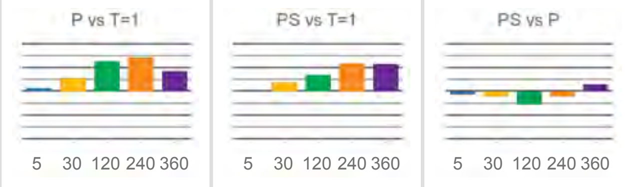

Locus		5m	30m	120m	240m	360m
**PGN_0039**	P vs T=1	0.507	0.747	0.921	0.718	0.786
	PS vs T=1	0.924	0.793	0.379	0.150	0.381
	PS vs P	0.403	0.060	-0.501	-0.548	-0.383

beta-hexosaminidase

cell envelope

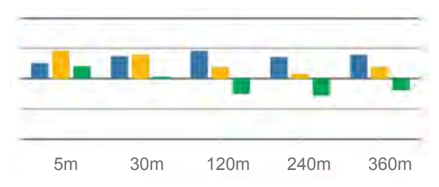

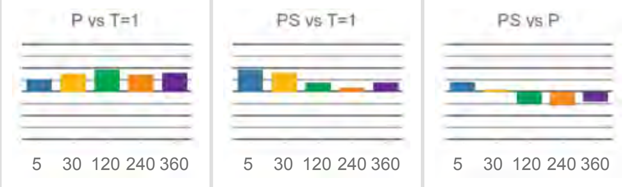

Locus		5m	30m	120m	240m	360m
**PGN_0040**	P vs T=1	-0.063	0.271	0.631	0.411	0.943
	PS vs T=1	0.704	0.901	1.032	0.854	0.662
	PS vs P	0.569	0.525	0.429	0.303	-0.147

hypothetical protein

hypothetical proteins

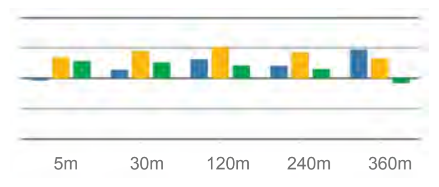

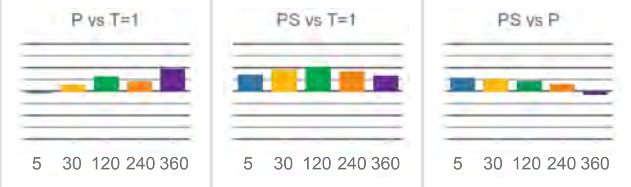

Locus		5m	30m	120m	240m	360m
**PGN_0041** *htpG*	P vs T=1	2.230	2.692	2.793	2.663	2.119
	PS vs T=1	0.367	0.555	0.460	0.624	0.719
	PS vs P	-1.762	-1.989	-2.212	-1.930	-1.354

heat shock protein 90

protein fate

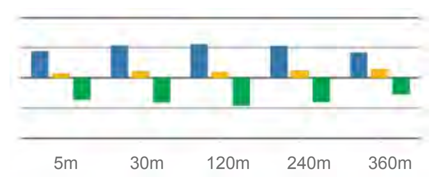

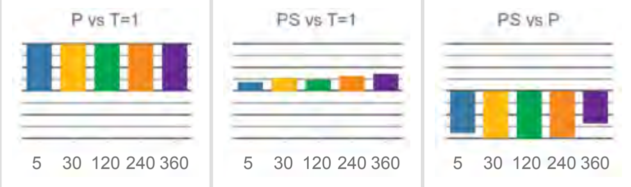

Locus		5m	30m	120m	240m	360m
**PGN_0042**	P vs T=1	0.501	1.072	1.400	1.549	1.231
	PS vs T=1	-0.019	0.540	2.026	2.525	2.427
	PS vs P	-0.555	-0.520	0.631	0.982	1.180

probable phosphatidate cytidylyltransferase

regulatory functions

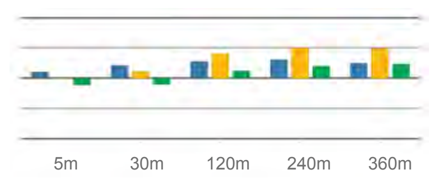

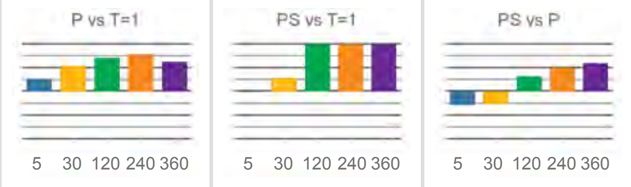

PgSg Time Course

Legend: P vs T=1 | PS vs T=1 | PS vs P

log$_2$ (Fold Change)

Locus		5m	30m	120m	240m	360m
**PGN_0043**	P vs T=1	0.816	1.601	1.826	1.806	1.407
	PS vs T=1	0.153	2.391	3.769	3.969	3.590
	PS vs P	-0.753	0.772	1.916	2.116	2.122
	putative transmembrane AAA-metalloprotease FtsH					
	cellular processes					
**PGN_0044**	P vs T=1	-0.758	-1.480	-2.259	-2.197	-1.558
	PS vs T=1	-1.063	-1.123	-1.540	-1.457	-1.742
	PS vs P	-0.243	0.342	0.612	0.642	-0.177
	GTP-binding protein					
	hypothetical proteins-Conserved					
**PGN_0045**	P vs T=1	1.470	0.900	0.219	0.292	1.696
	PS vs T=1	0.697	0.927	0.644	1.156	0.682
	PS vs P	-0.455	0.096	0.150	0.563	-0.555
	conserved hypothetical protein					
	hypothetical proteins-Conserved					
**PGN_0046**	P vs T=1	1.233	0.958	1.360	2.547	3.104
	PS vs T=1	0.776	0.369	1.629	1.873	1.920
	PS vs P	-0.356	-0.573	0.196	-0.100	-0.647
	hypothetical protein					
	hypothetical proteins					
**PGN_0047**	P vs T=1	-0.136	-0.937	0.328	0.638	2.569
	PS vs T=1	-1.025	-0.085	0.028	0.159	1.196
	PS vs P	-0.735	0.142	-0.116	-0.227	-0.381
	conserved hypothetical protein					
	hypothetical proteins-Conserved					
**PGN_0048**	P vs T=1	0.609	0.178	1.168	1.715	2.716
	PS vs T=1	-0.121	0.822	1.615	1.719	2.036
	PS vs P	-0.701	0.373	0.475	0.203	-0.380
	conserved hypothetical protein					
	hypothetical proteins-Conserved					
**PGN_0049**	P vs T=1	1.098	1.081	1.699	2.168	3.295
	PS vs T=1	0.519	1.427	2.239	2.158	2.015
	PS vs P	-0.581	0.271	0.549	0.111	-1.056
	probable anti-restriction protein					
	anti-restriction					

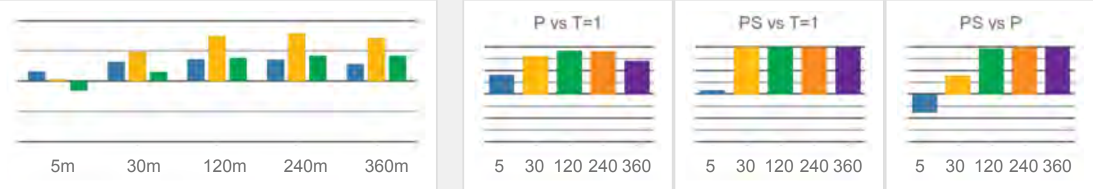

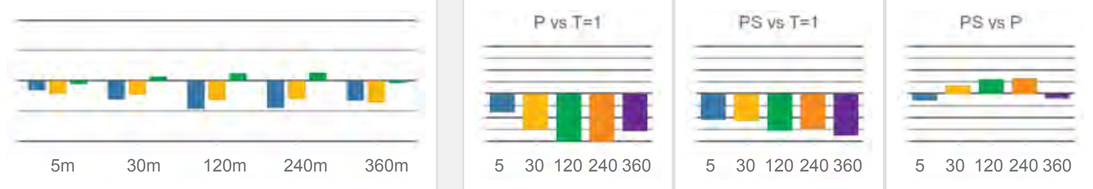

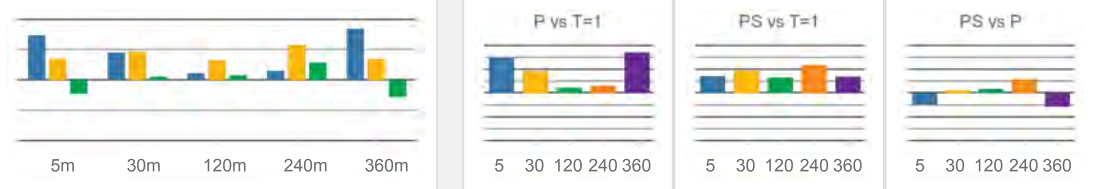

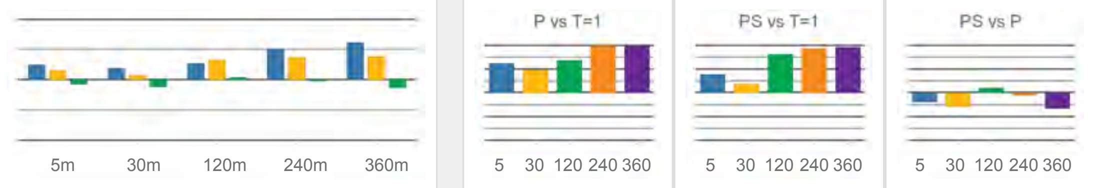

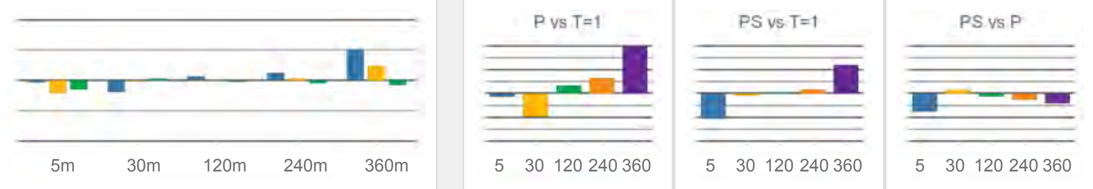

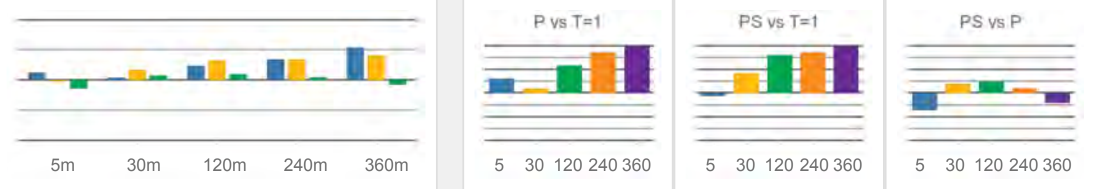

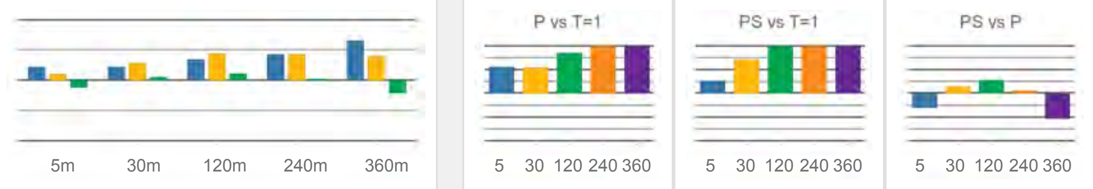

PgSg Time Course

log$_2$ (Fold Change)

Locus		5m	30m	120m	240m	360m
**PGN_0050**	P vs T=1	0.547	0.566	1.196	1.772	2.239
	PS vs T=1	-0.063	0.526	1.534	1.334	1.342
	PS vs P	-0.621	-0.081	0.353	-0.324	-0.784
	conserved hypothetical protein					
	hypothetical proteins-Conserved					
**PGN_0051**	P vs T=1	0.548	0.527	0.977	1.713	1.957
	PS vs T=1	-0.216	0.320	0.995	0.844	1.139
	PS vs P	-0.732	-0.234	0.038	-0.553	-0.588
	conserved hypothetical protein					
	hypothetical proteins-Conserved					
**PGN_0052**	P vs T=1	0.130	0.257	1.423	1.843	1.875
	PS vs T=1	-0.521	-0.103	0.682	1.105	1.439
	PS vs P	-0.685	-0.415	-0.578	-0.483	-0.292
	hypothetical protein					
	hypothetical proteins					
**PGN_0053**	P vs T=1	2.026	1.595	1.436	1.119	0.697
	PS vs T=1	-0.578	-0.182	0.006	-0.365	-0.739
	PS vs P	-2.258	-1.543	-1.261	-1.358	-1.380
	conserved hypothetical protein					
	hypothetical proteins-Conserved					
**PGN_0054**	P vs T=1	1.733	2.139	2.667	2.601	1.995
	PS vs T=1	-0.915	-0.335	0.169	0.047	-0.179
	PS vs P	-2.501	-2.291	-2.315	-2.382	-2.090
	hypothetical protein					
	hypothetical proteins					
**PGN_0055**	P vs T=1	1.426	1.543	1.690	1.467	1.074
	PS vs T=1	-0.628	-0.331	0.143	0.145	0.095
	PS vs P	-1.977	-1.790	-1.471	-1.253	-0.950
	probable lysozyme					
	cellular processes					
**PGN_0056**	P vs T=1	1.614	2.164	2.928	3.027	2.753
	PS vs T=1	0.886	1.471	2.424	2.421	2.629
	PS vs P	-0.739	-0.648	-0.409	-0.506	-0.080
	probable conserved protein found in conjugate transposon					
	other categories					

P vs T=1 | PS vs T=1 | PS vs P

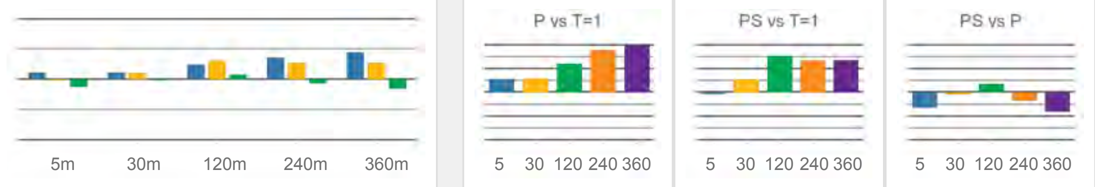

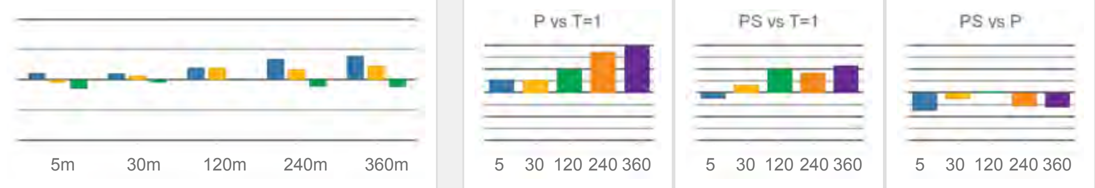

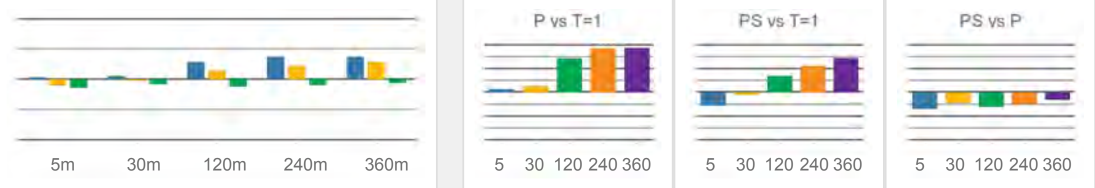

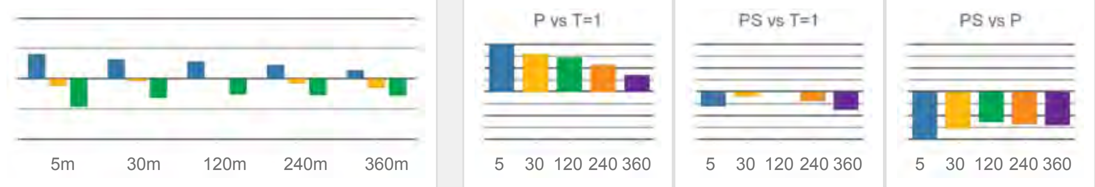

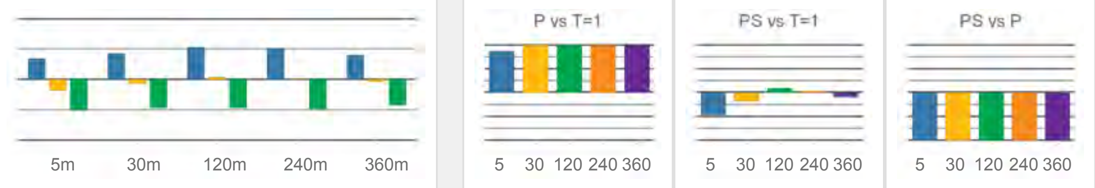

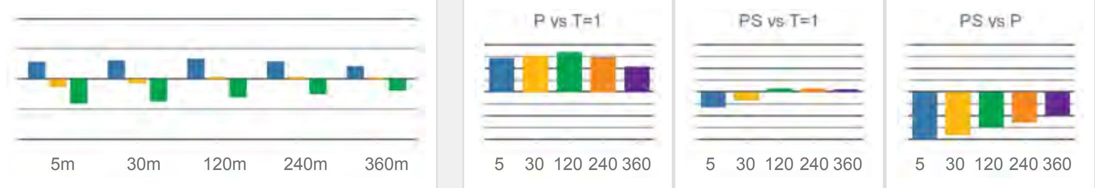

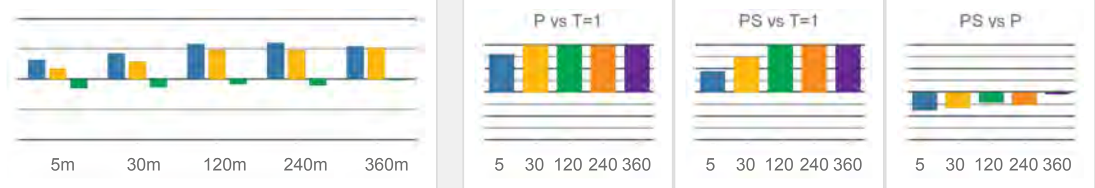

PgSg Time Course

log$_2$ (Fold Change)

Legend: P vs T=1 · PS vs T=1 · PS vs P

Locus		5m	30m	120m	240m	360m
**PGN_0057** *traP*	P vs T=1	0.746	1.290	1.758	1.828	1.636
	PS vs T=1	0.277	0.961	1.289	1.066	1.104
	PS vs P	-0.486	-0.305	-0.419	-0.701	-0.503
	probable conserved protein found in conjugate transposon TraP					
	other categories					
**PGN_0058**	P vs T=1	1.313	2.220	2.849	2.847	2.511
	PS vs T=1	0.422	0.984	1.645	1.626	1.946
	PS vs P	-0.911	-1.111	-1.049	-1.070	-0.498
	probable conserved protein found in conjugate transposon					
	other categories					
**PGN_0059** *traN*	P vs T=1	0.872	1.554	2.311	2.432	2.093
	PS vs T=1	0.292	0.904	1.490	1.655	1.898
	PS vs P	-0.608	-0.623	-0.757	-0.709	-0.172
	conserved protein found in conjugate transposon TraN					
	other categories					
**PGN_0060** *traM*	P vs T=1	0.809	1.580	2.731	3.167	3.013
	PS vs T=1	0.253	0.889	1.576	2.023	2.422
	PS vs P	-0.613	-0.675	-1.066	-1.029	-0.531
	conserved protein found in conjugate transposon TraM					
	other categories					
**PGN_0061**	P vs T=1	1.250	1.960	3.197	3.664	3.670
	PS vs T=1	0.368	1.202	2.098	3.013	3.544
	PS vs P	-0.940	-0.711	-0.914	-0.423	0.009
	hypothetical protein					
	hypothetical proteins					
**PGN_0062** *traK*	P vs T=1	1.181	1.676	2.613	3.067	3.146
	PS vs T=1	0.223	0.818	1.286	2.099	2.460
	PS vs P	-0.966	-0.811	-1.196	-0.794	-0.572
	putative conserved protein found in conjugate transposon TraK					
	other categories					
**PGN_0063** *traJ*	P vs T=1	1.167	1.898	2.851	3.128	3.016
	PS vs T=1	0.501	0.901	1.620	2.136	2.351
	PS vs P	-0.705	-0.942	-1.112	-0.861	-0.588
	conserved transmembrane protein found in conjugate transposon TraJ					
	other categories					

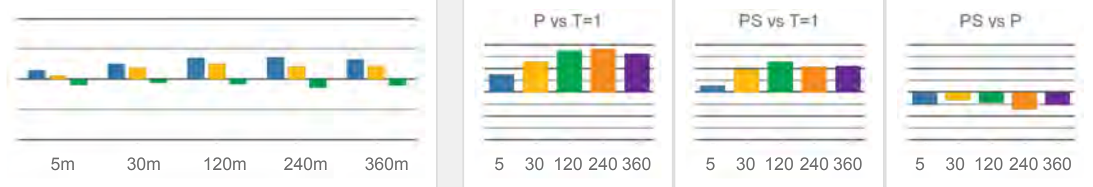

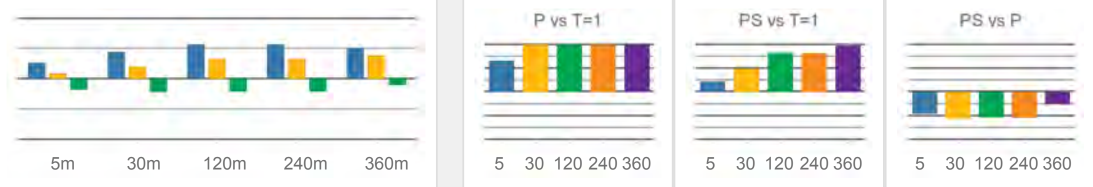

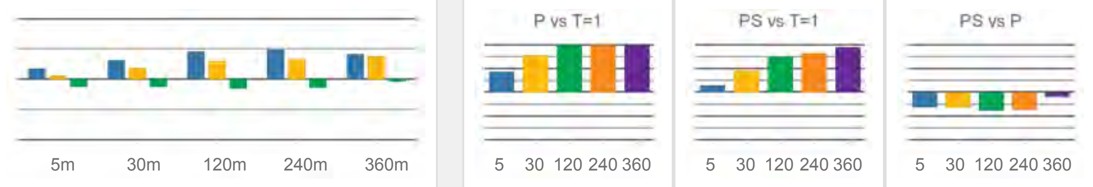

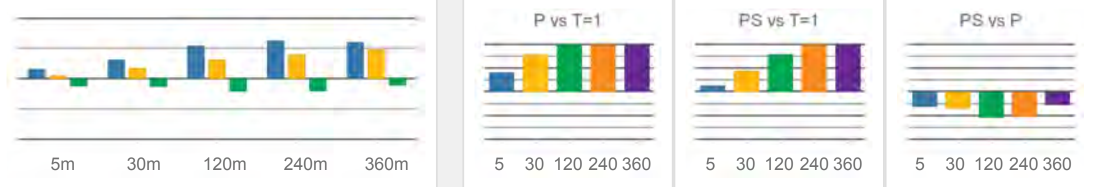

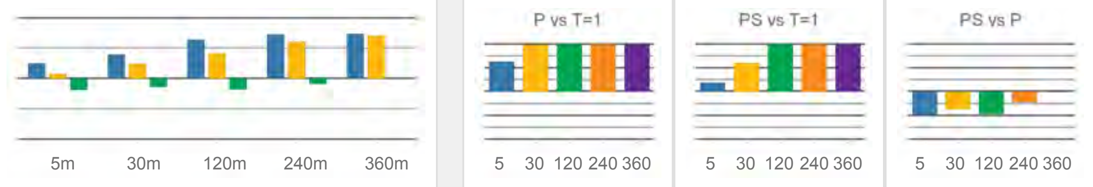

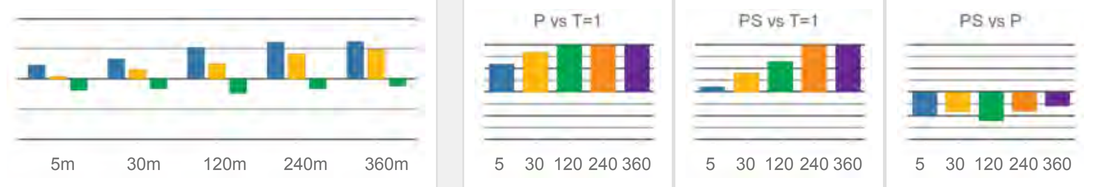

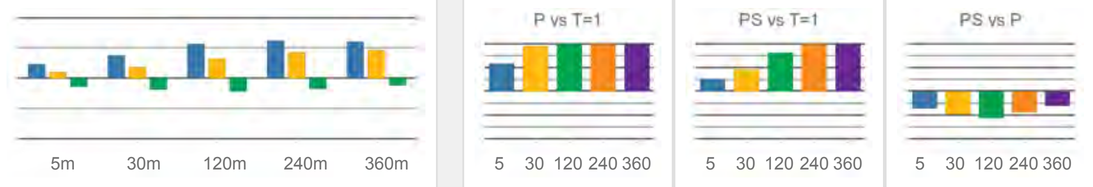

log$_2$ (Fold Change)

Legend: P vs T=1 · PS vs T=1 · PS vs P

Locus		5m	30m	120m	240m	360m
**PGN_0064**	P vs T=1	1.102	2.130	3.455	3.616	3.182
traI	PS vs T=1	0.685	1.309	2.293	2.627	2.848
	PS vs P	-0.533	-0.765	-0.975	-0.811	-0.257

putative conserved protein found in conjugate transposon TraI

other categories

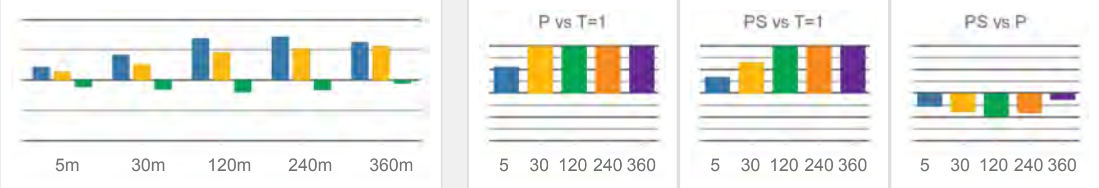

Locus		5m	30m	120m	240m	360m
**PGN_0065**	P vs T=1	0.493	1.145	1.851	2.081	1.961
traG	PS vs T=1	-0.273	0.150	0.615	0.705	0.914
	PS vs P	-0.797	-0.919	-1.108	-1.231	-0.961

conserved protein found in conjugate transposon TraG

other categories

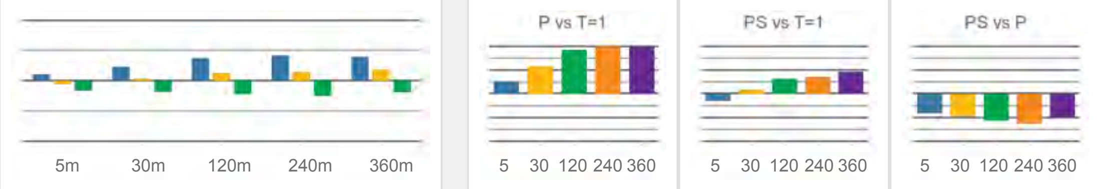

Locus		5m	30m	120m	240m	360m
**PGN_0066**	P vs T=1	0.854	1.683	2.598	2.972	2.558
traF	PS vs T=1	0.092	0.575	1.201	1.691	1.936
	PS vs P	-0.839	-0.962	-1.141	-0.978	-0.484

probable conserved transmembrane protein found in conjugate transposon TraF

other categories

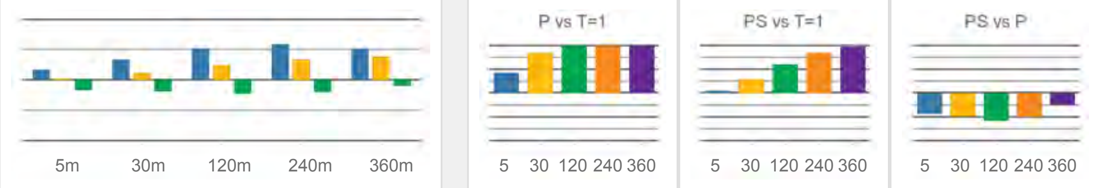

Locus		5m	30m	120m	240m	360m
**PGN_0067**	P vs T=1	1.262	1.928	2.769	2.991	2.698
traF	PS vs T=1	0.697	1.190	1.656	2.020	2.220
	PS vs P	-0.589	-0.694	-1.011	-0.849	-0.422

probable conserved transmembrane protein found in conjugate transposon TraE

other categories

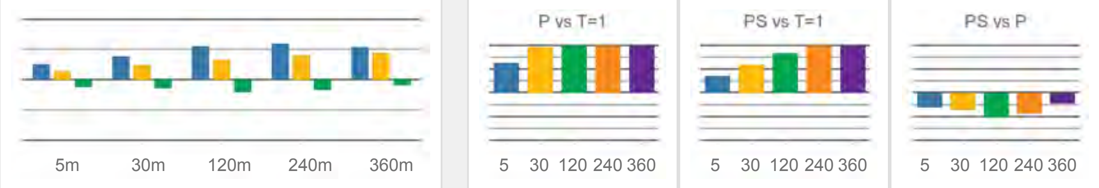

Locus		5m	30m	120m	240m	360m
**PGN_0068**	P vs T=1	-0.826	-0.337	-0.127	-0.236	-0.924
	PS vs T=1	-0.644	-0.205	-0.117	-0.356	-0.580
	PS vs P	0.107	0.131	0.033	-0.108	0.289

hypothetical protein

hypothetical proteins

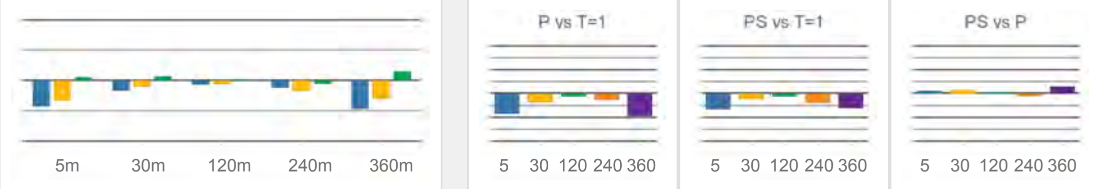

Locus		5m	30m	120m	240m	360m
**PGN_0069**	P vs T=1	0.942	1.676	2.848	3.187	3.299
traA	PS vs T=1	0.906	1.242	2.105	2.498	2.703
	PS vs P	-0.160	-0.440	-0.622	-0.541	-0.492

probable conserved protein found in conjugate transposon TraA

other categories

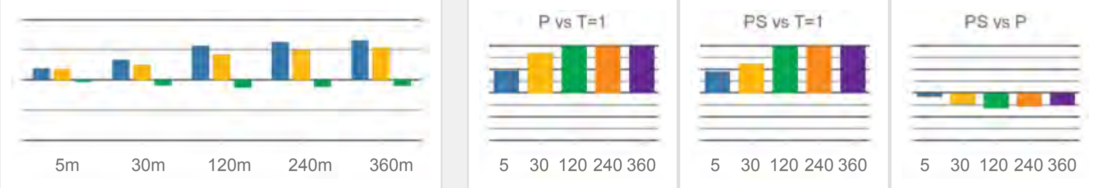

Locus		5m	30m	120m	240m	360m
**PGN_0070**	P vs T=1	0.946	1.692	2.376	2.698	2.626
	PS vs T=1	0.889	1.079	1.754	2.005	1.888
	PS vs P	-0.128	-0.575	-0.532	-0.544	-0.646

hypothetical protein

hypothetical proteins

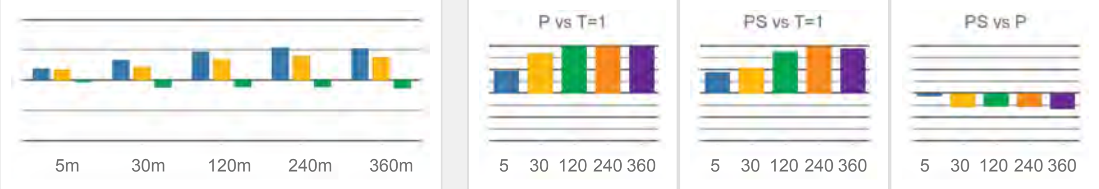

PgSg Time Course

Hackett Laboratory UW

log$_2$ (Fold Change)

Legend: P vs T=1 | PS vs T=1 | PS vs P

Locus		5m	30m	120m	240m	360m
**PGN_0071**	P vs T=1	1.545	2.220	3.106	3.239	3.157
	PS vs T=1	1.343	1.779	2.544	2.630	2.573
	PS vs P	-0.252	-0.414	-0.459	-0.483	-0.503

hypothetical protein

hypothetical proteins

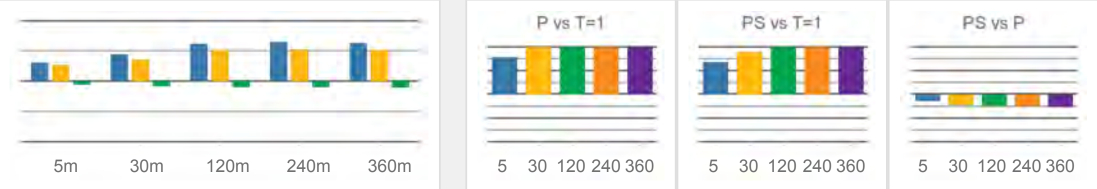

Locus		5m	30m	120m	240m	360m
**PGN_0072**	P vs T=1	0.335	1.288	2.199	2.350	2.405
	PS vs T=1	0.134	0.572	1.589	1.719	1.691
	PS vs P	-0.308	-0.682	-0.509	-0.519	-0.634

hypothetical protein

hypothetical proteins

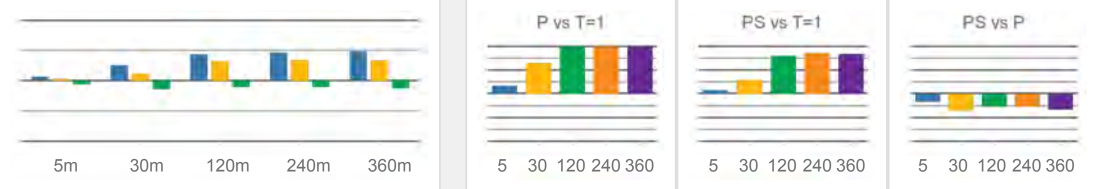

Locus		5m	30m	120m	240m	360m
**PGN_0073** *traA*	P vs T=1	2.409	3.110	4.007	4.329	4.492
	PS vs T=1	1.940	2.344	3.196	3.651	3.562
	PS vs P	-0.500	-0.711	-0.698	-0.538	-0.817

putative conserved protein found in conjugate transposon TraA

other categories

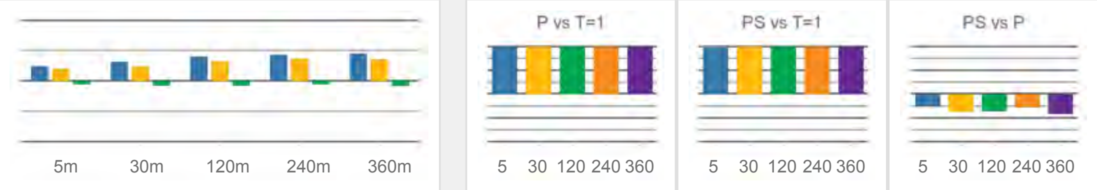

Locus		5m	30m	120m	240m	360m
**PGN_0074**	P vs T=1	0.855	0.650	0.515	0.791	1.571
	PS vs T=1	-0.379	-0.334	-0.027	-0.159	0.277
	PS vs P	-1.110	-0.888	-0.510	-0.812	-1.091

conserved hypothetical protein

hypothetical proteins-Conserved

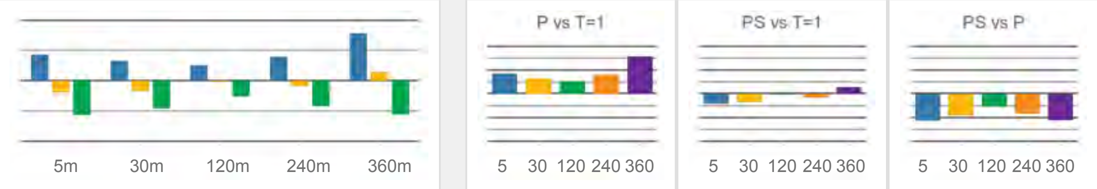

Locus		5m	30m	120m	240m	360m
**PGN_0075**	P vs T=1	0.307	0.007	-0.146	0.614	1.686
	PS vs T=1	-0.433	-0.569	0.212	0.333	0.536
	PS vs P	-0.705	-0.572	0.283	-0.217	-1.044

conserved hypothetical protein

hypothetical proteins-Conserved

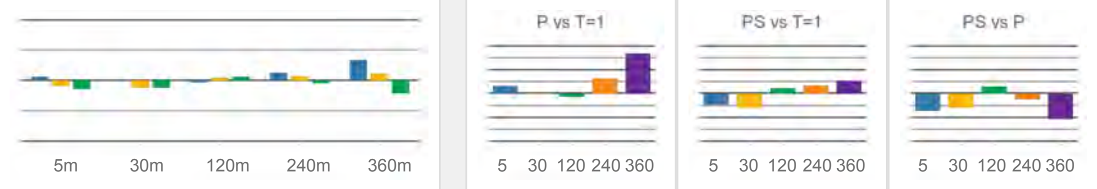

Locus		5m	30m	120m	240m	360m
**PGN_0076**	P vs T=1	0.244	0.145	0.463	1.118	1.814
	PS vs T=1	-0.116	-0.406	-0.141	0.003	0.327
	PS vs P	-0.351	-0.549	-0.592	-1.033	-1.411

putative mobilization protein TraG family

other categories

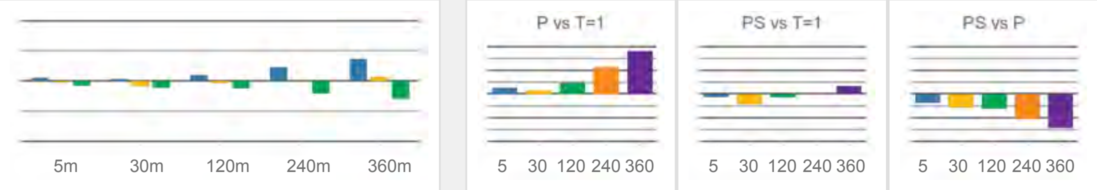

Locus		5m	30m	120m	240m	360m
**PGN_0077**	P vs T=1	-0.075	-0.864	-1.411	-1.631	-1.613
	PS vs T=1	-0.904	-1.408	-1.601	-1.853	-2.025
	PS vs P	-0.681	-0.517	-0.234	-0.296	-0.454

hypothetical protein

hypothetical proteins

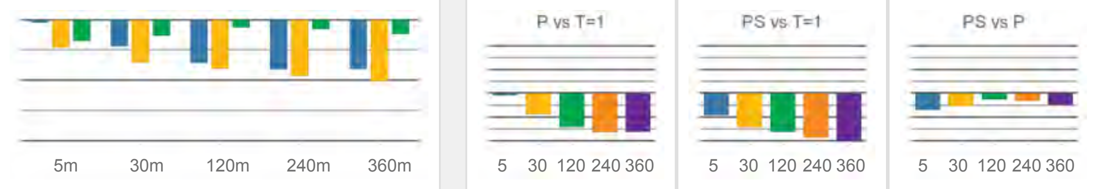

PgSg Time Course

log2 (Fold Change)

Legend: P vs T=1 | PS vs T=1 | PS vs P

Locus		5m	30m	120m	240m	360m
PGN_0078	P vs T=1	0.272	-0.817	-1.401	-1.403	-1.713
	PS vs T=1	-0.736	-1.058	-1.185	-1.359	-1.555
	PS vs P	-0.869	-0.249	0.148	-0.007	0.087
	hypothetical protein					
	hypothetical proteins					
PGN_0079	P vs T=1	0.447	-0.130	-0.457	-0.536	-0.732
	PS vs T=1	-0.191	-0.234	-0.298	-0.482	-0.586
	PS vs P	-0.571	-0.106	0.132	0.030	0.116
	conserved hypothetical protein with DUF1016 domain					
	hypothetical proteins-Conserved					
PGN_0080	P vs T=1	-0.501	-0.563	-0.238	-0.178	-0.641
	PS vs T=1	-0.712	-0.342	-0.052	-0.088	-0.021
	PS vs P	-0.222	0.192	0.192	0.100	0.587
	probable tetracycline resistance element mobilization regulatory protein RteC					
	mobile and extrachromosomal element functions					
PGN_0081	P vs T=1	0.126	-0.429	-0.970	-1.094	-1.095
	PS vs T=1	-0.247	-0.739	-0.924	-1.129	-1.008
	PS vs P	-0.338	-0.300	0.032	-0.053	0.076
	putative Na driven multidrug efflux pump					
	transport and binding proteins					
PGN_0082	P vs T=1	0.662	-0.801	-1.707	-1.492	-1.207
	PS vs T=1	0.224	-0.621	-0.975	-1.092	-1.211
	PS vs P	-0.351	0.162	0.652	0.353	-0.016
	probable transcriptional regulator AraC family					
	regulatory functions					
PGN_0083	P vs T=1	0.915	1.835	3.060	3.378	3.133
	PS vs T=1	0.686	1.195	2.015	2.334	2.507
	PS vs P	-0.338	-0.605	-0.895	-0.880	-0.538
	conserved hypothetical protein					
	hypothetical proteins-Conserved					
PGN_0084	P vs T=1	0.977	1.750	2.848	3.335	3.080
	PS vs T=1	0.432	0.893	1.566	2.096	2.292
	PS vs P	-0.604	-0.823	-1.173	-1.099	-0.716
	DNA topoisomerase I					
	DNA metabolism					

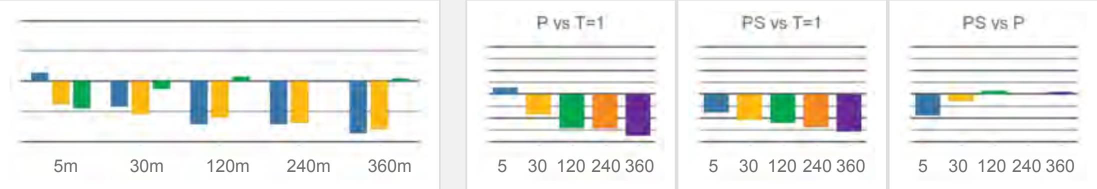

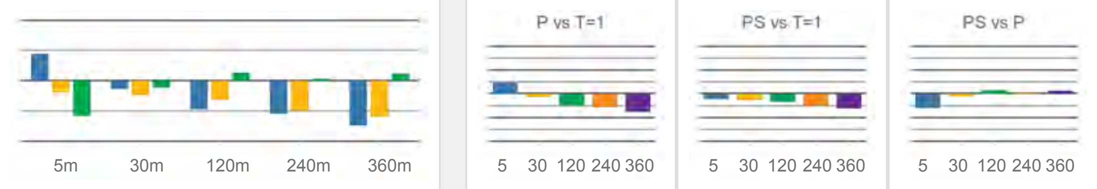

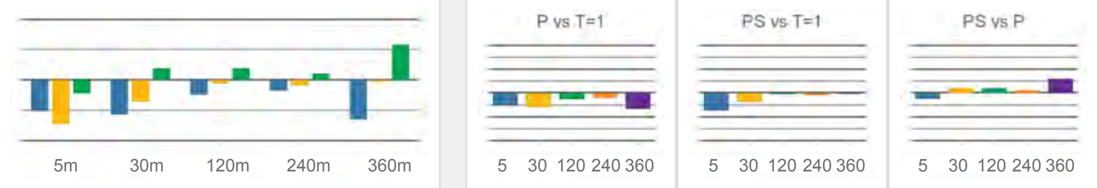

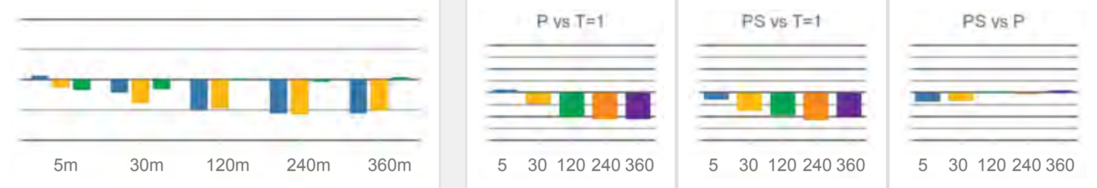

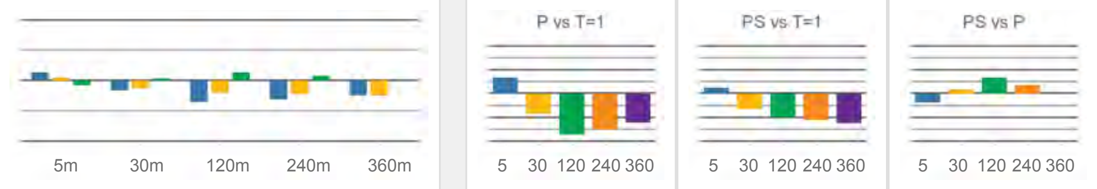

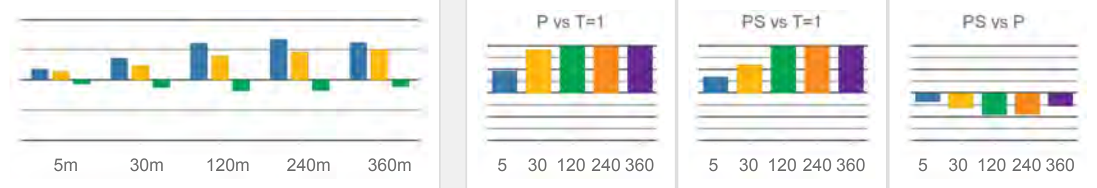

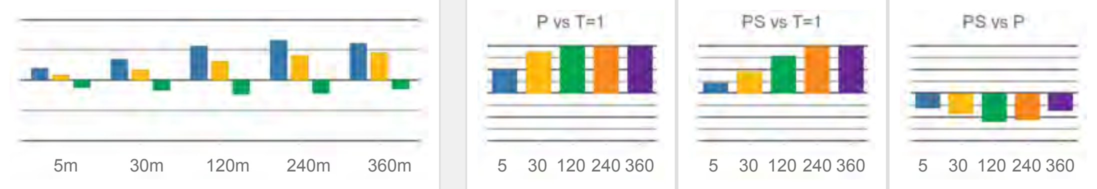

PgSg Time Course

Hackett Laboratory UW

log$_2$ (Fold Change)

Locus		5m	30m	120m	240m	360m
**PGN_0085**	P vs T=1	0.563	0.956	1.685	2.578	2.717
	PS vs T=1	-0.017	0.282	0.609	1.165	1.607
	PS vs P	-0.611	-0.618	-0.912	-1.005	-0.845

hypothetical protein

hypothetical proteins

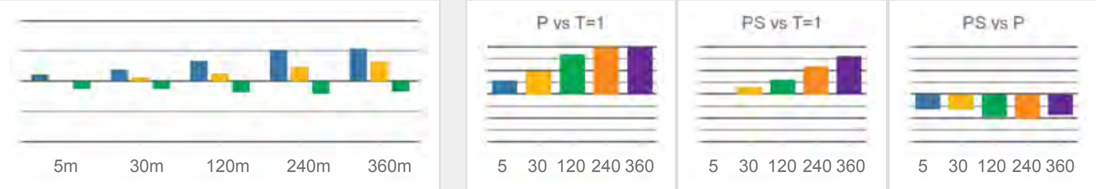

Locus		5m	30m	120m	240m	360m
**PGN_0086**	P vs T=1	0.768	1.141	1.796	2.351	2.781
	PS vs T=1	0.366	0.494	0.704	1.143	1.333
	PS vs P	-0.407	-0.638	-1.067	-1.163	-1.411

putative DNA methylase

DNA metabolism

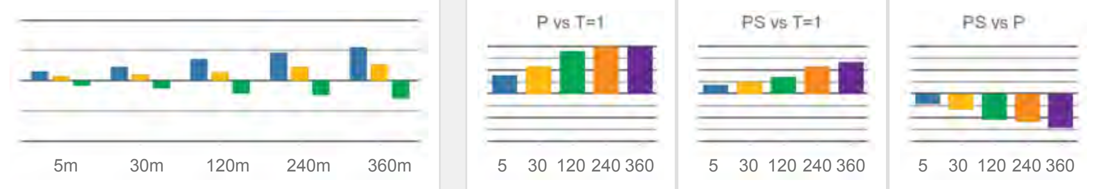

Locus		5m	30m	120m	240m	360m
**PGN_0087**	P vs T=1	-0.032	-0.564	-0.186	0.195	0.227
	PS vs T=1	-0.153	0.189	0.705	0.764	0.740
	PS vs P	-0.131	0.610	0.822	0.559	0.500

conserved hypothetical protein

hypothetical proteins-Conserved

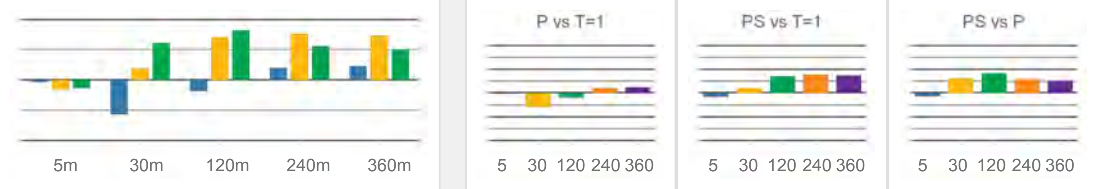

Locus		5m	30m	120m	240m	360m
**PGN_0088**	P vs T=1	0.138	-0.085	0.171	0.753	0.845
	PS vs T=1	-0.124	-0.050	0.645	0.840	0.713
	PS vs P	-0.269	-0.005	0.424	0.121	-0.113

putative transcriptional regulator

regulatory functions

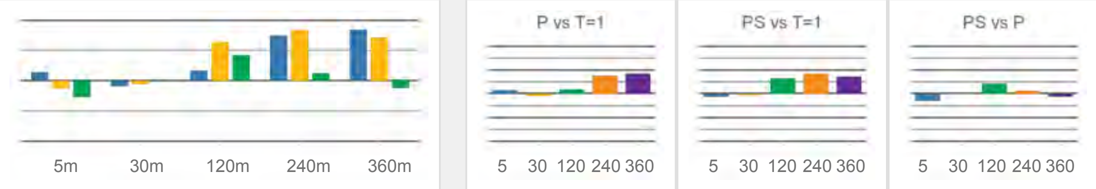

Locus		5m	30m	120m	240m	360m
**PGN_0089**	P vs T=1	-0.294	-0.532	0.105	0.999	1.241
	PS vs T=1	-0.632	-0.246	0.029	-0.049	0.084
	PS vs P	-0.350	0.200	-0.077	-0.862	-1.030

hypothetical protein

hypothetical proteins

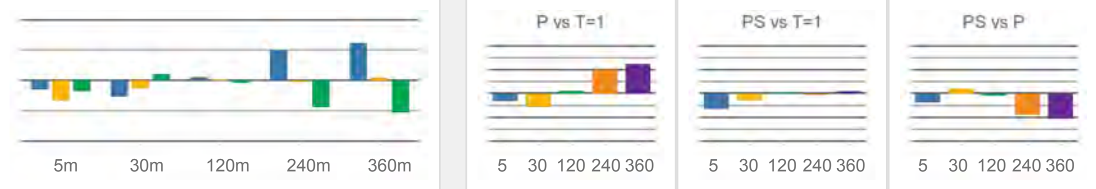

Locus		5m	30m	120m	240m	360m
**PGN_0090**	P vs T=1	0.486	0.596	1.119	1.738	1.932
	PS vs T=1	0.194	0.540	1.257	0.873	0.791
	PS vs P	-0.307	-0.072	0.184	-0.681	-0.992

hypothetical protein

hypothetical proteins

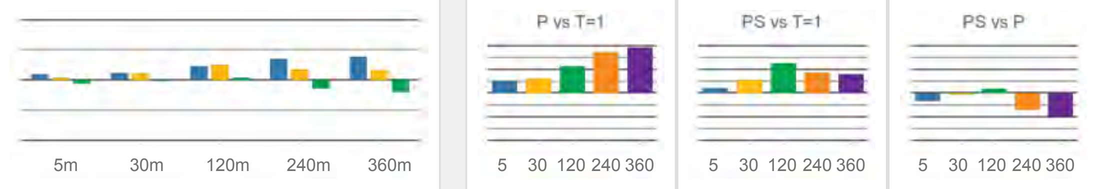

Locus		5m	30m	120m	240m	360m
**PGN_0091**	P vs T=1	1.207	1.507	2.116	2.324	2.074
	PS vs T=1	0.452	1.211	2.468	2.734	2.692
	PS vs P	-0.762	-0.293	0.379	0.446	0.627

hypothetical protein

hypothetical proteins

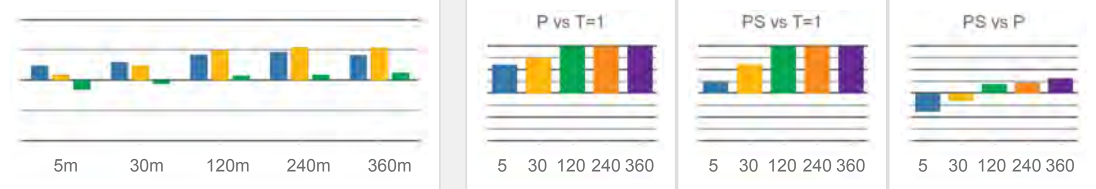

P vs T=1 | PS vs T=1 | PS vs P

Locus		log₂ (Fold Change) 5m	30m	120m	240m	360m
**PGN_0092**	P vs T=1	0.375	0.728	1.336	1.624	1.377
	PS vs T=1	-0.503	0.293	1.554	1.660	1.662
	PS vs P	-0.898	-0.430	0.251	0.091	0.300
	conserved hypothetical protein					
	hypothetical proteins-Conserved					

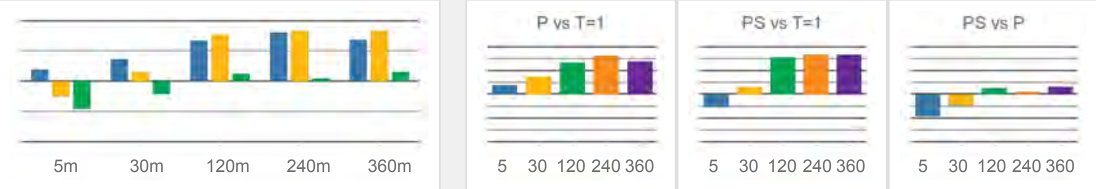

Locus		5m	30m	120m	240m	360m
**PGN_0093**	P vs T=1	0.400	0.681	1.162	1.362	1.090
	PS vs T=1	0.218	0.558	1.026	0.790	1.010
	PS vs P	-0.209	-0.120	-0.088	-0.495	-0.058
	conserved hypothetical protein					
	hypothetical proteins-Conserved					

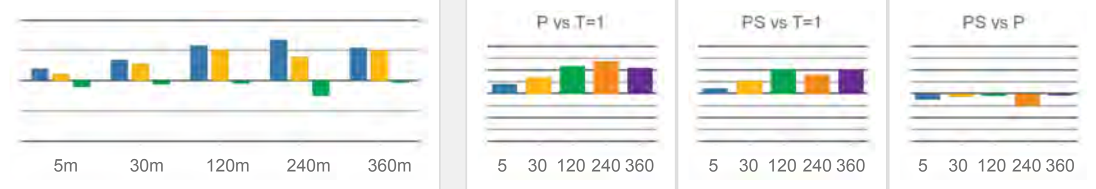

Locus		5m	30m	120m	240m	360m
**PGN_0094**	P vs T=1	0.493	0.318	0.229	0.348	0.369
	PS vs T=1	0.039	0.256	0.420	0.158	-0.103
	PS vs P	-0.415	-0.053	0.187	-0.171	-0.455
	putative bacteriophage integrase					
	mobile and extrachromosomal element functions					

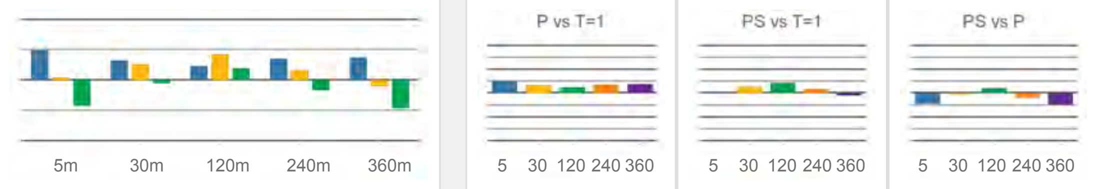

Locus		5m	30m	120m	240m	360m
**PGN_0095**	P vs T=1	0.349	0.064	-0.341	-0.486	-0.210
	PS vs T=1	0.353	0.274	0.086	-0.214	-0.371
	PS vs P	0.064	0.212	0.344	0.133	-0.164
	hypothetical protein					
	hypothetical proteins					

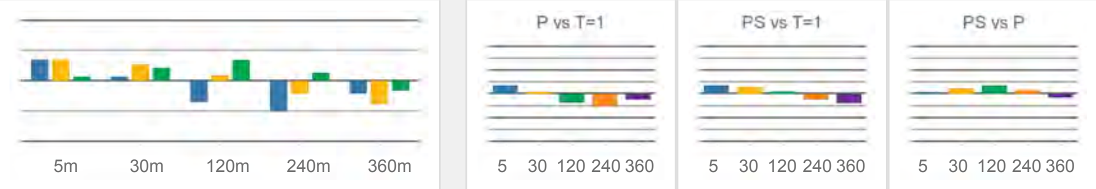

Locus		5m	30m	120m	240m	360m
**PGN_0096**	P vs T=1	0.323	0.046	-0.586	-0.932	-0.885
	PS vs T=1	0.266	0.099	-0.362	-0.678	-0.688
	PS vs P	-0.044	0.060	0.208	0.208	0.177
	aspartate kinase					
	amino acid biosynthesis					

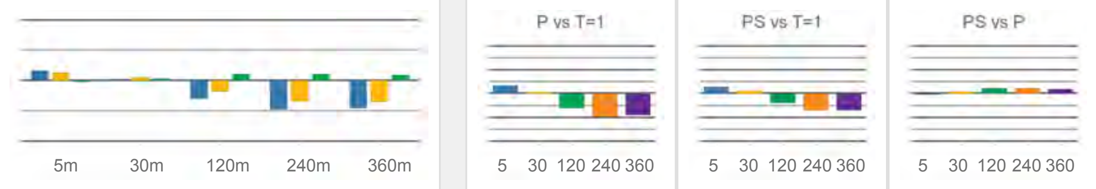

Locus		5m	30m	120m	240m	360m
**PGN_0097**	P vs T=1	0.997	0.843	0.255	-0.220	-0.593
	PS vs T=1	0.868	0.890	0.571	0.463	0.152
	PS vs P	-0.099	0.066	0.300	0.617	0.681
	putative cell-division ATP-binding protein					
	cellular processes					

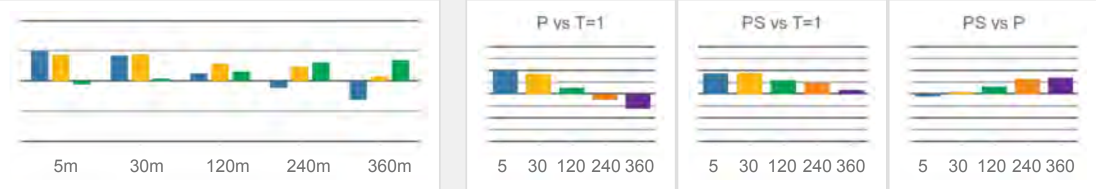

Locus		5m	30m	120m	240m	360m
**PGN_0098**	P vs T=1	3.920	2.666	2.001	2.975	3.595
	PS vs T=1	2.137	2.051	2.262	2.553	2.403
	PS vs P	-1.379	-0.480	0.019	-0.073	-0.726
	hypothetical protein					
	hypothetical proteins					

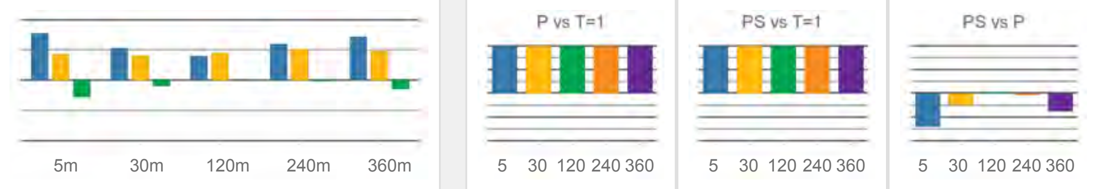

PgSg Time Course

log₂ (Fold Change)

Legend: P vs T=1 | PS vs T=1 | PS vs P

PGN_0099

Locus	5m	30m	120m	240m	360m
P vs T=1	0.167	0.165	0.082	-0.505	-1.396
PS vs T=1	-1.266	-1.143	-1.206	-1.282	-1.297
PS vs P	-1.265	-1.141	-1.148	-0.733	0.033

probable peptidase

protein fate

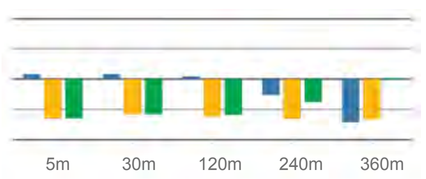

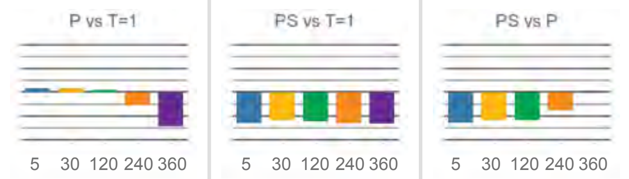

PGN_0100

Locus	5m	30m	120m	240m	360m
P vs T=1	-0.164	-0.334	-0.789	-0.965	-1.154
PS vs T=1	-0.837	-0.743	-1.008	-0.833	-0.907
PS vs P	-0.645	-0.389	-0.228	0.104	0.216

diaminopimelate decarboxylase

amino acid biosynthesis

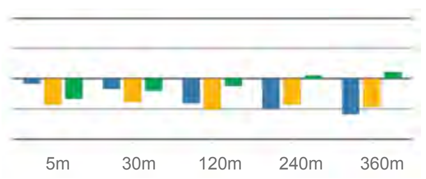

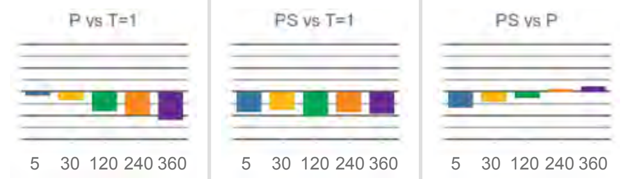

PGN_0101

MenA

Locus	5m	30m	120m	240m	360m
P vs T=1	0.338	0.152	0.080	0.174	0.317
PS vs T=1	-0.820	-0.283	-0.392	-0.427	-0.013
PS vs P	-1.111	-0.411	-0.456	-0.557	-0.295

putative 1,4-dihydroxy-2-naphthoate octaprenyltransferase

biosynthesis of cofactors, prosthetic groups, and carriers

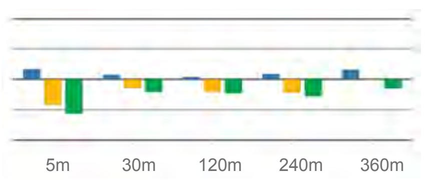

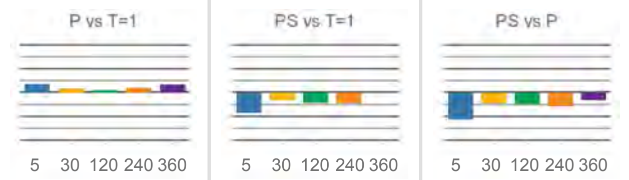

PGN_0102

Locus	5m	30m	120m	240m	360m
P vs T=1	0.155	0.302	0.618	0.607	0.392
PS vs T=1	-0.521	0.024	0.501	0.773	0.457
PS vs P	-0.685	-0.283	-0.095	0.173	0.039

conserved hypothetical protein

hypothetical proteins-Conserved

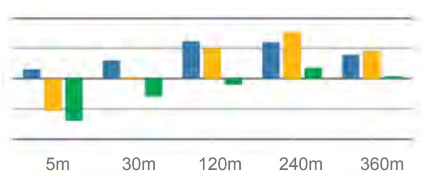

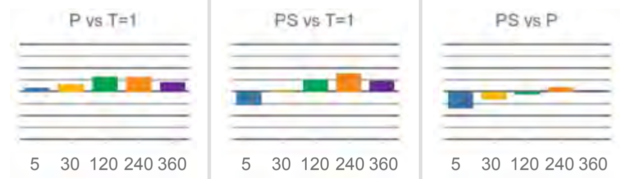

PGN_0103

Locus	5m	30m	120m	240m	360m
P vs T=1	1.133	1.845	2.558	2.555	2.114
PS vs T=1	1.036	1.621	2.243	2.345	2.123
PS vs P	-0.156	-0.213	-0.258	-0.164	0.013

putative TonB

transport and binding proteins

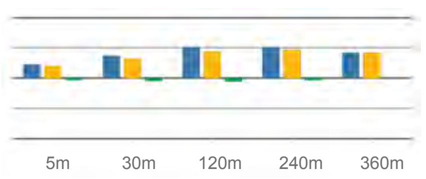

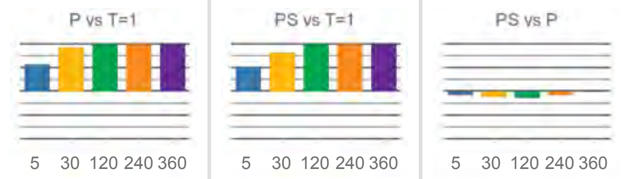

PGN_0104

Locus	5m	30m	120m	240m	360m
P vs T=1	0.329	0.266	0.322	0.498	0.158
PS vs T=1	0.135	-0.019	0.106	0.177	0.236
PS vs P	-0.195	-0.293	-0.226	-0.313	0.058

transposase in ISPg1

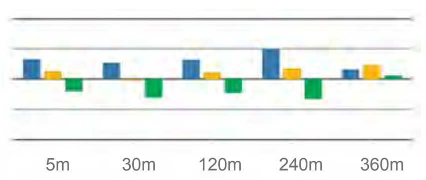

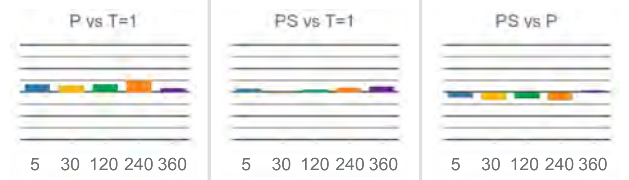

PGN_0105

Locus	5m	30m	120m	240m	360m
P vs T=1	-0.126	-0.008	0.510	1.340	2.611
PS vs T=1	0.545	0.439	0.049	1.121	0.635
PS vs P	0.392	0.187	-0.482	0.142	-1.143

conserved hypothetical protein

hypothetical proteins-Conserved

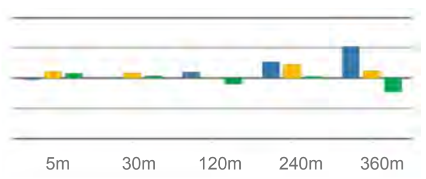

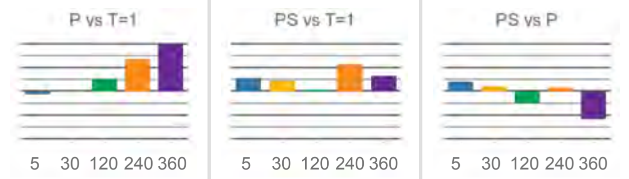

log2 (Fold Change)

Legend: P vs T=1 | PS vs T=1 | PS vs P

Locus		5m	30m	120m	240m	360m
**PGN_0106**	P vs T=1	0.428	-0.179	-0.185	0.309	1.357
	PS vs T=1	0.104	-0.030	0.581	0.327	0.786
	PS vs P	-0.282	0.046	0.597	-0.016	-0.441

partial transposase in ISPg3

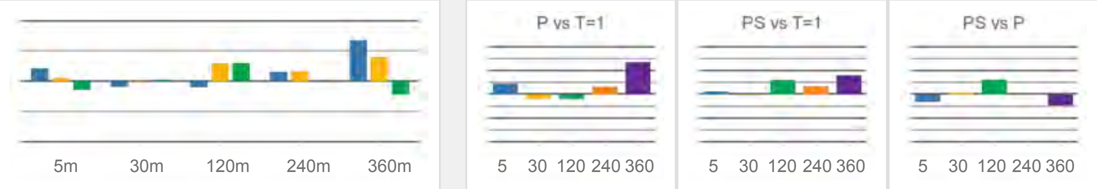

Locus		5m	30m	120m	240m	360m
**PGN_0107**	P vs T=1	0.183	-0.148	-0.159	-0.005	0.620
	PS vs T=1	-0.123	0.113	0.240	0.422	0.259
	PS vs P	-0.302	0.173	0.242	0.272	-0.306

partial transposase in ISPg3

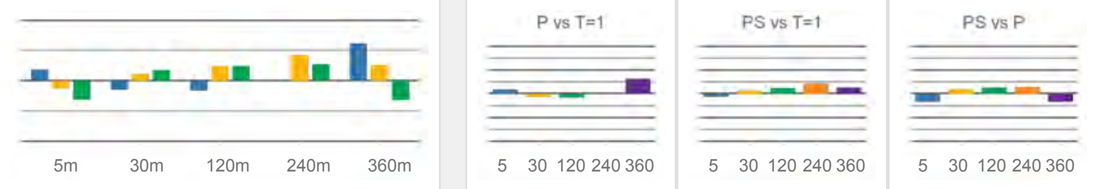

Locus		5m	30m	120m	240m	360m
**PGN_0108**	P vs T=1	-0.357	-0.431	-0.578	-0.346	-0.581
	PS vs T=1	-0.550	-0.847	-0.581	-0.467	-0.302
	PS vs P	-0.196	-0.422	-0.038	-0.131	0.247

partial transposase in ISPg1

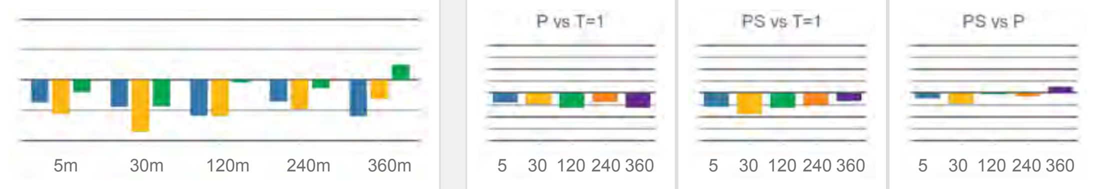

Locus		5m	30m	120m	240m	360m
**PGN_0109**	P vs T=1	-1.026	-1.379	-1.620	-1.422	-1.152
	PS vs T=1	-1.472	-1.614	-1.383	-1.220	-1.037
	PS vs P	-0.392	-0.251	0.134	0.157	0.127

conserved hypothetical protein

hypothetical proteins-Conserved

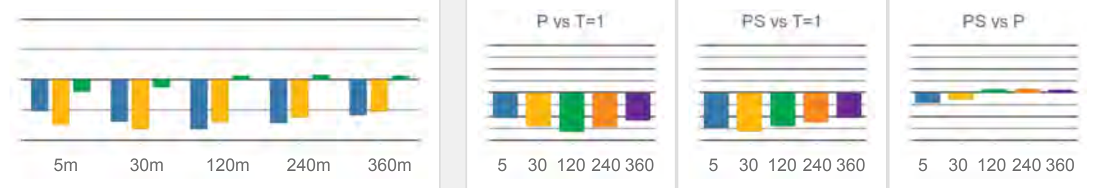

Locus		5m	30m	120m	240m	360m
**PGN_0110**	P vs T=1	0.160	0.067	-0.260	-0.975	-0.722
	PS vs T=1	-0.478	-0.605	-1.324	-1.242	-1.190
	PS vs P	-0.423	-0.441	-0.808	-0.414	-0.394

hypothetical protein

hypothetical proteins

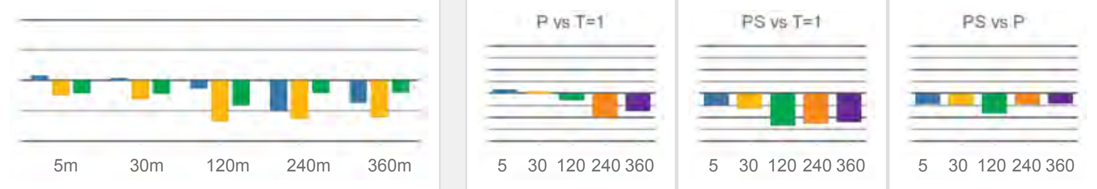

Locus		5m	30m	120m	240m	360m
**PGN_0111**	P vs T=1	0.687	-0.098	-0.106	0.254	0.395
	PS vs T=1	-0.763	-0.338	-0.210	0.504	-0.267
	PS vs P	-1.067	-0.258	-0.159	0.395	-0.380

partial transposase in ISPg6

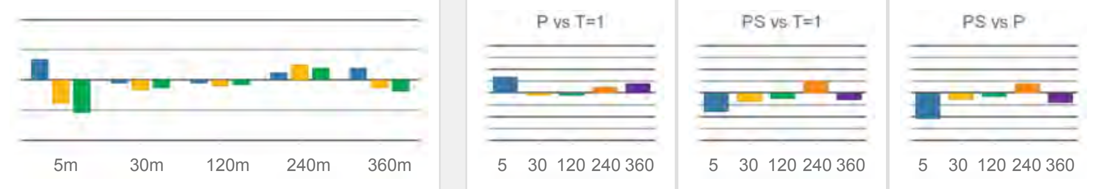

Locus		5m	30m	120m	240m	360m
**PGN_0112**	P vs T=1	0.147	0.423	0.669	0.677	0.623
	PS vs T=1	-0.280	-0.013	0.373	1.101	1.101
	PS vs P	-0.448	-0.411	-0.253	0.432	0.465

partial transposase Orf1 in ISPg5

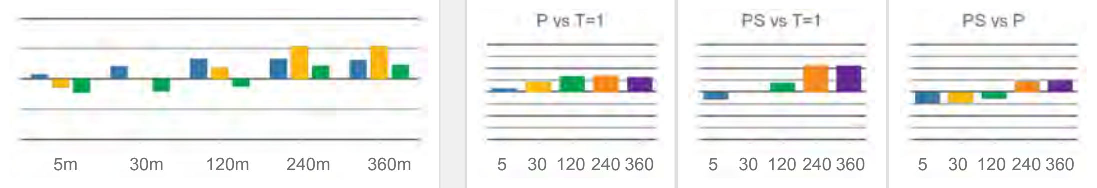

log$_2$ (Fold Change)

Locus		5m	30m	120m	240m	360m
**PGN_0113**	P vs T=1	0.063	-0.231	-0.235	-1.143	-0.099
	PS vs T=1	-0.626	-0.863	-0.749	-0.494	-0.032
	PS vs P	-0.523	-0.545	-0.434	-0.113	0.121
	hypothetical protein					
	hypothetical proteins					
**PGN_0114**	P vs T=1	-0.808	-0.524	-0.513	-0.519	-0.663
	PS vs T=1	-0.971	-0.553	-0.307	0.109	-0.112
	PS vs P	-0.182	-0.031	0.198	0.613	0.532
	Na translocating NADH-quinone reductase subunit A					
	energy metabolism					
**PGN_0115**	P vs T=1	-1.543	-1.419	-1.759	-1.878	-2.125
	PS vs T=1	-0.625	-1.073	-1.624	-1.435	-1.630
	PS vs P	0.854	0.324	0.077	0.364	0.404
	Na translocating NADH-quinone reductase subunit B					
	energy metabolism					
**PGN_0116**	P vs T=1	-0.192	0.079	-0.153	-0.796	-0.646
	PS vs T=1	0.354	0.115	-0.087	-0.212	-0.383
	PS vs P	0.530	0.048	0.070	0.474	0.227
	probable Na translocating NADH-quinone reductase subunit C					
	energy metabolism					
**PGN_0117**	P vs T=1	-0.967	-0.532	-0.498	-1.060	-1.418
	PS vs T=1	-0.633	-0.663	-0.729	-0.888	-1.138
	PS vs P	0.294	-0.113	-0.200	0.122	0.215
	putative Na translocating NADH-quinone reductase subunit D					
	transport and binding proteins					
**PGN_0118**	P vs T=1	-0.333	-0.095	-0.531	-1.109	-1.387
	PS vs T=1	0.133	0.101	-0.304	-0.677	-0.922
	PS vs P	0.457	0.215	0.224	0.344	0.390
	putative Na translocating NADH-quinone reductase subunit E					
	transport and binding proteins					
**PGN_0119**	P vs T=1	-0.093	-0.040	-0.233	-0.830	-1.260
	PS vs T=1	0.528	0.354	-0.156	-0.678	-1.053
	PS vs P	0.616	0.399	0.087	0.113	0.160
	Na translocating NADH-quinone reductase subunit F					
	energy metabolism					

P vs T=1 | PS vs T=1 | PS vs P

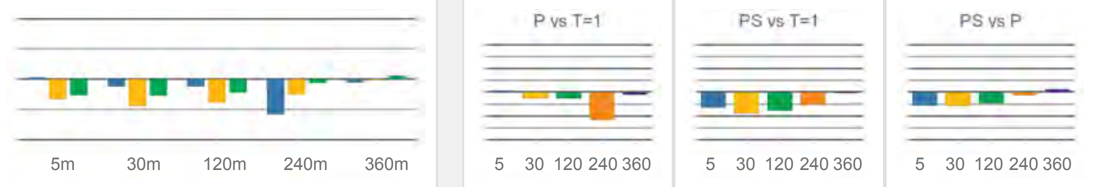

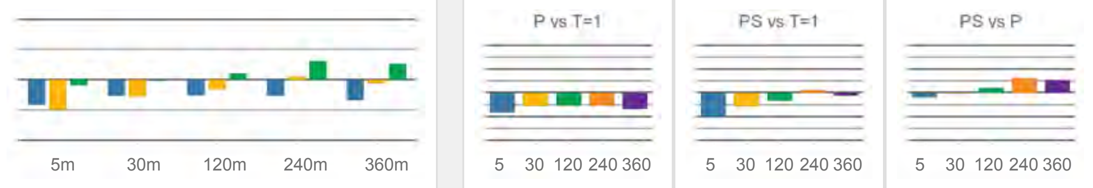

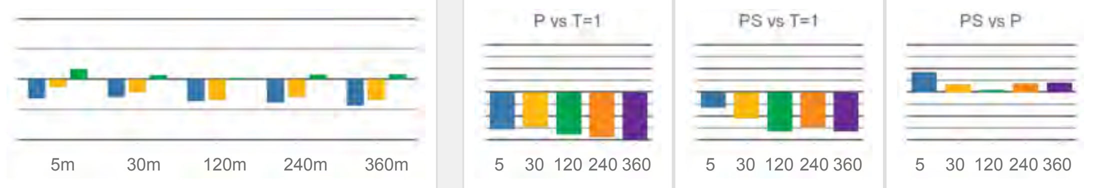

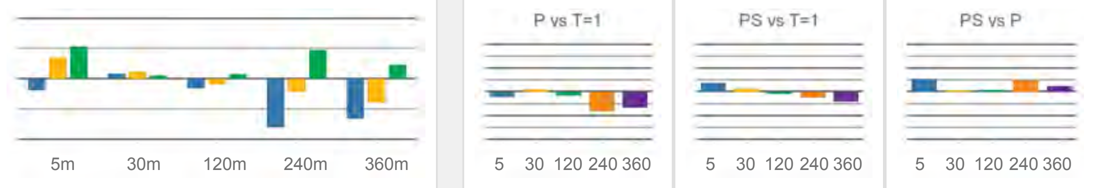

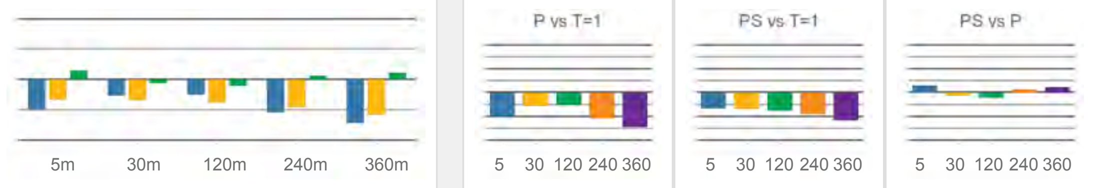

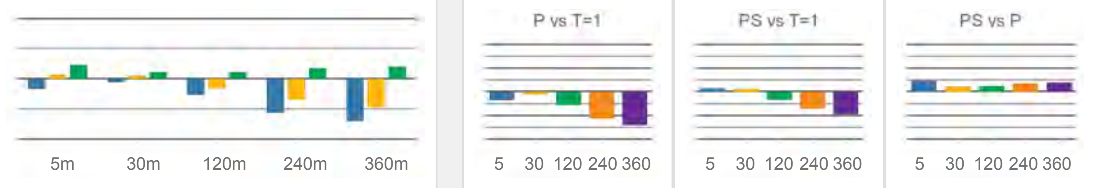

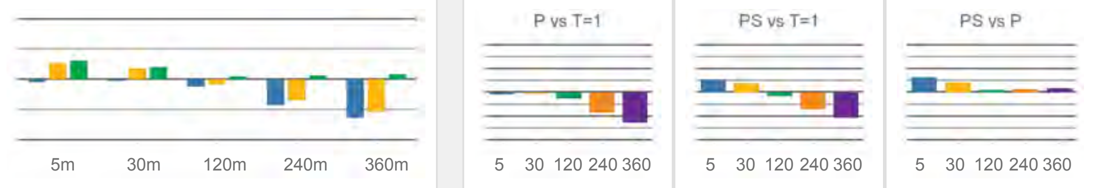

log$_2$ (Fold Change)

Legend: P vs T=1 | PS vs T=1 | PS vs P

Locus		5m	30m	120m	240m	360m
**PGN_0120**	P vs T=1	-0.235	-0.834	-1.612	-1.742	-2.253
	PS vs T=1	-0.850	-0.745	-0.417	-0.379	-0.670
	PS vs P	-0.545	0.096	1.114	1.264	1.456

conserved hypothetical protein

hypothetical proteins-Conserved

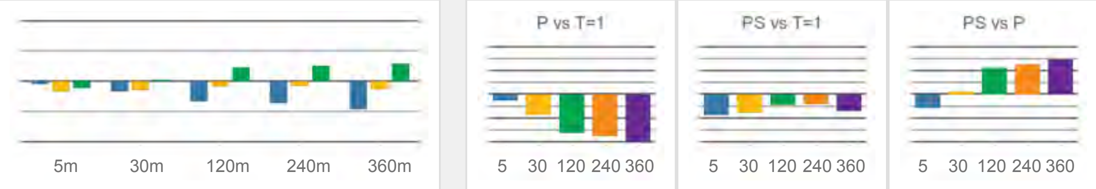

Locus		5m	30m	120m	240m	360m
**PGN_0121**	P vs T=1	-0.611	-0.712	-1.190	-1.530	-1.823
	PS vs T=1	-0.415	-0.418	-0.466	-0.537	-0.843
	PS vs P	0.196	0.291	0.696	0.937	0.930

conserved hypothetical protein

hypothetical proteins-Conserved

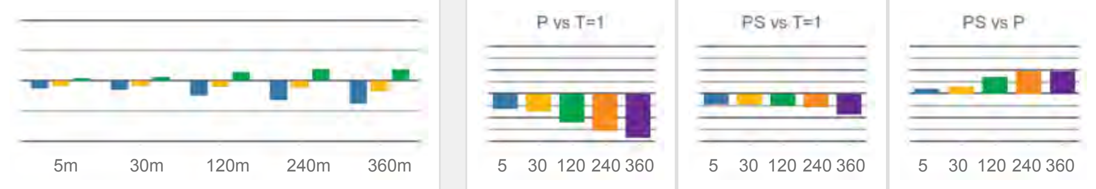

Locus		5m	30m	120m	240m	360m
**PGN_0122**	P vs T=1	0.660	0.418	-0.108	-0.930	-1.893
	PS vs T=1	0.869	1.043	0.588	-0.483	-1.287
	PS vs P	0.273	0.653	0.701	0.358	0.454

28 kDa outer membrane protein Omp28

cell envelope

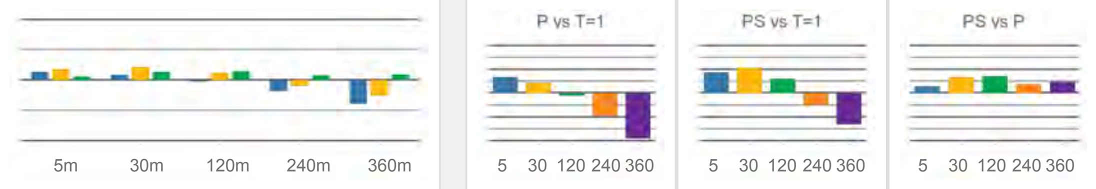

Locus		5m	30m	120m	240m	360m
**PGN_0123**	P vs T=1	0.743	0.672	0.586	0.266	-0.514
	PS vs T=1	0.189	0.497	0.409	-0.034	-0.097
	PS vs P	-0.487	-0.129	-0.136	-0.292	0.351

conserved hypothetical protein

hypothetical proteins-Conserved

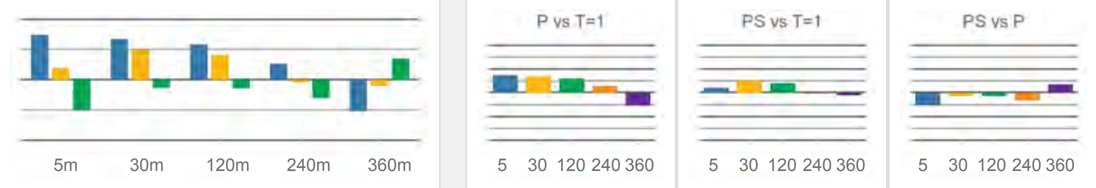

Locus		5m	30m	120m	240m	360m
**PGN_0124**	P vs T=1	1.329	1.243	1.259	0.768	-0.153
	PS vs T=1	1.153	1.360	1.217	0.622	0.534
	PS vs P	-0.088	0.165	0.045	-0.158	0.500

hypothetical protein

hypothetical proteins

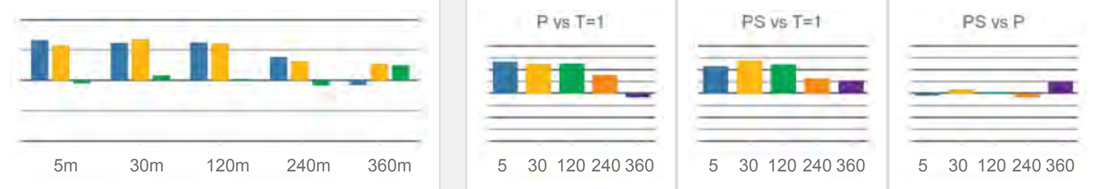

Locus		5m	30m	120m	240m	360m
**PGN_0125**	P vs T=1	-0.526	-0.394	-0.501	-0.849	-1.293
	PS vs T=1	-0.284	-0.354	-0.878	-1.278	-1.461
	PS vs P	0.237	0.044	-0.371	-0.438	-0.193

D-isomer specific 2-hydroxyacid dehydrogenase family protein

unknown function

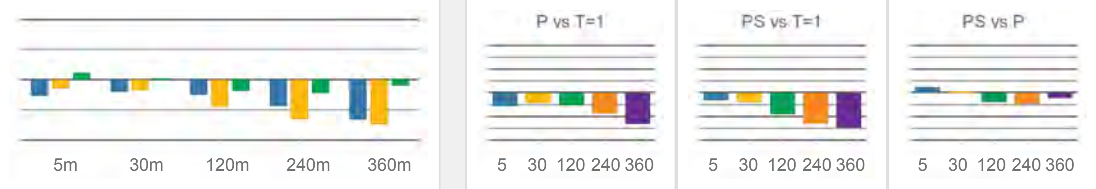

Locus		5m	30m	120m	240m	360m
**PGN_0126**	P vs T=1	-0.568	-0.342	0.069	-0.080	-0.384
	PS vs T=1	-0.139	0.026	0.024	0.327	0.395
	PS vs P	0.387	0.344	-0.032	0.399	0.755

putative transmembrane glucose/galactose transporter

transport and binding proteins

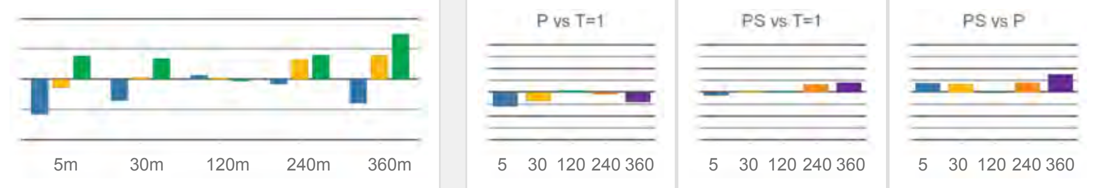

log$_2$ (Fold Change)

Legend: P vs T=1 | PS vs T=1 | PS vs P

Locus		5m	30m	120m	240m	360m
**PGN_0127**	P vs T=1	-1.141	-1.042	-0.741	-0.917	-0.873
	PS vs T=1	-0.509	-0.635	-0.689	-0.460	-0.801
	PS vs P	0.464	0.269	0.076	0.317	-0.010
	hypothetical protein					
	hypothetical proteins					
**PGN_0128**	P vs T=1	-0.867	-0.822	-0.967	-1.476	-2.253
	PS vs T=1	-1.532	-1.214	-1.271	-1.884	-2.167
	PS vs P	-0.637	-0.360	-0.278	-0.413	0.046
	immunoreactive 53 kDa antigen					
	cell envelope					
**PGN_0129**	P vs T=1	-1.013	-0.989	-1.388	-2.189	-3.103
	PS vs T=1	-1.730	-1.210	-1.226	-2.070	-2.624
	PS vs P	-0.663	-0.164	0.188	0.059	0.359
	conserved hypothetical protein					
	hypothetical proteins-Conserved					
**PGN_0130**	P vs T=1	0.649	0.523	0.626	0.776	0.490
	PS vs T=1	0.488	0.294	0.476	0.481	0.436
	PS vs P	-0.156	-0.238	-0.156	-0.283	-0.069
	partial transposase in ISPg1					
**PGN_0131**	P vs T=1	-0.375	-0.527	-0.595	-0.437	-0.618
	PS vs T=1	-0.568	-0.947	-0.702	-0.632	-0.436
	PS vs P	-0.193	-0.432	-0.140	-0.207	0.149
	partial transposase in ISPg1					
**PGN_0132**	P vs T=1	-0.075	0.750	1.428	1.752	2.153
	PS vs T=1	0.508	0.719	1.036	1.630	1.700
	PS vs P	0.328	-0.043	-0.277	0.053	-0.268
	conserved hypothetical protein					
	hypothetical proteins-Conserved					
**PGN_0133** *BioA*	P vs T=1	-1.562	-1.426	-0.887	-0.421	0.413
	PS vs T=1	-0.786	-0.981	-1.075	-0.522	-0.594
	PS vs P	0.625	0.302	-0.225	-0.024	-0.837
	adenosylmethionine--8-amino-7-oxononanoate aminotransferase					
	biosynthesis of cofactors, prosthetic groups, and carriers					

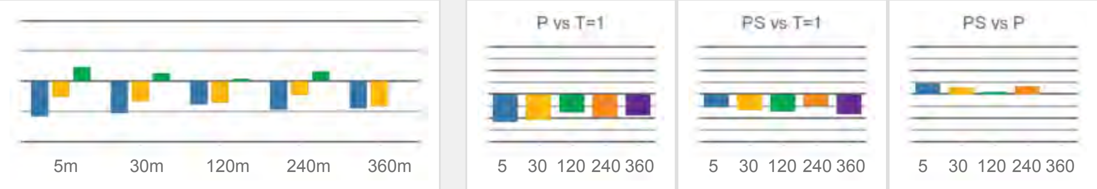

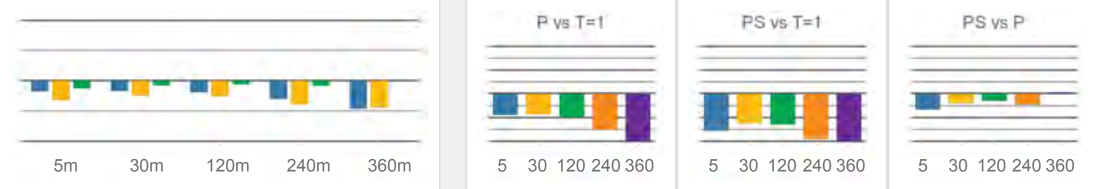

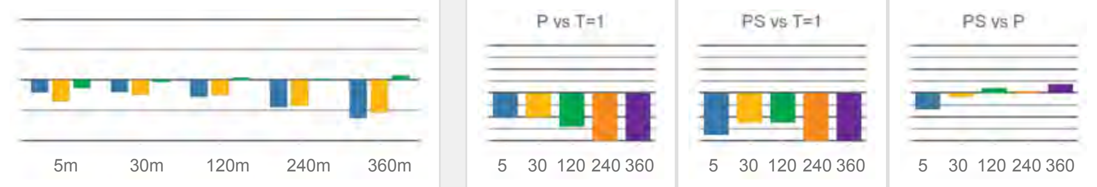

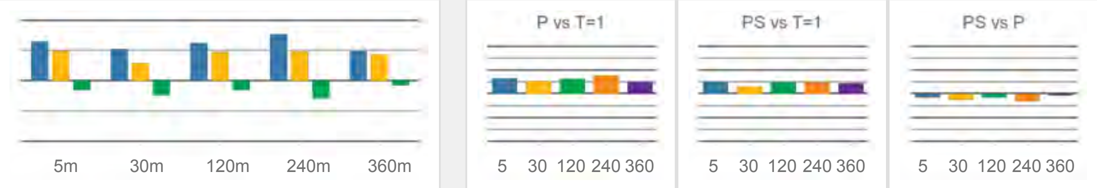

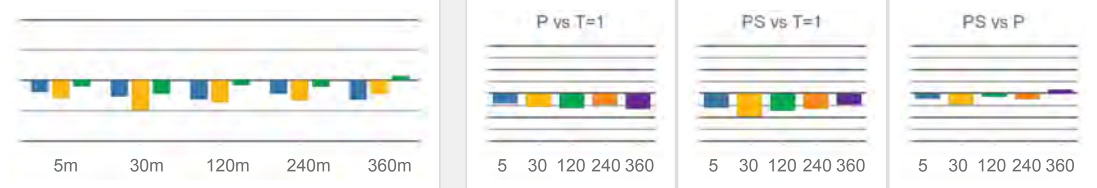

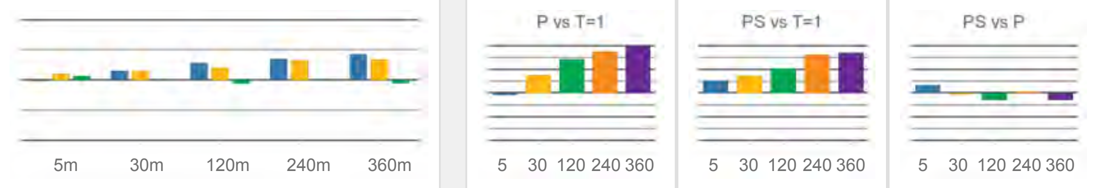

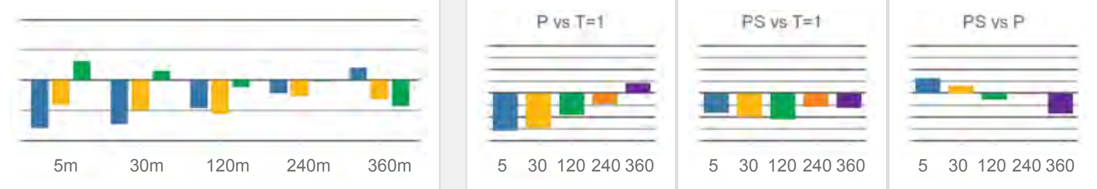

Legend: P vs T=1 | PS vs T=1 | PS vs P

Locus		5m	30m	120m	240m	360m
		log₂ (Fold Change)				
**PGN_0134**	P vs T=1	-0.664	-0.991	-1.004	-0.736	0.172
BioB	PS vs T=1	-0.221	-0.751	-0.519	-0.299	-0.234
	PS vs P	0.428	0.167	0.369	0.365	-0.312
	putative biotin synthetase					
	biosynthesis of cofactors, prosthetic groups, and carriers					
**PGN_0135**	P vs T=1	-1.696	-1.969	-2.162	-2.577	-3.063
	PS vs T=1	-1.648	-1.604	-2.068	-2.603	-2.690
	PS vs P	0.066	0.353	0.091	-0.064	0.311
	putative H+/peptide symporter					
	transport and binding proteins					
**PGN_0136**	P vs T=1	-0.500	-1.111	-1.939	-1.958	-1.458
	PS vs T=1	-0.352	-0.755	-0.753	-0.558	-0.715
	PS vs P	0.174	0.337	1.069	1.258	0.711
	conserved hypothetical protein					
	hypothetical proteins-Conserved					
**PGN_0137**	P vs T=1	-0.454	-0.475	-0.855	-1.460	-1.996
	PS vs T=1	-0.754	-0.558	-0.385	-0.287	-0.610
	PS vs P	-0.282	-0.064	0.464	1.090	1.287
	putative tryptophanyl-tRNA synthetase					
	protein synthesis					
**PGN_0138**	P vs T=1	0.099	0.636	0.975	0.749	0.190
	PS vs T=1	0.059	0.216	0.252	0.153	0.051
	PS vs P	-0.052	-0.400	-0.685	-0.569	-0.145
	conserved hypothetical protein					
	hypothetical proteins-Conserved					
**PGN_0139**	P vs T=1	-0.298	-0.108	-0.039	-0.317	-0.679
	PS vs T=1	-0.124	-0.454	-0.621	-0.682	-0.902
	PS vs P	0.169	-0.333	-0.553	-0.361	-0.251
	conserved hypothetical protein					
	hypothetical proteins-Conserved					
**PGN_0140**	P vs T=1	-0.292	-0.283	-0.762	-1.200	-0.930
	PS vs T=1	0.079	-0.360	-0.761	-0.752	-0.867
	PS vs P	0.376	-0.062	-0.007	0.365	0.049
	peptide methionine sulfoxide reductase					
	protein fate					

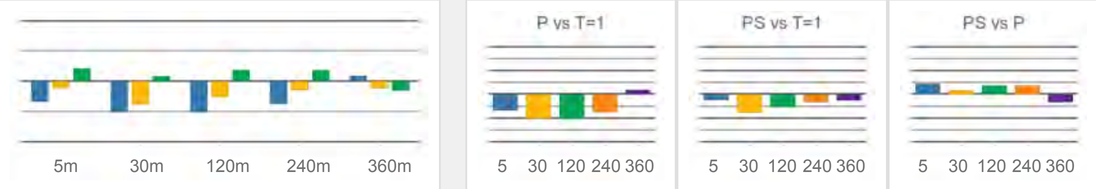

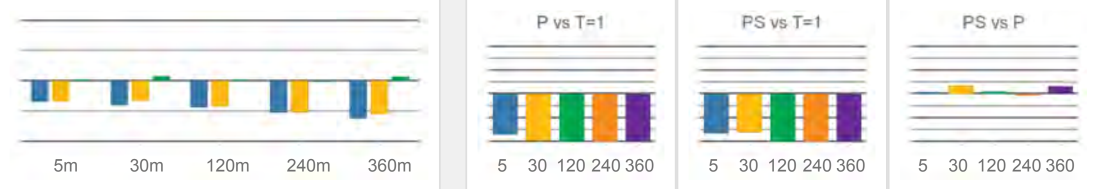

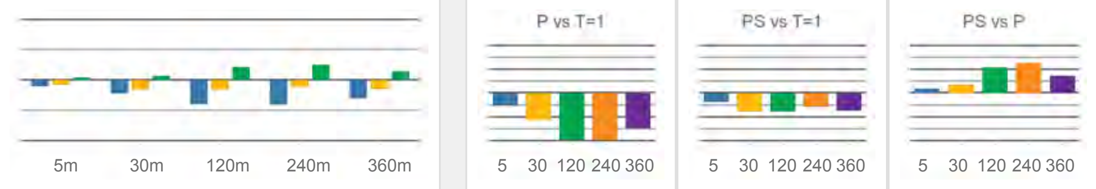

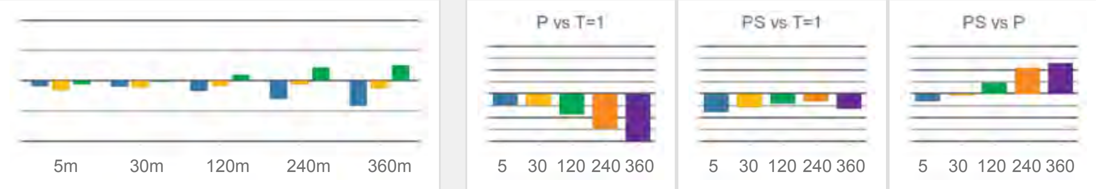

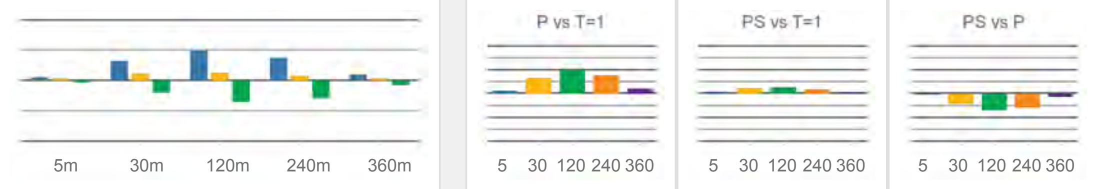

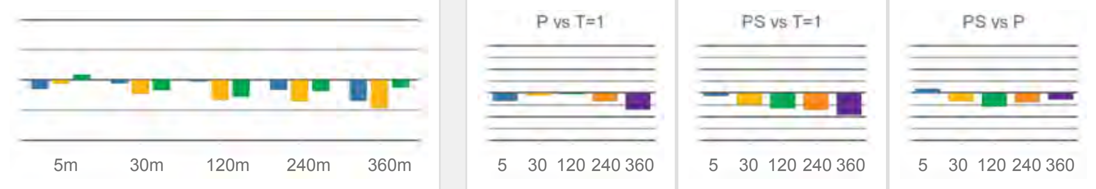

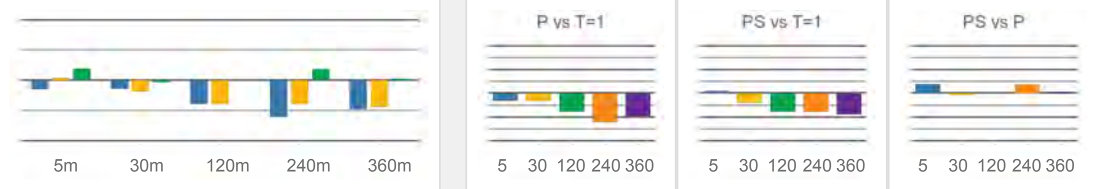

PgSg Time Course

log$_2$ (Fold Change)

Legend: P vs T=1 | PS vs T=1 | PS vs P

Locus		5m	30m	120m	240m	360m
**PGN_0141**	P vs T=1	0.077	-0.002	-0.329	-0.463	-0.392
	PS vs T=1	0.474	-0.051	-0.363	-0.345	-0.384
	PS vs P	0.402	-0.041	-0.046	0.089	-0.001

conserved hypothetical protein

hypothetical proteins-Conserved

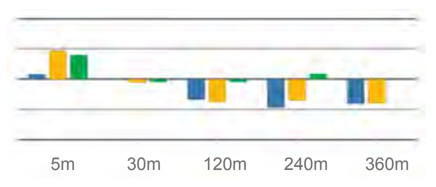

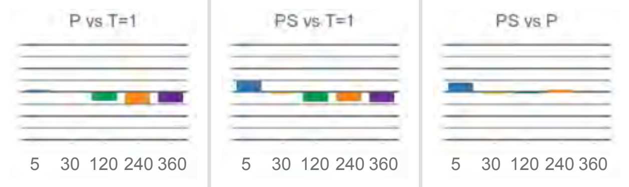

Locus		5m	30m	120m	240m	360m
**PGN_0142**	P vs T=1	-0.664	-1.123	-1.670	-1.954	-1.411
	PS vs T=1	-1.392	-1.589	-1.847	-1.763	-1.650
	PS vs P	-0.658	-0.433	-0.205	0.078	-0.222

putative cation efflux protein

transport and binding proteins

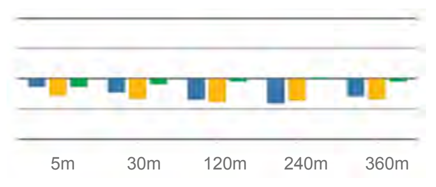

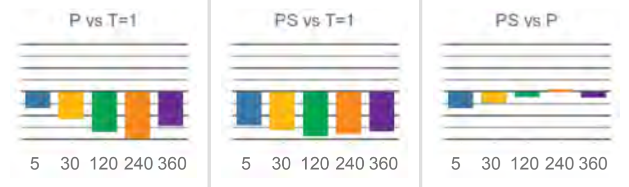

Locus		5m	30m	120m	240m	360m
**PGN_0143**	P vs T=1	-0.888	-0.728	-0.739	-0.827	-0.908
	PS vs T=1	-1.410	-1.054	-1.076	-0.855	-0.924
	PS vs P	-0.519	-0.295	-0.308	-0.030	-0.038

probable dihydroneopterin aldolase

biosynthesis of cofactors, prosthetic groups, and carriers

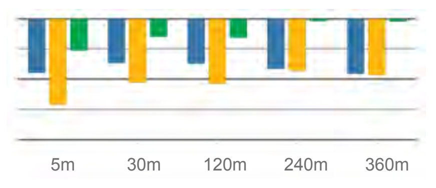

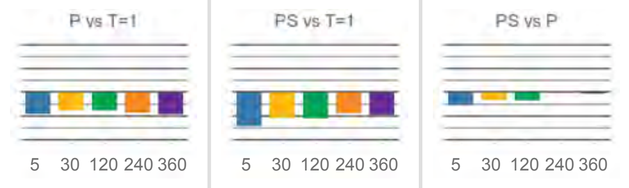

Locus		5m	30m	120m	240m	360m
**PGN_0144**	P vs T=1	-0.506	-0.732	-1.140	-1.033	-0.723
	PS vs T=1	-0.293	-0.388	-0.218	0.012	-0.096
	PS vs P	0.203	0.310	0.814	0.936	0.594

hypothetical protein

hypothetical proteins

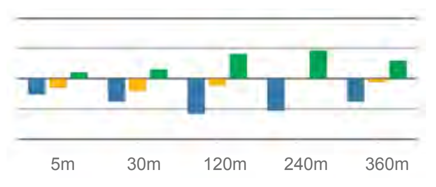

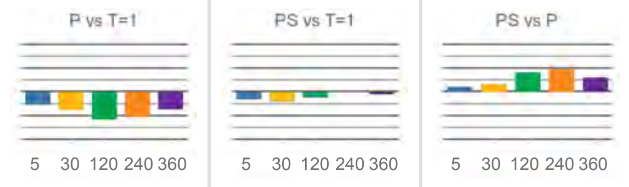

Locus		5m	30m	120m	240m	360m
**PGN_0145**	P vs T=1	-0.720	-0.955	-1.197	-1.083	-0.171
	PS vs T=1	-0.706	-1.149	-1.690	-1.409	-1.288
	PS vs P	0.027	-0.193	-0.511	-0.336	-1.067

conserved hypothetical protein

hypothetical proteins-Conserved

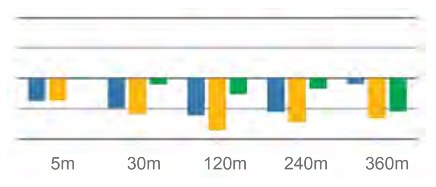

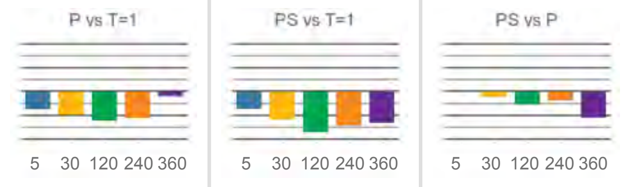

Locus		5m	30m	120m	240m	360m
**PGN_0146**	P vs T=1	0.171	-0.214	0.041	0.626	1.877
	PS vs T=1	0.682	-0.101	0.771	0.409	0.427
	PS vs P	0.442	-0.141	0.479	-0.017	-0.502

hypothetical protein

hypothetical proteins

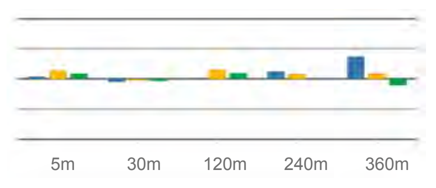

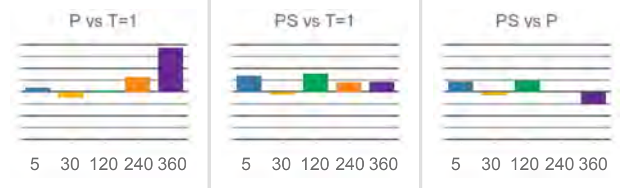

Locus		5m	30m	120m	240m	360m
**PGN_0147**	P vs T=1	-0.641	-0.551	-0.740	-0.514	0.072
	PS vs T=1	0.331	-0.039	-0.632	-0.956	-0.920
	PS vs P	0.942	0.495	0.072	-0.443	-0.952

conserved hypothetical protein

hypothetical proteins-Conserved

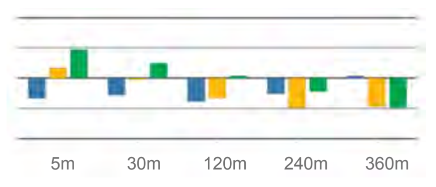

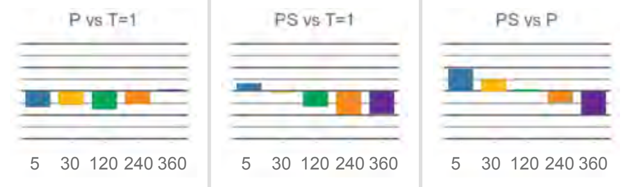

PgSg Time Course

log$_2$ (Fold Change)

Legend: P vs T=1 | PS vs T=1 | PS vs P

Locus		5m	30m	120m	240m	360m
**PGN_0148**	P vs T=1	-0.518	-0.511	-1.185	-1.196	-0.650
	PS vs T=1	-0.132	-0.431	-1.264	-1.415	-1.556
	PS vs P	0.388	0.090	-0.107	-0.244	-0.892
	conserved hypothetical protein					
	hypothetical proteins-Conserved					

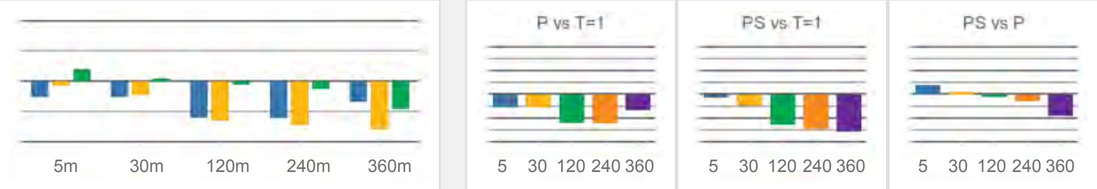

Locus		5m	30m	120m	240m	360m
**PGN_0149**	P vs T=1	0.321	-0.036	-0.336	-0.914	-1.368
	PS vs T=1	-0.043	-0.377	-0.591	-0.592	-0.773
	PS vs P	-0.315	-0.316	-0.239	0.286	0.546
	ribose-phosphate pyrophosphokinase					
	purines, pyrimidines, nucleosides and nucleotides					

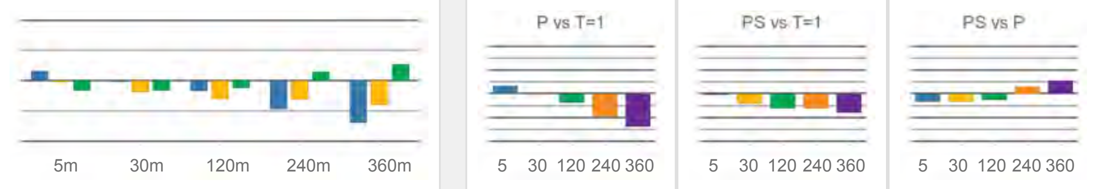

Locus		5m	30m	120m	240m	360m
**PGN_0150**	P vs T=1	-0.794	-0.474	-0.083	0.233	0.046
	PS vs T=1	-0.782	-0.779	-0.647	-0.399	-0.164
	PS vs P	-0.034	-0.310	-0.538	-0.567	-0.192
	putative ATP-dependent RNA helicase					
	transcription					

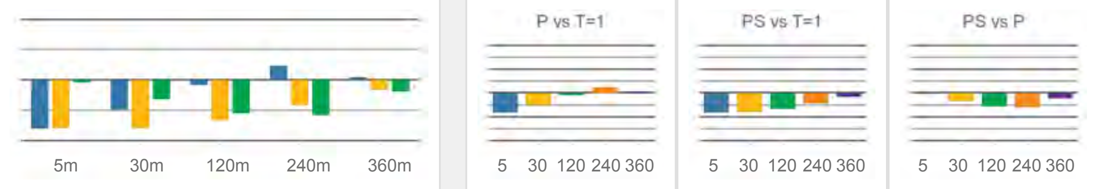

Locus		5m	30m	120m	240m	360m
**PGN_0151**	P vs T=1	0.368	0.905	1.288	0.904	0.122
	PS vs T=1	0.319	0.035	-0.364	-0.906	-0.994
	PS vs P	-0.043	-0.738	-1.461	-1.675	-1.108
	conserved hypothetical protein					
	hypothetical proteins-Conserved					

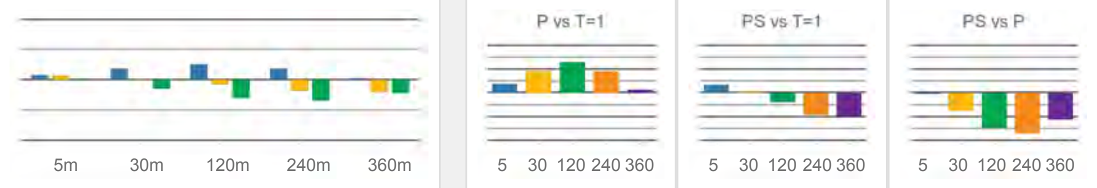

Locus		5m	30m	120m	240m	360m
**PGN_0152**	P vs T=1	0.765	1.212	1.851	1.741	1.030
	PS vs T=1	-0.196	-0.110	-0.090	-0.614	-0.698
	PS vs P	-0.933	-1.259	-1.848	-2.270	-1.699
	immunoreactive 61 kDa antigen					
	cell envelope					

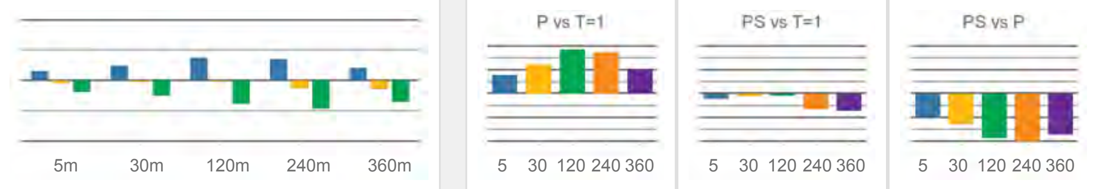

Locus		5m	30m	120m	240m	360m
**PGN_0153**	P vs T=1	2.020	1.959	1.965	2.086	2.072
	PS vs T=1	0.506	0.587	0.582	0.605	1.271
	PS vs P	-1.233	-1.085	-1.103	-1.049	-0.530
	conserved hypothetical protein					
	hypothetical proteins-Conserved					

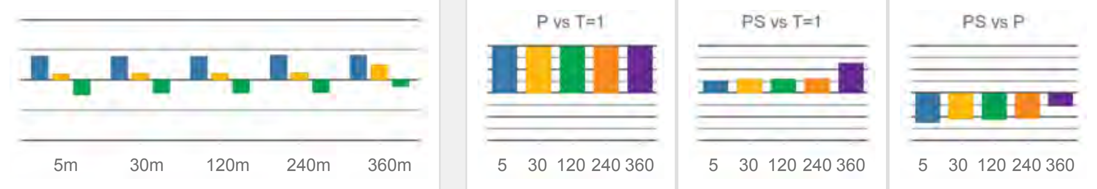

Locus		5m	30m	120m	240m	360m
**PGN_0154**	P vs T=1	-1.243	-1.084	-0.622	-0.616	-1.180
	PS vs T=1	-1.081	-0.549	-0.367	-0.554	-0.469
	PS vs P	0.108	0.491	0.273	0.080	0.673
	conserved hypothetical protein					
	hypothetical proteins-Conserved					

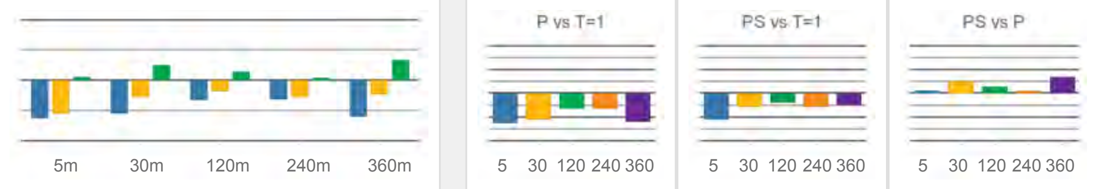

PgSg Time Course

Hackett Laboratory UW

Legend: P vs T=1 | PS vs T=1 | PS vs P

Locus		5m	30m	120m	240m	360m
		log$_2$ (Fold Change)				
**PGN_0155**	P vs T=1	-0.891	-0.779	-1.094	-1.349	-1.234
	PS vs T=1	-2.262	-1.429	-1.521	-2.143	-1.695
	PS vs P	-1.201	-0.468	-0.338	-0.775	-0.424
	hypothetical protein					
	hypothetical proteins					
**PGN_0156**	P vs T=1	0.355	0.477	0.626	0.177	-0.852
	PS vs T=1	-0.474	0.094	0.171	0.090	-0.025
	PS vs P	-0.786	-0.335	-0.392	-0.070	0.766
	conserved hypothetical protein					
	hypothetical proteins-Conserved					
**PGN_0157** *ThiH*	P vs T=1	0.662	0.899	2.010	2.604	3.449
	PS vs T=1	0.321	0.407	1.584	2.355	2.653
	PS vs P	-0.429	-0.546	-0.294	-0.009	-0.544
	putative thiamine biosynthesis protein ThiH					
	biosynthesis of cofactors, prosthetic groups, and carriers					
**PGN_0158** *ThiG*	P vs T=1	-0.124	-0.143	1.066	2.111	2.982
	PS vs T=1	-0.316	0.070	0.717	1.774	1.739
	PS vs P	-0.429	-0.166	-0.269	0.148	-0.666
	putative thiazole biosynthesis protein ThiG					
	biosynthesis of cofactors, prosthetic groups, and carriers					
**PGN_0159** *ThiD/E*	P vs T=1	-0.586	-0.274	0.822	1.757	2.646
	PS vs T=1	-0.389	-0.054	0.588	1.118	1.467
	PS vs P	-0.072	0.000	-0.184	-0.319	-0.859
	probable thiamin-phosphate pyrophosphorylase					
	biosynthesis of cofactors, prosthetic groups, and carriers					
**PGN_0160** *ThiC*	P vs T=1	-0.503	-0.351	0.632	1.567	2.420
	PS vs T=1	-0.232	-0.176	0.305	1.096	1.418
	PS vs P	0.061	-0.037	-0.316	-0.209	-0.736
	thiamine biosynthesis protein					
	biosynthesis of cofactors, prosthetic groups, and carriers					
**PGN_0161** *ThiS*	P vs T=1	-0.993	-2.650	-2.400	-1.196	-0.413
	PS vs T=1	-1.511	-2.126	-1.918	-1.495	-1.424
	PS vs P	-0.344	-0.001	0.002	-0.061	-0.664
	putative thiamine biosynthesis protein ThiS					
	biosynthesis of cofactors, prosthetic groups, and carriers					

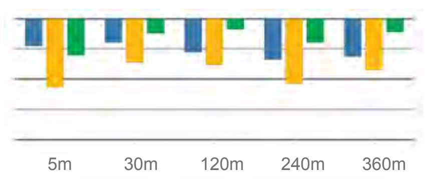

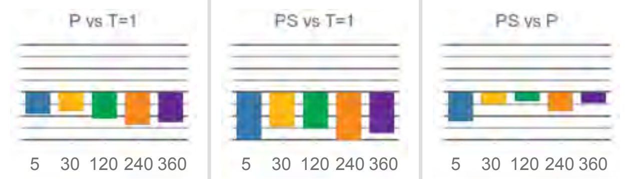

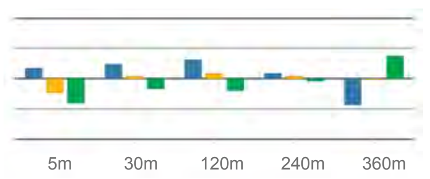

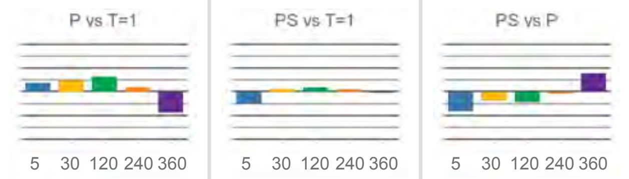

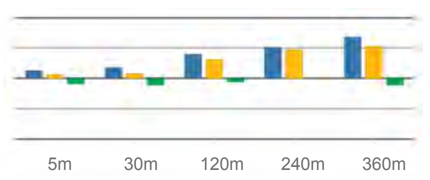

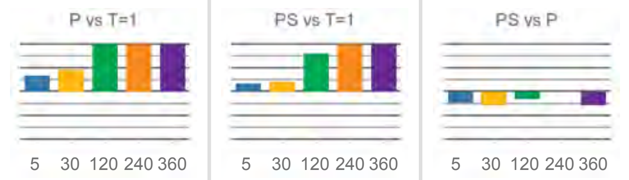

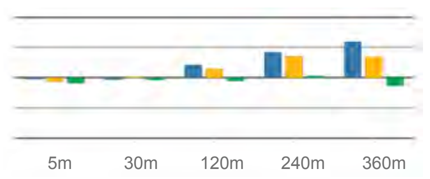

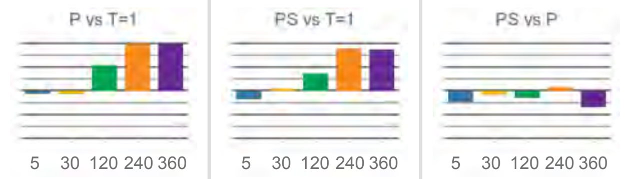

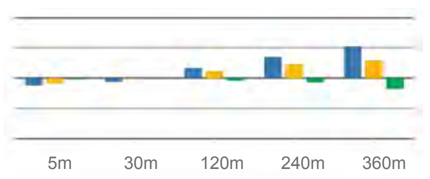

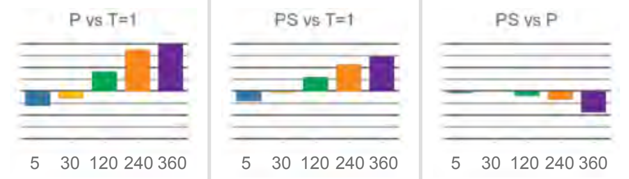

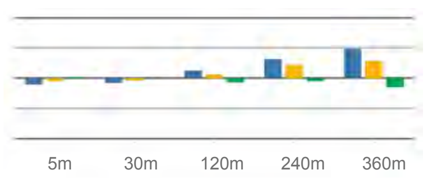

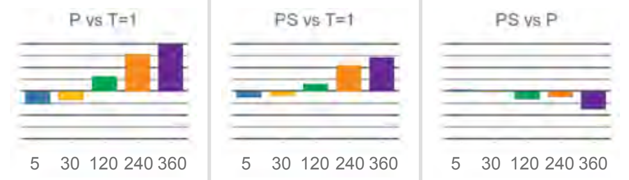

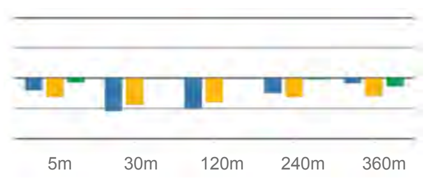

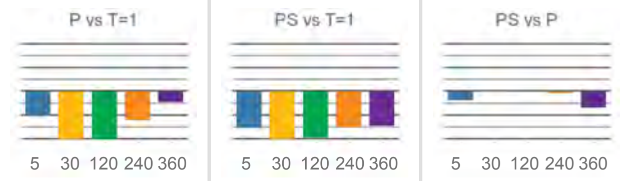

log$_2$ (Fold Change)

Legend: P vs T=1 | PS vs T=1 | PS vs P

Locus		5m	30m	120m	240m	360m
**PGN_0162**	P vs T=1	-0.317	-0.459	0.127	0.499	0.955
	PS vs T=1	-0.829	-0.895	-0.234	-0.076	0.358
	PS vs P	-0.502	-0.470	-0.299	-0.417	-0.450

conserved hypothetical protein

hypothetical proteins-Conserved

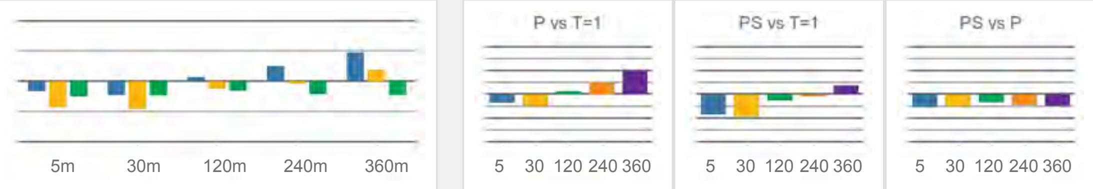

Locus		5m	30m	120m	240m	360m
**PGN_0163**	P vs T=1	0.046	0.192	0.484	0.390	-0.218
	PS vs T=1	-0.221	0.397	1.085	0.658	0.586
	PS vs P	-0.279	0.199	0.635	0.291	0.742

hypothetical protein

hypothetical proteins

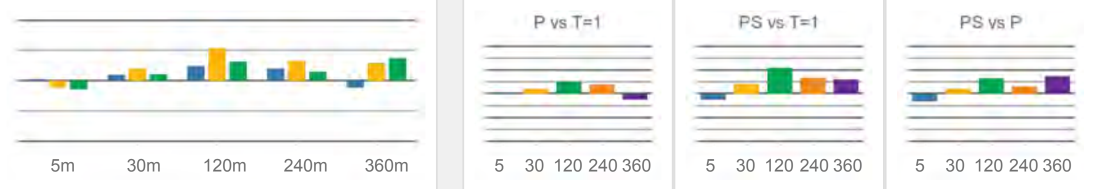

Locus		5m	30m	120m	240m	360m
**PGN_0164**	P vs T=1	0.563	0.243	-0.045	1.746	2.682
	PS vs T=1	-0.074	-0.309	0.231	0.992	0.823
	PS vs P	-0.441	-0.479	-0.184	0.024	-0.894

conserved hypothetical protein

hypothetical proteins-Conserved

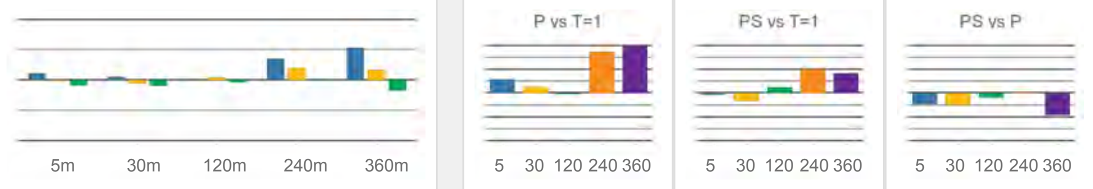

Locus		5m	30m	120m	240m	360m
**PGN_0165**	P vs T=1	0.835	0.123	0.352	0.086	1.248
	PS vs T=1	-0.818	-0.712	-0.837	0.078	-0.176
	PS vs P	-0.932	-0.571	-0.726	-0.002	-0.476

conserved hypothetical protein

hypothetical proteins-Conserved

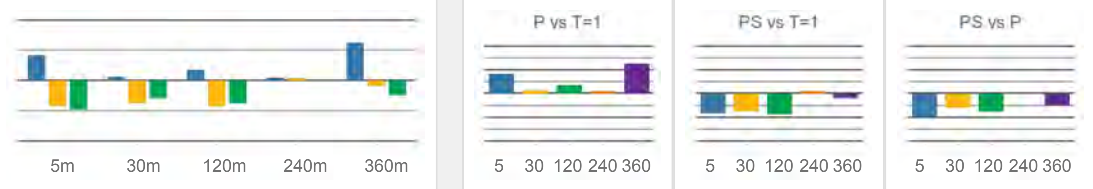

Locus		5m	30m	120m	240m	360m
**PGN_0166**	P vs T=1	0.525	-0.068	0.774	1.382	2.573
	PS vs T=1	-0.806	-0.560	-1.231	0.823	0.970
	PS vs P	-0.991	-0.516	-1.422	0.004	-0.819

conserved hypothetical protein

hypothetical proteins-Conserved

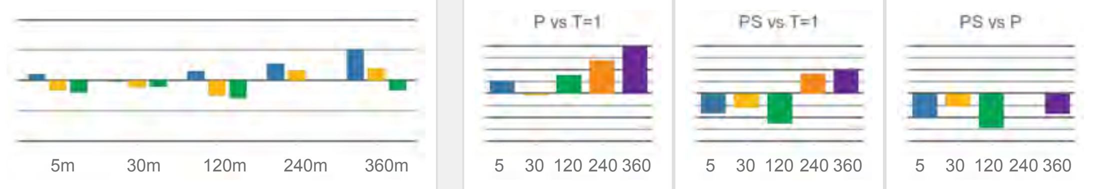

Locus		5m	30m	120m	240m	360m
**PGN_0167**	P vs T=1	0.291	0.497	0.899	0.781	-0.001
rpsP	PS vs T=1	-1.387	-1.042	-0.667	-0.714	-0.669
	PS vs P	-1.624	-1.473	-1.491	-1.428	-0.659

30S ribosomal protein S16

protein synthesis

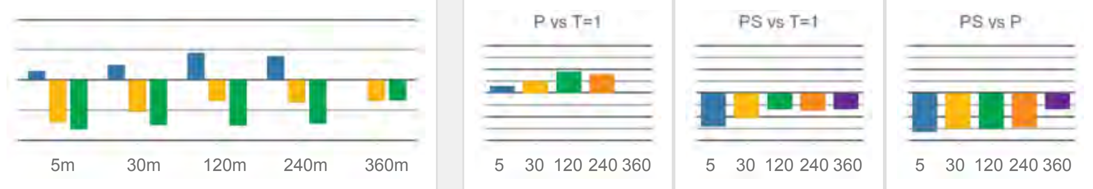

Locus		5m	30m	120m	240m	360m
**PGN_0168**	P vs T=1	0.245	-0.314	-1.001	-0.642	-0.438
wbpB	PS vs T=1	0.280	-0.281	-0.607	-0.555	-0.789
	PS vs P	0.091	0.030	0.274	0.060	-0.345

lipopolysaccharide biosynthesis protein WbpB

unknown function

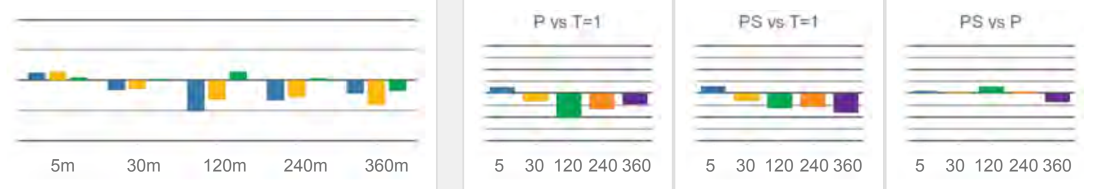

PgSg Time Course

Hackett Laboratory UW

Legend: P vs T=1 | PS vs T=1 | PS vs P

log2 (Fold Change)

Locus		5m	30m	120m	240m	360m
PGN_0169	P vs T=1	-0.083	-0.587	-1.222	-0.933	-0.373
	PS vs T=1	-0.239	-0.601	-0.519	-0.448	-0.466
	PS vs P	-0.122	-0.019	0.594	0.428	-0.073

probable metallo-beta-lactamase superfamily protein

unknown function

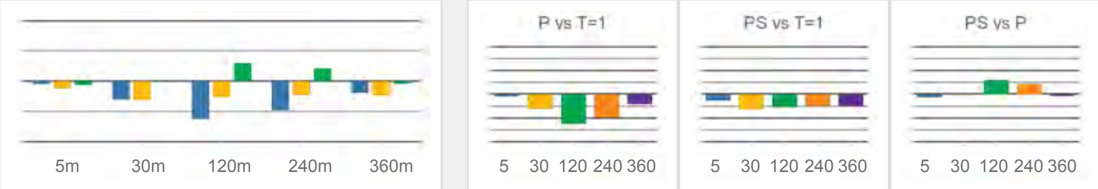

Locus		5m	30m	120m	240m	360m
PGN_0170	P vs T=1	-0.039	-0.538	-1.641	-1.653	-1.374
	PS vs T=1	-0.416	-0.664	-0.632	-0.582	-1.107
	PS vs P	-0.320	-0.104	0.911	0.970	0.238

L-asparaginase

energy metabolism

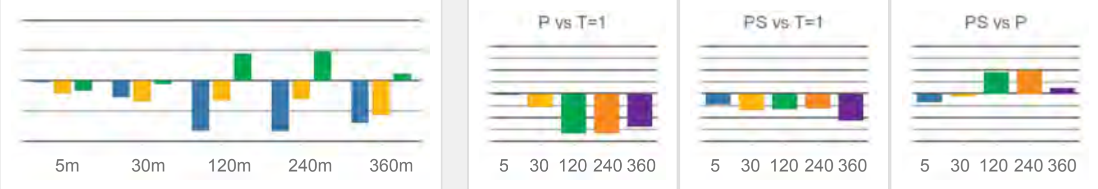

Locus		5m	30m	120m	240m	360m
PGN_0171	P vs T=1	0.824	-0.173	-0.589	-0.667	0.219
	PS vs T=1	0.614	0.246	0.831	0.520	0.158
	PS vs P	-0.029	0.310	1.151	0.826	0.032

partial transposase in ISPg2

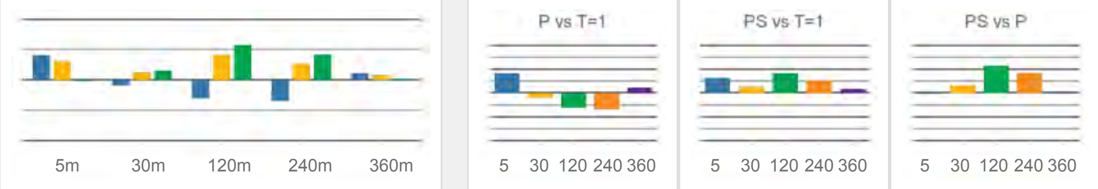

Locus		5m	30m	120m	240m	360m
PGN_0172	P vs T=1	1.712	1.878	1.263	0.379	-0.174
	PS vs T=1	1.289	1.707	2.270	2.346	1.668
	PS vs P	-0.318	-0.030	1.020	1.742	1.615

conserved hypothetical protein

hypothetical proteins-Conserved

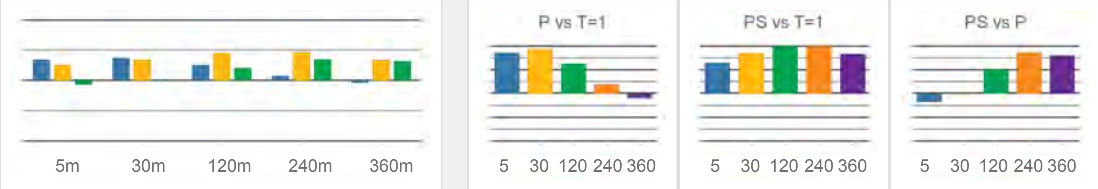

Locus		5m	30m	120m	240m	360m
PGN_0173	P vs T=1	0.466	0.859	0.888	0.509	-0.256
	PS vs T=1	0.090	0.480	0.939	1.025	0.873
	PS vs P	-0.375	-0.336	0.084	0.509	1.079

glyceraldehyde 3-phosphate dehydrogenase type I

energy metabolism

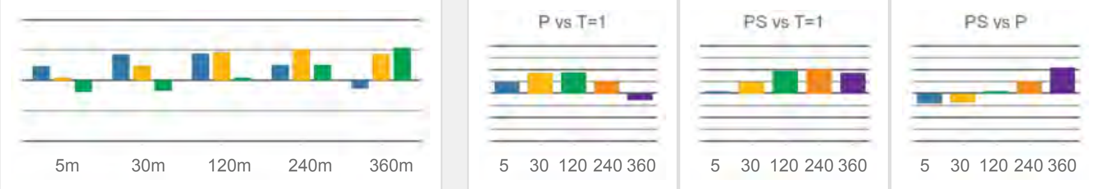

Locus		5m	30m	120m	240m	360m
PGN_0174	P vs T=1	-0.166	-0.267	-0.468	-0.167	0.440
	PS vs T=1	0.082	-0.090	0.049	0.270	0.187
	PS vs P	0.241	0.159	0.432	0.406	-0.202

probable transcriptional regulator AraC family

regulatory functions

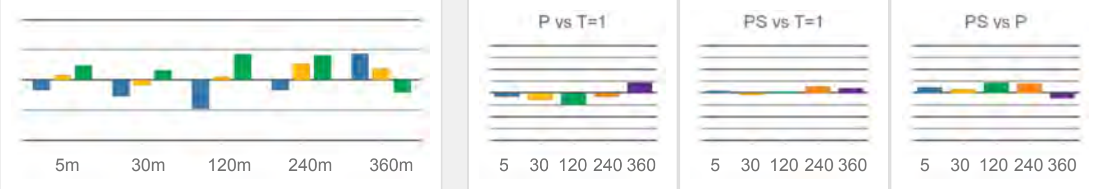

Locus		5m	30m	120m	240m	360m
PGN_0175	P vs T=1	-0.075	-0.123	-0.226	-0.394	-0.296
	PS vs T=1	0.475	0.048	-0.665	-0.397	-0.520
	PS vs P	0.544	0.168	-0.435	-0.037	-0.227

conserved hypothetical protein

hypothetical proteins-Conserved

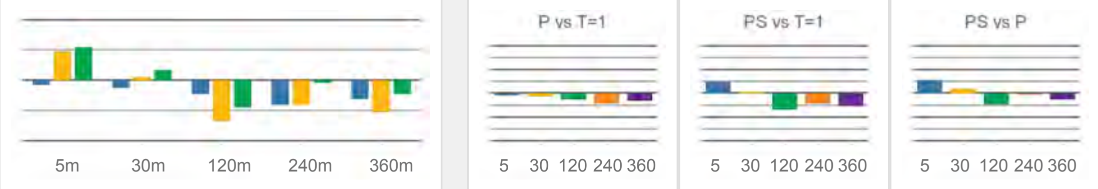

log$_2$ (Fold Change)

Legend: P vs T=1 | PS vs T=1 | PS vs P

Locus		5m	30m	120m	240m	360m
**PGN_0176**	P vs T=1	-0.629	-0.629	-0.412	-0.635	-1.003
	PS vs T=1	-0.366	-0.443	-0.269	-0.267	-0.450
	PS vs P	0.224	0.146	0.163	0.323	0.450
	conserved hypothetical protein					
	hypothetical proteins-Conserved					
**PGN_0177**	P vs T=1	-0.580	-0.239	0.062	0.257	1.598
	PS vs T=1	-1.443	-0.672	0.177	0.391	-0.035
	PS vs P	-0.899	-0.387	0.152	0.180	-0.891
	hypothetical protein					
	hypothetical proteins					
**PGN_0178**	P vs T=1	-2.200	-2.216	-2.200	-2.696	-3.538
	PS vs T=1	-1.695	-2.063	-1.772	-1.554	-1.748
	PS vs P	0.452	0.110	0.404	1.038	1.621
	conserved hypothetical protein					
	hypothetical proteins-Conserved					
**PGN_0179**	P vs T=1	-0.762	-0.326	-0.451	-1.325	-2.159
	PS vs T=1	0.355	0.263	-0.110	-0.448	-1.076
	PS vs P	1.052	0.584	0.347	0.795	0.974
	60 kDa protein					
	unknown function					
**PGN_0180** *fimA*	P vs T=1	0.134	0.751	1.097	0.552	-0.407
	PS vs T=1	1.471	1.993	2.035	1.276	0.779
	PS vs P	1.231	1.202	0.950	0.688	1.108
	FimA type I fimbrilin					
	cellular processes					
**PGN_0181**	P vs T=1	-0.427	-0.127	0.419	0.253	-0.252
	PS vs T=1	1.047	0.699	0.308	0.270	0.036
	PS vs P	1.314	0.729	-0.085	0.007	0.210
	conserved hypothetical protein					
	hypothetical proteins-Conserved					
**PGN_0182**	P vs T=1	0.024	0.010	-0.308	-0.229	-0.772
	PS vs T=1	1.591	1.237	0.205	-0.254	-0.657
	PS vs P	1.412	1.091	0.364	-0.087	-0.085
	conserved hypothetical protein					
	hypothetical proteins-Conserved					

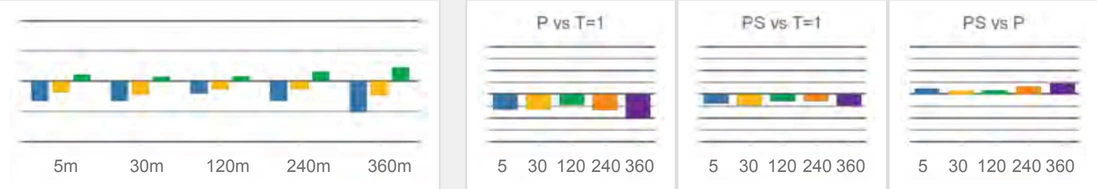

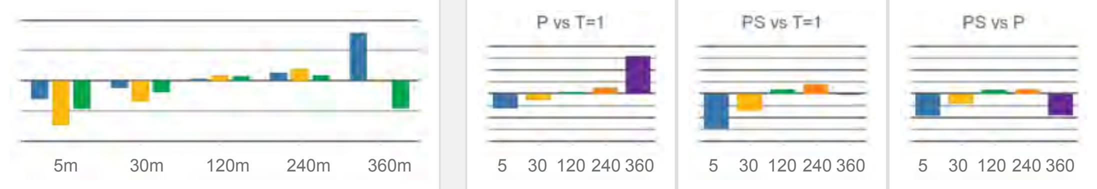

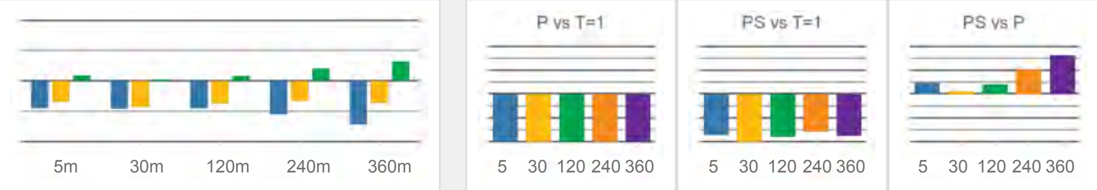

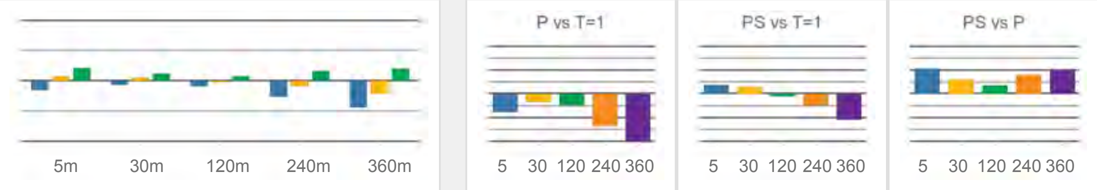

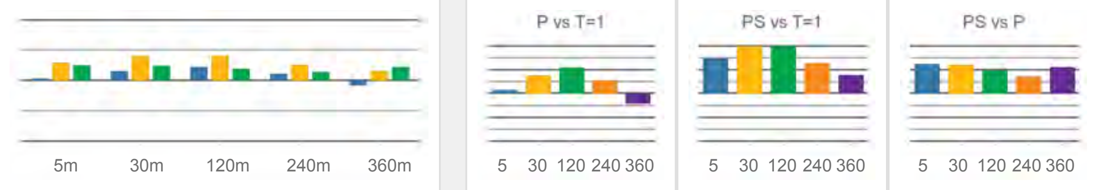

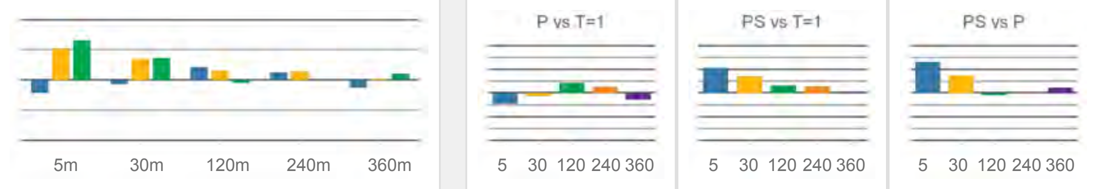

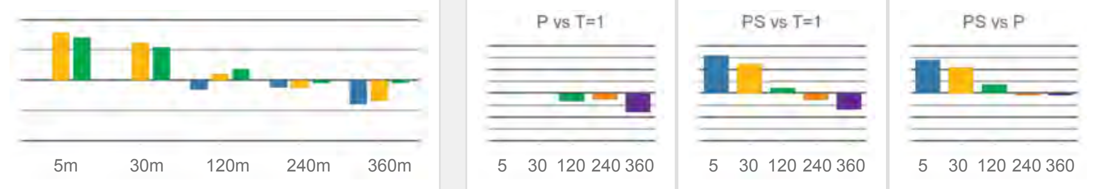

log2 (Fold Change)

Legend: P vs T=1 | PS vs T=1 | PS vs P

PGN_0183 *fimC*

Locus	5m	30m	120m	240m	360m
P vs T=1	-0.450	-0.264	-0.872	-1.545	-2.069
PS vs T=1	0.846	0.608	-0.851	-1.600	-2.038
PS vs P	1.270	0.885	0.014	-0.133	-0.064

minor component FimC

cellular processes

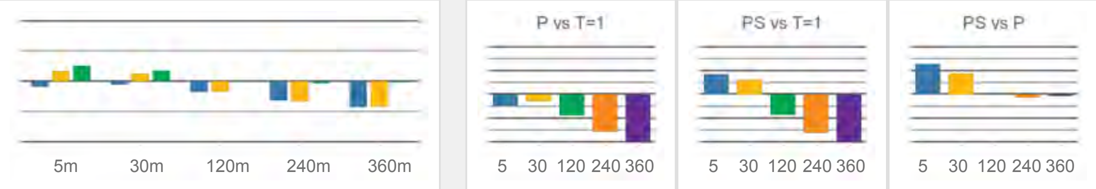

PGN_0184 *fimD*

Locus	5m	30m	120m	240m	360m
P vs T=1	-1.317	-0.912	-1.420	-2.224	-2.747
PS vs T=1	0.156	0.270	-0.699	-1.643	-1.927
PS vs P	1.380	1.162	0.687	0.455	0.692

minor component FimD

cellular processes

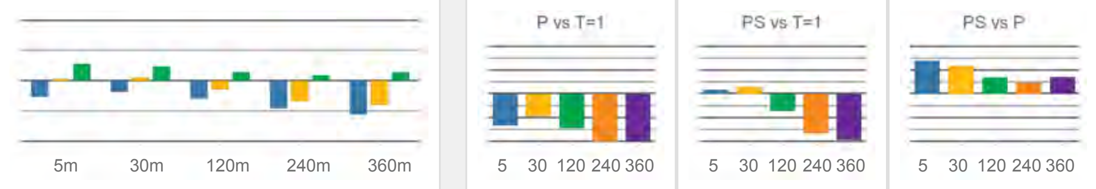

PGN_0185 *fimE*

Locus	5m	30m	120m	240m	360m
P vs T=1	-1.000	-0.906	-1.147	-1.702	-2.412
PS vs T=1	0.288	0.366	-0.345	-1.347	-1.493
PS vs P	1.237	1.236	0.778	0.291	0.830

minor component FimE

cellular processes

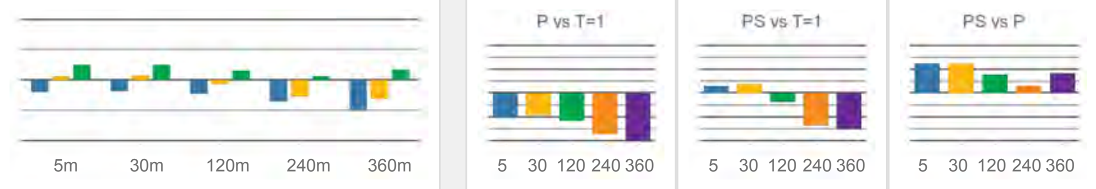

PGN_0186

Locus	5m	30m	120m	240m	360m
P vs T=1	-1.348	-1.005	-0.822	-0.710	-0.956
PS vs T=1	-0.655	-0.619	-0.462	-0.350	-0.433
PS vs P	0.516	0.290	0.276	0.288	0.379

hypothetical protein

hypothetical proteins

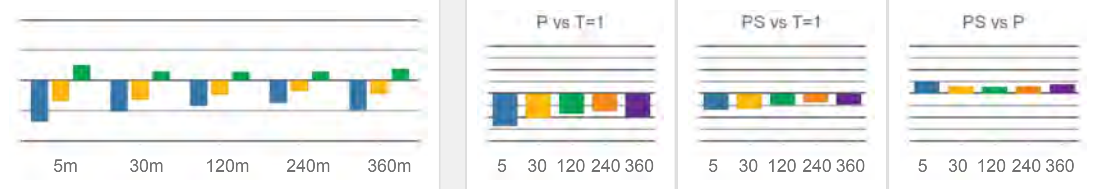

PGN_0187

Locus	5m	30m	120m	240m	360m
P vs T=1	0.018	-0.092	0.038	-0.077	-0.664
PS vs T=1	-1.342	-1.013	-0.757	-1.042	-0.917
PS vs P	-1.305	-0.879	-0.747	-0.916	-0.257

conserved hypothetical protein

hypothetical proteins-Conserved

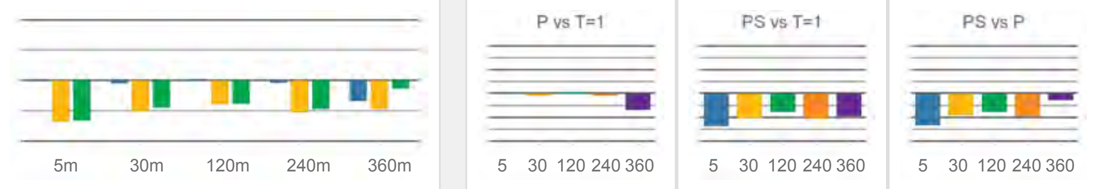

PGN_0188 *rpmF*

Locus	5m	30m	120m	240m	360m
P vs T=1	0.234	0.742	1.558	1.761	0.884
PS vs T=1	-0.706	-0.361	-0.106	0.201	0.391
PS vs P	-0.928	-1.030	-1.529	-1.411	-0.465

50S ribosomal protein L32

protein synthesis

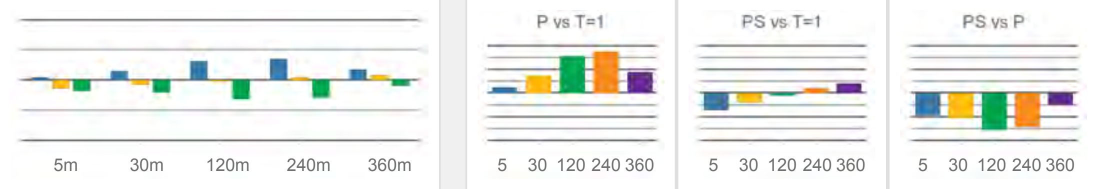

PGN_0189

Locus	5m	30m	120m	240m	360m
P vs T=1	0.158	0.288	0.624	0.699	0.164
PS vs T=1	-0.538	-0.476	-0.028	0.129	0.070
PS vs P	-0.685	-0.747	-0.628	-0.544	-0.093

beta-ketoacyl-acyl-carrier-protein synthase III

fatty acid and phospholipid metabolism

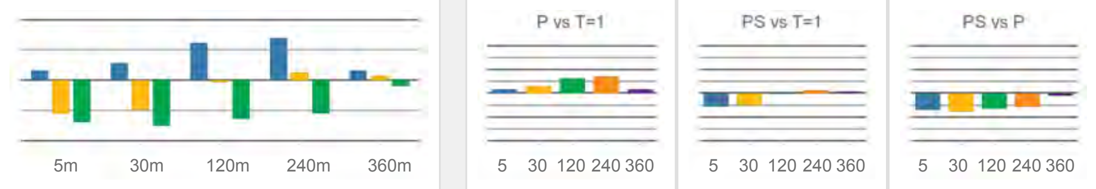

log2 (Fold Change)

Legend: P vs T=1 · PS vs T=1 · PS vs P

Locus		5m	30m	120m	240m	360m
**PGN_0190**	P vs T=1	-0.649	-0.852	-1.285	-1.398	-1.216
	PS vs T=1	-0.460	-1.024	-1.279	-1.627	-1.488
	PS vs P	0.211	-0.157	-0.013	-0.259	-0.271

putative GTP-binding protein

regulatory functions

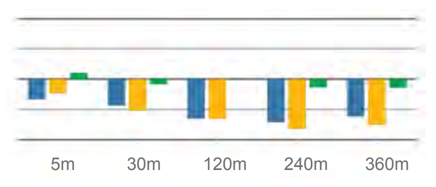

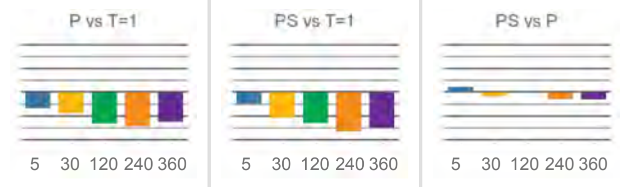

Locus		5m	30m	120m	240m	360m
**PGN_0191**	P vs T=1	-0.617	-1.103	-1.699	-1.650	-1.456
	PS vs T=1	-0.694	-1.627	-2.189	-2.053	-2.246
	PS vs P	-0.046	-0.508	-0.513	-0.410	-0.778

GTP-binding protein EngA

unknown function

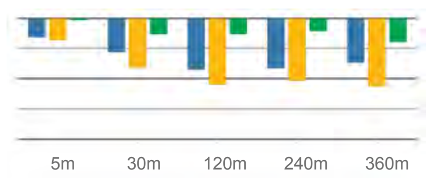

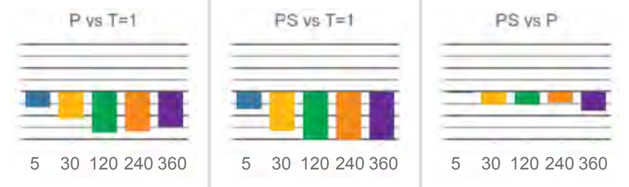

Locus		5m	30m	120m	240m	360m
**PGN_0192**	P vs T=1	0.178	0.465	0.263	-0.379	-0.669
	PS vs T=1	0.343	0.241	-0.507	-1.094	-1.057
	PS vs P	0.174	-0.192	-0.737	-0.719	-0.399

conserved hypothetical protein

hypothetical proteins-Conserved

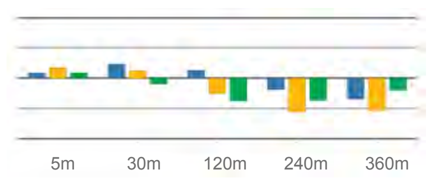

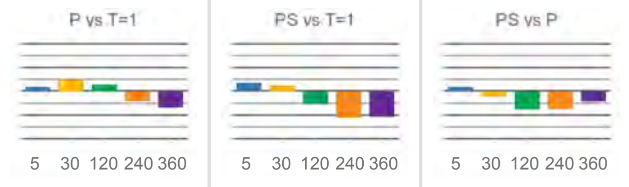

Locus		5m	30m	120m	240m	360m
**PGN_0193**	P vs T=1	-0.477	-0.492	-0.518	-0.354	-0.038
	PS vs T=1	0.102	-0.284	-1.205	-0.910	-0.868
	PS vs P	0.563	0.197	-0.679	-0.492	-0.740

putative polysaccharide deacetylase

energy metabolism

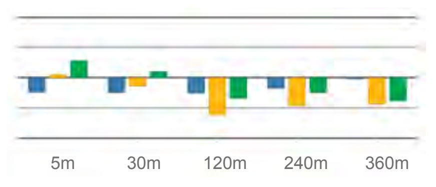

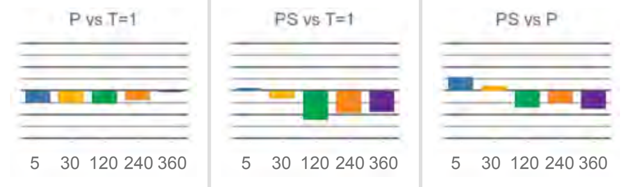

Locus		5m	30m	120m	240m	360m
**PGN_0194**	P vs T=1	-0.471	-0.367	-0.266	-0.438	0.238
	PS vs T=1	0.050	-0.114	-0.510	-0.368	-0.460
	PS vs P	0.494	0.244	-0.211	0.036	-0.596

conserved hypothetical protein

hypothetical proteins-Conserved

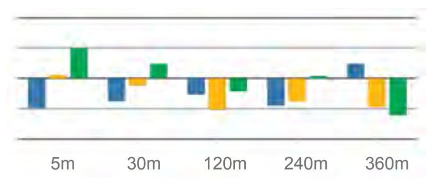

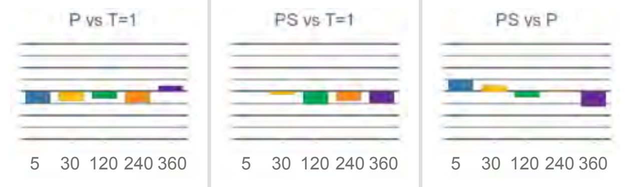

Locus		5m	30m	120m	240m	360m
**PGN_0195**	P vs T=1	-0.097	0.027	0.112	-0.009	-0.291
	PS vs T=1	-0.236	-0.183	0.116	0.499	0.329
	PS vs P	-0.140	-0.196	0.023	0.502	0.588

putative xanthine phosphoribosyltransferase

purines, pyrimidines, nucleosides and nucleotides

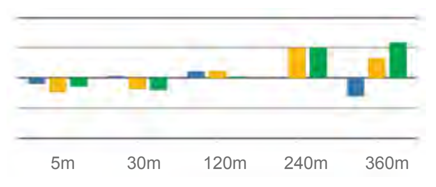

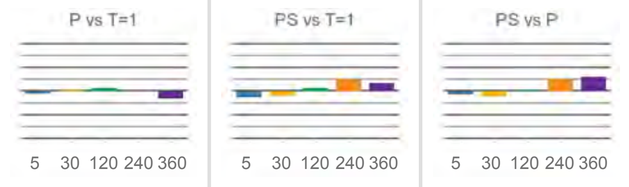

Locus		5m	30m	120m	240m	360m
**PGN_0196**	P vs T=1	0.305	0.236	0.285	0.452	0.886
	PS vs T=1	0.308	0.150	0.266	0.713	0.702
	PS vs P	0.004	-0.091	-0.029	0.264	-0.150

xanthine/uracil permease

transport and binding proteins

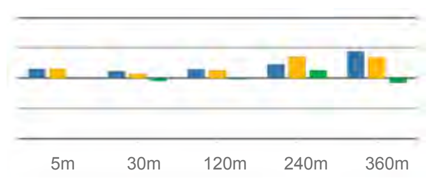

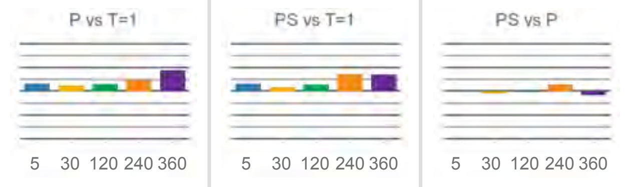

PgSg Time Course

log$_2$ (Fold Change)

Legend: P vs T=1 · PS vs T=1 · PS vs P

Locus		5m	30m	120m	240m	360m
**PGN_0197**	P vs T=1	-0.578	-0.381	-0.305	-0.548	-0.487
	PS vs T=1	-0.666	-0.992	-1.006	-0.881	-0.791
	PS vs P	-0.095	-0.583	-0.662	-0.341	-0.300

conserved hypothetical protein

hypothetical proteins-Conserved

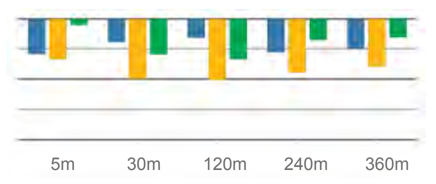

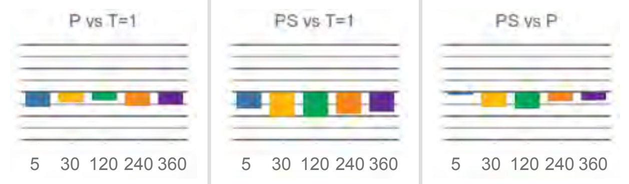

Locus		5m	30m	120m	240m	360m
**PGN_0198**	P vs T=1	0.068	0.171	-0.045	-0.161	-0.292
	PS vs T=1	-0.314	-0.382	-0.116	0.004	-0.176
	PS vs P	-0.372	-0.533	-0.069	0.156	0.106

conserved hypothetical protein

unknown function

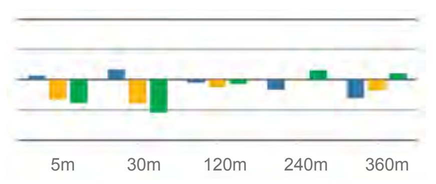

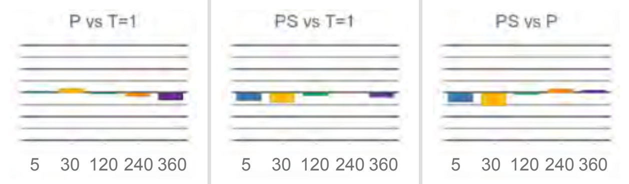

Locus		5m	30m	120m	240m	360m
**PGN_0199**	P vs T=1	0.314	0.975	1.639	1.829	1.458
	PS vs T=1	0.627	1.124	1.535	1.748	1.942
	PS vs P	0.222	0.136	-0.060	-0.023	0.485

hypothetical protein

hypothetical proteins

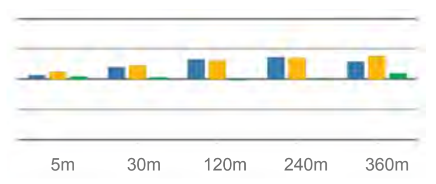

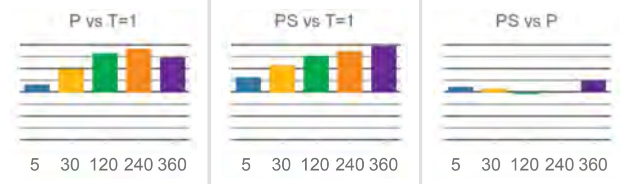

Locus		5m	30m	120m	240m	360m
**PGN_0200**	P vs T=1	1.054	1.506	1.557	1.124	0.557
	PS vs T=1	1.344	1.731	1.669	1.286	0.999
	PS vs P	0.275	0.243	0.138	0.156	0.412

conserved hypothetical protein

hypothetical proteins-Conserved

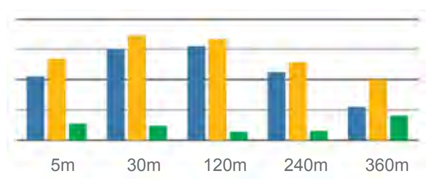

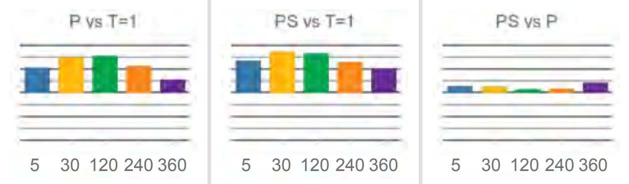

Locus		5m	30m	120m	240m	360m
**PGN_0201**	P vs T=1	0.623	0.776	0.672	0.379	0.215
	PS vs T=1	0.823	0.779	0.776	1.062	0.867
	PS vs P	0.194	0.011	0.105	0.640	0.617

conserved hypothetical protein with DUF558 domain

hypothetical proteins-Conserved

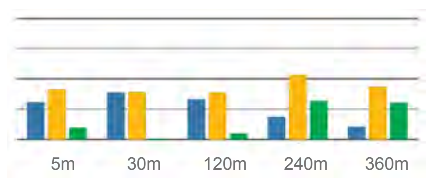

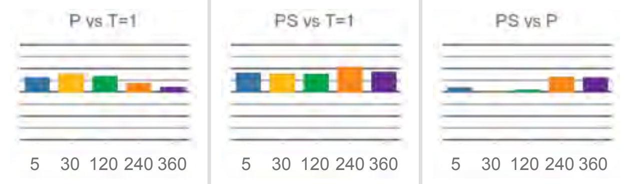

Locus		5m	30m	120m	240m	360m
**PGN_0202**	P vs T=1	-0.305	-0.151	-0.179	-0.239	-0.094
	PS vs T=1	-0.416	-0.542	-0.496	-0.246	-0.357
	PS vs P	-0.108	-0.356	-0.288	0.007	-0.230

probable leucine aminopeptidase precursor

unknown function

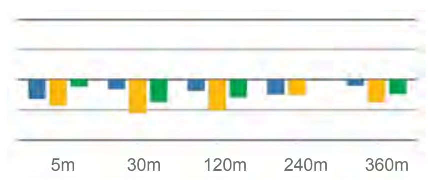

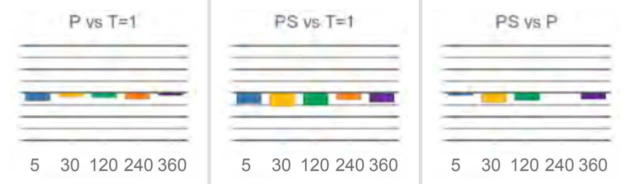

Locus		5m	30m	120m	240m	360m
**PGN_0203**	P vs T=1	-0.715	0.052	0.642	0.602	-0.151
	PS vs T=1	-0.395	-0.471	-0.482	-0.145	-0.100
	PS vs P	0.262	-0.496	-1.037	-0.648	0.029

probable SufE Fe/S-cluster-related protein

hypothetical proteins-Conserved

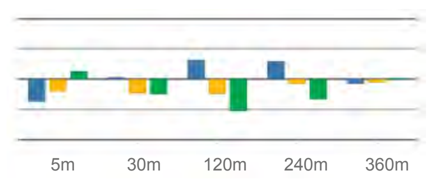

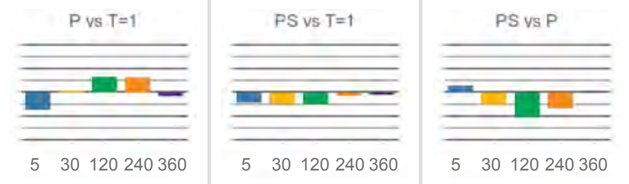

log$_2$ (Fold Change)

Legend: P vs T=1 | PS vs T=1 | PS vs P

Locus		5m	30m	120m	240m	360m
**PGN_0204**	P vs T=1	-0.966	-0.676	-0.469	-0.392	-0.375
HemY	PS vs T=1	-0.605	-0.791	-0.953	-0.986	-0.883
	PS vs P	0.309	-0.116	-0.455	-0.545	-0.479

protoporphyrinogen oxidase

biosynthesis of cofactors, prosthetic groups, and carriers

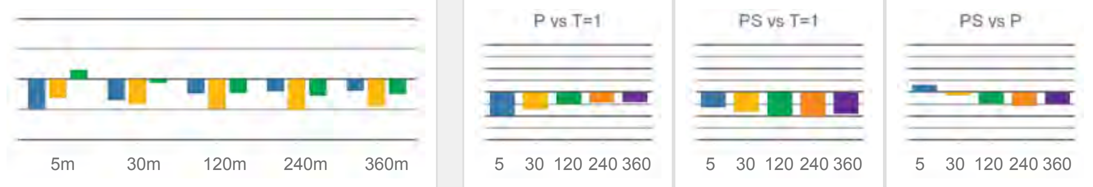

Locus		5m	30m	120m	240m	360m
**PGN_0205**	P vs T=1	-0.791	-1.172	-1.077	-0.579	0.819
	PS vs T=1	-0.445	-0.769	-0.768	-0.608	-0.283
	PS vs P	0.332	0.297	0.183	-0.013	-0.898

conserved hypothetical protein

hypothetical proteins-Conserved

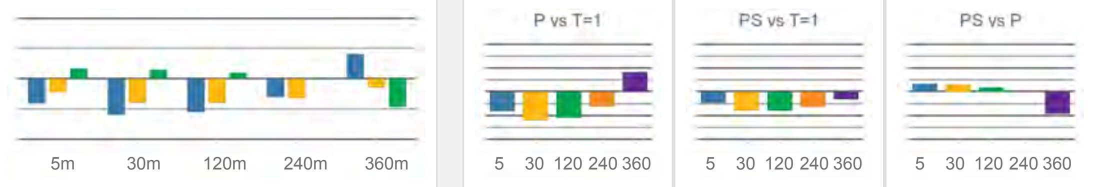

Locus		5m	30m	120m	240m	360m
**PGN_0206**	P vs T=1	-0.834	-0.727	-0.470	-0.203	0.356
	PS vs T=1	0.038	-0.186	-0.450	-0.354	-0.436
	PS vs P	0.796	0.479	-0.000	-0.123	-0.713

putative lipid A disaccharide synthase

cell envelope

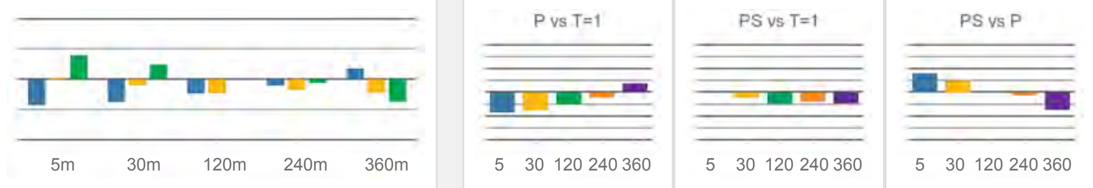

Locus		5m	30m	120m	240m	360m
**PGN_0207**	P vs T=1	-0.665	-0.463	-0.060	-0.522	-0.434
	PS vs T=1	0.031	-0.470	-0.602	-0.458	-0.745
	PS vs P	0.638	-0.019	-0.465	0.029	-0.306

probable stationary-phase survival protein

cellular processes

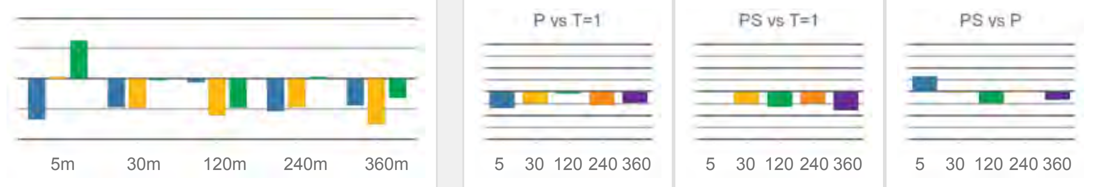

Locus		5m	30m	120m	240m	360m
**PGN_0208**	P vs T=1	0.371	0.927	0.942	0.457	-0.083
	PS vs T=1	0.636	0.625	0.645	0.629	0.303
	PS vs P	0.240	-0.247	-0.237	0.164	0.329

conserved hypothetical protein

protein fate

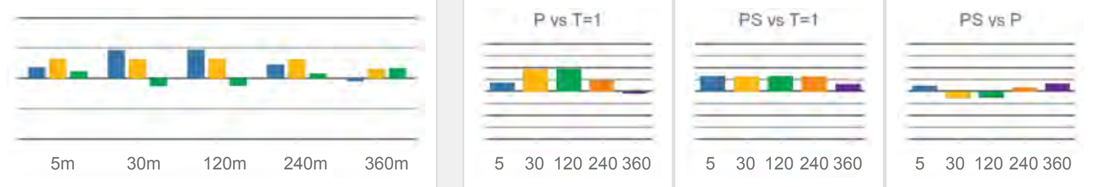

Locus		5m	30m	120m	240m	360m
**PGN_0209**	P vs T=1	-0.290	-0.425	-0.845	-1.094	-1.228
	PS vs T=1	-0.205	-0.163	-0.002	0.271	-0.070
	PS vs P	0.088	0.259	0.814	1.301	1.114

glycyl-tRNA synthetase

protein synthesis

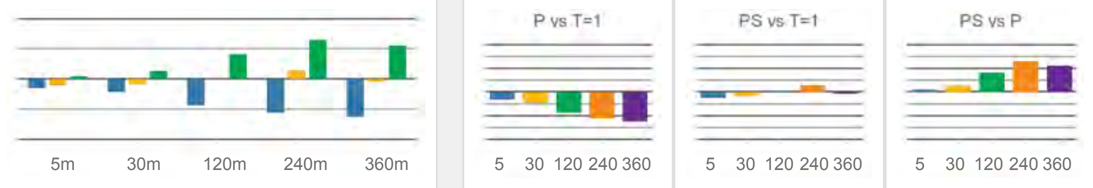

Locus		5m	30m	120m	240m	360m
**PGN_0210**	P vs T=1	0.358	0.279	0.374	0.405	-0.077
	PS vs T=1	0.193	0.011	0.143	0.239	0.269
	PS vs P	-0.164	-0.272	-0.233	-0.165	0.320

transposase in ISPg1

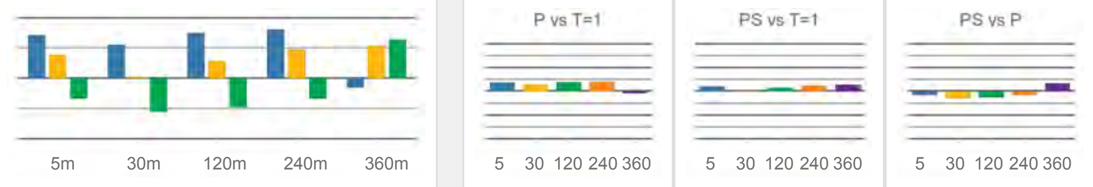

PgSg Time Course

log$_2$ (Fold Change)

Legend: P vs T=1 | PS vs T=1 | PS vs P

Locus		5m	30m	120m	240m	360m
**PGN_0211**	P vs T=1	-0.299	-0.240	-0.088	-0.103	-0.386
	PS vs T=1	-0.148	-0.135	0.436	0.732	0.668
	PS vs P	0.126	0.082	0.506	0.805	1.013
	conserved hypothetical protein					
	hypothetical proteins-Conserved					
**PGN_0212**	P vs T=1	0.338	0.260	0.370	0.513	0.212
	PS vs T=1	0.194	0.013	0.178	0.271	0.219
	PS vs P	-0.146	-0.255	-0.198	-0.238	-0.007
	transposase in ISPg1					
**PGN_0213**	P vs T=1	-0.414	-0.327	0.729	1.899	3.153
	PS vs T=1	-0.240	-0.269	0.739	0.997	1.878
	PS vs P	-0.208	-0.344	-0.082	-0.355	-0.669
	conserved hypothetical protein					
	hypothetical proteins-Conserved					
**PGN_0214**	P vs T=1	-2.311	-1.658	-2.208	-2.200	-2.288
	PS vs T=1	-2.601	-2.352	-2.488	-2.330	-2.234
	PS vs P	-0.408	-0.505	-0.332	-0.163	-0.018
	conserved hypothetical protein					
	hypothetical proteins-Conserved					
**PGN_0215**	P vs T=1	1.806	2.244	3.037	3.119	2.116
	PS vs T=1	2.277	2.728	2.800	3.039	3.007
	PS vs P	0.383	0.479	-0.074	0.078	0.854
	conserved hypothetical protein					
	hypothetical proteins-Conserved					
**PGN_0216**	P vs T=1	-1.741	-1.018	-0.508	-0.864	-1.464
	PS vs T=1	-1.607	-1.175	-1.068	-0.864	-0.699
	PS vs P	-0.169	-0.158	-0.412	-0.011	0.585
	conserved hypothetical protein					
	hypothetical proteins-Conserved					
**PGN_0217**	P vs T=1	-0.988	-0.746	-1.239	-1.704	-2.522
	PS vs T=1	-1.325	-1.277	-1.976	-2.929	-2.900
	PS vs P	-0.243	-0.350	-0.639	-1.179	-0.478
	conserved hypothetical protein					
	hypothetical proteins-Conserved					

PGN_0211

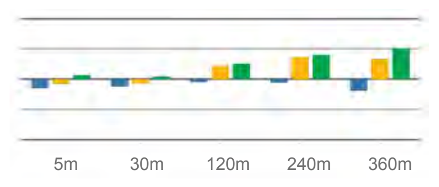

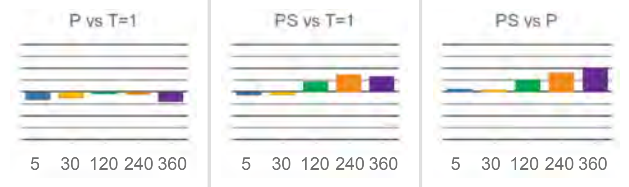

PGN_0212

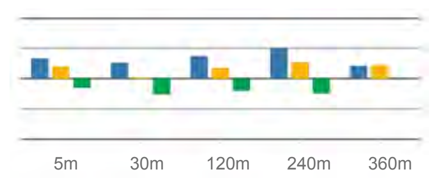

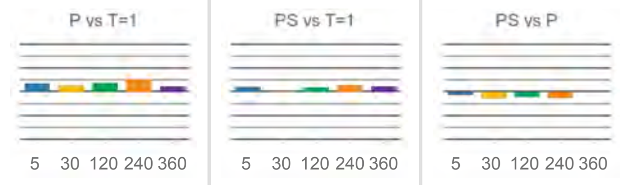

PGN_0213

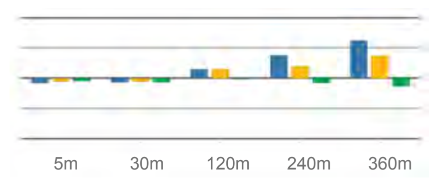

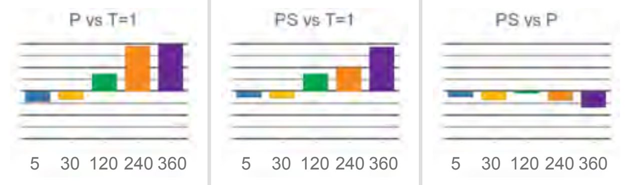

PGN_0214

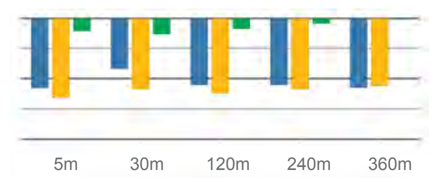

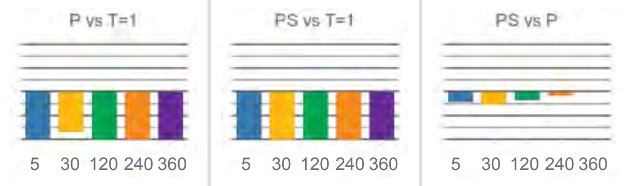

PGN_0215

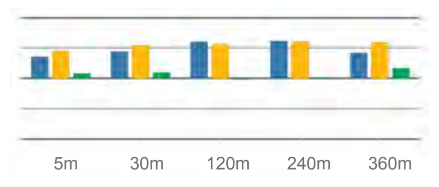

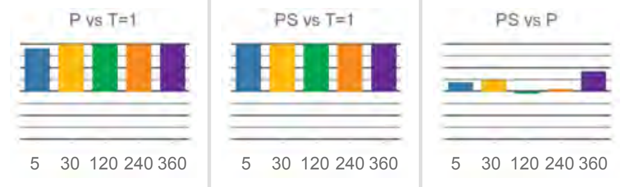

PGN_0216

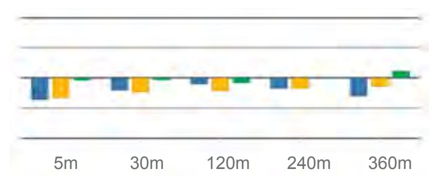

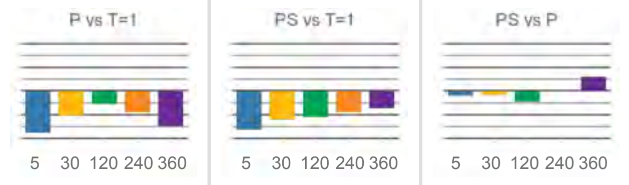

PGN_0217

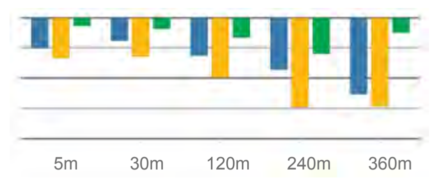

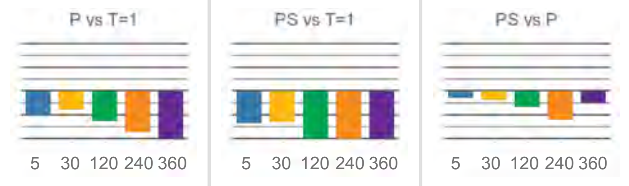

log$_2$ (Fold Change)

P vs T=1 | PS vs T=1 | PS vs P

Locus		5m	30m	120m	240m	360m
**PGN_0218**	P vs T=1	0.373	0.323	0.394	0.517	0.166
	PS vs T=1	0.171	-0.003	0.178	0.275	0.248
	PS vs P	-0.201	-0.330	-0.222	-0.236	0.062

transposase in ISPg1

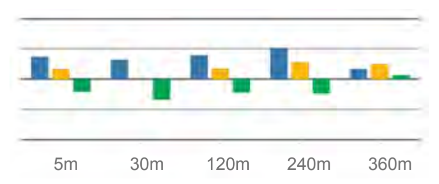

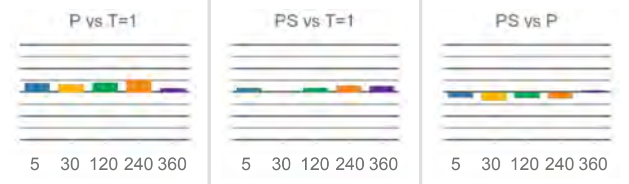

Locus		5m	30m	120m	240m	360m
**PGN_0219**	P vs T=1	0.525	0.443	-0.077	-0.266	-0.191
	PS vs T=1	-0.096	-0.623	-1.139	-0.822	-0.714
	PS vs P	-0.578	-1.015	-1.066	-0.589	-0.535

partial transposase in ISPg1

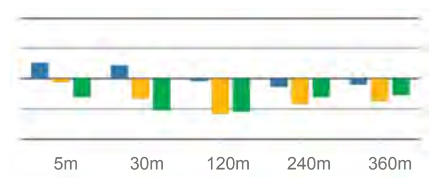

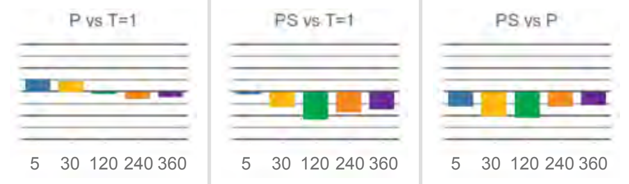

Locus		5m	30m	120m	240m	360m
**PGN_0220**	P vs T=1	1.130	1.065	0.955	0.862	0.780
	PS vs T=1	0.504	0.240	0.262	0.256	0.293
	PS vs P	-0.594	-0.793	-0.678	-0.597	-0.485

partial transposase in ISPg1

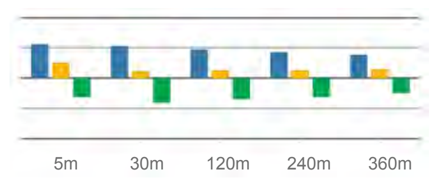

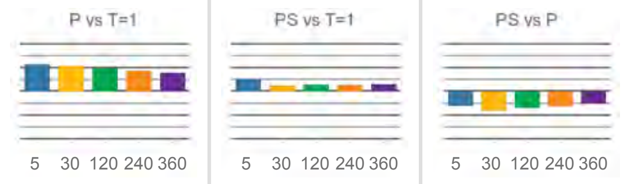

Locus		5m	30m	120m	240m	360m
**PGN_0221**	P vs T=1	0.446	0.597	0.836	0.911	0.585
	PS vs T=1	0.352	0.226	0.150	0.350	0.176
	PS vs P	-0.094	-0.358	-0.658	-0.527	-0.404

DNA topoisomerase III

DNA metabolism

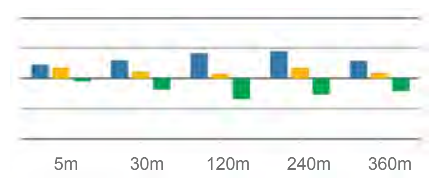

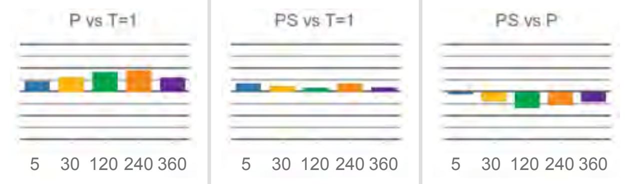

Locus		5m	30m	120m	240m	360m
**PGN_0222**	P vs T=1	0.304	0.330	0.269	1.892	3.059
	PS vs T=1	0.520	0.349	1.031	0.879	1.609
	PS vs P	0.058	-0.131	0.268	-0.451	-0.786

conserved hypothetical protein

hypothetical proteins-Conserved

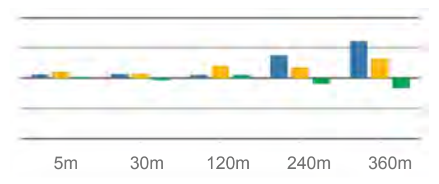

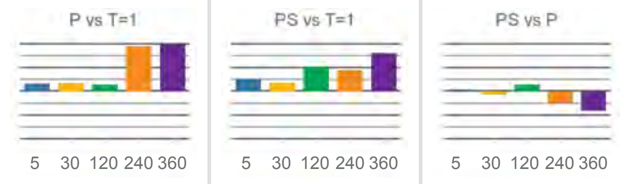

Locus		5m	30m	120m	240m	360m
**PGN_0223** *wecA*	P vs T=1	-1.163	-2.571	-2.832	-2.361	-1.219
	PS vs T=1	-1.889	-2.096	-1.793	-1.576	-1.860
	PS vs P	-0.582	0.310	0.804	0.676	-0.541

glycosyl transferase family 4

cell envelope

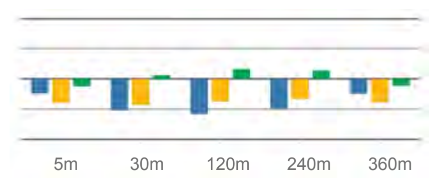

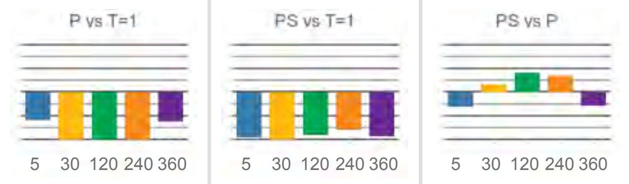

Locus		5m	30m	120m	240m	360m
**PGN_0224** *cap50*	P vs T=1	-1.629	-2.748	-3.497	-3.040	-2.599
	PS vs T=1	-1.920	-2.767	-3.228	-3.563	-3.270
	PS vs P	-0.155	-0.061	0.099	-0.547	-0.644

UDP-N-acetyl-D-mannosaminuronic acid dehydrogenase

cell envelope

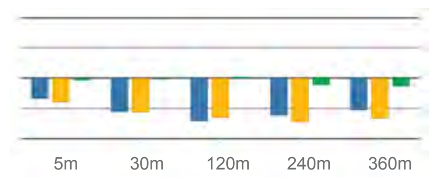

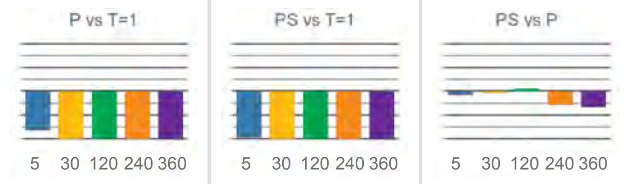

PgSg Time Course

log$_2$ (Fold Change)

Legend: P vs T=1 · PS vs T=1 · PS vs P

Locus		5m	30m	120m	240m	360m
**PGN_0225**	P vs T=1	-1.441	-3.032	-3.128	-2.778	-1.746
	PS vs T=1	-1.300	-2.025	-2.282	-2.458	-2.216
	PS vs P	0.299	0.645	0.529	0.139	-0.366
	probable glycosyltransferase					
	cell envelope					
**PGN_0226**	P vs T=1	-1.080	-2.078	-2.114	-1.678	-0.669
	PS vs T=1	-0.514	-1.248	-1.698	-1.727	-1.698
	PS vs P	0.625	0.589	0.222	-0.085	-0.853
	conserved hypothetical protein					
	hypothetical proteins-Conserved					
**PGN_0227**	P vs T=1	-0.981	-1.524	-1.714	-1.411	-1.098
	PS vs T=1	-0.758	-1.319	-2.236	-2.149	-2.068
	PS vs P	0.292	0.162	-0.564	-0.674	-0.882
	probable glycosyl transferase family 1					
	cell envelope					
**PGN_0228**	P vs T=1	-1.062	-2.123	-2.537	-2.622	-1.715
	PS vs T=1	-0.856	-1.066	-2.143	-2.509	-2.394
	PS vs P	0.296	0.945	0.267	-0.041	-0.632
	probable coenzyme F390 synthetase					
	biosynthesis of cofactors, prosthetic groups, and carriers					
**PGN_0229**	P vs T=1	-1.033	-0.798	-0.347	-0.432	-1.175
	PS vs T=1	-0.823	-0.399	-0.059	-0.455	-0.640
	PS vs P	0.162	0.371	0.310	-0.006	0.483
	putative DNA-binding protein histone-like family					
	hypothetical proteins-Conserved					
**PGN_0230**	P vs T=1	-0.625	-1.232	-1.838	-1.809	-1.570
	PS vs T=1	-0.680	-0.783	-1.480	-1.952	-1.940
	PS vs P	0.026	0.424	0.238	-0.231	-0.384
	probable serine acetyltransferase					
	cellular processes					
**PGN_0231**	P vs T=1	-0.937	-1.358	-1.963	-1.893	-1.368
	PS vs T=1	-0.474	-0.619	-1.822	-2.231	-2.230
	PS vs P	0.485	0.712	0.042	-0.406	-0.831
	probable delta-aminolevulinic acid dehydratase					
	biosynthesis of cofactors, prosthetic groups, and carriers					

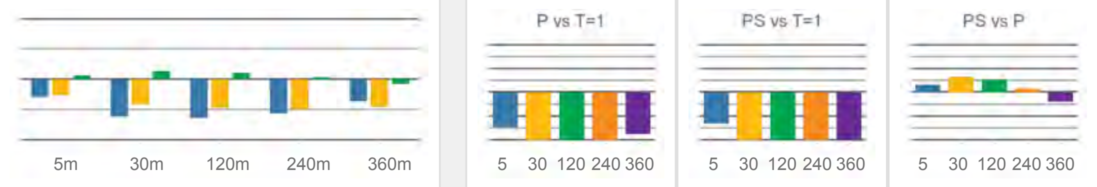

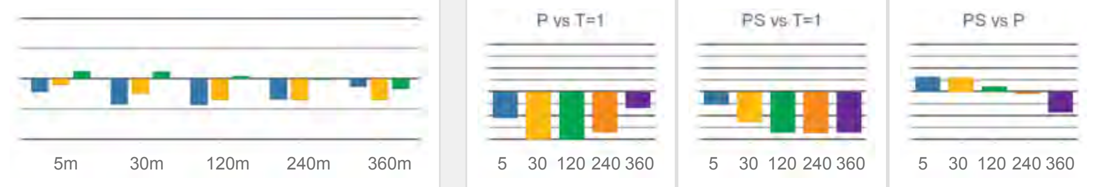

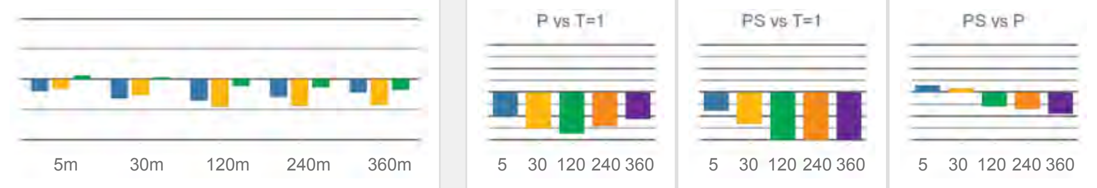

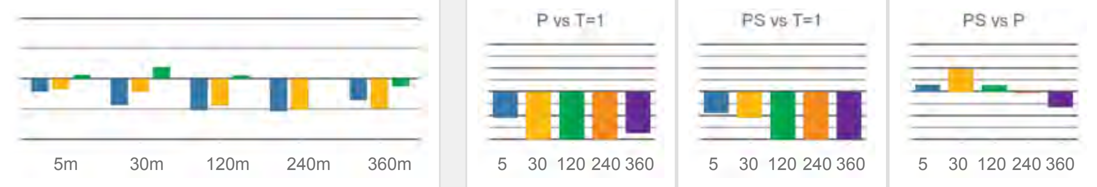

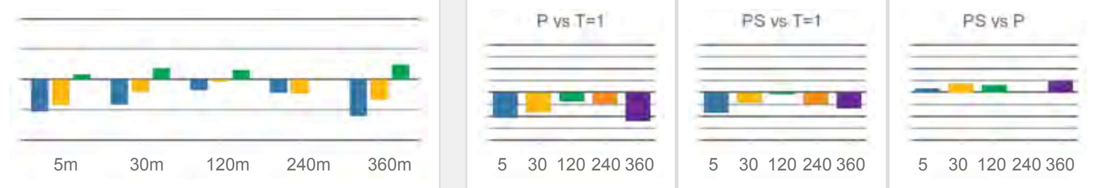

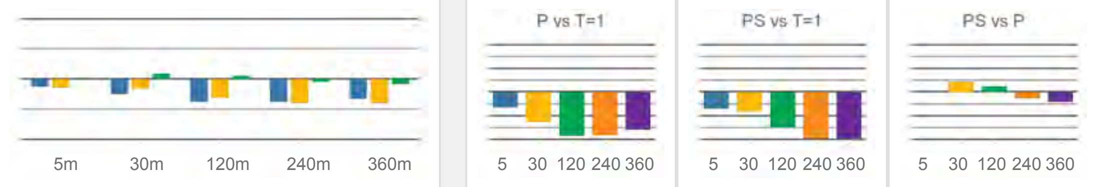

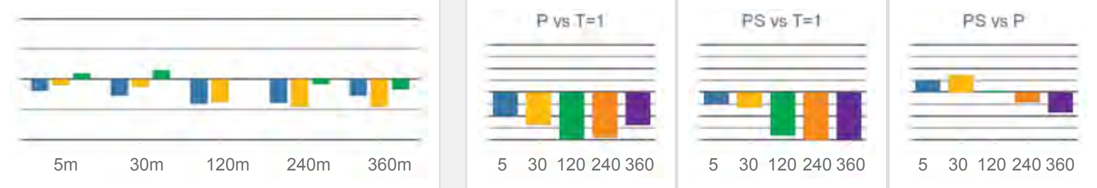

P vs T=1 | PS vs T=1 | PS vs P

log2 (Fold Change)

Locus		5m	30m	120m	240m	360m
**PGN_0232**	P vs T=1	-0.916	-1.476	-1.919	-1.997	-1.544
	PS vs T=1	-0.447	-0.500	-1.128	-1.472	-1.456
	PS vs P	0.491	0.877	0.636	0.343	0.061

probable glycosyl transferase family 2

cell envelope

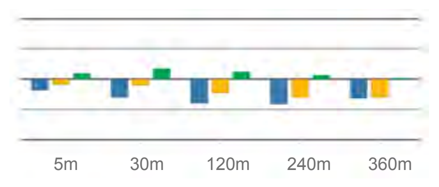

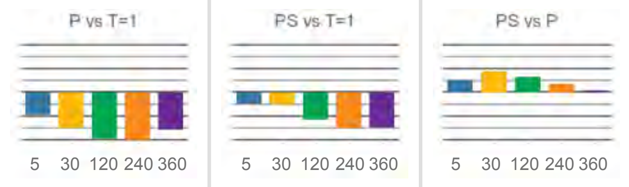

Locus		5m	30m	120m	240m	360m
**PGN_0233**	P vs T=1	-0.782	-1.525	-2.078	-2.374	-2.260
	PS vs T=1	-0.323	-0.621	-1.110	-1.780	-2.154
	PS vs P	0.515	0.834	0.838	0.374	-0.004

conserved hypothetical protein with glycosyl transferase WecB/TagA/CpsF family domain

cell envelope

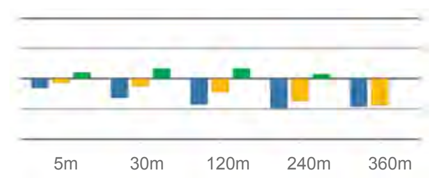

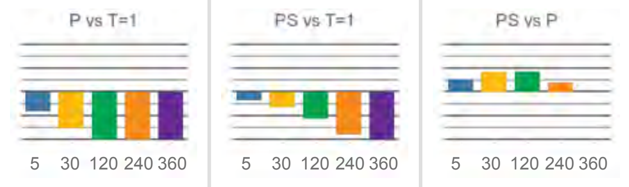

Locus		5m	30m	120m	240m	360m
**PGN_0234**	P vs T=1	-0.997	-1.316	-1.599	-2.100	-2.411
	PS vs T=1	-0.673	-0.645	-1.219	-1.834	-2.173
	PS vs P	0.332	0.658	0.368	0.204	0.180

UDP-N-acetylglucosamine 2-epimerase

cell envelope

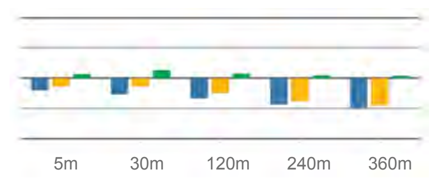

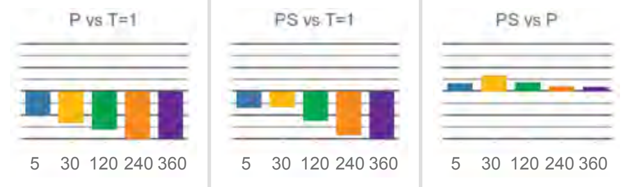

Locus		5m	30m	120m	240m	360m
**PGN_0235**	P vs T=1	-0.814	-0.727	-0.166	-0.057	-0.833
	PS vs T=1	-0.983	-0.599	-0.348	-0.405	-0.367
	PS vs P	-0.199	0.098	-0.144	-0.298	0.431

DNA-binding protein HU

DNA metabolism

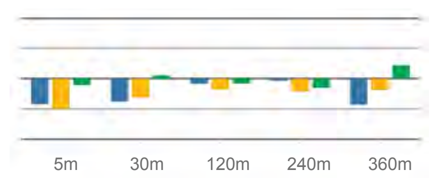

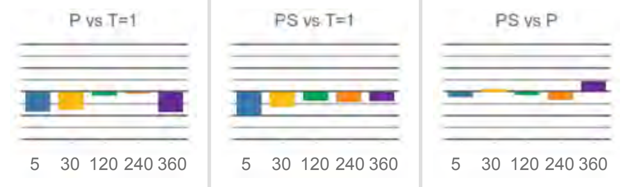

Locus		5m	30m	120m	240m	360m
**PGN_0236**	P vs T=1	-0.362	-0.891	-1.274	-1.028	-0.506
	PS vs T=1	-0.045	-0.216	-0.732	-0.746	-0.926
	PS vs P	0.343	0.621	0.400	0.214	-0.371

conserved hypothetical protein

hypothetical proteins-Conserved

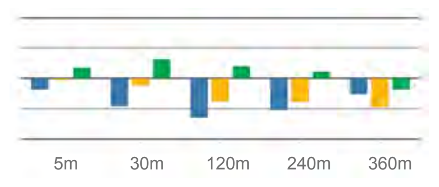

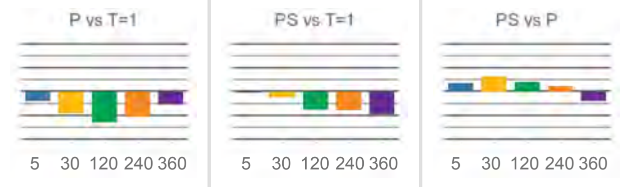

Locus		5m	30m	120m	240m	360m
**PGN_0237**	P vs T=1	-0.837	-0.875	-0.916	-0.660	-0.336
	PS vs T=1	-0.339	-0.634	-0.910	-0.720	-0.674
	PS vs P	0.469	0.207	-0.051	-0.064	-0.305

conserved hypothetical protein

hypothetical proteins-Conserved

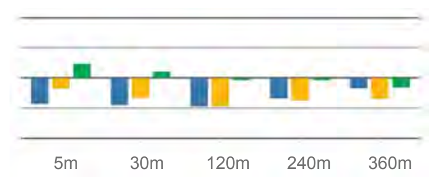

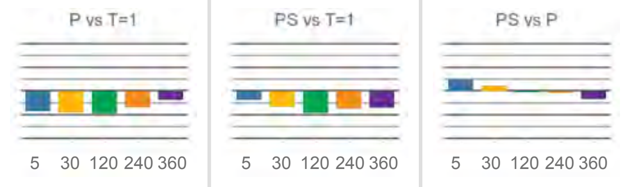

Locus		5m	30m	120m	240m	360m
**PGN_0238**	P vs T=1	-0.126	0.306	0.828	1.032	1.054
	PS vs T=1	0.170	-0.015	0.113	0.315	0.406
	PS vs P	0.236	-0.310	-0.632	-0.583	-0.565

conserved hypothetical protein

hypothetical proteins-Conserved

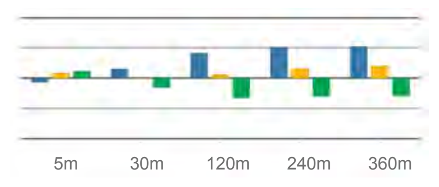

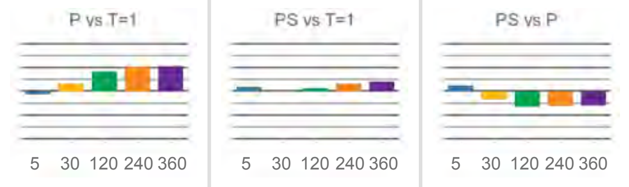

PgSg Time Course

log$_2$ (Fold Change)

Legend: P vs T=1 | PS vs T=1 | PS vs P

PGN_0239

Locus	5m	30m	120m	240m	360m
P vs T=1	-0.836	-0.809	-0.765	-0.521	-0.285
PS vs T=1	-0.701	-0.802	-0.527	0.021	-0.012
PS vs P	0.117	-0.011	0.203	0.528	0.279

putative type I phosphodiesterase-nucleotide pyrophosphatase

central intermediary metabolism

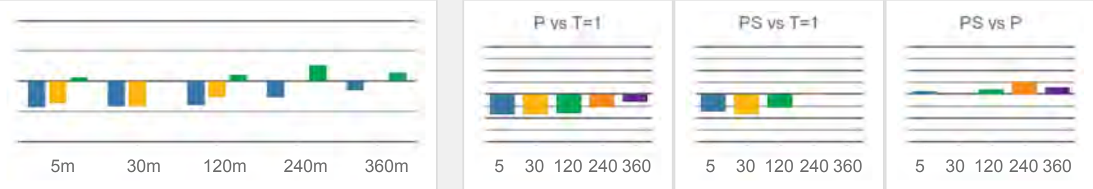

PGN_0240

HemH

Locus	5m	30m	120m	240m	360m
P vs T=1	-0.330	-0.571	-0.418	-0.371	0.052
PS vs T=1	-0.247	-0.407	0.054	0.486	0.468
PS vs P	0.065	0.098	0.413	0.768	0.421

putative ferrochelatase

biosynthesis of cofactors, prosthetic groups, and carriers

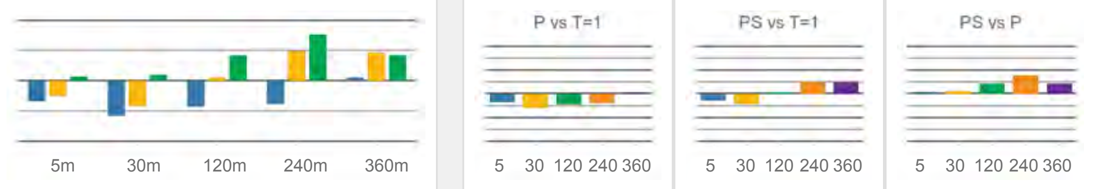

PGN_0241

Locus	5m	30m	120m	240m	360m
P vs T=1	-0.630	-0.410	0.291	0.618	0.817
PS vs T=1	-0.488	-0.355	-0.411	-0.129	0.156
PS vs P	0.052	-0.002	-0.605	-0.568	-0.530

conserved hypothetical protein

hypothetical proteins-Conserved

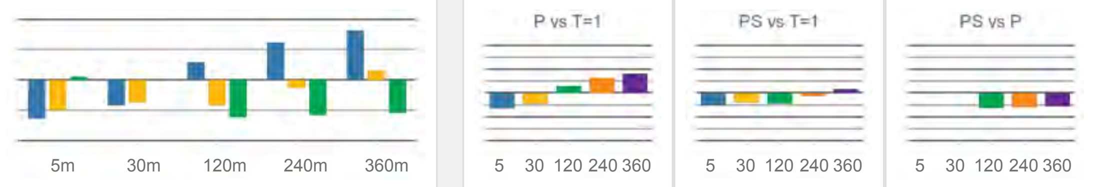

PGN_0242

Locus	5m	30m	120m	240m	360m
P vs T=1	-0.294	0.055	0.930	1.332	1.445
PS vs T=1	0.251	0.186	0.370	0.829	0.804
PS vs P	0.428	0.068	-0.493	-0.382	-0.555

probable glycosyl transferase family 1

cell envelope

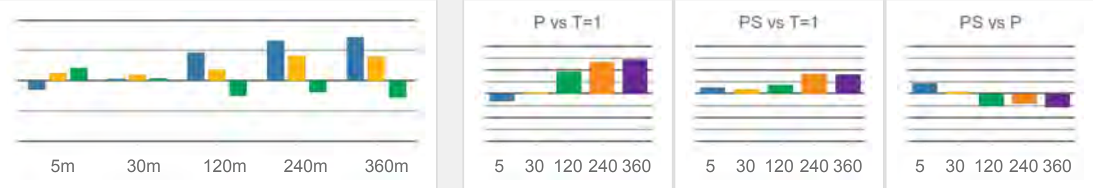

PGN_0243

Locus	5m	30m	120m	240m	360m
P vs T=1	1.556	1.788	1.595	1.010	0.547
PS vs T=1	2.131	2.337	2.319	2.079	1.912
PS vs P	0.566	0.571	0.738	1.001	1.288

phosphoglycerate mutase

energy metabolism

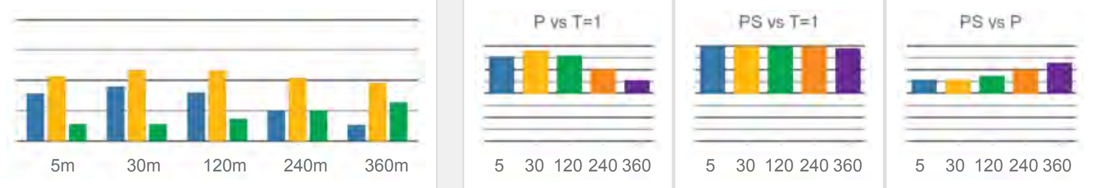

PGN_0244

Locus	5m	30m	120m	240m	360m
P vs T=1	0.043	0.685	0.684	-0.052	2.349
PS vs T=1	0.390	0.750	0.971	0.891	0.836
PS vs P	0.092	0.132	0.306	0.228	-0.734

hypothetical protein

hypothetical proteins

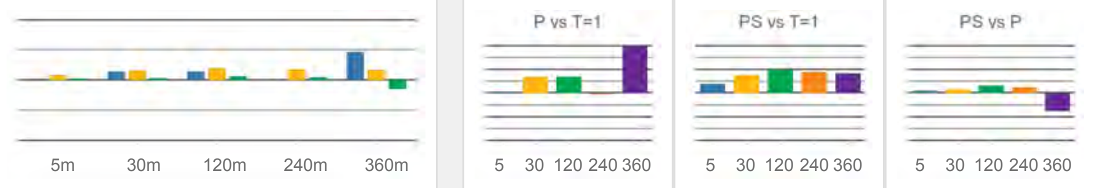

PGN_0245

Locus	5m	30m	120m	240m	360m
P vs T=1	-0.690	-0.606	-0.985	-1.214	-0.219
PS vs T=1	-0.310	-0.596	-1.620	-1.642	-1.572
PS vs P	0.376	0.030	-0.648	-0.513	-1.250

hypothetical protein

hypothetical proteins

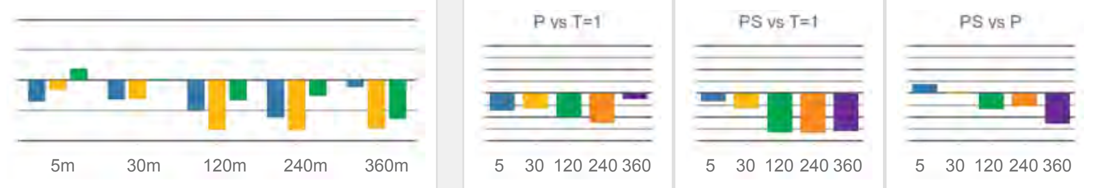

log$_2$ (Fold Change)

Legend: P vs T=1 | PS vs T=1 | PS vs P

Locus		5m	30m	120m	240m	360m
**PGN_0246**	P vs T=1	-0.220	-0.336	-0.517	-0.459	0.232
	PS vs T=1	-0.056	-0.539	-1.182	-1.102	-1.096
	PS vs P	0.180	-0.190	-0.664	-0.627	-1.238

conserved hypothetical protein

hypothetical proteins-Conserved

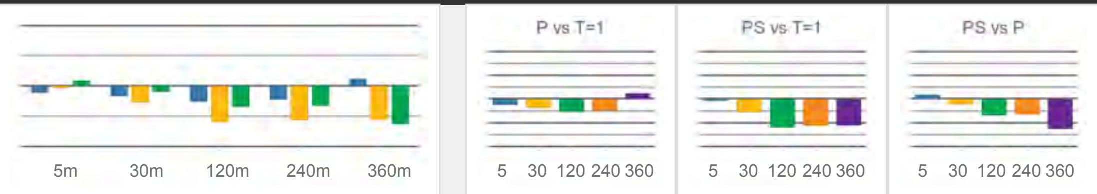

Locus		5m	30m	120m	240m	360m
**PGN_0247**	P vs T=1	0.121	0.306	0.125	-0.160	-0.264
	PS vs T=1	0.382	-0.217	-0.867	-1.103	-0.707
	PS vs P	0.266	-0.501	-0.970	-0.935	-0.443

putative magnesium transporter

transport and binding proteins

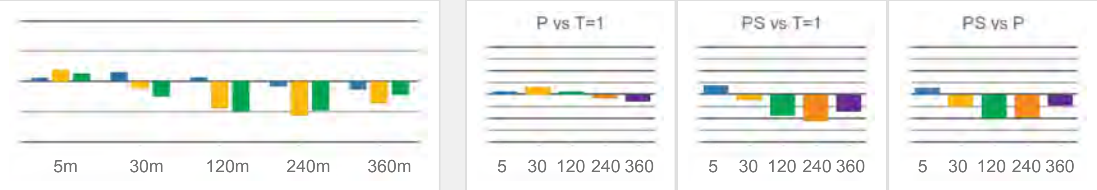

Locus		5m	30m	120m	240m	360m
**PGN_0248**	P vs T=1	0.225	-0.261	-0.762	-1.218	-1.125
	PS vs T=1	0.067	-0.962	-1.538	-1.172	-1.347
	PS vs P	-0.109	-0.672	-0.770	-0.015	-0.241

putative dimethyladenosine transferase

protein synthesis

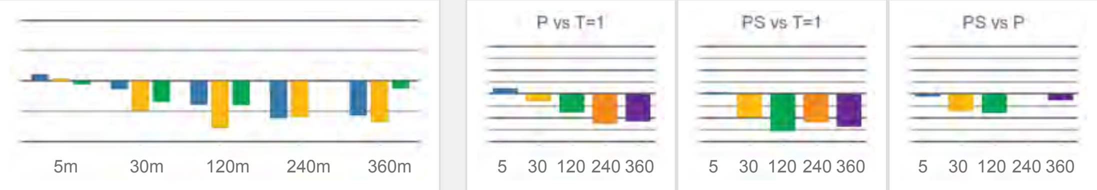

Locus		5m	30m	120m	240m	360m
**PGN_0249**	P vs T=1	-0.618	-0.951	-0.946	-1.202	-1.218
	PS vs T=1	-0.760	-0.809	-0.852	-0.700	-0.826
	PS vs P	-0.132	0.132	0.092	0.463	0.369

conserved hypothetical protein

hypothetical proteins-Conserved

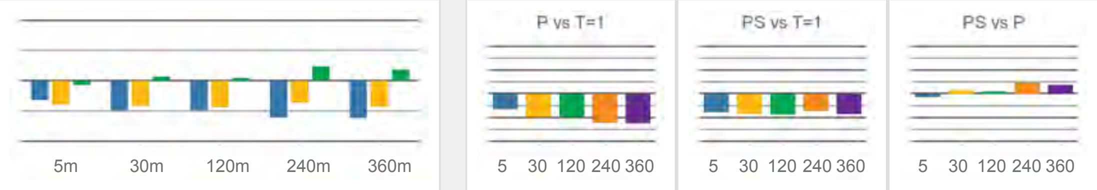

Locus		5m	30m	120m	240m	360m
**PGN_0250**	P vs T=1	0.016	0.586	0.999	0.595	-0.047
	PS vs T=1	0.593	0.646	0.434	0.196	-0.137
	PS vs P	0.539	0.077	-0.508	-0.376	-0.109

aminoacyl-histidine dipeptidase

protein fate

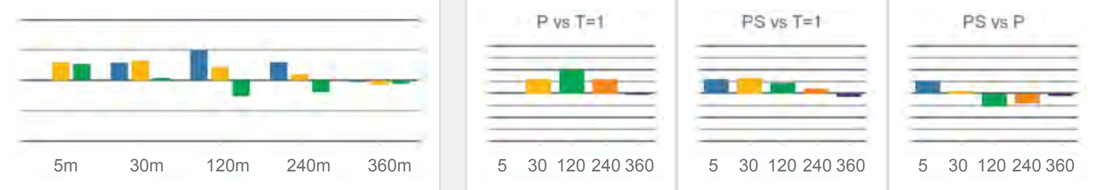

Locus		5m	30m	120m	240m	360m
**PGN_0251**	P vs T=1	1.115	1.693	2.047	1.737	1.287
	PS vs T=1	2.182	2.520	2.280	1.872	1.494
	PS vs P	1.004	0.820	0.268	0.141	0.189

malonyl CoA-acyl carrier protein transacylase

fatty acid and phospholipid metabolism

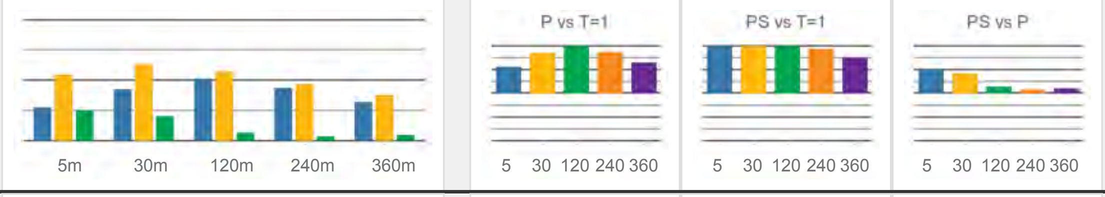

Locus		5m	30m	120m	240m	360m
**PGN_0252**	P vs T=1	0.159	0.032	-0.113	-0.203	0.037
	PS vs T=1	1.009	0.636	-0.007	-0.220	-0.254
	PS vs P	0.843	0.589	0.092	-0.042	-0.283

putative membrane-bound lytic murein transglycosylase D

cell envelope

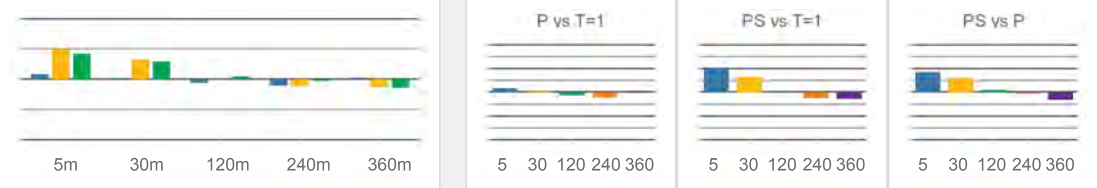

PgSg Time Course

log$_2$ (Fold Change)

Locus		5m	30m	120m	240m	360m
PGN_0253	P vs T=1	-1.383	-1.154	-0.516	0.062	0.364
	PS vs T=1	-1.159	-0.934	-0.735	-0.882	-0.349
	PS vs P	0.049	0.088	-0.210	-0.755	-0.570
	conserved hypothetical protein					
	hypothetical proteins-Conserved					
PGN_0254	P vs T=1	-0.610	-0.013	0.723	0.935	0.563
	PS vs T=1	-0.533	-0.596	-0.349	-0.242	-0.130
	PS vs P	-0.009	-0.568	-0.980	-1.056	-0.656
	putative ParB chromosome partitioning protein					
	cellular processes					
PGN_0255	P vs T=1	-1.193	-1.045	-0.473	-0.267	-0.457
	PS vs T=1	-1.019	-1.081	-0.622	-0.622	-0.254
	PS vs P	0.111	-0.085	-0.111	-0.270	0.206
	putative ParA chromosome partitioning protein					
	regulatory functions					
PGN_0256	P vs T=1	-2.822	-2.086	-1.226	-1.066	-1.416
	PS vs T=1	-1.882	-1.765	-1.811	-1.503	-1.602
	PS vs P	0.463	0.146	-0.495	-0.328	-0.215
	hydrolase					
	unknown function					
PGN_0257	P vs T=1	-1.878	-1.877	-0.915	-0.323	-0.357
	PS vs T=1	-1.430	-1.453	-1.104	-0.533	-0.392
	PS vs P	0.278	0.220	-0.189	-0.095	0.004
	putative arginine deiminase					
	central intermediary metabolism					
PGN_0258	P vs T=1	-1.660	-1.129	-1.003	-1.245	-1.915
	PS vs T=1	-1.744	-1.386	-1.306	-1.498	-1.622
	PS vs P	-0.146	-0.231	-0.268	-0.248	0.230
	hypothetical protein					
	hypothetical proteins-Conserved					
PGN_0259	P vs T=1	-0.959	-0.814	-0.104	-0.228	0.041
	PS vs T=1	-0.419	-0.187	-0.219	-0.379	-0.265
	PS vs P	0.404	0.497	-0.063	-0.165	-0.263
	conserved hypothetical protein					
	hypothetical proteins-Conserved					

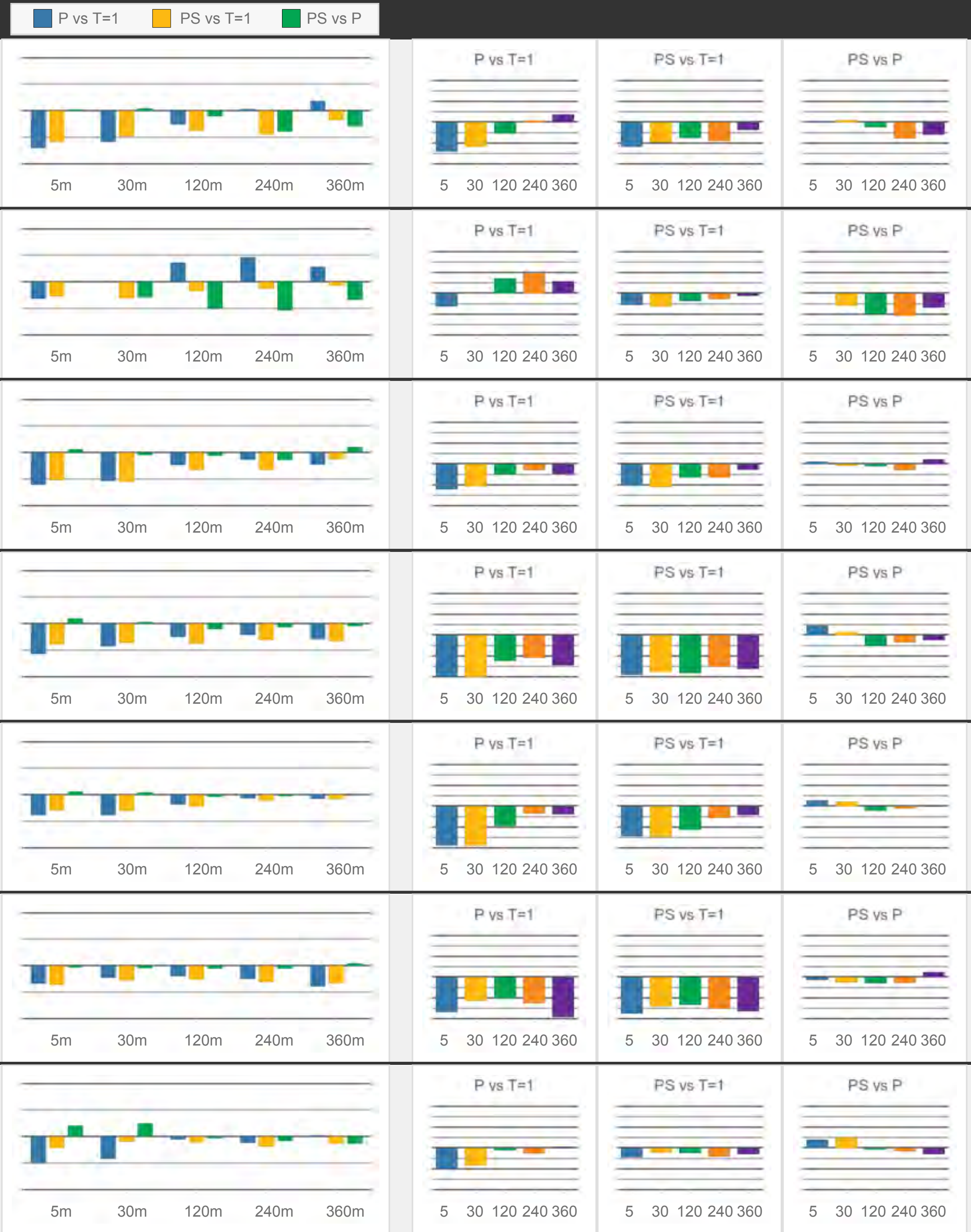

log2 (Fold Change)

Legend: P vs T=1 · PS vs T=1 · PS vs P

Locus		5m	30m	120m	240m	360m
**PGN_0260**	P vs T=1	-1.945	-1.349	-0.893	-0.716	-0.609
	PS vs T=1	-1.600	-1.435	-1.319	-1.294	-1.185
	PS vs P	0.196	-0.118	-0.393	-0.505	-0.530
	conserved hypothetical protein					
	hypothetical proteins-Conserved					
**PGN_0261**	P vs T=1	-1.261	-0.968	-0.428	-0.109	-0.214
	PS vs T=1	-1.023	-1.090	-1.141	-0.998	-0.972
	PS vs P	0.159	-0.150	-0.660	-0.775	-0.706
	sigma-54-dependent transcriptional regulator					
	regulatory functions					
**PGN_0262**	P vs T=1	-1.037	-0.945	-0.383	-0.386	-0.062
	PS vs T=1	-0.698	-0.917	-1.093	-1.158	-0.870
	PS vs P	0.301	-0.002	-0.667	-0.731	-0.759
	conserved hypothetical protein					
	hypothetical proteins-Conserved					
**PGN_0263**	P vs T=1	-0.387	-0.009	0.351	0.179	-0.147
	PS vs T=1	-0.548	-0.579	-1.028	-0.916	-0.863
	PS vs P	-0.175	-0.539	-1.307	-1.036	-0.706
	putative Fe-S oxidoreductase					
	hypothetical proteins-Conserved					
**PGN_0264**	P vs T=1	-0.112	0.438	1.304	1.349	0.727
	PS vs T=1	0.181	0.541	0.808	0.737	0.310
	PS vs P	0.220	0.084	-0.433	-0.542	-0.418
	signal recognition particle-docking protein					
	protein fate					
**PGN_0265**	P vs T=1	-0.580	-0.098	0.558	0.760	0.319
	PS vs T=1	-0.472	-0.320	-0.299	0.049	0.073
	PS vs P	0.041	-0.228	-0.794	-0.624	-0.236
	carboxynorspermidine decarboxylase					
	central intermediary metabolism					
**PGN_0266**	P vs T=1	-0.405	0.183	0.891	1.083	0.771
	PS vs T=1	-0.125	-0.087	0.061	0.562	0.526
	PS vs P	0.187	-0.275	-0.763	-0.440	-0.225
	aspartyl-tRNA synthetase					
	protein synthesis					

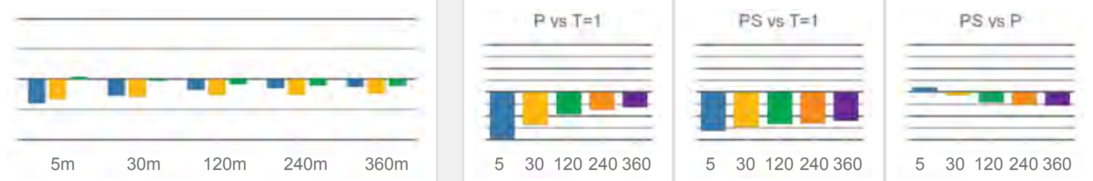

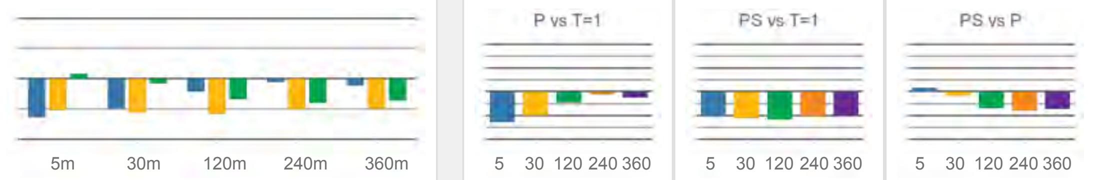

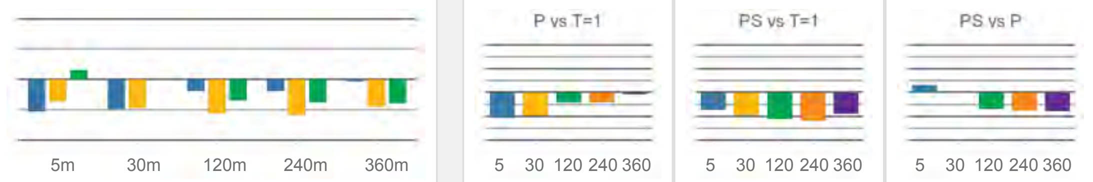

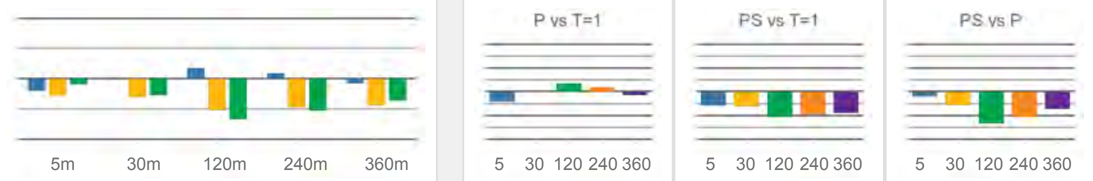

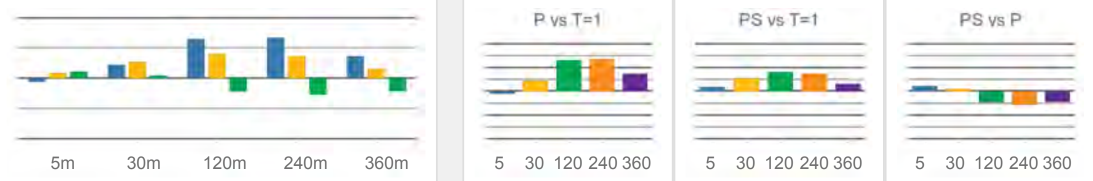

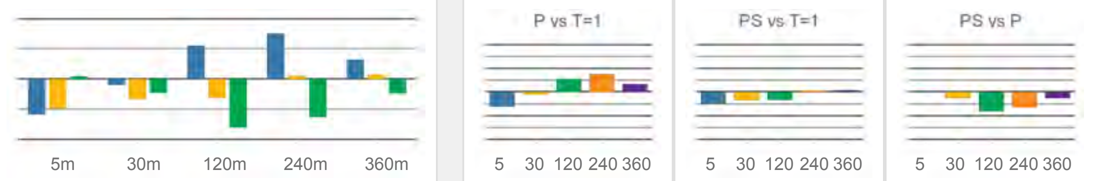

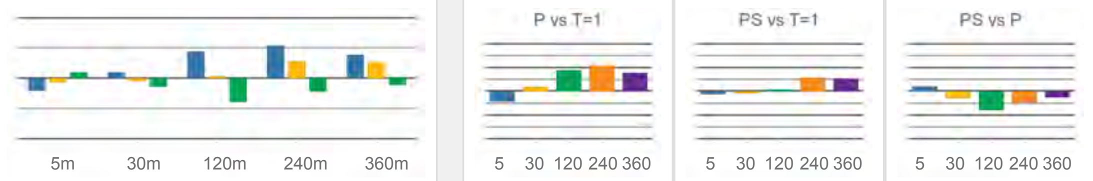

log$_2$ (Fold Change)

Legend: P vs T=1 | PS vs T=1 | PS vs P

Locus		5m	30m	120m	240m	360m
**PGN_0267**	P vs T=1	-0.929	-0.886	-0.623	-0.226	0.272
RibD	PS vs T=1	-0.782	-1.121	-0.682	-0.361	-0.357
	PS vs P	0.102	-0.272	-0.082	-0.071	-0.534

putative riboflavin biosynthesis protein

biosynthesis of cofactors, prosthetic groups, and carriers

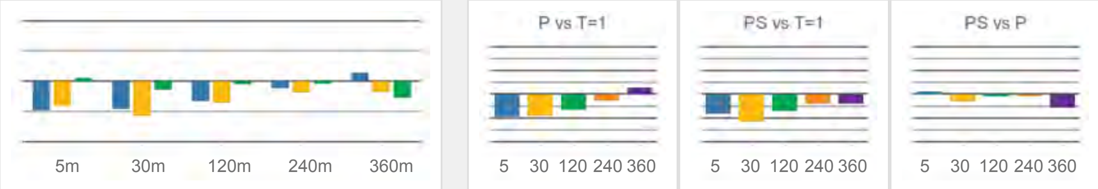

Locus		5m	30m	120m	240m	360m
**PGN_0268**	P vs T=1	-0.648	-0.922	-0.719	-0.597	-0.287
	PS vs T=1	-0.313	-0.700	-1.034	-0.572	-0.779
	PS vs P	0.327	0.177	-0.326	0.026	-0.456

protoporphyrinogen oxidase

biosynthesis of cofactors, prosthetic groups, and carriers

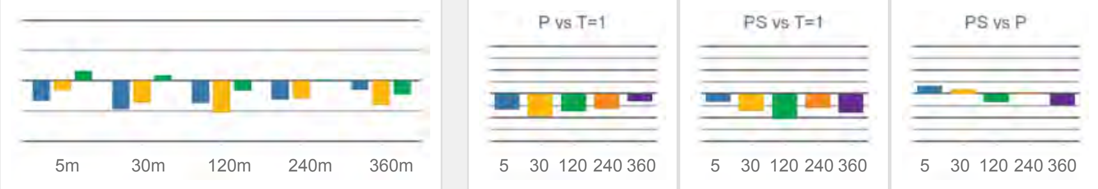

Locus		5m	30m	120m	240m	360m
**PGN_0269**	P vs T=1	-0.152	-0.283	0.348	0.676	0.696
	PS vs T=1	0.307	-0.052	-0.229	0.047	0.535
	PS vs P	0.391	0.110	-0.531	-0.490	-0.095

putative transcriptional regulatory protein

regulatory functions

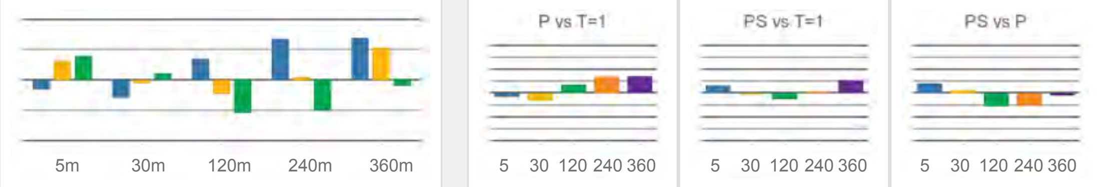

Locus		5m	30m	120m	240m	360m
**PGN_0270**	P vs T=1	0.270	0.372	1.053	1.270	1.334
	PS vs T=1	0.530	0.405	0.248	0.660	0.581
	PS vs P	0.228	0.005	-0.735	-0.505	-0.679

probable amidophosphoribosyl-transferase

purines, pyrimidines, nucleosides and nucleotides

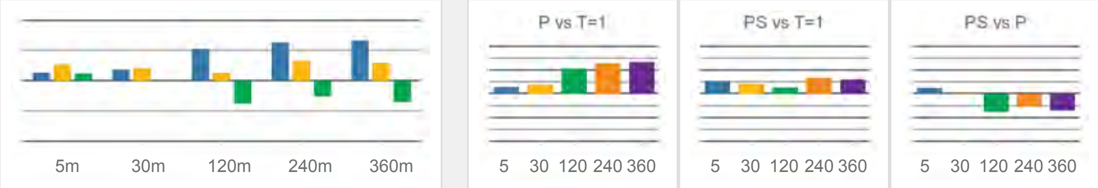

Locus		5m	30m	120m	240m	360m
**PGN_0271**	P vs T=1	0.562	0.778	0.539	-0.128	-1.053
	PS vs T=1	0.889	0.883	-0.091	-0.837	-0.995
	PS vs P	0.342	0.142	-0.593	-0.709	0.018

endopeptidase PepO

protein fate

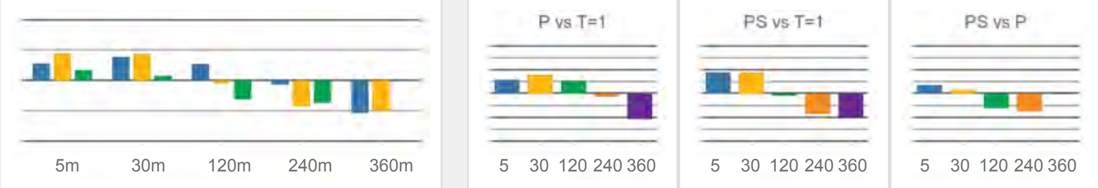

Locus		5m	30m	120m	240m	360m
**PGN_0272**	P vs T=1	0.692	1.071	0.839	0.109	-0.551
	PS vs T=1	1.102	1.140	0.610	0.298	-0.073
	PS vs P	0.409	0.108	-0.193	0.155	0.412

conserved hypothetical protein

hypothetical proteins-Conserved

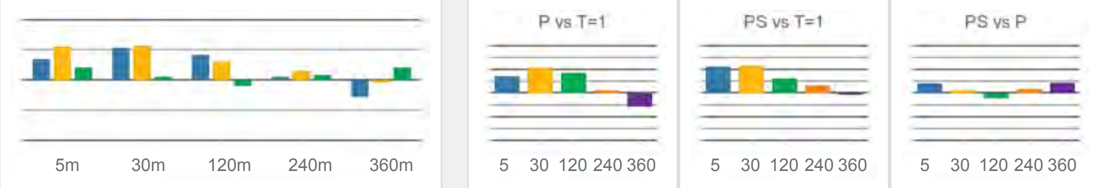

Locus		5m	30m	120m	240m	360m
**PGN_0273**	P vs T=1	0.911	0.508	0.441	0.309	-0.384
	PS vs T=1	2.493	2.745	2.746	1.856	1.589
	PS vs P	1.500	2.042	2.178	1.419	1.782

conserved hypothetical protein

hypothetical proteins-Conserved

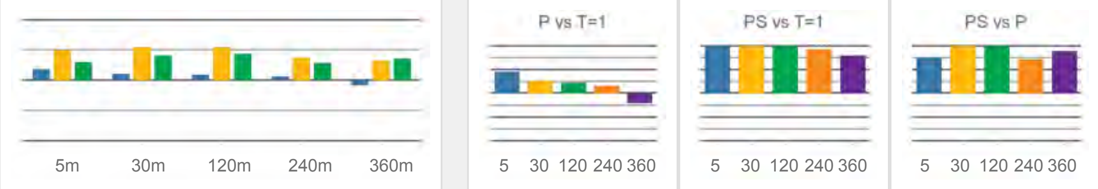

log$_2$ (Fold Change)

Legend: P vs T=1 | PS vs T=1 | PS vs P

PGN_0274

Locus	5m	30m	120m	240m	360m
P vs T=1	-0.310	-0.713	-1.533	-1.681	-1.986
PS vs T=1	0.090	-0.687	-1.143	-0.886	-0.981
PS vs P	0.419	0.027	0.336	0.725	0.938

putative RNA polymerase sigma-70 factor ECF subfamily

transcription

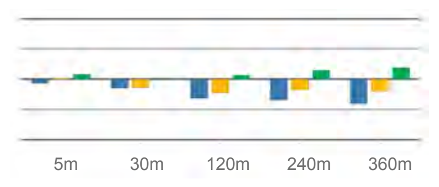

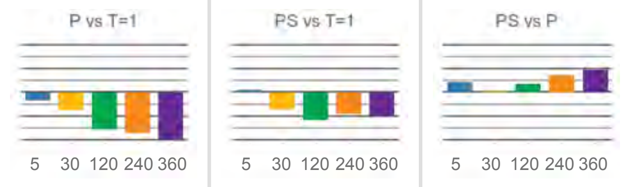

PGN_0275

Locus	5m	30m	120m	240m	360m
P vs T=1	-0.338	-0.321	-0.615	-0.517	-0.214
PS vs T=1	-0.047	-0.167	-0.641	-0.586	-0.521
PS vs P	0.284	0.154	-0.057	-0.086	-0.291

phosphofructokinase

energy metabolism

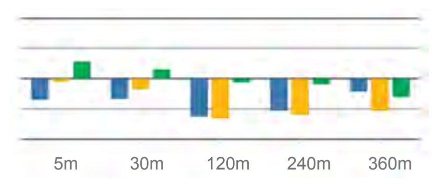

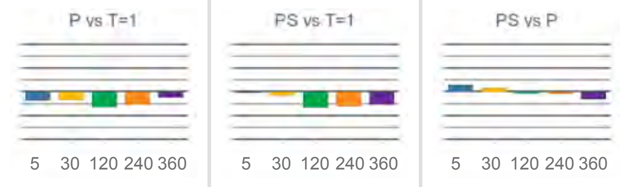

PGN_0276

Locus	5m	30m	120m	240m	360m
P vs T=1	-1.425	-1.136	-1.231	-0.046	0.341
PS vs T=1	-1.385	-1.529	-1.554	-1.105	-1.083
PS vs P	-0.103	-0.399	-0.529	-0.579	-1.012

hypothetical protein

hypothetical proteins

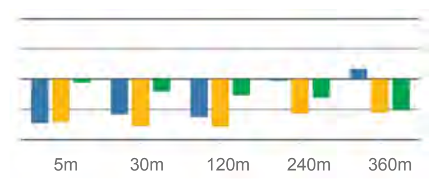

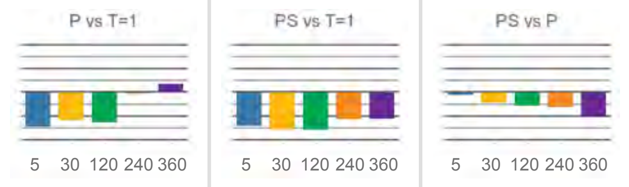

PGN_0277

hslR

Locus	5m	30m	120m	240m	360m
P vs T=1	-0.526	-0.235	-0.041	-0.089	0.692
PS vs T=1	-0.558	-0.992	-0.730	-0.342	-0.121
PS vs P	-0.075	-0.691	-0.600	-0.228	-0.614

putative heat shock protein 15

protein fate

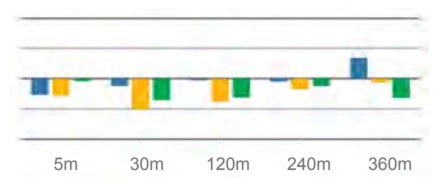

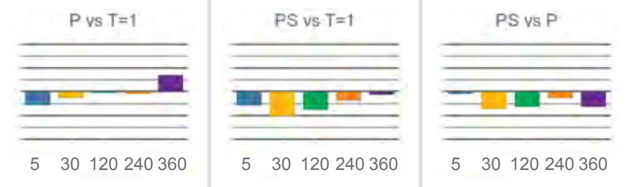

PGN_0278

Locus	5m	30m	120m	240m	360m
P vs T=1	-0.274	-0.204	-0.233	0.597	0.823
PS vs T=1	-0.789	-1.096	-0.999	-0.834	0.017
PS vs P	-0.486	-0.826	-0.763	-1.162	-0.654

putative peptidyl-tRNA hydrolase

protein synthesis

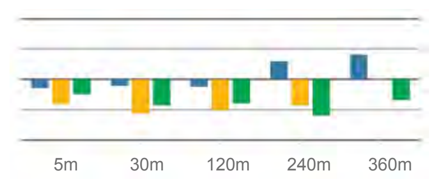

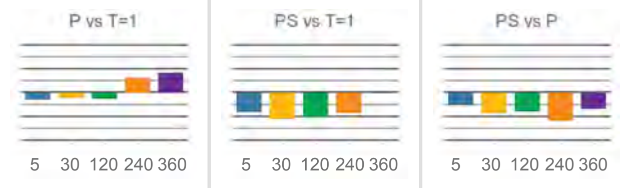

PGN_0279

rplY

Locus	5m	30m	120m	240m	360m
P vs T=1	0.554	0.373	0.682	0.663	0.149
PS vs T=1	-1.435	-1.292	-1.260	-1.265	-0.926
PS vs P	-1.893	-1.588	-1.853	-1.830	-1.041

putative 50S ribosomal protein L25

protein synthesis

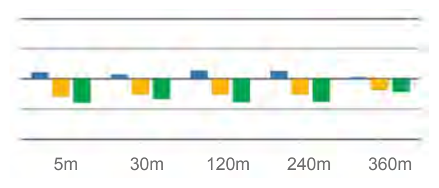

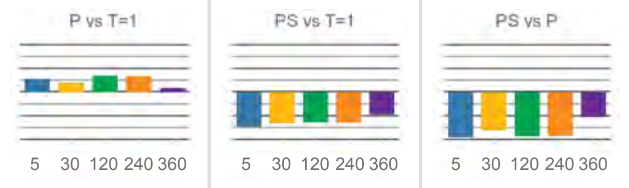

PGN_0280

Locus	5m	30m	120m	240m	360m
P vs T=1	0.540	0.602	1.411	1.805	3.271
PS vs T=1	-1.339	-0.814	0.940	1.354	1.711
PS vs P	-1.466	-1.084	-0.219	-0.092	-0.796

hypothetical protein

hypothetical proteins

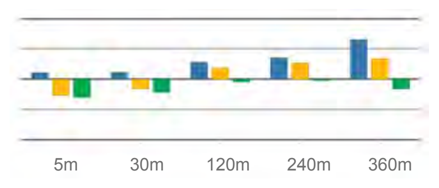

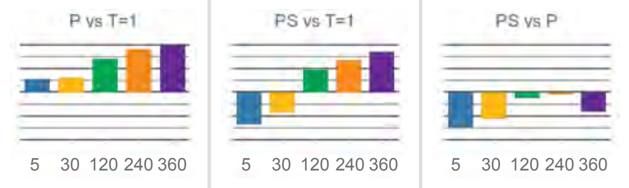

log₂ (Fold Change)

Legend: P vs T=1 | PS vs T=1 | PS vs P

Locus		5m	30m	120m	240m	360m
**PGN_0281**	P vs T=1	-0.478	-0.094	-0.087	-0.443	-0.421
	PS vs T=1	-0.218	-0.320	-0.452	-0.479	-0.606
	PS vs P	0.246	-0.207	-0.342	-0.047	-0.186

methionyl-tRNA synthetase

protein synthesis

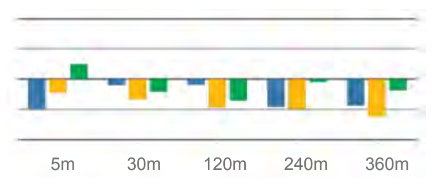

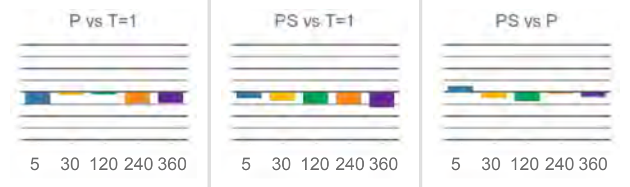

Locus		5m	30m	120m	240m	360m
**PGN_0282**	P vs T=1	-0.631	-0.388	0.034	0.135	0.144
	PS vs T=1	-0.050	-0.272	-0.490	-0.421	-0.172
	PS vs P	0.536	0.096	-0.502	-0.523	-0.300

2',3'-cyclic-nucleotide 2'-phosphodiesterase precursor

purines, pyrimidines, nucleosides and nucleotides

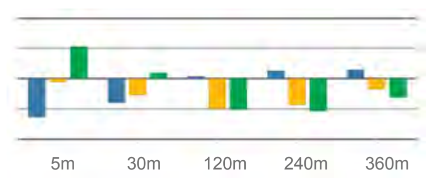

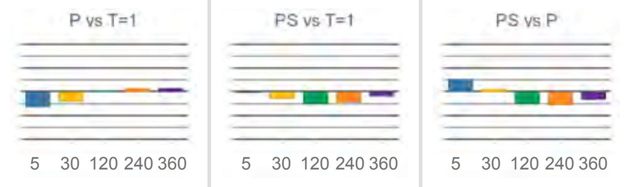

Locus		5m	30m	120m	240m	360m
**PGN_0283**	P vs T=1	-0.929	-0.625	-0.099	0.293	-0.194
	PS vs T=1	-0.402	-0.204	-0.183	-0.244	-0.076
	PS vs P	0.418	0.361	-0.063	-0.446	0.110

probable exonuclease

DNA metabolism

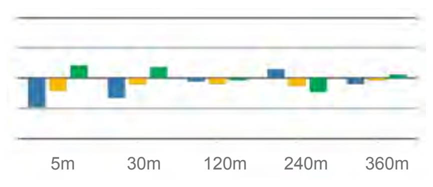

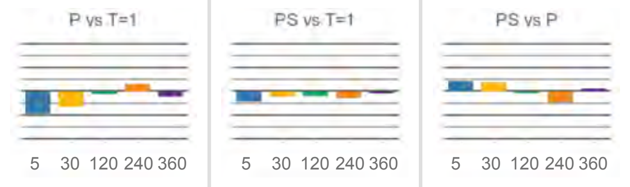

Locus		5m	30m	120m	240m	360m
**PGN_0284**	P vs T=1	0.393	1.013	1.297	0.952	0.134
	PS vs T=1	0.071	0.313	0.372	0.414	0.362
	PS vs P	-0.331	-0.605	-0.809	-0.475	0.188

probable transcriptional regulator

regulatory functions

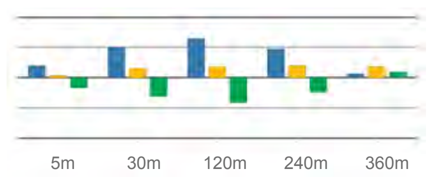

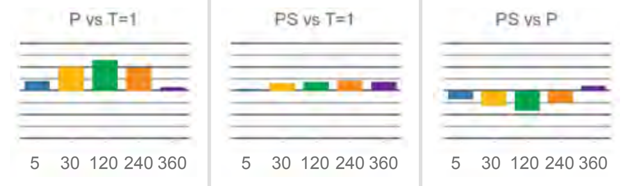

Locus		5m	30m	120m	240m	360m
**PGN_0285**	P vs T=1	2.280	2.731	3.006	2.758	2.273
	PS vs T=1	1.922	1.673	1.317	1.311	1.143
	PS vs P	-0.299	-0.926	-1.538	-1.325	-1.075

pyridine nucleotide-disulphide oxidoreductase

hypothetical proteins-Conserved

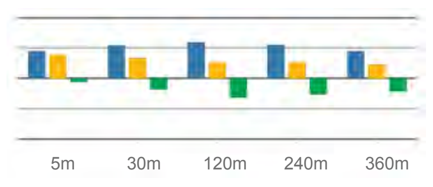

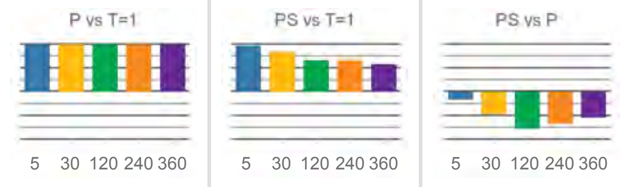

Locus		5m	30m	120m	240m	360m
**PGN_0286**	P vs T=1	0.463	-0.003	1.610	2.197	3.753
	PS vs T=1	-0.517	-0.121	1.231	1.454	1.795
	PS vs P	-0.742	-0.447	0.074	-0.000	-0.561

hypothetical protein frame-shifted with PGN0285

hypothetical proteins

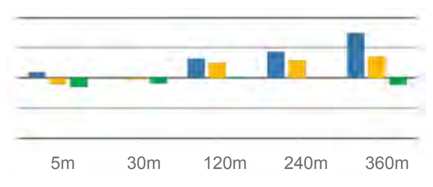

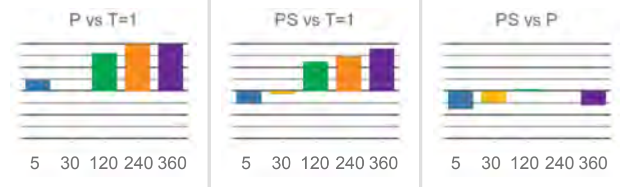

Locus		5m	30m	120m	240m	360m
**PGN_0287** *mfa1*	P vs T=1	-0.886	-0.544	-0.624	-1.194	-1.942
	PS vs T=1	-0.531	-0.154	-0.680	-1.378	-1.506
	PS vs P	0.314	0.400	-0.033	-0.217	0.357

Mfa1fimbrilin

cellular processes

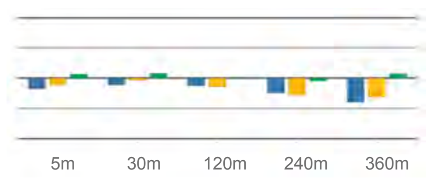

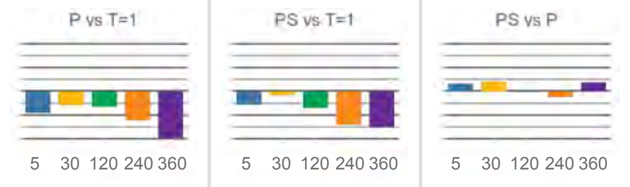

Legend: P vs T=1 | PS vs T=1 | PS vs P

Locus		log$_2$ (Fold Change) 5m	30m	120m	240m	360m
**PGN_0288**	P vs T=1	-0.705	-0.802	-1.632	-2.363	-2.737
	PS vs T=1	-0.495	-0.385	-1.395	-2.263	-2.271
	PS vs P	0.238	0.443	0.201	-0.018	0.355

conserved hypothetical protein

hypothetical proteins-Conserved

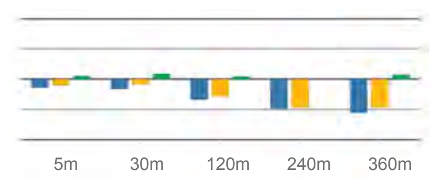

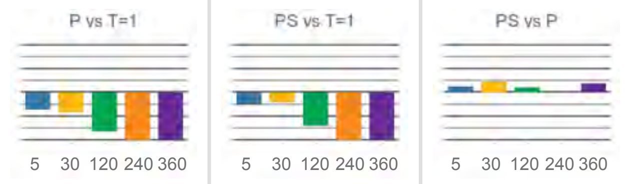

Locus		5m	30m	120m	240m	360m
**PGN_0289**	P vs T=1	-1.041	-1.098	-2.079	-2.490	-2.393
	PS vs T=1	-0.420	-0.124	-1.312	-2.320	-2.320
	PS vs P	0.619	0.974	0.715	0.083	0.030

conserved hypothetical protein

hypothetical proteins-Conserved

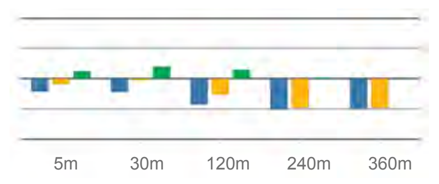

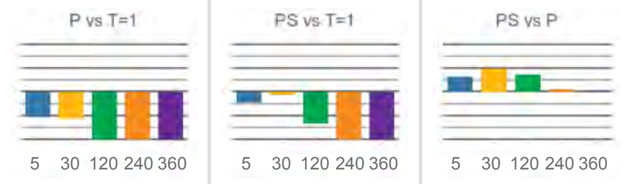

Locus		5m	30m	120m	240m	360m
**PGN_0290**	P vs T=1	-0.560	-0.469	-1.132	-1.503	-1.773
	PS vs T=1	0.252	0.438	-0.378	-1.250	-1.428
	PS vs P	0.794	0.903	0.715	0.180	0.281

immunoreactive 32 kDa antigen

cell envelope

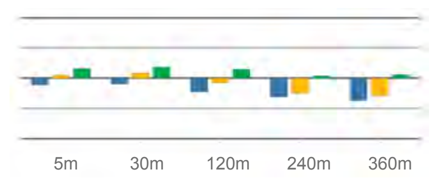

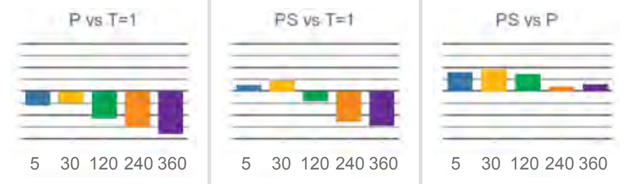

Locus		5m	30m	120m	240m	360m
**PGN_0291**	P vs T=1	-1.354	-1.209	-1.396	-1.598	-1.897
	PS vs T=1	-0.329	-0.416	-0.865	-1.387	-1.635
	PS vs P	0.984	0.770	0.509	0.182	0.228

conserved hypothetical protein

unknown function

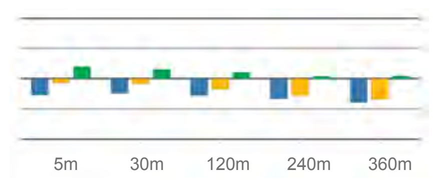

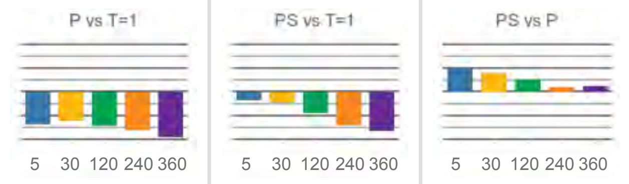

Locus		5m	30m	120m	240m	360m
**PGN_0292**	P vs T=1	1.300	1.481	1.978	2.327	2.228
	PS vs T=1	-0.580	-0.207	0.117	0.408	0.745
	PS vs P	-1.522	-1.286	-1.386	-1.232	-1.011

hypothetical protein

hypothetical proteins

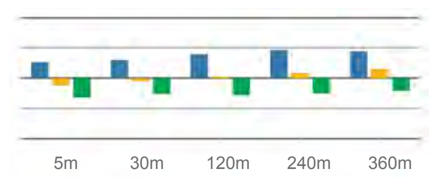

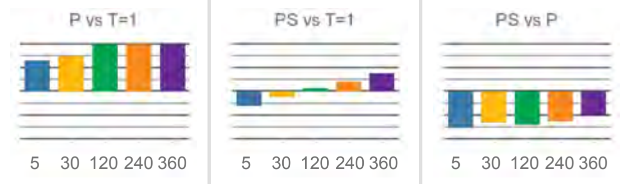

Locus		5m	30m	120m	240m	360m
**PGN_0293** *ragA*	P vs T=1	1.012	1.269	0.808	-0.326	-1.504
	PS vs T=1	0.615	0.827	-0.303	-1.581	-1.670
	PS vs P	-0.281	-0.274	-0.973	-1.254	-0.269

receptor antigen A

cell envelope

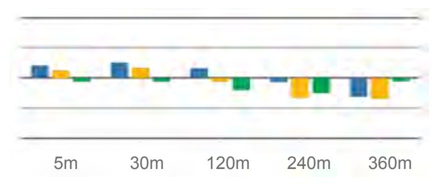

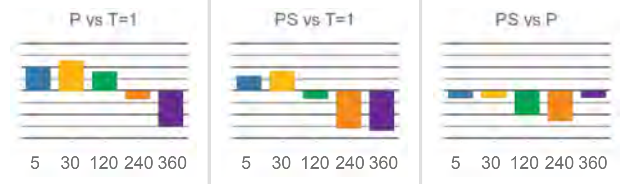

Locus		5m	30m	120m	240m	360m
**PGN_0294** *ragB*	P vs T=1	1.460	1.935	2.271	1.984	1.303
	PS vs T=1	1.170	1.525	0.959	0.136	0.176
	PS vs P	-0.266	-0.325	-1.187	-1.749	-1.108

receptor antigen B

cell envelope

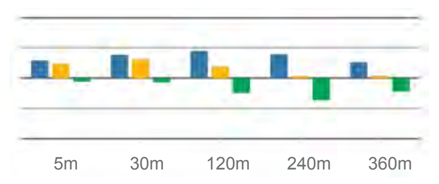

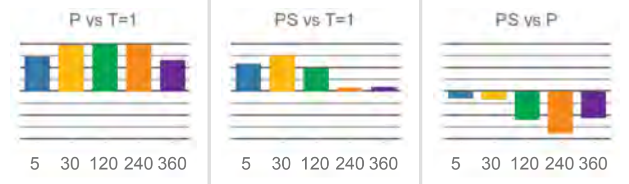

PgSg **RNA Seq**

PgSg Time Course

Hackett Laboratory UW

log₂ (Fold Change)

P vs T=1 | PS vs T=1 | PS vs P

Locus		5m	30m	120m	240m	360m
**PGN_0295**	P vs T=1	0.395	0.602	0.500	0.014	-0.879
	PS vs T=1	-0.862	-0.573	-1.149	-1.983	-2.194
	PS vs P	-1.098	-0.977	-1.473	-1.876	-1.319

C-terminal domain of Arg- and Lys-gingipain proteinase

hypothetical proteins-Conserved

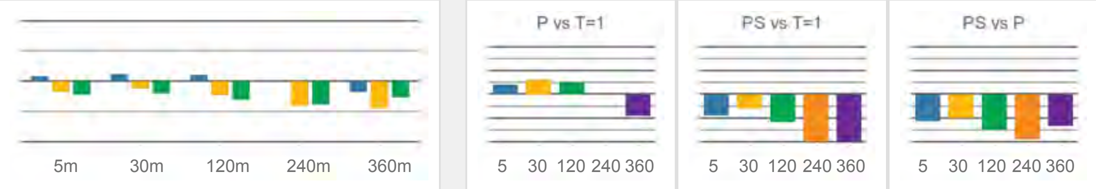

Locus		5m	30m	120m	240m	360m
**PGN_0296**	P vs T=1	0.368	-0.397	-1.274	-1.465	-1.870
	PS vs T=1	-0.141	-0.399	-0.449	-0.429	-0.673
	PS vs P	-0.423	0.007	0.756	0.954	1.109

conserved hypothetical protein

hypothetical proteins-Conserved

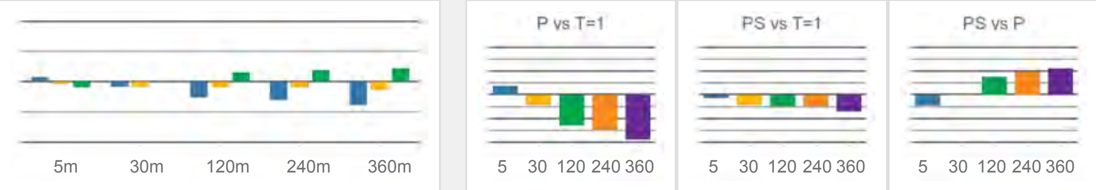

Locus		5m	30m	120m	240m	360m
**PGN_0297**	P vs T=1	0.482	0.486	0.541	0.628	-0.026
	PS vs T=1	0.387	0.187	-0.164	-0.086	-0.116
	PS vs P	-0.087	-0.289	-0.691	-0.691	-0.099

conserved hypothetical protein

hypothetical proteins-Conserved

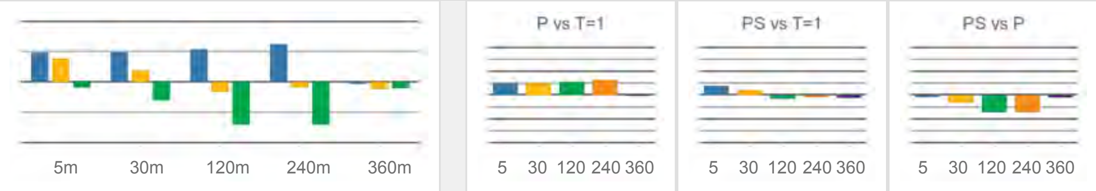

Locus		5m	30m	120m	240m	360m
**PGN_0298**	P vs T=1	-0.005	-0.322	-0.539	-0.382	-0.484
	PS vs T=1	-0.015	-0.279	-0.996	-1.372	-1.100
	PS vs P	0.015	0.048	-0.458	-0.961	-0.607

putative undecaprenyl diphosphate synthase

biosynthesis of cofactors, prosthetic groups, and carriers

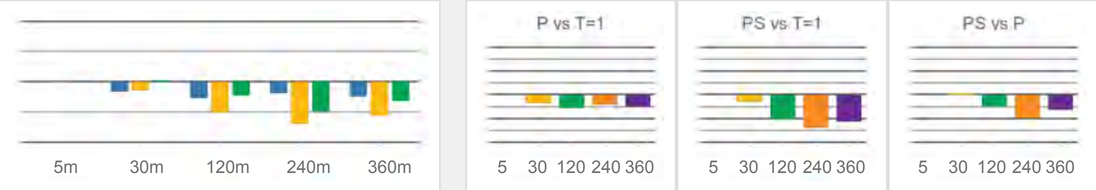

Locus		5m	30m	120m	240m	360m
**PGN_0299**	P vs T=1	-0.231	-0.956	-1.577	-1.678	-1.747
	PS vs T=1	-0.229	-0.630	-1.402	-1.961	-2.005
	PS vs P	0.041	0.324	0.153	-0.301	-0.272

putative outer membrane protein

cell envelope

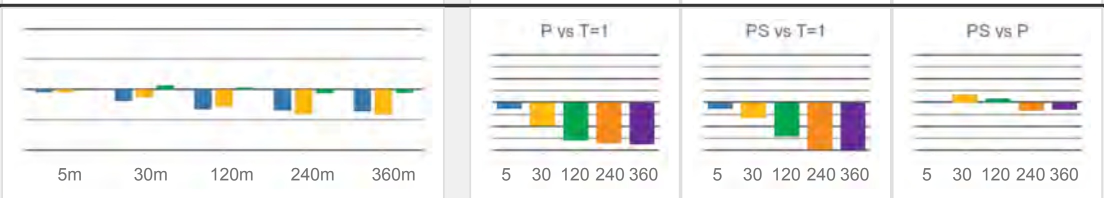

Locus		5m	30m	120m	240m	360m
**PGN_0300**	P vs T=1	0.676	1.025	1.453	1.538	1.103
	PS vs T=1	0.610	1.767	2.497	2.501	2.423
	PS vs P	-0.110	0.715	1.044	0.965	1.297

conserved hypothetical protein

cell envelope

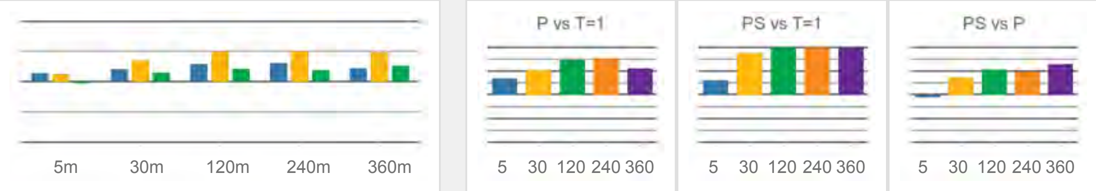

Locus		5m	30m	120m	240m	360m
**PGN_0301**	P vs T=1	0.304	0.492	0.940	0.976	0.677
	PS vs T=1	0.142	1.116	1.787	1.654	1.518
	PS vs P	-0.197	0.592	0.856	0.684	0.827

conserved hypothetical protein

cell envelope

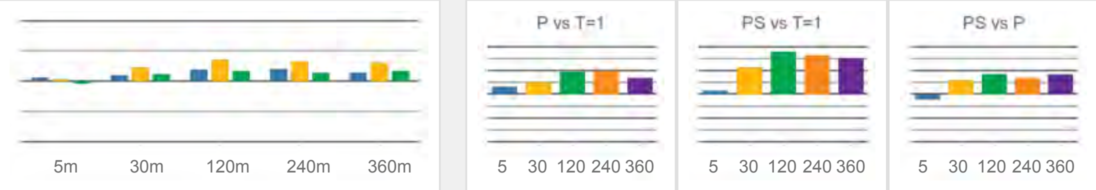

PgSg Time Course

log2 (Fold Change)

Legend: P vs T=1 | PS vs T=1 | PS vs P

Locus		5m	30m	120m	240m	360m
**PGN_0302**	P vs T=1	-0.685	-0.464	0.017	0.128	-0.593
	PS vs T=1	-0.808	-0.474	-0.068	0.124	0.135
	PS vs P	-0.154	-0.027	-0.074	0.011	0.701
	rubrerythrin					
	energy metabolism					
**PGN_0303**	P vs T=1	1.341	1.589	1.399	1.076	0.531
	PS vs T=1	1.164	1.264	1.607	1.635	1.286
	PS vs P	-0.168	-0.293	0.219	0.545	0.723
	putative zinc protease					
	protein fate					
**PGN_0304**	P vs T=1	-0.099	-0.096	1.848	2.253	3.235
	PS vs T=1	-0.432	-0.269	0.959	2.161	2.057
	PS vs P	-0.485	-0.442	-0.091	0.597	0.105
	conserved hypothetical protein					
	hypothetical proteins-Conserved					
**PGN_0305**	P vs T=1	-0.020	-0.087	1.710	2.132	3.327
	PS vs T=1	-0.734	0.226	1.181	2.148	2.172
	PS vs P	-0.807	-0.185	-0.007	0.524	-0.108
	conserved hypothetical protein					
	hypothetical proteins-Conserved					
**PGN_0306**	P vs T=1	-0.113	-0.239	0.190	0.605	0.836
	PS vs T=1	-0.853	-0.225	0.549	0.989	0.873
	PS vs P	-0.737	-0.019	0.352	0.427	0.076
	conserved hypothetical protein					
	hypothetical proteins-Conserved					
**PGN_0307**	P vs T=1	-0.902	-1.036	-0.857	-0.560	-0.124
	PS vs T=1	-1.668	-1.170	-0.563	-0.325	-0.282
	PS vs P	-0.761	-0.157	0.268	0.253	-0.112
	conserved hypothetical protein					
	hypothetical proteins-Conserved					
**PGN_0308**	P vs T=1	-0.126	-0.305	-0.089	0.649	1.071
	PS vs T=1	-0.570	-0.847	-0.700	-0.299	0.724
	PS vs P	-0.385	-0.530	-0.600	-0.554	-0.050
	conserved hypothetical protein					
	hypothetical proteins-Conserved					

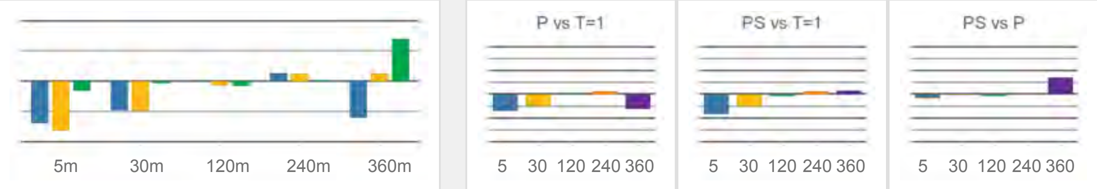

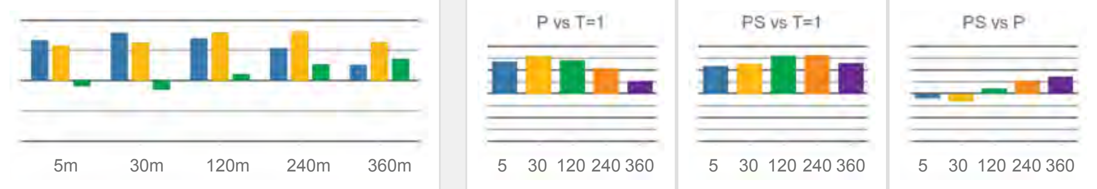

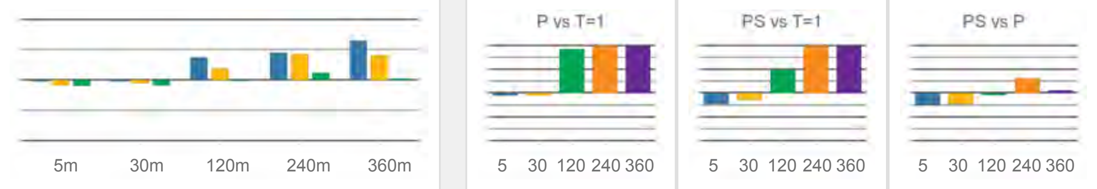

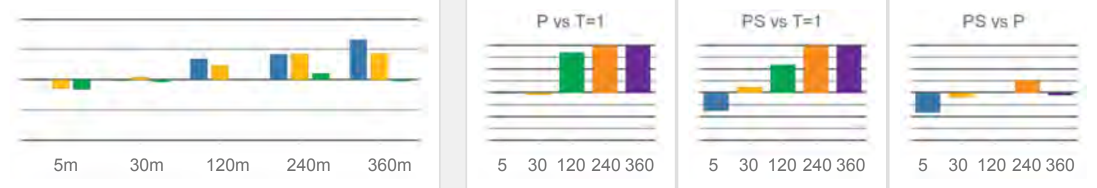

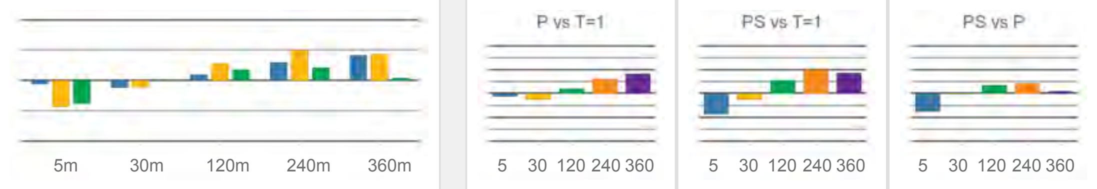

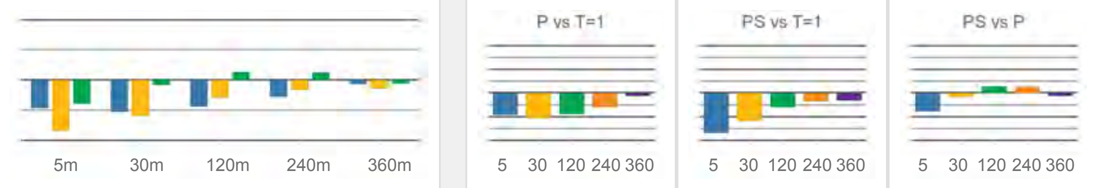

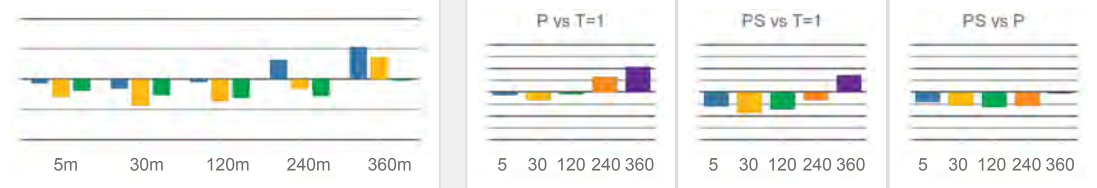

log2 (Fold Change)

Legend: P vs T=1, PS vs T=1, PS vs P

Locus		5m	30m	120m	240m	360m
**PGN_0309**	P vs T=1	-0.782	-1.216	-1.359	-1.136	-0.296
	PS vs T=1	-1.546	-1.729	-1.629	-1.367	-1.094
	PS vs P	-0.685	-0.513	-0.335	-0.240	-0.652

probable ribonuclease P protein component

transcription

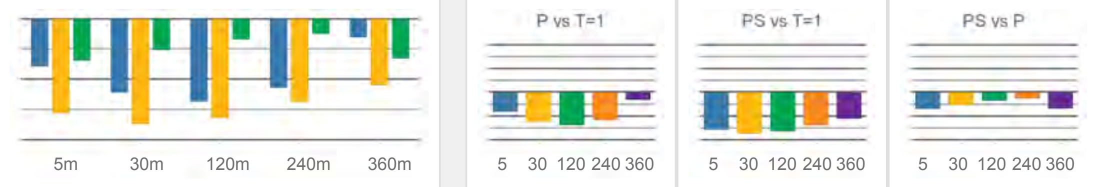

Locus		5m	30m	120m	240m	360m
**PGN_0310** *HemD*	P vs T=1	-0.611	-1.163	-1.725	-1.766	-1.615
	PS vs T=1	-2.014	-2.178	-2.118	-1.707	-1.698
	PS vs P	-1.322	-0.975	-0.417	0.027	-0.090

putative uroporphyrinogen-III synthase

biosynthesis of cofactors, prosthetic groups, and carriers

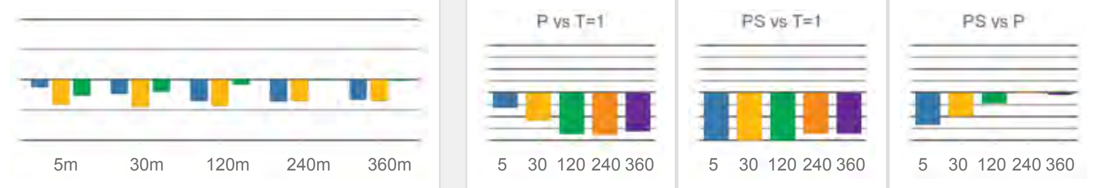

Locus		5m	30m	120m	240m	360m
**PGN_0311**	P vs T=1	-0.021	-0.864	-1.000	-0.319	0.283
	PS vs T=1	-0.414	-0.642	-0.069	0.141	0.045
	PS vs P	-0.320	0.090	0.695	0.443	-0.161

conserved hypothetical protein

hypothetical proteins-Conserved

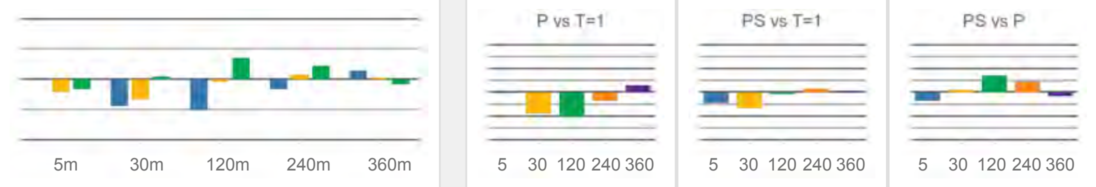

Locus		5m	30m	120m	240m	360m
**PGN_0312**	P vs T=1	0.537	-0.118	-0.623	-0.030	-0.139
	PS vs T=1	-0.059	-0.220	0.002	0.310	-0.097
	PS vs P	-0.487	-0.125	0.381	0.358	0.009

hypothetical protein

hypothetical proteins

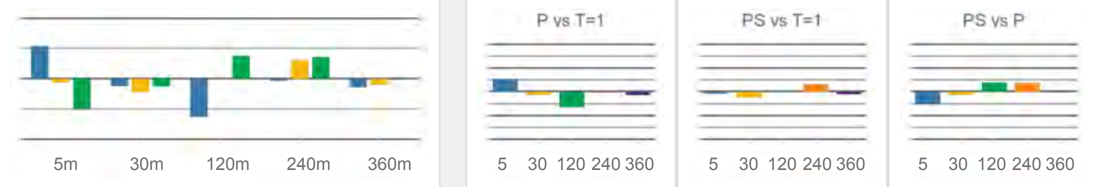

Locus		5m	30m	120m	240m	360m
**PGN_0313**	P vs T=1	-0.245	-0.427	-0.764	-0.550	-0.147
	PS vs T=1	-0.524	-0.822	-1.136	-1.051	-0.896
	PS vs P	-0.255	-0.376	-0.388	-0.475	-0.704

peptide chain release factor 3

protein synthesis

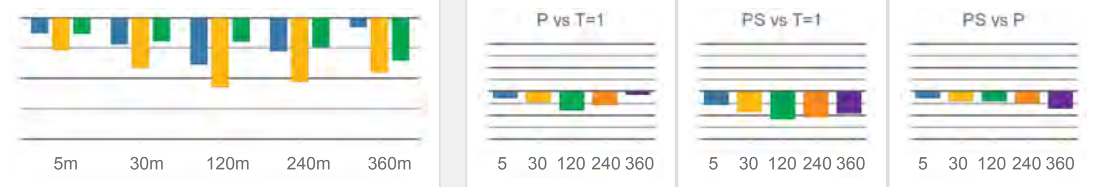

Locus		5m	30m	120m	240m	360m
**PGN_0314**	P vs T=1	0.437	0.146	0.226	0.671	1.676
	PS vs T=1	0.205	0.671	1.414	1.144	0.654
	PS vs P	-0.221	0.433	1.061	0.483	-0.817

probable formate/nitrite transporter

transport and binding proteins

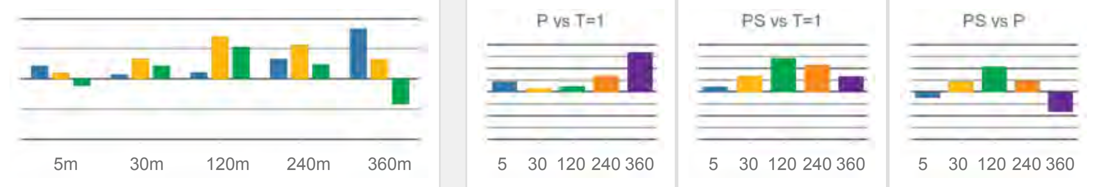

Locus		5m	30m	120m	240m	360m
**PGN_0315** *CbiD*	P vs T=1	-0.893	-0.659	-0.420	-0.239	0.212
	PS vs T=1	-0.063	-0.104	-0.563	-0.523	-0.388
	PS vs P	0.756	0.513	-0.145	-0.256	-0.536

precorrin-6x reductase/cobalamin biosynthetic protein CbiD

biosynthesis of cofactors, prosthetic groups, and carriers

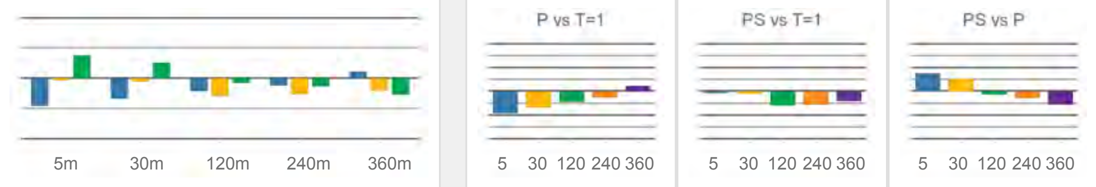

log$_2$ (Fold Change)

Legend: P vs T=1 | PS vs T=1 | PS vs P

PGN_0316

CbiG

Locus	5m	30m	120m	240m	360m
P vs T=1	-0.888	-0.800	-0.539	-0.076	0.297
PS vs T=1	-0.195	-0.369	-0.805	-0.884	-0.759
PS vs P	0.647	0.392	-0.274	-0.724	-0.979

precorrin-4 C11-methyltransferase

biosynthesis of cofactors, prosthetic groups, and carriers

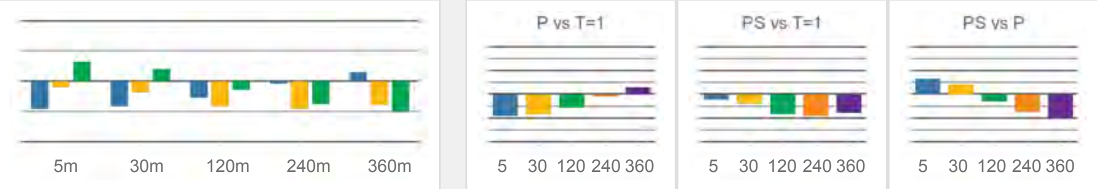

PGN_0317

CobL

Locus	5m	30m	120m	240m	360m
P vs T=1	-0.739	-0.631	-0.453	0.072	0.556
PS vs T=1	-0.170	-0.523	-0.788	-0.801	-0.105
PS vs P	0.514	0.076	-0.350	-0.748	-0.550

decarboxylating precorrin-6Y C5,15-methyltransferase

biosynthesis of cofactors, prosthetic groups, and carriers

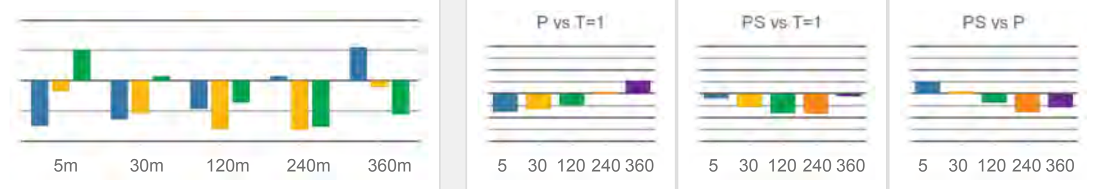

PGN_0318

CobH/C

Locus	5m	30m	120m	240m	360m
P vs T=1	-1.025	-0.613	-0.223	-0.014	-0.145
PS vs T=1	-0.534	-0.743	-0.766	-0.220	-0.188
PS vs P	0.410	-0.146	-0.518	-0.157	-0.034

precorrin-3B C17-methyltransferase

biosynthesis of cofactors, prosthetic groups, and carriers

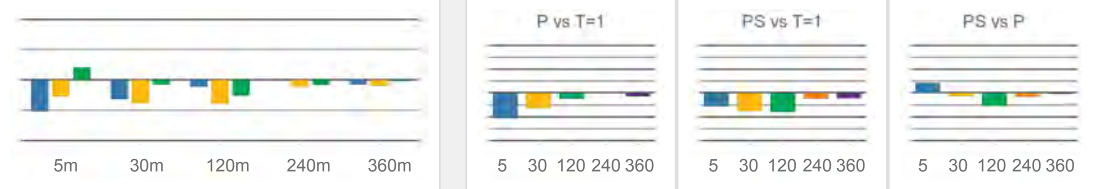

PGN_0319

Locus	5m	30m	120m	240m	360m
P vs T=1	0.175	0.547	1.134	1.883	1.776
PS vs T=1	-0.208	0.565	1.504	1.926	2.147
PS vs P	-0.475	-0.031	0.365	0.177	0.426

probable RNA polymerase sigma-70 factor ECF subfamily

transcription

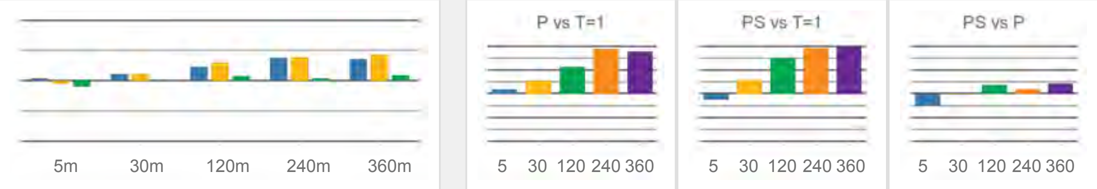

PGN_0320

Locus	5m	30m	120m	240m	360m
P vs T=1	-0.434	-0.099	0.477	1.189	1.421
PS vs T=1	-0.776	0.000	0.862	1.282	1.496
PS vs P	-0.439	0.030	0.357	0.229	0.162

conserved hypothetical protein

hypothetical proteins-Conserved

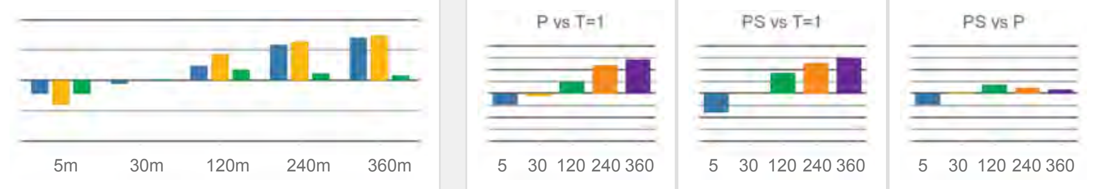

PGN_0321

Locus	5m	30m	120m	240m	360m
P vs T=1	0.319	0.470	1.256	1.902	1.854
PS vs T=1	-0.264	0.401	2.014	2.245	2.009
PS vs P	-0.629	-0.133	0.765	0.463	0.214

conserved hypothetical protein

hypothetical proteins-Conserved

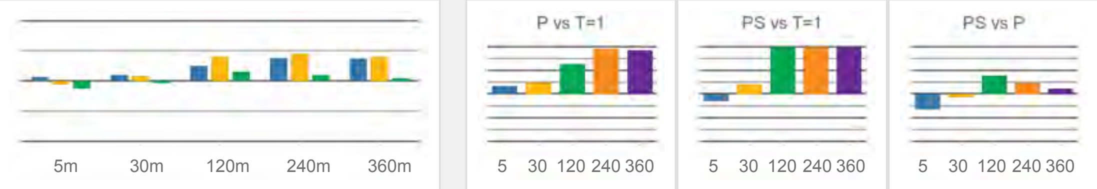

PGN_0322

Locus	5m	30m	120m	240m	360m
P vs T=1	0.628	1.058	1.606	1.884	1.837
PS vs T=1	-0.023	0.241	1.081	1.650	1.816
PS vs P	-0.663	-0.783	-0.470	-0.161	0.020

conserved hypothetical protein

hypothetical proteins-Conserved

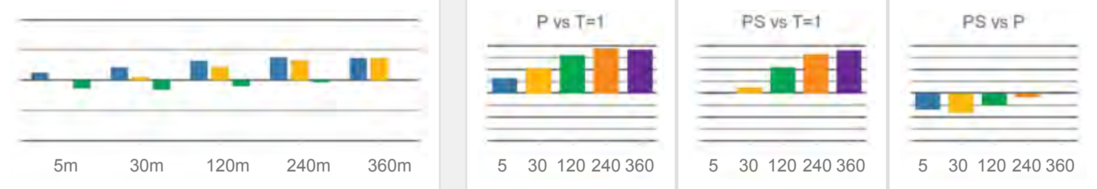

PgSg Time Course

log$_2$ (Fold Change)

Legend: P vs T=1 | PS vs T=1 | PS vs P

Locus		5m	30m	120m	240m	360m
**PGN_0323**	P vs T=1	0.836	1.318	1.793	2.255	2.300
	PS vs T=1	0.164	0.306	0.902	1.450	1.709
	PS vs P	-0.675	-0.980	-0.852	-0.735	-0.548
	conserved hypothetical protein					
	hypothetical proteins-Conserved					

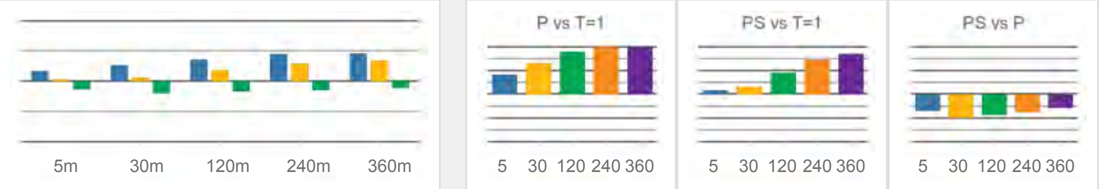

Locus		5m	30m	120m	240m	360m
**PGN_0324**	P vs T=1	0.281	0.206	0.237	0.310	-0.035
	PS vs T=1	0.089	-0.131	0.043	0.179	0.180
	PS vs P	-0.190	-0.341	-0.202	-0.132	0.190
	transposase in ISPg1					

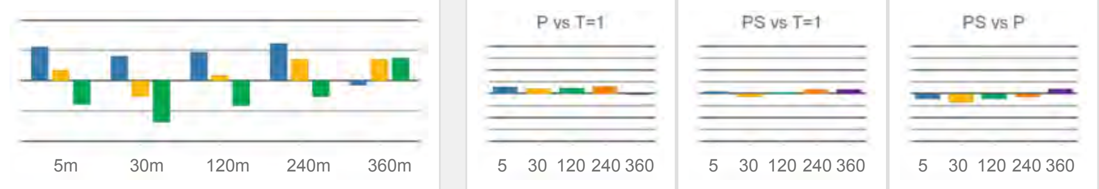

Locus		5m	30m	120m	240m	360m
**PGN_0325**	P vs T=1	-0.712	-1.492	-0.971	-1.144	0.117
	PS vs T=1	-0.630	-0.545	0.043	0.216	0.257
	PS vs P	0.071	0.514	0.844	0.901	0.347
	hypothetical protein					
	hypothetical proteins					

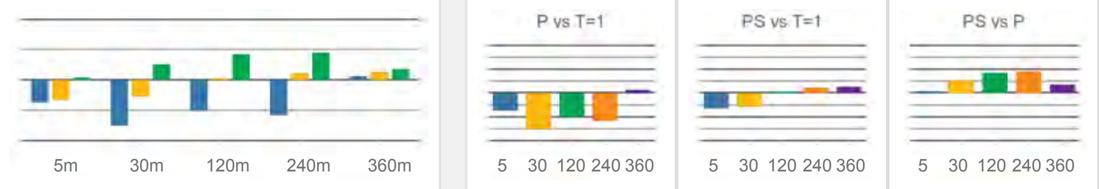

Locus		5m	30m	120m	240m	360m
**PGN_0326**	P vs T=1	-0.835	-0.554	-0.022	0.170	0.518
	PS vs T=1	-1.277	-0.975	-0.070	0.067	0.412
	PS vs P	-0.505	-0.430	0.024	0.004	0.012
	DNA-binding protein histone-like family					
	DNA metabolism					

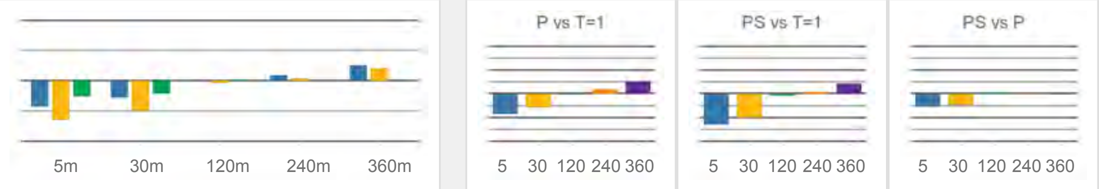

Locus		5m	30m	120m	240m	360m
**PGN_0327**	P vs T=1	-0.740	-0.884	-0.867	-0.519	-0.484
	PS vs T=1	-1.022	-0.900	-0.627	-0.476	-0.456
	PS vs P	-0.286	-0.071	0.153	0.080	0.038
	putative DNA polymerase III epsilon chain					
	DNA metabolism					

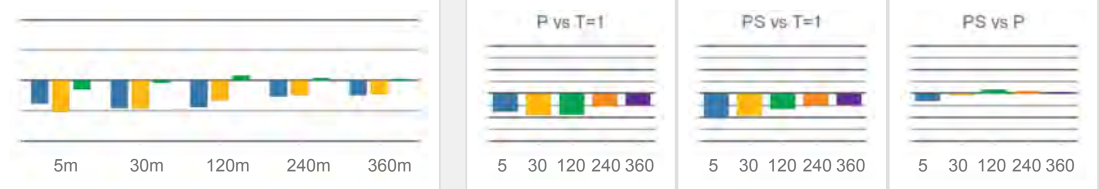

Locus		5m	30m	120m	240m	360m
**PGN_0328**	P vs T=1	0.293	0.392	0.483	0.388	0.212
	PS vs T=1	0.142	0.506	0.869	0.917	0.809
	PS vs P	-0.158	0.109	0.385	0.505	0.566
	conserved hypothetical protein					
	hypothetical proteins-Conserved					

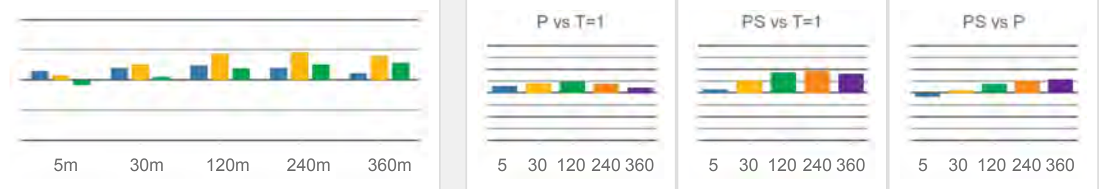

Locus		5m	30m	120m	240m	360m
**PGN_0329**	P vs T=1	-0.279	0.298	1.087	1.325	0.960
	PS vs T=1	-0.411	-0.245	-0.118	0.011	0.094
	PS vs P	-0.187	-0.523	-1.116	-1.206	-0.824
	conserved hypothetical protein					
	unknown function					

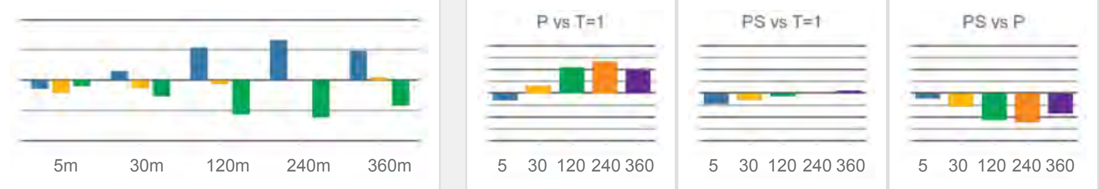

PgSg Time Course

log2 (Fold Change)

Legend: P vs T=1 | PS vs T=1 | PS vs P

Locus		5m	30m	120m	240m	360m
**PGN_0330**	P vs T=1	-0.898	-1.042	-1.244	-0.887	0.011
	PS vs T=1	-1.012	-1.558	-1.473	-1.078	-0.972
	PS vs P	-0.106	-0.512	-0.290	-0.180	-0.887

DNA repair protein

DNA metabolism

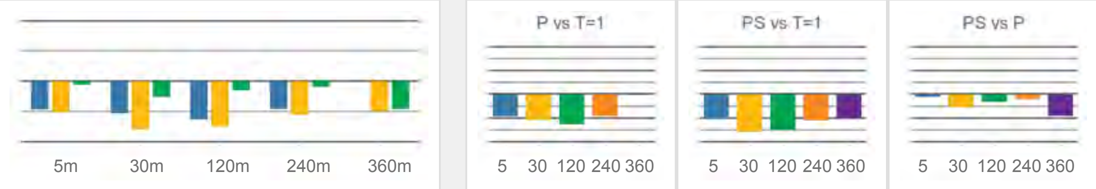

Locus		5m	30m	120m	240m	360m
**PGN_0331**	P vs T=1	-0.913	-1.059	-1.538	-1.283	-0.923
	PS vs T=1	-1.415	-1.426	-0.911	-0.798	-1.041
	PS vs P	-0.478	-0.353	0.536	0.448	-0.099

conserved hypothetical protein

unknown function

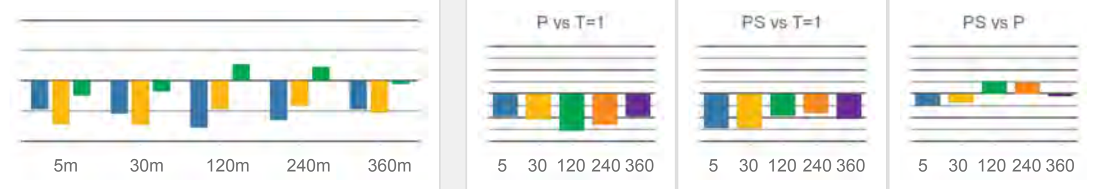

Locus		5m	30m	120m	240m	360m
**PGN_0332**	P vs T=1	2.047	1.704	1.131	1.174	2.526
	PS vs T=1	1.328	1.294	1.801	1.682	1.789
	PS vs P	-0.510	-0.277	0.461	0.285	-0.308

conserved hypothetical protein

hypothetical proteins-Conserved

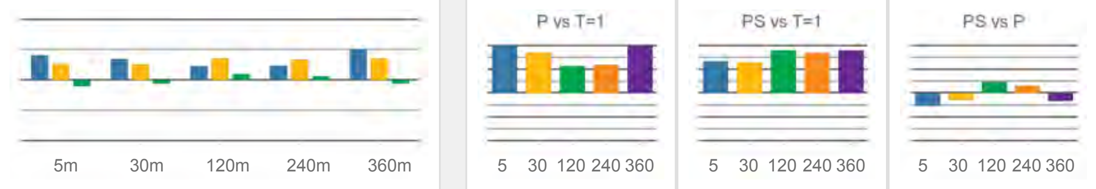

Locus		5m	30m	120m	240m	360m
**PGN_0333**	P vs T=1	-0.699	-1.023	-1.530	-1.779	-2.361
	PS vs T=1	-1.268	-1.461	-1.483	-1.331	-1.402
	PS vs P	-0.522	-0.413	0.034	0.414	0.892

putative transaldolase

energy metabolism

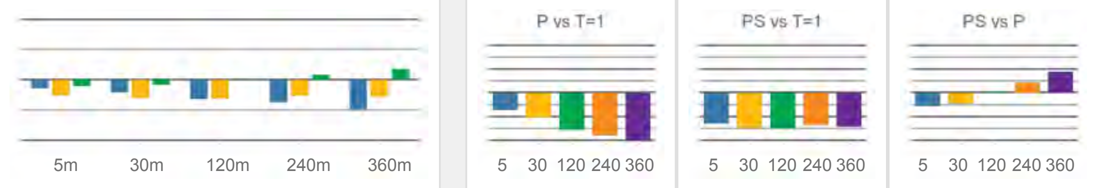

Locus		5m	30m	120m	240m	360m
**PGN_0334**	P vs T=1	0.628	0.947	1.645	1.917	1.690
	PS vs T=1	1.086	0.880	0.716	0.867	1.115
	PS vs P	0.418	-0.072	-0.865	-0.960	-0.534

conserved hypothetical protein with DUF1343 domain

hypothetical proteins-Conserved

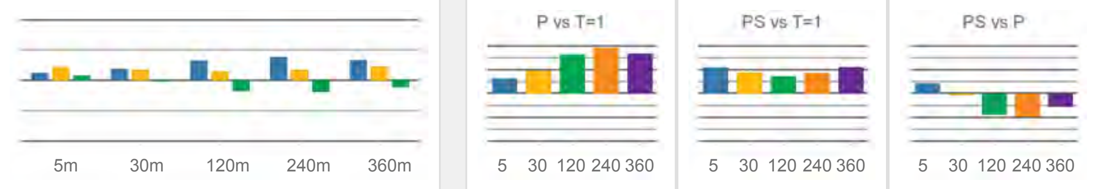

Locus		5m	30m	120m	240m	360m
**PGN_0335**	P vs T=1	1.172	1.288	1.042	0.622	-0.181
	PS vs T=1	0.955	1.164	0.919	0.745	0.480
	PS vs P	-0.194	-0.091	-0.104	0.118	0.624

conserved hypothetical protein with Zinc carboxypeptidase domain

protein fate

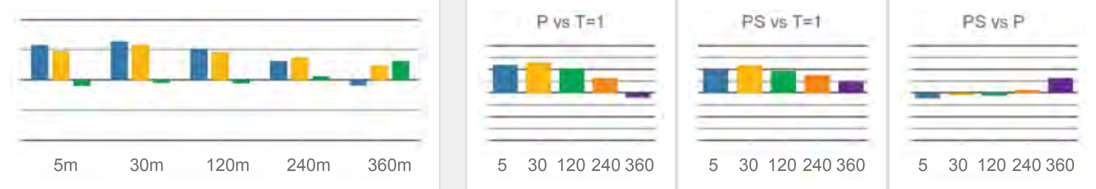

Locus		5m	30m	120m	240m	360m
**PGN_0336**	P vs T=1	-0.915	-1.791	-1.990	-1.186	-0.970
	PS vs T=1	-1.339	-0.934	0.169	-0.017	-0.221
	PS vs P	-0.399	0.683	1.819	1.105	0.721

immunoreactive 23 kDa antigen

cell envelope

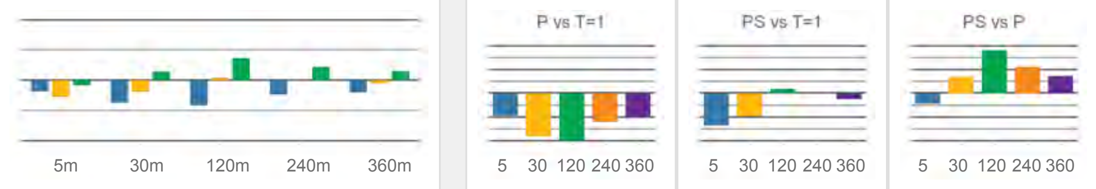

log$_2$ (Fold Change)

Legend: P vs T=1 | PS vs T=1 | PS vs P

PGN_0337

Locus	5m	30m	120m	240m	360m
P vs T=1	-0.012	-0.598	1.487	1.685	3.253
PS vs T=1	-0.706	-0.774	0.931	1.026	1.859
PS vs P	-0.618	-0.650	0.073	-0.068	-0.101

conserved hypothetical protein

hypothetical proteins-Conserved

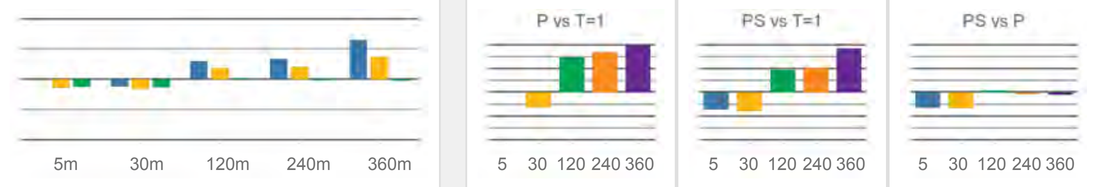

PGN_0338

Locus	5m	30m	120m	240m	360m
P vs T=1	0.196	0.059	1.682	1.591	3.822
PS vs T=1	-0.779	0.508	2.051	1.647	1.697
PS vs P	-0.890	-0.050	0.762	0.201	-0.770

hypothetical protein

hypothetical proteins

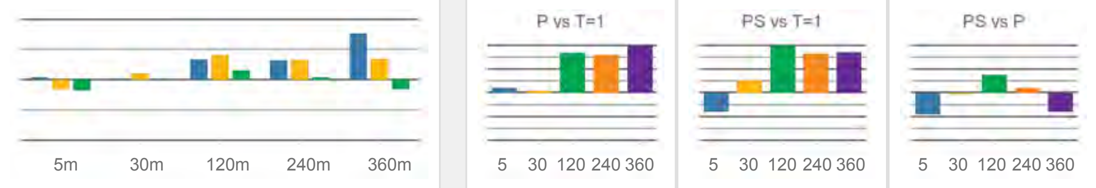

PGN_0339

Locus	5m	30m	120m	240m	360m
P vs T=1	-0.169	-1.121	-0.391	0.940	3.042
PS vs T=1	-0.350	0.042	0.916	2.391	2.005
PS vs P	-0.307	-0.007	0.421	1.287	0.336

hypothetical protein

hypothetical proteins

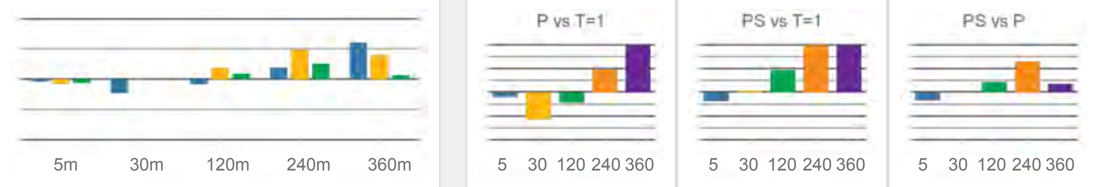

PGN_0340

Locus	5m	30m	120m	240m	360m
P vs T=1	-0.577	-0.675	-0.907	-0.694	0.123
PS vs T=1	-1.420	-1.147	-0.581	-0.522	-0.566
PS vs P	-0.812	-0.452	0.289	0.167	-0.613

carboxyl-terminal processing protease

protein fate

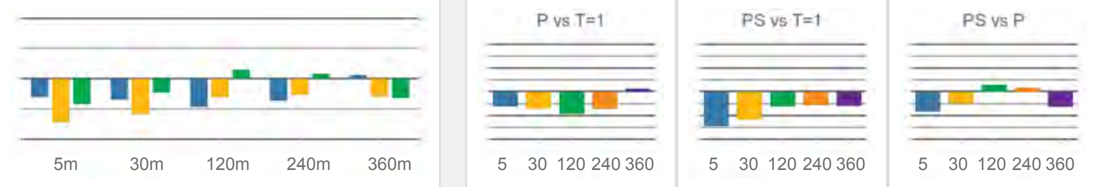

PGN_0341

Locus	5m	30m	120m	240m	360m
P vs T=1	-0.536	-0.725	-0.557	-0.440	-0.281
PS vs T=1	0.392	-0.095	-0.502	-0.309	-0.445
PS vs P	0.911	0.599	0.042	0.126	-0.157

conserved hypothetical protein

hypothetical proteins-Conserved

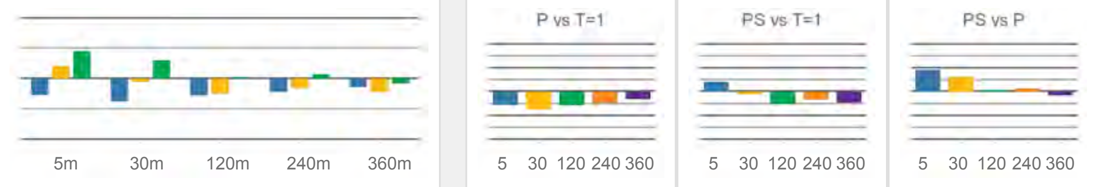

PGN_0342

Locus	5m	30m	120m	240m	360m
P vs T=1	-0.076	-0.095	0.021	0.585	1.426
PS vs T=1	0.813	0.653	0.523	0.861	0.872
PS vs P	0.806	0.645	0.374	0.304	-0.419

putative uracil-DNA glycosylase

DNA metabolism

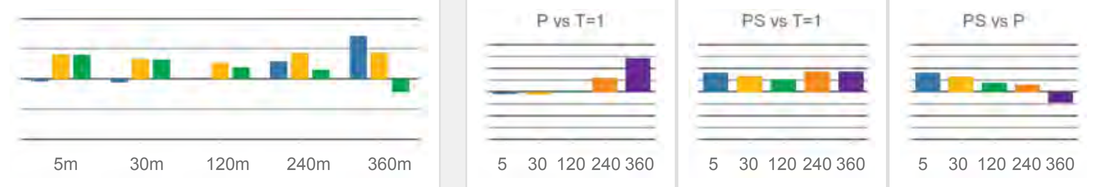

PGN_0343

Locus	5m	30m	120m	240m	360m
P vs T=1	-0.582	-1.025	-1.712	-1.108	-1.054
PS vs T=1	-1.348	-2.215	-2.418	-2.324	-1.277
PS vs P	-0.513	-0.938	-0.839	-0.825	-0.051

hypothetical protein

hypothetical proteins

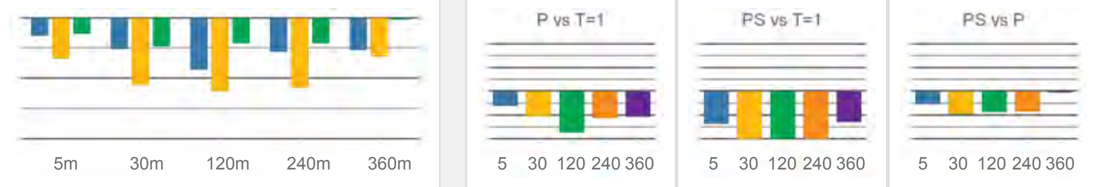

PgSg Time Course

log2 (Fold Change)

Legend: P vs T=1 | PS vs T=1 | PS vs P

Locus		5m	30m	120m	240m	360m
**PGN_0344**	P vs T=1	0.187	-0.319	-1.089	-1.427	-1.041
	PS vs T=1	-0.512	-1.178	-1.728	-1.503	-1.815
	PS vs P	-0.559	-0.763	-0.640	-0.153	-0.739

probable haloacid dehalogenase-like hydrolase

unknown function

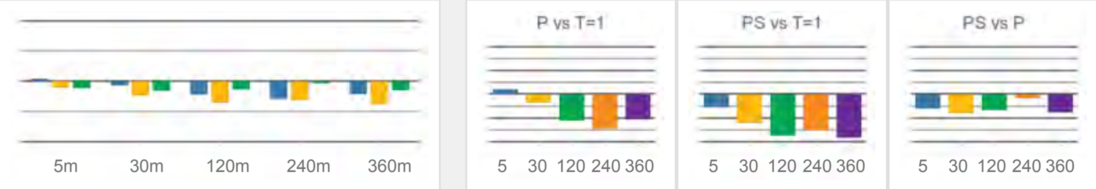

Locus		5m	30m	120m	240m	360m
**PGN_0345**	P vs T=1	0.605	1.321	1.924	1.941	1.261
	PS vs T=1	0.362	0.833	1.013	0.464	0.235
	PS vs P	-0.263	-0.440	-0.829	-1.393	-1.002

conserved hypothetical protein

hypothetical proteins-Conserved

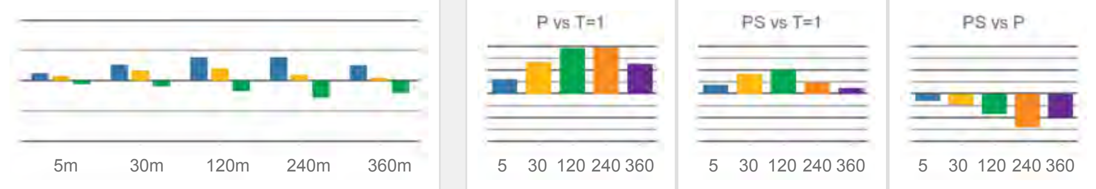

Locus		5m	30m	120m	240m	360m
**PGN_0346**	P vs T=1	1.046	1.502	1.330	0.679	-0.072
	PS vs T=1	0.713	0.874	0.601	0.113	-0.304
	PS vs P	-0.313	-0.569	-0.682	-0.559	-0.257

putative methyltransferase

hypothetical proteins-Conserved

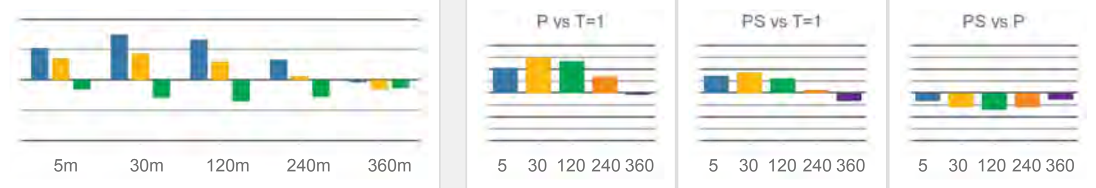

Locus		5m	30m	120m	240m	360m
**PGN_0347**	P vs T=1	0.447	0.299	-0.478	-1.337	-1.880
	PS vs T=1	-0.177	-0.408	-0.634	-0.920	-1.120
	PS vs P	-0.573	-0.658	-0.149	0.359	0.697

conserved hypothetical protein

hypothetical proteins-Conserved

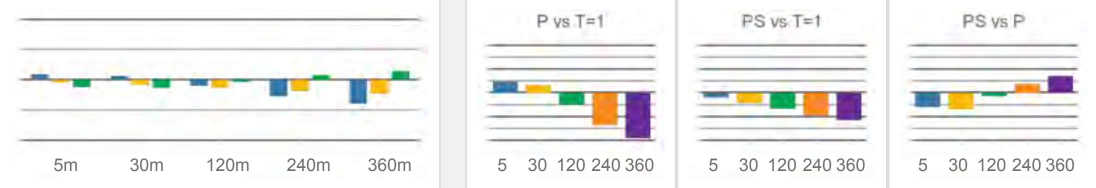

Locus		5m	30m	120m	240m	360m
**PGN_0348** *uspA*	P vs T=1	1.049	1.281	1.219	0.638	-0.258
	PS vs T=1	1.024	1.500	1.624	1.172	0.955
	PS vs P	-0.041	0.231	0.416	0.469	1.087

putative universal stress protein UspA

cellular processes

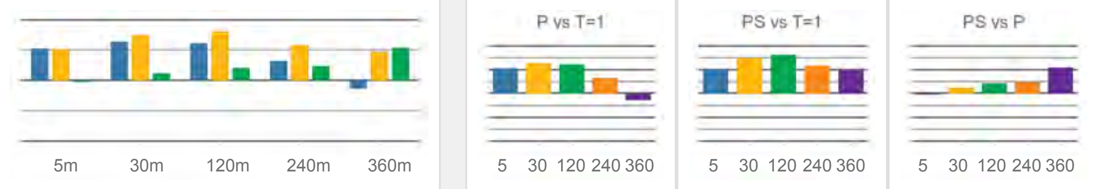

Locus		5m	30m	120m	240m	360m
**PGN_0349** *ustA*	P vs T=1	-0.726	-0.566	-0.449	-0.660	-1.473
	PS vs T=1	-0.821	-0.569	-0.781	-1.036	-0.955
	PS vs P	-0.132	-0.010	-0.306	-0.379	0.400

upregulated in stationary phase protein A

hypothetical proteins

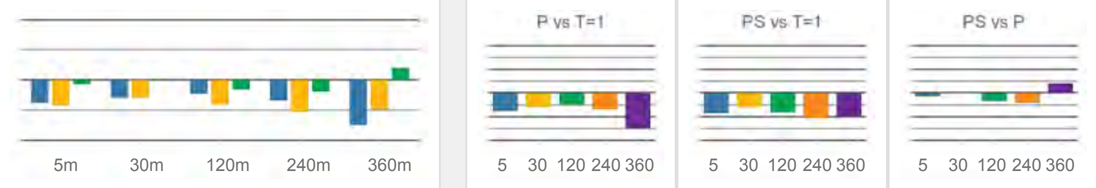

Locus		5m	30m	120m	240m	360m
**PGN_0350**	P vs T=1	1.135	1.355	1.638	1.481	1.215
	PS vs T=1	0.854	1.135	1.460	1.555	1.448
	PS vs P	-0.284	-0.194	-0.115	0.095	0.211

probable translation initation factor SUI1

protein synthesis

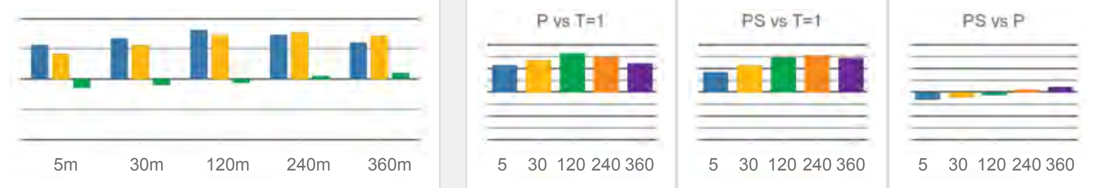

PgSg Time Course

Hackett Laboratory UW

log$_2$ (Fold Change)

Legend: P vs T=1 | PS vs T=1 | PS vs P

Locus		5m	30m	120m	240m	360m
**PGN_0351**	P vs T=1	0.721	1.145	1.380	0.998	0.165
	PS vs T=1	0.694	0.768	0.445	0.237	0.143
	PS vs P	-0.029	-0.349	-0.894	-0.742	-0.039

pyruvate carboxylase subunit B

energy metabolism

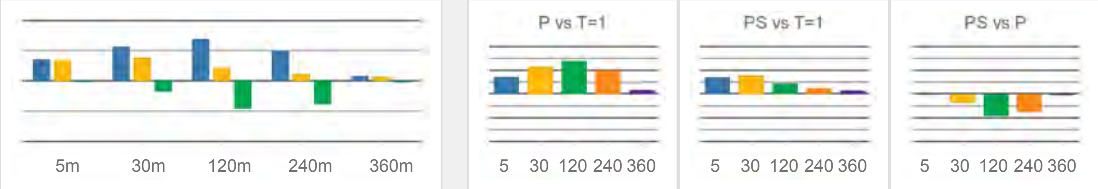

Locus		5m	30m	120m	240m	360m
**PGN_0352**	P vs T=1	-0.395	-0.996	-0.566	0.618	1.660
	PS vs T=1	-0.810	-0.780	-0.571	0.663	1.023
	PS vs P	-0.421	-0.250	-0.381	0.336	-0.026

conserved hypothetical protein

hypothetical proteins-Conserved

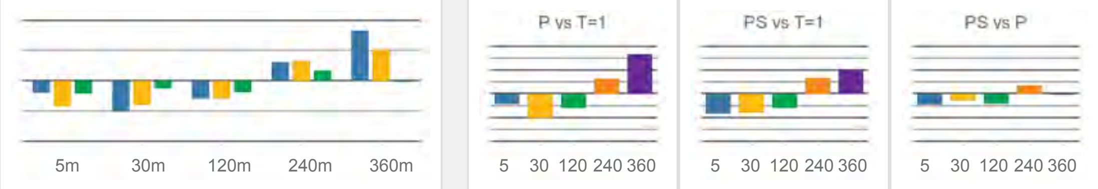

Locus		5m	30m	120m	240m	360m
**PGN_0353**	P vs T=1	0.331	-0.274	-0.868	-1.058	-0.987
	PS vs T=1	-0.357	-0.558	-0.981	-1.040	-0.923
	PS vs P	-0.628	-0.261	-0.137	-0.039	0.042

conserved hypothetical protein

hypothetical proteins-Conserved

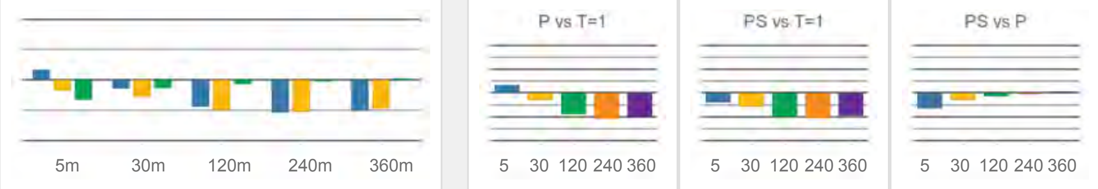

Locus		5m	30m	120m	240m	360m
**PGN_0354**	P vs T=1	0.380	0.287	-0.337	-0.692	-0.648
	PS vs T=1	-0.064	-0.230	-0.999	-1.039	-0.732
	PS vs P	-0.400	-0.472	-0.655	-0.375	-0.094

putative nitrogen utilization substance protein A

transcription

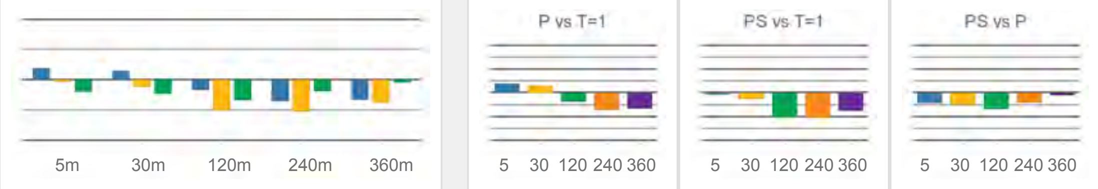

Locus		5m	30m	120m	240m	360m
**PGN_0355**	P vs T=1	0.224	0.602	0.468	-0.023	-0.387
	PS vs T=1	-0.238	-0.419	-1.040	-1.142	-1.045
	PS vs P	-0.446	-0.983	-1.475	-1.104	-0.659

translation initiation factor IF-2

protein synthesis

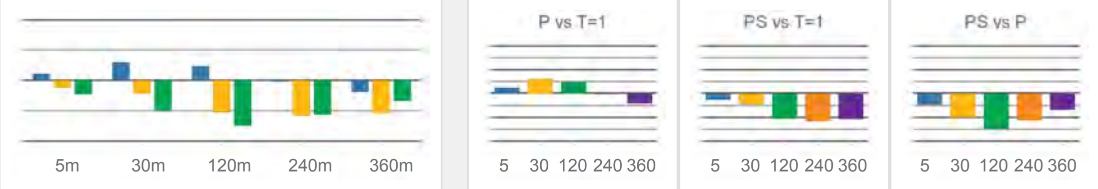

Locus		5m	30m	120m	240m	360m
**PGN_0356**	P vs T=1	-0.099	-0.046	0.310	0.003	-0.357
	PS vs T=1	-0.403	-0.469	-0.178	0.061	-0.267
	PS vs P	-0.292	-0.403	-0.404	0.069	0.046

conserved hypothetical protein

hypothetical proteins-Conserved

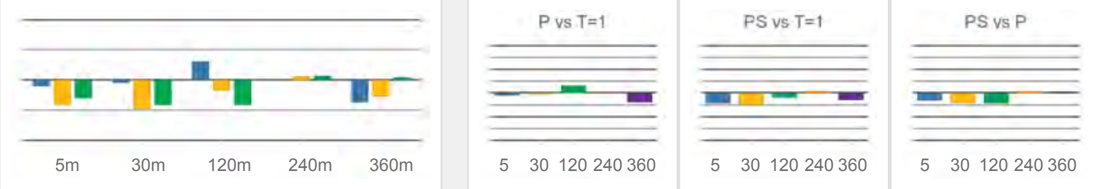

Locus		5m	30m	120m	240m	360m
**PGN_0357**	P vs T=1	0.149	0.369	0.524	0.502	0.130
	PS vs T=1	-0.874	1.319	3.183	3.042	2.570
	PS vs P	-1.052	0.911	2.616	2.485	2.382

ABC transporter membrane protein

transport and binding proteins

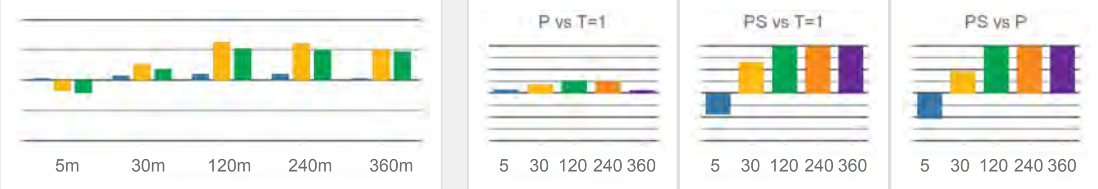

log$_2$ (Fold Change)

P vs T=1 | PS vs T=1 | PS vs P

Locus		5m	30m	120m	240m	360m
**PGN_0358**	P vs T=1	0.865	1.157	1.532	1.573	1.282
	PS vs T=1	0.704	1.702	3.646	3.460	3.087
	PS vs P	-0.193	0.521	2.098	1.864	1.777
	putative ABC transporter ATP-binding protein					
	transport and binding proteins					
**PGN_0359**	P vs T=1	0.518	0.690	0.839	1.082	0.974
	PS vs T=1	0.873	1.041	2.560	2.713	2.405
	PS vs P	0.312	0.319	1.681	1.606	1.408
	putative ABC transporter permease protein					
	transport and binding proteins					
**PGN_0360**	P vs T=1	-0.031	-0.027	-0.250	-0.675	-0.731
	PS vs T=1	0.837	0.431	-0.026	-0.144	-0.269
	PS vs P	0.858	0.452	0.216	0.486	0.436
	tyrosyl-tRNA synthetase					
	protein synthesis					
**PGN_0361** *wbbL*	P vs T=1	0.004	-0.480	-1.543	-1.487	-1.067
	PS vs T=1	0.676	-0.038	-0.722	-1.016	-1.138
	PS vs P	0.697	0.442	0.696	0.355	-0.086
	putative glycosyl transferase family 2					
	cell envelope					
**PGN_0362**	P vs T=1	0.615	-0.695	0.261	-0.478	1.989
	PS vs T=1	0.098	-0.084	0.825	2.008	1.074
	PS vs P	-0.222	-0.134	0.436	1.281	0.053
	hypothetical protein					
	hypothetical proteins					
**PGN_0363**	P vs T=1	0.135	0.202	1.526	2.588	3.434
	PS vs T=1	-0.214	-0.014	1.330	1.979	1.643
	PS vs P	-0.504	-0.426	0.111	0.216	-0.626
	conserved hypothetical protein					
	hypothetical proteins-Conserved					
**PGN_0364**	P vs T=1	-0.372	-0.963	0.467	1.722	2.931
	PS vs T=1	-0.295	-0.581	1.025	1.007	1.738
	PS vs P	-0.198	-0.382	0.480	0.035	-0.121
	hypothetical protein					
	hypothetical proteins					

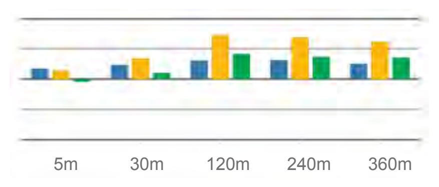

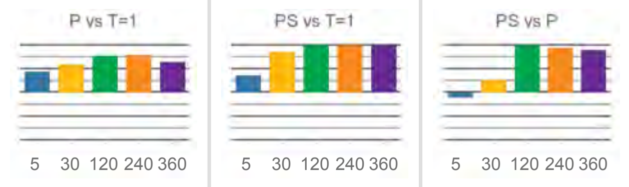

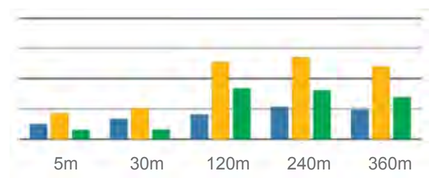

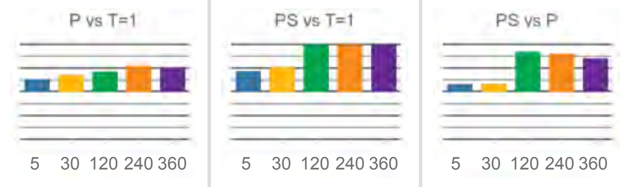

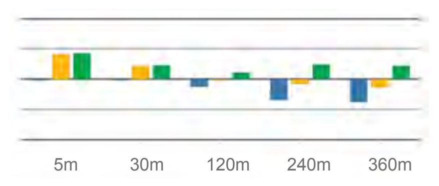

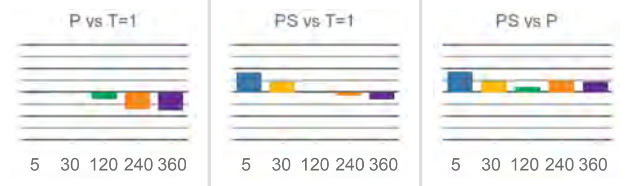

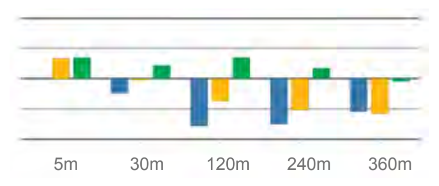

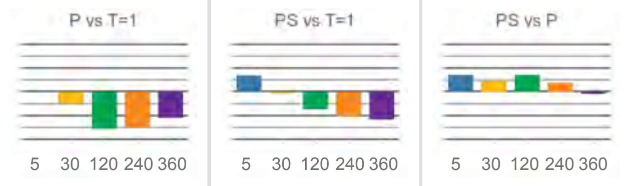

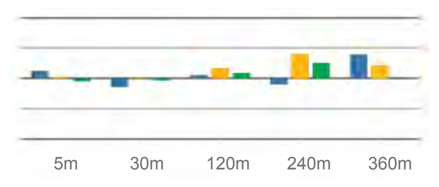

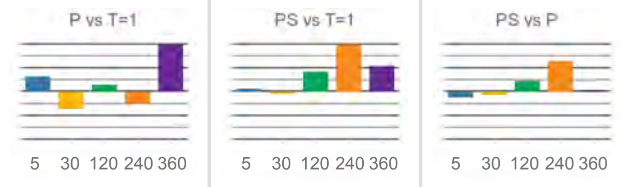

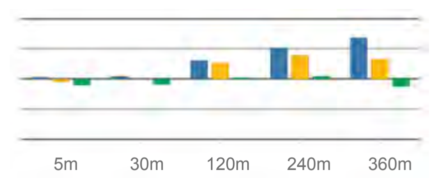

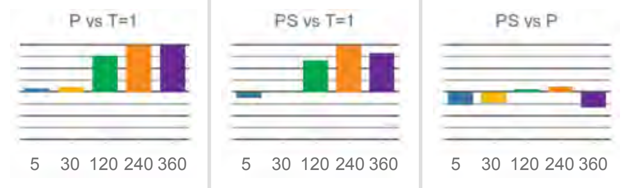

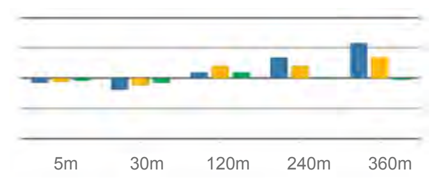

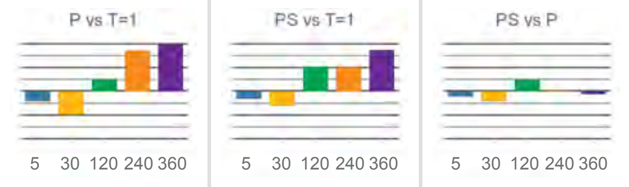

log2 (Fold Change)

Legend: P vs T=1 | PS vs T=1 | PS vs P

Locus		5m	30m	120m	240m	360m
**PGN_0365**	P vs T=1	-0.447	-0.848	-1.595	-1.767	-1.590
	PS vs T=1	-0.031	-0.240	-0.241	-0.366	-0.564
	PS vs P	0.420	0.580	1.260	1.278	0.967

arginyl-tRNA synthetase

protein synthesis

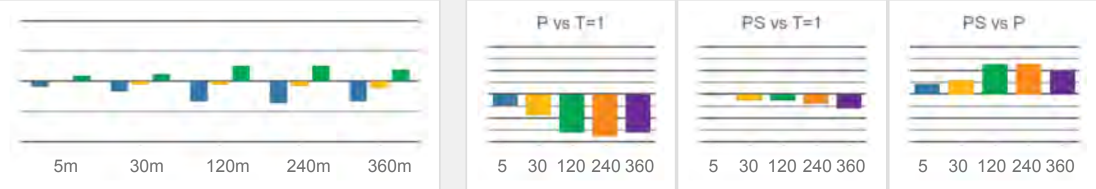

Locus		5m	30m	120m	240m	360m
**PGN_0366**	P vs T=1	-0.393	-0.722	-1.459	-1.540	-1.404
	PS vs T=1	0.618	0.092	-0.327	-0.514	-0.803
	PS vs P	1.006	0.795	1.045	0.908	0.549

putative tRNA 5-methylaminomethyl-2-thiouridylate 5-methyltransferase

protein synthesis

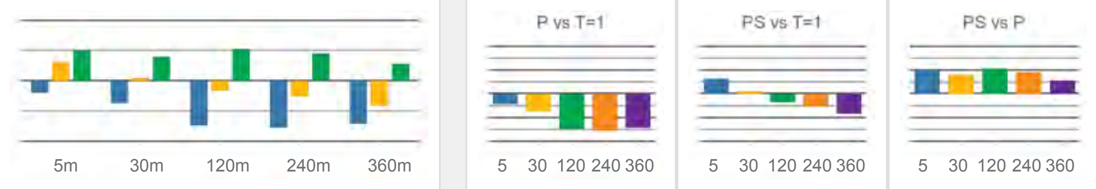

Locus		5m	30m	120m	240m	360m
**PGN_0367**	P vs T=1	-0.142	-0.725	-1.954	-1.818	-1.542
	PS vs T=1	0.560	0.085	-0.788	-1.142	-1.082
	PS vs P	0.738	0.786	0.911	0.464	0.377

exodeoxyribonuclease III

DNA metabolism

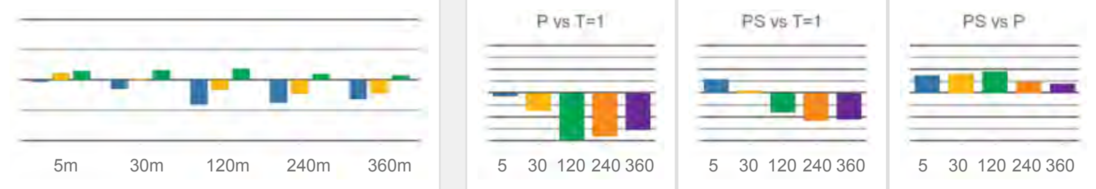

Locus		5m	30m	120m	240m	360m
**PGN_0368**	P vs T=1	0.692	0.241	-0.490	-0.821	-0.411
oxyR	PS vs T=1	1.228	0.801	-0.089	-0.710	-1.032
	PS vs P	0.567	0.563	0.348	-0.009	-0.621

redox-sensitive transcriptional activator OxyR

regulatory functions

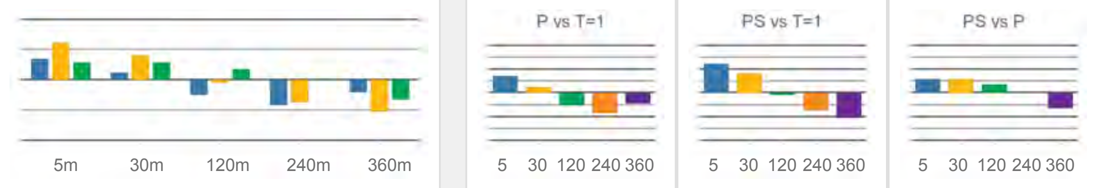

Locus		5m	30m	120m	240m	360m
**PGN_0369**	P vs T=1	0.285	-0.628	-1.414	-1.460	-1.369
	PS vs T=1	0.163	-0.123	-0.278	-0.104	-0.323
	PS vs P	-0.036	0.467	0.949	1.133	0.928

probable single-stranded binding protein

DNA metabolism

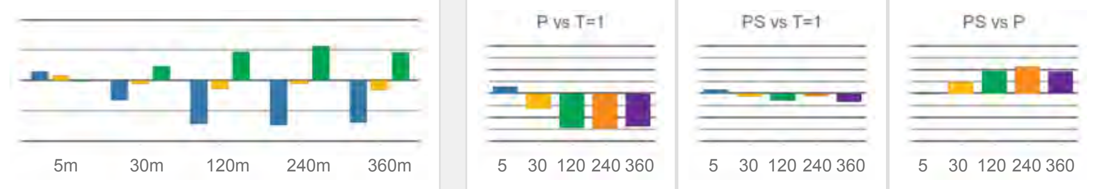

Locus		5m	30m	120m	240m	360m
**PGN_0370**	P vs T=1	-0.196	-1.180	-1.903	-1.617	-1.294
	PS vs T=1	-0.220	-0.602	-0.918	-0.896	-1.068
	PS vs P	0.041	0.538	0.851	0.646	0.211

conserved hypothetical protein

cellular processes

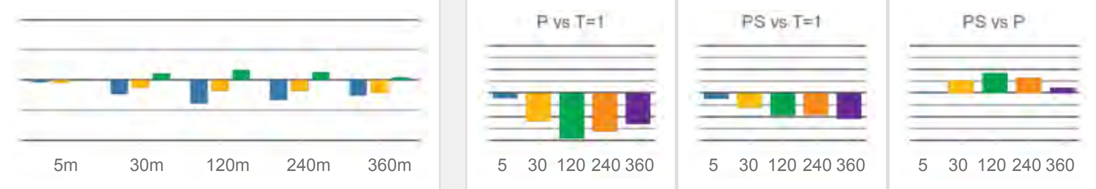

Locus		5m	30m	120m	240m	360m
**PGN_0371**	P vs T=1	-0.244	-0.746	-0.724	-0.317	-0.390
	PS vs T=1	-0.679	-0.707	0.001	-0.172	-0.032
	PS vs P	-0.420	0.010	0.673	0.154	0.347

conserved hypothetical protein

hypothetical proteins-Conserved

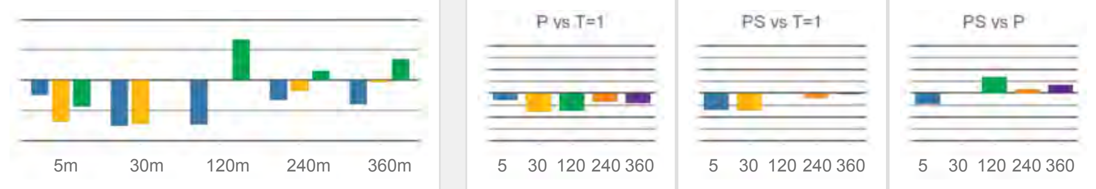

PgSg Time Course

Legend: P vs T=1 · PS vs T=1 · PS vs P

Locus		log$_2$ (Fold Change) 5m	30m	120m	240m	360m
**PGN_0372**	P vs T=1	0.627	-0.094	0.486	1.093	0.549
	PS vs T=1	-0.424	0.156	1.386	1.231	1.393
	PS vs P	-0.949	0.104	0.866	0.340	0.801

hypothetical protein

hypothetical proteins

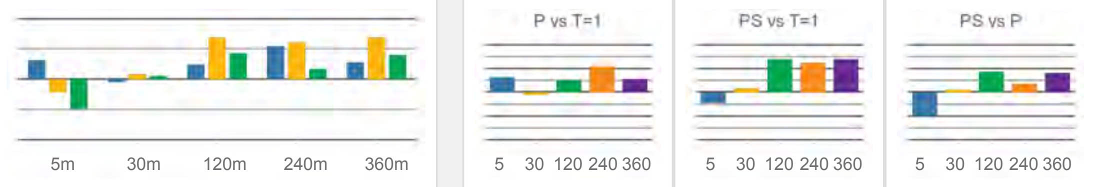

Locus		5m	30m	120m	240m	360m
**PGN_0373**	P vs T=1	0.674	1.519	1.692	1.492	0.836
	PS vs T=1	-0.674	1.681	3.317	2.851	2.580
	PS vs P	-1.389	0.226	1.656	1.349	1.673

putative thioredoxin

energy metabolism

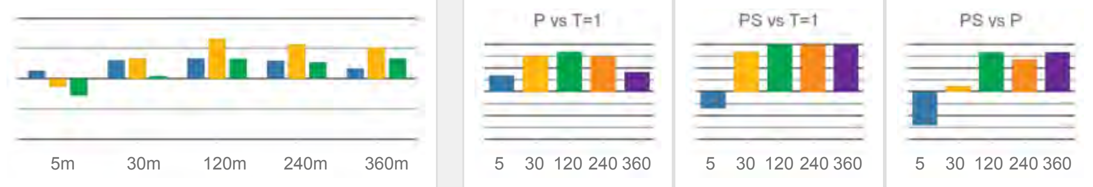

Locus		5m	30m	120m	240m	360m
**PGN_0374**	P vs T=1	0.849	0.329	-0.236	-0.494	-1.108
	PS vs T=1	0.544	0.451	0.351	0.184	-0.103
	PS vs P	-0.238	0.131	0.554	0.628	0.928

conserved hypothetical protein

hypothetical proteins-Conserved

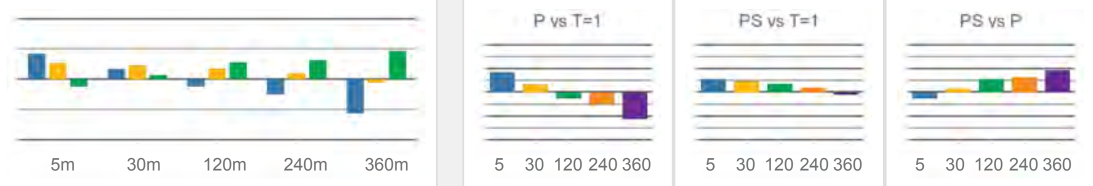

Locus		5m	30m	120m	240m	360m
**PGN_0375**	P vs T=1	0.675	0.844	0.915	0.773	0.154
	PS vs T=1	0.788	0.474	0.430	0.639	0.424
	PS vs P	0.113	-0.356	-0.469	-0.126	0.251

phosphoribulose/uridine kinase

unknown function

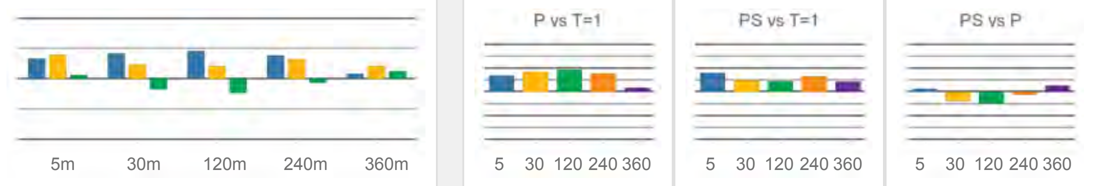

Locus		5m	30m	120m	240m	360m
**PGN_0376**	P vs T=1	-0.625	-0.167	-0.127	-0.103	-0.121
	PS vs T=1	-0.583	-0.328	0.023	0.544	0.547
	PS vs P	-0.002	-0.157	0.140	0.626	0.651

2-dehydro-3-deoxyphosphooctonate aldolase

cell envelope

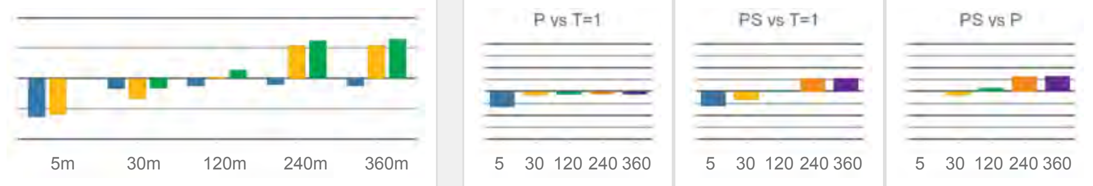

Locus		5m	30m	120m	240m	360m
**PGN_0377**	P vs T=1	0.662	0.834	0.854	0.716	0.202
	PS vs T=1	0.926	0.817	0.589	0.900	0.603
	PS vs P	0.261	0.000	-0.243	0.191	0.376

aspartate ammonia-lyase

energy metabolism

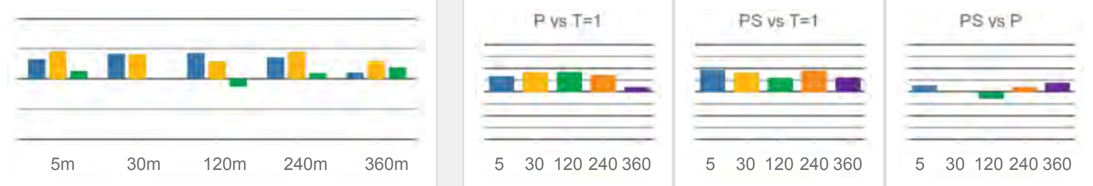

Locus		5m	30m	120m	240m	360m
**PGN_0378**	P vs T=1	0.324	0.037	-0.590	-0.700	-0.728
	PS vs T=1	0.628	0.027	-0.263	-0.223	-0.634
	PS vs P	0.331	0.005	0.284	0.419	0.062

putative exopolyphosphatase

hypothetical proteins-Conserved

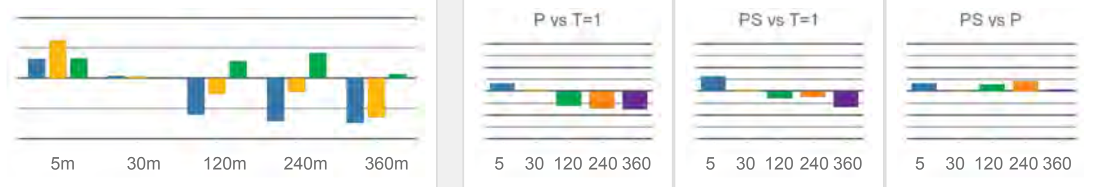

PgSg Time Course

Hackett Laboratory UW

log$_2$ (Fold Change)

Locus		5m	30m	120m	240m	360m
**PGN_0379**	P vs T=1	0.561	0.245	-0.560	-1.023	-0.331
	PS vs T=1	0.905	0.260	-0.612	-0.636	-0.974
	PS vs P	0.375	0.039	-0.083	0.242	-0.618
	conserved hypothetical protein					
	hypothetical proteins-Conserved					
**PGN_0380** *GlcK*	P vs T=1	0.371	0.259	-0.452	-0.894	-1.338
	PS vs T=1	0.056	0.361	0.762	0.936	0.649
	PS vs P	-0.292	0.117	1.171	1.729	1.891
	partial ROK family transcriptional repressor with glucose kinase domain					
	energy metabolism					
**PGN_0381**	P vs T=1	0.919	1.261	1.193	0.898	0.415
	PS vs T=1	0.742	1.015	1.473	1.667	1.502
	PS vs P	-0.181	-0.226	0.289	0.740	1.032
	partial ROK family transcriptional repressor with glucose kinase domain					
	regulatory functions					
**PGN_0382**	P vs T=1	-0.701	-0.328	-0.369	-0.230	0.336
	PS vs T=1	-0.499	-0.623	0.032	0.612	0.482
	PS vs P	0.108	-0.301	0.301	0.726	0.198
	conserved hypothetical protein					
	hypothetical proteins-Conserved					
**PGN_0383**	P vs T=1	0.502	0.305	0.167	0.973	1.741
	PS vs T=1	0.629	0.439	0.663	1.150	0.942
	PS vs P	0.127	0.103	0.362	0.260	-0.672
	probable transporter					
	transport and binding proteins					
**PGN_0384**	P vs T=1	-0.815	-1.134	-1.316	-1.232	-0.749
	PS vs T=1	-0.492	-0.721	-0.798	-0.609	-1.089
	PS vs P	0.318	0.354	0.396	0.497	-0.309
	conserved hypothetical protein					
	hypothetical proteins-Conserved					
**PGN_0385**	P vs T=1	-0.458	-0.760	-1.220	-1.051	-0.401
	PS vs T=1	-0.241	-0.141	0.678	0.725	0.385
	PS vs P	0.197	0.547	1.685	1.567	0.764
	putative integrase/recombinase XerD					
	cellular processes					

P vs T=1 PS vs T=1 PS vs P

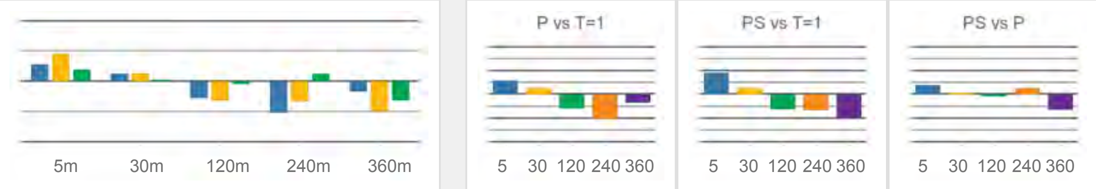

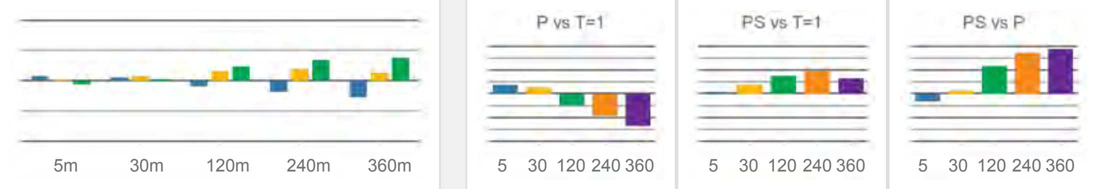

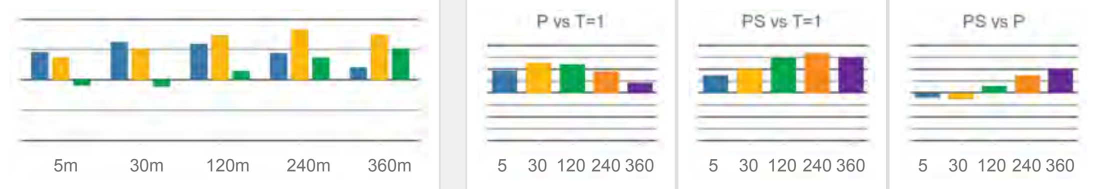

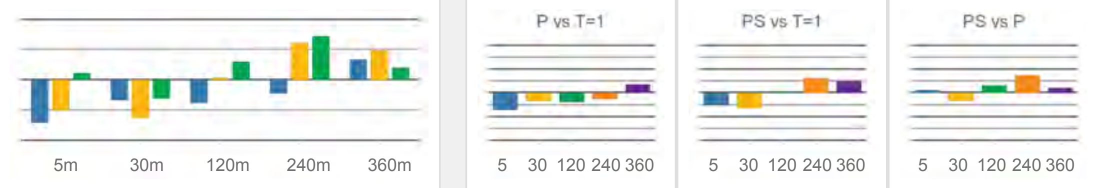

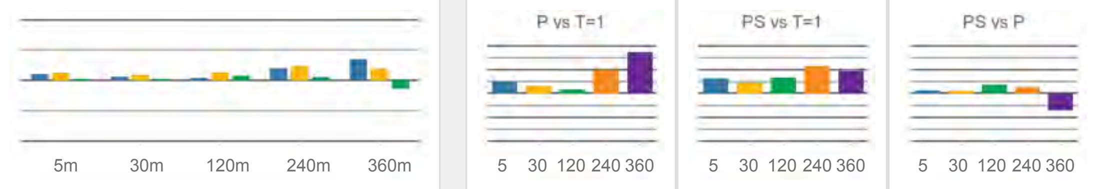

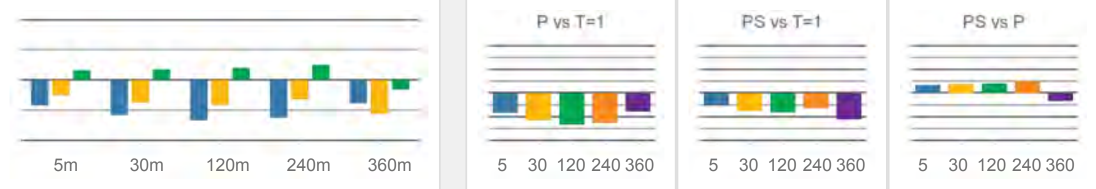

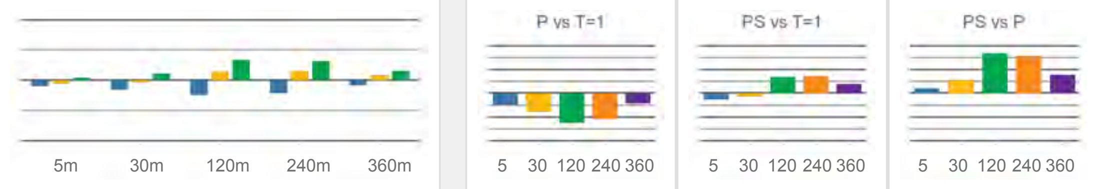

log$_2$ (Fold Change)

Legend: P vs T=1 | PS vs T=1 | PS vs P

Locus		5m	30m	120m	240m	360m
**PGN_0386**	P vs T=1	-0.491	-0.764	-1.385	-1.313	-1.329
	PS vs T=1	-0.759	-1.259	-1.400	-1.384	-1.458
	PS vs P	-0.225	-0.462	-0.092	-0.118	-0.169
	probable 3-dehydroquinate dehydratase type II					
	amino acid biosynthesis					

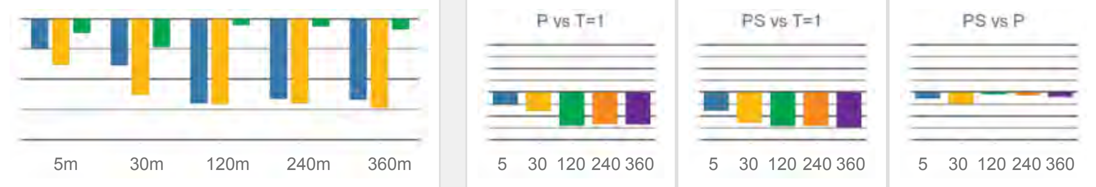

Locus		5m	30m	120m	240m	360m
**PGN_0387**	P vs T=1	0.267	0.074	-0.185	-0.086	0.284
	PS vs T=1	0.327	0.359	0.305	0.125	0.131
	PS vs P	0.066	0.272	0.425	0.155	-0.136
	putative O-methyltransferase					
	unknown function					

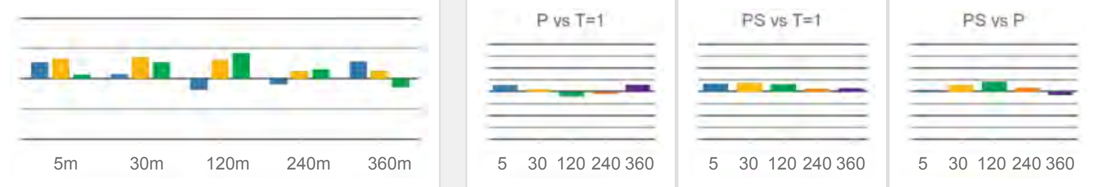

Locus		5m	30m	120m	240m	360m
**PGN_0388**	P vs T=1	-0.406	0.294	0.661	0.634	-0.042
	PS vs T=1	-1.742	0.447	2.065	2.375	2.293
	PS vs P	-1.385	0.157	1.407	1.726	2.278
	putative thiol peroxidase					
	cellular processes					

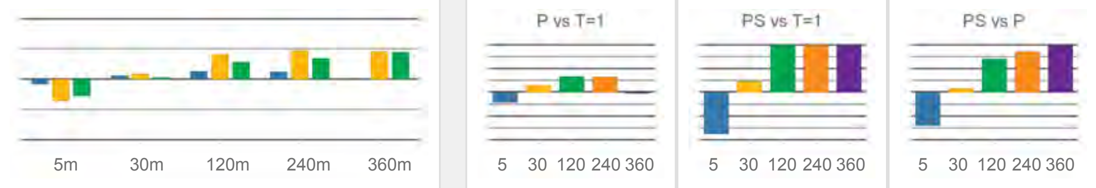

Locus		5m	30m	120m	240m	360m
**PGN_0389**	P vs T=1	-1.072	-1.265	-1.219	-1.015	-1.129
	PS vs T=1	-0.931	-1.321	-0.935	-0.901	-0.839
	PS vs P	0.135	-0.086	0.251	0.126	0.268
	putative cytidine/deoxycytidylate deaminase					
	unknown function					

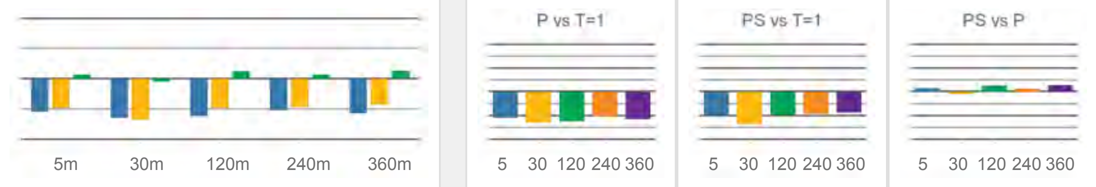

Locus		5m	30m	120m	240m	360m
**PGN_0390**	P vs T=1	-0.289	-0.448	-0.538	-0.495	-0.384
	PS vs T=1	0.001	-0.558	-0.905	-0.778	-0.624
	PS vs P	0.297	-0.110	-0.372	-0.276	-0.227
	conserved hypothetical protein					
	unknown function					

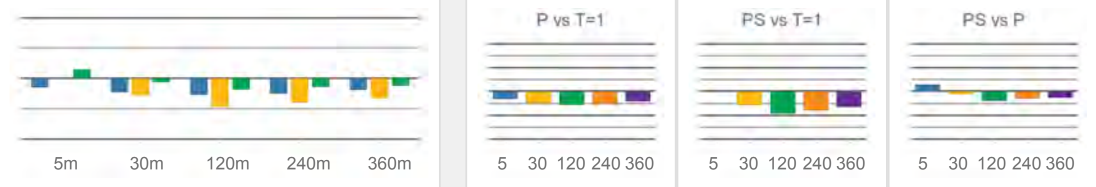

Locus		5m	30m	120m	240m	360m
**PGN_0391**	P vs T=1	0.159	0.354	0.360	0.028	-0.563
	PS vs T=1	-0.022	0.026	0.198	0.302	0.184
	PS vs P	-0.178	-0.310	-0.144	0.268	0.715
	conserved hypothetical protein					
	unknown function					

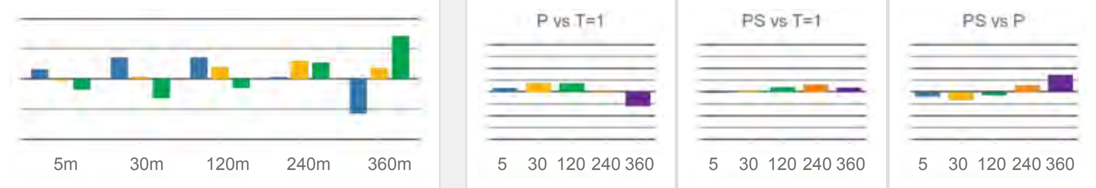

Locus		5m	30m	120m	240m	360m
**PGN_0392**	P vs T=1	-0.291	-0.529	-0.317	-0.326	0.524
	PS vs T=1	-0.489	-0.422	-0.271	0.314	0.236
	PS vs P	-0.193	0.043	0.020	0.538	-0.162
	conserved hypothetical protein with competence-damaged protein domain					
	hypothetical proteins-Conserved					

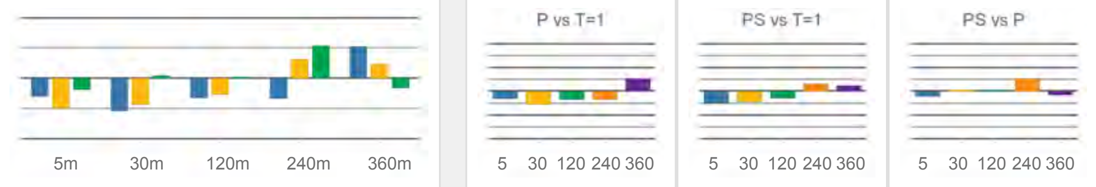

log$_2$ (Fold Change)

Legend: P vs T=1 | PS vs T=1 | PS vs P

Locus		5m	30m	120m	240m	360m
**PGN_0393**	P vs T=1	-0.226	-0.381	-0.639	-0.036	0.098
	PS vs T=1	-0.648	-0.693	-0.214	0.484	0.547
	PS vs P	-0.415	-0.319	0.339	0.531	0.455

putative O-sialoglycoprotein endopeptidase

protein fate

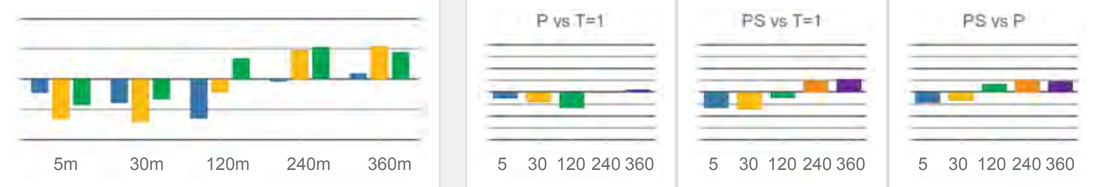

Locus		5m	30m	120m	240m	360m
**PGN_0394** *rpsT*	P vs T=1	-0.189	-0.559	-0.900	-0.874	-1.389
	PS vs T=1	-1.772	-1.406	-0.857	-0.597	-0.533
	PS vs P	-1.442	-0.779	0.025	0.271	0.759

probable 30S ribosomal protein S20

protein synthesis

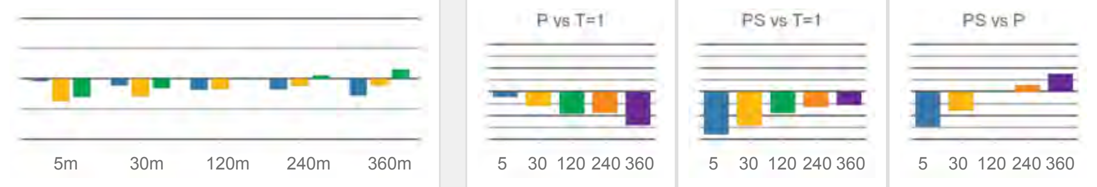

Locus		5m	30m	120m	240m	360m
**PGN_0395**	P vs T=1	0.073	-0.175	-0.248	0.309	1.411
	PS vs T=1	0.066	-0.221	-0.840	0.176	0.269
	PS vs P	0.026	-0.075	-0.674	-0.017	-0.825

conserved hypothetical protein

hypothetical proteins-Conserved

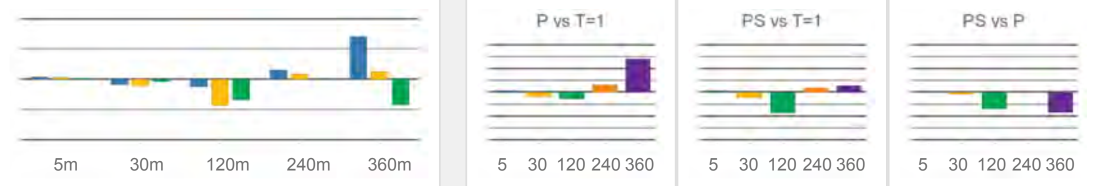

Locus		5m	30m	120m	240m	360m
**PGN_0396**	P vs T=1	0.444	0.593	0.405	0.066	0.194
	PS vs T=1	0.831	0.541	-0.131	-0.150	-0.200
	PS vs P	0.389	-0.034	-0.519	-0.227	-0.387

ribonuclease R

transcription

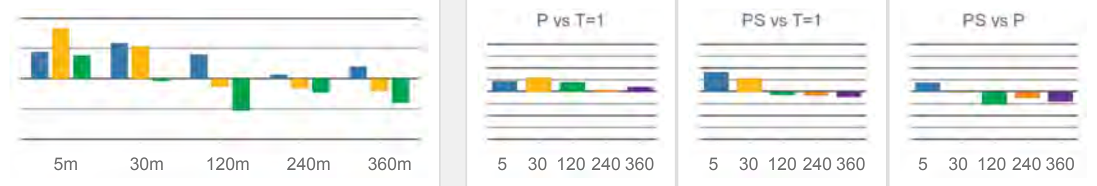

Locus		5m	30m	120m	240m	360m
**PGN_0397**	P vs T=1	-0.726	-0.721	-1.174	-1.340	-1.172
	PS vs T=1	-0.530	-0.693	-1.061	-0.811	-1.052
	PS vs P	0.195	0.033	0.088	0.478	0.104

putative auxin-regulated protein

hypothetical proteins-Conserved

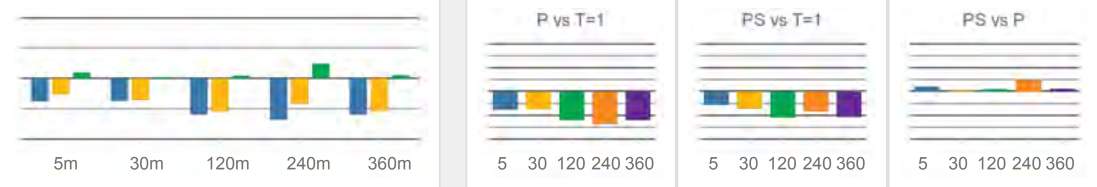

Locus		5m	30m	120m	240m	360m
**PGN_0398**	P vs T=1	-0.012	0.022	-0.378	-0.557	-0.349
	PS vs T=1	-0.248	-0.606	-1.177	-1.190	-1.079
	PS vs P	-0.220	-0.602	-0.785	-0.630	-0.710

ABC transporter ATP-binding protein MsbA family

transport and binding proteins

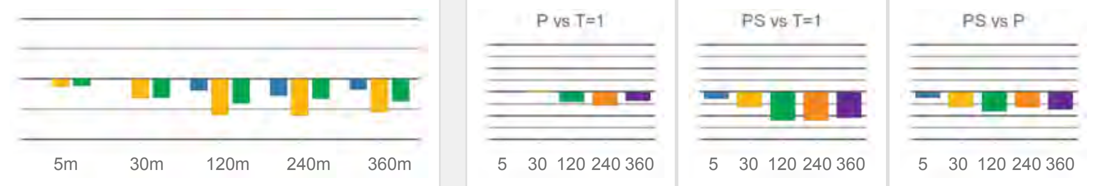

Locus		5m	30m	120m	240m	360m
**PGN_0399**	P vs T=1	1.385	0.918	0.529	2.031	3.120
	PS vs T=1	0.437	0.384	0.872	1.631	1.071
	PS vs P	-0.561	-0.389	-0.081	0.242	-0.882

conserved hypothetical protein

hypothetical proteins-Conserved

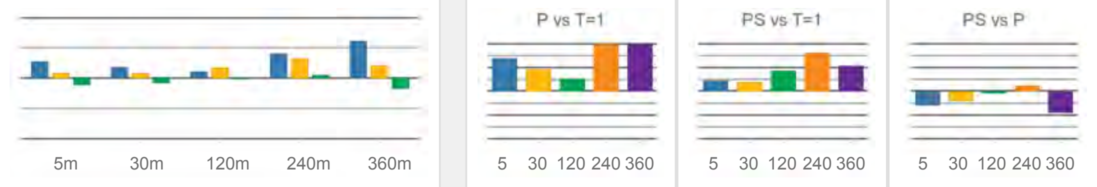

PgSg Time Course

log$_2$ (Fold Change)

Legend: P vs T=1 | PS vs T=1 | PS vs P

Locus		5m	30m	120m	240m	360m
**PGN_0400**	P vs T=1	1.721	2.052	2.444	2.707	2.461
	PS vs T=1	0.792	1.253	1.601	1.685	1.864
	PS vs P	-0.903	-0.751	-0.788	-0.953	-0.562

conserved hypothetical protein

hypothetical proteins-Conserved

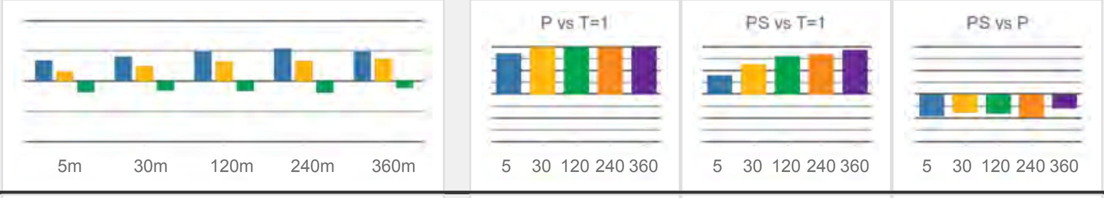

Locus		5m	30m	120m	240m	360m
**PGN_0401**	P vs T=1	0.204	0.231	1.711	2.507	3.646
	PS vs T=1	-0.646	-0.164	1.010	2.316	2.321
	PS vs P	-0.775	-0.510	-0.177	0.522	-0.096

conserved hypothetical protein

hypothetical proteins-Conserved

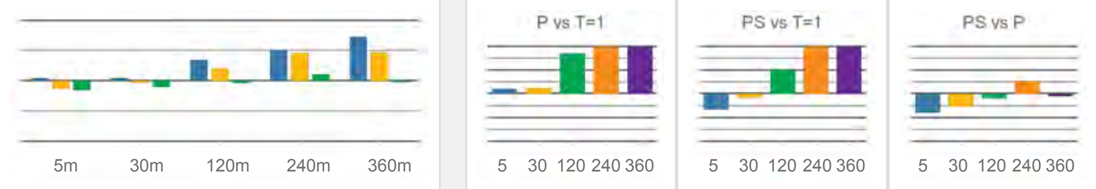

Locus		5m	30m	120m	240m	360m
**PGN_0402**	P vs T=1	1.186	0.725	1.501	2.378	3.504
	PS vs T=1	-0.126	0.317	0.905	1.438	2.152
	PS vs P	-0.929	-0.423	-0.353	-0.235	-0.533

hypothetical protein

hypothetical proteins

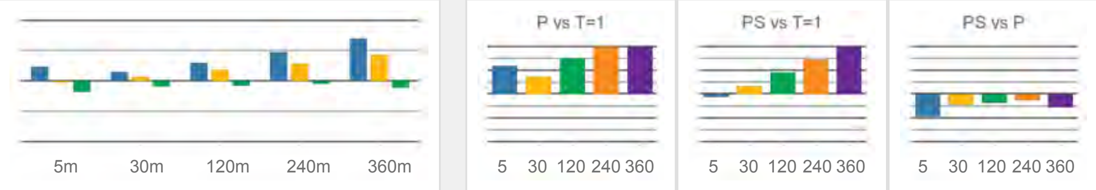

Locus		5m	30m	120m	240m	360m
**PGN_0403**	P vs T=1	-0.641	-0.718	-1.014	-0.907	-0.680
	PS vs T=1	-1.494	-1.262	-0.487	-0.267	-0.357
	PS vs P	-0.830	-0.526	0.480	0.598	0.322

putative pyridoxamine-phosphate oxidase

biosynthesis of cofactors, prosthetic groups, and carriers

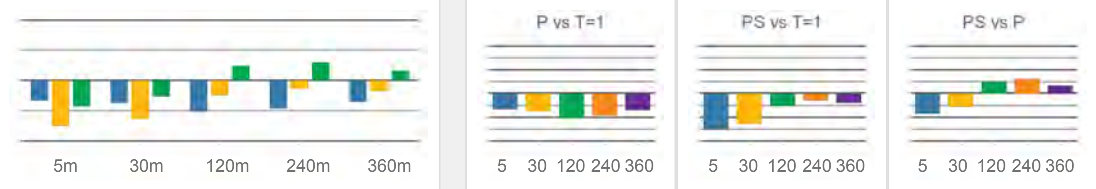

Locus		5m	30m	120m	240m	360m
**PGN_0404**	P vs T=1	-0.463	-0.526	-0.889	-0.647	-0.246
	PS vs T=1	-0.849	-0.922	-1.210	-1.030	-0.700
	PS vs P	-0.351	-0.355	-0.361	-0.336	-0.368

conserved hypothetical protein

hypothetical proteins-Conserved

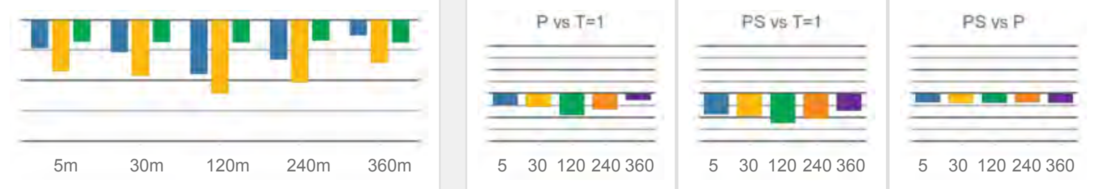

Locus		5m	30m	120m	240m	360m
**PGN_0405**	P vs T=1	-0.116	-0.350	-0.278	0.180	0.828
	PS vs T=1	-0.205	-0.662	-1.003	-0.758	-0.399
	PS vs P	-0.078	-0.311	-0.722	-0.877	-1.167

alpha-1,2-mannosidase family protein

cell envelope

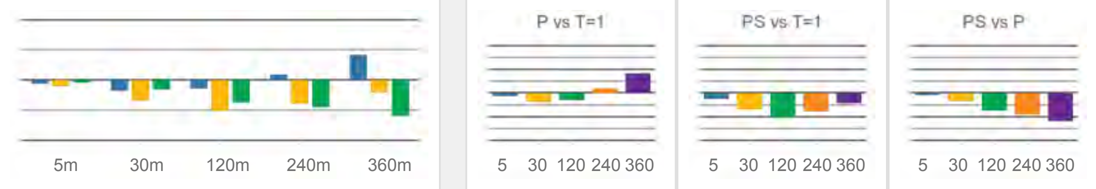

Locus		5m	30m	120m	240m	360m
**PGN_0406**	P vs T=1	0.002	-0.048	-0.078	-0.026	0.346
	PS vs T=1	0.356	-0.180	-1.002	-0.902	-0.779
	PS vs P	0.358	-0.126	-0.910	-0.849	-1.090

conserved hypothetical protein with glycosyl hydrolase family 92 domain

cell envelope

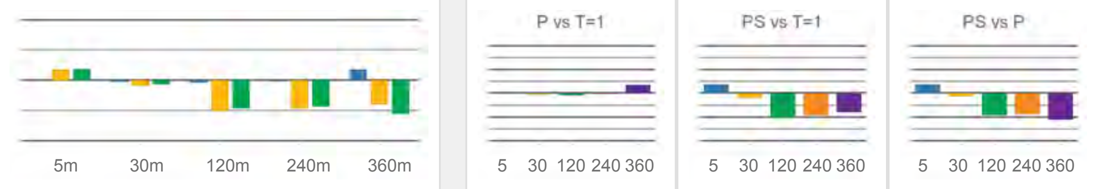

log$_2$ (Fold Change)

Legend: P vs T=1 | PS vs T=1 | PS vs P

Locus		5m	30m	120m	240m	360m
**PGN_0407**	P vs T=1	0.482	0.329	-0.173	0.242	0.768
	PS vs T=1	0.675	0.751	-0.483	-0.315	-0.017
	PS vs P	0.247	0.446	-0.438	-0.405	-0.508
	hypothetical protein					
	hypothetical proteins					

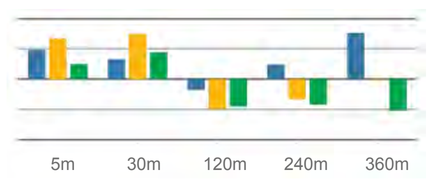

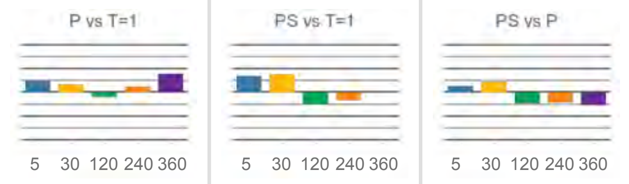

Locus		5m	30m	120m	240m	360m
**PGN_0408**	P vs T=1	0.484	-0.646	-1.328	-1.371	-1.673
	PS vs T=1	-0.558	-1.017	-1.255	-1.387	-1.379
	PS vs P	-0.899	-0.352	0.023	-0.055	0.230
	conserved hypothetical protein					
	hypothetical proteins-Conserved					

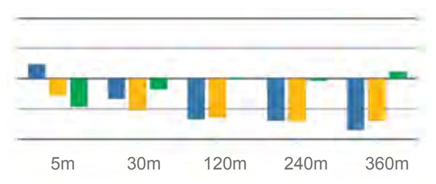

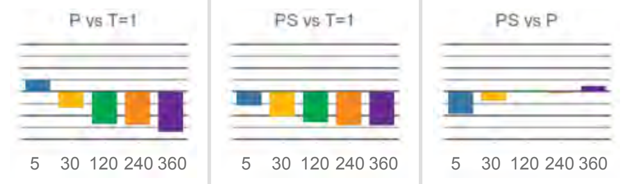

Locus		5m	30m	120m	240m	360m
**PGN_0409**	P vs T=1	-0.215	-1.092	-1.817	-1.667	-0.979
	PS vs T=1	-1.071	-1.653	-1.999	-1.677	-1.359
	PS vs P	-0.724	-0.541	-0.345	-0.135	-0.327
	conserved hypothetical protein					
	hypothetical proteins-Conserved					

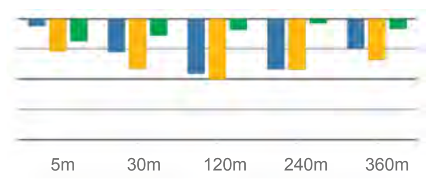

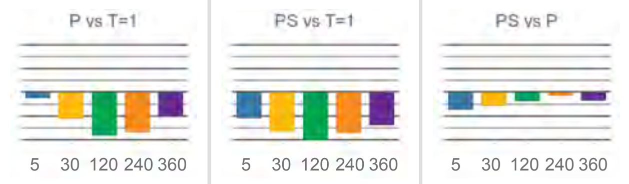

Locus		5m	30m	120m	240m	360m
**PGN_0410**	P vs T=1	-0.400	-0.867	-1.281	-1.296	-1.352
	PS vs T=1	-0.836	-1.258	-1.698	-1.857	-1.465
	PS vs P	-0.399	-0.378	-0.430	-0.565	-0.126
	probable RNA pseudouridylate synthase					
	transcription					

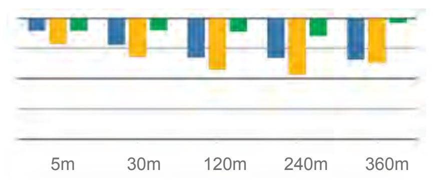

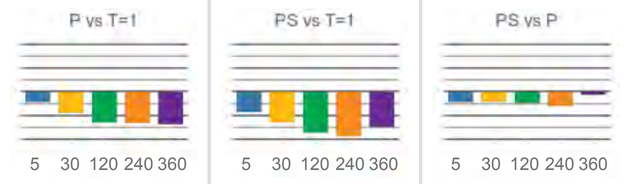

Locus		5m	30m	120m	240m	360m
**PGN_0411**	P vs T=1	0.481	0.872	0.777	0.315	-0.176
	PS vs T=1	1.263	1.196	0.765	0.535	0.525
	PS vs P	0.758	0.340	0.007	0.203	0.666
	thiol-disulfide interchange protein					
	protein fate					

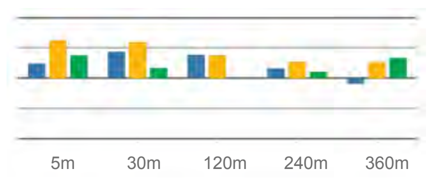

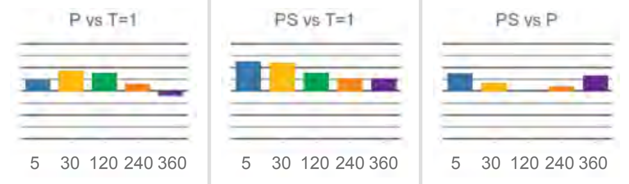

Locus		5m	30m	120m	240m	360m
**PGN_0412**	P vs T=1	-0.010	0.269	0.059	-0.598	-1.044
	PS vs T=1	1.194	1.159	0.480	-0.073	-0.349
	PS vs P	1.179	0.887	0.425	0.465	0.632
	conserved hypothetical protein					
	unknown function					

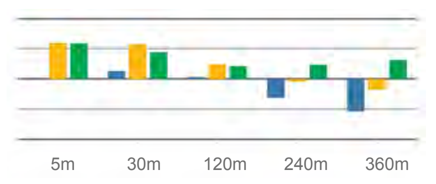

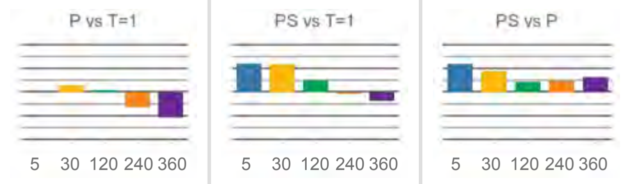

Locus		5m	30m	120m	240m	360m
**PGN_0413**	P vs T=1	0.072	0.254	0.023	-0.505	-1.303
gyrB	PS vs T=1	0.436	0.444	-0.128	-0.573	-0.817
	PS vs P	0.364	0.204	-0.139	-0.078	0.451
	DNA gyrase B subunit					
	DNA metabolism					

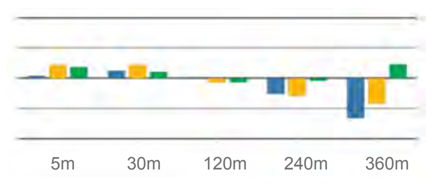

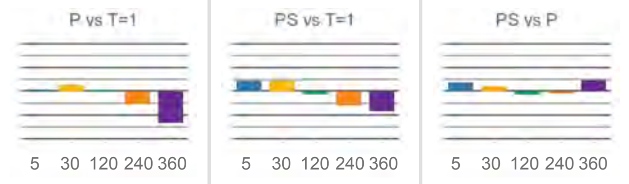

PgSg Time Course

log2 (Fold Change)

Legend: P vs T=1 | PS vs T=1 | PS vs P

Locus		5m	30m	120m	240m	360m
**PGN_0414**	P vs T=1	-0.113	-0.040	-0.053	-0.062	-0.007
	PS vs T=1	0.661	0.116	-0.637	-0.581	-0.361
	PS vs P	0.760	0.155	-0.575	-0.506	-0.344

probable membrane-bound dipeptidase

protein fate

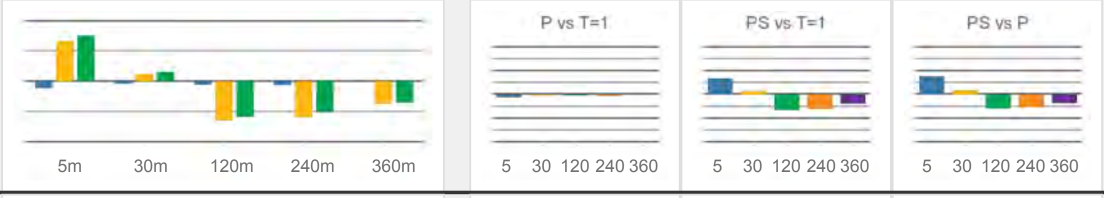

Locus		5m	30m	120m	240m	360m
**PGN_0415**	P vs T=1	-0.072	-0.058	-0.772	-0.773	-0.456
	PS vs T=1	0.355	0.474	-0.095	-0.162	-0.297
	PS vs P	0.423	0.532	0.639	0.570	0.151

restriction endonuclease

DNA metabolism

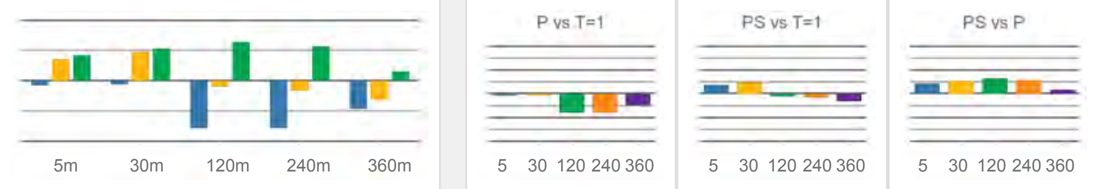

Locus		5m	30m	120m	240m	360m
**PGN_0416**	P vs T=1	-0.547	-1.208	-1.615	-1.515	-1.516
	PS vs T=1	0.074	-0.119	0.098	0.174	-0.218
	PS vs P	0.611	0.970	1.544	1.536	1.195

putative type II DNA modification methyltransferase

DNA metabolism

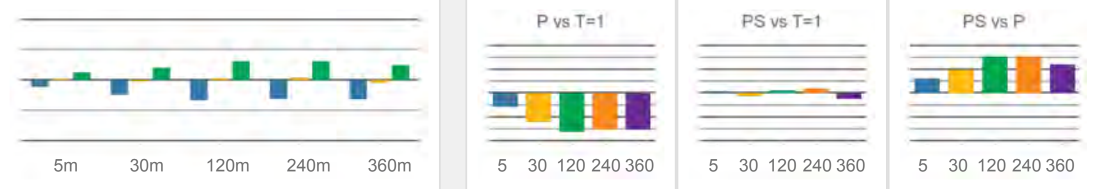

Locus		5m	30m	120m	240m	360m
**PGN_0417**	P vs T=1	-0.447	-1.431	-1.067	-0.606	0.212
	PS vs T=1	-0.486	-1.117	-0.550	-0.470	0.039
	PS vs P	0.059	-0.019	0.327	0.129	0.036

conserved hypothetical protein

hypothetical proteins-Conserved

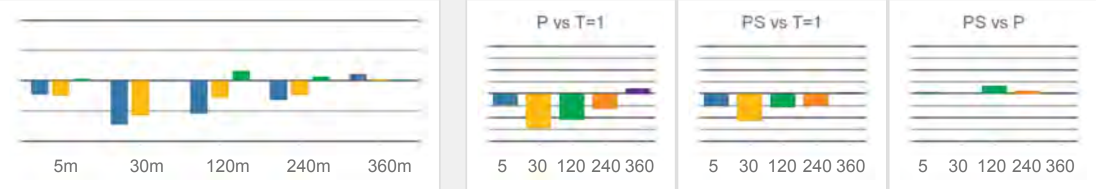

Locus		5m	30m	120m	240m	360m
**PGN_0418**	P vs T=1	-0.687	-0.260	0.069	0.180	-0.001
	PS vs T=1	-0.516	-0.313	-0.271	-0.045	-0.155
	PS vs P	0.125	-0.058	-0.323	-0.200	-0.153

conserved hypothetical protein with aminodeoxychorismate lyase domain

hypothetical proteins-Conserved

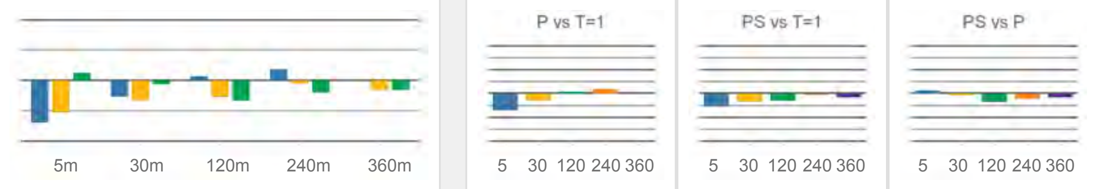

Locus		5m	30m	120m	240m	360m
**PGN_0419**	P vs T=1	-1.152	-0.866	-0.854	-0.837	-0.985
	PS vs T=1	-0.889	-0.999	-0.856	-0.635	-0.749
	PS vs P	0.213	-0.140	-0.020	0.178	0.191

putative dinucleotide-utilizing enzyme involved in molybdopterin and thiamine biosynthesis

unknown function

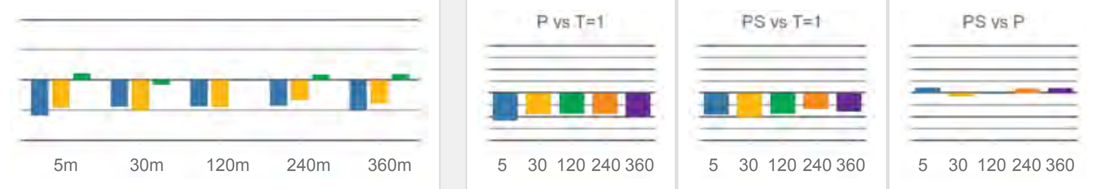

Locus		5m	30m	120m	240m	360m
**PGN_0420**	P vs T=1	-0.922	-0.795	-0.972	-0.833	-0.613
	PS vs T=1	-0.664	-0.981	-1.485	-1.244	-1.409
	PS vs P	0.243	-0.169	-0.520	-0.374	-0.738

putative lipoprotein releasing system ATP-binding protein

transport and binding proteins

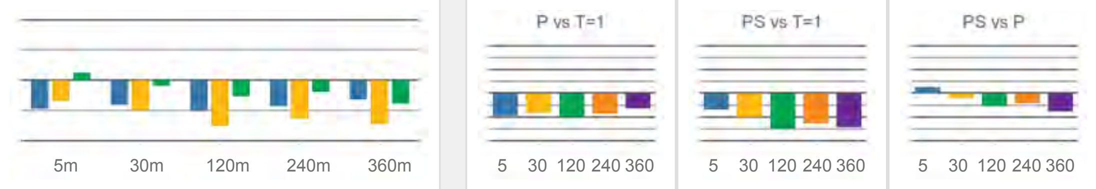

PgSg Time Course

log2 (Fold Change)

Legend: P vs T=1 | PS vs T=1 | PS vs P

Locus		5m	30m	120m	240m	360m
**PGN_0421**	P vs T=1	-0.037	-0.054	0.038	0.233	0.714
	PS vs T=1	0.545	0.228	-0.015	0.017	0.199
	PS vs P	0.538	0.233	-0.085	-0.188	-0.401

conserved hypothetical protein

hypothetical proteins-Conserved

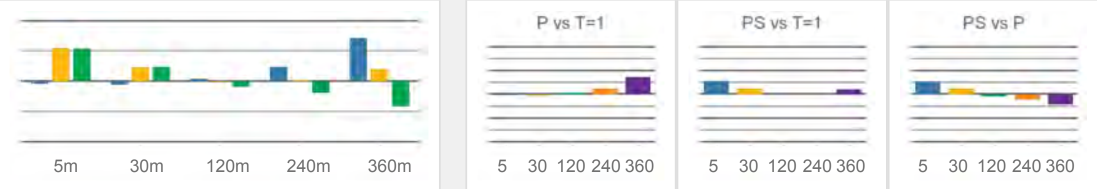

Locus		5m	30m	120m	240m	360m
**PGN_0422**	P vs T=1	-0.390	0.049	0.221	0.150	0.042
	PS vs T=1	0.678	0.819	0.767	0.590	0.433
	PS vs P	1.002	0.746	0.541	0.417	0.368

conserved hypothetical protein

unknown function

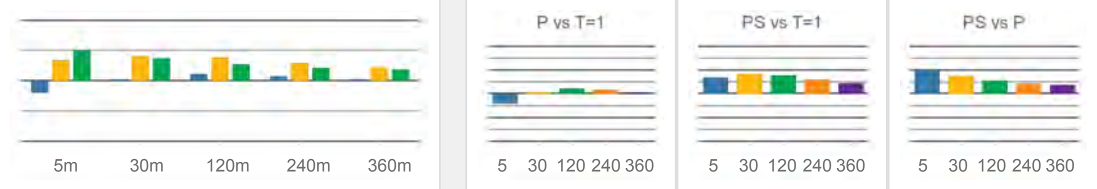

Locus		5m	30m	120m	240m	360m
**PGN_0423**	P vs T=1	-0.407	-0.518	-0.787	-1.137	-1.826
	PS vs T=1	-0.695	-0.066	0.392	0.456	0.380
	PS vs P	-0.279	0.443	1.147	1.511	2.086

putative transcription elongation factor

transcription

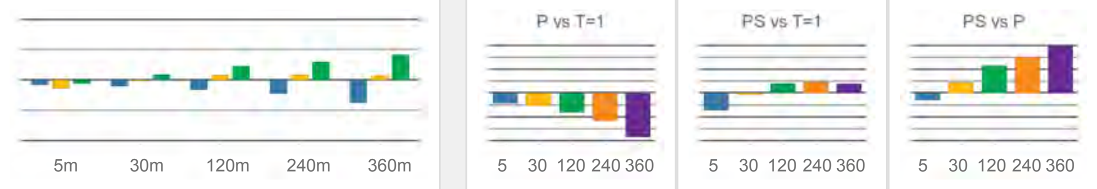

Locus		5m	30m	120m	240m	360m
**PGN_0424**	P vs T=1	-0.758	-0.676	-0.590	-0.553	-0.979
	PS vs T=1	-1.276	-0.616	-0.119	0.250	0.236
	PS vs P	-0.525	0.053	0.463	0.793	1.167

putative HIT family protein

unknown function

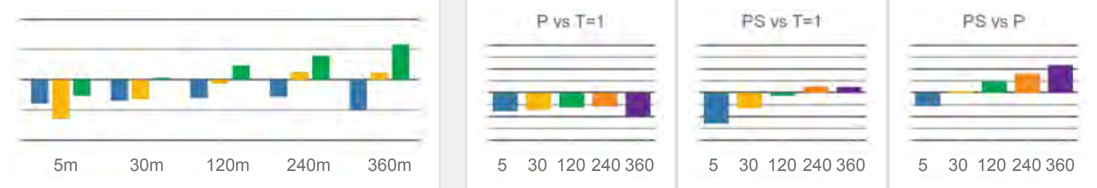

Locus		5m	30m	120m	240m	360m
**PGN_0425**	P vs T=1	0.314	0.158	0.508	-0.043	-0.050
	PS vs T=1	-0.227	0.065	0.375	0.367	0.536
	PS vs P	-0.469	-0.099	-0.001	0.255	0.491

partial transposase in ISPg3

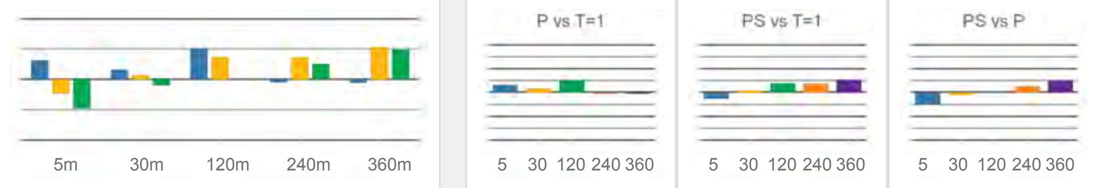

Locus		5m	30m	120m	240m	360m
**PGN_0426**	P vs T=1	0.608	1.307	1.634	1.120	0.162
	PS vs T=1	0.534	1.286	1.771	1.754	1.588
	PS vs P	-0.148	0.008	0.195	0.608	1.316

conserved hypothetical protein

hypothetical proteins-Conserved

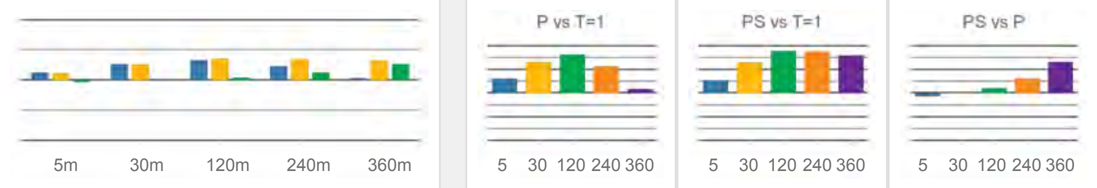

Locus		5m	30m	120m	240m	360m
**PGN_0427**	P vs T=1	1.321	1.705	1.628	1.045	0.200
	PS vs T=1	1.989	2.523	2.550	2.295	2.102
	PS vs P	0.620	0.808	0.916	1.182	1.797

putative glycosyl hydrolase

hypothetical proteins-Conserved

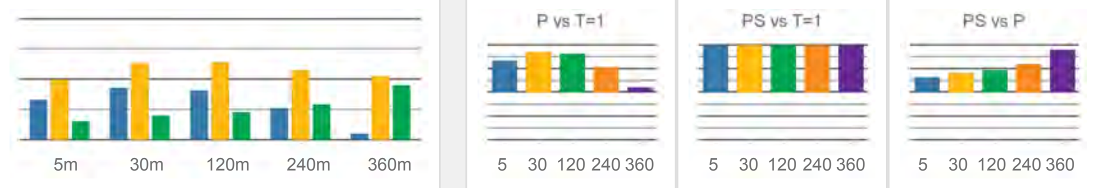

PgSg Time Course

log2 (Fold Change) — Legend: P vs T=1, PS vs T=1, PS vs P

Locus		5m	30m	120m	240m	360m
PGN_0428	P vs T=1	1.292	1.739	1.235	0.713	0.466
	PS vs T=1	2.031	1.916	1.567	1.255	0.937
	PS vs P	0.712	0.203	0.319	0.467	0.413

putative glycosyl transferase family 1

cell envelope

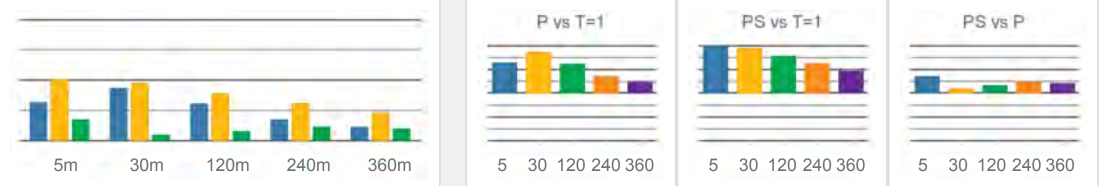

Locus		5m	30m	120m	240m	360m
PGN_0429	P vs T=1	-0.086	0.195	0.201	0.260	-0.118
	PS vs T=1	-0.039	-0.148	-0.126	0.105	0.172
	PS vs P	0.037	-0.342	-0.330	-0.156	0.277

putative 4-alpha-glucanotransferase

unknown function

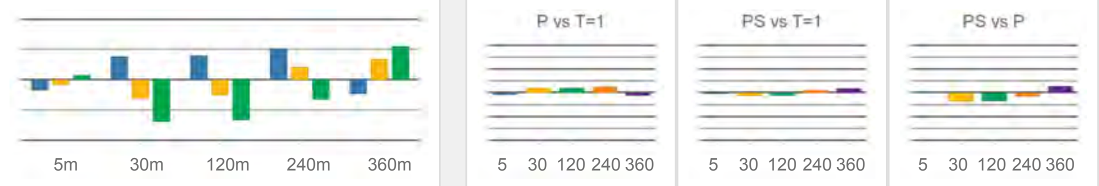

Locus		5m	30m	120m	240m	360m
PGN_0430	P vs T=1	0.989	1.125	1.229	1.586	2.194
	PS vs T=1	1.648	1.798	1.935	2.071	1.934
	PS vs P	0.585	0.613	0.634	0.511	-0.126

putative ATP-binding component of ABC transporter protein

transport and binding proteins

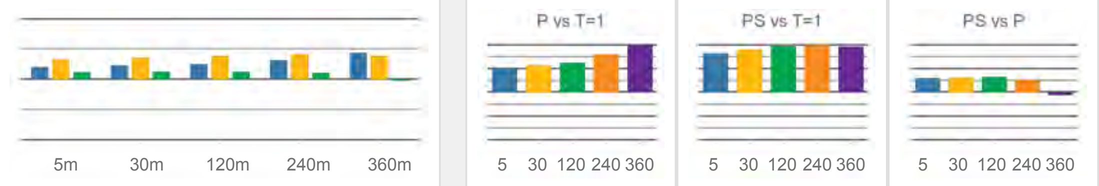

Locus		5m	30m	120m	240m	360m
PGN_0431	P vs T=1	-0.174	-0.348	0.097	1.081	1.947
	PS vs T=1	-0.221	-0.574	-0.064	0.174	0.432
	PS vs P	-0.061	-0.286	-0.189	-0.652	-1.287

conserved hypothetical protein

hypothetical proteins-Conserved

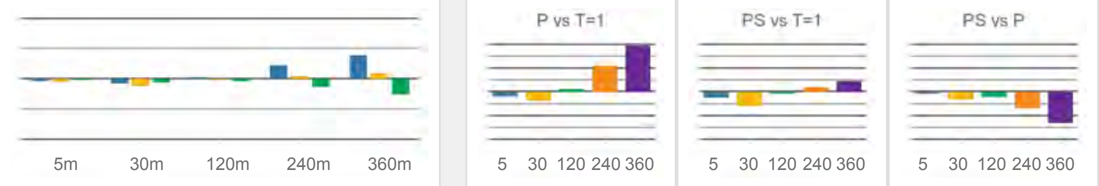

Locus		5m	30m	120m	240m	360m
PGN_0432	P vs T=1	-0.719	-0.111	-0.098	1.076	1.971
	PS vs T=1	-0.566	-0.958	-0.677	0.534	0.040
	PS vs P	-0.227	-0.548	-0.568	0.147	-0.573

conserved hypothetical protein

hypothetical proteins-Conserved

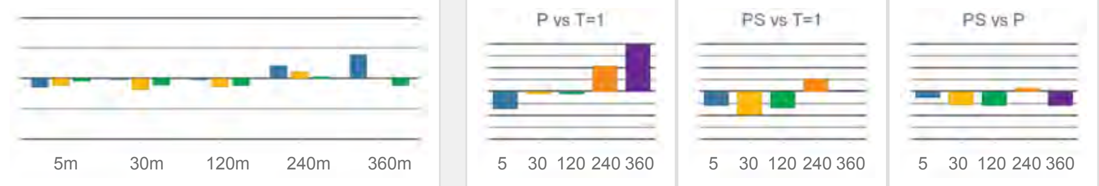

Locus		5m	30m	120m	240m	360m
PGN_0433	P vs T=1	-0.032	0.355	1.026	1.097	0.542
	PS vs T=1	0.499	0.437	0.955	1.647	1.245
	PS vs P	0.474	0.063	-0.039	0.577	0.685

phosphoglycerate kinase

energy metabolism

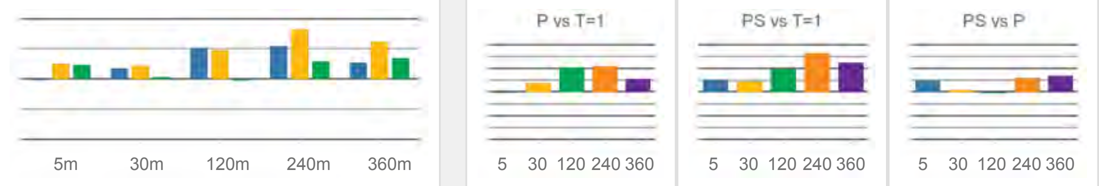

Locus		5m	30m	120m	240m	360m
PGN_0434	P vs T=1	-0.132	0.168	0.914	0.881	0.334
	PS vs T=1	0.472	0.628	0.842	1.233	1.154
	PS vs P	0.514	0.410	-0.020	0.382	0.788

phosphoenolpyruvate carboxykinase

energy metabolism

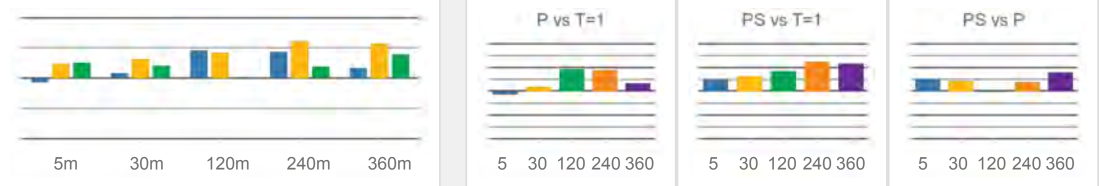

PgSg Time Course

log$_2$ (Fold Change)

Locus		5m	30m	120m	240m	360m
**PGN_0435**	P vs T=1	-0.017	0.230	1.096	2.377	2.898
	PS vs T=1	-0.316	-0.053	0.542	1.002	1.607
	PS vs P	-0.489	-0.369	-0.342	-0.280	-0.279
	probable partial hemagglutinin-related protein					
	unknown function					
**PGN_0436**	P vs T=1	1.176	1.077	2.327	3.351	3.773
	PS vs T=1	0.254	0.556	1.926	2.278	3.113
	PS vs P	-0.798	-0.578	-0.147	-0.299	-0.108
	probable partial hemagglutinin-related protein					
	unknown function					
**PGN_0437**	P vs T=1	-2.293	-1.626	-2.023	-2.083	-2.271
	PS vs T=1	-2.426	-2.206	-2.308	-2.189	-2.132
	PS vs P	-0.295	-0.414	-0.303	-0.136	0.038
	conserved hypothetical protein					
	hypothetical proteins-Conserved					
**PGN_0438**	P vs T=1	1.802	2.238	3.032	3.104	2.129
	PS vs T=1	2.274	2.722	2.798	3.033	2.991
	PS vs P	0.384	0.479	-0.071	0.085	0.828
	conserved hypothetical protein					
	hypothetical proteins-Conserved					
**PGN_0439**	P vs T=1	-1.761	-1.035	-0.542	-0.840	-1.474
	PS vs T=1	-1.614	-1.195	-1.093	-0.823	-0.705
	PS vs P	-0.158	-0.163	-0.414	0.016	0.588
	conserved hypothetical protein					
	hypothetical proteins-Conserved					
**PGN_0440**	P vs T=1	-0.994	-0.747	-1.248	-1.709	-2.543
	PS vs T=1	-1.312	-1.283	-1.986	-2.896	-2.870
	PS vs P	-0.227	-0.353	-0.641	-1.142	-0.433
	conserved hypothetical protein					
	hypothetical proteins-Conserved					
**PGN_0441**	P vs T=1	0.647	0.876	1.853	2.513	3.164
	PS vs T=1	0.353	0.923	2.092	2.375	2.424
	PS vs P	-0.414	-0.079	0.349	0.198	-0.380
	hypothetical protein					
	hypothetical proteins					

P vs T=1 PS vs T=1 PS vs P

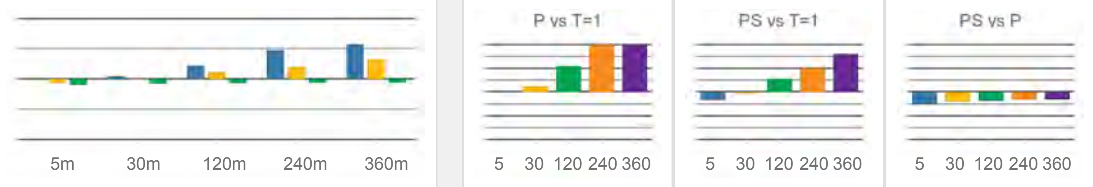

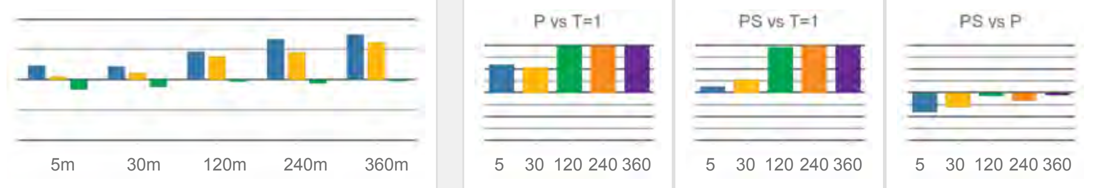

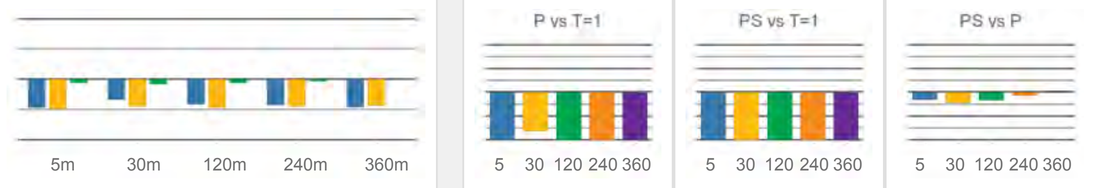

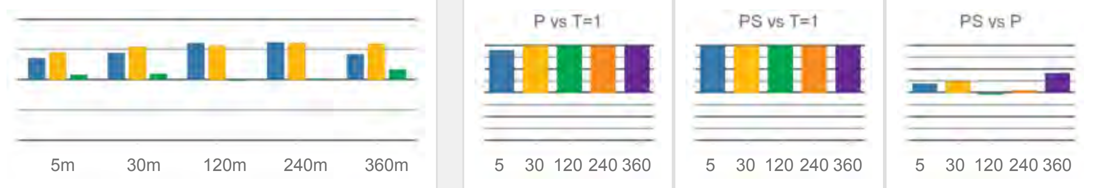

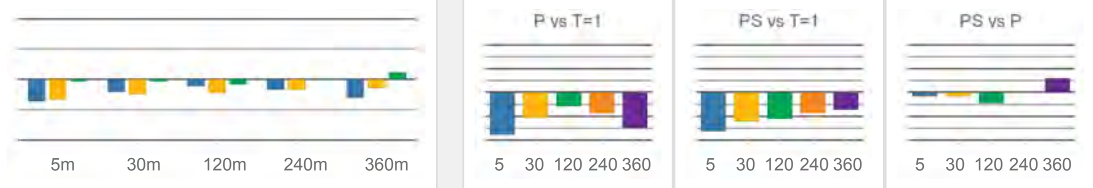

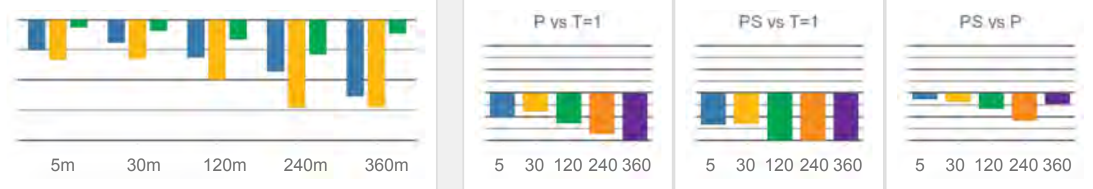

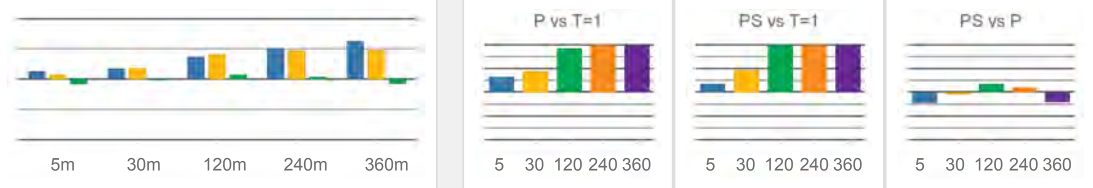

PgSg Time Course

log$_2$ (Fold Change)

P vs T=1 | PS vs T=1 | PS vs P

Locus		5m	30m	120m	240m	360m
**PGN_0442**	P vs T=1	0.413	-0.176	0.055	0.527	1.647
	PS vs T=1	-0.121	0.062	0.509	0.521	1.022
	PS vs P	-0.490	0.121	0.345	-0.009	-0.480

transposase in ISPg3

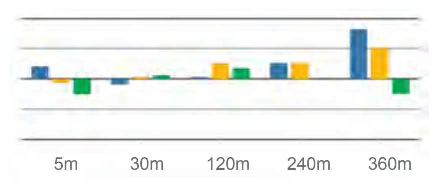

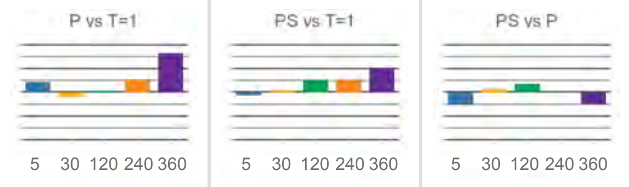

Locus		5m	30m	120m	240m	360m
**PGN_0443**	P vs T=1	-0.338	-0.742	-0.460	0.632	1.064
	PS vs T=1	-0.971	-0.887	-0.500	-0.236	0.221
	PS vs P	-0.541	-0.325	-0.217	-0.273	-0.279

hypothetical protein

hypothetical proteins

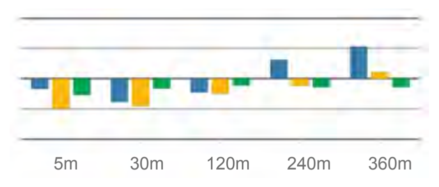

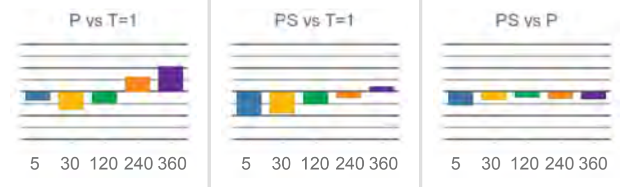

Locus		5m	30m	120m	240m	360m
**PGN_0444**	P vs T=1	0.999	1.471	2.109	2.775	2.775
	PS vs T=1	1.043	1.236	1.525	2.050	2.449
	PS vs P	-0.013	-0.235	-0.539	-0.611	-0.260

probable outer membrane efflux protein

transport and binding proteins

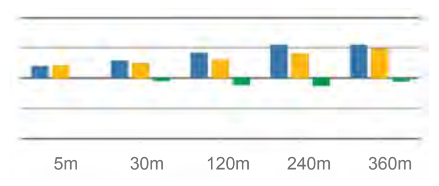

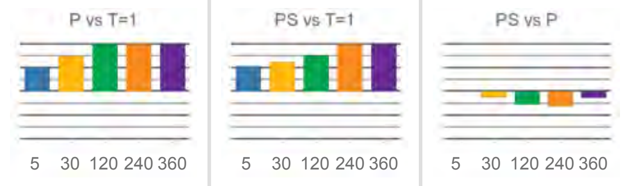

Locus		5m	30m	120m	240m	360m
**PGN_0445**	P vs T=1	0.762	1.050	1.420	1.805	1.648
	PS vs T=1	0.666	0.829	0.937	1.383	1.670
	PS vs P	-0.111	-0.216	-0.462	-0.370	0.041

membrane fusion efflux protein

transport and binding proteins

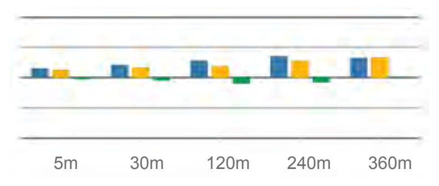

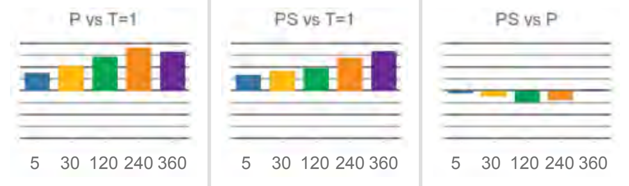

Locus		5m	30m	120m	240m	360m
**PGN_0446**	P vs T=1	0.740	0.823	1.248	1.837	2.192
	PS vs T=1	0.712	0.802	1.363	1.921	2.417
	PS vs P	-0.045	-0.040	0.105	0.116	0.251

putative ABC transporter ATP-binding protein

transport and binding proteins

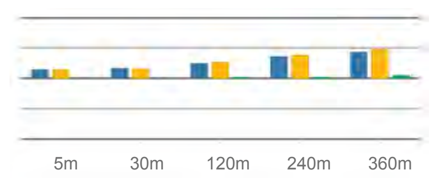

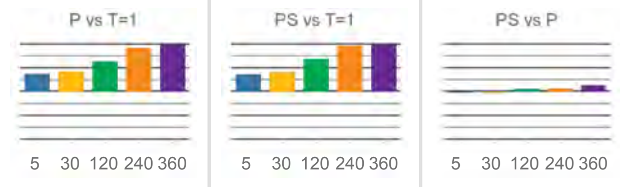

Locus		5m	30m	120m	240m	360m
**PGN_0447**	P vs T=1	0.597	0.744	1.189	1.697	1.848
	PS vs T=1	0.752	0.934	1.381	2.043	2.570
	PS vs P	0.129	0.167	0.183	0.364	0.733

putative ABC transporter permease protein

transport and binding proteins

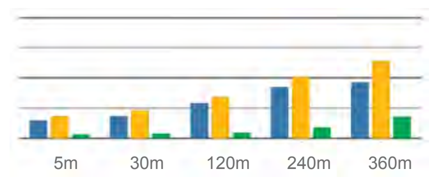

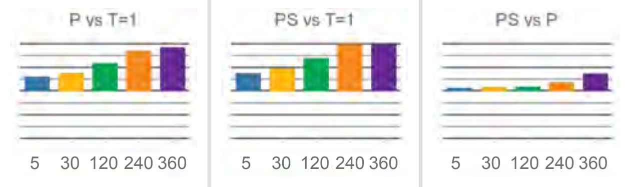

Locus		5m	30m	120m	240m	360m
**PGN_0448**	P vs T=1	0.665	0.983	1.406	1.868	1.704
	PS vs T=1	0.707	0.874	1.367	1.904	2.227
	PS vs P	-0.016	-0.126	-0.031	0.098	0.538

putative ABC transporter ATP-binding protein

transport and binding proteins

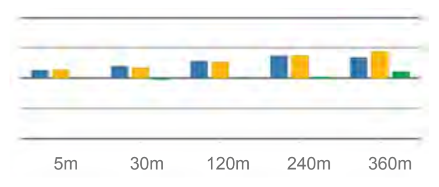

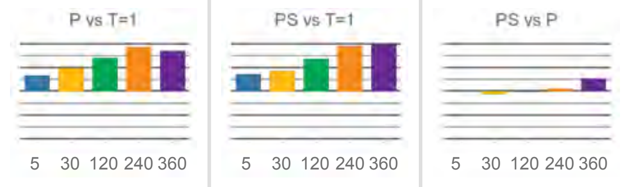

log$_2$ (Fold Change)

P vs T=1 | PS vs T=1 | PS vs P

Locus		5m	30m	120m	240m	360m
**PGN_0449**	P vs T=1	0.785	1.355	1.850	2.051	1.637
	PS vs T=1	0.841	1.235	1.908	2.251	2.599
	PS vs P	0.012	-0.119	0.080	0.233	0.951

conserved hypothetical protein

hypothetical proteins-Conserved

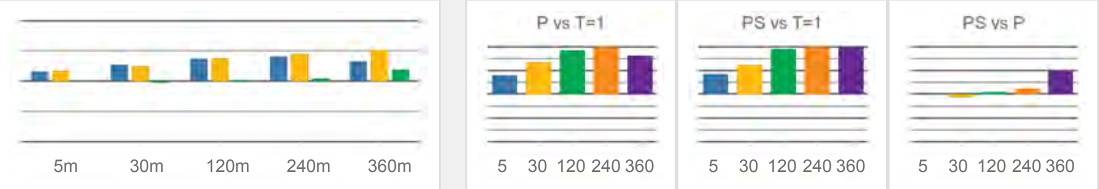

Locus		5m	30m	120m	240m	360m
**PGN_0450**	P vs T=1	-0.276	0.042	0.428	0.571	0.638
	PS vs T=1	-0.098	-0.068	0.357	0.924	1.342
	PS vs P	0.099	-0.138	-0.065	0.358	0.704

putative RNA polymerase sigma-70 factor ECF subfamily

transcription

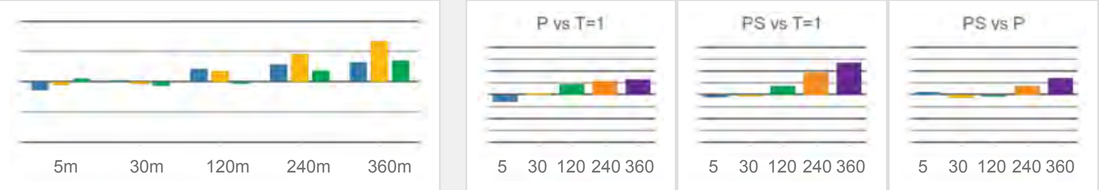

Locus		5m	30m	120m	240m	360m
**PGN_0451**	P vs T=1	0.946	1.081	1.367	1.732	1.749
	PS vs T=1	0.973	1.193	1.635	2.093	2.506
	PS vs P	-0.005	0.085	0.248	0.384	0.763

conserved hypothetical protein

hypothetical proteins-Conserved

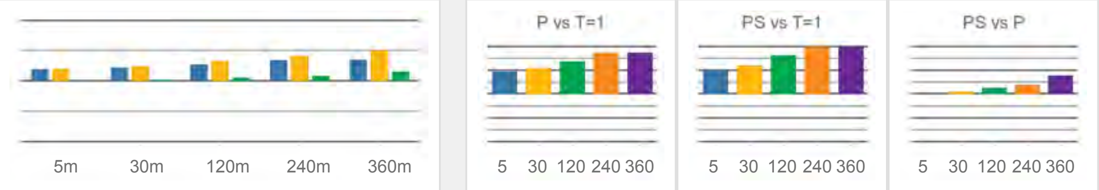

Locus		5m	30m	120m	240m	360m
**PGN_0452**	P vs T=1	1.010	1.188	1.371	1.755	1.753
	PS vs T=1	0.514	0.826	1.327	1.276	2.338
	PS vs P	-0.482	-0.319	-0.035	-0.289	0.651

conserved hypothetical protein

hypothetical proteins-Conserved

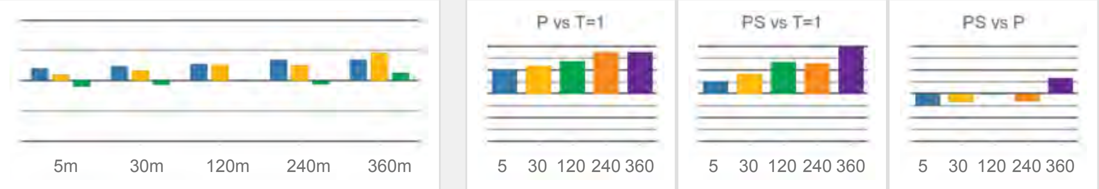

Locus		5m	30m	120m	240m	360m
**PGN_0453**	P vs T=1	1.424	1.468	1.974	2.582	2.471
	PS vs T=1	1.007	1.424	2.050	2.263	2.945
	PS vs P	-0.416	-0.076	0.102	-0.107	0.558

partial transposase Orf1 in ISPg5

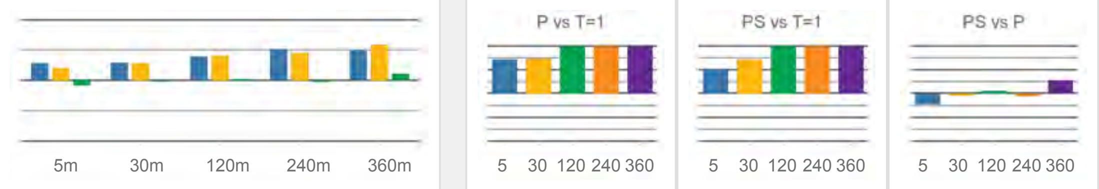

Locus		5m	30m	120m	240m	360m
**PGN_0454**	P vs T=1	0.440	-0.028	0.138	0.857	1.800
	PS vs T=1	0.058	0.101	0.667	0.742	1.003
	PS vs P	-0.358	0.037	0.408	-0.056	-0.655

transposase in ISPg3

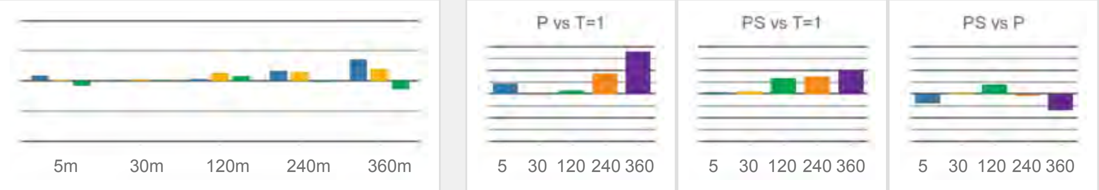

Locus		5m	30m	120m	240m	360m
**PGN_0455**	P vs T=1	-0.581	-0.950	-0.323	-0.285	0.281
	PS vs T=1	-0.886	-0.926	-0.386	-0.492	-0.065
	PS vs P	-0.292	-0.141	0.025	-0.151	-0.123

partial transposase Orf1 in ISPg5

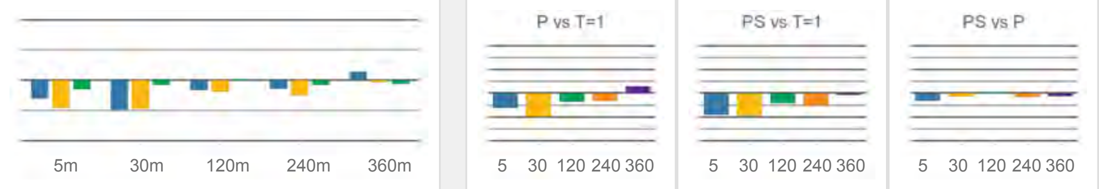

PgSg Time Course

log$_2$ (Fold Change)

Legend: P vs T=1 | PS vs T=1 | PS vs P

Locus		5m	30m	120m	240m	360m
**PGN_0456**	P vs T=1	0.503	1.008	1.475	1.431	1.085
	PS vs T=1	0.913	1.039	1.104	1.343	1.118
	PS vs P	0.372	0.032	-0.343	-0.069	0.030
	methylmalonyl-CoA mutase large subunit					
	energy metabolism					
**PGN_0457**	P vs T=1	0.003	0.243	0.305	0.072	-0.541
	PS vs T=1	-0.053	-0.311	-0.433	-0.098	-0.086
	PS vs P	-0.056	-0.542	-0.724	-0.167	0.436
	methylmalonyl-CoA mutase small subunit					
	energy metabolism					
**PGN_0458**	P vs T=1	0.179	0.567	0.864	0.825	0.642
	PS vs T=1	1.033	1.982	3.543	3.646	3.264
	PS vs P	0.766	1.349	2.627	2.750	2.568
	hypothetical protein					
	hypothetical proteins					
**PGN_0459**	P vs T=1	0.306	-0.280	0.066	0.753	1.625
	PS vs T=1	0.021	0.085	0.461	0.642	0.937
	PS vs P	-0.271	0.236	0.288	-0.059	-0.568
	transposase in ISPg3					
**PGN_0460**	P vs T=1	0.547	0.727	0.609	0.766	1.555
	PS vs T=1	-0.103	0.434	0.937	1.322	1.404
	PS vs P	-0.634	-0.254	0.268	0.503	-0.021
	DNA-binding protein histone-like family					
	DNA metabolism					
**PGN_0461**	P vs T=1	0.854	1.280	1.478	1.267	0.781
	PS vs T=1	1.620	1.751	1.479	0.989	0.663
	PS vs P	0.710	0.472	0.041	-0.256	-0.147
	probable D-alanyl-D-alanine dipeptidase					
	protein fate					
**PGN_0462**	P vs T=1	0.097	0.770	0.435	-0.249	-0.607
	PS vs T=1	0.991	1.013	0.272	-0.377	-0.737
	PS vs P	0.868	0.265	-0.149	-0.161	-0.164
	probable haloacid dehalogenase-like hydrolase					
	hypothetical proteins-Conserved					

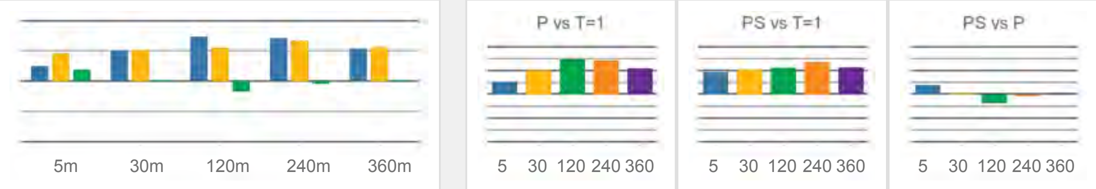

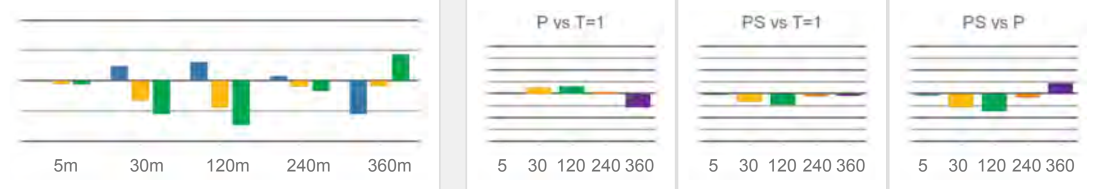

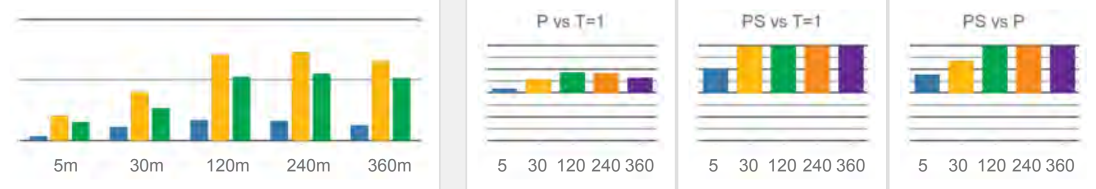

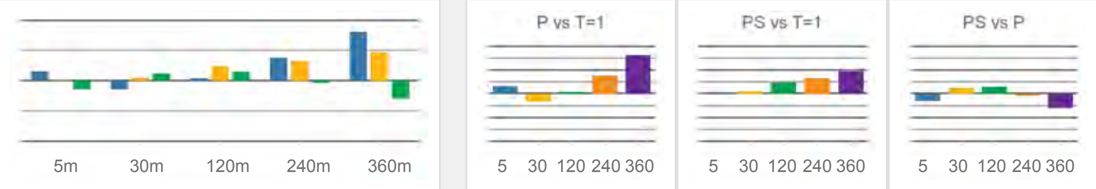

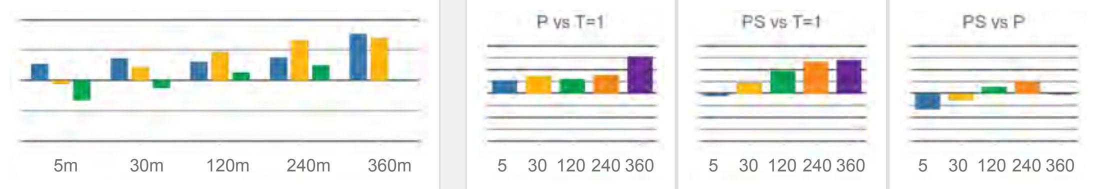

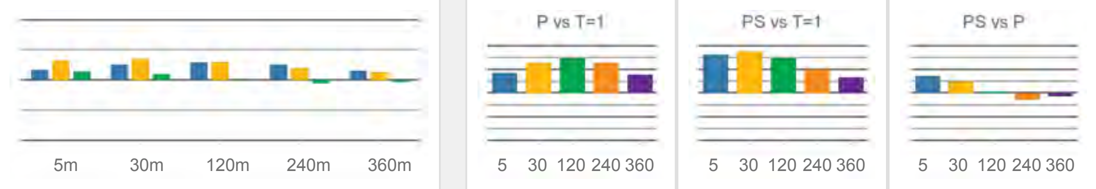

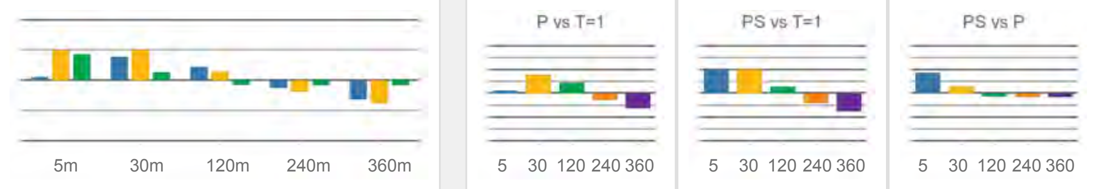

PgSg Time Course

log$_2$ (Fold Change)

Locus		5m	30m	120m	240m	360m
**PGN_0463**	P vs T=1	0.423	0.625	0.295	-0.177	-0.313
	PS vs T=1	1.113	1.029	0.305	-0.078	-0.201
	PS vs P	0.681	0.410	0.009	0.061	0.087
	conserved hypothetical protein					
	hypothetical proteins-Conserved					
**PGN_0464**	P vs T=1	0.273	0.276	-0.062	-0.350	-0.514
	PS vs T=1	0.728	0.407	-0.224	-0.299	-0.176
	PS vs P	0.454	0.133	-0.168	0.027	0.318
	conserved hypothetical protein					
	unknown function					
**PGN_0465**	P vs T=1	-0.299	-0.035	-0.394	-0.652	-0.652
	PS vs T=1	0.199	-0.131	-0.567	-0.701	-0.882
	PS vs P	0.489	-0.086	-0.178	-0.079	-0.244
	GTP pyrophosphokinase					
	cellular processes					
**PGN_0466**	P vs T=1	-1.215	-1.022	-1.345	-1.069	-0.836
	PS vs T=1	-0.887	-0.905	-0.875	-0.849	-0.849
	PS vs P	0.300	0.108	0.404	0.191	-0.017
	putative cardiolipin synthetase					
	fatty acid and phospholipid metabolism					
**PGN_0467**	P vs T=1	-0.643	0.427	1.272	1.732	1.019
	PS vs T=1	-0.619	-0.472	0.101	0.419	0.178
	PS vs P	-0.343	-0.690	-0.731	-0.601	-0.561
	conserved hypothetical protein					
	hypothetical proteins-Conserved					
**PGN_0468** *BioG*	P vs T=1	-0.121	-0.428	-0.718	-0.341	0.337
	PS vs T=1	-0.127	-0.736	-1.357	-1.160	-0.852
	PS vs P	0.044	-0.286	-0.682	-0.714	-1.016
	conserved hypothetical protein with DUF452 domain					
	biosynthesis of cofactors, prosthetic groups, and carriers/ hypothetical [illegible]					
**PGN_0469** *BioC*	P vs T=1	0.019	-0.249	-0.219	0.061	1.105
	PS vs T=1	0.533	0.353	-0.286	-0.138	0.059
	PS vs P	0.502	0.541	-0.136	-0.206	-0.909
	probable biotin synthesis protein					
	biosynthesis of cofactors, prosthetic groups, and carriers					

P vs T=1 | PS vs T=1 | PS vs P

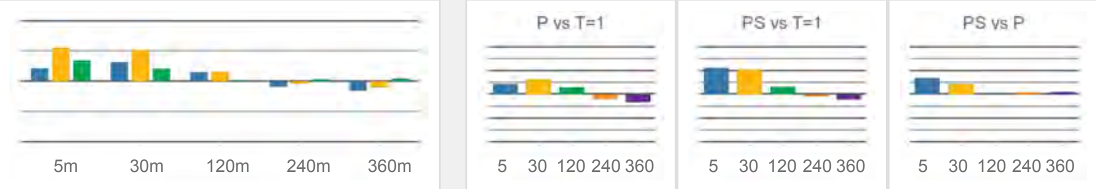

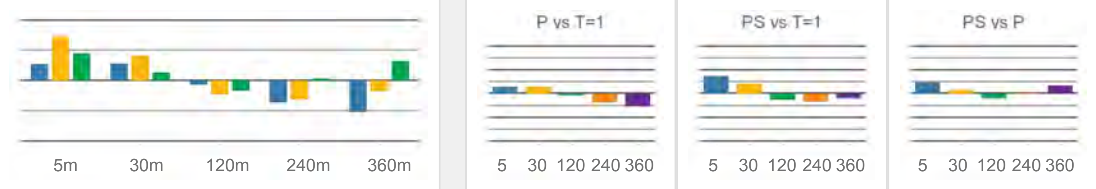

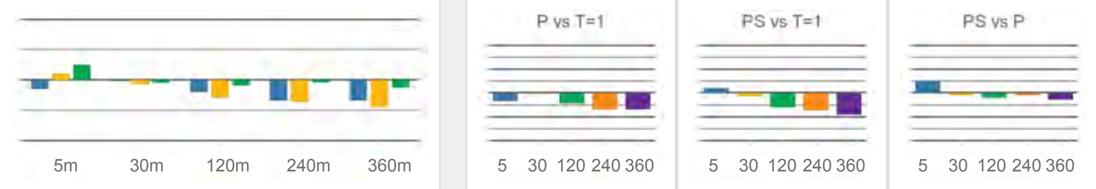

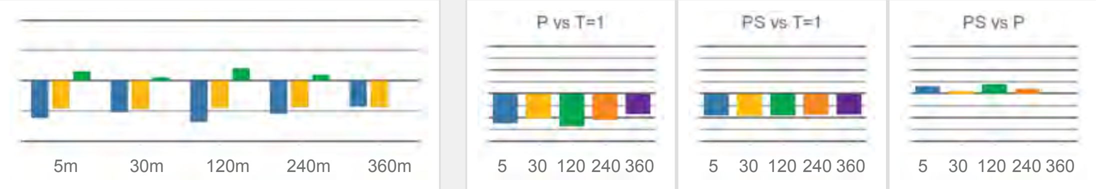

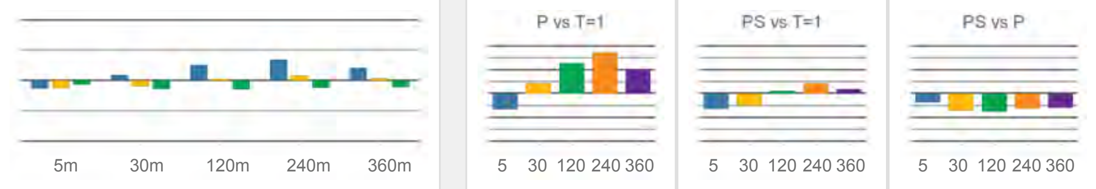

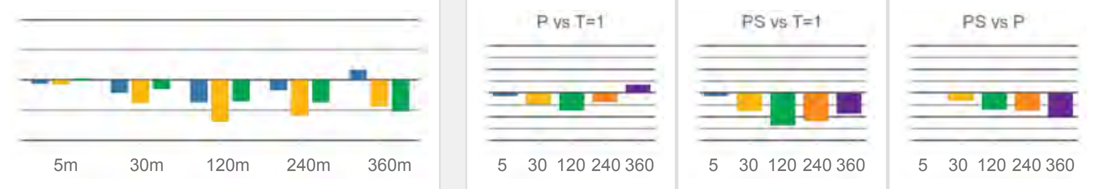

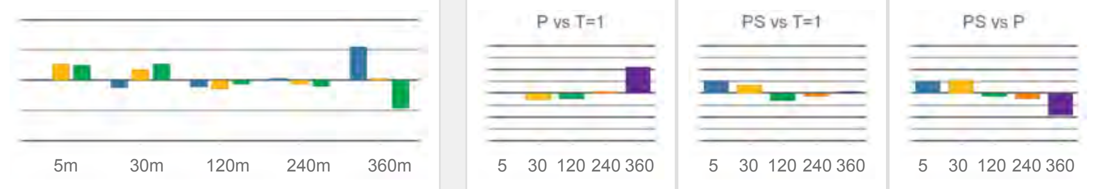

log$_2$ (Fold Change)

Legend: P vs T=1 | PS vs T=1 | PS vs P

Locus		5m	30m	120m	240m	360m
**PGN_0470**	P vs T=1	-0.346	-0.332	-0.848	-0.814	-0.763
	PS vs T=1	0.296	0.305	-0.441	-0.709	-1.084
	PS vs P	0.634	0.634	0.362	0.070	-0.333

conserved hypothetical protein

unknown function

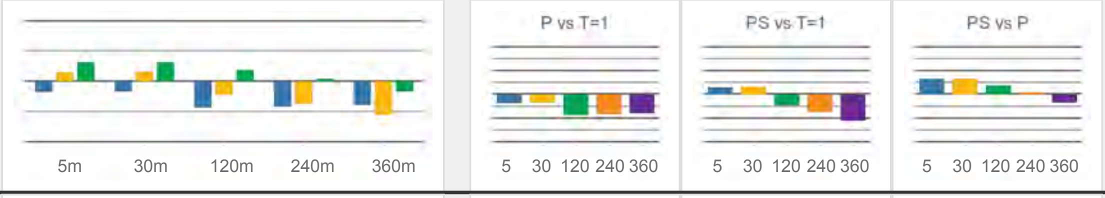

Locus		5m	30m	120m	240m	360m
**PGN_0471**	P vs T=1	0.404	0.565	-0.130	-0.648	-0.661
	PS vs T=1	0.613	0.487	-0.557	-0.993	-1.015
	PS vs P	0.219	-0.053	-0.425	-0.411	-0.382

conserved hypothetical protein

hypothetical proteins-Conserved

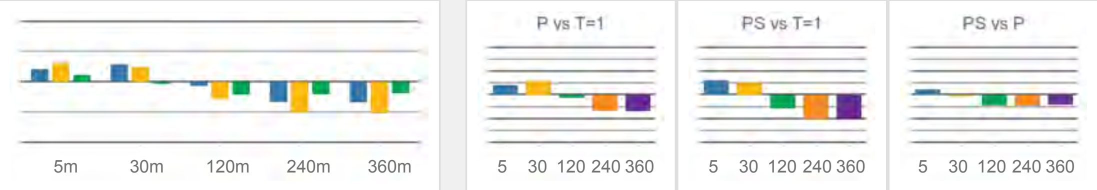

Locus		5m	30m	120m	240m	360m
**PGN_0472**	P vs T=1	-1.001	-0.561	-0.178	0.091	-0.112
	PS vs T=1	-0.759	-0.677	-0.458	-0.130	0.162
	PS vs P	0.176	-0.133	-0.276	-0.199	0.268

DNA topoisomerase IV A subunit

DNA metabolism

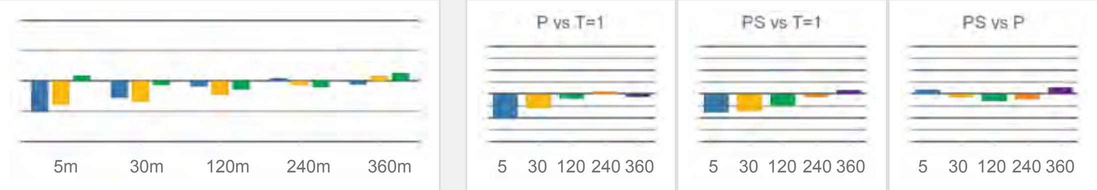

Locus		5m	30m	120m	240m	360m
**PGN_0473**	P vs T=1	0.463	0.290	0.175	0.151	0.006
	PS vs T=1	0.425	0.297	0.467	0.692	0.656
	PS vs P	-0.032	0.001	0.276	0.518	0.627

conserved hypothetical protein

regulatory functions

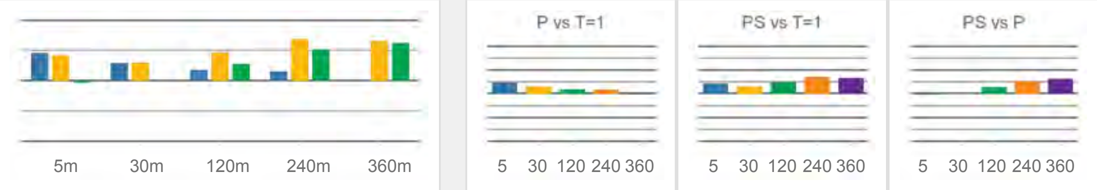

Locus		5m	30m	120m	240m	360m
**PGN_0474**	P vs T=1	-0.415	0.370	0.508	0.596	3.596
	PS vs T=1	-0.215	-0.129	0.730	0.895	1.159
	PS vs P	-0.245	-0.214	0.164	0.121	-0.172

hypothetical protein

hypothetical proteins

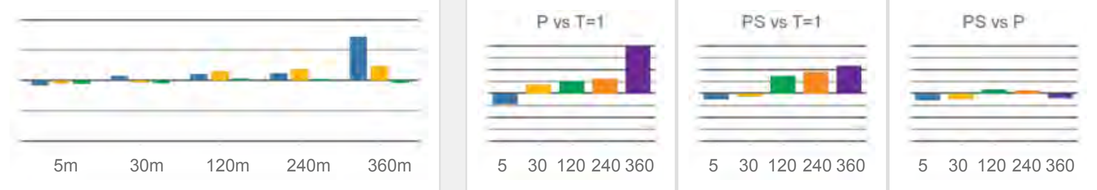

Locus		5m	30m	120m	240m	360m
**PGN_0475**	P vs T=1	0.814	0.402	0.515	2.830	3.588
	PS vs T=1	0.921	1.223	0.968	1.768	2.410
	PS vs P	0.048	0.423	-0.163	-0.249	-0.568

hypothetical protein

hypothetical proteins

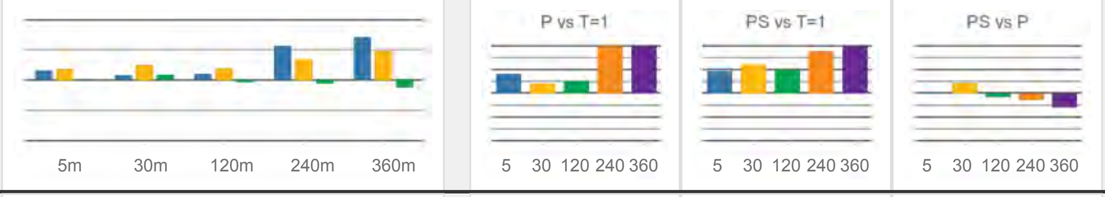

Locus		5m	30m	120m	240m	360m
**PGN_0476**	P vs T=1	0.572	0.786	0.883	1.008	0.539
	PS vs T=1	0.522	1.122	1.423	1.538	1.359
	PS vs P	-0.074	0.322	0.528	0.527	0.793

conserved hypothetical protein

hypothetical proteins-Conserved

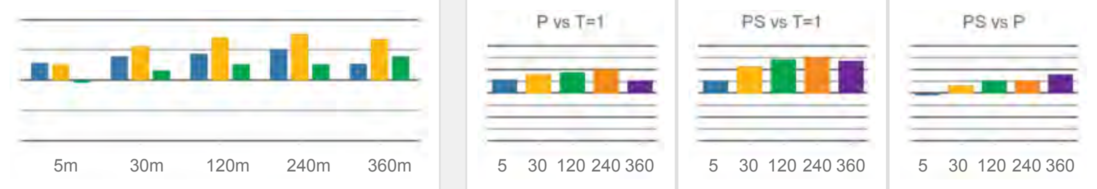

PgSg Time Course

log$_2$ (Fold Change)

Legend: P vs T=1 | PS vs T=1 | PS vs P

Locus		5m	30m	120m	240m	360m
**PGN_0477**	P vs T=1	0.766	1.027	1.268	1.383	1.156
	PS vs T=1	0.743	1.222	1.637	1.839	1.723
	PS vs P	-0.040	0.190	0.369	0.459	0.561
	conserved hypothetical protein					
	hypothetical proteins-Conserved					
**PGN_0478**	P vs T=1	0.446	0.499	0.683	0.801	1.287
	PS vs T=1	-0.153	-0.171	-0.075	-0.276	-0.049
	PS vs P	-0.526	-0.573	-0.607	-0.830	-1.067
	partial transposase in ISPg4					
**PGN_0479**	P vs T=1	0.351	-0.235	0.098	0.250	1.707
	PS vs T=1	-0.430	-0.339	-0.340	0.034	-0.579
	PS vs P	-0.521	-0.222	-0.347	-0.126	-1.103
	hypothetical protein					
	hypothetical proteins					
**PGN_0480**	P vs T=1	2.327	2.197	2.372	2.633	2.868
	PS vs T=1	-0.477	-0.262	0.749	0.864	1.496
	PS vs P	-2.302	-1.970	-1.281	-1.261	-0.998
	partial transposase in ISPg4					
**PGN_0481**	P vs T=1	1.621	1.339	1.574	1.548	1.166
	PS vs T=1	-1.232	-0.887	-0.078	0.268	0.607
	PS vs P	-2.615	-2.050	-1.506	-1.144	-0.503
	hypothetical protein					
	hypothetical proteins					
**PGN_0482**	P vs T=1	0.408	0.358	0.849	0.920	0.645
	PS vs T=1	-2.134	-1.975	-1.234	-0.712	-0.233
	PS vs P	-2.454	-2.244	-1.992	-1.531	-0.833
	probable immunoreactive 23 kDa antigen					
	cell envelope					
**PGN_0483**	P vs T=1	-0.135	0.083	0.513	0.616	0.347
	PS vs T=1	0.064	-0.066	-0.006	0.214	0.133
	PS vs P	0.181	-0.152	-0.490	-0.359	-0.207
	putative aldose 1-epimerase					
	energy metabolism					

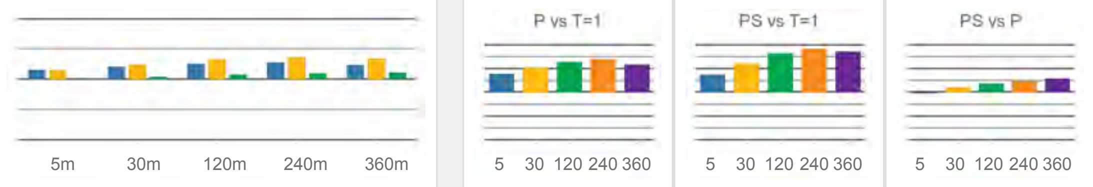

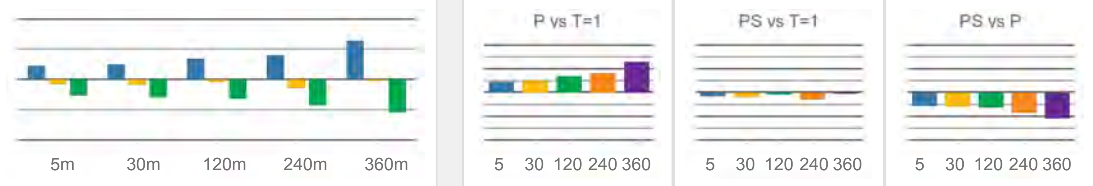

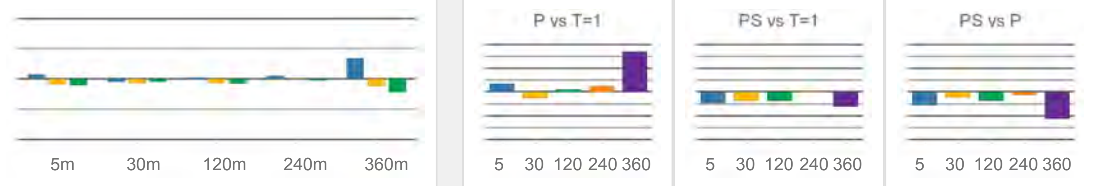

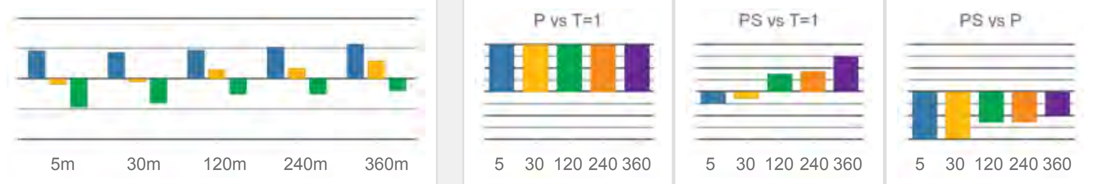

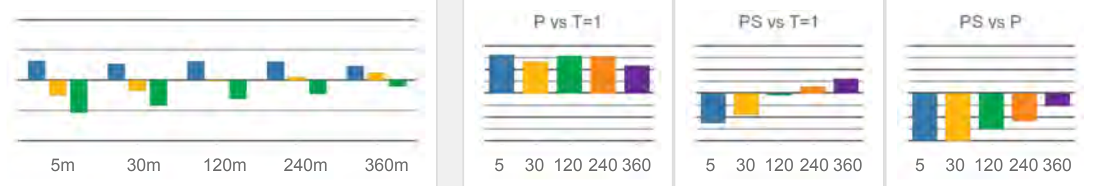

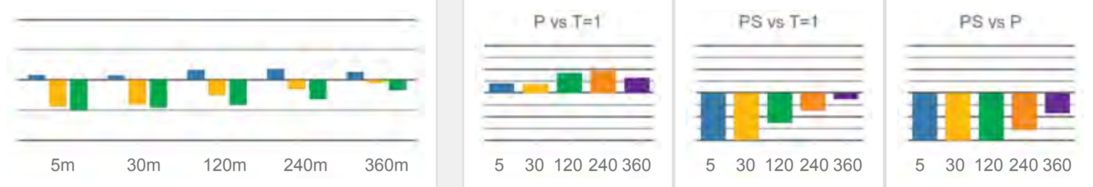

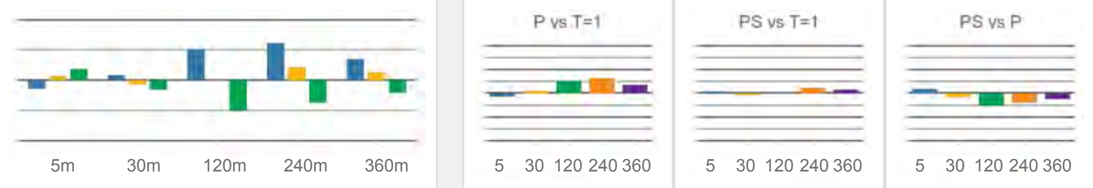

PgSg Time Course

log2 (Fold Change)

Legend: P vs T=1 | PS vs T=1 | PS vs P

Locus		5m	30m	120m	240m	360m
**PGN_0484**	P vs T=1	-0.169	0.399	0.514	0.176	-0.291
	PS vs T=1	0.570	0.746	0.202	-0.317	-0.393
	PS vs P	0.707	0.356	-0.287	-0.486	-0.119

putative galactokinase

energy metabolism

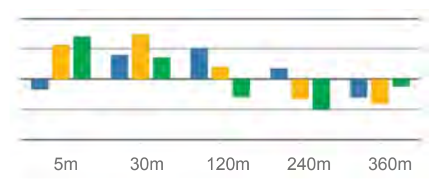

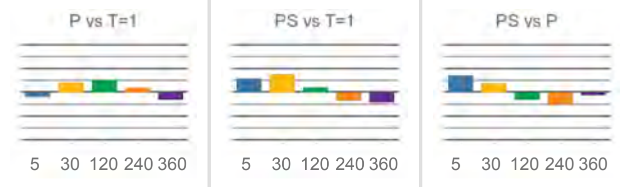

Locus		5m	30m	120m	240m	360m
**PGN_0485**	P vs T=1	0.263	0.580	0.986	1.291	1.116
	PS vs T=1	0.327	1.054	1.279	1.515	1.574
	PS vs P	0.004	0.444	0.302	0.262	0.467

conserved hypothetical protein

hypothetical proteins-Conserved

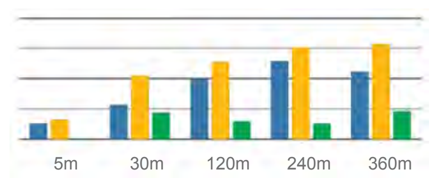

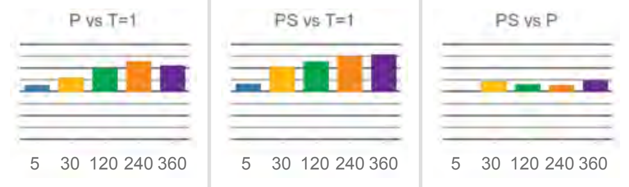

Locus		5m	30m	120m	240m	360m
**PGN_0486**	P vs T=1	2.121	2.644	2.899	3.161	3.250
	PS vs T=1	2.020	2.903	3.242	3.458	3.522
	PS vs P	-0.154	0.278	0.368	0.350	0.320

conserved hypothetical protein

hypothetical proteins-Conserved

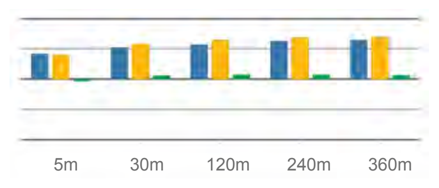

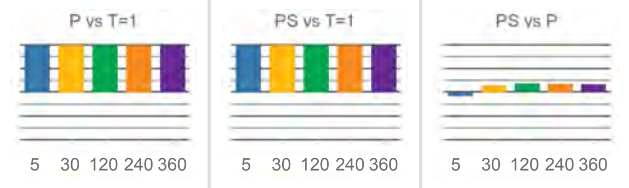

Locus		5m	30m	120m	240m	360m
**PGN_0487**	P vs T=1	0.612	0.777	0.620	0.646	0.624
	PS vs T=1	0.552	0.639	0.475	0.772	0.810
	PS vs P	-0.055	-0.110	-0.140	0.132	0.189

FtsK/SpoIIIE family cell division protein

unknown function

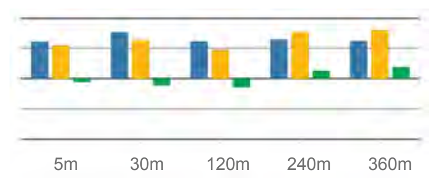

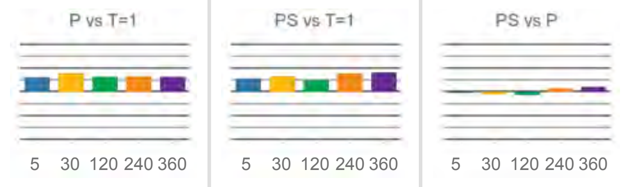

Locus		5m	30m	120m	240m	360m
**PGN_0488**	P vs T=1	0.930	1.421	2.073	2.205	2.184
	PS vs T=1	1.264	1.365	1.618	1.717	1.737
	PS vs P	0.272	-0.050	-0.381	-0.405	-0.391

conserved hypothetical protein

energy metabolism

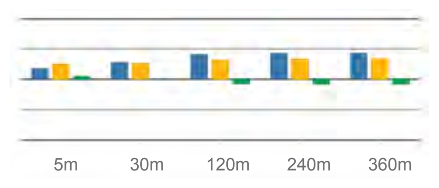

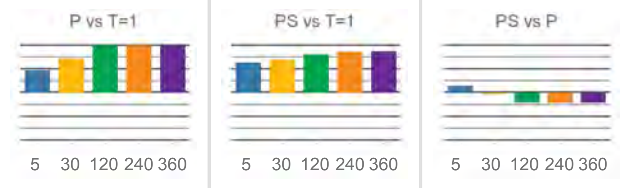

Locus		5m	30m	120m	240m	360m
**PGN_0489**	P vs T=1	-0.610	-0.392	0.756	1.428	1.265
	PS vs T=1	0.033	0.470	0.656	0.800	0.967
	PS vs P	0.269	0.495	0.049	-0.148	-0.050

conserved hypothetical protein

hypothetical proteins-Conserved

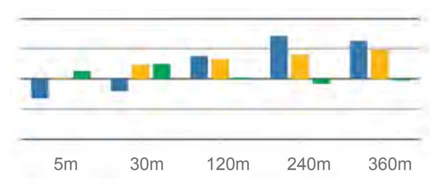

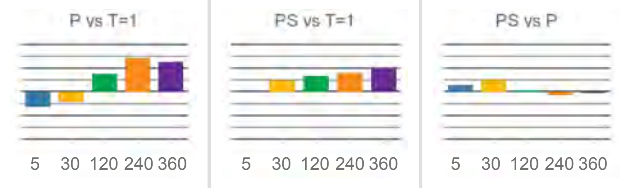

Locus		5m	30m	120m	240m	360m
**PGN_0490**	P vs T=1	-0.678	-0.103	0.610	1.016	1.122
	PS vs T=1	-0.602	-0.375	-0.005	0.537	0.785
	PS vs P	-0.021	-0.293	-0.574	-0.395	-0.286

putative DNA-damage-inducible protein F

transport and binding proteins

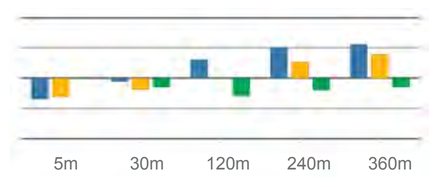

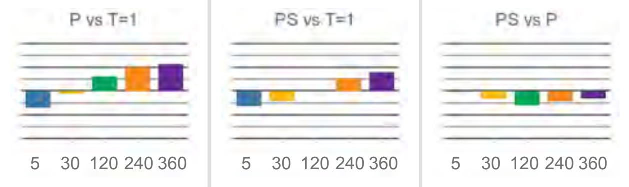

PgSg Time Course

log$_2$ (Fold Change)

P vs T=1 | PS vs T=1 | PS vs P

Locus		5m	30m	120m	240m	360m
**PGN_0491**	P vs T=1	-0.624	-0.025	0.528	0.667	0.786
ltp1	PS vs T=1	-0.577	-0.238	0.045	0.052	0.215
	PS vs P	-0.076	-0.211	-0.398	-0.501	-0.484

probable phosphotyrosine protein phosphatase

regulatory functions

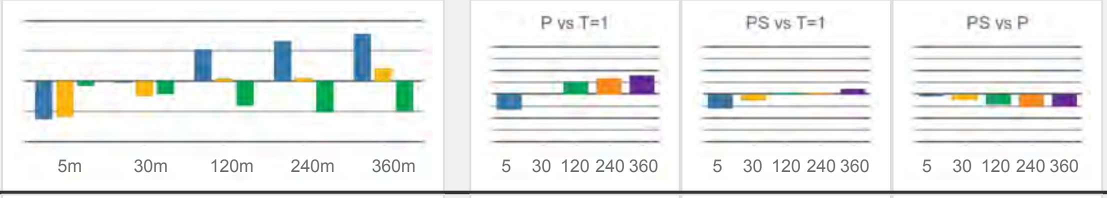

Locus		5m	30m	120m	240m	360m
**PGN_0492**	P vs T=1	0.320	1.011	1.409	1.380	0.829
	PS vs T=1	-0.139	0.391	0.992	1.167	1.070
	PS vs P	-0.482	-0.596	-0.387	-0.188	0.233

cation-transporting ATPase

transport and binding proteins

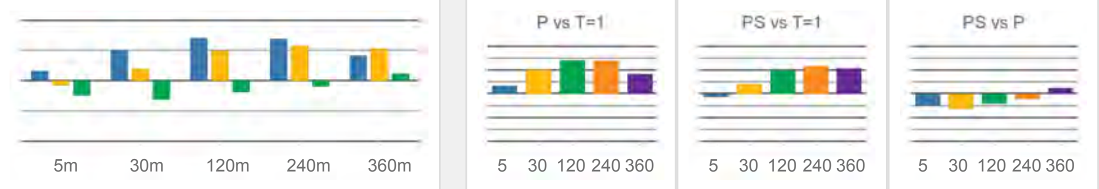

Locus		5m	30m	120m	240m	360m
**PGN_0493**	P vs T=1	0.962	1.621	1.983	1.963	1.272
	PS vs T=1	-0.118	0.855	1.635	1.679	1.586
	PS vs P	-1.076	-0.696	-0.277	-0.220	0.310

hypothetical protein

hypothetical proteins

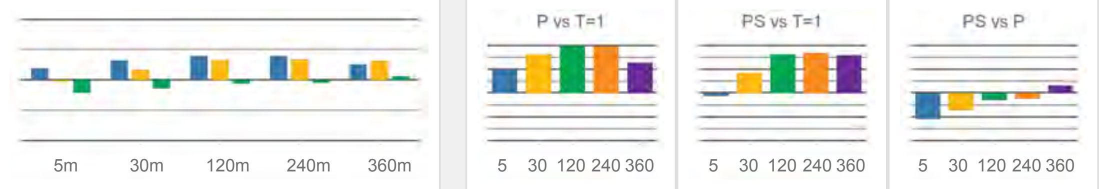

Locus		5m	30m	120m	240m	360m
**PGN_0494**	P vs T=1	0.049	0.274	0.544	1.147	3.893
	PS vs T=1	0.017	0.174	1.011	1.311	1.190
	PS vs P	-0.204	-0.122	0.255	0.260	-0.338

hypothetical protein

hypothetical proteins

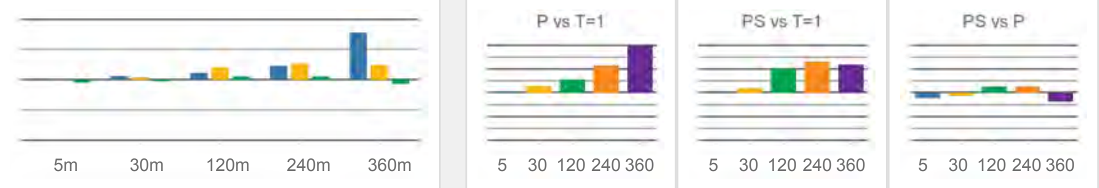

Locus		5m	30m	120m	240m	360m
**PGN_0495**	P vs T=1	0.008	0.000	-0.585	0.609	0.954
	PS vs T=1	0.255	0.592	0.937	1.206	1.107
	PS vs P	0.171	0.492	0.961	0.707	0.290

conserved hypothetical protein

hypothetical proteins-Conserved

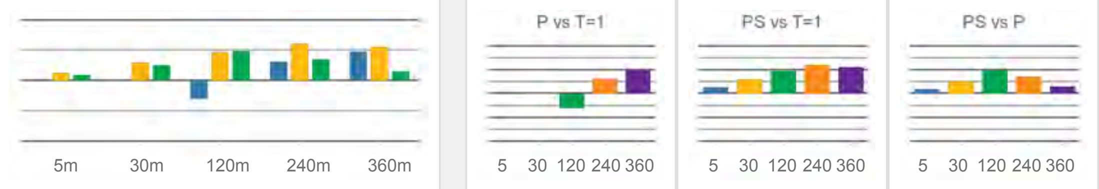

Locus		5m	30m	120m	240m	360m
**PGN_0496**	P vs T=1	-0.352	-0.095	0.020	-0.300	-0.622
	PS vs T=1	-0.766	-0.351	0.074	0.281	0.391
	PS vs P	-0.422	-0.244	0.071	0.556	0.975

putative cytochrome B subunit

energy metabolism

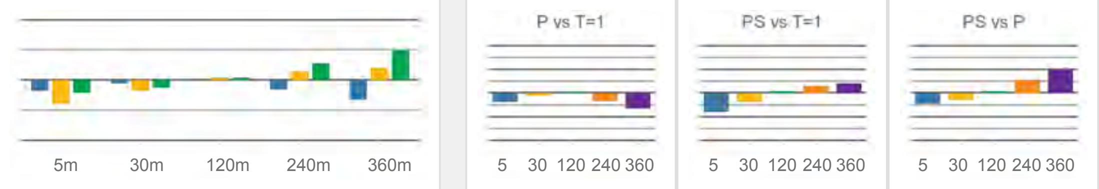

Locus		5m	30m	120m	240m	360m
**PGN_0497**	P vs T=1	1.043	1.237	1.297	0.926	0.338
	PS vs T=1	0.553	0.401	0.147	0.020	-0.072
	PS vs P	-0.452	-0.774	-1.086	-0.873	-0.420

fumarate reductase flavoprotein subunit

energy metabolism

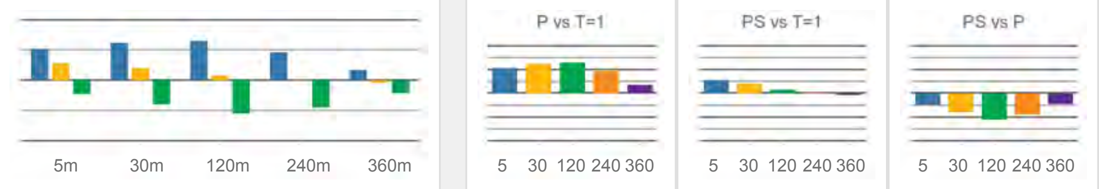

log$_2$ (Fold Change)

P vs T=1 | PS vs T=1 | PS vs P

Locus		5m	30m	120m	240m	360m
**PGN_0498**	P vs T=1	1.528	2.156	2.800	2.855	2.313
	PS vs T=1	0.717	1.113	1.200	1.192	1.271
	PS vs P	-0.810	-1.010	-1.549	-1.613	-1.025
	fumarate reductase iron-sulfur protein					
	energy metabolism					
**PGN_0499**	P vs T=1	-1.490	-1.515	-1.931	-2.314	-3.012
	PS vs T=1	-1.359	-1.012	-0.646	-0.616	-1.076
	PS vs P	0.108	0.478	1.211	1.554	1.742
	conserved hypothetical protein					
	unknown function					
**PGN_0500**	P vs T=1	0.624	0.945	0.528	-0.179	-0.824
	PS vs T=1	0.908	0.993	0.713	0.570	0.175
	PS vs P	0.285	0.084	0.197	0.705	0.941
	methylmalonyl-CoA decarboxylase alpha subunit					
	energy metabolism					
**PGN_0501**	P vs T=1	0.347	0.305	0.229	-0.134	-0.664
	PS vs T=1	0.545	0.450	0.128	-0.107	-0.149
	PS vs P	0.211	0.156	-0.082	0.008	0.460
	conserved hypothetical protein					
	hypothetical proteins-Conserved					
**PGN_0502**	P vs T=1	1.278	1.686	1.950	1.475	0.653
	PS vs T=1	1.995	2.391	2.372	1.833	1.567
	PS vs P	0.640	0.699	0.486	0.335	0.785
	conserved hypothetical protein					
	hypothetical proteins-Conserved					
**PGN_0503**	P vs T=1	0.874	1.403	1.412	0.825	0.120
	PS vs T=1	1.337	1.685	1.683	1.292	0.846
	PS vs P	0.426	0.305	0.302	0.431	0.648
	putative biotin carboxyl carrier protein					
	energy metabolism					
**PGN_0504**	P vs T=1	-0.191	0.146	0.463	0.209	-0.434
	PS vs T=1	0.240	0.468	0.532	0.523	0.366
	PS vs P	0.357	0.307	0.109	0.309	0.728
	methylmalonyl-CoA decarboxylase beta subunit					
	energy metabolism					

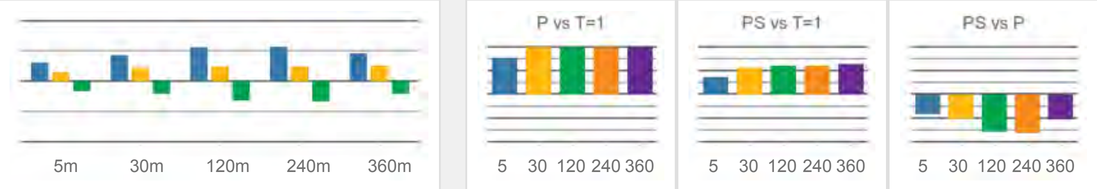

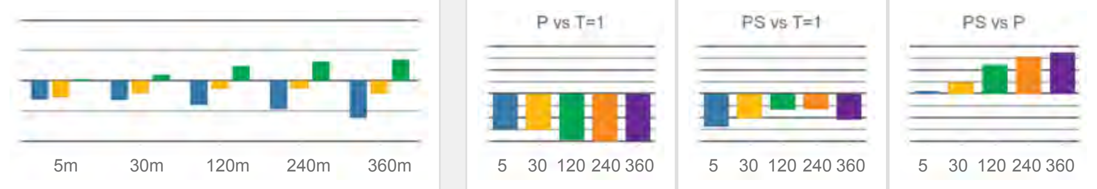

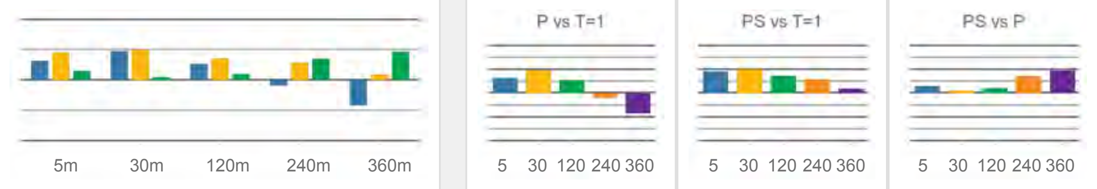

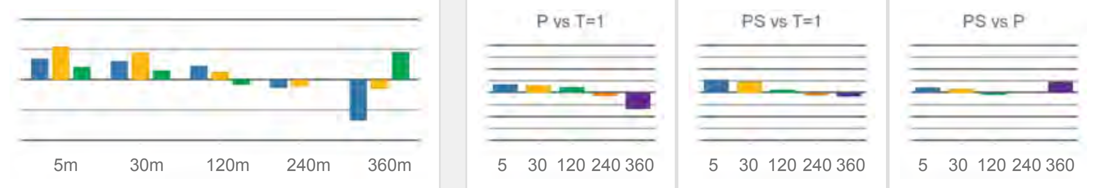

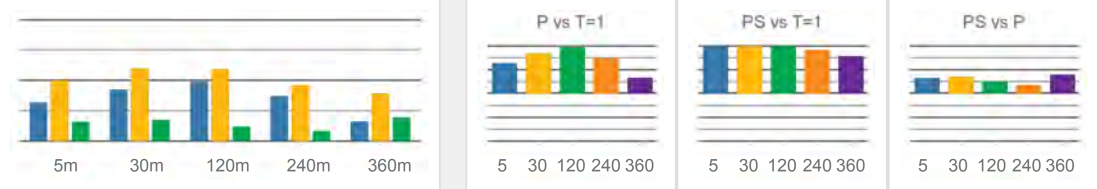

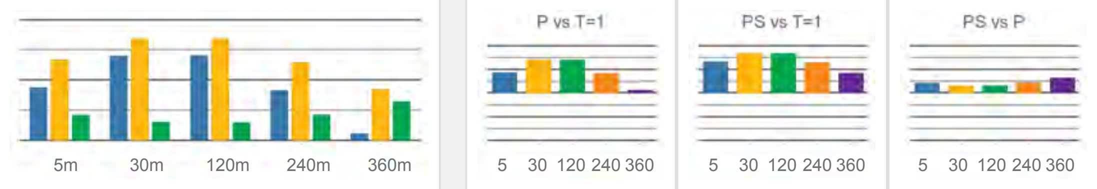

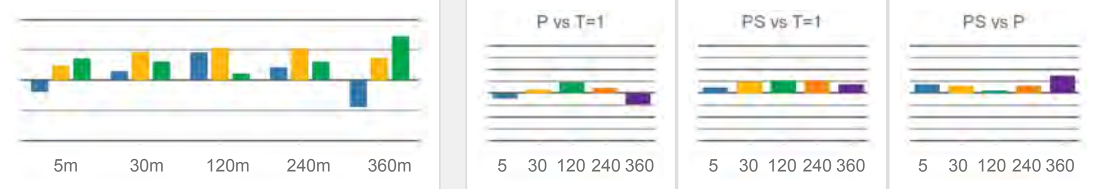

log$_2$ (Fold Change)

Locus		5m	30m	120m	240m	360m
**PGN_0505**	P vs T=1	-1.272	-0.806	-0.339	-0.331	-0.440
	PS vs T=1	-0.737	-0.560	-0.258	-0.487	-0.343
	PS vs P	0.371	0.188	0.137	-0.103	0.086
	hypothetical protein					
	hypothetical proteins					

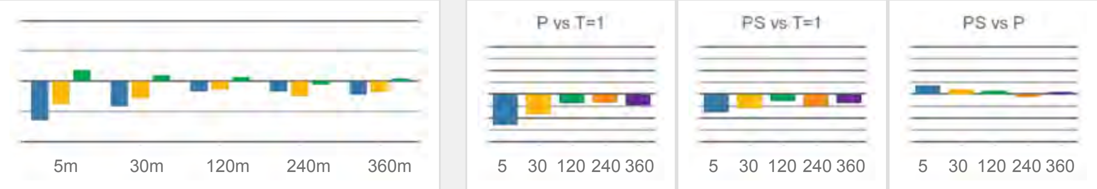

Locus		5m	30m	120m	240m	360m
**PGN_0506**	P vs T=1	-0.513	-0.680	-0.024	0.472	0.026
	PS vs T=1	-1.066	-1.048	-0.385	0.001	-0.120
	PS vs P	-0.499	-0.427	-0.156	0.012	-0.010
	hypothetical protein					
	hypothetical proteins					

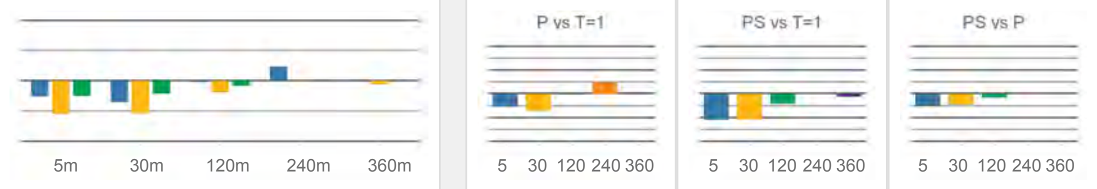

Locus		5m	30m	120m	240m	360m
**PGN_0507**	P vs T=1	-0.301	-1.064	-0.663	-0.200	0.420
	PS vs T=1	-0.933	-0.549	0.599	0.878	0.893
	PS vs P	-0.571	0.287	1.112	1.043	0.564
	putative ion transporter					
	transport and binding proteins					

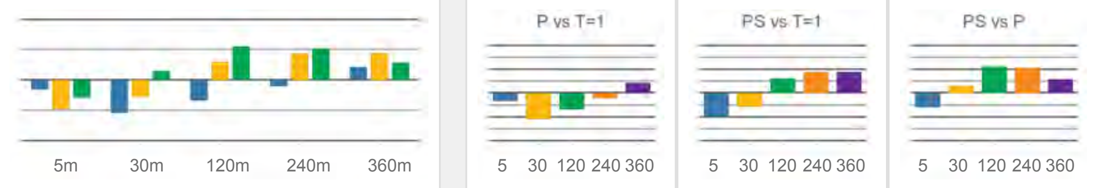

Locus		5m	30m	120m	240m	360m
**PGN_0508**	P vs T=1	1.473	1.611	1.493	1.184	0.647
	PS vs T=1	2.138	1.908	1.211	0.654	0.467
	PS vs P	0.679	0.335	-0.236	-0.504	-0.195
	putative aminopeptidase					
	protein fate					

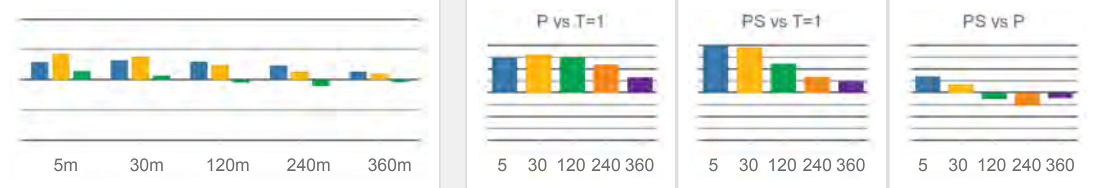

Locus		5m	30m	120m	240m	360m
**PGN_0509**	P vs T=1	-0.221	-0.423	-1.202	-1.558	-1.297
	PS vs T=1	-0.307	-0.769	-1.350	-1.505	-1.551
	PS vs P	-0.067	-0.327	-0.159	0.010	-0.260
	immunoreactive 84 kDa antigen					
	cell envelope					

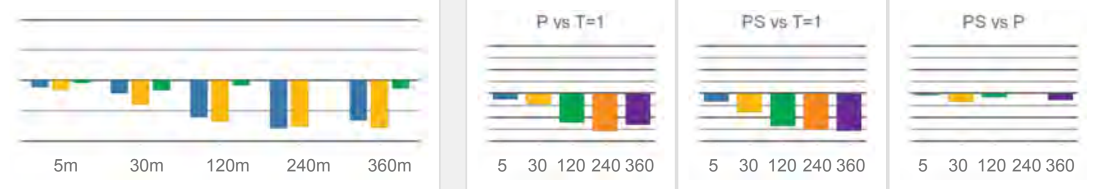

Locus		5m	30m	120m	240m	360m
**PGN_0510**	P vs T=1	0.135	-0.866	-1.986	-1.890	-1.388
	PS vs T=1	-0.559	-0.635	-0.609	-0.751	-1.246
	PS vs P	-0.584	0.226	1.162	0.944	0.115
	probable xanthosine triphosphate pyrophosphatase					
	unknown function					

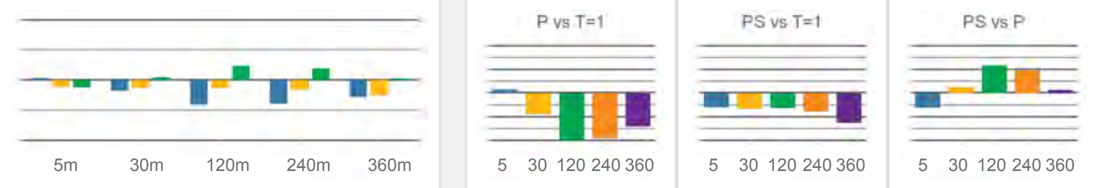

Locus		5m	30m	120m	240m	360m
**PGN_0511**	P vs T=1	0.092	-0.701	-1.037	-1.220	-1.462
	PS vs T=1	-0.227	-0.369	-0.317	-0.647	-0.743
	PS vs P	-0.218	0.287	0.640	0.449	0.592
	conserved hypothetical protein					
	unknown function					

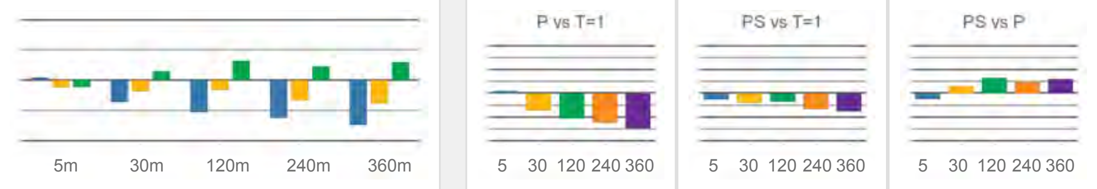

log$_2$ (Fold Change)

Legend: P vs T=1 | PS vs T=1 | PS vs P

Locus		5m	30m	120m	240m	360m
**PGN_0512**	P vs T=1	0.298	-0.326	-0.684	-0.629	-0.587
	PS vs T=1	0.373	-0.141	-0.106	-0.315	-0.519
	PS vs P	0.110	0.172	0.524	0.272	0.050
	probable biotin--acetyl-CoA-carboxylase ligase					
	biosynthesis of cofactors, prosthetic groups, and carriers					
**PGN_0513**	P vs T=1	-0.383	-1.121	-1.734	-1.519	-0.899
	PS vs T=1	-0.027	-0.463	-0.723	-1.171	-1.330
	PS vs P	0.387	0.626	0.886	0.269	-0.405
	conserved hypothetical protein					
	hypothetical proteins-Conserved					
**PGN_0514**	P vs T=1	-0.714	-1.355	-1.998	-2.251	-2.122
	PS vs T=1	-0.249	-0.639	-1.650	-2.133	-2.204
	PS vs P	0.493	0.699	0.286	0.003	-0.130
	conserved hypothetical protein					
	hypothetical proteins-Conserved					
**PGN_0515**	P vs T=1	-0.804	-1.443	-2.055	-2.218	-2.371
	PS vs T=1	-0.509	-0.933	-1.856	-2.728	-2.614
	PS vs P	0.323	0.498	0.148	-0.577	-0.306
	probable lipoprotein signal peptidase					
	protein fate					
**PGN_0516**	P vs T=1	-0.478	-0.048	-0.114	-0.765	-1.839
	PS vs T=1	0.074	0.373	0.071	-0.601	-1.212
	PS vs P	0.529	0.431	0.204	0.138	0.531
	putative DnaK suppressor protein					
	DNA metabolism					
**PGN_0517**	P vs T=1	0.490	0.390	-0.356	-0.712	-1.035
	PS vs T=1	0.914	0.208	-0.718	-0.955	-0.924
	PS vs P	0.443	-0.159	-0.366	-0.262	0.088
	isoleucyl-tRNA synthetase					
	protein synthesis					
**PGN_0518**	P vs T=1	0.125	0.140	0.112	0.038	-0.121
	PS vs T=1	0.293	0.180	0.144	0.424	0.333
	PS vs P	0.153	0.029	0.014	0.344	0.404
	putative ribulose-phosphate 3-epimerase					
	energy metabolism					

PGN_0512

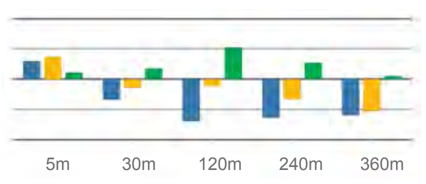

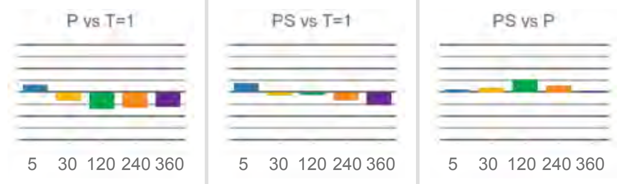

PGN_0513

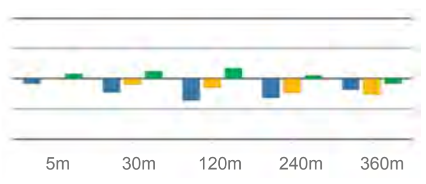

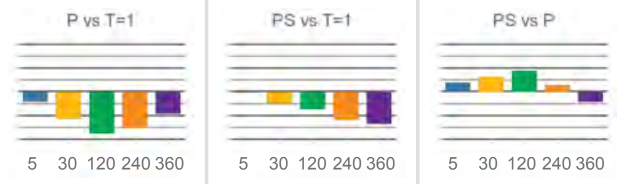

PGN_0514

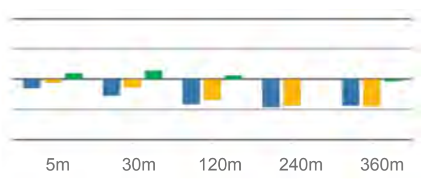

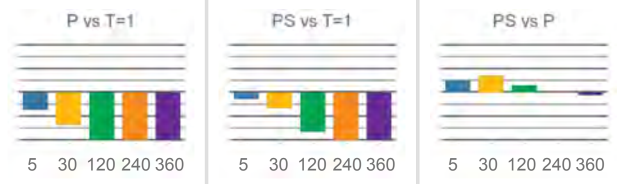

PGN_0515

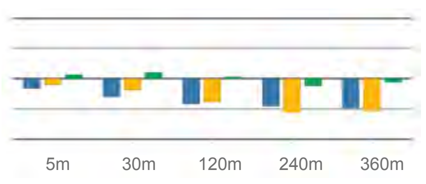

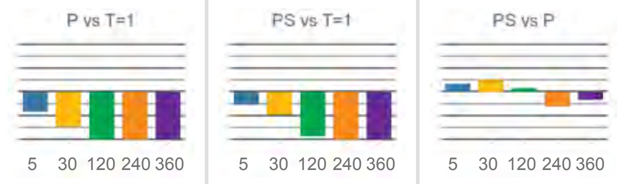

PGN_0516

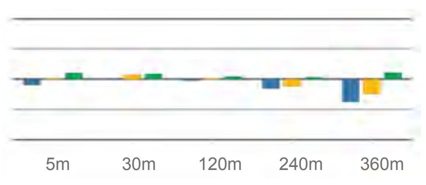

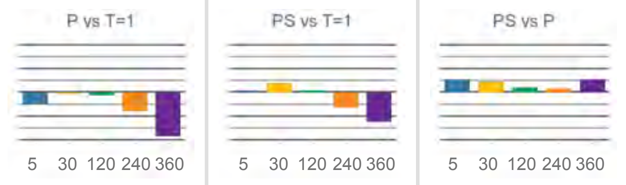

PGN_0517

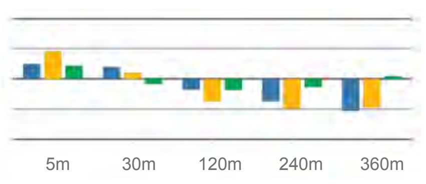

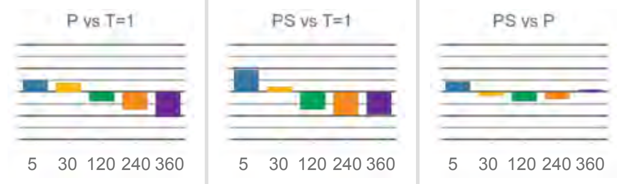

PGN_0518

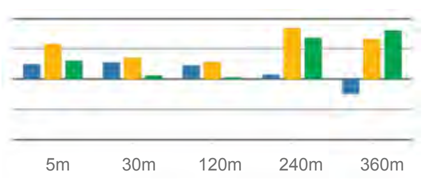

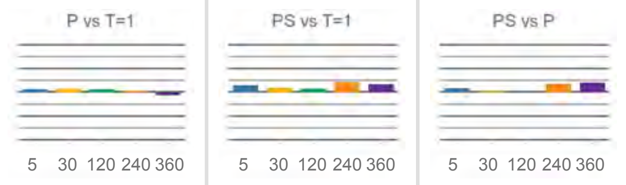

log2 (Fold Change)

Legend: P vs T=1 | PS vs T=1 | PS vs P

Locus		5m	30m	120m	240m	360m
**PGN_0519**	P vs T=1	-0.216	-0.703	-0.443	-0.127	0.785
	PS vs T=1	0.379	-0.181	-0.531	-0.403	-0.074
	PS vs P	0.587	0.431	-0.144	-0.267	-0.749
	probable competence protein					
	cellular processes					
**PGN_0520**	P vs T=1	-0.845	-1.174	-1.705	-1.702	-1.211
	PS vs T=1	-0.846	-1.370	-1.693	-2.222	-1.695
	PS vs P	0.042	-0.180	-0.097	-0.615	-0.432
	probable shikimate kinase					
	central intermediary metabolism					
**PGN_0521**	P vs T=1	-0.229	-0.641	-1.346	-1.344	-0.912
	PS vs T=1	-0.514	-0.955	-1.056	-1.251	-1.451
	PS vs P	-0.256	-0.300	0.253	0.057	-0.526
	conserved hypothetical protein					
	hypothetical proteins-Conserved					
**PGN_0522**	P vs T=1	0.934	1.027	0.983	1.567	1.662
	PS vs T=1	0.676	0.361	0.436	0.902	0.993
	PS vs P	-0.230	-0.611	-0.532	-0.521	-0.574
	putative dihydropteroate synthase					
	biosynthesis of cofactors, prosthetic groups, and carriers					
**PGN_0523**	P vs T=1	-0.481	-0.497	-0.372	-0.247	-0.336
	PS vs T=1	-0.072	-0.449	-0.708	-0.307	-0.418
	PS vs P	0.392	0.032	-0.329	-0.026	-0.078
	conserved hypothetical protein					
	hypothetical proteins-Conserved					
**PGN_0524**	P vs T=1	-0.780	-1.742	-1.894	-1.319	-0.995
	PS vs T=1	-0.289	-1.013	-1.579	-1.655	-1.404
	PS vs P	0.513	0.646	0.179	-0.329	-0.386
	lipid A 4'-phosphatase					
	unknown function					
**PGN_0525** *batE*	P vs T=1	-0.589	-1.202	-1.759	-1.601	-1.272
	PS vs T=1	-0.102	-0.701	-1.652	-2.131	-2.162
	PS vs P	0.512	0.486	0.037	-0.552	-0.867
	probable aerotolerance-related exported protein BatE					
	cellular processes					

PGN_0519
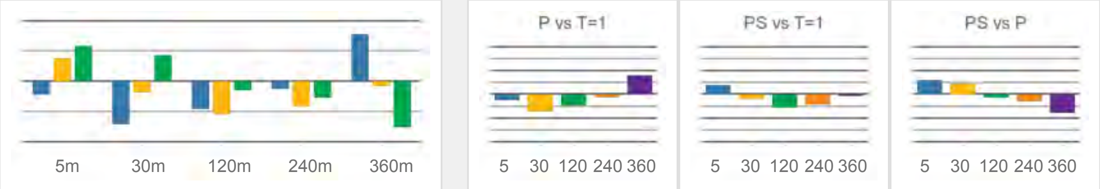

PGN_0520
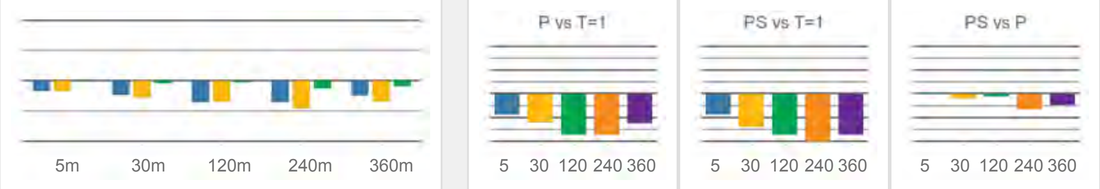

PGN_0521
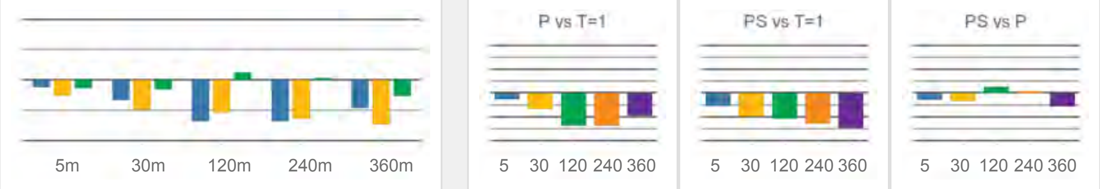

PGN_0522
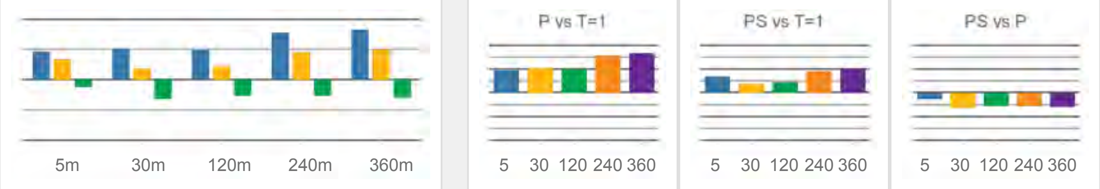

PGN_0523
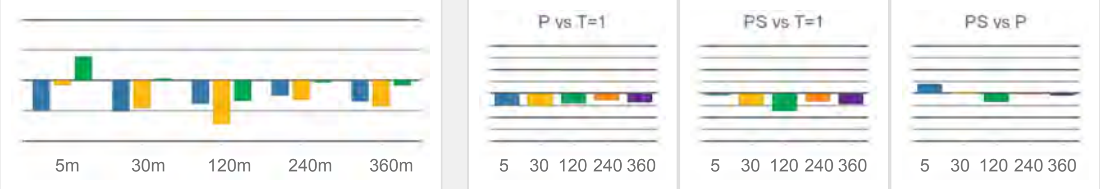

PGN_0524
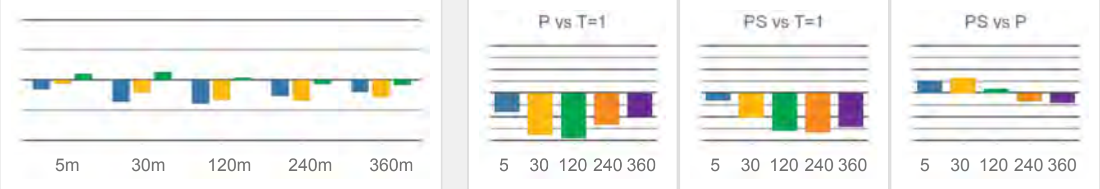

PGN_0525
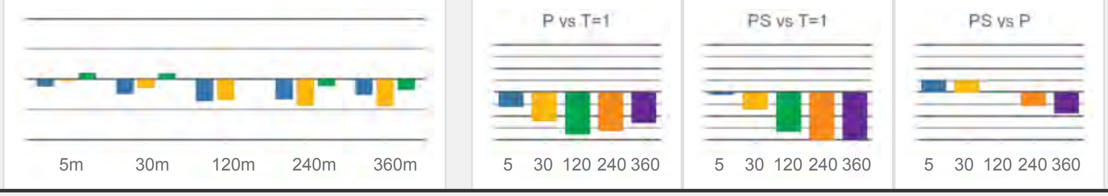

PgSg Time Course

log$_2$ (Fold Change)

Legend: P vs T=1 | PS vs T=1 | PS vs P

PGN_0526 — *batD*

Locus	5m	30m	120m	240m	360m
P vs T=1	0.009	-0.564	-1.200	-1.013	-0.484
PS vs T=1	0.297	-0.236	-1.151	-1.446	-1.149
PS vs P	0.331	0.325	-0.002	-0.446	-0.636

aerotolerance-related exported protein BatD

cellular processes

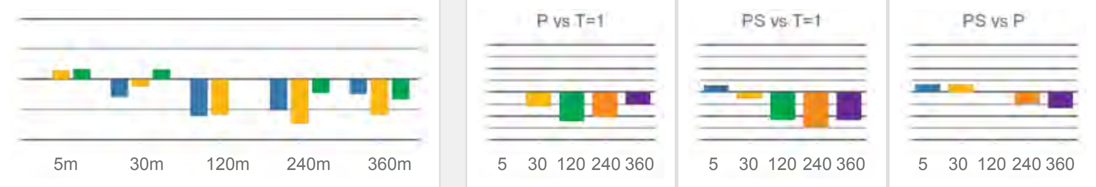

PGN_0527 — *batC*

Locus	5m	30m	120m	240m	360m
P vs T=1	0.486	0.081	-0.340	-0.043	-0.084
PS vs T=1	1.076	0.785	0.136	-0.505	-0.536
PS vs P	0.605	0.694	0.442	-0.445	-0.444

probable aerotolerance-related exported protein BatC

cellular processes

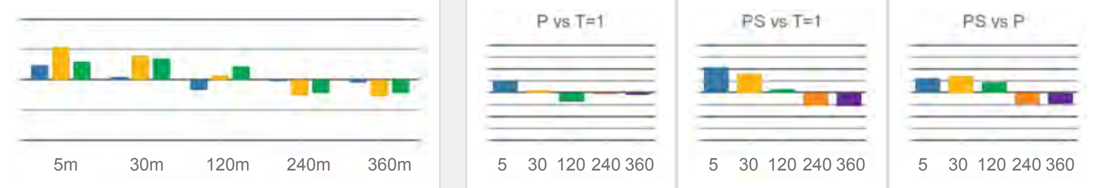

PGN_0528 — *batB*

Locus	5m	30m	120m	240m	360m
P vs T=1	0.158	-0.264	-0.957	-0.975	-0.904
PS vs T=1	0.128	-0.115	-0.908	-1.280	-1.063
PS vs P	0.021	0.167	0.015	-0.325	-0.165

putative aerotolerance-related exported protein BatB

cellular processes

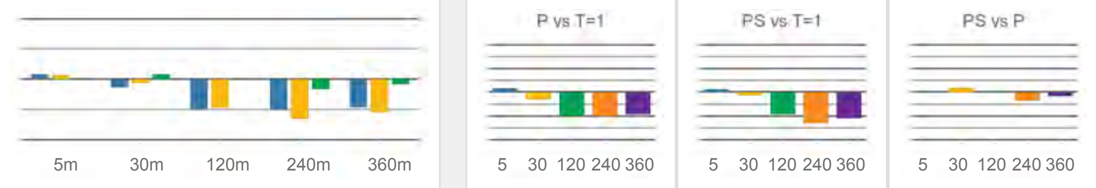

PGN_0529 — *batA*

Locus	5m	30m	120m	240m	360m
P vs T=1	0.225	-0.082	-0.504	-0.326	-0.392
PS vs T=1	0.105	-0.209	-0.642	-0.947	-0.610
PS vs P	-0.085	-0.113	-0.155	-0.606	-0.217

aerotolerance-related membrane protein BatA

cellular processes

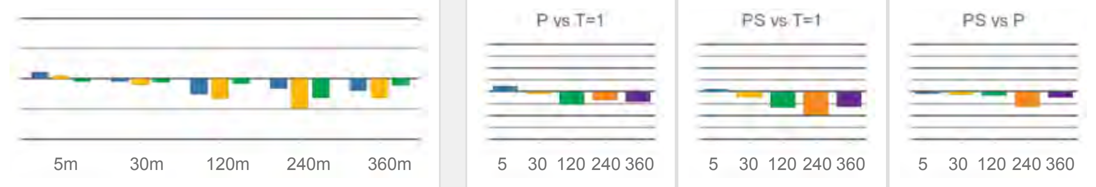

PGN_0530

Locus	5m	30m	120m	240m	360m
P vs T=1	0.377	0.112	-0.408	-0.284	-0.147
PS vs T=1	0.288	0.030	-0.338	-0.673	-0.183
PS vs P	-0.063	-0.069	0.048	-0.389	-0.031

conserved hypothetical protein

hypothetical proteins-Conserved

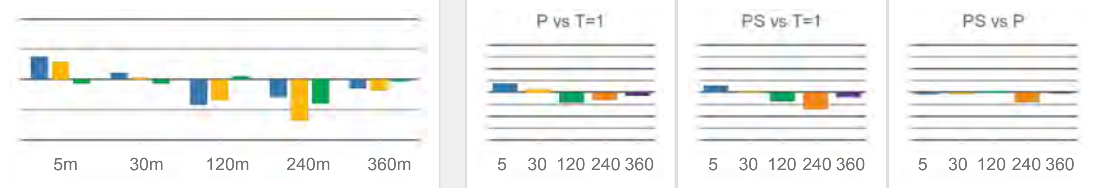

PGN_0531

Locus	5m	30m	120m	240m	360m
P vs T=1	0.406	0.440	0.217	0.217	0.096
PS vs T=1	0.399	0.505	0.353	0.098	0.193
PS vs P	-0.000	0.073	0.134	-0.117	0.094

putative von Willebrand factor type A

hypothetical proteins-Conserved

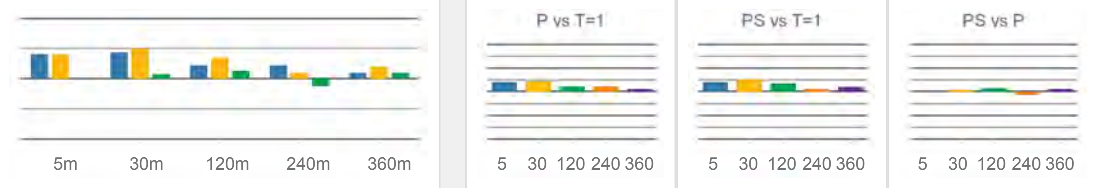

PGN_0532

Locus	5m	30m	120m	240m	360m
P vs T=1	1.499	1.668	1.348	1.361	1.274
PS vs T=1	1.391	1.993	1.882	1.370	1.391
PS vs P	-0.096	0.349	0.527	0.014	0.117

magnesium chelatase subunit I

unknown function

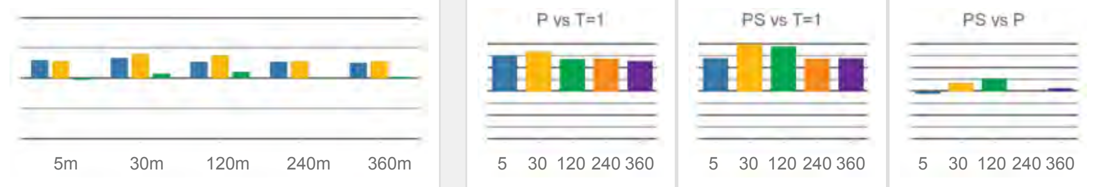

log2 (Fold Change)

Legend: P vs T=1 | PS vs T=1 | PS vs P

Locus		5m	30m	120m	240m	360m
**PGN_0533**	P vs T=1	-0.436	-0.199	-0.771	-1.268	-1.137
	PS vs T=1	0.096	0.083	-0.738	-1.113	-1.106
	PS vs P	0.526	0.306	0.025	0.070	0.003

putative quinolinate synthetase complex subunit A

biosynthesis of cofactors, prosthetic groups, and carriers

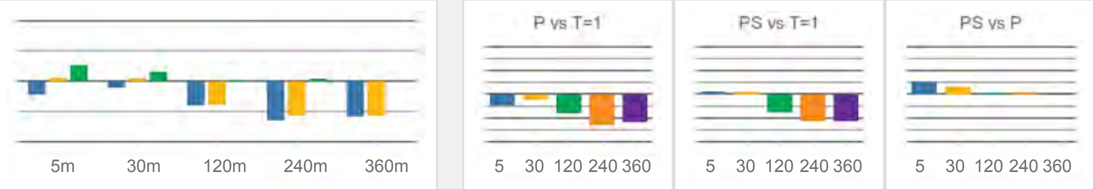

Locus		5m	30m	120m	240m	360m
**PGN_0534**	P vs T=1	-0.307	-0.135	-0.916	-1.411	-1.486
	PS vs T=1	0.082	-0.089	-0.929	-1.303	-1.222
	PS vs P	0.396	0.077	-0.030	-0.019	0.187

putative nicotinate-nucleotide pyrophosphorylase

biosynthesis of cofactors, prosthetic groups, and carriers

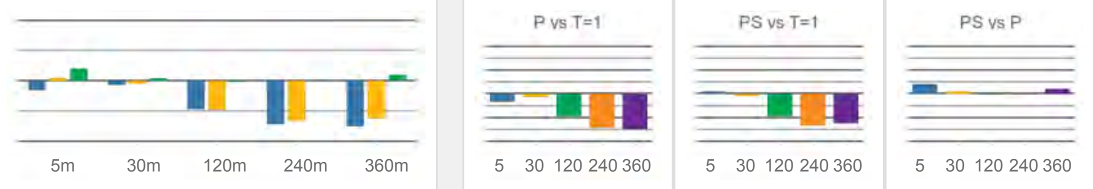

Locus		5m	30m	120m	240m	360m
**PGN_0535**	P vs T=1	-1.035	-0.989	-1.043	-1.108	-1.254
	PS vs T=1	-0.788	-1.155	-1.717	-1.741	-1.627
	PS vs P	0.242	-0.157	-0.657	-0.616	-0.380

L-aspartate oxidase

biosynthesis of cofactors, prosthetic groups, and carriers

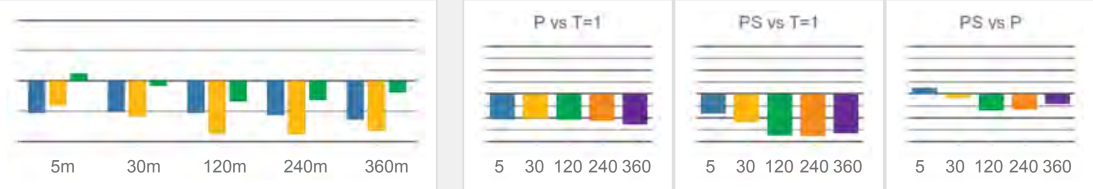

Locus		5m	30m	120m	240m	360m
**PGN_0536**	P vs T=1	-0.541	-0.177	0.910	1.293	1.176
	PS vs T=1	-1.911	-1.857	-0.938	-0.538	-0.086
	PS vs P	-1.308	-1.470	-1.470	-1.309	-0.967

conserved hypothetical protein

hypothetical proteins-Conserved

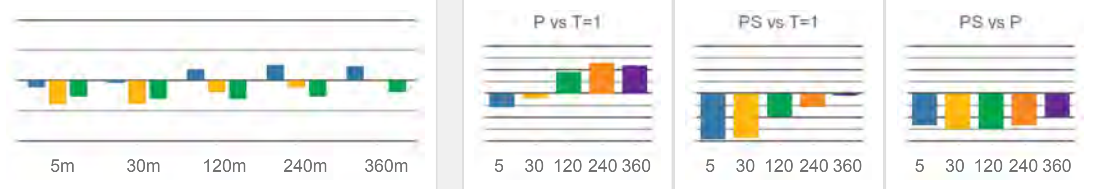

Locus		5m	30m	120m	240m	360m
**PGN_0537**	P vs T=1	-0.239	0.236	0.258	-0.378	-0.997
	PS vs T=1	-0.816	-0.392	-0.057	-0.165	-0.485
	PS vs P	-0.584	-0.539	-0.239	0.163	0.403

probable transcriptional regulator Crp family

regulatory functions

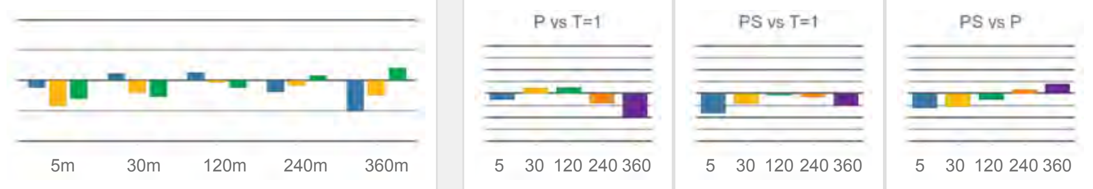

Locus		5m	30m	120m	240m	360m
**PGN_0538**	P vs T=1	-0.490	-0.466	-0.186	0.556	1.221
	PS vs T=1	-0.368	-0.528	0.135	0.282	0.568
	PS vs P	0.069	-0.124	0.238	-0.142	-0.513

conserved hypothetical protein

hypothetical proteins-Conserved

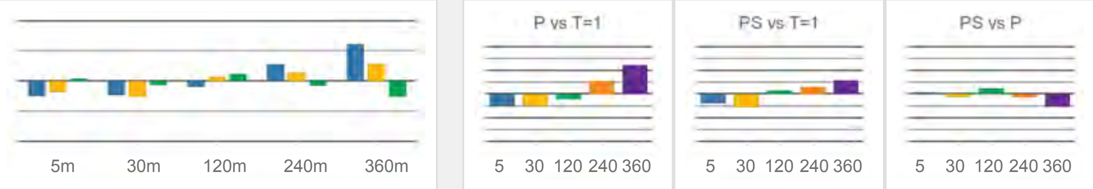

Locus		5m	30m	120m	240m	360m
**PGN_0539**	P vs T=1	0.593	0.861	1.174	1.857	2.601
	PS vs T=1	1.321	1.247	1.538	1.640	1.691
	PS vs P	0.636	0.334	0.323	-0.084	-0.744

metallo-beta-lactamase superfamily protein

unknown function

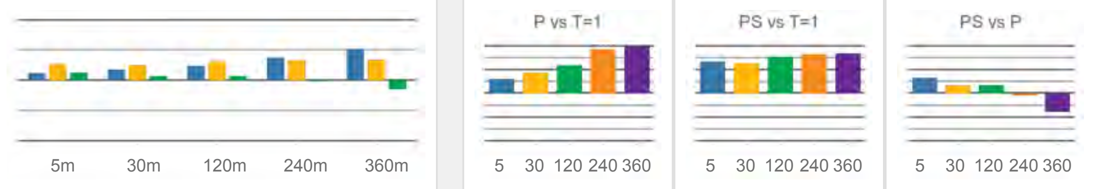

PgSg Time Course

log2 (Fold Change)

Locus		5m	30m	120m	240m	360m
**PGN_0540**	P vs T=1	0.139	0.680	1.633	1.662	1.449
	PS vs T=1	0.188	0.594	1.119	0.969	0.994
	PS vs P	-0.069	-0.098	-0.334	-0.490	-0.353
	conserved hypothetical protein					
	hypothetical proteins-Conserved					
**PGN_0541**	P vs T=1	-0.903	-0.232	0.427	0.227	2.274
	PS vs T=1	-0.229	0.026	0.902	1.088	1.441
	PS vs P	-0.144	-0.064	0.390	0.345	0.135
	hypothetical protein					
	hypothetical proteins					
**PGN_0542**	P vs T=1	-0.330	-0.273	0.185	0.341	0.533
	PS vs T=1	-0.184	-0.119	0.762	0.814	0.933
	PS vs P	0.048	0.052	0.575	0.493	0.456
	partial transposase in ISPg2					
**PGN_0543**	P vs T=1	-0.122	-0.463	-1.236	-1.609	-1.452
	PS vs T=1	-0.362	-0.874	-0.882	-0.917	-1.182
	PS vs P	-0.215	-0.394	0.328	0.609	0.242
	glutamyl-tRNA synthetase					
	protein synthesis					
**PGN_0544**	P vs T=1	-0.634	-0.881	-1.318	-1.128	-0.534
	PS vs T=1	-0.297	-0.650	-0.959	-1.066	-0.969
	PS vs P	0.344	0.223	0.284	0.022	-0.395
	3-deoxy-D-manno-octulosonic-acid transferase					
	cell envelope					
**PGN_0545**	P vs T=1	-0.898	-1.757	-2.613	-2.230	-1.331
	PS vs T=1	-0.534	-1.423	-2.560	-2.776	-2.421
	PS vs P	0.402	0.323	-0.068	-0.579	-1.038
	putative sulfatase					
	cell envelope					
**PGN_0546**	P vs T=1	-0.550	-1.002	-1.303	-1.236	-1.390
	PS vs T=1	-0.350	-0.600	-0.547	-0.552	-0.719
	PS vs P	0.209	0.366	0.691	0.627	0.618
	glucose-1-phosphate thymidylyltransferase					
	cell envelope					

P vs T=1 | PS vs T=1 | PS vs P

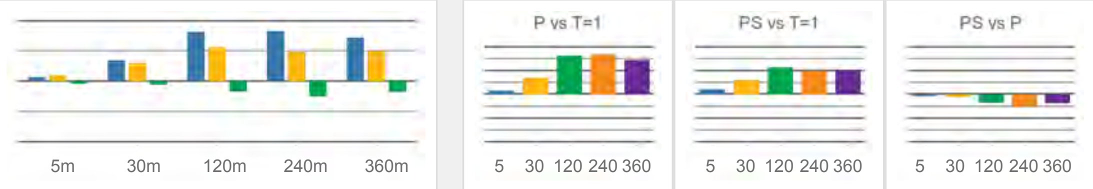

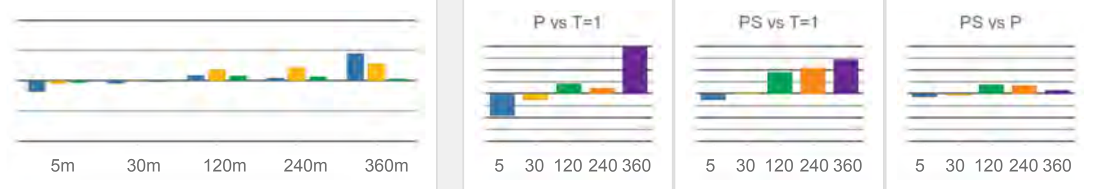

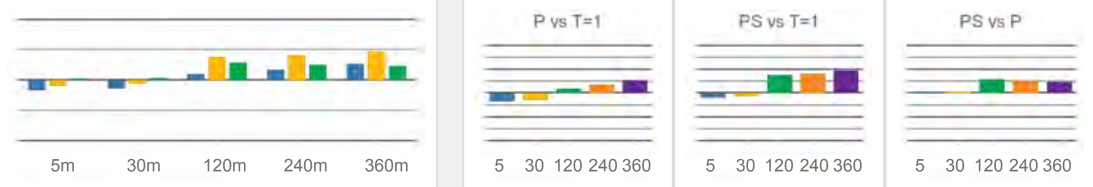

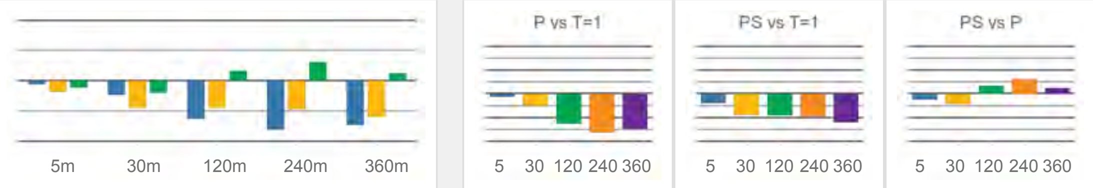

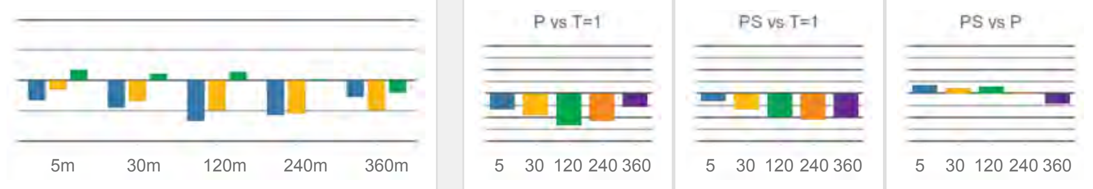

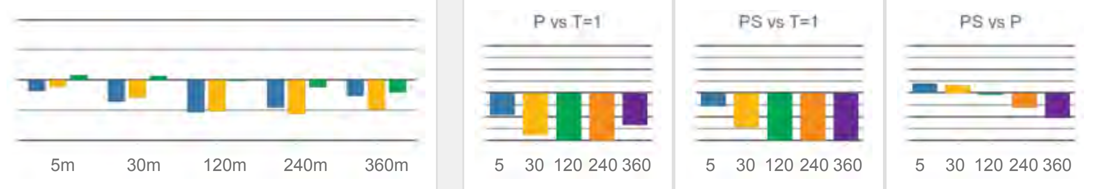

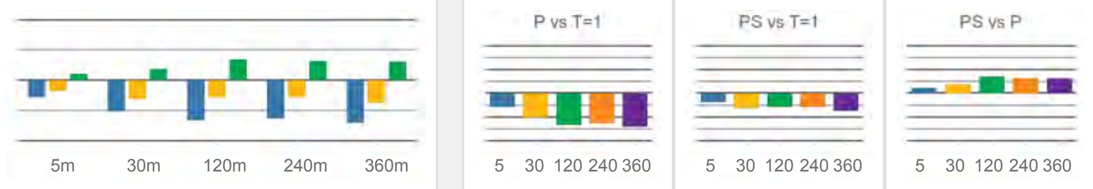

log2 (Fold Change)

Legend: P vs T=1 | PS vs T=1 | PS vs P

Locus		5m	30m	120m	240m	360m
**PGN_0547**	P vs T=1	-0.233	-1.129	-1.684	-1.813	-1.476
	PS vs T=1	0.236	-0.343	-0.631	-0.816	-1.089
	PS vs P	0.497	0.753	0.961	0.855	0.353
	dTDP-4-dehydrorhamnose 3,5-epimerase					
	cell envelope					

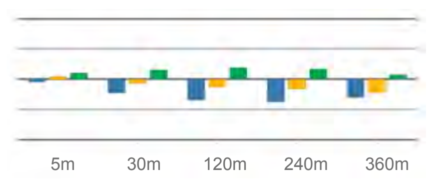

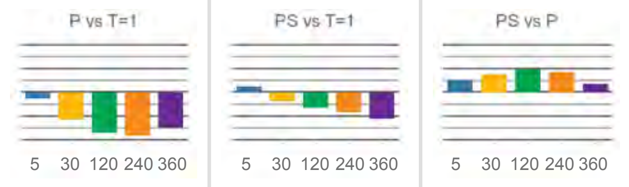

Locus		5m	30m	120m	240m	360m
**PGN_0548**	P vs T=1	-1.195	-1.515	-2.249	-2.254	-1.906
	PS vs T=1	-0.201	-0.818	-1.597	-1.961	-1.675
	PS vs P	0.991	0.674	0.503	0.121	0.186
	putative dTDP-4-dehydrorhamnose reductase					
	cell envelope					

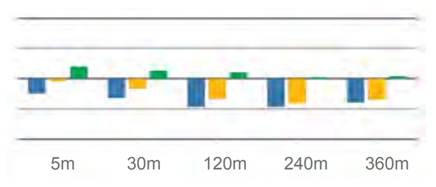

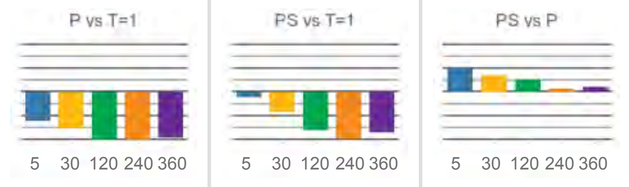

Locus		5m	30m	120m	240m	360m
**PGN_0549**	P vs T=1	-0.651	-0.592	-1.024	-1.588	-1.573
	PS vs T=1	0.554	0.304	-0.587	-1.324	-1.519
	PS vs P	1.192	0.892	0.424	0.164	0.008
	dTDP-glucose 4,6-dehydratase					
	cell envelope					

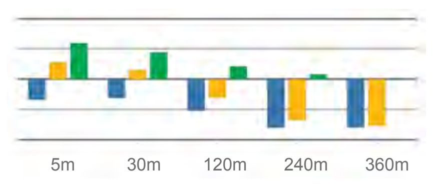

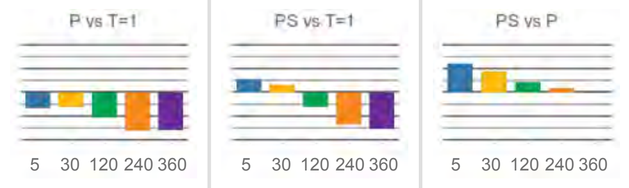

Locus		5m	30m	120m	240m	360m
**PGN_0550**	P vs T=1	0.508	0.945	1.277	1.271	0.606
	PS vs T=1	1.056	1.289	1.329	0.828	0.496
	PS vs P	0.522	0.344	0.071	-0.420	-0.118
	aminomethyltransferase					
	energy metabolism					

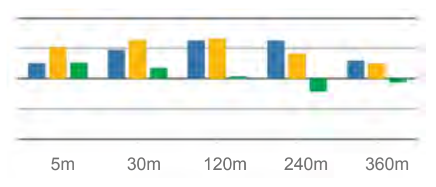

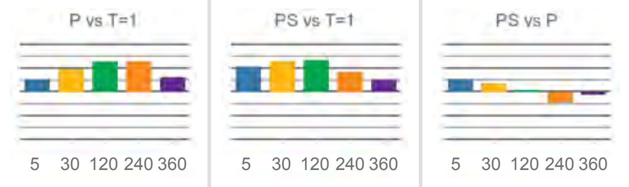

Locus		5m	30m	120m	240m	360m
**PGN_0551**	P vs T=1	0.493	0.353	1.846	2.751	4.024
	PS vs T=1	-0.686	0.462	2.197	2.314	2.691
	PS vs P	-0.981	-0.241	0.619	0.284	-0.160
	conserved hypothetical protein					
	hypothetical proteins-Conserved					

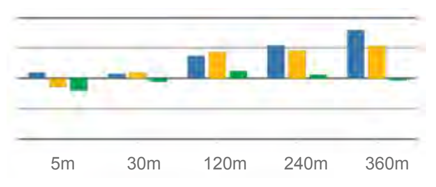

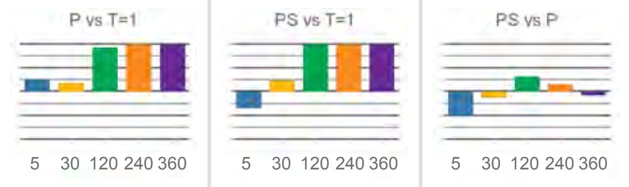

Locus		5m	30m	120m	240m	360m
**PGN_0552**	P vs T=1	0.154	-0.143	1.927	2.677	4.374
	PS vs T=1	-0.055	-0.048	2.120	2.707	2.722
	PS vs P	-0.368	-0.432	0.646	0.717	-0.102
	hypothetical protein					
	hypothetical proteins					

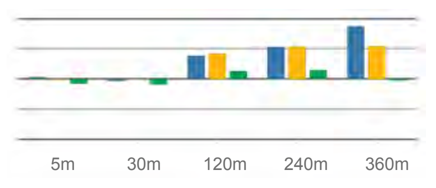

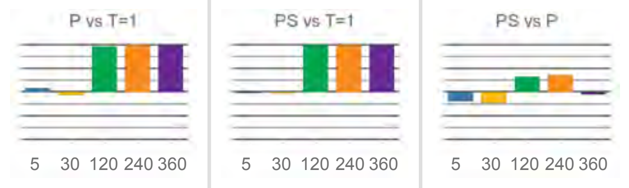

Locus		5m	30m	120m	240m	360m
**PGN_0553**	P vs T=1	0.568	0.922	0.844	2.811	4.055
	PS vs T=1	-0.120	-0.384	0.269	1.948	1.371
	PS vs P	-0.582	-0.788	-0.662	0.176	-1.245
	conserved hypothetical protein					
	hypothetical proteins-Conserved					

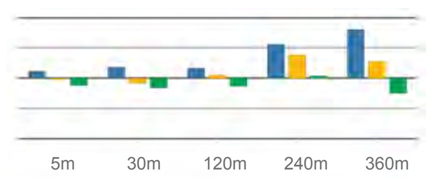

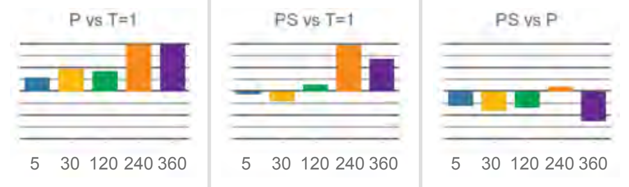

PgSg Time Course

log$_2$ (Fold Change)

Legend: P vs T=1 | PS vs T=1 | PS vs P

Locus		5m	30m	120m	240m	360m
**PGN_0554**	P vs T=1	0.627	1.016	2.057	3.028	4.350
	PS vs T=1	0.286	0.102	1.580	1.126	1.461
	PS vs P	-0.427	-0.667	-0.076	-0.692	-1.454
	conserved hypothetical protein					
	hypothetical proteins-Conserved					
**PGN_0555**	P vs T=1	0.197	0.566	1.319	2.768	4.223
	PS vs T=1	0.219	0.614	1.342	1.704	2.544
	PS vs P	-0.318	-0.157	-0.038	-0.259	-0.731
	conserved hypothetical protein					
	hypothetical proteins-Conserved					
**PGN_0556** *CobNST*	P vs T=1	0.785	0.433	0.708	1.446	2.566
	PS vs T=1	0.130	-0.321	0.349	0.929	1.390
	PS vs P	-0.624	-0.755	-0.376	-0.425	-1.069
	putative cobalamin biosynthesis-related protein					
	biosynthesis of cofactors, prosthetic groups, and carriers/ unknown f[illegible]					
**PGN_0557** *hmuR*	P vs T=1	0.537	0.556	1.241	1.653	2.469
	PS vs T=1	-0.425	-0.139	0.985	1.505	1.653
	PS vs P	-0.940	-0.695	-0.195	-0.032	-0.654
	TonB-dependent receptor HmuR					
	transport and binding proteins					
**PGN_0558**	P vs T=1	2.308	2.092	2.224	2.268	2.627
	PS vs T=1	-0.480	-0.191	0.389	0.292	0.760
	PS vs P	-2.516	-2.040	-1.627	-1.693	-1.638
	conserved hypothetical protein					
	transport and binding proteins					
**PGN_0559**	P vs T=1	-0.097	0.228	1.529	1.825	2.423
	PS vs T=1	-0.841	-0.423	0.502	1.262	1.731
	PS vs P	-0.781	-0.569	-0.380	0.091	0.084
	conserved hypothetical protein					
	hypothetical proteins-Conserved					
**PGN_0560**	P vs T=1	-0.602	-0.928	-0.189	0.032	0.467
	PS vs T=1	-0.575	-1.017	0.770	0.964	1.179
	PS vs P	-0.131	-0.437	0.846	0.822	0.821
	hypothetical protein					
	hypothetical proteins					

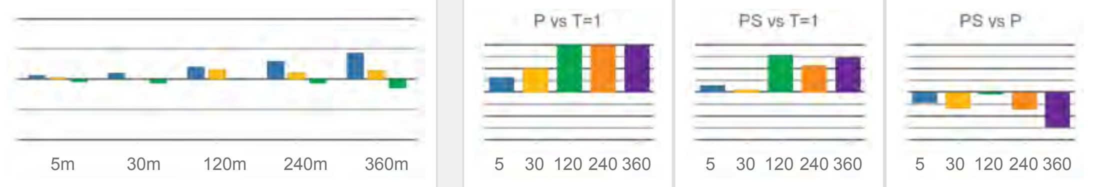

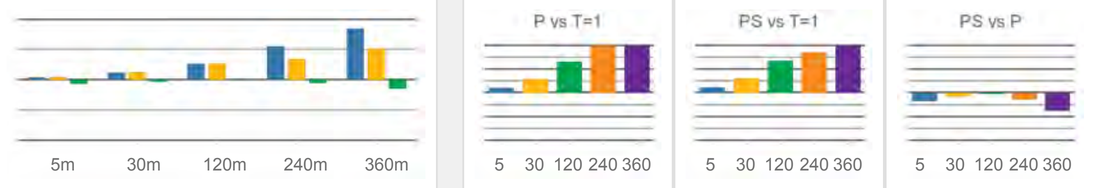

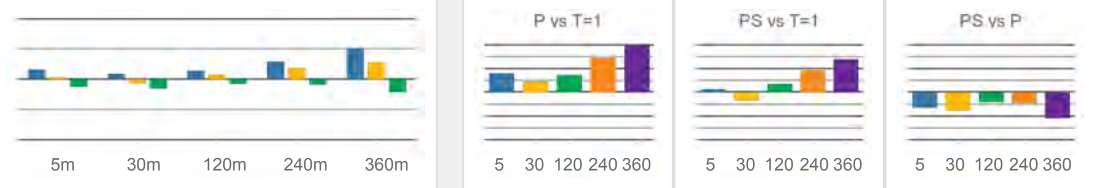

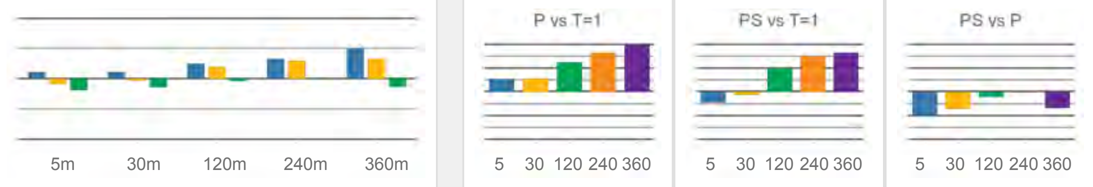

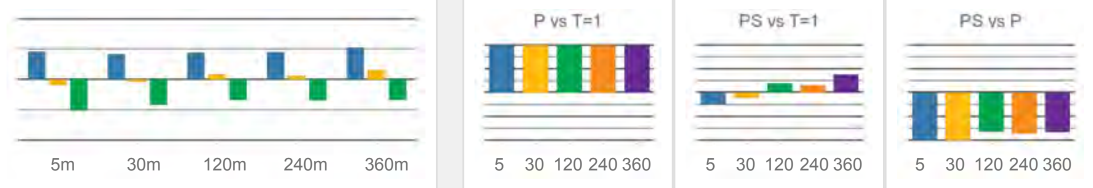

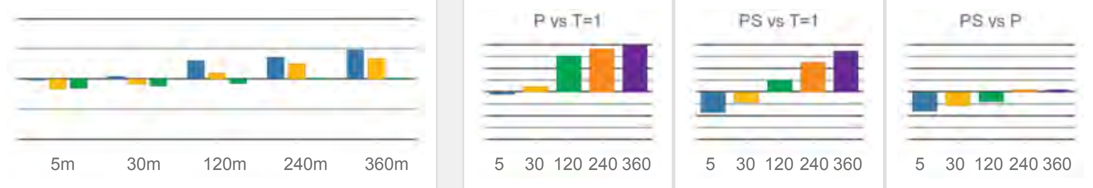

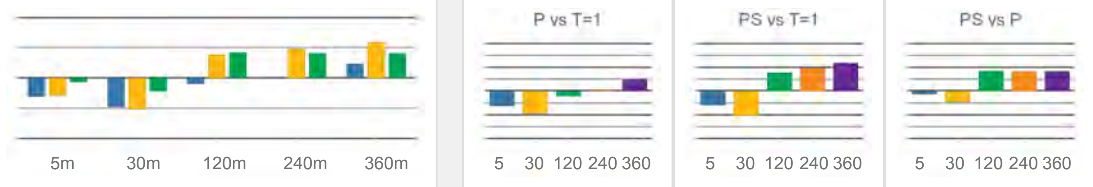

log2 (Fold Change)

Legend: P vs T=1 | PS vs T=1 | PS vs P

Locus		5m	30m	120m	240m	360m
**PGN_0561**	P vs T=1	-0.763	-0.628	-0.613	-0.521	-0.554
prtT	PS vs T=1	-1.469	-1.257	0.094	0.147	-0.057
	PS vs P	-0.705	-0.619	0.702	0.666	0.493

trypsin like proteinase PrtT

protein fate

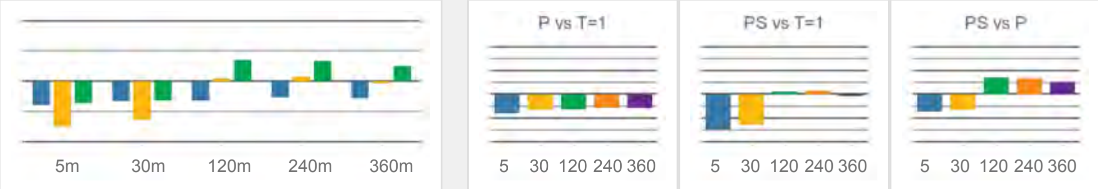

Locus		5m	30m	120m	240m	360m
**PGN_0562**	P vs T=1	0.965	2.066	2.758	2.817	2.205
	PS vs T=1	-0.535	2.020	4.409	4.309	4.014
	PS vs P	-1.606	-0.006	1.711	1.532	1.754

conserved hypothetical protein

hypothetical proteins-Conserved

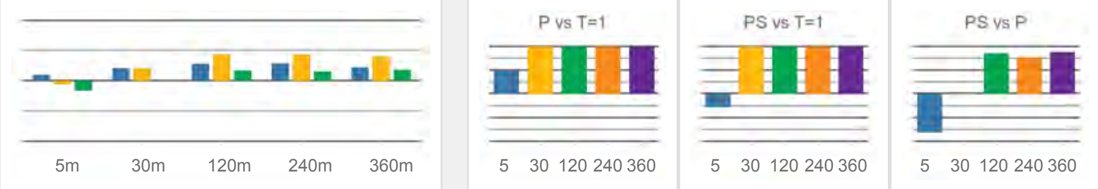

Locus		5m	30m	120m	240m	360m
**PGN_0563**	P vs T=1	-0.285	0.528	1.526	1.601	0.952
	PS vs T=1	-1.601	0.364	2.294	2.374	2.355
	PS vs P	-1.403	-0.173	0.835	0.825	1.369

conserved hypothetical protein

hypothetical proteins-Conserved

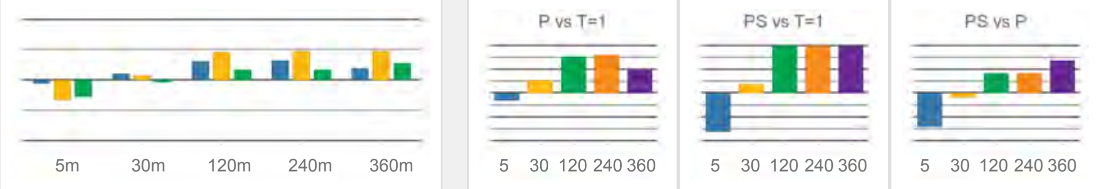

Locus		5m	30m	120m	240m	360m
**PGN_0564**	P vs T=1	0.653	1.448	1.900	1.715	1.028
sod	PS vs T=1	-0.442	1.381	2.904	3.046	2.824
	PS vs P	-1.128	-0.044	1.033	1.333	1.763

superoxide dismutase Fe-Mn

cellular processes

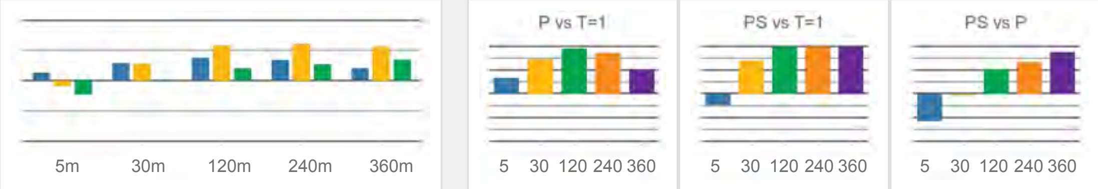

Locus		5m	30m	120m	240m	360m
**PGN_0565**	P vs T=1	0.640	0.636	0.114	0.329	1.148
	PS vs T=1	1.607	1.688	1.527	1.027	0.845
	PS vs P	0.931	1.014	1.233	0.562	-0.242

conserved hypothetical protein

hypothetical proteins-Conserved

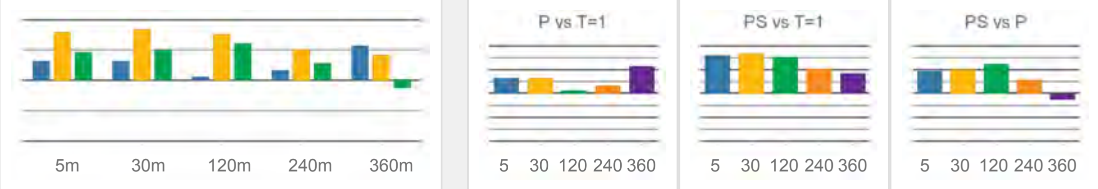

Locus		5m	30m	120m	240m	360m
**PGN_0566**	P vs T=1	-0.691	-0.597	-0.493	-0.456	-0.762
	PS vs T=1	0.088	0.371	0.624	0.317	0.168
	PS vs P	0.727	0.922	1.089	0.750	0.886

probable thioesterase protein

hypothetical proteins-Conserved

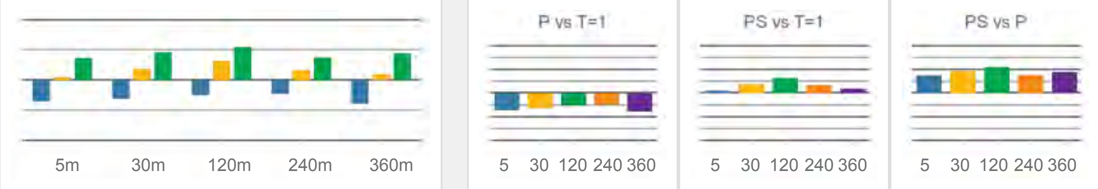

Locus		5m	30m	120m	240m	360m
**PGN_0567**	P vs T=1	-0.070	-0.204	-0.254	-0.069	0.021
prtC	PS vs T=1	0.795	0.744	0.678	0.470	0.159
	PS vs P	0.838	0.903	0.885	0.521	0.136

collagenase

protein fate

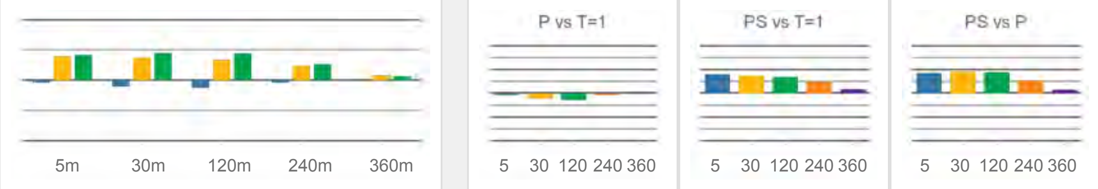

Locus		5m	30m	120m	240m	360m
		log$_2$ (Fold Change)				
**PGN_0568**	P vs T=1	-0.770	-0.376	-0.283	-0.320	-0.833
	PS vs T=1	-0.798	-0.462	0.516	0.329	0.007
	PS vs P	-0.077	-0.087	0.790	0.633	0.774
	2-amino-4-hydroxy-6- hydroxymethyldihydropteridine pyrophosphokinase					
	biosynthesis of cofactors, prosthetic groups, and carriers					
**PGN_0569**	P vs T=1	0.736	1.370	1.498	1.334	0.641
	PS vs T=1	0.185	1.319	2.702	2.791	2.540
	PS vs P	-0.583	-0.031	1.209	1.439	1.846
	S-adenosylmethionine:tRNA ribosyltransferase-isomerase					
	protein synthesis					
**PGN_0570**	P vs T=1	0.716	0.612	0.528	0.168	0.490
	PS vs T=1	0.802	1.094	1.216	1.111	0.999
	PS vs P	0.088	0.463	0.665	0.818	0.490
	putative tRNA pseudouridine synthase B					
	protein synthesis					
**PGN_0571**	P vs T=1	-0.053	-0.294	-0.604	-0.369	0.368
	PS vs T=1	0.135	-0.159	-0.757	-0.699	-0.746
	PS vs P	0.216	0.133	-0.213	-0.313	-0.982
	putative undecaprenol kinase					
	cellular processes					
**PGN_0572**	P vs T=1	-0.624	-0.517	-0.944	-1.469	-1.076
	PS vs T=1	-0.742	-0.900	-1.459	-1.408	-1.457
	PS vs P	-0.090	-0.314	-0.490	-0.136	-0.378
	conserved hypothetical protein					
	hypothetical proteins-Conserved					
**PGN_0573**	P vs T=1	0.071	0.079	-0.468	-0.554	-0.323
	PS vs T=1	0.086	-0.109	-0.459	-0.694	-0.345
	PS vs P	0.023	-0.173	-0.014	-0.176	-0.022
	putative cell division protein FtsX					
	cellular processes					
**PGN_0574**	P vs T=1	-0.492	-0.402	0.295	1.075	1.646
	PS vs T=1	-0.029	0.767	1.861	2.011	2.149
	PS vs P	0.194	0.840	1.342	0.941	0.586
	conserved hypothetical protein					
	hypothetical proteins-Conserved					

P vs T=1 | PS vs T=1 | PS vs P

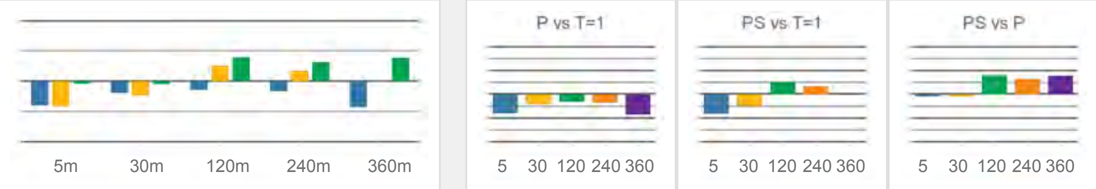

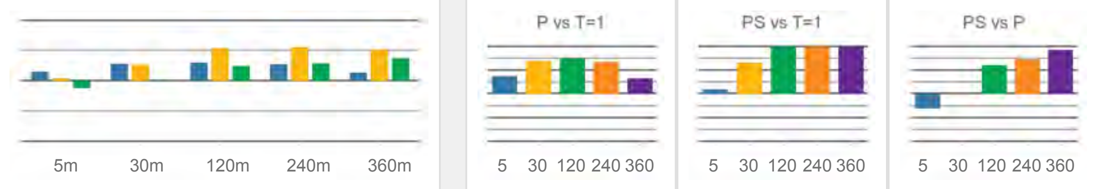

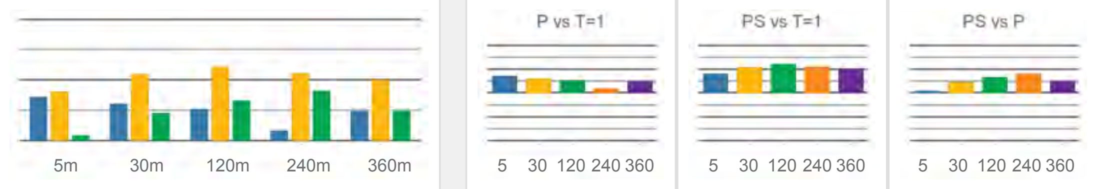

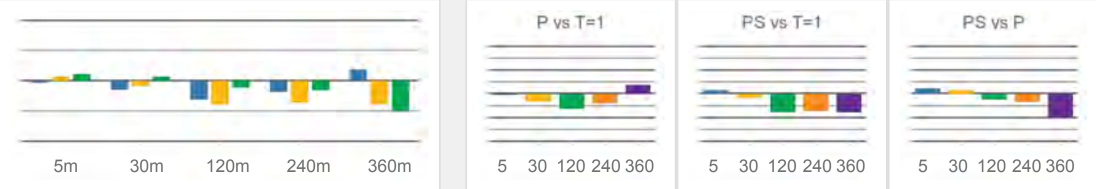

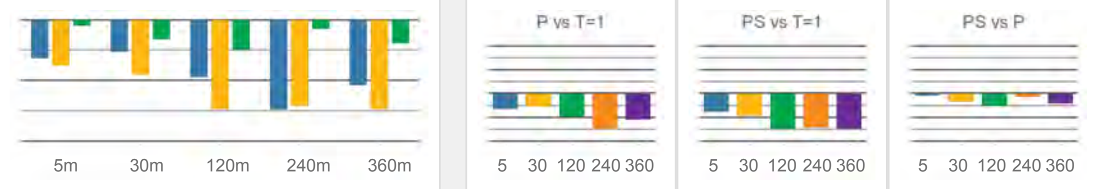

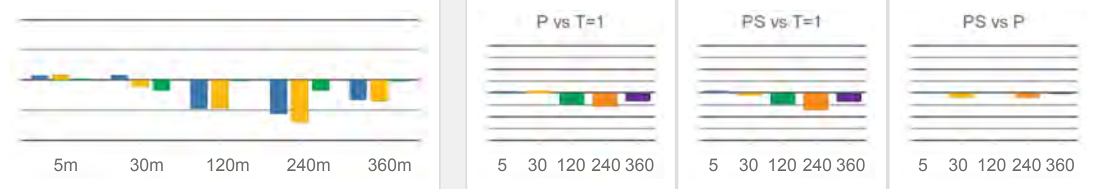

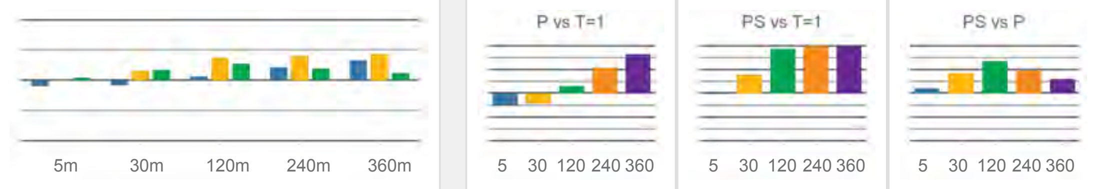

log$_2$ (Fold Change)

P vs T=1 | PS vs T=1 | PS vs P

Locus		5m	30m	120m	240m	360m
**PGN_0575**	P vs T=1	0.537	0.458	0.535	0.639	0.375
	PS vs T=1	0.329	0.162	0.374	0.345	0.327
	PS vs P	-0.206	-0.302	-0.167	-0.290	-0.062

transposase in ISPg1

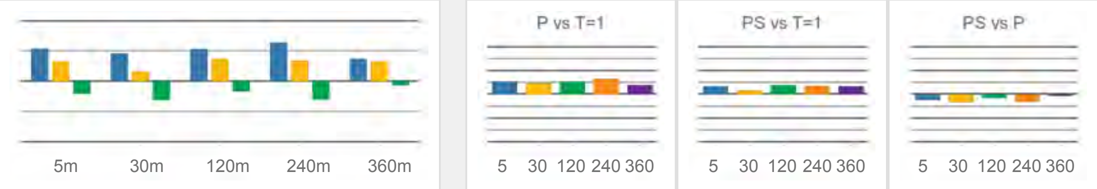

Locus		5m	30m	120m	240m	360m
**PGN_0576**	P vs T=1	0.244	-0.344	-0.005	0.712	1.735
	PS vs T=1	1.064	0.675	0.727	0.490	0.662
	PS vs P	0.672	0.417	0.369	0.004	-0.235

hypothetical protein

hypothetical proteins

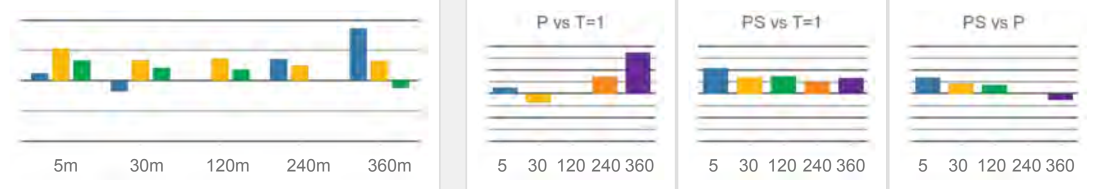

Locus		5m	30m	120m	240m	360m
**PGN_0577**	P vs T=1	0.337	0.302	0.311	0.459	0.117
	PS vs T=1	0.172	-0.042	0.069	0.187	0.206
	PS vs P	-0.164	-0.346	-0.249	-0.266	0.071

transposase in ISPg1

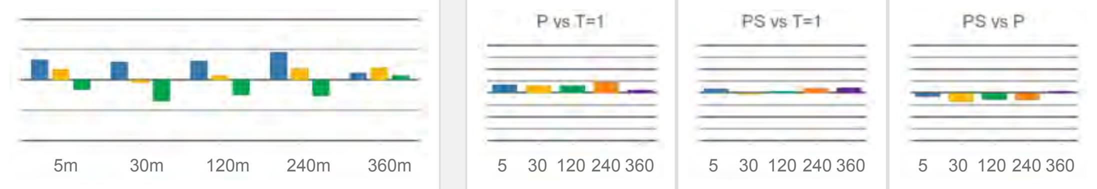

Locus		5m	30m	120m	240m	360m
**PGN_0578**	P vs T=1	1.113	0.643	0.766	1.477	1.766
	PS vs T=1	0.927	0.496	1.238	1.125	1.408
	PS vs P	-0.149	-0.227	0.319	-0.229	-0.246

conserved hypothetical protein found in conjugate transposon

hypothetical proteins-Conserved

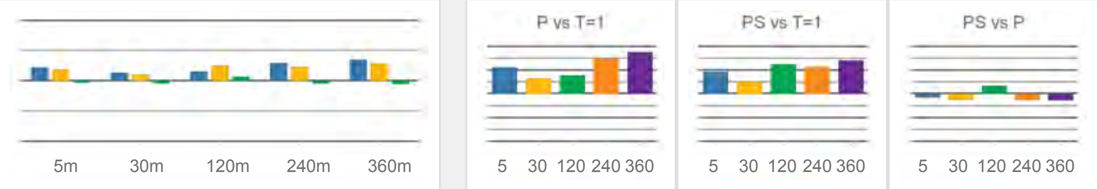

Locus		5m	30m	120m	240m	360m
**PGN_0579**	P vs T=1	0.932	0.158	0.424	1.024	1.990
	PS vs T=1	0.961	0.848	1.264	1.227	1.236
	PS vs P	0.065	0.486	0.672	0.212	-0.583

conserved hypothetical protein

hypothetical proteins-Conserved

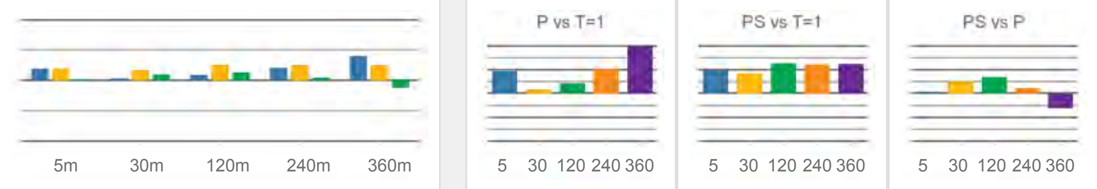

Locus		5m	30m	120m	240m	360m
**PGN_0580**	P vs T=1	-0.557	-1.439	-1.712	-1.587	-0.341
	PS vs T=1	0.071	0.247	0.359	0.282	-0.107
	PS vs P	0.577	1.224	1.606	1.446	0.282

conserved hypothetical protein

hypothetical proteins-Conserved

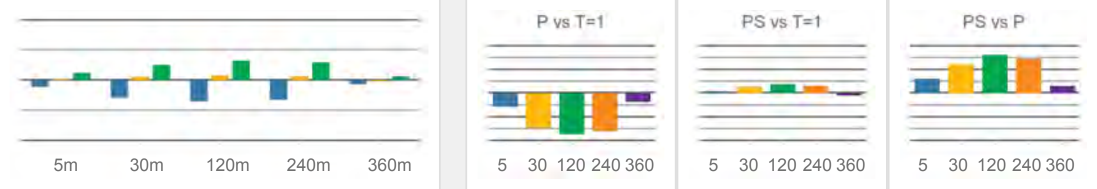

Locus		5m	30m	120m	240m	360m
**PGN_0581**	P vs T=1	-0.038	-0.735	0.041	0.984	2.797
	PS vs T=1	-0.896	-0.083	1.101	1.214	1.487
	PS vs P	-0.817	0.199	0.845	0.369	-0.773

conserved hypothetical protein

hypothetical proteins-Conserved

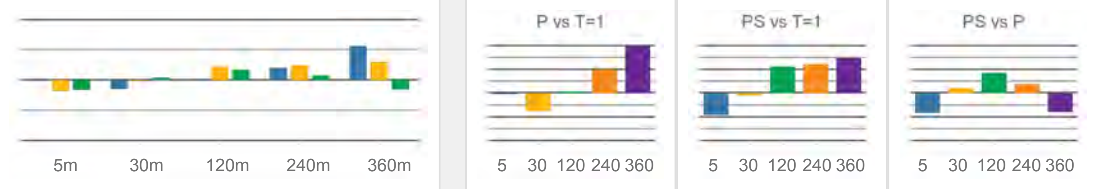

PgSg Time Course

log$_2$ (Fold Change)

Legend: P vs T=1 | PS vs T=1 | PS vs P

Locus		5m	30m	120m	240m	360m
**PGN_0582**	P vs T=1	0.117	-0.592	-0.096	0.652	2.384
	PS vs T=1	-0.584	-0.435	1.198	1.483	1.369
	PS vs P	-0.656	-0.114	1.073	0.820	-0.691

DNA topoisomerase I

DNA metabolism

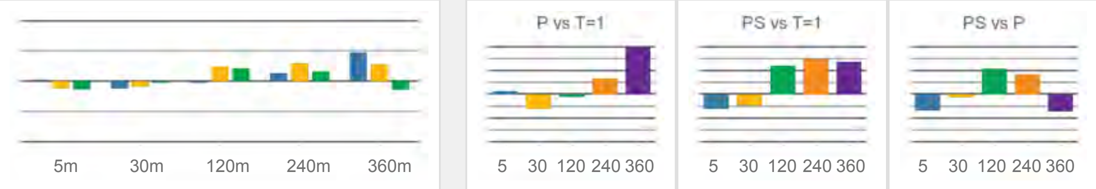

Locus		5m	30m	120m	240m	360m
**PGN_0583**	P vs T=1	-1.173	-2.102	-2.818	-3.035	-1.914
	PS vs T=1	-0.820	-1.217	-1.789	-1.786	-1.821
	PS vs P	0.452	0.652	0.624	0.716	0.078

conserved hypothetical protein

hypothetical proteins-Conserved

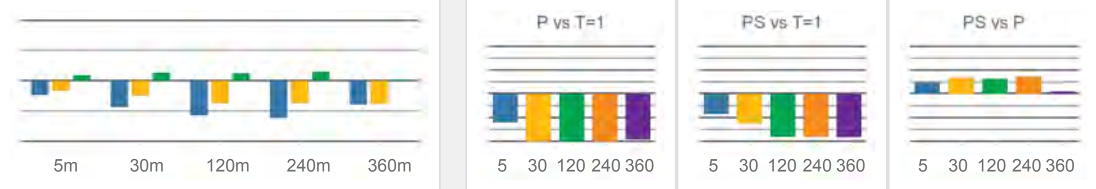

Locus		5m	30m	120m	240m	360m
**PGN_0584**	P vs T=1	-0.298	-1.217	-1.033	0.102	1.535
	PS vs T=1	-0.342	-0.547	-0.036	0.149	0.631
	PS vs P	-0.021	0.402	0.669	0.128	-0.678

conserved hypothetical protein

hypothetical proteins-Conserved

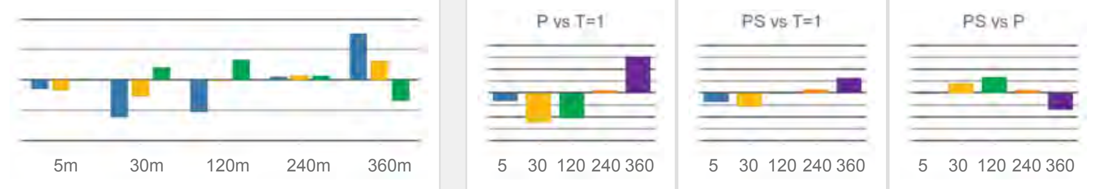

Locus		5m	30m	120m	240m	360m
**PGN_0585**	P vs T=1	0.302	-0.340	-0.220	0.385	1.680
	PS vs T=1	-0.099	0.012	0.326	0.761	0.695
	PS vs P	-0.369	0.224	0.382	0.354	-0.828

transposase in ISPg3

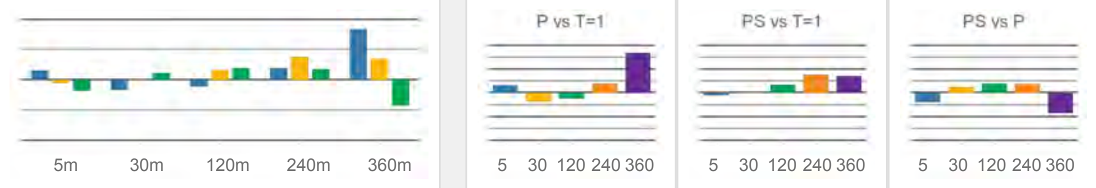

Locus		5m	30m	120m	240m	360m
**PGN_0586**	P vs T=1	0.518	-0.300	0.883	1.856	3.540
	PS vs T=1	-0.063	0.280	0.809	1.611	1.603
	PS vs P	-0.515	-0.004	-0.098	0.143	-1.163

conserved hypothetical protein

hypothetical proteins-Conserved

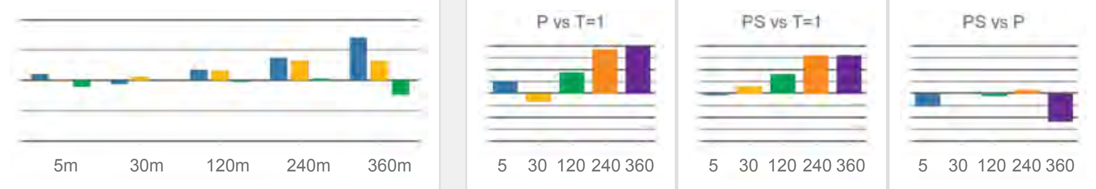

Locus		5m	30m	120m	240m	360m
**PGN_0587**	P vs T=1	0.271	0.221	0.273	0.339	-0.004
	PS vs T=1	0.060	-0.123	-0.052	0.068	0.104
	PS vs P	-0.209	-0.346	-0.329	-0.268	0.085

transposase in ISPg1

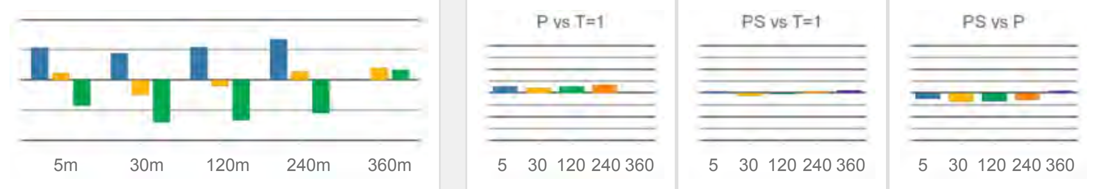

Locus		5m	30m	120m	240m	360m
**PGN_0588**	P vs T=1	1.868	1.746	1.498	1.552	1.976
	PS vs T=1	1.805	1.574	1.560	1.370	1.285
	PS vs P	-0.035	-0.154	0.039	-0.191	-0.635

conserved hypothetical protein

hypothetical proteins-Conserved

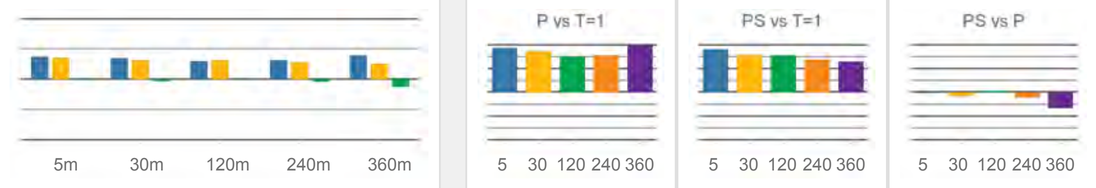

PgSg Time Course

log2 (Fold Change)

Legend: P vs T=1 | PS vs T=1 | PS vs P

Locus		5m	30m	120m	240m	360m
**PGN_0589**	P vs T=1	1.258	1.002	1.005	1.080	0.830
	PS vs T=1	1.188	1.098	0.741	-0.078	-0.178
	PS vs P	-0.005	0.105	-0.224	-1.004	-0.933

conserved hypothetical protein

hypothetical proteins-Conserved

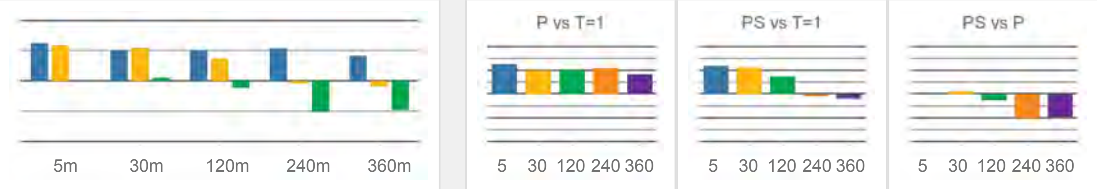

Locus		5m	30m	120m	240m	360m
**PGN_0590**	P vs T=1	-0.255	-0.522	-0.364	-0.382	-0.963
	PS vs T=1	-0.544	-0.414	-0.250	-0.136	-0.299
	PS vs P	-0.281	0.093	0.111	0.243	0.635

putative Fic family protein

unknown function

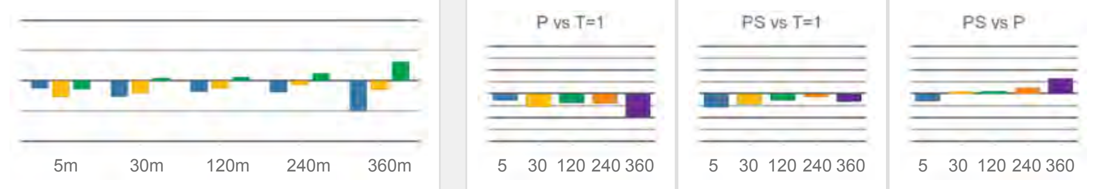

Locus		5m	30m	120m	240m	360m
**PGN_0591**	P vs T=1	-0.463	-0.947	-0.916	-0.997	-0.932
	PS vs T=1	-0.369	-0.019	0.470	0.718	0.636
	PS vs P	0.045	0.647	1.152	1.416	1.360

conserved hypothetical protein

hypothetical proteins-Conserved

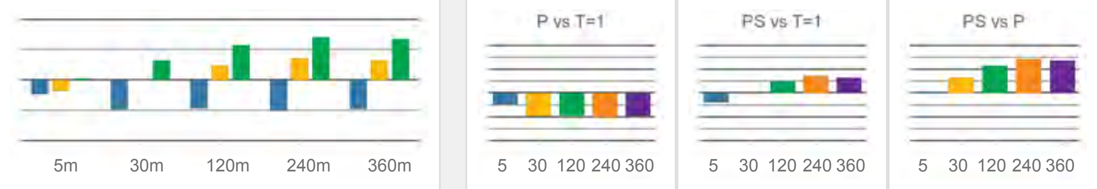

Locus		5m	30m	120m	240m	360m
**PGN_0592** *traQ*	P vs T=1	-0.006	0.309	1.245	1.089	2.016
	PS vs T=1	0.574	1.108	1.463	1.815	1.557
	PS vs P	0.403	0.653	0.306	0.676	-0.290

putative conserved protein found in conjugate transposon TraQ

mobile and extrachromosomal element functions

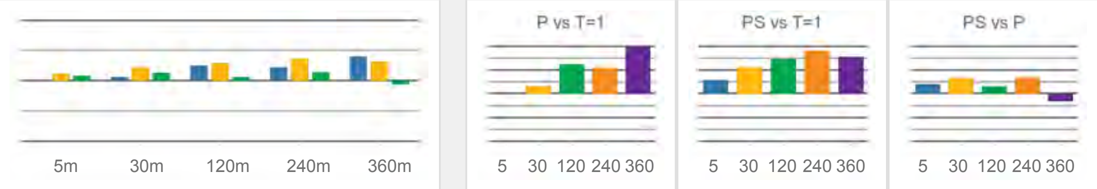

Locus		5m	30m	120m	240m	360m
**PGN_0593** *traO*	P vs T=1	0.899	1.015	1.856	2.369	2.998
	PS vs T=1	1.314	1.792	2.392	2.537	2.573
	PS vs P	0.250	0.570	0.538	0.320	-0.203

putative conserved protein found in conjugate transposon TraO

mobile and extrachromosomal element functions

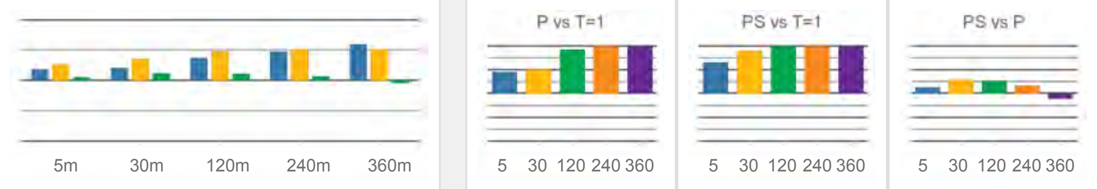

Locus		5m	30m	120m	240m	360m
**PGN_0594** *traN*	P vs T=1	0.506	0.870	1.840	2.384	3.176
	PS vs T=1	0.752	1.119	2.179	2.478	2.505
	PS vs P	0.061	0.114	0.388	0.271	-0.445

conserved protein found in conjugate transposon TraN

other categories

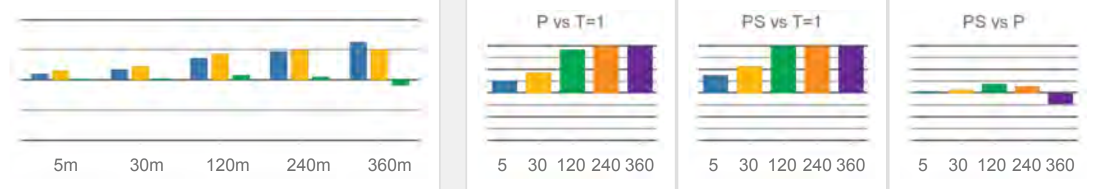

Locus		5m	30m	120m	240m	360m
**PGN_0595** *traM*	P vs T=1	0.412	0.477	1.301	2.473	3.394
	PS vs T=1	0.271	0.732	1.367	1.625	2.182
	PS vs P	-0.267	0.064	0.038	-0.448	-0.851

putative conserved protein found in conjugate transposon TraM

mobile and extrachromosomal element functions

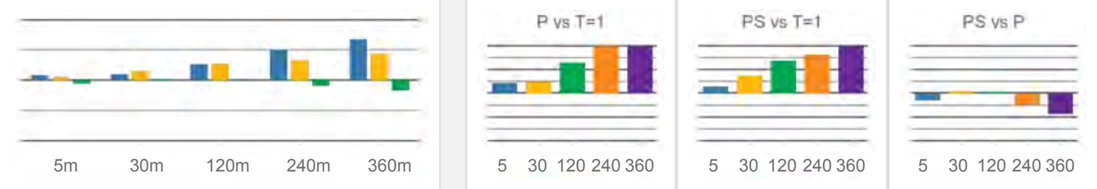

log$_2$ (Fold Change)

Legend: P vs T=1 | PS vs T=1 | PS vs P

PGN_0596

Locus		5m	30m	120m	240m	360m
PGN_0596	P vs T=1	0.570	0.318	1.162	2.254	3.449
	PS vs T=1	-0.050	0.311	1.740	2.229	2.829
	PS vs P	-0.577	-0.309	0.414	0.461	0.235

conserved hypothetical protein found in conjugate transposon

hypothetical proteins-Conserved

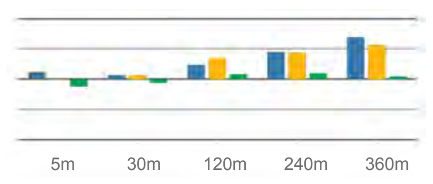

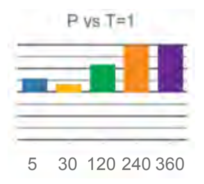

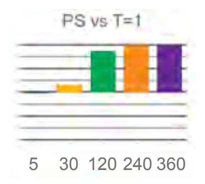

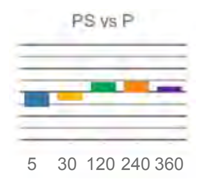

PGN_0597

Locus		5m	30m	120m	240m	360m
PGN_0597 *traK*	P vs T=1	0.497	0.397	1.210	2.557	3.992
	PS vs T=1	-0.060	-0.029	1.511	2.417	2.603
	PS vs P	-0.602	-0.580	0.160	0.379	-0.715

putative conserved protein found in conjugate transposon TraK

mobile and extrachromosomal element functions

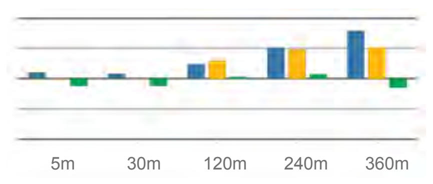

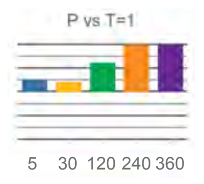

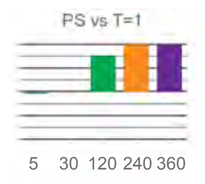

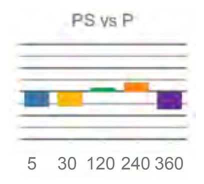

PGN_0598

Locus		5m	30m	120m	240m	360m
PGN_0598 *traJ*	P vs T=1	0.171	0.546	0.569	1.883	3.495
	PS vs T=1	-0.554	-0.498	0.825	1.659	1.291
	PS vs P	-0.756	-0.890	0.029	0.159	-1.620

conserved transmembrane protein found in conjugate transposon TraJ

other categories

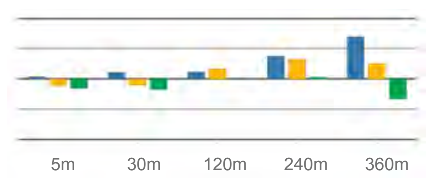

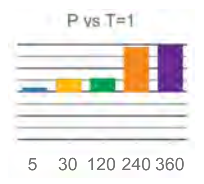

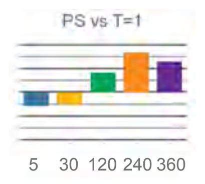

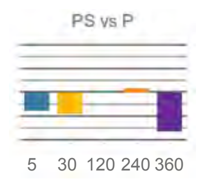

PGN_0599

Locus		5m	30m	120m	240m	360m
PGN_0599 *traI*	P vs T=1	-0.462	-0.718	0.107	0.842	2.153
	PS vs T=1	-0.771	-0.850	0.371	1.171	1.086
	PS vs P	-0.392	-0.387	0.200	0.518	-0.659

putative conserved protein found in conjugate transposon TraI

mobile and extrachromosomal element functions

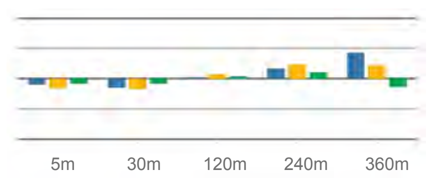

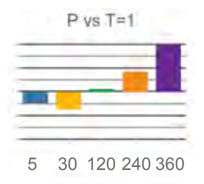

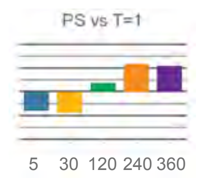

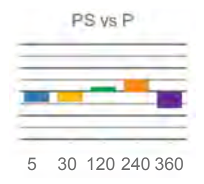

PGN_0600

Locus		5m	30m	120m	240m	360m
PGN_0600	P vs T=1	-0.298	-0.137	0.640	1.983	2.662
	PS vs T=1	-1.229	-0.574	0.309	1.565	1.083
	PS vs P	-0.977	-0.531	-0.301	0.261	-0.824

conserved hypothetical protein

hypothetical proteins-Conserved

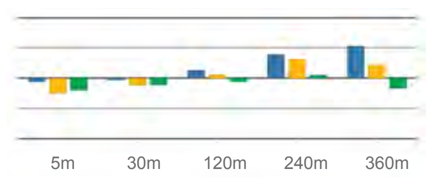

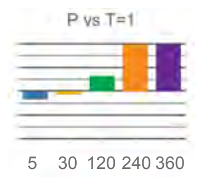

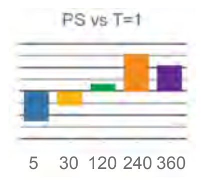

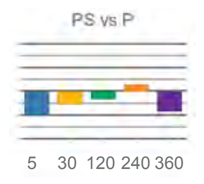

PGN_0601

Locus		5m	30m	120m	240m	360m
PGN_0601	P vs T=1	-0.110	0.071	1.044	1.874	2.545
	PS vs T=1	-0.037	-0.166	1.466	1.556	2.232
	PS vs P	-0.230	-0.449	0.436	0.126	0.173

conserved hypothetical protein

hypothetical proteins-Conserved

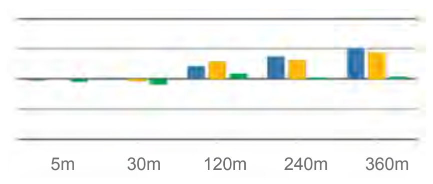

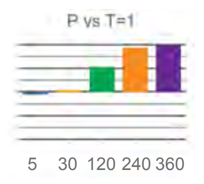

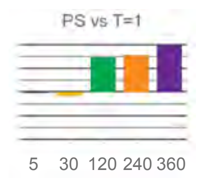

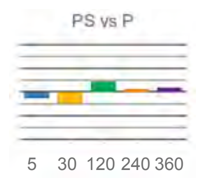

PGN_0602

Locus		5m	30m	120m	240m	360m
PGN_0602	P vs T=1	0.318	0.279	0.338	0.446	0.170
	PS vs T=1	0.092	-0.048	0.089	0.246	0.153
	PS vs P	-0.225	-0.331	-0.254	-0.197	-0.032

transposase in ISPg1

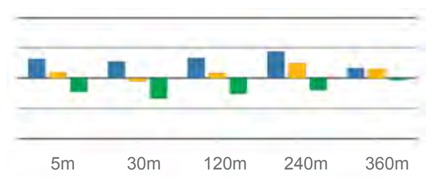

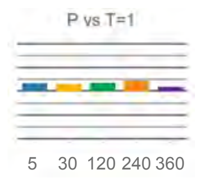

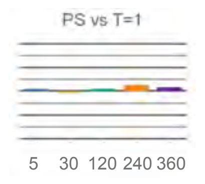

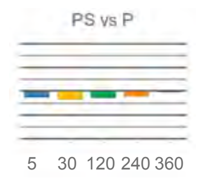

PgSg Time Course

log$_2$ (Fold Change)

Legend: P vs T=1 | PS vs T=1 | PS vs P

Locus		5m	30m	120m	240m	360m
**PGN_0603**	P vs T=1	-0.917	-0.237	0.159	0.727	0.040
	PS vs T=1	-1.019	-1.067	-0.998	-0.098	-0.692
	PS vs P	-0.349	-0.628	-0.836	-0.200	-0.548
	hypothetical protein					
	hypothetical proteins					

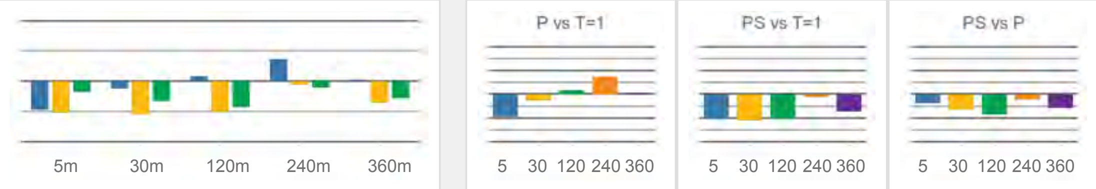

Locus		5m	30m	120m	240m	360m
**PGN_0604**	P vs T=1	1.541	2.151	2.327	2.009	1.339
	PS vs T=1	0.947	2.085	2.891	2.466	2.071
	PS vs P	-0.599	-0.042	0.588	0.462	0.717
	ferritin					
	transport and binding proteins					

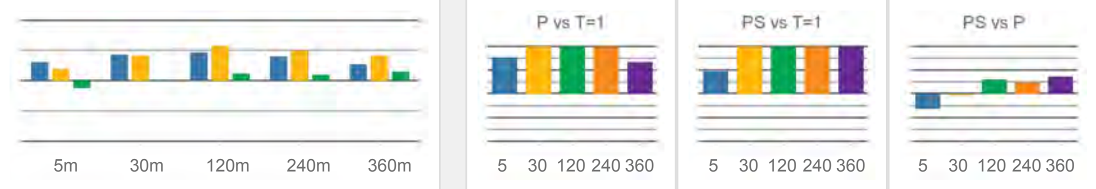

Locus		5m	30m	120m	240m	360m
**PGN_0605**	P vs T=1	0.190	0.115	0.183	0.202	-0.065
	PS vs T=1	0.001	-0.189	-0.050	-0.010	0.005
	PS vs P	-0.188	-0.308	-0.236	-0.214	0.051
	transposase in ISPg1					

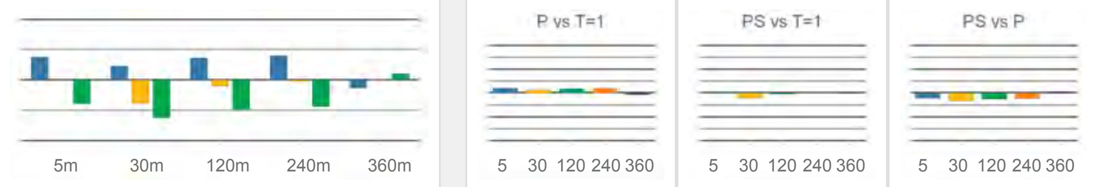

Locus		5m	30m	120m	240m	360m
**PGN_0606**	P vs T=1	0.252	0.574	0.661	1.165	1.706
	PS vs T=1	0.594	0.582	0.338	0.145	0.551
	PS vs P	0.282	0.012	-0.326	-0.887	-1.012
	glucosamine-6-phosphate isomerase					
	central intermediary metabolism					

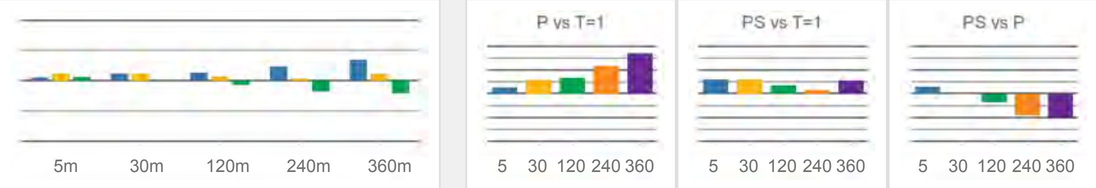

Locus		5m	30m	120m	240m	360m
**PGN_0607**	P vs T=1	0.847	1.289	1.228	0.806	0.178
dpp11	PS vs T=1	1.111	0.902	0.531	0.224	-0.061
	PS vs P	0.263	-0.366	-0.675	-0.574	-0.252
	dipeptidyl peptidase 11					
	protein fate					

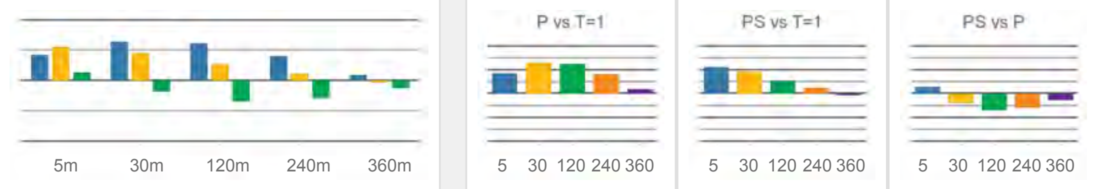

Locus		5m	30m	120m	240m	360m
**PGN_0608**	P vs T=1	-0.064	0.224	0.666	0.551	0.303
	PS vs T=1	0.076	0.247	0.727	0.992	0.900
	PS vs P	0.100	0.008	0.089	0.439	0.568
	conserved hypothetical protein					
	hypothetical proteins-Conserved					

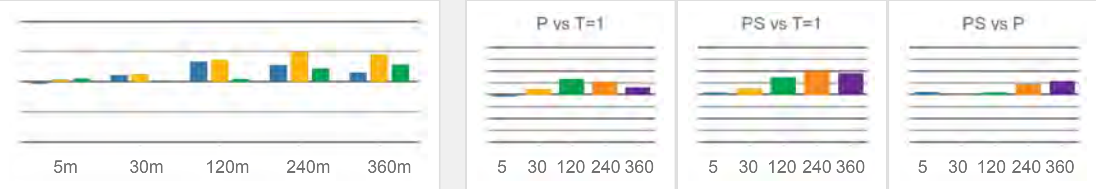

Locus		5m	30m	120m	240m	360m
**PGN_0609**	P vs T=1	-0.583	-0.407	-0.341	-0.387	-0.436
	PS vs T=1	-0.681	-0.282	0.204	0.829	0.490
	PS vs P	-0.144	0.100	0.512	1.151	0.878
	probable DNA mismatch repair protein					
	hypothetical proteins					

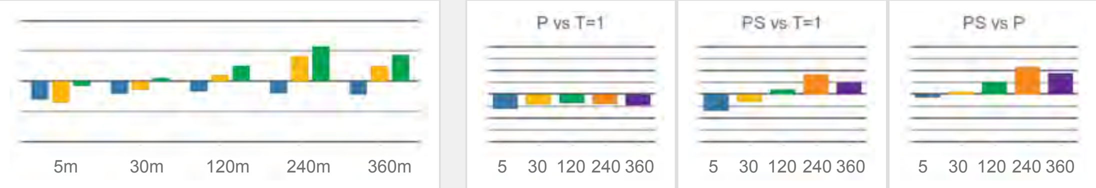

PgSg Time Course

log$_2$ (Fold Change)

Legend: P vs T=1 | PS vs T=1 | PS vs P

Locus		5m	30m	120m	240m	360m
**PGN_0610**	P vs T=1	1.526	2.079	2.281	1.862	0.949
	PS vs T=1	1.280	1.569	1.514	1.053	0.823
	PS vs P	-0.240	-0.468	-0.718	-0.783	-0.140
	conserved hypothetical protein with DUF1015 domain					
	hypothetical proteins-Conserved					
**PGN_0611**	P vs T=1	1.423	1.768	1.466	0.780	-0.145
	PS vs T=1	1.048	1.261	1.155	0.668	0.339
	PS vs P	-0.339	-0.439	-0.264	-0.112	0.438
	D-3-phosphoglycerate dehydrogenase					
	unknown function					
**PGN_0612**	P vs T=1	-0.500	-0.525	-0.394	-0.731	-1.441
	PS vs T=1	-1.023	-1.063	-1.169	-1.279	-1.251
	PS vs P	-0.490	-0.506	-0.730	-0.528	0.165
	phosphoserine aminotransferase					
	biosynthesis of cofactors, prosthetic groups, and carriers					
**PGN_0613**	P vs T=1	-0.749	-1.972	-2.679	-2.215	-1.770
ugdA	PS vs T=1	-1.585	-1.609	-1.313	-1.106	-1.285
	PS vs P	-0.732	0.297	1.171	1.014	0.468
	UDP-glucose 6-dehydrogenase					
	cell envelope					
**PGN_0614**	P vs T=1	0.156	0.678	1.458	1.946	1.947
	PS vs T=1	-0.079	0.077	0.344	0.623	0.728
	PS vs P	-0.275	-0.589	-1.053	-1.222	-1.155
	DNA-binding protein histone-like family					
	DNA metabolism					
**PGN_0615**	P vs T=1	1.897	2.235	2.756	3.188	3.441
	PS vs T=1	1.171	1.516	1.822	2.587	2.775
	PS vs P	-0.590	-0.489	-0.604	-0.138	-0.238
	hypothetical protein					
	hypothetical proteins					
**PGN_0616**	P vs T=1	0.652	0.882	0.900	0.821	0.298
	PS vs T=1	0.503	0.562	0.738	0.915	0.911
	PS vs P	-0.146	-0.287	-0.137	0.109	0.578
	probable elongation factor P					
	protein synthesis					

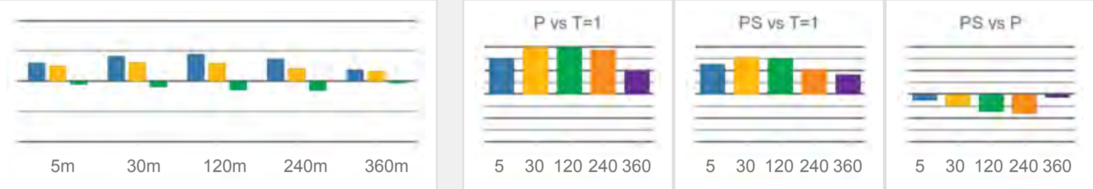

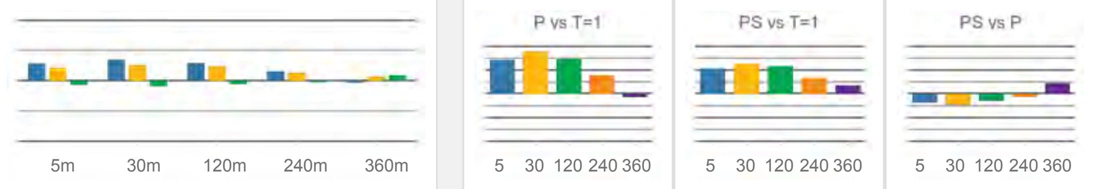

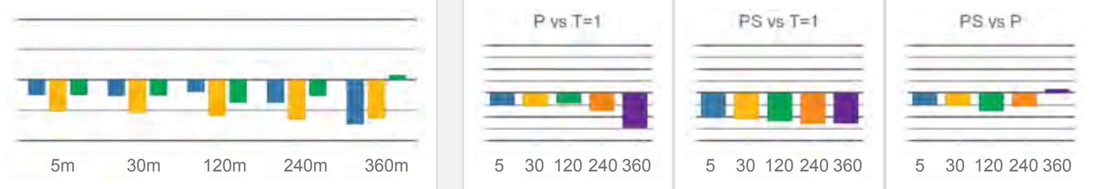

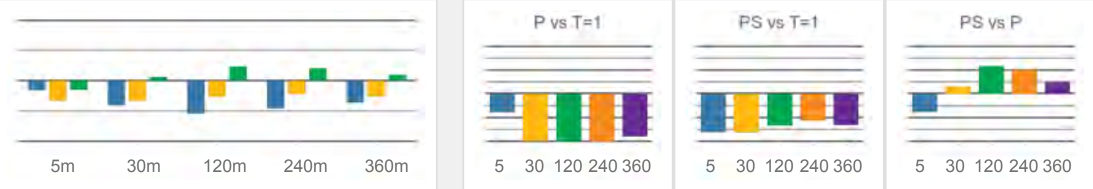

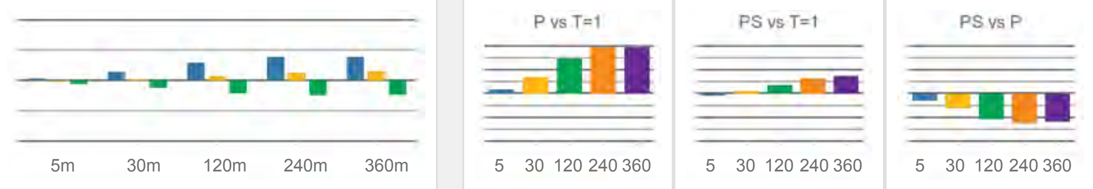

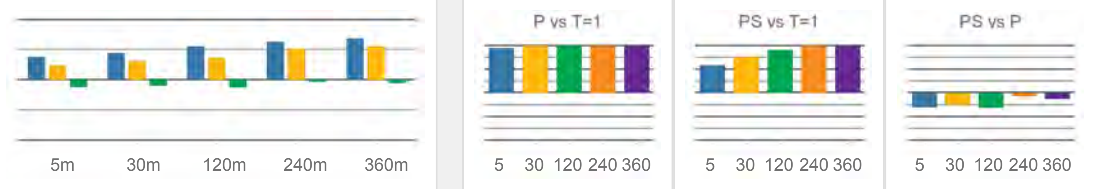

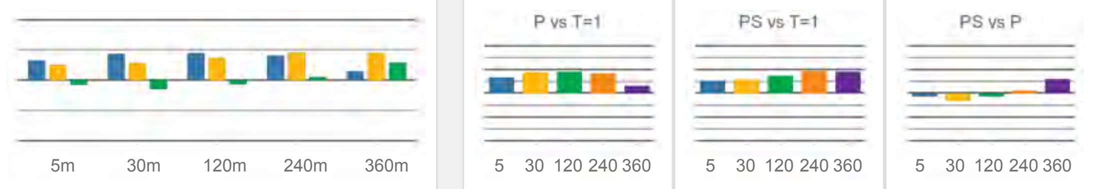

PgSg Time Course

log$_2$ (Fold Change)

Legend: P vs T=1, PS vs T=1, PS vs P

Locus		5m	30m	120m	240m	360m
**PGN_0617**	P vs T=1	-1.373	-1.252	-0.832	-0.355	0.237
	PS vs T=1	-1.069	-1.148	-0.965	-0.890	-0.605
	PS vs P	0.226	0.039	-0.153	-0.476	-0.760

conserved hypothetical protein

hypothetical proteins-Conserved

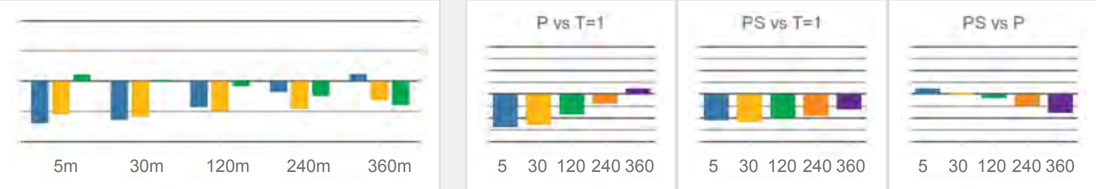

Locus		5m	30m	120m	240m	360m
**PGN_0618**	P vs T=1	0.987	1.038	0.877	0.499	0.116
	PS vs T=1	0.944	0.805	0.528	0.386	0.208
	PS vs P	-0.026	-0.208	-0.329	-0.122	0.067

aspartate-semialdehyde dehydrogenase

amino acid biosynthesis

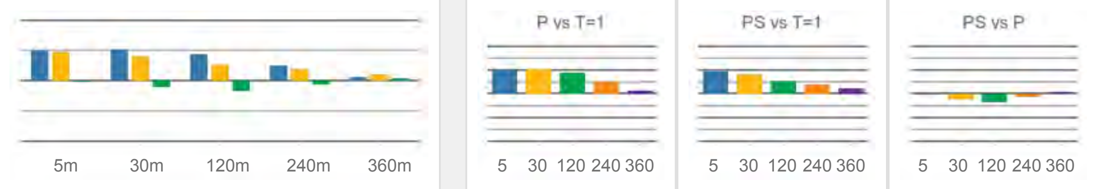

Locus		5m	30m	120m	240m	360m
**PGN_0619**	P vs T=1	0.301	0.170	0.178	-0.017	0.501
	PS vs T=1	-0.211	-0.079	-0.409	-0.307	-0.161
	PS vs P	-0.433	-0.220	-0.533	-0.365	-0.494

conserved hypothetical protein

hypothetical proteins-Conserved

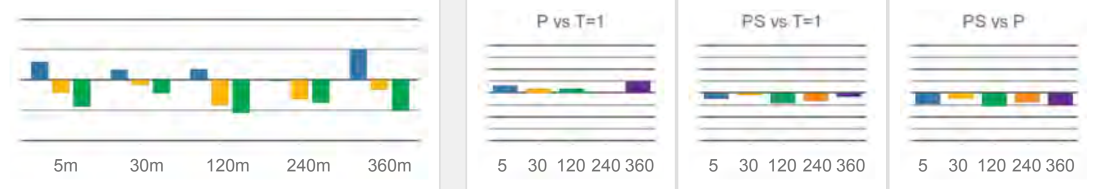

Locus		5m	30m	120m	240m	360m
**PGN_0620**	P vs T=1	0.003	-0.209	-0.628	-0.614	-0.432
	PS vs T=1	0.586	0.146	-0.203	-0.052	-0.306
	PS vs P	0.580	0.342	0.371	0.497	0.105

putative S-adenosyl-methyltransferase MraW

hypothetical proteins-Conserved

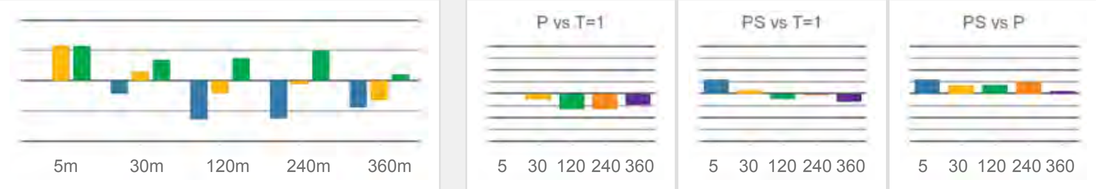

Locus		5m	30m	120m	240m	360m
**PGN_0621**	P vs T=1	-0.525	-0.649	-0.519	-0.782	-0.478
	PS vs T=1	-0.096	-0.480	-0.439	-0.463	-0.566
	PS vs P	0.390	0.104	0.059	0.170	-0.106

conserved hypothetical protein

hypothetical proteins-Conserved

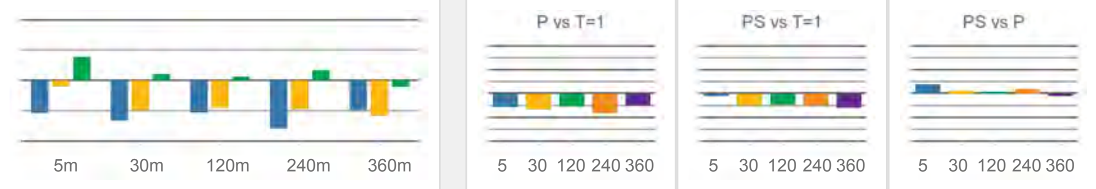

Locus		5m	30m	120m	240m	360m
**PGN_0622**	P vs T=1	-0.791	-0.859	-0.852	-0.810	-0.420
	PS vs T=1	-0.384	-0.815	-1.330	-1.478	-1.470
	PS vs P	0.403	0.039	-0.476	-0.657	-1.023

penicillin-binding protein

cell envelope

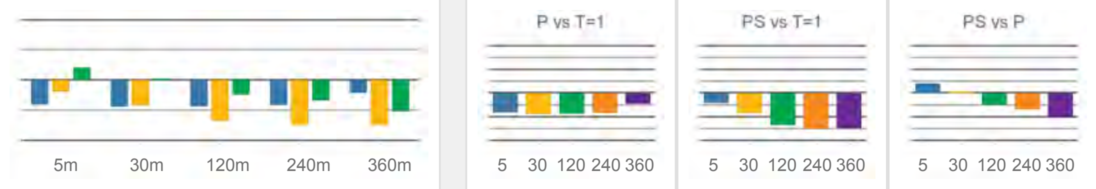

Locus		5m	30m	120m	240m	360m
**PGN_0623**	P vs T=1	-0.614	-0.910	-1.992	-2.299	-2.011
	PS vs T=1	-0.205	-0.603	-2.167	-2.763	-2.783
	PS vs P	0.434	0.324	-0.228	-0.560	-0.792

UDP-N-acetylmuramoylalanyl-D-glutamyl-2, 6-diaminopimelate ligase

cell envelope

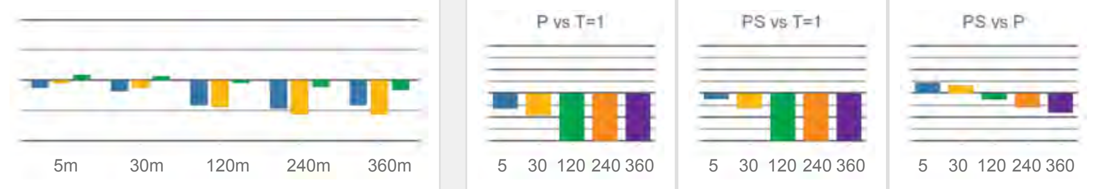

log2 (Fold Change)

Legend: P vs T=1 | PS vs T=1 | PS vs P

Locus		5m	30m	120m	240m	360m
**PGN_0624**	P vs T=1	0.329	0.259	-0.154	-0.372	-0.128
	PS vs T=1	0.873	0.629	-0.581	-1.100	-0.817
	PS vs P	0.555	0.382	-0.433	-0.750	-0.677

phospho-N-acetylmuramoyl-pentapeptide- transferase

cell envelope

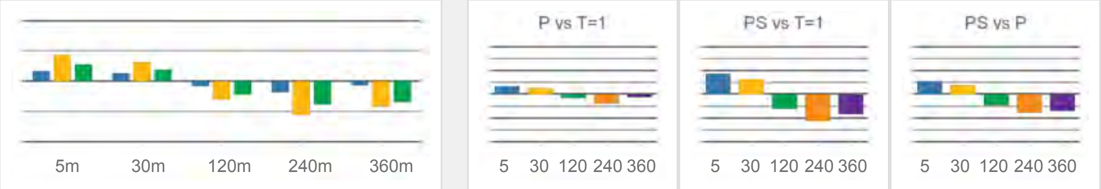

Locus		5m	30m	120m	240m	360m
**PGN_0625**	P vs T=1	-0.597	-0.987	-1.668	-1.950	-2.099
	PS vs T=1	-0.205	-0.299	-1.523	-2.054	-2.101
	PS vs P	0.410	0.683	0.107	-0.175	-0.059

UDP-N-acetylmuramoylalanine--D-glutamate ligase

cell envelope

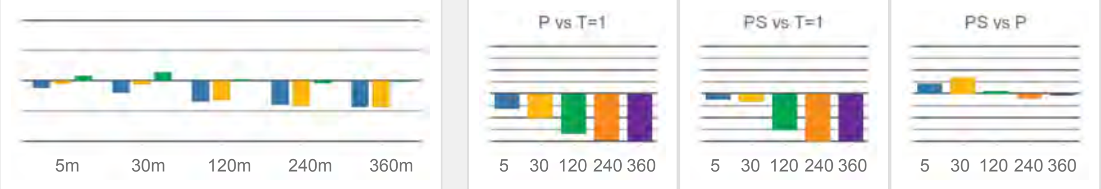

Locus		5m	30m	120m	240m	360m
**PGN_0626**	P vs T=1	-0.124	-0.660	-1.548	-1.750	-1.092
	PS vs T=1	0.353	0.276	-0.836	-1.470	-1.452
	PS vs P	0.516	0.919	0.593	0.106	-0.359

putative rod shape-determining protein RodA

cellular processes

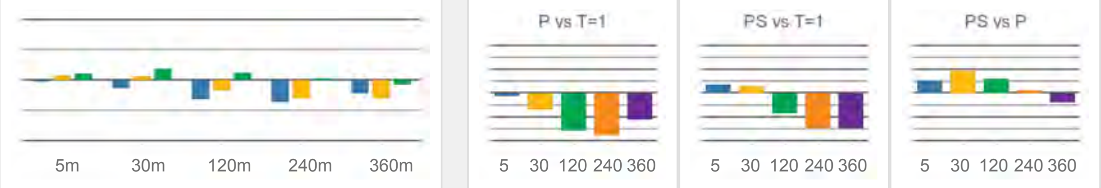

Locus		5m	30m	120m	240m	360m
**PGN_0627**	P vs T=1	0.136	-0.055	-0.580	-0.646	0.057
	PS vs T=1	0.815	0.728	-0.158	-0.409	-0.415
	PS vs P	0.676	0.762	0.331	0.116	-0.438

N-acetylglucosaminyl transferase

cell envelope

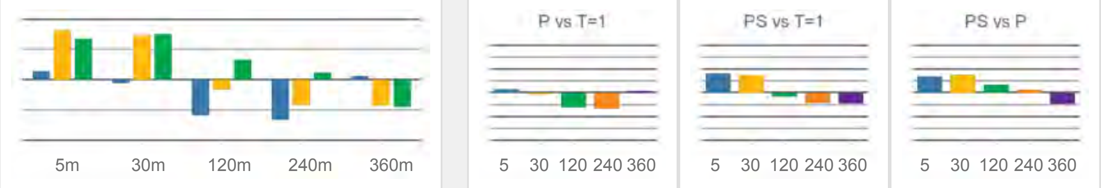

Locus		5m	30m	120m	240m	360m
**PGN_0628**	P vs T=1	-0.627	-0.769	-1.828	-1.455	-0.735
	PS vs T=1	-0.203	-0.393	-1.247	-1.675	-1.821
	PS vs P	0.431	0.384	0.410	-0.282	-1.031

UDP-N-acetylmuramate-alanine ligase

cell envelope

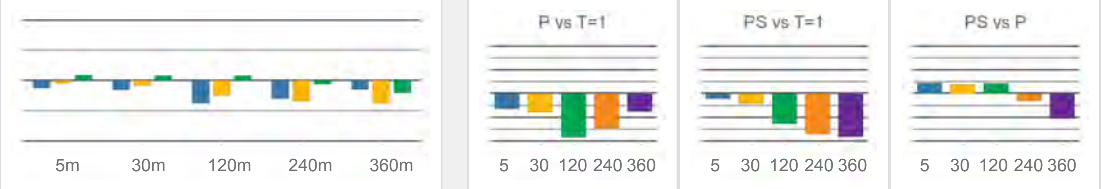

Locus		5m	30m	120m	240m	360m
**PGN_0629**	P vs T=1	0.490	-0.024	-0.465	-0.241	0.139
	PS vs T=1	1.035	0.726	-0.210	-0.402	-0.240
	PS vs P	0.582	0.711	0.148	-0.189	-0.336

putative cell division protein FtsQ

cellular processes

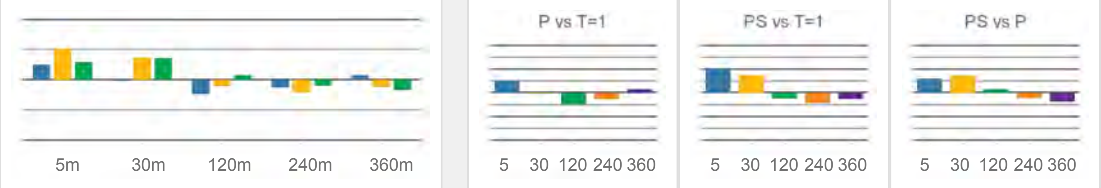

Locus		5m	30m	120m	240m	360m
**PGN_0630**	P vs T=1	-0.090	-0.235	-0.459	-0.896	-1.357
	PS vs T=1	0.532	0.159	-0.317	-0.760	-1.083
	PS vs P	0.623	0.389	0.140	0.101	0.227

probable cell division protein FtsA

cellular processes

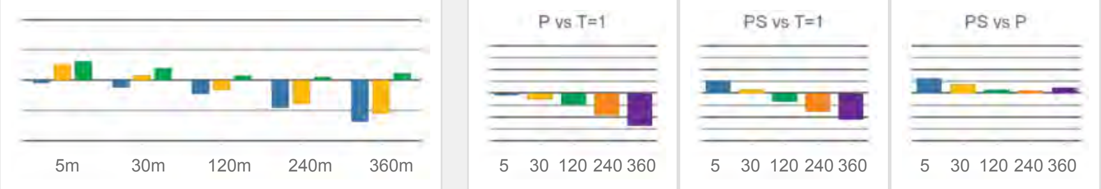

PgSg Time Course

log$_2$ (Fold Change)

P vs T=1 | PS vs T=1 | PS vs P

Locus		5m	30m	120m	240m	360m
**PGN_0631**	P vs T=1	-0.114	0.292	0.156	-0.460	-0.893
	PS vs T=1	0.748	0.858	0.297	-0.550	-0.966
	PS vs P	0.833	0.571	0.152	-0.123	-0.115

putative cell division protein FtsZ

cellular processes

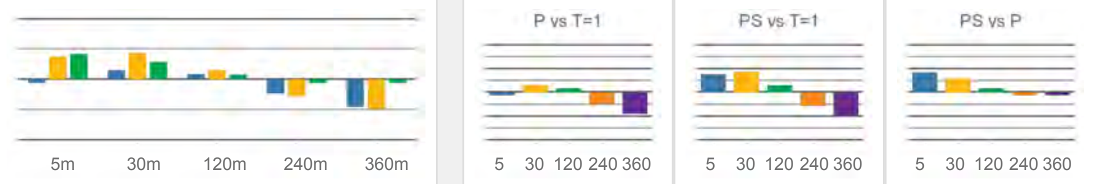

Locus		5m	30m	120m	240m	360m
**PGN_0632**	P vs T=1	0.212	0.879	1.154	0.934	0.284
	PS vs T=1	0.561	1.253	1.235	0.569	0.269
	PS vs P	0.303	0.380	0.109	-0.349	-0.037

conserved hypothetical protein

unknown function

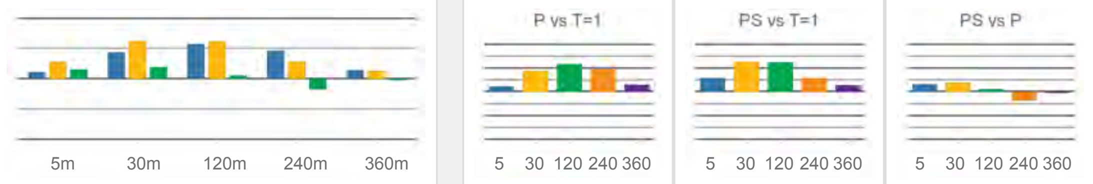

Locus		5m	30m	120m	240m	360m
**PGN_0633**	P vs T=1	-0.369	-0.666	-0.776	-0.822	0.073
	PS vs T=1	-0.121	-0.547	-0.728	-0.561	-0.267
	PS vs P	0.260	0.091	-0.003	0.154	-0.251

conserved hypothetical protein

hypothetical proteins-Conserved

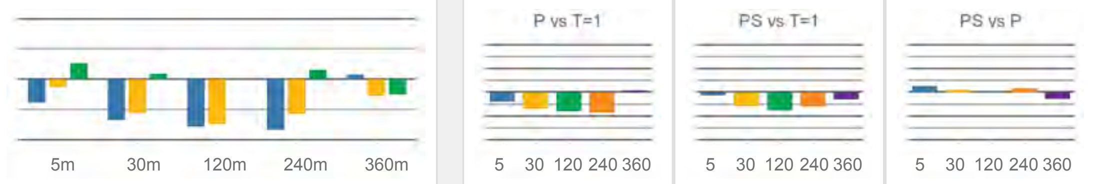

Locus		5m	30m	120m	240m	360m
**PGN_0634**	P vs T=1	-0.146	-0.007	0.007	0.001	0.377
	PS vs T=1	0.055	-0.281	-0.352	-0.453	-0.388
	PS vs P	0.189	-0.253	-0.336	-0.426	-0.690

3-methyl-2-oxobutanoate hydroxymethyltransferase

biosynthesis of cofactors, prosthetic groups, and carriers

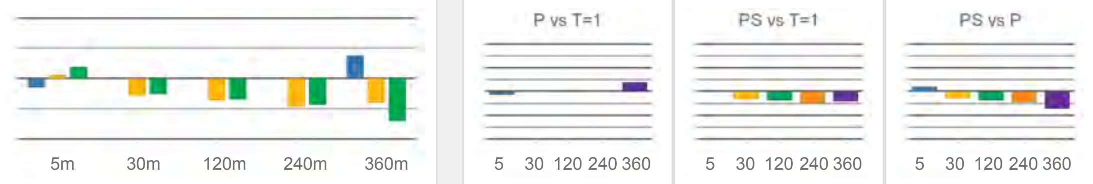

Locus		5m	30m	120m	240m	360m
**PGN_0635**	P vs T=1	-0.354	-0.214	-0.275	-0.475	-0.394
	PS vs T=1	-0.740	-1.004	-1.053	-0.515	-0.402
	PS vs P	-0.375	-0.761	-0.756	-0.044	-0.005

glutamine-hydrolyzing GMP synthase

purines, pyrimidines, nucleosides and nucleotides

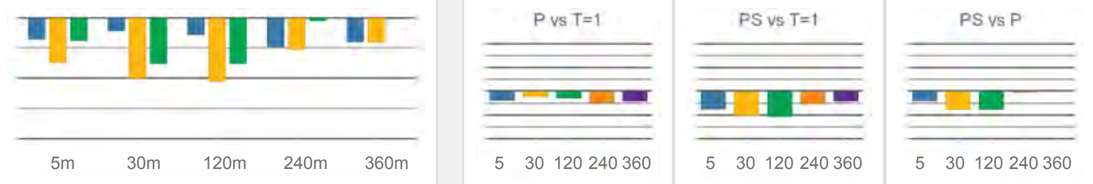

Locus		5m	30m	120m	240m	360m
**PGN_0636**	P vs T=1	-0.334	-0.742	-1.378	-1.623	-2.007
rpmE	PS vs T=1	-1.721	-1.536	-1.579	-1.549	-1.241
	PS vs P	-1.263	-0.723	-0.213	0.030	0.687

50S ribosomal protein L31

protein synthesis

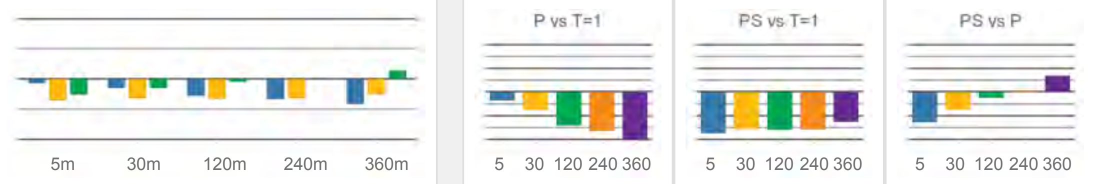

Locus		5m	30m	120m	240m	360m
**PGN_0637**	P vs T=1	1.518	2.221	2.723	2.943	2.673
	PS vs T=1	0.382	1.459	2.457	2.780	2.699
	PS vs P	-1.143	-0.699	-0.198	-0.088	0.060

putative heat shock-related protease htrA protein

protein fate

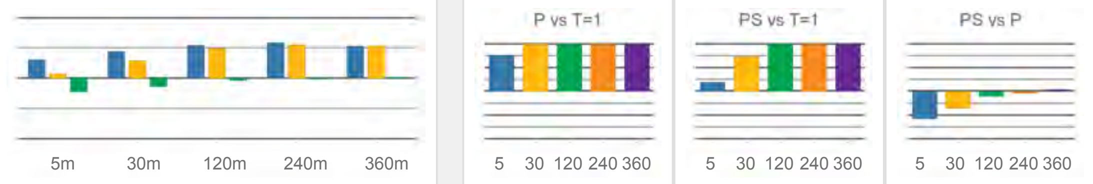

PgSg Time Course

Hackett Laboratory UW

log2 (Fold Change)

Legend: P vs T=1 | PS vs T=1 | PS vs P

PGN_0638 — *rpoD*

Locus	5m	30m	120m	240m	360m
P vs T=1	0.670	0.647	0.747	0.913	0.621
PS vs T=1	-0.103	0.436	1.297	1.442	1.184
PS vs P	-0.763	-0.208	0.549	0.536	0.557

RNA polymerase sigma factor RpoD

transcription

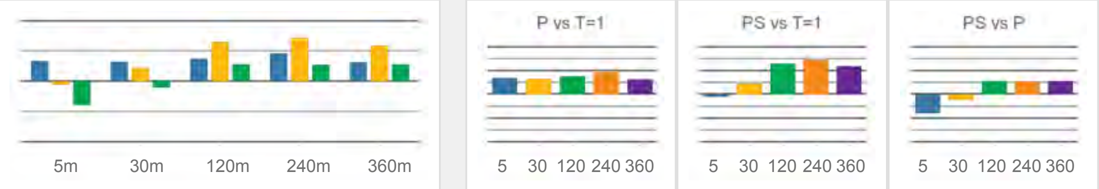

PGN_0639 — *rpsF*

Locus	5m	30m	120m	240m	360m
P vs T=1	0.592	1.097	1.656	1.666	1.114
PS vs T=1	0.054	0.823	1.715	1.869	1.787
PS vs P	-0.568	-0.249	0.122	0.252	0.664

30S ribosomal protein S6

protein synthesis

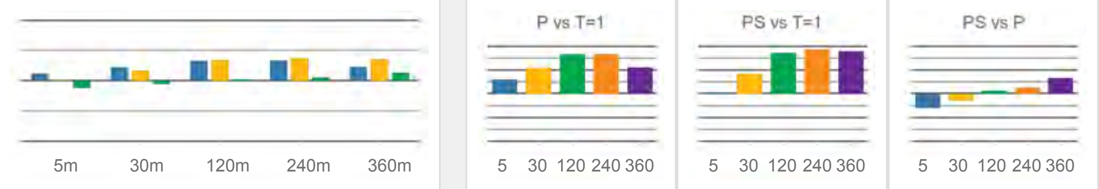

PGN_0640 — *rpsR*

Locus	5m	30m	120m	240m	360m
P vs T=1	0.605	0.998	1.689	1.869	1.327
PS vs T=1	0.023	0.724	1.814	1.939	1.822
PS vs P	-0.602	-0.267	0.172	0.122	0.497

30S ribosomal protein S18

protein synthesis

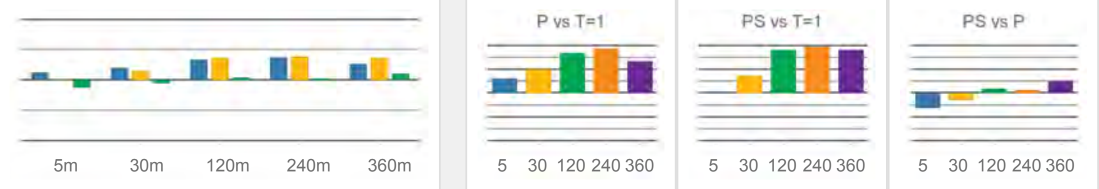

PGN_0641 — *rplI*

Locus	5m	30m	120m	240m	360m
P vs T=1	0.290	0.645	1.376	1.656	1.188
PS vs T=1	-0.240	0.332	1.180	1.228	1.283
PS vs P	-0.547	-0.309	-0.154	-0.371	0.108

50S ribosomal protein L9

protein synthesis

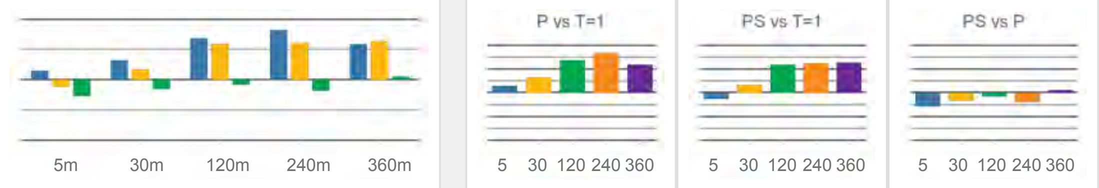

PGN_0642

Locus	5m	30m	120m	240m	360m
P vs T=1	-0.151	-0.021	0.096	0.134	0.297
PS vs T=1	0.068	0.122	0.495	0.977	0.658
PS vs P	0.202	0.132	0.387	0.824	0.358

conserved hypothetical protein

hypothetical proteins-Conserved

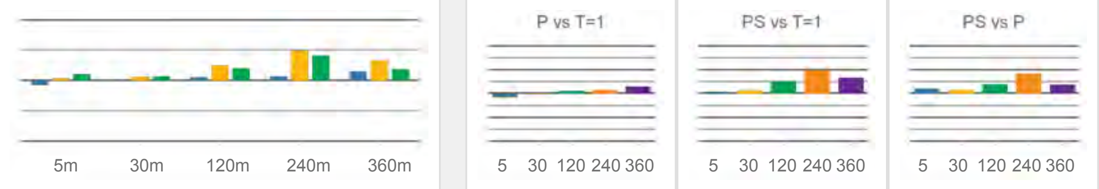

PGN_0643 — *RibA*

Locus	5m	30m	120m	240m	360m
P vs T=1	-0.106	0.546	1.325	1.550	1.137
PS vs T=1	0.617	0.893	1.343	1.555	1.465
PS vs P	0.676	0.332	0.033	0.026	0.327

3,4-dihydroxy-2-butanone 4-phosphate synthase

biosynthesis of cofactors, prosthetic groups, and carriers

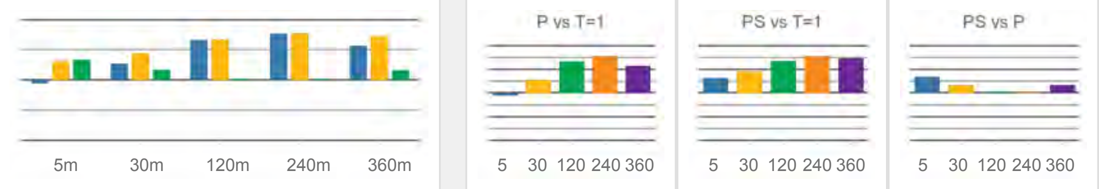

PGN_0644

Locus	5m	30m	120m	240m	360m
P vs T=1	0.443	0.362	0.456	0.624	0.244
PS vs T=1	0.236	0.058	0.229	0.323	0.346
PS vs P	-0.207	-0.310	-0.233	-0.293	0.085

transposase in ISPg1

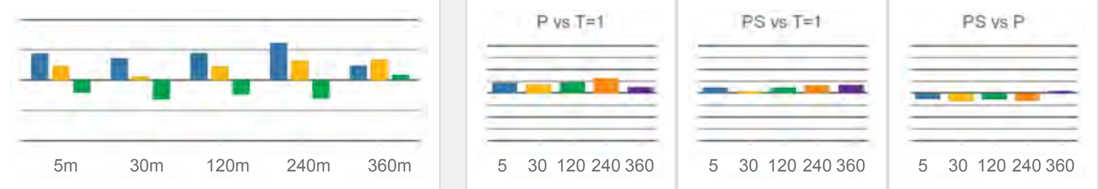

log$_2$ (Fold Change)

P vs T=1 | PS vs T=1 | PS vs P

PGN_0645
porQ

Locus		5m	30m	120m	240m	360m
PGN_0645	P vs T=1	0.250	0.052	-0.387	-0.568	-0.990
	PS vs T=1	0.323	0.209	0.253	0.418	-0.162
	PS vs P	0.090	0.159	0.605	0.933	0.761

Por secretion system protein porQ

hypothetical proteins

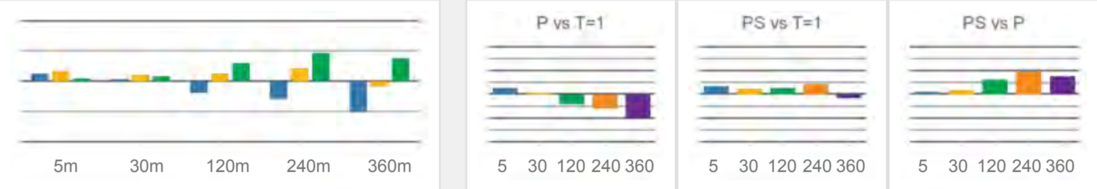

PGN_0646

Locus		5m	30m	120m	240m	360m
PGN_0646	P vs T=1	-0.280	-0.227	-0.739	-1.057	-1.393
	PS vs T=1	-0.162	-0.260	-0.489	-0.692	-1.057
	PS vs P	0.127	-0.010	0.236	0.308	0.268

probable cytidylate kinase

purines, pyrimidines, nucleosides and nucleotides

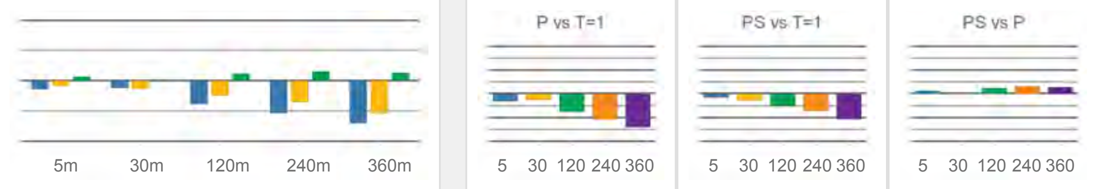

PGN_0647

Locus		5m	30m	120m	240m	360m
PGN_0647	P vs T=1	-0.391	-0.473	-0.520	-0.490	-0.420
	PS vs T=1	0.023	-0.274	-0.689	-0.727	-0.602
	PS vs P	0.406	0.188	-0.182	-0.246	-0.185

hydroxymethylbutenyl pyrophosphate reductase

cellular processes

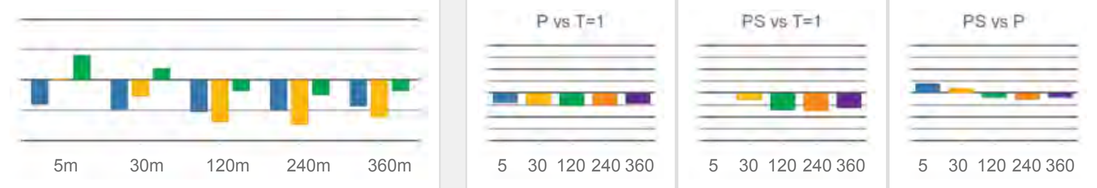

PGN_0648

Locus		5m	30m	120m	240m	360m
PGN_0648	P vs T=1	-0.666	-0.265	0.216	0.409	0.106
	PS vs T=1	-0.266	-0.343	-0.238	0.176	0.291
	PS vs P	0.315	-0.101	-0.424	-0.184	0.177

conserved hypothetical protein

hypothetical proteins-Conserved

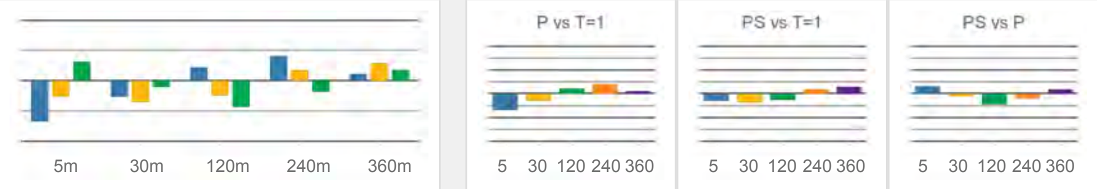

PGN_0649

Locus		5m	30m	120m	240m	360m
PGN_0649	P vs T=1	0.205	-0.055	-0.084	-0.508	-1.408
	PS vs T=1	-1.083	-1.468	-2.009	-2.047	-1.808
	PS vs P	-1.082	-1.242	-1.743	-1.414	-0.474

conserved hypothetical protein

hypothetical proteins-Conserved

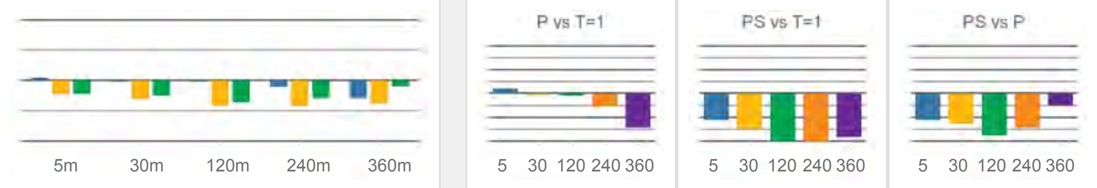

PGN_0650

Locus		5m	30m	120m	240m	360m
PGN_0650	P vs T=1	0.724	1.183	2.118	2.423	1.816
	PS vs T=1	-0.793	-0.609	-0.533	-0.188	-0.029
	PS vs P	-1.471	-1.704	-2.513	-2.456	-1.776

conserved hypothetical protein

hypothetical proteins-Conserved

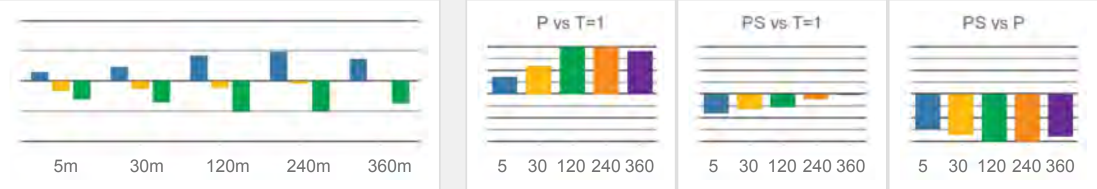

PGN_0651

Locus		5m	30m	120m	240m	360m
PGN_0651	P vs T=1	1.140	1.383	1.192	1.182	1.505
	PS vs T=1	0.143	1.069	1.179	1.294	1.440
	PS vs P	-0.798	-0.110	0.049	0.155	0.151

conserved hypothetical protein

hypothetical proteins-Conserved

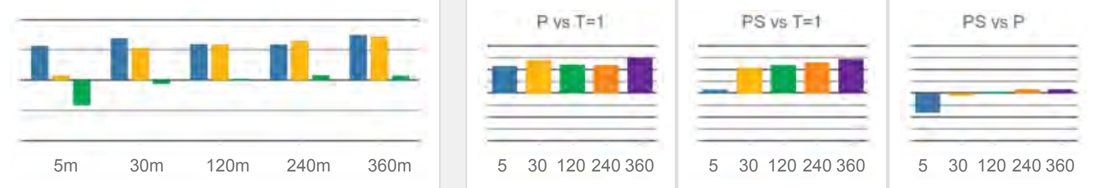

PgSg Time Course

log2 (Fold Change)

Legend: P vs T=1 | PS vs T=1 | PS vs P

PGN_0652

Locus	5m	30m	120m	240m	360m
P vs T=1	-0.214	-0.314	0.505	1.123	1.262
PS vs T=1	-2.297	-1.519	-0.698	-0.348	0.259
PS vs P	-1.874	-1.113	-0.983	-1.032	-0.743

conserved hypothetical protein

hypothetical proteins-Conserved

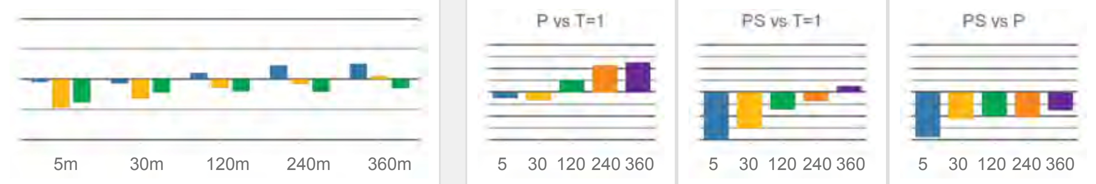

PGN_0653

Locus	5m	30m	120m	240m	360m
P vs T=1	1.790	2.092	2.180	1.600	0.883
PS vs T=1	1.343	1.751	1.653	1.114	1.025
PS vs P	-0.387	-0.239	-0.401	-0.445	0.083

conserved hypothetical protein

hypothetical proteins-Conserved

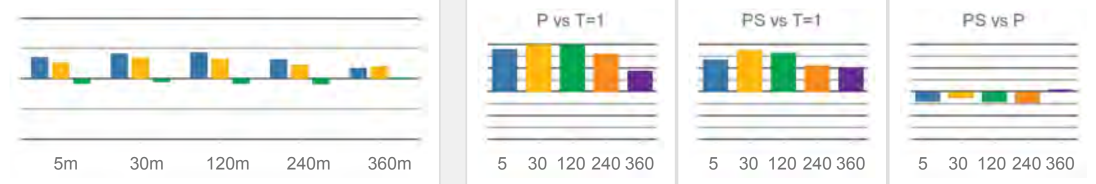

PGN_0654

Locus	5m	30m	120m	240m	360m
P vs T=1	0.903	1.427	1.367	0.532	-0.580
PS vs T=1	0.743	1.157	1.094	0.363	-0.115
PS vs P	-0.159	-0.205	-0.210	-0.188	0.376

conserved hypothetical protein

hypothetical proteins-Conserved

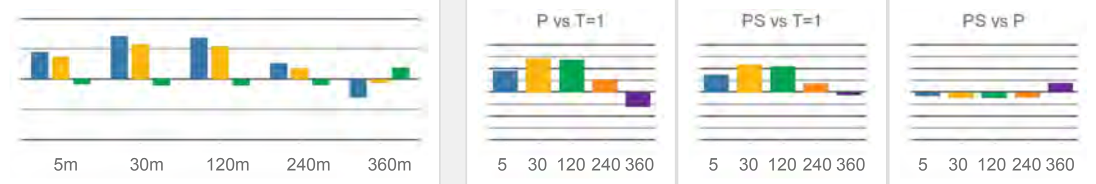

PGN_0655

Locus	5m	30m	120m	240m	360m
P vs T=1	1.984	2.410	1.961	1.149	0.450
PS vs T=1	1.874	2.264	1.871	0.946	0.619
PS vs P	-0.093	-0.091	-0.057	-0.249	0.085

conserved hypothetical protein

hypothetical proteins-Conserved

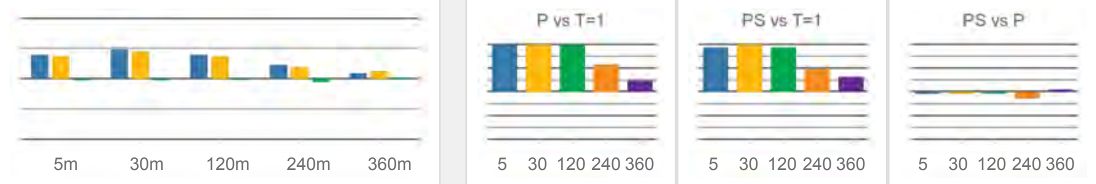

PGN_0656

Locus	5m	30m	120m	240m	360m
P vs T=1	1.415	1.889	1.663	0.926	0.178
PS vs T=1	1.707	2.099	1.623	0.808	0.474
PS vs P	0.282	0.265	0.006	-0.154	0.217

conserved hypothetical protein

hypothetical proteins-Conserved

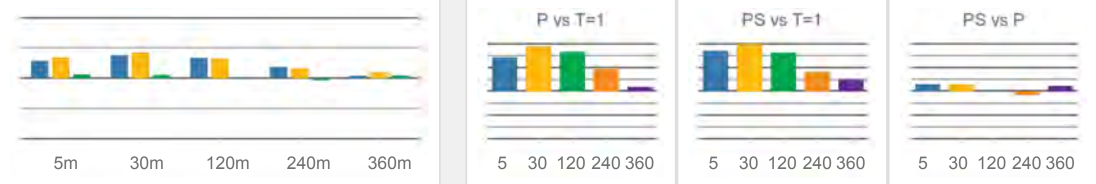

PGN_0657

Locus	5m	30m	120m	240m	360m
P vs T=1	-0.024	0.627	0.846	0.245	-0.734
PS vs T=1	-0.055	0.325	0.179	-0.609	-0.931
PS vs P	-0.055	-0.268	-0.615	-0.842	-0.238

conserved hypothetical protein

hypothetical proteins-Conserved

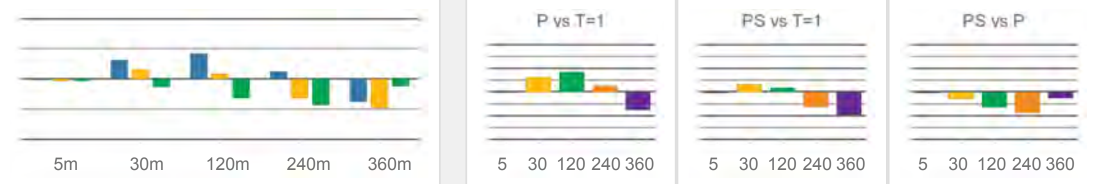

PGN_0658

Locus	5m	30m	120m	240m	360m
P vs T=1	0.216	0.141	-0.095	0.133	0.393
PS vs T=1	0.080	-0.280	-0.592	-0.445	-0.360
PS vs P	-0.107	-0.390	-0.497	-0.534	-0.704

GTP-binding elongation factor family protein TypA/BipA

unknown function

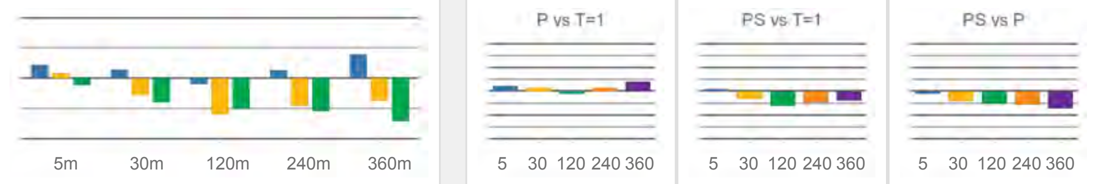

log$_2$ (Fold Change)

P vs T=1 | PS vs T=1 | PS vs P

Locus		5m	30m	120m	240m	360m
**PGN_0659**	P vs T=1	-1.413	-1.818	-1.688	-2.032	-2.798
	PS vs T=1	-1.859	-1.770	-1.640	-1.643	-1.762
	PS vs P	-0.378	0.019	0.077	0.353	0.905

35 kDa hemin binding protein

energy metabolism

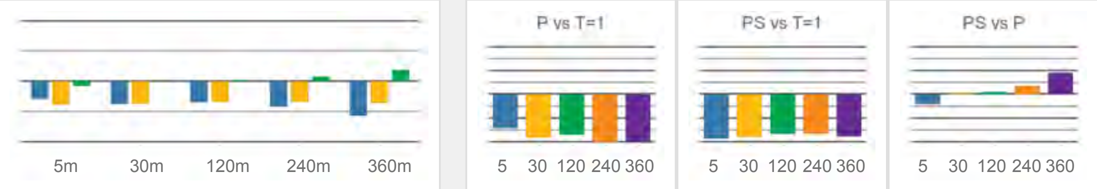

Locus		5m	30m	120m	240m	360m
**PGN_0660**	P vs T=1	4.924	5.326	5.907	5.886	4.977
	PS vs T=1	3.733	4.696	4.483	4.020	3.802
	PS vs P	-1.119	-0.541	-1.303	-1.747	-1.136

putative alkyl hydroperoxide reductase C subunit

cellular processes

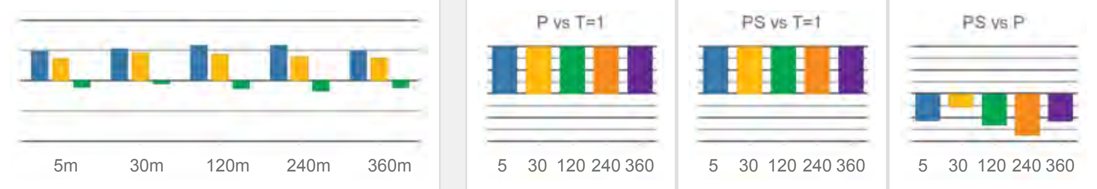

Locus		5m	30m	120m	240m	360m
**PGN_0661**	P vs T=1	4.741	5.224	5.219	4.675	3.677
	PS vs T=1	4.033	4.848	3.910	3.207	2.849
	PS vs P	-0.560	-0.166	-1.096	-1.326	-0.816

alkyl hydroperoxide reductase F subunit

cellular processes

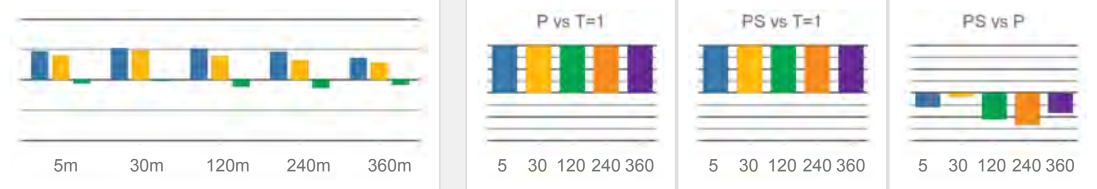

Locus		5m	30m	120m	240m	360m
**PGN_0662** *serB*	P vs T=1	0.671	0.636	0.142	-0.211	-0.728
	PS vs T=1	0.686	0.354	-0.370	-0.634	-0.689
	PS vs P	0.031	-0.265	-0.505	-0.424	0.025

HAD family serine phosphatase

cellular processes

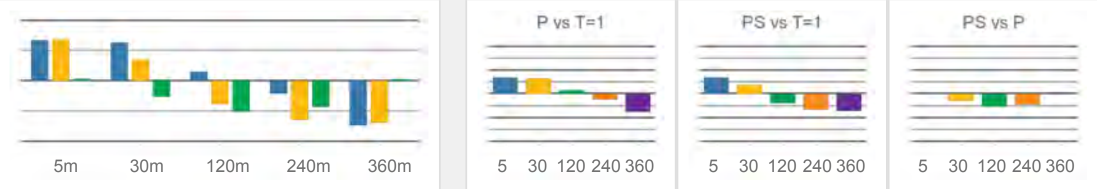

Locus		5m	30m	120m	240m	360m
**PGN_0663**	P vs T=1	-0.497	-0.495	-1.121	-1.525	-1.936
	PS vs T=1	-0.572	-0.775	-1.478	-1.935	-2.019
	PS vs P	-0.040	-0.230	-0.355	-0.449	-0.151

conserved hypothetical protein

hypothetical proteins-Conserved

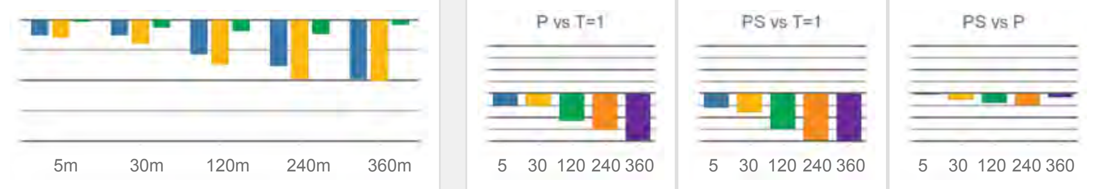

Locus		5m	30m	120m	240m	360m
**PGN_0664**	P vs T=1	0.515	0.015	-1.329	-1.891	-1.764
	PS vs T=1	0.788	0.216	-1.115	-1.939	-2.018
	PS vs P	0.319	0.230	0.180	-0.140	-0.287

conserved hypothetical protein

hypothetical proteins-Conserved

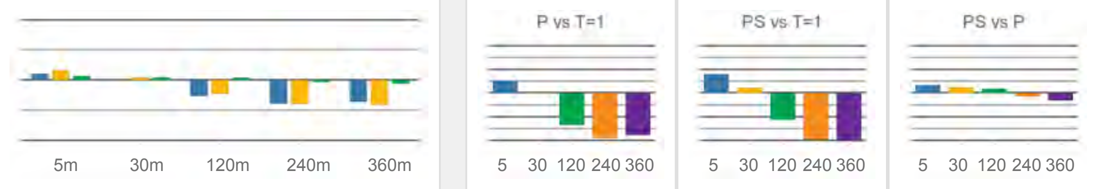

Locus		5m	30m	120m	240m	360m
**PGN_0665**	P vs T=1	0.622	0.957	0.644	-0.065	-0.890
	PS vs T=1	1.411	1.512	1.290	0.778	0.416
	PS vs P	0.774	0.564	0.649	0.797	1.232

putative triosephosphate isomerase

energy metabolism

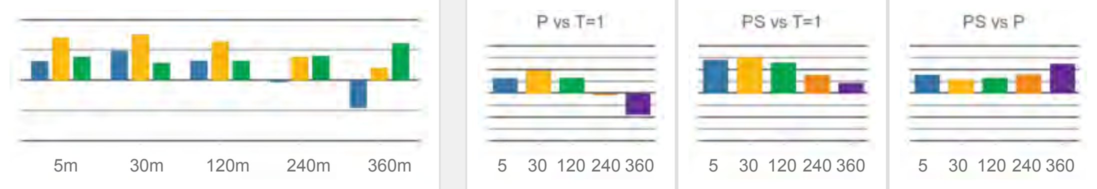

PgSg Time Course

log$_2$ (Fold Change)

Legend: P vs T=1 | PS vs T=1 | PS vs P

Locus		5m	30m	120m	240m	360m
**PGN_0666**	P vs T=1	1.934	2.375	2.229	1.498	0.764
	PS vs T=1	2.901	3.113	3.147	2.758	2.418
	PS vs P	0.914	0.754	0.934	1.162	1.519

conserved hypothetical protein

hypothetical proteins-Conserved

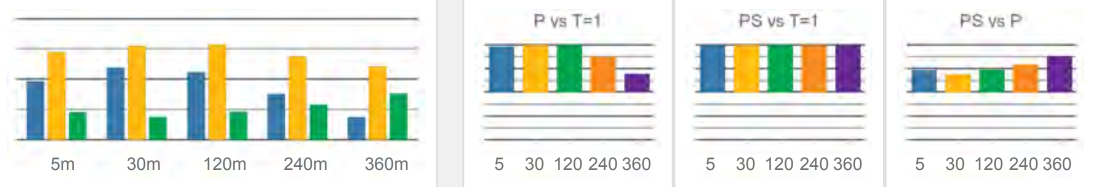

Locus		5m	30m	120m	240m	360m
**PGN_0667**	P vs T=1	1.041	1.611	1.960	1.657	1.102
	PS vs T=1	1.900	2.253	2.411	2.298	2.260
	PS vs P	0.814	0.634	0.464	0.630	1.128

putative GTP cyclohydrolase I

biosynthesis of cofactors, prosthetic groups, and carriers

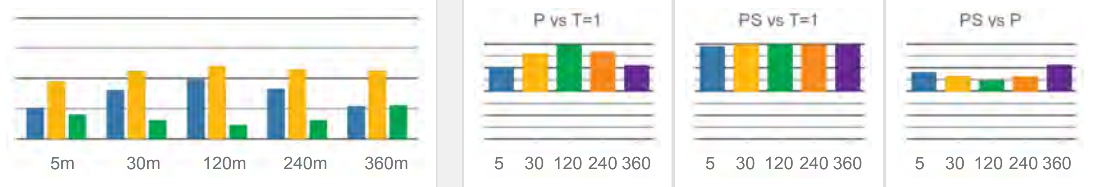

Locus		5m	30m	120m	240m	360m
**PGN_0668**	P vs T=1	0.317	0.313	0.261	0.268	-0.359
	PS vs T=1	-0.524	-0.202	-0.484	-0.491	-0.645
	PS vs P	-0.643	-0.336	-0.587	-0.578	-0.282

RNA-binding protein

transcription

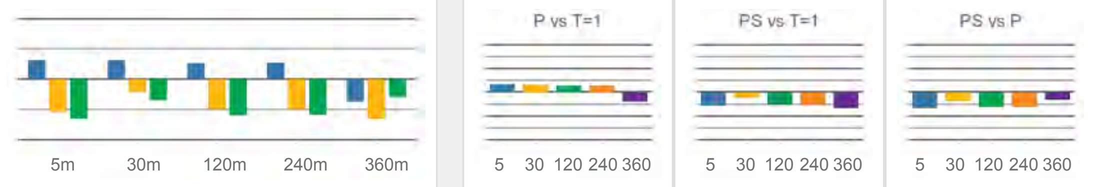

Locus		5m	30m	120m	240m	360m
**PGN_0669**	P vs T=1	0.034	0.380	0.377	0.169	0.551
	PS vs T=1	0.428	0.645	0.707	0.751	0.498
	PS vs P	0.338	0.264	0.327	0.500	-0.028

ABC transporter ATP-binding protein

transport and binding proteins

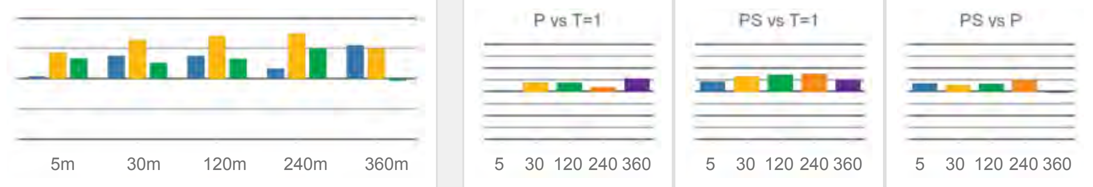

Locus		5m	30m	120m	240m	360m
**PGN_0670**	P vs T=1	-0.542	-0.609	-0.528	0.218	0.139
	PS vs T=1	-0.642	-0.553	-0.334	0.194	0.105
	PS vs P	-0.125	0.010	0.113	0.067	-0.007

probable inorganic polyphosphate/ATP-NAD kinase

hypothetical proteins-Conserved

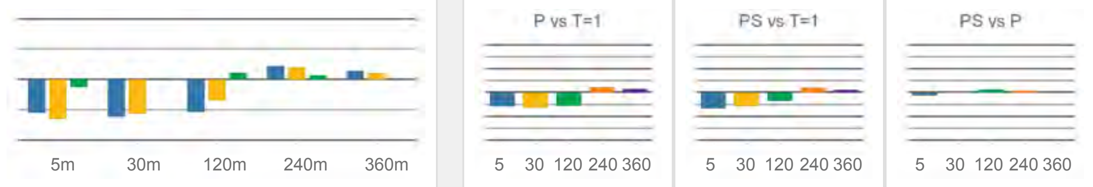

Locus		5m	30m	120m	240m	360m
**PGN_0671**	P vs T=1	1.370	1.155	0.910	0.202	-0.053
	PS vs T=1	0.849	1.379	1.787	1.684	1.347
	PS vs P	-0.446	0.249	0.880	1.326	1.282

putative pyridoxal phosphate biosynthetic protein

biosynthesis of cofactors, prosthetic groups, and carriers

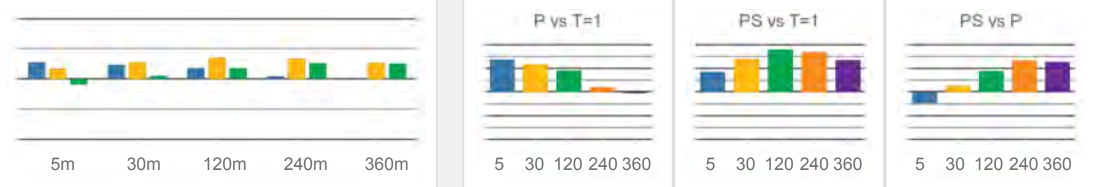

Locus		5m	30m	120m	240m	360m
**PGN_0672**	P vs T=1	0.482	0.974	1.164	0.795	0.535
	PS vs T=1	0.398	0.386	0.476	0.851	0.732
	PS vs P	-0.097	-0.535	-0.619	0.067	0.185

probable biopolymer transport protein

transport and binding proteins

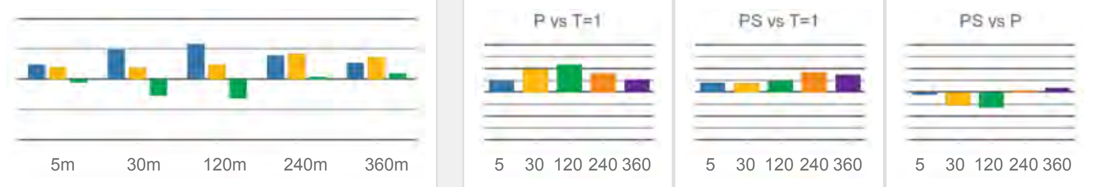

PgSg Time Course

log$_2$ (Fold Change)

Legend: P vs T=1, PS vs T=1, PS vs P

Locus		5m	30m	120m	240m	360m
**PGN_0673**	P vs T=1	0.723	1.371	2.114	2.318	2.033
	PS vs T=1	0.667	0.848	1.377	1.819	1.809
	PS vs P	-0.139	-0.476	-0.602	-0.342	-0.154

probable biopolymer transport protein

transport and binding proteins

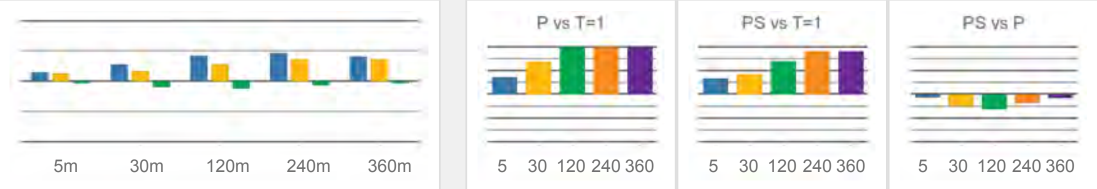

Locus		5m	30m	120m	240m	360m
**PGN_0674**	P vs T=1	0.388	0.658	1.513	1.687	1.221
	PS vs T=1	0.988	1.002	1.373	1.599	1.332
	PS vs P	0.485	0.275	-0.047	0.025	0.125

conserved hypothetical protein

hypothetical proteins-Conserved

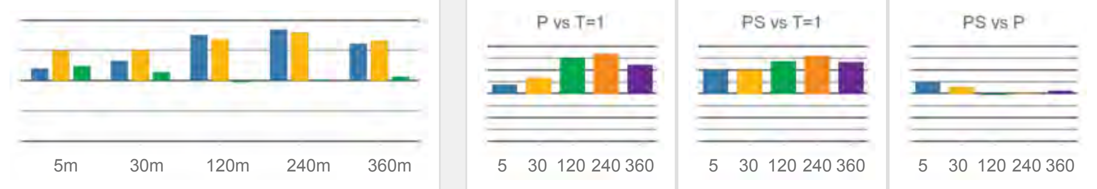

Locus		5m	30m	120m	240m	360m
**PGN_0675**	P vs T=1	-0.140	0.557	1.557	1.826	1.362
	PS vs T=1	0.165	0.543	1.000	1.141	1.149
	PS vs P	0.236	-0.022	-0.493	-0.601	-0.188

conserved hypothetical protein

unknown function

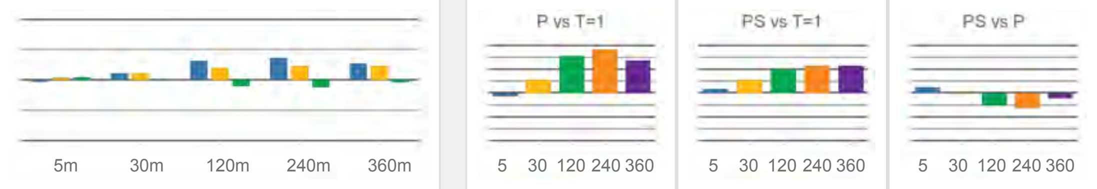

Locus		5m	30m	120m	240m	360m
**PGN_0676**	P vs T=1	0.749	0.941	1.023	1.214	0.788
	PS vs T=1	0.990	1.094	1.064	1.043	0.705
	PS vs P	0.234	0.159	0.053	-0.129	-0.083

putative ribosomal protein L11 methyltransferase

protein synthesis

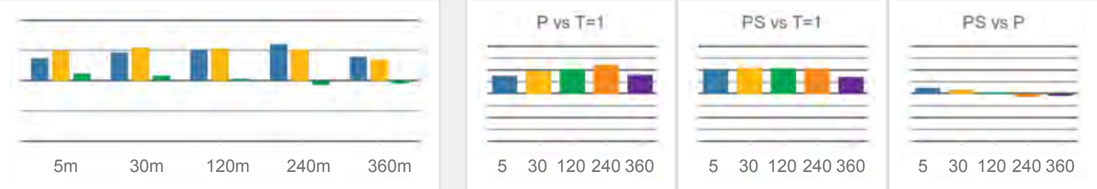

Locus		5m	30m	120m	240m	360m
**PGN_0677**	P vs T=1	0.370	0.072	-0.303	-0.041	0.556
	PS vs T=1	0.867	0.439	-0.220	-0.665	-0.293
	PS vs P	0.510	0.360	0.030	-0.610	-0.786

putative multi antimicrobial extrusion protein MatE

transport and binding proteins

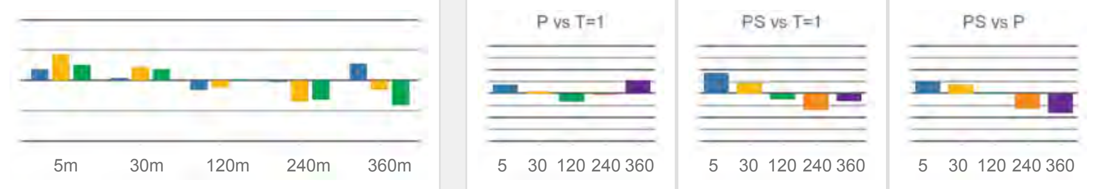

Locus		5m	30m	120m	240m	360m
**PGN_0678** *ThiL*	P vs T=1	1.176	1.036	0.574	0.327	0.036
	PS vs T=1	1.475	1.174	0.312	0.310	-0.171
	PS vs P	0.327	0.161	-0.264	-0.046	-0.246

thiamine monophosphate kinase

biosynthesis of cofactors, prosthetic groups, and carriers

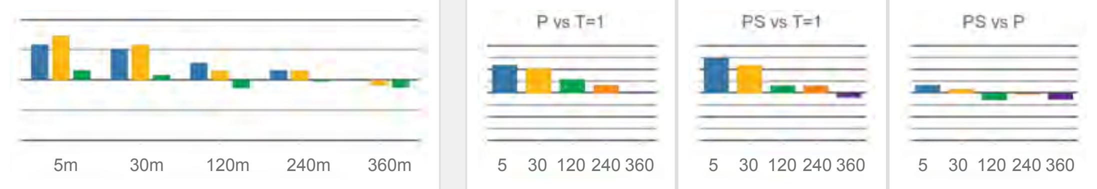

Locus		5m	30m	120m	240m	360m
**PGN_0679**	P vs T=1	0.810	0.685	-0.117	-0.413	-0.372
	PS vs T=1	1.017	0.501	-0.109	0.072	-0.417
	PS vs P	0.249	-0.139	-0.032	0.392	-0.084

putative tetraacyldisaccharide 4'-kinase

cell envelope

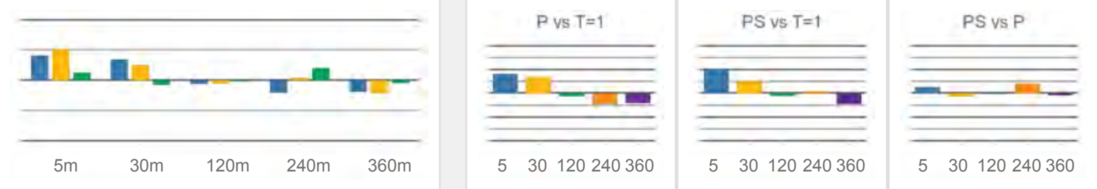

PgSg Time Course

log$_2$ (Fold Change)

Legend: P vs T=1 · PS vs T=1 · PS vs P

Locus		5m	30m	120m	240m	360m
**PGN_0680**	P vs T=1	0.966	0.572	-0.302	-0.971	-1.473
	PS vs T=1	0.576	0.256	-0.205	-0.200	-0.693
	PS vs P	-0.323	-0.274	0.087	0.704	0.710
	signal peptide peptidase SppA 67K type					
	protein fate					
**PGN_0681**	P vs T=1	0.453	-1.732	-0.401	0.795	2.600
	PS vs T=1	0.308	0.431	1.046	0.330	0.621
	PS vs P	0.050	0.303	0.702	-0.086	-0.380
	hypothetical protein					
	hypothetical proteins					
**PGN_0682**	P vs T=1	-1.486	-0.488	0.564	0.924	0.802
	PS vs T=1	-1.467	-0.981	-0.267	-0.051	0.116
	PS vs P	-0.216	-0.516	-0.725	-0.810	-0.610
	hypothetical protein					
	hypothetical proteins					
**PGN_0683**	P vs T=1	1.278	1.901	2.683	2.621	2.090
	PS vs T=1	1.719	2.114	2.344	2.015	1.776
	PS vs P	0.321	0.212	-0.209	-0.498	-0.297
	TonB-linked receptor Tlr					
	transport and binding proteins					
**PGN_0684**	P vs T=1	-0.300	-0.234	-0.512	-0.389	-0.297
	PS vs T=1	-0.036	0.015	-0.111	-0.201	-0.391
	PS vs P	0.249	0.243	0.351	0.159	-0.102
	conserved hypothetical protein with adenosylcobinamide amidohydrolase domain					
	hypothetical proteins-Conserved					
**PGN_0685**	P vs T=1	-0.926	-1.029	-1.300	-1.088	-0.984
	PS vs T=1	-0.751	-0.917	-1.128	-1.514	-1.341
	PS vs P	0.172	0.103	0.118	-0.426	-0.354
	putative iron compound ABC transporter ATP-binding protein					
	transport and binding proteins					
**PGN_0686**	P vs T=1	-0.233	-0.243	-0.229	-0.226	0.222
	PS vs T=1	-0.363	-0.833	-1.226	-1.019	-0.483
	PS vs P	-0.121	-0.571	-0.971	-0.766	-0.647
	putative iron compound ABC transporter permease protein					
	transport and binding proteins					

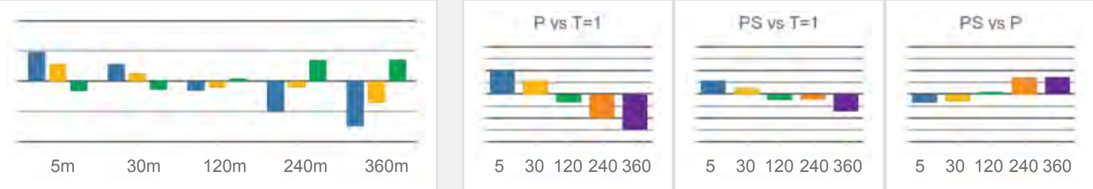

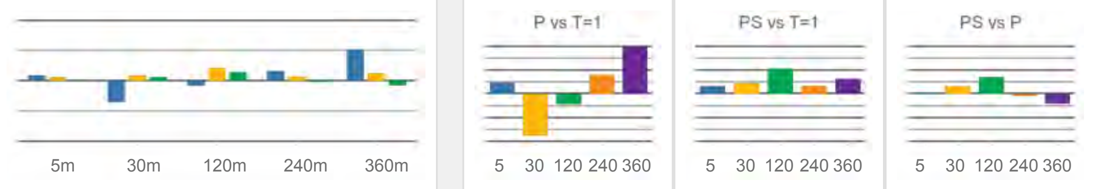

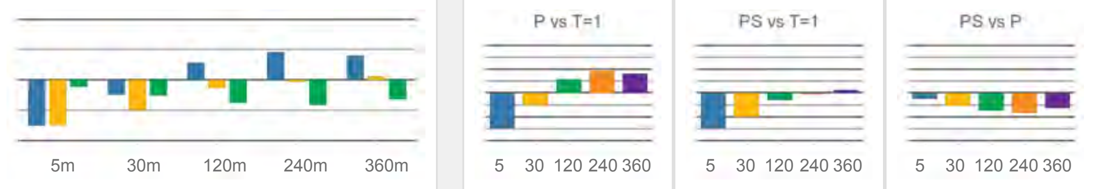

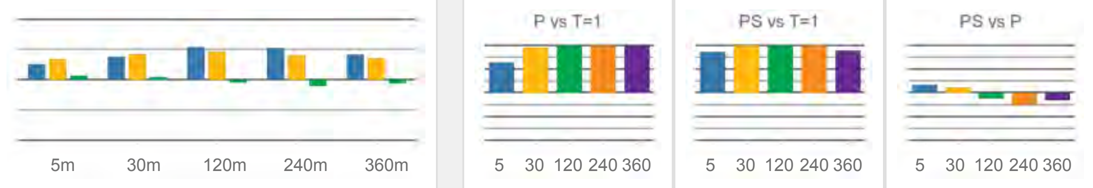

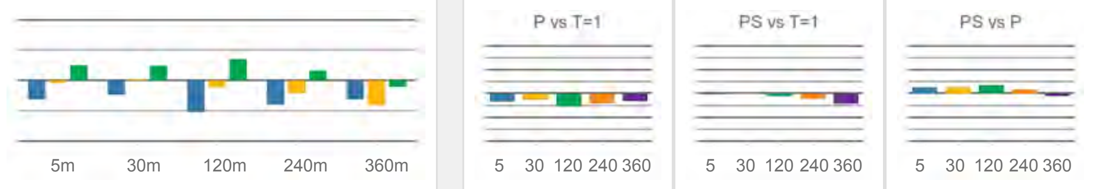

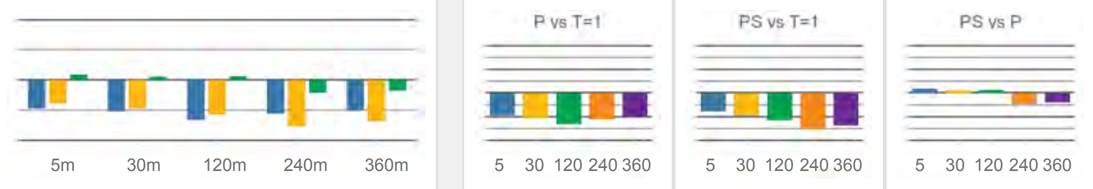

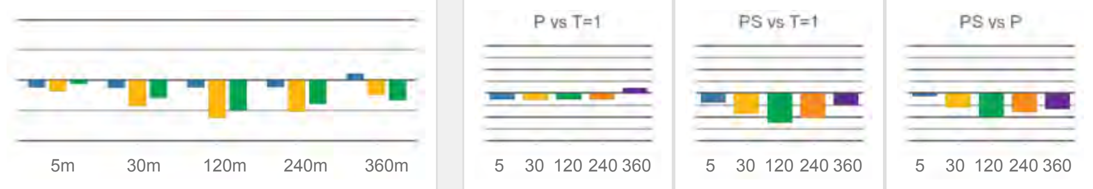

log2 (Fold Change)

Locus		5m	30m	120m	240m	360m
**PGN_0687**	P vs T=1	-0.622	-0.855	-1.006	-0.805	-0.670
	PS vs T=1	-0.465	-1.199	-1.396	-1.321	-0.962
	PS vs P	0.163	-0.348	-0.412	-0.510	-0.287

putative iron compound ABC transporter periplasmic iron compound-binding protein

transport and binding proteins

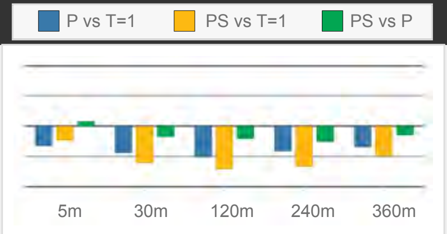

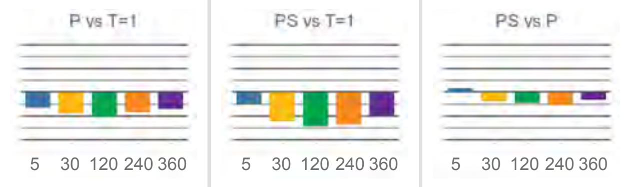

Locus		5m	30m	120m	240m	360m
**PGN_0688**	P vs T=1	-0.489	-0.642	-0.835	-0.048	0.326
	PS vs T=1	-0.980	-1.165	-0.553	0.058	0.419
	PS vs P	-0.467	-0.521	0.098	0.222	0.199

conserved hypothetical protein

hypothetical proteins-Conserved

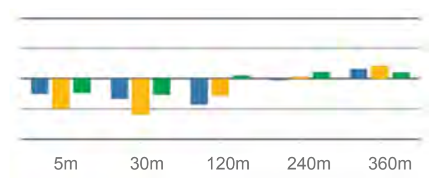

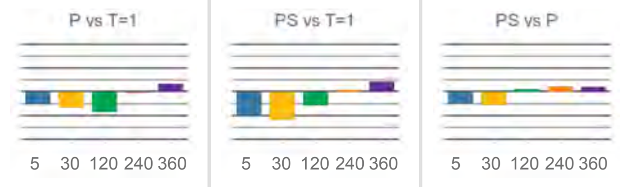

Locus		5m	30m	120m	240m	360m
**PGN_0689**	P vs T=1	-0.634	-1.302	-0.987	-0.267	0.609
	PS vs T=1	-1.615	-1.162	-0.399	-0.073	0.340
	PS vs P	-0.907	-0.033	0.425	0.297	-0.067

conserved hypothetical protein

hypothetical proteins-Conserved

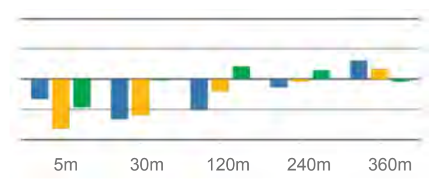

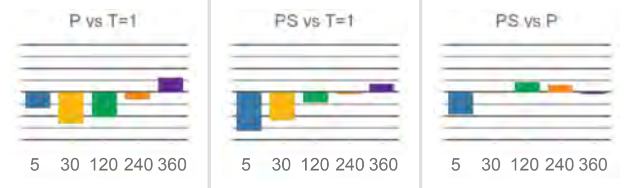

Locus		5m	30m	120m	240m	360m
**PGN_0690**	P vs T=1	-0.767	-1.484	-1.512	-1.251	-0.179
	PS vs T=1	-1.609	-1.407	-0.179	0.514	0.635
	PS vs P	-0.800	-0.083	1.040	1.464	0.863

conserved hypothetical protein

unknown function

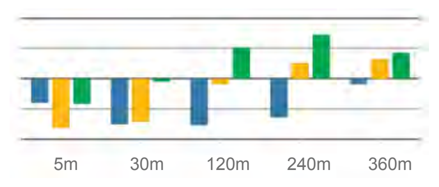

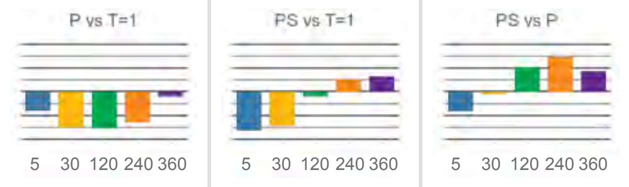

Locus		5m	30m	120m	240m	360m
**PGN_0691**	P vs T=1	-0.234	-0.009	-0.303	-0.991	-1.672
	PS vs T=1	-0.499	-0.445	-0.211	-0.346	-0.628
	PS vs P	-0.240	-0.370	0.131	0.561	0.892

conserved hypothetical protein

hypothetical proteins-Conserved

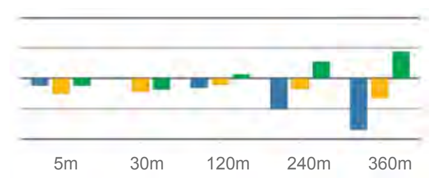

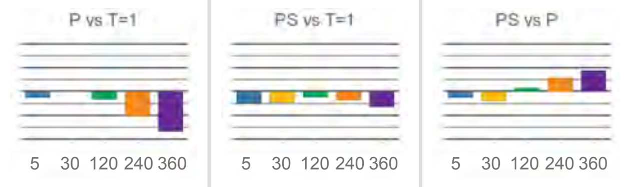

Locus		5m	30m	120m	240m	360m
**PGN_0692**	P vs T=1	-0.159	-0.237	-0.871	-1.645	-2.106
	PS vs T=1	-0.731	-0.836	-0.824	-0.919	-1.158
	PS vs P	-0.517	-0.542	0.056	0.655	0.874

phosphoserine phosphatase

amino acid biosynthesis

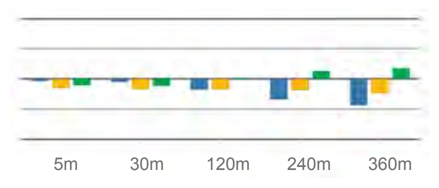

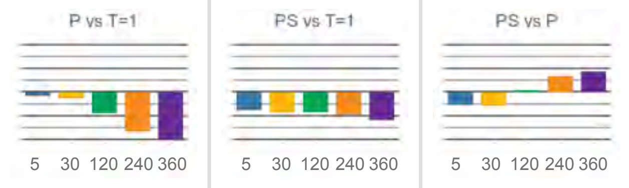

Locus		5m	30m	120m	240m	360m
**PGN_0693**	P vs T=1	0.153	-0.070	-0.304	-0.853	-1.712
	PS vs T=1	-0.835	-0.502	-0.026	-0.010	-0.249
	PS vs P	-0.907	-0.383	0.302	0.804	1.370

conserved hypothetical protein

hypothetical proteins-Conserved

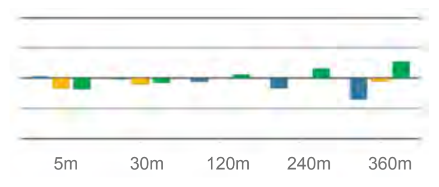

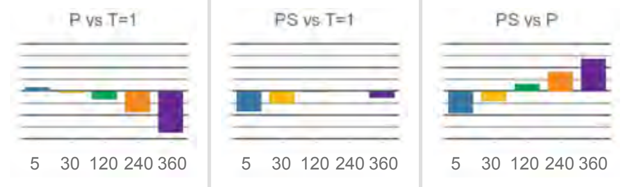

PgSg Time Course

log_2 (Fold Change)

Legend: P vs T=1 | PS vs T=1 | PS vs P

Locus		5m	30m	120m	240m	360m
**PGN_0694**	P vs T=1	0.238	0.575	0.637	0.756	0.163
rpmH	PS vs T=1	-0.627	-0.168	-0.048	0.183	0.446
	PS vs P	-0.823	-0.640	-0.606	-0.465	0.263

50S ribosomal protein L34

protein synthesis

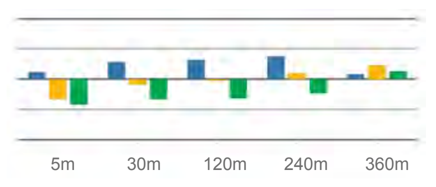

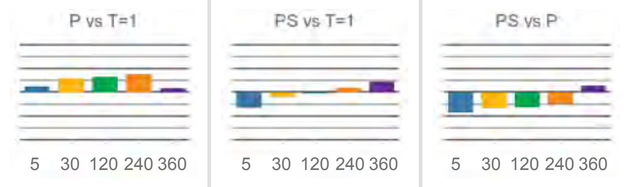

Locus		5m	30m	120m	240m	360m
**PGN_0695**	P vs T=1	-0.125	0.014	0.262	0.154	0.288
	PS vs T=1	0.380	0.554	0.239	-0.044	-0.061
	PS vs P	0.460	0.507	-0.005	-0.202	-0.328

probable septum formation protein Maf

cellular processes

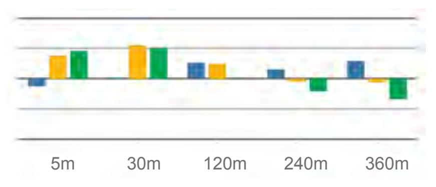

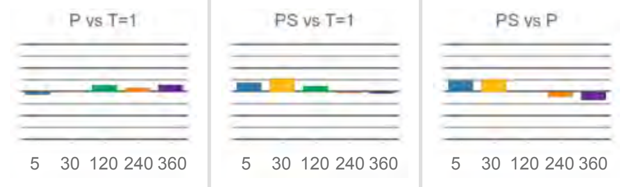

Locus		5m	30m	120m	240m	360m
**PGN_0696**	P vs T=1	0.063	0.278	0.266	0.136	-0.066
	PS vs T=1	0.951	0.838	0.578	0.197	0.265
	PS vs P	0.842	0.542	0.301	0.027	0.284

probable hydrolase

hypothetical proteins-Conserved

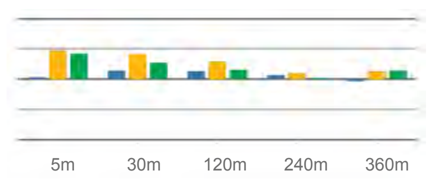

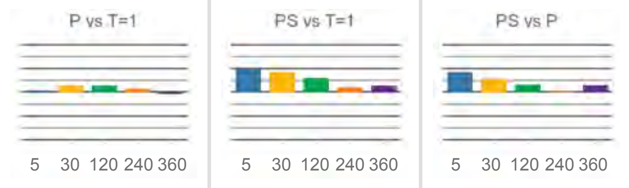

Locus		5m	30m	120m	240m	360m
**PGN_0697**	P vs T=1	-0.033	0.294	0.277	0.234	-0.119
	PS vs T=1	0.684	0.398	0.526	0.626	0.621
	PS vs P	0.692	0.100	0.236	0.369	0.698

conserved hypothetical protein

hypothetical proteins-Conserved

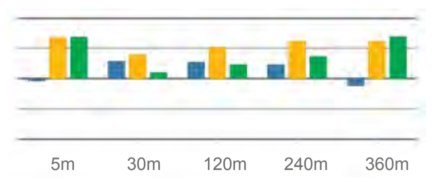

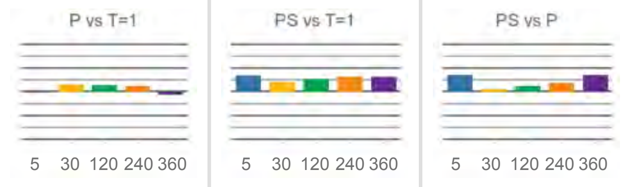

Locus		5m	30m	120m	240m	360m
**PGN_0698**	P vs T=1	-0.311	-0.284	-0.623	-0.933	-1.264
	PS vs T=1	-0.230	-0.424	-0.435	-0.005	-0.250
	PS vs P	0.077	-0.134	0.159	0.841	0.925

probable nitroreductase

unknown function

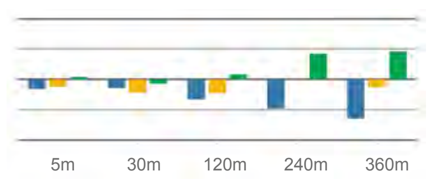

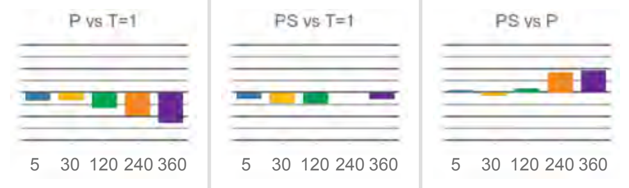

Locus		5m	30m	120m	240m	360m
**PGN_0699**	P vs T=1	0.286	-0.336	-0.363	0.861	1.491
	PS vs T=1	-0.129	-0.939	-0.128	0.623	1.061
	PS vs P	-0.232	-0.642	-0.114	0.198	0.088

conserved hypothetical protein

hypothetical proteins-Conserved

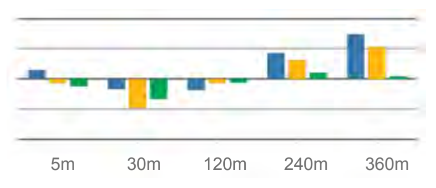

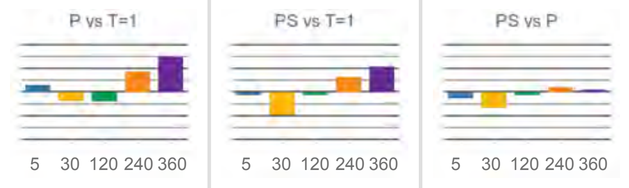

Locus		5m	30m	120m	240m	360m
**PGN_0700**	P vs T=1	-0.259	-0.080	0.652	1.094	1.466
	PS vs T=1	0.163	0.121	0.740	1.325	1.218
	PS vs P	0.317	0.112	0.103	0.310	-0.162

putative oxidoreductase Gfo/Idh/MocA family

unknown function

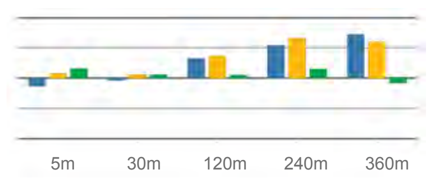

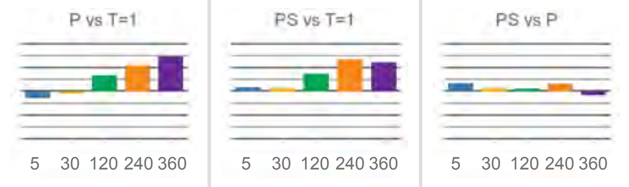

Supplement: Table S1 — This is the first part of three files containing the master list of differential expression data for annotated genes discussed in this study, ordered by PGN number. Four different bar graphs showing the three possible differential expression comparisons are presented for each ORF, as an aid to visualizing the gene's behavior throughout the time course. [file Table1.PDF]
