# Supplementary material for: Insights into Dynamic Polymicrobial Synergy Revealed by Time-Coursed RNA-Seq
Source: Front Microbiol. 2017 Feb 28;8:261. doi: 10.3389/fmicb.2017.00261 (PMC5329018; doi:10.3389/fmicb.2017.00261)

log$_2$ (Fold Change)

Locus		5m	30m	120m	240m	360m
**PGN_0701**	P vs T=1	-0.609	-0.678	-0.770	-0.153	0.680
lacZI	PS vs T=1	0.248	-0.370	-1.107	-0.849	-0.265
	PS vs P	0.826	0.276	-0.397	-0.635	-0.852
	beta-galactosidase					
	energy metabolism					
**PGN_0702**	P vs T=1	-0.329	-0.590	-1.309	-1.424	-0.635
	PS vs T=1	0.405	0.292	-0.862	-1.343	-1.397
	PS vs P	0.742	0.870	0.335	-0.074	-0.716
	conserved hypothetical protein					
	hypothetical proteins-Conserved					
**PGN_0703**	P vs T=1	-0.363	-0.918	-1.524	-1.998	-1.338
	PS vs T=1	0.386	0.332	-0.435	-1.063	-1.104
	PS vs P	0.778	1.185	0.961	0.627	0.199
	hypothetical protein					
	hypothetical proteins					
**PGN_0704**	P vs T=1	0.234	0.244	-0.144	-0.560	-0.944
	PS vs T=1	0.022	0.186	-0.277	-0.825	-0.928
	PS vs P	-0.192	-0.035	-0.125	-0.278	-0.009
	putative tonB-linked outer membrane receptor					
	transport and binding proteins					
**PGN_0705**	P vs T=1	-0.512	-0.162	0.159	-0.383	-1.624
	PS vs T=1	-0.330	0.235	0.159	-1.061	-1.561
	PS vs P	0.163	0.406	0.048	-0.662	0.012
	heme-binding protein FetB					
	transport and binding proteins					
**PGN_0706**	P vs T=1	-0.424	-0.494	-0.527	-0.874	-0.478
	PS vs T=1	0.229	-0.057	-1.200	-1.226	-1.129
	PS vs P	0.646	0.428	-0.650	-0.397	-0.622
	putative exported periplasmic protein					
	hypothetical proteins-Conserved					
**PGN_0707**	P vs T=1	-0.619	-0.458	-0.079	0.297	0.613
	PS vs T=1	0.042	-0.147	-0.808	-0.382	-0.240
	PS vs P	0.604	0.275	-0.699	-0.578	-0.764
	putative iron compound ABC transporter permease protein					
	transport and binding proteins					

P vs T=1 | PS vs T=1 | PS vs P

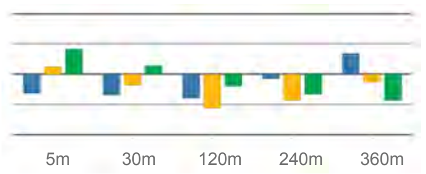

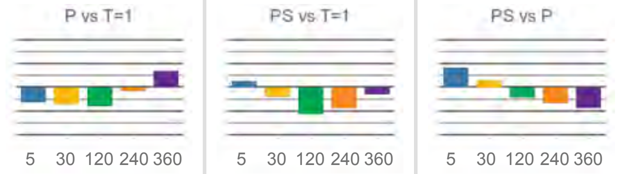

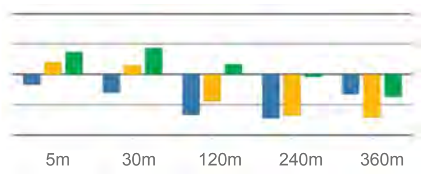

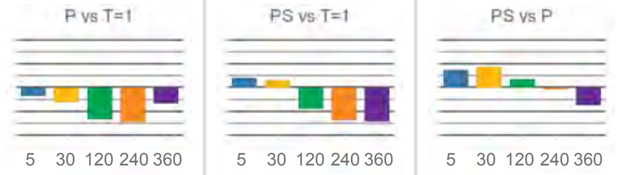

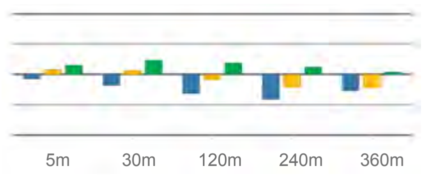

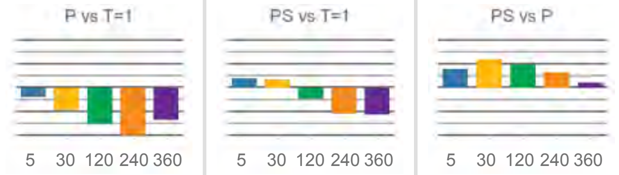

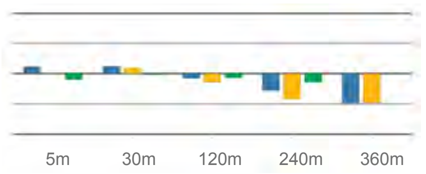

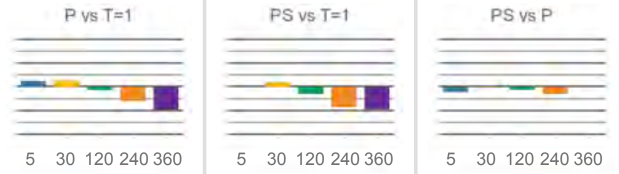

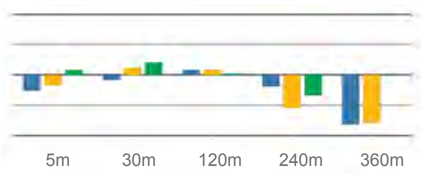

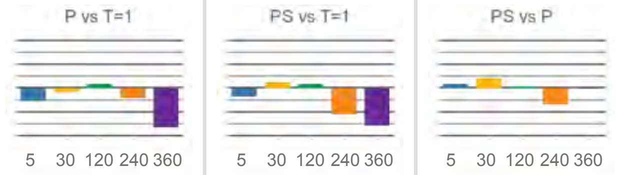

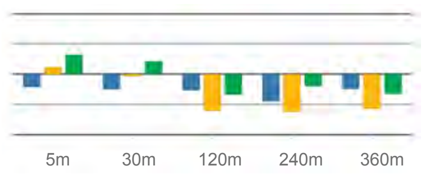

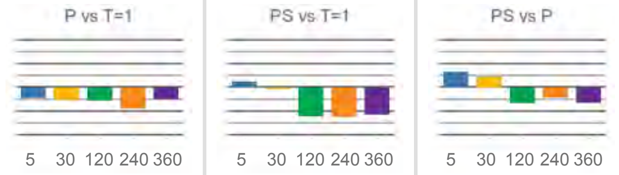

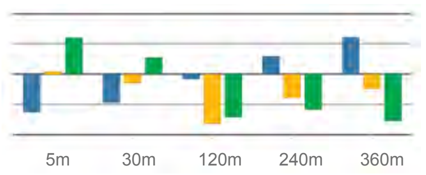

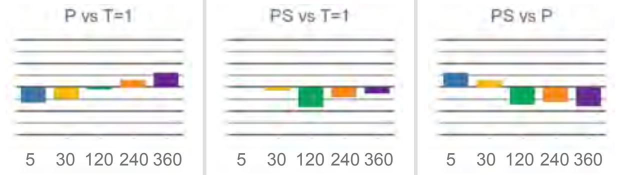

log$_2$ (Fold Change)

P vs T=1 | PS vs T=1 | PS vs P

Locus		5m	30m	120m	240m	360m
**PGN_0708**	P vs T=1	-0.467	-0.515	-0.236	-0.121	0.163
	PS vs T=1	-0.126	-0.440	-1.185	-0.861	-0.580
	PS vs P	0.326	0.054	-0.907	-0.672	-0.673

putative iron compound ABC transporter ATP-binding protein

transport and binding proteins

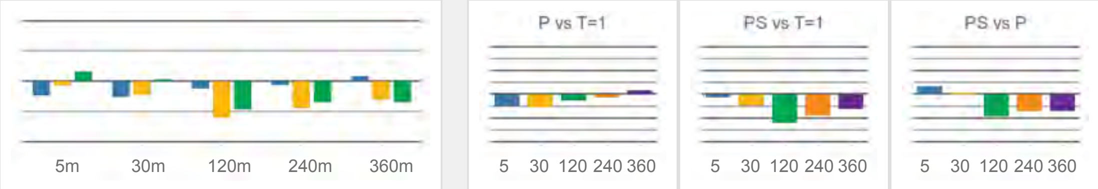

Locus		5m	30m	120m	240m	360m
**PGN_0709**	P vs T=1	0.488	1.102	1.524	0.960	-0.137
	PS vs T=1	0.465	0.648	0.609	0.200	-0.098
	PS vs P	-0.034	-0.429	-0.869	-0.740	0.008

putative indolepyruvate ferredoxin oxidoreductase beta subunit

energy metabolism

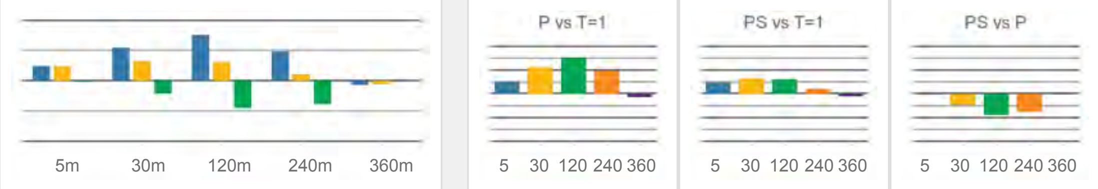

Locus		5m	30m	120m	240m	360m
**PGN_0710**	P vs T=1	0.600	1.130	1.562	1.142	0.348
	PS vs T=1	1.287	1.182	1.138	0.866	0.622
	PS vs P	0.626	0.074	-0.343	-0.251	0.227

indolepyruvate ferredoxin oxidoreductase alpha subunit

energy metabolism

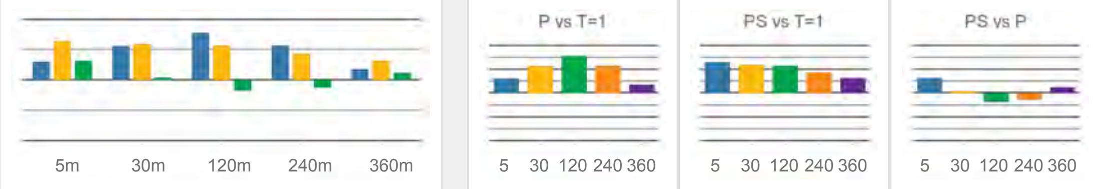

Locus		5m	30m	120m	240m	360m
**PGN_0711**	P vs T=1	0.179	0.082	0.096	0.471	0.370
	PS vs T=1	0.210	0.085	0.497	1.007	0.586
	PS vs P	0.025	-0.023	0.350	0.563	0.215

putative oxidoreductase short chain dehydrogenase/reductase family

unknown function

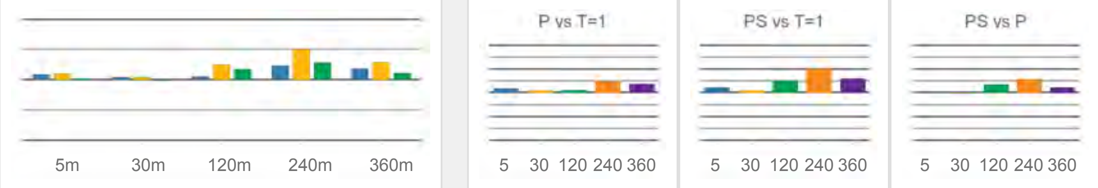

Locus		5m	30m	120m	240m	360m
**PGN_0712**	P vs T=1	-0.443	-0.070	0.099	1.844	3.326
	PS vs T=1	-0.789	-0.496	0.520	1.125	2.486
	PS vs P	-0.594	-0.411	-0.055	0.109	0.496

conserved hypothetical protein

hypothetical proteins-Conserved

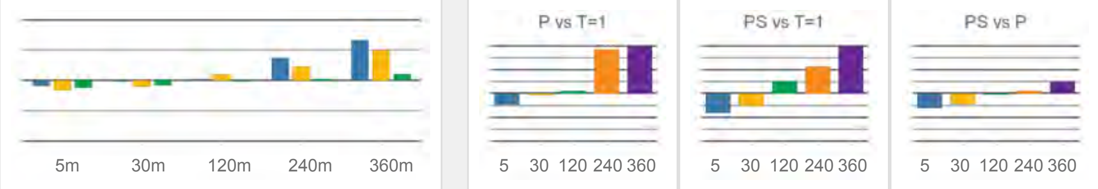

Locus		5m	30m	120m	240m	360m
**PGN_0713**	P vs T=1	-0.250	-0.105	-0.049	0.091	-0.022
	PS vs T=1	0.224	-0.012	0.123	0.566	0.174
	PS vs P	0.443	0.080	0.157	0.472	0.183

saccharopine dehydrogenase

hypothetical proteins-Conserved

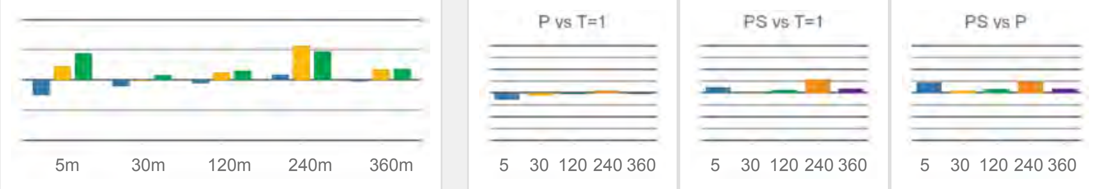

Locus		5m	30m	120m	240m	360m
**PGN_0714**	P vs T=1	0.596	0.678	0.694	0.835	0.736
	PS vs T=1	0.727	0.183	0.123	0.798	0.650
	PS vs P	0.129	-0.469	-0.559	-0.006	-0.080

probable pyrazinamidase/nicotinamidase

biosynthesis of cofactors, prosthetic groups, and carriers

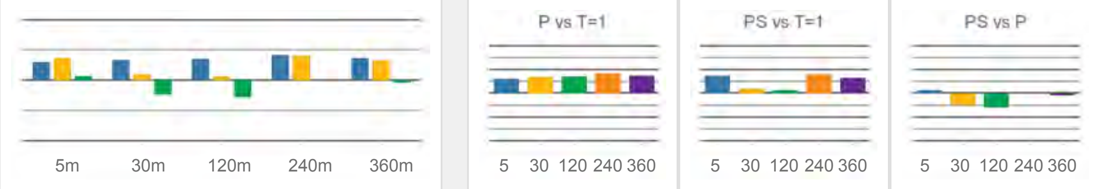

PgSg Time Course

log2 (Fold Change)

Legend: P vs T=1 · PS vs T=1 · PS vs P

Locus		5m	30m	120m	240m	360m
PGN_0715	P vs T=1	0.230	0.560	1.277	1.757	2.322
	PS vs T=1	-0.481	-0.670	0.016	0.753	1.299
	PS vs P	-0.722	-1.144	-1.110	-0.757	-0.819
	outer membrane efflux protein					
	transport and binding proteins					

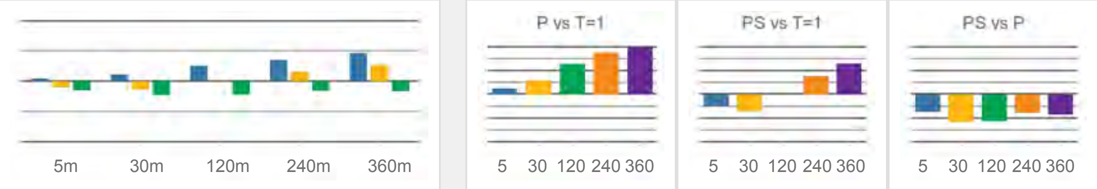

Locus		5m	30m	120m	240m	360m
PGN_0716	P vs T=1	-0.301	0.052	0.964	1.946	2.376
	PS vs T=1	-0.924	-0.655	-0.295	0.722	0.819
	PS vs P	-0.712	-0.709	-1.090	-0.802	-1.224
	ABC transporter permease					
	transport and binding proteins					

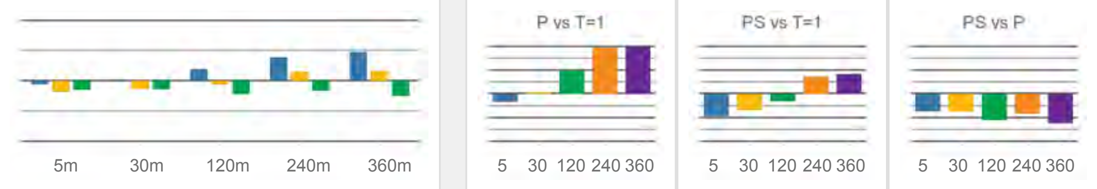

Locus		5m	30m	120m	240m	360m
PGN_0717	P vs T=1	-0.314	0.489	0.811	0.887	1.064
	PS vs T=1	-0.834	-0.837	-0.428	0.143	0.711
	PS vs P	-0.614	-1.071	-0.973	-0.477	-0.150
	conserved hypothetical protein					
	hypothetical proteins-Conserved					

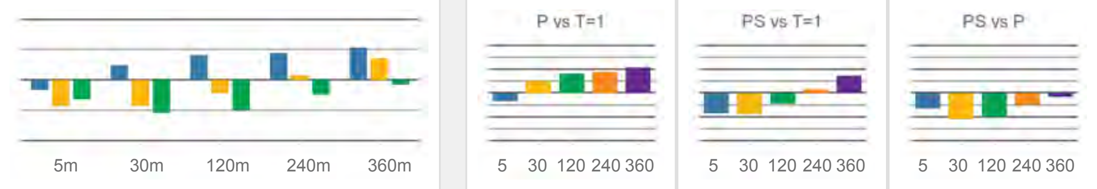

Locus		5m	30m	120m	240m	360m
PGN_0718	P vs T=1	0.566	0.652	1.267	2.295	3.449
	PS vs T=1	0.477	0.420	1.073	1.433	1.838
	PS vs P	-0.164	-0.307	-0.205	-0.563	-1.273
	putative ABC transporter permease protein					
	transport and binding proteins					

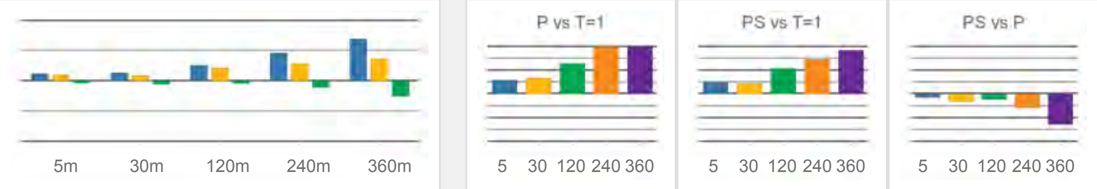

Locus		5m	30m	120m	240m	360m
PGN_0719	P vs T=1	-0.329	-0.634	-0.256	0.532	1.720
	PS vs T=1	-0.303	-0.077	0.915	1.069	0.898
	PS vs P	-0.017	0.422	1.026	0.564	-0.701
	probable ABC transporter permease protein					
	transport and binding proteins					

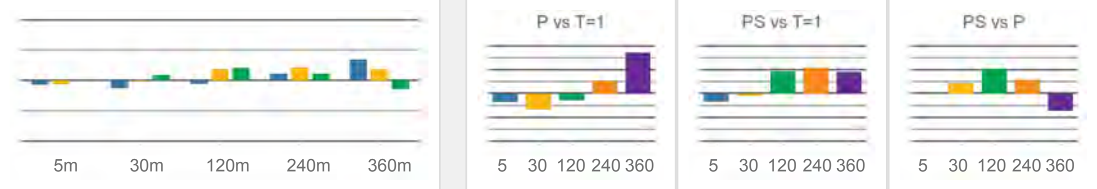

Locus		5m	30m	120m	240m	360m
PGN_0720	P vs T=1	-0.037	0.180	1.069	1.730	2.623
	PS vs T=1	-0.194	-0.068	0.517	0.992	1.418
	PS vs P	-0.234	-0.308	-0.497	-0.569	-1.033
	putative ABC transporter permease protein					
	transport and binding proteins					

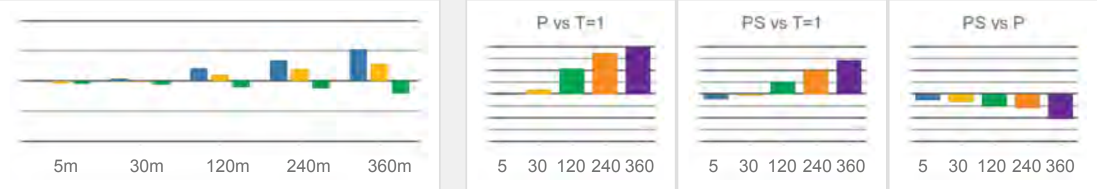

Locus		5m	30m	120m	240m	360m
PGN_0721	P vs T=1	-0.172	0.531	1.223	1.606	1.925
	PS vs T=1	0.431	0.639	1.302	2.045	2.332
	PS vs P	0.327	0.023	0.091	0.503	0.486
	putative ABC transporter ATP-binding protein					
	transport and binding proteins					

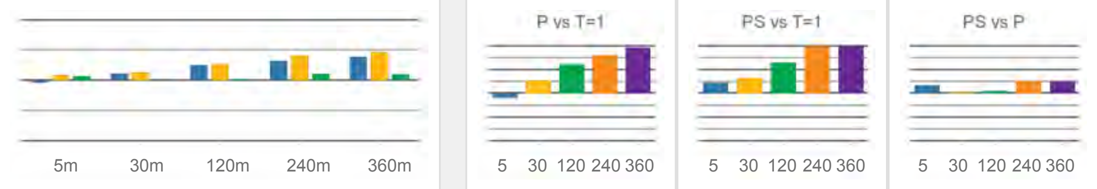

PgSg Time Course

log$_2$ (Fold Change)

Legend: P vs T=1 | PS vs T=1 | PS vs P

Locus		5m	30m	120m	240m	360m
**PGN_0722**	P vs T=1	2.157	3.115	2.992	2.944	2.451
	PS vs T=1	1.493	3.388	4.466	4.475	4.080
	PS vs P	-0.742	0.346	1.461	1.501	1.559

conserved hypothetical protein

hypothetical proteins-Conserved

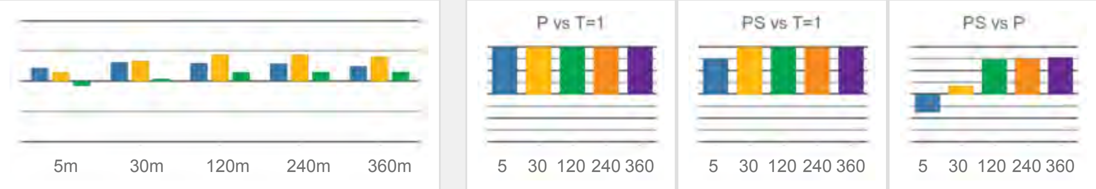

Locus		5m	30m	120m	240m	360m
**PGN_0723**	P vs T=1	1.368	1.878	2.155	1.879	1.146
	PS vs T=1	0.585	0.989	1.252	1.092	0.834
	PS vs P	-0.731	-0.762	-0.768	-0.694	-0.305

succinate-semialdehyde dehydrogenase

energy metabolism

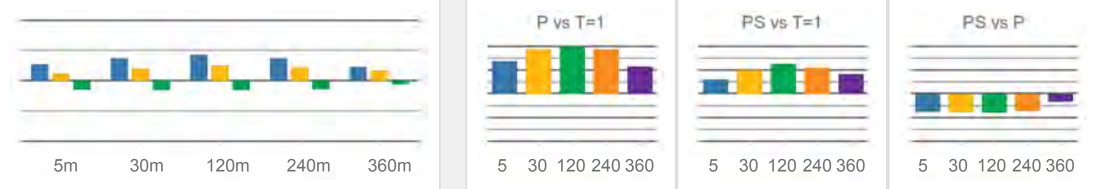

Locus		5m	30m	120m	240m	360m
**PGN_0724**	P vs T=1	0.212	0.064	-0.343	-1.120	-2.034
	PS vs T=1	-0.461	-0.289	-0.686	-1.542	-1.873
	PS vs P	-0.563	-0.264	-0.283	-0.447	0.066

NAD-dependent 4-hydroxybutyrate dehydrogenase

energy metabolism

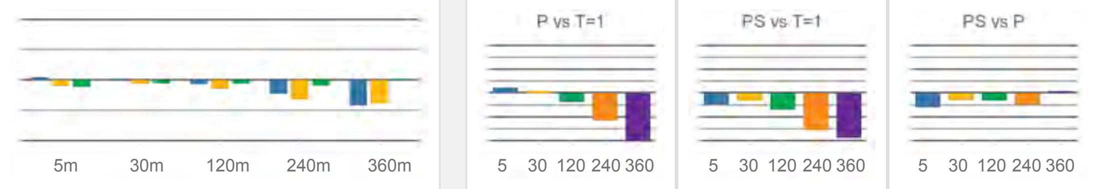

Locus		5m	30m	120m	240m	360m
**PGN_0725**	P vs T=1	0.498	-0.209	-1.344	-2.015	-2.314
	PS vs T=1	-0.027	-0.137	-0.795	-1.620	-2.011
	PS vs P	-0.380	0.129	0.502	0.284	0.212

4-hydroxybutyrate CoA-transferase

energy metabolism

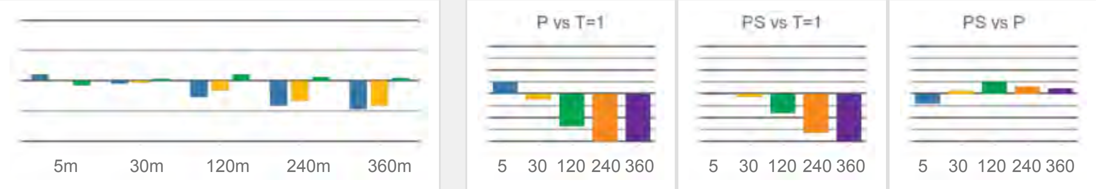

Locus		5m	30m	120m	240m	360m
**PGN_0726**	P vs T=1	-0.398	-0.760	-1.001	-1.150	-1.608
	PS vs T=1	-1.066	-1.048	-1.181	-1.646	-1.663
	PS vs P	-0.591	-0.271	-0.187	-0.506	-0.118

conserved hypothetical protein

unknown function

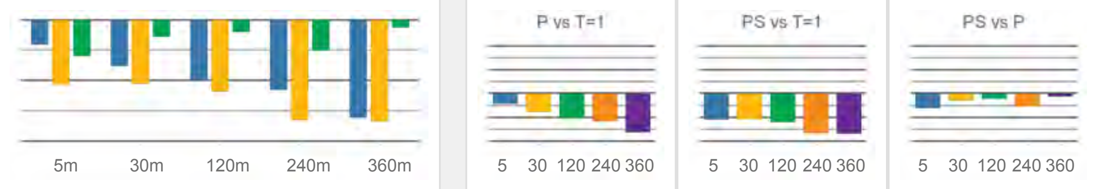

Locus		5m	30m	120m	240m	360m
**PGN_0727**	P vs T=1	-0.238	-0.203	-0.511	-1.340	-2.277
	PS vs T=1	-1.309	-0.922	-0.784	-1.374	-1.715
	PS vs P	-1.025	-0.670	-0.243	-0.054	0.513

4-hydroxybutyryl-CoA dehydratase

energy metabolism

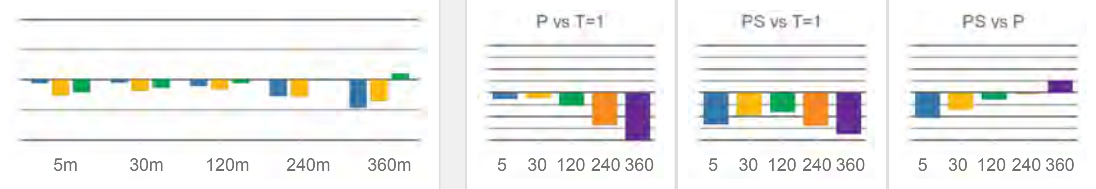

Locus		5m	30m	120m	240m	360m
**PGN_0728**	P vs T=1	0.229	0.373	0.675	0.449	-0.491
	PS vs T=1	-0.321	0.042	-0.206	-0.846	-0.929
	PS vs P	-0.525	-0.297	-0.817	-1.244	-0.461

outer membrane protein 40 precursor

unknown function

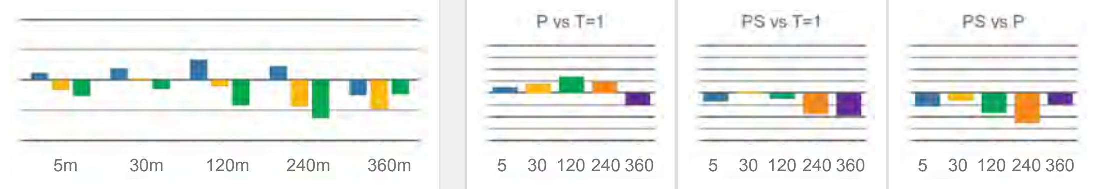

log$_2$ (Fold Change)

Legend: P vs T=1 | PS vs T=1 | PS vs P

PGN_0729

Locus		5m	30m	120m	240m	360m
PGN_0729	P vs T=1	0.328	0.427	0.620	0.166	-1.002
	PS vs T=1	-0.326	0.083	-0.076	-0.793	-1.034
	PS vs P	-0.609	-0.295	-0.617	-0.922	-0.096

outer membrane protein 41 precursor

unknown function

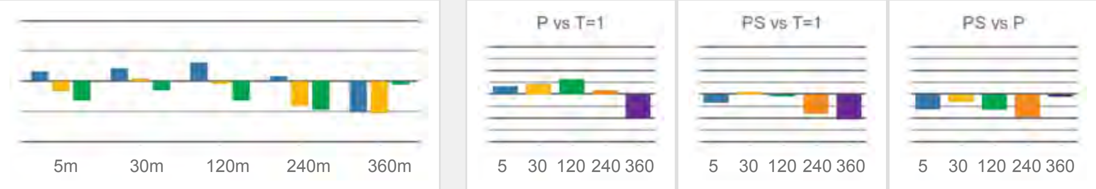

PGN_0730

Locus		5m	30m	120m	240m	360m
PGN_0730	P vs T=1	1.234	0.946	0.241	0.169	-0.823
	PS vs T=1	1.762	2.174	1.354	0.862	0.239
	PS vs P	0.607	1.180	0.877	0.509	0.437

hypothetical protein

hypothetical proteins

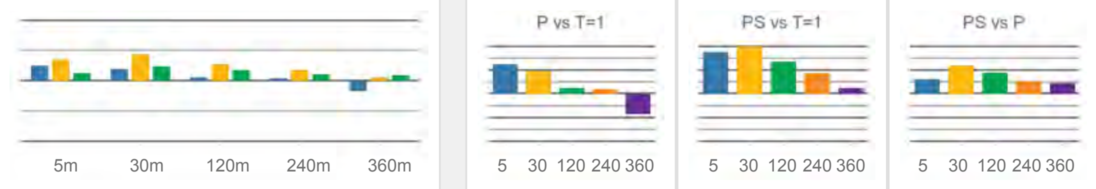

PGN_0731

Locus		5m	30m	120m	240m	360m
PGN_0731	P vs T=1	-0.799	-0.899	-2.078	-2.685	-2.966
	PS vs T=1	-0.625	-0.473	-1.412	-2.397	-1.916
	PS vs P	0.240	0.493	0.399	-0.292	0.452

hypothetical protein

hypothetical proteins

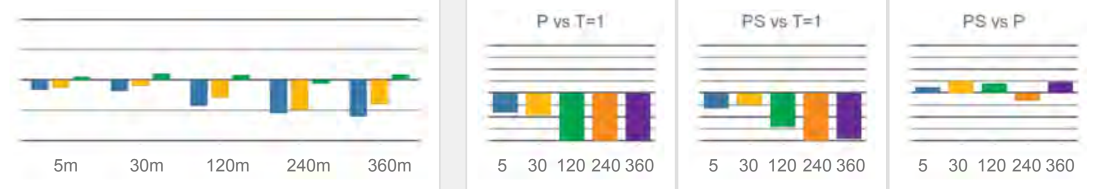

PGN_0732

Locus		5m	30m	120m	240m	360m
PGN_0732	P vs T=1	-0.318	-0.537	-1.487	-2.214	-2.179
	PS vs T=1	0.106	-0.045	-0.790	-1.328	-1.426
	PS vs P	0.452	0.509	0.639	0.685	0.649

conserved hypothetical protein

hypothetical proteins-Conserved

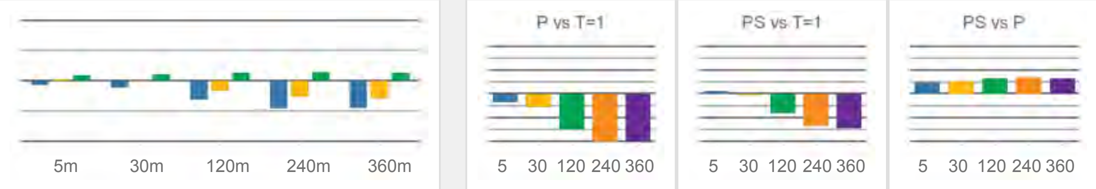

PGN_0733

Locus		5m	30m	120m	240m	360m
PGN_0733	P vs T=1	0.368	0.082	-0.709	-0.926	-0.960
	PS vs T=1	0.861	0.294	-0.544	-0.595	-0.747
	PS vs P	0.504	0.219	0.145	0.297	0.194

alpha-glucan phosphorylase

energy metabolism

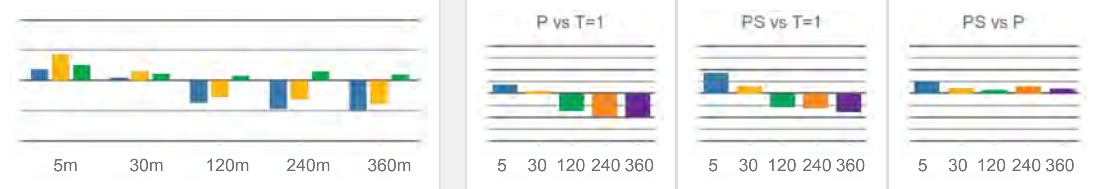

PGN_0734

Locus		5m	30m	120m	240m	360m
PGN_0734	P vs T=1	0.071	0.092	0.842	1.207	2.015
	PS vs T=1	-0.997	0.159	1.030	1.286	1.216
	PS vs P	-1.020	-0.128	0.245	0.262	-0.276

conserved hypothetical protein

hypothetical proteins-Conserved

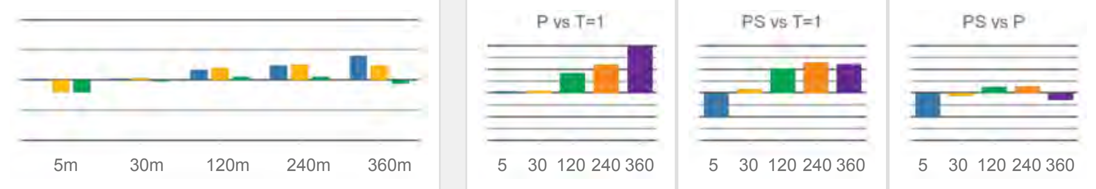

PGN_0735

CobU/P

Locus		5m	30m	120m	240m	360m
PGN_0735	P vs T=1	-0.337	-0.320	-0.710	-0.751	-0.935
	PS vs T=1	-0.447	-0.362	-0.275	0.199	0.206
	PS vs P	-0.110	-0.038	0.389	0.880	1.076

putative bifunctional cobalamin biosynthesis protein

biosynthesis of cofactors, prosthetic groups, and carriers

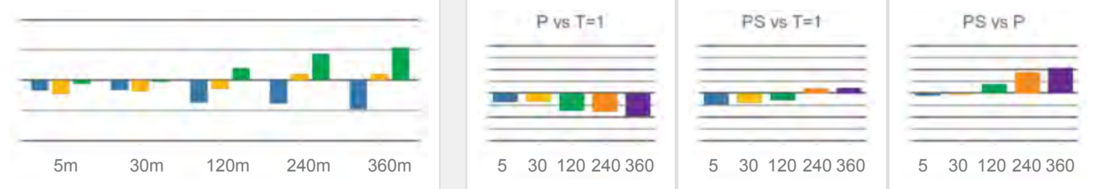

PgSg Time Course

log$_2$ (Fold Change)

Legend: P vs T=1 | PS vs T=1 | PS vs P

Locus		5m	30m	120m	240m	360m
**PGN_0736**	P vs T=1	0.538	0.886	0.921	0.536	0.336
CobU/T	PS vs T=1	0.888	0.922	0.790	0.756	0.492
	PS vs P	0.334	0.047	-0.111	0.197	0.134

putative nicotinate-nucleotide-dimethylbenzimidazole phosphoribosyltransferase

biosynthesis of cofactors, prosthetic groups, and carriers

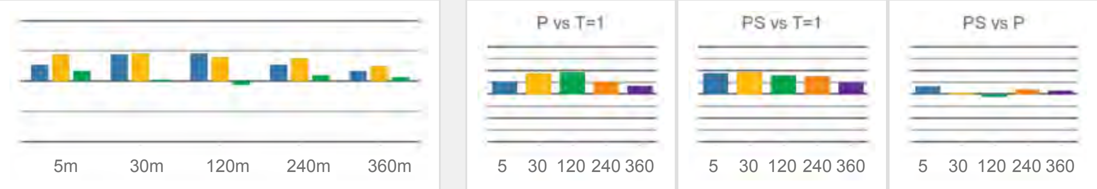

Locus		5m	30m	120m	240m	360m
**PGN_0737**	P vs T=1	0.218	0.492	0.704	0.461	0.552
CobS/V	PS vs T=1	0.639	0.650	0.421	0.544	0.527
	PS vs P	0.400	0.155	-0.264	0.062	-0.025

probable cobalamin-5'-phosphate synthase

biosynthesis of cofactors, prosthetic groups, and carriers

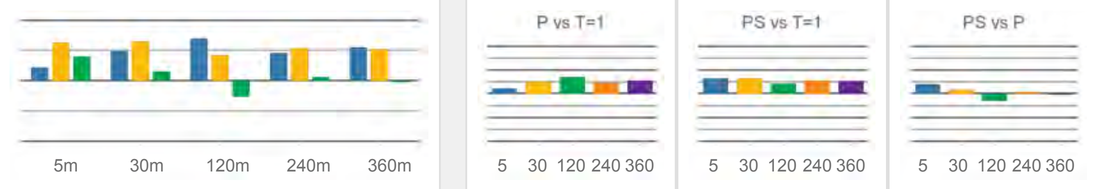

Locus		5m	30m	120m	240m	360m
**PGN_0738**	P vs T=1	0.226	0.447	0.636	0.356	0.693
CobC	PS vs T=1	1.095	1.153	0.775	0.632	0.597
	PS vs P	0.799	0.664	0.143	0.185	-0.083

putative phosphoglycerate mutase

biosynthesis of cofactors, prosthetic groups, and carriers/ unknown

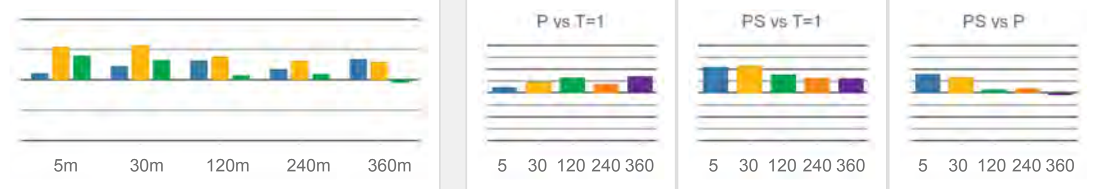

Locus		5m	30m	120m	240m	360m
**PGN_0739**	P vs T=1	1.062	1.123	1.162	0.764	-0.120
	PS vs T=1	0.962	1.475	2.031	2.096	1.848
	PS vs P	-0.103	0.346	0.868	1.288	1.881

glutamate racemase

cell envelope

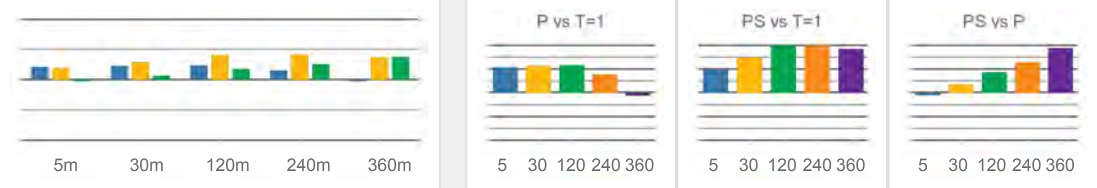

Locus		5m	30m	120m	240m	360m
**PGN_0740**	P vs T=1	0.220	0.841	1.629	1.701	0.890
	PS vs T=1	0.171	0.943	1.722	1.910	1.878
	PS vs P	-0.119	0.088	0.135	0.247	0.959

conserved hypothetical protein

hypothetical proteins-Conserved

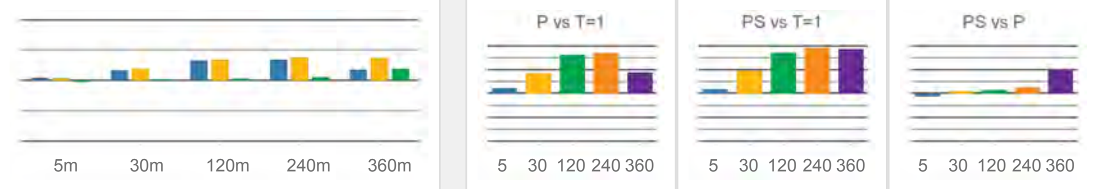

Locus		5m	30m	120m	240m	360m
**PGN_0741**	P vs T=1	0.445	0.865	1.034	1.143	0.788
	PS vs T=1	-0.105	0.336	1.533	1.944	1.800
	PS vs P	-0.563	-0.518	0.501	0.804	0.998

TonB-dependent receptor

transport and binding proteins

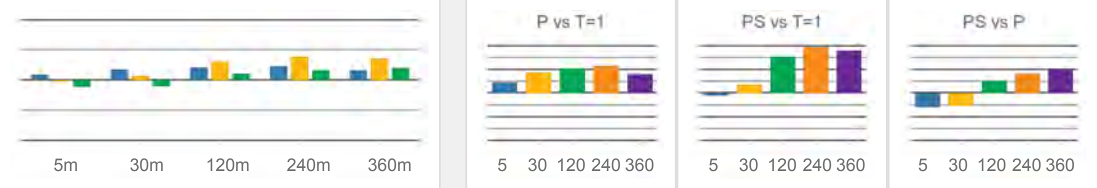

Locus		5m	30m	120m	240m	360m
**PGN_0742**	P vs T=1	-0.165	-0.078	-0.048	-0.472	-1.358
	PS vs T=1	-0.058	0.219	0.187	-0.344	-0.467
	PS vs P	0.110	0.308	0.261	0.122	0.833

probable FKBP-type peptidyl-prolyl cis-trans isomerase

protein fate

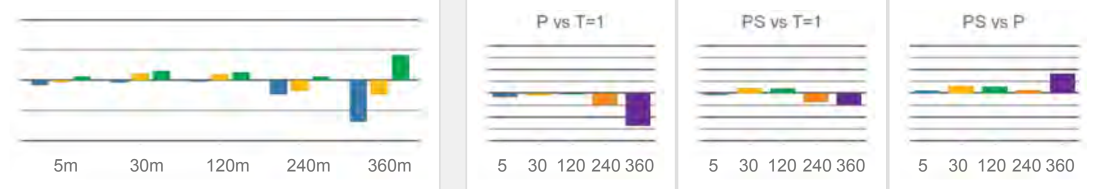

PgSg Time Course

log2 (Fold Change)

Locus		5m	30m	120m	240m	360m
PGN_0743	P vs T=1	-0.575	-0.217	-0.386	-0.973	-1.697
	PS vs T=1	-0.880	-0.571	-0.677	-1.047	-1.442
	PS vs P	-0.302	-0.303	-0.255	-0.100	0.181
PGN_0744	P vs T=1	-0.164	-0.672	-1.480	-1.656	-1.144
	PS vs T=1	-0.911	-0.653	-0.255	-0.014	-0.096
	PS vs P	-0.671	0.023	1.058	1.374	0.988
PGN_0745	P vs T=1	-0.475	-1.475	-1.075	-0.855	0.069
	PS vs T=1	-0.985	-1.011	-0.806	-0.135	-0.094
	PS vs P	-0.383	0.058	0.060	0.506	0.104
PGN_0746	P vs T=1	-0.303	0.048	0.643	0.768	0.111
	PS vs T=1	-0.967	-0.560	-0.325	-0.402	-0.259
	PS vs P	-0.660	-0.575	-0.889	-1.064	-0.350
PGN_0747	P vs T=1	-0.237	-0.540	-0.422	-0.211	0.086
	PS vs T=1	-0.798	-0.960	-1.162	-0.653	-0.435
	PS vs P	-0.513	-0.414	-0.709	-0.355	-0.431
PGN_0748 *gslA*	P vs T=1	1.387	2.146	2.019	1.403	0.659
	PS vs T=1	1.580	1.650	0.901	0.560	0.557
	PS vs P	0.181	-0.433	-1.067	-0.836	-0.131
PGN_0749	P vs T=1	0.190	0.087	0.122	0.222	-0.061
	PS vs T=1	-0.007	-0.225	-0.083	0.036	0.070
	PS vs P	-0.195	-0.318	-0.213	-0.186	0.113

PGN_0743: probable FKBP-type peptidyl-prolyl cis-trans isomerase FkpA — *protein fate*

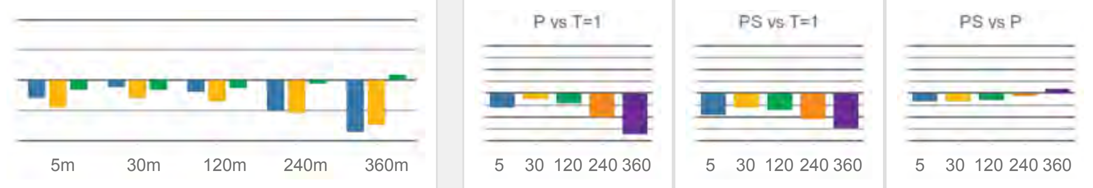

PGN_0744: probable FKBP-type peptidyl-prolyl cis-trans isomerase — *protein fate*

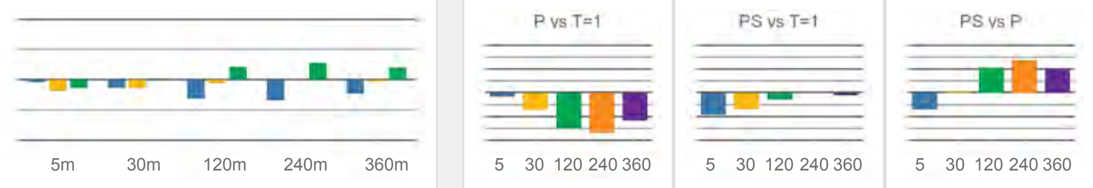

PGN_0745: hypothetical protein — *hypothetical proteins*

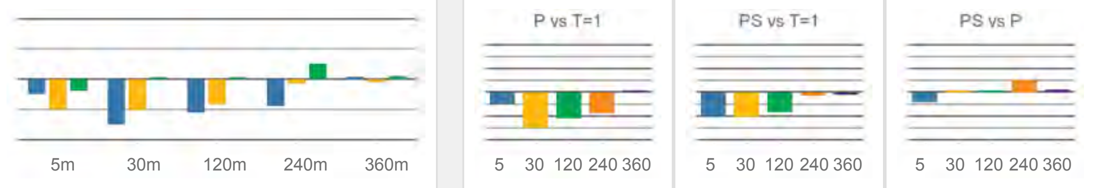

PGN_0746: conserved hypothetical protein — *hypothetical proteins-Conserved*

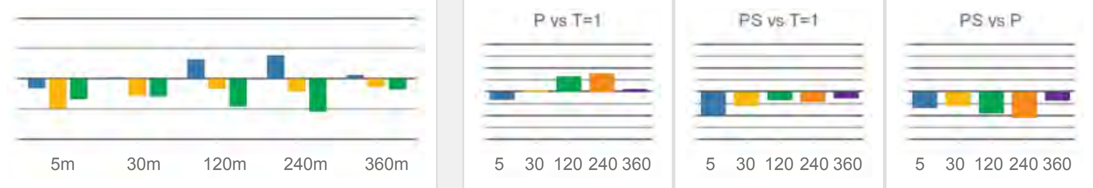

PGN_0747: probable anthranilate synthase component II — *biosynthesis of cofactors, prosthetic groups, and carriers*

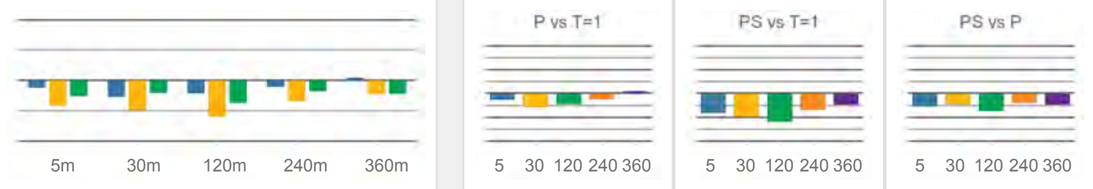

PGN_0748 (*gslA*): gingipain-sensitive ligand A — *cellular processes*

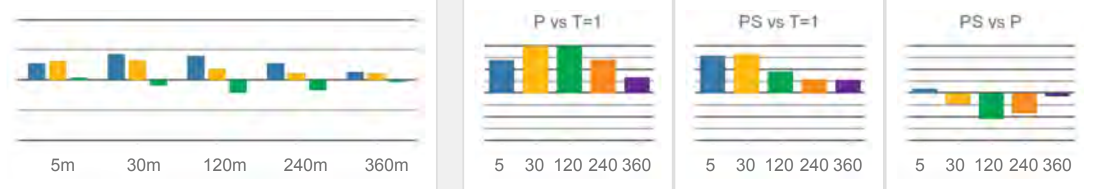

PGN_0749: transposase in ISPg1

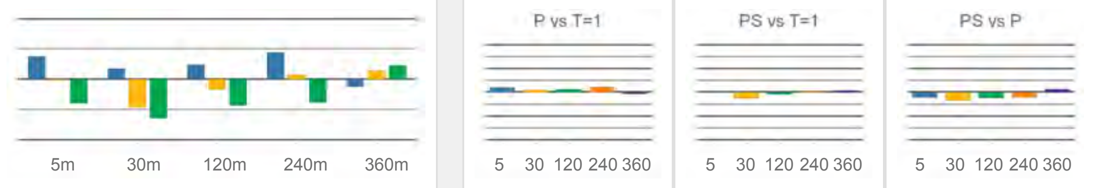

log$_2$ (Fold Change)

Legend: P vs T=1 | PS vs T=1 | PS vs P

Locus		5m	30m	120m	240m	360m
**PGN_0750**	P vs T=1	-0.748	-0.896	-0.767	-0.735	-0.173
	PS vs T=1	-1.009	-1.345	-1.461	-1.515	-1.316
	PS vs P	-0.244	-0.442	-0.660	-0.731	-1.046

putative copper homeostasis protein CutC

transport and binding proteins

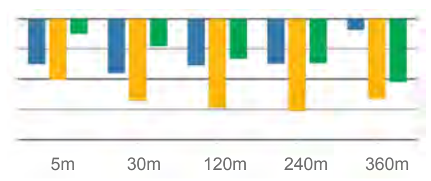

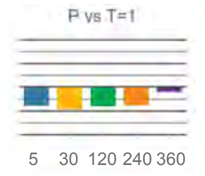

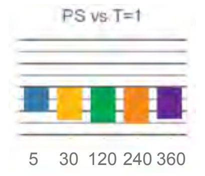

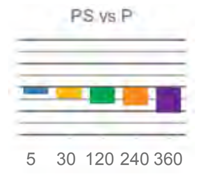

Locus		5m	30m	120m	240m	360m
**PGN_0751**	P vs T=1	-0.108	-0.392	-0.906	-0.853	-0.682
	PS vs T=1	-1.074	-0.799	-1.091	-1.170	-1.024
	PS vs P	-0.909	-0.373	-0.208	-0.319	-0.326

putative cationic transporter

transport and binding proteins

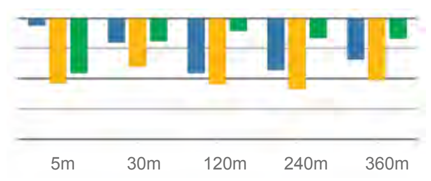

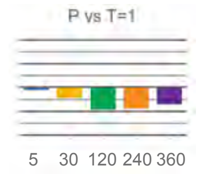

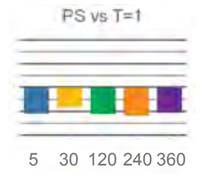

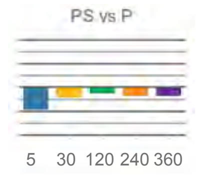

Locus		5m	30m	120m	240m	360m
**PGN_0752**	P vs T=1	0.030	0.199	1.203	1.302	0.821
	PS vs T=1	-0.004	0.539	2.548	3.008	3.055
	PS vs P	-0.225	0.139	1.321	1.641	2.077

hypothetical protein

hypothetical proteins

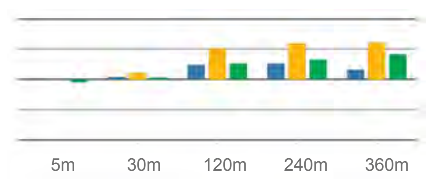

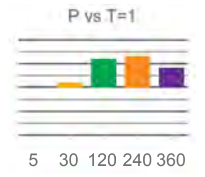

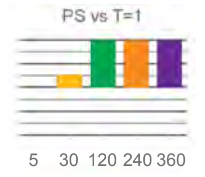

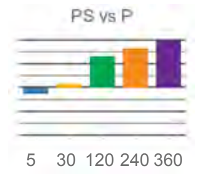

Locus		5m	30m	120m	240m	360m
**PGN_0753**	P vs T=1	0.022	0.171	0.717	1.111	0.717
	PS vs T=1	-0.107	0.797	2.554	2.812	2.588
	PS vs P	-0.211	0.537	1.777	1.672	1.812

probable two component system response regulator

regulatory functions

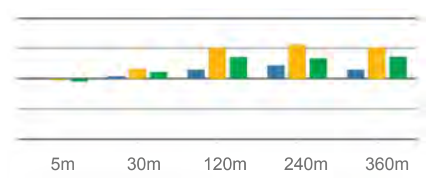

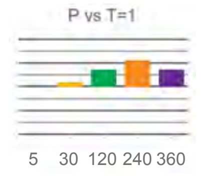

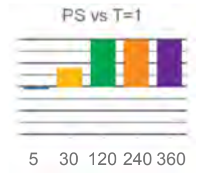

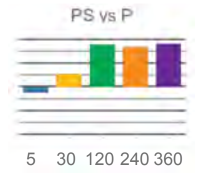

Locus		5m	30m	120m	240m	360m
**PGN_0754**	P vs T=1	0.553	0.172	-0.079	-0.100	-0.549
	PS vs T=1	0.252	0.121	0.078	0.179	0.152
	PS vs P	-0.277	-0.046	0.150	0.274	0.674

conserved hypothetical protein

hypothetical proteins-Conserved

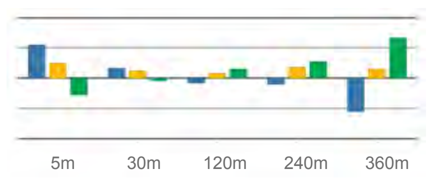

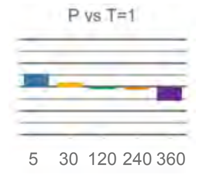

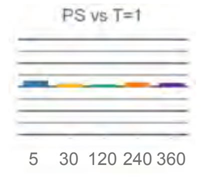

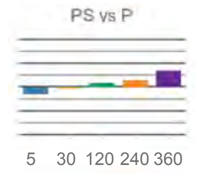

Locus		5m	30m	120m	240m	360m
**PGN_0755**	P vs T=1	0.036	-0.012	0.227	0.886	1.481
	PS vs T=1	-0.453	-0.607	0.033	0.087	0.541
	PS vs P	-0.418	-0.531	-0.171	-0.380	-0.426

hypothetical protein

hypothetical proteins

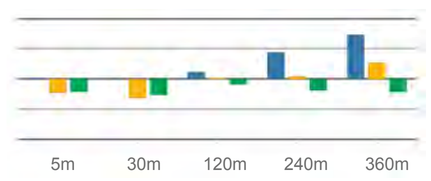

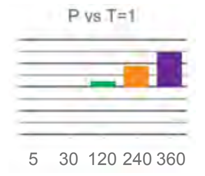

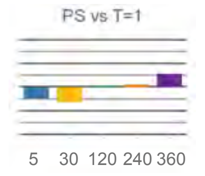

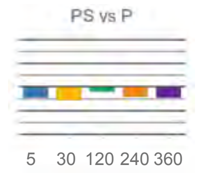

Locus		5m	30m	120m	240m	360m
**PGN_0756**	P vs T=1	0.291	0.806	1.495	1.568	1.041
	PS vs T=1	0.255	0.317	0.779	1.147	1.066
	PS vs P	-0.072	-0.477	-0.665	-0.374	0.027

dipeptidyl-peptidase 5 (paper)

protein fate

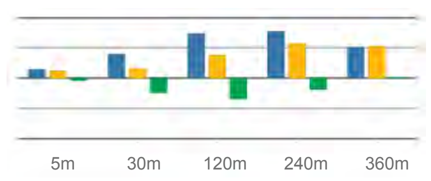

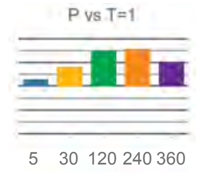

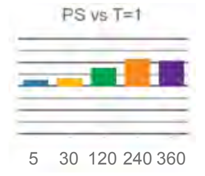

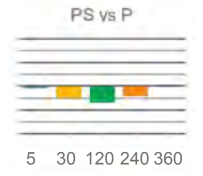

PgSg Time Course

Legend: P vs T=1 | PS vs T=1 | PS vs P

log$_2$ (Fold Change)

Locus		5m	30m	120m	240m	360m
**PGN_0757**	P vs T=1	0.434	1.089	1.164	0.890	0.429
	PS vs T=1	0.835	0.734	0.518	0.078	0.007
	PS vs P	0.375	-0.303	-0.577	-0.753	-0.422

conserved hypothetical protein

unknown function

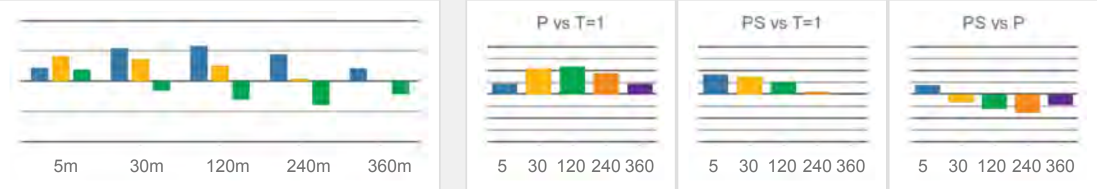

Locus		5m	30m	120m	240m	360m
**PGN_0758**	P vs T=1	-1.840	-1.748	-1.431	-1.523	-2.245
	PS vs T=1	-1.512	-1.127	-1.473	-2.595	-2.698
	PS vs P	0.262	0.574	0.048	-0.956	-0.502

conserved hypothetical protein

hypothetical proteins-Conserved

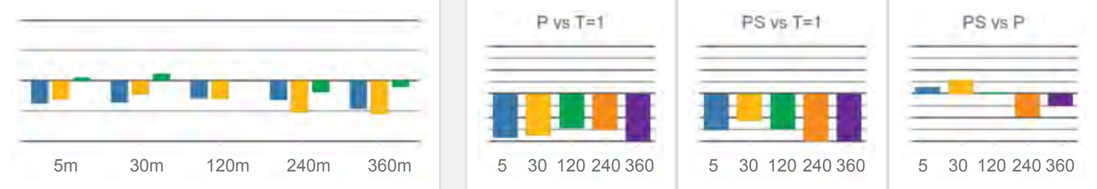

Locus		5m	30m	120m	240m	360m
**PGN_0759**	P vs T=1	-0.162	-0.302	-0.688	-0.628	-0.474
	PS vs T=1	0.569	0.183	-1.021	-1.130	-1.160
	PS vs P	0.733	0.484	-0.353	-0.502	-0.671

conserved hypothetical protein

hypothetical proteins-Conserved

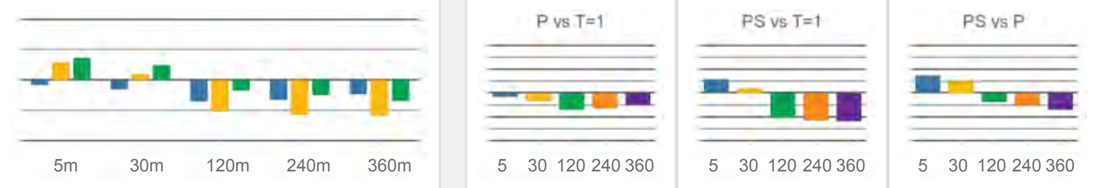

Locus		5m	30m	120m	240m	360m
**PGN_0760**	P vs T=1	-0.289	-0.349	-1.042	-1.300	-1.325
	PS vs T=1	0.072	-0.384	-1.129	-1.644	-1.770
	PS vs P	0.374	-0.014	-0.102	-0.386	-0.466

putative D-alanine--D-alanine ligase

cell envelope

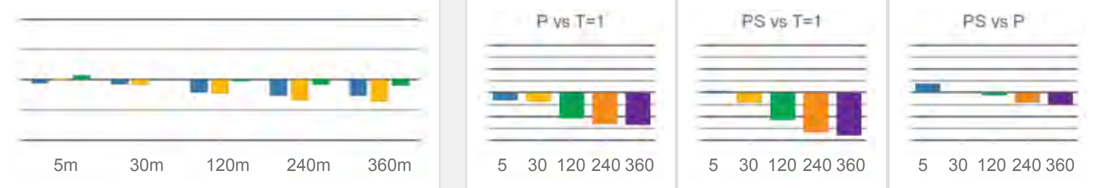

Locus		5m	30m	120m	240m	360m
**PGN_0761**	P vs T=1	-0.180	-0.545	-1.238	-1.271	-1.053
	PS vs T=1	-0.280	-0.935	-1.235	-1.148	-1.460
	PS vs P	-0.052	-0.363	-0.055	0.062	-0.413

ribosomal large subunit pseudouridine synthase

protein synthesis

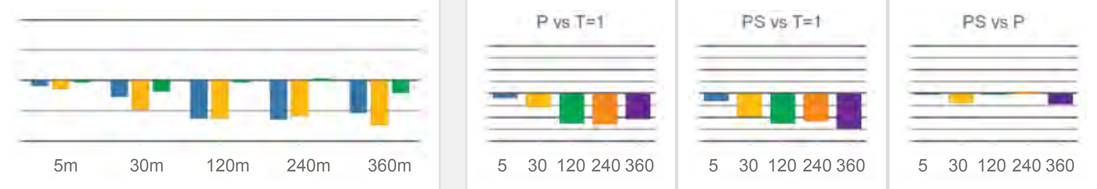

Locus		5m	30m	120m	240m	360m
**PGN_0762**	P vs T=1	0.034	-0.262	-0.781	-1.010	-1.379
	PS vs T=1	-0.423	-0.804	-0.880	-0.689	-0.977
	PS vs P	-0.430	-0.523	-0.106	0.293	0.359

conserved hypothetical protein

hypothetical proteins-Conserved

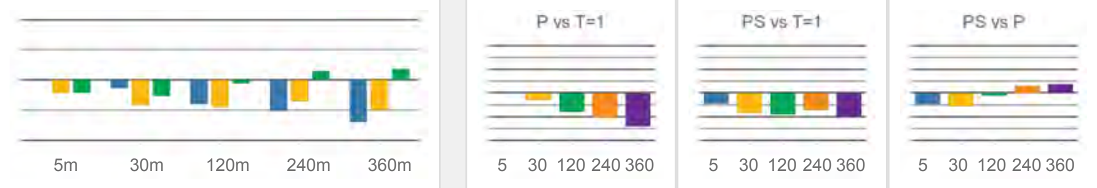

Locus		5m	30m	120m	240m	360m
**PGN_0763**	P vs T=1	0.642	0.738	1.827	2.366	3.249
	PS vs T=1	-0.736	-0.533	0.645	1.149	1.716
	PS vs P	-1.048	-0.907	-0.578	-0.361	-0.571

conserved hypothetical protein

hypothetical proteins-Conserved

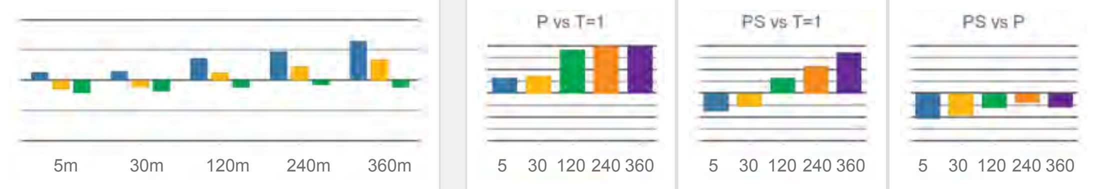

PgSg Time Course

log2 (Fold Change)

P vs T=1 · PS vs T=1 · PS vs P

Locus		5m	30m	120m	240m	360m
**PGN_0764**	P vs T=1	-0.170	0.277	0.714	0.562	0.052
RibC	PS vs T=1	-0.381	0.067	0.260	-0.124	-0.354
	PS vs P	-0.233	-0.189	-0.395	-0.634	-0.408

putative riboflavin synthase alpha subunit

biosynthesis of cofactors, prosthetic groups, and carriers

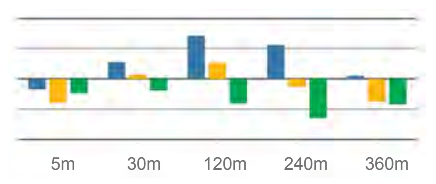

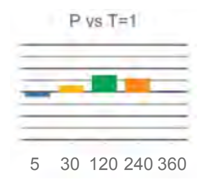

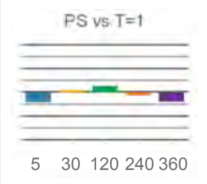

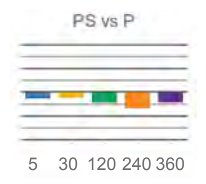

Locus		5m	30m	120m	240m	360m
**PGN_0765**	P vs T=1	-1.060	-0.659	-0.467	-0.433	-0.520
	PS vs T=1	-1.136	-1.388	-1.162	-0.860	-1.229
	PS vs P	-0.109	-0.697	-0.646	-0.366	-0.680

probable nitroreductase

unknown function

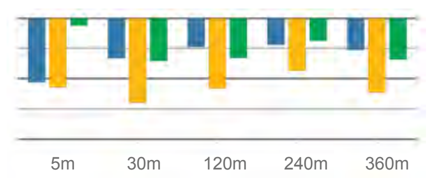

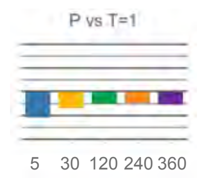

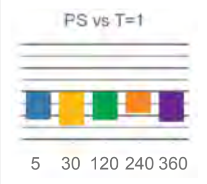

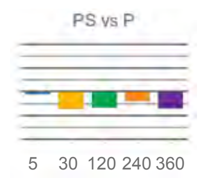

Locus		5m	30m	120m	240m	360m
**PGN_0766**	P vs T=1	-0.683	-0.361	-0.381	-0.380	-0.557
	PS vs T=1	-0.322	-0.860	-1.264	-1.047	-0.909
	PS vs P	0.342	-0.450	-0.825	-0.593	-0.334

aminotransferase class V

unknown function

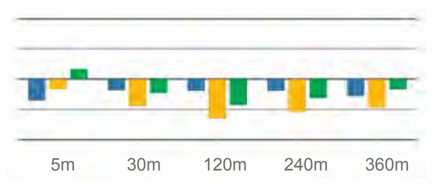

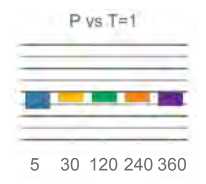

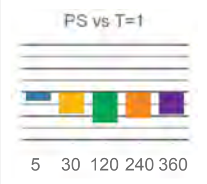

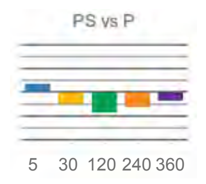

Locus		5m	30m	120m	240m	360m
**PGN_0767**	P vs T=1	-0.435	-0.076	-0.103	-0.047	0.082
	PS vs T=1	-0.128	-0.403	-0.795	-1.319	-0.684
	PS vs P	0.267	-0.221	-0.565	-1.026	-0.589

putative ribonuclease HII

transcription

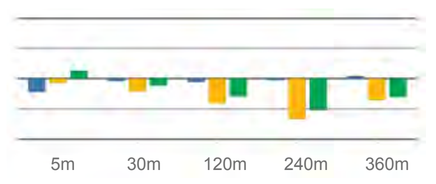

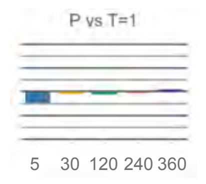

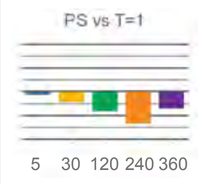

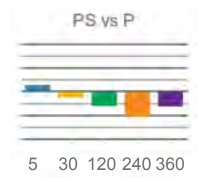

Locus		5m	30m	120m	240m	360m
**PGN_0768**	P vs T=1	0.186	-0.462	-0.703	-0.450	-0.274
	PS vs T=1	-0.209	-0.370	-0.039	0.106	-0.042
	PS vs P	-0.324	0.047	0.513	0.491	0.228

conserved hypothetical protein

hypothetical proteins-Conserved

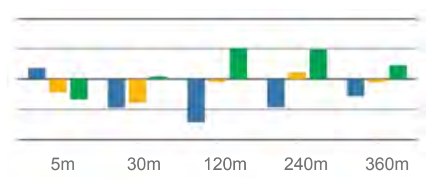

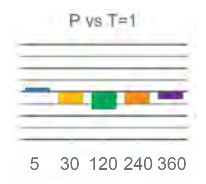

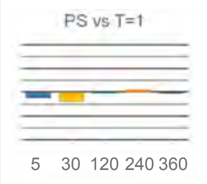

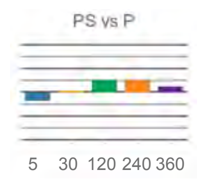

Locus		5m	30m	120m	240m	360m
**PGN_0769**	P vs T=1	-0.493	-0.799	-1.223	-0.892	-0.642
	PS vs T=1	-0.688	-0.813	-0.276	-0.578	-0.493
	PS vs P	-0.166	-0.056	0.698	0.208	0.129

putative cytosine/adenosine deaminase

unknown function

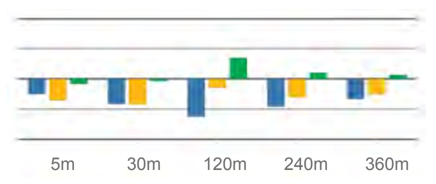

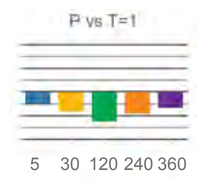

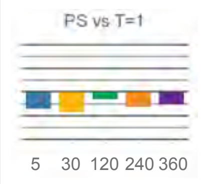

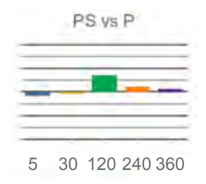

Locus		5m	30m	120m	240m	360m
**PGN_0770**	P vs T=1	-0.758	-0.924	-1.047	-0.647	-0.015
	PS vs T=1	-0.486	-0.530	-0.205	-0.051	-0.283
	PS vs P	0.251	0.344	0.711	0.561	-0.205

conserved hypothetical protein

unknown function

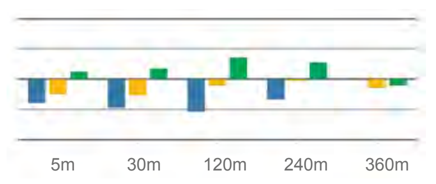

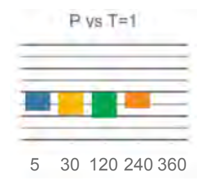

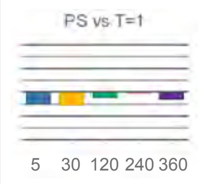

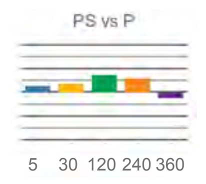

PgSg Time Course

log$_2$ (Fold Change)

P vs T=1 | PS vs T=1 | PS vs P

Locus		5m	30m	120m	240m	360m
**PGN_0771**	P vs T=1	-0.065	-0.369	-0.477	-0.208	0.720
	PS vs T=1	0.440	-0.113	0.314	0.357	0.298
	PS vs P	0.492	0.177	0.629	0.467	-0.285
	conserved hypothetical protein					
	hypothetical proteins-Conserved					
**PGN_0772**	P vs T=1	0.112	0.269	0.213	0.445	0.515
	PS vs T=1	0.291	0.037	-0.362	0.147	0.072
	PS vs P	0.175	-0.201	-0.554	-0.223	-0.387
	probable tRNA/rRNA methyltransferase					
	transcription					
**PGN_0773**	P vs T=1	0.577	0.462	0.196	-0.203	-0.312
	PS vs T=1	-0.189	-0.113	-0.206	-0.399	-0.647
	PS vs P	-0.727	-0.536	-0.360	-0.201	-0.335
	putative lactoylglutathione lyase					
	energy metabolism					
**PGN_0774**	P vs T=1	-0.397	-0.137	0.266	0.648	1.206
	PS vs T=1	-0.128	-0.174	-0.367	-0.031	0.210
	PS vs P	0.218	-0.051	-0.595	-0.573	-0.882
	two-component system sensor histidine kinase					
	signal transduction					
**PGN_0775**	P vs T=1	0.080	0.395	0.888	0.780	0.694
	PS vs T=1	0.059	-0.071	-0.184	0.323	0.283
	PS vs P	-0.036	-0.443	-0.994	-0.396	-0.377
	two-component system response regulator					
	signal transduction					
**PGN_0776**	P vs T=1	0.156	-0.422	-0.283	1.319	2.362
	PS vs T=1	0.292	-0.414	0.123	0.794	0.439
	PS vs P	0.165	-0.275	-0.036	0.148	-0.851
	conserved hypothetical protein					
	hypothetical proteins-Conserved					
**PGN_0777**	P vs T=1	-0.519	-0.848	-1.012	-1.264	-1.432
	PS vs T=1	0.016	-0.187	-0.316	-1.002	-0.869
	PS vs P	0.535	0.609	0.650	0.169	0.477
	probable glycosyl transferase					
	cell envelope					

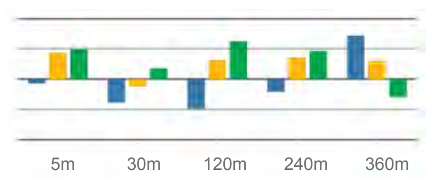

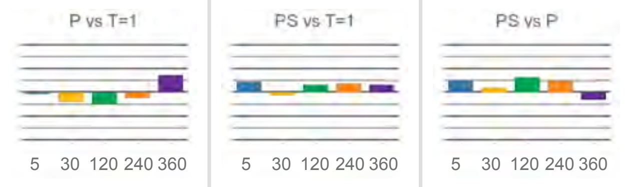

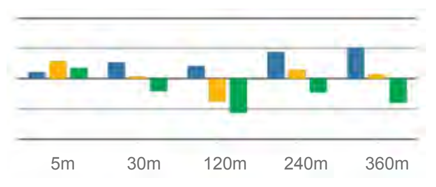

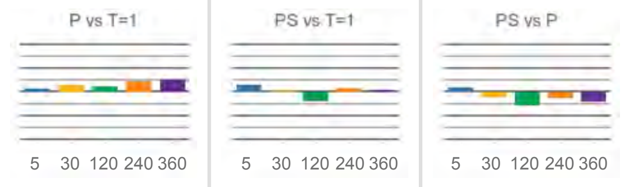

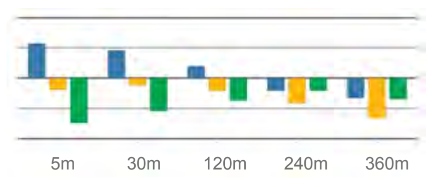

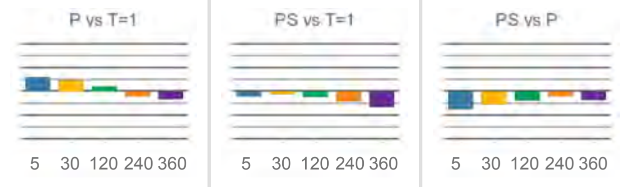

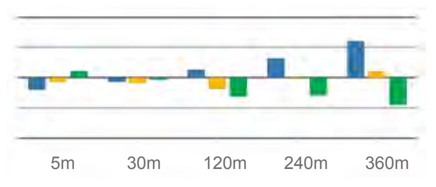

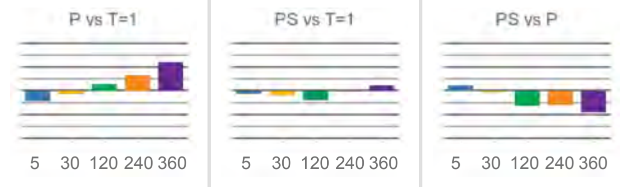

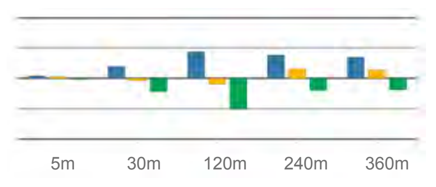

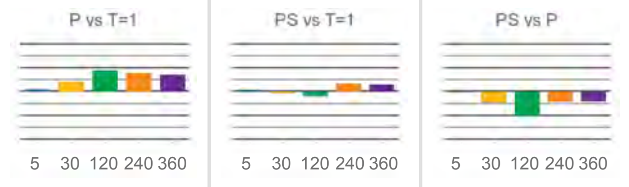

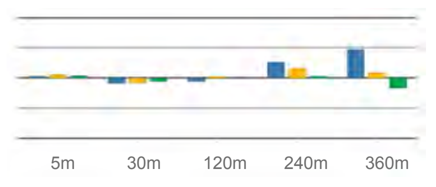

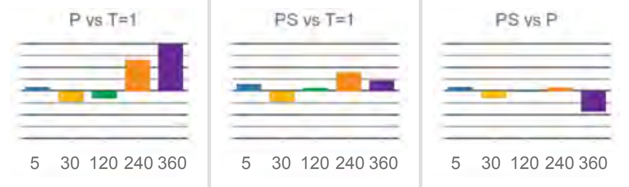

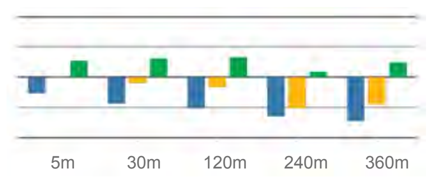

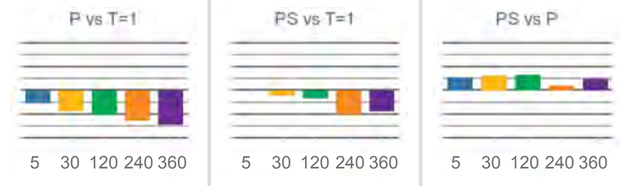

PgSg Time Course

log2 (Fold Change)

Legend: P vs T=1 · PS vs T=1 · PS vs P

PGN_0778

porT

Locus	5m	30m	120m	240m	360m
P vs T=1	0.210	-0.341	-1.184	-0.946	-0.713
PS vs T=1	1.419	1.097	0.909	0.706	0.582
PS vs P	1.201	1.389	1.935	1.524	1.243

Por secretion system protein porT/sprT

unknown function

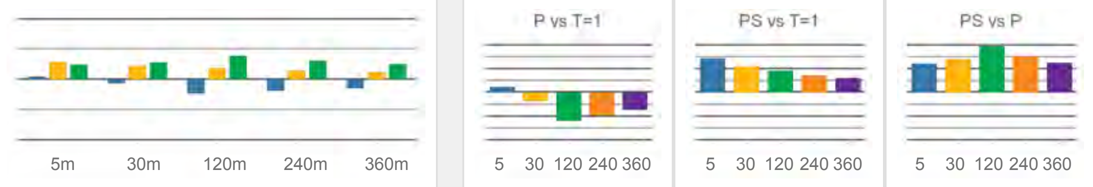

PGN_0779

Locus	5m	30m	120m	240m	360m
P vs T=1	-0.357	-1.025	-1.302	-1.255	-0.535
PS vs T=1	-1.047	-1.104	-0.279	-0.355	-0.908
PS vs P	-0.629	-0.110	0.905	0.757	-0.318

probable uracil phosphoribosyltransferase

purines, pyrimidines, nucleosides and nucleotides

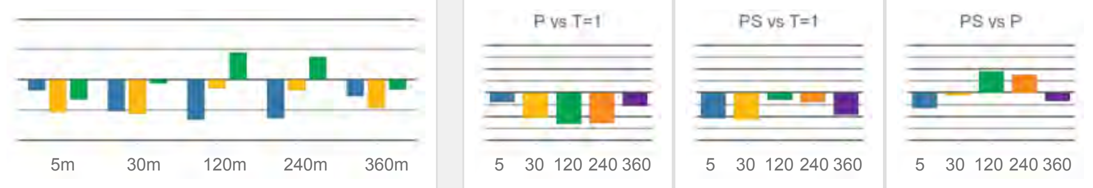

PGN_0780

prtQ

Locus	5m	30m	120m	240m	360m
P vs T=1	-1.130	-1.274	-1.145	-0.429	0.291
PS vs T=1	-1.223	-1.549	-1.141	-0.469	-0.535
PS vs P	-0.120	-0.328	-0.083	0.027	-0.718

PrtQ, protease

protein fate

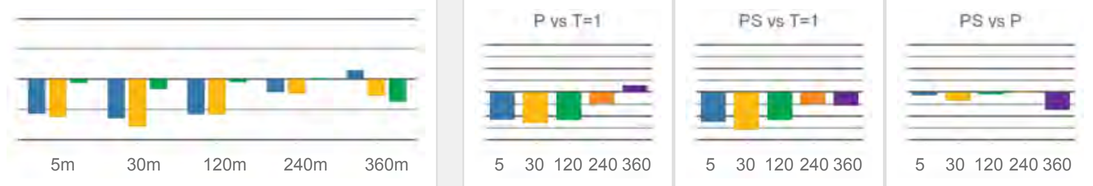

PGN_0781

Locus	5m	30m	120m	240m	360m
P vs T=1	-0.847	-1.222	-1.483	-0.977	-0.303
PS vs T=1	-1.266	-1.251	-0.878	-0.596	-0.722
PS vs P	-0.401	-0.046	0.532	0.385	-0.375

DNA topoisomerase I

DNA metabolism

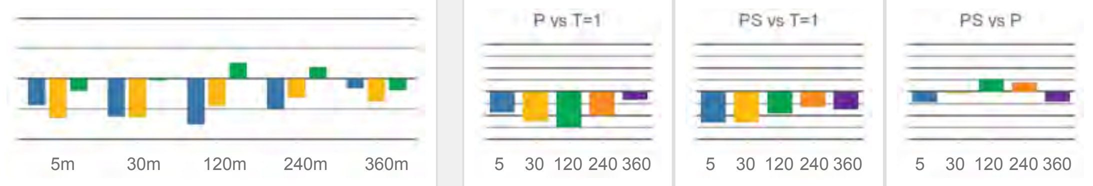

PGN_0782

Locus	5m	30m	120m	240m	360m
P vs T=1	-0.952	-1.293	-1.021	-0.044	0.582
PS vs T=1	-1.558	-1.522	-1.090	-1.125	-0.374
PS vs P	-0.585	-0.322	-0.172	-0.781	-0.723

putative tRNA pseudouridine synthase A

protein synthesis

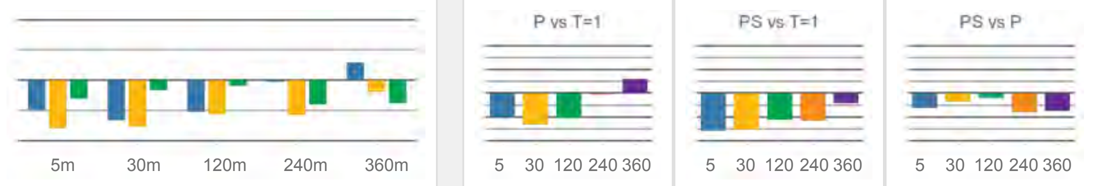

PGN_0783

Locus	5m	30m	120m	240m	360m
P vs T=1	-2.665	-2.940	-3.100	-3.400	-3.844
PS vs T=1	-3.123	-3.182	-3.807	-4.309	-4.396
PS vs P	-0.406	-0.225	-0.668	-0.910	-0.662

putative DNA-binding protein histone-like family

hypothetical proteins-Conserved

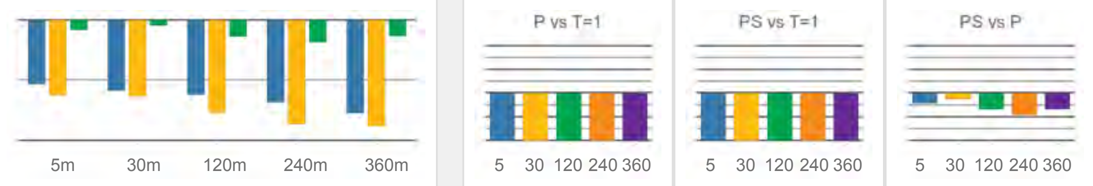

PGN_0784

Locus	5m	30m	120m	240m	360m
P vs T=1	-2.142	-2.389	-2.940	-3.713	-3.879
PS vs T=1	-2.551	-2.481	-2.794	-3.650	-4.146
PS vs P	-0.326	-0.045	0.140	-0.098	-0.390

hypothetical protein

hypothetical proteins

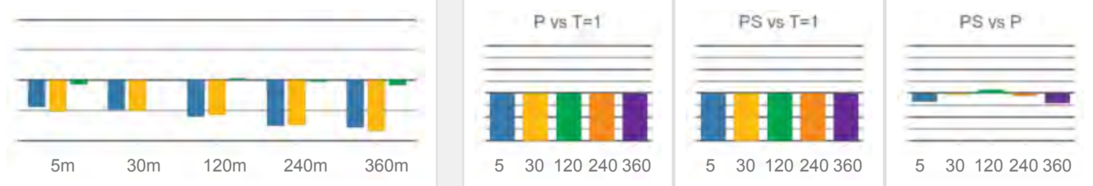

PgSg Time Course

log$_2$ (Fold Change)

P vs T=1 | PS vs T=1 | PS vs P

Locus		5m	30m	120m	240m	360m
**PGN_0785**	P vs T=1	-1.846	-1.904	-2.106	-2.742	-2.818
	PS vs T=1	-1.804	-1.713	-2.052	-2.624	-2.541
	PS vs P	0.039	0.185	0.058	-0.081	0.128

hypothetical protein

hypothetical proteins

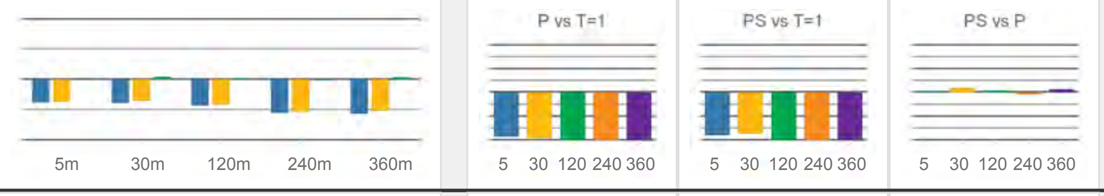

Locus		5m	30m	120m	240m	360m
**PGN_0786**	P vs T=1	0.868	0.726	0.367	-0.021	-0.079
	PS vs T=1	1.660	1.150	0.073	-0.298	-0.356
	PS vs P	0.804	0.433	-0.285	-0.306	-0.288

conserved hypothetical protein

hypothetical proteins-Conserved

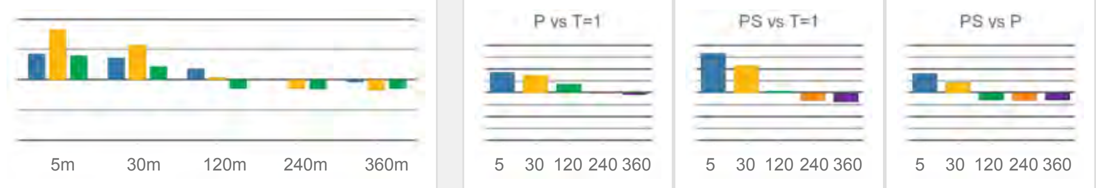

Locus		5m	30m	120m	240m	360m
**PGN_0787**	P vs T=1	0.563	0.457	0.463	0.238	0.463
	PS vs T=1	1.506	1.048	0.292	-0.010	-0.036
	PS vs P	0.933	0.573	-0.154	-0.268	-0.469

conserved hypothetical protein

hypothetical proteins-Conserved

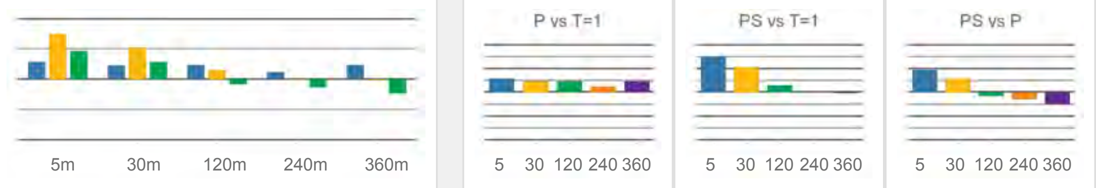

Locus		5m	30m	120m	240m	360m
**PGN_0788**	P vs T=1	1.300	2.009	2.532	2.270	1.706
	PS vs T=1	2.131	2.409	2.376	2.455	2.083
	PS vs P	0.764	0.403	-0.110	0.202	0.362

peptidyl-dipeptidase

protein fate

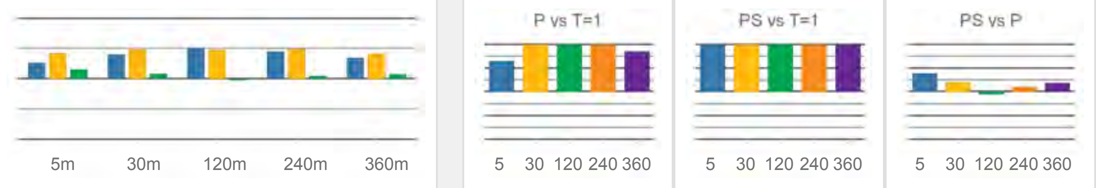

Locus		5m	30m	120m	240m	360m
**PGN_0789**	P vs T=1	0.158	0.609	0.629	0.182	-0.250
	PS vs T=1	0.524	0.468	0.343	0.633	0.256
	PS vs P	0.350	-0.126	-0.269	0.437	0.481

conserved hypothetical protein

unknown function

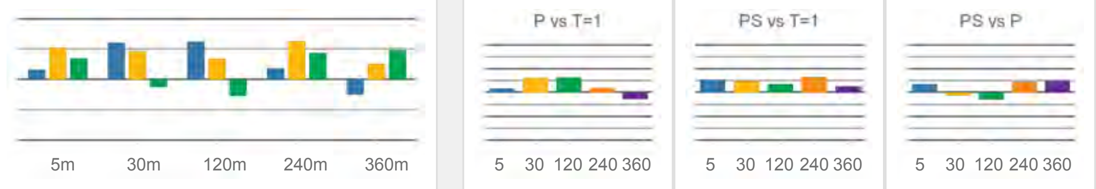

Locus		5m	30m	120m	240m	360m
**PGN_0790**	P vs T=1	0.318	-0.260	-0.348	0.491	1.553
	PS vs T=1	-0.185	-0.059	0.427	0.564	0.578
	PS vs P	-0.456	0.087	0.547	0.106	-0.805

transposase in ISPg3

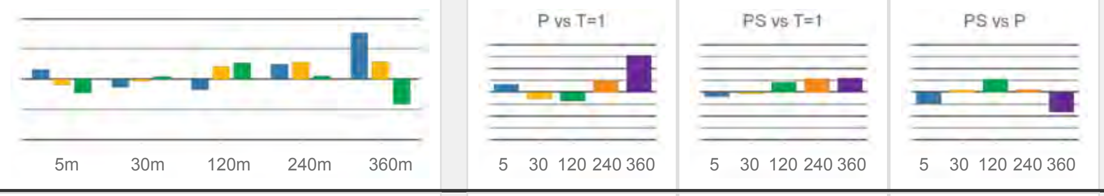

Locus		5m	30m	120m	240m	360m
**PGN_0791**	P vs T=1	1.132	0.861	0.577	0.293	-0.156
	PS vs T=1	0.253	-0.065	-0.339	-0.308	-0.259
	PS vs P	-0.807	-0.872	-0.881	-0.583	-0.113

putative cell division trigger factor

protein fate

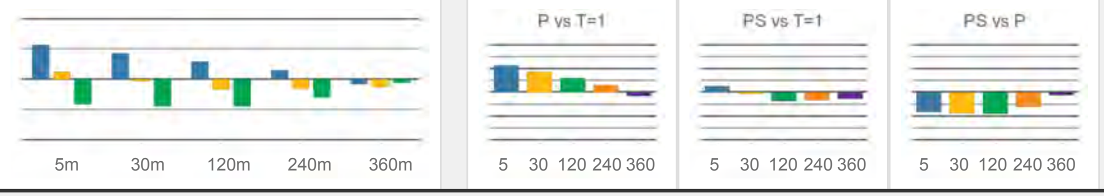

PgSg Time Course

log₂ (Fold Change)

P vs T=1 | PS vs T=1 | PS vs P

Locus		5m	30m	120m	240m	360m
**PGN_0792**	P vs T=1	0.379	1.043	1.730	1.541	0.871
	PS vs T=1	0.478	0.519	0.712	0.792	0.615
	PS vs P	0.071	-0.499	-0.957	-0.707	-0.255
	polyribonucleotide nucleotidyltransferase					
	transcription					
**PGN_0793**	P vs T=1	-0.353	-0.363	-0.747	-0.664	-0.234
	PS vs T=1	-0.018	-0.573	-1.245	-1.273	-1.048
	PS vs P	0.337	-0.201	-0.511	-0.608	-0.790
	4-alpha-glucanotransferase					
	energy metabolism					
**PGN_0794**	P vs T=1	-0.145	-0.186	-0.405	-0.636	-0.395
	PS vs T=1	0.120	-0.273	-0.518	-0.360	-0.643
	PS vs P	0.270	-0.078	-0.118	0.225	-0.245
	conserved hypothetical protein					
	hypothetical proteins-Conserved					
**PGN_0795**	P vs T=1	0.239	0.591	0.332	-0.192	-0.494
	PS vs T=1	0.328	0.503	-0.128	-0.825	-0.906
	PS vs P	0.091	-0.063	-0.441	-0.639	-0.425
	conserved hypothetical protein					
	unknown function					
**PGN_0796**	P vs T=1	0.746	0.613	1.324	0.764	3.135
	PS vs T=1	0.010	0.192	1.229	1.825	2.150
	PS vs P	-0.542	-0.391	0.101	0.433	0.091
	hypothetical protein					
	hypothetical proteins					
**PGN_0797**	P vs T=1	0.138	-0.447	1.012	1.001	2.746
	PS vs T=1	-0.827	-0.594	0.757	2.374	2.445
	PS vs P	-0.795	-0.565	-0.008	1.099	0.542
	conserved hypothetical protein					
	hypothetical proteins-Conserved					
**PGN_0798**	P vs T=1	-0.473	-0.792	-0.665	-0.279	-0.263
	PS vs T=1	-0.023	0.975	2.473	2.611	2.321
	PS vs P	0.335	1.546	2.904	2.712	2.462
	conserved hypothetical protein					
	hypothetical proteins-Conserved					

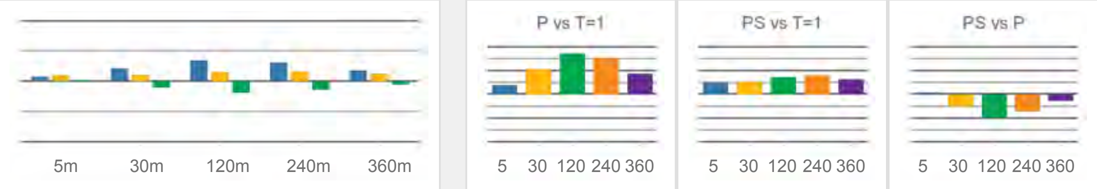

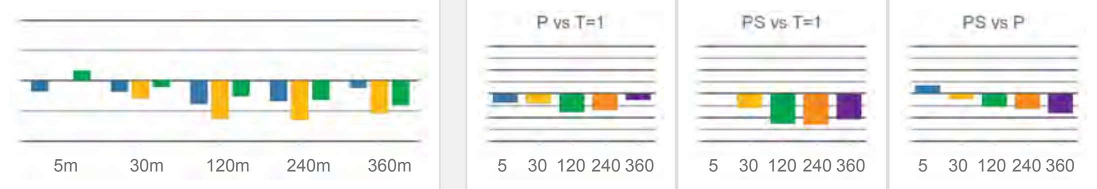

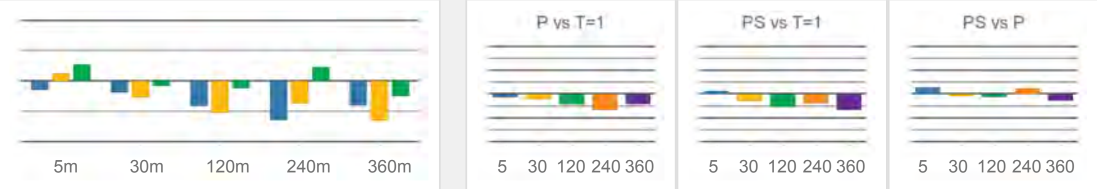

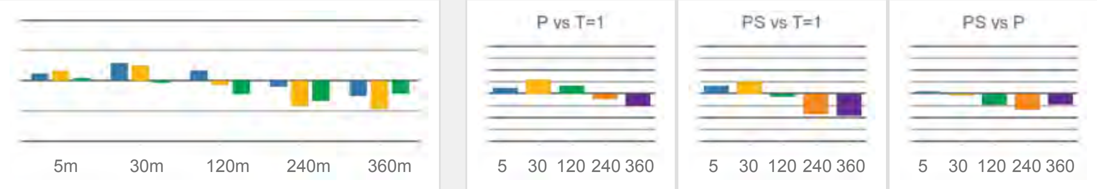

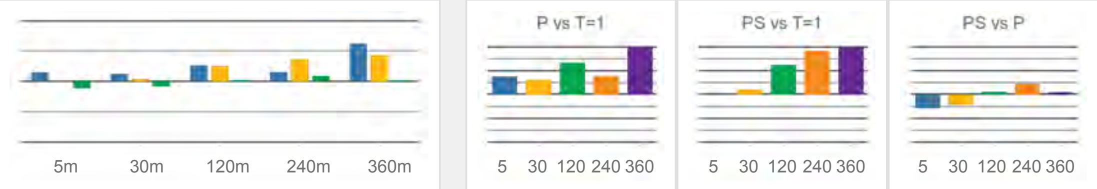

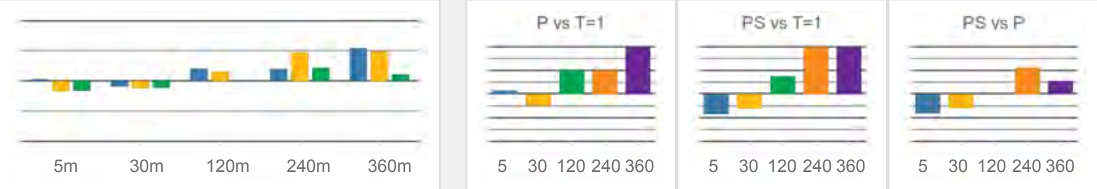

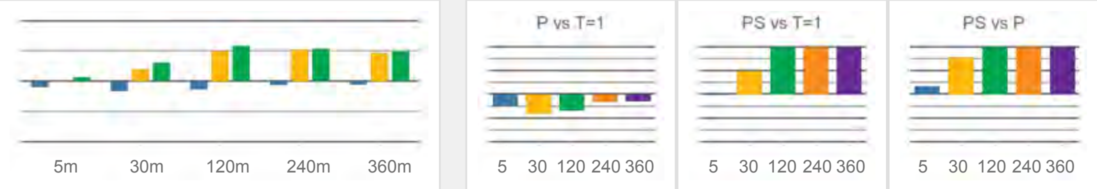

PgSg Time Course

log$_2$ (Fold Change)

P vs T=1 PS vs T=1 PS vs P

Locus		5m	30m	120m	240m	360m
PGN_0799	P vs T=1	0.819	1.016	0.751	0.207	-0.451
	PS vs T=1	0.824	1.236	1.308	0.937	0.588
	PS vs P	0.010	0.245	0.563	0.680	0.961

putative acyl-CoA dehydrogenase

fatty acid and phospholipid metabolism

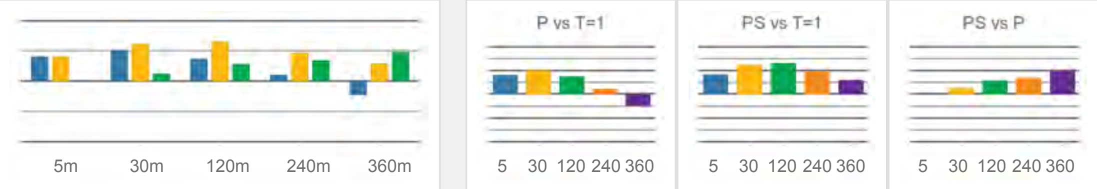

Locus		5m	30m	120m	240m	360m
PGN_0800	P vs T=1	1.570	1.686	1.340	0.748	0.246
	PS vs T=1	2.016	2.745	3.014	2.484	1.956
	PS vs P	0.414	1.033	1.613	1.558	1.533

putative electron transfer flavoprotein alpha subunit

energy metabolism

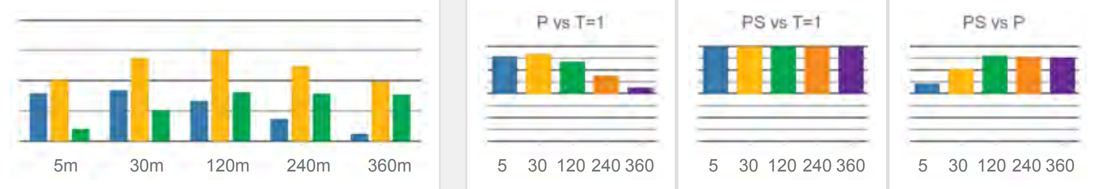

Locus		5m	30m	120m	240m	360m
PGN_0801	P vs T=1	1.206	1.381	1.089	0.494	0.024
	PS vs T=1	1.473	2.183	2.646	2.325	2.011
	PS vs P	0.231	0.786	1.500	1.639	1.797

probable electron transfer flavoprotein beta subunit

energy metabolism

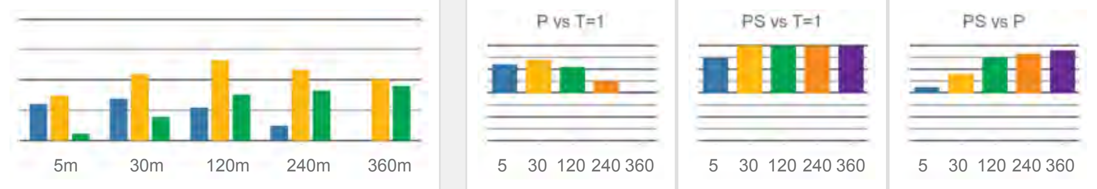

Locus		5m	30m	120m	240m	360m
PGN_0802	P vs T=1	0.672	0.052	-0.472	-0.771	-1.013
	PS vs T=1	1.438	1.826	2.112	1.230	0.364
	PS vs P	0.759	1.576	2.324	1.671	1.114

probable glycoprotease

hypothetical proteins-Conserved

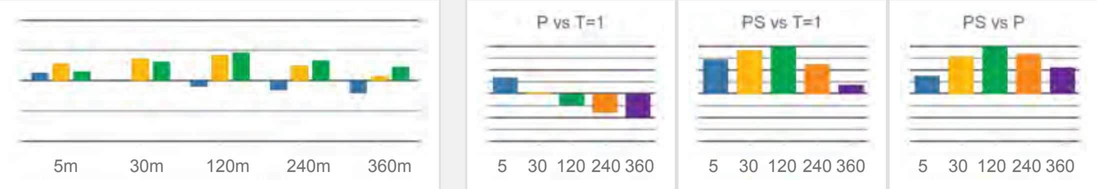

Locus		5m	30m	120m	240m	360m
PGN_0803	P vs T=1	0.721	1.009	1.524	1.329	0.362
	PS vs T=1	0.492	0.889	1.394	1.420	1.531
	PS vs P	-0.232	-0.095	-0.056	0.134	1.125

conserved hypothetical protein

hypothetical proteins-Conserved

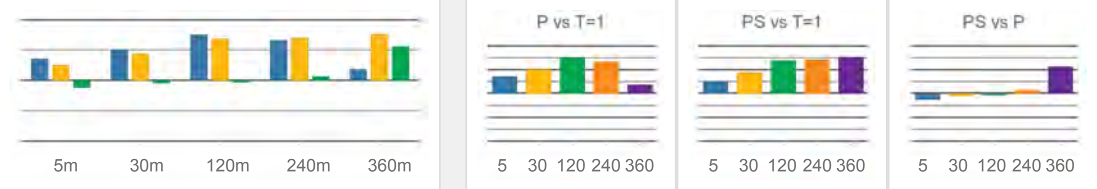

Locus		5m	30m	120m	240m	360m
PGN_0804	P vs T=1	0.560	0.773	0.884	0.302	-0.808
	PS vs T=1	0.410	0.760	0.905	0.646	0.460
	PS vs P	-0.136	0.021	0.070	0.339	1.200

conserved hypothetical protein

hypothetical proteins-Conserved

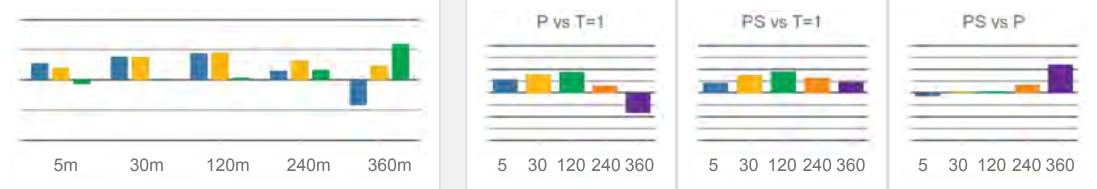

Locus		5m	30m	120m	240m	360m
PGN_0805	P vs T=1	0.864	1.076	0.959	0.336	-0.733
	PS vs T=1	0.555	0.791	0.814	0.588	0.476
	PS vs P	-0.279	-0.234	-0.102	0.243	1.143

conserved hypothetical protein

hypothetical proteins-Conserved

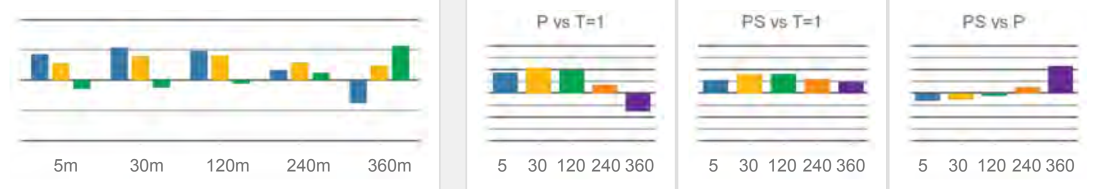

PgSg Time Course

Hackett Laboratory UW

log2 (Fold Change)

Legend: P vs T=1 | PS vs T=1 | PS vs P

Locus		5m	30m	120m	240m	360m
PGN_0806	P vs T=1	1.059	1.263	0.653	-0.347	-1.045
	PS vs T=1	0.528	0.827	0.686	0.283	0.059
	PS vs P	-0.498	-0.386	0.054	0.593	1.058

putative MotA/TolQ/ExbB proton channel protein

transport and binding proteins

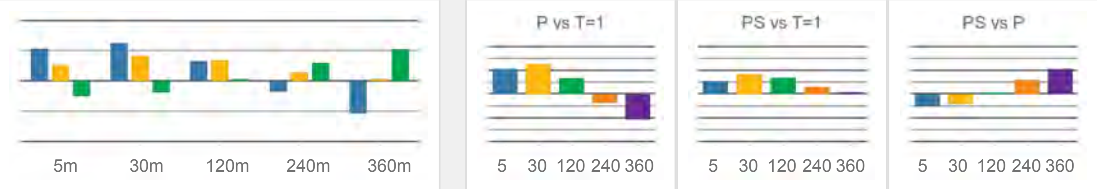

Locus		5m	30m	120m	240m	360m
PGN_0807	P vs T=1	-0.119	-0.497	-0.798	-1.107	-0.531
	PS vs T=1	0.471	0.529	0.569	0.383	-0.127
	PS vs P	0.581	0.949	1.242	1.216	0.370

putative DNAse related protein

hypothetical proteins-Conserved

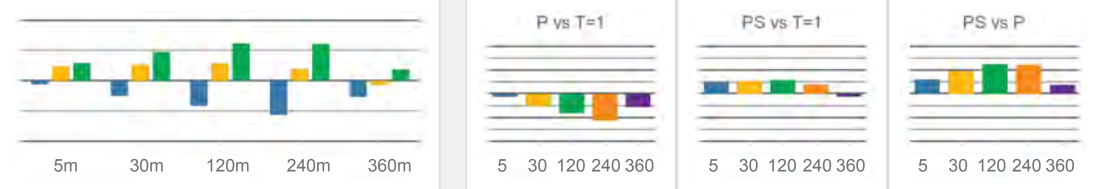

Locus		5m	30m	120m	240m	360m
PGN_0808	P vs T=1	-0.255	-0.494	-1.132	-0.723	-0.397
	PS vs T=1	0.119	0.446	0.557	0.646	0.235
	PS vs P	0.347	0.855	1.411	1.221	0.595

probable isoprenyl synthetase

central intermediary metabolism

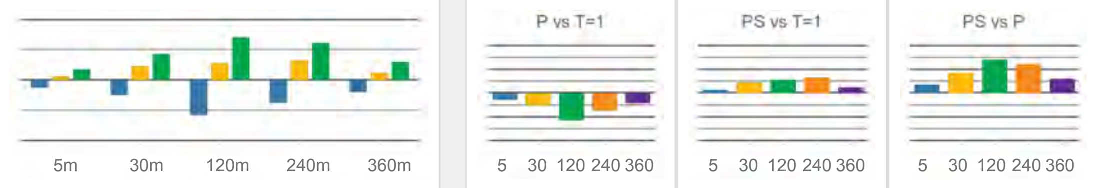

Locus		5m	30m	120m	240m	360m
PGN_0809	P vs T=1	-0.719	-0.262	0.059	0.181	-0.259
	PS vs T=1	-0.646	-0.095	0.525	1.387	1.244
	PS vs P	0.034	0.156	0.463	1.201	1.478

putative TonB protein

transport and binding proteins

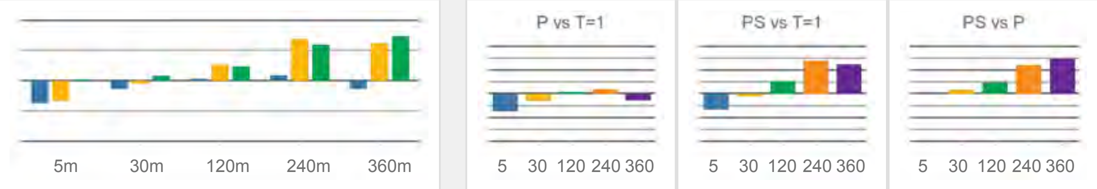

Locus		5m	30m	120m	240m	360m
PGN_0810	P vs T=1	-0.213	-0.205	0.467	0.326	1.502
	PS vs T=1	-0.153	-0.303	-0.638	-0.479	0.019
	PS vs P	0.021	-0.146	-0.807	-0.622	-0.968

conserved hypothetical protein

hypothetical proteins-Conserved

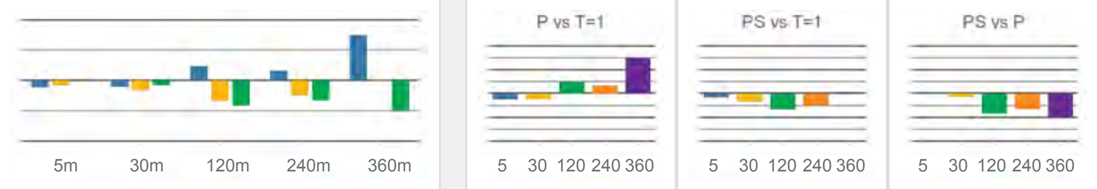

Locus		5m	30m	120m	240m	360m
PGN_0811	P vs T=1	-0.843	-0.889	-0.983	-0.622	-0.226
	PS vs T=1	-0.055	-0.514	-0.872	-0.871	-0.685
	PS vs P	0.768	0.355	0.078	-0.237	-0.432

conserved hypothetical protein

hypothetical proteins-Conserved

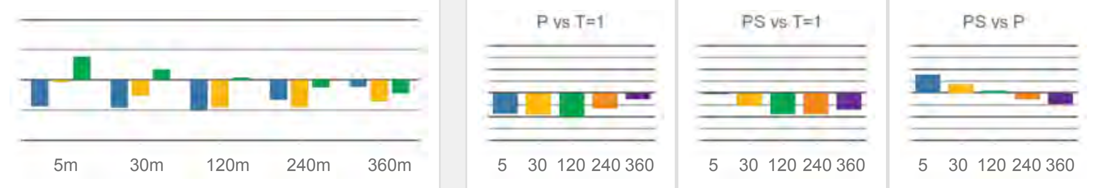

Locus		5m	30m	120m	240m	360m
PGN_0812	P vs T=1	-1.178	-1.003	-1.264	-1.055	-0.574
	PS vs T=1	-0.749	-0.941	-1.472	-1.675	-1.294
	PS vs P	0.398	0.060	-0.267	-0.613	-0.656

conserved hypothetical protein

hypothetical proteins-Conserved

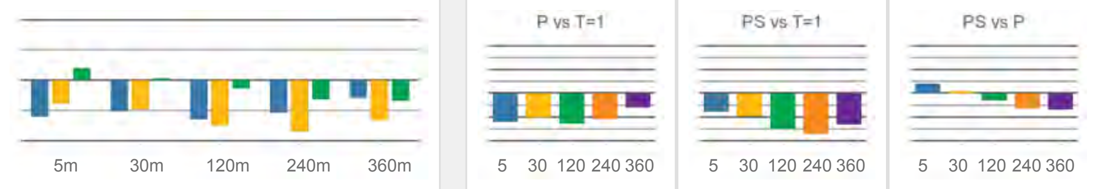

log2 (Fold Change)

Locus		5m	30m	120m	240m	360m
PGN_0813	P vs T=1	-1.419	-1.209	-0.944	-0.877	-0.993
	PS vs T=1	-1.105	-1.292	-1.701	-1.729	-1.705
	PS vs P	0.253	-0.097	-0.723	-0.802	-0.698

GTP-binding protein

unknown function

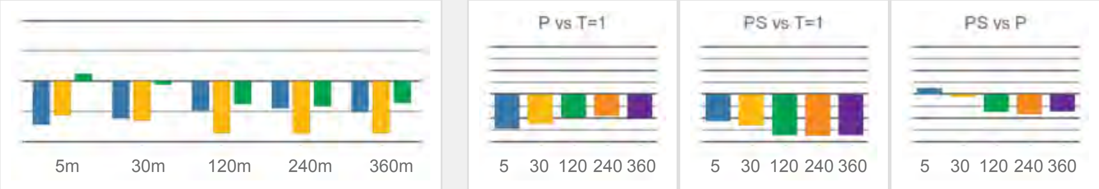

Locus		5m	30m	120m	240m	360m
PGN_0814	P vs T=1	-0.136	0.146	0.237	-0.028	-0.540
	PS vs T=1	-0.272	-0.161	-0.445	-0.643	-0.777
	PS vs P	-0.135	-0.282	-0.645	-0.592	-0.254

probable adenylate kinase

purines, pyrimidines, nucleosides and nucleotides

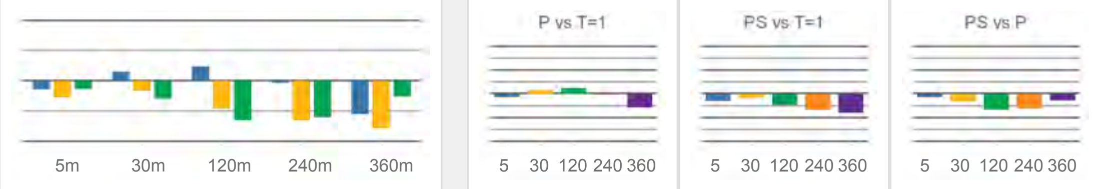

Locus		5m	30m	120m	240m	360m
PGN_0815	P vs T=1	-0.454	-1.013	-1.498	-1.751	-1.931
	PS vs T=1	-0.591	-0.509	-0.352	-0.029	-0.380
	PS vs P	-0.094	0.460	1.019	1.487	1.365

putative hypoxanthine phosphoribosyltransferase

purines, pyrimidines, nucleosides and nucleotides

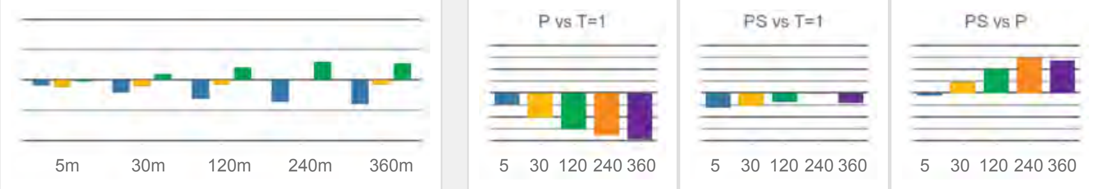

Locus		5m	30m	120m	240m	360m
PGN_0816	P vs T=1	0.092	-0.387	-1.464	-2.107	-2.507
	PS vs T=1	-0.412	-1.082	-1.527	-1.884	-2.001
	PS vs P	-0.454	-0.661	-0.074	0.175	0.460

fructose-1,6-bisphosphatase

energy metabolism

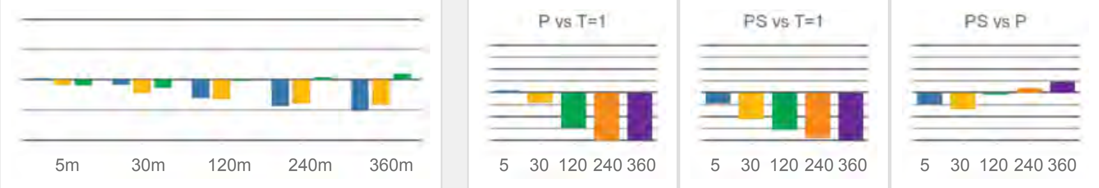

Locus		5m	30m	120m	240m	360m
PGN_0817	P vs T=1	0.557	0.304	-0.686	-1.426	-1.762
	PS vs T=1	0.813	0.345	-0.962	-1.779	-1.974
	PS vs P	0.286	0.067	-0.277	-0.392	-0.245

penicillin-binding protein 1A

cell envelope

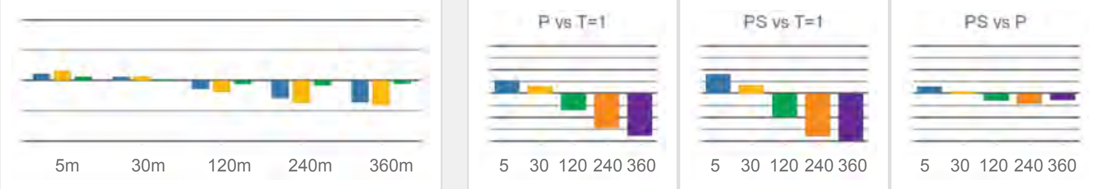

Locus		5m	30m	120m	240m	360m
PGN_0818	P vs T=1	0.962	1.661	2.037	1.685	0.912
	PS vs T=1	1.353	2.158	2.256	1.647	1.709
	PS vs P	0.347	0.510	0.262	-0.023	0.767

conserved hypothetical protein

hypothetical proteins-Conserved

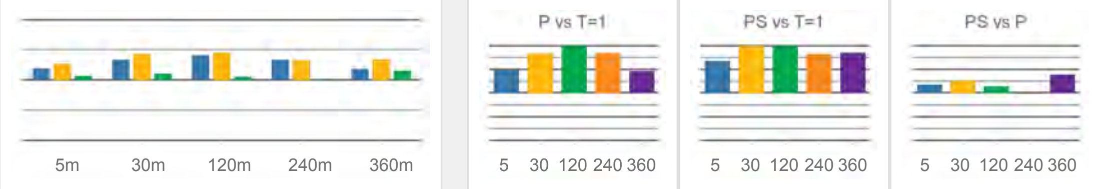

Locus		5m	30m	120m	240m	360m
PGN_0819	P vs T=1	0.081	0.198	-0.162	-0.588	-0.673
	PS vs T=1	-0.079	-0.385	-0.423	-0.404	-0.574
	PS vs P	-0.146	-0.554	-0.253	0.158	0.083

leucyl-tRNA synthetase

protein synthesis

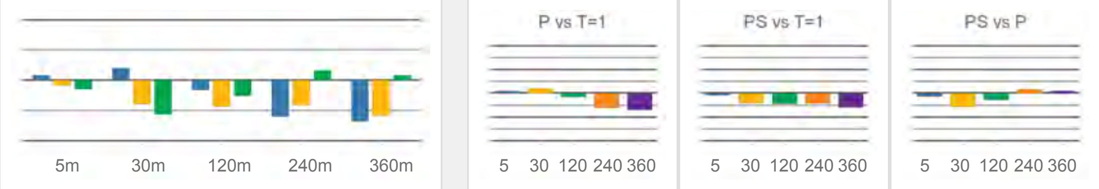

log2 (Fold Change)

Legend: P vs T=1 | PS vs T=1 | PS vs P

Locus		5m	30m	120m	240m	360m
PGN_0820	P vs T=1	2.066	1.744	1.299	1.569	1.442
	PS vs T=1	1.151	1.416	2.018	2.078	1.880
	PS vs P	-0.759	-0.252	0.588	0.515	0.414
	hypothetical protein					
	hypothetical proteins					
PGN_0821	P vs T=1	0.226	-0.103	-0.437	1.588	3.310
	PS vs T=1	-0.654	-0.196	0.737	1.066	1.711
	PS vs P	-0.699	-0.247	0.312	0.118	-0.589
	hypothetical protein					
	hypothetical proteins					
PGN_0822	P vs T=1	0.064	-0.073	-0.361	0.851	2.787
	PS vs T=1	-1.040	-0.303	0.363	0.652	1.799
	PS vs P	-0.861	-0.264	0.109	0.045	0.007
	conserved hypothetical protein					
	hypothetical proteins-Conserved					
PGN_0823	P vs T=1	-0.330	-0.337	-0.453	-0.346	0.274
	PS vs T=1	0.175	-0.220	-0.568	-0.774	-0.465
	PS vs P	0.494	0.112	-0.134	-0.423	-0.676
	NAD-utilizing dehydrogenases					
	hypothetical proteins-Conserved					
PGN_0824	P vs T=1	0.067	-0.613	-0.810	-0.460	0.268
	PS vs T=1	0.154	-0.278	-0.619	-0.767	-0.912
	PS vs P	0.115	0.309	0.137	-0.294	-1.111
	tRNA nucleotidyltransferase					
	transcription					
PGN_0825	P vs T=1	0.693	0.494	0.436	1.339	1.819
	PS vs T=1	1.275	0.883	0.555	0.823	0.440
	PS vs P	0.552	0.283	-0.090	-0.199	-0.954
	conserved hypothetical protein					
	hypothetical proteins-Conserved					
PGN_0826	P vs T=1	0.848	1.170	1.539	1.497	1.172
	PS vs T=1	2.157	2.210	2.063	1.729	1.623
	PS vs P	1.202	0.981	0.537	0.239	0.426
	dihydrolipoamide dehydrogenase					
	energy metabolism					

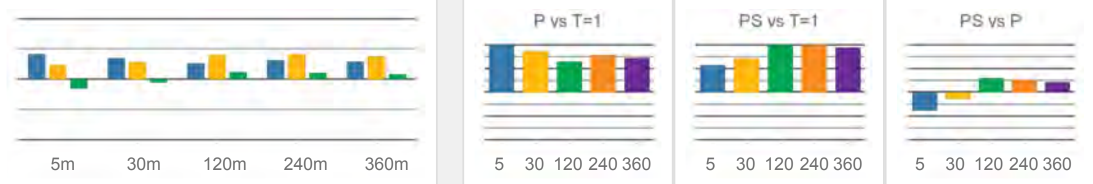

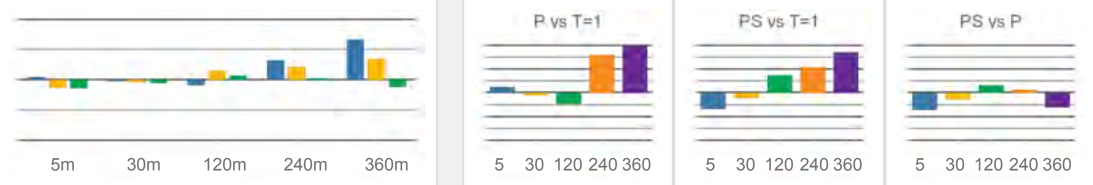

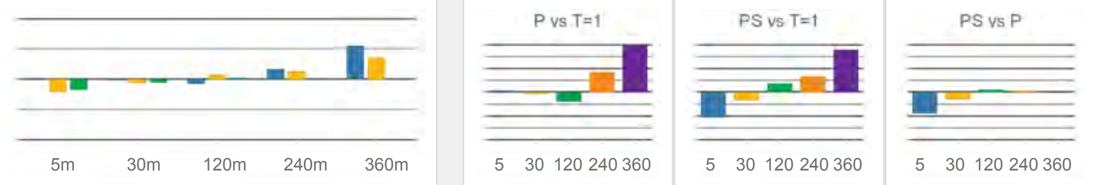

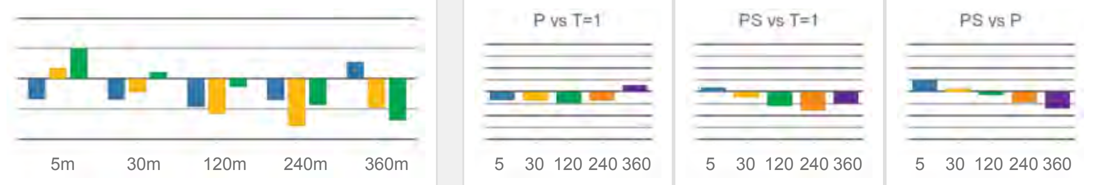

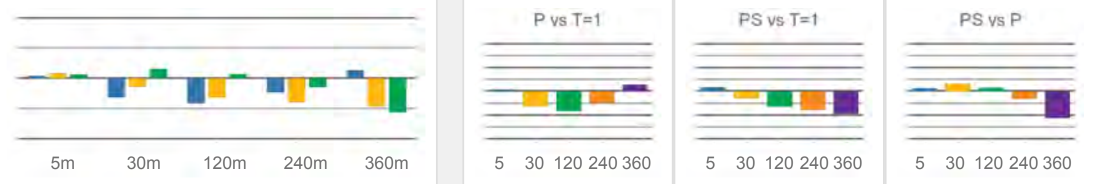

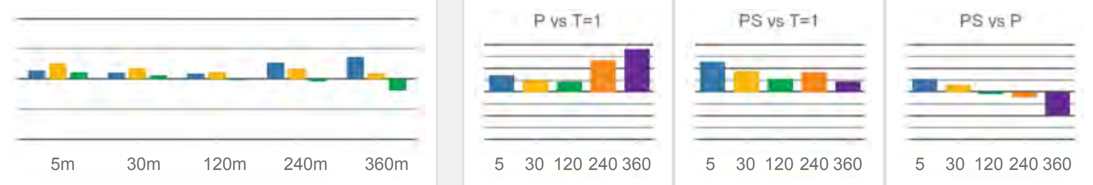

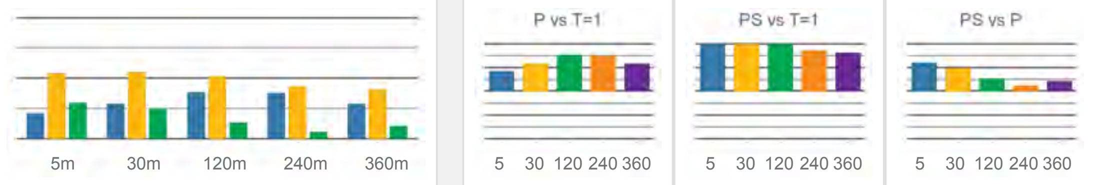

PgSg Time Course

log$_2$ (Fold Change)

Legend: P vs T=1 | PS vs T=1 | PS vs P

Locus		5m	30m	120m	240m	360m
**PGN_0827**	P vs T=1	0.207	0.120	-0.373	-0.798	-0.969
	PS vs T=1	1.263	0.827	0.153	-0.592	-0.735
	PS vs P	1.053	0.705	0.501	0.111	0.164

glucosamine-6-phosphate isomerase

central intermediary metabolism

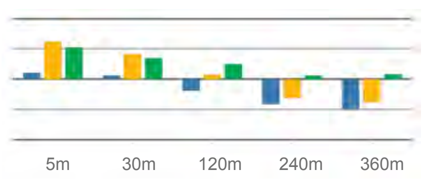

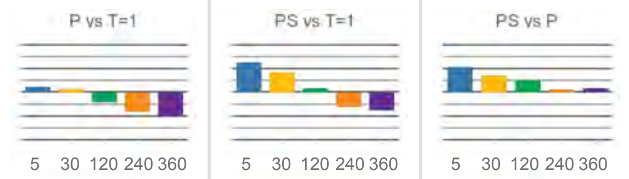

Locus		5m	30m	120m	240m	360m
**PGN_0828**	P vs T=1	-0.410	-0.599	-1.309	-1.757	-1.760
	PS vs T=1	0.007	-0.420	-1.215	-1.676	-1.837
	PS vs P	0.426	0.187	0.078	0.014	-0.109

flavoprotein

energy metabolism

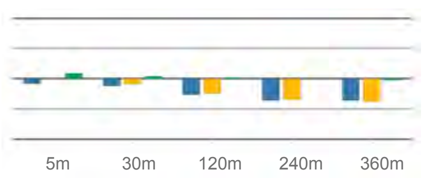

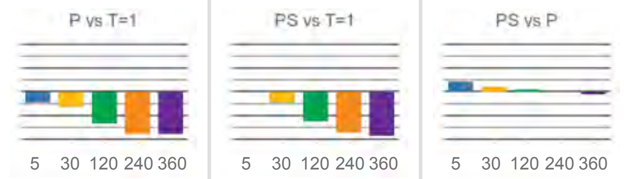

Locus		5m	30m	120m	240m	360m
**PGN_0829**	P vs T=1	-1.123	-1.342	-1.482	-1.216	-0.519
	PS vs T=1	-0.892	-1.393	-1.720	-1.657	-1.194
	PS vs P	0.232	-0.073	-0.302	-0.436	-0.589

putative prolipoprotein diacylglyceryl transferase

protein fate

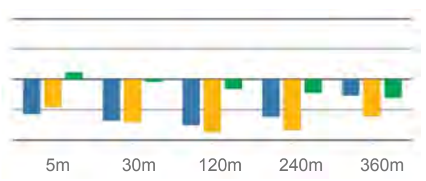

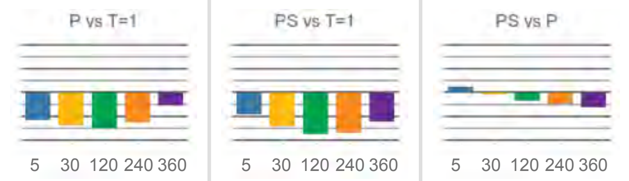

Locus		5m	30m	120m	240m	360m
**PGN_0830**	P vs T=1	-0.562	-0.780	-1.388	-1.229	-1.144
	PS vs T=1	-0.913	-1.508	-2.104	-1.743	-2.038
	PS vs P	-0.297	-0.677	-0.739	-0.487	-0.867

meso-diaminopimelate D-dehydrogenase

unknown function

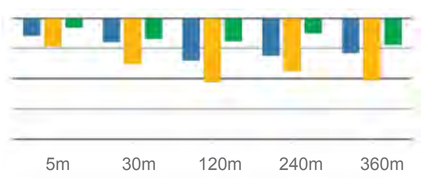

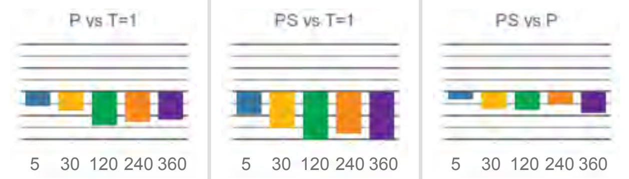

Locus		5m	30m	120m	240m	360m
**PGN_0831**	P vs T=1	-0.288	-0.682	-0.849	-0.877	-0.861
	PS vs T=1	-1.067	-1.279	-1.436	-1.206	-1.305
	PS vs P	-0.714	-0.570	-0.572	-0.311	-0.432

probable nitrogen utilization substance protein

transcription

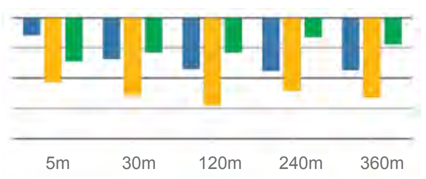

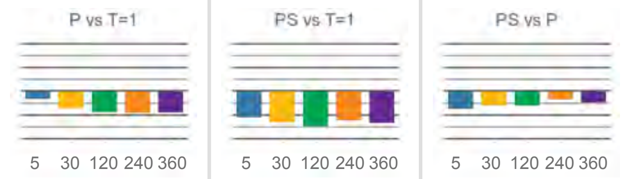

Locus		5m	30m	120m	240m	360m
**PGN_0832** *sov*	P vs T=1	-0.353	-0.810	-1.592	-1.559	-0.996
	PS vs T=1	0.626	0.317	-0.557	-1.099	-1.211
	PS vs P	0.980	1.114	0.997	0.424	-0.218

Por secretion system protein sov/gliding motility protein SprA

hypothetical proteins

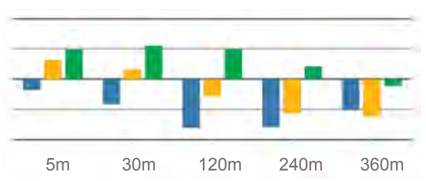

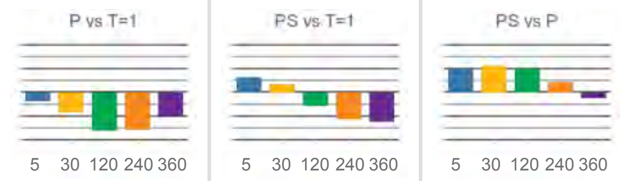

Locus		5m	30m	120m	240m	360m
**PGN_0833** *ruvA*	P vs T=1	0.562	0.490	0.377	0.287	-0.117
	PS vs T=1	1.370	1.320	1.074	0.722	0.600
	PS vs P	0.797	0.814	0.680	0.410	0.675

probable Holliday junction DNA helicase RuvA

DNA metabolism

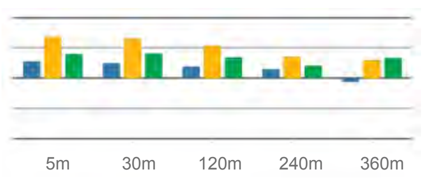

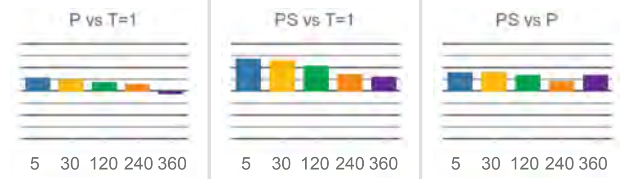

PgSg Time Course

log2 (Fold Change)

P vs T=1 | PS vs T=1 | PS vs P

Locus		5m	30m	120m	240m	360m
**PGN_0834**	P vs T=1	0.345	0.246	0.305	0.422	0.100
	PS vs T=1	0.176	-0.010	0.084	0.170	0.222
	PS vs P	-0.167	-0.262	-0.228	-0.249	0.105
	transposase in ISPg1					
**PGN_0835**	P vs T=1	-0.544	-1.570	-1.812	-1.956	0.390
	PS vs T=1	-0.240	-0.526	-0.756	-0.109	0.334
	PS vs P	0.336	0.389	0.167	0.552	0.405
	conserved hypothetical protein<br>*hypothetical proteins-Conserved*					
**PGN_0836**	P vs T=1	0.051	-1.139	-2.031	-2.164	-1.528
	PS vs T=1	-0.423	-1.055	-1.464	-1.789	-1.858
	PS vs P	-0.291	0.051	0.410	0.208	-0.348
	conserved hypothetical protein<br>*hypothetical proteins-Conserved*					
**PGN_0837**	P vs T=1	0.222	0.028	0.114	-0.283	-1.164
	PS vs T=1	-0.283	-0.195	-0.593	-0.903	-0.827
	PS vs P	-0.442	-0.197	-0.649	-0.609	0.254
	conserved hypothetical protein<br>*hypothetical proteins-Conserved*					
**PGN_0838**	P vs T=1	-0.114	0.122	0.628	0.654	-0.040
	PS vs T=1	-0.802	-0.530	-0.453	-0.323	-0.267
	PS vs P	-0.684	-0.601	-0.939	-0.837	-0.262
	conserved hypothetical protein<br>*hypothetical proteins-Conserved*					
**PGN_0839**	P vs T=1	0.114	-0.691	-1.036	-0.445	0.252
	PS vs T=1	0.471	-0.084	-0.356	-0.438	-0.441
	PS vs P	0.393	0.552	0.549	0.017	-0.622
	transposase in ISPg2					
**PGN_0840**	P vs T=1	-0.810	-0.775	-0.798	-0.639	-0.968
	PS vs T=1	-0.888	-0.893	0.066	0.508	0.593
	PS vs P	-0.136	-0.170	0.732	1.030	1.367
	conserved hypothetical protein<br>*unknown function*					

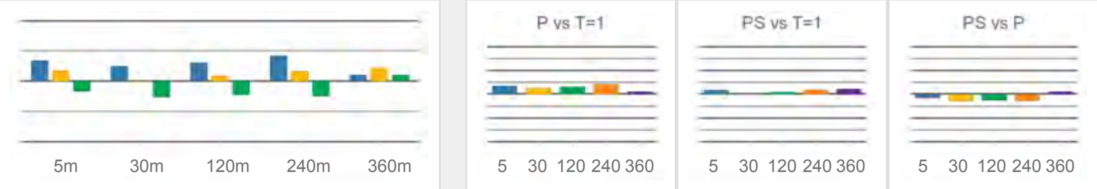

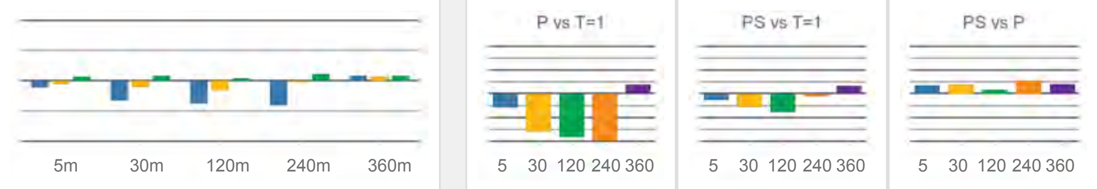

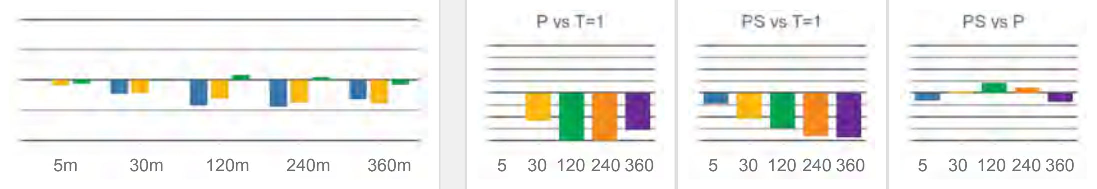

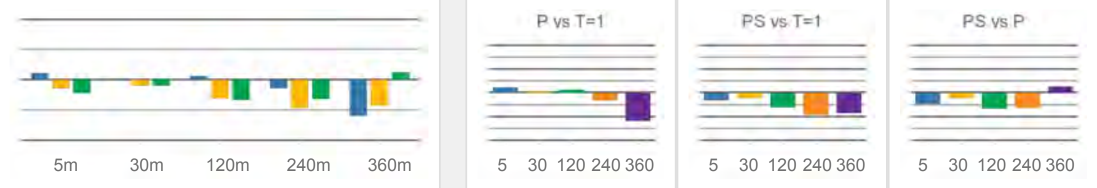

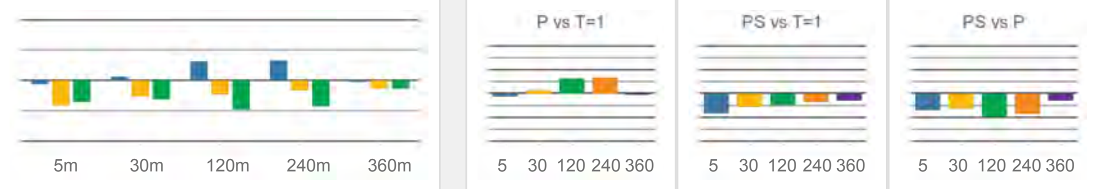

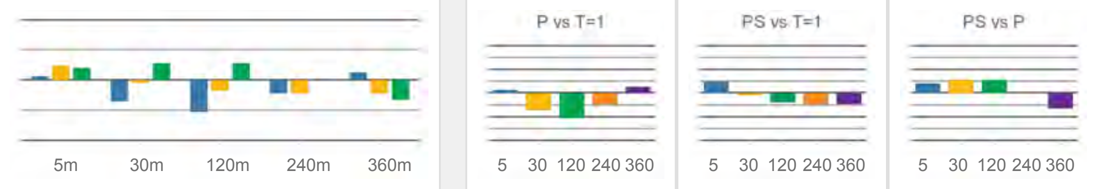

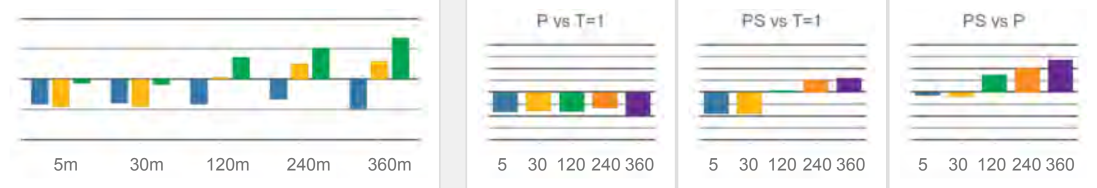

PgSg Time Course

log$_2$ (Fold Change)

Legend: P vs T=1 | PS vs T=1 | PS vs P

Locus		5m	30m	120m	240m	360m
PGN_0841	P vs T=1	-0.166	-0.920	-1.230	-1.289	-0.632
	PS vs T=1	-0.289	-0.796	-0.473	0.133	-0.093
	PS vs P	-0.074	0.061	0.614	1.203	0.521

putative 4-diphosphocytidyl-2C-methyl-D-erythritol synthase

biosynthesis of cofactors, prosthetic groups, and carriers

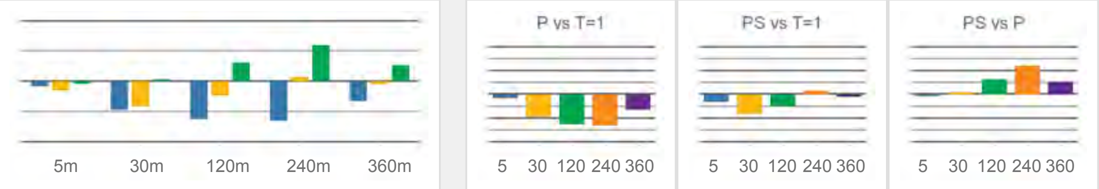

Locus		5m	30m	120m	240m	360m
PGN_0842	P vs T=1	0.343	0.240	0.307	0.402	0.082
	PS vs T=1	0.133	-0.025	0.119	0.163	0.248
	PS vs P	-0.208	-0.271	-0.194	-0.237	0.149

transposase in ISPg1

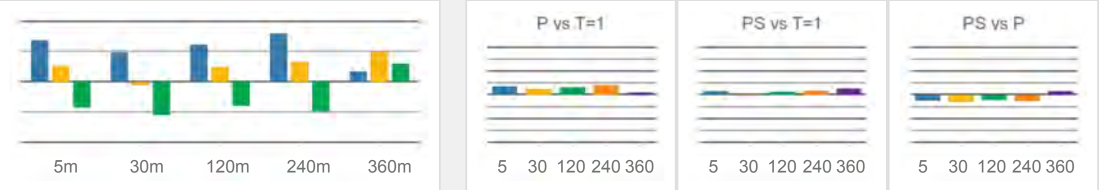

Locus		5m	30m	120m	240m	360m
PGN_0843	P vs T=1	-0.022	0.204	1.019	1.301	2.438
	PS vs T=1	-0.465	-0.374	0.449	0.883	1.372
	PS vs P	-0.505	-0.581	-0.390	-0.190	-0.701

conserved hypothetical protein

hypothetical proteins-Conserved

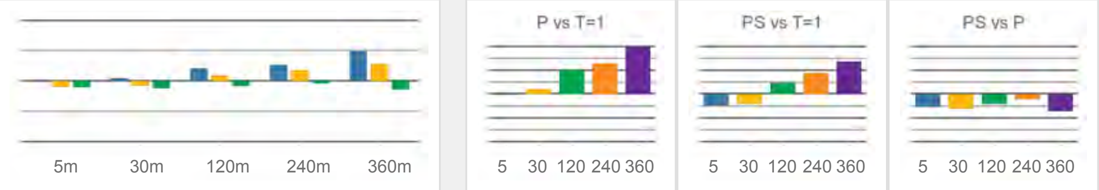

Locus		5m	30m	120m	240m	360m
PGN_0844	P vs T=1	-0.637	-0.123	0.426	0.895	1.165
	PS vs T=1	-1.112	-0.585	0.044	0.415	0.465
	PS vs P	-0.598	-0.459	-0.309	-0.254	-0.510

conserved hypothetical protein

hypothetical proteins-Conserved

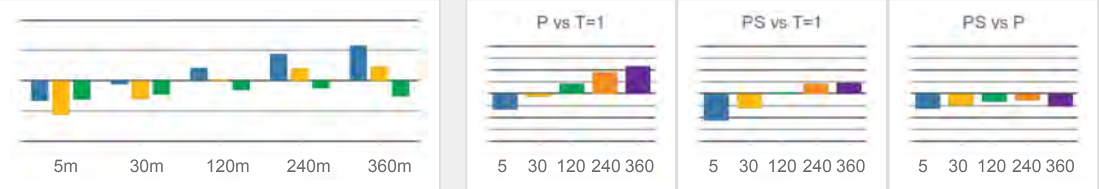

Locus		5m	30m	120m	240m	360m
PGN_0845	P vs T=1	0.824	0.510	1.144	1.621	3.719
	PS vs T=1	-0.146	-0.778	1.858	1.833	1.855
	PS vs P	-0.737	-1.016	0.693	0.359	-0.901

conserved hypothetical protein

hypothetical proteins-Conserved

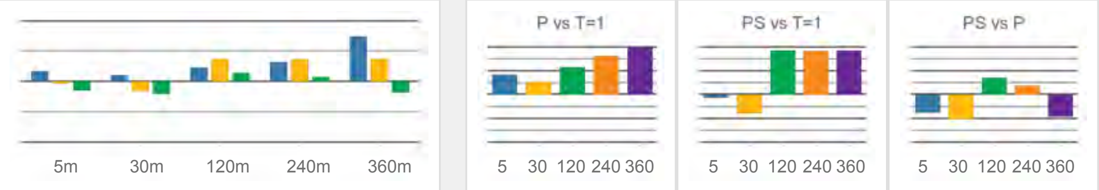

Locus		5m	30m	120m	240m	360m
PGN_0846	P vs T=1	0.414	-0.309	0.461	1.595	3.379
	PS vs T=1	-0.085	0.301	1.970	1.899	2.222
	PS vs P	-0.492	0.051	1.132	0.504	-0.571

conserved hypothetical protein

hypothetical proteins-Conserved

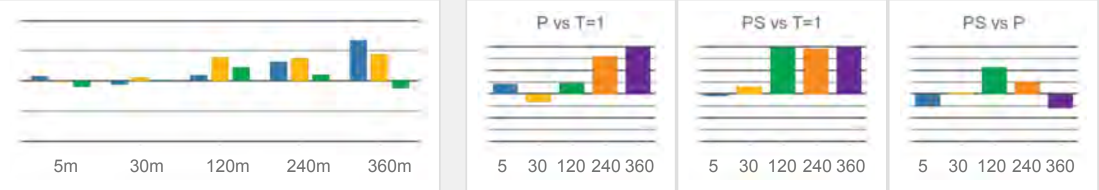

Locus		5m	30m	120m	240m	360m
PGN_0847	P vs T=1	-0.952	-1.635	-2.051	-0.183	1.311
	PS vs T=1	-0.814	-0.796	0.853	0.914	0.813
	PS vs P	-0.004	0.230	1.601	1.064	-0.085

conserved hypothetical protein

hypothetical proteins-Conserved

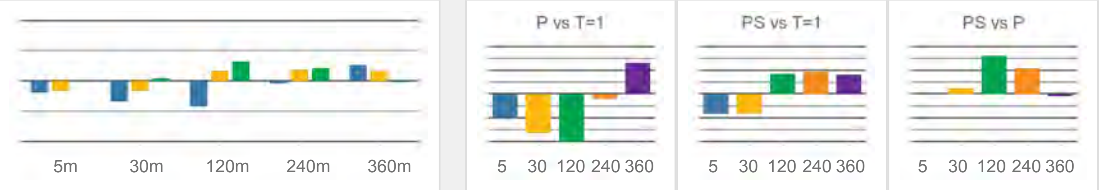

PgSg Time Course

log$_2$ (Fold Change)

Legend: P vs T=1, PS vs T=1, PS vs P

PGN_0848

Locus	5m	30m	120m	240m	360m
P vs T=1	-1.317	-1.967	-1.329	-0.644	-0.207
PS vs T=1	-1.332	-1.407	0.097	0.598	0.852
PS vs P	-0.161	-0.028	1.006	1.112	1.078

conserved hypothetical protein

hypothetical proteins-Conserved

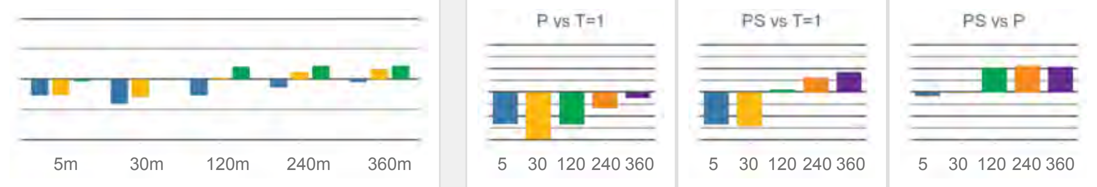

PGN_0849

Locus	5m	30m	120m	240m	360m
P vs T=1	-0.006	-0.565	-0.110	0.409	1.898
PS vs T=1	-0.035	0.334	1.215	1.131	1.575
PS vs P	-0.093	0.422	0.968	0.570	0.117

conserved hypothetical protein

hypothetical proteins-Conserved

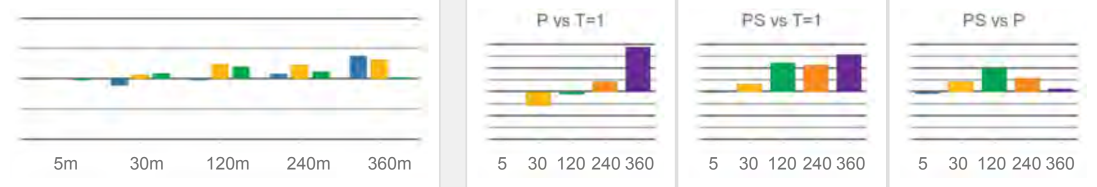

PGN_0850

Locus	5m	30m	120m	240m	360m
P vs T=1	-0.308	-0.883	-0.592	0.155	0.562
PS vs T=1	-0.348	-0.272	0.433	0.563	0.293
PS vs P	-0.044	0.168	0.674	0.560	0.095

conserved hypothetical protein

hypothetical proteins-Conserved

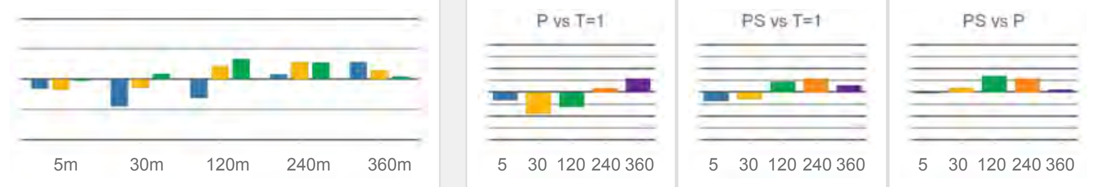

PGN_0851

Locus	5m	30m	120m	240m	360m
P vs T=1	-0.581	-0.897	-0.159	0.187	1.462
PS vs T=1	-0.560	-0.447	0.345	0.695	0.384
PS vs P	-0.071	0.141	0.476	0.552	-0.635

conserved hypothetical protein

hypothetical proteins-Conserved

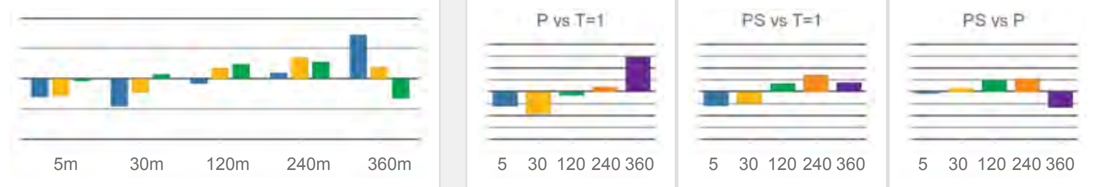

PGN_0852

Locus	5m	30m	120m	240m	360m
P vs T=1	0.900	0.609	0.679	0.529	0.745
PS vs T=1	-0.970	-0.711	-0.540	-0.245	0.086
PS vs P	-1.714	-1.214	-1.117	-0.696	-0.583

immunoreactive 47 kDa antigen

cell envelope

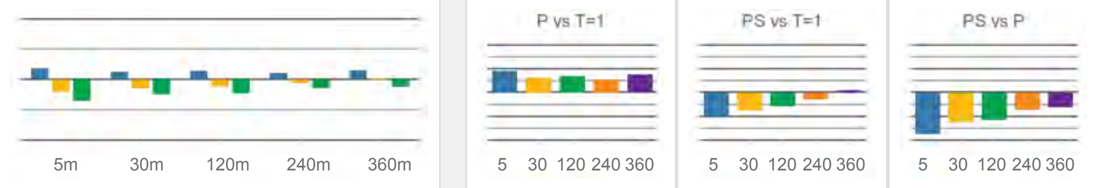

PGN_0853

Locus	5m	30m	120m	240m	360m
P vs T=1	1.191	0.350	0.274	0.874	2.589
PS vs T=1	-0.619	-0.575	0.294	1.272	1.681
PS vs P	-1.336	-0.806	-0.185	0.420	-0.312

conserved hypothetical protein

hypothetical proteins-Conserved

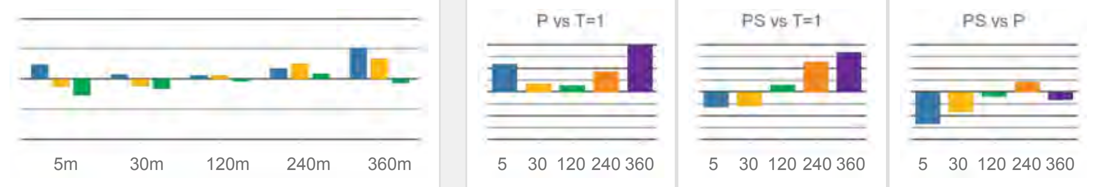

PGN_0854

Locus	5m	30m	120m	240m	360m
P vs T=1	-0.657	-0.633	-0.308	0.341	0.372
PS vs T=1	-1.350	-1.428	0.760	1.777	2.065
PS vs P	-0.754	-0.842	0.878	1.459	1.666

hypothetical protein

hypothetical proteins

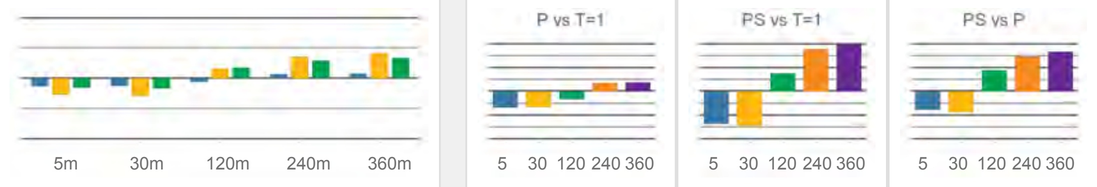

PgSg Time Course

log$_2$ (Fold Change)

Legend: P vs T=1 | PS vs T=1 | PS vs P

Locus		5m	30m	120m	240m	360m
**PGN_0855**	P vs T=1	0.462	0.290	0.499	0.146	1.846
	PS vs T=1	0.224	0.216	1.726	3.495	3.675
	PS vs P	-0.308	-0.281	0.831	2.284	1.986
	hypothetical protein					
	hypothetical proteins					
**PGN_0856**	P vs T=1	-0.289	-0.696	-0.351	0.344	1.496
	PS vs T=1	0.196	0.877	2.257	2.845	2.638
	PS vs P	0.363	1.291	2.301	2.336	1.196
	putative A/G-specific adenine glycosylase					
	DNA metabolism					
**PGN_0857**	P vs T=1	-0.281	-1.143	-2.293	-2.458	-1.918
	PS vs T=1	-0.773	-0.968	-1.012	-0.849	-1.017
	PS vs P	-0.375	0.171	1.067	1.322	0.827
	conserved hypothetical protein					
	transport and binding proteins					
**PGN_0858**	P vs T=1	-0.518	-0.860	-1.604	-1.869	-1.155
	PS vs T=1	-0.530	-0.797	-0.763	-0.597	-0.697
	PS vs P	0.020	0.061	0.683	0.961	0.427
	probable ABC transporter ATP-binding protein					
	transport and binding proteins					
**PGN_0859**	P vs T=1	-0.240	-0.483	-0.808	-0.627	0.044
	PS vs T=1	-0.121	-0.023	-0.145	-0.147	-0.635
	PS vs P	0.126	0.423	0.535	0.395	-0.593
	probable ABC transporter permease protein					
	transport and binding proteins					
**PGN_0860**	P vs T=1	-1.772	-1.902	-1.866	-1.899	-2.135
	PS vs T=1	-1.240	-1.186	-1.003	-1.287	-1.553
	PS vs P	0.491	0.657	0.826	0.574	0.531
	conserved hypothetical protein					
	hypothetical proteins-Conserved					
**PGN_0861**	P vs T=1	-0.264	-0.356	-0.384	-0.232	-0.377
	PS vs T=1	0.008	0.180	0.256	0.148	0.358
	PS vs P	0.257	0.505	0.601	0.367	0.703
	conserved hypothetical protein					
	hypothetical proteins-Conserved					

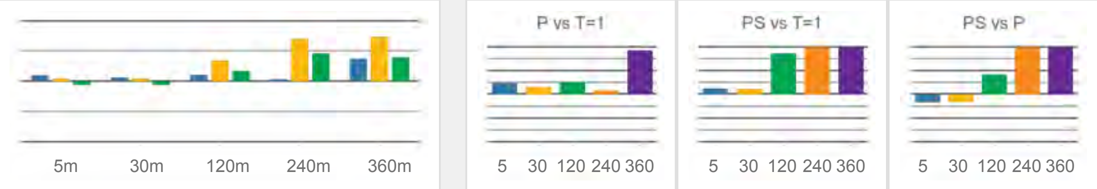

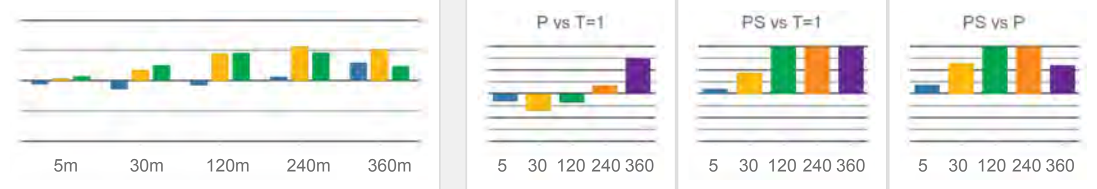

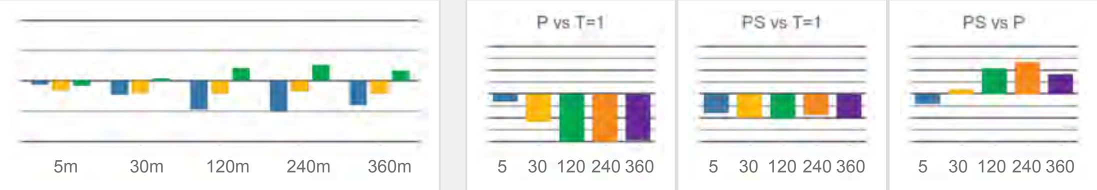

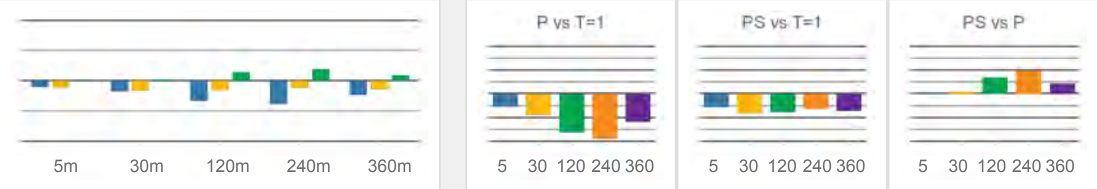

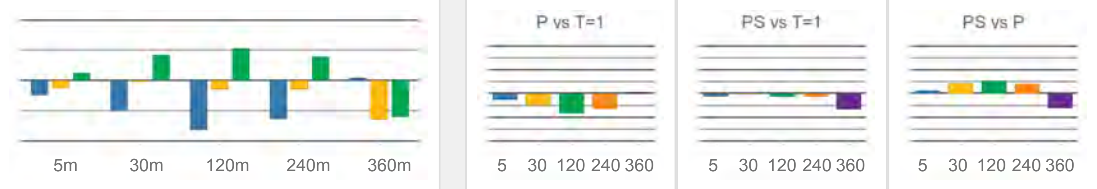

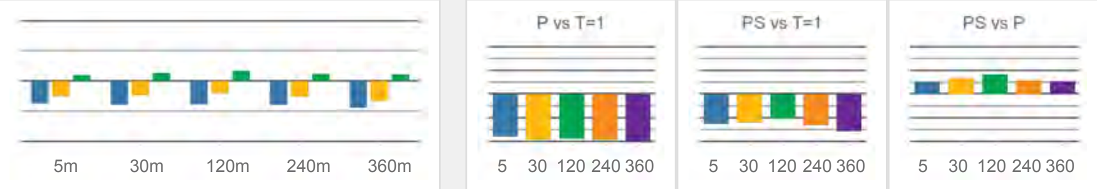

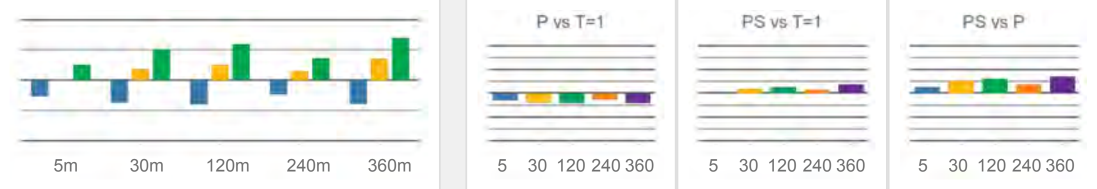

log₂ (Fold Change)

Legend: P vs T=1 · PS vs T=1 · PS vs P

Locus		5m	30m	120m	240m	360m
**PGN_0862**	P vs T=1	-0.262	-0.174	-0.648	-0.619	-0.143
	PS vs T=1	-0.255	-0.513	-0.988	-0.937	-0.665
	PS vs P	0.016	-0.315	-0.352	-0.325	-0.493

Type III restriction enzyme, res subunit

DNA metabolism

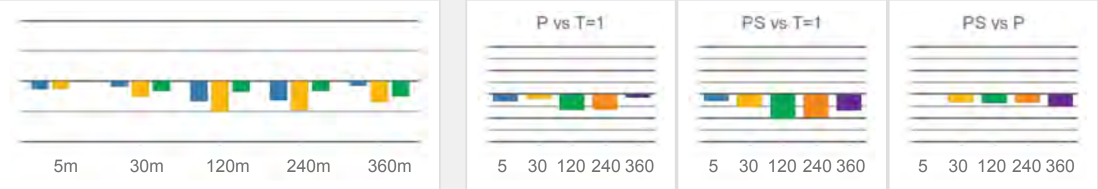

Locus		5m	30m	120m	240m	360m
**PGN_0863**	P vs T=1	-0.730	-0.945	-0.716	-0.182	0.190
	PS vs T=1	-1.249	-1.079	-0.403	0.043	0.006
	PS vs P	-0.516	-0.165	0.277	0.270	-0.138

DNA methylase N-4/N-6

DNA metabolism

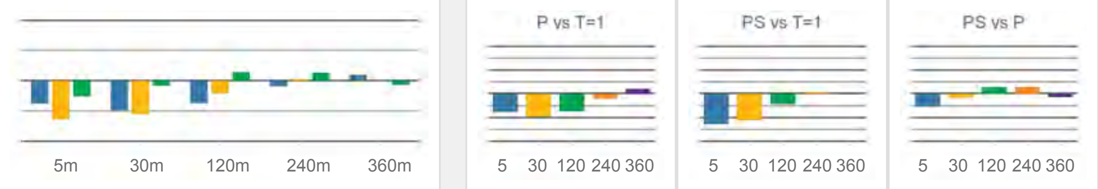

Locus		5m	30m	120m	240m	360m
**PGN_0864**	P vs T=1	0.316	-0.392	-0.144	0.558	1.620
	PS vs T=1	0.017	-0.098	0.308	0.587	0.757
	PS vs P	-0.261	0.132	0.297	0.063	-0.695

transposase in ISPg3

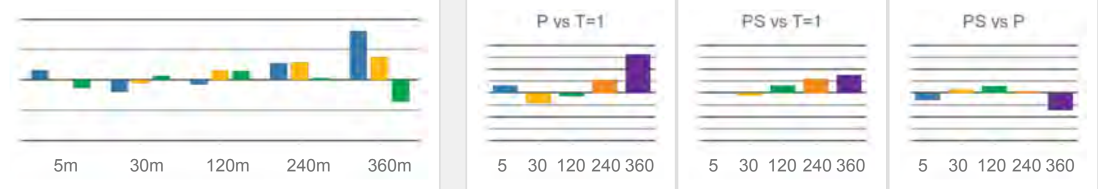

Locus		5m	30m	120m	240m	360m
**PGN_0865**	P vs T=1	-0.610	-0.484	-0.629	-1.090	-1.744
	PS vs T=1	-0.787	-0.832	-1.052	-1.065	-1.142
	PS vs P	-0.176	-0.342	-0.418	0.016	0.582

bifunctional phosphoribosylaminoimidazolecarboxamide formyltransferase/IMP cyclohydrolase

purines, pyrimidines, nucleosides and nucleotides

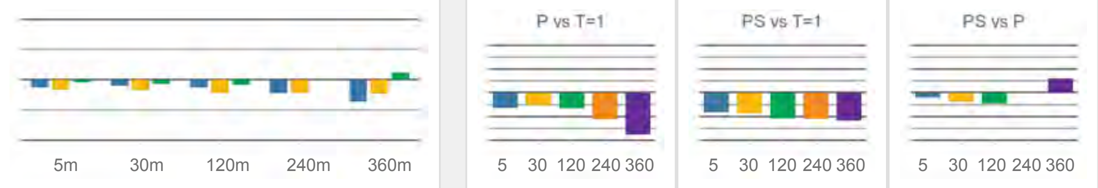

Locus		5m	30m	120m	240m	360m
**PGN_0866**	P vs T=1	0.444	0.762	0.855	0.513	-0.037
	PS vs T=1	0.900	1.053	0.957	0.570	0.328
	PS vs P	0.431	0.299	0.125	0.051	0.328

cell shape-determining protein MreB

cell envelope

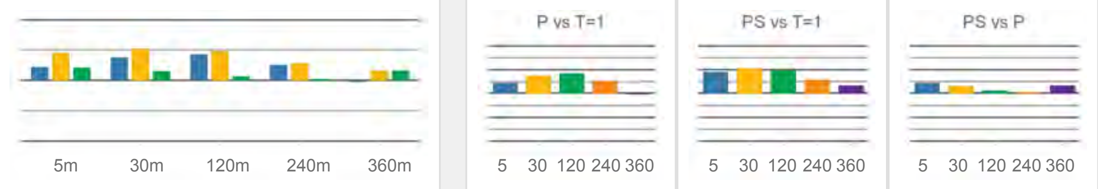

Locus		5m	30m	120m	240m	360m
**PGN_0867**	P vs T=1	-1.416	-1.233	-1.069	-0.719	-0.633
	PS vs T=1	-1.406	-1.406	-1.317	-1.337	-0.993
	PS vs P	-0.023	-0.185	-0.255	-0.559	-0.331

probable cell shape-determining protein MreC

cell envelope

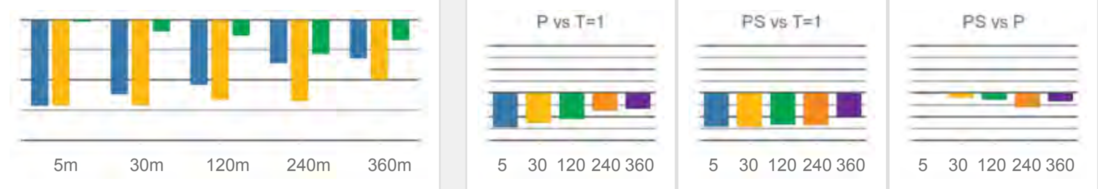

Locus		5m	30m	120m	240m	360m
**PGN_0868**	P vs T=1	-1.770	-2.275	-1.989	-1.574	-0.499
	PS vs T=1	-1.881	-2.416	-2.273	-1.561	-2.103
	PS vs P	-0.111	-0.321	-0.386	0.055	-1.243

conserved hypothetical protein

hypothetical proteins-Conserved

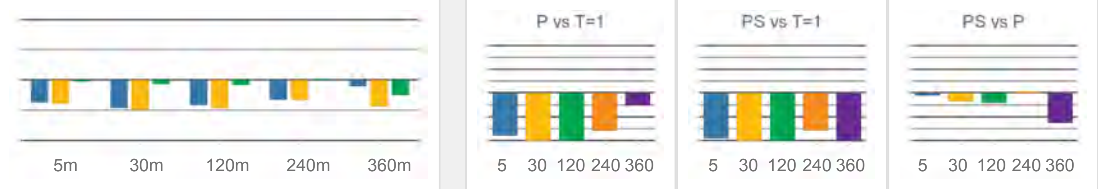

PgSg Time Course

log_2 (Fold Change)

Legend: P vs T=1 | PS vs T=1 | PS vs P

PGN_0869

Locus		5m	30m	120m	240m	360m
PGN_0869	P vs T=1	-1.422	-1.771	-1.305	-0.683	0.085
	PS vs T=1	-0.821	-1.535	-1.852	-1.230	-0.950
	PS vs P	0.568	0.138	-0.576	-0.458	-0.927

penicillin-binding protein 2

cell envelope

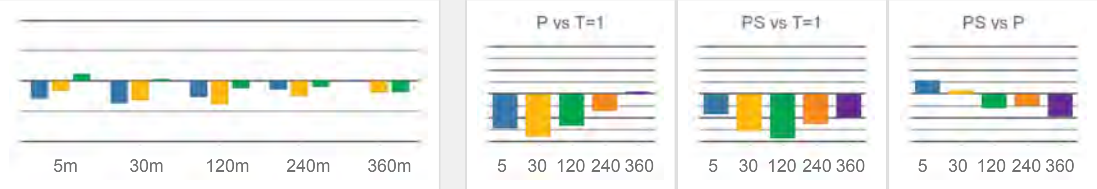

PGN_0870

Locus		5m	30m	120m	240m	360m
PGN_0870	P vs T=1	-1.649	-1.581	-1.238	-1.053	-0.623
	PS vs T=1	-1.035	-1.360	-1.621	-1.641	-1.213
	PS vs P	0.562	0.175	-0.385	-0.563	-0.547

putative rod shape-determining protein RodA

cellular processes

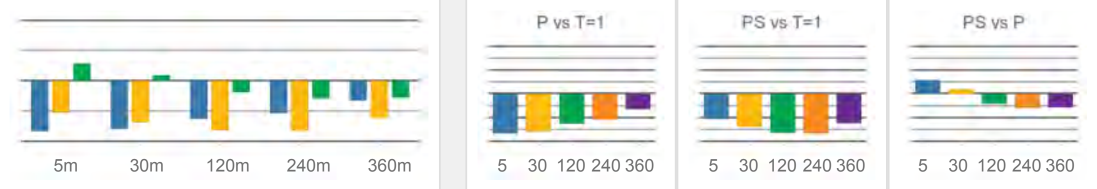

PGN_0871

Locus		5m	30m	120m	240m	360m
PGN_0871	P vs T=1	-0.497	-0.640	-0.412	-0.113	-0.233
	PS vs T=1	-0.512	-0.708	-0.459	-0.144	-0.362
	PS vs P	-0.025	-0.097	-0.060	-0.004	-0.129

conserved hypothetical protein

hypothetical proteins-Conserved

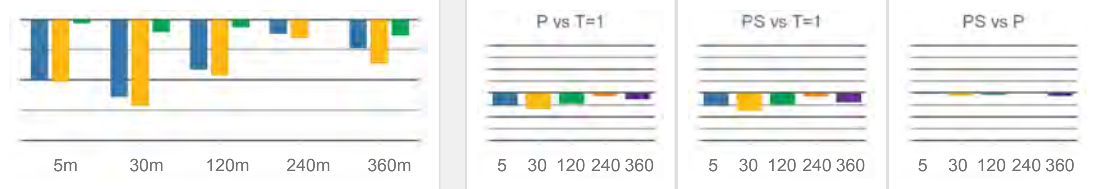

PGN_0872

Locus		5m	30m	120m	240m	360m
PGN_0872	P vs T=1	-0.169	-0.060	-0.351	-0.798	-1.123
	PS vs T=1	-0.105	-0.246	-0.686	-0.645	-0.963
	PS vs P	0.079	-0.143	-0.314	0.090	0.081

DNA-binding protein histone-like family

DNA metabolism

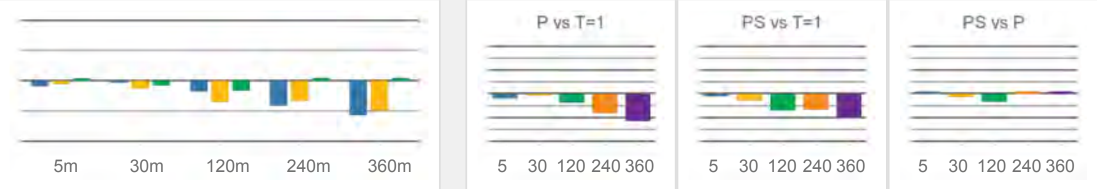

PGN_0873

Locus		5m	30m	120m	240m	360m
PGN_0873	P vs T=1	-0.420	-0.117	0.196	-0.016	-0.324
	PS vs T=1	-0.417	-0.496	-0.494	-0.165	0.088
	PS vs P	-0.015	-0.369	-0.651	-0.141	0.386

conserved hypothetical protein

hypothetical proteins-Conserved

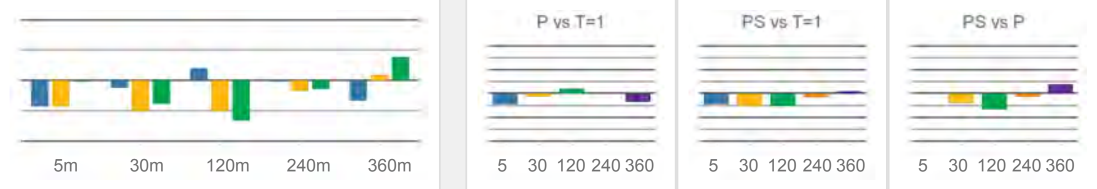

PGN_0874

Locus		5m	30m	120m	240m	360m
PGN_0874	P vs T=1	-0.326	0.110	0.222	0.159	0.116
	PS vs T=1	-0.005	0.182	-0.155	0.102	0.235
	PS vs P	0.298	0.075	-0.366	-0.056	0.116

conserved hypothetical protein

hypothetical proteins-Conserved

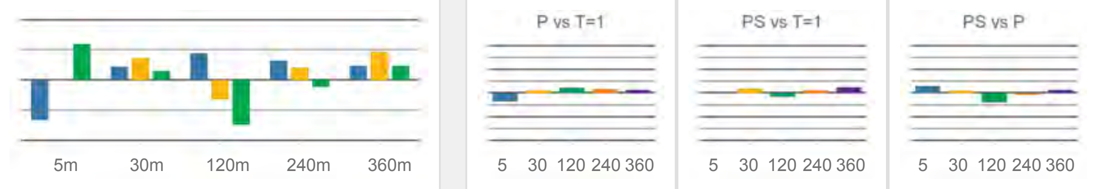

PGN_0875

gyrA

Locus		5m	30m	120m	240m	360m
PGN_0875	P vs T=1	1.097	1.253	0.438	-0.506	-1.018
	PS vs T=1	1.489	1.427	0.349	-0.642	-0.783
	PS vs P	0.404	0.197	-0.082	-0.163	0.207

DNA gyrase A subunit

DNA metabolism

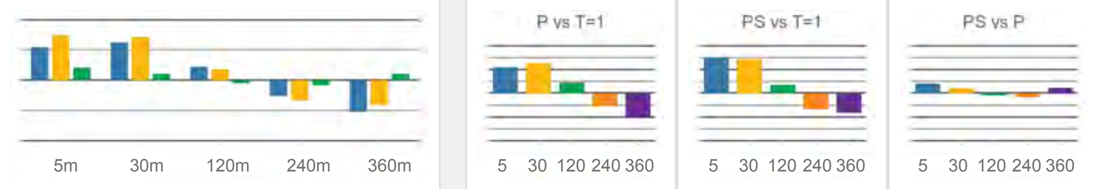

PgSg Time Course

log$_2$ (Fold Change)

Legend: P vs T=1 | PS vs T=1 | PS vs P

Locus		5m	30m	120m	240m	360m
**PGN_0876**	P vs T=1	-0.572	-0.085	0.152	-0.286	-1.227
	PS vs T=1	-0.155	0.328	0.033	-0.789	-0.858
	PS vs P	0.382	0.424	-0.079	-0.491	0.327

TPR domain protein

unknown function

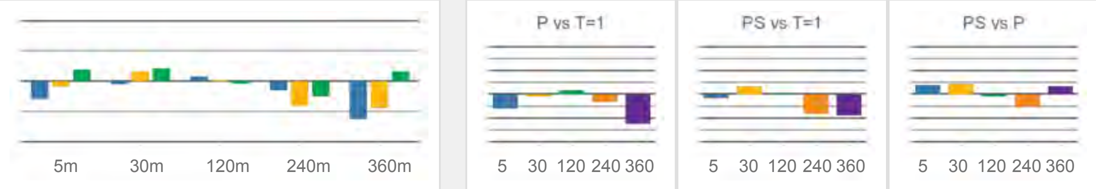

Locus		5m	30m	120m	240m	360m
**PGN_0877**	P vs T=1	0.018	0.061	0.404	0.776	1.211
	PS vs T=1	-0.247	-0.479	-0.369	0.020	0.351
	PS vs P	-0.262	-0.532	-0.748	-0.698	-0.811

SNF2-related helicase

DNA metabolism

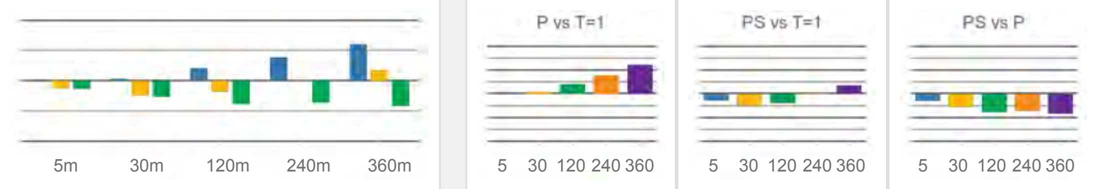

Locus		5m	30m	120m	240m	360m
**PGN_0878**	P vs T=1	-0.519	-0.309	-0.355	-0.255	-0.383
	PS vs T=1	-0.411	-0.700	-0.951	-0.795	-0.734
	PS vs P	0.101	-0.355	-0.556	-0.449	-0.323

hypothetical protein

hypothetical proteins

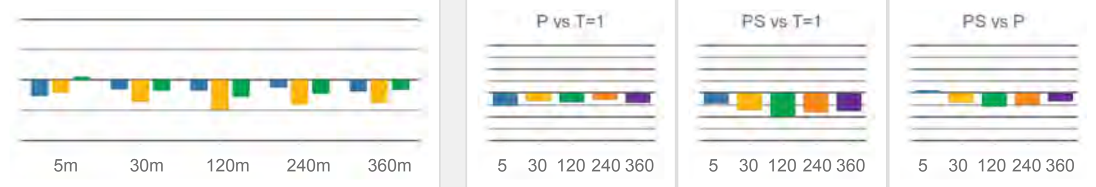

Locus		5m	30m	120m	240m	360m
**PGN_0879**	P vs T=1	0.258	-0.403	-0.103	0.520	1.402
	PS vs T=1	-0.223	-0.076	0.312	0.363	0.476
	PS vs P	-0.448	0.210	0.315	-0.110	-0.800

transposase in ISPg3

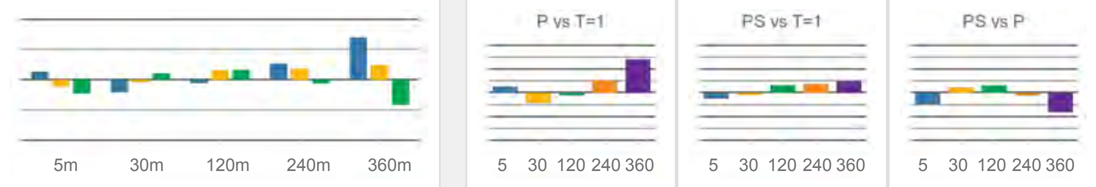

Locus		5m	30m	120m	240m	360m
**PGN_0880**	P vs T=1	0.922	0.821	0.179	-0.528	-1.180
	PS vs T=1	0.669	0.538	0.076	-0.447	-0.883
	PS vs P	-0.214	-0.245	-0.090	0.058	0.259

tyrosine phenol-lyase

energy metabolism

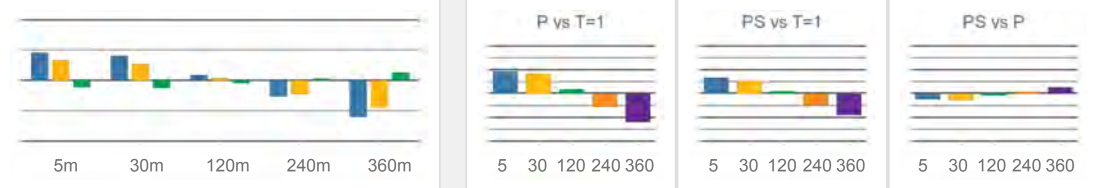

Locus		5m	30m	120m	240m	360m
**PGN_0881**	P vs T=1	-1.242	-2.002	-2.396	-1.893	-1.135
	PS vs T=1	-0.382	-1.290	-2.111	-2.234	-1.899
	PS vs P	0.878	0.613	0.075	-0.374	-0.679

conserved hypothetical protein

unknown function

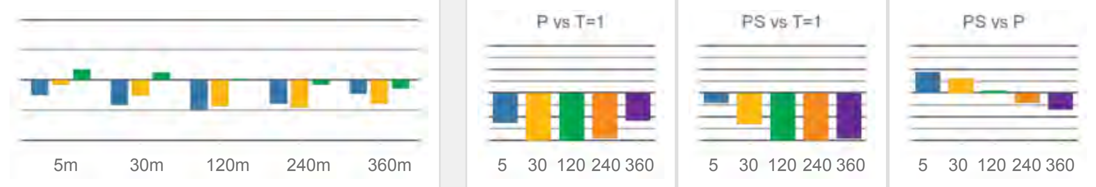

Locus		5m	30m	120m	240m	360m
**PGN_0882**	P vs T=1	-0.469	-0.888	-1.149	-1.426	-0.713
	PS vs T=1	0.366	-0.396	-1.288	-1.771	-1.396
	PS vs P	0.851	0.470	-0.161	-0.438	-0.633

conserved hypothetical protein

hypothetical proteins-Conserved

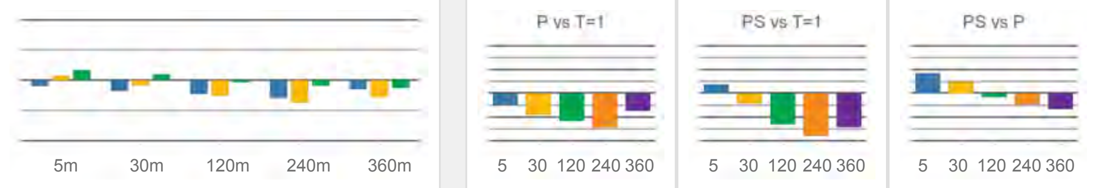

PgSg Time Course

log2 (Fold Change)

P vs T=1 | PS vs T=1 | PS vs P

Locus		5m	30m	120m	240m	360m
PGN_0883	P vs T=1	-0.461	-0.860	-1.529	-1.693	-0.833
	PS vs T=1	-0.085	-0.707	-1.282	-1.363	-1.393
	PS vs P	0.395	0.151	0.149	0.153	-0.527

conserved hypothetical protein

hypothetical proteins-Conserved

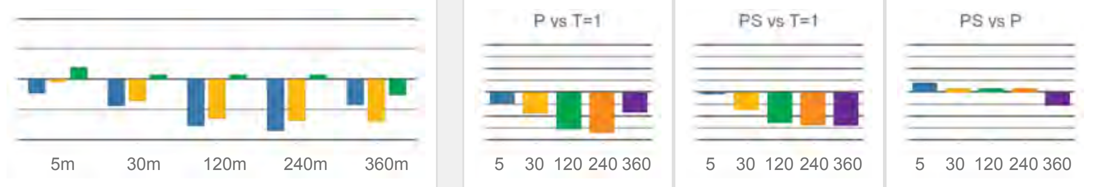

Locus		5m	30m	120m	240m	360m
PGN_0884	P vs T=1	-0.275	-1.006	-1.152	-0.692	-0.506
	PS vs T=1	-0.761	-1.211	-1.219	-1.111	-0.932
	PS vs P	-0.438	-0.228	-0.107	-0.401	-0.410

organic solvent tolerance protein OstA

hypothetical proteins

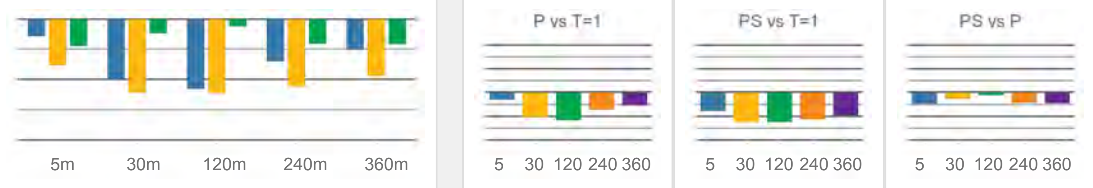

Locus		5m	30m	120m	240m	360m
PGN_0885	P vs T=1	0.207	-1.019	-1.363	-0.819	-0.145
	PS vs T=1	-0.895	-0.446	0.331	0.544	0.552
	PS vs P	-0.966	0.407	1.299	1.169	0.726

probable nitroimidazole resistance protein

cellular processes

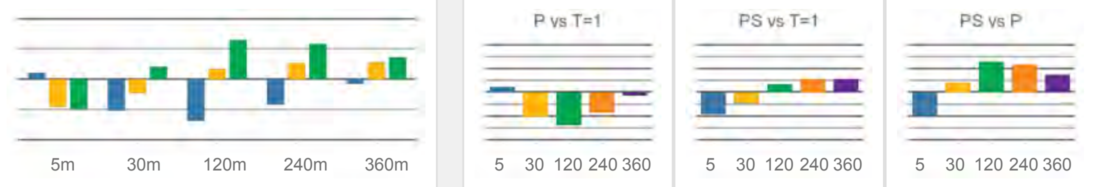

Locus		5m	30m	120m	240m	360m
PGN_0886	P vs T=1	0.783	0.751	0.495	0.268	0.086
	PS vs T=1	0.603	0.875	0.857	0.733	0.445
	PS vs P	-0.167	0.130	0.342	0.412	0.311

putative cation efflux protein

transport and binding proteins

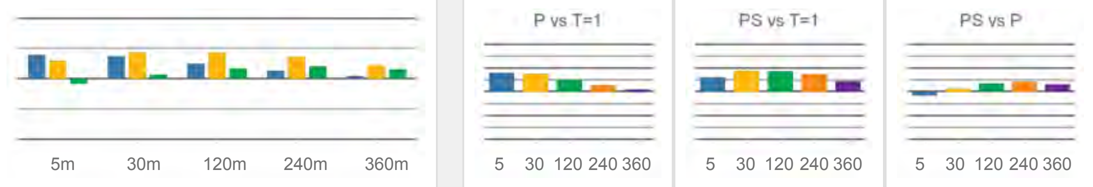

Locus		5m	30m	120m	240m	360m
PGN_0887	P vs T=1	-1.598	-1.632	-1.348	-1.165	-0.994
	PS vs T=1	-1.574	-1.543	-1.749	-1.504	-1.336
	PS vs P	-0.018	0.030	-0.389	-0.273	-0.288

conserved hypothetical protein

hypothetical proteins-Conserved

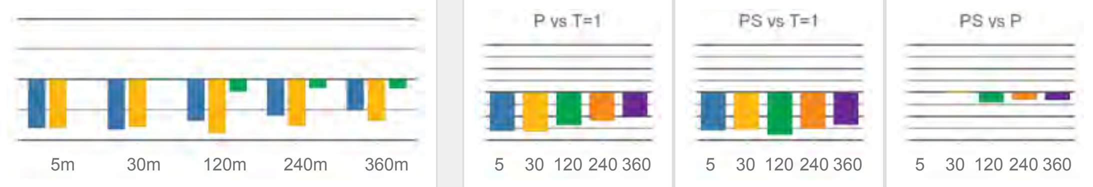

Locus		5m	30m	120m	240m	360m
PGN_0888	P vs T=1	-1.759	-1.668	-1.752	-1.974	-1.904
	PS vs T=1	-2.124	-1.937	-2.278	-2.296	-1.798
	PS vs P	-0.370	-0.226	-0.467	-0.414	0.022

conserved hypothetical protein

hypothetical proteins-Conserved

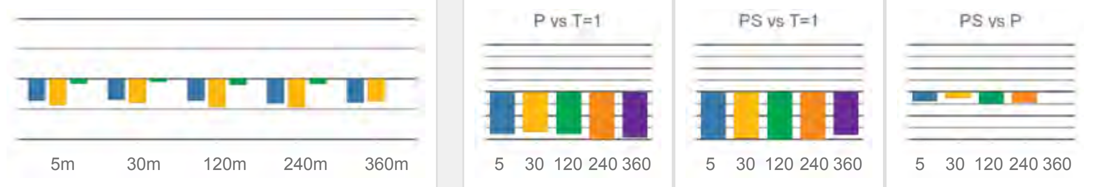

Locus		5m	30m	120m	240m	360m
PGN_0889	P vs T=1	-0.424	-0.859	-0.898	-0.756	0.005
	PS vs T=1	-0.963	-1.160	-0.980	-0.789	-0.227
	PS vs P	-0.514	-0.310	-0.106	-0.051	-0.188

potassium uptake protein TrkA

transport and binding proteins

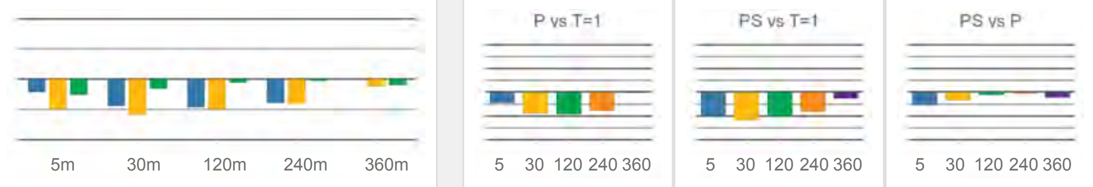

PgSg Time Course

log$_2$ (Fold Change)

Legend: P vs T=1 · PS vs T=1 · PS vs P

PGN_0890

Locus	5m	30m	120m	240m	360m
P vs T=1	-0.083	0.324	0.417	0.206	-0.142
PS vs T=1	-0.212	0.106	0.595	0.652	0.299
PS vs P	-0.149	-0.208	0.187	0.436	0.419

putative TonB-dependent outer membrane receptor protein

transport and binding proteins

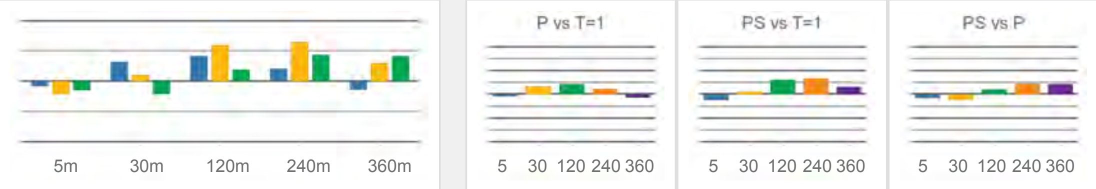

PGN_0891

Locus	5m	30m	120m	240m	360m
P vs T=1	-0.331	-0.001	0.220	-0.101	-0.602
PS vs T=1	-0.402	-0.435	-0.494	-0.602	-0.784
PS vs P	-0.075	-0.414	-0.677	-0.482	-0.194

dioxygenase

fatty acid and phospholipid metabolism

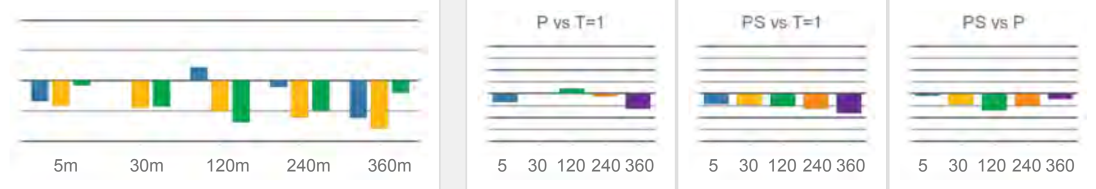

PGN_0892

Locus	5m	30m	120m	240m	360m
P vs T=1	-0.048	0.156	0.796	0.287	-0.024
PS vs T=1	0.137	0.260	0.371	0.070	-0.302
PS vs P	0.144	0.096	-0.246	-0.176	-0.296

hypothetical protein

hypothetical proteins

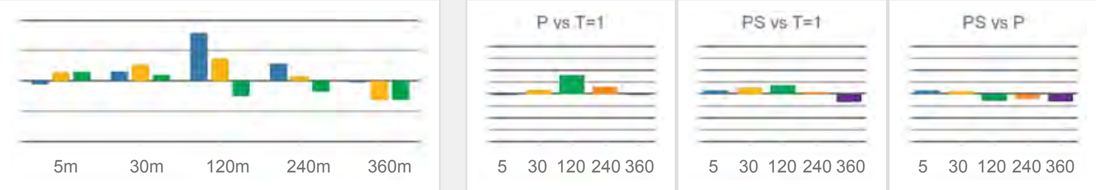

PGN_0893

Locus	5m	30m	120m	240m	360m
P vs T=1	-0.493	0.030	0.771	0.714	0.202
PS vs T=1	0.316	0.381	0.338	0.080	0.201
PS vs P	0.757	0.336	-0.396	-0.601	-0.005

fumarate hydratase class I anaerobic

energy metabolism

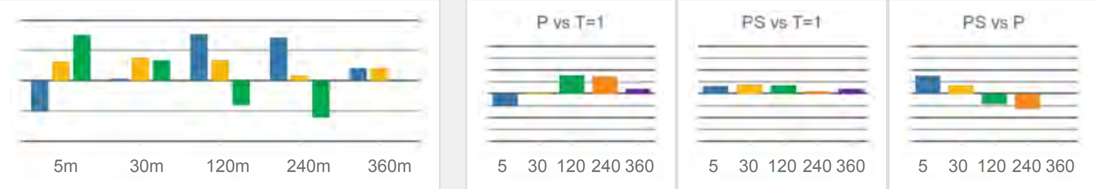

PGN_0894

Locus	5m	30m	120m	240m	360m
P vs T=1	0.184	0.129	-0.168	-0.250	-0.221
PS vs T=1	0.519	0.044	-0.076	0.308	0.083
PS vs P	0.337	-0.081	0.081	0.537	0.295

DNA polymerase III, gamma and tau subunits

DNA metabolism

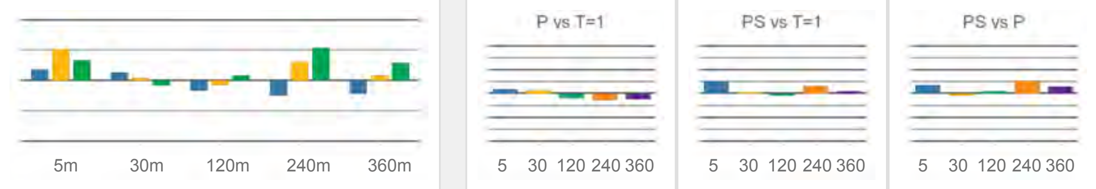

PGN_0895

Locus	5m	30m	120m	240m	360m
P vs T=1	-0.480	-0.150	0.448	0.734	0.057
PS vs T=1	-0.084	0.194	-0.373	-0.766	-1.003
PS vs P	0.306	0.313	-0.735	-1.354	-1.040

ferredoxin 4Fe-4S

energy metabolism

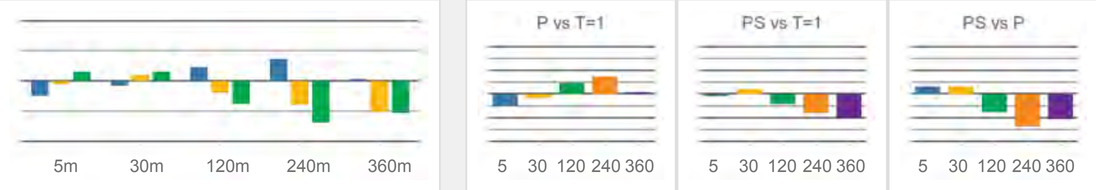

PGN_0896

Locus	5m	30m	120m	240m	360m
P vs T=1	0.020	-0.868	-1.221	-0.883	-0.429
PS vs T=1	-0.454	-0.381	-0.129	-0.061	-0.130
PS vs P	-0.427	0.438	0.972	0.759	0.306

probable D-alanyl-D-alanine carboxypeptidase

protein fate

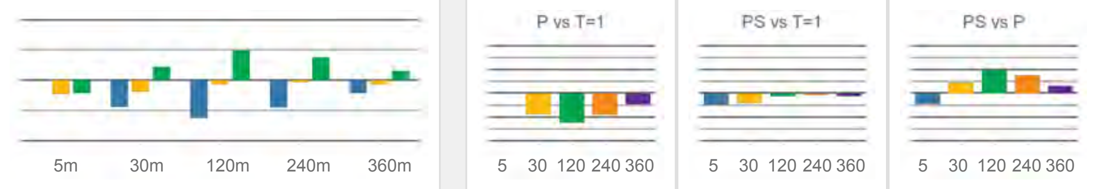

PgSg RNA Seq

PgSg Time Course

Hackett Laboratory UW

log$_2$ (Fold Change)

Legend: P vs T=1 | PS vs T=1 | PS vs P

Locus		5m	30m	120m	240m	360m
**PGN_0897**	P vs T=1	1.292	0.575	0.788	1.412	1.173
	PS vs T=1	0.845	1.002	1.298	1.135	1.467
	PS vs P	-0.300	0.279	0.408	-0.019	0.354

conserved hypothetical protein

hypothetical proteins-Conserved

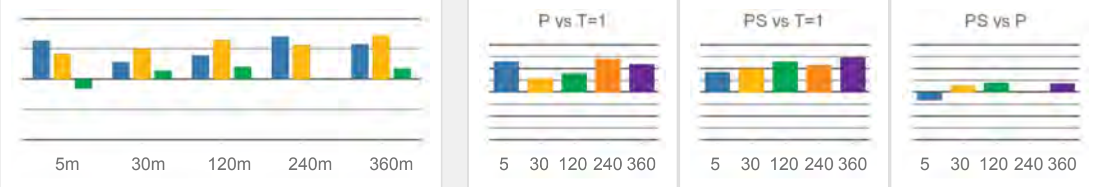

Locus		5m	30m	120m	240m	360m
**PGN_0898**	P vs T=1	0.100	0.239	0.514	0.224	-0.441
	PS vs T=1	0.139	-0.109	-0.235	-0.538	-0.695
	PS vs P	0.044	-0.333	-0.720	-0.742	-0.260

probable peptidylarginine deiminase

cellular processes

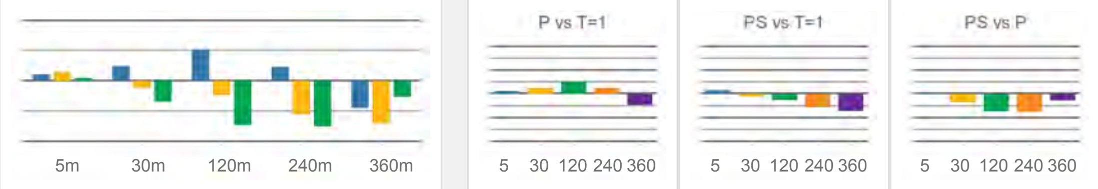

Locus		5m	30m	120m	240m	360m
**PGN_0899**	P vs T=1	0.290	0.293	0.628	2.454	2.294
	PS vs T=1	0.005	-0.529	2.102	3.067	2.876
	PS vs P	-0.390	-0.688	0.879	1.324	1.015

conserved hypothetical protein

hypothetical proteins-Conserved

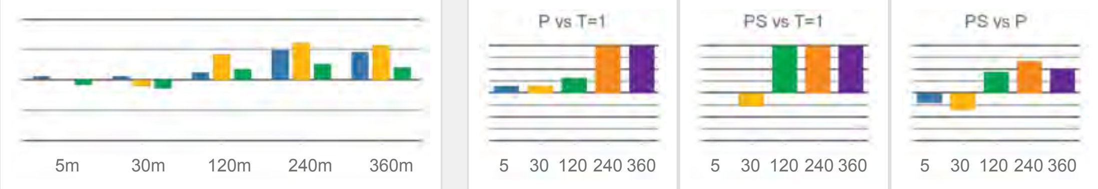

Locus		5m	30m	120m	240m	360m
**PGN_0900**	P vs T=1	0.359	-0.139	-0.185	0.091	0.068
	PS vs T=1	-0.109	-0.314	-0.904	-1.228	-0.881
	PS vs P	-0.421	-0.164	-0.702	-1.266	-0.918

thiol protease

protein fate

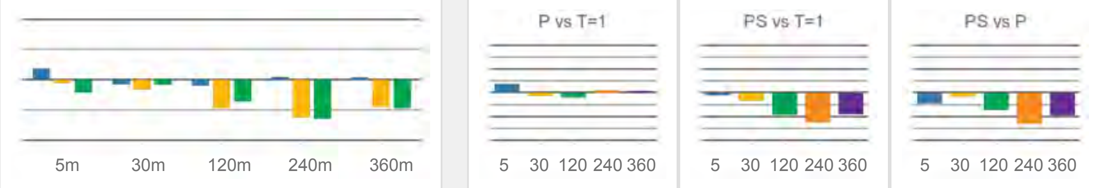

Locus		5m	30m	120m	240m	360m
**PGN_0901** *RibH*	P vs T=1	-0.071	-1.016	-1.558	-1.883	-1.737
	PS vs T=1	-0.853	-1.438	-2.006	-2.629	-2.561
	PS vs P	-0.690	-0.392	-0.433	-0.770	-0.807

putative riboflavin synthase beta subunit

biosynthesis of cofactors, prosthetic groups, and carriers

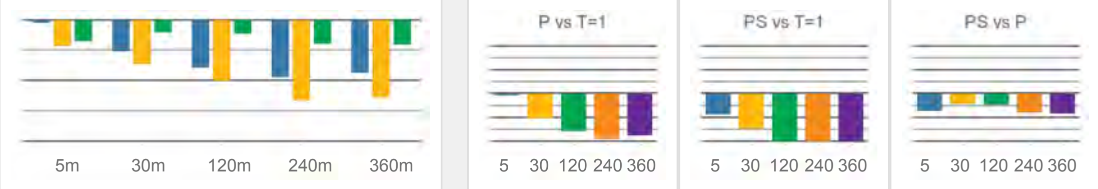

Locus		5m	30m	120m	240m	360m
**PGN_0902**	P vs T=1	-0.821	-1.519	-1.588	-1.578	-2.296
	PS vs T=1	-1.070	-0.674	-0.376	-0.308	-0.431
	PS vs P	-0.207	0.756	1.141	1.207	1.740

conserved hypothetical protein

unknown function

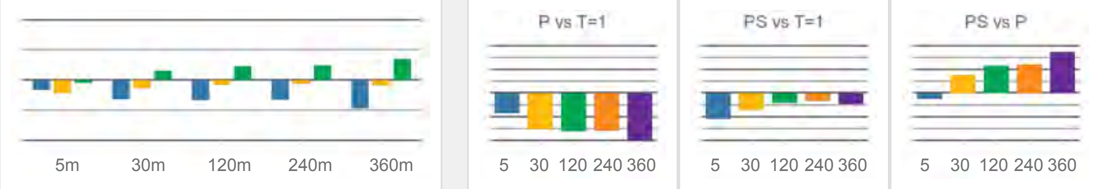

Locus		5m	30m	120m	240m	360m
**PGN_0903** *fimR*	P vs T=1	-0.714	-1.585	-2.295	-2.293	-2.272
	PS vs T=1	-0.596	-0.880	-1.155	-1.506	-1.662
	PS vs P	0.180	0.651	1.000	0.654	0.525

two-component system response regulator FimR

signal transduction

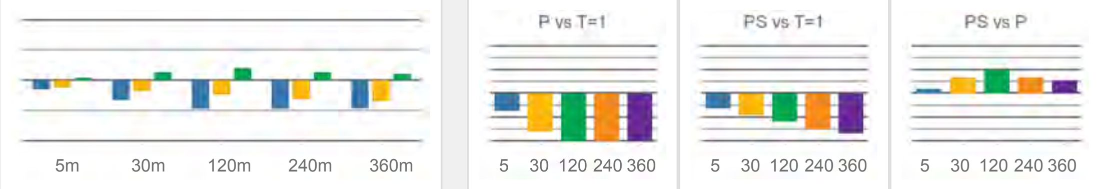

PgSg Time Course

log₂ (Fold Change)

Legend: P vs T=1 | PS vs T=1 | PS vs P

Locus		5m	30m	120m	240m	360m
**PGN_0904**	P vs T=1	-0.697	-1.911	-2.731	-2.282	-1.688
fimS	PS vs T=1	-0.300	-0.940	-1.025	-1.544	-1.645
	PS vs P	0.518	0.772	1.318	0.548	0.021

two-component system sensor histidine kinase FimS

signal transduction

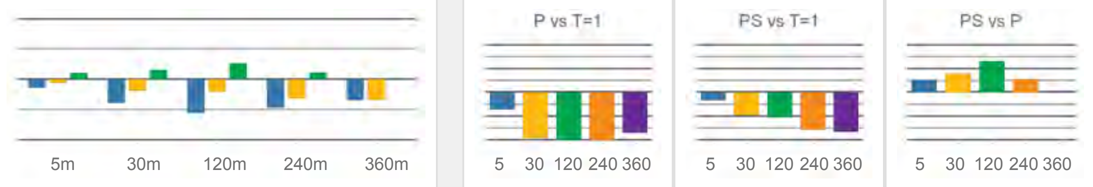

Locus		5m	30m	120m	240m	360m
**PGN_0905**	P vs T=1	-0.543	-1.223	-1.584	-1.648	-1.436
	PS vs T=1	-0.663	-0.976	-1.241	-1.565	-1.682
	PS vs P	-0.076	0.220	0.283	0.010	-0.264

putative dihydroorotate dehydrogenase

purines, pyrimidines, nucleosides and nucleotides

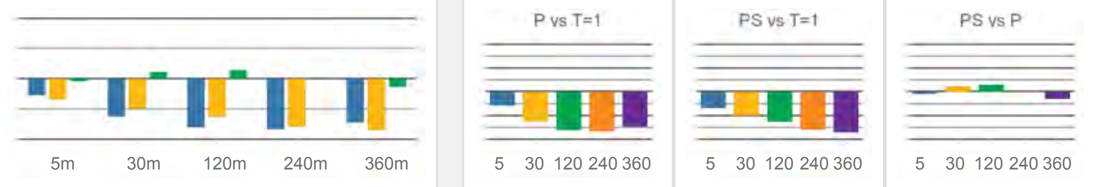

Locus		5m	30m	120m	240m	360m
**PGN_0906**	P vs T=1	-0.863	-1.502	-1.851	-2.002	-1.986
	PS vs T=1	-1.321	-1.387	-1.560	-1.696	-1.884
	PS vs P	-0.397	0.091	0.241	0.232	0.056

probable dihydroorotate dehydrogenase electron transfer subunit

purines, pyrimidines, nucleosides and nucleotides

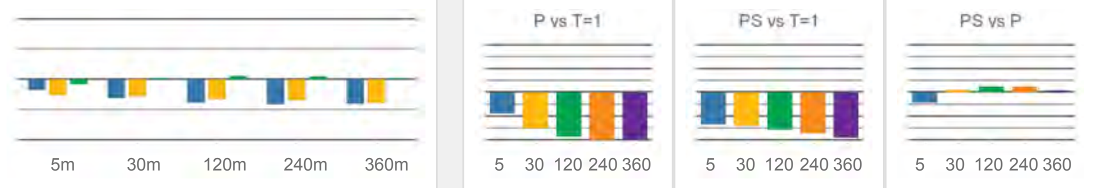

Locus		5m	30m	120m	240m	360m
**PGN_0907**	P vs T=1	0.077	-0.309	-0.301	-0.078	-0.288
	PS vs T=1	-0.457	-0.062	0.451	0.701	0.688
	PS vs P	-0.510	0.199	0.686	0.747	0.919

conserved hypothetical protein

regulatory functions

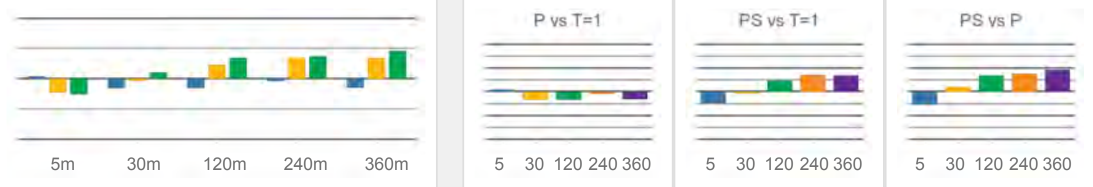

Locus		5m	30m	120m	240m	360m
**PGN_0908**	P vs T=1	0.508	-0.561	0.712	1.017	3.236
	PS vs T=1	-0.160	0.293	0.689	1.889	2.243
	PS vs P	-0.466	-0.034	-0.030	0.666	0.126

hypothetical protein

hypothetical proteins

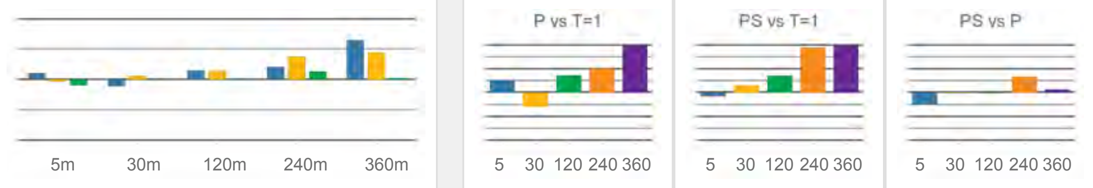

Locus		5m	30m	120m	240m	360m
**PGN_0909**	P vs T=1	0.292	-0.326	-0.363	0.403	1.523
	PS vs T=1	-0.191	-0.033	0.487	0.415	0.684
	PS vs P	-0.434	0.153	0.619	0.026	-0.660

transposase in ISPg3

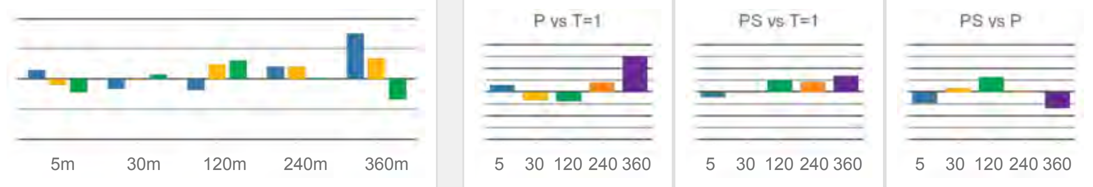

Locus		5m	30m	120m	240m	360m
**PGN_0910**	P vs T=1	0.039	0.232	0.182	1.085	1.599
	PS vs T=1	0.693	1.118	1.201	1.230	1.681
	PS vs P	0.564	0.807	0.830	0.210	0.158

conserved hypothetical protein

hypothetical proteins-Conserved

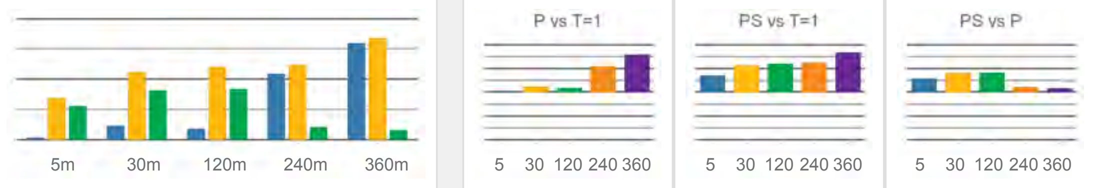

PgSg Time Course

log$_2$ (Fold Change)

Legend: P vs T=1 | PS vs T=1 | PS vs P

Locus		5m	30m	120m	240m	360m
**PGN_0911**	P vs T=1	0.403	0.497	0.022	-0.700	-0.757
	PS vs T=1	1.105	0.902	-0.340	-0.822	-1.094
	PS vs P	0.705	0.419	-0.350	-0.239	-0.391

putative ribonuclease H

transcription

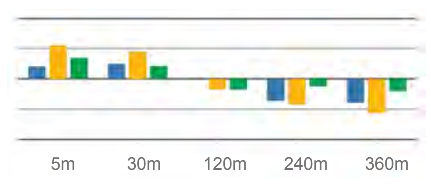

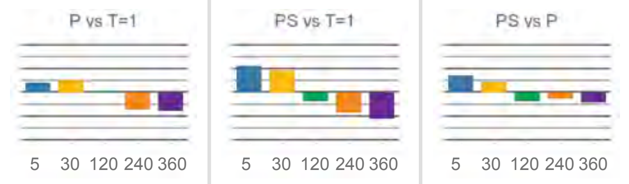

Locus		5m	30m	120m	240m	360m
**PGN_0912**	P vs T=1	1.322	1.520	0.617	-0.241	-0.556
	PS vs T=1	1.914	1.677	0.108	-0.702	-1.397
	PS vs P	0.605	0.184	-0.496	-0.507	-0.869

conserved hypothetical protein

unknown function

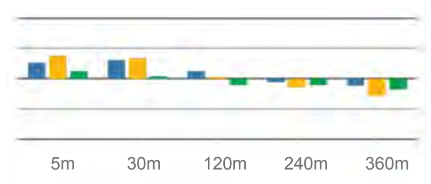

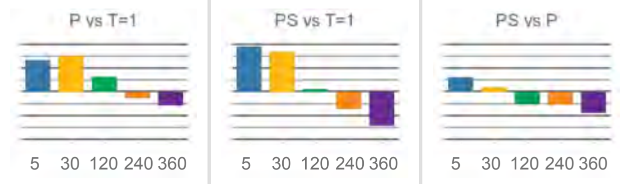

Locus		5m	30m	120m	240m	360m
**PGN_0913**	P vs T=1	1.971	2.065	1.383	0.633	-0.019
	PS vs T=1	2.511	2.089	0.862	0.201	-0.244
	PS vs P	0.562	0.059	-0.501	-0.466	-0.287

putative acetyltransferase

unknown function

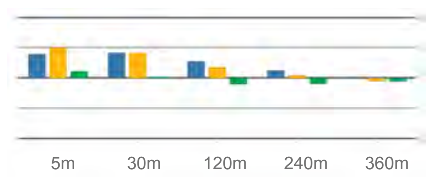

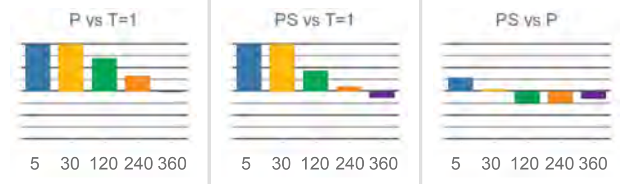

Locus		5m	30m	120m	240m	360m
**PGN_0914**	P vs T=1	1.640	2.080	2.008	1.261	0.383
	PS vs T=1	2.522	2.558	1.952	1.217	0.767
	PS vs P	0.862	0.503	-0.018	-0.063	0.327

peptidase M24 family

protein fate

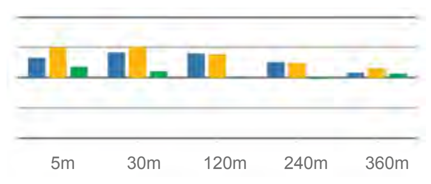

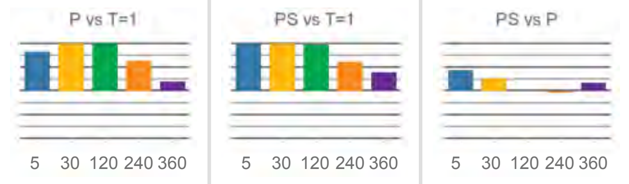

Locus		5m	30m	120m	240m	360m
**PGN_0915**	P vs T=1	-0.322	-1.263	-2.255	-2.346	-2.323
	PS vs T=1	-0.170	-0.980	-1.131	-0.789	-1.336
	PS vs P	0.225	0.238	0.959	1.383	0.876

conserved hypothetical protein

hypothetical proteins-Conserved

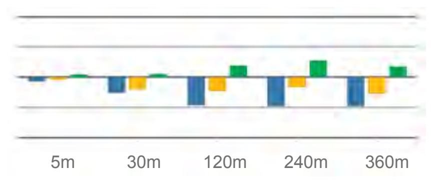

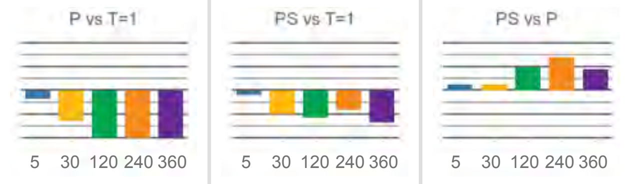

Locus		5m	30m	120m	240m	360m
**PGN_0916** *dnaK*	P vs T=1	2.322	2.936	3.367	3.260	2.711
	PS vs T=1	0.205	0.062	0.148	0.263	0.301
	PS vs P	-1.993	-2.673	-3.032	-2.827	-2.322

molecular chaperone DnaK

protein fate

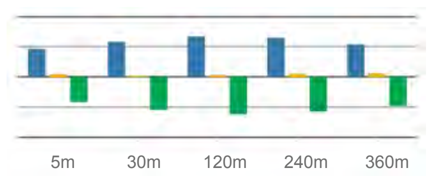

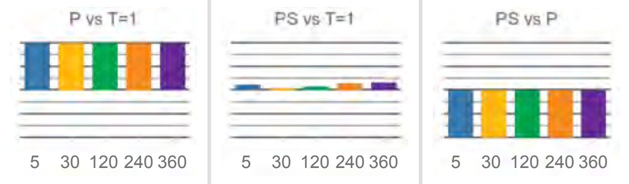

Locus		5m	30m	120m	240m	360m
**PGN_0917**	P vs T=1	-0.175	-0.846	-0.906	-0.811	-0.505
	PS vs T=1	-0.676	-1.285	-1.081	-1.011	-0.782
	PS vs P	-0.417	-0.465	-0.214	-0.223	-0.259

tyrosine type site-specific recombinase

DNA metabolism

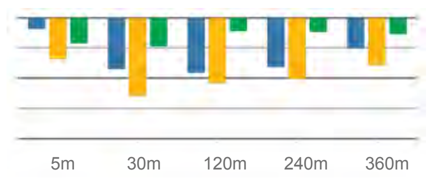

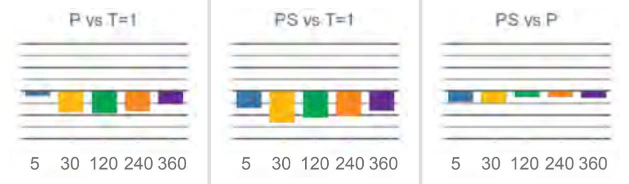

log$_2$ (Fold Change)

Locus		5m	30m	120m	240m	360m
PGN_0918	P vs T=1	0.397	0.341	0.390	0.417	0.038
	PS vs T=1	0.209	0.048	0.172	0.292	0.344
	PS vs P	-0.186	-0.296	-0.222	-0.127	0.281
	transposase in ISPg1					
PGN_0919	P vs T=1	-0.554	-1.403	-1.874	-1.470	-1.249
	PS vs T=1	-0.220	-0.057	0.470	0.500	0.352
	PS vs P	0.284	0.668	1.382	1.311	1.151
	hypothetical protein; *hypothetical proteins*					
PGN_0920	P vs T=1	0.378	0.121	1.105	1.640	3.023
	PS vs T=1	-0.576	0.512	1.598	1.993	3.134
	PS vs P	-0.789	-0.070	0.439	0.488	0.906
	putative partial excisionase; *hypothetical proteins*					
PGN_0921	P vs T=1	0.351	0.761	1.230	2.122	3.596
	PS vs T=1	-0.617	-0.702	1.181	1.159	2.197
	PS vs P	-0.768	-0.793	0.097	-0.120	-0.095
	hypothetical protein; *hypothetical proteins*					
PGN_0922	P vs T=1	0.254	0.004	0.791	2.020	3.425
	PS vs T=1	-0.834	-0.428	1.207	1.431	2.141
	PS vs P	-0.979	-0.606	0.316	-0.056	-0.494
	conserved hypothetical protein; *hypothetical proteins-Conserved*					
PGN_0923	P vs T=1	0.696	0.074	1.025	1.419	3.567
	PS vs T=1	-0.668	-0.061	1.063	1.284	1.780
	PS vs P	-1.123	-0.360	0.126	0.022	-1.114
	putative DNA primase; *DNA metabolism*					
PGN_0924	P vs T=1	0.245	-0.433	0.873	1.223	2.895
	PS vs T=1	-0.612	0.066	0.969	1.480	1.051
	PS vs P	-0.752	-0.077	0.220	0.405	-0.916
	mobilization protein; *cellular processes*					

Legend: P vs T=1, PS vs T=1, PS vs P

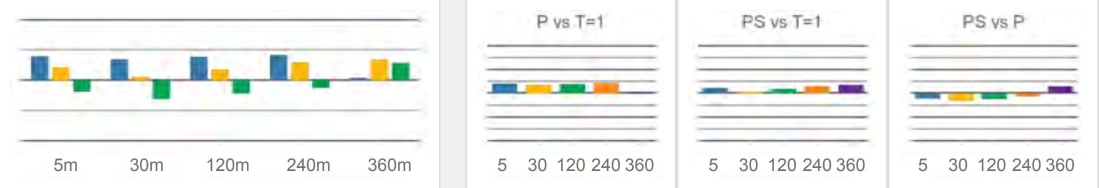

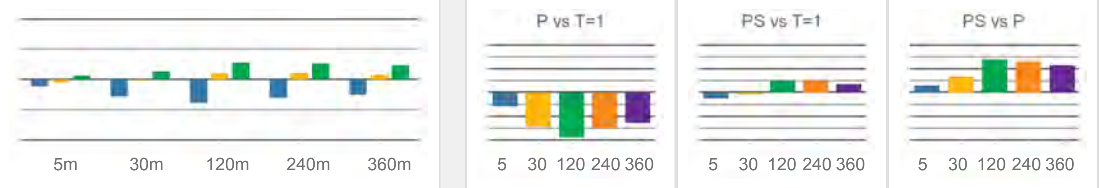

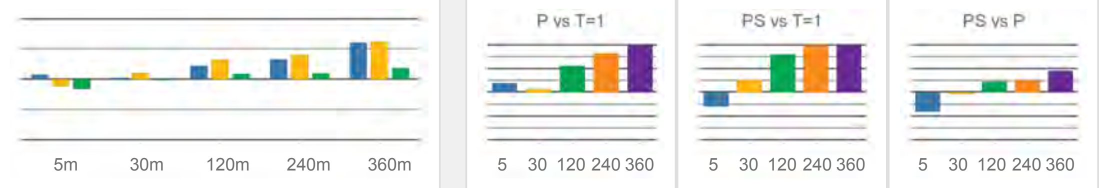

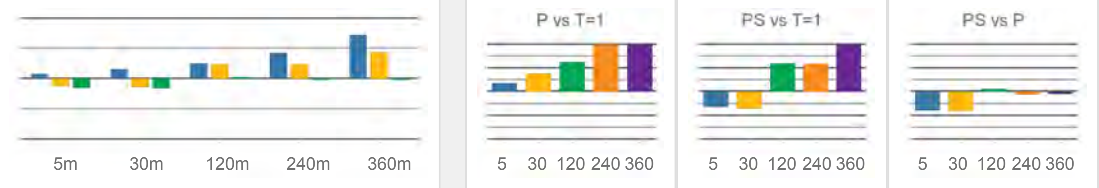

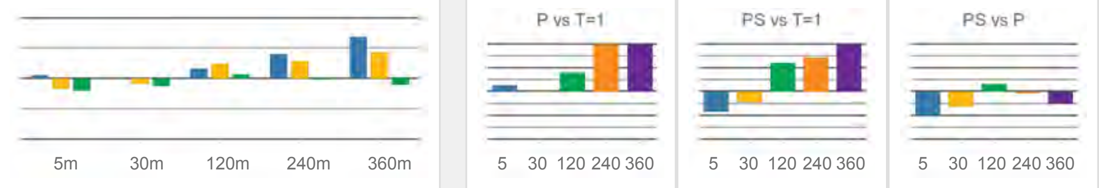

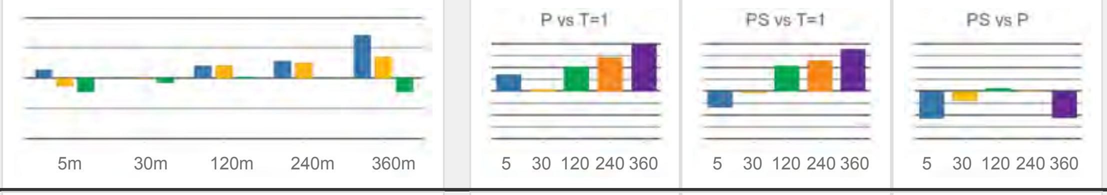

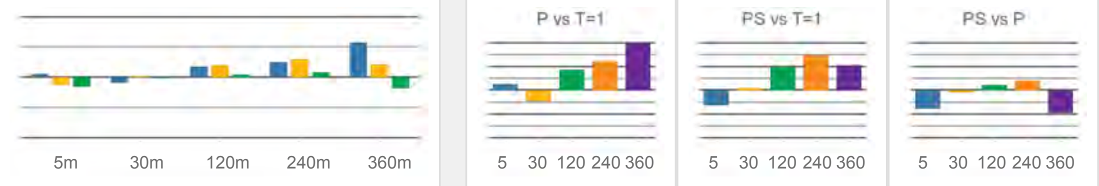

***PgSg* RNA Seq**

PgSg Time Course

Hackett Laboratory UW

log$_2$ (Fold Change)

P vs T=1 | PS vs T=1 | PS vs P

Locus		5m	30m	120m	240m	360m
**PGN_0925**	P vs T=1	0.702	0.144	0.974	1.950	3.264
	PS vs T=1	-0.185	0.170	1.349	1.249	1.914
	PS vs P	-0.750	-0.249	0.334	-0.295	-0.730

putative mobilization protein

other categories

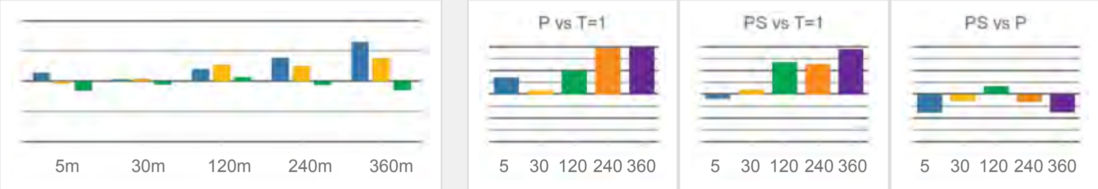

Locus		5m	30m	120m	240m	360m
**PGN_0926**	P vs T=1	0.252	-0.140	0.542	1.355	2.683
	PS vs T=1	-0.360	0.533	1.465	1.547	1.690
	PS vs P	-0.622	0.407	0.798	0.343	-0.676

conserved hypothetical protein

hypothetical proteins-Conserved

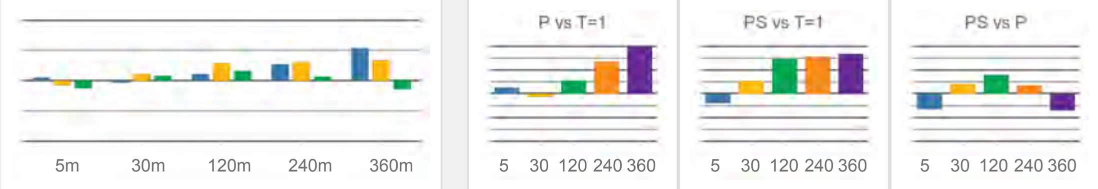

Locus		5m	30m	120m	240m	360m
**PGN_0927**	P vs T=1	-0.818	-0.377	-0.047	0.157	-0.054
	PS vs T=1	-0.981	0.249	1.561	2.136	2.201
	PS vs P	-0.299	0.539	1.525	1.895	2.158

conserved hypothetical protein

hypothetical proteins-Conserved

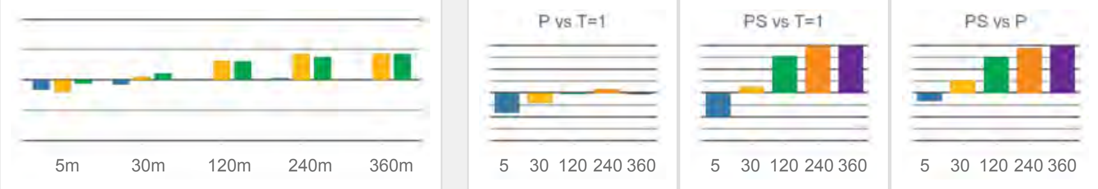

Locus		5m	30m	120m	240m	360m
**PGN_0928**	P vs T=1	-1.045	-1.279	-0.902	-0.610	-0.574
	PS vs T=1	-1.076	-0.244	1.214	1.484	1.418
	PS vs P	-0.144	0.829	1.966	1.978	1.904

conserved hypothetical protein

hypothetical proteins-Conserved

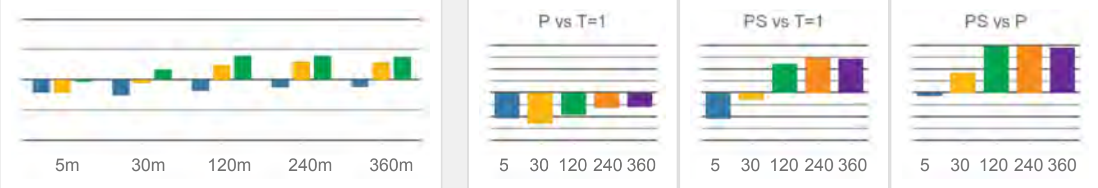

Locus		5m	30m	120m	240m	360m
**PGN_0929**	P vs T=1	-1.085	-1.440	-1.566	-1.595	-1.548
	PS vs T=1	-0.766	-0.513	0.298	0.243	0.039
	PS vs P	0.258	0.770	1.697	1.660	1.467

conserved hypothetical protein

hypothetical proteins-Conserved

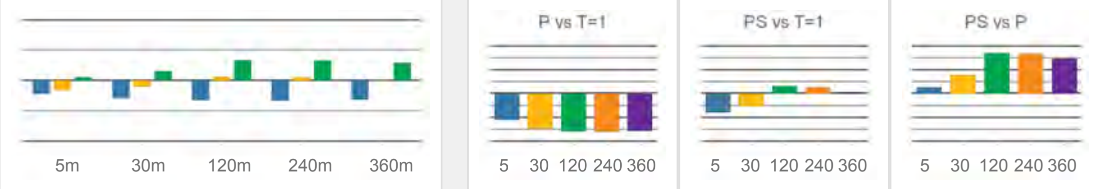

Locus		5m	30m	120m	240m	360m
**PGN_0930**	P vs T=1	-1.038	-1.264	-1.386	-1.861	-1.747
	PS vs T=1	-0.378	-0.117	-0.057	-0.261	-0.533
	PS vs P	0.550	0.951	1.176	1.278	1.025

hypothetical protein

hypothetical proteins

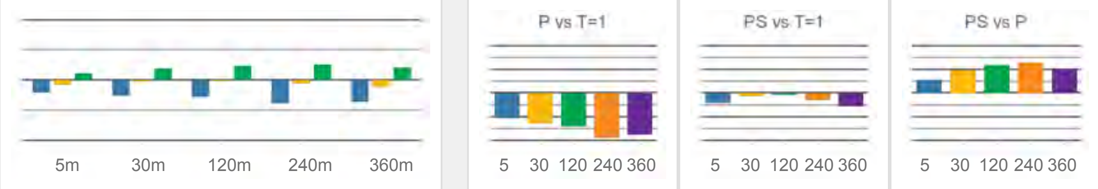

Locus		5m	30m	120m	240m	360m
**PGN_0931**	P vs T=1	-1.492	-2.044	-2.396	-2.354	-2.112
	PS vs T=1	-0.603	-0.469	-0.699	-1.325	-1.809
	PS vs P	0.851	1.437	1.536	0.885	0.243

conserved hypothetical protein

hypothetical proteins-Conserved

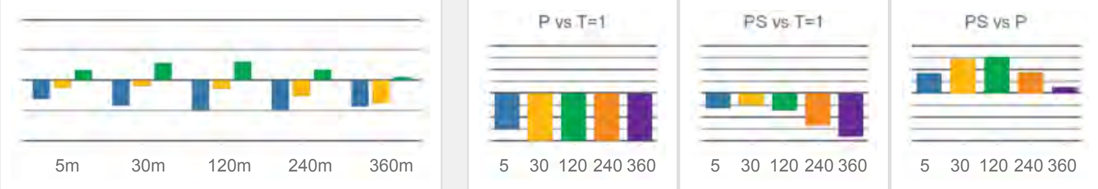

PgSg Time Course

Hackett Laboratory UW

log2 (Fold Change)

Legend: P vs T=1 · PS vs T=1 · PS vs P

Locus		5m	30m	120m	240m	360m
**PGN_0932**	P vs T=1	-1.134	-1.421	-1.550	-1.523	-1.673
	PS vs T=1	-0.107	0.087	-0.444	-0.727	-0.933
	PS vs P	0.941	1.349	0.965	0.665	0.617
	probable GCN5-related N-acetyltransferase					
	unknown function					
**PGN_0933**	P vs T=1	-1.243	-2.043	-2.767	-2.719	-2.059
	PS vs T=1	-0.710	-0.749	-1.217	-1.614	-1.732
	PS vs P	0.557	1.185	1.347	0.914	0.296
	probable transcriptional regulator					
	regulatory functions					
**PGN_0934**	P vs T=1	0.324	-0.315	-0.115	0.578	1.534
	PS vs T=1	-0.047	-0.002	0.280	0.480	0.848
	PS vs P	-0.341	0.195	0.264	-0.059	-0.561
	transposase in ISPg3					
**PGN_0935**	P vs T=1	0.485	-0.045	-0.635	-0.897	-1.419
	PS vs T=1	0.246	-0.210	-0.651	-0.660	-0.798
	PS vs P	-0.192	-0.150	-0.028	0.216	0.585
	conserved hypothetical protein					
	hypothetical proteins-Conserved					
**PGN_0936**	P vs T=1	1.498	2.427	2.627	2.487	1.965
	PS vs T=1	0.698	2.809	4.246	4.154	3.761
	PS vs P	-0.844	0.401	1.626	1.653	1.760
	putative glycerate dehydrogenase					
	central intermediary metabolism					
**PGN_0937**	P vs T=1	0.423	0.935	1.888	1.940	1.683
	PS vs T=1	-0.358	1.220	3.901	4.195	3.971
	PS vs P	-0.971	0.142	2.025	2.188	2.171
	hypothetical protein					
	hypothetical proteins					
**PGN_0938** *BioF*	P vs T=1	-0.293	-0.600	-0.674	-0.549	-0.790
	PS vs T=1	-0.728	-0.591	1.040	1.028	0.613
	PS vs P	-0.424	-0.021	1.657	1.530	1.352
	8-amino-7-oxononanoate synthase					
	biosynthesis of cofactors, prosthetic groups, and carriers					

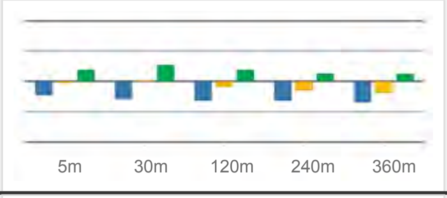
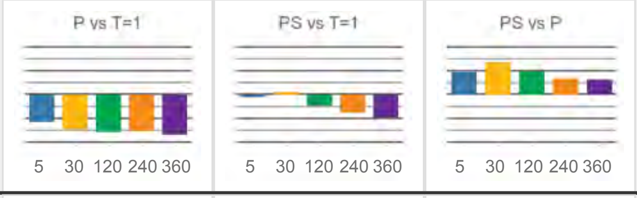
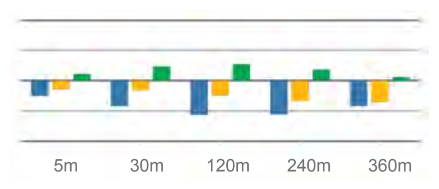
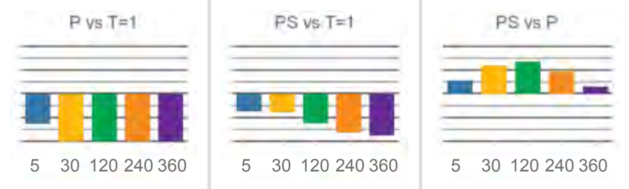
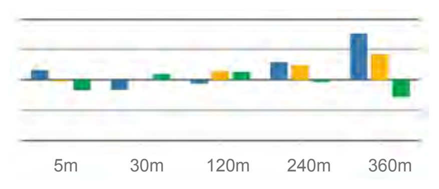
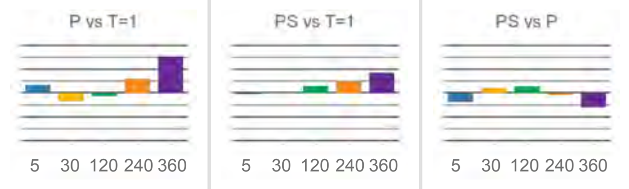
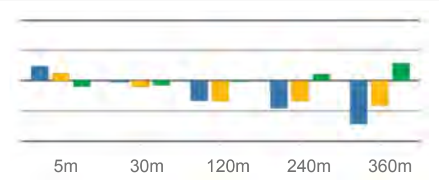
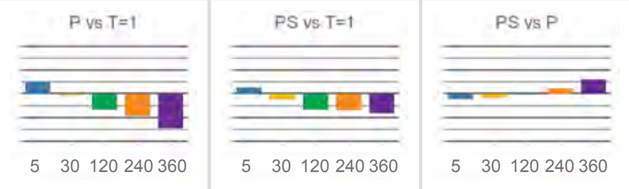
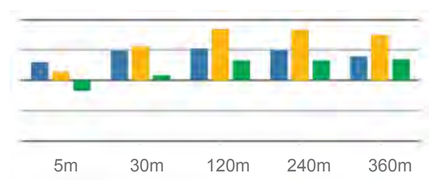
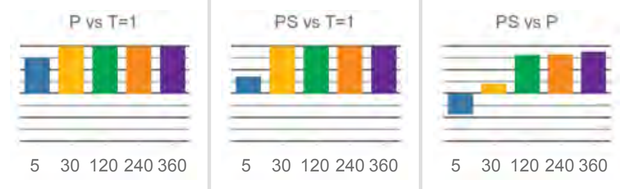
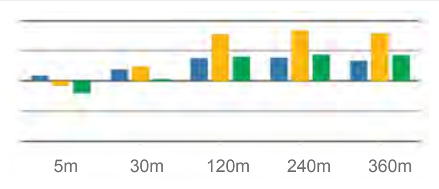
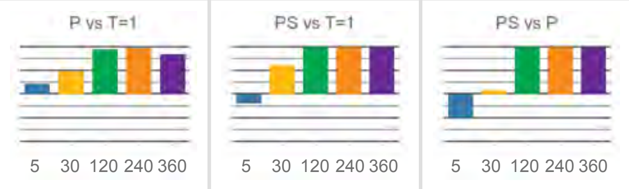
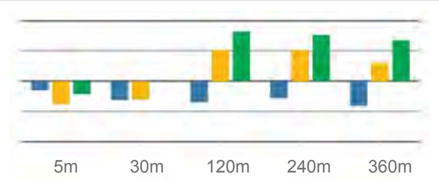
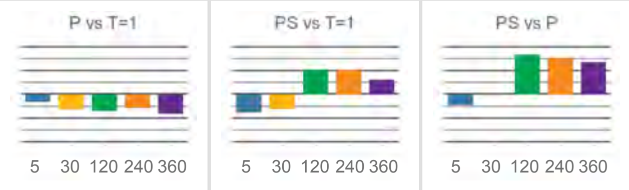

PgSg Time Course

log$_2$ (Fold Change)

P vs T=1 | PS vs T=1 | PS vs P

Locus		5m	30m	120m	240m	360m
**PGN_0939**	P vs T=1	-0.801	-0.940	-0.902	-0.731	-0.964
	PS vs T=1	-0.789	-0.634	0.413	0.400	0.135
	PS vs P	-0.018	0.245	1.238	1.078	1.026
	conserved hypothetical protein					
	hypothetical proteins-Conserved					
**PGN_0940**	P vs T=1	-0.667	-0.532	-0.251	-0.251	-0.422
	PS vs T=1	-0.466	-0.267	0.420	0.625	0.749
	PS vs P	0.106	0.190	0.642	0.813	1.080
	partial transposase in ISPg2					
**PGN_0941**	P vs T=1	-0.189	-0.751	-1.008	-1.093	-0.798
	PS vs T=1	-0.594	-1.014	-0.941	-0.701	-0.884
	PS vs P	-0.377	-0.266	0.039	0.337	-0.089
	conserved hypothetical protein					
	hypothetical proteins-Conserved					
**PGN_0942**	P vs T=1	-0.379	-0.624	-0.354	-0.496	0.054
	PS vs T=1	-0.429	-0.719	-0.905	-0.853	-0.744
	PS vs P	-0.039	-0.111	-0.519	-0.361	-0.736
	conserved hypothetical protein					
	hypothetical proteins-Conserved					
**PGN_0943**	P vs T=1	-0.968	-1.060	-1.061	-0.441	0.575
	PS vs T=1	-0.970	-1.499	-1.641	-1.377	-0.912
	PS vs P	-0.008	-0.447	-0.624	-0.812	-1.293
	alginate O-acetyltransferase					
	fatty acid and phospholipid metabolism					
**PGN_0944**	P vs T=1	0.122	-0.576	-0.653	0.115	1.185
	PS vs T=1	-0.256	-0.288	0.012	0.014	0.277
	PS vs P	-0.327	0.167	0.454	-0.077	-0.753
	transposase in ISPg3					
**PGN_0945**	P vs T=1	0.296	-0.341	-0.035	0.430	2.123
	PS vs T=1	-0.305	-0.865	-0.111	0.581	0.958
	PS vs P	-0.501	-0.614	-0.179	0.162	-0.804
	putative TetR family transcriptional regulator					
	regulatory functions					

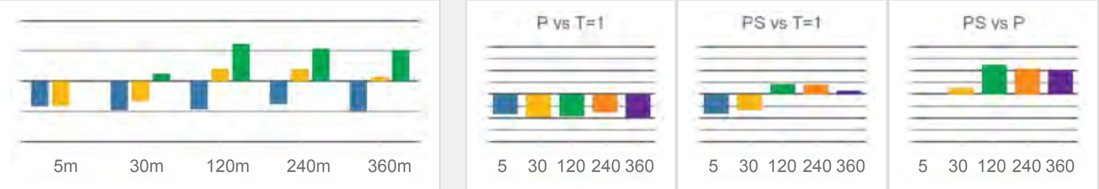

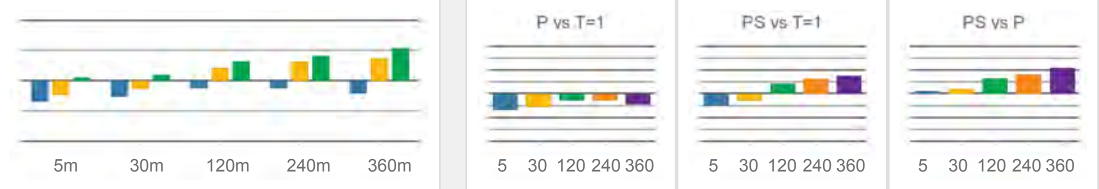

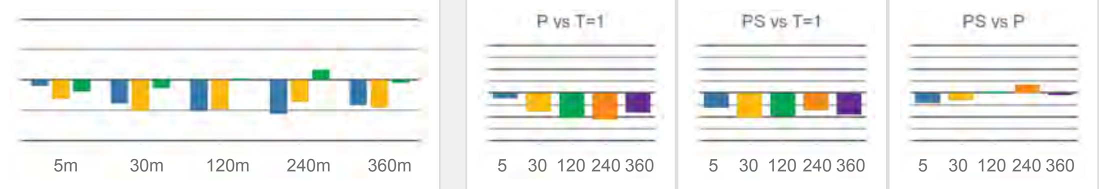

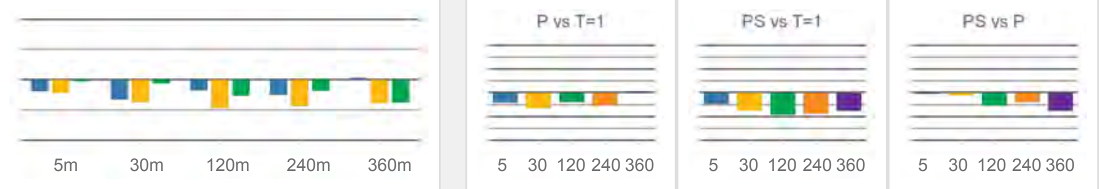

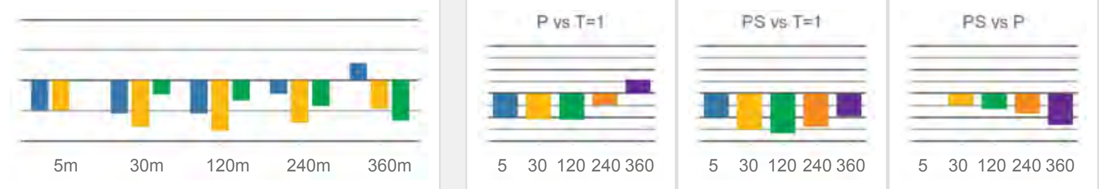

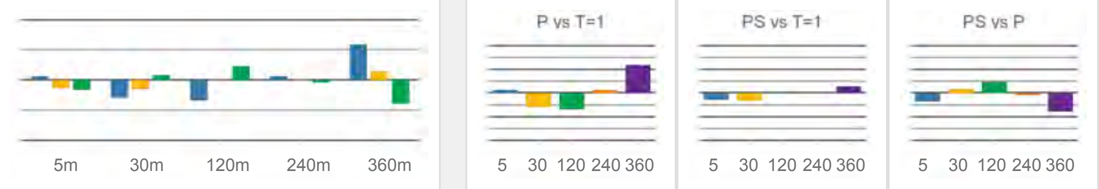

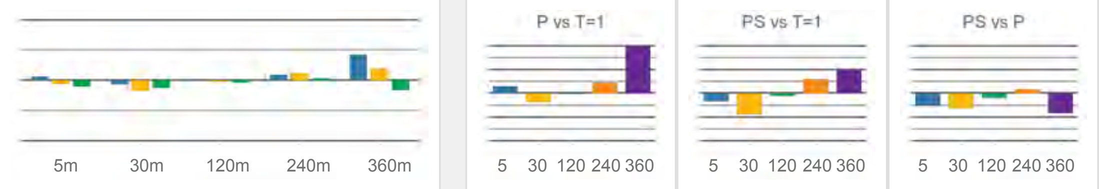

PgSg Time Course

log₂ (Fold Change)

Legend: P vs T=1 | PS vs T=1 | PS vs P

Locus		5m	30m	120m	240m	360m
**PGN_0946**	P vs T=1	0.480	0.353	0.882	1.487	2.612
	PS vs T=1	-0.095	-0.125	0.489	0.685	0.959
	PS vs P	-0.560	-0.502	-0.368	-0.651	-1.446
	conserved hypothetical protein					
	hypothetical proteins-Conserved					
**PGN_0947**	P vs T=1	0.417	1.055	1.391	1.157	2.106
	PS vs T=1	0.191	0.393	0.671	0.987	1.043
	PS vs P	-0.298	-0.546	-0.543	-0.138	-0.779
	conserved hypothetical protein					
	hypothetical proteins-Conserved					
**PGN_0948**	P vs T=1	0.347	0.498	1.009	1.701	2.372
	PS vs T=1	0.205	0.389	0.615	0.801	1.259
	PS vs P	-0.209	-0.159	-0.357	-0.639	-0.839
	conserved hypothetical protein					
	hypothetical proteins-Conserved					
**PGN_0949**	P vs T=1	0.936	0.827	0.737	1.698	2.735
	PS vs T=1	0.838	0.596	0.824	1.136	1.478
	PS vs P	-0.092	-0.248	-0.056	-0.407	-1.035
	ABC transporter ATP-binding protein					
	transport and binding proteins					
**PGN_0950**	P vs T=1	0.184	0.133	0.253	0.889	2.234
	PS vs T=1	0.056	-0.041	0.540	0.755	1.124
	PS vs P	-0.161	-0.227	0.175	-0.089	-0.914
	ABC transporter ATP-binding protein					
	transport and binding proteins					
**PGN_0951**	P vs T=1	0.248	0.343	0.810	1.357	2.715
	PS vs T=1	0.657	0.740	1.052	1.557	1.665
	PS vs P	0.245	0.216	0.125	0.249	-0.723
	conserved hypothetical protein					
	hypothetical proteins-Conserved					
**PGN_0952**	P vs T=1	-0.305	-0.732	-1.034	-1.602	-2.224
	PS vs T=1	-1.017	-1.146	-1.274	-1.367	-1.421
	PS vs P	-0.661	-0.398	-0.236	0.197	0.745
	carboxyl-terminal processing protease					
	protein fate					

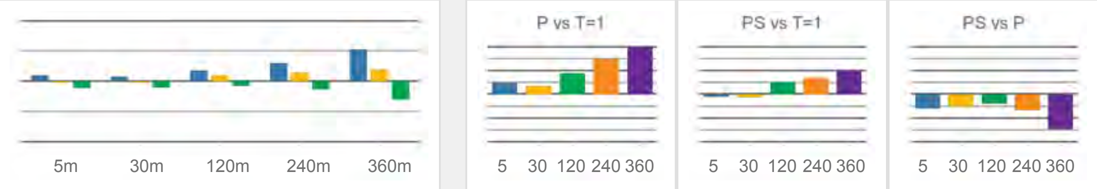

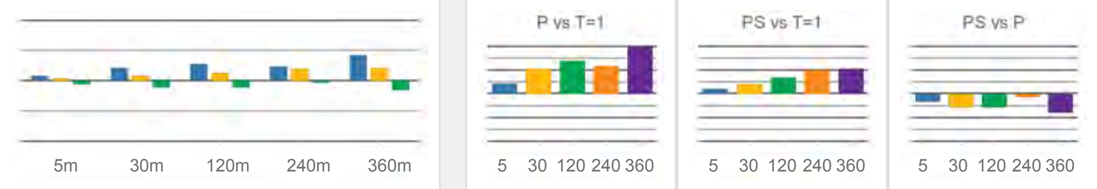

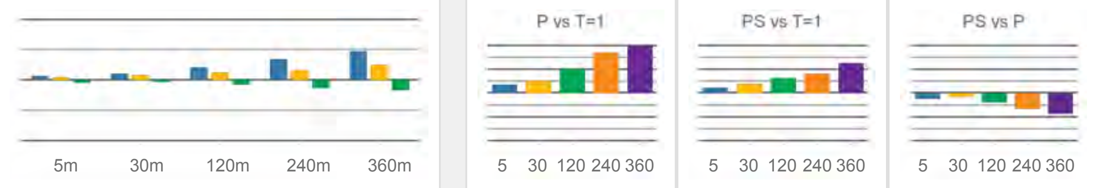

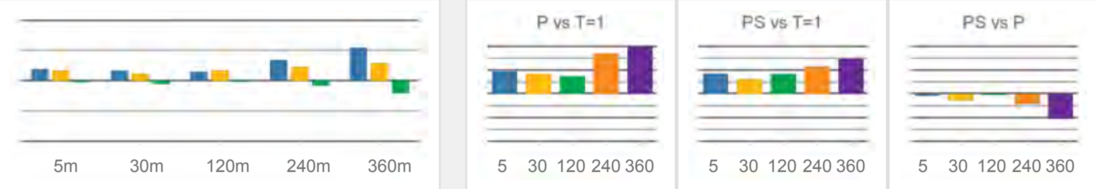

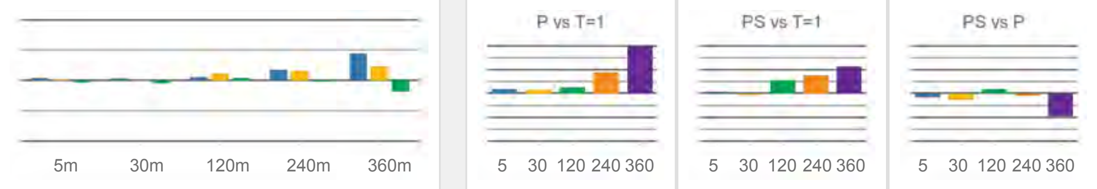

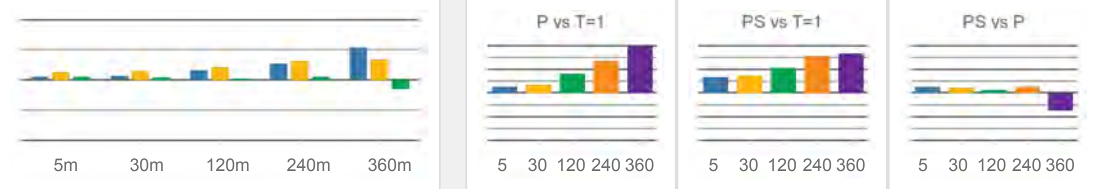

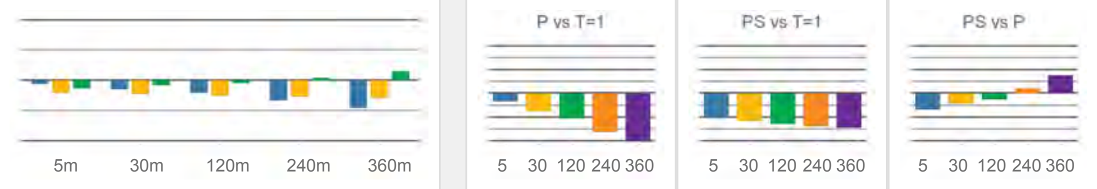

PgSg Time Course

log2 (Fold Change)

P vs T=1 | PS vs T=1 | PS vs P

Locus		5m	30m	120m	240m	360m
**PGN_0953**	P vs T=1	0.361	-0.219	-0.079	0.508	1.692
	PS vs T=1	0.022	-0.057	0.554	0.564	0.777
	PS vs P	-0.313	0.061	0.498	0.058	-0.775
	transposase in ISPg3					
**PGN_0954**	P vs T=1	0.661	-0.031	0.892	1.923	2.997
	PS vs T=1	-0.132	-0.253	0.749	0.944	1.524
	PS vs P	-0.682	-0.444	-0.131	-0.556	-1.033
	partial transposase in ISPg6					
**PGN_0955**	P vs T=1	0.237	-0.357	-0.375	0.471	1.487
	PS vs T=1	-0.071	-0.077	0.351	0.273	0.734
	PS vs P	-0.284	0.166	0.523	-0.159	-0.621
	transposase in ISPg3					
**PGN_0956**	P vs T=1	-0.910	-1.026	-0.471	-0.187	-0.622
	PS vs T=1	-0.808	-0.517	0.281	0.482	0.422
	PS vs P	0.007	0.346	0.705	0.686	0.932
	hypothetical protein					
	hypothetical proteins					
**PGN_0957**	P vs T=1	-0.541	-1.057	-0.830	-0.486	0.004
	PS vs T=1	-0.397	0.018	0.694	0.910	0.868
	PS vs P	0.074	0.803	1.272	1.215	0.843
	conserved hypothetical protein					
	hypothetical proteins-Conserved					
**PGN_0958**	P vs T=1	-0.272	-0.519	-0.864	-0.166	1.383
	PS vs T=1	-0.313	-0.143	0.744	1.071	0.999
	PS vs P	-0.180	0.024	0.707	0.717	0.045
	conserved hypothetical protein					
	hypothetical proteins-Conserved					
**PGN_0959**	P vs T=1	0.060	-0.149	0.086	0.642	0.851
	PS vs T=1	0.147	0.413	1.139	1.264	1.108
	PS vs P	0.026	0.399	0.842	0.597	0.270
	probable transcriptional regulator					
	regulatory functions					

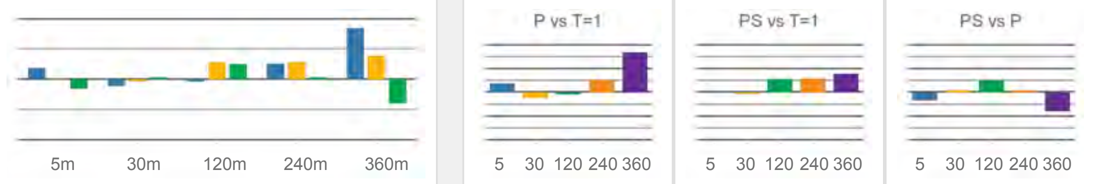

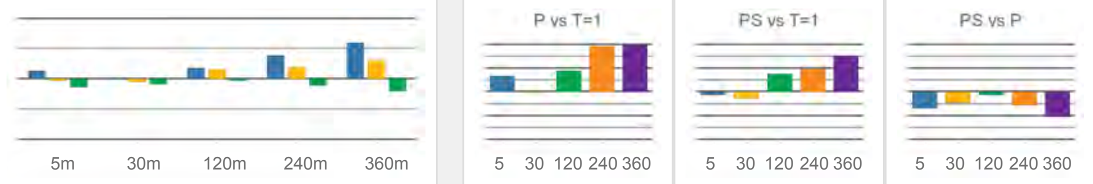

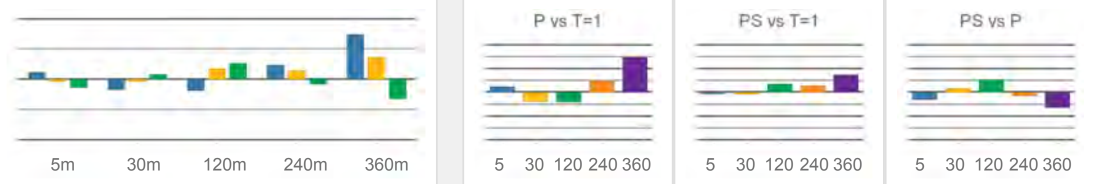

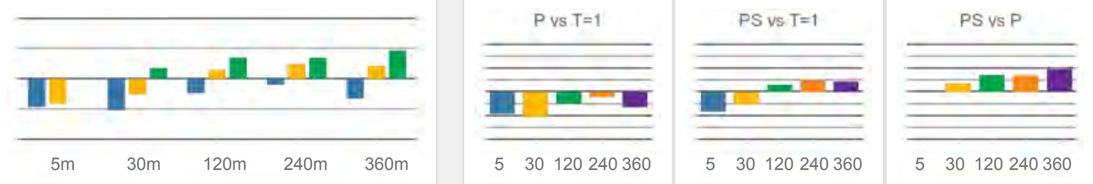

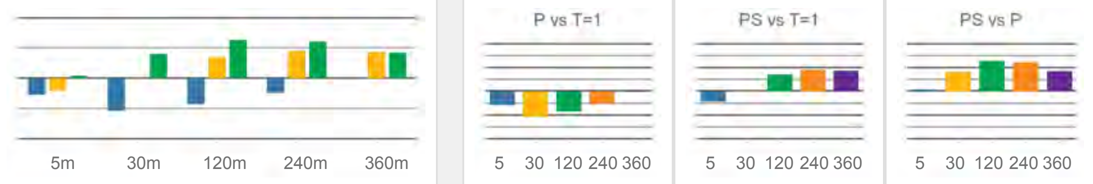

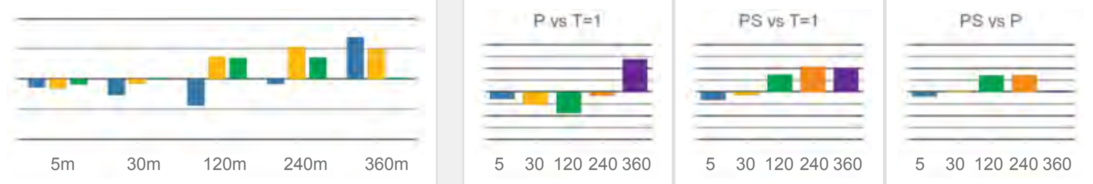

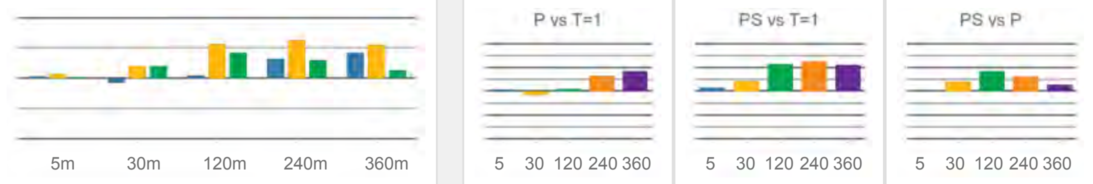

PgSg Time Course

log$_2$ (Fold Change)

Legend: P vs T=1 | PS vs T=1 | PS vs P

Locus		5m	30m	120m	240m	360m
PGN_0960	P vs T=1	0.206	0.020	-0.610	-0.302	-0.324
	PS vs T=1	0.168	0.201	0.226	-0.110	-0.121
	PS vs P	-0.027	0.181	0.752	0.165	0.180

conserved hypothetical protein

hypothetical proteins-Conserved

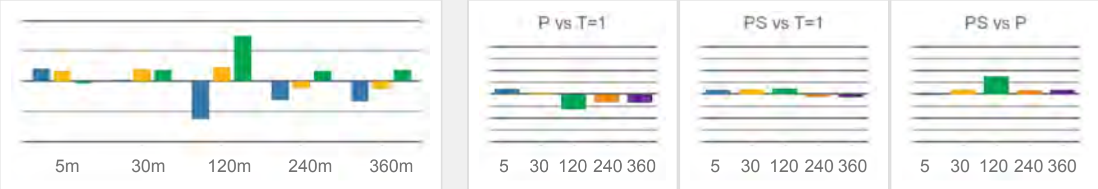

Locus		5m	30m	120m	240m	360m
PGN_0961	P vs T=1	0.574	0.081	1.553	2.005	3.560
	PS vs T=1	-0.013	0.425	1.458	0.827	1.581
	PS vs P	-0.411	-0.114	0.333	-0.266	-0.356

conserved hypothetical protein

hypothetical proteins-Conserved

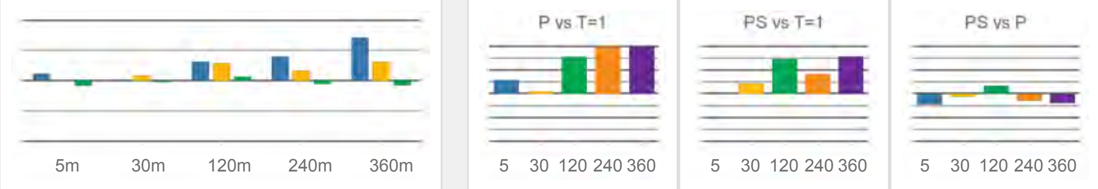

Locus		5m	30m	120m	240m	360m
PGN_0962	P vs T=1	-0.118	-0.673	-1.585	-2.154	-2.535
	PS vs T=1	-0.771	-0.850	-1.146	-1.258	-1.572
	PS vs P	-0.569	-0.141	0.411	0.816	0.890

threonyl-tRNA synthetase

protein synthesis

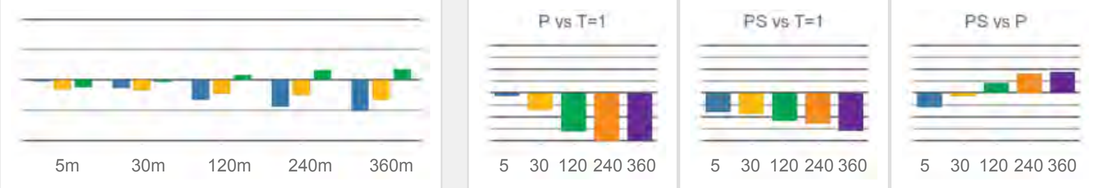

Locus		5m	30m	120m	240m	360m
PGN_0963	P vs T=1	-0.373	-0.572	-0.765	-1.198	-1.988
	PS vs T=1	-0.835	-0.685	-0.857	-1.147	-1.419
	PS vs P	-0.430	-0.096	-0.078	0.039	0.517

translation initiation factor IF-3

protein synthesis

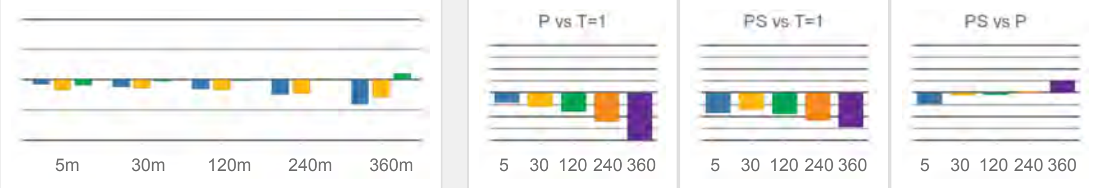

Locus		5m	30m	120m	240m	360m
PGN_0964 *rpmI*	P vs T=1	-0.560	-0.485	-0.195	-0.582	-1.720
	PS vs T=1	-0.874	-0.270	0.124	-0.292	-0.467
	PS vs P	-0.315	0.213	0.348	0.288	1.176

probable 50S ribosomal protein L35

protein synthesis

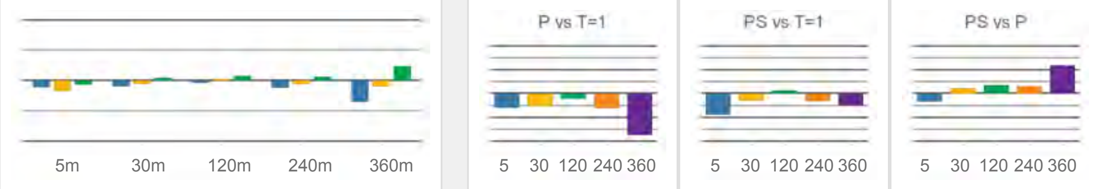

Locus		5m	30m	120m	240m	360m
PGN_0965 *rplT*	P vs T=1	-0.756	-0.547	-0.086	-0.280	-0.888
	PS vs T=1	-1.394	-0.830	-0.280	-0.205	-0.189
	PS vs P	-0.642	-0.275	-0.157	0.091	0.676

putative 50S ribosomal protein L20

protein synthesis

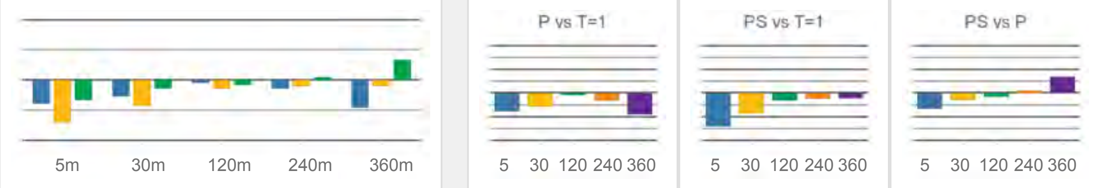

Locus		5m	30m	120m	240m	360m
PGN_0966	P vs T=1	0.126	-0.131	-0.382	-0.313	-0.107
	PS vs T=1	-0.498	-1.052	-1.239	-0.939	-0.536
	PS vs P	-0.589	-0.899	-0.876	-0.637	-0.424

partial transposase in ISPg1

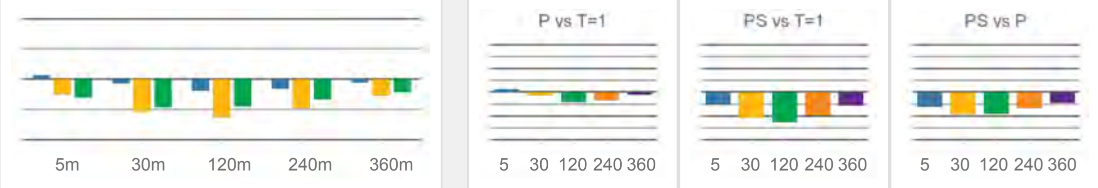

log2 (Fold Change)

Legend: P vs T=1 | PS vs T=1 | PS vs P

Locus		5m	30m	120m	240m	360m
**PGN_0967**	P vs T=1	0.881	0.823	0.951	0.791	0.706
	PS vs T=1	0.452	0.180	0.111	0.132	0.435
	PS vs P	-0.409	-0.625	-0.809	-0.646	-0.273

partial transposase in ISPg1

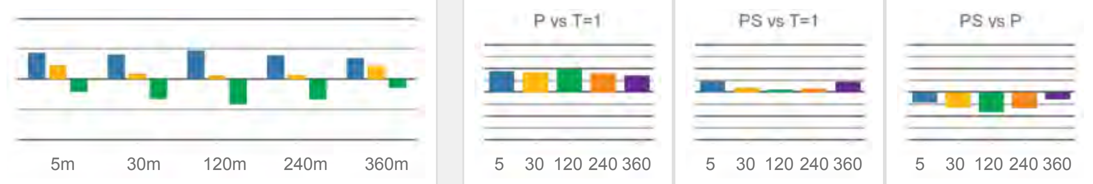

Locus		5m	30m	120m	240m	360m
**PGN_0968**	P vs T=1	1.349	1.755	2.555	2.850	2.871
	PS vs T=1	1.167	1.539	2.145	2.438	2.748
	PS vs P	-0.243	-0.221	-0.319	-0.294	-0.045

conserved hypothetical protein

hypothetical proteins-Conserved

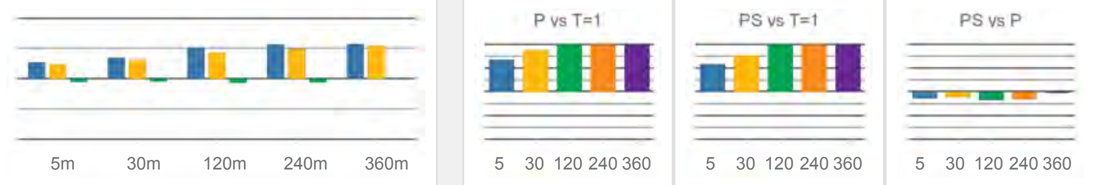

Locus		5m	30m	120m	240m	360m
**PGN_0969**	P vs T=1	-0.590	-0.013	0.943	1.361	1.519
	PS vs T=1	-0.423	0.089	0.883	1.336	1.644
	PS vs P	-0.101	-0.022	-0.004	0.091	0.210

conserved hypothetical protein

hypothetical proteins-Conserved

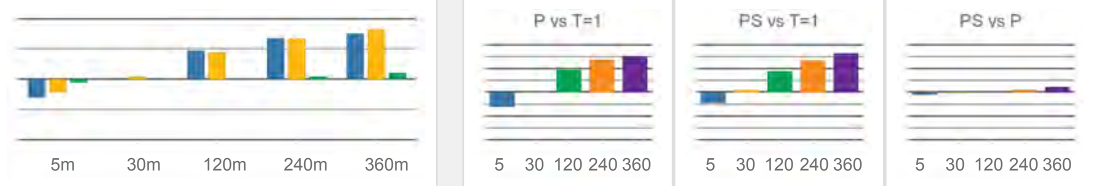

Locus		5m	30m	120m	240m	360m
**PGN_0970**	P vs T=1	0.543	0.966	1.648	2.144	1.992
	PS vs T=1	0.386	0.683	1.335	1.913	2.221
	PS vs P	-0.225	-0.296	-0.269	-0.129	0.268

putative RNA polymerase sigma-70 factor ECF subfamily

transcription

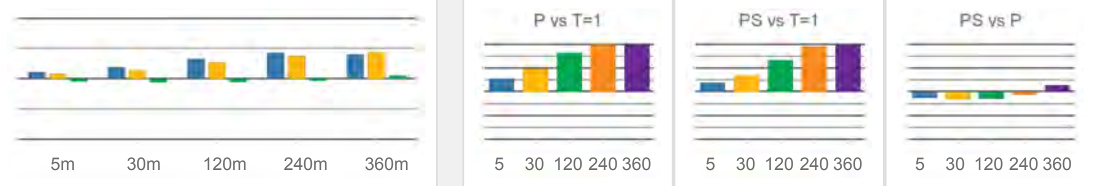

Locus		5m	30m	120m	240m	360m
**PGN_0971**	P vs T=1	0.375	0.310	0.397	0.481	0.145
	PS vs T=1	0.204	-0.018	0.129	0.251	0.241
	PS vs P	-0.170	-0.331	-0.271	-0.226	0.078

transposase in ISPg1

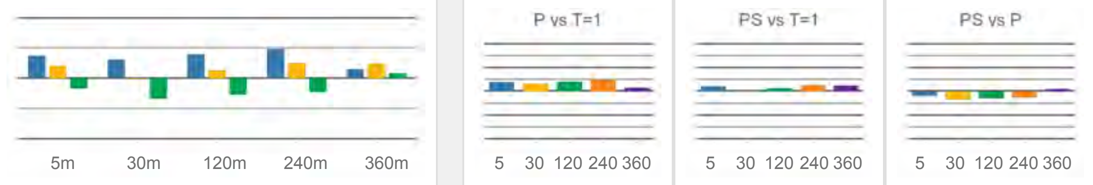

Locus		5m	30m	120m	240m	360m
**PGN_0972**	P vs T=1	-1.049	-1.331	-1.586	-1.825	-2.252
	PS vs T=1	-0.650	-0.299	0.171	0.144	-0.233
	PS vs P	0.332	0.890	1.599	1.765	1.813

TPR domain protein

unknown function

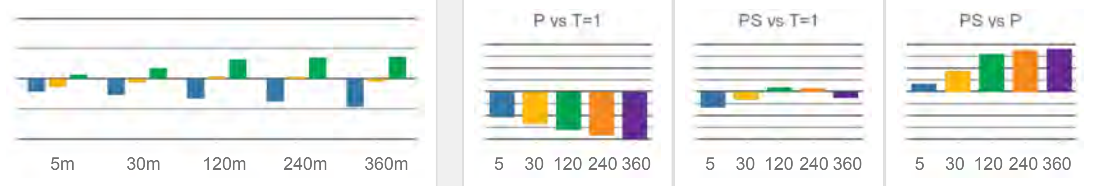

Locus		5m	30m	120m	240m	360m
**PGN_0973**	P vs T=1	-0.810	-0.917	-0.901	-0.860	-0.276
	PS vs T=1	-0.278	-0.848	-1.550	-1.522	-1.281
	PS vs P	0.525	0.059	-0.649	-0.657	-0.965

conserved hypothetical protein

hypothetical proteins-Conserved

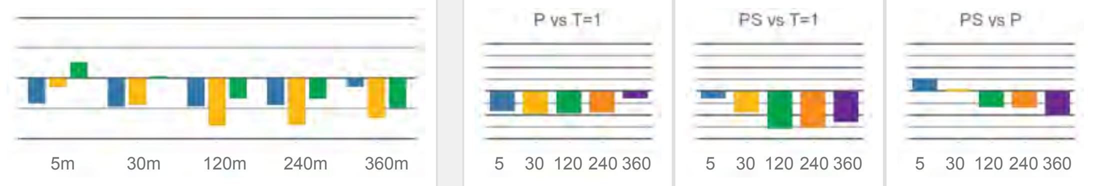

PgSg Time Course

log$_2$ (Fold Change)

Legend: P vs T=1 | PS vs T=1 | PS vs P

PGN_0974

Locus		5m	30m	120m	240m	360m
PGN_0974	P vs T=1	-1.188	-1.328	-1.369	-1.136	-0.323
	PS vs T=1	-1.083	-1.861	-2.203	-1.900	-1.390
	PS vs P	0.111	-0.527	-0.839	-0.714	-0.952

putative shikimate 5-dehydrogenase

amino acid biosynthesis

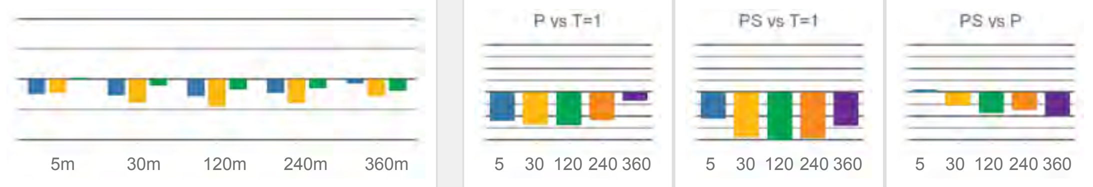

PGN_0975

Locus		5m	30m	120m	240m	360m
PGN_0975 *UbiE*	P vs T=1	-0.897	-0.945	-1.116	-1.090	-0.924
	PS vs T=1	-0.801	-1.504	-1.677	-1.785	-1.947
	PS vs P	0.107	-0.538	-0.550	-0.663	-0.984

putative ubiquinone/menaquinone biosynthesis methyltransferase

biosynthesis of cofactors, prosthetic groups, and carriers

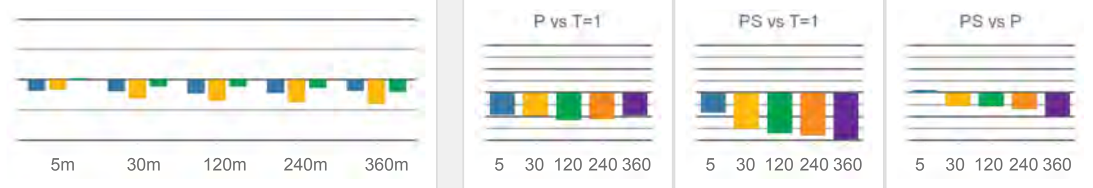

PGN_0976

Locus		5m	30m	120m	240m	360m
PGN_0976	P vs T=1	0.200	0.408	0.241	-0.123	-0.316
	PS vs T=1	0.366	0.098	-0.334	-0.516	-0.423
	PS vs P	0.176	-0.272	-0.537	-0.391	-0.117

phosphoribosylaminoimidazole-succinocarboxamide synthase

purines, pyrimidines, nucleosides and nucleotides

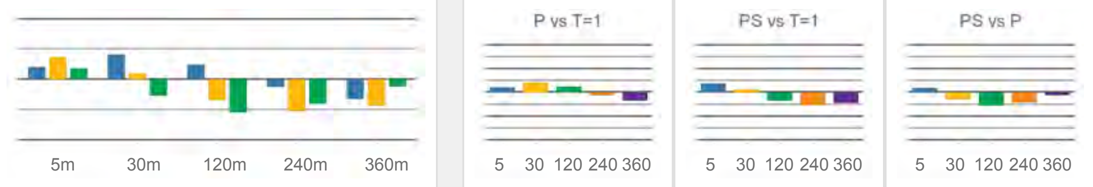

PGN_0977

Locus		5m	30m	120m	240m	360m
PGN_0977	P vs T=1	0.030	-0.396	-0.707	-0.692	-0.485
	PS vs T=1	-0.286	-0.599	-0.851	-0.994	-0.863
	PS vs P	-0.284	-0.197	-0.165	-0.314	-0.364

phosphate starvation-inducible PhoH-like protein

unknown function

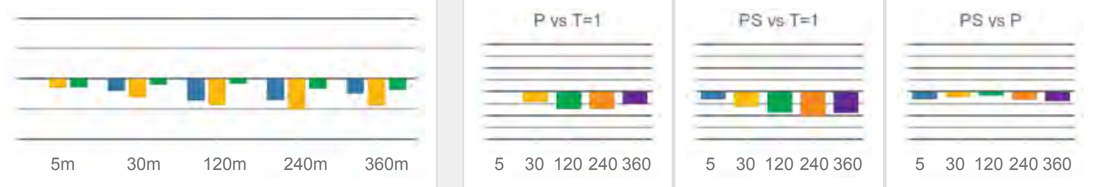

PGN_0978

Locus		5m	30m	120m	240m	360m
PGN_0978	P vs T=1	-0.427	-0.571	0.005	0.117	0.266
	PS vs T=1	-0.435	-0.369	0.217	0.340	0.530
	PS vs P	-0.076	0.025	0.261	0.276	0.342

hypothetical protein

hypothetical proteins

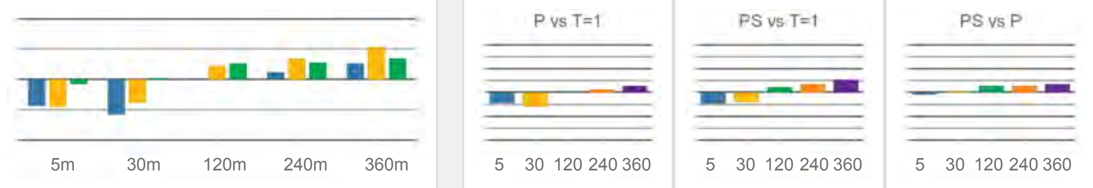

PGN_0979

Locus		5m	30m	120m	240m	360m
PGN_0979	P vs T=1	-0.594	-0.282	0.019	0.044	0.934
	PS vs T=1	-0.041	-0.680	-0.222	-0.324	0.763
	PS vs P	0.252	-0.363	-0.136	-0.281	0.321

hypothetical protein

hypothetical proteins

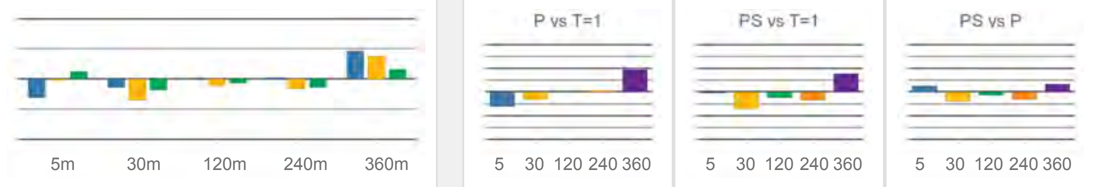

PGN_0980

Locus		5m	30m	120m	240m	360m
PGN_0980	P vs T=1	-0.659	-0.647	-0.781	-0.643	0.072
	PS vs T=1	-0.643	-0.952	-0.862	-0.659	-0.811
	PS vs P	0.013	-0.302	-0.101	-0.025	-0.834

putative alpha-1,2-mannosidase family protein

cell envelope

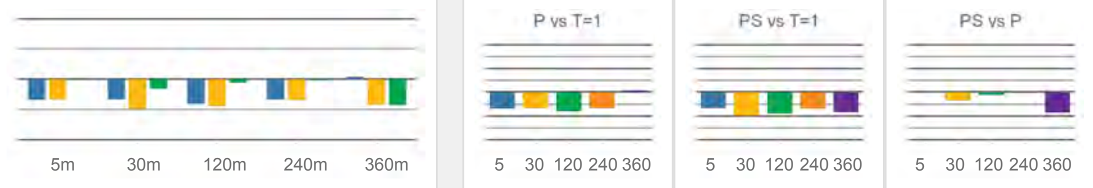

log2 (Fold Change)

Legend: P vs T=1 · PS vs T=1 · PS vs P

Locus		5m	30m	120m	240m	360m
**PGN_0981**	P vs T=1	0.042	0.107	0.404	1.013	2.037
	PS vs T=1	-0.374	-0.227	0.111	0.802	0.878
	PS vs P	-0.424	-0.342	-0.299	-0.105	-1.017
	S-adenosylmethionine:tRNA ribosyltransferase-isomerase					
	protein synthesis					
**PGN_0982**	P vs T=1	-0.546	-1.212	-1.205	-1.209	-0.696
	PS vs T=1	-0.831	-0.167	0.989	0.894	0.536
	PS vs P	-0.273	0.864	2.015	1.886	1.182
	putative Mrr restriction system protein					
	DNA metabolism					
**PGN_0983**	P vs T=1	1.884	1.428	1.736	1.784	2.345
	PS vs T=1	0.887	0.156	0.351	0.297	1.638
	PS vs P	-0.704	-0.980	-0.963	-0.918	-0.242
	hypothetical protein					
	hypothetical proteins					
**PGN_0984**	P vs T=1	-1.223	-1.670	-2.165	-1.875	-2.091
	PS vs T=1	-0.812	-1.173	-1.227	-1.217	-1.482
	PS vs P	0.406	0.436	0.781	0.572	0.505
	probable phosphatidylserine decarboxylase proenzyme					
	unknown function					
**PGN_0985**	P vs T=1	0.302	-0.392	-0.934	-0.947	-0.422
	PS vs T=1	0.792	0.081	-0.116	-0.205	-0.237
	PS vs P	0.526	0.428	0.661	0.549	0.169
	probable phosphatidylserine synthase					
	central intermediary metabolism					
**PGN_0986**	P vs T=1	0.554	-0.202	-0.849	-0.788	-0.598
	PS vs T=1	1.524	0.888	0.333	-0.493	-0.826
	PS vs P	1.022	0.999	0.914	0.084	-0.287
	hypothetical protein					
	hypothetical proteins					
**PGN_0987**	P vs T=1	0.562	0.311	-0.292	-0.864	-1.268
	PS vs T=1	0.685	0.623	-0.063	-0.575	-0.803
	PS vs P	0.141	0.322	0.226	0.250	0.426
	prolyl-tRNA synthetase					
	protein synthesis					

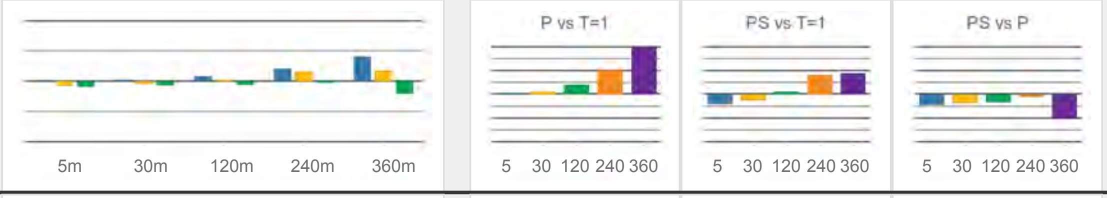
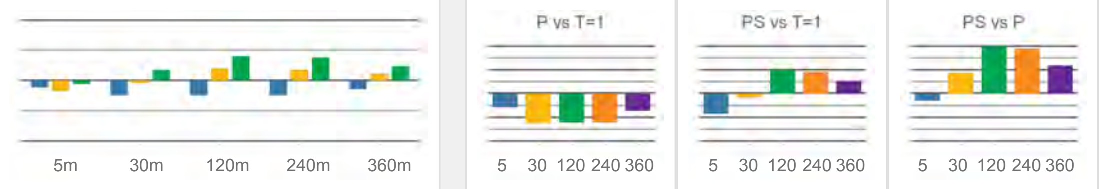
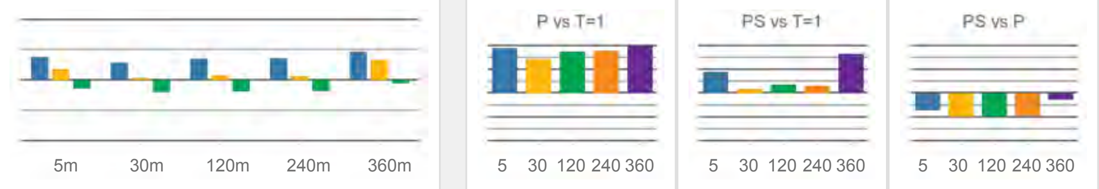
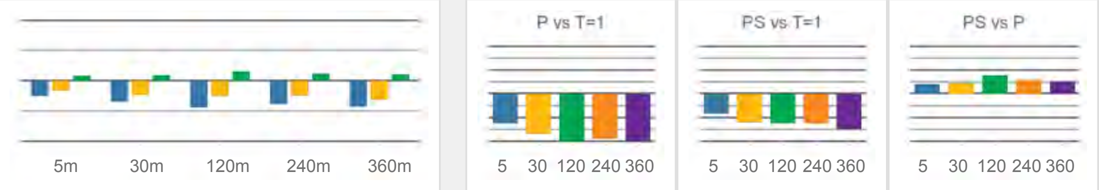
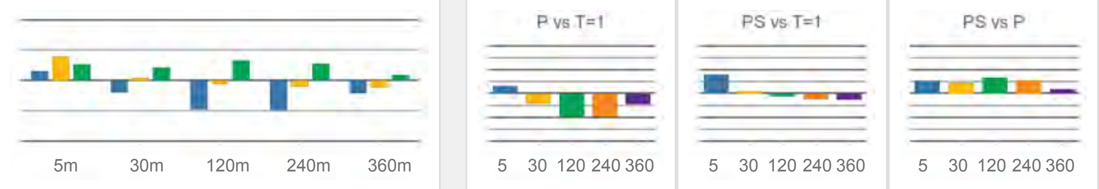
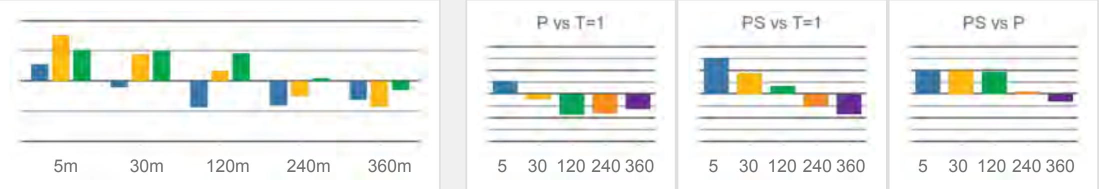
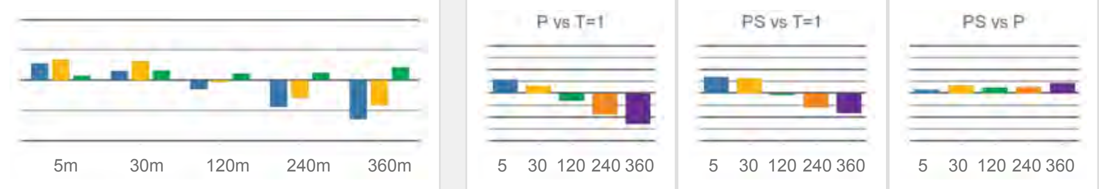

log$_2$ (Fold Change)

Locus		5m	30m	120m	240m	360m
**PGN_0988**	P vs T=1	0.291	0.712	0.637	0.149	-0.463
	PS vs T=1	0.417	0.689	0.093	-0.316	-0.347
	PS vs P	0.119	-0.000	-0.510	-0.471	0.057

hypothetical protein

hypothetical proteins

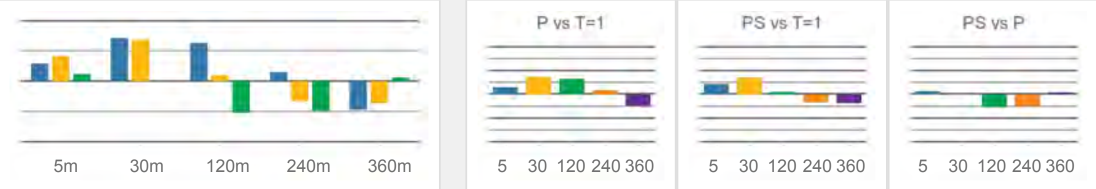

Locus		5m	30m	120m	240m	360m
**PGN_0989**	P vs T=1	0.104	0.264	-0.107	-0.647	-0.892
	PS vs T=1	0.628	0.565	0.033	-0.577	-0.746
	PS vs P	0.519	0.316	0.142	0.000	0.087

putative methyltransferase

hypothetical proteins-Conserved

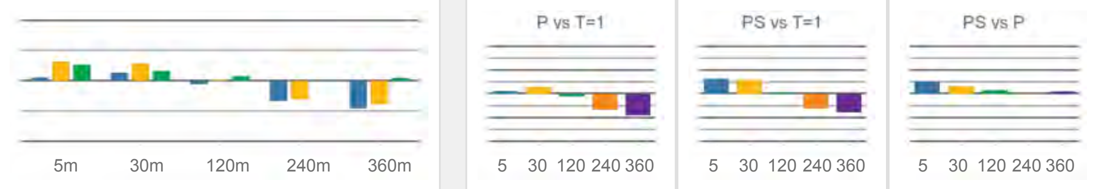

Locus		5m	30m	120m	240m	360m
**PGN_0990**	P vs T=1	0.223	0.070	0.044	-0.060	-0.413
	PS vs T=1	0.350	-0.063	-0.424	-0.844	-0.876
	PS vs P	0.147	-0.121	-0.445	-0.758	-0.468

ATP-binding protein Mrp/Nbp35 family

unknown function

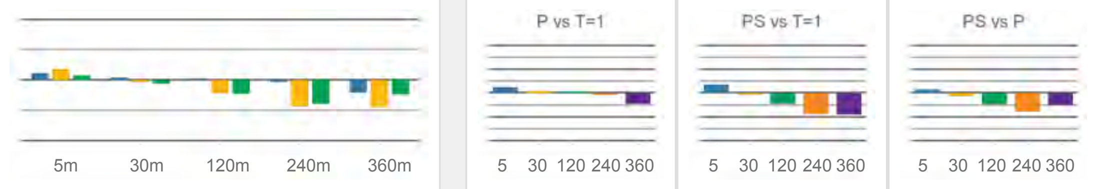

Locus		5m	30m	120m	240m	360m
**PGN_0991**	P vs T=1	-0.533	-0.939	-1.307	-0.932	-0.252
	PS vs T=1	-0.169	-0.611	-1.497	-1.692	-1.275
	PS vs P	0.375	0.313	-0.247	-0.749	-0.975

putative ribonuclease BN

transcription

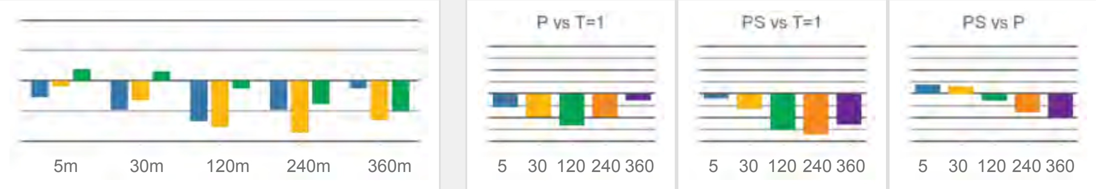

Locus		5m	30m	120m	240m	360m
**PGN_0992**	P vs T=1	-0.384	-0.751	-1.002	-1.224	-0.467
RibF	PS vs T=1	0.587	0.219	-0.624	-1.051	-0.940
	PS vs P	0.964	0.930	0.311	0.021	-0.452

putative riboflavin biosynthesis protein

biosynthesis of cofactors, prosthetic groups, and carriers

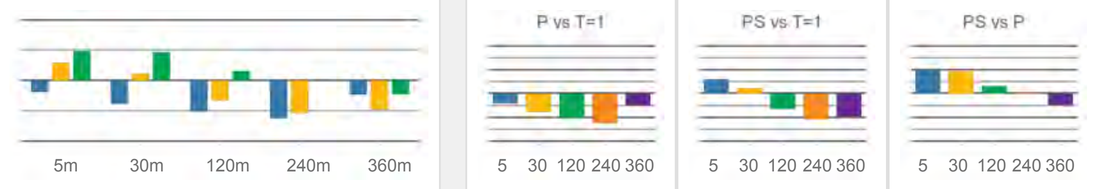

Locus		5m	30m	120m	240m	360m
**PGN_0993**	P vs T=1	-0.015	-0.075	-0.338	-0.838	-1.192
	PS vs T=1	0.902	0.843	-0.100	-0.823	-1.049
	PS vs P	0.911	0.910	0.239	-0.037	0.085

putative peptidase M23/M37 family

protein fate

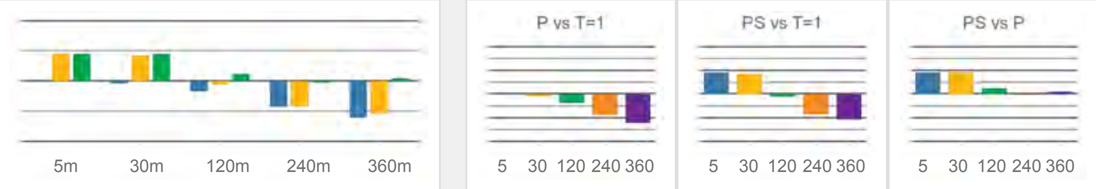

Locus		5m	30m	120m	240m	360m
**PGN_0994**	P vs T=1	0.556	0.253	-0.389	-0.682	-0.512
	PS vs T=1	1.172	0.825	-0.386	-0.476	-0.626
	PS vs P	0.627	0.573	-0.039	0.100	-0.144

conserved hypothetical protein

hypothetical proteins-Conserved

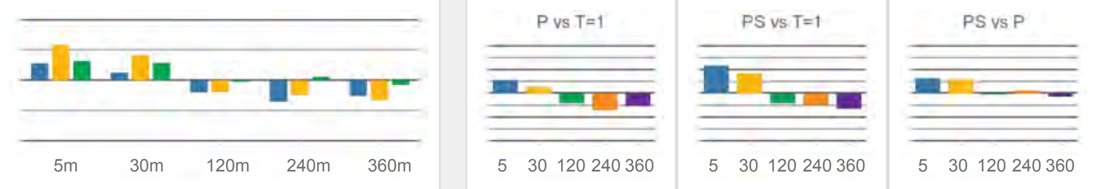

log2 (Fold Change)

P vs T=1 | PS vs T=1 | PS vs P

Locus		5m	30m	120m	240m	360m
**PGN_0995**	P vs T=1	0.256	-0.212	-1.134	-1.377	-0.879
	PS vs T=1	0.593	-0.019	-1.146	-1.458	-1.701
	PS vs P	0.373	0.205	-0.105	-0.246	-0.819
	TPR domain protein					
	unknown function					
**PGN_0996**	P vs T=1	-0.521	-1.155	-1.662	-1.772	-0.996
	PS vs T=1	0.236	-0.498	-1.722	-1.862	-1.860
	PS vs P	0.789	0.628	-0.131	-0.193	-0.815
	TPR domain protein					
	unknown function					
**PGN_0997**	P vs T=1	-0.215	-0.234	-0.917	-1.015	-0.743
	PS vs T=1	0.524	0.016	-1.109	-1.132	-1.067
	PS vs P	0.748	0.278	-0.263	-0.212	-0.315
	putative deoxyuridine 5'-triphosphate nucleotidohydrolase					
	purines, pyrimidines, nucleosides and nucleotides					
**PGN_0998**	P vs T=1	-0.148	-0.133	-0.618	-1.006	-1.003
	PS vs T=1	0.285	-0.342	-1.318	-1.593	-1.458
	PS vs P	0.465	-0.151	-0.676	-0.612	-0.463
	4-hydroxy-3-methylbut-2-en-1-yl diphosphate synthase					
	unknown function					
**PGN_0999**	P vs T=1	0.029	0.346	0.087	-0.545	-1.100
	PS vs T=1	-0.228	-0.068	-0.222	-0.496	-0.643
	PS vs P	-0.249	-0.381	-0.286	0.018	0.403
	putative phosphoribosylaminoimidazole carboxylase					
	purines, pyrimidines, nucleosides and nucleotides					
**PGN_1000**	P vs T=1	-0.253	-0.096	-0.486	-1.359	-2.016
	PS vs T=1	-0.514	-0.315	-0.474	-0.599	-0.790
	PS vs P	-0.245	-0.185	0.028	0.669	1.109
	putative glycine cleavage system H protein					
	central intermediary metabolism					
**PGN_1001**	P vs T=1	0.029	-0.218	-0.509	-0.592	-0.446
	PS vs T=1	0.563	0.362	0.017	0.077	-0.225
	PS vs P	0.535	0.559	0.473	0.586	0.196
	conserved hypothetical protein					
	hypothetical proteins-Conserved					

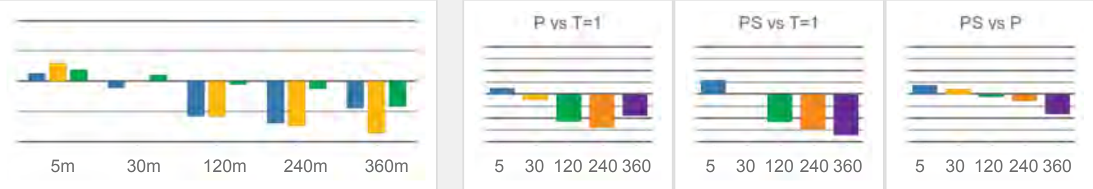

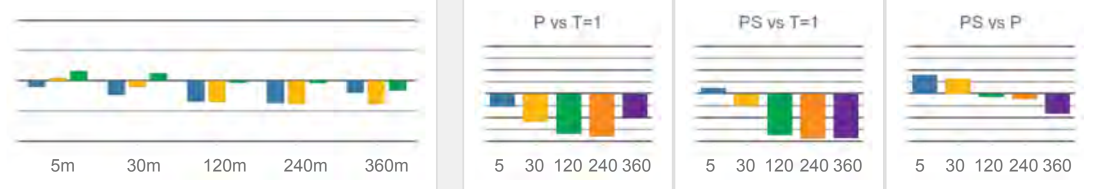

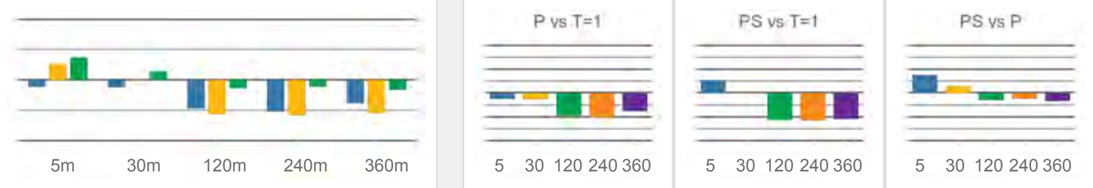

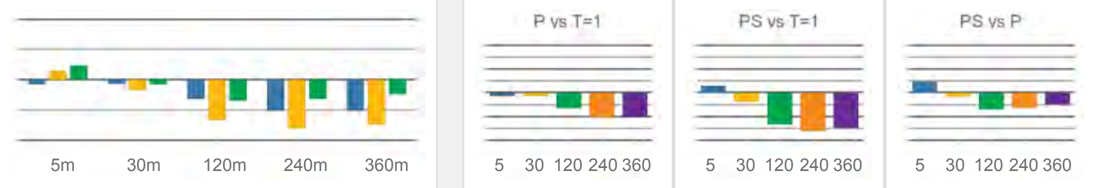

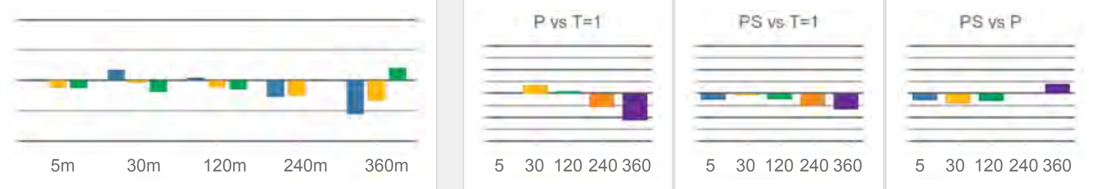

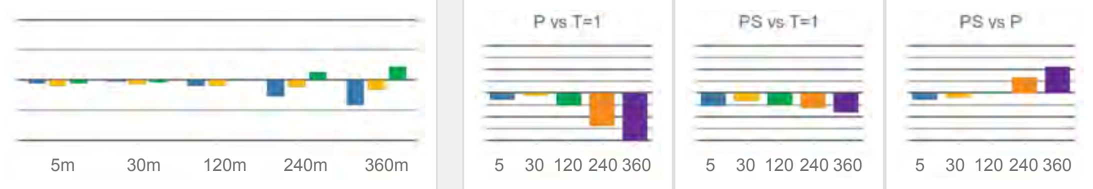

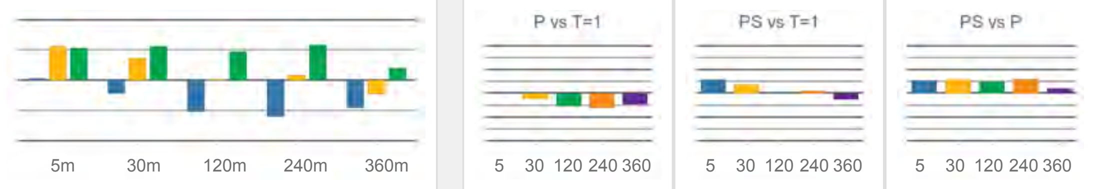

PgSg Time Course

log₂ (Fold Change) — Legend: P vs T=1, PS vs T=1, PS vs P

Locus		5m	30m	120m	240m	360m
**PGN_1002**	P vs T=1	-0.618	-0.408	-0.538	-0.662	-0.725
	PS vs T=1	-0.376	-0.508	-0.686	-0.427	-0.443
	PS vs P	0.226	-0.095	-0.155	0.203	0.256
	AMP nucleosidase					
	purines, pyrimidines, nucleosides and nucleotides					
**PGN_1003**	P vs T=1	-0.338	-0.381	-0.519	-0.747	-1.453
	PS vs T=1	-0.380	-0.008	0.669	0.629	0.309
	PS vs P	-0.049	0.357	1.155	1.302	1.628
	conserved hypothetical protein					
	hypothetical proteins-Conserved					
**PGN_1004**	P vs T=1	-0.383	-0.051	0.313	0.251	-0.205
	PS vs T=1	0.067	0.127	0.496	0.503	0.194
	PS vs P	0.397	0.161	0.203	0.263	0.364
	putative ABC transporter ATP-binding protein					
	transport and binding proteins					
**PGN_1005**	P vs T=1	0.104	0.336	0.815	0.850	0.433
	PS vs T=1	0.456	0.378	0.631	1.076	0.975
	PS vs P	0.323	0.028	-0.168	0.239	0.526
	probable ABC transporter permease protein					
	transport and binding proteins					
**PGN_1006**	P vs T=1	0.387	-0.241	-0.227	0.539	1.604
	PS vs T=1	-0.036	-0.009	0.464	0.520	0.643
	PS vs P	-0.374	0.103	0.490	0.015	-0.785
	transposase in ISPg3					
**PGN_1007**	P vs T=1	-0.366	-1.610	-0.126	0.635	1.610
	PS vs T=1	-0.659	-0.648	-0.492	0.035	0.276
	PS vs P	-0.223	0.054	-0.245	-0.092	-0.558
	conserved hypothetical protein					
	hypothetical proteins-Conserved					
**PGN_1008**	P vs T=1	0.664	0.652	0.792	0.815	0.535
	PS vs T=1	0.366	0.366	0.510	0.538	0.415
	PS vs P	-0.289	-0.283	-0.270	-0.261	-0.137
	partial transposase in ISPg1					

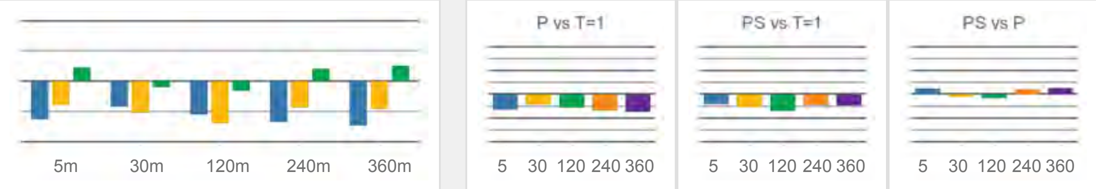

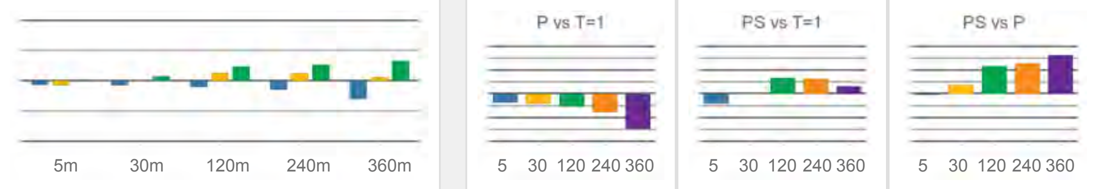

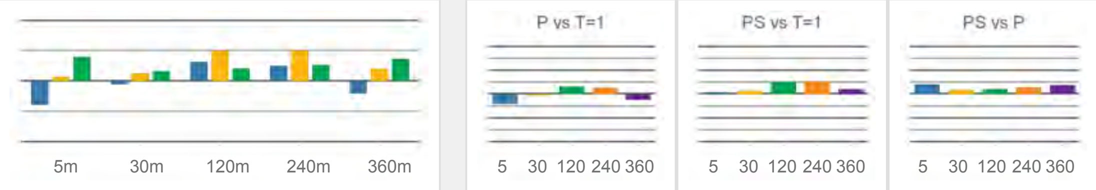

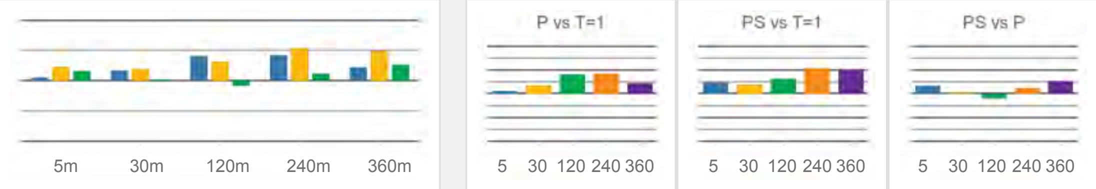

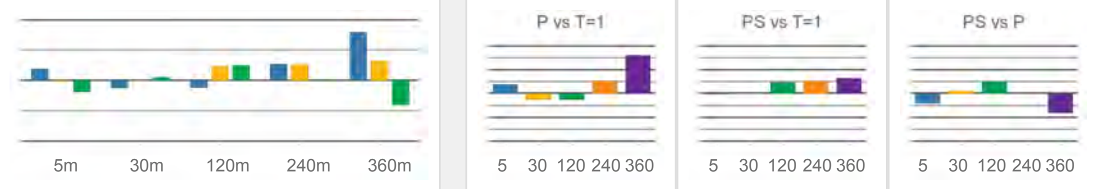

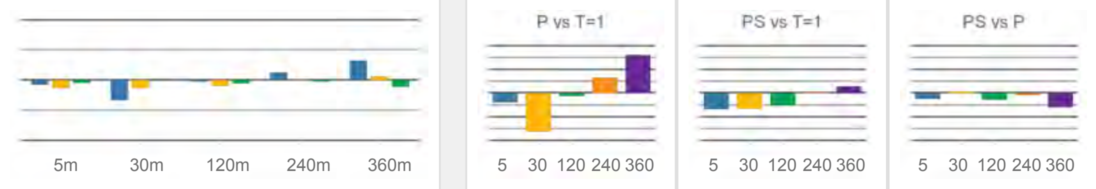

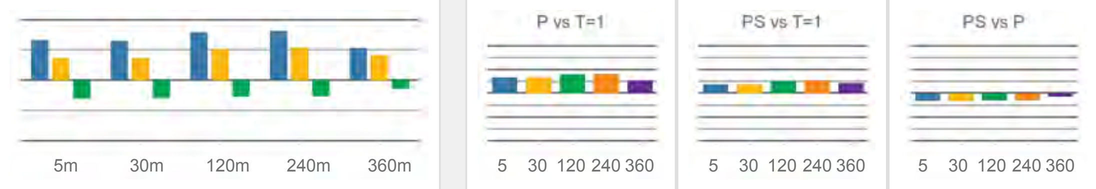

PgSg Time Course

log_2 (Fold Change)

Locus		5m	30m	120m	240m	360m
PGN_1009	P vs T=1	-2.706	-2.061	-1.324	-0.844	-0.787
	PS vs T=1	-2.031	-1.748	-1.229	-1.115	-1.039
	PS vs P	0.331	0.155	0.074	-0.196	-0.218
	calcium-transporting ATPase					
	transport and binding proteins					
PGN_1010	P vs T=1	0.121	0.516	0.590	0.183	-0.379
	PS vs T=1	-0.152	0.207	0.544	0.611	0.403
	PS vs P	-0.277	-0.281	-0.009	0.416	0.722
	conserved hypothetical protein					
	hypothetical proteins-Conserved					
PGN_1011	P vs T=1	-0.253	0.290	0.741	0.511	0.701
	PS vs T=1	0.394	0.432	0.486	0.923	0.784
	PS vs P	0.584	0.136	-0.218	0.404	0.098
	xanthine/uracil/vitamin C permease					
	transport and binding proteins					
PGN_1012	P vs T=1	-0.644	-0.299	0.120	-0.164	0.063
	PS vs T=1	-0.097	-0.229	-0.424	-0.396	-0.319
	PS vs P	0.477	0.056	-0.486	-0.240	-0.354
	putative 4-diphosphocytidyl-2C-methyl-D-erythritol kinase					
	biosynthesis of cofactors, prosthetic groups, and carriers					
PGN_1013	P vs T=1	-0.451	-0.519	-0.437	-0.168	0.891
	PS vs T=1	0.020	-0.113	0.096	0.294	0.073
	PS vs P	0.443	0.365	0.479	0.436	-0.740
	putative Fe-S oxidoreductase					
	hypothetical proteins-Conserved					
PGN_1014	P vs T=1	0.304	0.804	1.375	1.403	0.809
	PS vs T=1	0.836	0.799	0.820	0.981	0.944
	PS vs P	0.487	-0.006	-0.520	-0.390	0.127
	elongation factor G					
	protein synthesis					
PGN_1015	P vs T=1	-0.268	-0.018	0.166	0.232	-0.068
	PS vs T=1	-0.234	-0.195	-0.167	-0.115	-0.111
	PS vs P	0.019	-0.173	-0.319	-0.324	-0.049
	putative DNA polymerase III delta prime subunit					
	DNA metabolism					

P vs T=1 | PS vs T=1 | PS vs P

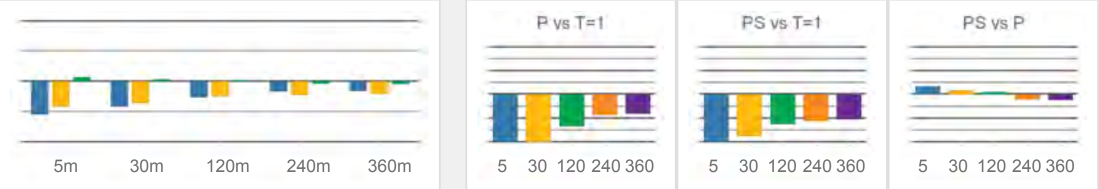

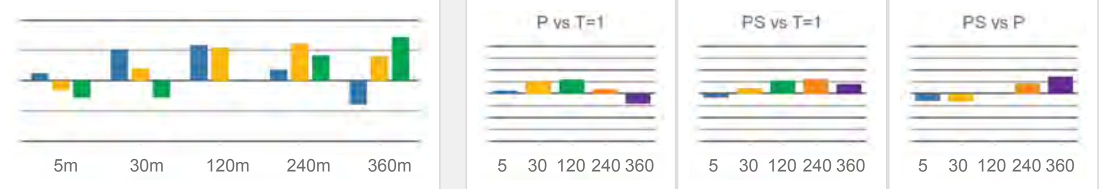

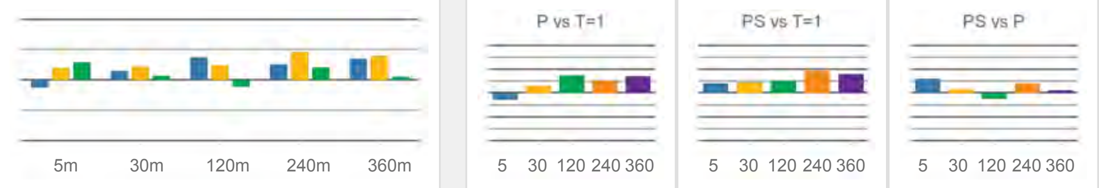

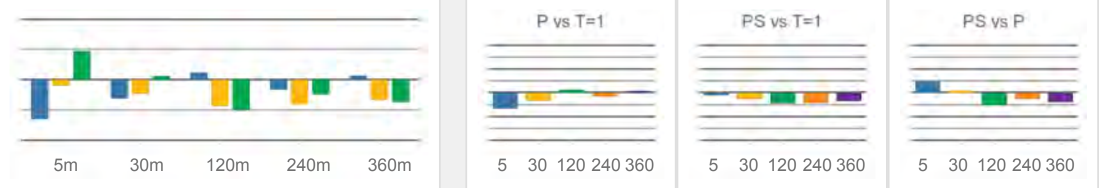

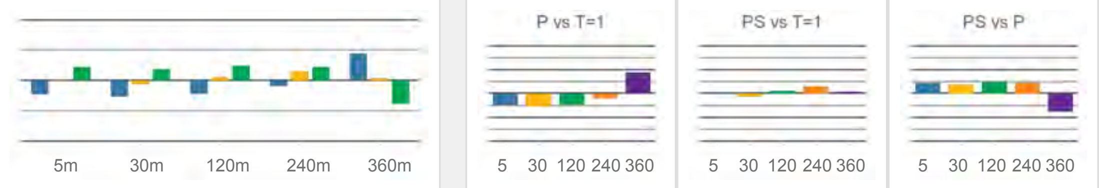

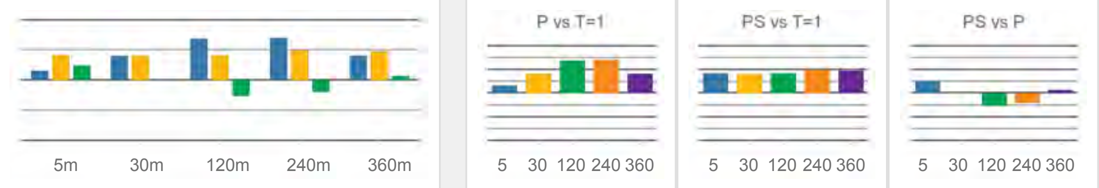

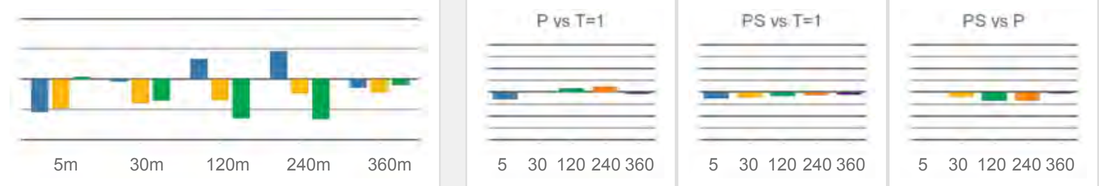

Legend: P vs T=1 | PS vs T=1 | PS vs P

Locus		log$_2$ (Fold Change) 5m	30m	120m	240m	360m
**PGN_1016**	P vs T=1	-0.112	-0.290	-0.504	0.235	0.721
	PS vs T=1	-0.289	-0.532	-0.328	0.273	0.392
	PS vs P	-0.158	-0.244	0.079	0.120	-0.238
	conserved hypothetical protein					
	hypothetical proteins-Conserved					
**PGN_1017**	P vs T=1	0.508	0.696	0.810	1.630	2.506
	PS vs T=1	0.341	0.127	0.896	1.465	1.647
	PS vs P	-0.189	-0.532	0.031	0.033	-0.621
	conserved hypothetical protein					
	hypothetical proteins-Conserved					
**PGN_1018**	P vs T=1	0.068	0.433	0.482	0.682	0.727
	PS vs T=1	-0.077	0.330	1.004	1.546	1.477
	PS vs P	-0.192	-0.111	0.487	0.841	0.735
	conserved hypothetical protein					
	hypothetical proteins-Conserved					
**PGN_1019** *porX*	P vs T=1	1.661	1.604	1.276	1.660	1.706
	PS vs T=1	1.968	2.060	1.826	1.819	1.926
	PS vs P	0.312	0.456	0.528	0.167	0.226
	Por secretion system response regulatr protein porX					
	signal transduction					
**PGN_1020**	P vs T=1	-0.700	-0.917	-1.140	-0.881	-1.055
	PS vs T=1	-0.508	-0.638	-1.131	-0.823	-0.700
	PS vs P	0.194	0.268	-0.032	0.065	0.331
	probable ATP/GTP-binding transmembrane protein					
	unknown function					
**PGN_1021**	P vs T=1	-0.718	-1.051	-1.027	-0.326	-0.104
	PS vs T=1	-0.312	-0.569	-1.352	-0.739	-0.434
	PS vs P	0.395	0.378	-0.454	-0.230	-0.202
	hypothetical protein					
	hypothetical proteins					
**PGN_1022**	P vs T=1	-0.731	-0.849	-0.847	-0.733	-0.428
	PS vs T=1	-0.714	-0.854	-1.025	-0.483	-0.592
	PS vs P	0.014	-0.020	-0.198	0.238	-0.143
	putative thymidine kinase					
	purines, pyrimidines, nucleosides and nucleotides					

PGN_1016

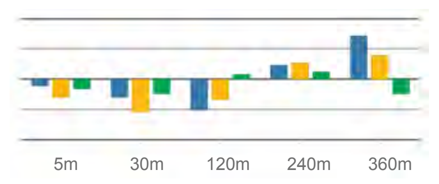

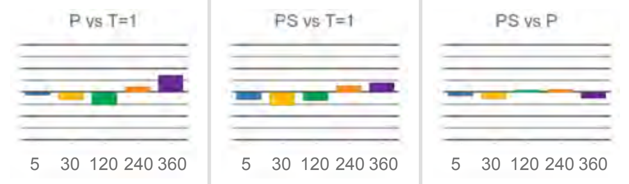

PGN_1017

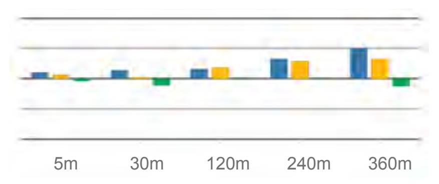

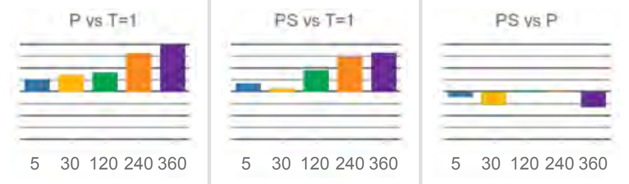

PGN_1018

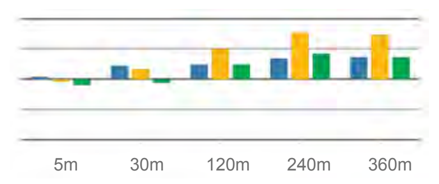

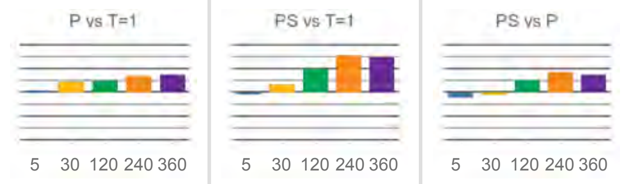

PGN_1019

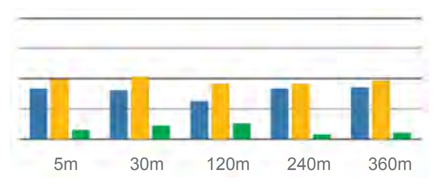

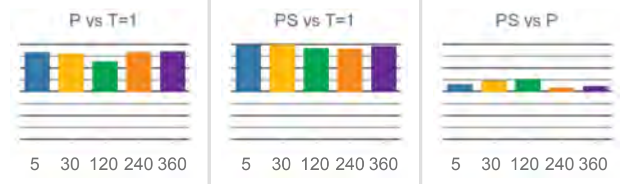

PGN_1020

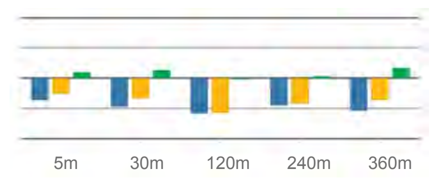

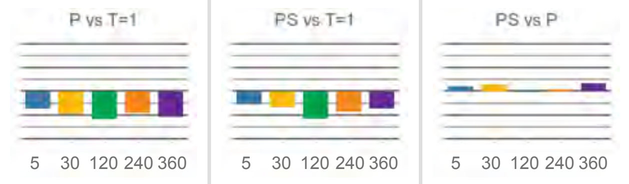

PGN_1021

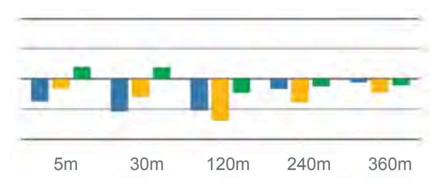

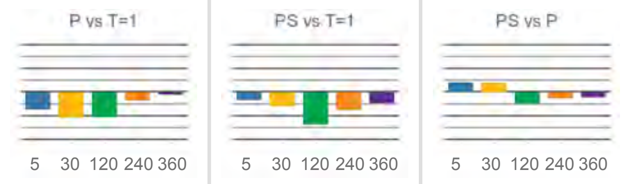

PGN_1022

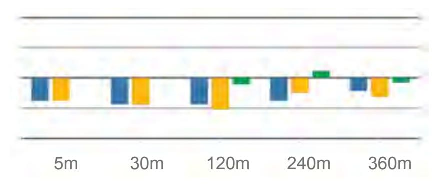

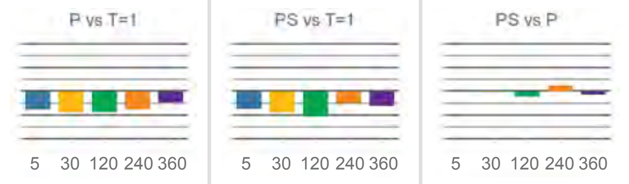

PgSg Time Course

log_2 (Fold Change)

Legend: P vs T=1 | PS vs T=1 | PS vs P

PGN_1023

Locus	5m	30m	120m	240m	360m
P vs T=1	0.663	0.924	1.155	1.067	1.031
PS vs T=1	0.769	0.950	0.918	1.049	0.910
PS vs P	0.090	0.038	-0.198	0.007	-0.103

acid phosphatase OlpA

cell envelope

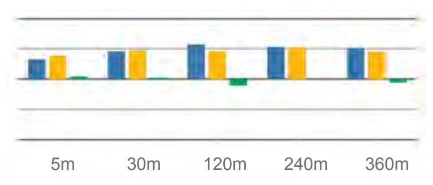

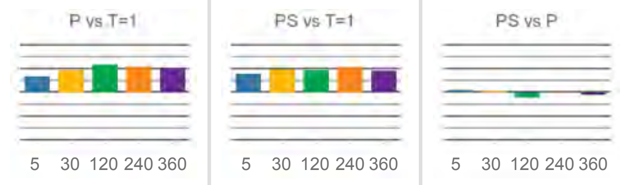

PGN_1024

Locus	5m	30m	120m	240m	360m
P vs T=1	0.269	0.294	-0.105	0.213	0.575
PS vs T=1	0.744	0.504	0.053	0.093	0.018
PS vs P	0.468	0.211	0.076	-0.117	-0.505

putative ribosome-binding factor A

transcription

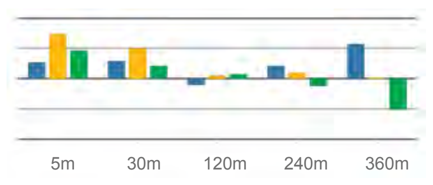

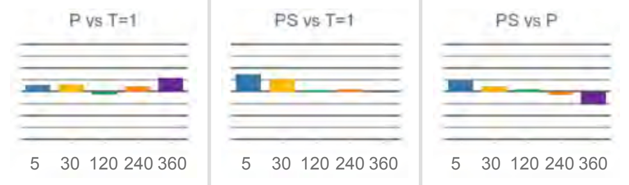

PGN_1025

Locus	5m	30m	120m	240m	360m
P vs T=1	0.139	-0.175	-0.274	-0.065	0.247
PS vs T=1	0.547	0.023	-0.592	-0.613	-0.561
PS vs P	0.419	0.187	-0.332	-0.521	-0.766

conserved hypothetical protein

hypothetical proteins-Conserved

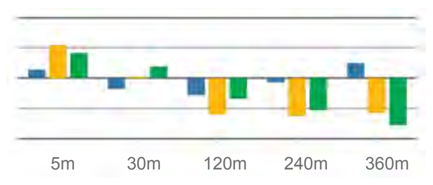

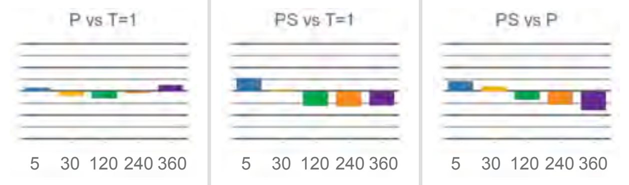

PGN_1026

Locus	5m	30m	120m	240m	360m
P vs T=1	-0.359	-0.919	-1.244	-1.260	-1.191
PS vs T=1	-0.051	-0.542	-0.430	-0.191	-0.366
PS vs P	0.317	0.337	0.734	0.968	0.769

glycosyl transferase family 2

cell envelope

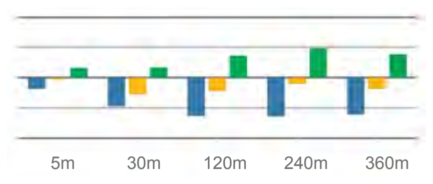

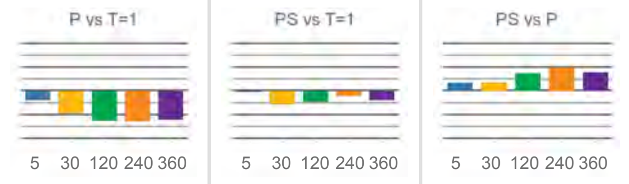

PGN_1027

Locus	5m	30m	120m	240m	360m
P vs T=1	-0.020	-0.221	-0.258	-0.060	-0.036
PS vs T=1	0.757	0.113	0.106	0.345	0.308
PS vs P	0.763	0.307	0.329	0.391	0.334

dihydroorotase

purines, pyrimidines, nucleosides and nucleotides

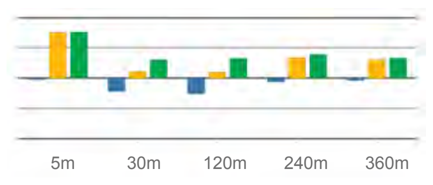

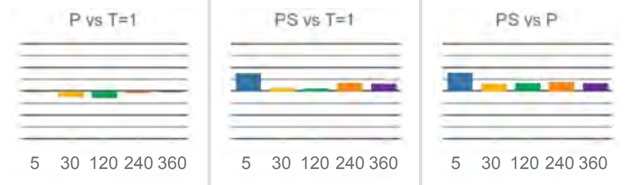

PGN_1028

Locus	5m	30m	120m	240m	360m
P vs T=1	-0.267	-0.637	-1.100	-1.204	-1.033
PS vs T=1	0.021	-0.260	-0.192	-0.120	-0.397
PS vs P	0.297	0.354	0.819	0.948	0.581

conserved hypothetical protein

hypothetical proteins-Conserved

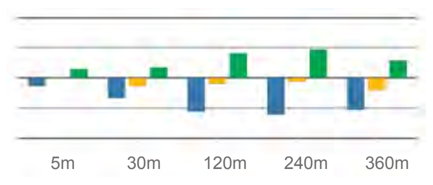

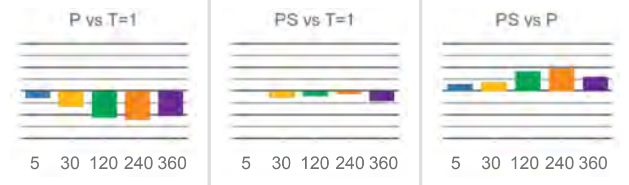

PGN_1029

Locus	5m	30m	120m	240m	360m
P vs T=1	-0.207	-0.726	-0.996	-1.402	-0.886
PS vs T=1	-0.090	-0.686	-0.391	-0.265	-0.575
PS vs P	0.164	0.014	0.536	0.870	0.281

conserved hypothetical protein

hypothetical proteins-Conserved

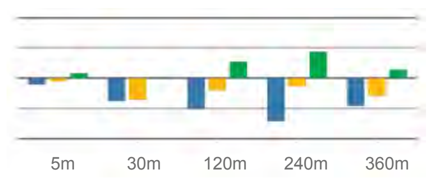

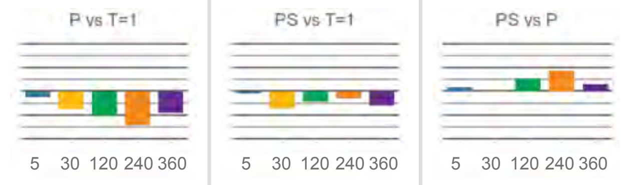

PgSg Time Course

log$_2$ (Fold Change)

Legend: P vs T=1 | PS vs T=1 | PS vs P

Locus		5m	30m	120m	240m	360m
**PGN_1030**	P vs T=1	-0.031	-0.131	0.574	0.817	0.426
	PS vs T=1	-0.855	-0.855	-0.658	-0.550	-0.625
	PS vs P	-0.727	-0.667	-0.977	-0.983	-0.867

hypothetical protein

hypothetical proteins

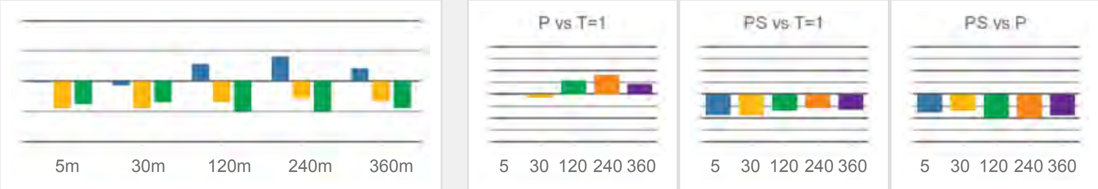

Locus		5m	30m	120m	240m	360m
**PGN_1031**	P vs T=1	0.623	-0.755	-0.302	0.536	2.532
	PS vs T=1	-0.362	-1.265	0.644	1.062	1.431
	PS vs P	-0.653	-0.831	0.520	0.505	-0.414

conserved hypothetical protein

hypothetical proteins-Conserved

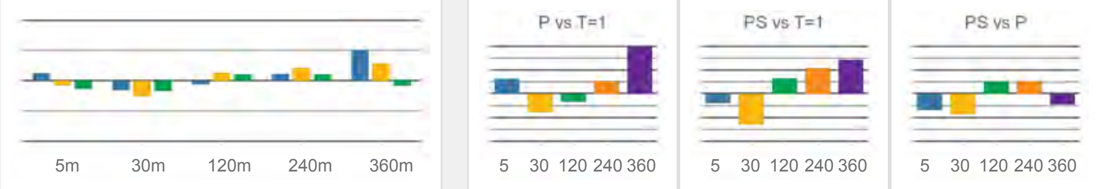

Locus		5m	30m	120m	240m	360m
**PGN_1032**	P vs T=1	-1.346	-1.104	-0.812	-0.863	-1.313
	PS vs T=1	-0.508	0.190	0.787	0.633	0.196
	PS vs P	0.604	1.098	1.482	1.364	1.346

conserved hypothetical protein

hypothetical proteins-Conserved

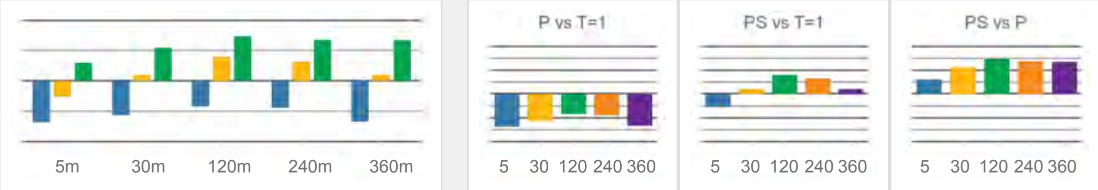

Locus		5m	30m	120m	240m	360m
**PGN_1033**	P vs T=1	-0.538	-2.107	-2.638	-2.415	-1.361
wzx	PS vs T=1	-0.664	-1.293	-1.510	-1.478	-1.822
	PS vs P	0.028	0.638	0.857	0.723	-0.416

O-antigen flippase

transport and binding proteins

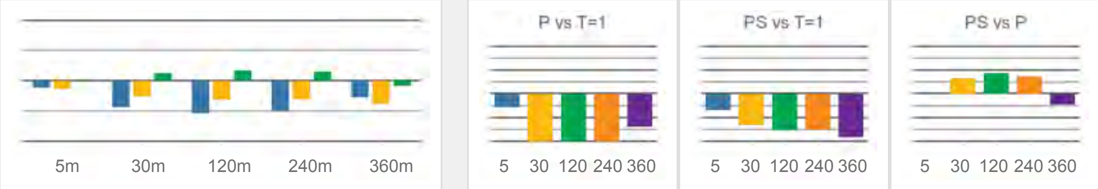

Locus		5m	30m	120m	240m	360m
**PGN_1034**	P vs T=1	-0.486	-1.223	-1.560	-1.544	-1.210
	PS vs T=1	-0.500	-0.579	-0.410	-0.267	-0.713
	PS vs P	0.022	0.577	1.022	1.124	0.458

conserved hypothetical protein

unknown function

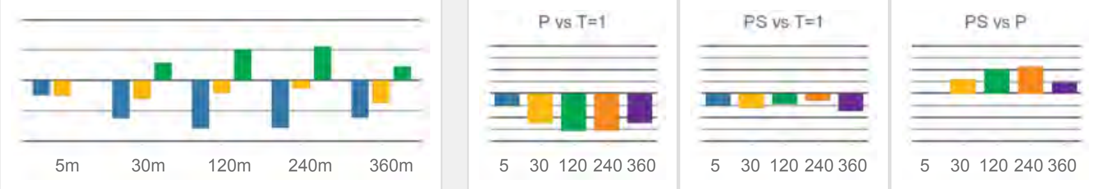

Locus		5m	30m	120m	240m	360m
**PGN_1035**	P vs T=1	0.245	-0.230	-0.670	-0.907	-1.004
	PS vs T=1	0.393	0.116	-0.164	-0.372	-0.549
	PS vs P	0.170	0.342	0.482	0.484	0.422

conserved hypothetical protein with DUF1063 domain

hypothetical proteins-Conserved

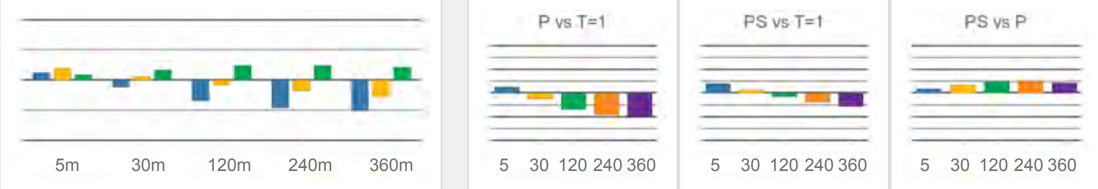

Locus		5m	30m	120m	240m	360m
**PGN_1036**	P vs T=1	0.409	0.298	0.216	0.385	0.067
	PS vs T=1	0.231	-0.018	-0.122	-0.301	-0.309
	PS vs P	-0.154	-0.297	-0.325	-0.631	-0.371

putative G/U mismatch-specific DNA glycosylase

DNA metabolism

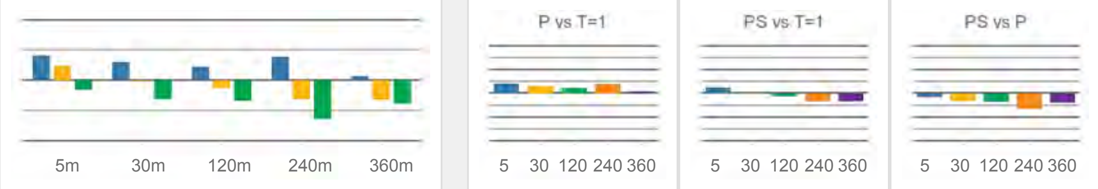

PgSg Time Course

log$_2$ (Fold Change)

Legend: P vs T=1 | PS vs T=1 | PS vs P

Locus		5m	30m	120m	240m	360m
**PGN_1037**	P vs T=1	0.064	0.613	0.973	0.627	-0.124
	PS vs T=1	-0.656	-0.048	0.079	-0.629	-0.745
	PS vs P	-0.713	-0.607	-0.821	-1.207	-0.625

conserved hypothetical protein

cell envelope

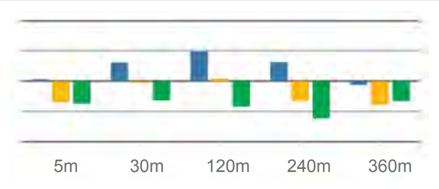

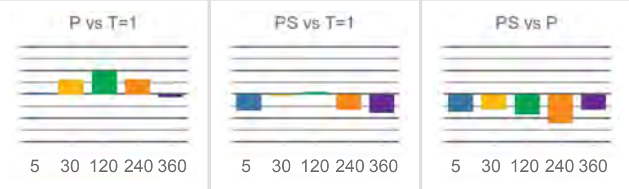

Locus		5m	30m	120m	240m	360m
**PGN_1038**	P vs T=1	-0.316	-0.441	-0.545	-0.662	-1.364
	PS vs T=1	-0.985	-0.713	-0.148	0.229	0.107
	PS vs P	-0.650	-0.266	0.389	0.871	1.416

conserved hypothetical protein

hypothetical proteins-Conserved

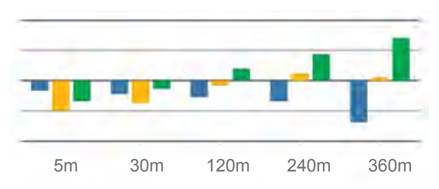

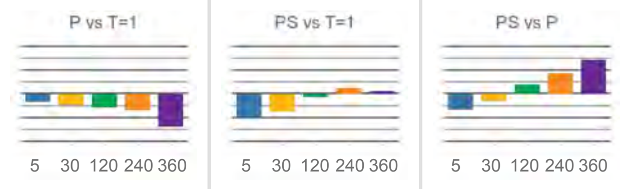

Locus		5m	30m	120m	240m	360m
**PGN_1039**	P vs T=1	-0.566	-0.860	-1.001	-0.367	0.711
	PS vs T=1	-0.455	-0.752	-1.144	-0.933	-0.716
	PS vs P	0.119	0.069	-0.241	-0.493	-1.268

putative alpha-1,2-mannosidase precursor

cell envelope

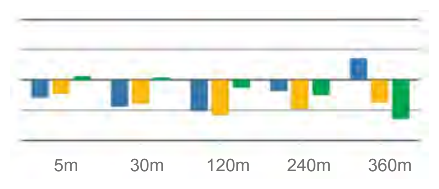

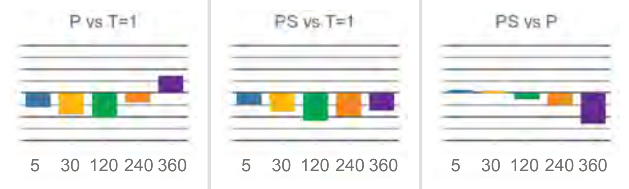

Locus		5m	30m	120m	240m	360m
**PGN_1040**	P vs T=1	2.728	2.160	1.231	0.739	-0.225
	PS vs T=1	2.448	2.282	1.315	0.950	0.399
	PS vs P	-0.173	0.172	0.065	0.141	0.458

conserved hypothetical protein

hypothetical proteins-Conserved

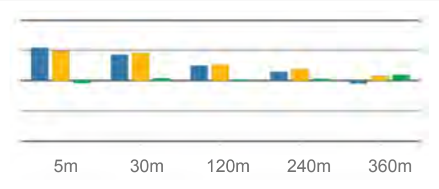

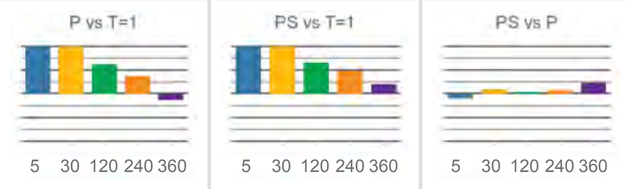

Locus		5m	30m	120m	240m	360m
**PGN_1041**	P vs T=1	3.287	3.488	2.901	2.203	1.264
	PS vs T=1	3.722	3.883	3.090	2.228	1.667
	PS vs P	0.489	0.484	0.223	-0.019	0.291

cytochrome d ubiquinol oxidase subunit I

energy metabolism

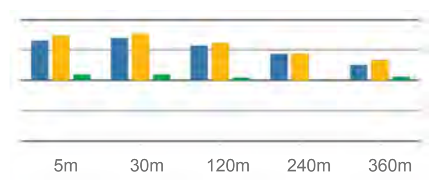

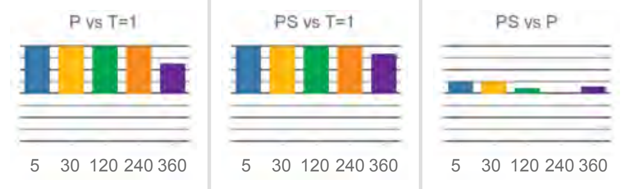

Locus		5m	30m	120m	240m	360m
**PGN_1042**	P vs T=1	1.596	2.300	2.484	2.142	1.366
	PS vs T=1	1.874	2.753	2.630	2.074	2.065
	PS vs P	0.193	0.496	0.220	-0.049	0.620

cytochrome d ubiquinol oxidase subunit II

energy metabolism

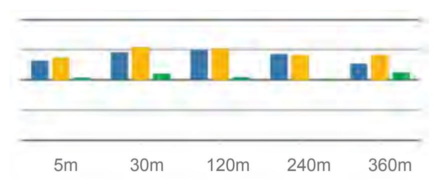

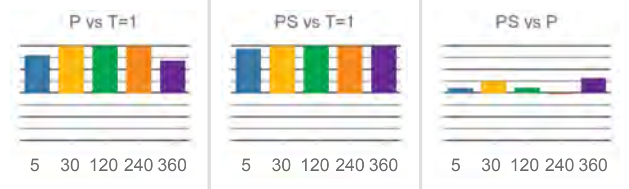

Locus		5m	30m	120m	240m	360m
**PGN_1043**	P vs T=1	0.816	0.810	0.622	0.624	0.297
	PS vs T=1	0.601	1.006	2.021	2.362	1.994
	PS vs P	-0.222	0.182	1.350	1.680	1.636

conserved hypothetical protein

hypothetical proteins-Conserved

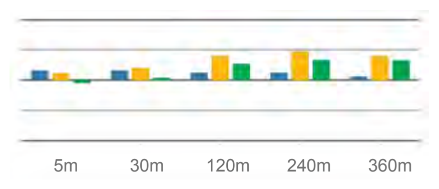

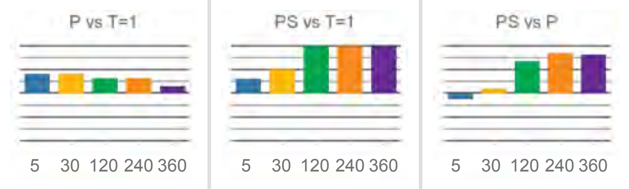

PgSg Time Course

Hackett Laboratory UW

log$_2$ (Fold Change)

Legend: P vs T=1 | PS vs T=1 | PS vs P

Locus		5m	30m	120m	240m	360m
**PGN_1044**	P vs T=1	0.354	0.271	0.019	0.154	0.509
	PS vs T=1	1.017	0.582	1.343	1.482	1.134
	PS vs P	0.643	0.287	1.257	1.262	0.614
	alpha-amylase					
	energy metabolism					

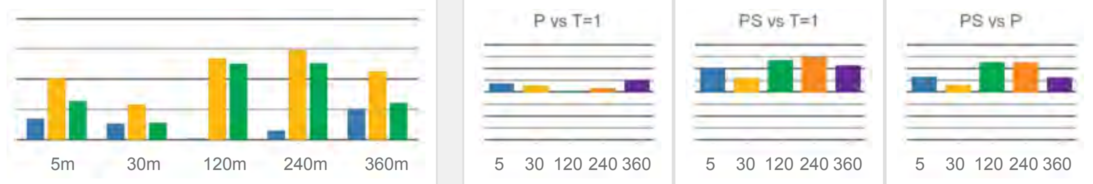

Locus		5m	30m	120m	240m	360m
**PGN_1045**	P vs T=1	-0.542	-0.732	-0.918	-0.150	1.095
lacZII	PS vs T=1	-0.360	-0.275	0.412	0.728	0.495
	PS vs P	0.157	0.401	1.178	0.855	-0.530
	beta-galactosidase					
	energy metabolism					

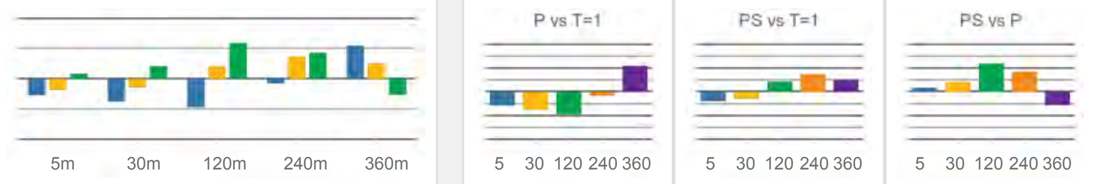

Locus		5m	30m	120m	240m	360m
**PGN_1046**	P vs T=1	0.687	0.520	0.357	1.023	2.044
	PS vs T=1	0.529	0.274	0.858	1.046	1.115
	PS vs P	-0.134	-0.254	0.334	0.100	-0.703
	putative DNA repair protein					
	DNA metabolism					

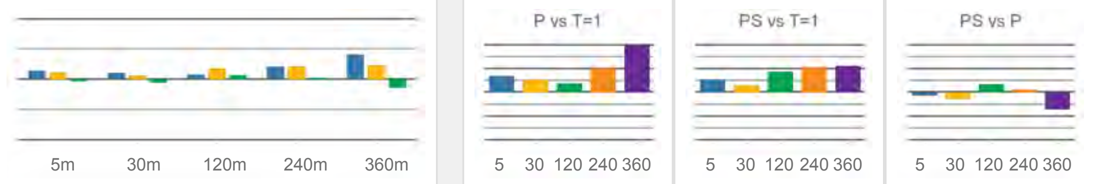

Locus		5m	30m	120m	240m	360m
**PGN_1047**	P vs T=1	2.406	2.663	2.260	1.642	2.118
	PS vs T=1	-0.225	-0.295	0.080	0.536	0.554
	PS vs P	-2.496	-2.775	-2.054	-1.035	-1.461
	hydroxylamine reductase					
	unknown function					

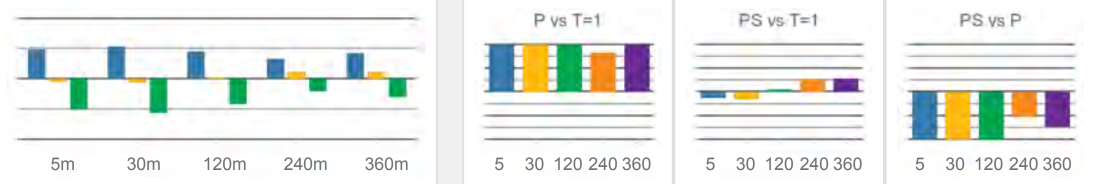

Locus		5m	30m	120m	240m	360m
**PGN_1048**	P vs T=1	0.108	0.147	0.473	0.741	0.366
	PS vs T=1	0.033	-0.242	0.477	0.265	0.030
	PS vs P	-0.104	-0.386	0.068	-0.212	-0.298
	hypothetical protein					
	hypothetical proteins					

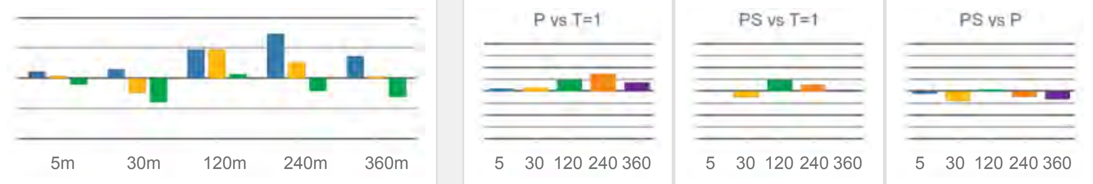

Locus		5m	30m	120m	240m	360m
**PGN_1049**	P vs T=1	-0.161	0.306	0.258	-0.304	-0.668
	PS vs T=1	-0.111	0.376	0.829	0.244	-0.200
	PS vs P	0.034	0.088	0.586	0.525	0.441
	putative alkaline phosphatase					
	biosynthesis of cofactors, prosthetic groups, and carriers/ central [illegible]					

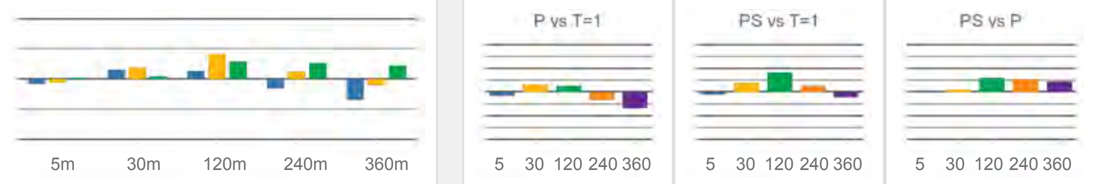

Locus		5m	30m	120m	240m	360m
**PGN_1050**	P vs T=1	1.100	1.207	0.953	0.596	0.320
	PS vs T=1	1.167	1.743	2.064	1.470	0.930
	PS vs P	0.070	0.542	1.106	0.842	0.582
	conserved hypothetical protein					
	protein fate					

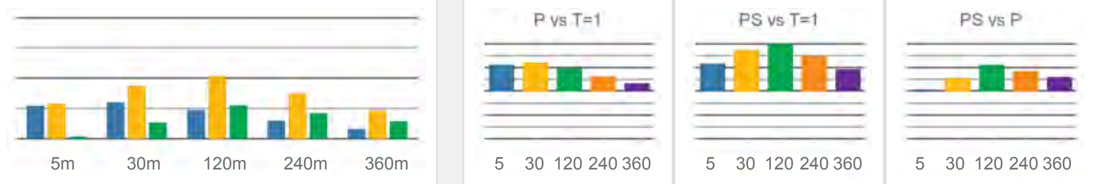

PgSg Time Course

log$_2$ (Fold Change)

P vs T=1 | PS vs T=1 | PS vs P

Locus		5m	30m	120m	240m	360m
**PGN_1051**	P vs T=1	-0.488	-0.767	-1.646	-1.679	-1.603
	PS vs T=1	-0.401	-0.108	-0.145	-0.827	-1.308
	PS vs P	0.133	0.650	1.254	0.606	0.161
	hypothetical protein					
	hypothetical proteins					
**PGN_1052**	P vs T=1	0.135	0.128	-0.230	-0.638	-1.274
	PS vs T=1	0.460	0.975	1.196	0.390	-0.064
	PS vs P	0.326	0.844	1.405	0.964	1.116
	conserved hypothetical protein					
	hypothetical proteins-Conserved					
**PGN_1053**	P vs T=1	0.003	0.066	-0.377	-0.449	0.095
	PS vs T=1	1.075	1.080	0.003	-0.619	-0.505
	PS vs P	1.058	1.006	0.337	-0.227	-0.568
	putative phospho-2-dehydro-3-deoxyheptonate aldolase/chorismate mutase					
	amino acid biosynthesis					
**PGN_1054** *vimF*	P vs T=1	-0.470	-0.397	-0.819	-1.138	-0.593
	PS vs T=1	0.478	0.126	-0.891	-1.337	-1.147
	PS vs P	0.938	0.526	-0.089	-0.275	-0.537
	virulence modulating gene F					
	hypothetical proteins					
**PGN_1055** *vimE*	P vs T=1	0.056	0.106	-0.630	-0.867	-0.664
	PS vs T=1	0.609	0.088	-1.033	-1.394	-1.127
	PS vs P	0.560	-0.000	-0.415	-0.560	-0.463
	virulence modulating gene E					
	hypothetical proteins					
**PGN_1056** *vimA*	P vs T=1	-0.120	0.174	0.311	-0.082	-0.614
	PS vs T=1	-0.148	-0.446	-1.107	-1.425	-1.491
	PS vs P	-0.022	-0.580	-1.359	-1.307	-0.883
	virulence modulating gene A					
	hypothetical proteins					
**PGN_1057** *recA*	P vs T=1	-0.052	0.397	0.648	0.211	-0.468
	PS vs T=1	0.282	0.417	0.015	-0.376	-0.725
	PS vs P	0.310	0.039	-0.587	-0.574	-0.281
	recombinase A					
	DNA metabolism					

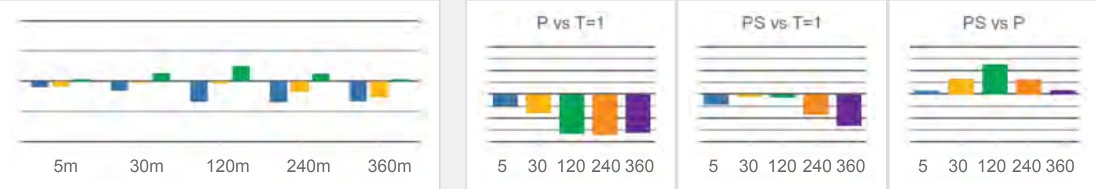

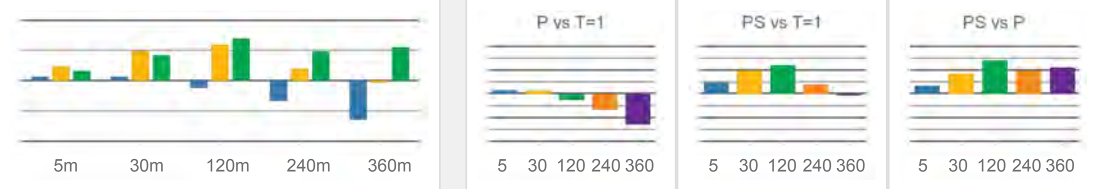

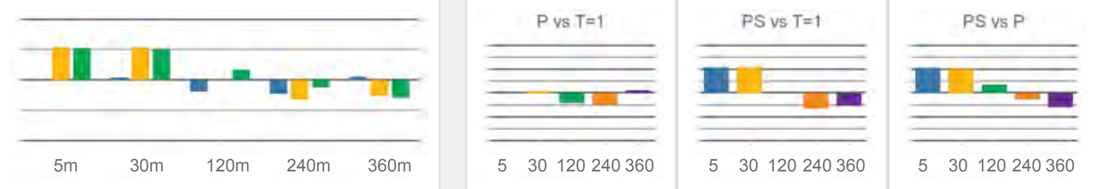

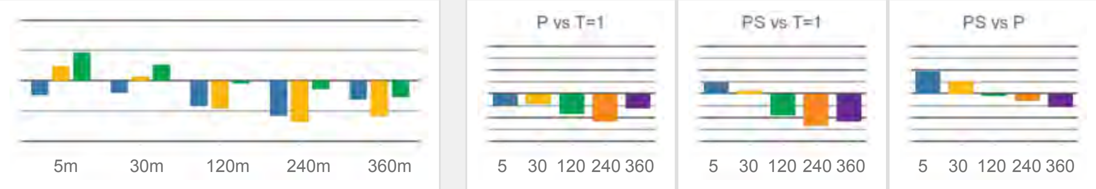

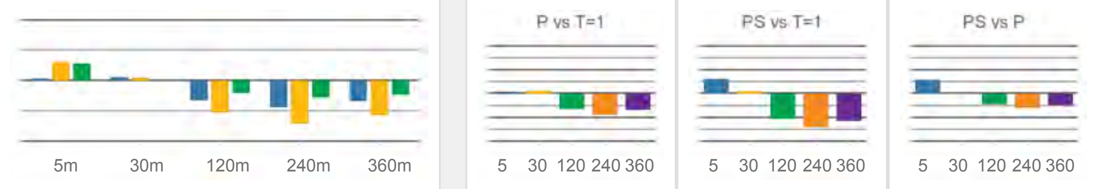

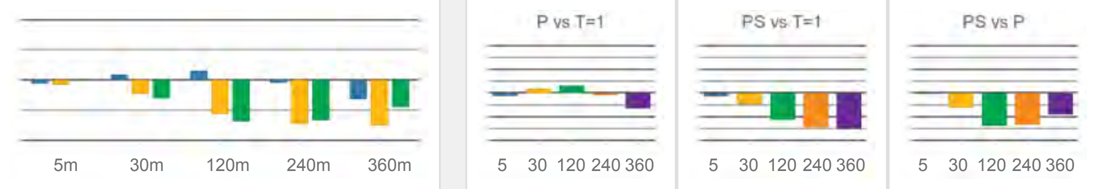

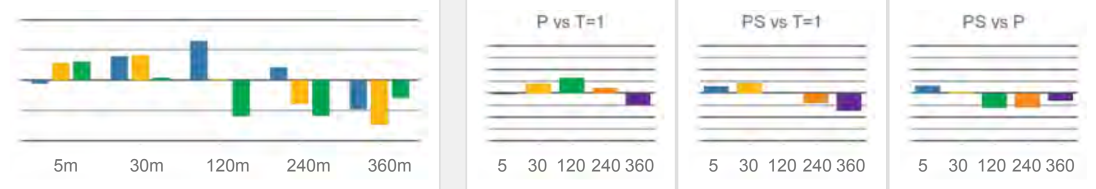

log$_2$ (Fold Change)

Locus		5m	30m	120m	240m	360m
**PGN_1058**	P vs T=1	0.882	0.834	0.999	0.731	-0.066
bcp	PS vs T=1	1.188	1.190	1.436	1.716	1.539
	PS vs P	0.299	0.337	0.446	0.953	1.511

putative bacterioferritin comigratory protein

unknown function

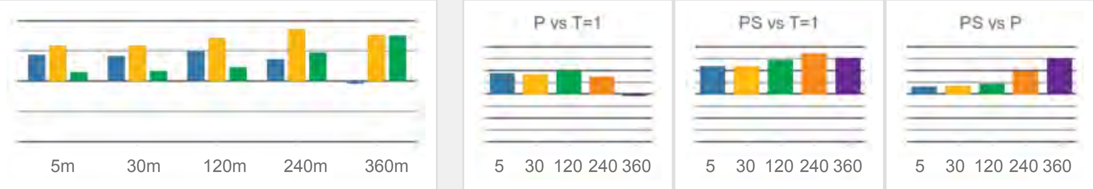

Locus		5m	30m	120m	240m	360m
**PGN_1059**	P vs T=1	0.350	0.115	0.939	1.472	2.884
	PS vs T=1	0.146	0.392	1.009	1.406	1.902
	PS vs P	-0.269	-0.033	0.098	0.170	-0.317

conserved hypothetical protein

hypothetical proteins-Conserved

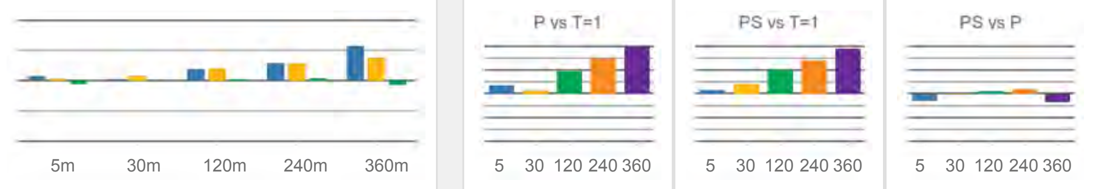

Locus		5m	30m	120m	240m	360m
**PGN_1060**	P vs T=1	0.424	-0.342	-0.232	0.670	1.669
	PS vs T=1	0.012	0.125	0.479	0.448	0.841
	PS vs P	-0.379	0.345	0.529	-0.163	-0.705

transposase in ISPg3

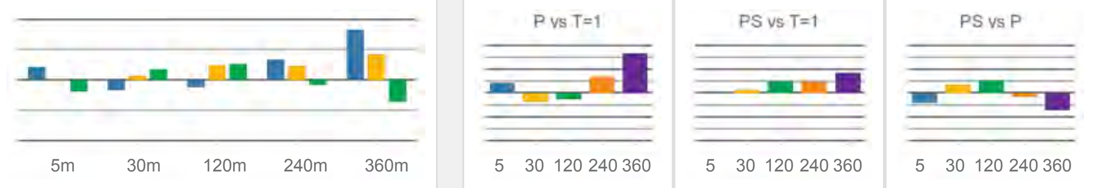

Locus		5m	30m	120m	240m	360m
**PGN_1061**	P vs T=1	0.011	-0.256	-0.298	0.084	0.311
	PS vs T=1	-0.224	-0.759	-0.461	-0.515	-0.449
	PS vs P	-0.212	-0.498	-0.184	-0.530	-0.701

conserved hypothetical protein

hypothetical proteins-Conserved

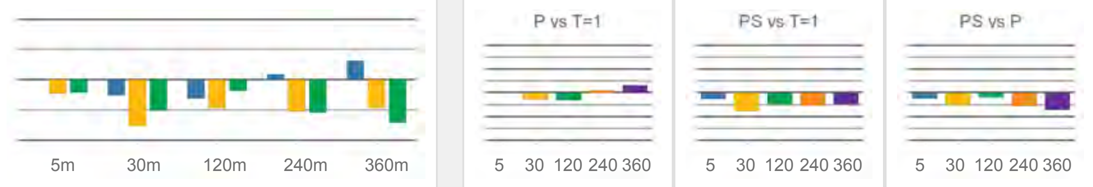

Locus		5m	30m	120m	240m	360m
**PGN_1062**	P vs T=1	-0.672	-0.726	-0.743	-0.242	0.135
	PS vs T=1	-0.574	-0.841	-0.934	-0.551	-0.305
	PS vs P	0.085	-0.132	-0.236	-0.249	-0.375

tRNA modification GTPase

cellular processes

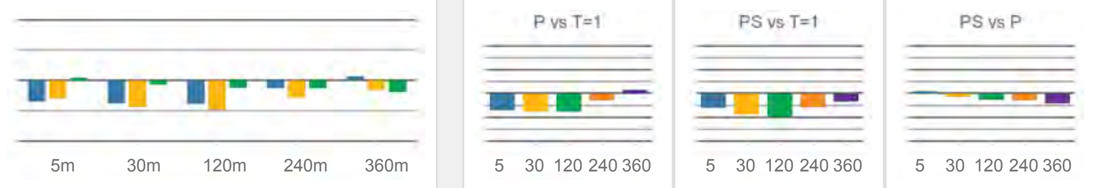

Locus		5m	30m	120m	240m	360m
**PGN_1063**	P vs T=1	0.440	0.119	0.686	1.458	2.267
	PS vs T=1	0.448	0.375	0.941	1.285	1.548
	PS vs P	-0.045	-0.027	0.136	0.123	-0.232

partial transposase Orf2 in ISPg5

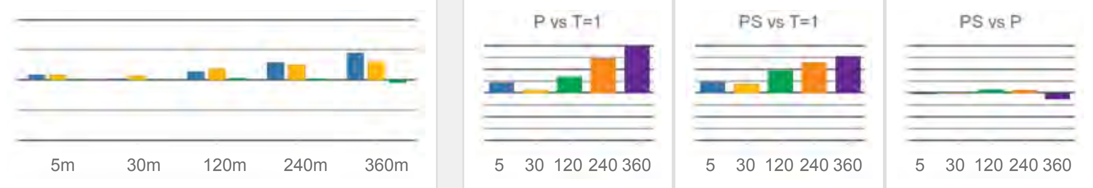

Locus		5m	30m	120m	240m	360m
**PGN_1064**	P vs T=1	0.484	0.117	0.299	0.820	1.658
	PS vs T=1	0.064	0.224	0.591	0.823	1.200
	PS vs P	-0.398	0.034	0.209	0.035	-0.341

transposase in ISPg3

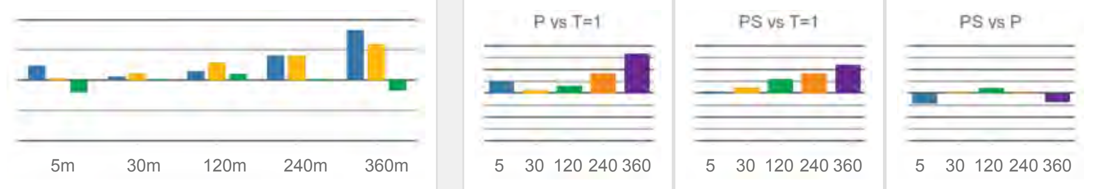

PgSg Time Course

log$_2$ (Fold Change)

Locus		5m	30m	120m	240m	360m
**PGN_1065**	P vs T=1	0.368	-0.323	-0.214	0.254	1.566
	PS vs T=1	-0.112	-0.013	0.428	0.497	0.673
	PS vs P	-0.424	0.168	0.477	0.192	-0.717
	transposase in ISPg3					
**PGN_1066**	P vs T=1	0.286	-0.311	-0.185	0.398	1.603
	PS vs T=1	-0.119	-0.028	0.358	0.552	0.892
	PS vs P	-0.375	0.161	0.387	0.139	-0.566
	transposase in ISPg3					
**PGN_1067**	P vs T=1	-1.095	-2.044	-2.018	-1.460	-0.114
	PS vs T=1	-0.666	-0.397	-0.074	0.042	-0.156
	PS vs P	0.349	1.086	1.409	1.193	0.126
	hypothetical protein					
	hypothetical proteins					
**PGN_1068**	P vs T=1	-1.134	-2.145	-1.925	-1.573	0.429
	PS vs T=1	-0.390	-0.643	-0.462	-0.271	-0.715
	PS vs P	0.654	0.913	1.002	0.917	-0.789
	conserved hypothetical protein					
	hypothetical proteins-Conserved					
**PGN_1069**	P vs T=1	-0.713	-1.705	-1.286	-0.645	0.998
	PS vs T=1	-0.079	-0.161	-0.316	-0.472	-0.467
	PS vs P	0.571	0.908	0.627	0.134	-0.968
	hypothetical protein					
	hypothetical proteins					
**PGN_1070**	P vs T=1	-1.065	-2.297	-1.778	-1.550	0.452
	PS vs T=1	-0.420	-0.952	-1.060	-0.956	-0.822
	PS vs P	0.649	0.740	0.459	0.343	-0.840
	hypothetical protein					
	hypothetical proteins					
**PGN_1071**	P vs T=1	-1.231	-2.656	-2.137	-1.489	0.055
	PS vs T=1	-0.587	-1.251	-0.981	-1.512	-1.191
	PS vs P	0.672	0.753	0.802	-0.073	-0.864
	hypothetical protein					
	hypothetical proteins					

P vs T=1 | PS vs T=1 | PS vs P

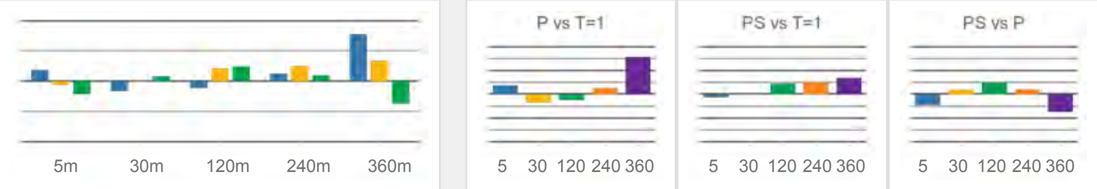

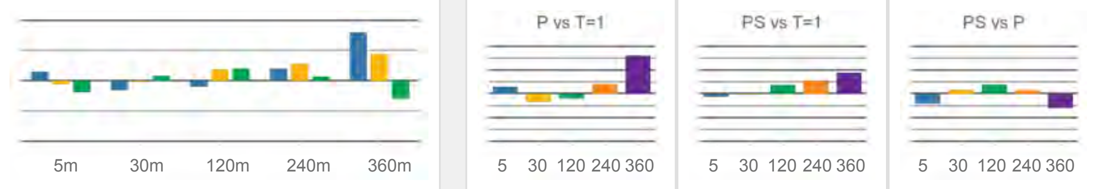

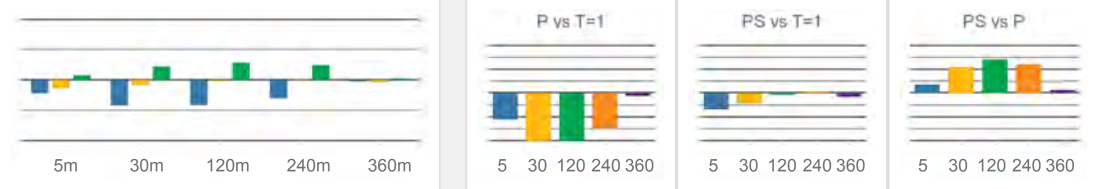

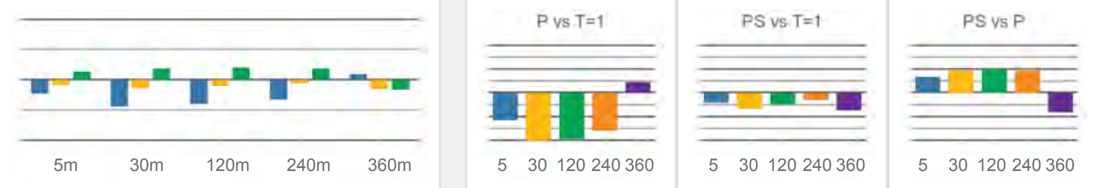

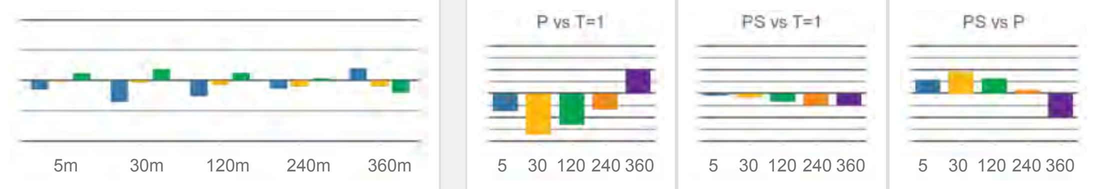

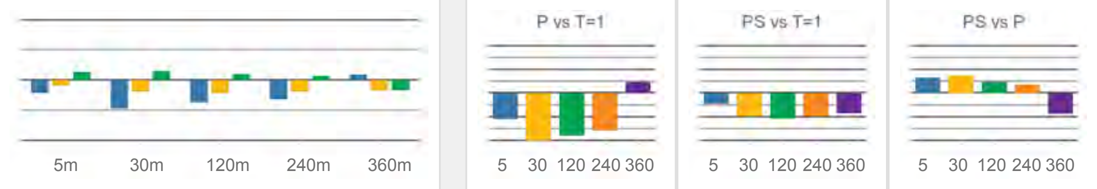

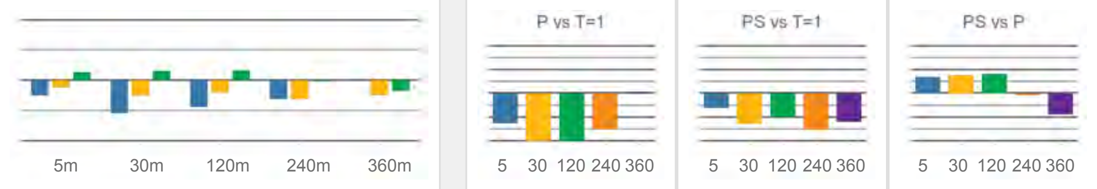

PgSg Time Course

log$_2$ (Fold Change)

Legend: P vs T=1 | PS vs T=1 | PS vs P

Locus		5m	30m	120m	240m	360m
**PGN_1072**	P vs T=1	-1.016	-2.385	-2.244	-1.478	0.286
	PS vs T=1	-0.539	-0.880	-0.730	-0.578	-0.640
	PS vs P	0.488	0.693	0.821	0.622	-0.459

hypothetical protein

hypothetical proteins

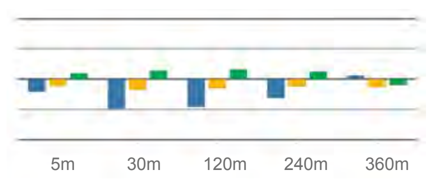

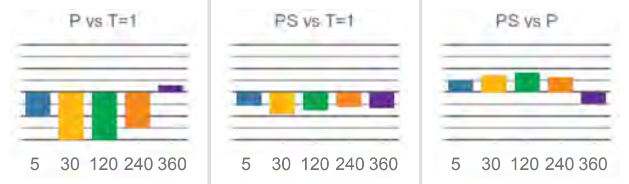

Locus		5m	30m	120m	240m	360m
**PGN_1073**	P vs T=1	-1.412	-3.150	-2.383	-1.501	0.307
	PS vs T=1	-0.910	-1.582	-1.187	-0.784	-0.432
	PS vs P	0.518	0.757	0.738	0.605	-0.433

hypothetical protein

hypothetical proteins

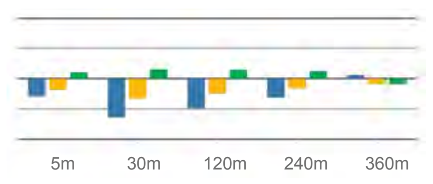

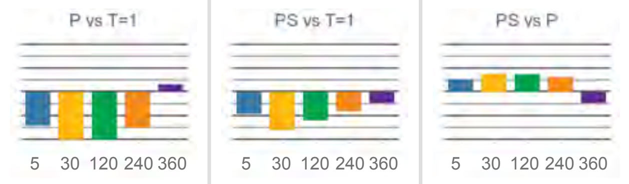

Locus		5m	30m	120m	240m	360m
**PGN_1074**	P vs T=1	-0.912	-1.686	-1.446	-1.084	0.202
	PS vs T=1	-0.674	-0.824	0.126	0.420	0.154
	PS vs P	0.190	0.534	1.301	1.299	0.073

hypothetical protein

hypothetical proteins

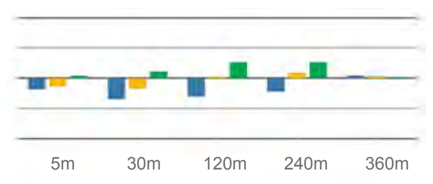

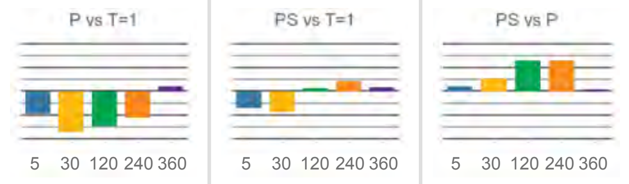

Locus		5m	30m	120m	240m	360m
**PGN_1075**	P vs T=1	-0.171	-0.164	0.380	0.054	1.033
	PS vs T=1	-0.215	0.006	0.368	0.270	1.143
	PS vs P	-0.112	0.060	0.094	0.021	0.333

hypothetical protein

hypothetical proteins

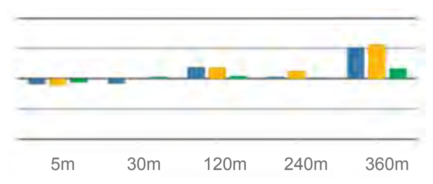

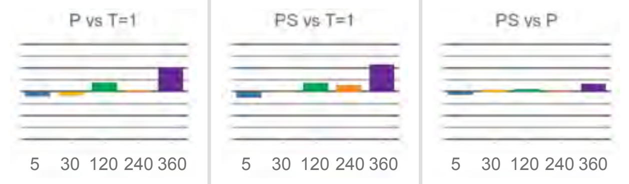

Locus		5m	30m	120m	240m	360m
**PGN_1076**	P vs T=1	0.447	0.169	0.276	0.680	1.219
	PS vs T=1	0.092	-0.064	-0.037	0.219	0.431
	PS vs P	-0.335	-0.250	-0.332	-0.428	-0.731

putative DNA methylase

DNA metabolism

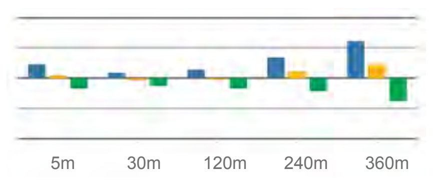

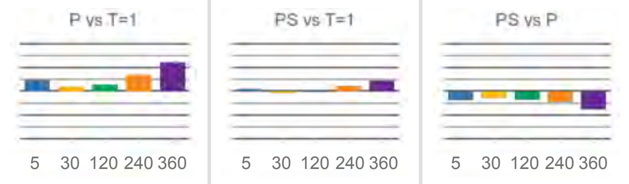

Locus		5m	30m	120m	240m	360m
**PGN_1077**	P vs T=1	0.468	0.417	0.446	0.546	0.260
	PS vs T=1	0.235	0.067	0.181	0.325	0.334
	PS vs P	-0.231	-0.351	-0.270	-0.219	0.059

transposase in ISPg1

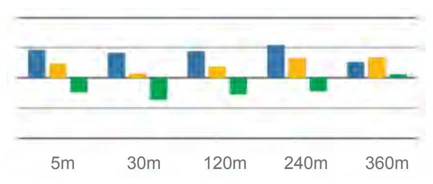

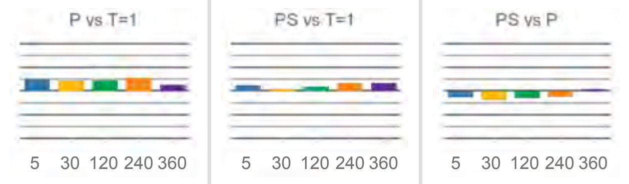

Locus		5m	30m	120m	240m	360m
**PGN_1078**	P vs T=1	0.647	0.762	0.074	-0.401	-0.393
	PS vs T=1	0.905	0.633	0.452	0.725	0.146
	PS vs P	0.277	-0.083	0.354	1.019	0.488

GDP-mannose 4,6-dehydratase

cell envelope

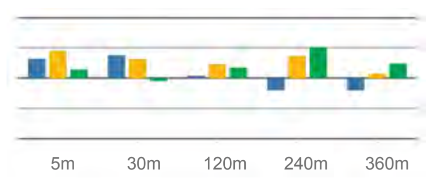

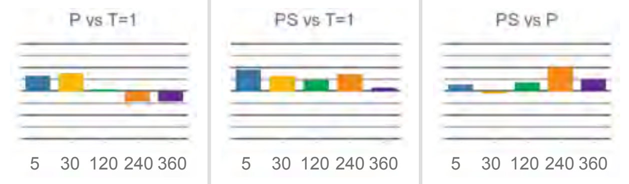

log$_2$ (Fold Change)

Legend: P vs T=1 | PS vs T=1 | PS vs P

Locus		5m	30m	120m	240m	360m
**PGN_1079**	P vs T=1	0.599	0.621	0.456	0.445	0.212
	PS vs T=1	0.808	0.653	0.346	0.546	0.510
	PS vs P	0.214	0.043	-0.113	0.100	0.276
	GDP-fucose synthetase					
	cell envelope					

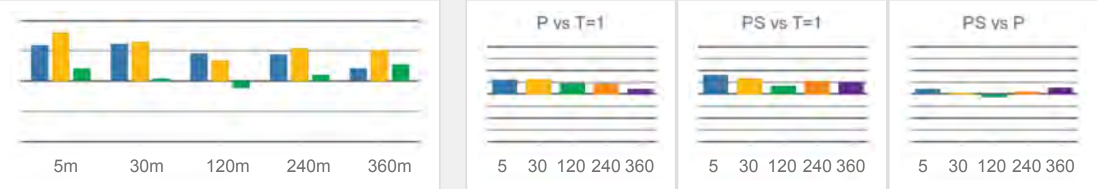

Locus		5m	30m	120m	240m	360m
**PGN_1080**	P vs T=1	1.809	1.794	1.406	0.736	-0.271
	PS vs T=1	0.785	0.935	0.117	-0.380	-0.514
	PS vs P	-0.928	-0.764	-1.218	-1.087	-0.270
	branched-chain amino acid aminotransferase					
	amino acid biosynthesis					

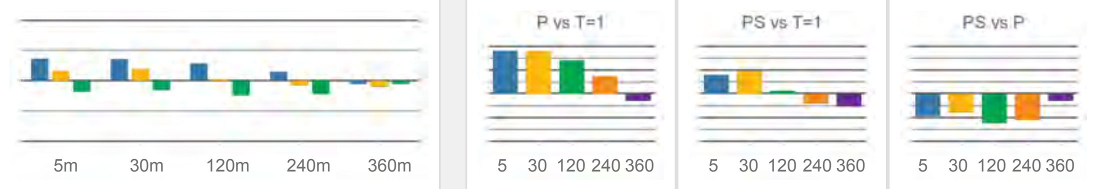

Locus		5m	30m	120m	240m	360m
**PGN_1081**	P vs T=1	0.248	-0.036	-0.359	-0.681	-1.226
	PS vs T=1	0.285	0.073	-0.008	0.085	0.066
	PS vs P	0.049	0.105	0.335	0.729	1.242
	conserved hypothetical protein					
	hypothetical proteins-Conserved					

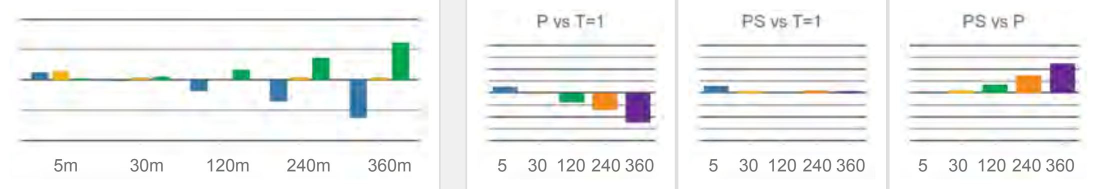

Locus		5m	30m	120m	240m	360m
**PGN_1082**	P vs T=1	0.091	-0.159	0.552	1.637	1.501
	PS vs T=1	0.128	-0.016	0.393	0.874	1.447
	PS vs P	-0.060	-0.149	-0.170	-0.084	0.283
	hypothetical protein					
	hypothetical proteins					

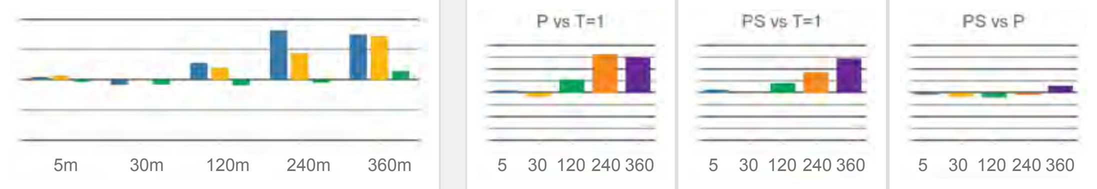

Locus		5m	30m	120m	240m	360m
**PGN_1083**	P vs T=1	-0.526	-0.307	0.784	1.552	2.017
	PS vs T=1	0.012	-0.401	0.343	1.115	0.699
	PS vs P	0.043	-0.356	-0.164	0.234	-0.394
	hypothetical protein					
	hypothetical proteins					

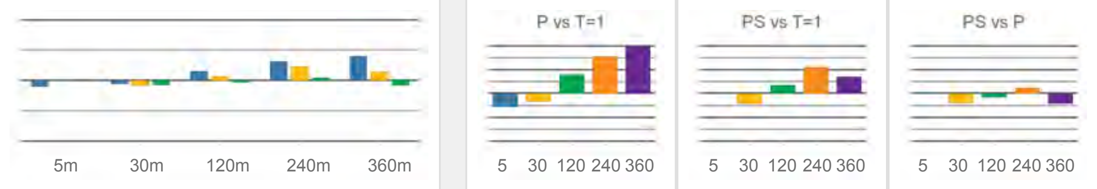

Locus		5m	30m	120m	240m	360m
**PGN_1084**	P vs T=1	-0.163	-0.166	-0.195	0.147	0.589
	PS vs T=1	0.102	0.004	-0.072	-0.012	0.470
	PS vs P	0.211	0.112	0.003	-0.084	0.078
	hypothetical protein					
	hypothetical proteins					

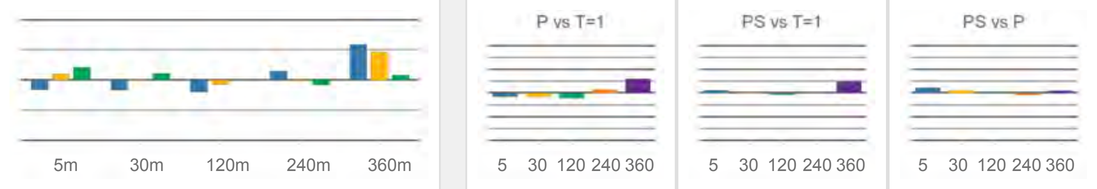

Locus		5m	30m	120m	240m	360m
**PGN_1085**	P vs T=1	0.788	0.602	0.415	0.810	1.197
	PS vs T=1	0.845	0.416	0.531	0.700	0.800
	PS vs P	0.079	-0.179	0.085	-0.076	-0.346
	ferrous iron transport protein B					
	transport and binding proteins					

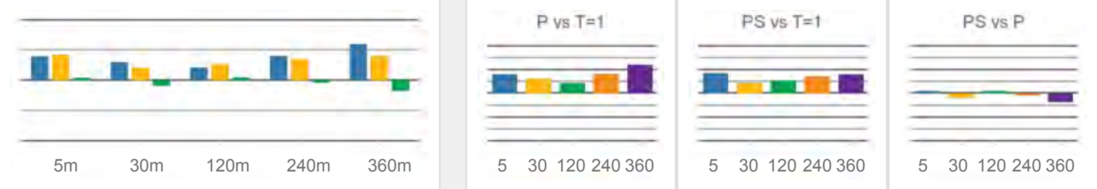

PgSg Time Course

Hackett Laboratory UW

log$_2$ (Fold Change)

Legend: P vs T=1 | PS vs T=1 | PS vs P

Locus		5m	30m	120m	240m	360m
**PGN_1086**	P vs T=1	0.160	0.244	0.988	1.018	0.913
	PS vs T=1	0.137	0.134	0.187	0.185	0.138
	PS vs P	-0.044	-0.128	-0.599	-0.577	-0.611

hypothetical protein

hypothetical proteins

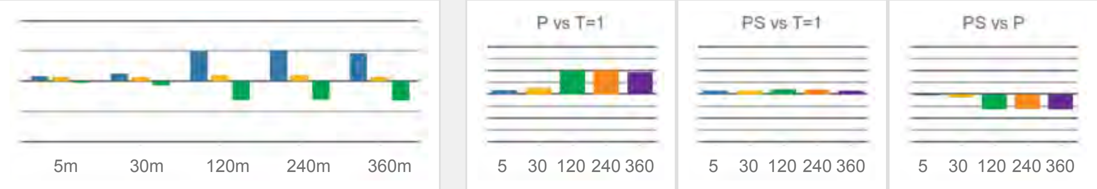

Locus		5m	30m	120m	240m	360m
**PGN_1087**	P vs T=1	0.726	0.804	0.933	0.945	1.332
	PS vs T=1	0.747	0.839	0.812	0.926	0.807
	PS vs P	0.022	0.043	-0.088	0.015	-0.434

conserved hypothetical protein

hypothetical proteins-Conserved

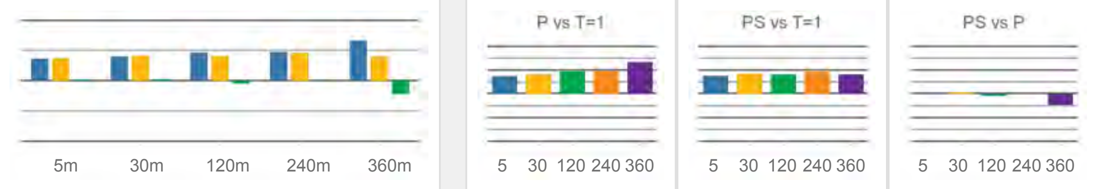

Locus		5m	30m	120m	240m	360m
**PGN_1088**	P vs T=1	1.872	2.223	1.984	1.284	0.522
rpsA	PS vs T=1	1.261	1.699	1.482	0.803	0.531
	PS vs P	-0.579	-0.469	-0.460	-0.476	-0.013

30S ribosomal protein S1

protein synthesis

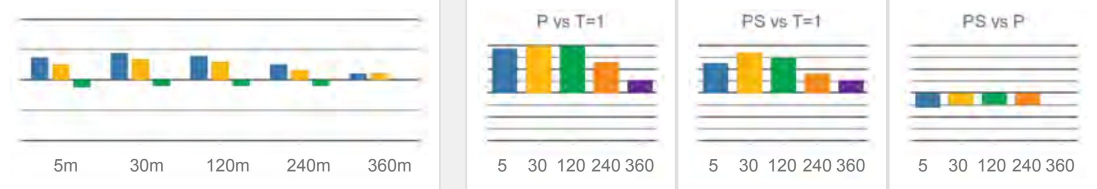

Locus		5m	30m	120m	240m	360m
**PGN_1089**	P vs T=1	-0.966	-1.176	-1.397	-0.797	0.098
	PS vs T=1	-1.008	-1.299	-0.388	0.224	-0.067
	PS vs P	-0.058	-0.182	0.764	0.974	-0.042

probable methyltransferase

hypothetical proteins-Conserved

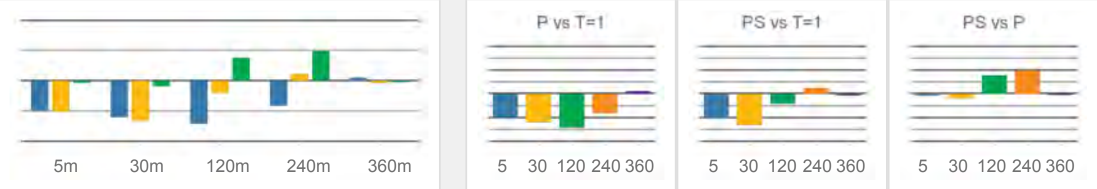

Locus		5m	30m	120m	240m	360m
**PGN_1090**	P vs T=1	-0.011	0.023	-0.250	-0.652	-0.771
	PS vs T=1	-1.307	-1.191	-0.584	-0.200	-0.469
	PS vs P	-1.220	-1.129	-0.306	0.402	0.263

conserved hypothetical protein

hypothetical proteins-Conserved

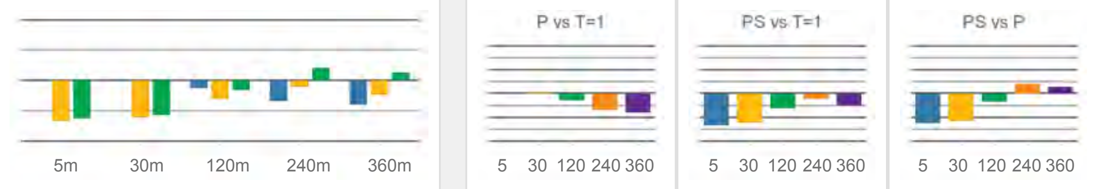

Locus		5m	30m	120m	240m	360m
**PGN_1091**	P vs T=1	0.936	0.787	0.390	-0.189	-1.029
	PS vs T=1	-0.140	0.303	0.810	0.770	0.468
	PS vs P	-1.024	-0.447	0.426	0.921	1.429

conserved hypothetical protein

hypothetical proteins-Conserved

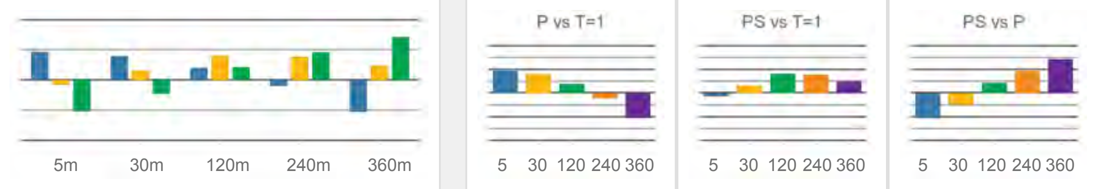

Locus		5m	30m	120m	240m	360m
**PGN_1092**	P vs T=1	0.378	0.200	0.286	0.672	1.030
	PS vs T=1	-0.399	-0.701	-0.127	0.155	0.127
	PS vs P	-0.748	-0.878	-0.399	-0.451	-0.842

ATP-dependent exoDNAse alpha subunit

DNA metabolism

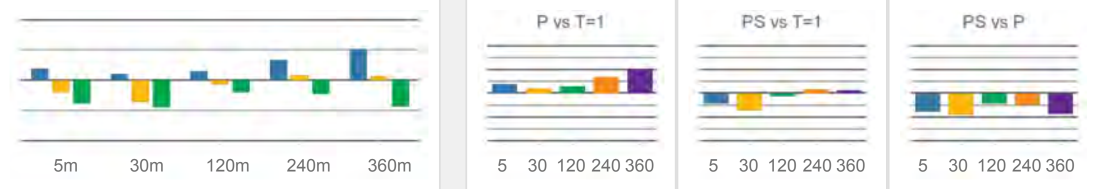

log$_2$ (Fold Change)

Legend: P vs T=1, PS vs T=1, PS vs P

Locus		5m	30m	120m	240m	360m
**PGN_1093**	P vs T=1	0.278	0.231	0.104	0.672	0.898
	PS vs T=1	-0.303	0.208	0.325	0.326	0.504
	PS vs P	-0.572	-0.020	0.200	-0.306	-0.365
	conserved hypothetical protein					
	hypothetical proteins-Conserved					
**PGN_1094**	P vs T=1	0.532	1.296	2.008	1.873	1.194
	PS vs T=1	1.301	1.483	1.546	1.598	1.487
	PS vs P	0.689	0.185	-0.407	-0.240	0.277
	glycine dehydrogenase					
	energy metabolism					
**PGN_1095**	P vs T=1	-0.413	-0.611	-0.689	-0.239	-0.025
	PS vs T=1	-0.265	-0.102	-0.243	-0.004	-0.103
	PS vs P	0.134	0.456	0.337	0.242	-0.054
	probable metallo-beta-lactamase					
	unknown function					
**PGN_1096**	P vs T=1	-0.962	-0.906	-0.943	-0.977	-0.959
	PS vs T=1	-1.151	-1.163	-0.878	-0.475	-0.613
	PS vs P	-0.200	-0.262	0.045	0.462	0.321
	glucose-inhibited division protein B					
	unknown function					
**PGN_1097**	P vs T=1	-0.568	-1.396	-1.691	-1.536	-1.581
	PS vs T=1	-0.879	-1.166	-0.916	-0.545	-0.921
	PS vs P	-0.237	0.122	0.605	0.854	0.550
	conserved hypothetical protein					
	hypothetical proteins-Conserved					
**PGN_1098**	P vs T=1	0.850	0.799	0.792	0.845	0.653
	PS vs T=1	1.084	1.057	1.240	1.082	0.718
	PS vs P	0.235	0.251	0.439	0.244	0.054
	probable succinoglycan biosynthesis regulator ExsB protein					
	unknown function					
**PGN_1099**	P vs T=1	-0.242	-0.393	-0.306	-0.512	-0.042
	PS vs T=1	0.282	0.097	-0.013	-0.231	-0.142
	PS vs P	0.504	0.442	0.267	0.154	-0.077
	probable phosphoesterase					
	hypothetical proteins-Conserved					

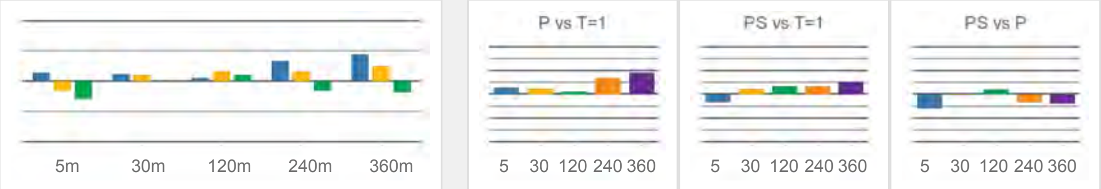

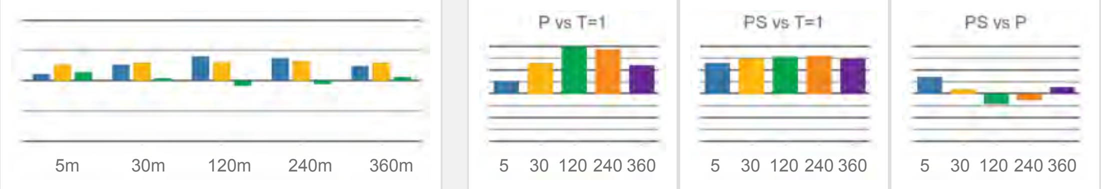

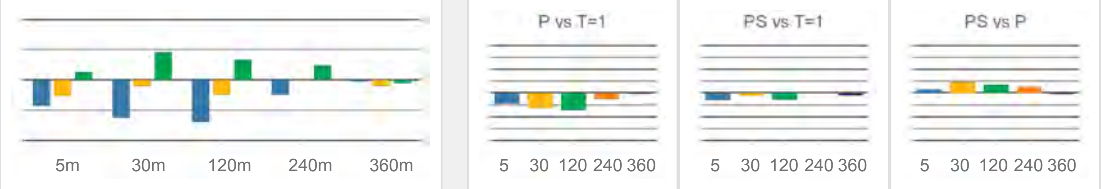

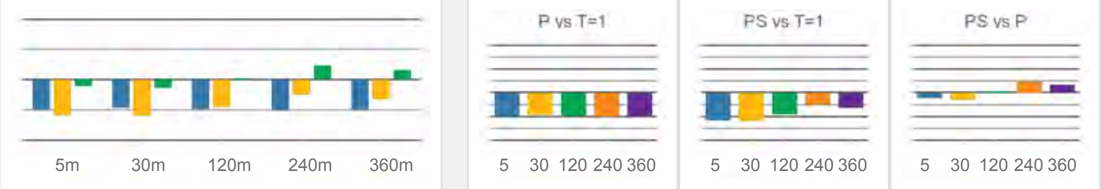

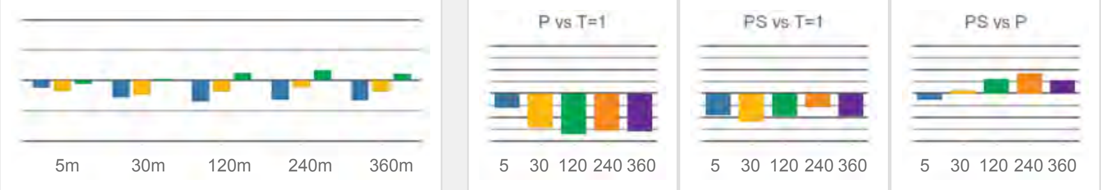

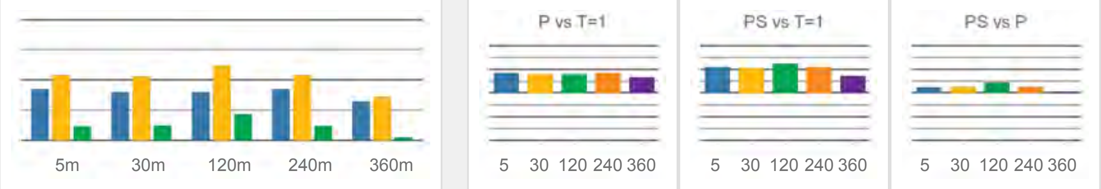

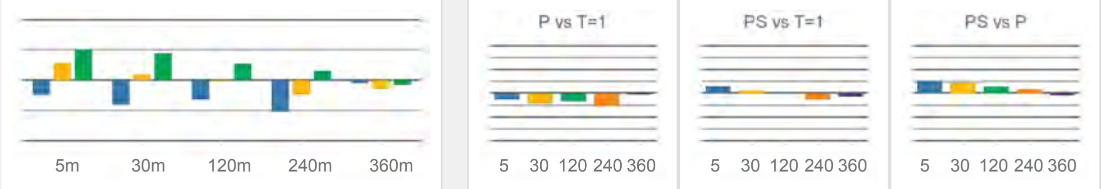

PgSg Time Course

Legend: P vs T=1 · PS vs T=1 · PS vs P

log$_2$ (Fold Change)

Locus		5m	30m	120m	240m	360m
**PGN_1100**	P vs T=1	-0.245	-0.255	-0.459	-0.291	0.346
	PS vs T=1	0.506	0.158	-0.003	0.056	0.143
	PS vs P	0.734	0.397	0.402	0.310	-0.164

putative capsule biosynthesis protein CapA

cell envelope

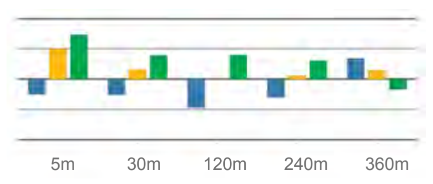

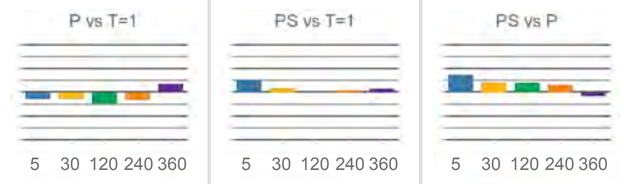

Locus		5m	30m	120m	240m	360m
**PGN_1101**	P vs T=1	-0.386	-0.594	0.034	0.218	0.378
	PS vs T=1	0.400	0.511	0.554	0.661	0.652
	PS vs P	0.639	0.808	0.514	0.488	0.360

conserved hypothetical protein

hypothetical proteins-Conserved

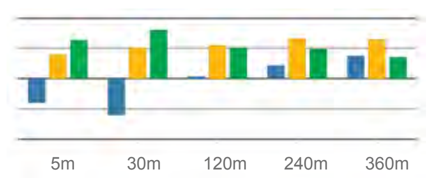

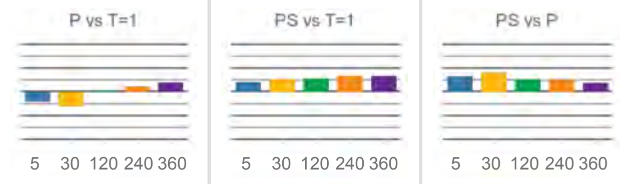

Locus		5m	30m	120m	240m	360m
**PGN_1102**	P vs T=1	-0.069	-0.161	0.007	1.246	1.344
	PS vs T=1	-0.099	-0.074	-0.261	0.341	1.173
	PS vs P	-0.099	-0.068	-0.442	-0.226	0.239

hypothetical protein

hypothetical proteins

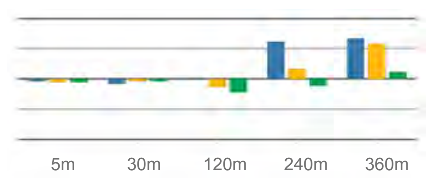

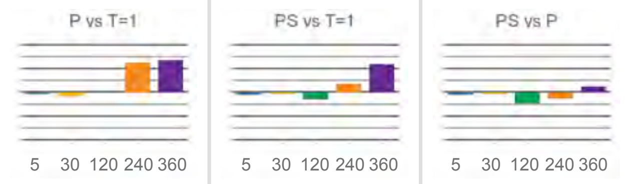

Locus		5m	30m	120m	240m	360m
**PGN_1103**	P vs T=1	0.540	0.416	0.002	-0.426	-1.049
	PS vs T=1	0.291	-0.031	-0.321	-0.403	-0.468
	PS vs P	-0.206	-0.407	-0.308	0.009	0.538

dipeptidase

hypothetical proteins-Conserved

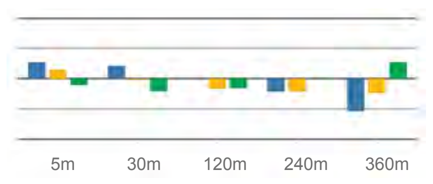

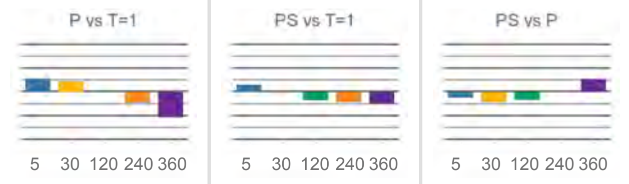

Locus		5m	30m	120m	240m	360m
**PGN_1104**	P vs T=1	1.859	2.084	2.280	2.537	2.324
	PS vs T=1	1.472	1.516	1.513	1.810	2.047
	PS vs P	-0.375	-0.544	-0.741	-0.685	-0.259

chorismate synthase

amino acid biosynthesis

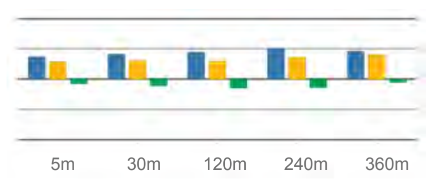

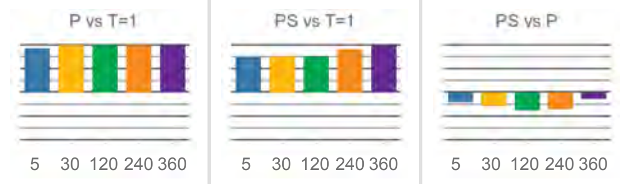

Locus		5m	30m	120m	240m	360m
**PGN_1105**	P vs T=1	0.499	0.760	1.185	1.268	0.714
	PS vs T=1	-0.175	0.282	0.294	0.178	0.295
	PS vs P	-0.659	-0.446	-0.834	-1.028	-0.407

probable FKBP-type peptidyl-prolyl cis-trans isomerase

protein fate

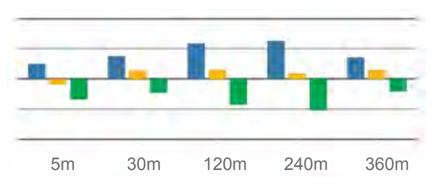

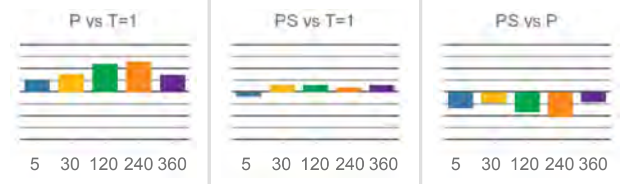

Locus		5m	30m	120m	240m	360m
**PGN_1106**	P vs T=1	1.687	1.591	1.721	1.928	1.541
	PS vs T=1	1.568	1.998	1.754	1.564	1.604
	PS vs P	-0.108	0.403	0.040	-0.340	0.062

conserved hypothetical protein

hypothetical proteins-Conserved

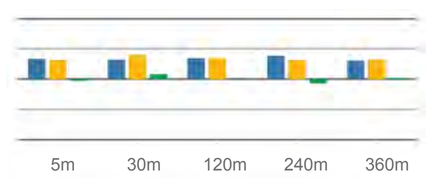

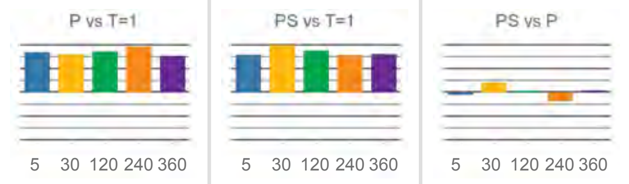

PgSg Time Course

log$_2$ (Fold Change)

Legend: P vs T=1 · PS vs T=1 · PS vs P

Locus		5m	30m	120m	240m	360m
**PGN_1107**	P vs T=1	1.515	1.457	1.356	1.594	1.229
	PS vs T=1	1.253	1.651	1.364	1.127	1.105
	PS vs P	-0.248	0.197	0.006	-0.448	-0.127

conserved hypothetical protein

hypothetical proteins-Conserved

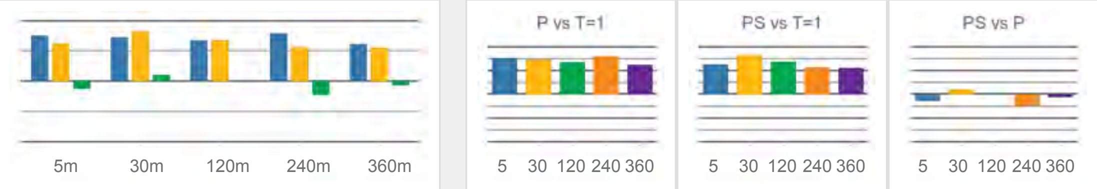

Locus		5m	30m	120m	240m	360m
**PGN_1108**	P vs T=1	0.936	-0.195	-1.346	-1.433	-1.391
	PS vs T=1	0.314	-0.439	-0.138	0.238	0.056
	PS vs P	-0.466	-0.247	0.992	1.419	1.294

putative RNA polymerase ECF-type sigma factor

transcription

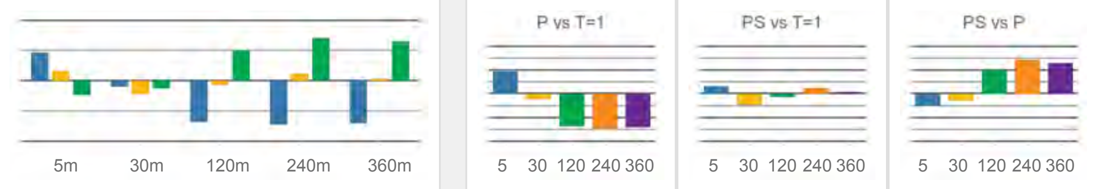

Locus		5m	30m	120m	240m	360m
**PGN_1109**	P vs T=1	0.740	0.315	0.592	0.965	2.221
	PS vs T=1	-0.385	-0.867	-0.317	0.138	0.713
	PS vs P	-0.859	-0.973	-0.699	-0.452	-0.897

hypothetical protein

hypothetical proteins

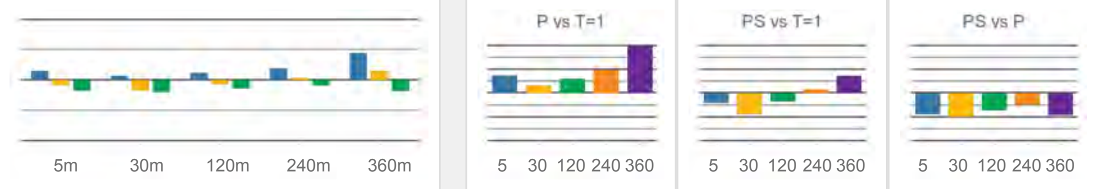

Locus		5m	30m	120m	240m	360m
**PGN_1110**	P vs T=1	0.232	0.251	0.254	0.579	0.228
	PS vs T=1	-0.046	-0.169	0.015	0.333	0.425
	PS vs P	-0.282	-0.423	-0.256	-0.236	0.178

partial transposase in ISPg1

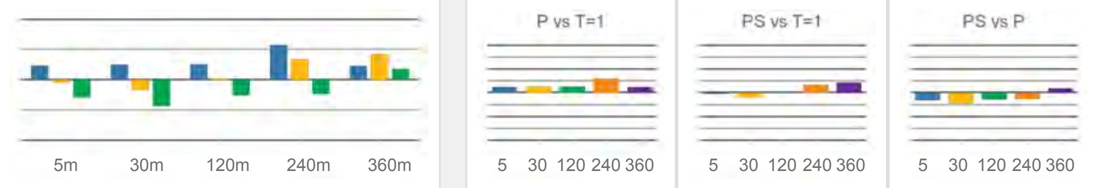

Locus		5m	30m	120m	240m	360m
**PGN_1111**	P vs T=1	1.443	1.902	2.346	2.473	2.031
	PS vs T=1	0.904	1.906	3.117	3.097	2.735
	PS vs P	-0.558	0.011	0.792	0.649	0.699

formate-tetrahydrofolate ligase

central intermediary metabolism

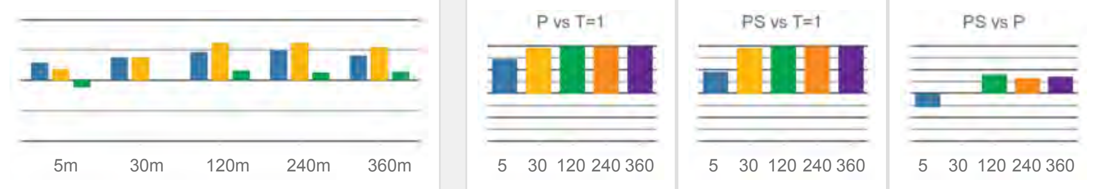

Locus		5m	30m	120m	240m	360m
**PGN_1112**	P vs T=1	0.626	0.531	-0.203	-1.037	-1.701
	PS vs T=1	0.509	0.321	-0.388	-0.763	-0.943
	PS vs P	-0.083	-0.174	-0.175	0.235	0.707

PhoH-like protein

unknown function

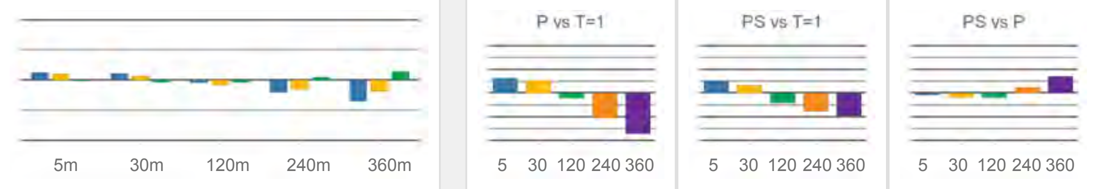

Locus		5m	30m	120m	240m	360m
**PGN_1113** *ruvC*	P vs T=1	0.925	1.067	0.644	0.178	-0.259
	PS vs T=1	0.820	0.705	0.397	0.430	0.270
	PS vs P	-0.093	-0.339	-0.238	0.218	0.481

putative crossover junction endodeoxyribonuclease RuvC

DNA metabolism

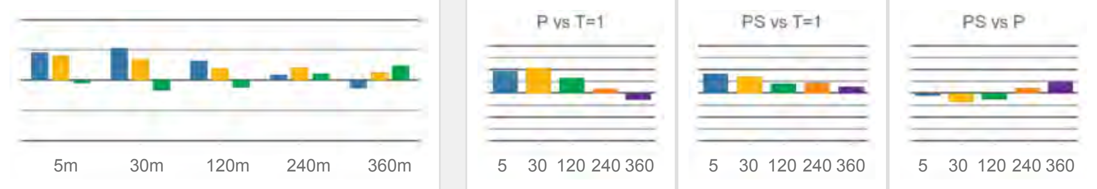

PgSg Time Course

Hackett Laboratory UW

log2 (Fold Change)

Legend: P vs T=1 | PS vs T=1 | PS vs P

Locus		5m	30m	120m	240m	360m
**PGN_1114**	P vs T=1	-0.099	-0.593	-1.216	-1.033	-0.903
	PS vs T=1	0.001	0.073	0.407	0.467	0.261
	PS vs P	0.120	0.606	1.355	1.266	1.042
	conserved hypothetical protein					
	hypothetical proteins-Conserved					
**PGN_1115**	P vs T=1	-0.950	-0.905	-0.784	-1.357	-2.370
	PS vs T=1	-1.613	-1.159	-1.134	-1.857	-2.299
	PS vs P	-0.620	-0.213	-0.263	-0.511	-0.090
	putative hemagglutinin					
	cellular processes					
**PGN_1116**	P vs T=1	-0.078	0.655	1.030	0.771	-0.014
	PS vs T=1	0.965	1.186	1.084	0.686	0.494
	PS vs P	1.018	0.531	0.065	-0.081	0.494
	putative aminotransferase					
	unknown function					
**PGN_1117**	P vs T=1	-0.163	0.154	0.292	-0.107	-0.523
	PS vs T=1	0.855	0.552	0.108	0.010	-0.167
	PS vs P	0.986	0.396	-0.164	0.105	0.329
	acetyl-CoA synthetase					
	energy metabolism					
**PGN_1118**	P vs T=1	0.380	0.315	0.374	0.424	0.052
	PS vs T=1	0.180	-0.005	0.151	0.214	0.215
	PS vs P	-0.196	-0.323	-0.227	-0.207	0.134
	transposase in ISPg1					
**PGN_1119**	P vs T=1	-0.038	0.446	0.495	0.053	-0.703
	PS vs T=1	0.227	0.892	1.423	1.337	1.084
	PS vs P	0.223	0.438	0.923	1.241	1.718
	probable large conductance mechanosensitive channel protein					
	cellular processes					
**PGN_1120**	P vs T=1	-0.457	-0.515	-0.531	-0.743	-0.995
	PS vs T=1	-0.256	-0.252	-0.004	0.238	-0.071
	PS vs P	0.192	0.249	0.517	0.945	0.885
	putative NADPH-NAD transhydrogenase					
	central intermediary metabolism					

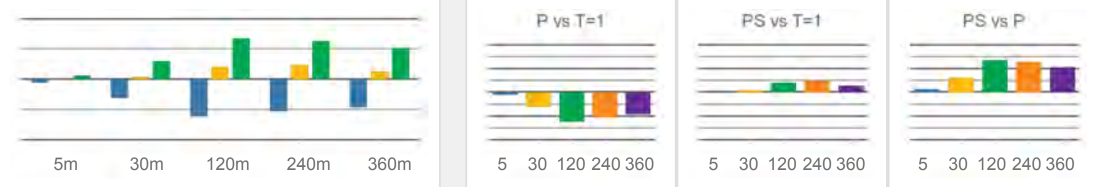

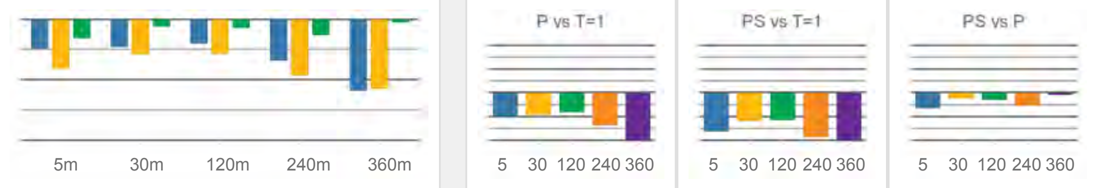

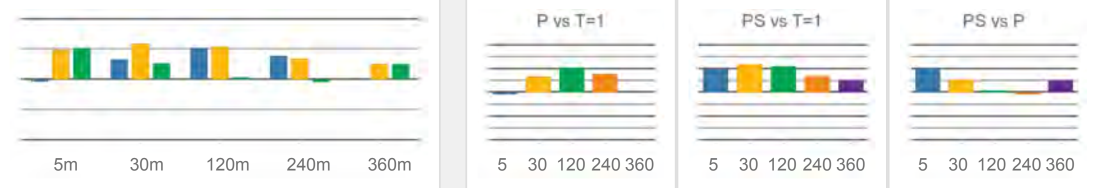

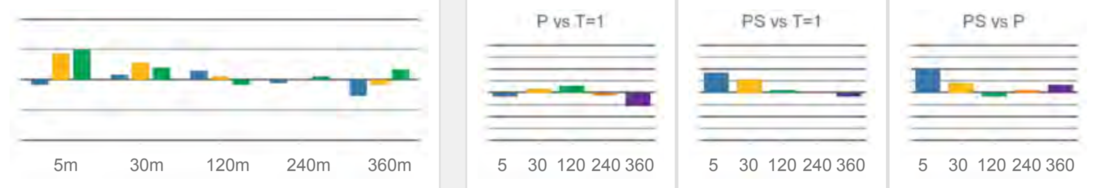

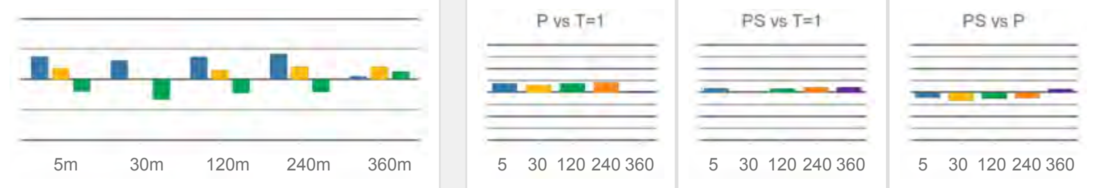

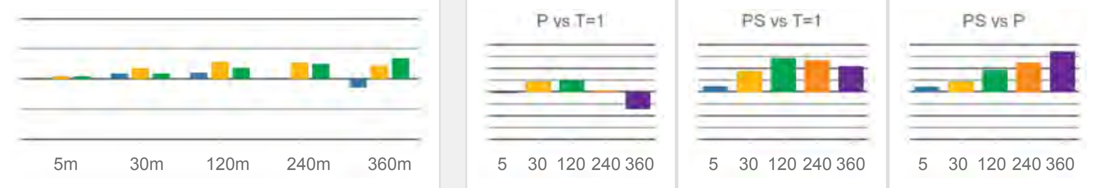

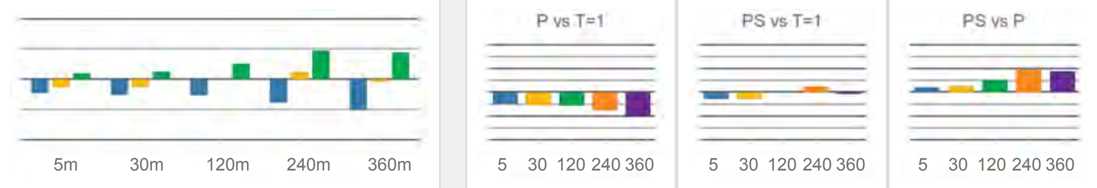

PgSg Time Course

log_2 (Fold Change)

P vs T=1 | PS vs T=1 | PS vs P

Locus		5m	30m	120m	240m	360m
**PGN_1121**	P vs T=1	-0.422	0.592	1.262	1.038	0.641
	PS vs T=1	-0.058	0.179	0.857	1.116	1.114
	PS vs P	0.214	-0.382	-0.297	0.130	0.443

probable NADPH-NAD transhydrogenase alpha subunit

central intermediary metabolism

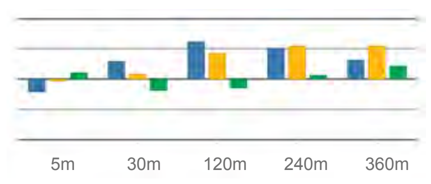

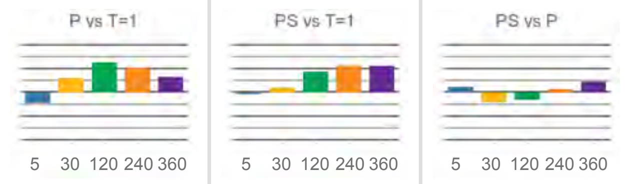

Locus		5m	30m	120m	240m	360m
**PGN_1122**	P vs T=1	-0.366	-0.082	0.492	0.514	0.285
	PS vs T=1	0.223	-0.015	-0.218	0.189	0.212
	PS vs P	0.528	0.046	-0.661	-0.283	-0.066

NADPH-NAD transhydrogenase beta subunit

energy metabolism

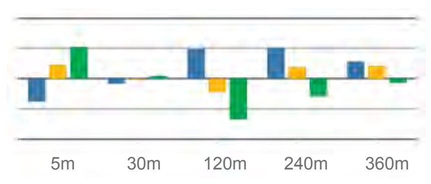

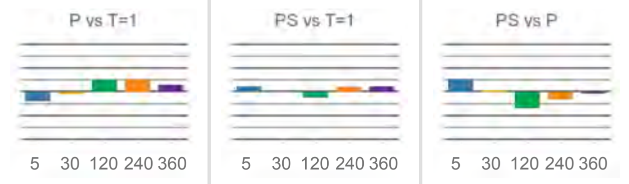

Locus		5m	30m	120m	240m	360m
**PGN_1123**	P vs T=1	-0.162	0.337	0.863	0.867	0.794
	PS vs T=1	0.428	0.406	0.021	0.118	0.112
	PS vs P	0.529	0.070	-0.778	-0.684	-0.645

conserved hypothetical protein

hypothetical proteins-Conserved

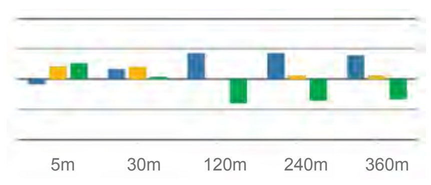

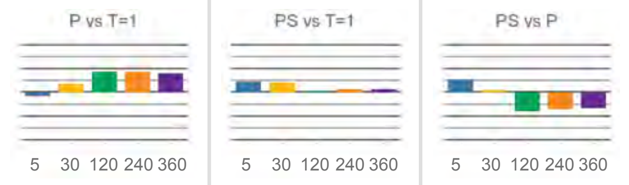

Locus		5m	30m	120m	240m	360m
**PGN_1124**	P vs T=1	1.264	1.595	1.693	1.369	0.673
	PS vs T=1	1.325	1.497	1.134	0.814	0.691
	PS vs P	0.062	-0.081	-0.536	-0.543	0.005

Band 7 protein

unknown function

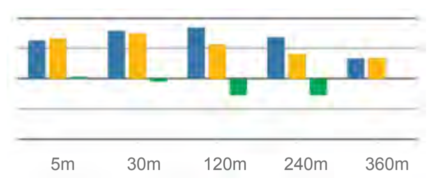

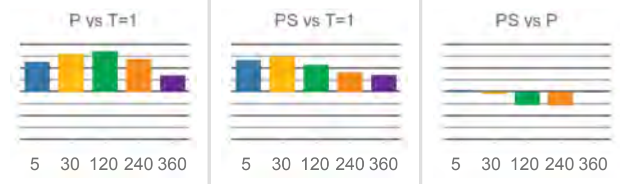

Locus		5m	30m	120m	240m	360m
**PGN_1125**	P vs T=1	0.242	0.090	-0.432	-0.587	-0.852
	PS vs T=1	0.383	0.358	0.326	0.166	-0.195
	PS vs P	0.158	0.277	0.715	0.680	0.581

conserved hypothetical protein

cell envelope

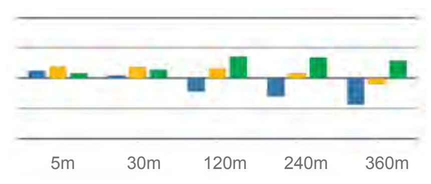

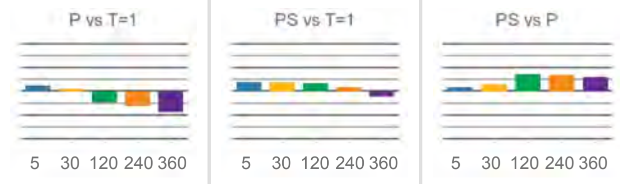

Locus		5m	30m	120m	240m	360m
**PGN_1126**	P vs T=1	-0.621	-0.841	-0.786	0.059	-0.030
	PS vs T=1	-0.628	-0.481	0.461	0.931	0.768
	PS vs P	-0.042	0.272	1.087	0.904	0.783

putative error-prone repair: SOS-response transcriptional repressor UmuD homolog

DNA metabolism

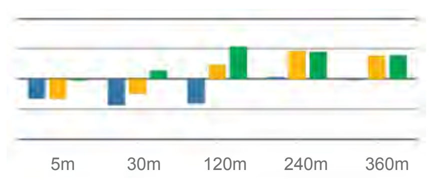

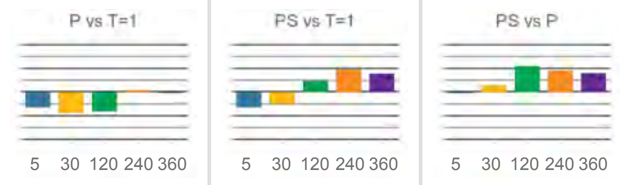

Locus		5m	30m	120m	240m	360m
**PGN_1127**	P vs T=1	-1.093	-0.951	-0.569	-0.305	-0.124
	PS vs T=1	-0.548	-0.467	-0.207	0.270	0.420
	PS vs P	0.448	0.399	0.315	0.548	0.533

putative SOS mutagenesis and repair protein UmuC homolog

DNA metabolism

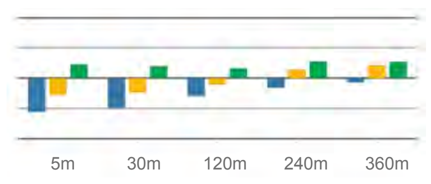

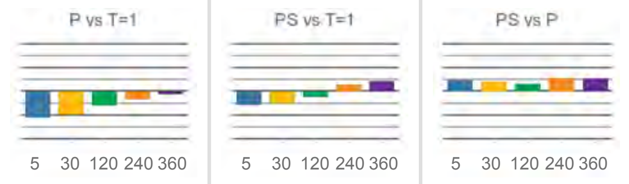

log2 (Fold Change)

Legend: P vs T=1 | PS vs T=1 | PS vs P

Locus		5m	30m	120m	240m	360m
**PGN_1128**	P vs T=1	0.342	0.368	0.216	0.112	-0.178
	PS vs T=1	-0.106	0.116	0.485	0.864	0.683
	PS vs P	-0.432	-0.236	0.263	0.731	0.829
	L-lactate permease					
	transport and binding proteins					
**PGN_1129**	P vs T=1	0.577	1.051	1.854	1.981	1.194
	PS vs T=1	-0.047	0.531	1.011	0.879	0.751
	PS vs P	-0.636	-0.501	-0.784	-1.039	-0.436
	conserved hypothetical protein					
	hypothetical proteins-Conserved					
**PGN_1130**	P vs T=1	-0.342	-0.005	0.953	1.344	0.740
	PS vs T=1	-0.746	-0.281	0.314	0.399	0.410
	PS vs P	-0.457	-0.299	-0.571	-0.843	-0.314
	putative UDP-N-acetylenolpyruvoylglucosamine reductase					
	cell envelope					
**PGN_1131**	P vs T=1	0.834	0.608	0.342	0.228	0.763
	PS vs T=1	0.918	0.398	-0.145	0.084	-0.066
	PS vs P	0.108	-0.200	-0.501	-0.186	-0.789
	conserved hypothetical protein					
	protein fate					
**PGN_1132**	P vs T=1	0.405	0.341	0.390	0.554	0.111
	PS vs T=1	0.241	0.043	0.180	0.297	0.274
	PS vs P	-0.163	-0.304	-0.219	-0.247	0.137
	transposase in ISPg1					
**PGN_1133**	P vs T=1	0.037	0.024	0.015	1.252	2.851
	PS vs T=1	-0.380	-0.030	0.349	1.546	1.507
	PS vs P	-0.468	-0.212	-0.148	0.533	-0.248
	hypothetical protein					
	hypothetical proteins					
**PGN_1134**	P vs T=1	0.491	0.268	0.238	0.728	1.385
	PS vs T=1	0.773	0.640	1.218	2.117	1.812
	PS vs P	0.255	0.294	0.844	1.337	0.476
	conserved hypothetical protein					
	cell envelope					

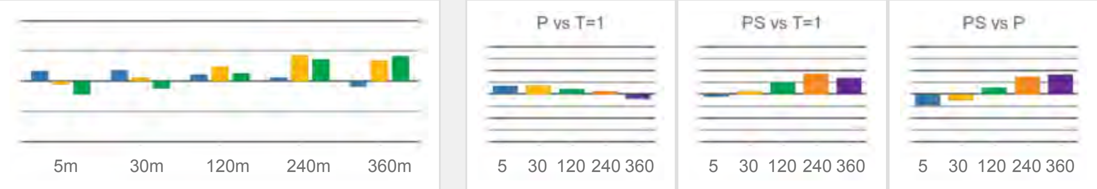

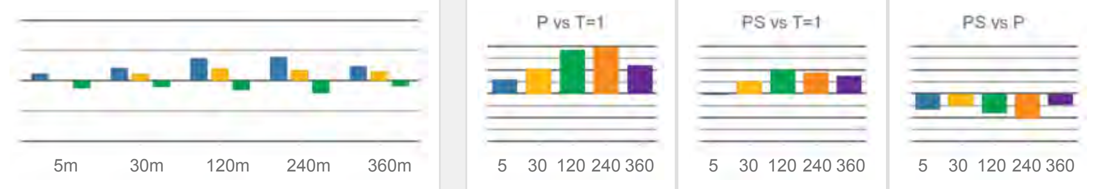

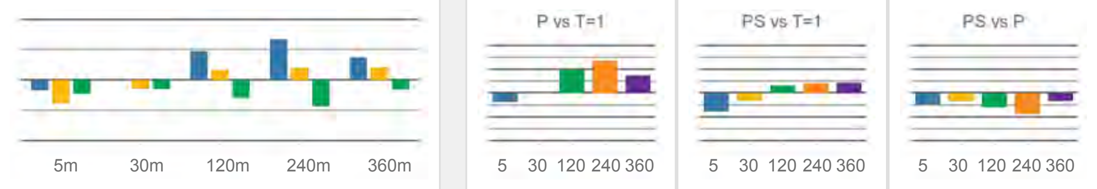

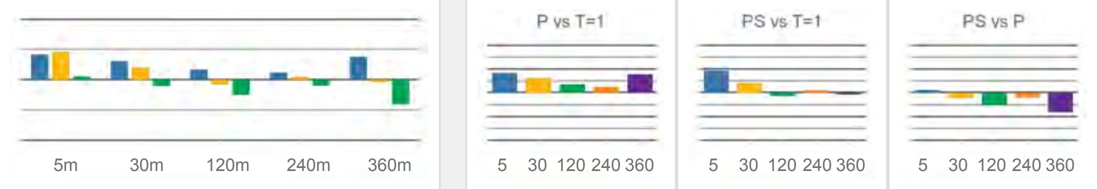

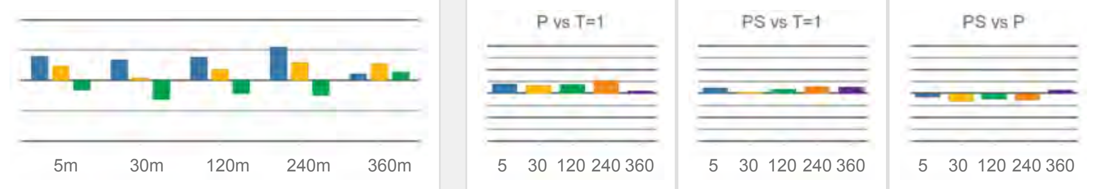

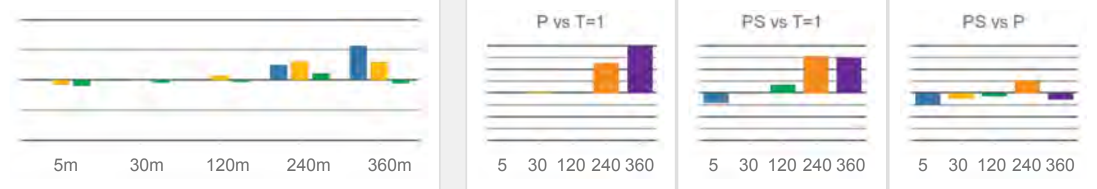

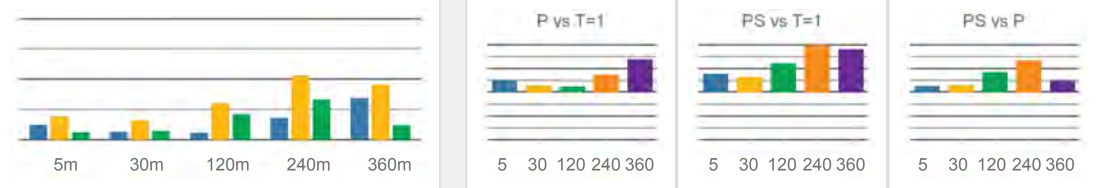

PgSg Time Course

log2 (Fold Change)

Legend: P vs T=1 | PS vs T=1 | PS vs P

Locus		5m	30m	120m	240m	360m
**PGN_1135**	P vs T=1	0.164	-0.037	-0.171	0.315	0.812
	PS vs T=1	0.556	0.009	-0.313	0.011	0.069
	PS vs P	0.397	0.032	-0.199	-0.233	-0.644

putative glycosyltransferase

cell envelope

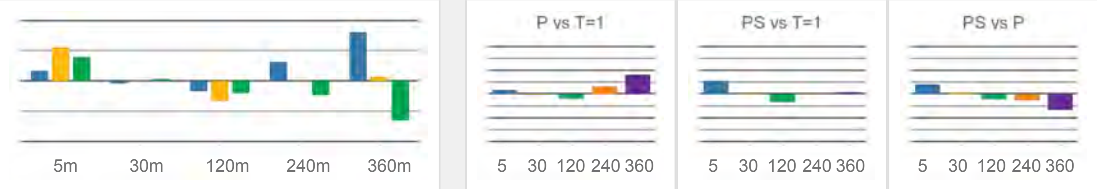

Locus		5m	30m	120m	240m	360m
**PGN_1136**	P vs T=1	-0.747	-0.991	-1.500	-1.209	-1.456
	PS vs T=1	-0.604	-0.580	-0.041	0.286	0.082
	PS vs P	0.132	0.368	1.303	1.394	1.428

probable GTP-cyclohydrolase protein

hypothetical proteins-Conserved

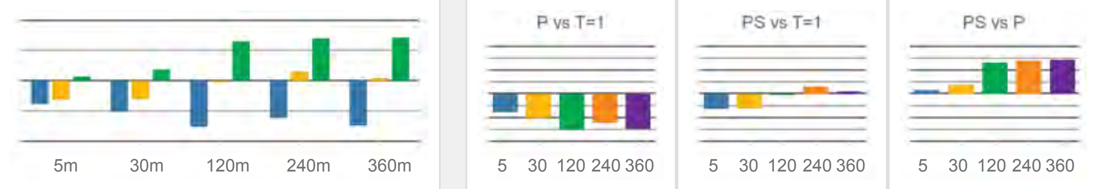

Locus		5m	30m	120m	240m	360m
**PGN_1137**	P vs T=1	-0.080	-0.341	-0.772	-0.753	-0.905
	PS vs T=1	-0.076	-0.169	-0.082	0.217	0.039
	PS vs P	0.019	0.161	0.621	0.898	0.880

conserved hypothetical protein

hypothetical proteins-Conserved

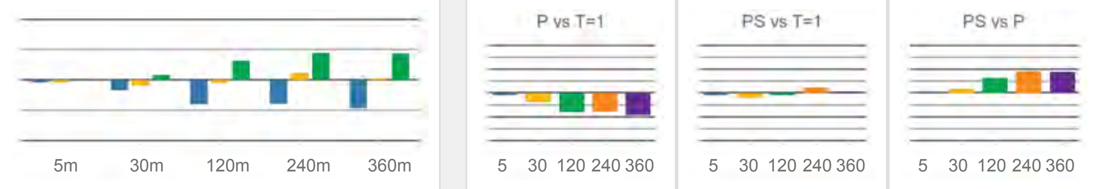

Locus		5m	30m	120m	240m	360m
**PGN_1138**	P vs T=1	-0.366	0.049	0.386	0.112	-0.057
	PS vs T=1	0.107	0.232	-0.044	0.063	0.029
	PS vs P	0.436	0.179	-0.391	-0.054	0.067

conserved hypothetical protein

hypothetical proteins-Conserved

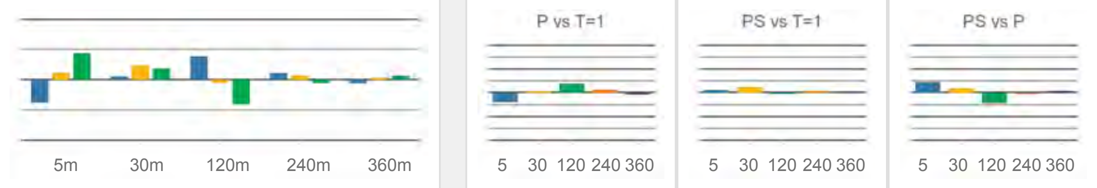

Locus		5m	30m	120m	240m	360m
**PGN_1139**	P vs T=1	1.082	1.558	1.909	1.564	0.911
	PS vs T=1	1.239	1.786	2.109	1.859	1.632
	PS vs P	0.136	0.232	0.223	0.294	0.692

conserved hypothetical protein

hypothetical proteins-Conserved

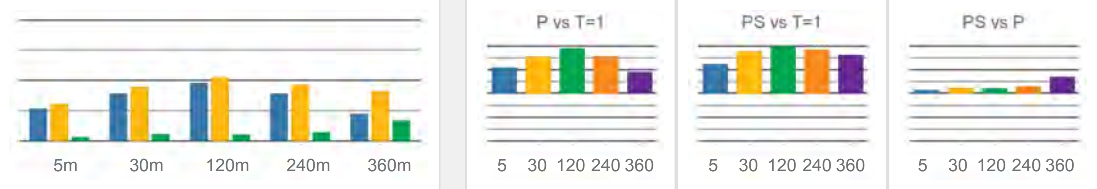

Locus		5m	30m	120m	240m	360m
**PGN_1140**	P vs T=1	0.316	0.670	0.552	-0.003	-0.611
	PS vs T=1	0.315	0.704	0.878	0.725	0.492
	PS vs P	-0.008	0.048	0.337	0.692	1.047

putative orotate phosphoribosyltransferase

purines, pyrimidines, nucleosides and nucleotides

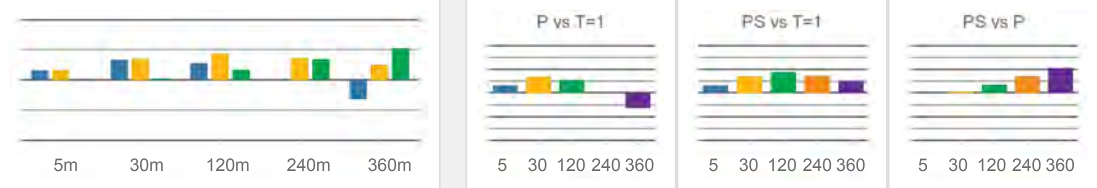

Locus		5m	30m	120m	240m	360m
**PGN_1141**	P vs T=1	-0.196	0.045	0.167	-0.053	-0.445
	PS vs T=1	0.570	0.346	0.070	0.296	0.150
	PS vs P	0.743	0.293	-0.093	0.326	0.551

putative amidohydrolase

unknown function

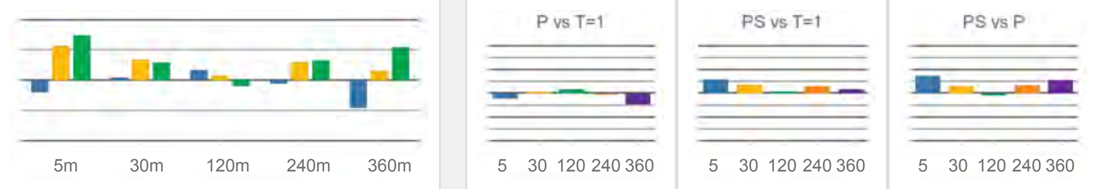

PgSg Time Course

log2 (Fold Change)

Legend: P vs T=1 · PS vs T=1 · PS vs P

Locus		5m	30m	120m	240m	360m
**PGN_1142**	P vs T=1	-0.327	-0.368	-0.484	-0.850	-1.116
	PS vs T=1	0.504	0.273	0.001	0.243	-0.002
	PS vs P	0.812	0.620	0.464	1.014	1.046

probable acetyltransferase

unknown function

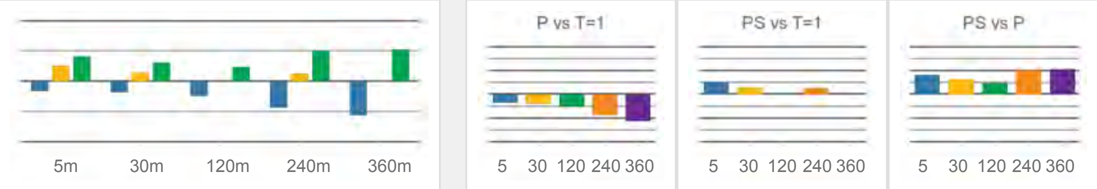

Locus		5m	30m	120m	240m	360m
**PGN_1143**	P vs T=1	-0.224	-0.314	-1.101	-1.681	-2.184
	PS vs T=1	0.179	0.252	-0.061	-0.279	-0.602
	PS vs P	0.415	0.575	0.953	1.187	1.360

conserved hypothetical protein

hypothetical proteins-Conserved

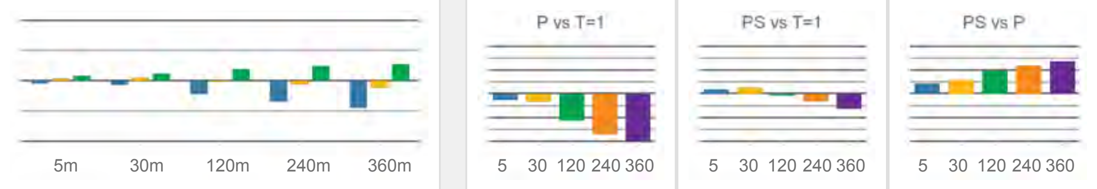

Locus		5m	30m	120m	240m	360m
**PGN_1144**	P vs T=1	-1.338	-1.544	-1.675	-1.589	-1.225
	PS vs T=1	-0.988	-1.379	-0.361	0.247	0.484
	PS vs P	0.189	-0.070	0.895	1.339	1.470

hypothetical protein

hypothetical proteins

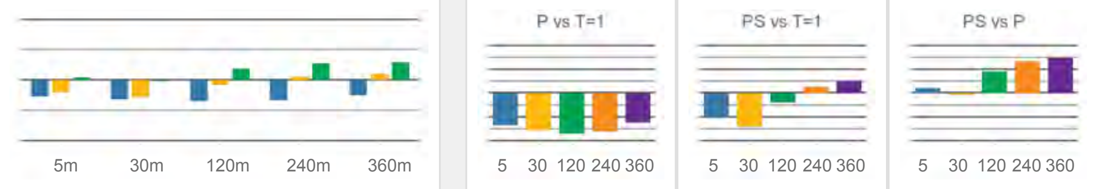

Locus		5m	30m	120m	240m	360m
**PGN_1145**	P vs T=1	-0.093	-0.445	-0.352	-0.081	-0.710
	PS vs T=1	0.015	0.354	0.995	1.453	1.336
	PS vs P	0.082	0.650	1.205	1.459	1.828

conserved hypothetical protein

hypothetical proteins-Conserved

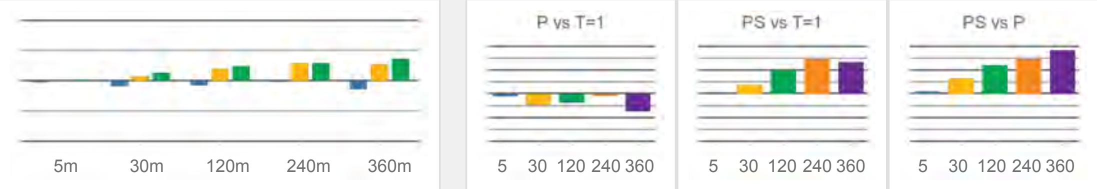

Locus		5m	30m	120m	240m	360m
**PGN_1146**	P vs T=1	-0.228	-0.431	-0.673	-0.801	-1.067
	PS vs T=1	-0.239	0.223	0.619	0.663	0.485
	PS vs P	-0.012	0.625	1.241	1.391	1.483

conserved hypothetical protein

hypothetical proteins-Conserved

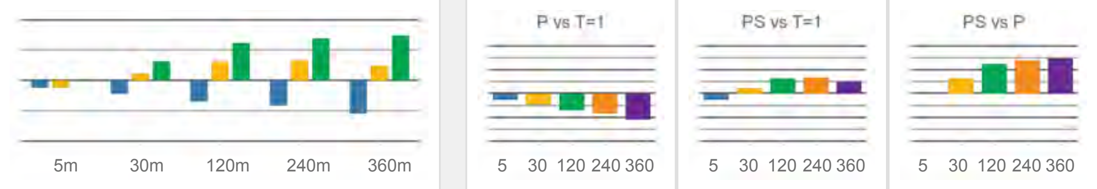

Locus		5m	30m	120m	240m	360m
**PGN_1147**	P vs T=1	0.298	0.435	0.722	0.725	0.722
	PS vs T=1	0.574	0.816	0.654	0.606	0.510
	PS vs P	0.257	0.367	-0.056	-0.107	-0.202

conserved hypothetical protein

hypothetical proteins-Conserved

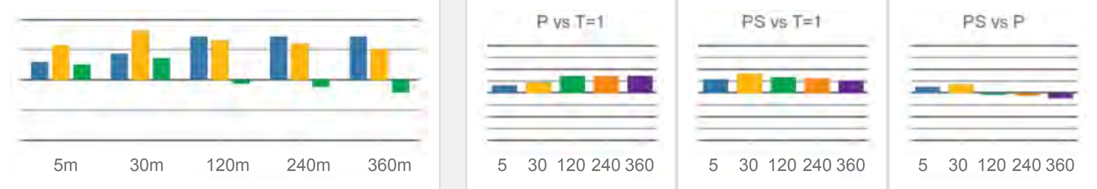

Locus		5m	30m	120m	240m	360m
**PGN_1148**	P vs T=1	0.819	1.334	1.492	1.157	0.477
	PS vs T=1	1.247	1.537	1.311	0.690	0.446
	PS vs P	0.410	0.213	-0.160	-0.458	-0.049

phosphoribosylamine-glycine ligase

purines, pyrimidines, nucleosides and nucleotides

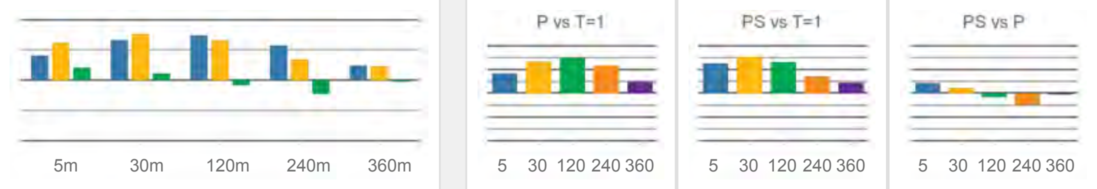

PgSg Time Course

Legend: P vs T=1 | PS vs T=1 | PS vs P

Locus		log₂ (Fold Change) 5m	30m	120m	240m	360m
**PGN_1149**	P vs T=1	0.874	1.086	0.966	0.479	-0.118
	PS vs T=1	1.315	1.278	0.817	0.272	0.102
	PS vs P	0.437	0.201	-0.138	-0.218	0.192

prolyl tripeptidase A

protein fate

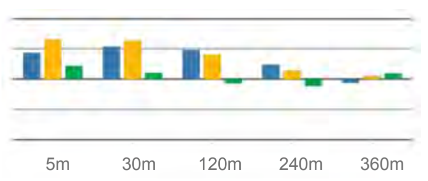

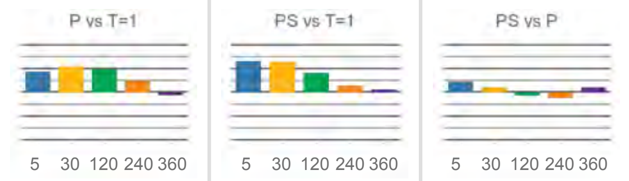

Locus		5m	30m	120m	240m	360m
**PGN_1150**	P vs T=1	-0.141	-0.680	-1.472	-1.760	-1.929
	PS vs T=1	-0.235	-0.693	-0.980	-0.943	-1.254
	PS vs P	-0.052	-0.015	0.427	0.718	0.600

putative N6-adenine-specific DNA methylase

hypothetical proteins-Conserved

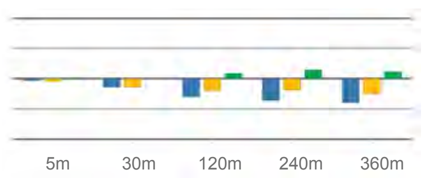

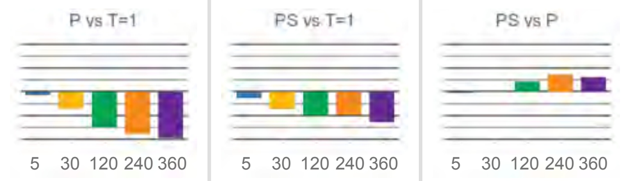

Locus		5m	30m	120m	240m	360m
**PGN_1151**	P vs T=1	-0.661	-0.925	-1.405	-1.375	-0.797
	PS vs T=1	0.070	-0.301	-1.034	-1.429	-1.301
	PS vs P	0.726	0.600	0.292	-0.131	-0.485

1-deoxy-D-xylulose-5-phosphate reductoisomerase

biosynthesis of cofactors, prosthetic groups, and carriers

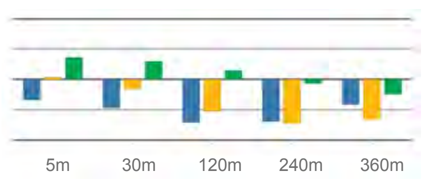

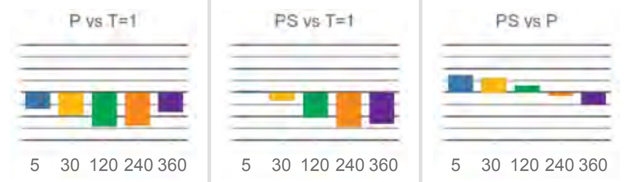

Locus		5m	30m	120m	240m	360m
**PGN_1152**	P vs T=1	-0.039	-0.082	-0.437	-0.789	-0.625
	PS vs T=1	0.546	0.365	-0.233	-0.442	-0.665
	PS vs P	0.577	0.441	0.170	0.223	-0.079

probable 16S rRNA processing protein

transcription

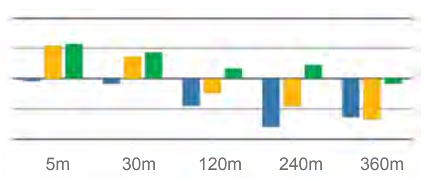

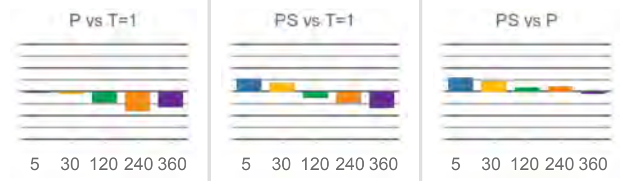

Locus		5m	30m	120m	240m	360m
**PGN_1153**	P vs T=1	-0.117	0.051	-0.210	-0.675	-0.925
	PS vs T=1	0.480	0.503	-0.185	-0.611	-0.850
	PS vs P	0.587	0.456	0.026	0.024	0.039

UDP-N-acetylglucosamine 1-carboxyvinyltransferase

cell envelope

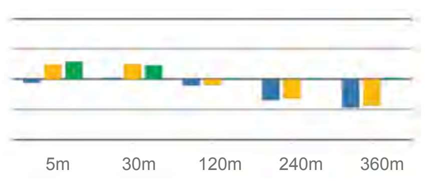

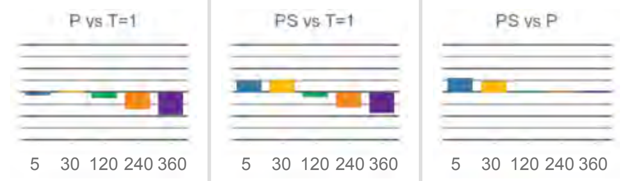

Locus		5m	30m	120m	240m	360m
**PGN_1154**	P vs T=1	-0.457	-0.591	-1.438	-1.821	-1.649
	PS vs T=1	0.236	0.313	-0.537	-1.047	-1.019
	PS vs P	0.691	0.898	0.816	0.593	0.554

conserved hypothetical protein

hypothetical proteins-Conserved

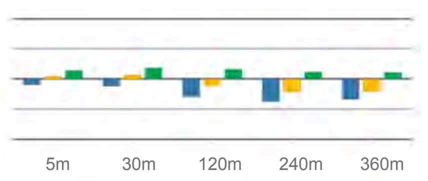

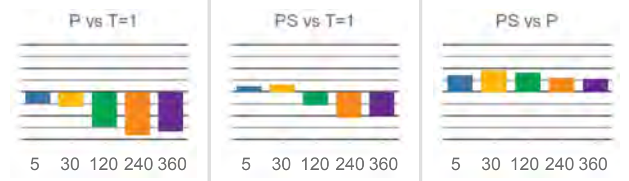

Locus		5m	30m	120m	240m	360m
**PGN_1155**	P vs T=1	0.219	0.089	-0.292	-0.489	-0.653
	PS vs T=1	0.899	0.738	-0.260	-0.723	-0.557
	PS vs P	0.681	0.645	0.018	-0.261	0.069

glucose-6-phosphate isomerase

energy metabolism

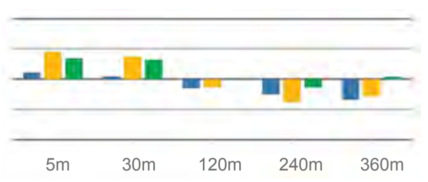

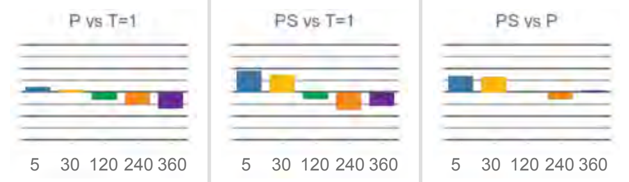

log2 (Fold Change)

Locus		5m	30m	120m	240m	360m
PGN_1156	P vs T=1	-0.072	-0.229	-0.875	-1.265	-1.410
	PS vs T=1	0.291	-0.006	-1.126	-1.827	-2.060
	PS vs P	0.383	0.239	-0.261	-0.601	-0.679
	glycerol-3-phosphate dehydrogenase					
	energy metabolism					
PGN_1157	P vs T=1	0.102	-0.009	-0.693	-1.069	-1.507
	PS vs T=1	0.373	-0.126	-1.094	-1.457	-1.604
	PS vs P	0.295	-0.091	-0.407	-0.411	-0.133
	lysyl-tRNA synthetase					
	protein synthesis					
PGN_1158	P vs T=1	0.028	0.433	0.303	-0.294	-0.954
	PS vs T=1	0.358	0.359	-0.299	-0.654	-0.713
	PS vs P	0.317	-0.048	-0.573	-0.383	0.176
	putative purine nucleoside phosphorylase					
	unknown function					
PGN_1159	P vs T=1	0.285	0.307	-0.228	-0.822	-1.091
	PS vs T=1	0.630	0.290	-0.532	-0.705	-0.900
	PS vs P	0.353	-0.002	-0.305	0.067	0.150
	conserved hypothetical protein					
	hypothetical proteins-Conserved					
PGN_1160	P vs T=1	0.056	-0.659	-0.766	-0.336	0.394
	PS vs T=1	0.394	0.018	-0.147	-0.262	-0.119
	PS vs P	0.352	0.627	0.534	0.063	-0.465
	transposase in ISPg2					
PGN_1161	P vs T=1	0.421	0.349	0.406	0.567	0.210
	PS vs T=1	0.222	0.062	0.169	0.353	0.309
	PS vs P	-0.198	-0.293	-0.246	-0.207	0.078
	transposase in ISPg1					
PGN_1162	P vs T=1	0.641	0.675	0.811	0.998	0.809
	PS vs T=1	0.533	0.841	1.235	1.318	1.212
	PS vs P	-0.113	0.159	0.419	0.324	0.399
	putative CoA transferase subunit A					
	energy metabolism					

P vs T=1 | PS vs T=1 | PS vs P

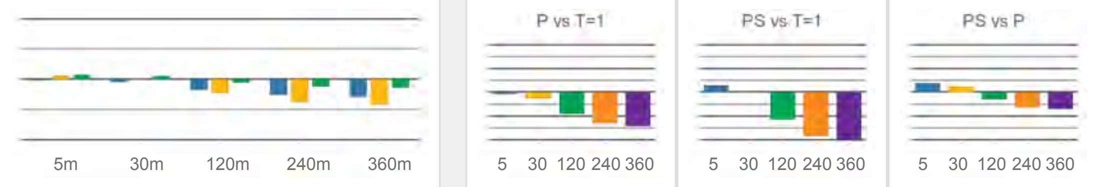

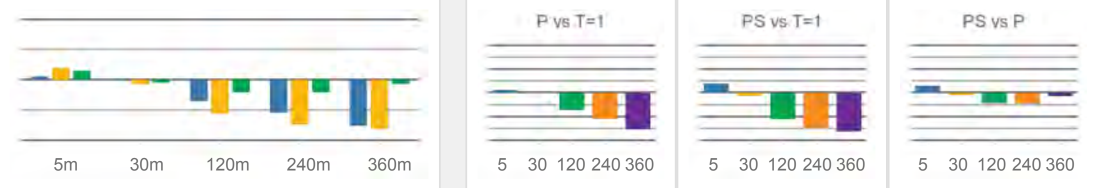

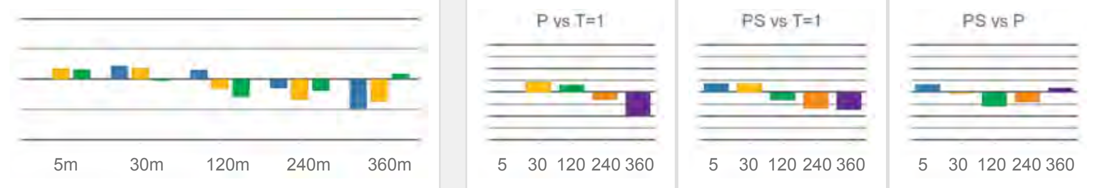

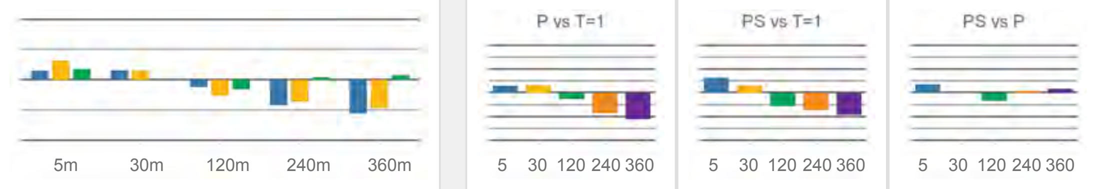

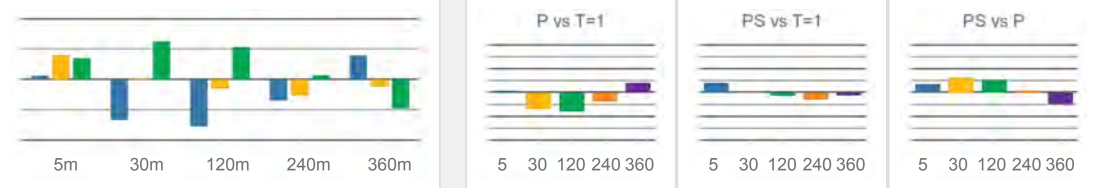

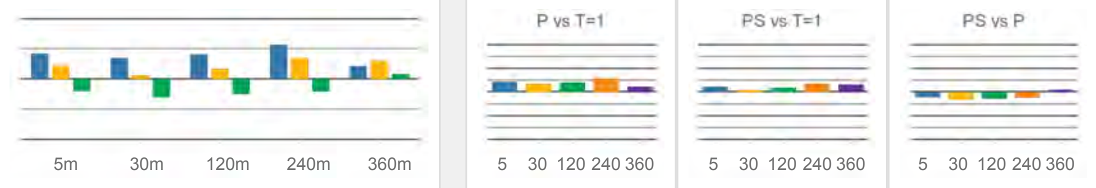

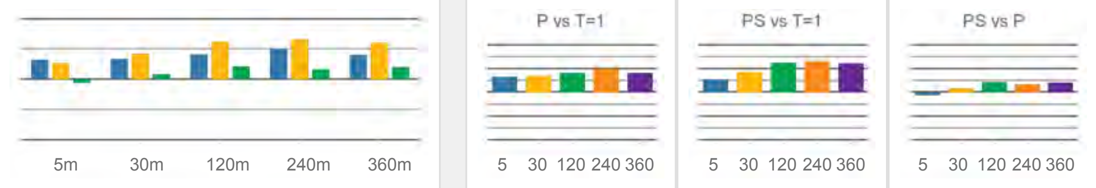

log2 (Fold Change)

Legend: P vs T=1 | PS vs T=1 | PS vs P

Locus		5m	30m	120m	240m	360m
**PGN_1163**	P vs T=1	0.727	0.523	0.123	0.045	-0.081
	PS vs T=1	0.706	0.740	1.190	0.982	0.896
	PS vs P	-0.012	0.212	1.016	0.860	0.920
	conserved hypothetical protein					
	hypothetical proteins-Conserved					

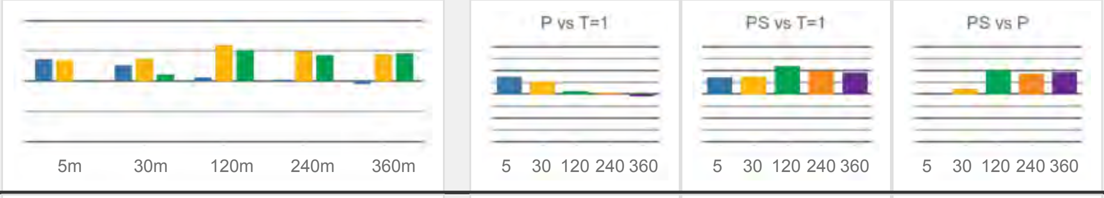

Locus		5m	30m	120m	240m	360m
**PGN_1164**	P vs T=1	1.280	1.381	1.327	1.179	1.058
	PS vs T=1	1.744	1.926	2.167	1.982	1.772
	PS vs P	0.443	0.531	0.823	0.765	0.684
	conserved hypothetical protein with prokaryotic DUF849 domain					
	hypothetical proteins-Conserved					

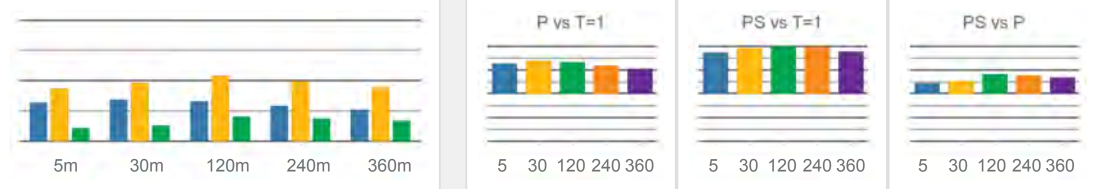

Locus		5m	30m	120m	240m	360m
**PGN_1165**	P vs T=1	0.552	1.094	1.440	1.185	0.539
	PS vs T=1	0.883	1.280	1.428	1.405	1.309
	PS vs P	0.295	0.188	0.011	0.221	0.743
	conserved hypothetical protein					
	energy metabolism					

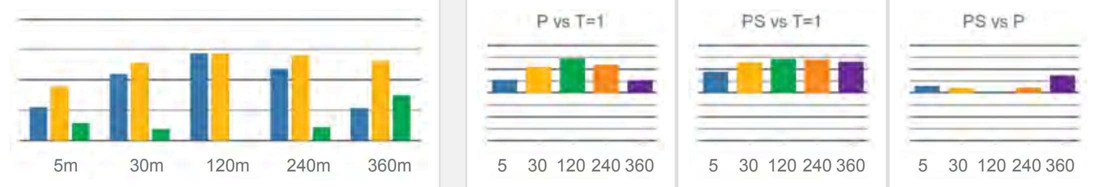

Locus		5m	30m	120m	240m	360m
**PGN_1166**	P vs T=1	0.721	1.285	1.915	1.913	1.305
	PS vs T=1	1.218	1.689	2.072	2.006	1.923
	PS vs P	0.459	0.392	0.171	0.103	0.606
	L-lysine 2,3-aminomutase					
	energy metabolism					

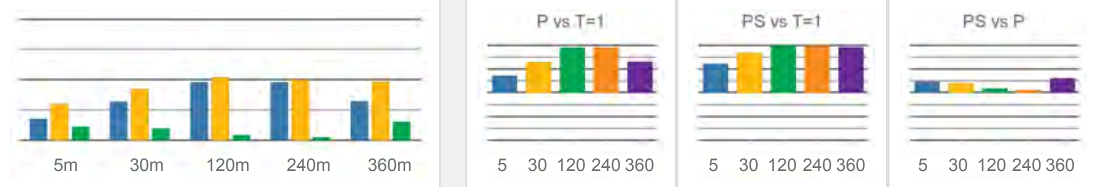

Locus		5m	30m	120m	240m	360m
**PGN_1167**	P vs T=1	0.032	0.090	-0.420	-0.870	-1.067
	PS vs T=1	0.475	0.547	-0.272	-1.033	-1.031
	PS vs P	0.446	0.470	0.137	-0.216	-0.008
	conserved hypothetical protein					
	hypothetical proteins-Conserved					

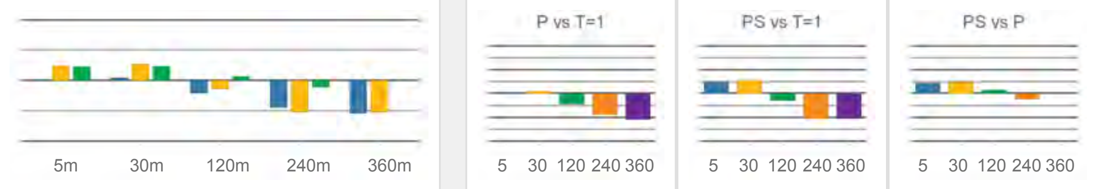

Locus		5m	30m	120m	240m	360m
**PGN_1168**	P vs T=1	-0.043	-0.473	-1.188	-1.541	-1.077
	PS vs T=1	0.535	0.350	-0.935	-1.870	-1.828
	PS vs P	0.610	0.819	0.210	-0.416	-0.748
	probable DNA mismatch repair protein MutS					
	DNA metabolism					

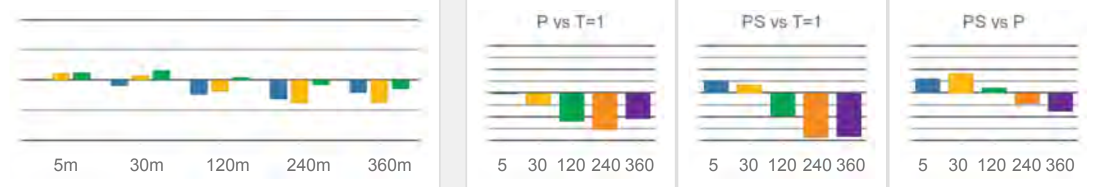

Locus		5m	30m	120m	240m	360m
**PGN_1169**	P vs T=1	-0.747	-1.170	-1.794	-2.041	-1.867
	PS vs T=1	-0.052	-0.394	-1.536	-2.222	-2.353
	PS vs P	0.707	0.764	0.223	-0.234	-0.503
	D-lysine 5,6-aminomutase alpha subunit					
	energy metabolism					

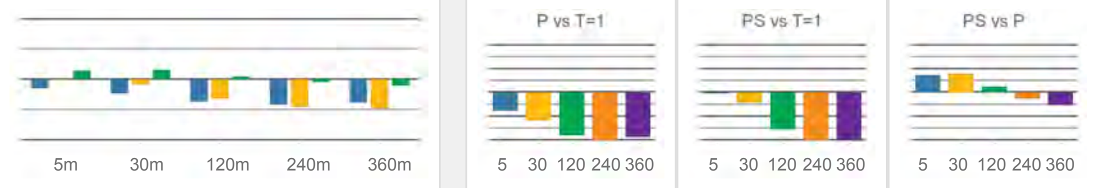

log$_2$ (Fold Change)

P vs T=1 | PS vs T=1 | PS vs P

Locus		5m	30m	120m	240m	360m
**PGN_1170**	P vs T=1	-0.819	-0.962	-0.977	-0.984	-1.204
	PS vs T=1	-0.242	-0.342	-0.912	-1.211	-1.005
	PS vs P	0.556	0.581	0.042	-0.244	0.157
	D-lysine 5,6-aminomutase beta subunit					
	energy metabolism					

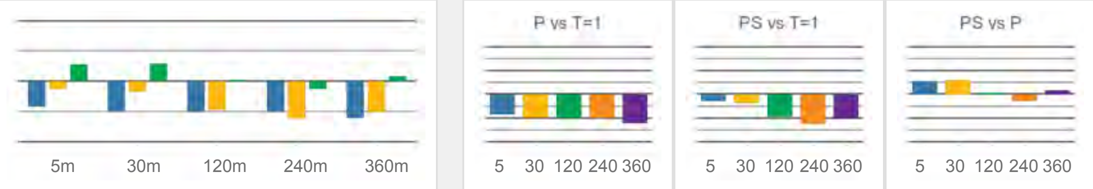

Locus		5m	30m	120m	240m	360m
**PGN_1171**	P vs T=1	0.462	0.797	0.791	0.544	0.608
	PS vs T=1	1.379	1.533	0.947	0.671	0.597
	PS vs P	0.883	0.730	0.162	0.094	-0.015
	coenzyme A transferase beta subunit					
	central intermediary metabolism					

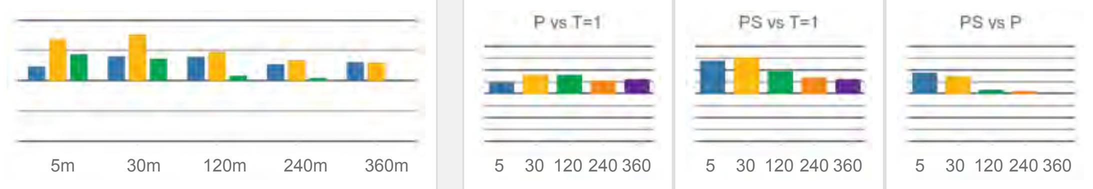

Locus		5m	30m	120m	240m	360m
**PGN_1172**	P vs T=1	0.615	1.035	0.731	-0.164	-0.838
	PS vs T=1	1.607	1.907	1.357	0.544	-0.101
	PS vs P	0.952	0.886	0.639	0.627	0.640
	acyl-CoA dehydrogenase short-chain specific					
	fatty acid and phospholipid metabolism					

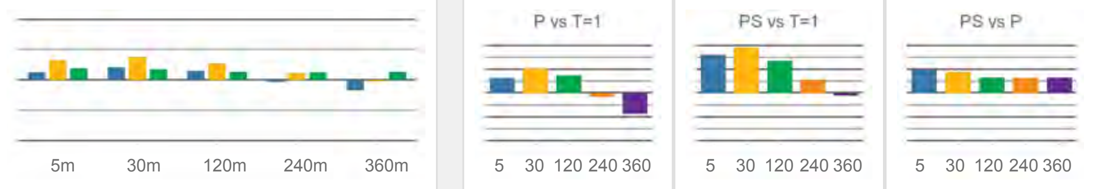

Locus		5m	30m	120m	240m	360m
**PGN_1173**	P vs T=1	1.057	1.529	1.327	0.549	-0.337
	PS vs T=1	1.932	2.241	2.097	1.262	0.773
	PS vs P	0.836	0.732	0.790	0.656	1.012
	putative electron transfer flavoprotein beta subunit					
	energy metabolism					

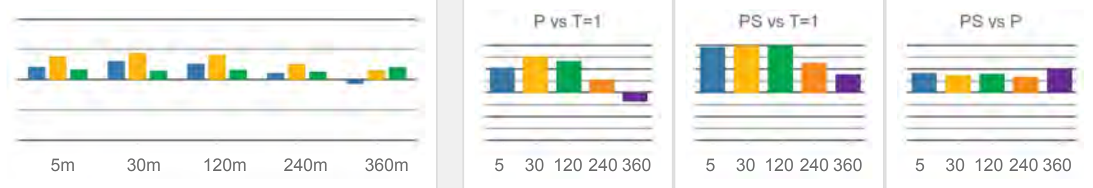

Locus		5m	30m	120m	240m	360m
**PGN_1174**	P vs T=1	0.313	0.889	1.082	0.751	0.003
	PS vs T=1	0.979	1.326	1.322	1.058	0.997
	PS vs P	0.588	0.439	0.268	0.295	0.927
	electron transfer flavoprotein alpha subunit					
	energy metabolism					

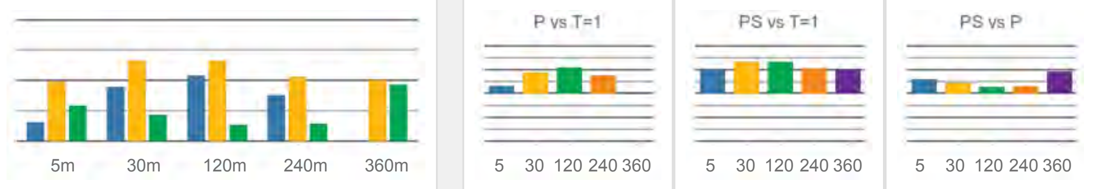

Locus		5m	30m	120m	240m	360m
**PGN_1175**	P vs T=1	0.999	1.540	1.358	0.433	-0.041
	PS vs T=1	1.829	1.885	1.384	0.752	0.443
	PS vs P	0.796	0.383	0.072	0.214	0.373
	putative enoyl-CoA hydratase					
	fatty acid and phospholipid metabolism					

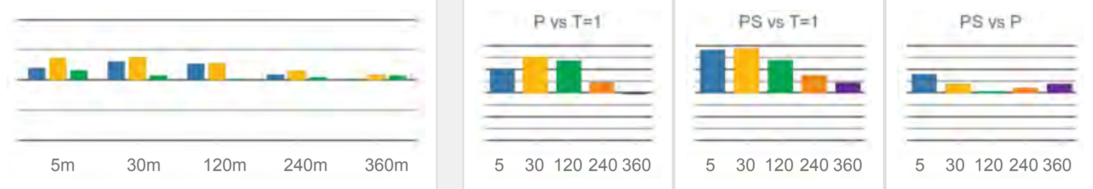

Locus		5m	30m	120m	240m	360m
**PGN_1176**	P vs T=1	0.447	0.883	0.723	0.150	-0.534
	PS vs T=1	0.898	1.075	0.566	0.043	-0.095
	PS vs P	0.434	0.224	-0.123	-0.133	0.359
	putative 3-hydroxybutyryl-CoA dehydrogenase					
	fatty acid and phospholipid metabolism					

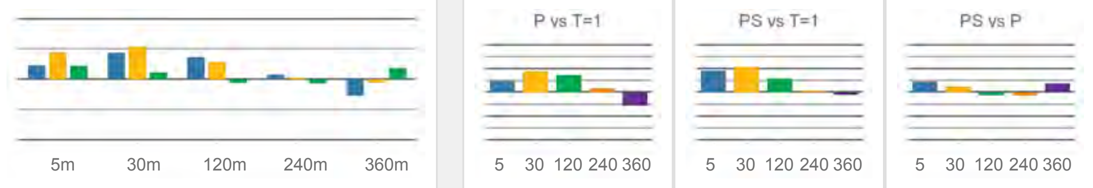

log₂ (Fold Change)

Legend: P vs T=1 | PS vs T=1 | PS vs P

Locus		5m	30m	120m	240m	360m
**PGN_1177**	P vs T=1	0.293	0.233	0.252	0.349	0.100
	PS vs T=1	0.176	-0.059	0.069	0.157	0.211
	PS vs P	-0.117	-0.296	-0.190	-0.193	0.097
	transposase in ISPg1					

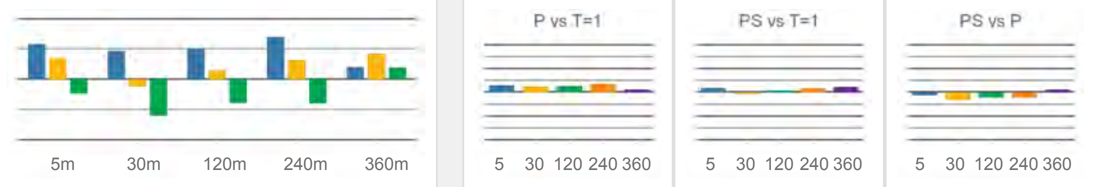

Locus		5m	30m	120m	240m	360m
**PGN_1178**	P vs T=1	1.514	1.797	1.814	1.274	0.556
	PS vs T=1	1.561	1.800	1.658	1.199	1.039
	PS vs P	0.055	0.031	-0.122	-0.076	0.453
	acetate kinase					
	energy metabolism					

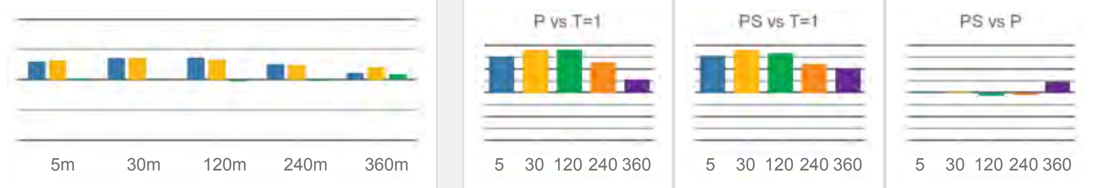

Locus		5m	30m	120m	240m	360m
**PGN_1179**	P vs T=1	0.606	0.438	-0.374	-1.185	-1.542
	PS vs T=1	0.213	0.427	0.260	-0.033	-0.319
	PS vs P	-0.349	0.024	0.624	1.059	1.148
	phosphotransacetylase					
	energy metabolism					

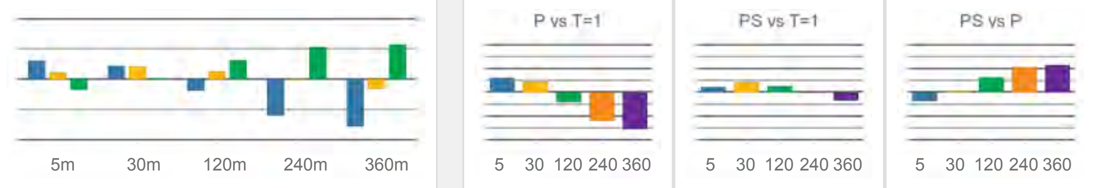

Locus		5m	30m	120m	240m	360m
**PGN_1180**	P vs T=1	0.093	-0.407	-0.951	-1.401	-1.407
	PS vs T=1	-0.053	-0.035	0.198	0.123	-0.431
	PS vs P	-0.062	0.357	1.061	1.330	0.864
	conserved hypothetical protein					
	hypothetical proteins-Conserved					

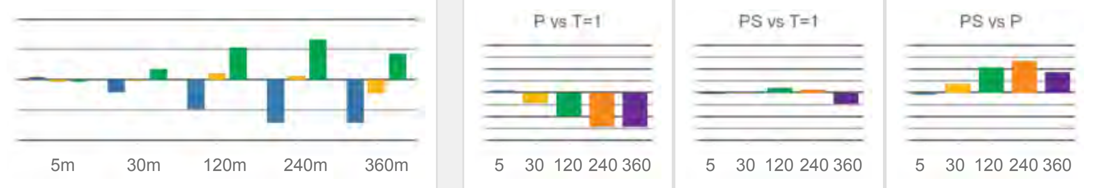

Locus		5m	30m	120m	240m	360m
**PGN_1181**	P vs T=1	-0.473	-0.762	-1.385	-2.201	-2.917
	PS vs T=1	-1.560	-1.180	-1.396	-1.728	-2.057
	PS vs P	-0.905	-0.296	0.026	0.371	0.705
	probable thiol:disulfide oxidoreductase					
	energy metabolism					

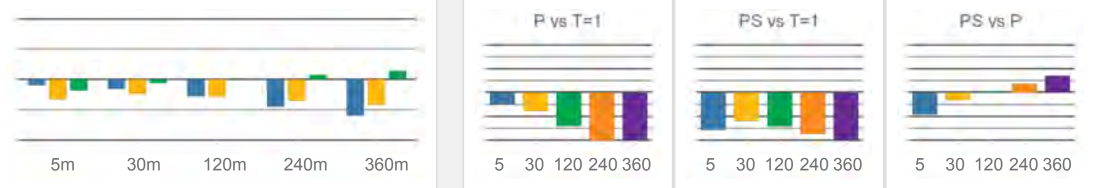

Locus		5m	30m	120m	240m	360m
**PGN_1182**	P vs T=1	-0.901	-0.374	-0.162	-0.511	-1.365
	PS vs T=1	-1.140	-0.660	-0.659	-0.819	-0.687
	PS vs P	-0.321	-0.221	-0.394	-0.290	0.533
	conserved hypothetical protein					
	hypothetical proteins-Conserved					

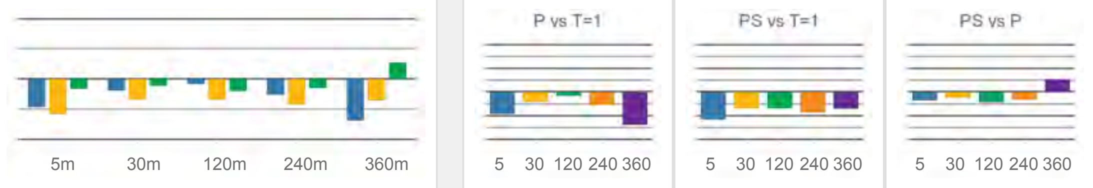

Locus		5m	30m	120m	240m	360m
**PGN_1183**	P vs T=1	-0.419	-1.288	-1.092	-1.403	0.028
	PS vs T=1	-1.486	-1.782	-1.959	-1.585	-0.404
	PS vs P	-0.850	-0.577	-0.824	-0.508	-0.088
	hypothetical protein					
	hypothetical proteins					

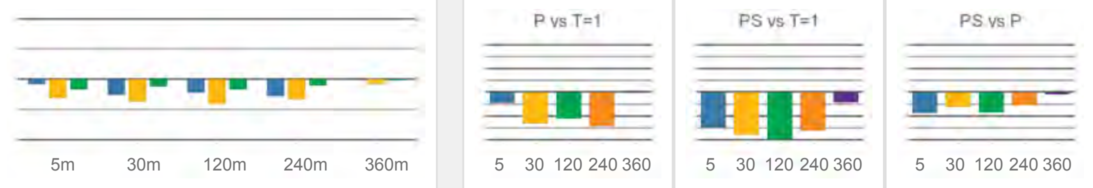

PgSg Time Course

log$_2$ (Fold Change)

Legend: P vs T=1 · PS vs T=1 · PS vs P

Locus		5m	30m	120m	240m	360m
**PGN_1184**	P vs T=1	0.409	-0.061	-0.374	-0.281	-0.155
	PS vs T=1	0.493	0.186	0.520	0.614	0.405
	PS vs P	0.100	0.228	0.840	0.846	0.540

conserved hypothetical protein

hypothetical proteins-Conserved

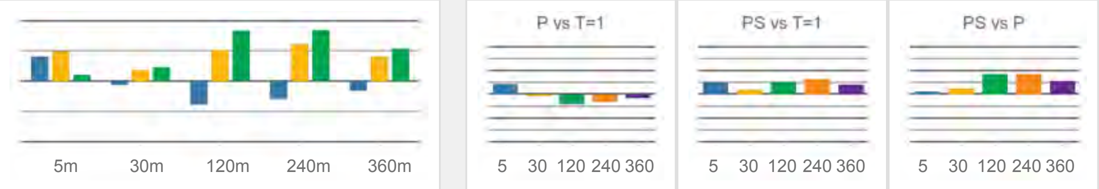

Locus		5m	30m	120m	240m	360m
**PGN_1185**	P vs T=1	-0.584	-0.733	-0.811	-0.648	-0.517
	PS vs T=1	-0.468	-0.214	0.523	0.311	0.083
	PS vs P	0.105	0.490	1.281	0.920	0.587

conserved hypothetical protein

hypothetical proteins-Conserved

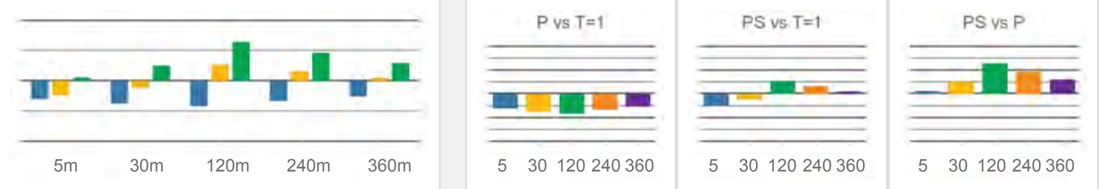

Locus		5m	30m	120m	240m	360m
**PGN_1186** *rprY*	P vs T=1	0.176	0.567	0.951	0.835	0.154
	PS vs T=1	-0.673	0.182	1.018	0.939	0.880
	PS vs P	-0.852	-0.366	0.097	0.123	0.709

putative DNA-binding response regulator RprY

signal transduction

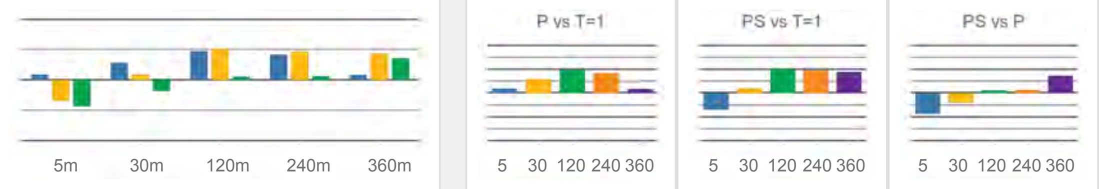

Locus		5m	30m	120m	240m	360m
**PGN_1187**	P vs T=1	0.012	-0.084	-0.378	-0.532	-0.266
	PS vs T=1	-0.225	-0.214	0.189	0.351	0.139
	PS vs P	-0.223	-0.123	0.534	0.812	0.392

conserved hypothetical protein

unknown function

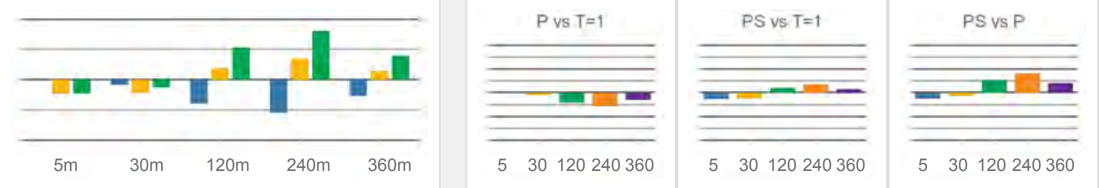

Locus		5m	30m	120m	240m	360m
**PGN_1188**	P vs T=1	0.286	0.945	1.445	1.083	0.371
	PS vs T=1	0.611	1.162	1.497	1.241	0.865
	PS vs P	0.265	0.228	0.113	0.177	0.450

conserved hypothetical protein

hypothetical proteins-Conserved

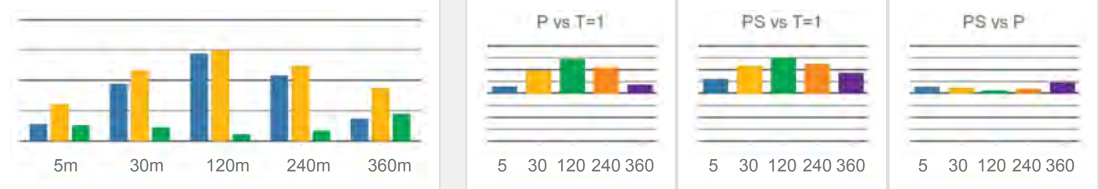

Locus		5m	30m	120m	240m	360m
**PGN_1189**	P vs T=1	1.254	1.944	2.578	2.424	1.581
	PS vs T=1	1.704	2.155	2.452	2.522	2.332
	PS vs P	0.388	0.221	-0.063	0.138	0.723

phosphoglucomutase/phosphomannomutase

energy metabolism

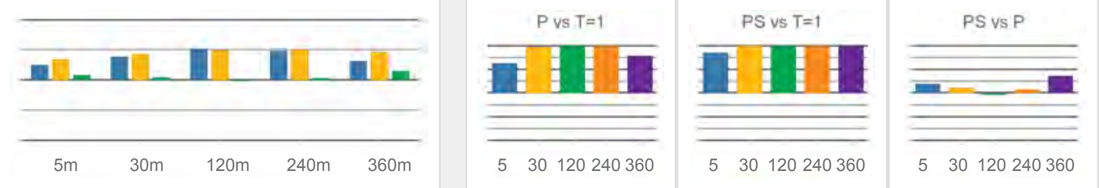

Locus		5m	30m	120m	240m	360m
**PGN_1190**	P vs T=1	0.247	0.611	1.613	1.820	1.535
	PS vs T=1	0.437	0.558	1.104	1.462	1.693
	PS vs P	0.120	-0.086	-0.438	-0.274	0.182

RNA methyltransferase

protein synthesis

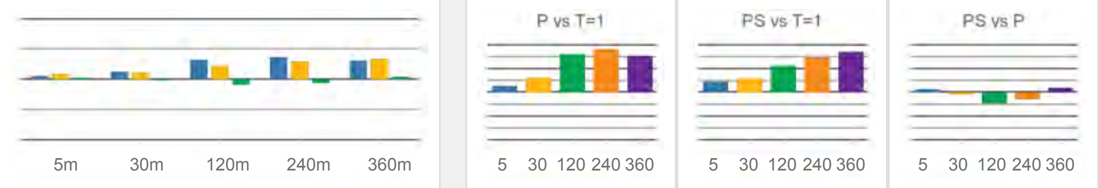

PgSg Time Course

Hackett Laboratory UW

log2 (Fold Change)

Locus		5m	30m	120m	240m	360m
PGN_1191	P vs T=1	0.364	0.296	0.315	0.475	0.078
	PS vs T=1	0.185	-0.005	0.089	0.189	0.230
	PS vs P	-0.177	-0.304	-0.233	-0.279	0.133
	transposase in ISPg1					
PGN_1192	P vs T=1	0.623	0.884	1.543	2.270	3.220
	PS vs T=1	-0.045	-0.426	0.390	0.980	1.535
	PS vs P	-0.665	-1.108	-0.936	-0.771	-1.159
	DNA-binding protein histone-like family					
	DNA metabolism					
PGN_1193	P vs T=1	0.149	-0.284	-0.701	-0.569	-0.170
	PS vs T=1	0.206	0.468	1.149	1.263	0.790
	PS vs P	0.060	0.691	1.702	1.687	0.923
	conserved hypothetical protein					
	hypothetical proteins-Conserved					
PGN_1194	P vs T=1	-0.988	-1.653	-2.169	-1.715	-0.989
	PS vs T=1	-0.500	-0.886	-0.543	-0.400	-0.612
	PS vs P	0.487	0.672	1.420	1.197	0.381
	alanine racemase					
	unknown function					
PGN_1195	P vs T=1	0.007	-0.355	-0.425	0.080	0.971
	PS vs T=1	-0.750	-0.996	-0.919	-0.444	-0.124
	PS vs P	-0.705	-0.624	-0.520	-0.434	-0.964
	conserved hypothetical protein					
	hypothetical proteins-Conserved					
PGN_1196	P vs T=1	-0.216	-0.056	-0.297	-0.215	0.514
	PS vs T=1	0.207	0.382	-0.076	-0.521	-0.434
	PS vs P	0.378	0.435	0.166	-0.318	-0.788
	putative sugar phosphate isomerase					
	energy metabolism					
PGN_1197	P vs T=1	-0.788	-0.422	-0.256	-0.238	0.423
	PS vs T=1	-0.716	-0.541	-1.026	-0.503	-0.662
	PS vs P	0.024	-0.108	-0.719	-0.230	-0.950
	conserved hypothetical protein					
	hypothetical proteins-Conserved					

P vs T=1 | PS vs T=1 | PS vs P

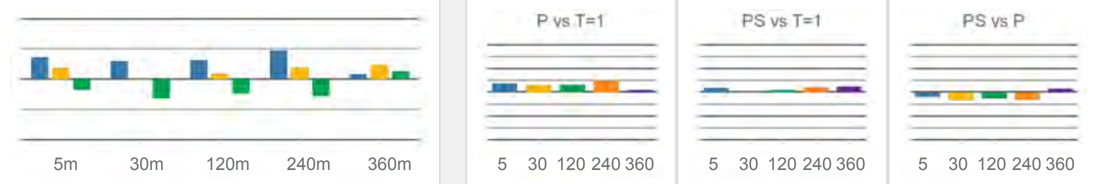

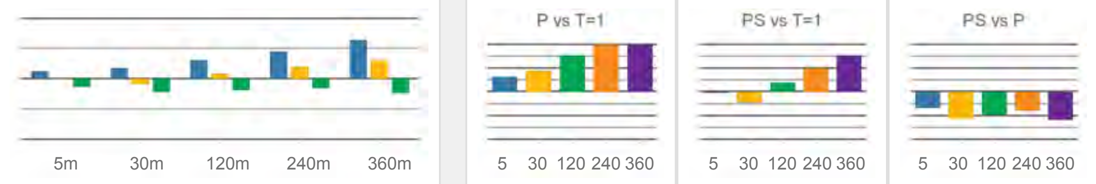

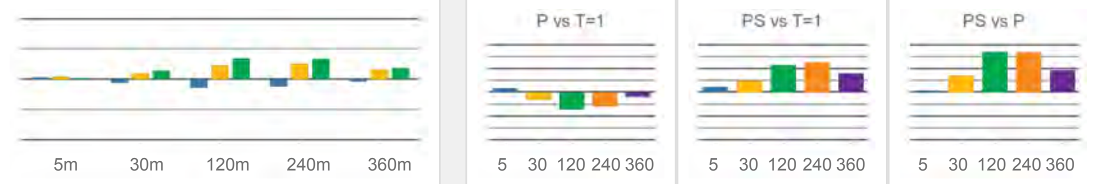

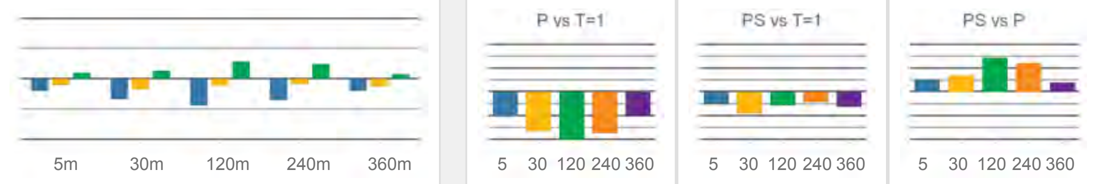

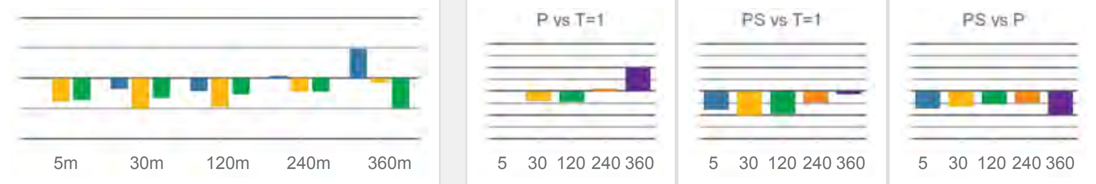

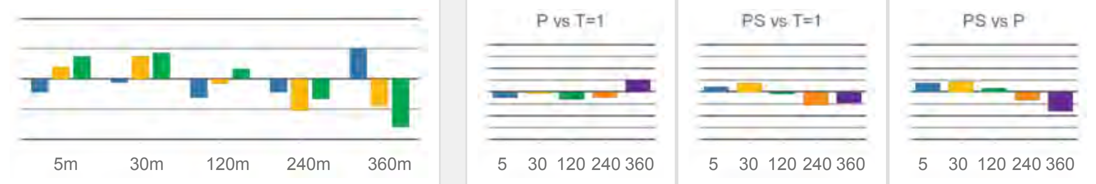

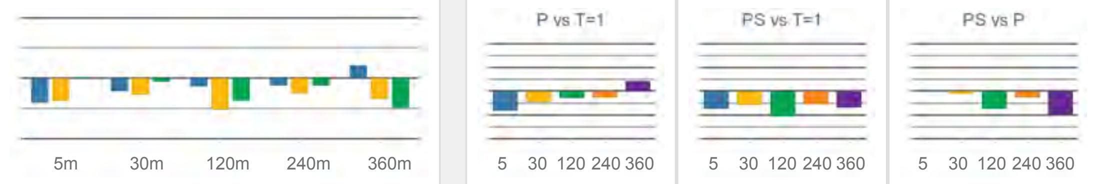

Legend: P vs T=1 | PS vs T=1 | PS vs P

log2 (Fold Change)

Locus		5m	30m	120m	240m	360m
**PGN_1198**	P vs T=1	-0.483	-0.806	-1.096	-0.498	0.263
	PS vs T=1	-0.653	-1.003	-1.001	-0.435	-0.428
	PS vs P	-0.146	-0.211	-0.019	0.095	-0.593

putative sodium-solute transporter

transport and binding proteins

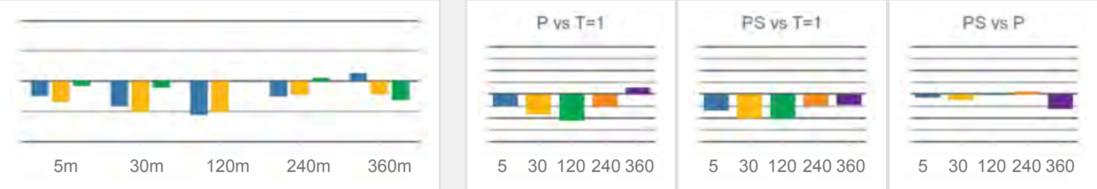

Locus		5m	30m	120m	240m	360m
**PGN_1199**	P vs T=1	-1.263	-0.966	-0.560	-0.626	0.109
	PS vs T=1	-0.914	-0.743	-0.489	-0.202	0.006
	PS vs P	0.177	0.132	0.094	0.336	0.036

DNA-binding protein histone-like family

DNA metabolism

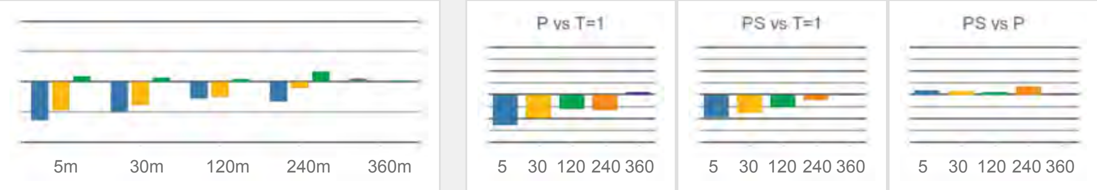

Locus		5m	30m	120m	240m	360m
**PGN_1200**	P vs T=1	-0.347	-0.184	-0.425	0.162	0.530
	PS vs T=1	0.048	-0.142	-0.224	-0.012	0.278
	PS vs P	0.364	0.034	0.125	-0.140	-0.212

ATPase AAA family

unknown function

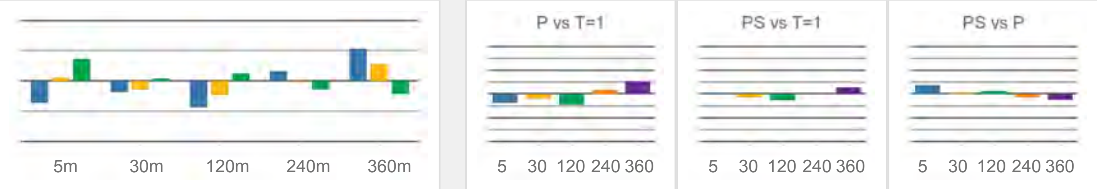

Locus		5m	30m	120m	240m	360m
**PGN_1201**	P vs T=1	0.049	0.467	0.651	0.632	0.629
	PS vs T=1	-0.411	-0.273	-0.321	0.037	0.004
	PS vs P	-0.464	-0.665	-0.879	-0.507	-0.563

conserved hypothetical protein

hypothetical proteins-Conserved

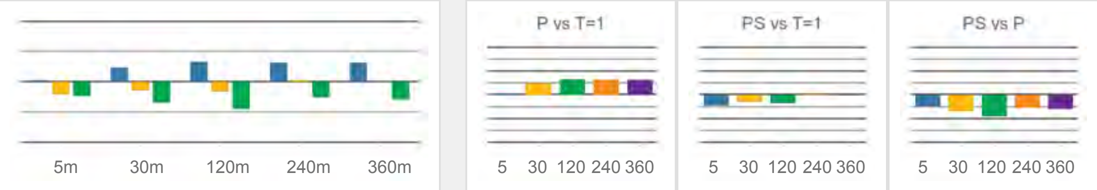

Locus		5m	30m	120m	240m	360m
**PGN_1202**	P vs T=1	1.008	1.220	0.767	0.274	-0.028
rpoN	PS vs T=1	1.084	0.801	0.356	0.572	0.195
	PS vs P	0.098	-0.367	-0.391	0.270	0.192

RNA polymerase sigma-54 factor

transcription

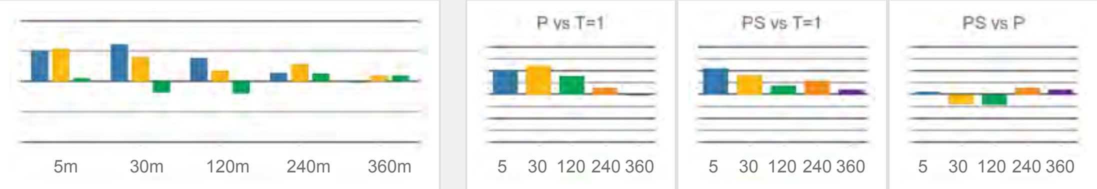

Locus		5m	30m	120m	240m	360m
**PGN_1203**	P vs T=1	0.511	0.727	0.743	0.454	0.031
	PS vs T=1	0.908	0.760	0.757	1.214	0.919
	PS vs P	0.384	0.042	0.026	0.738	0.849

UDP-N-acetylmuramoylalanyl-D-glutamyl-2, 6-diaminopimelate--D-alanyl-D-alanyl ligase

cell envelope

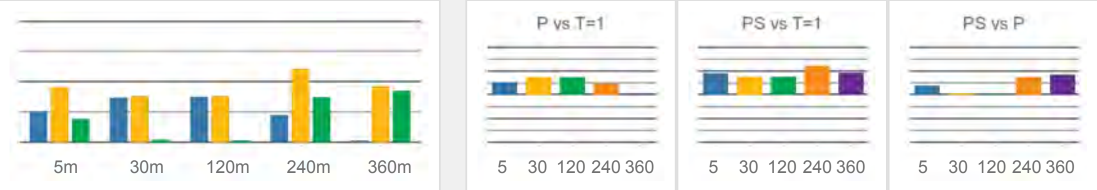

Locus		5m	30m	120m	240m	360m
**PGN_1204**	P vs T=1	-0.356	-1.102	-0.710	-0.618	-0.895
	PS vs T=1	-0.778	-0.386	0.841	1.571	1.397
	PS vs P	-0.410	0.587	1.465	2.079	2.174

probable aspartate-1-decarboxylase

biosynthesis of cofactors, prosthetic groups, and carriers

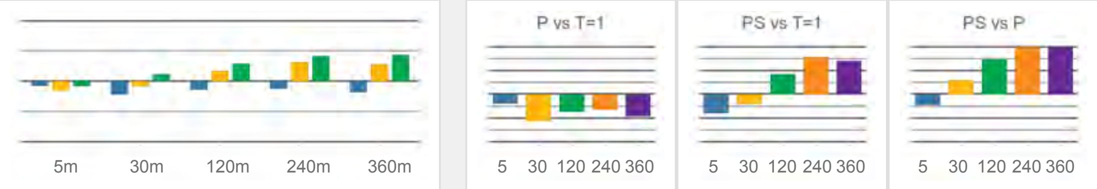

PgSg Time Course

log2 (Fold Change)

Legend: P vs T=1 | PS vs T=1 | PS vs P

Locus		5m	30m	120m	240m	360m
**PGN_1205**	P vs T=1	-0.701	-0.760	-0.921	-0.942	-0.962
	PS vs T=1	-0.644	-0.554	-0.323	0.037	-0.116
	PS vs P	0.051	0.197	0.573	0.940	0.822

signal recognition particle protein

protein fate

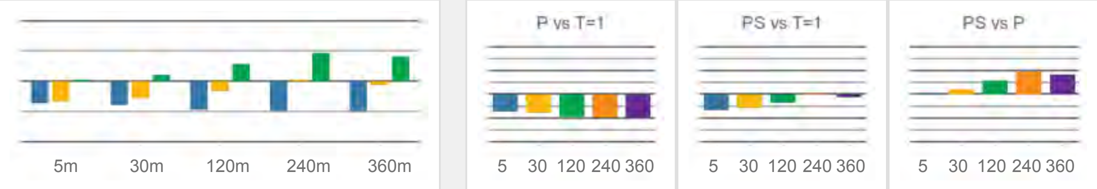

Locus		5m	30m	120m	240m	360m
**PGN_1206**	P vs T=1	1.138	1.872	2.517	2.647	2.387
	PS vs T=1	1.133	1.793	2.730	3.188	2.887
	PS vs P	-0.069	-0.077	0.245	0.569	0.506

putative methylenetetrahydrofolate dehydrogenase/methenyltetrahydrofolate cyclohydrolase

biosynthesis of cofactors, prosthetic groups, and carriers

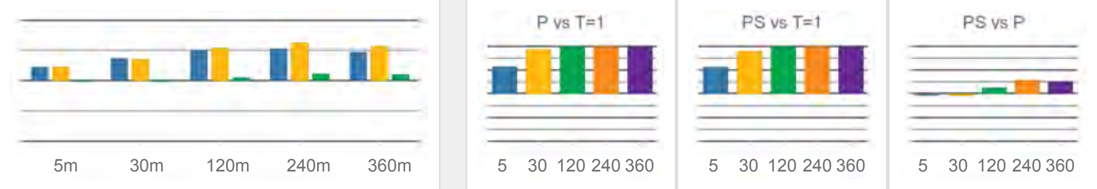

Locus		5m	30m	120m	240m	360m
**PGN_1207**	P vs T=1	-0.283	-0.193	0.205	0.717	1.419
	PS vs T=1	-0.335	-0.455	0.033	0.527	0.459
	PS vs P	-0.089	-0.292	-0.176	-0.091	-0.830

putative transport multidrug efflux protein

transport and binding proteins

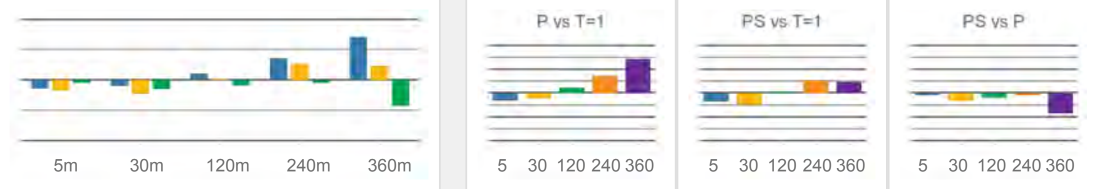

Locus		5m	30m	120m	240m	360m
**PGN_1208**	P vs T=1	1.297	2.042	3.206	3.524	3.074
clpB	PS vs T=1	-0.781	-0.423	0.001	0.431	0.637
	PS vs P	-2.015	-2.314	-2.996	-2.867	-2.314

ClpB protein

protein fate

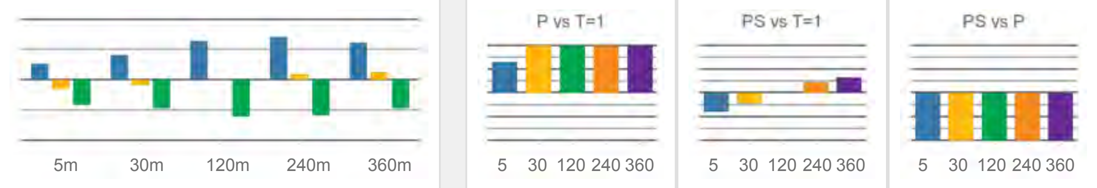

Locus		5m	30m	120m	240m	360m
**PGN_1209**	P vs T=1	0.442	0.953	0.713	0.207	-0.460
	PS vs T=1	0.023	0.564	1.329	1.863	1.518
	PS vs P	-0.431	-0.281	0.633	1.530	1.786

probable flavodoxin

energy metabolism

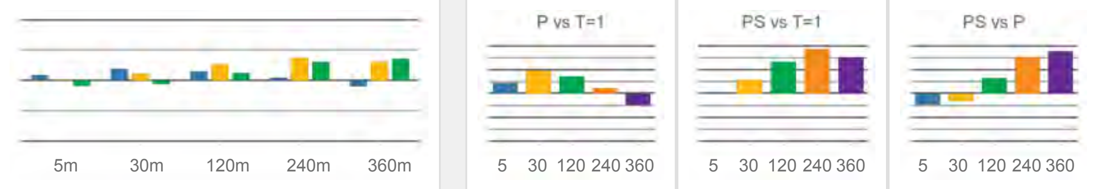

Locus		5m	30m	120m	240m	360m
**PGN_1210**	P vs T=1	-1.123	-2.206	-2.671	-2.176	-1.486
	PS vs T=1	-0.979	-0.979	0.008	0.131	-0.183
	PS vs P	0.173	0.912	2.185	1.985	1.230

hypothetical protein

hypothetical proteins

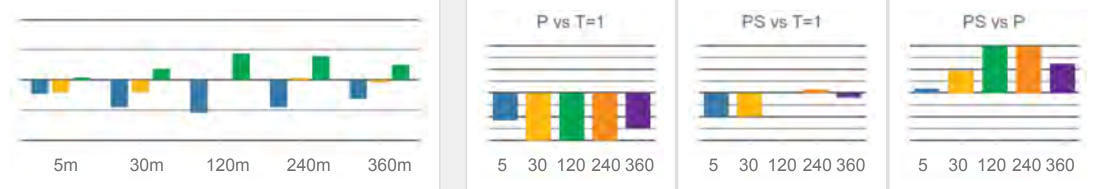

Locus		5m	30m	120m	240m	360m
**PGN_1211**	P vs T=1	-0.211	-0.768	-1.341	-1.300	-0.855
	PS vs T=1	0.125	0.326	0.651	0.357	-0.128
	PS vs P	0.354	1.002	1.800	1.469	0.684

hypothetical protein

hypothetical proteins

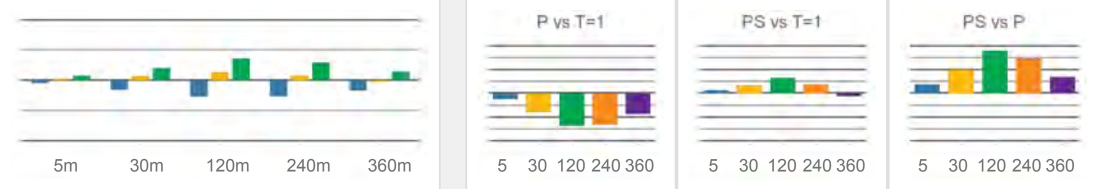

log2 (Fold Change)

P vs T=1 | PS vs T=1 | PS vs P

Locus		5m	30m	120m	240m	360m
**PGN_1212**	P vs T=1	-0.812	-1.229	-1.412	-1.459	-0.942
	PS vs T=1	-0.486	-0.344	0.047	-0.093	-0.241
	PS vs P	0.314	0.792	1.330	1.189	0.680
	hypothetical protein					
	hypothetical proteins					
**PGN_1213**	P vs T=1	-0.586	-1.504	-1.909	-1.732	-0.970
	PS vs T=1	-0.105	-0.432	-0.489	-0.851	-1.134
	PS vs P	0.510	0.985	1.298	0.789	-0.151
	putative ATP-binding protein					
	transport and binding proteins					
**PGN_1214**	P vs T=1	-0.653	-1.260	-1.748	-1.680	-0.847
	PS vs T=1	0.019	-0.180	-0.356	-0.817	-1.232
	PS vs P	0.677	0.971	1.194	0.671	-0.355
	hypothetical protein					
	hypothetical proteins					
**PGN_1215**	P vs T=1	-0.801	-1.395	-1.885	-1.786	-1.000
	PS vs T=1	-0.179	-0.308	-0.739	-1.045	-1.143
	PS vs P	0.634	0.939	0.861	0.479	-0.101
	hypothetical protein					
	hypothetical proteins					
**PGN_1216**	P vs T=1	0.338	0.241	0.319	0.478	0.147
	PS vs T=1	0.184	0.040	0.179	0.278	0.250
	PS vs P	-0.154	-0.212	-0.151	-0.195	0.083
	transposase in ISPg1					
**PGN_1217**	P vs T=1	-0.587	-0.331	0.090	0.411	0.247
	PS vs T=1	-0.188	-0.189	0.374	0.100	0.462
	PS vs P	0.290	0.076	0.275	-0.224	0.214
	hypothetical protein					
	hypothetical proteins					
**PGN_1218**	P vs T=1	0.109	0.186	0.104	-0.001	-0.435
	PS vs T=1	0.917	0.902	0.811	0.467	0.222
	PS vs P	0.792	0.705	0.699	0.457	0.635
	asparaginyl-tRNA synthetase					
	protein synthesis					

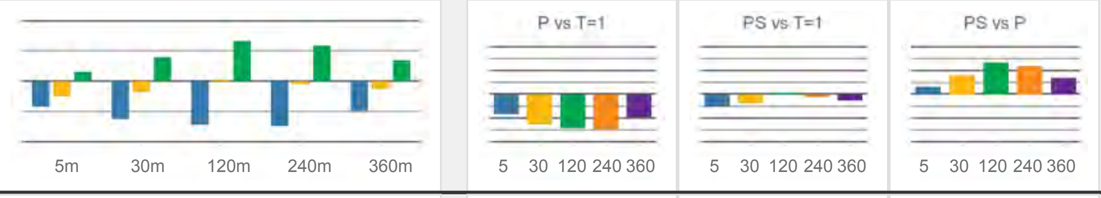

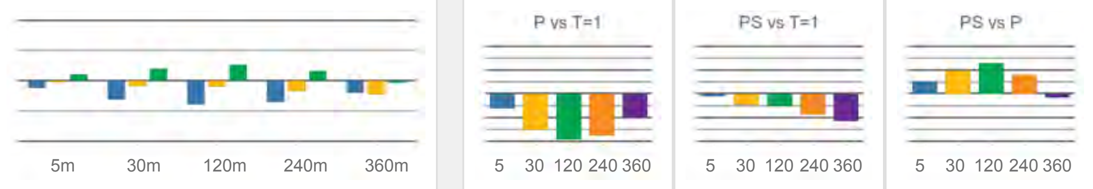

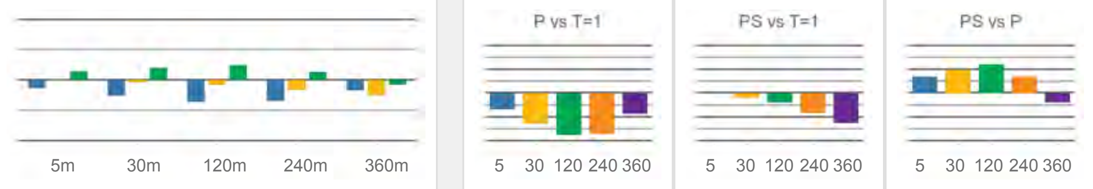

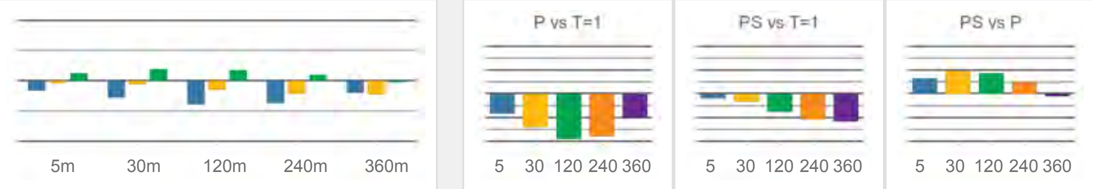

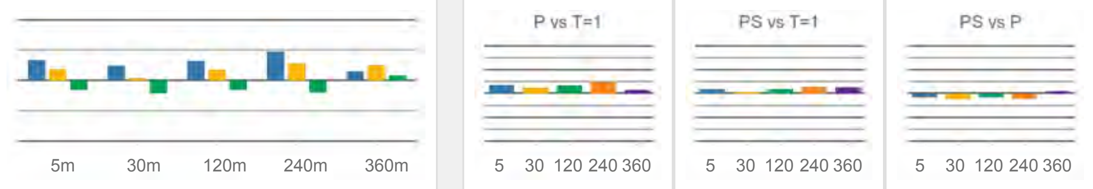

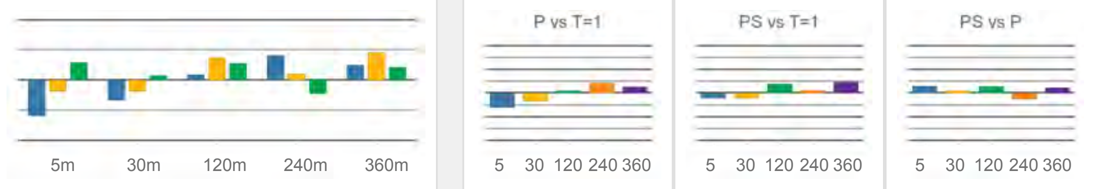

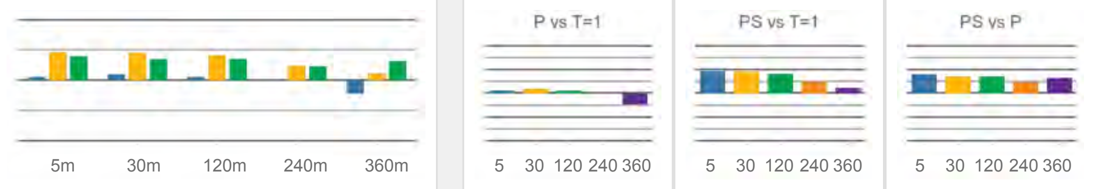

log$_2$ (Fold Change)

Legend: P vs T=1, PS vs T=1, PS vs P

Locus		5m	30m	120m	240m	360m
**PGN_1219**	P vs T=1	-0.717	-0.841	-1.288	-1.514	-1.766
	PS vs T=1	-0.226	-0.312	-0.114	-0.548	-1.140
	PS vs P	0.488	0.521	1.141	0.921	0.585

ribosomal large subunit pseudouridine synthase B

protein synthesis

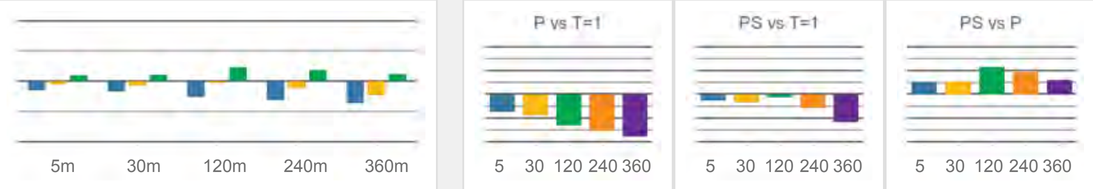

Locus		5m	30m	120m	240m	360m
**PGN_1220**	P vs T=1	-1.016	-1.194	-1.522	-1.754	-2.135
	PS vs T=1	-1.454	-1.269	-0.518	-0.690	-1.010
	PS vs P	-0.412	-0.070	0.973	1.010	1.061

adenylosuccinate lyase

purines, pyrimidines, nucleosides and nucleotides

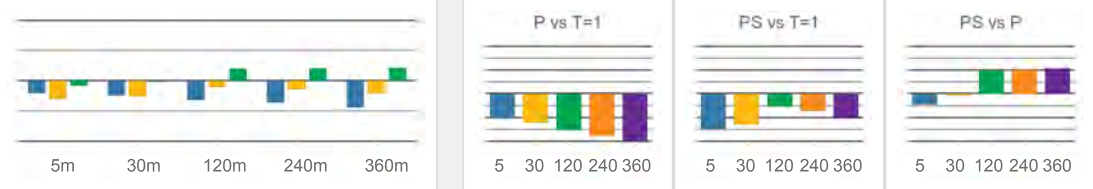

Locus		5m	30m	120m	240m	360m
**PGN_1221**	P vs T=1	-0.177	0.527	0.702	0.765	0.308
	PS vs T=1	-1.151	0.154	1.211	1.136	0.870
	PS vs P	-1.006	-0.344	0.523	0.388	0.544

probable ATP:corrinoid adenosyltransferase

unknown function

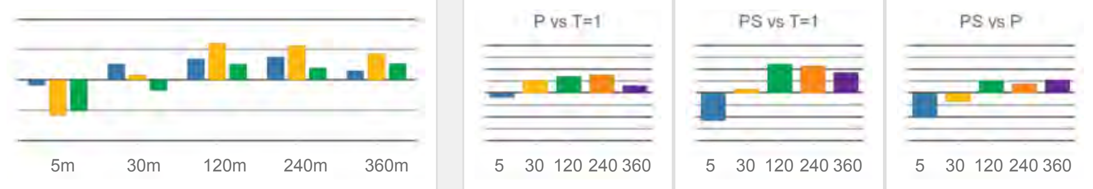

Locus		5m	30m	120m	240m	360m
**PGN_1222**	P vs T=1	0.403	0.100	-0.091	0.751	1.297
	PS vs T=1	0.947	0.147	-0.671	-0.511	0.226
	PS vs P	0.574	0.035	-0.656	-0.985	-0.848

conserved hypothetical protein

hypothetical proteins-Conserved

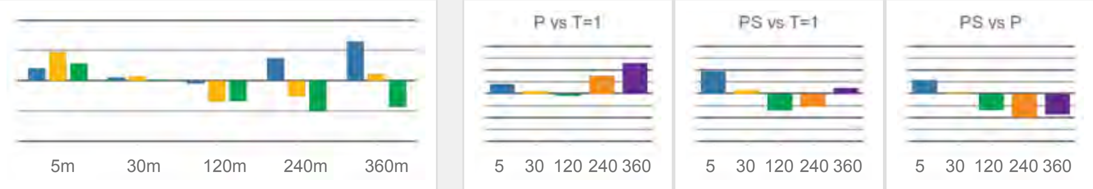

Locus		5m	30m	120m	240m	360m
**PGN_1223**	P vs T=1	0.328	0.049	0.294	0.949	1.789
	PS vs T=1	0.159	-0.775	-0.506	0.040	0.603
	PS vs P	-0.125	-0.803	-0.773	-0.733	-1.004

uracil permease

transport and binding proteins

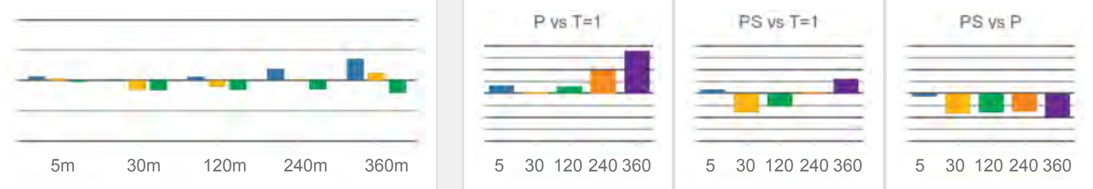

Locus		5m	30m	120m	240m	360m
**PGN_1224**	P vs T=1	-0.330	-0.568	-0.847	-1.177	-1.408
	PS vs T=1	-0.976	-0.713	0.111	0.498	0.389
	PS vs P	-0.625	-0.145	0.927	1.580	1.720

probable transcriptional regulator

regulatory functions

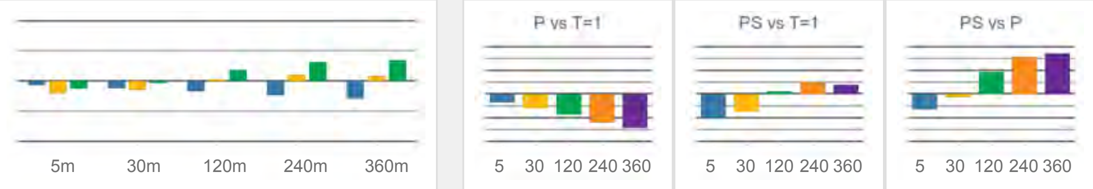

Locus		5m	30m	120m	240m	360m
**PGN_1225**	P vs T=1	-1.038	-1.129	-0.818	-0.503	0.084
	PS vs T=1	-0.982	-1.270	-0.742	0.214	0.138
	PS vs P	0.028	-0.186	0.042	0.709	0.094

probable exodeoxyribonuclease VII large subunit

DNA metabolism

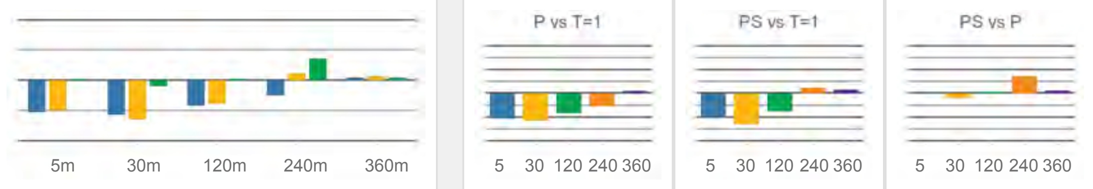

PgSg Time Course

Legend: P vs T=1 | PS vs T=1 | PS vs P

Locus		log$_2$ (Fold Change) 5m	30m	120m	240m	360m
**PGN_1226**	P vs T=1	0.136	0.759	1.896	2.098	1.530
	PS vs T=1	0.610	0.710	1.145	1.219	1.185
	PS vs P	0.288	-0.094	-0.596	-0.711	-0.309
	ribonucleotide reductase					
	purines, pyrimidines, nucleosides and nucleotides					
**PGN_1227**	P vs T=1	-0.719	-1.437	-1.709	-1.777	-1.022
	PS vs T=1	-0.807	-0.377	0.785	1.049	0.700
	PS vs P	-0.077	0.847	2.221	2.493	1.645
	TPR domain protein					
	unknown function					
**PGN_1228**	P vs T=1	0.072	-0.510	0.195	1.325	3.987
	PS vs T=1	-0.714	-0.177	-0.681	2.480	-0.378
	PS vs P	-0.424	-0.139	-0.476	1.034	-0.481
	hypothetical protein					
	hypothetical proteins					
**PGN_1229**	P vs T=1	-0.455	-0.697	-0.851	-0.606	-0.641
	PS vs T=1	-0.595	-1.009	-1.055	-0.763	-0.645
	PS vs P	-0.125	-0.309	-0.213	-0.144	-0.001
	valyl-tRNA synthetase					
	protein synthesis					
**PGN_1230**	P vs T=1	-1.182	-1.057	-1.026	-1.324	-2.111
	PS vs T=1	-1.546	-1.614	-1.975	-2.205	-2.186
	PS vs P	-0.345	-0.520	-0.897	-0.843	-0.121
	conserved hypothetical protein					
	hypothetical proteins-Conserved					
**PGN_1231**	P vs T=1	-1.409	-1.491	-1.043	-1.150	-0.505
	PS vs T=1	-2.417	-2.573	-2.155	-1.935	-2.298
	PS vs P	-0.899	-0.961	-0.821	-0.568	-1.335
	hypothetical protein					
	hypothetical proteins					
**PGN_1232**	P vs T=1	0.945	1.812	2.031	1.951	1.584
	PS vs T=1	0.255	1.711	3.101	3.376	2.996
	PS vs P	-0.722	-0.084	1.078	1.419	1.397
	thioredoxin reductase					
	energy metabolism					

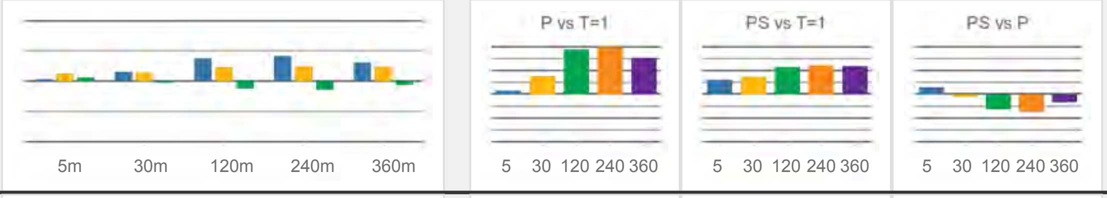

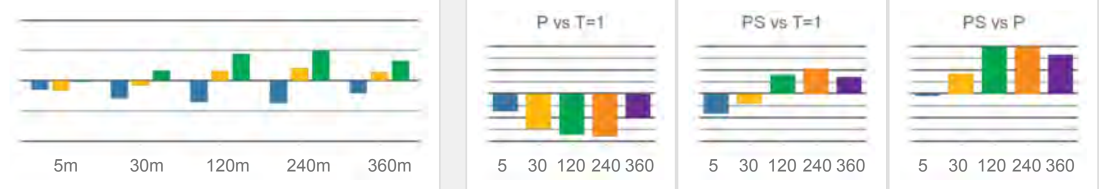

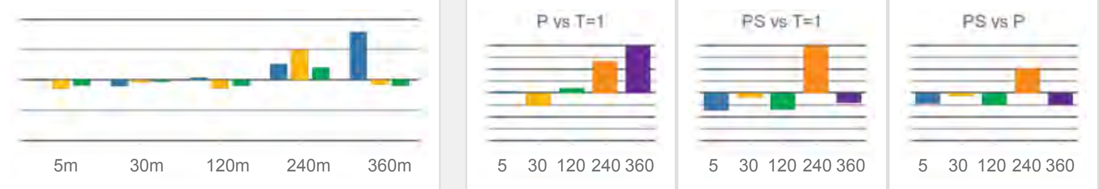

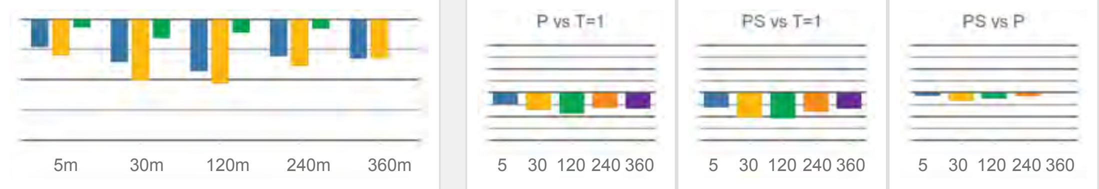

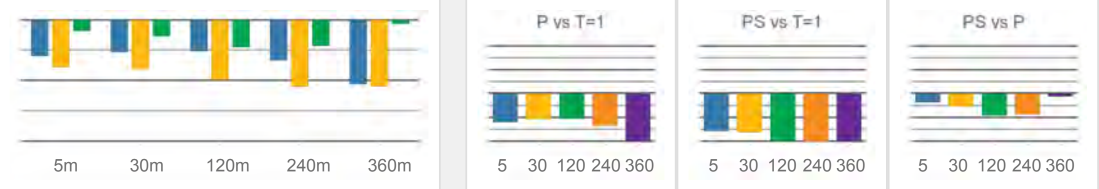

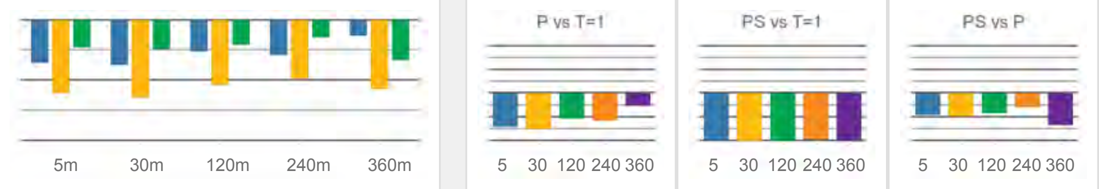

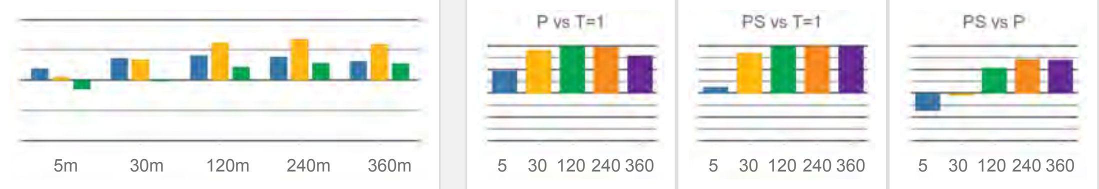

log2 (Fold Change)

Legend: P vs T=1 | PS vs T=1 | PS vs P

Locus		5m	30m	120m	240m	360m
**PGN_1233**	P vs T=1	-0.500	-1.599	-2.042	-1.656	-1.327
	PS vs T=1	-1.780	-2.015	-0.857	-0.807	-1.103
	PS vs P	-1.117	-0.454	1.002	0.781	0.228

conserved hypothetical protein

cell envelope

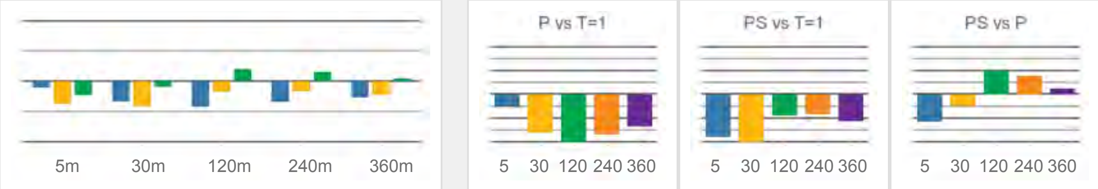

Locus		5m	30m	120m	240m	360m
**PGN_1234**	P vs T=1	-1.038	-2.069	-1.932	-1.473	-0.089
	PS vs T=1	-0.990	-1.657	-1.500	-1.527	-1.180
	PS vs P	0.106	0.256	0.303	-0.076	-0.932

conserved hypothetical protein

hypothetical proteins-Conserved

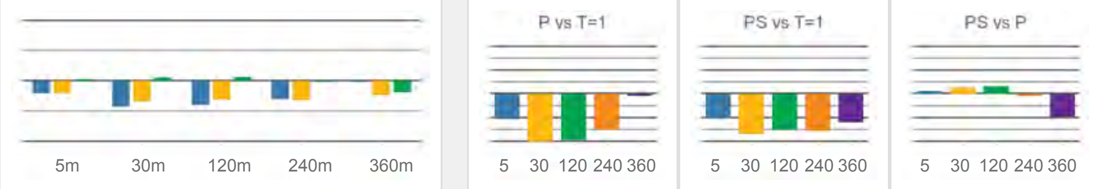

Locus		5m	30m	120m	240m	360m
**PGN_1235** *porS*	P vs T=1	-0.995	-1.622	-1.726	-0.811	-0.191
	PS vs T=1	-0.923	-1.198	-0.921	-0.787	-0.602
	PS vs P	0.080	0.316	0.595	0.083	-0.326

membrane protein PorS

cell envelope

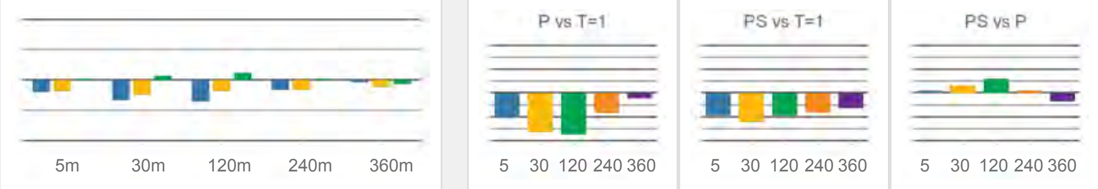

Locus		5m	30m	120m	240m	360m
**PGN_1236** *porR*	P vs T=1	-1.540	-2.619	-2.651	-1.907	-0.957
	PS vs T=1	-1.625	-2.118	-1.985	-2.067	-1.836
	PS vs P	-0.009	0.298	0.430	-0.130	-0.720

regulator PorR

regulatory functions

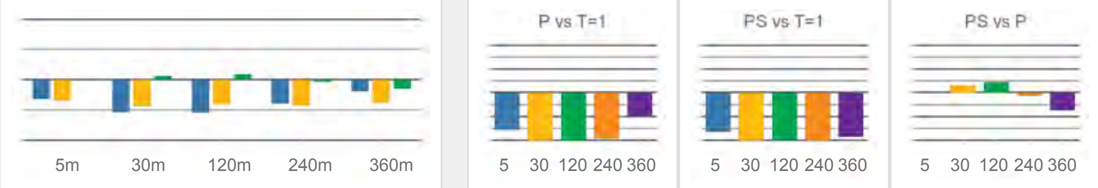

Locus		5m	30m	120m	240m	360m
**PGN_1237**	P vs T=1	0.461	0.126	0.931	0.460	1.901
	PS vs T=1	-0.075	0.336	0.455	0.722	0.486
	PS vs P	-0.480	-0.017	-0.259	-0.009	-0.782

hypothetical protein

hypothetical proteins

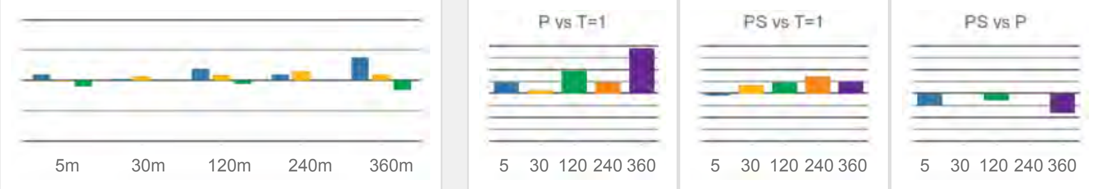

Locus		5m	30m	120m	240m	360m
**PGN_1238**	P vs T=1	-0.350	-1.090	-1.505	-1.471	-1.459
	PS vs T=1	-0.168	-0.036	1.012	1.188	0.811
	PS vs P	0.201	0.928	2.316	2.449	2.136

conserved hypothetical protein

hypothetical proteins-Conserved

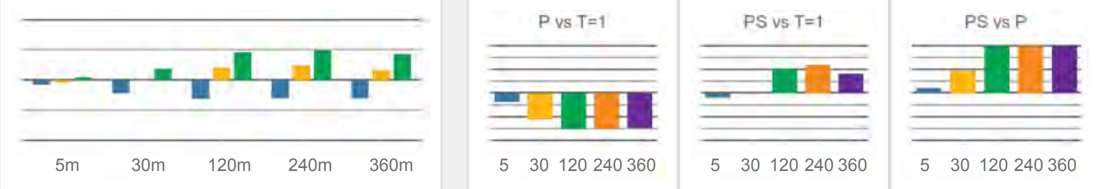

Locus		5m	30m	120m	240m	360m
**PGN_1239**	P vs T=1	-0.897	-1.914	-2.681	-2.731	-2.072
	PS vs T=1	-0.619	-0.728	-0.918	-1.111	-1.200
	PS vs P	0.363	1.009	1.410	1.223	0.775

probable lipopolysaccharide biosynthesis glycosyltransferase

cell envelope

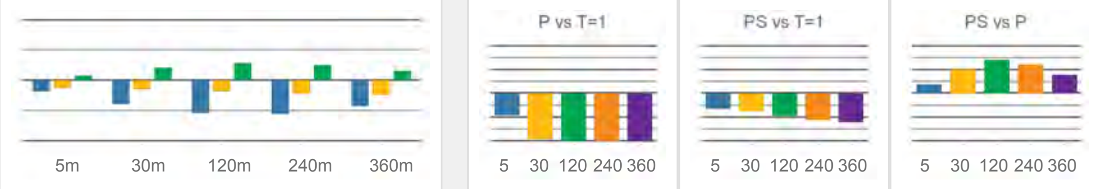

PgSg Time Course

Legend: P vs T=1 | PS vs T=1 | PS vs P

log2 (Fold Change)

Locus		5m	30m	120m	240m	360m
**PGN_1240**	P vs T=1	-1.127	-2.304	-3.368	-3.143	-2.378
	PS vs T=1	-1.035	-1.766	-2.135	-2.545	-2.657
	PS vs P	0.269	0.432	0.859	0.344	-0.283
	conserved hypothetical protein					
	cell envelope					

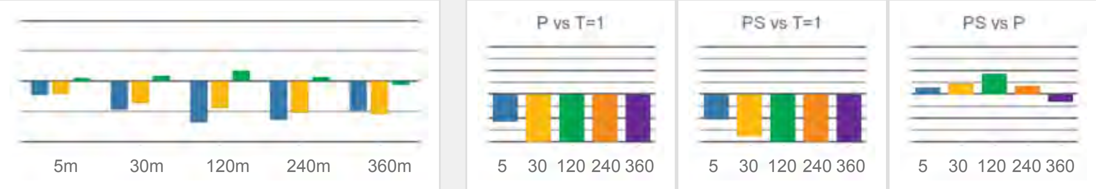

Locus		5m	30m	120m	240m	360m
**PGN_1241**	P vs T=1	-0.781	-2.029	-2.466	-2.806	-1.946
	PS vs T=1	-1.153	-2.064	-2.028	-2.052	-2.442
	PS vs P	-0.108	-0.134	0.244	0.327	-0.450
	hypothetical protein					
	hypothetical proteins					

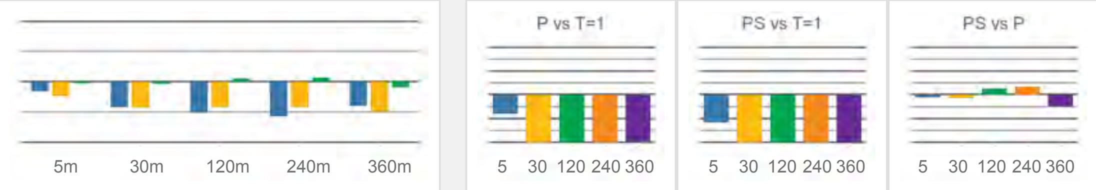

Locus		5m	30m	120m	240m	360m
**PGN_1242** *wzy*	P vs T=1	-0.196	-1.811	-2.612	-2.179	-1.148
	PS vs T=1	-0.594	-1.123	-0.969	-1.160	-1.174
	PS vs P	-0.135	0.444	1.107	0.693	0.017
	putative O-antigen polymerase Wzy					
	cell envelope					

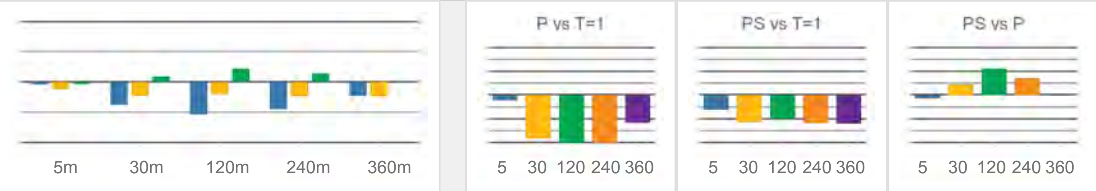

Locus		5m	30m	120m	240m	360m
**PGN_1243**	P vs T=1	-0.381	-1.286	-2.241	-2.185	-1.723
	PS vs T=1	-0.981	-1.171	-1.043	-0.996	-1.150
	PS vs P	-0.528	0.098	1.058	1.049	0.538
	UDP-glucose 6-dehydrogenase					
	cell envelope					

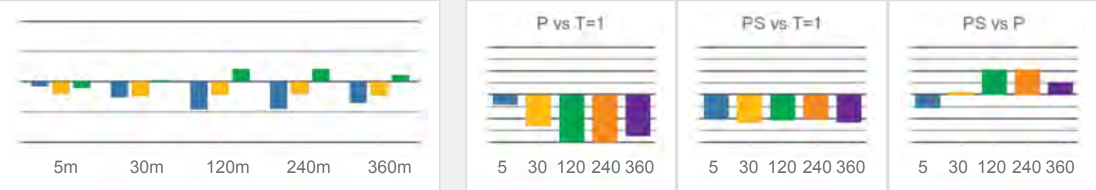

Locus		5m	30m	120m	240m	360m
**PGN_1244**	P vs T=1	0.645	0.655	0.524	0.218	-0.136
	PS vs T=1	-0.067	-0.103	-0.274	-0.410	-0.383
	PS vs P	-0.673	-0.715	-0.760	-0.608	-0.255
	putative peptide chain release factor RF-2					
	protein synthesis					

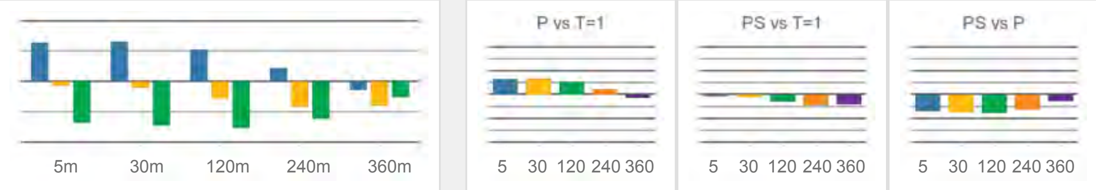

Locus		5m	30m	120m	240m	360m
**PGN_1245**	P vs T=1	0.250	0.206	0.012	-0.045	0.222
	PS vs T=1	0.456	0.383	0.181	0.214	0.031
	PS vs P	0.206	0.176	0.156	0.236	-0.185
	long-chain-fatty-acid-CoA ligase					
	fatty acid and phospholipid metabolism					

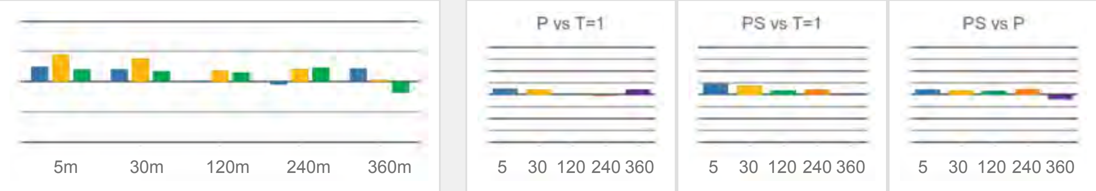

Locus		5m	30m	120m	240m	360m
**PGN_1246**	P vs T=1	0.032	-0.557	-1.372	-1.622	-1.378
	PS vs T=1	-0.185	-0.623	-0.389	-0.776	-1.112
	PS vs P	-0.154	-0.061	0.908	0.747	0.228
	hypothetical protein					
	hypothetical proteins					

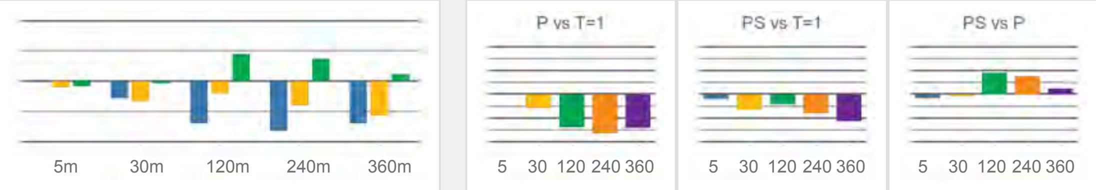

log2 (Fold Change)

P vs T=1 | PS vs T=1 | PS vs P

Locus		5m	30m	120m	240m	360m
**PGN_1247**	P vs T=1	-0.933	-1.394	-0.767	-0.424	-0.314
	PS vs T=1	-2.347	-2.970	-1.549	-1.456	-1.086
	PS vs P	-1.232	-1.435	-0.556	-0.633	-0.514

hypothetical protein

hypothetical proteins

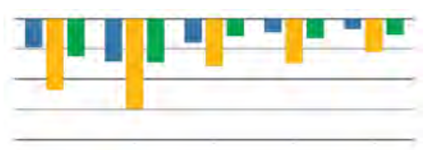

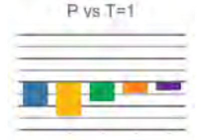

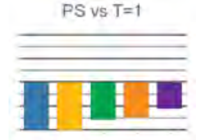

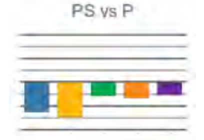

Locus		5m	30m	120m	240m	360m
**PGN_1248**	P vs T=1	0.305	-0.618	1.938	0.845	3.565
	PS vs T=1	-0.435	-0.556	-0.426	0.667	0.870
	PS vs P	-0.350	-0.345	-0.479	0.032	-0.134

hypothetical protein

hypothetical proteins

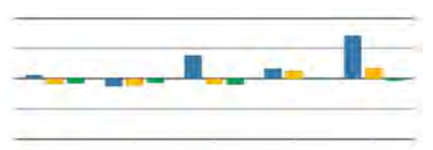

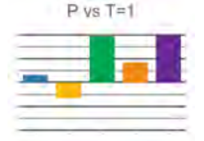

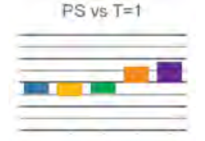

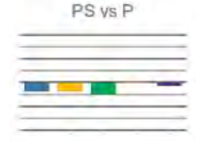

Locus		5m	30m	120m	240m	360m
**PGN_1249**	P vs T=1	-0.133	-0.220	0.422	0.955	2.853
	PS vs T=1	-0.150	-0.635	1.061	1.238	1.080
	PS vs P	-0.180	-0.494	0.548	0.425	-0.390

conserved hypothetical protein

hypothetical proteins-Conserved

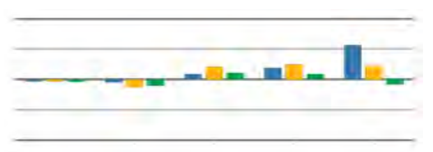

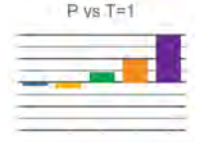

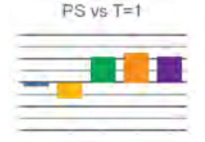

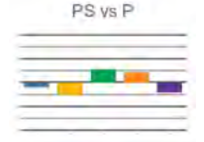

Locus		5m	30m	120m	240m	360m
**PGN_1250**	P vs T=1	0.127	0.012	0.670	0.898	3.545
	PS vs T=1	-0.783	0.048	1.421	1.660	1.485
	PS vs P	-0.733	-0.188	0.633	0.546	-0.427

hypothetical protein

hypothetical proteins

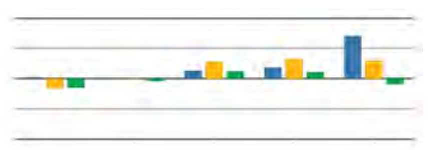

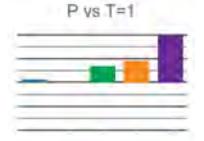

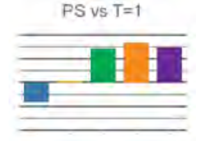

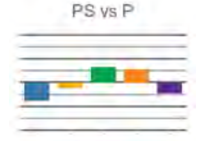

Locus		5m	30m	120m	240m	360m
**PGN_1251** *gtfB*	P vs T=1	-1.322	-2.347	-3.031	-3.092	-2.779
	PS vs T=1	-1.221	-1.474	-1.180	-1.124	-1.526
	PS vs P	0.159	0.738	1.607	1.697	1.127

probable glycosyltransferase

cell envelope

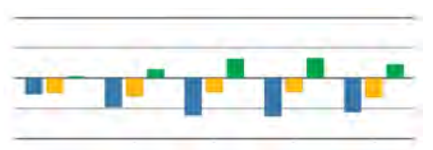

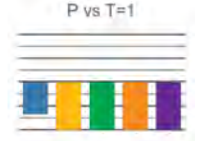

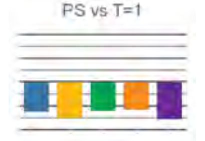

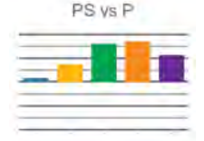

Locus		5m	30m	120m	240m	360m
**PGN_1252**	P vs T=1	1.006	1.360	1.084	0.548	-0.236
	PS vs T=1	1.353	1.547	1.523	1.300	0.936
	PS vs P	0.338	0.205	0.443	0.723	1.120

iron-containing alcohol dehydrogenase

energy metabolism

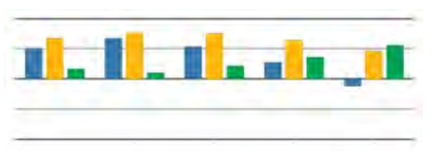

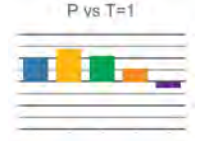

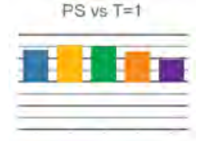

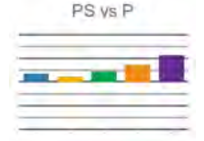

Locus		5m	30m	120m	240m	360m
**PGN_1253**	P vs T=1	-0.271	-0.081	0.383	0.498	-0.054
	PS vs T=1	-0.621	0.091	0.717	0.570	0.461
	PS vs P	-0.364	0.164	0.353	0.096	0.506

hypothetical protein

hypothetical proteins

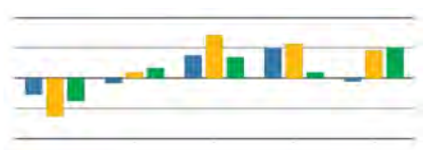

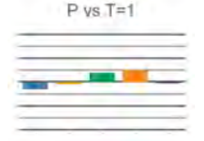

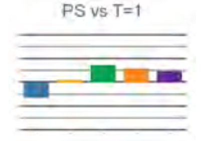

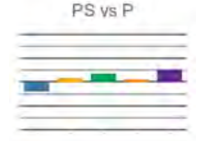

PgSg Time Course

P vs T=1 · PS vs T=1 · PS vs P

Locus		log2 (Fold Change) 5m	30m	120m	240m	360m
**PGN_1254**	P vs T=1	-0.133	-0.903	-1.379	-1.346	-1.469
	PS vs T=1	-0.136	-0.324	-0.212	-0.360	-0.548
	PS vs P	0.023	0.557	1.111	0.932	0.879

hypothetical protein

hypothetical proteins

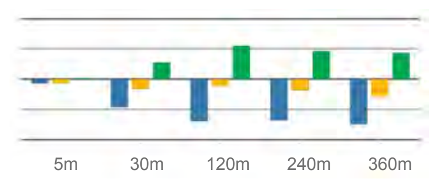

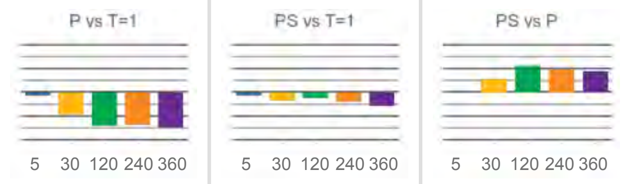

Locus		5m	30m	120m	240m	360m
**PGN_1255** *rfa*	P vs T=1	0.128	-0.243	-0.335	-0.070	0.177
	PS vs T=1	0.520	0.244	0.468	0.514	0.204
	PS vs P	0.395	0.457	0.761	0.573	0.036

putative heptosyltransferase

cell envelope

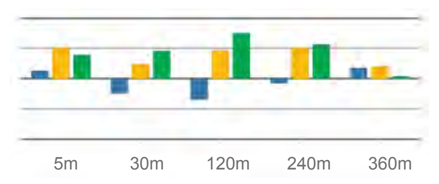

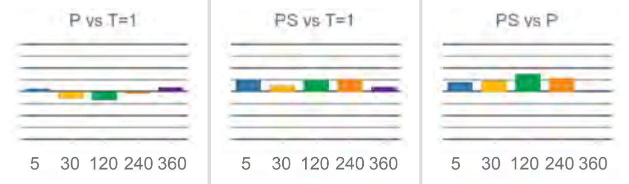

Locus		5m	30m	120m	240m	360m
**PGN_1256**	P vs T=1	-0.787	-0.570	-0.321	-0.213	-0.478
	PS vs T=1	-0.609	-0.363	-0.033	0.066	0.118
	PS vs P	0.142	0.193	0.295	0.297	0.582

conserved hypothetical protein

unknown function

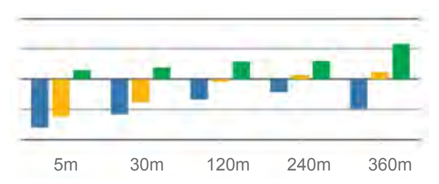

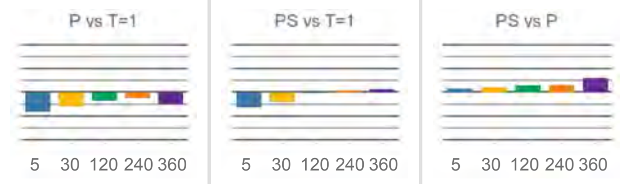

Locus		5m	30m	120m	240m	360m
**PGN_1257**	P vs T=1	0.563	0.693	0.457	1.045	1.890
	PS vs T=1	0.616	0.627	0.576	1.177	0.467
	PS vs P	0.035	-0.013	-0.059	0.306	-0.715

hypothetical protein

hypothetical proteins

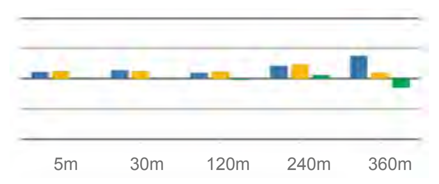

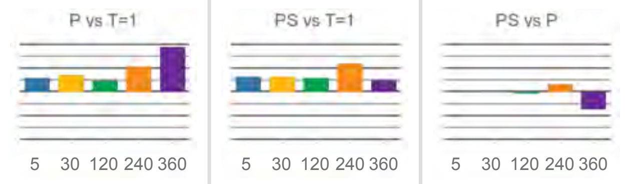

Locus		5m	30m	120m	240m	360m
**PGN_1258** *CobC/D*	P vs T=1	-0.399	-0.739	-0.911	-0.694	-0.331
	PS vs T=1	0.366	0.358	-0.337	-0.607	-0.342
	PS vs P	0.751	1.036	0.497	0.054	0.003

cobalamin biosynthesis protein

biosynthesis of cofactors, prosthetic groups, and carriers

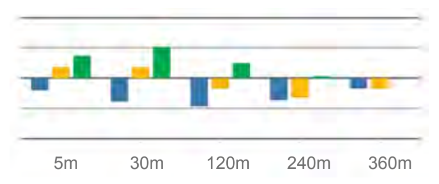

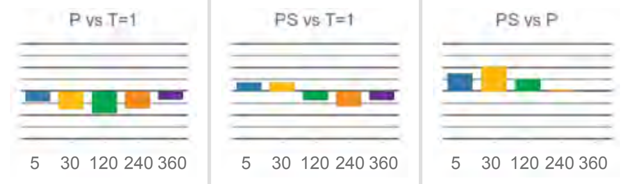

Locus		5m	30m	120m	240m	360m
**PGN_1259**	P vs T=1	-0.485	-0.508	-0.725	-0.306	-0.128
	PS vs T=1	0.295	0.127	-0.954	-0.986	-0.892
	PS vs P	0.753	0.609	-0.285	-0.613	-0.707

probable histidinol-phosphate aminotransferase

amino acid biosynthesis

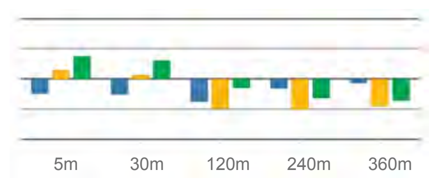

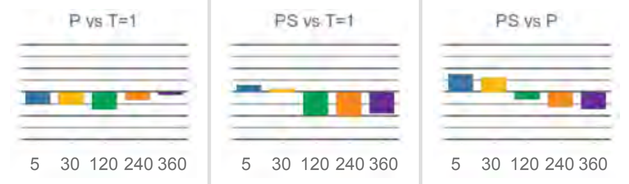

Locus		5m	30m	120m	240m	360m
**PGN_1260** *CobQ*	P vs T=1	-0.284	-0.435	-0.445	-0.289	0.213
	PS vs T=1	0.513	0.091	-0.658	-0.773	-0.400
	PS vs P	0.778	0.491	-0.240	-0.479	-0.562

cobyric acid synthase

biosynthesis of cofactors, prosthetic groups, and carriers

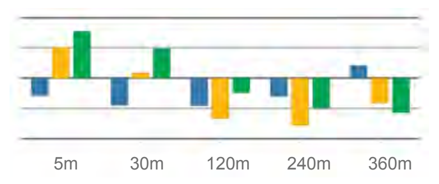

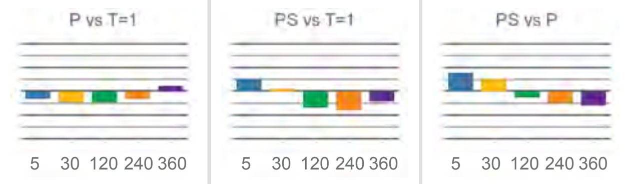

PgSg Time Course

log$_2$ (Fold Change)

Legend: P vs T=1 | PS vs T=1 | PS vs P

Locus		5m	30m	120m	240m	360m
**PGN_1261**	P vs T=1	-0.625	-0.396	-0.494	-0.400	-0.557
	PS vs T=1	-0.069	-0.229	-0.889	-0.603	-0.622
	PS vs P	0.518	0.165	-0.406	-0.188	-0.087
	probable cobalamin adenosyltransferase					
	hypothetical proteins-Conserved					

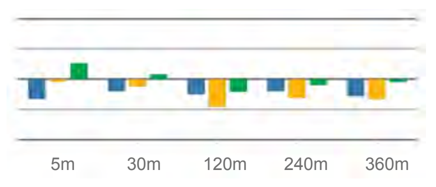

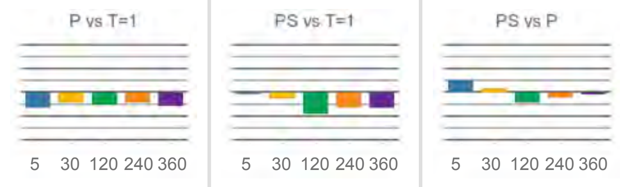

Locus		5m	30m	120m	240m	360m
**PGN_1262**	P vs T=1	-0.304	-0.449	-0.463	-0.060	-0.065
CobB	PS vs T=1	0.202	-0.109	-0.295	-0.104	-0.140
	PS vs P	0.492	0.313	0.133	-0.024	-0.069
	cobyrinic acid a,c-diamide synthase					
	biosynthesis of cofactors, prosthetic groups, and carriers					

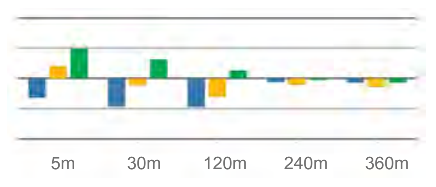

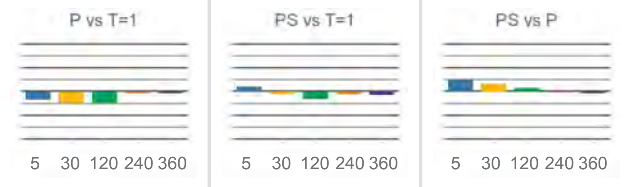

Locus		5m	30m	120m	240m	360m
**PGN_1263**	P vs T=1	0.326	0.443	-0.052	-0.518	-0.505
	PS vs T=1	0.702	0.320	-0.665	-1.050	-1.022
	PS vs P	0.382	-0.110	-0.606	-0.545	-0.521
	conserved hypothetical protein					
	hypothetical proteins-Conserved					

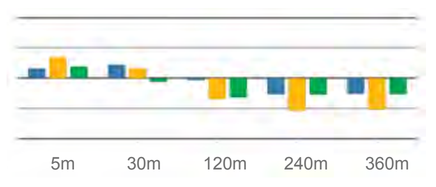

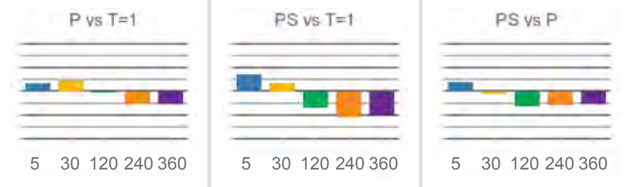

Locus		5m	30m	120m	240m	360m
**PGN_1264**	P vs T=1	-0.967	-1.532	-2.432	-2.104	-1.086
	PS vs T=1	-1.148	-1.722	-2.410	-2.152	-1.792
	PS vs P	-0.051	-0.166	-0.227	-0.161	-0.568
	conserved hypothetical protein					
	hypothetical proteins-Conserved					

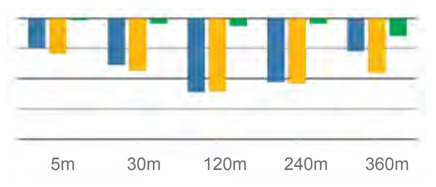

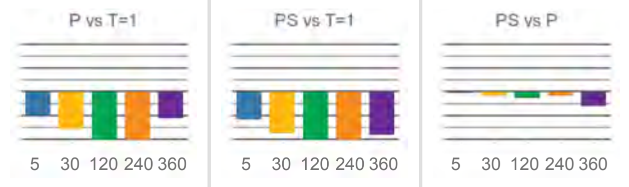

Locus		5m	30m	120m	240m	360m
**PGN_1265**	P vs T=1	0.790	-0.136	1.474	1.746	3.925
	PS vs T=1	0.275	-0.087	1.641	1.358	0.861
	PS vs P	-0.307	-0.431	0.502	0.044	-1.232
	hypothetical protein					
	hypothetical proteins					

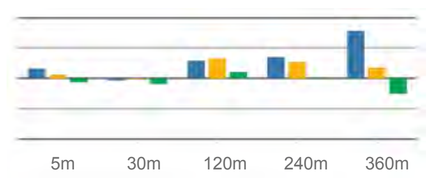

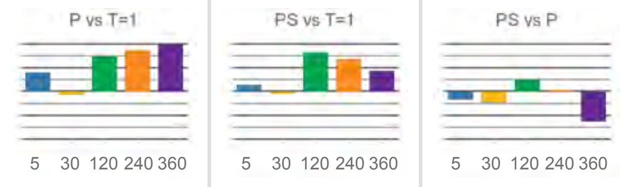

Locus		5m	30m	120m	240m	360m
**PGN_1266**	P vs T=1	2.338	1.023	1.503	1.907	2.839
	PS vs T=1	0.841	-0.204	1.023	0.427	0.255
	PS vs P	-0.766	-0.886	-0.130	-0.589	-1.101
	conserved hypothetical protein					
	hypothetical proteins-Conserved					

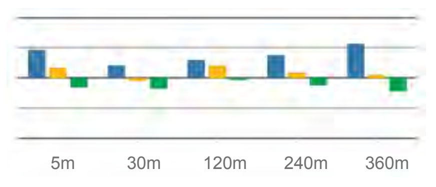

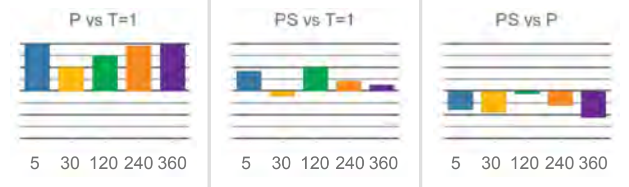

Locus		5m	30m	120m	240m	360m
**PGN_1267**	P vs T=1	0.130	-0.120	0.087	0.713	1.347
	PS vs T=1	0.097	0.440	0.971	1.352	1.098
	PS vs P	-0.060	0.442	0.745	0.676	-0.134
	conserved hypothetical protein					
	unknown function					

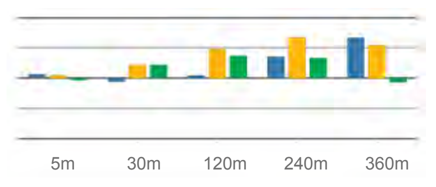

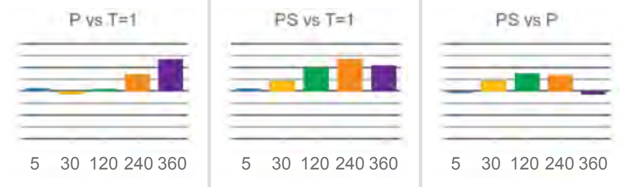

PgSg Time Course

log$_2$ (Fold Change)

Legend: P vs T=1 · PS vs T=1 · PS vs P

Locus		5m	30m	120m	240m	360m
**PGN_1268**	P vs T=1	0.044	0.044	0.128	0.132	-0.222
	PS vs T=1	0.439	0.345	0.121	0.298	0.047
	PS vs P	0.370	0.275	-0.019	0.153	0.231

putative oxidoreductase

unknown function

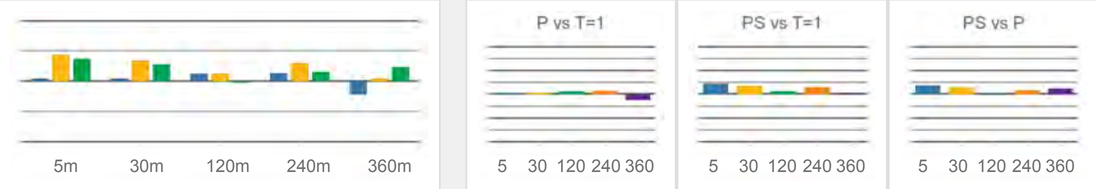

Locus		5m	30m	120m	240m	360m
**PGN_1269**	P vs T=1	1.043	1.197	1.427	1.065	0.652
	PS vs T=1	1.522	1.494	1.261	1.000	1.042
	PS vs P	0.457	0.291	-0.134	-0.077	0.352

putative electron transport protein

energy metabolism

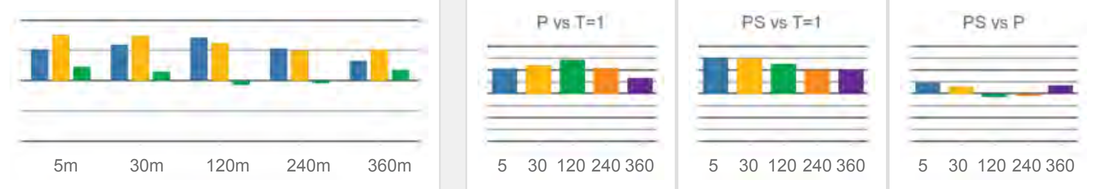

Locus		5m	30m	120m	240m	360m
**PGN_1270**	P vs T=1	0.700	0.847	0.611	0.192	-0.335
	PS vs T=1	1.174	1.468	1.321	0.669	0.563
	PS vs P	0.459	0.619	0.698	0.419	0.815

conserved hypothetical protein

unknown function

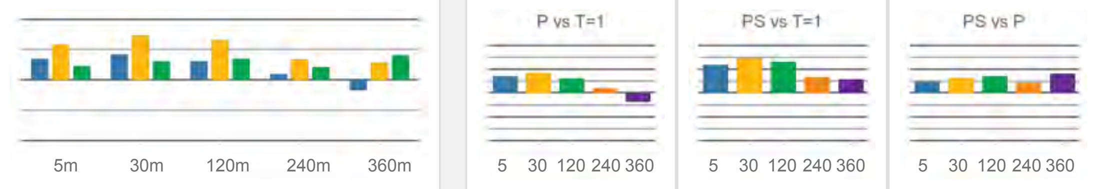

Locus		5m	30m	120m	240m	360m
**PGN_1271**	P vs T=1	0.378	0.290	0.333	0.495	0.040
	PS vs T=1	-0.371	-0.030	0.683	0.964	0.807
	PS vs P	-0.718	-0.306	0.351	0.491	0.738

probable thioesterase superfamily protein

hypothetical proteins

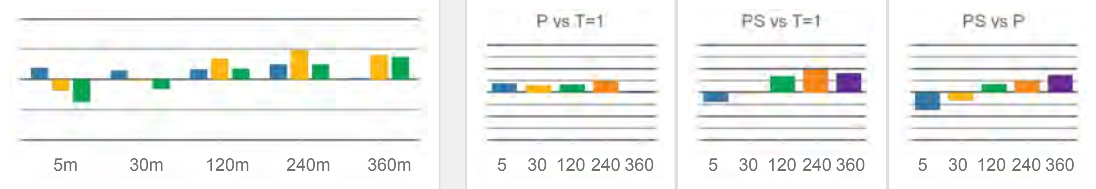

Locus		5m	30m	120m	240m	360m
**PGN_1272**	P vs T=1	-0.256	-0.421	-0.807	-1.133	-1.272
	PS vs T=1	-0.880	-0.767	-1.006	-0.754	-1.042
	PS vs P	-0.591	-0.324	-0.205	0.329	0.191

putative diaminopimelate decarboxylase

amino acid biosynthesis

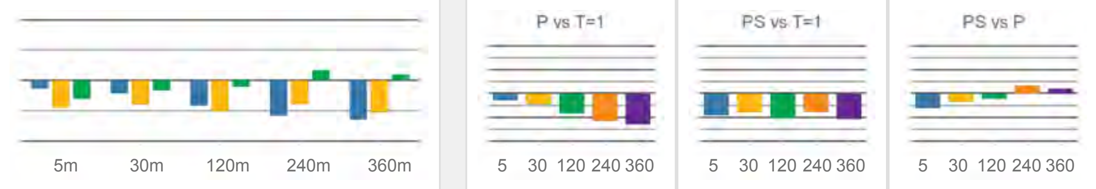

Locus		5m	30m	120m	240m	360m
**PGN_1273** *MenA*	P vs T=1	0.417	0.290	0.262	0.321	0.371
	PS vs T=1	-0.848	-0.310	-0.447	-0.135	-0.094
	PS vs P	-1.217	-0.569	-0.681	-0.413	-0.434

probable 1,4-dihydroxy-2-naphthoate octaprenyltransferase

biosynthesis of cofactors, prosthetic groups, and carriers

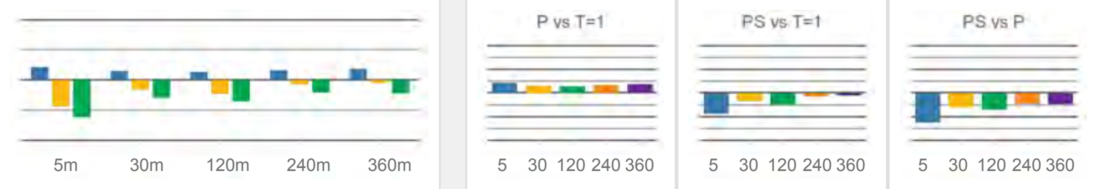

Locus		5m	30m	120m	240m	360m
**PGN_1274**	P vs T=1	0.035	0.215	0.700	0.583	0.330
	PS vs T=1	-0.596	-0.028	0.501	0.650	0.435
	PS vs P	-0.648	-0.253	-0.157	0.076	0.079

conserved hypothetical protein

hypothetical proteins-Conserved

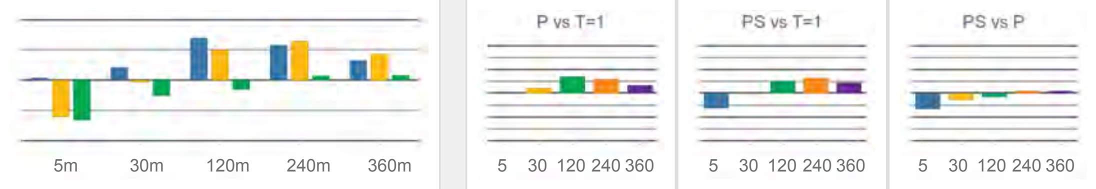

log$_2$ (Fold Change)

P vs T=1 | PS vs T=1 | PS vs P

Locus		5m	30m	120m	240m	360m
**PGN_1275**	P vs T=1	1.155	1.859	2.568	2.712	2.108
	PS vs T=1	1.103	1.661	2.261	2.302	2.079
	PS vs P	-0.091	-0.192	-0.271	-0.368	-0.028

conserved hypothetical protein

transport and binding proteins

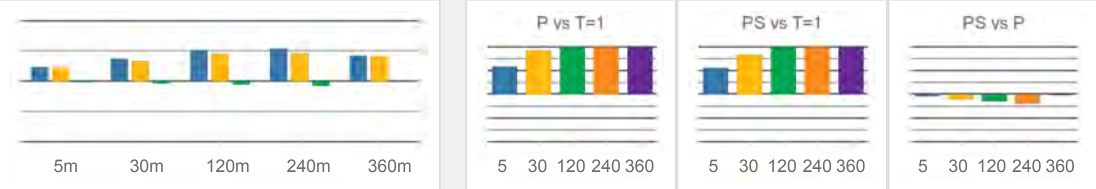

Locus		5m	30m	120m	240m	360m
**PGN_1276**	P vs T=1	0.354	0.290	0.379	0.472	0.205
	PS vs T=1	0.139	-0.040	0.127	0.309	0.242
	PS vs P	-0.214	-0.335	-0.257	-0.162	0.023

transposase in ISPg1

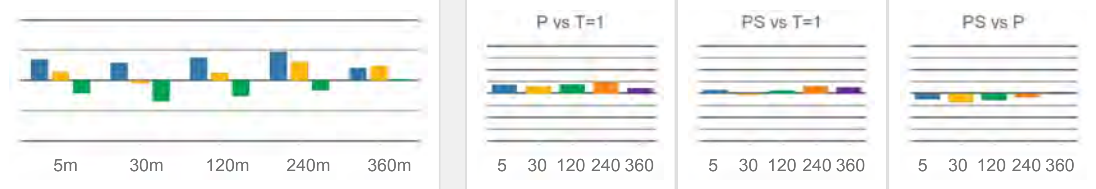

Locus		5m	30m	120m	240m	360m
**PGN_1277**	P vs T=1	0.114	-0.410	0.086	1.396	2.550
	PS vs T=1	0.251	-0.085	0.884	1.095	1.291
	PS vs P	0.033	-0.151	0.361	0.131	-0.506

conserved hypothetical protein

hypothetical proteins-Conserved

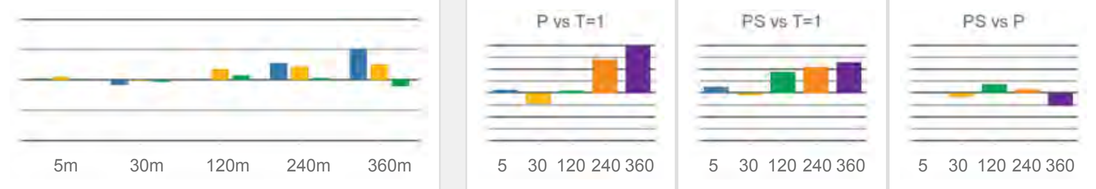

Locus		5m	30m	120m	240m	360m
**PGN_1278**	P vs T=1	0.421	-0.132	-0.116	0.656	1.332
	PS vs T=1	0.059	0.048	0.613	0.353	0.692
	PS vs P	-0.333	0.094	0.573	-0.244	-0.537

partial transposase in ISPg3

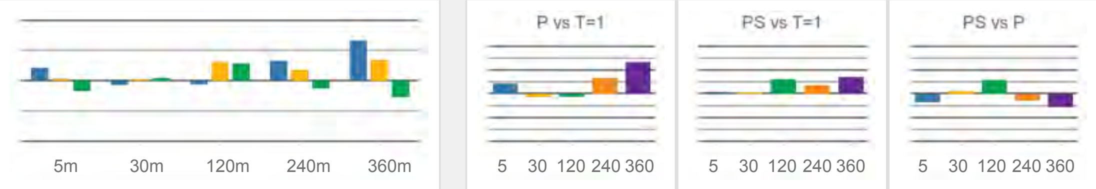

Locus		5m	30m	120m	240m	360m
**PGN_1279**	P vs T=1	0.168	-0.319	-0.246	0.230	0.595
	PS vs T=1	-0.148	0.049	0.297	0.583	0.346
	PS vs P	-0.310	0.237	0.341	0.300	-0.212

partial transposase in ISPg3

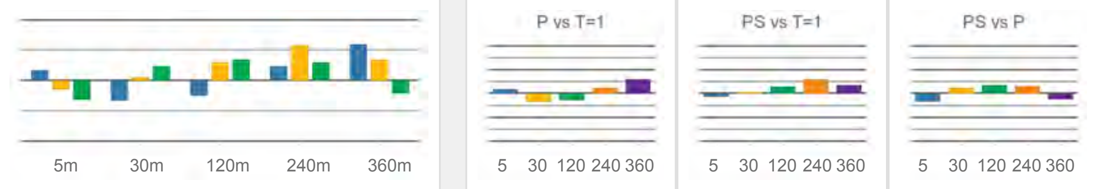

Locus		5m	30m	120m	240m	360m
**PGN_1280**	P vs T=1	-0.169	-0.291	-0.272	-0.332	-0.505
	PS vs T=1	-0.371	-0.733	-0.479	-0.307	-0.128
	PS vs P	-0.202	-0.449	-0.222	-0.004	0.340

partial transposase in ISPg1

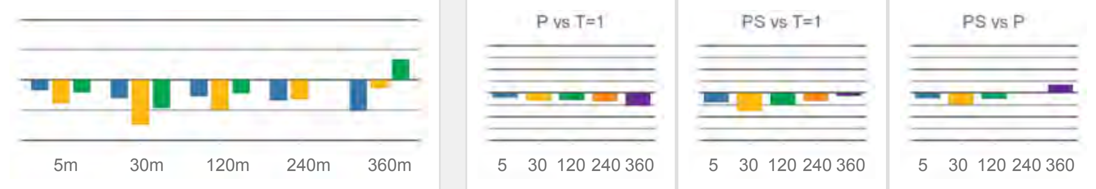

Locus		5m	30m	120m	240m	360m
**PGN_1281** *traM*	P vs T=1	0.525	1.287	2.420	2.857	2.752
	PS vs T=1	0.064	0.469	1.260	1.693	2.149
	PS vs P	-0.537	-0.797	-1.055	-1.028	-0.528

putative conserved protein found in conjugate transposon TraM

mobile and extrachromosomal element functions

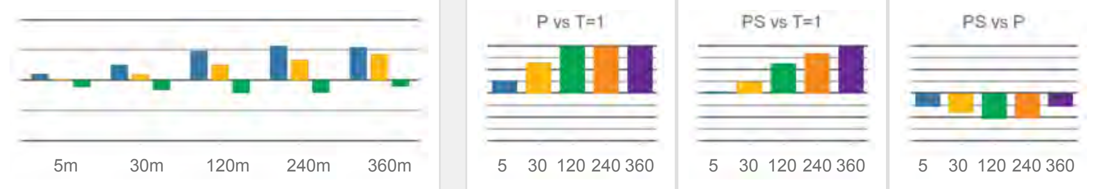

PgSg Time Course

log$_2$ (Fold Change)

Legend: P vs T=1 | PS vs T=1 | PS vs P

Locus		5m	30m	120m	240m	360m
**PGN_1282**	P vs T=1	0.816	1.521	2.260	2.323	1.966
traN	PS vs T=1	0.217	0.787	1.382	1.624	1.791
	PS vs P	-0.635	-0.693	-0.791	-0.618	-0.149

conserved protein found in conjugate transposon TraN

other categories

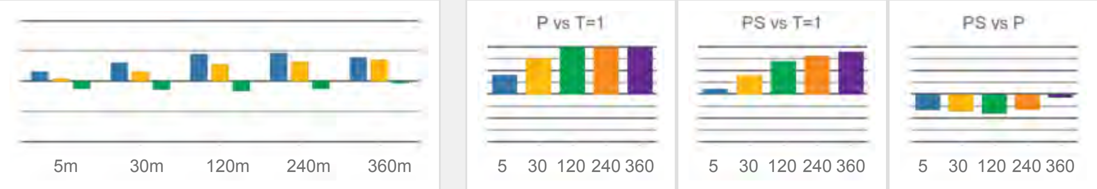

Locus		5m	30m	120m	240m	360m
**PGN_1283**	P vs T=1	1.424	2.315	2.954	2.889	2.512
traO	PS vs T=1	0.597	1.111	1.648	1.792	1.886
	PS vs P	-0.843	-1.097	-1.166	-0.969	-0.570

conserved protein found in conjugate transposon TraO

other categories

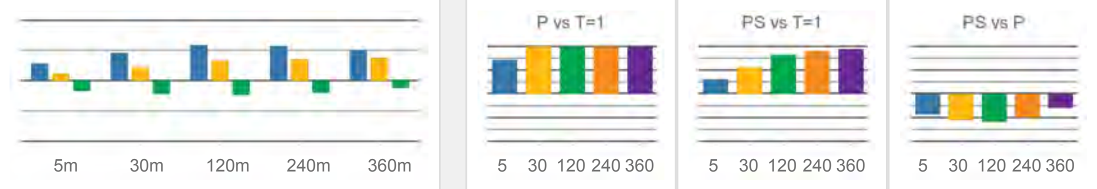

Locus		5m	30m	120m	240m	360m
**PGN_1284**	P vs T=1	0.639	1.270	1.778	1.810	1.605
traP	PS vs T=1	0.305	0.808	1.290	1.186	1.004
	PS vs P	-0.356	-0.439	-0.442	-0.572	-0.576

putative DNA primase involved in conjugation TraP

cellular processes

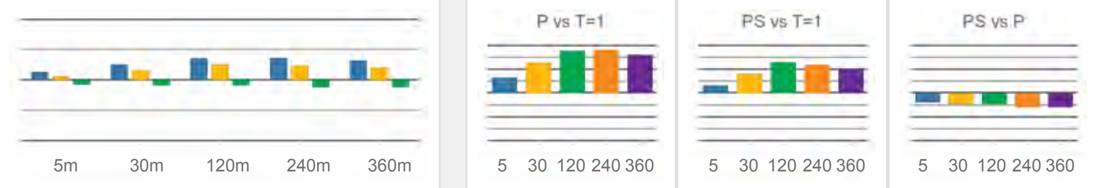

Locus		5m	30m	120m	240m	360m
**PGN_1285**	P vs T=1	1.597	2.116	2.939	3.116	2.633
traQ	PS vs T=1	0.876	1.579	2.335	2.421	2.557
	PS vs P	-0.733	-0.501	-0.504	-0.577	-0.041

conserved protein found in conjugate transposon TraQ

other categories

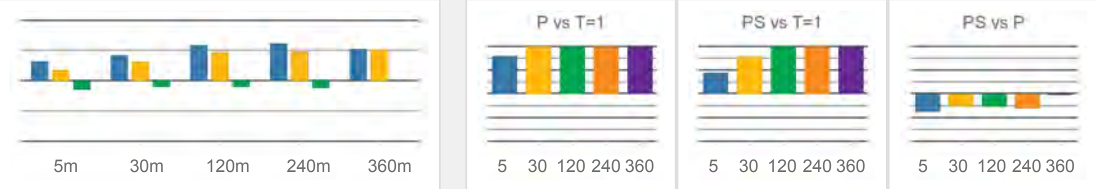

Locus		5m	30m	120m	240m	360m
**PGN_1286**	P vs T=1	1.351	1.506	1.647	1.355	0.916
	PS vs T=1	-0.750	-0.404	0.022	-0.195	0.045
	PS vs P	-2.038	-1.838	-1.558	-1.485	-0.849

probable lysozyme

cellular processes

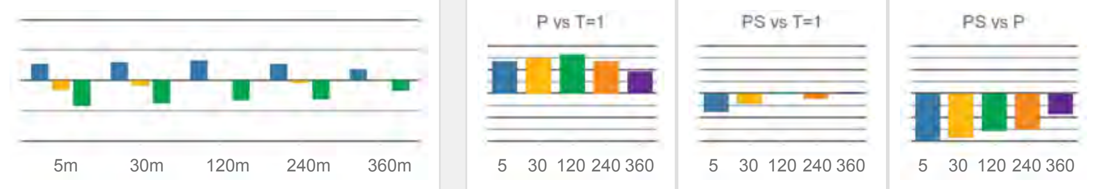

Locus		5m	30m	120m	240m	360m
**PGN_1287**	P vs T=1	1.710	2.126	2.636	2.614	2.013
	PS vs T=1	-0.881	-0.316	0.185	-0.019	-0.180
	PS vs P	-2.439	-2.249	-2.261	-2.447	-2.102

conserved hypothetical protein

hypothetical proteins-Conserved

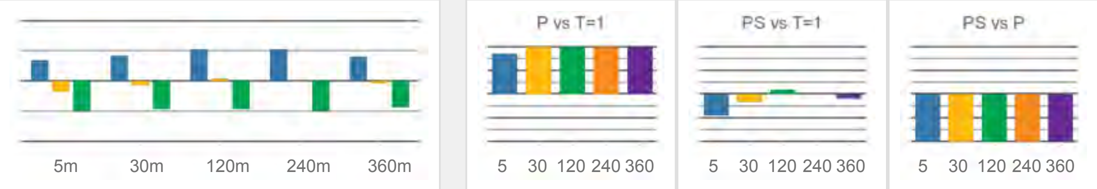

Locus		5m	30m	120m	240m	360m
**PGN_1288**	P vs T=1	2.015	1.580	1.425	1.174	0.722
	PS vs T=1	-0.530	-0.237	0.032	-0.428	-0.753
	PS vs P	-2.209	-1.588	-1.231	-1.466	-1.416

conserved hypothetical protein

hypothetical proteins-Conserved

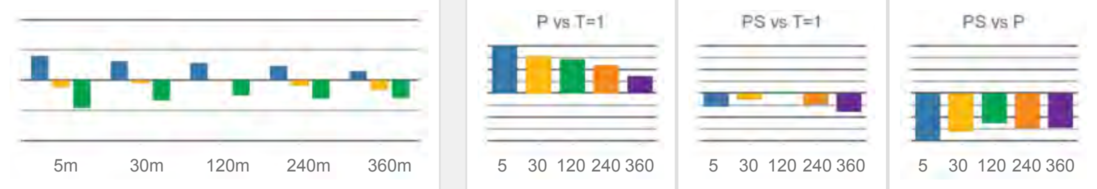

PgSg Time Course

Legend: P vs T=1 | PS vs T=1 | PS vs P

log$_2$ (Fold Change)

Locus		5m	30m	120m	240m	360m
**PGN_1289**	P vs T=1	0.069	0.222	1.302	1.249	1.802
	PS vs T=1	-0.784	-0.469	0.692	0.896	1.126
	PS vs P	-0.857	-0.694	-0.420	-0.208	-0.493
	conserved hypothetical protein					
	hypothetical proteins-Conserved					
**PGN_1290**	P vs T=1	0.569	0.612	1.383	1.523	1.901
	PS vs T=1	-0.043	0.371	1.283	0.983	0.815
	PS vs P	-0.601	-0.268	0.049	-0.327	-0.828
	conserved hypothetical protein					
	hypothetical proteins-Conserved					
**PGN_1291**	P vs T=1	0.609	0.532	1.056	1.691	2.292
	PS vs T=1	0.027	0.604	1.438	1.413	1.192
	PS vs P	-0.586	0.022	0.376	-0.169	-0.972
	conserved hypothetical protein related to phage					
	hypothetical proteins-Conserved					
**PGN_1292**	P vs T=1	1.085	1.130	1.800	2.361	3.356
	PS vs T=1	0.734	1.499	2.327	2.062	2.063
	PS vs P	-0.380	0.284	0.545	-0.125	-1.048
	probable anti-restriction protein					
	anti-restriction					
**PGN_1293**	P vs T=1	0.619	0.569	0.602	1.885	2.975
	PS vs T=1	0.131	0.725	1.634	1.855	1.914
	PS vs P	-0.500	0.053	0.705	0.254	-0.682
	conserved hypothetical protein					
	hypothetical proteins-Conserved					
**PGN_1294**	P vs T=1	-0.236	-0.983	-0.445	0.258	2.502
	PS vs T=1	-0.788	-0.609	-0.149	1.012	0.789
	PS vs P	-0.460	-0.142	-0.004	0.687	-0.672
	conserved hypothetical protein					
	hypothetical proteins-Conserved					
**PGN_1295**	P vs T=1	0.935	0.371	1.291	2.125	3.122
	PS vs T=1	0.298	0.628	0.975	1.289	1.605
	PS vs P	-0.448	-0.045	-0.158	-0.228	-0.678
	hypothetical protein					
	hypothetical proteins					

PGN_1289

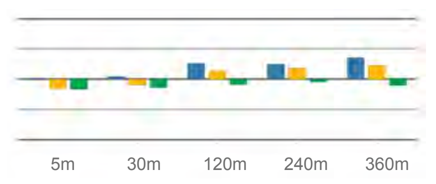

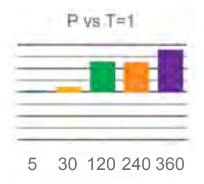

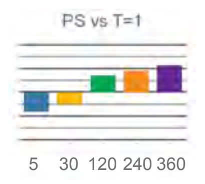

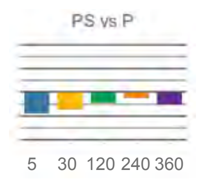

PGN_1290

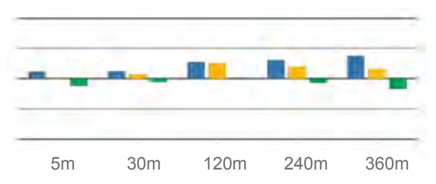

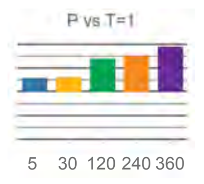

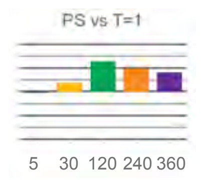

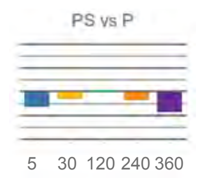

PGN_1291

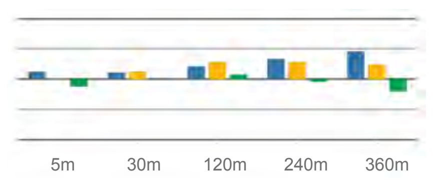

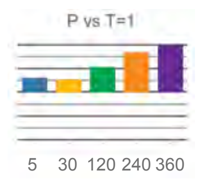

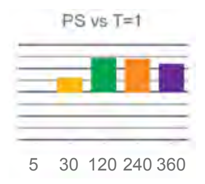

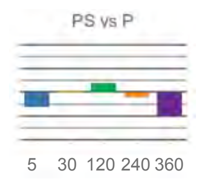

PGN_1292

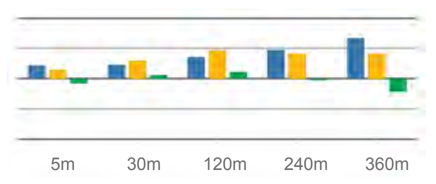

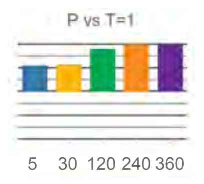

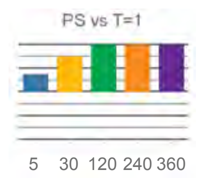

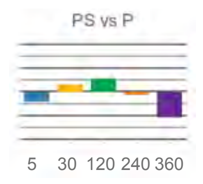

PGN_1293

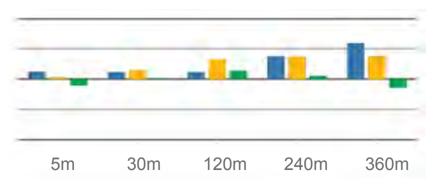

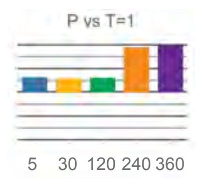

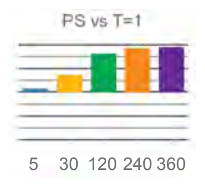

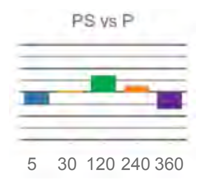

PGN_1294

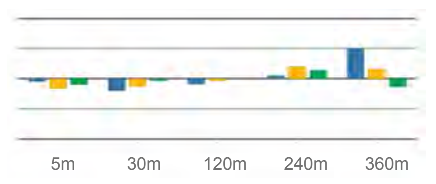

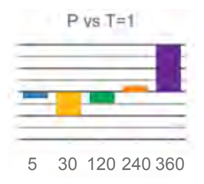

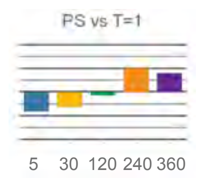

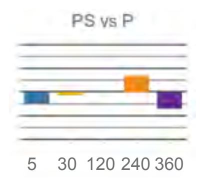

PGN_1295

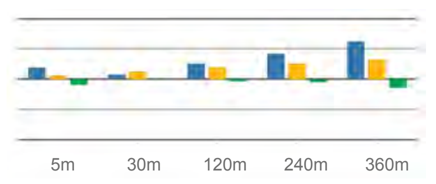

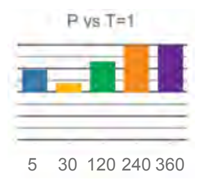

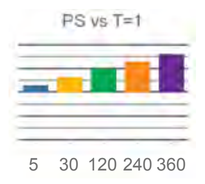

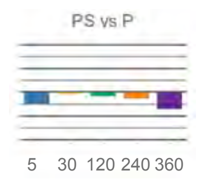

PgSg Time Course

Hackett Laboratory UW

Legend: P vs T=1 · PS vs T=1 · PS vs P

Locus		log2 (Fold Change) 5m	30m	120m	240m	360m
**PGN_1296**	P vs T=1	-0.012	0.044	-0.154	-0.312	-0.374
	PS vs T=1	0.533	0.353	0.050	0.025	-0.072
	PS vs P	0.539	0.307	0.195	0.314	0.288

putative OmpA family protein

cell envelope

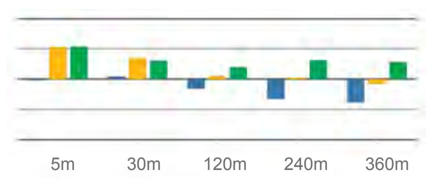

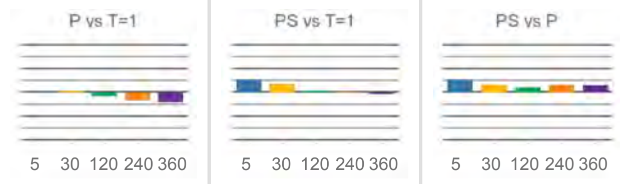

Locus		5m	30m	120m	240m	360m
**PGN_1297**	P vs T=1	0.819	0.994	1.012	0.772	0.219
	PS vs T=1	0.765	0.683	0.676	0.848	0.811
	PS vs P	-0.052	-0.298	-0.322	0.072	0.561

conserved hypothetical protein

hypothetical proteins-Conserved

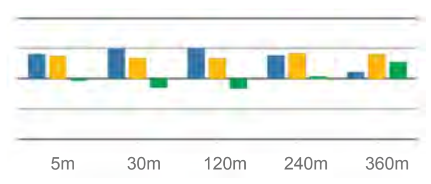

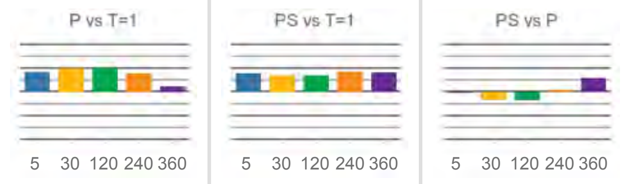

Locus		5m	30m	120m	240m	360m
**PGN_1298**	P vs T=1	-0.407	-0.405	-0.754	-1.080	-1.467
	PS vs T=1	-0.373	-0.571	-0.647	-0.704	-0.828
	PS vs P	0.035	-0.162	0.099	0.344	0.601

conserved hypothetical protein

hypothetical proteins-Conserved

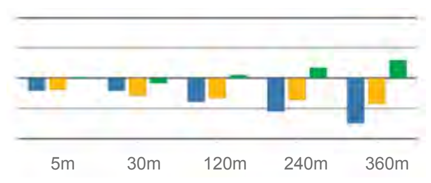

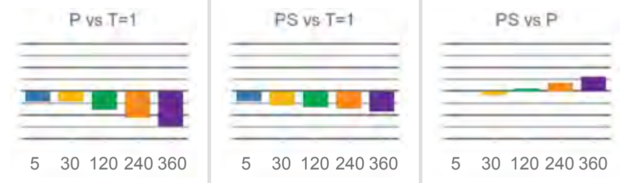

Locus		5m	30m	120m	240m	360m
**PGN_1299**	P vs T=1	0.419	0.103	-0.043	0.681	1.437
	PS vs T=1	0.418	0.392	1.101	0.589	0.631
	PS vs P	0.029	0.152	0.800	0.052	-0.394

hypothetical protein

hypothetical proteins

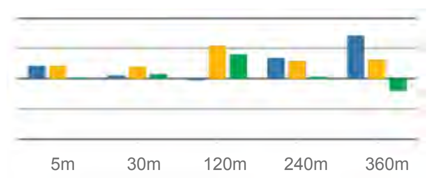

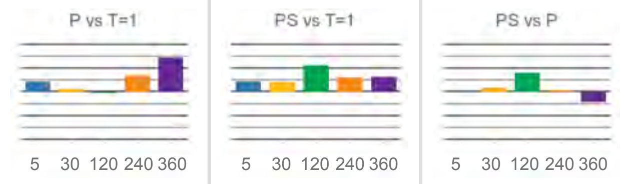

Locus		5m	30m	120m	240m	360m
**PGN_1300**	P vs T=1	-0.198	-0.850	-1.124	-0.802	-0.311
	PS vs T=1	-0.368	-0.580	-0.138	0.008	-0.164
	PS vs P	-0.120	0.198	0.825	0.726	0.170

probable transcriptional regulator

regulatory functions

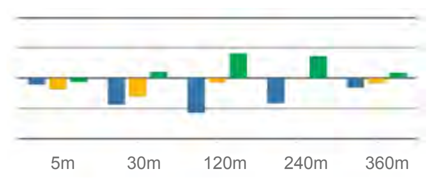

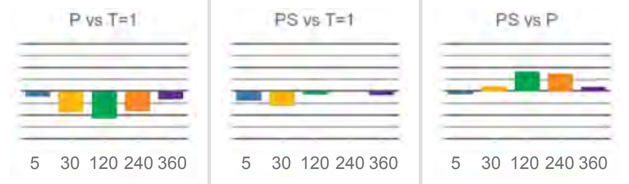

Locus		5m	30m	120m	240m	360m
**PGN_1301**	P vs T=1	-0.321	-1.099	-1.682	-1.513	-1.252
	PS vs T=1	-0.634	-0.722	-0.336	-0.370	-0.735
	PS vs P	-0.244	0.329	1.158	0.992	0.469

probable transcriptional regulator

regulatory functions

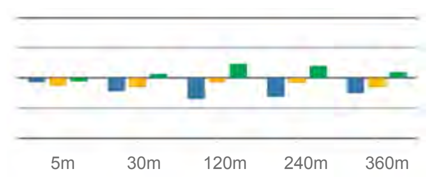

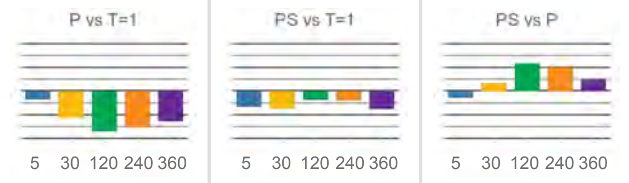

Locus		5m	30m	120m	240m	360m
**PGN_1302** *waaL*	P vs T=1	-1.264	-2.131	-2.579	-2.098	-1.369
	PS vs T=1	-1.327	-1.680	-1.269	-1.514	-1.588
	PS vs P	-0.006	0.361	1.119	0.517	-0.176

O-antigen ligase waaL

cell envelope

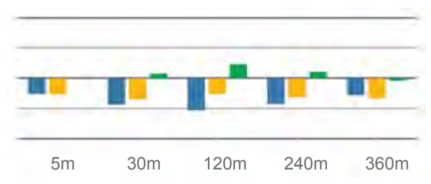

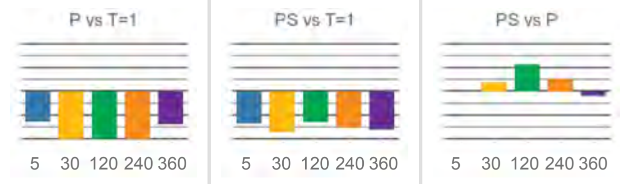

log$_2$ (Fold Change)

Legend: P vs T=1 | PS vs T=1 | PS vs P

Locus		5m	30m	120m	240m	360m
**PGN_1303**	P vs T=1	-0.603	-1.068	-1.227	-1.160	-1.125
	PS vs T=1	-1.094	-0.825	-0.096	-0.486	-0.619
	PS vs P	-0.468	0.213	1.073	0.621	0.475

hypothetical protein

hypothetical proteins

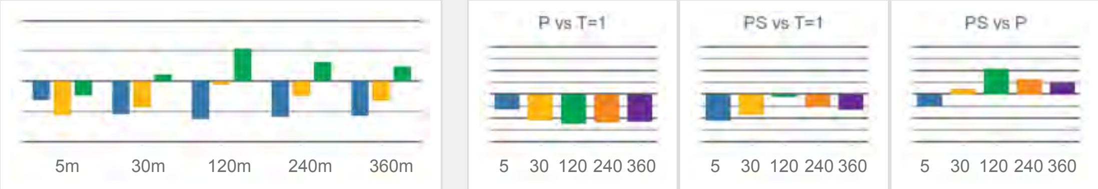

Locus		5m	30m	120m	240m	360m
**PGN_1304**	P vs T=1	-0.856	-1.110	-1.531	-1.660	-1.459
	PS vs T=1	-1.551	-1.042	-0.337	-0.618	-0.935
	PS vs P	-0.666	0.061	1.122	0.934	0.484

conserved hypothetical protein

hypothetical proteins-Conserved

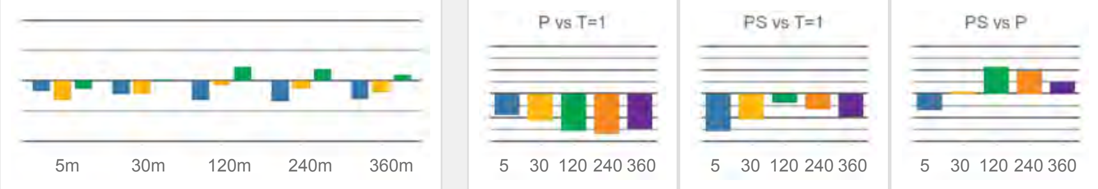

Locus		5m	30m	120m	240m	360m
**PGN_1305**	P vs T=1	0.212	0.141	-0.277	-0.264	-0.473
	PS vs T=1	-0.378	-0.088	0.013	-0.057	-0.345
	PS vs P	-0.544	-0.193	0.274	0.203	0.111

putative N-acetylmuramoyl-L-alanine amidase

cell envelope

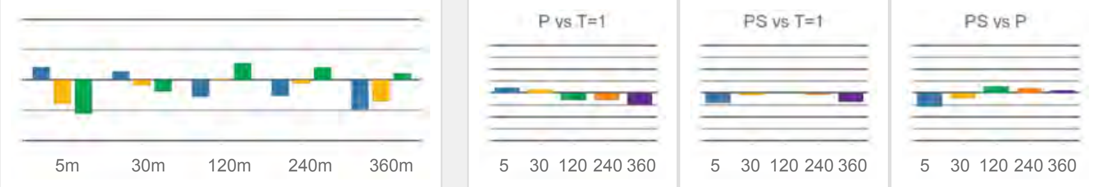

Locus		5m	30m	120m	240m	360m
**PGN_1306**	P vs T=1	0.891	0.396	1.006	1.192	1.853
	PS vs T=1	-1.030	-0.739	0.183	0.408	0.876
	PS vs P	-1.605	-1.003	-0.577	-0.465	-0.612

hypothetical protein

hypothetical proteins

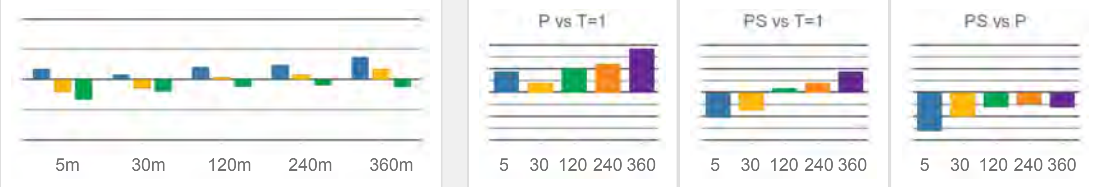

Locus		5m	30m	120m	240m	360m
**PGN_1307**	P vs T=1	0.191	0.003	1.084	1.406	1.775
	PS vs T=1	-0.793	-0.327	0.259	0.514	0.751
	PS vs P	-0.898	-0.398	-0.534	-0.469	-0.655

hypothetical protein

hypothetical proteins

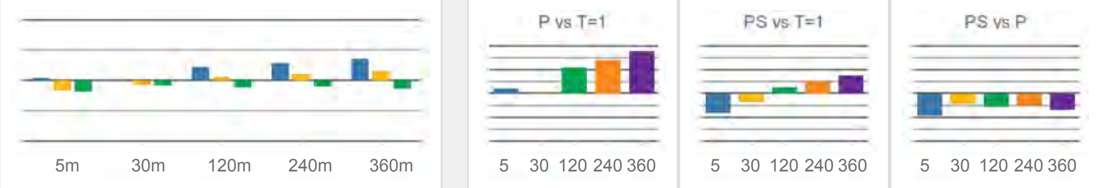

Locus		5m	30m	120m	240m	360m
**PGN_1308**	P vs T=1	0.086	0.106	0.035	0.464	0.493
	PS vs T=1	-0.081	0.387	0.907	1.003	0.902
	PS vs P	-0.178	0.264	0.818	0.546	0.410

probable iron dependent repressor

regulatory functions

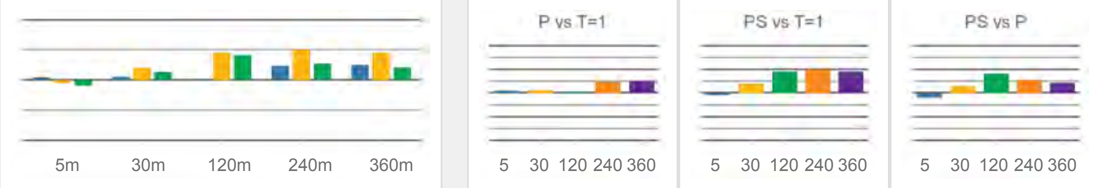

Locus		5m	30m	120m	240m	360m
**PGN_1309**	P vs T=1	-0.613	-0.481	-0.085	0.288	0.842
	PS vs T=1	-0.734	-0.762	-0.771	-0.440	-0.172
	PS vs P	-0.136	-0.287	-0.664	-0.665	-0.950

ferrous iron transport protein B

transport and binding proteins

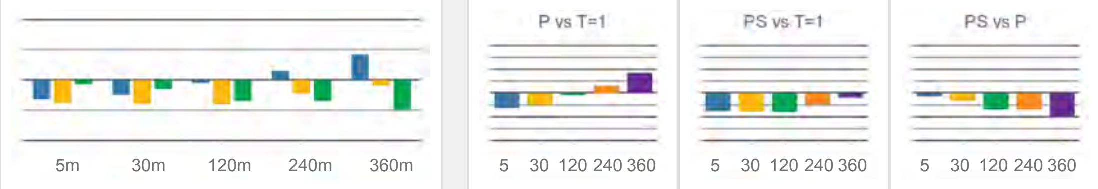

PgSg Time Course

log$_2$ (Fold Change)

P vs T=1 | PS vs T=1 | PS vs P

Locus		5m	30m	120m	240m	360m
**PGN_1310**	P vs T=1	-0.347	-0.134	0.250	1.009	1.661
	PS vs T=1	0.220	0.114	-0.034	0.481	0.685
	PS vs P	0.505	0.206	-0.301	-0.413	-0.871

glycogen synthase

energy metabolism

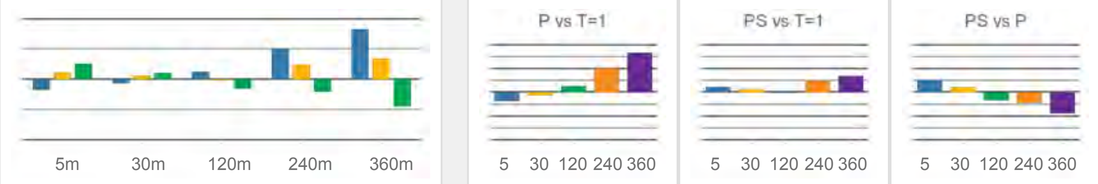

Locus		5m	30m	120m	240m	360m
**PGN_1311**	P vs T=1	-0.158	-0.557	-0.644	-0.573	-0.064
	PS vs T=1	-0.321	-0.411	-0.133	0.351	0.250
	PS vs P	-0.149	0.120	0.461	0.861	0.327

putative K+dependent Na+exchanger related-protein

hypothetical proteins

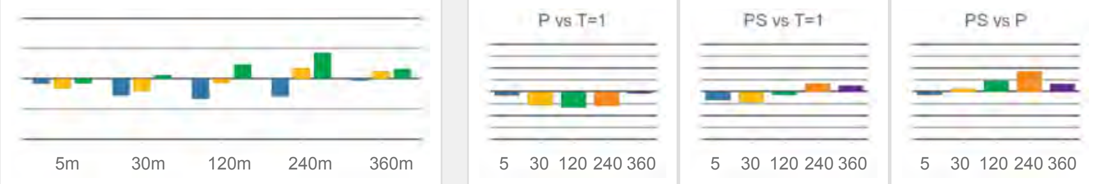

Locus		5m	30m	120m	240m	360m
**PGN_1312**	P vs T=1	-0.229	-0.415	-0.144	0.120	0.611
	PS vs T=1	-0.309	-0.346	-0.192	0.310	0.014
	PS vs P	-0.087	0.028	-0.065	0.214	-0.525

probable transcriptional regulator

regulatory functions

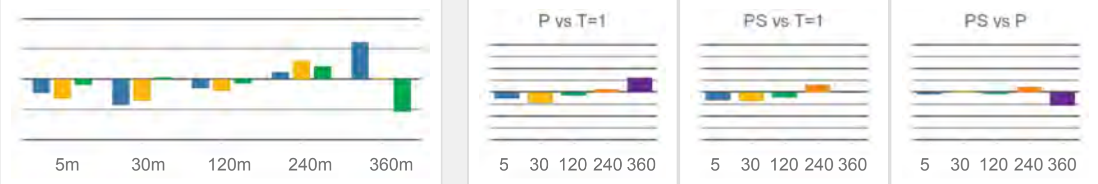

Locus		5m	30m	120m	240m	360m
**PGN_1313**	P vs T=1	0.739	0.197	-0.038	0.201	0.366
	PS vs T=1	0.013	-0.099	0.068	0.148	0.042
	PS vs P	-0.705	-0.292	0.099	-0.043	-0.312

conserved hypothetical protein

cell envelope

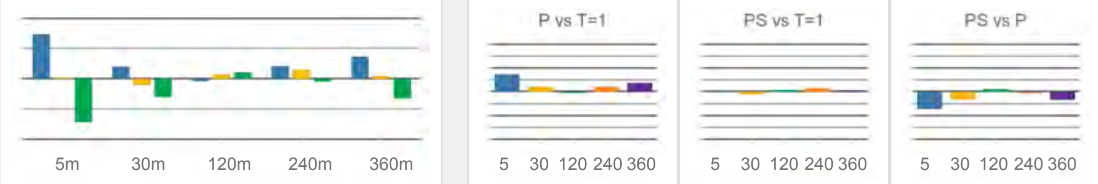

Locus		5m	30m	120m	240m	360m
**PGN_1314**	P vs T=1	0.626	0.539	0.512	0.485	0.361
	PS vs T=1	0.650	0.385	0.284	0.007	0.046
	PS vs P	0.033	-0.148	-0.221	-0.468	-0.312

ATP-dependent DNA helicase

DNA metabolism

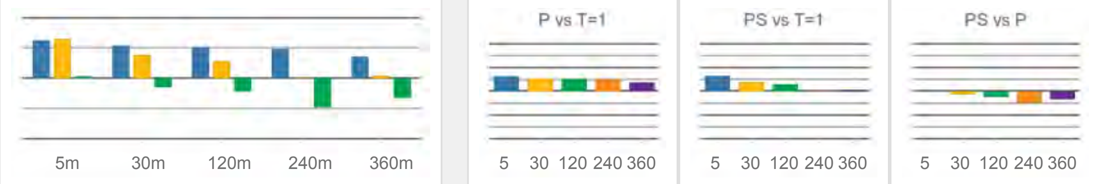

Locus		5m	30m	120m	240m	360m
**PGN_1315**	P vs T=1	0.526	0.695	0.645	0.371	-0.156
	PS vs T=1	0.194	0.792	0.838	0.162	-0.016
	PS vs P	-0.327	0.110	0.203	-0.208	0.125

conserved hypothetical protein

hypothetical proteins-Conserved

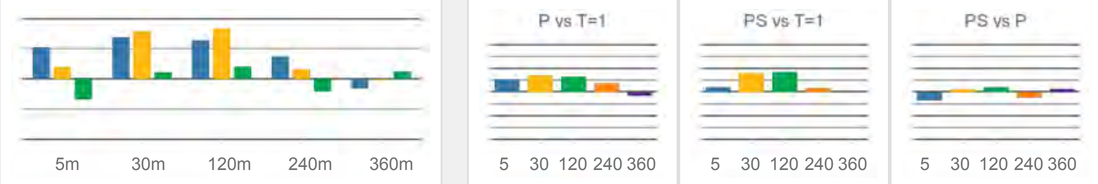

Locus		5m	30m	120m	240m	360m
**PGN_1316** *uvrAI*	P vs T=1	1.146	0.729	0.295	0.306	-0.248
	PS vs T=1	1.260	1.256	0.808	0.127	-0.143
	PS vs P	0.156	0.531	0.500	-0.174	0.083

excinuclease ABC A subunit

DNA metabolism

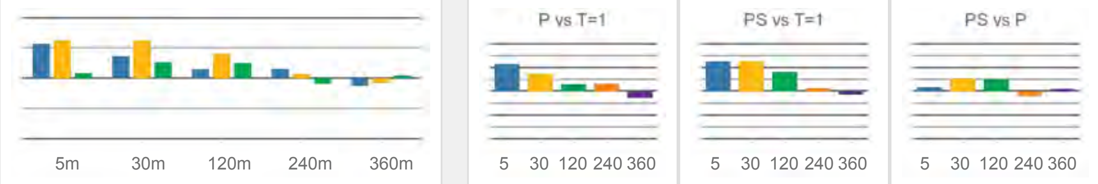

PgSg Time Course

log$_2$ (Fold Change)

Legend: P vs T=1 | PS vs T=1 | PS vs P

Locus		5m	30m	120m	240m	360m
**PGN_1317**	P vs T=1	-0.136	-0.508	-1.216	-1.290	-0.663
	PS vs T=1	0.311	-0.212	-0.890	-1.301	-1.262
	PS vs P	0.459	0.299	0.287	-0.063	-0.585

conserved hypothetical protein

hypothetical proteins-Conserved

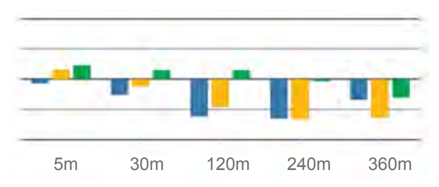

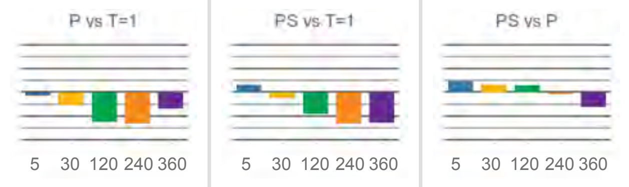

Locus		5m	30m	120m	240m	360m
**PGN_1318**	P vs T=1	-0.223	-0.581	-1.202	-1.402	-1.622
	PS vs T=1	-0.429	-0.640	-0.566	-1.060	-1.308
	PS vs P	-0.180	-0.049	0.609	0.296	0.271

putative ABC transporter ATP-binding protein

transport and binding proteins

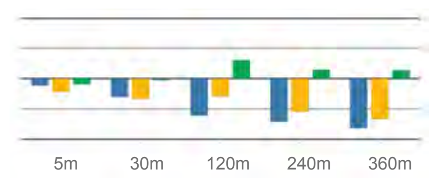

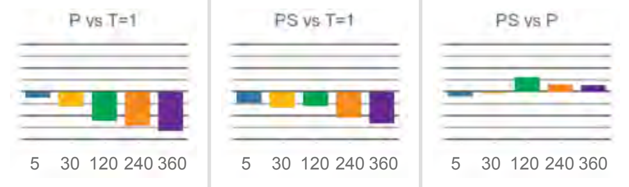

Locus		5m	30m	120m	240m	360m
**PGN_1319**	P vs T=1	0.357	0.257	0.375	0.552	0.164
	PS vs T=1	0.226	0.061	0.214	0.292	0.246
	PS vs P	-0.132	-0.203	-0.167	-0.254	0.068

transposase in ISPg1

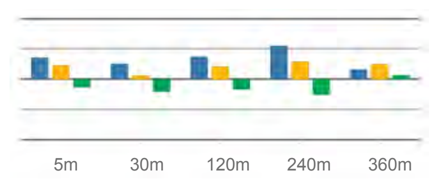

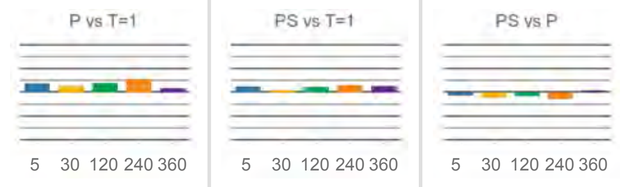

Locus		5m	30m	120m	240m	360m
**PGN_1320**	P vs T=1	-0.313	-0.947	-1.873	-2.009	-1.901
	PS vs T=1	-0.975	-1.017	-0.987	-0.931	-1.125
	PS vs P	-0.575	-0.059	0.765	0.921	0.695

conserved hypothetical protein

hypothetical proteins-Conserved

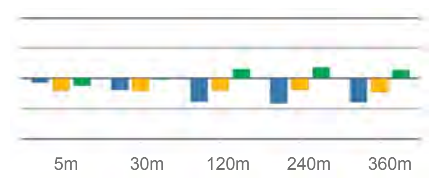

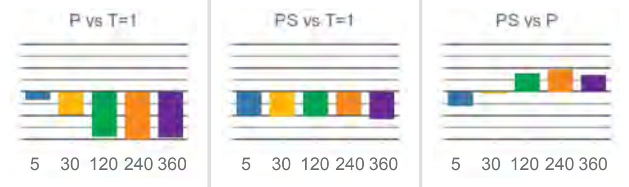

Locus		5m	30m	120m	240m	360m
**PGN_1321**	P vs T=1	0.037	-0.011	-0.096	-0.539	-1.225
	PS vs T=1	-0.693	-0.439	-0.639	-1.199	-1.395
	PS vs P	-0.672	-0.380	-0.498	-0.653	-0.213

conserved hypothetical protein

hypothetical proteins-Conserved

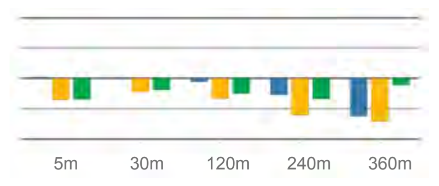

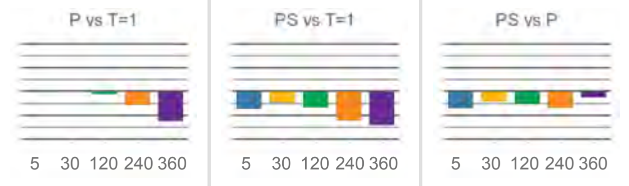

Locus		5m	30m	120m	240m	360m
**PGN_1322**	P vs T=1	0.334	0.413	0.346	1.819	2.707
	PS vs T=1	0.373	0.854	1.874	1.354	1.812
	PS vs P	-0.126	0.220	0.946	0.065	-0.189

conserved hypothetical protein

hypothetical proteins-Conserved

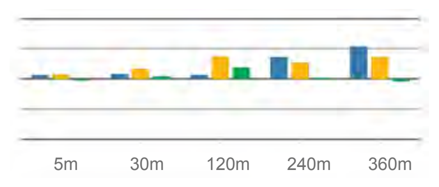

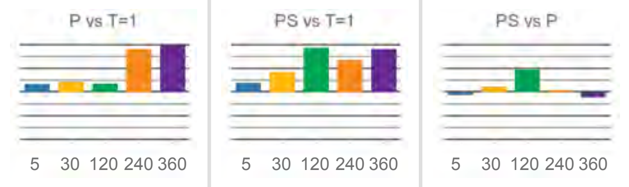

Locus		5m	30m	120m	240m	360m
**PGN_1323**	P vs T=1	0.259	0.284	-0.029	-0.732	-1.614
	PS vs T=1	-0.465	-0.210	-0.536	-1.224	-1.473
	PS vs P	-0.681	-0.447	-0.473	-0.494	0.103

TPR domain protein

unknown function

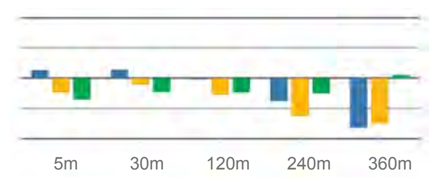

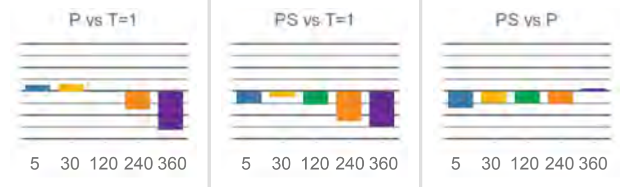

PgSg Time Course

log$_2$ (Fold Change)

P vs T=1 | PS vs T=1 | PS vs P

Locus		5m	30m	120m	240m	360m
**PGN_1324**	P vs T=1	-0.577	-0.741	-0.820	-0.314	0.361
	PS vs T=1	0.474	0.231	-0.346	-0.312	0.010
	PS vs P	1.001	0.893	0.354	0.003	-0.283

probable ABC transporter membrane protein

transport and binding proteins

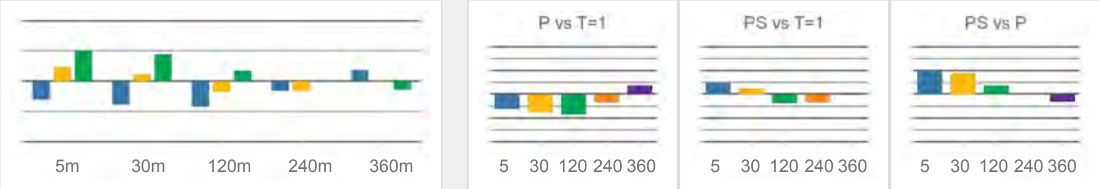

Locus		5m	30m	120m	240m	360m
**PGN_1325**	P vs T=1	-0.908	-1.712	-2.083	-1.804	-0.967
	PS vs T=1	-0.116	-0.551	-1.673	-2.319	-2.453
	PS vs P	0.807	1.113	0.319	-0.542	-1.420

probable ABC transporter membrane protein

transport and binding proteins

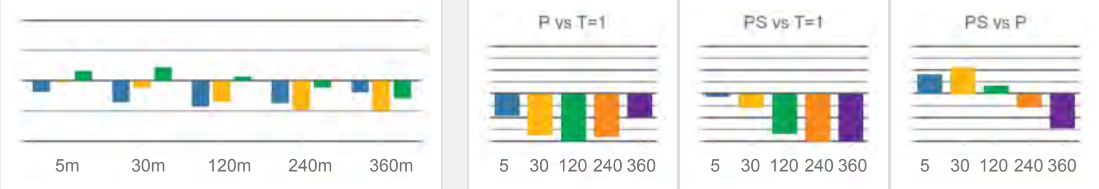

Locus		5m	30m	120m	240m	360m
**PGN_1326**	P vs T=1	-1.051	-2.123	-2.823	-3.058	-2.512
	PS vs T=1	-0.243	-0.510	-1.755	-2.635	-2.573
	PS vs P	0.879	1.442	0.853	0.166	-0.125

hypothetical protein

hypothetical proteins

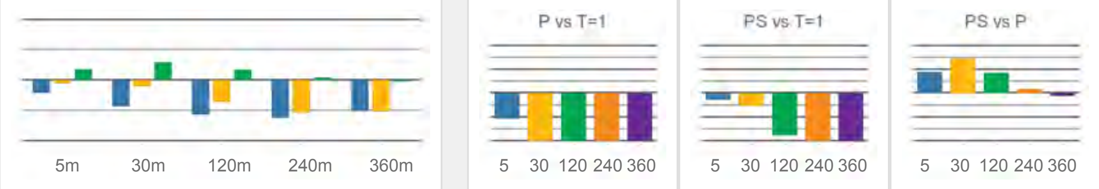

Locus		5m	30m	120m	240m	360m
**PGN_1327**	P vs T=1	-0.693	-1.363	-1.748	-2.201	-2.680
	PS vs T=1	-0.350	-0.448	-0.915	-1.995	-2.175
	PS vs P	0.398	0.862	0.785	0.108	0.383

hypothetical protein

hypothetical proteins

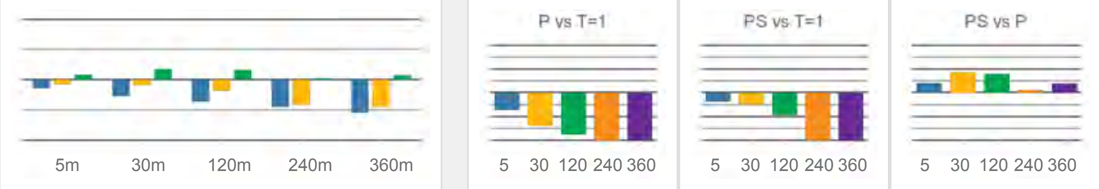

Locus		5m	30m	120m	240m	360m
**PGN_1328**	P vs T=1	-0.357	-0.920	-1.855	-2.371	-2.416
	PS vs T=1	-0.439	-0.400	-0.766	-1.747	-2.159
	PS vs P	0.016	0.533	1.005	0.468	0.163

hypothetical protein

hypothetical proteins

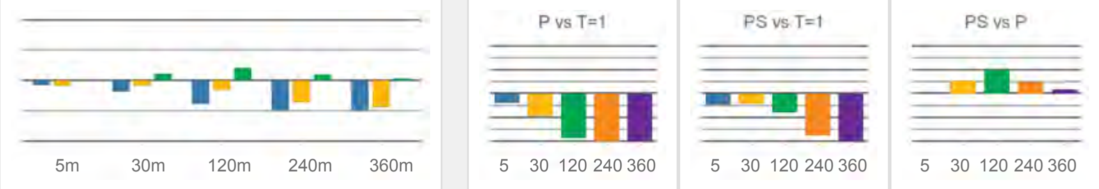

Locus		5m	30m	120m	240m	360m
**PGN_1329**	P vs T=1	0.430	-0.208	-1.023	-1.610	-1.354
	PS vs T=1	0.203	0.128	0.158	-0.407	-0.782
	PS vs P	-0.165	0.337	1.088	0.914	0.469

hypothetical protein

hypothetical proteins

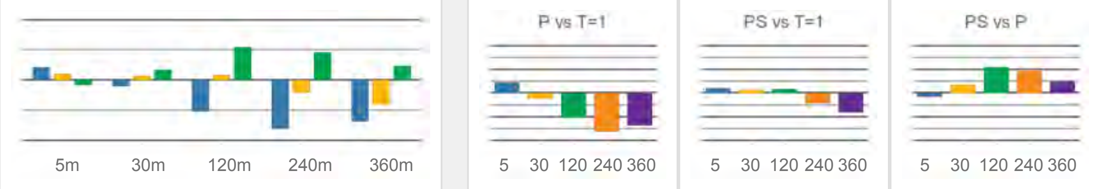

Locus		5m	30m	120m	240m	360m
**PGN_1330**	P vs T=1	-0.607	-1.325	-2.284	-2.730	-2.306
	PS vs T=1	-1.015	-1.132	-0.962	-1.459	-1.576
	PS vs P	-0.348	0.189	1.210	1.033	0.658

probable branched chain amino acid ABC transporter ATP-binding protein

transport and binding proteins

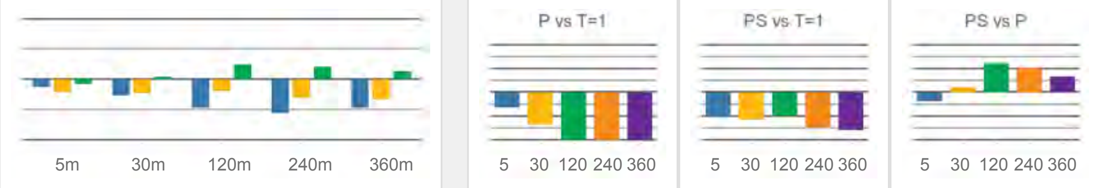

PgSg Time Course

log₂ (Fold Change)

Legend: P vs T=1 | PS vs T=1 | PS vs P

Locus		5m	30m	120m	240m	360m
PGN_1331	P vs T=1	0.955	0.750	1.432	2.205	3.902
	PS vs T=1	0.552	1.246	1.725	2.585	2.402
	PS vs P	-0.411	0.188	0.202	0.607	-0.740
	conserved hypothetical protein					
	hypothetical proteins-Conserved					
PGN_1332	P vs T=1	-0.244	-0.390	-0.126	0.794	1.476
	PS vs T=1	-0.533	-0.314	0.341	0.754	0.583
	PS vs P	-0.305	0.007	0.379	0.112	-0.732
	putative para-aminobenzoate synthase component I					
	biosynthesis of cofactors, prosthetic groups, and carriers					
PGN_1333	P vs T=1	-0.650	-0.962	-0.874	0.055	1.403
	PS vs T=1	-1.492	-1.357	-0.381	0.169	0.420
	PS vs P	-0.794	-0.487	0.230	0.269	-0.613
	probable para-aminobenzoate synthase component I					
	biosynthesis of cofactors, prosthetic groups, and carriers					
PGN_1334	P vs T=1	0.426	0.024	0.547	2.334	3.614
	PS vs T=1	-0.250	-0.351	0.642	1.341	1.978
	PS vs P	-0.645	-0.523	-0.115	-0.560	-1.270
	conserved hypothetical protein					
	hypothetical proteins-Conserved					
PGN_1335	P vs T=1	1.059	0.885	1.603	2.932	4.464
	PS vs T=1	0.480	0.506	1.335	1.960	2.698
	PS vs P	-0.570	-0.456	-0.290	-0.671	-1.448
	conserved hypothetical protein					
	hypothetical proteins-Conserved					
PGN_1336	P vs T=1	0.993	0.464	1.023	1.705	2.631
	PS vs T=1	0.079	0.052	0.700	0.982	1.711
	PS vs P	-0.831	-0.469	-0.315	-0.539	-0.722
	conserved hypothetical protein					
	hypothetical proteins-Conserved					
PGN_1337	P vs T=1	-0.044	0.679	1.504	1.702	1.428
	PS vs T=1	0.053	0.274	0.674	1.142	1.178
	PS vs P	-0.060	-0.390	-0.681	-0.380	-0.181
	conserved hypothetical protein					
	hypothetical proteins-Conserved					

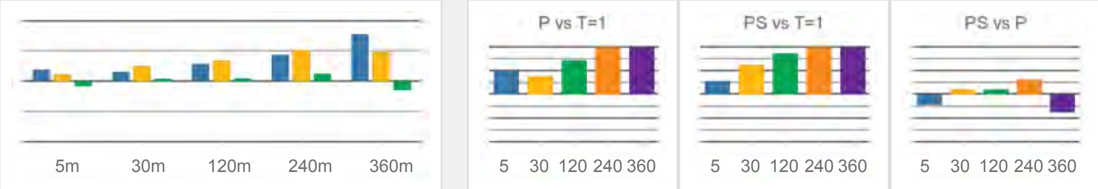

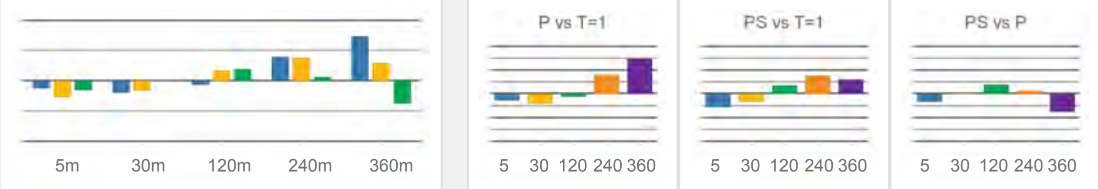

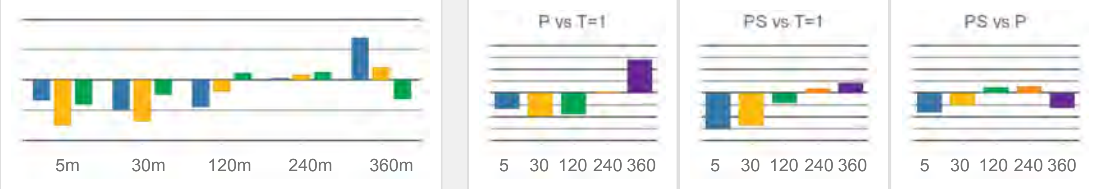

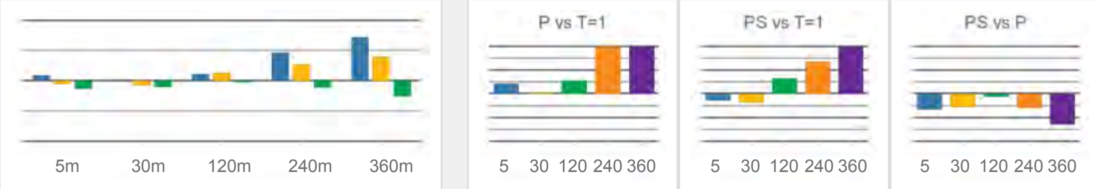

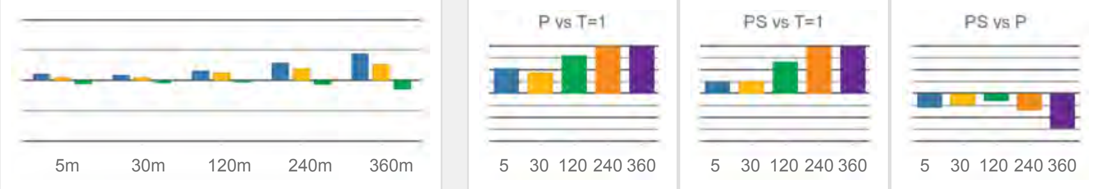

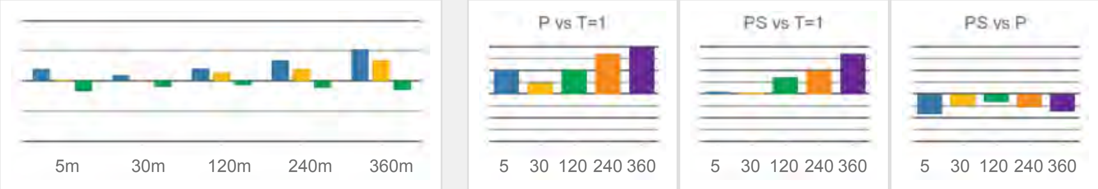

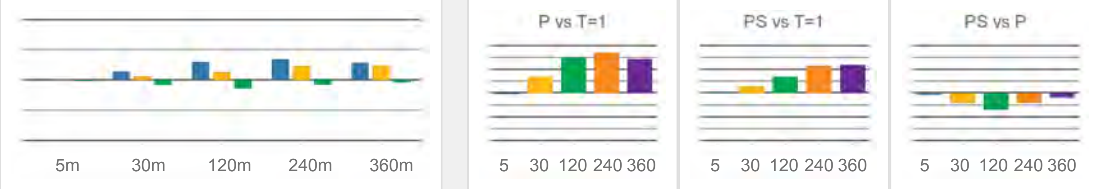

PgSg Time Course

Hackett Laboratory UW

log2 (Fold Change)

Locus		5m	30m	120m	240m	360m
PGN_1338	P vs T=1	0.214	0.661	1.111	1.079	0.441
	PS vs T=1	0.318	0.244	0.077	-0.009	0.053
	PS vs P	0.089	-0.396	-0.988	-1.046	-0.388
	pyruvate phosphate dikinase					
	energy metabolism					
PGN_1339	P vs T=1	-1.406	-1.721	-1.641	-1.594	-1.208
	PS vs T=1	-1.585	-1.647	-1.293	-1.497	-1.089
	PS vs P	-0.192	-0.026	0.239	-0.042	0.112
	conserved hypothetical protein					
	hypothetical proteins-Conserved					
PGN_1340	P vs T=1	-0.923	-1.996	-3.009	-3.004	-2.413
	PS vs T=1	-1.176	-1.264	-1.042	-1.308	-1.710
	PS vs P	-0.178	0.673	1.792	1.524	0.657
	conserved hypothetical protein					
	unknown function					
PGN_1341	P vs T=1	0.723	0.832	0.868	0.520	-0.276
	PS vs T=1	0.148	0.337	0.425	0.330	0.164
	PS vs P	-0.542	-0.453	-0.401	-0.176	0.407
	putative CoA transferase					
	central intermediary metabolism					
PGN_1342	P vs T=1	-0.128	-0.412	-0.319	-0.351	-0.411
	PS vs T=1	-0.515	-1.081	-1.303	-1.177	-1.103
	PS vs P	-0.359	-0.654	-0.953	-0.788	-0.673
	putative SAM/TRAM family methylase protein					
	hypothetical proteins-Conserved					
PGN_1343	P vs T=1	0.112	0.200	0.038	0.091	0.623
	PS vs T=1	-0.122	0.406	0.926	1.107	1.119
	PS vs P	-0.243	0.197	0.843	0.955	0.503
	probable ABC transporter ATP-binding protein					
	transport and binding proteins					
PGN_1344	P vs T=1	-0.293	-0.037	0.012	0.026	0.091
	PS vs T=1	-0.466	-0.036	0.468	0.685	0.672
	PS vs P	-0.208	-0.006	0.432	0.616	0.561
	conserved hypothetical protein					
	hypothetical proteins-Conserved					

P vs T=1 PS vs T=1 PS vs P

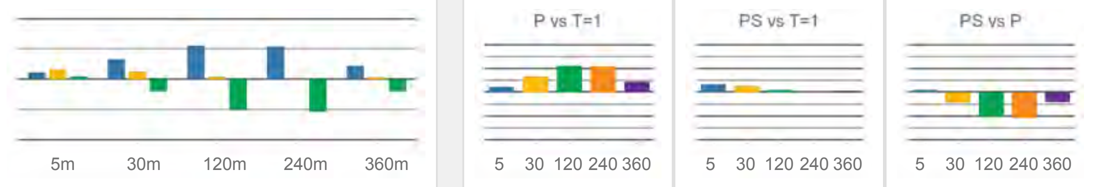

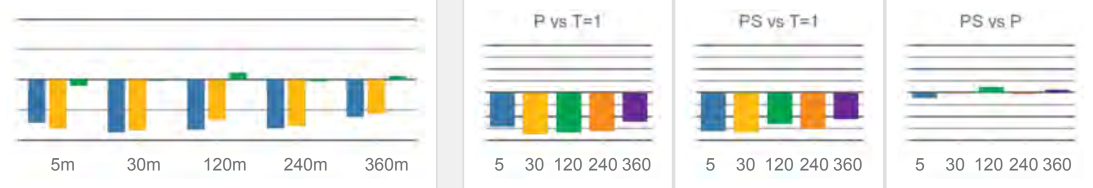

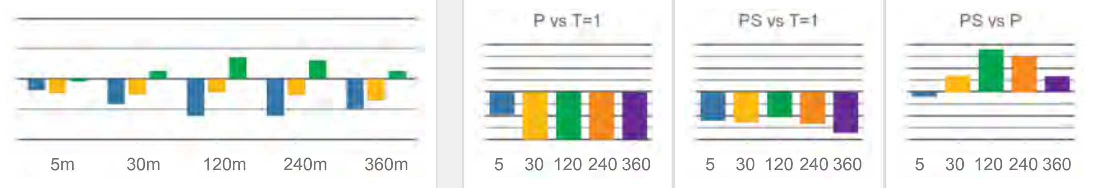

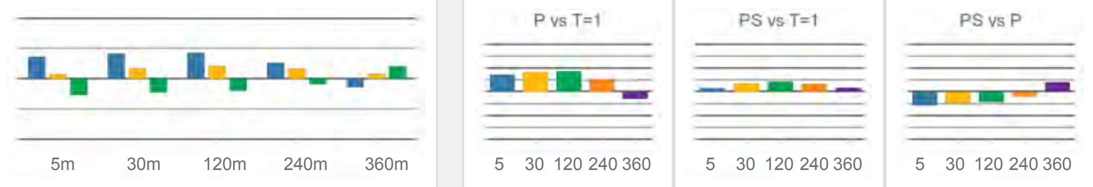

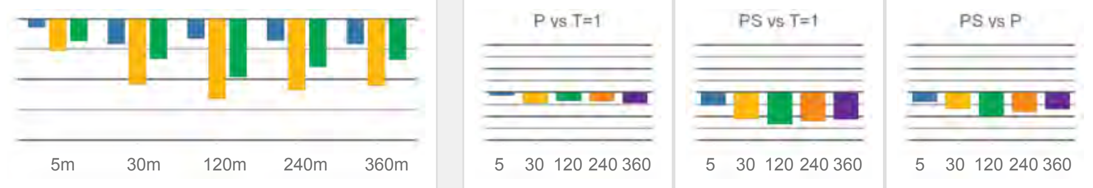

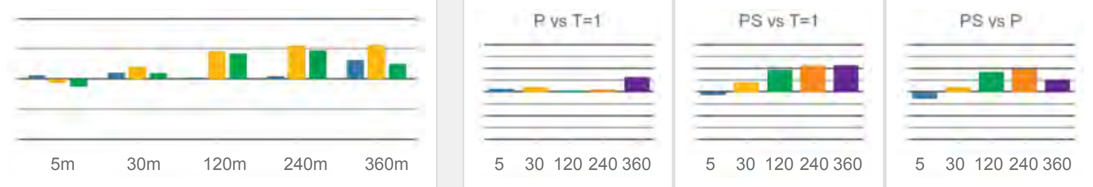

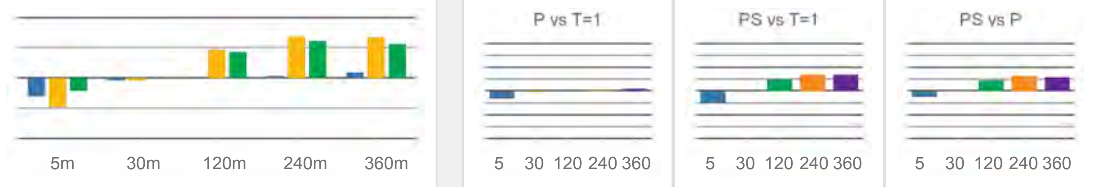

PgSg RNA Seq

PgSg Time Course

Hackett Laboratory UW

log2 (Fold Change)

P vs T=1 | PS vs T=1 | PS vs P

Locus		5m	30m	120m	240m	360m
**PGN_1345**	P vs T=1	-0.040	0.121	0.256	0.547	1.258
	PS vs T=1	-0.091	0.030	0.502	0.799	0.754
	PS vs P	-0.088	-0.107	0.211	0.267	-0.399

conserved hypothetical protein

hypothetical proteins-Conserved

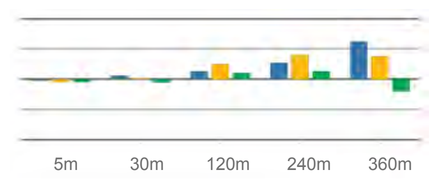

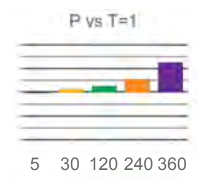

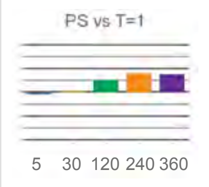

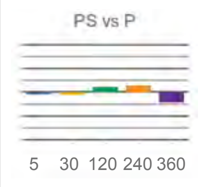

Locus		5m	30m	120m	240m	360m
**PGN_1346**	P vs T=1	-0.180	0.279	0.734	0.749	0.983
	PS vs T=1	0.187	0.253	0.363	0.880	0.827
	PS vs P	0.257	-0.041	-0.296	0.177	-0.075

putative transcriptional regulator

regulatory functions

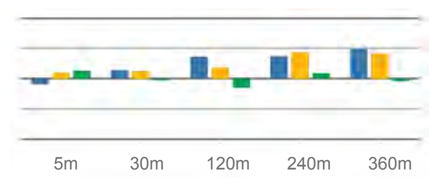

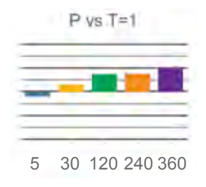

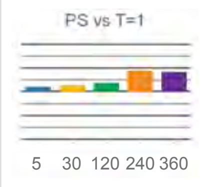

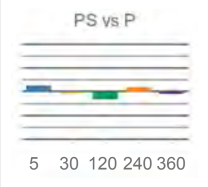

Locus		5m	30m	120m	240m	360m
**PGN_1347**	P vs T=1	0.142	0.086	0.308	0.846	1.439
	PS vs T=1	0.016	-0.102	0.026	0.111	0.694
	PS vs P	-0.129	-0.199	-0.289	-0.680	-0.689

putative TonB-dependent receptor exported protein

hypothetical proteins

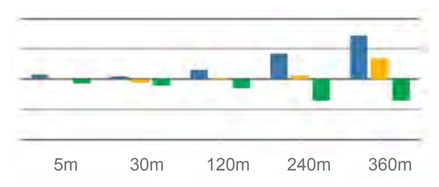

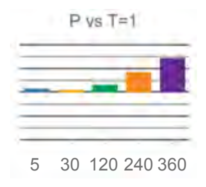

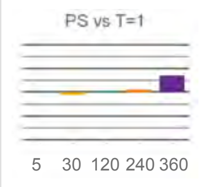

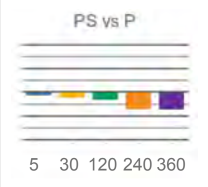

Locus		5m	30m	120m	240m	360m
**PGN_1348**	P vs T=1	-0.267	0.225	0.584	0.766	1.131
	PS vs T=1	-0.258	0.000	-0.051	0.304	0.510
	PS vs P	-0.055	-0.213	-0.577	-0.369	-0.523

conserved hypothetical protein

hypothetical proteins-Conserved

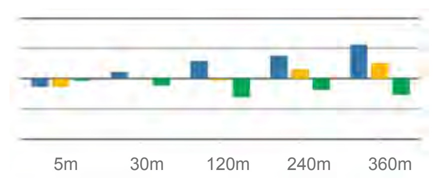

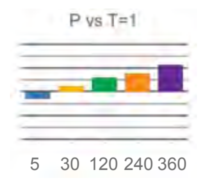

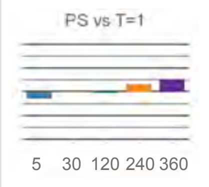

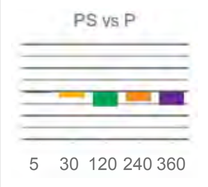

Locus		5m	30m	120m	240m	360m
**PGN_1349**	P vs T=1	0.623	1.141	1.785	1.754	1.369
	PS vs T=1	1.213	1.265	1.358	1.419	1.148
	PS vs P	0.506	0.124	-0.343	-0.265	-0.205

probable dipeptidyl anminopeptidase

protein fate

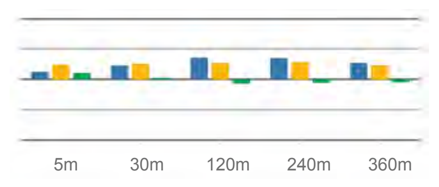

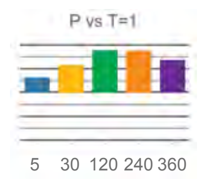

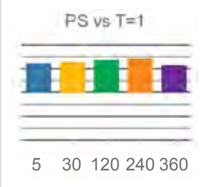

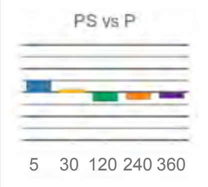

Locus		5m	30m	120m	240m	360m
**PGN_1350**	P vs T=1	0.386	0.305	0.271	0.114	-0.163
	PS vs T=1	0.418	0.785	1.134	0.726	0.656
	PS vs P	0.034	0.471	0.856	0.588	0.787

conserved hypothetical protein

hypothetical proteins-Conserved

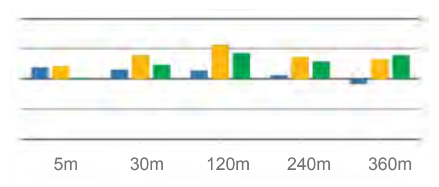

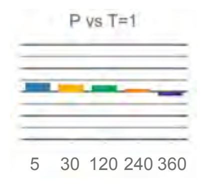

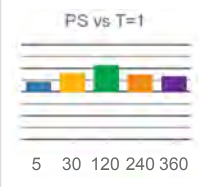

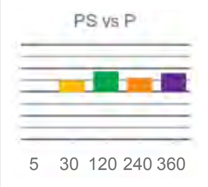

Locus		5m	30m	120m	240m	360m
**PGN_1351**	P vs T=1	0.344	0.044	-0.508	-0.401	0.216
	PS vs T=1	0.703	0.709	0.394	-0.400	-0.262
	PS vs P	0.379	0.646	0.756	-0.100	-0.406

conserved hypothetical protein

hypothetical proteins-Conserved

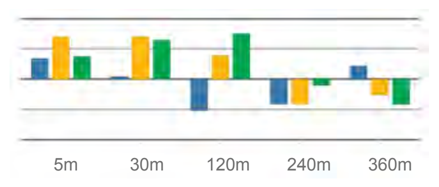

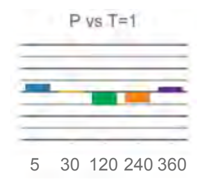

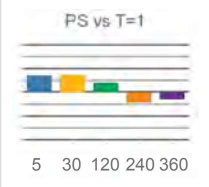

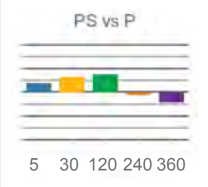

PgSg Time Course

Hackett Laboratory UW

log$_2$ (Fold Change)

Locus		5m	30m	120m	240m	360m
**PGN_1352**	P vs T=1	0.285	-0.022	-0.258	-0.331	0.048
	PS vs T=1	0.300	0.444	-0.044	-0.431	-0.267
	PS vs P	0.038	0.453	0.178	-0.154	-0.286
	conserved hypothetical protein with DUF1706 domain					
	hypothetical proteins-Conserved					
**PGN_1353**	P vs T=1	0.822	0.278	-0.084	-0.109	0.975
	PS vs T=1	0.834	0.507	0.356	-0.379	-0.187
	PS vs P	0.045	0.216	0.365	-0.362	-1.046
	conserved hypothetical protein					
	hypothetical proteins-Conserved					
**PGN_1354**	P vs T=1	1.406	0.853	-0.212	-0.996	-1.513
	PS vs T=1	0.772	0.604	0.115	-0.314	-0.612
	PS vs P	-0.549	-0.201	0.316	0.601	0.819
	conserved hypothetical protein					
	hypothetical proteins-Conserved					
**PGN_1355**	P vs T=1	1.806	1.490	0.636	-0.218	-0.940
	PS vs T=1	1.227	1.182	0.712	-0.006	-0.409
	PS vs P	-0.352	-0.141	0.116	0.083	0.327
	conserved hypothetical protein					
	hypothetical proteins-Conserved					
**PGN_1356**	P vs T=1	1.234	0.983	-0.217	-0.938	-1.378
	PS vs T=1	0.752	0.736	0.168	-0.483	-0.828
	PS vs P	-0.347	-0.136	0.354	0.321	0.421
	conserved hypothetical protein					
	hypothetical proteins-Conserved					
**PGN_1357**	P vs T=1	0.259	0.108	-0.599	-1.193	-1.422
	PS vs T=1	-0.322	-0.267	-0.709	-0.910	-0.859
	PS vs P	-0.491	-0.297	-0.107	0.187	0.483
	conserved hypothetical protein					
	hypothetical proteins-Conserved					
**PGN_1358**	P vs T=1	0.044	0.233	0.531	0.612	-0.036
	PS vs T=1	0.308	0.588	0.349	0.106	0.021
	PS vs P	0.233	0.340	-0.161	-0.469	0.031
	conserved hypothetical protein					
	hypothetical proteins-Conserved					

P vs T=1 | PS vs T=1 | PS vs P

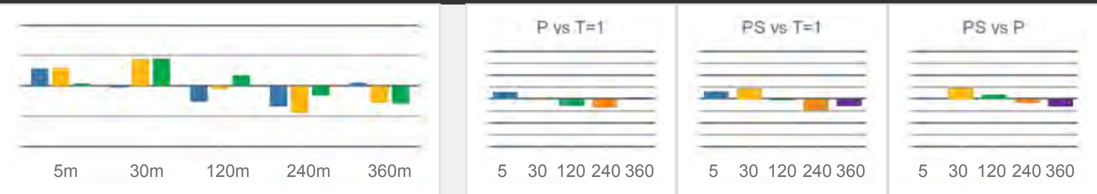

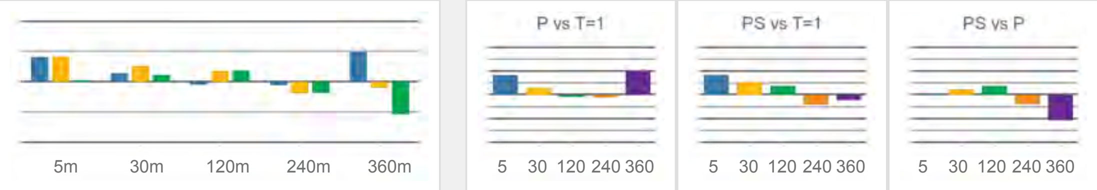

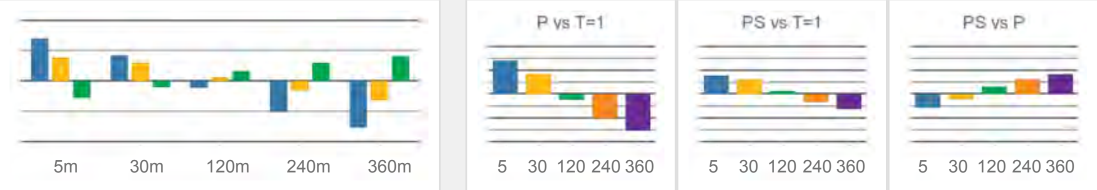

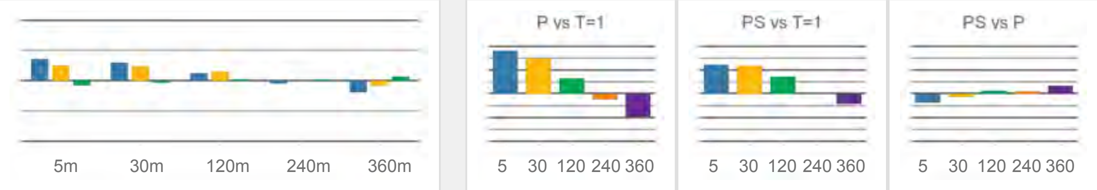

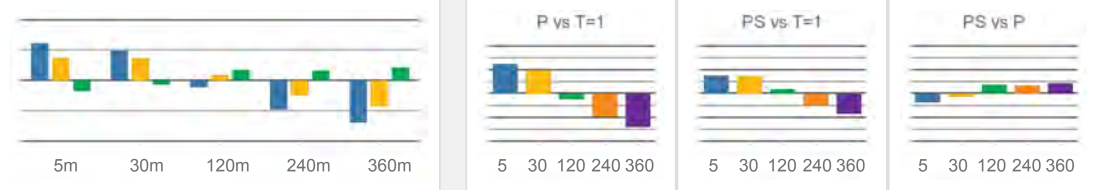

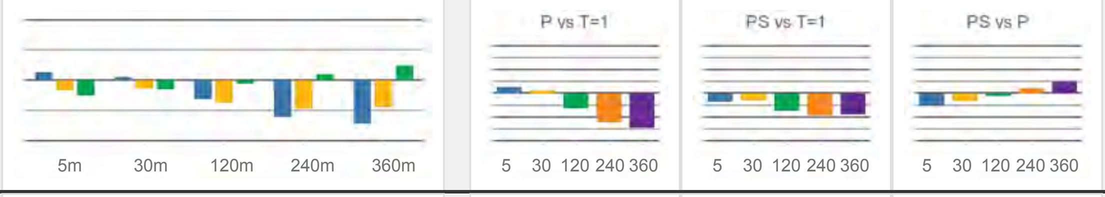

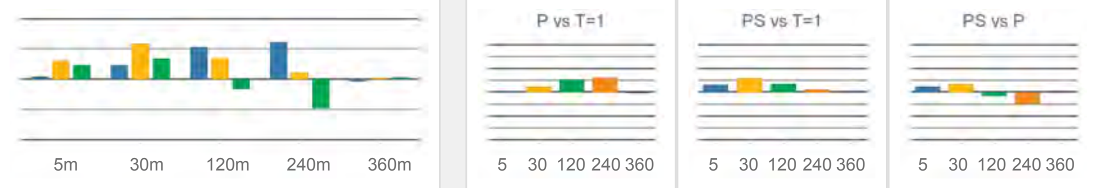

PgSg Time Course

Hackett Laboratory UW

log2 (Fold Change)

P vs T=1 | PS vs T=1 | PS vs P

Locus		5m	30m	120m	240m	360m
**PGN_1359**	P vs T=1	-0.067	0.356	0.839	1.007	0.382
PdxB	PS vs T=1	0.380	0.471	0.312	0.563	0.477
	PS vs P	0.380	0.102	-0.488	-0.385	0.076

putative erythronate-4-phosphate dehydrogenase

biosynthesis of cofactors, prosthetic groups, and carriers

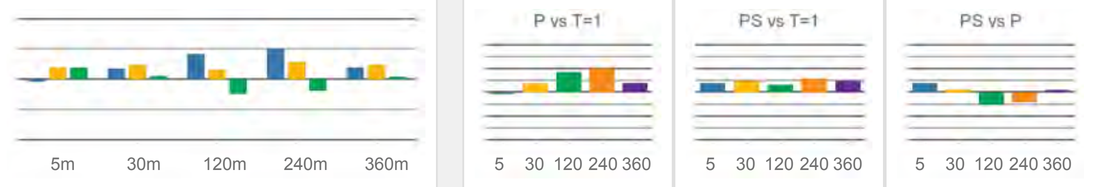

Locus		5m	30m	120m	240m	360m
**PGN_1360**	P vs T=1	0.114	0.620	1.170	1.090	1.041
	PS vs T=1	0.352	0.433	0.583	0.994	0.736
	PS vs P	0.195	-0.181	-0.533	-0.059	-0.281

probable oxidoreductase

unknown function

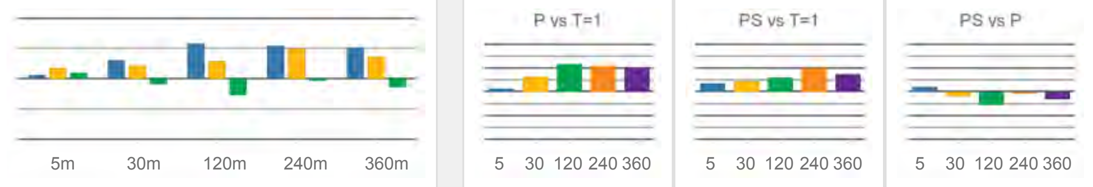

Locus		5m	30m	120m	240m	360m
**PGN_1361**	P vs T=1	-1.976	-2.160	-1.923	-1.493	-1.914
	PS vs T=1	-2.361	-2.504	-2.041	-1.820	-1.610
	PS vs P	-0.399	-0.398	-0.146	-0.242	0.254

conserved hypothetical protein

hypothetical proteins-Conserved

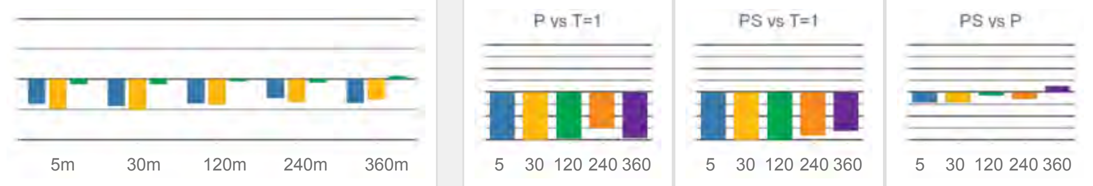

Locus		5m	30m	120m	240m	360m
**PGN_1362**	P vs T=1	-0.268	-0.337	-0.726	-1.302	-1.472
	PS vs T=1	-0.573	-0.823	-0.931	-0.946	-0.983
	PS vs P	-0.271	-0.450	-0.197	0.290	0.439

probable exported transglycosylase protein

unknown function

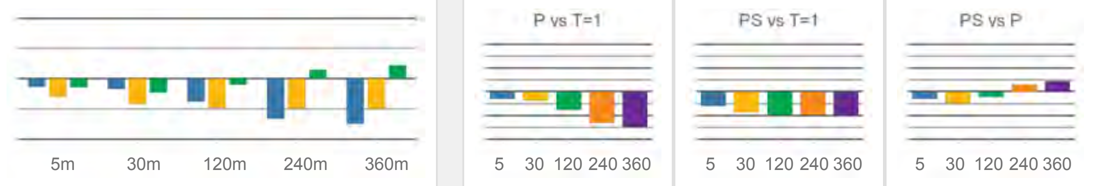

Locus		5m	30m	120m	240m	360m
**PGN_1363**	P vs T=1	-0.582	-0.409	-0.368	-0.311	0.076
wzt	PS vs T=1	-0.132	-0.284	-0.661	-0.749	-0.660
	PS vs P	0.408	0.118	-0.288	-0.421	-0.682

ABC transporter ATP-binding protein

transport and binding proteins

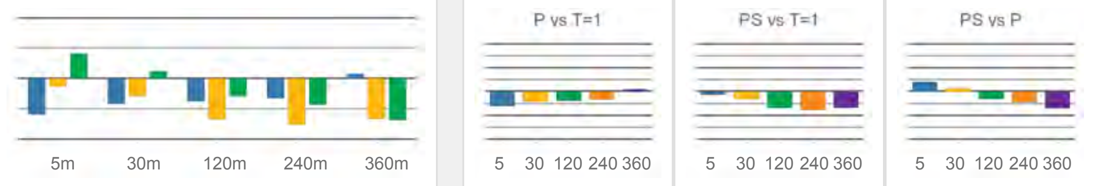

Locus		5m	30m	120m	240m	360m
**PGN_1364**	P vs T=1	-0.763	-0.296	0.439	0.671	0.129
	PS vs T=1	-0.399	0.178	0.294	0.251	0.202
	PS vs P	0.277	0.432	-0.109	-0.357	0.065

probable peptidyl-prolyl cis-trans isomerase cyclophilin-type

protein fate

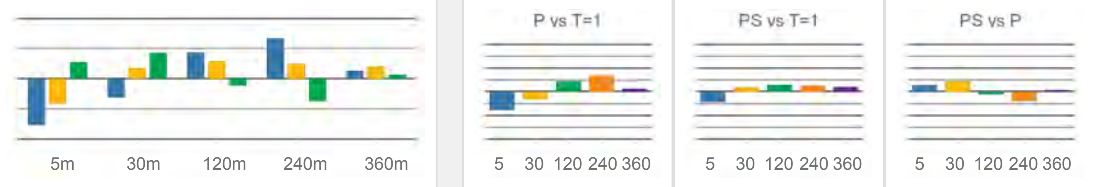

Locus		5m	30m	120m	240m	360m
**PGN_1365**	P vs T=1	-0.341	0.140	0.247	0.589	0.993
	PS vs T=1	0.189	0.729	0.529	0.575	0.272
	PS vs P	0.373	0.546	0.238	0.074	-0.552

conserved hypothetical protein

hypothetical proteins-Conserved

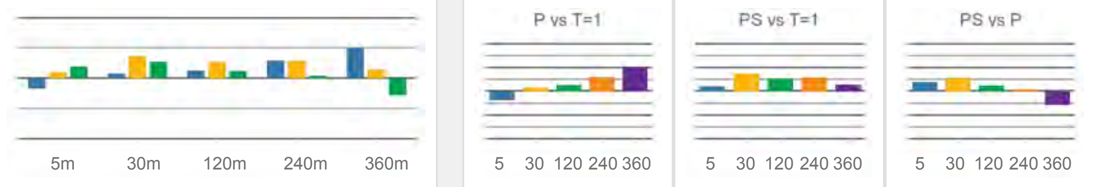

PgSg Time Course

log$_2$ (Fold Change)

Legend: P vs T=1 | PS vs T=1 | PS vs P

Locus		5m	30m	120m	240m	360m
PGN_1366	P vs T=1	0.243	0.761	0.659	0.200	-0.203
	PS vs T=1	0.721	0.644	0.524	0.494	0.228
	PS vs P	0.464	-0.106	-0.127	0.280	0.411

conserved hypothetical protein

hypothetical proteins-Conserved

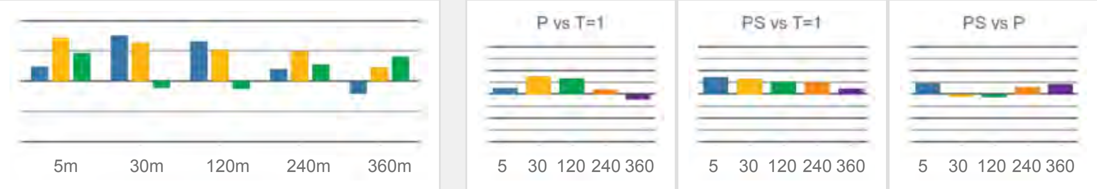

Locus		5m	30m	120m	240m	360m
PGN_1367	P vs T=1	-0.100	0.380	0.593	0.386	-0.167
	PS vs T=1	0.298	0.455	0.622	0.566	0.285
	PS vs P	0.366	0.082	0.047	0.183	0.432

NAD-specific glutamate dehydrogenase

energy metabolism

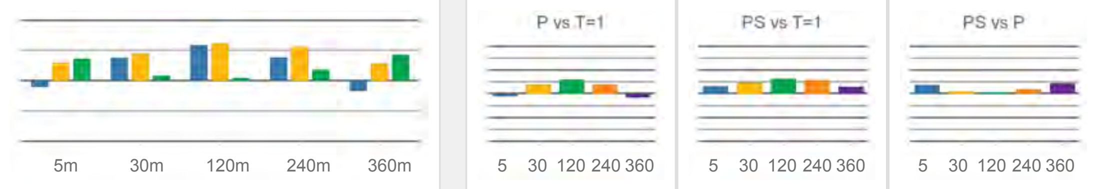

Locus		5m	30m	120m	240m	360m
PGN_1368	P vs T=1	-0.649	-0.864	-0.860	0.172	2.140
	PS vs T=1	-0.929	-1.246	-0.447	-0.368	0.327
	PS vs P	-0.349	-0.524	-0.081	-0.281	-0.673

conserved hypothetical protein

hypothetical proteins-Conserved

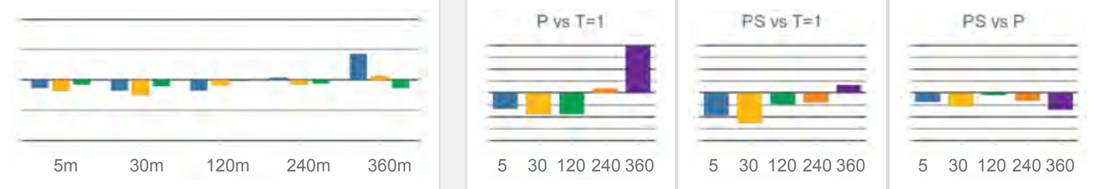

Locus		5m	30m	120m	240m	360m
PGN_1369	P vs T=1	-0.611	-0.359	0.191	0.403	0.057
	PS vs T=1	-0.996	-1.034	-0.417	-0.562	-0.231
	PS vs P	-0.406	-0.641	-0.483	-0.748	-0.231

conserved hypothetical protein

hypothetical proteins-Conserved

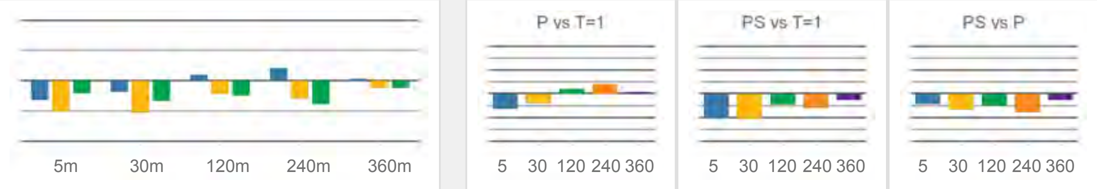

Locus		5m	30m	120m	240m	360m
PGN_1370	P vs T=1	0.489	0.501	0.641	0.552	0.439
	PS vs T=1	0.012	0.100	0.543	1.007	1.044
	PS vs P	-0.466	-0.390	-0.085	0.454	0.600

NAD-dependent nucleotide-diphosphate-sugar epimerase

unknown function

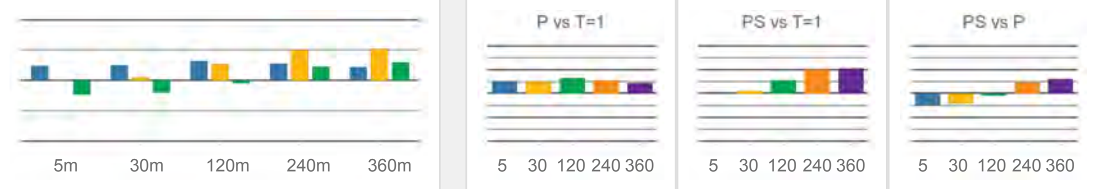

Locus		5m	30m	120m	240m	360m
PGN_1371	P vs T=1	0.879	0.767	1.632	0.896	2.902
	PS vs T=1	0.290	0.437	1.014	2.085	1.510
	PS vs P	-0.453	-0.322	-0.171	0.703	-0.415

hypothetical protein

hypothetical proteins

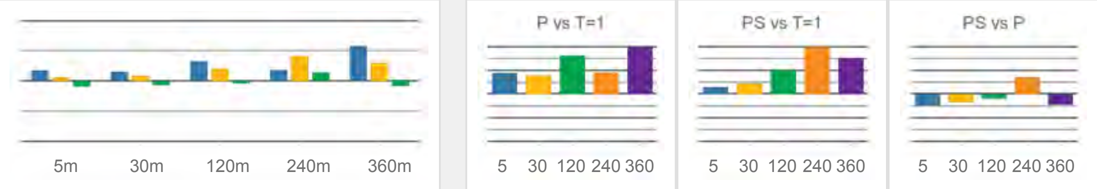

Locus		5m	30m	120m	240m	360m
PGN_1372	P vs T=1	-0.280	-0.651	-1.058	-1.202	-1.343
	PS vs T=1	-1.387	-1.313	-0.625	-0.473	-0.708
	PS vs P	-1.013	-0.627	0.389	0.655	0.569

conserved hypothetical protein

hypothetical proteins-Conserved

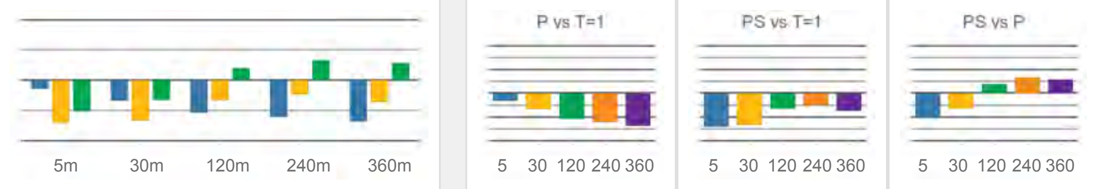

log$_2$ (Fold Change)

Locus		5m	30m	120m	240m	360m
**PGN_1373**	P vs T=1	0.362	-0.362	-0.902	-0.980	-1.001
cdhR	PS vs T=1	-0.575	-0.594	-0.334	-0.320	-0.437
	PS vs P	-0.845	-0.222	0.514	0.594	0.522
	probable transcriptional regulator					
	regulatory functions					
**PGN_1374**	P vs T=1	-0.013	-0.428	-0.935	-1.060	-0.765
	PS vs T=1	-0.268	-0.049	0.110	-0.179	-0.474
	PS vs P	-0.211	0.345	0.924	0.721	0.242
	putative ribosomal large subunit pseudouridine synthase D					
	protein synthesis					
**PGN_1375**	P vs T=1	0.487	0.542	0.341	-0.279	-1.088
	PS vs T=1	0.435	0.997	0.960	0.272	-0.106
	PS vs P	-0.040	0.467	0.629	0.494	0.876
	putative beta-ketoacyl-acyl carrier protein reductase					
	fatty acid and phospholipid metabolism					
**PGN_1376**	P vs T=1	0.256	-0.275	-1.052	-1.439	-1.450
	PS vs T=1	0.476	0.881	0.908	-0.082	-0.416
	PS vs P	0.258	1.094	1.797	1.110	0.883
	putative transcriptional regulator					
	regulatory functions					
**PGN_1377**	P vs T=1	-0.958	-1.320	-1.661	-1.448	-0.978
	PS vs T=1	-1.476	-1.549	-1.450	-1.102	-0.993
	PS vs P	-0.501	-0.232	0.173	0.325	-0.003
	GTP-binding protein LepA					
	unknown function					
**PGN_1378**	P vs T=1	-0.776	-0.861	-0.739	-0.454	0.129
	PS vs T=1	-0.787	-1.027	-0.877	-0.419	-0.316
	PS vs P	-0.023	-0.186	-0.163	0.038	-0.406
	replicative DNA helicase					
	DNA metabolism					
**PGN_1379**	P vs T=1	-0.324	-1.305	-1.576	-0.876	-0.838
	PS vs T=1	-1.323	-1.489	-0.923	-1.236	-1.101
	PS vs P	-0.783	-0.271	0.410	-0.244	-0.215
	hypothetical protein					
	hypothetical proteins					

P vs T=1 | PS vs T=1 | PS vs P

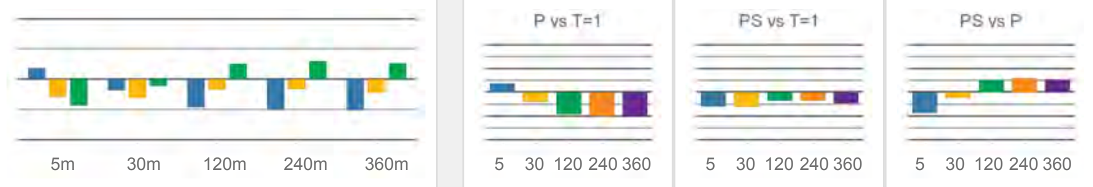

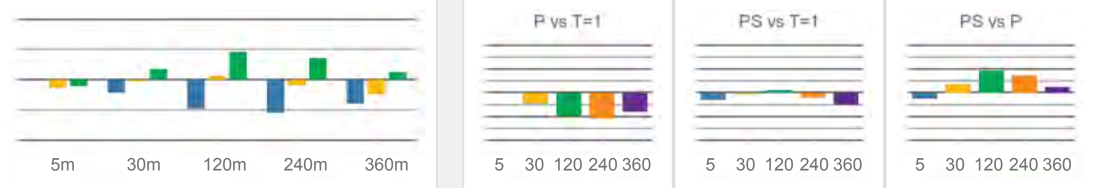

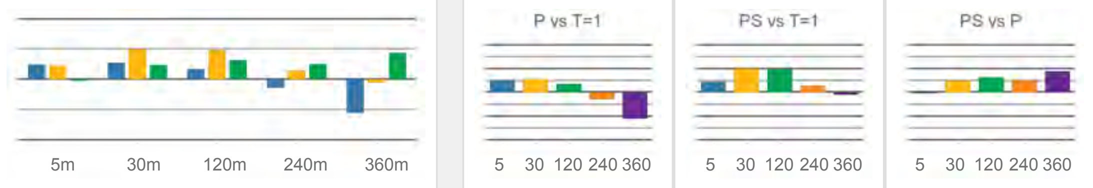

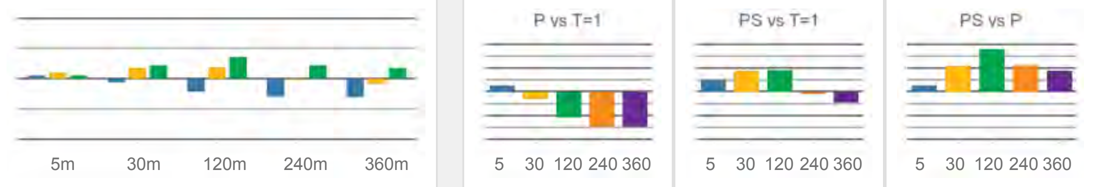

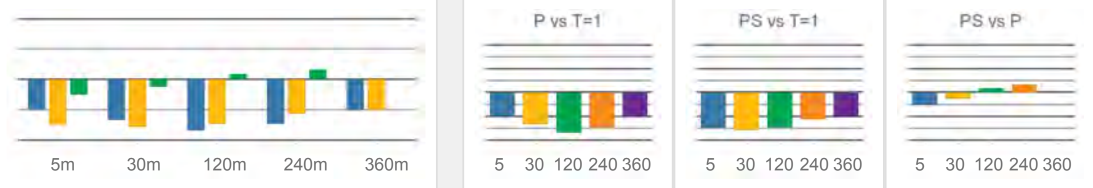

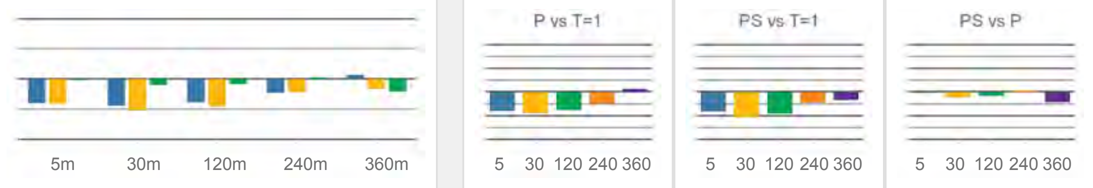

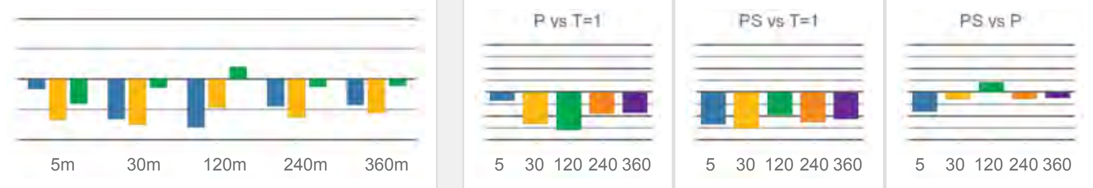

PgSg Time Course

log2 (Fold Change)

Legend: P vs T=1 · PS vs T=1 · PS vs P

Locus		5m	30m	120m	240m	360m
PGN_1380	P vs T=1	1.278	0.191	0.148	0.817	0.849
	PS vs T=1	0.261	0.020	0.680	0.786	0.105
	PS vs P	-0.704	-0.250	0.369	0.187	-0.535
	hypothetical protein					
	hypothetical proteins					
PGN_1381	P vs T=1	0.061	0.488	0.739	0.591	0.348
	PS vs T=1	-0.358	-0.423	-0.537	-0.490	-0.327
	PS vs P	-0.415	-0.880	-1.237	-1.049	-0.663
	alanyl-tRNA synthetase					
	protein synthesis					
PGN_1382	P vs T=1	-0.303	-0.504	-0.543	-0.591	-0.452
	PS vs T=1	-0.315	-0.919	-1.377	-1.421	-1.555
	PS vs P	0.010	-0.399	-0.806	-0.792	-1.061
	putative 3-dehydroquinate synthase					
	amino acid biosynthesis					
PGN_1383	P vs T=1	-0.238	-0.666	-0.315	0.345	1.042
	PS vs T=1	-0.240	-0.314	0.908	1.356	1.128
	PS vs P	-0.021	0.235	1.093	1.018	0.155
	conserved hypothetical protein					
	hypothetical proteins-Conserved					
PGN_1384	P vs T=1	-2.097	-2.044	-1.588	-1.092	-0.816
	PS vs T=1	-1.709	-1.224	-0.304	0.141	0.061
	PS vs P	0.224	0.638	1.145	1.166	0.848
	probable 1-acyl-sn-glycerol-3-phosphate acetyltransferase					
	fatty acid and phospholipid metabolism					
PGN_1385	P vs T=1	0.832	0.926	0.802	0.696	0.151
	PS vs T=1	-0.059	0.672	1.303	1.822	1.873
	PS vs P	-0.858	-0.220	0.499	1.103	1.662
	hypothetical protein					
	hypothetical proteins					
PGN_1386	P vs T=1	0.929	0.805	0.901	1.621	2.023
	PS vs T=1	0.664	0.669	0.646	0.859	1.266
	PS vs P	-0.222	-0.130	-0.271	-0.485	-0.527
	conserved hypothetical protein					
	hypothetical proteins-Conserved					

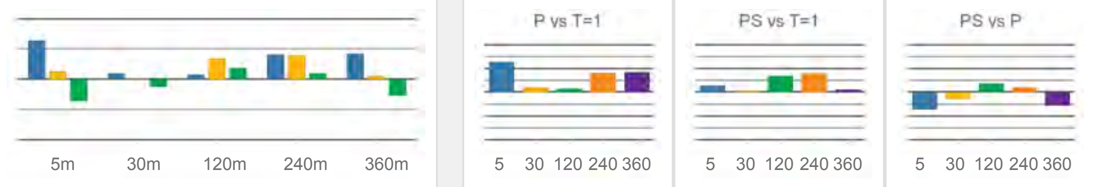

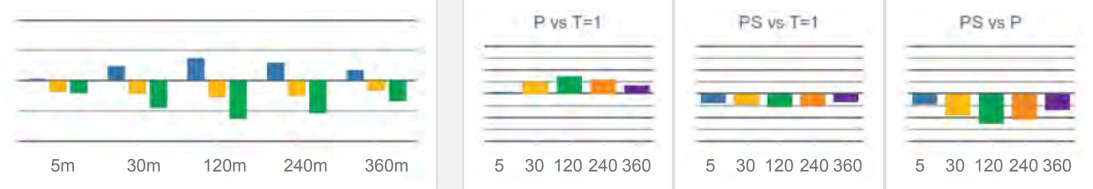

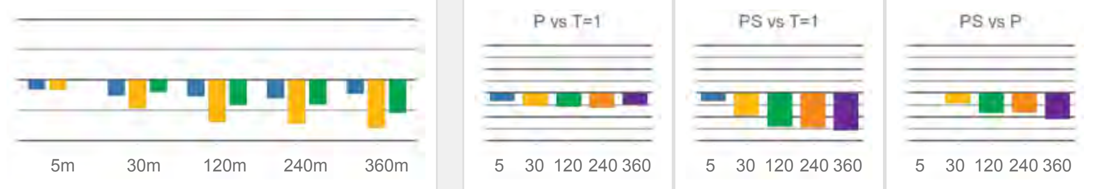

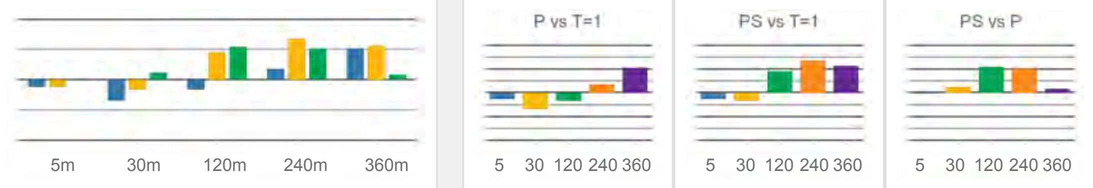

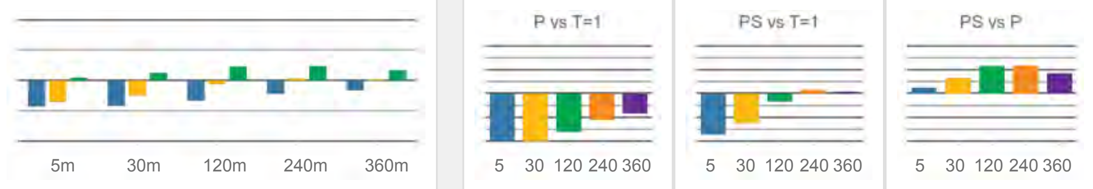

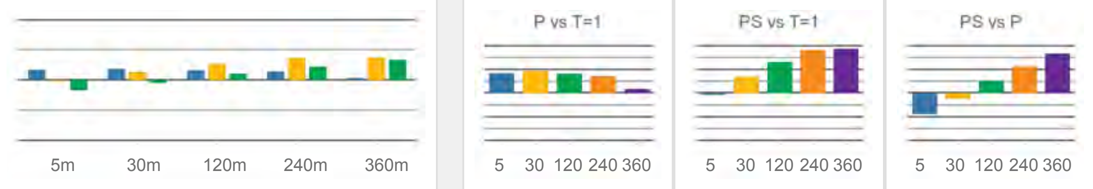

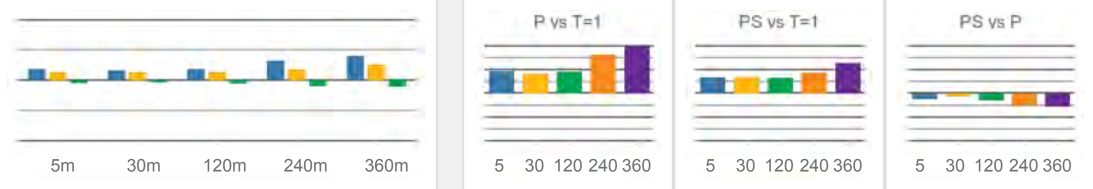

PgSg Time Course

Hackett Laboratory UW

log$_2$ (Fold Change)

P vs T=1 | PS vs T=1 | PS vs P

Locus		5m	30m	120m	240m	360m
**PGN_1387**	P vs T=1	-0.069	-0.340	-0.331	0.589	1.566
	PS vs T=1	-0.267	-0.634	-0.760	-0.427	0.112
	PS vs P	-0.176	-0.309	-0.487	-0.863	-1.296

putative ABC transporter permease protein

transport and binding proteins

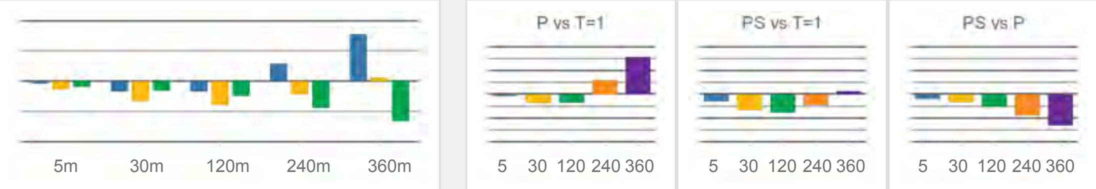

Locus		5m	30m	120m	240m	360m
**PGN_1388**	P vs T=1	0.204	-0.034	-0.246	0.181	0.902
	PS vs T=1	0.278	-0.293	-0.991	-0.752	-0.329
	PS vs P	0.123	-0.235	-0.755	-0.836	-1.093

DNA ligase

DNA metabolism

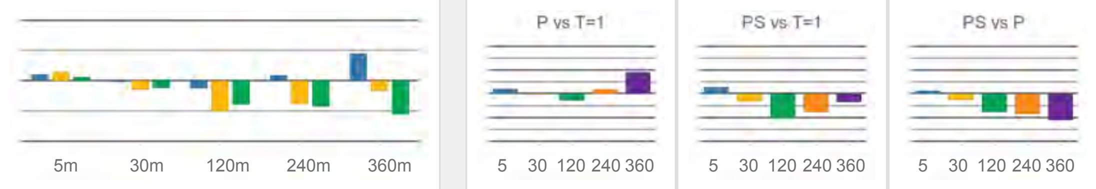

Locus		5m	30m	120m	240m	360m
**PGN_1389**	P vs T=1	-0.542	-0.891	-1.173	-0.569	0.667
	PS vs T=1	-0.285	-0.761	-1.137	-0.884	-0.685
	PS vs P	0.271	0.069	-0.194	-0.260	-1.042

probable acetyltransferase

unknown function

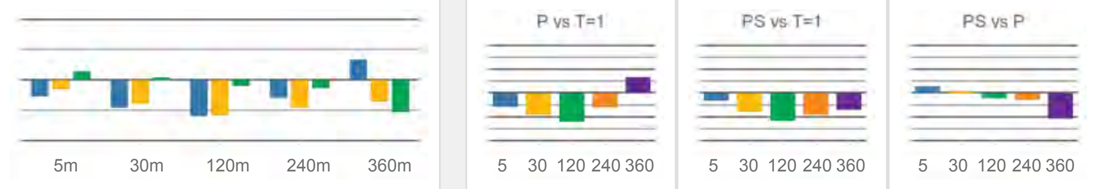

Locus		5m	30m	120m	240m	360m
**PGN_1390**	P vs T=1	-0.782	-0.899	-0.675	-0.703	-0.695
	PS vs T=1	-0.839	-1.324	-1.189	-0.926	-0.889
	PS vs P	-0.054	-0.430	-0.487	-0.205	-0.188

putative recombination protein RecR

DNA metabolism

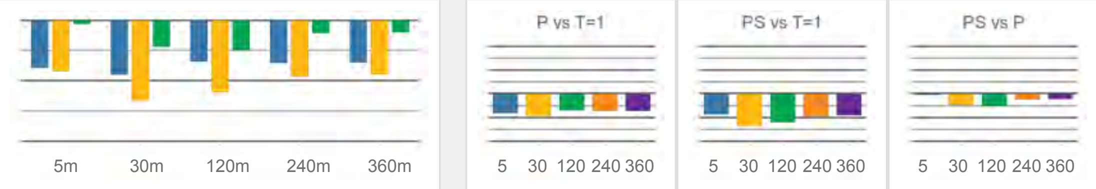

Locus		5m	30m	120m	240m	360m
**PGN_1391**	P vs T=1	0.126	-0.006	-0.239	-0.503	-0.722
	PS vs T=1	-0.113	-0.279	-0.504	-0.460	-0.497
	PS vs P	-0.217	-0.257	-0.260	0.031	0.208

putative ribonuclease E

transcription

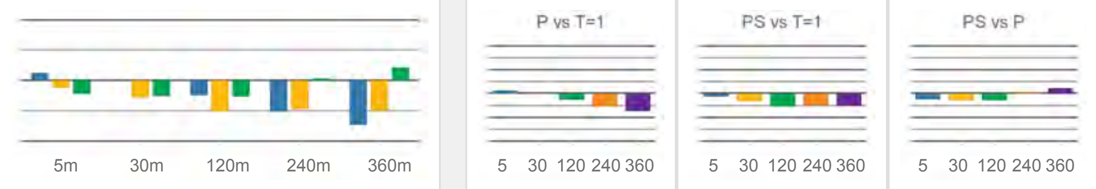

Locus		5m	30m	120m	240m	360m
**PGN_1392**	P vs T=1	-0.007	0.100	0.533	0.292	-0.670
	PS vs T=1	-0.071	0.409	1.007	0.867	0.736
	PS vs P	-0.093	0.283	0.537	0.585	1.269

conserved hypothetical protein

hypothetical proteins-Conserved

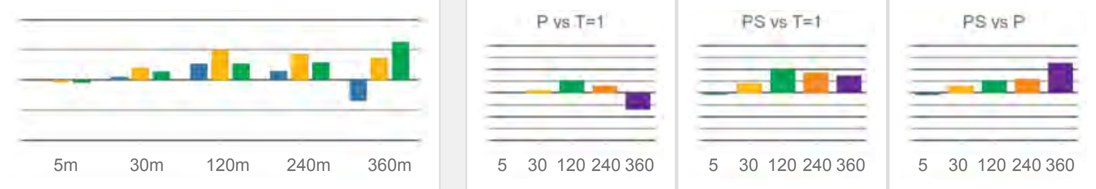

Locus		5m	30m	120m	240m	360m
**PGN_1393**	P vs T=1	0.916	0.670	0.473	-0.094	-0.819
	PS vs T=1	0.237	0.644	1.101	1.108	0.886
	PS vs P	-0.589	0.013	0.646	1.126	1.575

putative DNA-binding protein HU

DNA metabolism

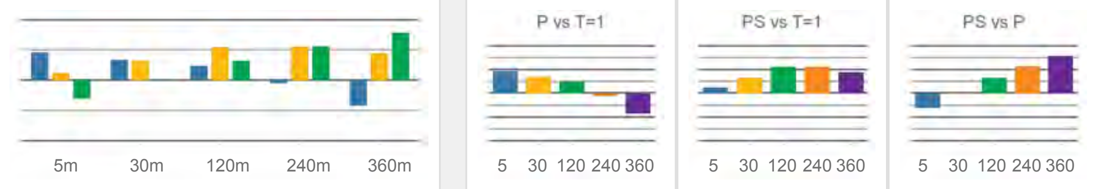

Legend: P vs T=1 | PS vs T=1 | PS vs P

Locus		5m	30m	120m	240m	360m
		log$_2$ (Fold Change)				
**PGN_1394**	P vs T=1	0.097	-0.796	-1.203	-0.923	-0.096
	PS vs T=1	0.549	0.081	-0.408	-0.722	-0.694
	PS vs P	0.503	0.807	0.662	0.138	-0.531

transposase in ISPg2

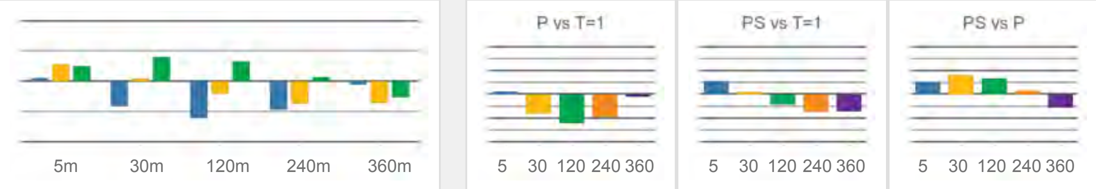

Locus		5m	30m	120m	240m	360m
**PGN_1395**	P vs T=1	-0.294	-0.353	-0.755	-1.288	-1.573
	PS vs T=1	-0.131	-0.683	-1.682	-2.055	-2.078
	PS vs P	0.184	-0.302	-0.904	-0.790	-0.539

putative anaerobic ribonucleoside-triphosphate reductase activating protein

purines, pyrimidines, nucleosides and nucleotides

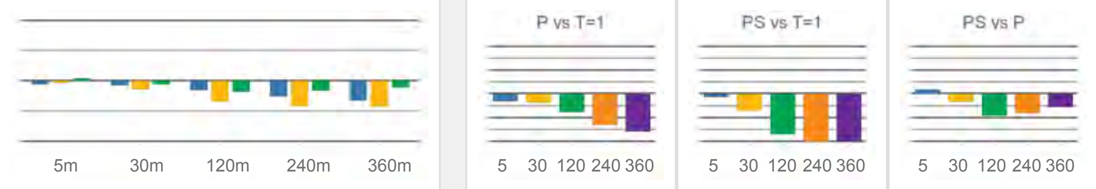

Locus		5m	30m	120m	240m	360m
**PGN_1396**	P vs T=1	-1.706	-1.497	-1.384	-1.414	-1.573
	PS vs T=1	-1.550	-1.717	-1.869	-1.602	-1.300
	PS vs P	0.053	-0.252	-0.496	-0.215	0.214

anaerobic ribonucleoside-triphosphate reductase

purines, pyrimidines, nucleosides and nucleotides

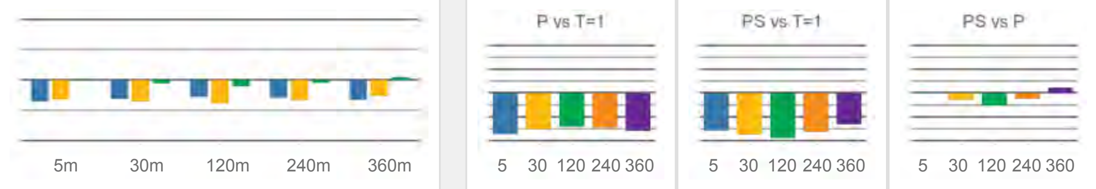

Locus		5m	30m	120m	240m	360m
**PGN_1397**	P vs T=1	0.204	0.117	0.176	0.290	-0.054
	PS vs T=1	0.019	-0.181	-0.070	0.071	0.064
	PS vs P	-0.183	-0.303	-0.253	-0.217	0.098

transposase in ISPg1

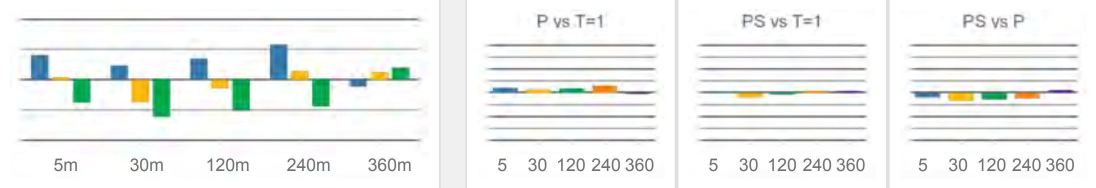

Locus		5m	30m	120m	240m	360m
**PGN_1398**	P vs T=1	-0.996	-0.774	-1.259	-1.710	-2.538
	PS vs T=1	-1.338	-1.307	-1.958	-2.912	-2.854
	PS vs P	-0.249	-0.358	-0.606	-1.157	-0.419

conserved hypothetical protein

hypothetical proteins-Conserved

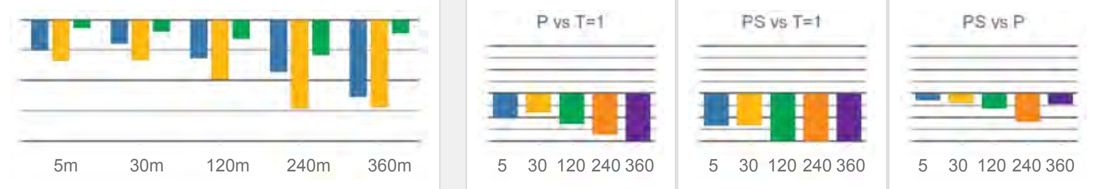

Locus		5m	30m	120m	240m	360m
**PGN_1399**	P vs T=1	-1.728	-1.025	-0.525	-0.836	-1.469
	PS vs T=1	-1.590	-1.166	-1.080	-0.818	-0.721
	PS vs P	-0.164	-0.148	-0.413	0.014	0.563

conserved hypothetical protein

hypothetical proteins-Conserved

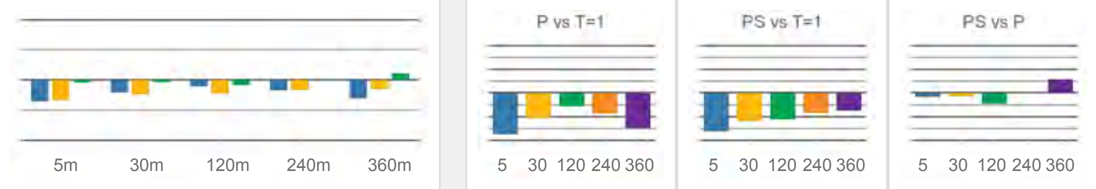

Locus		5m	30m	120m	240m	360m
**PGN_1400**	P vs T=1	0.227	-0.194	-0.311	-0.250	-0.747
	PS vs T=1	-0.305	-0.405	-0.623	-0.401	-0.177
	PS vs P	-0.432	-0.196	-0.307	-0.126	0.514

conserved hypothetical protein

hypothetical proteins-Conserved

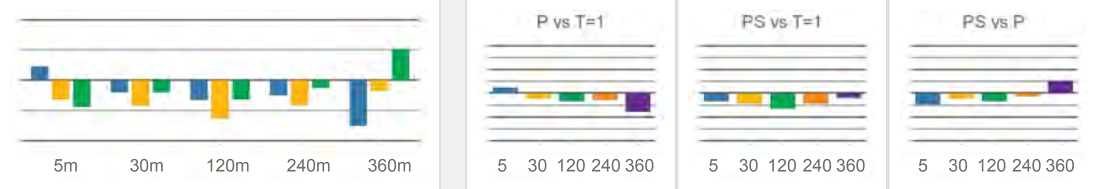

Supplement: Table S2 — The second of three sequential files making up the master list. [file Table2.PDF]
