# Supplementary material for: Insights into Dynamic Polymicrobial Synergy Revealed by Time-Coursed RNA-Seq
Source: Front Microbiol. 2017 Feb 28;8:261. doi: 10.3389/fmicb.2017.00261 (PMC5329018; doi:10.3389/fmicb.2017.00261)

log$_2$ (Fold Change)

P vs T=1 | PS vs T=1 | PS vs P

Locus		5m	30m	120m	240m	360m
**PGN_1401**	P vs T=1	-1.158	-0.326	0.580	0.686	0.027
	PS vs T=1	0.813	1.191	1.393	1.239	1.243
	PS vs P	1.836	1.451	0.816	0.559	1.186
	delta-1-pyrroline-5-carboxylate dehydrogenase					
	energy metabolism					

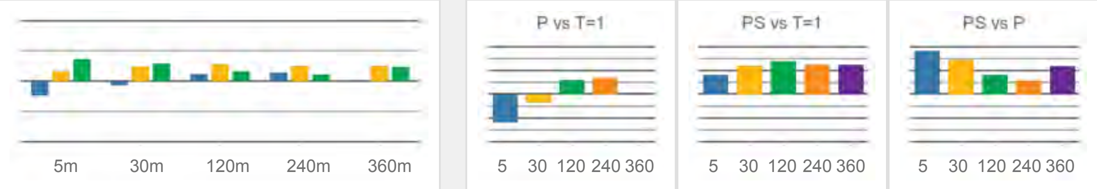

Locus		5m	30m	120m	240m	360m
**PGN_1402**	P vs T=1	0.015	0.577	0.775	0.221	-0.617
	PS vs T=1	1.180	1.309	0.918	0.294	-0.042
	PS vs P	1.121	0.730	0.165	0.062	0.532
	putative amidinotransferase					
	hypothetical proteins-Conserved					

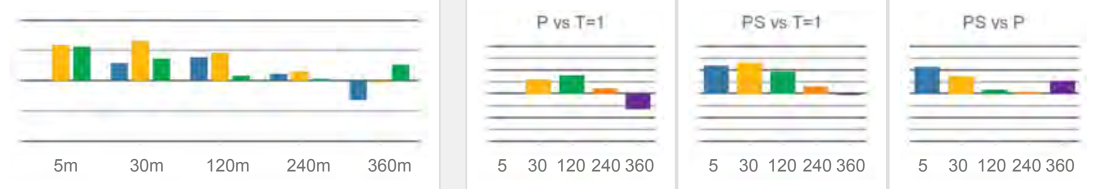

Locus		5m	30m	120m	240m	360m
**PGN_1403**	P vs T=1	-0.498	-0.024	0.083	-0.648	-1.754
	PS vs T=1	0.218	0.381	0.079	-0.570	-0.859
	PS vs P	0.684	0.414	0.025	0.061	0.834
	ornithine aminotransferase					
	amino acid biosynthesis					

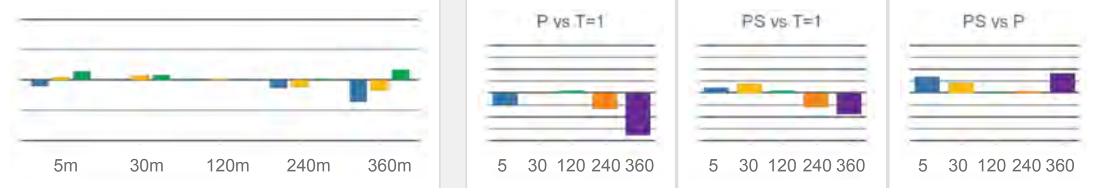

Locus		5m	30m	120m	240m	360m
**PGN_1404**	P vs T=1	1.340	1.105	1.126	1.875	2.587
	PS vs T=1	1.255	0.826	1.555	2.037	2.227
	PS vs P	0.027	-0.262	0.227	0.407	0.156
	conserved hypothetical protein					
	hypothetical proteins-Conserved					

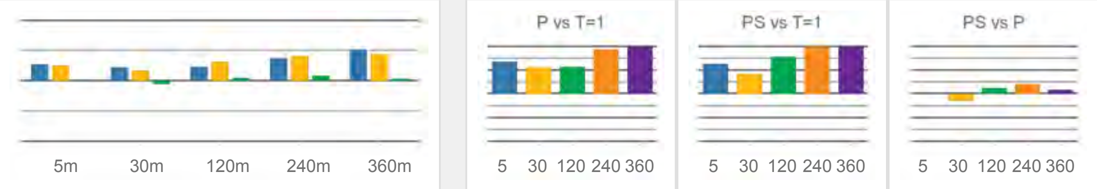

Locus		5m	30m	120m	240m	360m
**PGN_1405**	P vs T=1	0.698	0.943	0.960	0.749	0.286
	PS vs T=1	0.543	0.555	0.736	1.082	1.083
	PS vs P	-0.151	-0.352	-0.196	0.331	0.758
	elongation factor P					
	protein synthesis					

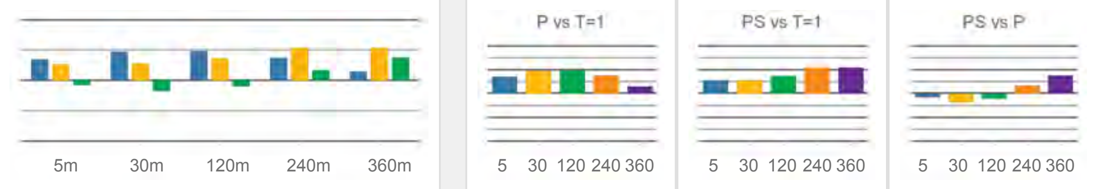

Locus		5m	30m	120m	240m	360m
**PGN_1406**	P vs T=1	2.233	2.494	3.181	3.873	3.775
	PS vs T=1	1.957	1.857	2.033	2.787	3.071
	PS vs P	-0.203	-0.473	-0.816	-0.511	-0.323
	hypothetical protein					
	hypothetical proteins					

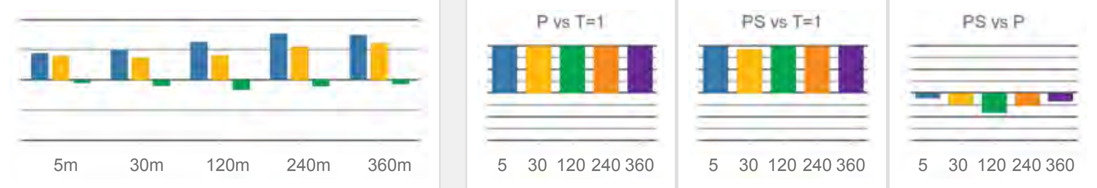

Locus		5m	30m	120m	240m	360m
**PGN_1407**	P vs T=1	0.167	0.685	1.456	1.973	1.916
	PS vs T=1	-0.104	0.109	0.366	0.639	0.800
	PS vs P	-0.305	-0.566	-1.038	-1.243	-1.063
	DNA-binding protein histone-like family					
	DNA metabolism					

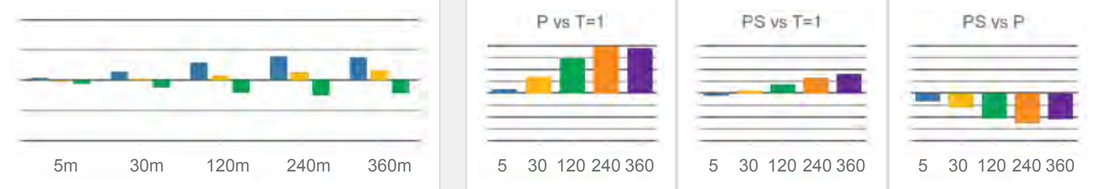

PgSg Time Course

Hackett Laboratory UW

log$_2$ (Fold Change)

Locus		5m	30m	120m	240m	360m
**PGN_1408**	P vs T=1	-0.709	-0.724	-0.489	-0.171	0.028
	PS vs T=1	-0.489	-0.460	-0.129	0.291	0.278
	PS vs P	0.185	0.223	0.329	0.458	0.255
	conserved hypothetical protein with predicted permease membrane region					
	transport and binding proteins					
**PGN_1409**	P vs T=1	0.276	0.625	0.486	-0.013	-0.745
	PS vs T=1	0.452	0.504	0.439	0.446	0.150
	PS vs P	0.171	-0.098	-0.030	0.442	0.850
	putative peptidase					
	protein fate					
**PGN_1410**	P vs T=1	1.037	1.849	2.331	2.011	1.330
	PS vs T=1	0.869	0.976	1.363	1.420	0.974
	PS vs P	-0.219	-0.733	-0.766	-0.449	-0.358
	conserved hypothetical protein					
	hypothetical proteins-Conserved					
**PGN_1411**	P vs T=1	1.202	1.471	1.235	0.783	0.650
	PS vs T=1	1.558	1.606	1.168	0.817	0.452
	PS vs P	0.355	0.183	-0.037	-0.006	-0.222
	putative N-ethylammeline chlorohydrolase					
	central intermediary metabolism					
**PGN_1412**	P vs T=1	1.332	2.025	2.709	2.670	1.967
	PS vs T=1	1.271	1.877	2.299	2.196	2.101
	PS vs P	-0.082	-0.133	-0.369	-0.439	0.134
	putative purine nucleoside phosphorylase I					
	purines, pyrimidines, nucleosides and nucleotides					
**PGN_1413**	P vs T=1	-1.587	-1.731	-2.115	-2.651	-2.832
	PS vs T=1	-1.069	-0.607	-0.223	-0.162	-0.426
	PS vs P	0.468	1.052	1.788	2.259	2.242
	conserved hypothetical protein					
	hypothetical proteins-Conserved					
**PGN_1414**	P vs T=1	-0.458	-0.880	-1.100	0.454	1.392
	PS vs T=1	-1.158	-1.046	-0.425	-0.141	-0.277
	PS vs P	-0.561	-0.340	0.043	-0.015	-0.649
	hypothetical protein					
	hypothetical proteins					

P vs T=1 | PS vs T=1 | PS vs P

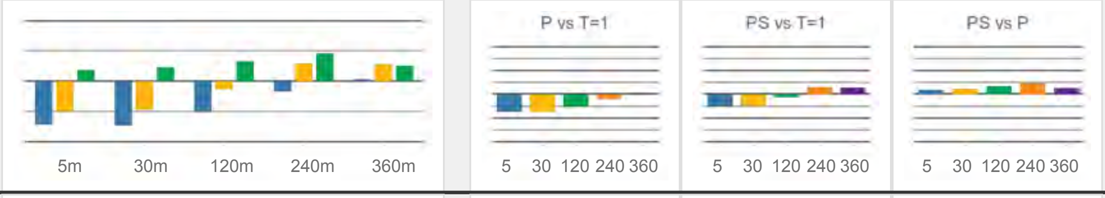

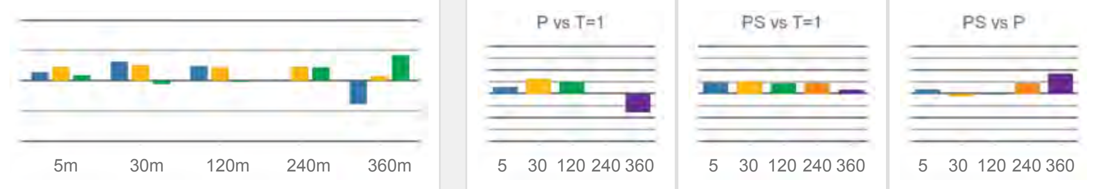

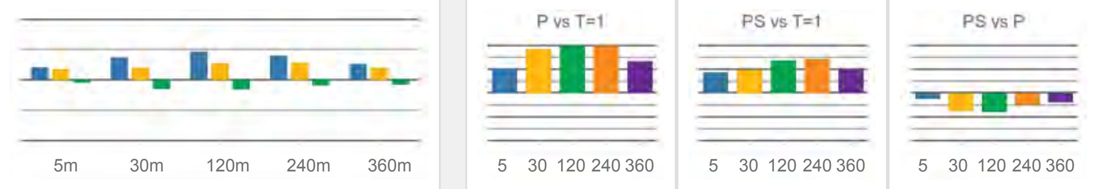

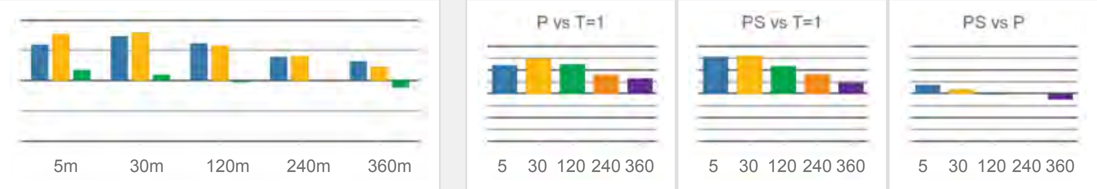

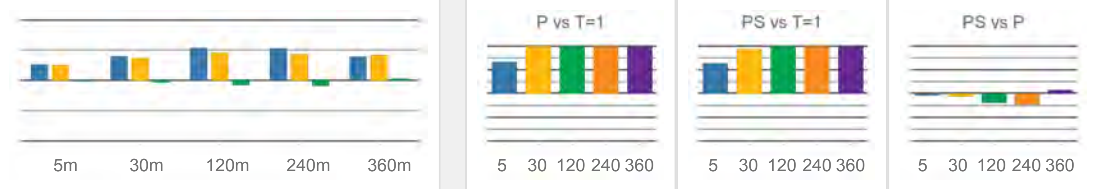

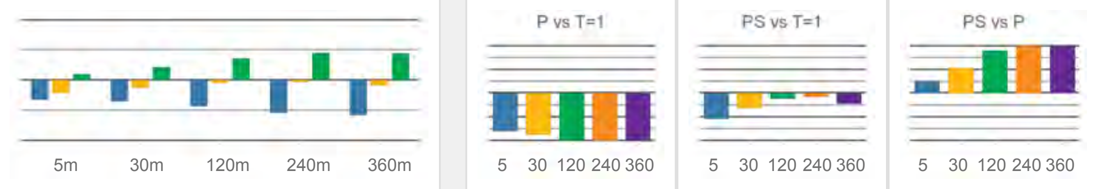

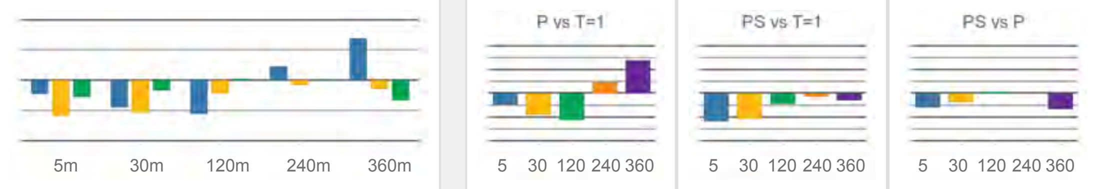

log2 (Fold Change)

P vs T=1 | PS vs T=1 | PS vs P

Locus		5m	30m	120m	240m	360m
**PGN_1415**	P vs T=1	-0.817	-0.701	-0.659	-0.504	-0.546
	PS vs T=1	-1.041	-1.024	-0.758	-0.376	-0.206
	PS vs P	-0.237	-0.317	-0.105	0.145	0.335
	DNA-binding protein histone-like family					
	DNA metabolism					
**PGN_1416**	P vs T=1	0.099	0.481	0.800	0.853	0.682
	PS vs T=1	0.578	0.417	0.520	0.637	0.596
	PS vs P	0.460	-0.064	-0.269	-0.205	-0.083
	probable lysyl endopeptidase precursor					
	protein fate					
**PGN_1417**	P vs T=1	0.416	1.004	2.085	1.920	2.558
	PS vs T=1	0.471	0.813	0.849	1.089	1.398
	PS vs P	-0.114	-0.174	-0.820	-0.502	-0.730
	hypothetical protein					
	hypothetical proteins					
**PGN_1418**	P vs T=1	1.968	2.811	3.534	3.238	2.408
	PS vs T=1	1.830	1.941	1.724	1.446	1.376
	PS vs P	-0.150	-0.807	-1.708	-1.715	-1.016
	pyruvate-flavodoxin oxidoreductase					
	energy metabolism					
**PGN_1419**	P vs T=1	0.206	0.080	-0.285	-0.886	-1.482
	PS vs T=1	-0.346	-0.359	-1.038	-1.120	-1.254
	PS vs P	-0.530	-0.420	-0.741	-0.249	0.199
	conserved hypothetical protein					
	hypothetical proteins-Conserved					
**PGN_1420**	P vs T=1	0.313	0.244	0.298	0.444	0.093
	PS vs T=1	0.161	-0.016	0.169	0.300	0.242
	PS vs P	-0.153	-0.269	-0.141	-0.139	0.123
	transposase in ISPg1					
**PGN_1421**	P vs T=1	0.914	1.220	1.610	1.743	1.528
	PS vs T=1	0.475	0.593	1.365	1.483	1.252
	PS vs P	-0.452	-0.577	-0.163	-0.129	-0.225
	hypothetical protein					
	hypothetical proteins					

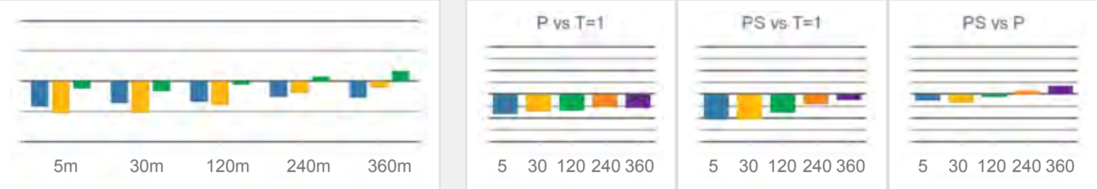

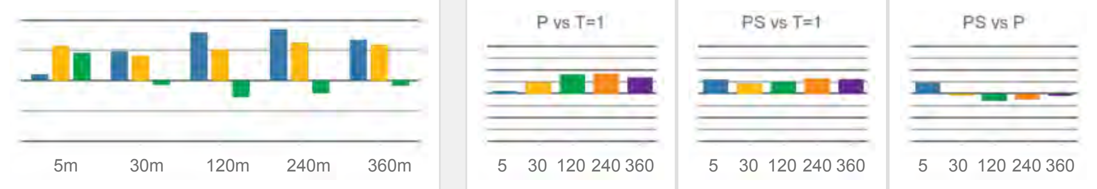

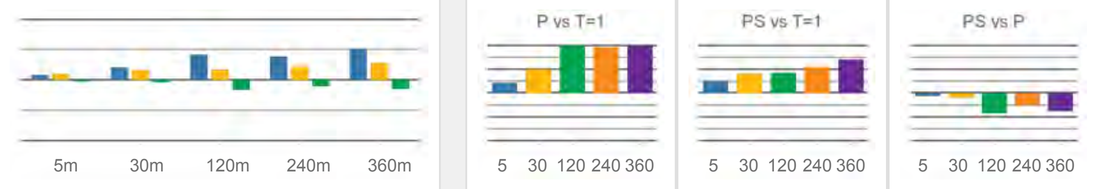

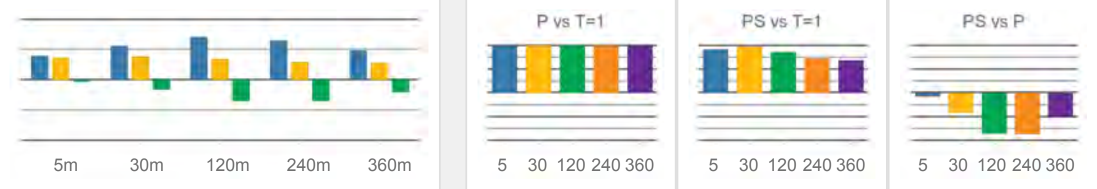

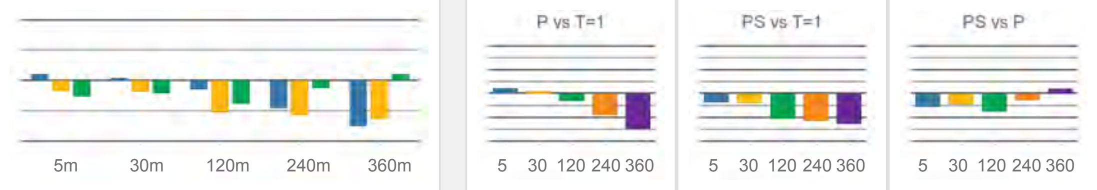

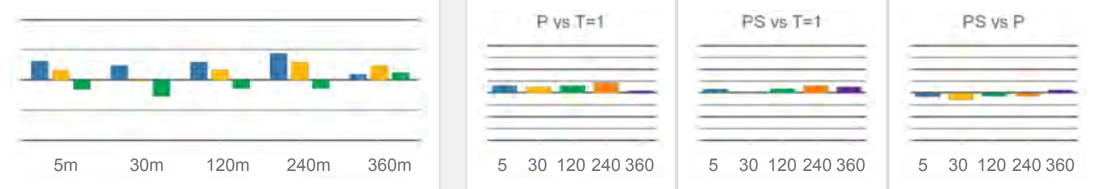

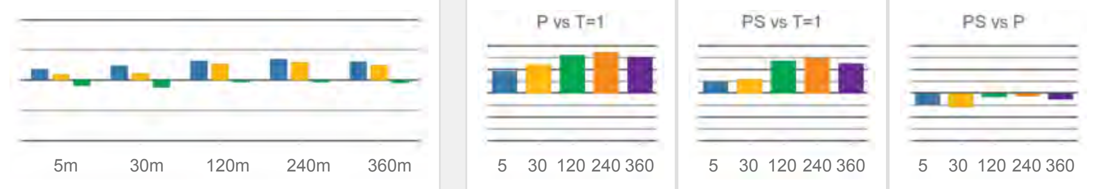

PgSg Time Course

log$_2$ (Fold Change)

Legend: P vs T=1 | PS vs T=1 | PS vs P

Locus		5m	30m	120m	240m	360m
**PGN_1422**	P vs T=1	-1.154	-0.888	-0.701	-0.914	-1.547
	PS vs T=1	-1.397	-0.851	-0.155	0.176	0.218
	PS vs P	-0.273	0.026	0.542	1.059	1.702
	putative partial DNA-binding protein histone-like family					
	cellular processes					
**PGN_1423**	P vs T=1	-0.695	-0.621	-0.513	-0.797	-1.727
	PS vs T=1	-0.711	-0.212	0.488	0.373	0.246
	PS vs P	-0.051	0.376	0.984	1.114	1.841
	putative partial DNA-binding protein histone-like family					
	cellular processes					
**PGN_1424**	P vs T=1	-0.356	-0.439	-0.431	-0.775	-1.692
	PS vs T=1	-0.189	0.200	0.846	0.702	0.516
	PS vs P	0.131	0.571	1.228	1.362	1.994
	putative partial Type II restriction enzyme					
	hypothetical proteins					
**PGN_1425**	P vs T=1	-1.202	-1.636	-2.157	-2.403	-2.649
	PS vs T=1	-1.205	-1.056	-0.721	-1.229	-1.391
	PS vs P	0.010	0.545	1.352	1.049	1.150
	putative partial Type II restriction enzyme					
	hypothetical proteins					
**PGN_1426**	P vs T=1	-1.055	-1.793	-2.343	-2.295	-2.512
	PS vs T=1	-0.960	-0.801	-0.447	-0.878	-1.279
	PS vs P	0.122	0.902	1.744	1.286	1.118
	adenine-specific methyltransferase					
	unknown function					
**PGN_1427**	P vs T=1	-0.847	-1.663	-1.316	-1.050	-0.544
	PS vs T=1	-0.390	-0.882	-0.052	0.103	-0.258
	PS vs P	0.427	0.387	1.001	0.956	0.317
	hypothetical protein					
	hypothetical proteins					
**PGN_1428**	P vs T=1	0.281	-0.380	-0.072	0.412	1.600
	PS vs T=1	-0.123	-0.097	0.431	0.504	0.701
	PS vs P	-0.374	0.153	0.395	0.085	-0.756
	transposase in ISPg3					

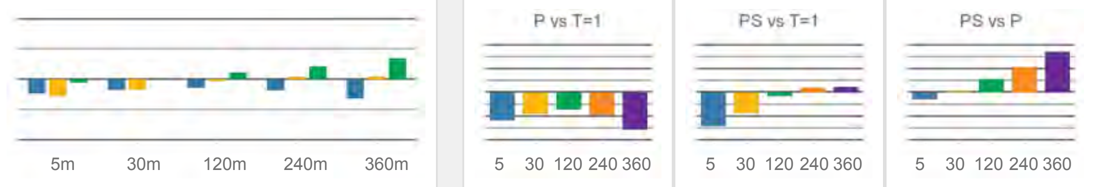

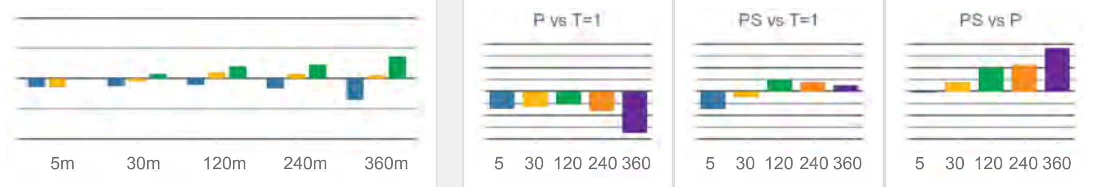

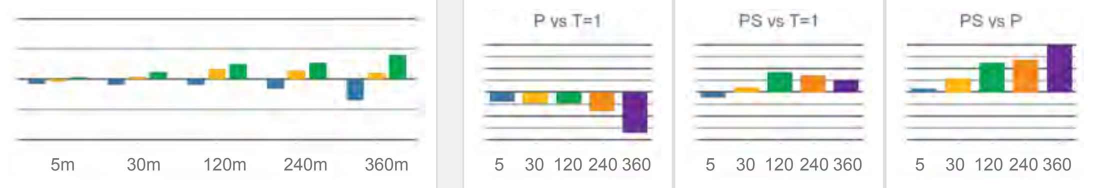

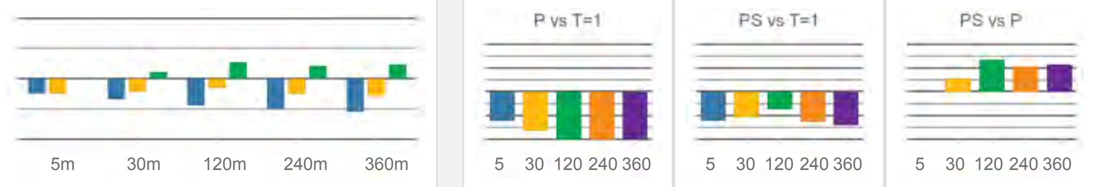

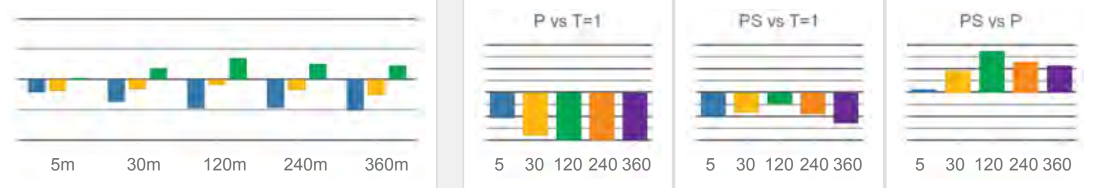

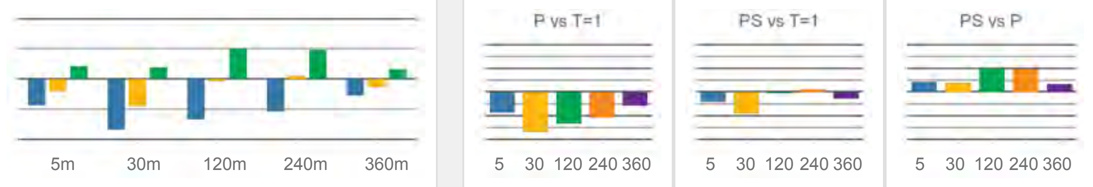

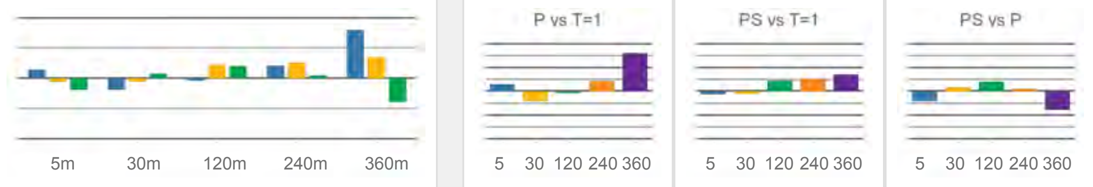

log$_2$ (Fold Change)

Legend: P vs T=1 | PS vs T=1 | PS vs P

Locus		5m	30m	120m	240m	360m
**PGN_1429**	P vs T=1	-0.522	0.344	1.019	0.883	0.324
	PS vs T=1	-0.226	0.395	0.915	0.544	0.276
	PS vs P	0.205	0.057	-0.039	-0.285	-0.059
	conserved hypothetical protein					
	hypothetical proteins-Conserved					
**PGN_1430**	P vs T=1	-0.183	0.000	0.002	-0.118	-0.125
	PS vs T=1	0.169	0.216	-0.115	-0.400	-0.429
	PS vs P	0.337	0.217	-0.110	-0.281	-0.302
	putative transmembrane Acr-type transport protein					
	transport and binding proteins					
**PGN_1431**	P vs T=1	0.088	0.417	0.092	-0.345	-0.513
	PS vs T=1	0.212	0.483	0.242	0.157	0.127
	PS vs P	0.115	0.086	0.149	0.440	0.593
	putative cation efflux system protein					
	hypothetical proteins-Conserved					
**PGN_1432**	P vs T=1	-0.343	-0.367	-0.920	-1.052	-1.044
	PS vs T=1	-0.286	0.129	0.172	0.509	0.255
	PS vs P	0.049	0.484	1.023	1.464	1.241
	probable outer membrane efflux protein					
	transport and binding proteins					
**PGN_1433**	P vs T=1	0.376	0.333	0.435	0.440	0.082
	PS vs T=1	0.185	0.045	0.174	0.255	0.293
	PS vs P	-0.189	-0.292	-0.260	-0.184	0.186
	transposase in ISPg1					
**PGN_1434**	P vs T=1	1.216	1.684	1.401	0.912	0.373
	PS vs T=1	1.273	1.535	1.637	1.745	1.534
	PS vs P	0.050	-0.119	0.243	0.806	1.124
	aminoacyl-histidine dipeptidase					
	protein fate					
**PGN_1435**	P vs T=1	0.735	-0.373	-1.111	-1.220	-1.044
	PS vs T=1	-0.079	-0.532	-0.618	-0.851	-0.936
	PS vs P	-0.669	-0.145	0.397	0.248	0.073
	hypothetical protein					
	hypothetical proteins					

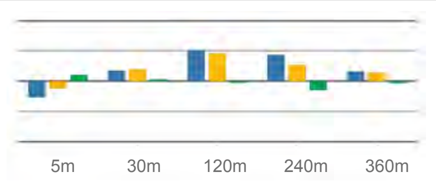

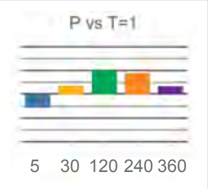

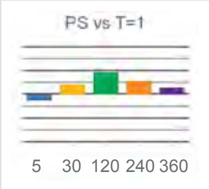

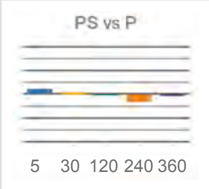

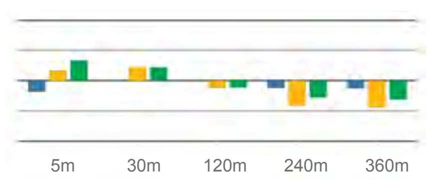

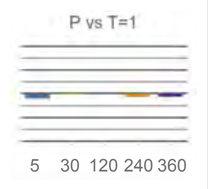

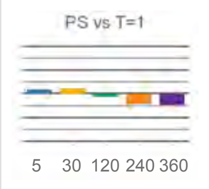

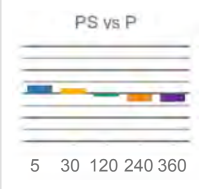

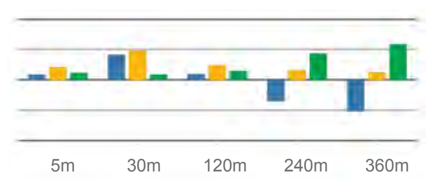

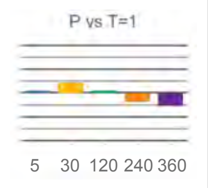

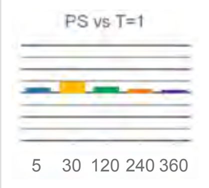

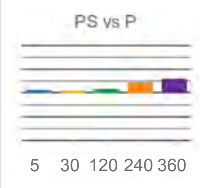

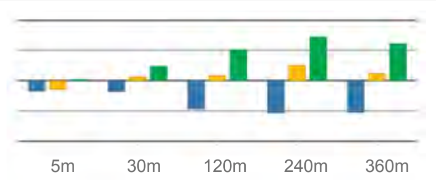

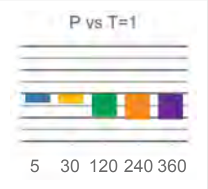

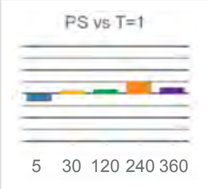

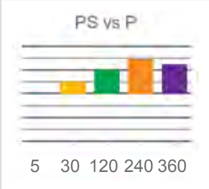

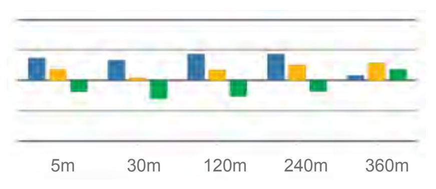

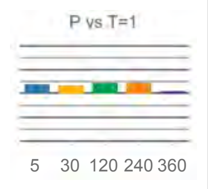

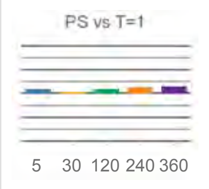

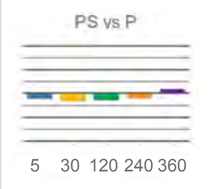

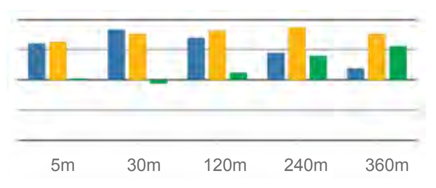

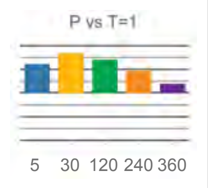

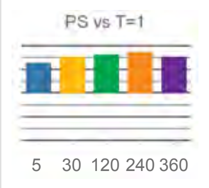

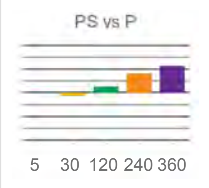

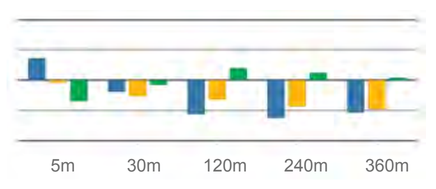

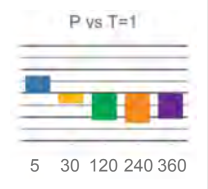

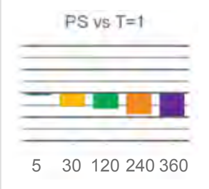

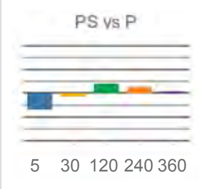

Legend: P vs T=1 | PS vs T=1 | PS vs P

Locus		log$_2$ (Fold Change) 5m	30m	120m	240m	360m
**PGN_1436**	P vs T=1	0.666	0.726	0.688	0.110	-0.932
	PS vs T=1	0.171	0.717	0.877	0.412	0.026
	PS vs P	-0.457	0.028	0.230	0.284	0.864
	conserved hypothetical protein					
	hypothetical proteins-Conserved					
**PGN_1437**	P vs T=1	-0.404	-0.056	0.263	0.372	0.100
	PS vs T=1	0.097	0.135	0.201	0.244	0.293
	PS vs P	0.475	0.184	-0.055	-0.116	0.189
	conserved hypothetical protein					
	hypothetical proteins-Conserved					
**PGN_1438**	P vs T=1	0.453	-0.604	-0.387	0.356	1.064
	PS vs T=1	-0.505	-0.259	-0.032	0.766	1.229
	PS vs P	-0.614	-0.061	-0.005	0.466	0.530
	hypothetical protein					
	hypothetical proteins					
**PGN_1439**	P vs T=1	-2.335	-2.996	-3.289	-1.912	-1.954
	PS vs T=1	-3.592	-4.457	-3.497	-3.838	-2.388
	PS vs P	-1.102	-1.422	-0.619	-1.304	-0.250
	hypothetical protein					
	hypothetical proteins					
**PGN_1440**	P vs T=1	0.042	-0.617	-0.863	0.105	1.190
	PS vs T=1	-0.110	-0.843	-0.583	-0.453	0.381
	PS vs P	-0.081	-0.289	0.030	-0.420	-0.596
	putative vancomycin B-type resistance protein VanW					
	hypothetical proteins-Conserved					
**PGN_1441**	P vs T=1	-0.051	-0.102	-0.436	-0.758	-0.701
	PS vs T=1	0.024	-0.072	0.114	0.322	0.112
	PS vs P	0.077	0.033	0.534	1.028	0.789
	glutamine-dependent NAD synthetase					
	biosynthesis of cofactors, prosthetic groups, and carriers					
**PGN_1442**	P vs T=1	0.228	0.168	0.164	0.331	-0.058
	PS vs T=1	0.018	-0.167	-0.066	0.131	0.118
	PS vs P	-0.209	-0.338	-0.239	-0.196	0.158
	transposase in ISPg1					

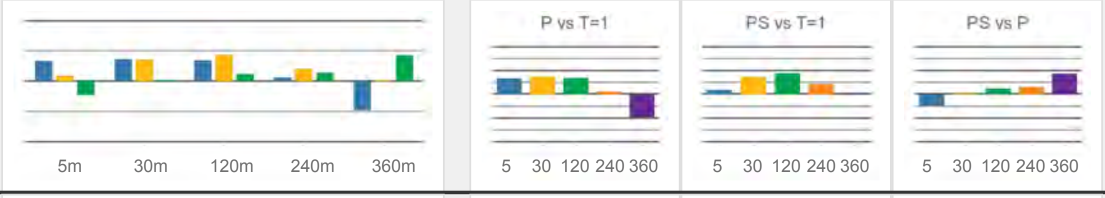

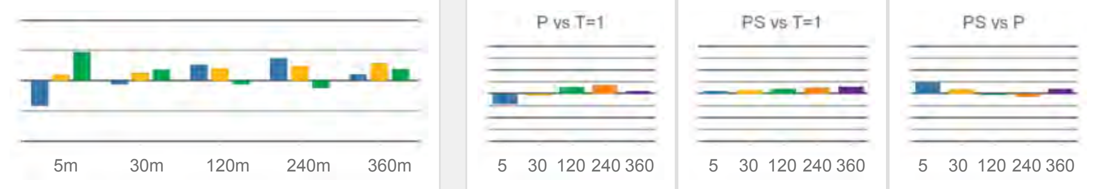

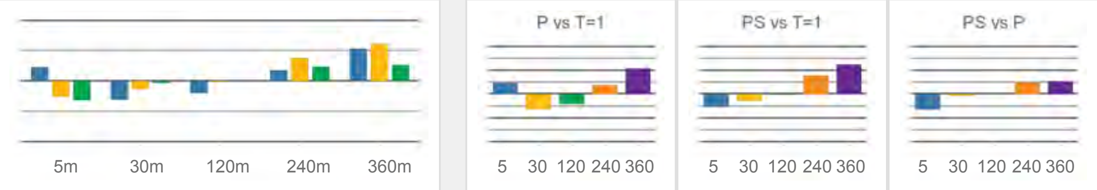

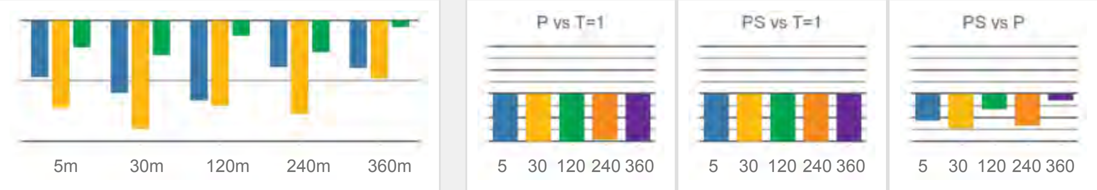

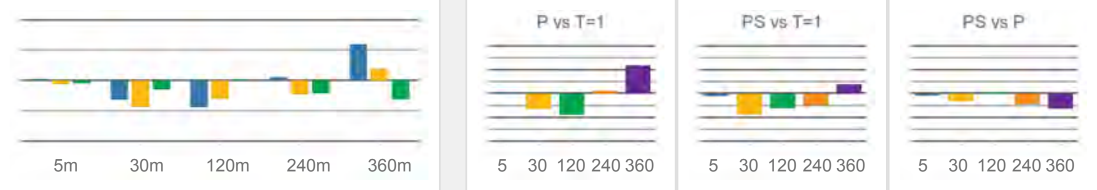

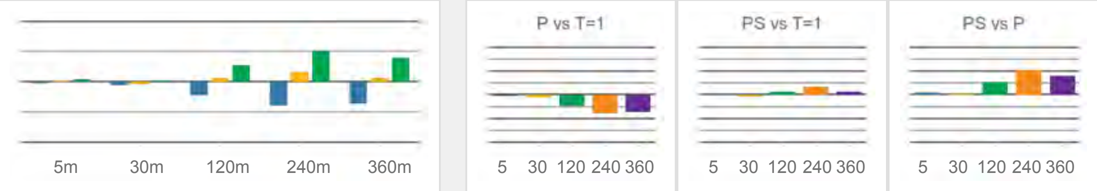

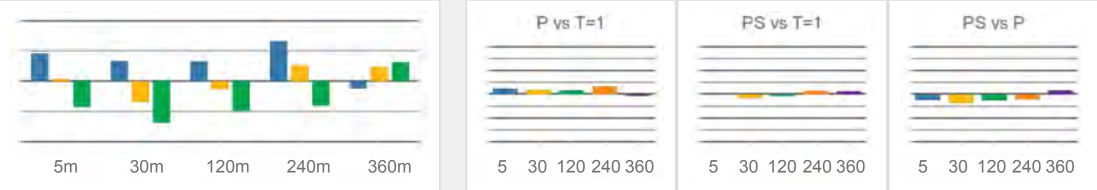

PgSg Time Course

Hackett Laboratory UW

log$_2$ (Fold Change)

P vs T=1 | PS vs T=1 | PS vs P

Locus		5m	30m	120m	240m	360m
PGN_1443	P vs T=1	1.239	1.253	0.611	0.009	-0.116
	PS vs T=1	1.305	0.939	-0.485	-0.915	-0.963
	PS vs P	0.126	-0.239	-1.058	-0.936	-0.853

carbamoyl-phosphate synthase large subunit

purines, pyrimidines, nucleosides and nucleotides

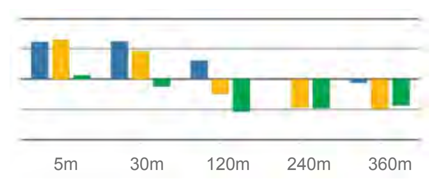

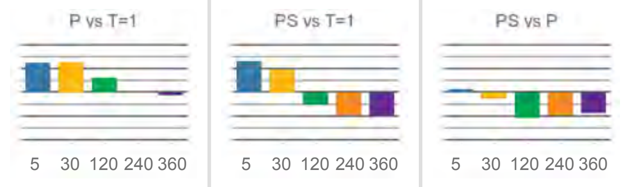

Locus		5m	30m	120m	240m	360m
PGN_1444	P vs T=1	0.740	1.135	1.392	1.157	0.556
	PS vs T=1	1.463	1.479	0.955	0.553	0.246
	PS vs P	0.704	0.348	-0.411	-0.584	-0.322

carbamoyl-phosphate synthase small subunit

purines, pyrimidines, nucleosides and nucleotides

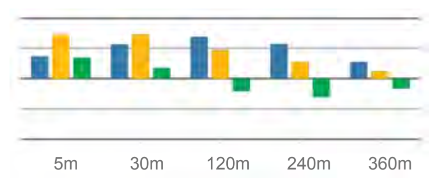

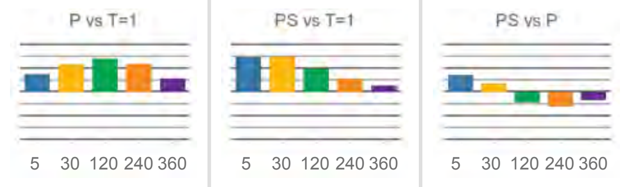

Locus		5m	30m	120m	240m	360m
PGN_1445	P vs T=1	-0.248	-0.050	-0.377	-0.863	-1.311
	PS vs T=1	0.103	-0.110	-0.695	-1.252	-1.344
	PS vs P	0.352	-0.041	-0.307	-0.406	-0.066

putative amidophosphoribosyltransferase

purines, pyrimidines, nucleosides and nucleotides

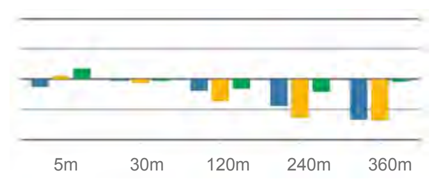

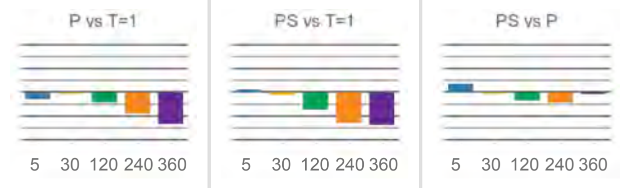

Locus		5m	30m	120m	240m	360m
PGN_1446	P vs T=1	-0.137	-0.071	-0.046	-0.100	-0.349
	PS vs T=1	-0.168	-0.520	-0.672	-0.800	-0.839
	PS vs P	-0.024	-0.434	-0.607	-0.679	-0.487

conserved hypothetical protein

cell envelope

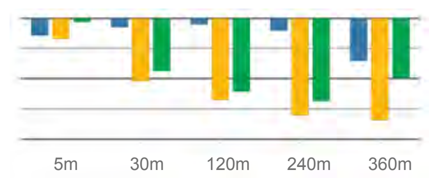

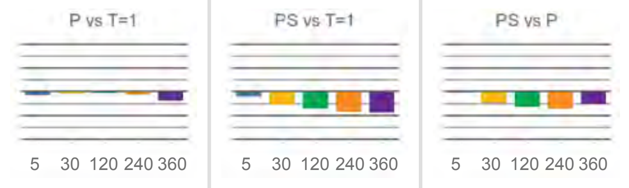

Locus		5m	30m	120m	240m	360m
PGN_1447	P vs T=1	-0.171	-0.056	-0.233	-0.343	-0.529
	PS vs T=1	-0.744	-0.673	-0.829	-0.709	-0.716
	PS vs P	-0.561	-0.598	-0.583	-0.357	-0.190

CTP synthase

purines, pyrimidines, nucleosides and nucleotides

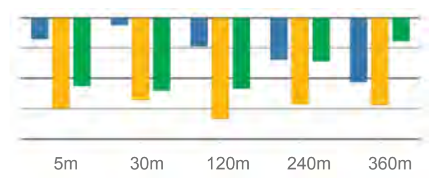

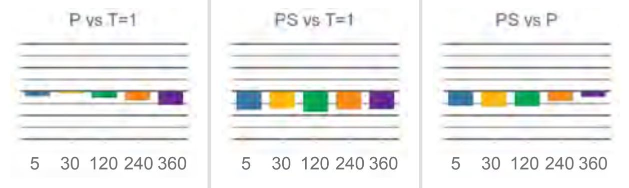

Locus		5m	30m	120m	240m	360m
PGN_1448	P vs T=1	0.074	-0.122	0.953	1.929	3.235
	PS vs T=1	-0.597	-0.053	0.725	1.488	1.571
	PS vs P	-0.705	-0.270	-0.144	0.109	-0.824

conserved hypothetical protein

hypothetical proteins-Conserved

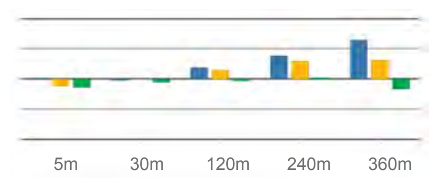

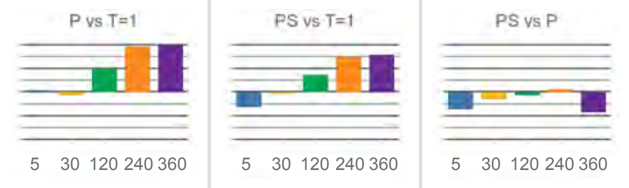

Locus		5m	30m	120m	240m	360m
PGN_1449	P vs T=1	-0.049	-0.280	-0.695	-0.844	-0.947
	PS vs T=1	-0.160	-0.242	-0.008	0.005	-0.354
	PS vs P	-0.089	0.043	0.657	0.802	0.559

inosine-5'-monophosphate dehydrogenase

purines, pyrimidines, nucleosides and nucleotides

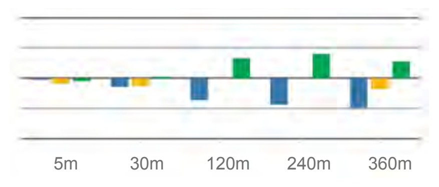

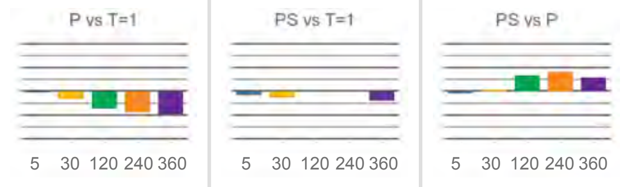

PgSg Time Course

log$_2$ (Fold Change)

Legend: P vs T=1, PS vs T=1, PS vs P

Locus		5m	30m	120m	240m	360m
**PGN_1450**	P vs T=1	-0.749	-0.676	-0.457	-0.463	-0.287
	PS vs T=1	-0.539	-0.839	-1.177	-0.878	-0.945
	PS vs P	0.195	-0.167	-0.690	-0.388	-0.625

putative tRNA isopentenyltransferase

protein synthesis

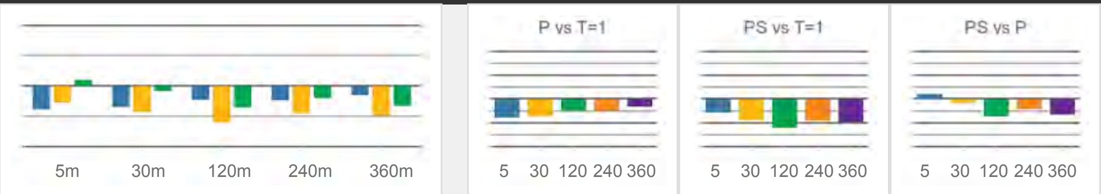

Locus		5m	30m	120m	240m	360m
**PGN_1451** *groES*	P vs T=1	2.425	2.758	3.674	3.621	2.728
	PS vs T=1	0.005	0.388	0.959	1.107	1.165
	PS vs P	-2.261	-2.183	-2.479	-2.296	-1.483

chaperonin GroES

protein fate

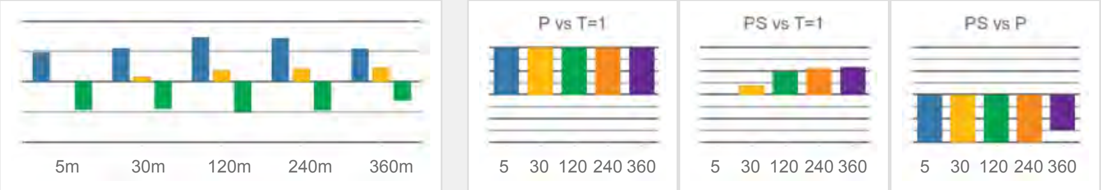

Locus		5m	30m	120m	240m	360m
**PGN_1452** *groEL*	P vs T=1	2.056	2.814	3.597	3.822	3.464
	PS vs T=1	0.857	1.039	1.347	1.614	1.702
	PS vs P	-1.166	-1.656	-2.103	-2.054	-1.677

chaperonin GroEL

protein fate

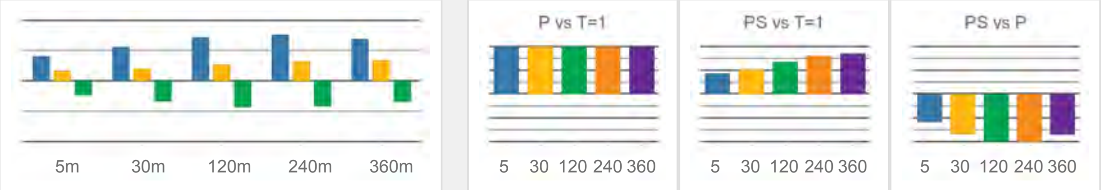

Locus		5m	30m	120m	240m	360m
**PGN_1453**	P vs T=1	0.658	0.204	1.209	0.914	2.979
	PS vs T=1	-0.433	0.028	1.518	1.957	1.453
	PS vs P	-0.803	-0.348	0.555	0.786	-0.511

conserved hypothetical protein

hypothetical proteins-Conserved

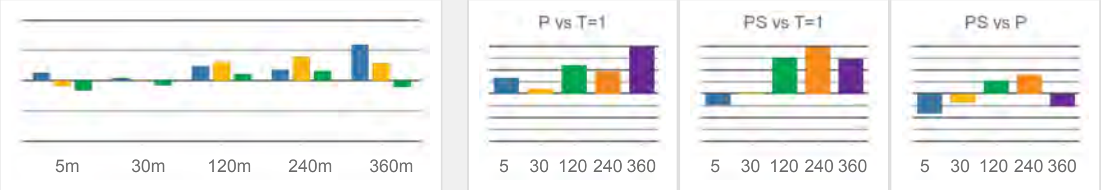

Locus		5m	30m	120m	240m	360m
**PGN_1454**	P vs T=1	0.122	-0.440	-0.472	-0.099	0.416
	PS vs T=1	-0.199	-0.433	0.285	0.461	0.463
	PS vs P	-0.307	-0.029	0.674	0.526	0.068

probable abortive infection protein

microbial immune system

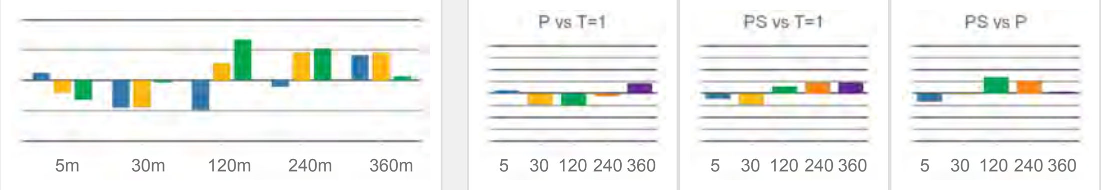

Locus		5m	30m	120m	240m	360m
**PGN_1455**	P vs T=1	0.181	-0.117	-0.734	-0.638	-0.314
	PS vs T=1	0.187	-0.410	-0.862	-1.107	-1.096
	PS vs P	0.020	-0.283	-0.141	-0.471	-0.771

conserved hypothetical protein

hypothetical proteins-Conserved

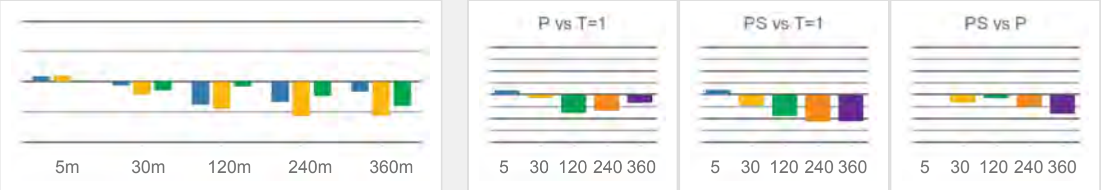

Locus		5m	30m	120m	240m	360m
**PGN_1456**	P vs T=1	-0.156	-0.183	-0.310	-0.622	-0.619
	PS vs T=1	0.127	-0.040	-0.561	-0.822	-0.899
	PS vs P	0.285	0.146	-0.244	-0.225	-0.290

conserved hypothetical protein

hypothetical proteins-Conserved

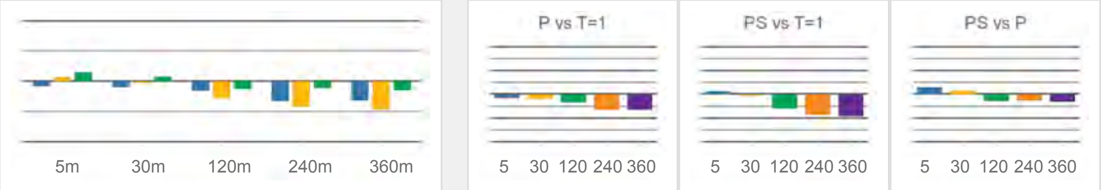

log$_2$ (Fold Change)

P vs T=1 PS vs T=1 PS vs P

Locus		5m	30m	120m	240m	360m
**PGN_1457**	P vs T=1	-0.036	-0.648	-1.508	-1.488	-1.137
	PS vs T=1	-0.172	-0.698	-1.591	-1.761	-1.771
	PS vs P	-0.111	-0.043	-0.114	-0.298	-0.631
	probable alkaline phosphatase					
	hypothetical proteins-Conserved					
**PGN_1458**	P vs T=1	0.457	0.336	-0.375	-0.855	-1.271
	PS vs T=1	0.567	0.400	-0.729	-1.625	-1.692
	PS vs P	0.130	0.084	-0.349	-0.776	-0.434
	preprotein translocase SecA subunit					
	protein fate					
**PGN_1459**	P vs T=1	1.391	1.196	-0.003	-0.519	0.499
	PS vs T=1	2.206	1.972	0.674	-0.216	-0.528
	PS vs P	0.834	0.794	0.605	0.102	-0.988
	conserved hypothetical protein					
	hypothetical proteins-Conserved					
**PGN_1460**	P vs T=1	1.062	0.865	-0.010	-0.374	0.095
	PS vs T=1	2.288	2.039	1.032	-0.008	-0.297
	PS vs P	1.230	1.172	0.945	0.158	-0.401
	putative guanylate kinase					
	purines, pyrimidines, nucleosides and nucleotides					
**PGN_1461**	P vs T=1	0.319	-0.164	-0.904	-0.489	0.771
	PS vs T=1	1.456	1.030	-0.120	-0.645	-0.375
	PS vs P	1.141	1.152	0.581	-0.236	-1.038
	putative spore maturation protein A/B					
	cellular processes					
**PGN_1462**	P vs T=1	-0.845	-0.980	-1.548	-1.411	-0.957
	PS vs T=1	-0.198	-0.181	-0.957	-1.476	-1.329
	PS vs P	0.634	0.773	0.433	-0.180	-0.355
	conserved hypothetical protein					
	hypothetical proteins-Conserved					
**PGN_1463**	P vs T=1	-0.246	-0.858	-1.085	-0.309	1.025
	PS vs T=1	0.386	0.480	-0.478	-0.348	-0.207
	PS vs P	0.622	1.154	0.284	-0.035	-0.993
	probable UbiA prenyltransferase					
	cell envelope					

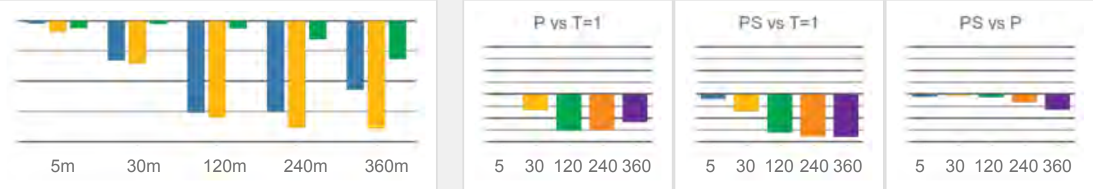

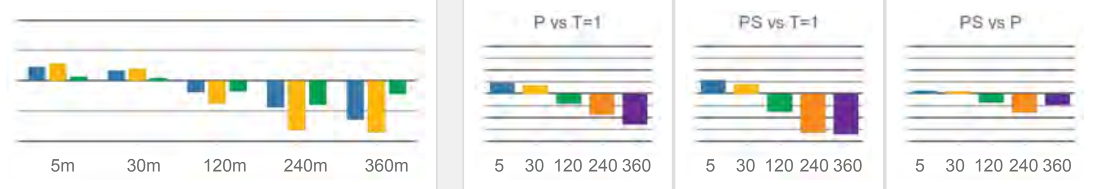

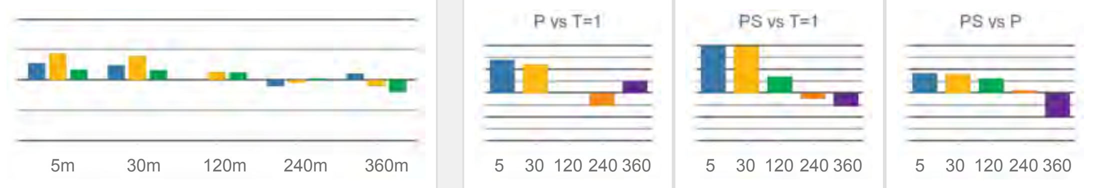

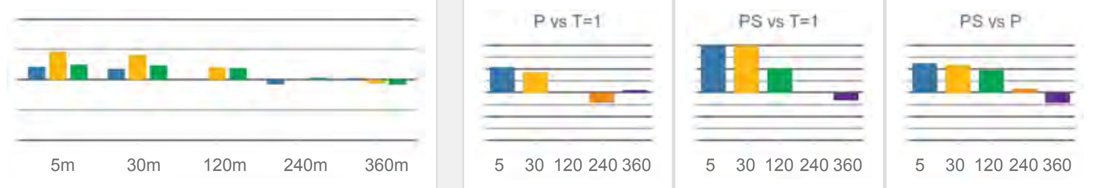

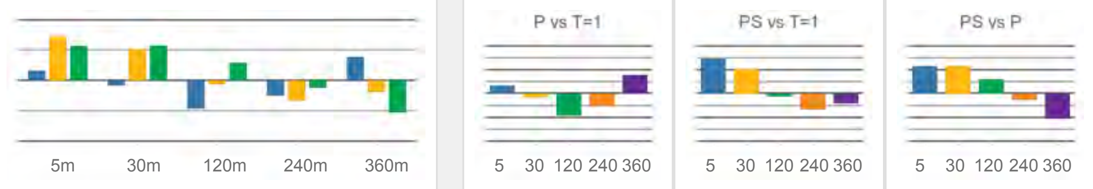

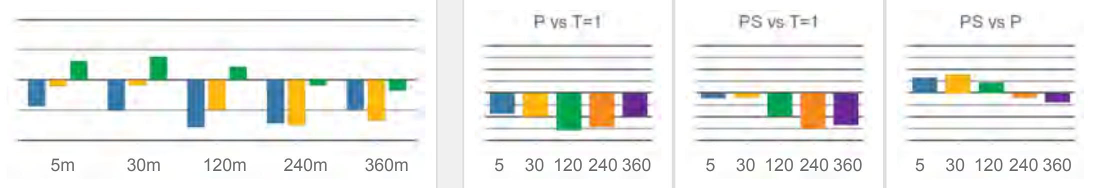

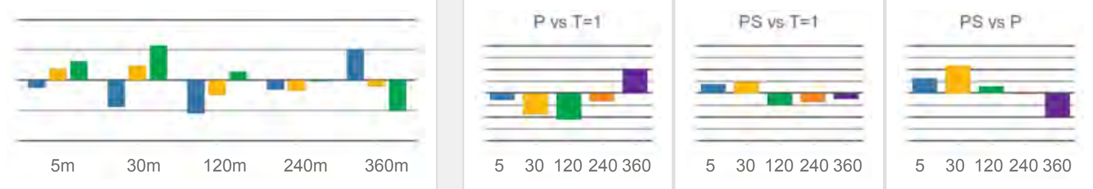

PgSg Time Course

log_2 (Fold Change)

P vs T=1 | PS vs T=1 | PS vs P

PGN_1464

Locus	5m	30m	120m	240m	360m
P vs T=1	-0.272	-0.632	-0.573	0.160	1.369
PS vs T=1	-0.381	-0.596	-1.137	-0.761	-0.705
PS vs P	-0.074	-0.038	-0.646	-0.683	-1.657

probable HAD-superfamily subfamily IB hydrolase

unknown function

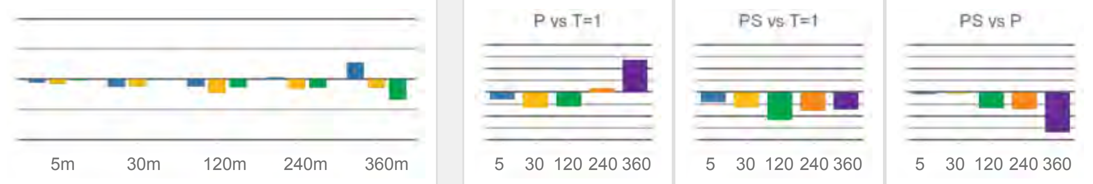

PGN_1465

Locus	5m	30m	120m	240m	360m
P vs T=1	-0.591	-0.410	-0.094	0.446	0.061
PS vs T=1	-0.994	-1.026	-0.840	-0.843	-0.334
PS vs P	-0.418	-0.584	-0.676	-1.011	-0.331

hypothetical protein

hypothetical proteins

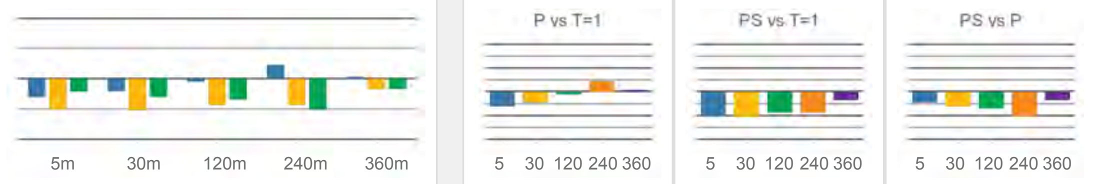

PGN_1466

rgpB

Locus	5m	30m	120m	240m	360m
P vs T=1	0.326	0.384	0.123	-0.568	-1.472
PS vs T=1	0.237	0.278	-0.309	-1.110	-1.300
PS vs P	-0.042	-0.047	-0.381	-0.546	0.117

arginine-specific cysteine proteinase RgpB

protein fate

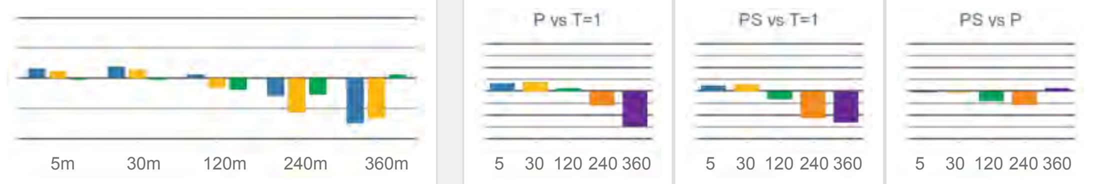

PGN_1467

Locus	5m	30m	120m	240m	360m
P vs T=1	2.145	2.082	1.763	1.107	0.344
PS vs T=1	1.230	1.466	1.237	0.937	0.745
PS vs P	-0.716	-0.433	-0.375	-0.158	0.259

conserved hypothetical protein

hypothetical proteins-Conserved

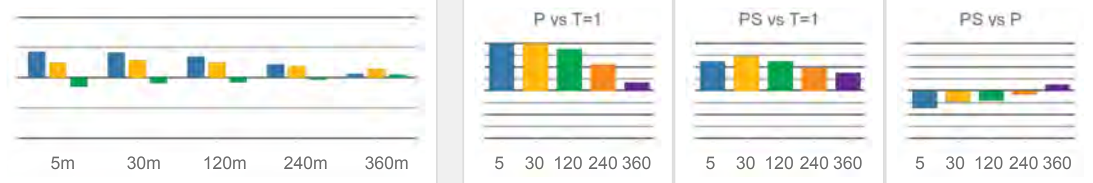

PGN_1468

Locus	5m	30m	120m	240m	360m
P vs T=1	0.052	0.038	-0.350	-0.952	-0.634
PS vs T=1	1.042	0.815	-0.278	-1.067	-0.963
PS vs P	0.988	0.779	0.073	-0.199	-0.335

putative lipoic acid synthase

biosynthesis of cofactors, prosthetic groups, and carriers

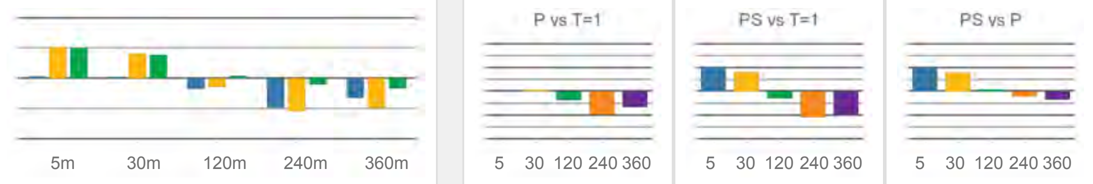

PGN_1469

dppIV

Locus	5m	30m	120m	240m	360m
P vs T=1	-0.168	-0.037	-0.321	-0.779	-0.671
PS vs T=1	0.655	0.331	-0.877	-1.474	-1.324
PS vs P	0.822	0.377	-0.544	-0.709	-0.651

dipeptidyl peptidase IV

protein fate

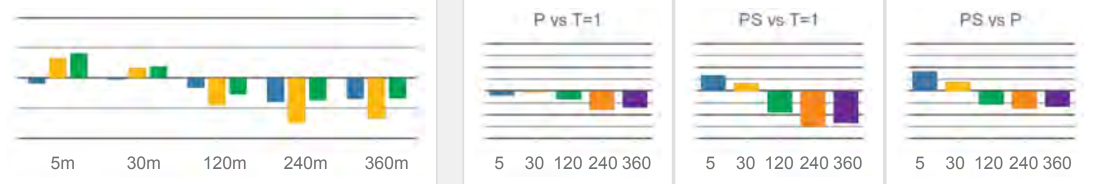

PGN_1470

Locus	5m	30m	120m	240m	360m
P vs T=1	-0.264	-0.470	-1.128	-0.972	-1.094
PS vs T=1	-0.085	-0.361	-1.148	-1.755	-1.521
PS vs P	0.222	0.142	-0.084	-0.743	-0.435

putative SsrA-binding protein

protein synthesis

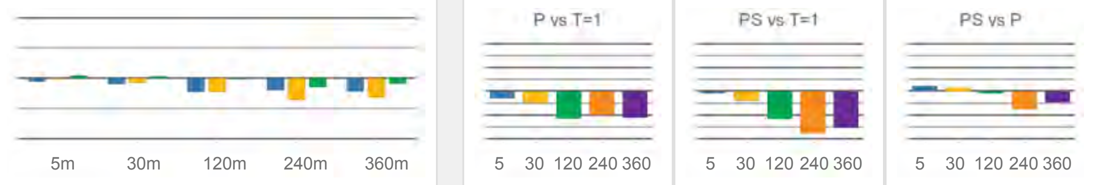

log$_2$ (Fold Change)

Legend: P vs T=1 | PS vs T=1 | PS vs P

Locus		5m	30m	120m	240m	360m
**PGN_1471**	P vs T=1	0.483	0.388	0.162	0.458	0.928
	PS vs T=1	0.753	0.386	-0.190	-0.137	0.319
	PS vs P	0.286	0.012	-0.370	-0.538	-0.529

conserved hypothetical protein

hypothetical proteins-Conserved

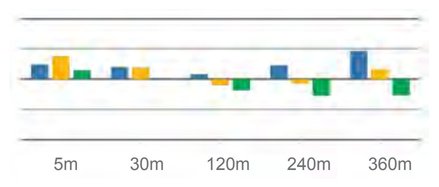

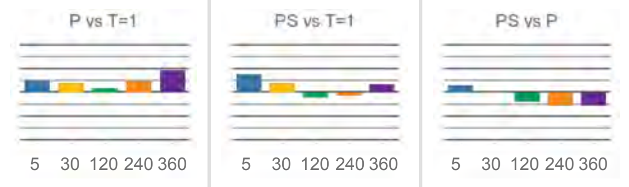

Locus		5m	30m	120m	240m	360m
**PGN_1472**	P vs T=1	-0.435	-0.616	-0.569	-0.526	-0.569
	PS vs T=1	-0.465	-1.235	-1.477	-1.064	-0.929
	PS vs P	-0.011	-0.604	-0.883	-0.504	-0.346

queuine tRNA-ribosyltransferase

protein synthesis

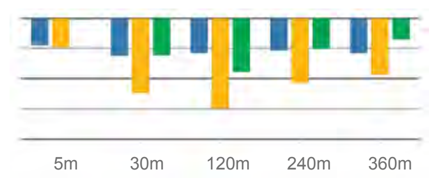

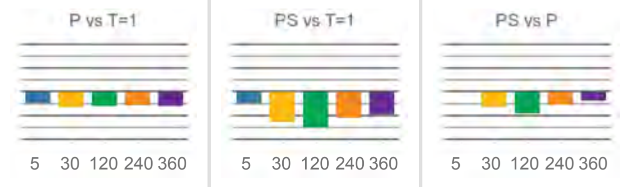

Locus		5m	30m	120m	240m	360m
**PGN_1473**	P vs T=1	-0.024	0.070	0.394	1.110	1.509
	PS vs T=1	0.158	0.067	0.571	0.916	0.435
	PS vs P	0.088	-0.079	0.125	0.087	-0.742

hypothetical protein

hypothetical proteins

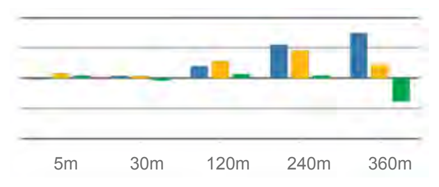

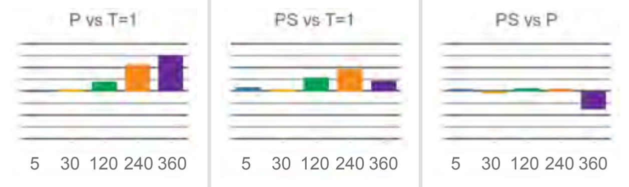

Locus		5m	30m	120m	240m	360m
**PGN_1474**	P vs T=1	0.550	0.924	1.156	1.162	1.329
	PS vs T=1	0.855	1.111	1.280	1.298	1.347
	PS vs P	0.243	0.189	0.163	0.168	0.079

putative autoinducer-2 production protein LuxS

cellular processes

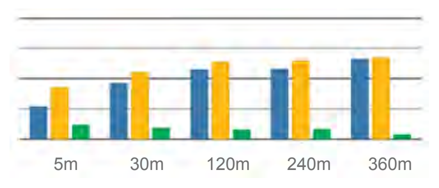

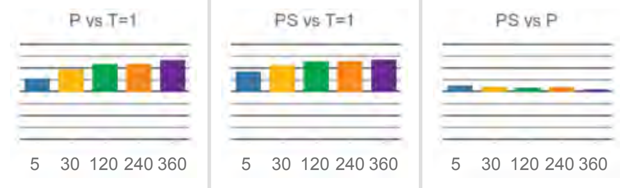

Locus		5m	30m	120m	240m	360m
**PGN_1475**	P vs T=1	0.066	0.226	0.277	0.437	0.603
	PS vs T=1	-0.360	-0.637	-0.163	-0.052	0.218
	PS vs P	-0.407	-0.805	-0.397	-0.403	-0.317

probable 5'-methylthioadenosine/S-adenosylhomocysteine nucleosidase

amino acid biosynthesis

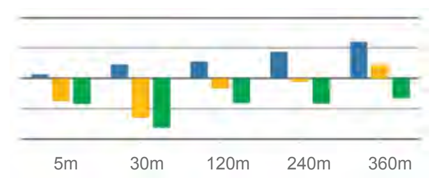

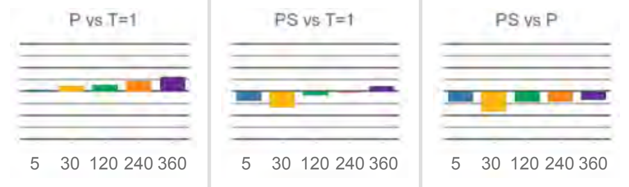

Locus		5m	30m	120m	240m	360m
**PGN_1476**	P vs T=1	1.553	1.215	1.823	2.466	2.965
	PS vs T=1	0.689	0.766	1.630	1.929	2.064
	PS vs P	-0.803	-0.468	-0.158	-0.393	-0.766

conserved hypothetical protein

hypothetical proteins-Conserved

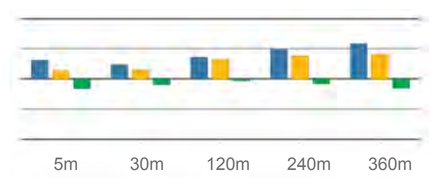

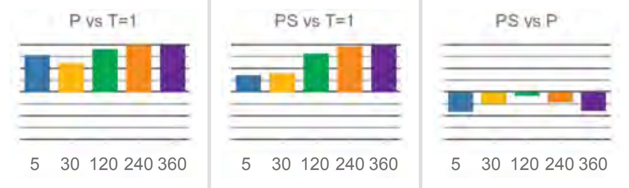

Locus		5m	30m	120m	240m	360m
**PGN_1477**	P vs T=1	-0.186	-0.622	1.069	1.840	2.781
	PS vs T=1	-0.819	-0.612	1.428	1.163	1.463
	PS vs P	-0.636	-0.509	0.603	0.083	-0.116

conserved hypothetical protein

hypothetical proteins-Conserved

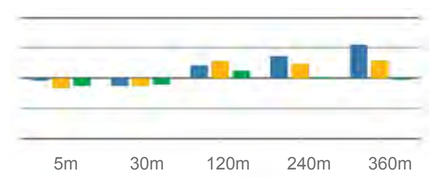

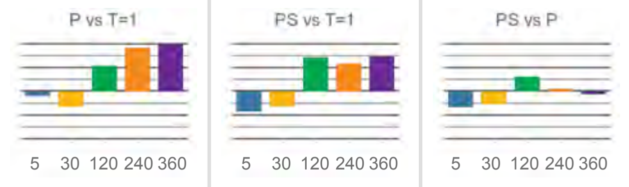

PgSg Time Course

Hackett Laboratory UW

log2 (Fold Change)

Locus		5m	30m	120m	240m	360m
**PGN_1478**	P vs T=1	0.145	-0.299	1.301	2.487	3.751
	PS vs T=1	-1.000	0.020	1.280	2.107	2.239
	PS vs P	-1.002	-0.298	0.156	0.356	-0.327

conserved hypothetical protein

hypothetical proteins-Conserved

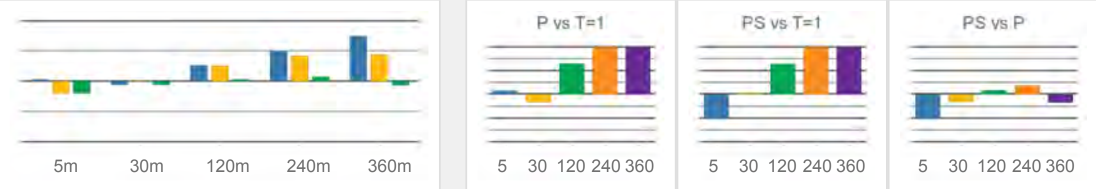

Locus		5m	30m	120m	240m	360m
**PGN_1479** *dpp7*	P vs T=1	-0.053	0.457	0.956	0.921	0.639
	PS vs T=1	0.985	0.980	1.167	1.520	1.288
	PS vs P	0.947	0.491	0.226	0.599	0.631

dipeptidyl peptidase 7

protein fate

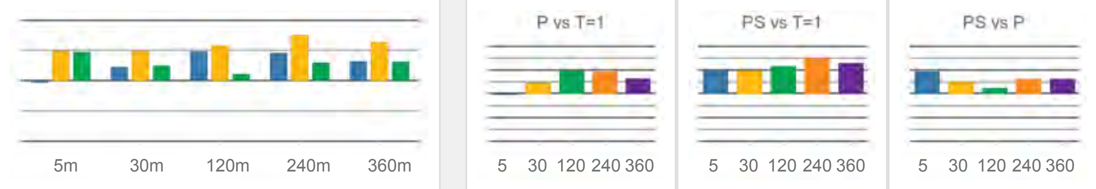

Locus		5m	30m	120m	240m	360m
**PGN_1480**	P vs T=1	-0.155	0.165	0.301	0.165	0.207
	PS vs T=1	0.283	0.782	1.694	2.037	1.615
	PS vs P	0.369	0.578	1.355	1.794	1.365

conserved hypothetical protein with DUF389 domain

cell envelope

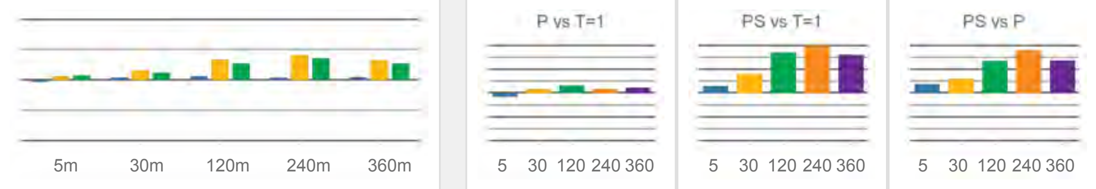

Locus		5m	30m	120m	240m	360m
**PGN_1481**	P vs T=1	-1.034	-1.281	-1.206	-1.017	-0.252
	PS vs T=1	-1.091	-1.081	-1.341	-1.366	-1.287
	PS vs P	-0.059	0.162	-0.163	-0.349	-0.962

putative polysaccharide biosynthesis protein

cell envelope

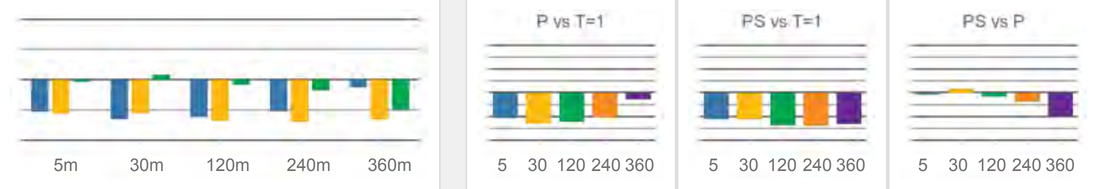

Locus		5m	30m	120m	240m	360m
**PGN_1482** *ruvB*	P vs T=1	-0.730	-0.731	-0.550	-0.139	0.125
	PS vs T=1	-0.649	-0.896	-1.170	-0.752	-0.682
	PS vs P	0.068	-0.180	-0.623	-0.549	-0.755

holliday junction DNA helicase RuvB

DNA metabolism

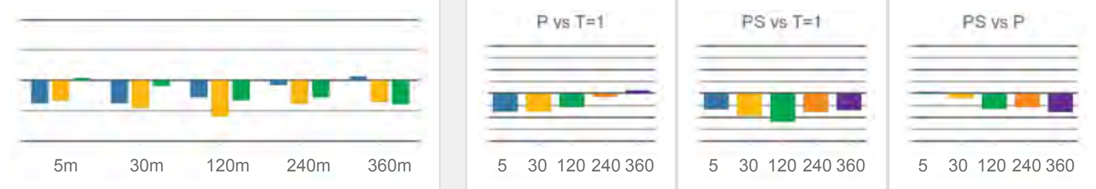

Locus		5m	30m	120m	240m	360m
**PGN_1483**	P vs T=1	1.141	0.245	0.766	2.239	3.286
	PS vs T=1	0.546	0.567	1.757	1.515	1.888
	PS vs P	-0.340	-0.098	0.621	0.022	-0.418

conserved hypothetical protein

hypothetical proteins-Conserved

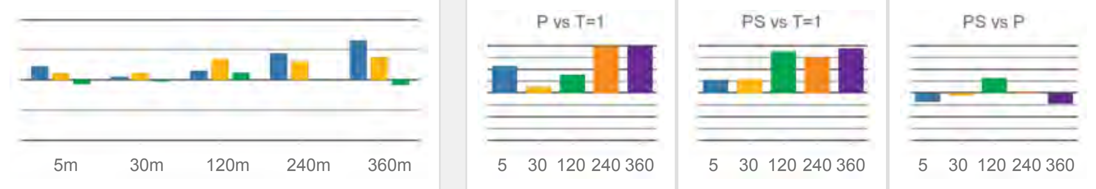

Locus		5m	30m	120m	240m	360m
**PGN_1484**	P vs T=1	0.420	-0.050	-0.392	-0.473	-0.322
	PS vs T=1	-0.365	-0.647	-0.631	-0.441	-0.569
	PS vs P	-0.736	-0.572	-0.243	0.015	-0.236

putative methylated-DNA-protein-cysteine methyltransferase

DNA metabolism

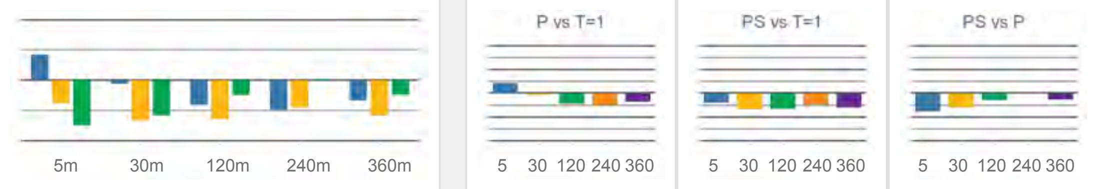

PgSg Time Course

log$_2$ (Fold Change)

Legend: P vs T=1 | PS vs T=1 | PS vs P

Locus		5m	30m	120m	240m	360m
**PGN_1485**	P vs T=1	1.454	1.724	2.083	2.055	1.267
	PS vs T=1	1.000	1.392	1.933	2.107	1.958
	PS vs P	-0.450	-0.316	-0.122	0.076	0.675

conserved hypothetical protein

protein fate

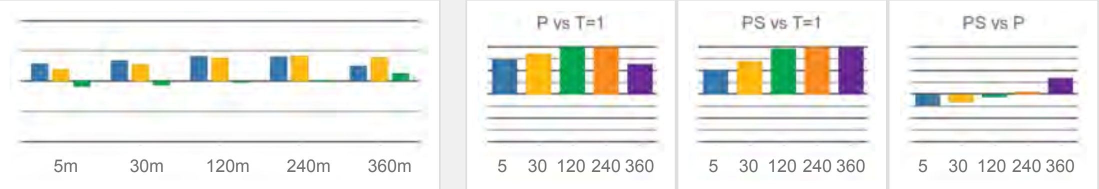

Locus		5m	30m	120m	240m	360m
**PGN_1486**	P vs T=1	0.028	0.266	0.463	0.340	0.089
	PS vs T=1	0.329	0.482	0.787	1.007	0.832
	PS vs P	0.283	0.209	0.326	0.653	0.721

conserved hypothetical protein

hypothetical proteins-Conserved

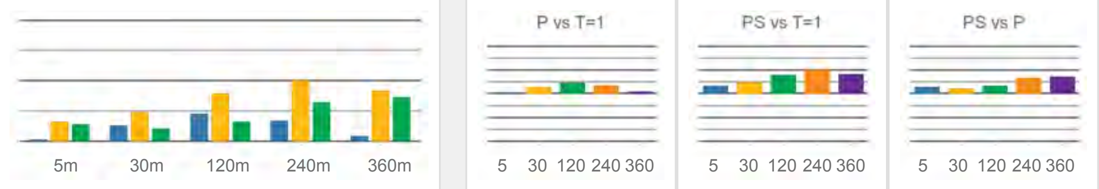

Locus		5m	30m	120m	240m	360m
**PGN_1487**	P vs T=1	-0.351	-0.192	-0.051	0.191	0.892
	PS vs T=1	0.233	0.323	0.376	0.977	0.680
	PS vs P	0.496	0.444	0.345	0.730	-0.132

probable dephospho-CoA kinase

central intermediary metabolism

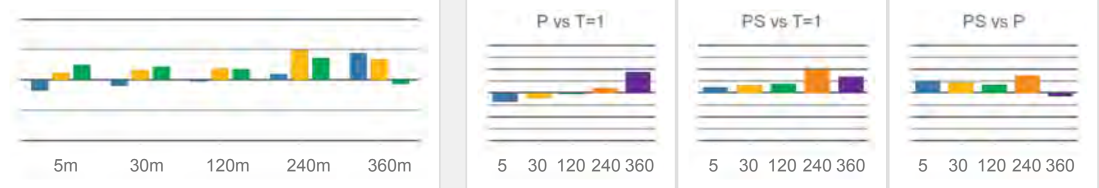

Locus		5m	30m	120m	240m	360m
**PGN_1488**	P vs T=1	0.381	0.207	-0.297	-0.269	-0.296
	PS vs T=1	0.481	0.750	1.122	0.947	0.505
	PS vs P	0.112	0.526	1.308	1.108	0.734

conserved hypothetical protein

hypothetical proteins-Conserved

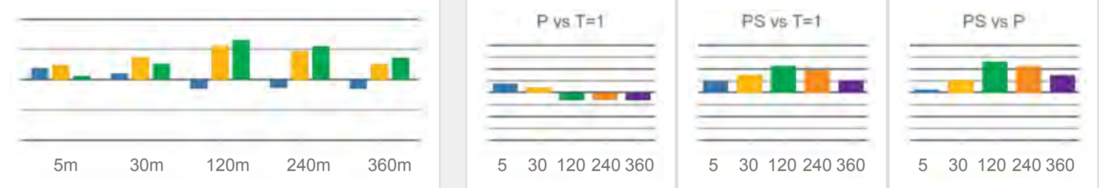

Locus		5m	30m	120m	240m	360m
**PGN_1489**	P vs T=1	1.043	1.620	1.972	1.776	1.137
	PS vs T=1	1.516	1.744	1.979	1.895	1.715
	PS vs P	0.427	0.142	0.051	0.140	0.554

2-amino-3-ketobutyrate CoA ligase

biosynthesis of cofactors, prosthetic groups, and carriers

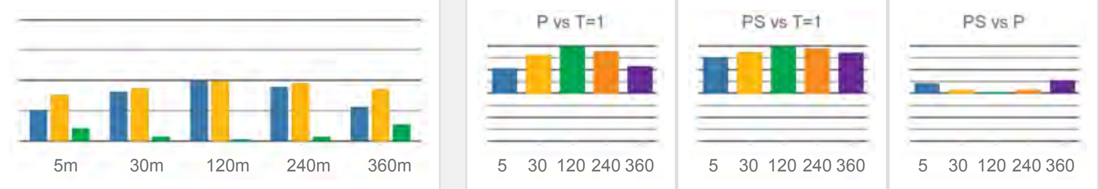

Locus		5m	30m	120m	240m	360m
**PGN_1490**	P vs T=1	1.284	1.242	1.725	2.013	1.885
CobI	PS vs T=1	-0.055	-0.061	0.409	0.738	0.597
	PS vs P	-1.280	-1.247	-1.238	-1.159	-1.215

probable precorrin-2 C20-methyltransferase

biosynthesis of cofactors, prosthetic groups, and carriers

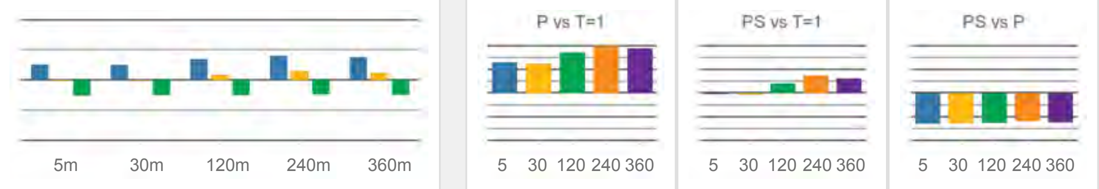

Locus		5m	30m	120m	240m	360m
**PGN_1491**	P vs T=1	1.118	0.835	0.508	0.522	0.581
	PS vs T=1	-0.374	-0.308	0.479	0.223	0.315
	PS vs P	-1.414	-1.081	-0.015	-0.267	-0.237

conserved hypothetical protein

hypothetical proteins-Conserved

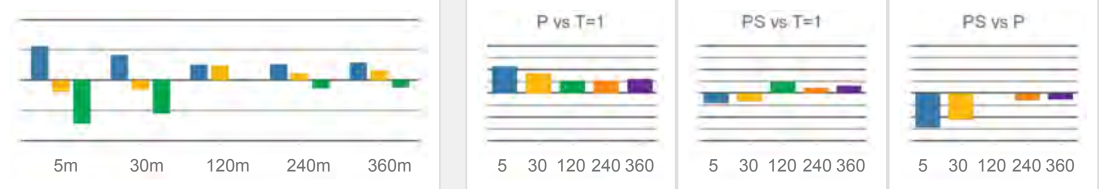

PgSg Time Course

log$_2$ (Fold Change)

P vs T=1 | PS vs T=1 | PS vs P

PGN_1492

Locus		5m	30m	120m	240m	360m
PGN_1492	P vs T=1	0.043	0.437	0.687	0.660	0.867
	PS vs T=1	-0.030	0.344	0.946	1.639	1.514
	PS vs P	-0.135	-0.089	0.274	0.954	0.661

putative pantoate-beta-alanine ligase

biosynthesis of cofactors, prosthetic groups, and carriers

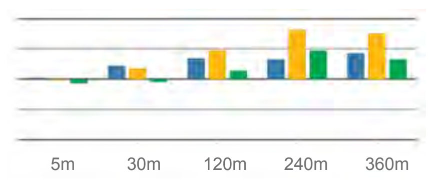

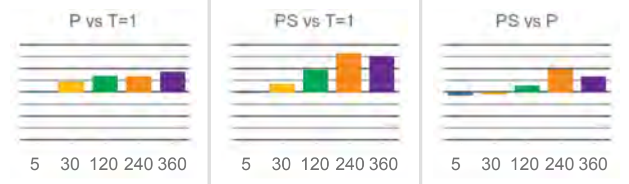

PGN_1493

Locus		5m	30m	120m	240m	360m
PGN_1493	P vs T=1	0.010	0.260	0.902	1.130	1.142
	PS vs T=1	-0.454	-0.469	-0.246	0.544	0.606
	PS vs P	-0.472	-0.707	-1.065	-0.487	-0.475

conserved hypothetical protein

unknown function

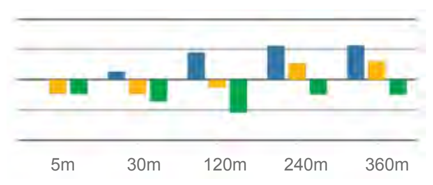

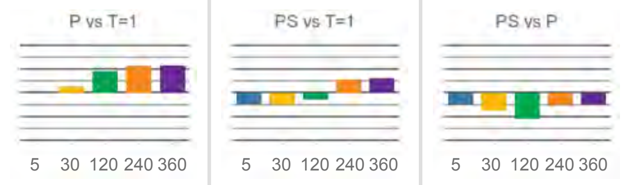

PGN_1494

HemN

Locus		5m	30m	120m	240m	360m
PGN_1494	P vs T=1	-0.788	-1.170	-0.749	-0.081	0.837
	PS vs T=1	-0.878	-1.239	-1.547	-0.619	-0.578
	PS vs P	-0.082	-0.163	-0.797	-0.347	-1.170

putative oxygen-independent coproporphyrinogen III oxidase

biosynthesis of cofactors, prosthetic groups, and carriers

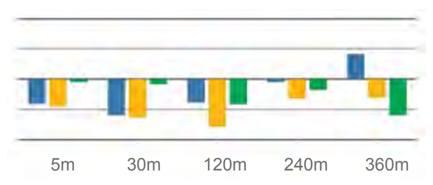

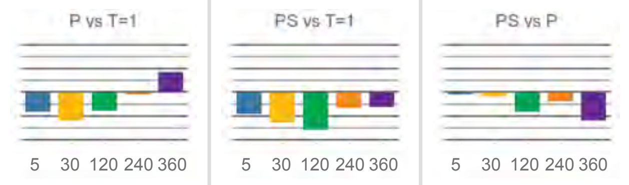

PGN_1495

Locus		5m	30m	120m	240m	360m
PGN_1495	P vs T=1	-0.601	-0.868	-1.099	-0.766	0.305
	PS vs T=1	-0.471	-1.269	-1.558	-1.470	-0.971
	PS vs P	0.159	-0.387	-0.503	-0.636	-1.096

putative low-specificity L-threonine aldolase

energy metabolism

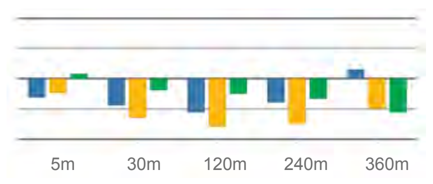

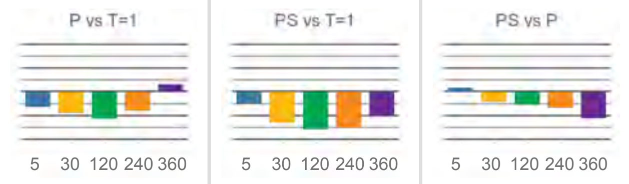

PGN_1496

Locus		5m	30m	120m	240m	360m
PGN_1496	P vs T=1	-1.387	-0.734	0.173	0.411	-0.104
	PS vs T=1	-1.051	-0.545	-0.111	-0.160	-0.172
	PS vs P	0.164	0.128	-0.224	-0.487	-0.073

hypothetical protein

hypothetical proteins

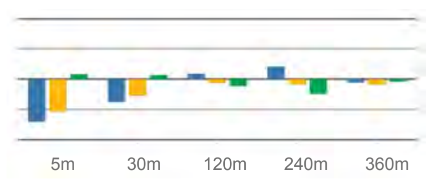

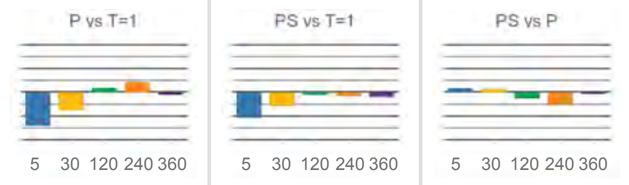

PGN_1497

Locus		5m	30m	120m	240m	360m
PGN_1497	P vs T=1	-0.835	-0.641	-0.176	0.243	0.469
	PS vs T=1	-0.425	-0.403	-0.518	-0.284	0.017
	PS vs P	0.318	0.172	-0.338	-0.442	-0.386

putative iron-sulfur cluster binding protein

transport and binding proteins

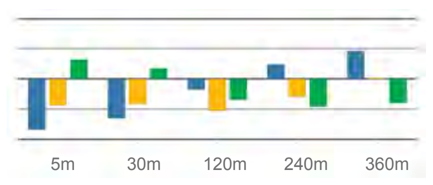

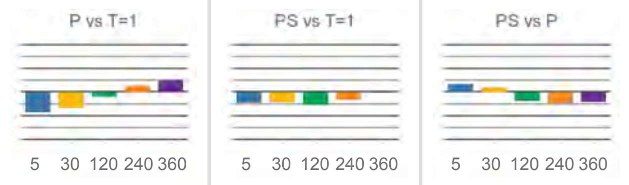

PGN_1498

Locus		5m	30m	120m	240m	360m
PGN_1498	P vs T=1	0.350	0.233	0.358	0.311	-0.186
	PS vs T=1	1.193	1.215	1.473	1.514	1.165
	PS vs P	0.818	0.946	1.093	1.175	1.308

conserved hypothetical protein

hypothetical proteins-Conserved

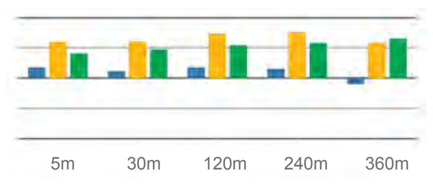

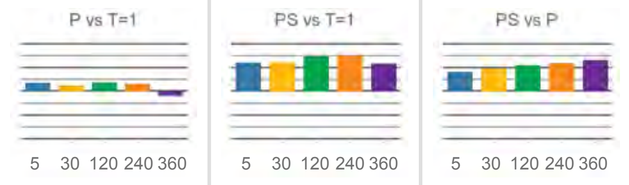

PgSg Time Course

log$_2$ (Fold Change)

Legend: P vs T=1 | PS vs T=1 | PS vs P

Locus		5m	30m	120m	240m	360m
**PGN_1499**	P vs T=1	0.099	0.098	0.743	2.723	4.120
	PS vs T=1	0.352	0.376	1.091	0.899	0.814
	PS vs P	-0.050	-0.076	0.134	-0.251	-1.008

conserved hypothetical protein

hypothetical proteins-Conserved

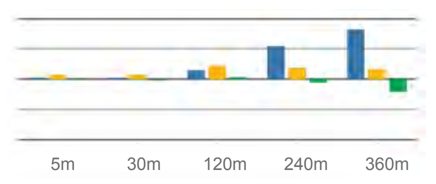

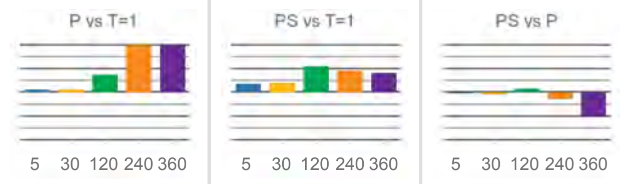

Locus		5m	30m	120m	240m	360m
**PGN_1500**	P vs T=1	0.381	0.341	0.093	-0.071	0.170
	PS vs T=1	0.597	0.097	0.019	0.360	0.200
	PS vs P	0.223	-0.228	-0.102	0.339	0.021

conserved hypothetical protein

hypothetical proteins-Conserved

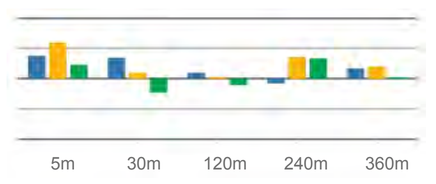

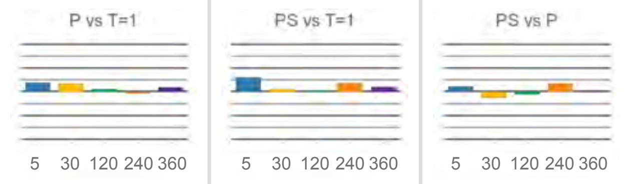

Locus		5m	30m	120m	240m	360m
**PGN_1501**	P vs T=1	0.542	0.718	0.626	0.451	0.208
Mpi	PS vs T=1	0.869	0.586	-0.017	0.022	-0.063
	PS vs P	0.323	-0.105	-0.615	-0.421	-0.287

putative mannose-6-phosphate isomerase

energy metabolism

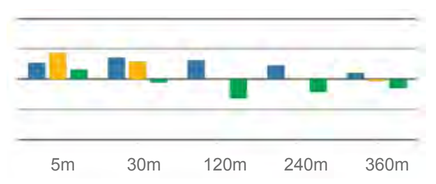

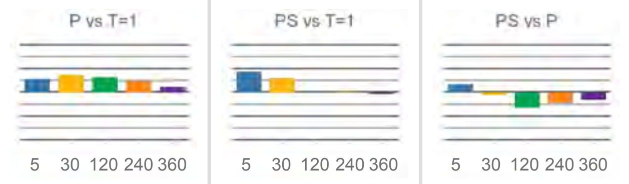

Locus		5m	30m	120m	240m	360m
**PGN_1502**	P vs T=1	0.157	0.713	0.640	0.313	0.196
	PS vs T=1	0.240	0.299	-0.014	-0.172	-0.022
	PS vs P	0.072	-0.388	-0.628	-0.483	-0.221

conserved hypothetical protein

hypothetical proteins-Conserved

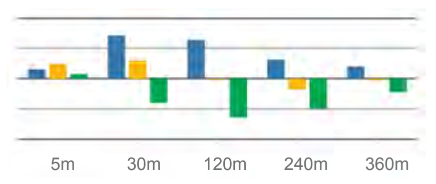

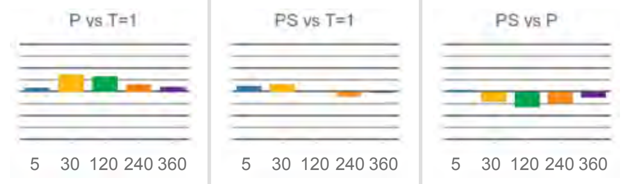

Locus		5m	30m	120m	240m	360m
**PGN_1503**	P vs T=1	-0.111	-0.235	-0.383	-0.646	-1.228
	PS vs T=1	-0.013	-0.200	-0.643	-1.131	-1.333
	PS vs P	0.116	0.046	-0.246	-0.482	-0.146

probable ferric uptake transcriptional regulator

regulatory functions

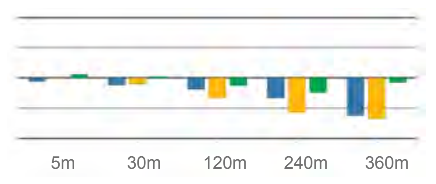

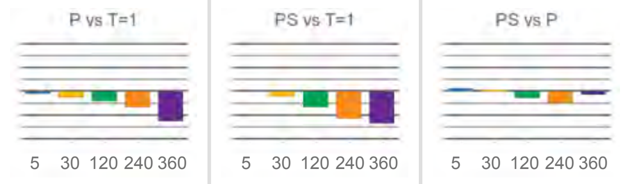

Locus		5m	30m	120m	240m	360m
**PGN_1504**	P vs T=1	0.015	0.236	0.197	-0.223	-0.643
	PS vs T=1	0.850	0.476	-0.491	-0.866	-0.853
	PS vs P	0.822	0.253	-0.658	-0.642	-0.233

adenylosuccinate synthetase

purines, pyrimidines, nucleosides and nucleotides

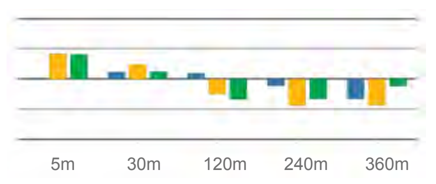

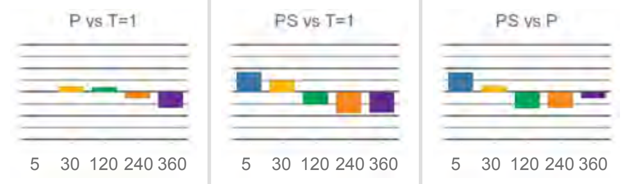

Locus		5m	30m	120m	240m	360m
**PGN_1505**	P vs T=1	-0.108	-0.202	-0.332	-0.543	-0.600
	PS vs T=1	1.061	0.601	-0.422	-0.912	-0.910
	PS vs P	1.149	0.780	-0.096	-0.394	-0.328

putative folylpolyglutamate synthase

biosynthesis of cofactors, prosthetic groups, and carriers

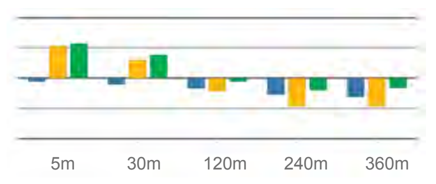

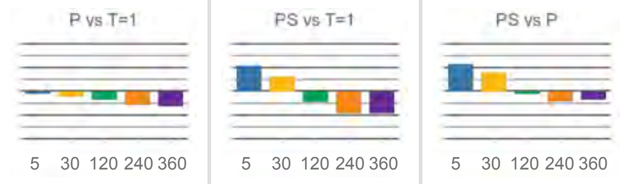

log$_2$ (Fold Change)

Locus		5m	30m	120m	240m	360m
**PGN_1506**	P vs T=1	0.760	0.787	0.777	0.378	0.034
	PS vs T=1	1.739	1.766	1.156	0.673	0.367
	PS vs P	0.959	0.962	0.386	0.266	0.298
	putative transporter					
	transport and binding proteins					
**PGN_1507**	P vs T=1	-1.692	-1.868	-1.918	-1.982	-2.103
	PS vs T=1	-0.907	-0.505	0.140	0.477	0.353
	PS vs P	0.572	1.078	1.784	2.126	2.179
	hypothetical protein					
	hypothetical proteins					
**PGN_1508**	P vs T=1	0.214	-0.221	-0.284	-0.124	0.727
	PS vs T=1	0.435	1.203	1.797	1.967	1.473
	PS vs P	0.171	1.126	1.657	1.647	0.817
	hypothetical protein					
	hypothetical proteins					
**PGN_1509**	P vs T=1	-0.434	-0.102	0.423	0.708	0.568
	PS vs T=1	-0.741	-0.720	-0.226	0.041	-0.097
	PS vs P	-0.357	-0.607	-0.590	-0.572	-0.620
	conserved hypothetical protein					
	hypothetical proteins-Conserved					
**PGN_1510**	P vs T=1	0.259	0.194	0.134	-0.126	-0.485
	PS vs T=1	0.034	-0.024	0.102	0.211	0.046
	PS vs P	-0.215	-0.211	-0.027	0.328	0.514
	putative peptidyl-prolyl cis-trans isomerase					
	hypothetical proteins					
**PGN_1511**	P vs T=1	-1.094	-2.074	-2.090	-1.394	-0.367
	PS vs T=1	-0.572	-1.320	-1.267	-1.306	-1.462
	PS vs P	0.544	0.582	0.633	0.085	-0.974
	probable hemolysin					
	unknown function					
**PGN_1512**	P vs T=1	-0.830	-0.377	0.265	0.540	0.502
	PS vs T=1	-0.725	-0.469	-0.031	0.040	0.245
	PS vs P	0.012	-0.122	-0.262	-0.440	-0.228
	conserved hypothetical protein					
	hypothetical proteins-Conserved					

P vs T=1 | PS vs T=1 | PS vs P

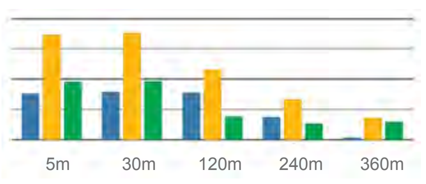

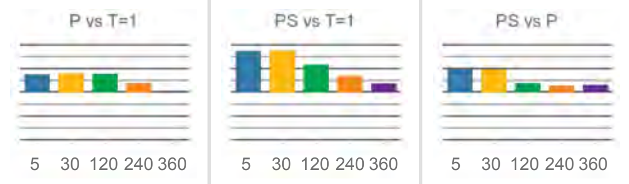

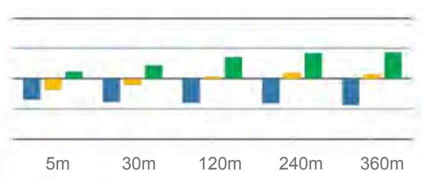

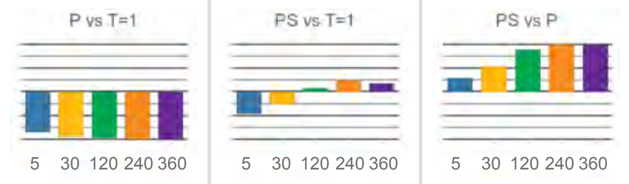

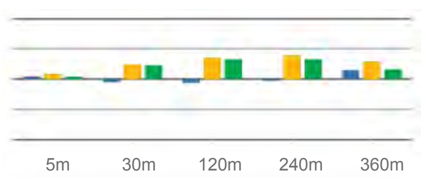

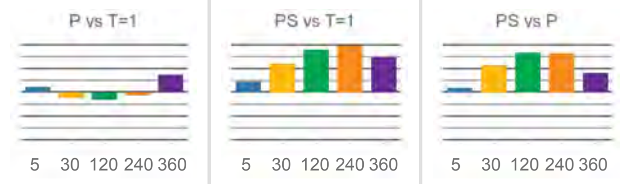

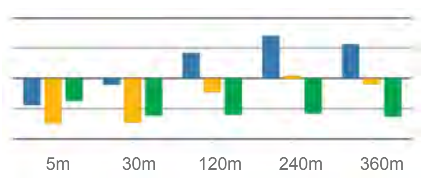

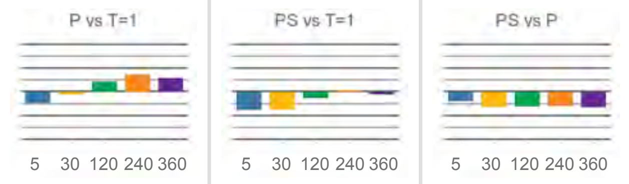

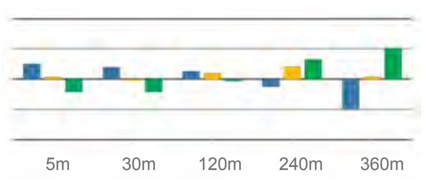

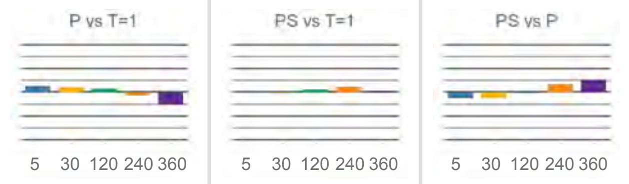

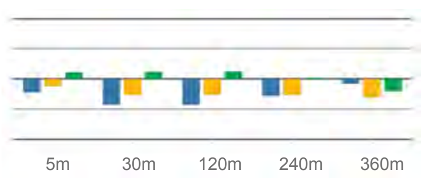

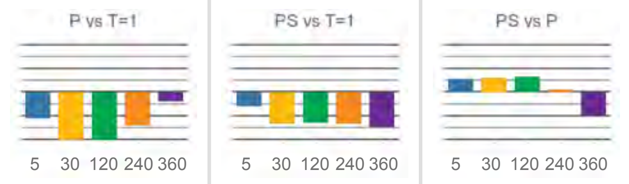

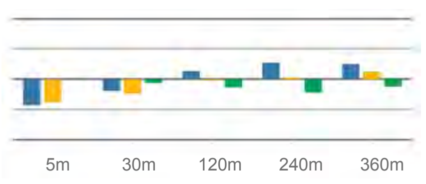

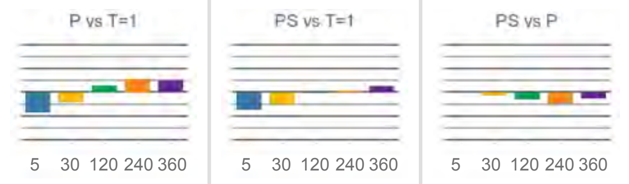

log$_2$ (Fold Change)

PGN_1513

Locus		5m	30m	120m	240m	360m
PGN_1513	P vs T=1	0.431	0.709	0.872	0.814	0.541
	PS vs T=1	0.407	1.001	1.150	0.811	0.752
	PS vs P	-0.037	0.292	0.286	0.003	0.206

conserved hypothetical protein

unknown function

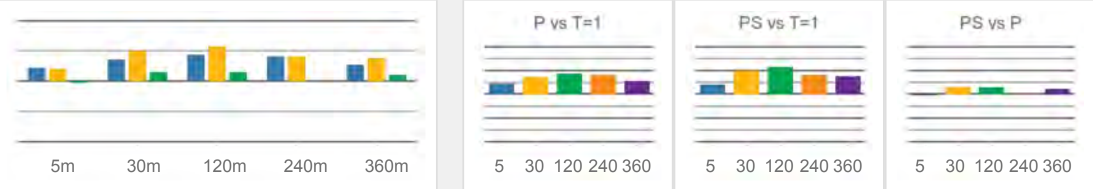

PGN_1514

Locus		5m	30m	120m	240m	360m
PGN_1514	P vs T=1	0.549	0.312	0.206	0.149	-0.155
	PS vs T=1	0.461	0.786	0.728	0.491	0.420
	PS vs P	-0.073	0.462	0.509	0.329	0.549

conserved hypothetical protein

hypothetical proteins-Conserved

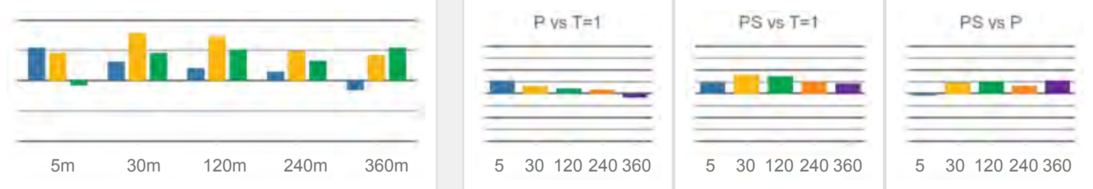

PGN_1515

Locus		5m	30m	120m	240m	360m
PGN_1515	P vs T=1	0.116	0.266	0.300	0.518	1.171
	PS vs T=1	1.245	1.198	0.602	0.401	0.324
	PS vs P	1.030	0.864	0.251	-0.095	-0.702

conserved hypothetical protein

hypothetical proteins-Conserved

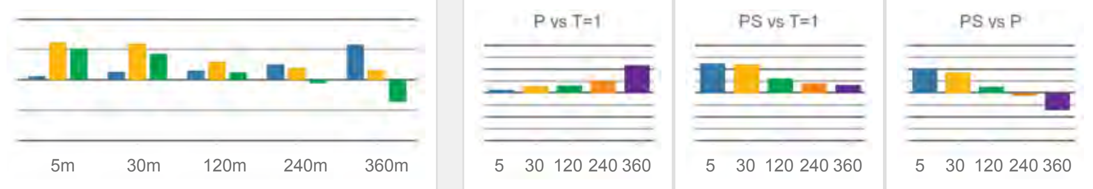

PGN_1516

Locus		5m	30m	120m	240m	360m
PGN_1516	P vs T=1	-0.463	-0.325	0.079	0.019	0.446
	PS vs T=1	0.018	-0.209	-0.743	-0.325	-0.227
	PS vs P	0.423	0.090	-0.728	-0.287	-0.555

probable molybdopterin biosynthesis MoeB protein

biosynthesis of cofactors, prosthetic groups, and carriers

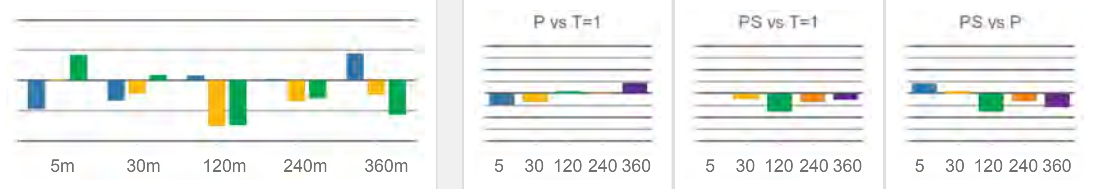

PGN_1517 *pepT*

Locus		5m	30m	120m	240m	360m
PGN_1517	P vs T=1	0.321	1.256	2.299	2.015	1.346
pepT	PS vs T=1	0.823	1.221	1.801	1.858	1.668
	PS vs P	0.347	-0.024	-0.351	-0.078	0.310

peptidase T

protein fate

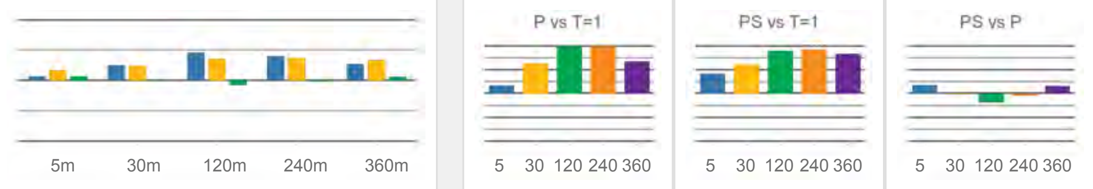

PGN_1518

Locus		5m	30m	120m	240m	360m
PGN_1518	P vs T=1	0.032	0.201	1.017	0.890	0.687
	PS vs T=1	0.347	0.505	0.480	0.583	0.707
	PS vs P	0.228	0.237	-0.424	-0.243	0.035

putative oligopeptide transporter

transport and binding proteins

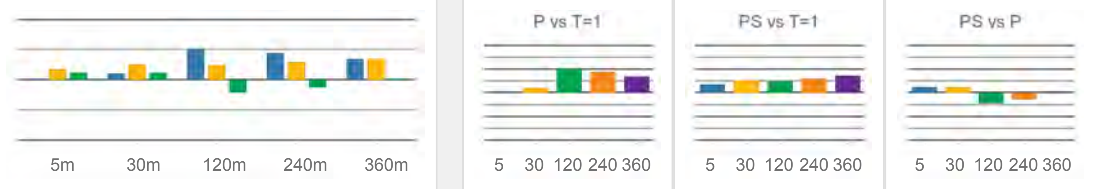

PGN_1519

Locus		5m	30m	120m	240m	360m
PGN_1519	P vs T=1	0.001	-0.174	-0.295	-0.587	-0.593
	PS vs T=1	0.336	-0.019	-0.992	-1.004	-0.764
	PS vs P	0.348	0.159	-0.683	-0.429	-0.177

hemagglutinin-related protein

unknown function

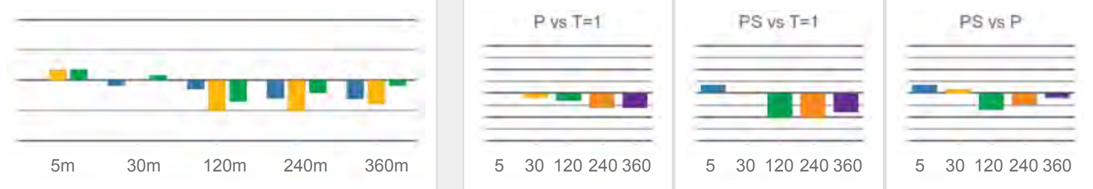

PgSg Time Course

log2 (Fold Change)

Legend: P vs T=1 | PS vs T=1 | PS vs P

PGN_1520

Locus		5m	30m	120m	240m	360m
PGN_1520	P vs T=1	-0.427	-0.060	0.491	0.765	0.764
	PS vs T=1	-0.288	-0.492	-0.594	-0.259	0.090
	PS vs P	0.092	-0.434	-1.041	-0.953	-0.636

conserved hypothetical protein

hypothetical proteins-Conserved

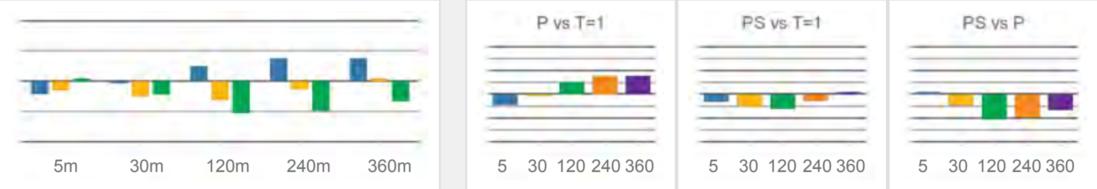

PGN_1521

Locus		5m	30m	120m	240m	360m
PGN_1521	P vs T=1	0.422	0.764	0.956	0.656	0.756
	PS vs T=1	0.784	0.743	0.601	0.506	0.341
	PS vs P	0.337	-0.016	-0.327	-0.167	-0.406

conserved hypothetical protein

hypothetical proteins-Conserved

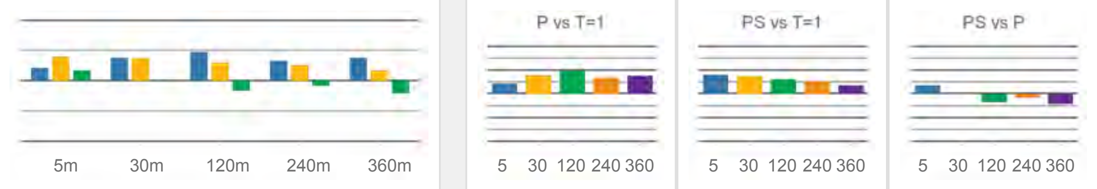

PGN_1522

Locus		5m	30m	120m	240m	360m
PGN_1522	P vs T=1	0.121	0.354	0.385	0.069	0.060
	PS vs T=1	0.285	0.300	0.520	0.337	0.140
	PS vs P	0.148	-0.049	0.142	0.234	0.064

conserved hypothetical protein

hypothetical proteins-Conserved

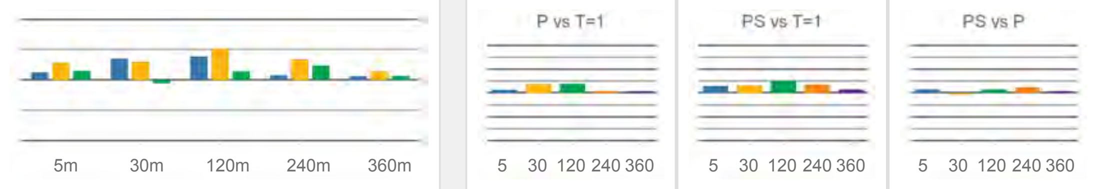

PGN_1523

Locus		5m	30m	120m	240m	360m
PGN_1523	P vs T=1	0.562	0.139	-0.564	-0.687	-0.061
	PS vs T=1	-0.090	0.589	1.041	0.911	0.587
	PS vs P	-0.617	0.432	1.463	1.383	0.618

putative polysaccharide export outer membrane protein

cell envelope

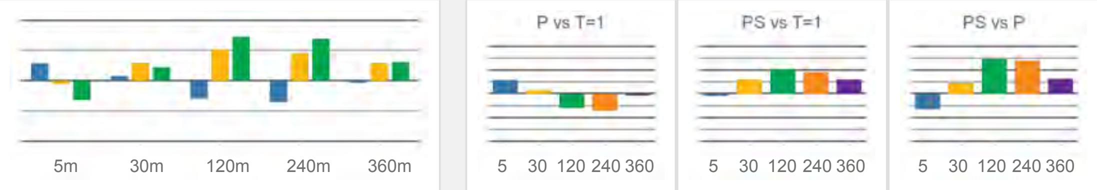

PGN_1524

ptk1

Locus		5m	30m	120m	240m	360m
PGN_1524	P vs T=1	-0.634	-1.415	-2.215	-2.237	-1.810
	PS vs T=1	-1.114	-1.151	-0.651	-0.917	-1.176
	PS vs P	-0.442	0.248	1.485	1.234	0.612

tyrosine kinase

transport and binding proteins

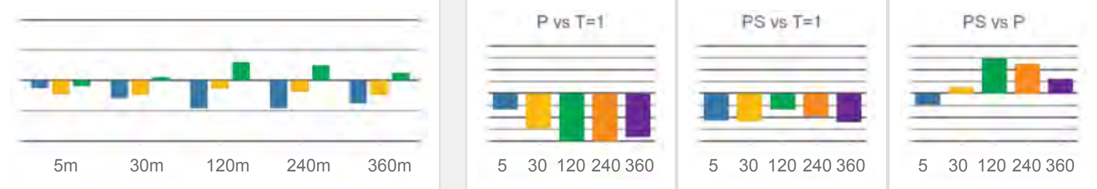

PGN_1525

Locus		5m	30m	120m	240m	360m
PGN_1525	P vs T=1	-0.126	-0.328	0.035	0.106	0.256
	PS vs T=1	0.482	0.231	0.596	0.860	0.852
	PS vs P	0.575	0.493	0.537	0.723	0.588

probable capsular polysaccharide biosynthesis protein

cell envelope

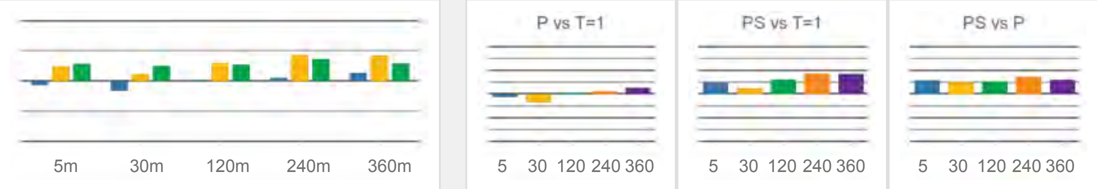

PGN_1526

Locus		5m	30m	120m	240m	360m
PGN_1526	P vs T=1	0.602	0.606	0.351	0.515	0.686
	PS vs T=1	-0.312	1.272	1.996	1.718	1.405
	PS vs P	-0.896	0.646	1.566	1.150	0.708

conserved hypothetical protein

hypothetical proteins-Conserved

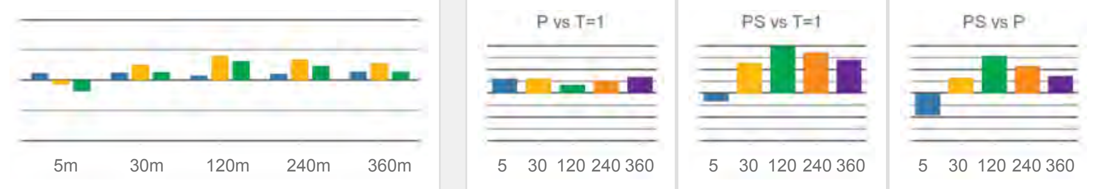

PgSg Time Course

log$_2$ (Fold Change)

P vs T=1 | PS vs T=1 | PS vs P

Locus		5m	30m	120m	240m	360m
**PGN_1527**	P vs T=1	0.120	0.405	0.222	0.281	0.527
	PS vs T=1	-0.816	0.230	1.278	1.283	0.841
	PS vs P	-0.927	-0.149	1.028	0.971	0.330

conserved hypothetical protein

unknown function

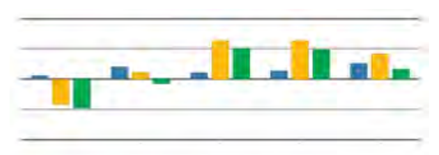

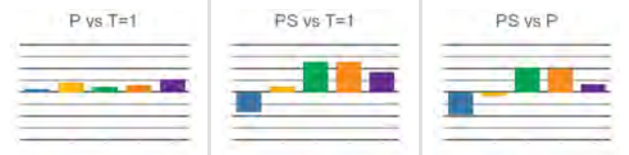

Locus		5m	30m	120m	240m	360m
**PGN_1528**	P vs T=1	0.033	0.644	1.072	1.291	1.632
	PS vs T=1	0.114	0.346	1.280	1.697	1.643
	PS vs P	0.007	-0.291	0.230	0.440	0.060

conserved hypothetical protein

unknown function

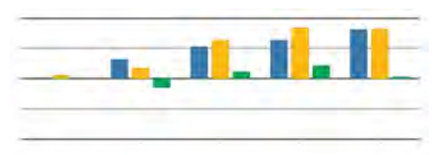

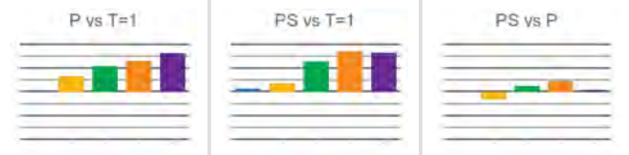

Locus		5m	30m	120m	240m	360m
**PGN_1529**	P vs T=1	1.789	2.060	2.667	2.611	1.783
	PS vs T=1	0.759	0.792	0.717	0.631	0.633
	PS vs P	-0.948	-1.156	-1.789	-1.826	-1.105

putative oxidoreductase

unknown function

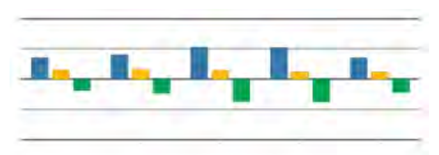

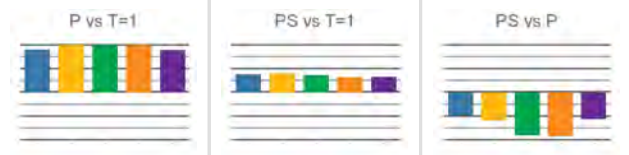

Locus		5m	30m	120m	240m	360m
**PGN_1530**	P vs T=1	0.571	0.518	1.061	1.301	1.063
	PS vs T=1	-0.585	-0.729	-0.671	-0.389	-0.081
	PS vs P	-1.030	-1.139	-1.562	-1.487	-1.035

putative 2-oxoglutarate ferredoxin oxidoreductase subunit

energy metabolism

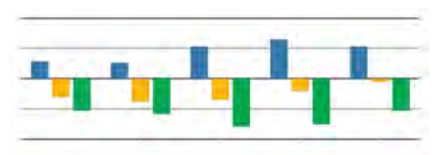

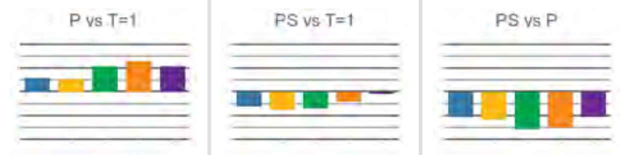

Locus		5m	30m	120m	240m	360m
**PGN_1531**	P vs T=1	-0.270	0.468	1.098	2.469	3.531
	PS vs T=1	0.273	-1.478	0.017	0.046	1.661
	PS vs P	0.029	-0.924	-0.512	-0.572	-0.202

hypothetical protein

hypothetical proteins

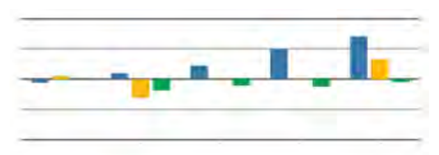

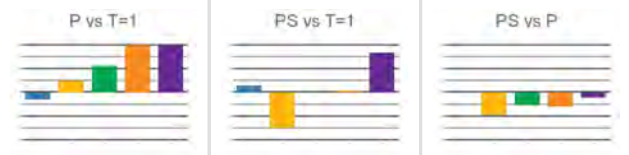

Locus		5m	30m	120m	240m	360m
**PGN_1532**	P vs T=1	0.198	0.260	1.621	1.494	2.996
	PS vs T=1	-0.356	-0.266	-0.014	1.184	1.753
	PS vs P	-0.532	-0.499	-0.768	0.061	-0.178

conserved hypothetical protein

hypothetical proteins-Conserved

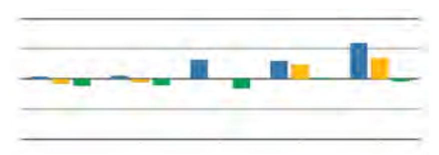

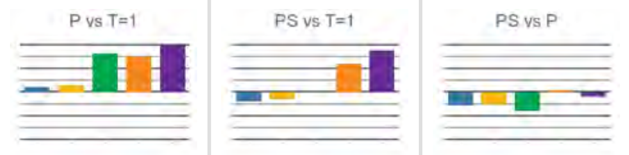

Locus		5m	30m	120m	240m	360m
**PGN_1533**	P vs T=1	1.869	2.385	2.881	2.919	2.220
	PS vs T=1	1.566	1.949	1.901	1.624	1.514
	PS vs P	-0.300	-0.390	-0.906	-1.217	-0.687

putative carbonic anhydrase

central intermediary metabolism

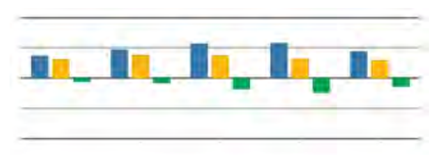

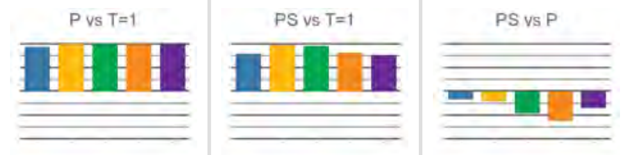

log2 (Fold Change)

Legend: P vs T=1 · PS vs T=1 · PS vs P

Locus		5m	30m	120m	240m	360m
**PGN_1534**	P vs T=1	0.006	-0.067	-0.289	-0.121	0.912
	PS vs T=1	1.410	2.041	1.889	1.448	1.178
	PS vs P	1.254	1.918	1.938	1.349	0.322
	hypothetical protein					
	hypothetical proteins					
**PGN_1535**	P vs T=1	-0.425	-1.023	-1.211	-0.990	-0.323
	PS vs T=1	1.014	1.394	1.199	0.723	0.549
	PS vs P	1.321	2.113	2.099	1.457	0.839
	hypothetical protein					
	hypothetical proteins					
**PGN_1536**	P vs T=1	-0.315	-1.627	-2.210	-2.132	-2.321
	PS vs T=1	-0.213	-0.978	-1.412	-1.877	-1.961
	PS vs P	0.178	0.596	0.712	0.195	0.302
	hypothetical protein					
	hypothetical proteins					
**PGN_1537**	P vs T=1	0.298	-0.151	-0.935	-1.256	-1.317
	PS vs T=1	0.751	-0.054	-0.981	-1.385	-1.481
	PS vs P	0.474	0.105	-0.071	-0.184	-0.196
	probable cation efflux system protein					
	transport and binding proteins					
**PGN_1538**	P vs T=1	-0.081	-0.228	-0.959	-0.998	-0.418
	PS vs T=1	0.811	0.494	-0.985	-1.255	-1.255
	PS vs P	0.892	0.720	-0.057	-0.290	-0.828
	putative cation efflux system					
	transport and binding proteins					
**PGN_1539**	P vs T=1	-0.812	-1.053	-2.006	-2.094	-1.374
	PS vs T=1	0.092	0.153	-1.809	-2.447	-2.407
	PS vs P	0.905	1.195	0.108	-0.453	-1.015
	putative ABC transport system exported protein					
	transport and binding proteins					
**PGN_1540**	P vs T=1	-1.078	-1.237	-1.609	-1.320	-0.391
	PS vs T=1	-0.024	-0.235	-1.379	-1.731	-1.661
	PS vs P	1.032	0.971	0.163	-0.439	-1.228
	putative ABC transport membrane protein					
	transport and binding proteins					

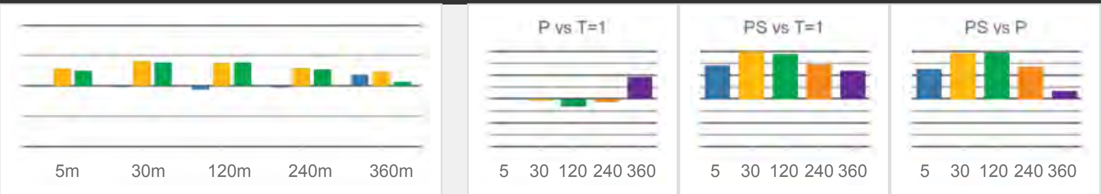

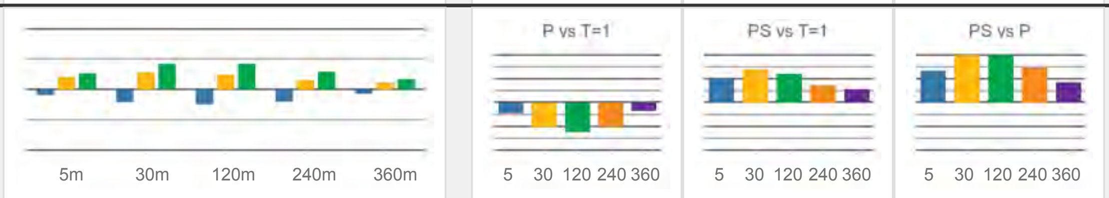

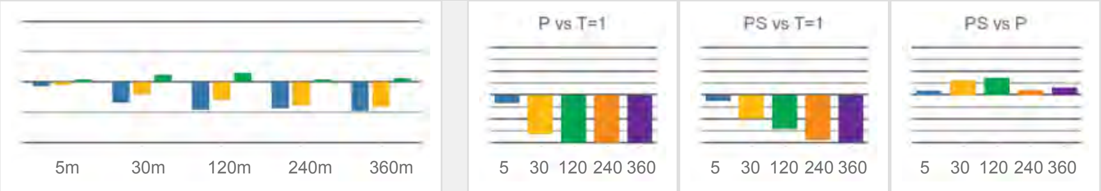

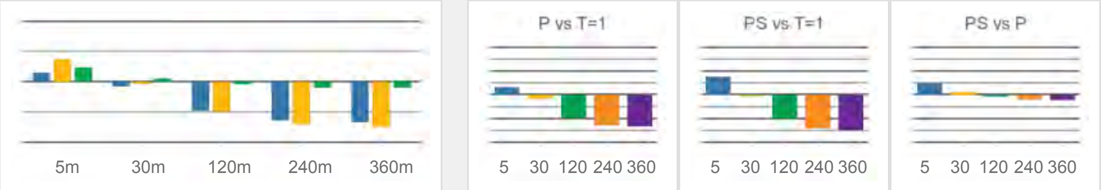

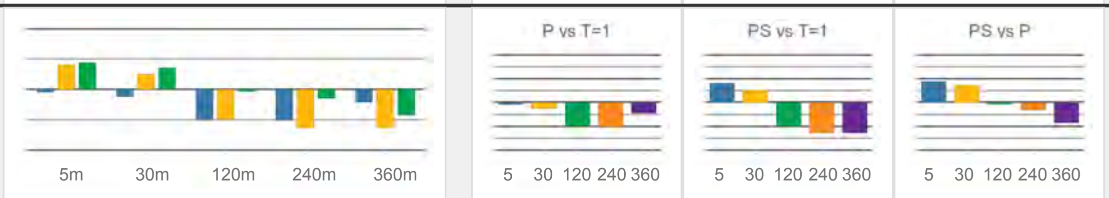

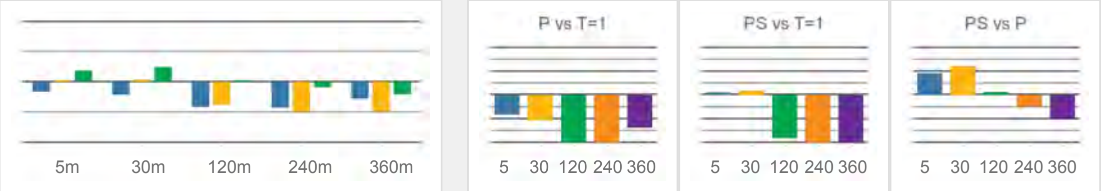

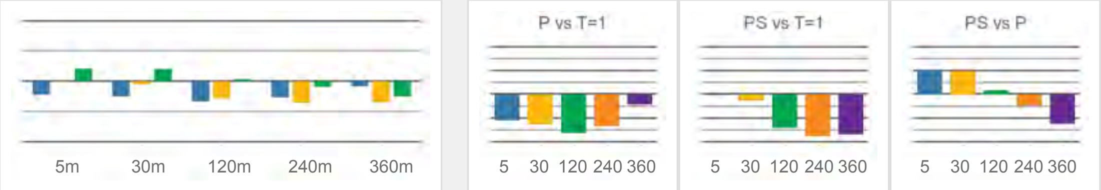

PgSg Time Course

log$_2$ (Fold Change)

Legend: P vs T=1 | PS vs T=1 | PS vs P

Locus		5m	30m	120m	240m	360m
PGN_1541	P vs T=1	0.203	0.408	0.514	0.266	0.913
	PS vs T=1	1.178	2.019	1.239	-0.785	-0.039
	PS vs P	0.811	1.476	0.722	-1.002	-0.673
	hypothetical protein					
	hypothetical proteins					
PGN_1542	P vs T=1	-0.309	-0.525	-0.540	-0.204	0.639
	PS vs T=1	0.264	0.619	0.590	0.773	0.671
	PS vs P	0.533	1.058	0.982	0.869	0.066
	putative esterase					
	hypothetical proteins-Conserved					
PGN_1543	P vs T=1	-1.266	-1.432	-1.710	-1.555	-1.087
	PS vs T=1	-1.170	-1.471	-1.243	-1.109	-1.096
	PS vs P	0.082	-0.068	0.368	0.362	-0.006
	conserved hypothetical protein					
	hypothetical proteins-Conserved					
PGN_1544	P vs T=1	-0.969	-1.070	-2.120	-2.357	-2.689
	PS vs T=1	-1.517	-1.496	-1.887	-2.040	-2.055
	PS vs P	-0.479	-0.354	0.162	0.215	0.518
	conserved hypothetical protein					
	hypothetical proteins-Conserved					
PGN_1545	P vs T=1	0.519	-0.110	0.910	1.662	3.273
	PS vs T=1	-1.150	-0.368	0.739	2.099	1.263
	PS vs P	-1.135	-0.501	-0.056	0.776	-0.724
	hypothetical protein					
	hypothetical proteins					
PGN_1546	P vs T=1	2.994	3.277	3.782	3.899	3.926
	PS vs T=1	1.476	3.442	4.529	4.300	4.106
	PS vs P	-1.376	0.213	0.855	0.547	0.307
	hypothetical protein					
	hypothetical proteins					
PGN_1547	P vs T=1	1.488	2.000	2.668	2.732	2.302
	PS vs T=1	0.054	1.695	3.276	3.756	3.440
	PS vs P	-1.437	-0.289	0.646	1.050	1.133
	conserved hypothetical protein					
	hypothetical proteins-Conserved					

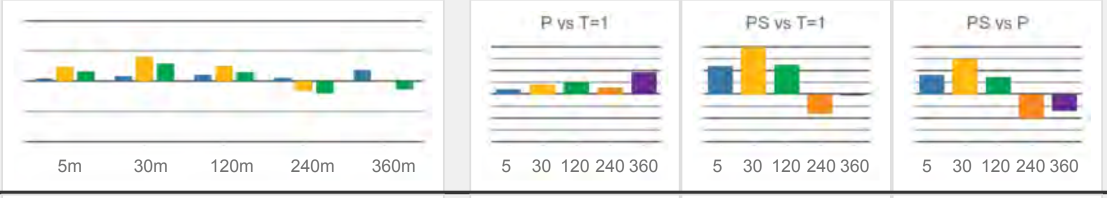

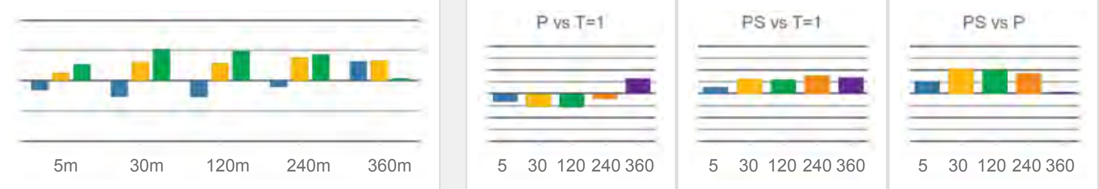

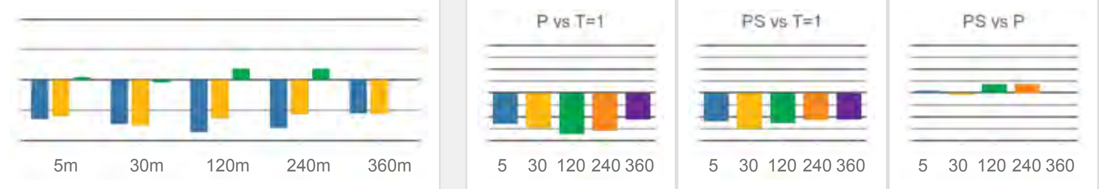

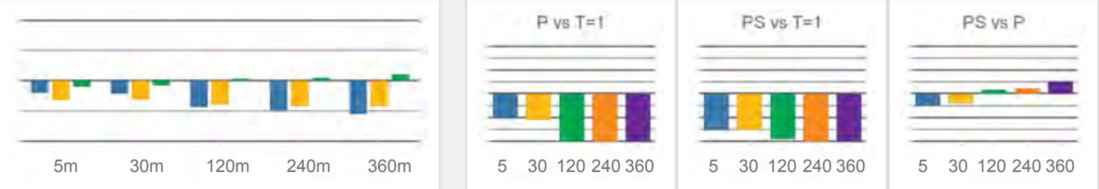

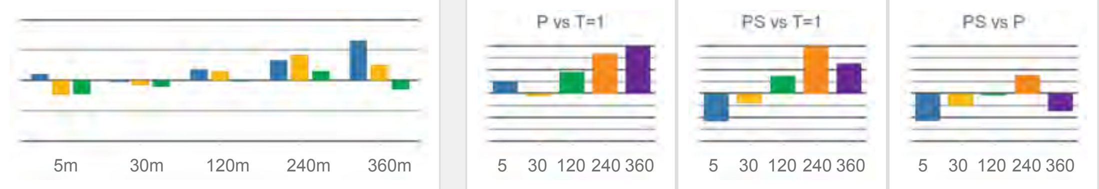

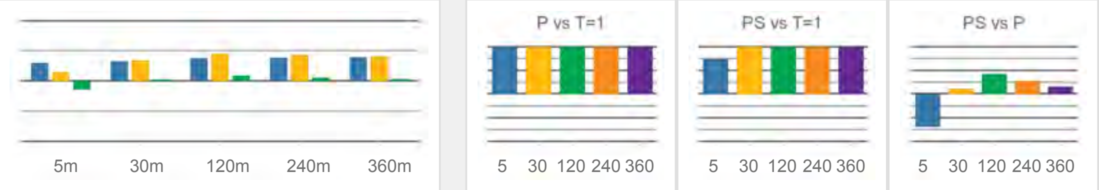

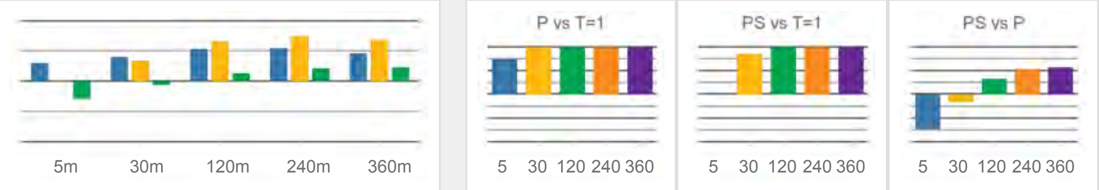

PgSg Time Course

log$_2$ (Fold Change)

Legend: P vs T=1 | PS vs T=1 | PS vs P

Locus		5m	30m	120m	240m	360m
**PGN_1548**	P vs T=1	1.293	1.632	2.276	2.803	2.567
	PS vs T=1	0.770	1.692	2.483	2.569	2.745
	PS vs P	-0.552	0.049	0.234	-0.162	0.207
	conserved hypothetical protein					
	hypothetical proteins-Conserved					
**PGN_1549** *clpP*	P vs T=1	0.239	-0.392	-1.479	-2.106	-2.387
	PS vs T=1	0.302	-0.281	-1.140	-1.580	-1.835
	PS vs P	0.127	0.138	0.307	0.430	0.476
	putative ATP-dependent Clp protease proteolytic subunit					
	protein fate					
**PGN_1550** *clpX*	P vs T=1	0.771	0.305	-0.744	-1.400	-1.806
	PS vs T=1	0.809	0.213	-1.048	-1.652	-1.790
	PS vs P	0.095	-0.055	-0.311	-0.299	-0.032
	ATP-dependent Clp protease ATP-binding subunit ClpX					
	protein fate					
**PGN_1551** *recQl*	P vs T=1	0.392	0.187	-1.083	-1.553	-1.806
	PS vs T=1	0.383	0.184	-1.334	-1.974	-2.163
	PS vs P	0.021	0.025	-0.264	-0.449	-0.381
	ATP-dependent DNA helicase RecQ					
	DNA metabolism					
**PGN_1552**	P vs T=1	-0.007	-0.228	-1.539	-2.403	-2.542
	PS vs T=1	-0.124	-0.245	-1.839	-2.804	-2.832
	PS vs P	-0.070	0.027	-0.310	-0.478	-0.339
	putative peptidyl-prolyl cis-trans isomerase					
	protein fate					
**PGN_1553**	P vs T=1	-0.402	-0.612	-1.068	-1.218	-1.163
	PS vs T=1	-0.611	-1.068	-1.946	-2.030	-1.809
	PS vs P	-0.192	-0.441	-0.877	-0.815	-0.645
	conserved hypothetical protein					
	hypothetical proteins-Conserved					
**PGN_1554**	P vs T=1	0.007	0.307	0.584	0.343	0.234
	PS vs T=1	0.264	-0.152	-0.396	-1.234	-0.839
	PS vs P	0.247	-0.418	-0.876	-1.447	-1.005
	conserved hypothetical protein					
	hypothetical proteins-Conserved					

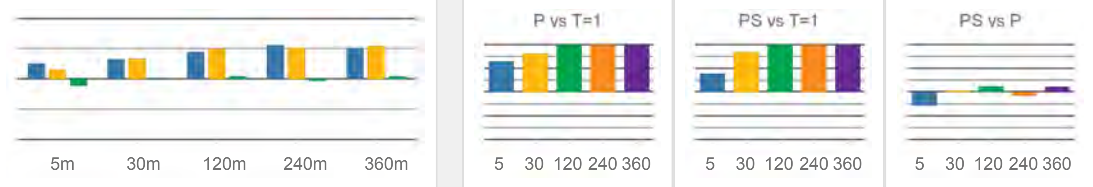

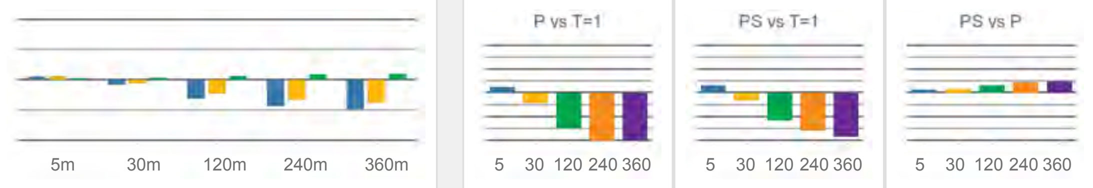

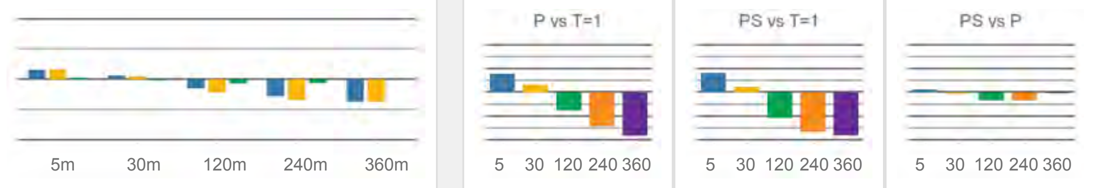

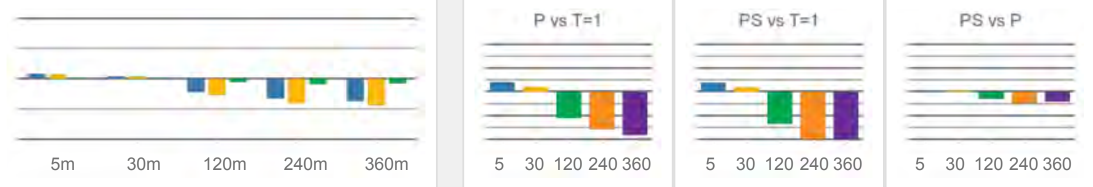

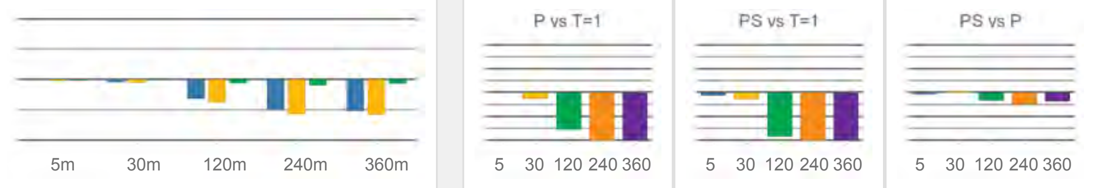

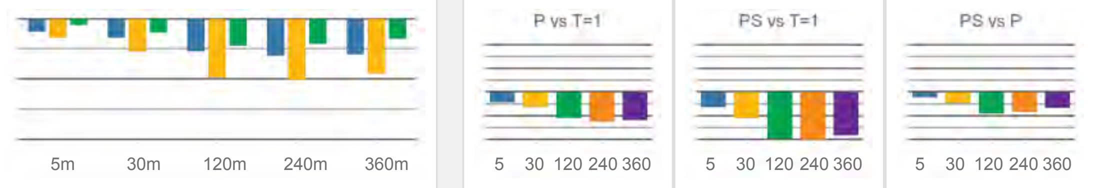

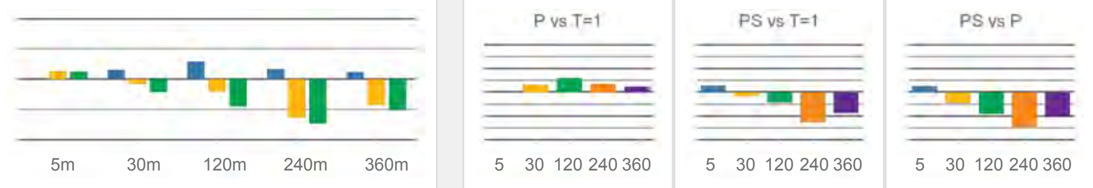

PgSg Time Course

log$_2$ (Fold Change)

P vs T=1 | PS vs T=1 | PS vs P

Locus		5m	30m	120m	240m	360m
**PGN_1555**	P vs T=1	-0.559	-0.725	-1.343	-1.267	-0.646
	PS vs T=1	-0.465	-1.156	-2.055	-2.058	-2.039
	PS vs P	0.117	-0.407	-0.740	-0.799	-1.346

DNA mismatch repair protein MutL

DNA metabolism

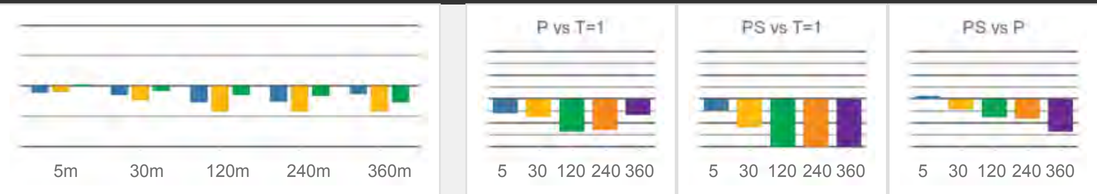

Locus		5m	30m	120m	240m	360m
**PGN_1556**	P vs T=1	0.343	-0.014	-0.597	-1.193	-1.780
	PS vs T=1	0.383	0.229	-0.291	-0.821	-0.969
	PS vs P	0.064	0.248	0.294	0.333	0.764

putative hemagglutinin

cellular processes

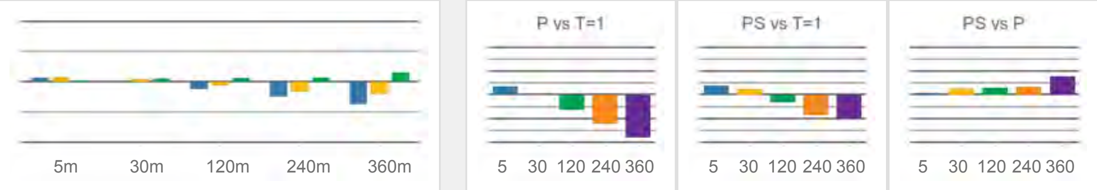

Locus		5m	30m	120m	240m	360m
**PGN_1557**	P vs T=1	0.713	1.054	1.561	1.428	0.460
	PS vs T=1	0.514	1.059	1.361	1.151	1.126
	PS vs P	-0.215	0.012	-0.157	-0.247	0.633

conserved hypothetical protein

hypothetical proteins-Conserved

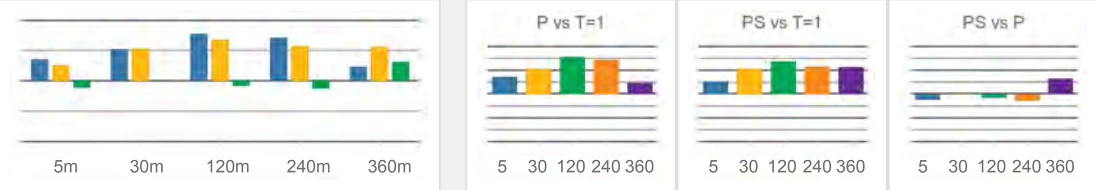

Locus		5m	30m	120m	240m	360m
**PGN_1558**	P vs T=1	0.281	0.440	0.471	0.164	-0.744
	PS vs T=1	-0.044	0.401	0.790	0.613	0.364
	PS vs P	-0.323	-0.029	0.332	0.439	1.045

conserved hypothetical protein

hypothetical proteins-Conserved

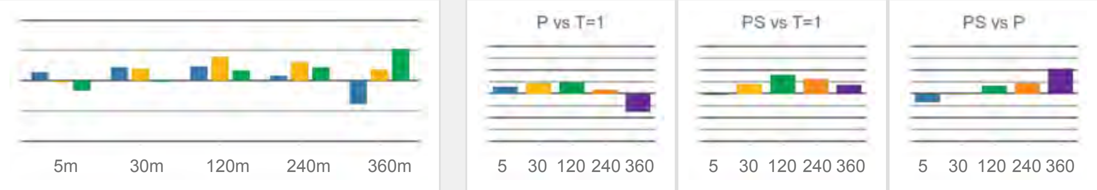

Locus		5m	30m	120m	240m	360m
**PGN_1559**	P vs T=1	-0.415	-1.492	-0.840	-0.204	1.395
	PS vs T=1	-0.377	-1.594	-0.932	0.204	0.514
	PS vs P	0.078	-0.652	-0.393	0.312	-0.238

hypothetical protein

hypothetical proteins

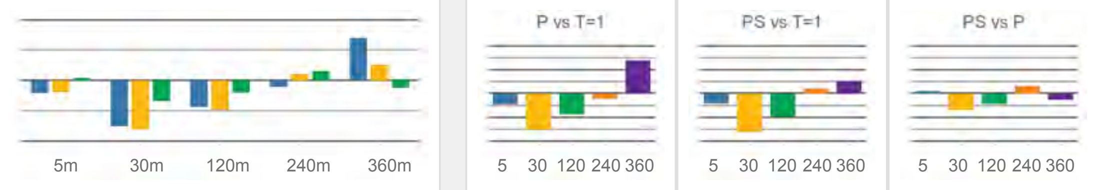

Locus		5m	30m	120m	240m	360m
**PGN_1560**	P vs T=1	-1.703	-1.564	-0.858	-0.732	-0.307
	PS vs T=1	-2.058	-1.904	-0.907	0.517	0.544
	PS vs P	-0.548	-0.507	-0.048	1.152	0.894

conserved hypothetical protein

hypothetical proteins-Conserved

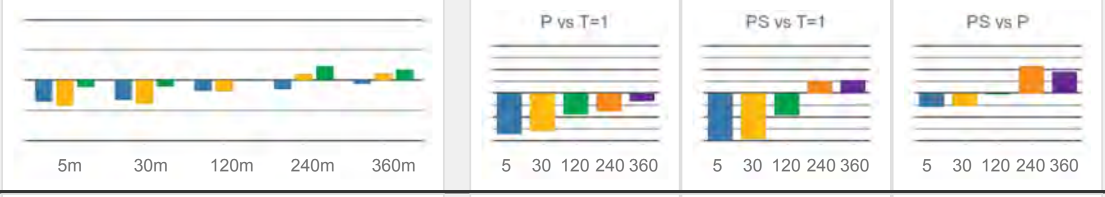

Locus		5m	30m	120m	240m	360m
**PGN_1561**	P vs T=1	0.353	-0.229	-0.506	-0.690	-1.126
	PS vs T=1	0.013	0.167	0.336	0.563	0.479
	PS vs P	-0.286	0.342	0.755	1.118	1.447

conserved hypothetical protein

hypothetical proteins-Conserved

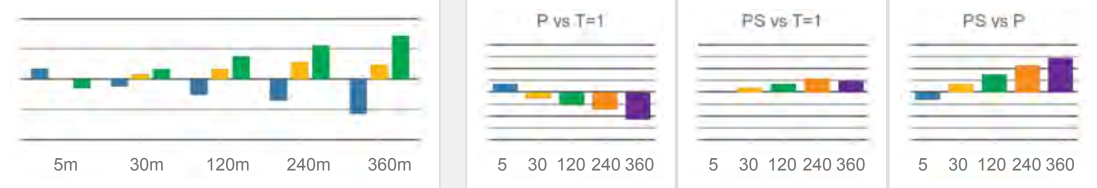

log2 (Fold Change)

Locus		5m	30m	120m	240m	360m
**PGN_1562**	P vs T=1	-0.378	-0.611	-0.970	-1.306	-1.462
	PS vs T=1	-0.643	-0.789	-0.607	-0.686	-0.708
	PS vs P	-0.250	-0.179	0.320	0.490	0.653
	conserved hypothetical protein					
	hypothetical proteins-Conserved					
**PGN_1563**	P vs T=1	0.352	0.269	0.432	0.348	0.174
	PS vs T=1	0.090	0.338	0.093	-0.071	0.160
	PS vs P	-0.243	0.060	-0.294	-0.384	-0.040
	hypothetical protein					
	hypothetical proteins					
**PGN_1564**	P vs T=1	0.487	0.649	0.842	0.561	0.148
	PS vs T=1	0.649	0.526	0.112	0.041	-0.076
	PS vs P	0.164	-0.103	-0.685	-0.500	-0.233
	conserved hypothetical protein					
	unknown function					
**PGN_1565**	P vs T=1	0.764	0.929	0.859	0.591	0.068
	PS vs T=1	1.093	0.813	0.121	-0.328	-0.271
	PS vs P	0.339	-0.081	-0.698	-0.892	-0.355
	conserved hypothetical protein with PSP1 C-terminal conserved region					
	hypothetical proteins-Conserved					
**PGN_1566**	P vs T=1	0.256	0.433	0.020	-0.132	-0.409
	PS vs T=1	0.845	0.831	-0.048	-0.450	-0.732
	PS vs P	0.583	0.413	-0.077	-0.333	-0.360
	conserved hypothetical protein					
	hypothetical proteins-Conserved					
**PGN_1567**	P vs T=1	-0.290	-0.134	-0.104	-0.206	-0.497
	PS vs T=1	-0.051	0.060	-0.446	-0.686	-0.947
	PS vs P	0.232	0.201	-0.321	-0.458	-0.454
	putative DNA replication and repair protein RecF					
	DNA metabolism					
**PGN_1568**	P vs T=1	-0.185	-0.432	-0.808	-0.368	-0.250
	PS vs T=1	0.194	0.219	-0.104	-0.715	-0.471
	PS vs P	0.374	0.603	0.518	-0.336	-0.207
	conserved hypothetical protein					
	hypothetical proteins-Conserved					

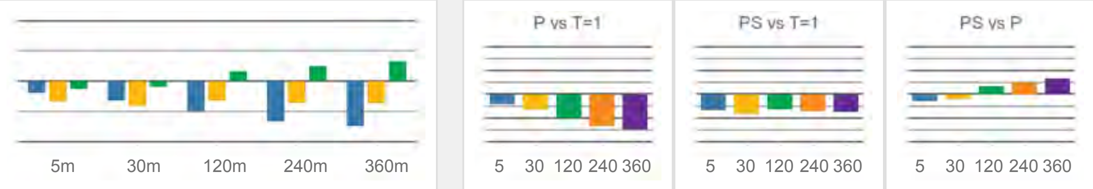

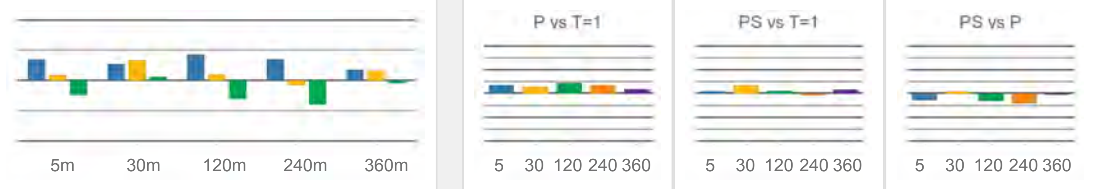

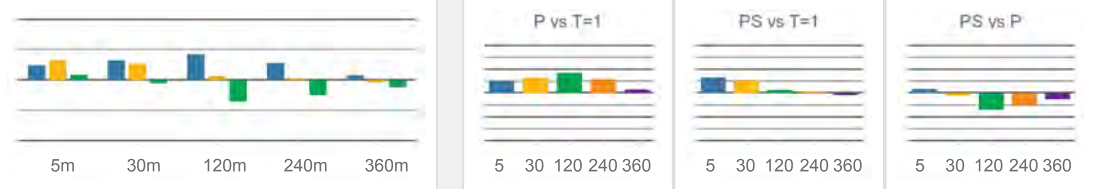

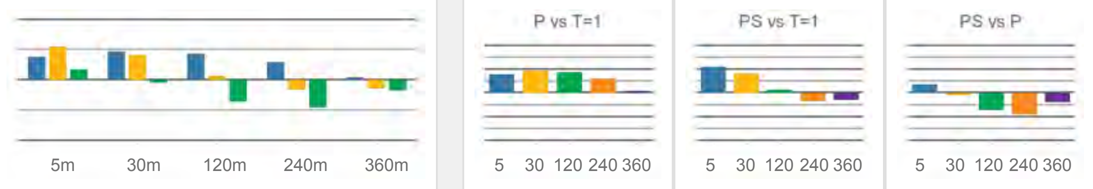

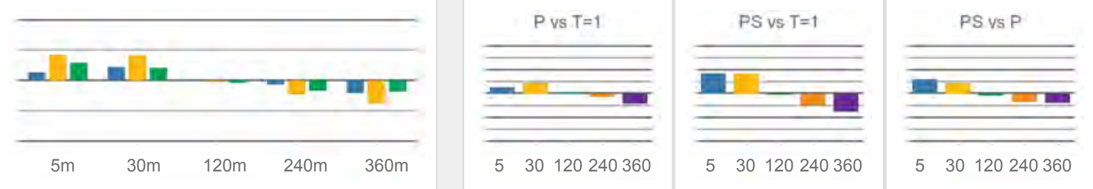

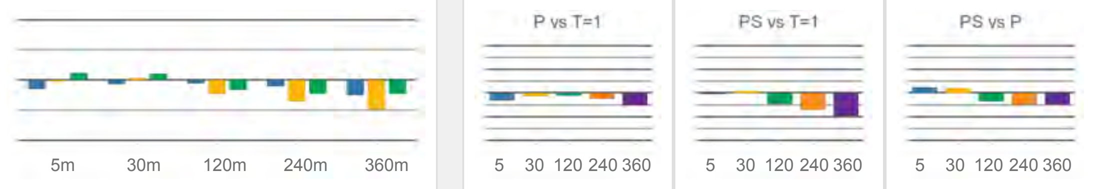

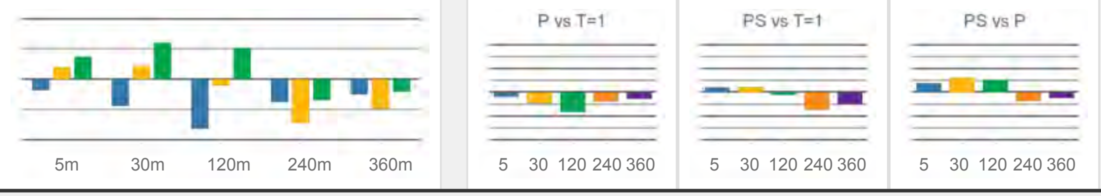

PgSg Time Course

log$_2$ (Fold Change)

Legend: P vs T=1 | PS vs T=1 | PS vs P

Locus		5m	30m	120m	240m	360m
**PGN_1569**	P vs T=1	-0.072	-0.087	-0.430	-0.504	-0.552
	PS vs T=1	0.075	0.686	0.797	0.333	0.257
	PS vs P	0.125	0.741	1.151	0.742	0.744
	probable transcriptional regulator Crp/Fnr family					
	regulatory functions					
**PGN_1570** *rpoC*	P vs T=1	0.524	0.508	-0.270	-0.969	-1.664
	PS vs T=1	0.731	0.686	-0.459	-1.558	-1.725
	PS vs P	0.233	0.207	-0.184	-0.608	-0.094
	DNA-directed RNA polymerase beta' subunit					
	transcription					
**PGN_1571** *rpoB*	P vs T=1	0.712	0.523	-0.909	-1.782	-2.113
	PS vs T=1	0.909	0.807	-0.913	-2.255	-2.304
	PS vs P	0.252	0.335	-0.019	-0.525	-0.233
	DNA-directed RNA polymerase beta subunit					
	transcription					
**PGN_1572** *rplL*	P vs T=1	0.239	0.707	1.236	1.072	0.141
	PS vs T=1	0.107	0.670	0.982	0.838	0.816
	PS vs P	-0.193	-0.000	-0.123	-0.145	0.599
	putative 50S ribosomal protein L7/L12					
	protein synthesis					
**PGN_1573** *rplJ*	P vs T=1	0.620	0.989	1.336	0.925	-0.129
	PS vs T=1	0.321	0.898	1.000	0.542	0.264
	PS vs P	-0.291	-0.030	-0.221	-0.328	0.324
	putative 50S ribosomal protein L10					
	protein synthesis					
**PGN_1574** *rplA*	P vs T=1	0.756	1.088	0.963	0.094	-1.161
	PS vs T=1	0.493	0.939	0.863	0.254	-0.168
	PS vs P	-0.222	-0.049	-0.005	0.122	0.834
	50S ribosomal protein L1					
	protein synthesis					
**PGN_1575** *rplK*	P vs T=1	1.070	1.198	0.804	-0.099	-1.178
	PS vs T=1	0.786	1.184	1.032	0.440	0.124
	PS vs P	-0.199	0.095	0.290	0.441	1.096
	50S ribosomal protein L11					
	protein synthesis					

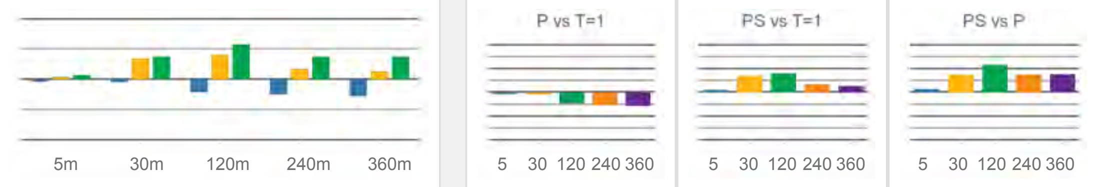

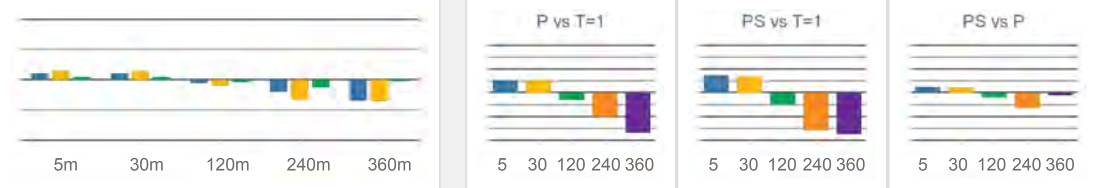

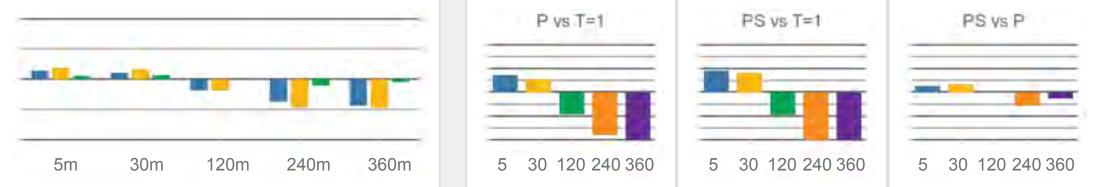

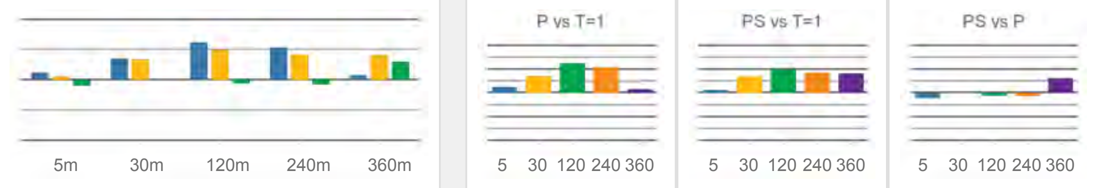

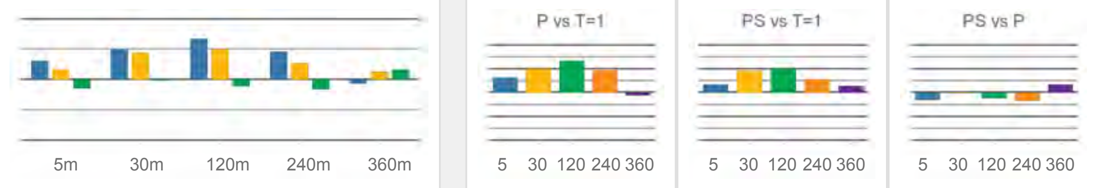

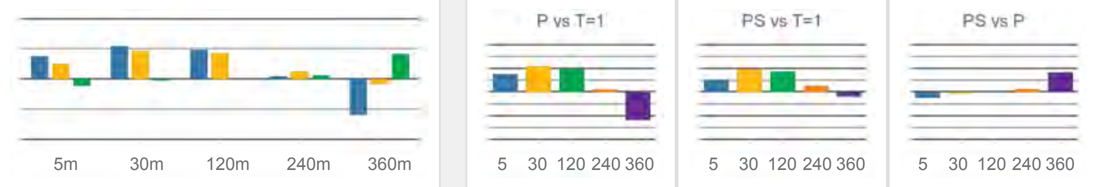

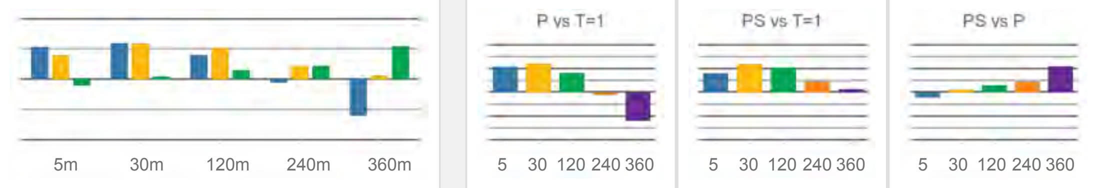

PgSg Time Course

log$_2$ (Fold Change)

Legend: P vs T=1 | PS vs T=1 | PS vs P

Locus		5m	30m	120m	240m	360m
**PGN_1576**	P vs T=1	0.766	0.632	-0.165	-1.141	-2.111
	PS vs T=1	0.440	0.665	0.052	-0.664	-1.100
	PS vs P	-0.201	0.139	0.236	0.353	0.820

putative transcription antitermination protein

transcription

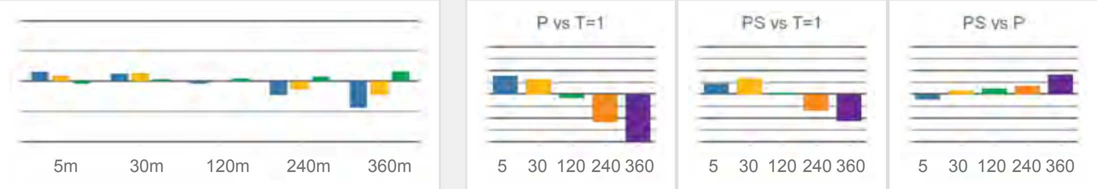

Locus		5m	30m	120m	240m	360m
**PGN_1577**	P vs T=1	0.471	0.247	-0.672	-1.912	-2.276
	PS vs T=1	-0.362	-0.204	-0.799	-1.611	-1.665
	PS vs P	-0.613	-0.271	-0.085	0.030	0.379

putative preprotein translocase SecE subunit

protein fate

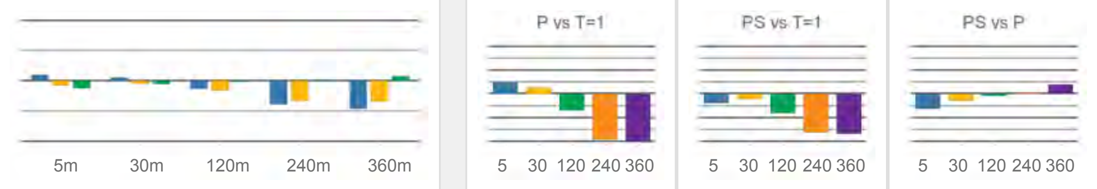

Locus		5m	30m	120m	240m	360m
**PGN_1578**	P vs T=1	1.469	1.872	2.106	1.827	1.087
	PS vs T=1	0.957	1.242	1.055	0.708	0.667
	PS vs P	-0.493	-0.580	-0.992	-1.078	-0.424

translation elongation factor Tu

protein synthesis

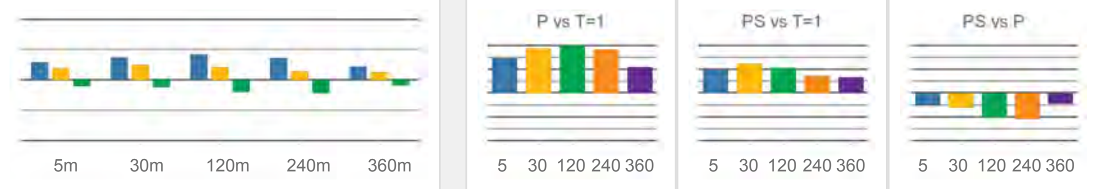

Locus		5m	30m	120m	240m	360m
**PGN_1579**	P vs T=1	0.543	0.766	0.892	0.366	-0.667
	PS vs T=1	0.629	1.103	1.276	0.570	0.154
	PS vs P	0.070	0.346	0.423	0.184	0.721

putative site-specific recombinase

protein synthesis

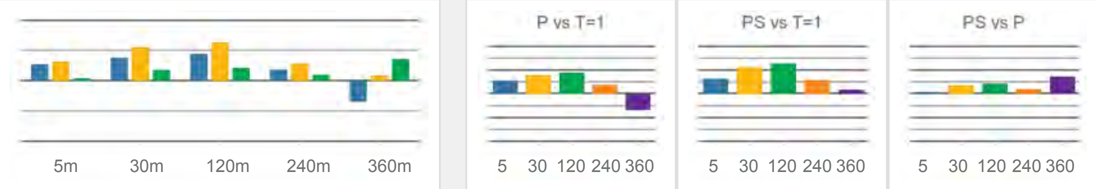

Locus		5m	30m	120m	240m	360m
**PGN_1580** *rpsU*	P vs T=1	1.258	1.028	1.184	1.313	0.640
	PS vs T=1	0.015	0.365	1.124	1.740	1.692
	PS vs P	-1.108	-0.600	-0.010	0.487	1.006

putative 30S ribosomal protein S21

protein synthesis

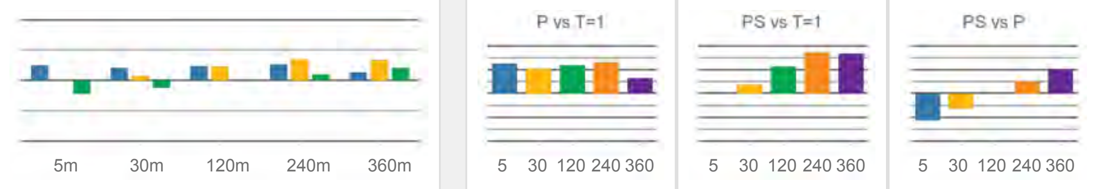

Locus		5m	30m	120m	240m	360m
**PGN_1581**	P vs T=1	-0.072	0.176	0.324	0.442	0.709
	PS vs T=1	0.350	0.528	1.736	1.692	1.406
	PS vs P	0.394	0.335	1.392	1.228	0.696

DNA mismatch repair protein MutS

DNA metabolism

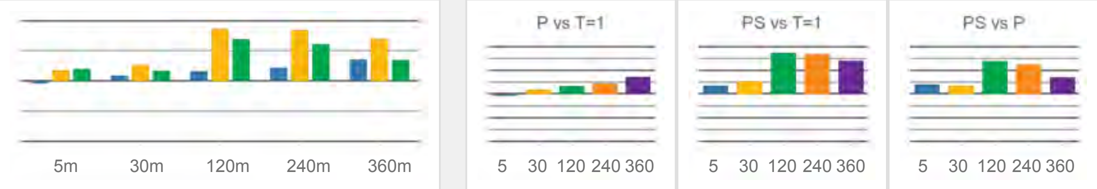

Locus		5m	30m	120m	240m	360m
**PGN_1582**	P vs T=1	-0.508	-0.126	0.231	0.517	0.565
	PS vs T=1	-0.523	0.089	1.240	1.385	0.966
	PS vs P	-0.089	0.179	0.987	0.875	0.408

membrane-associated zinc metalloprotease

protein fate

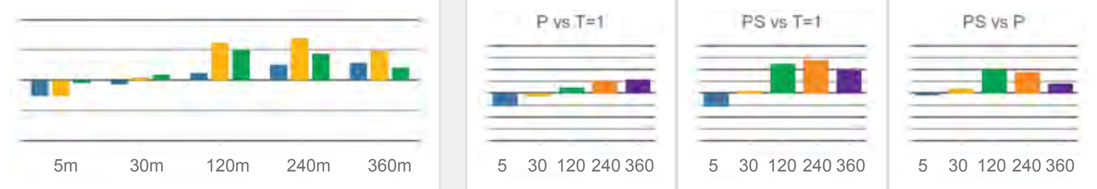

PgSg Time Course

Locus		log_2 (Fold Change) 5m	30m	120m	240m	360m
**PGN_1583**	P vs T=1	-0.009	-0.359	0.675	0.854	2.301
	PS vs T=1	-0.118	0.361	1.048	1.200	0.999
	PS vs P	-0.261	0.125	0.411	0.342	-0.327
	hypothetical protein					
	hypothetical proteins					
**PGN_1584**	P vs T=1	0.097	0.014	0.262	0.562	1.121
	PS vs T=1	-0.093	-0.141	0.710	1.077	1.128
	PS vs P	-0.200	-0.181	0.422	0.516	0.049
	putative sodium/hydrogen antiporter					
	transport and binding proteins					
**PGN_1585** *uvrB*	P vs T=1	-0.306	0.003	0.341	0.650	0.908
	PS vs T=1	-0.110	0.250	0.994	1.666	1.701
	PS vs P	0.154	0.222	0.632	1.009	0.797
	excinuclease ABC B subunit					
	DNA metabolism					
**PGN_1586**	P vs T=1	-0.665	-0.106	0.688	1.036	2.453
	PS vs T=1	-0.440	-0.539	-0.156	0.913	-0.030
	PS vs P	-0.146	-0.418	-0.483	0.238	-1.286
	hypothetical protein					
	hypothetical proteins					
**PGN_1587**	P vs T=1	0.265	0.759	1.273	1.135	0.599
	PS vs T=1	-0.071	0.277	0.432	0.307	0.196
	PS vs P	-0.344	-0.452	-0.783	-0.781	-0.396
	putative translation elongation factor Ts					
	protein synthesis					
**PGN_1588** *rpsB*	P vs T=1	1.299	1.831	2.751	3.048	2.515
	PS vs T=1	0.966	1.171	1.741	2.192	2.240
	PS vs P	-0.363	-0.613	-0.874	-0.701	-0.220
	putative 30S ribosomal protein S2					
	protein synthesis					
**PGN_1589** *rpsI*	P vs T=1	0.175	0.554	1.296	1.537	1.100
	PS vs T=1	-0.155	0.045	0.529	1.258	1.469
	PS vs P	-0.374	-0.492	-0.665	-0.161	0.390
	putative 30S ribosomal protein S9					
	protein synthesis					

P vs T=1 | PS vs T=1 | PS vs P

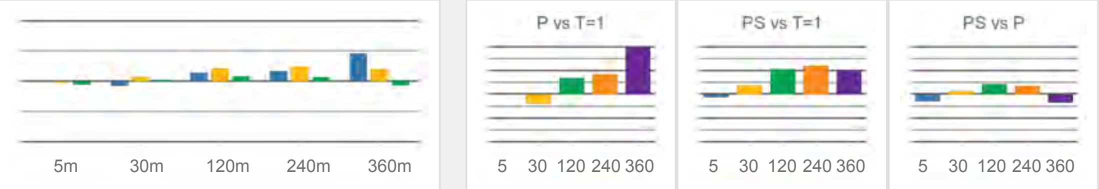

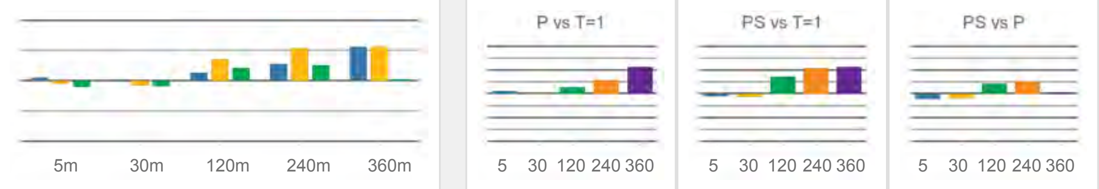

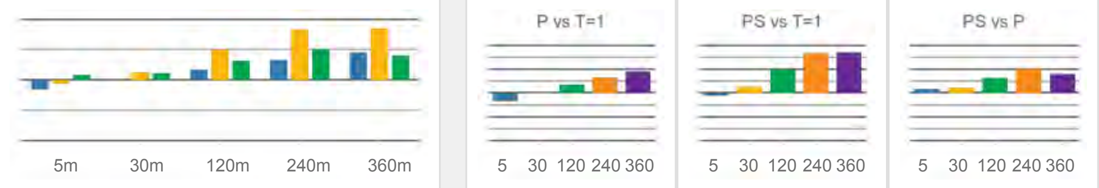

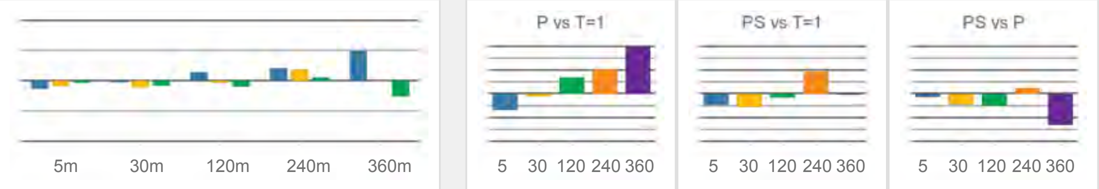

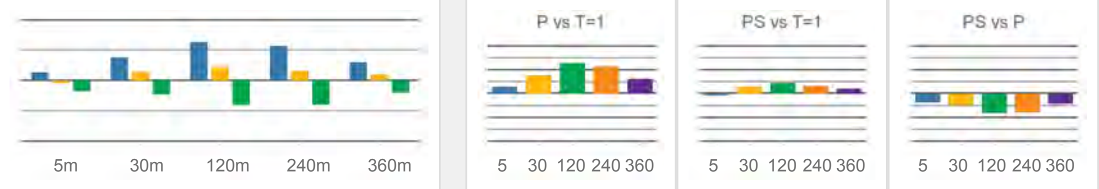

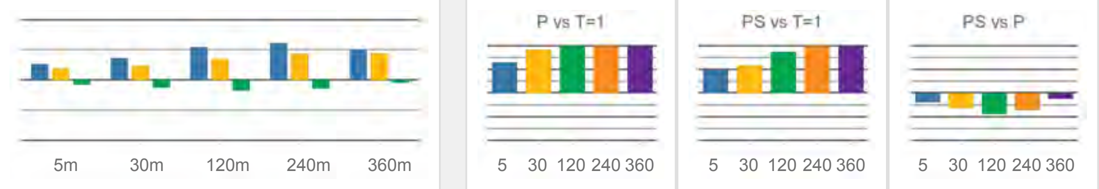

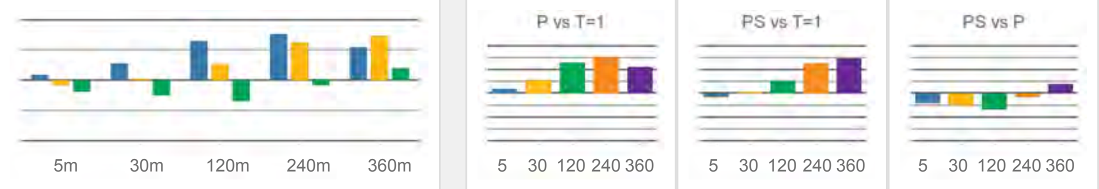

PgSg Time Course

log$_2$ (Fold Change)

Legend: P vs T=1 · PS vs T=1 · PS vs P

Locus		5m	30m	120m	240m	360m
**PGN_1590**	P vs T=1	0.356	0.599	1.106	1.123	0.832
rplM	PS vs T=1	-0.077	-0.108	0.309	0.715	0.784
	PS vs P	-0.424	-0.655	-0.692	-0.317	-0.019

putative 50S ribosomal protein L13

protein synthesis

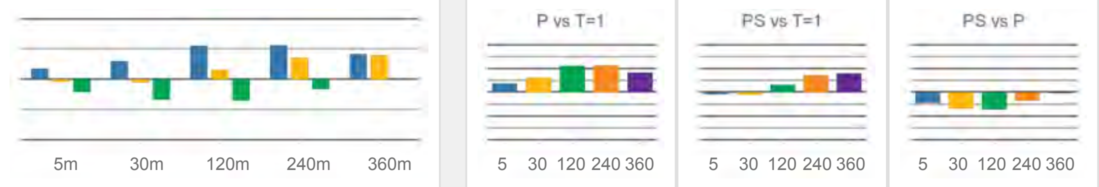

Locus		5m	30m	120m	240m	360m
**PGN_1591**	P vs T=1	-0.615	-0.780	-0.497	-0.261	-0.114
	PS vs T=1	-1.327	-0.704	0.009	0.241	0.370
	PS vs P	-0.707	0.031	0.484	0.507	0.495

conserved hypothetical protein

hypothetical proteins-Conserved

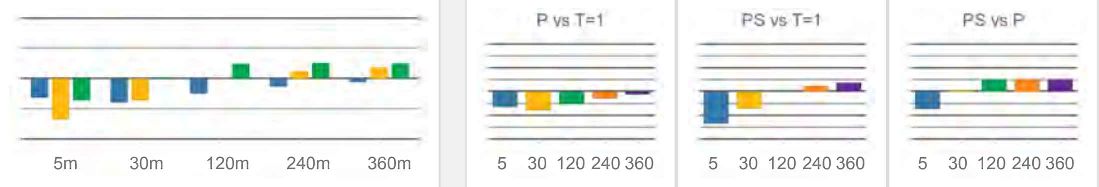

Locus		5m	30m	120m	240m	360m
**PGN_1592**	P vs T=1	-0.353	-0.880	-0.154	0.470	1.352
	PS vs T=1	-1.888	-1.451	-0.937	-0.207	-0.717
	PS vs P	-1.228	-0.656	-0.579	-0.178	-1.179

conserved hypothetical protein

hypothetical proteins-Conserved

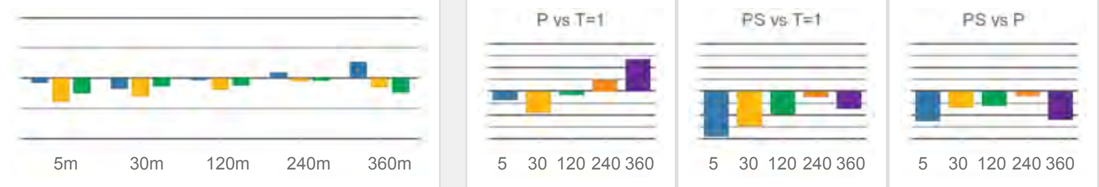

Locus		5m	30m	120m	240m	360m
**PGN_1593**	P vs T=1	0.025	0.639	1.250	1.104	0.984
	PS vs T=1	0.113	0.123	0.175	0.285	0.246
	PS vs P	0.013	-0.467	-0.930	-0.700	-0.667

putative phosphopantetheine adenylyltransferase

cell envelope

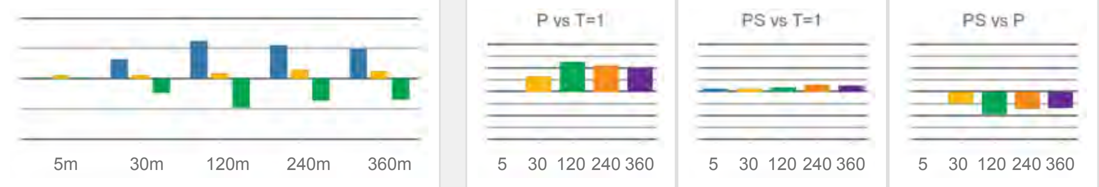

Locus		5m	30m	120m	240m	360m
**PGN_1594**	P vs T=1	0.527	0.545	0.235	0.095	0.278
	PS vs T=1	0.568	0.083	-0.555	-0.489	-0.387
	PS vs P	0.066	-0.425	-0.771	-0.573	-0.641

DNA topoisomerase IV B subunit

DNA metabolism

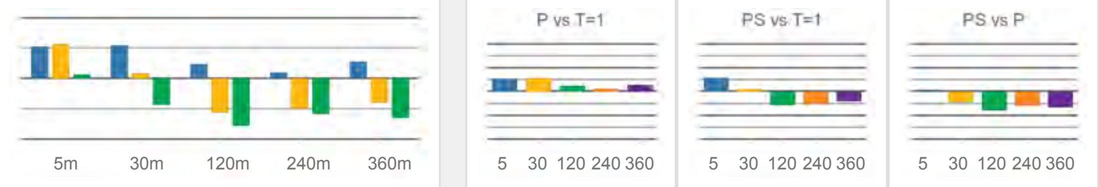

Locus		5m	30m	120m	240m	360m
**PGN_1595**	P vs T=1	-0.496	-0.194	-0.214	-0.148	0.301
	PS vs T=1	-0.015	-0.307	0.144	0.627	0.627
	PS vs P	0.405	-0.123	0.309	0.706	0.349

conserved hypothetical protein

hypothetical proteins-Conserved

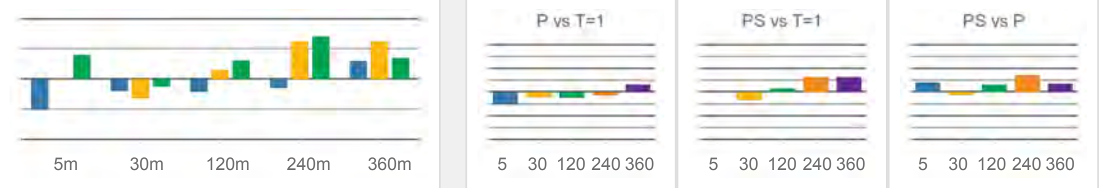

Locus		5m	30m	120m	240m	360m
**PGN_1596**	P vs T=1	-0.227	-0.181	0.388	0.994	1.753
	PS vs T=1	-0.017	-0.305	0.022	0.637	0.871
	PS vs P	0.135	-0.200	-0.356	-0.201	-0.695

probable 3'-5' exonuclease

unknown function

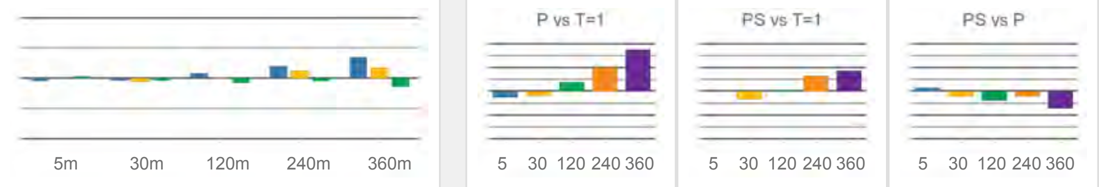

PgSg Time Course

log2 (Fold Change)

Legend: P vs T=1 | PS vs T=1 | PS vs P

Locus		5m	30m	120m	240m	360m
PGN_1597	P vs T=1	-0.739	-0.706	-0.492	-0.342	0.057
	PS vs T=1	-0.709	-1.128	-1.264	-0.929	-0.568
	PS vs P	0.024	-0.419	-0.743	-0.536	-0.566

conserved hypothetical protein

hypothetical proteins-Conserved

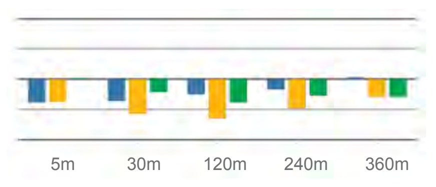

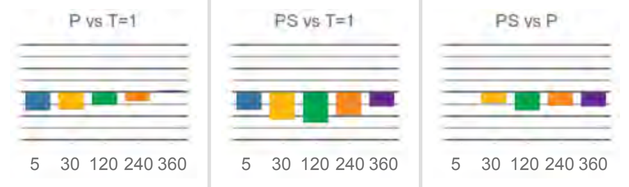

Locus		5m	30m	120m	240m	360m
PGN_1598	P vs T=1	-2.463	-2.174	-1.880	-1.892	-1.832
	PS vs T=1	-1.402	-1.321	-1.468	-1.578	-1.341
	PS vs P	0.952	0.778	0.373	0.255	0.451

probable glutamine ABC transporter

transport and binding proteins

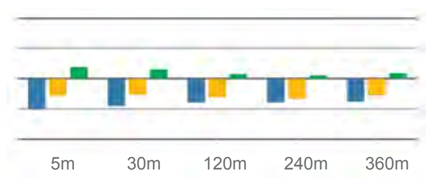

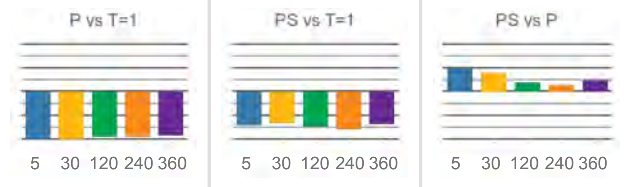

Locus		5m	30m	120m	240m	360m
PGN_1599	P vs T=1	-0.585	-0.781	-0.789	-0.940	-0.560
	PS vs T=1	0.220	0.043	-0.326	-0.989	-1.176
	PS vs P	0.791	0.798	0.448	-0.080	-0.609

conserved hypothetical protein

hypothetical proteins-Conserved

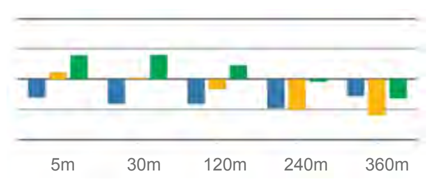

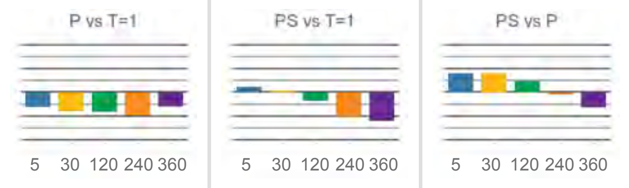

Locus		5m	30m	120m	240m	360m
PGN_1600	P vs T=1	-1.059	-0.760	-0.664	-1.169	-1.716
	PS vs T=1	-0.358	-0.151	-0.524	-0.873	-1.348
	PS vs P	0.679	0.601	0.144	0.272	0.329

conserved hypothetical protein

hypothetical proteins-Conserved

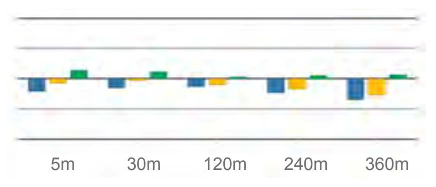

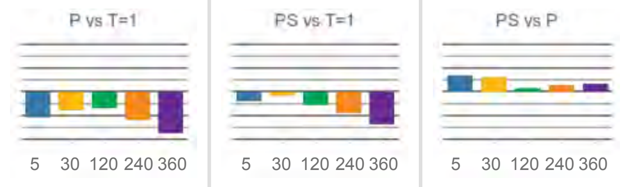

Locus		5m	30m	120m	240m	360m
PGN_1601	P vs T=1	-0.441	0.071	0.017	-0.439	-0.979
	PS vs T=1	0.036	0.235	-0.097	-0.494	-0.742
	PS vs P	0.448	0.178	-0.096	-0.075	0.188

conserved hypothetical protein with lemA family domain

unknown function

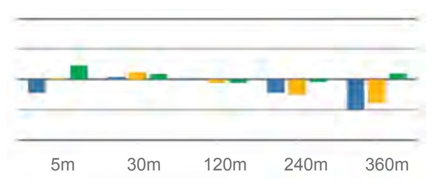

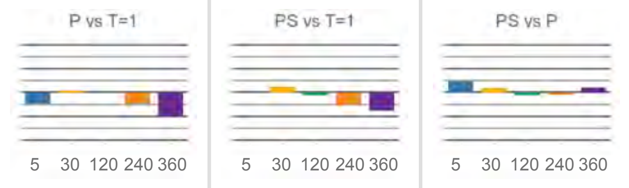

Locus		5m	30m	120m	240m	360m
PGN_1602	P vs T=1	0.090	0.456	0.350	0.315	0.132
	PS vs T=1	0.681	0.722	0.137	0.285	-0.272
	PS vs P	0.560	0.276	-0.207	-0.022	-0.409

putative flavoredoxin

unknown function

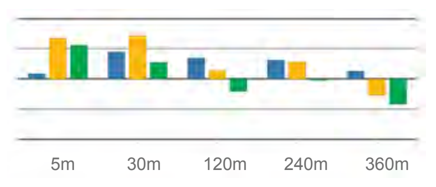

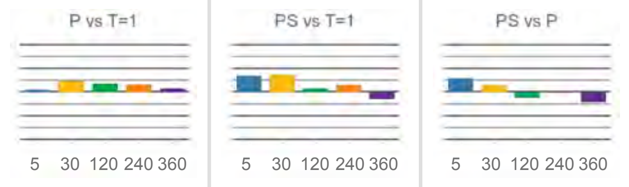

Locus		5m	30m	120m	240m	360m
PGN_1603	P vs T=1	-0.178	0.329	0.127	-0.511	-0.401
	PS vs T=1	0.822	0.839	0.161	-0.051	-0.603
	PS vs P	0.926	0.530	0.057	0.335	-0.238

aspartate carbamoyltransferase regulatory subunit

purines, pyrimidines, nucleosides and nucleotides

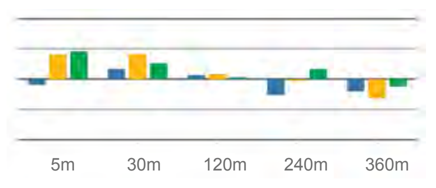

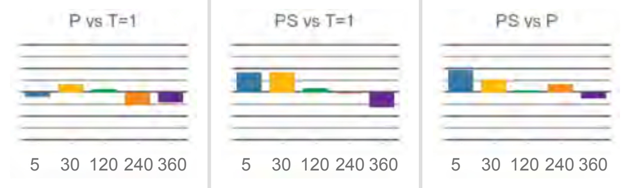

PgSg Time Course

log$_2$ (Fold Change)

Locus		5m	30m	120m	240m	360m
**PGN_1604**	P vs T=1	-0.047	0.260	-0.304	-0.483	-0.726
	PS vs T=1	0.774	0.636	0.013	-0.258	-0.520
	PS vs P	0.807	0.385	0.297	0.193	0.173
	aspartate carbamoyltransferase catalytic subunit					
	purines, pyrimidines, nucleosides and nucleotides					
**PGN_1605**	P vs T=1	0.434	0.541	-0.133	-0.741	-0.920
	PS vs T=1	0.933	0.875	0.524	0.576	0.001
	PS vs P	0.497	0.346	0.622	1.169	0.824
	conserved hypothetical protein					
	hypothetical proteins-Conserved					
**PGN_1606**	P vs T=1	0.361	0.220	-0.411	-0.534	-0.859
	PS vs T=1	0.366	0.200	0.445	0.845	0.683
	PS vs P	0.014	-0.016	0.800	1.300	1.466
	conserved hypothetical protein					
	hypothetical proteins-Conserved					
**PGN_1607**	P vs T=1	-0.027	-0.149	0.172	0.786	2.273
	PS vs T=1	-0.613	-0.338	0.173	0.226	0.849
	PS vs P	-0.565	-0.291	-0.095	-0.302	-0.727
	hypothetical protein					
	hypothetical proteins					
**PGN_1608**	P vs T=1	-0.633	-0.540	-0.033	0.746	1.643
	PS vs T=1	-0.533	-0.536	-0.283	0.199	0.254
	PS vs P	0.040	-0.056	-0.265	-0.418	-1.240
	neuraminidase					
**PGN_1609**	P vs T=1	0.281	-0.585	0.933	1.642	2.402
	PS vs T=1	-0.269	-0.027	0.110	0.332	2.299
	PS vs P	-0.397	-0.181	-0.366	-0.306	0.764
	hypothetical protein					
	hypothetical proteins					
**PGN_1610**	P vs T=1	1.089	1.168	1.447	1.482	0.902
	PS vs T=1	0.020	0.778	1.908	2.258	2.288
	PS vs P	-1.033	-0.370	0.482	0.787	1.314
	conserved hypothetical protein					
	hypothetical proteins-Conserved					

P vs T=1 | PS vs T=1 | PS vs P

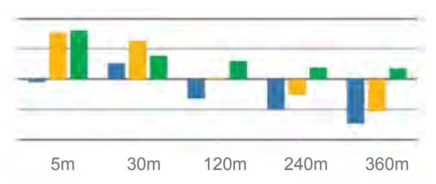

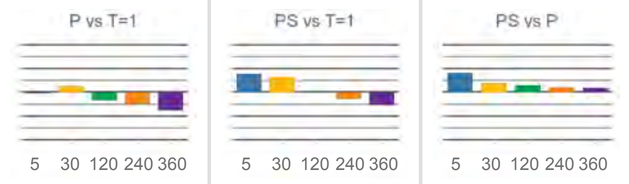

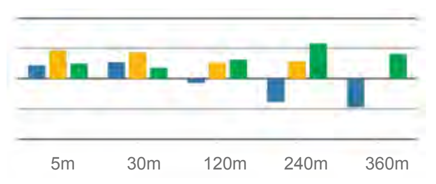

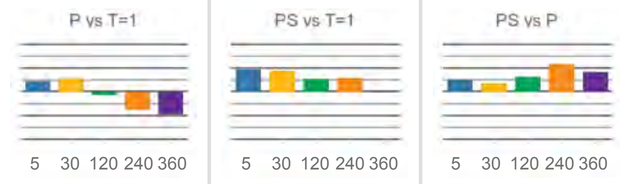

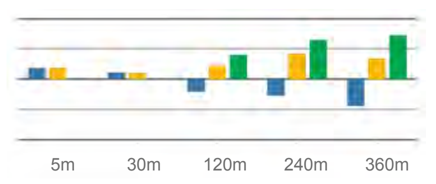

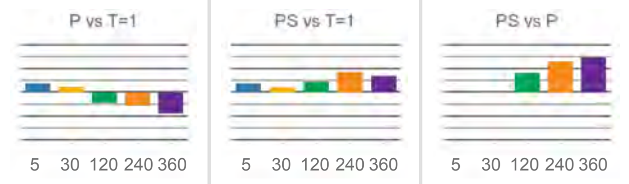

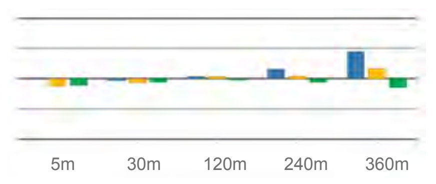

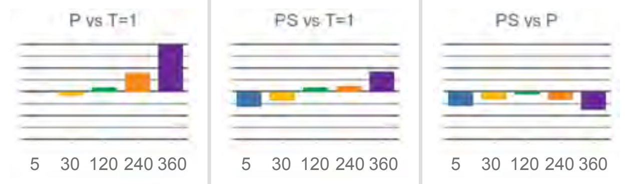

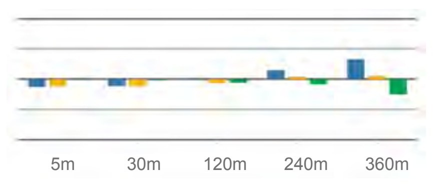

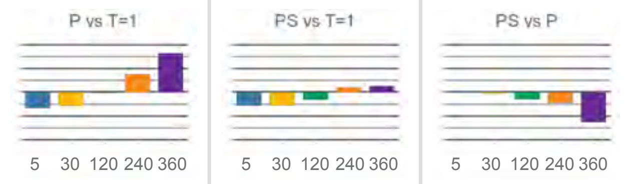

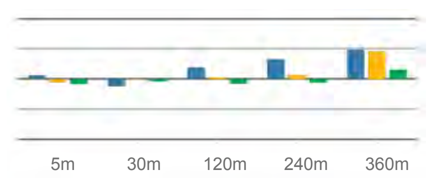

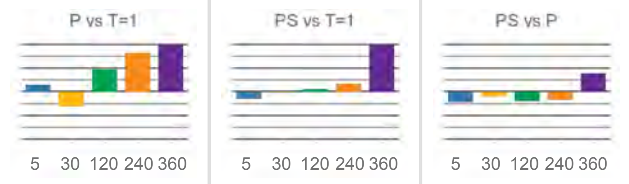

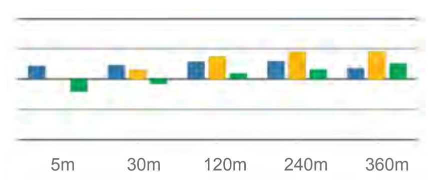

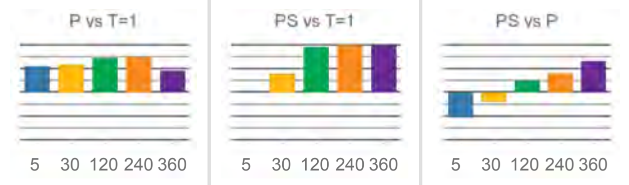

log$_2$ (Fold Change)

Legend: P vs T=1 | PS vs T=1 | PS vs P

Locus		5m	30m	120m	240m	360m
**PGN_1611**	P vs T=1	0.185	0.019	-0.150	-0.443	-0.813
	PS vs T=1	-0.840	-0.376	0.080	0.102	-0.192
	PS vs P	-0.963	-0.364	0.236	0.515	0.576

conserved hypothetical protein

unknown function

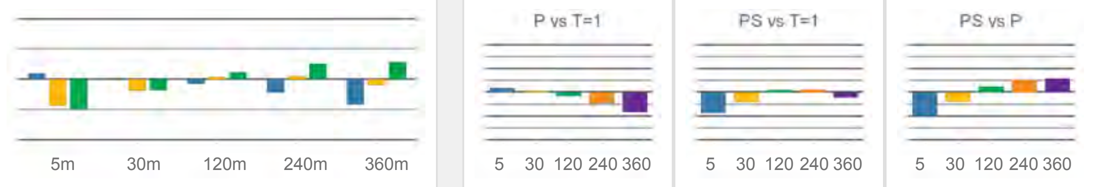

Locus		5m	30m	120m	240m	360m
**PGN_1612**	P vs T=1	-0.667	-0.319	-0.174	-0.250	-0.375
	PS vs T=1	-0.790	-0.802	-1.319	-1.472	-1.518
	PS vs P	-0.134	-0.438	-1.071	-1.136	-1.097

probable beta-phosphoglucomutase

energy metabolism

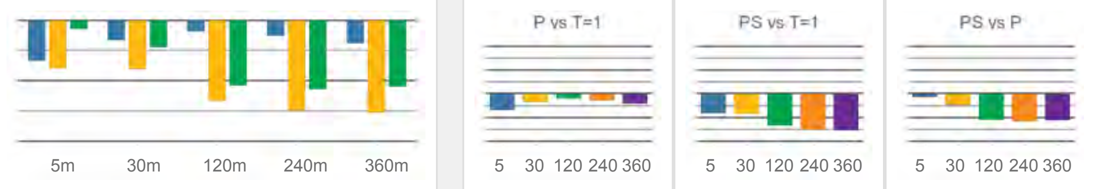

Locus		5m	30m	120m	240m	360m
**PGN_1613**	P vs T=1	-0.159	0.058	0.250	0.270	0.479
	PS vs T=1	-0.045	-0.226	-0.587	-0.405	-0.469
	PS vs P	0.105	-0.276	-0.815	-0.649	-0.919

ATP-dependent DNA helicase RecG

DNA metabolism

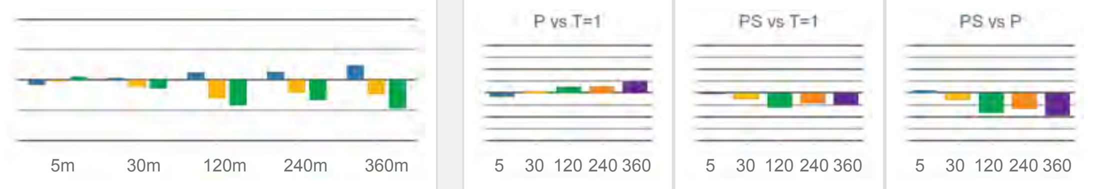

Locus		5m	30m	120m	240m	360m
**PGN_1614** *galE*	P vs T=1	0.078	0.623	0.914	0.606	0.198
	PS vs T=1	0.284	0.290	-0.073	-0.189	-0.111
	PS vs P	0.185	-0.313	-0.944	-0.771	-0.315

UDP-glucose 4-epimerase

energy metabolism

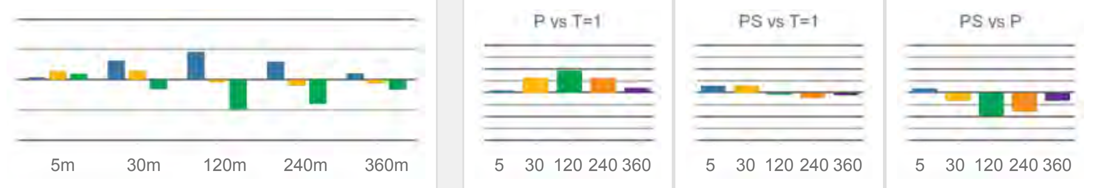

Locus		5m	30m	120m	240m	360m
**PGN_1615**	P vs T=1	-0.929	-0.859	-1.380	-1.686	-1.402
	PS vs T=1	-0.776	-0.902	-1.101	-1.142	-1.390
	PS vs P	0.140	-0.030	0.213	0.391	-0.033

putative GTP-binding protein

unknown function

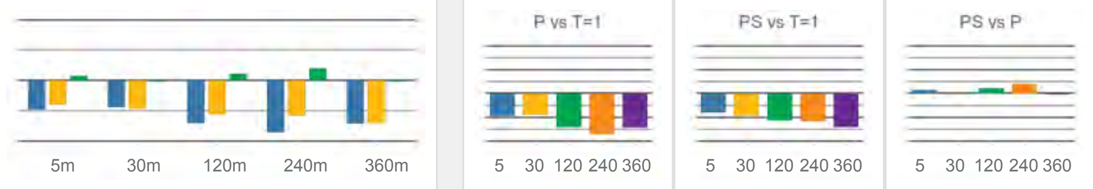

Locus		5m	30m	120m	240m	360m
**PGN_1616**	P vs T=1	-0.547	-0.412	-0.236	-0.146	-0.216
	PS vs T=1	-0.362	-0.211	0.918	1.634	1.314
	PS vs P	0.095	0.121	1.055	1.654	1.431

conserved hypothetical protein

hypothetical proteins-Conserved

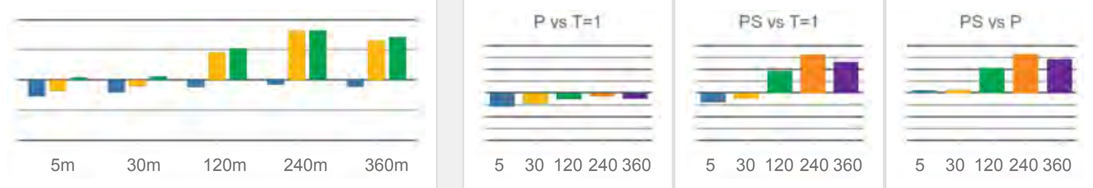

Locus		5m	30m	120m	240m	360m
**PGN_1617**	P vs T=1	-1.055	-0.960	-0.715	-0.553	-0.422
	PS vs T=1	-1.086	-1.409	-1.105	-0.504	-0.513
	PS vs P	-0.062	-0.466	-0.395	0.053	-0.085

probable metallophosphoesterase

central intermediary metabolism

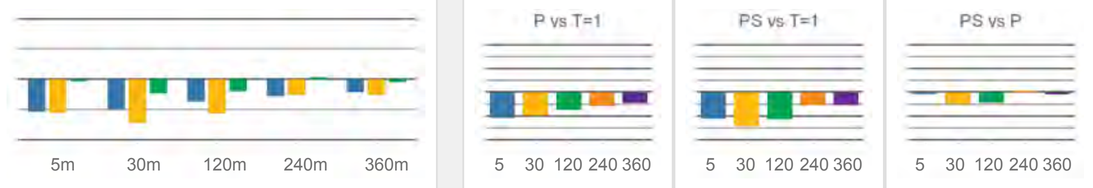

PgSg Time Course

log2 (Fold Change)

Legend: P vs T=1, PS vs T=1, PS vs P

Locus		5m	30m	120m	240m	360m
PGN_1618	P vs T=1	-0.348	-0.046	0.946	1.296	1.026
	PS vs T=1	-0.233	-0.507	-0.009	0.774	0.807
	PS vs P	0.035	-0.488	-0.858	-0.402	-0.171

methionine gamma-lyase

energy metabolism

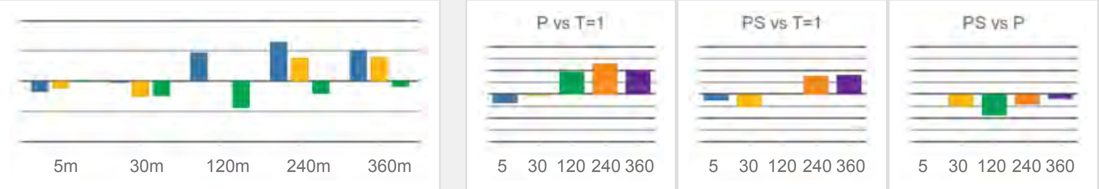

Locus		5m	30m	120m	240m	360m
PGN_1619	P vs T=1	0.422	0.360	0.394	0.508	0.134
	PS vs T=1	0.217	0.014	0.138	0.226	0.327
	PS vs P	-0.201	-0.348	-0.262	-0.277	0.172

transposase in ISPg1

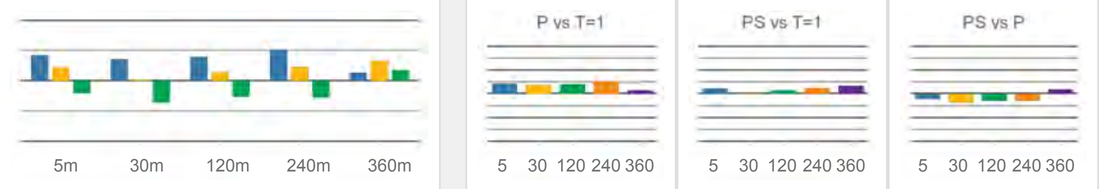

Locus		5m	30m	120m	240m	360m
PGN_1620	P vs T=1	-0.036	-0.217	-0.280	-0.300	-0.661
	PS vs T=1	-0.017	-0.343	-0.176	-0.015	-0.114
	PS vs P	0.027	-0.141	0.074	0.250	0.466

hypothetical protein

hypothetical proteins

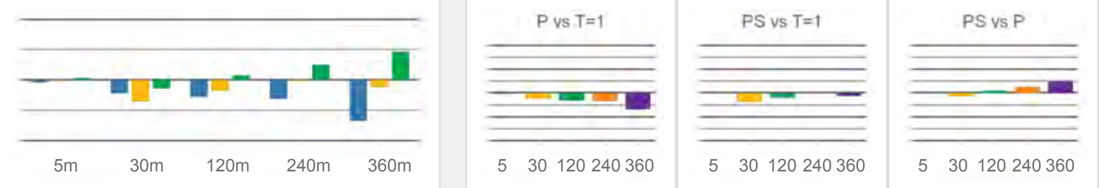

Locus		5m	30m	120m	240m	360m
PGN_1621	P vs T=1	-1.585	-1.701	-1.624	-1.639	-2.116
	PS vs T=1	-1.391	-1.283	-0.958	-0.717	-0.551
	PS vs P	0.101	0.293	0.564	0.806	1.384

conserved hypothetical protein

hypothetical proteins-Conserved

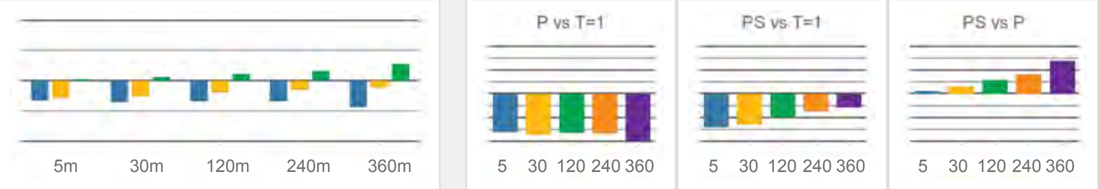

Locus		5m	30m	120m	240m	360m
PGN_1622	P vs T=1	0.171	-0.296	-0.285	-0.234	-0.635
	PS vs T=1	0.158	-0.238	-0.388	-0.306	-0.474
	PS vs P	0.009	0.030	-0.119	-0.080	0.123

conserved hypothetical protein

hypothetical proteins-Conserved

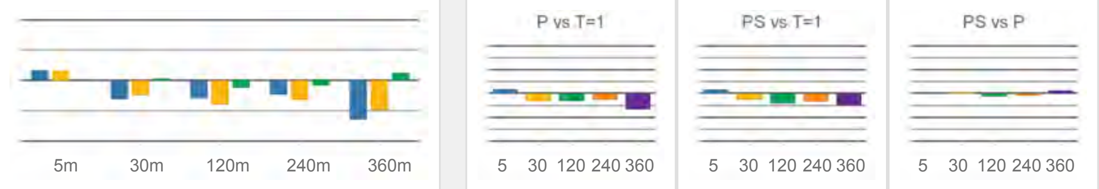

Locus		5m	30m	120m	240m	360m
PGN_1623	P vs T=1	-0.514	-0.222	-0.011	-0.028	0.243
	PS vs T=1	0.192	0.271	0.271	0.152	0.096
	PS vs P	0.676	0.478	0.277	0.170	-0.140

conserved hypothetical protein

hypothetical proteins-Conserved

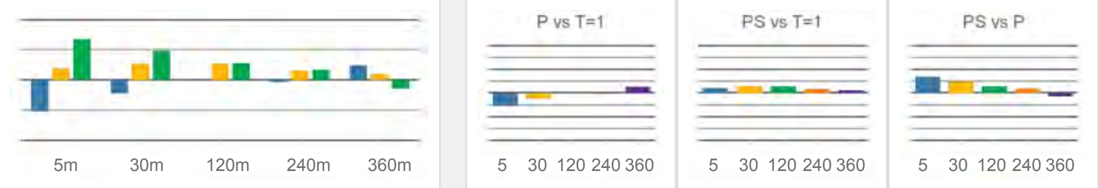

Locus		5m	30m	120m	240m	360m
PGN_1624	P vs T=1	1.351	1.653	1.387	1.223	2.808
	PS vs T=1	1.407	2.094	2.555	1.630	2.632
	PS vs P	0.051	0.504	0.965	0.107	0.464

conserved hypothetical protein

hypothetical proteins-Conserved

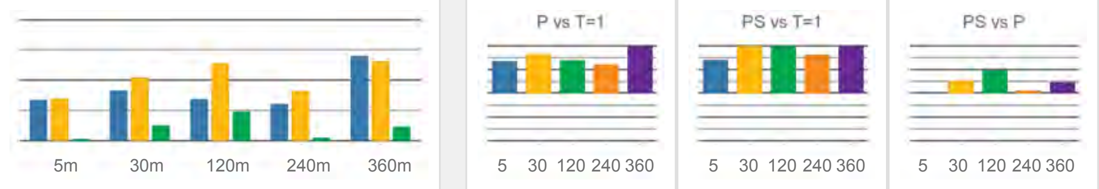

PgSg Time Course

log2 (Fold Change)

Locus		5m	30m	120m	240m	360m
PGN_1625	P vs T=1	1.059	1.673	2.083	2.048	1.759
	PS vs T=1	0.941	1.314	1.822	2.263	2.057
	PS vs P	-0.152	-0.328	-0.213	0.247	0.303
	conserved hypothetical protein					
	hypothetical proteins-Conserved					
PGN_1626	P vs T=1	-1.449	-1.032	-0.583	-0.217	-0.068
	PS vs T=1	-1.130	-1.224	-1.356	-0.496	-0.393
	PS vs P	0.259	-0.210	-0.758	-0.229	-0.294
	putative tRNA isopentenyltransferase					
	protein synthesis					
PGN_1627	P vs T=1	-0.798	-1.169	-1.599	-1.235	-0.656
	PS vs T=1	-0.189	-0.305	-0.373	-0.671	-0.834
	PS vs P	0.597	0.810	1.108	0.508	-0.162
	probable 4-amino-4-deoxy-L-arabinose transferase					
	cell envelope					
PGN_1628	P vs T=1	-0.481	-0.406	-0.581	-0.555	-0.856
	PS vs T=1	0.014	-0.148	-0.671	-1.086	-1.345
	PS vs P	0.484	0.257	-0.096	-0.520	-0.505
	putative glycosyltransferases					
	cell envelope					
PGN_1629	P vs T=1	-0.314	-0.070	0.545	0.760	0.879
	PS vs T=1	-0.292	-0.266	-0.340	-0.404	-0.428
	PS vs P	-0.002	-0.203	-0.832	-1.073	-1.239
	conserved hypothetical protein with integral membrane domain DUF6					
	hypothetical proteins-Conserved					
PGN_1630	P vs T=1	-0.243	0.043	0.717	0.811	0.725
	PS vs T=1	-0.548	-0.364	-0.263	0.088	0.173
	PS vs P	-0.326	-0.401	-0.917	-0.661	-0.518
	transcription termination factor Rho					
	transcription					
PGN_1631	P vs T=1	0.193	0.840	1.592	1.636	1.353
	PS vs T=1	-0.576	-0.832	-0.195	0.069	-0.116
	PS vs P	-0.744	-1.476	-1.518	-1.272	-1.283
	piutative DNA-binding protein histone-like family					
	DNA metabolism					

P vs T=1 | PS vs T=1 | PS vs P

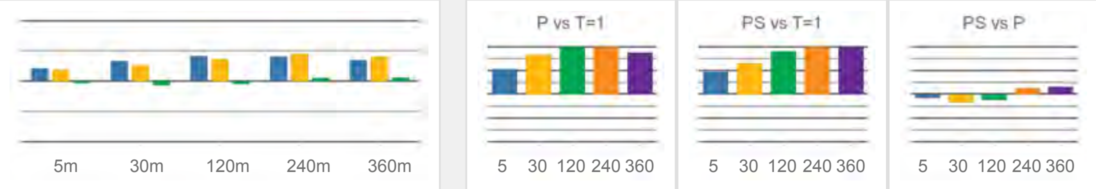

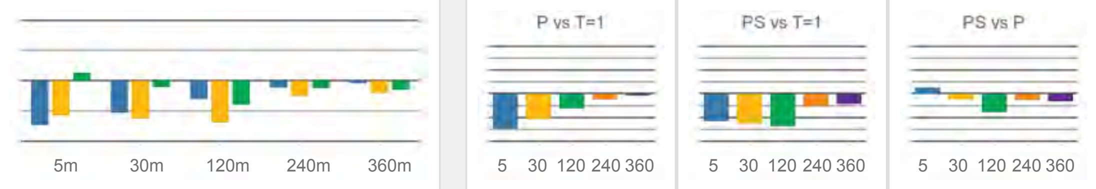

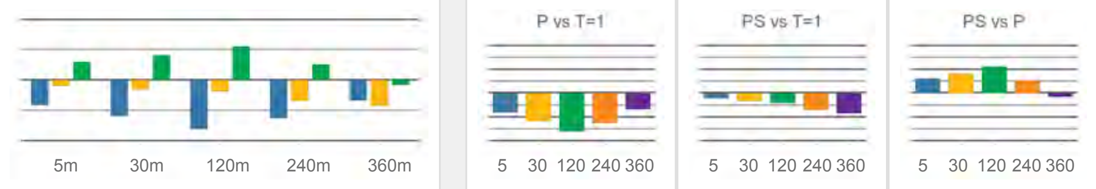

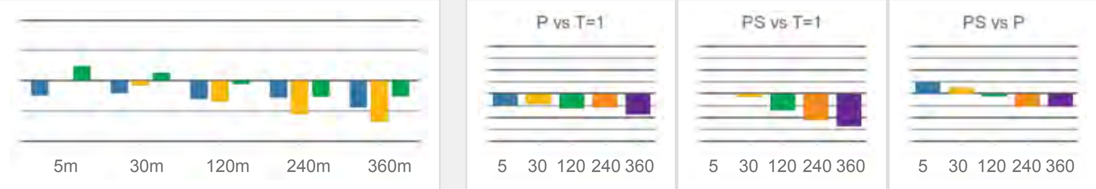

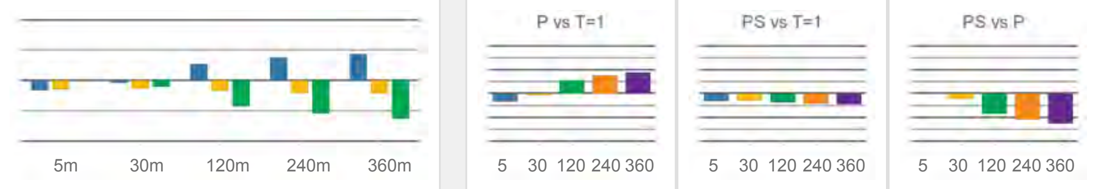

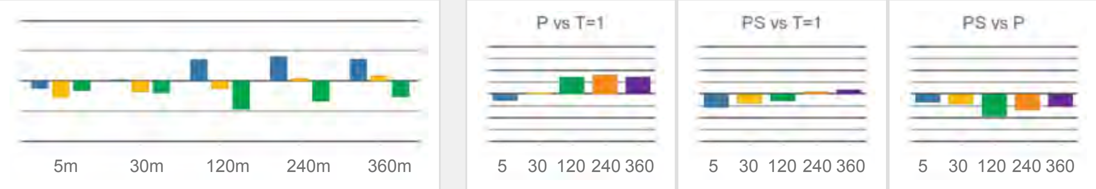

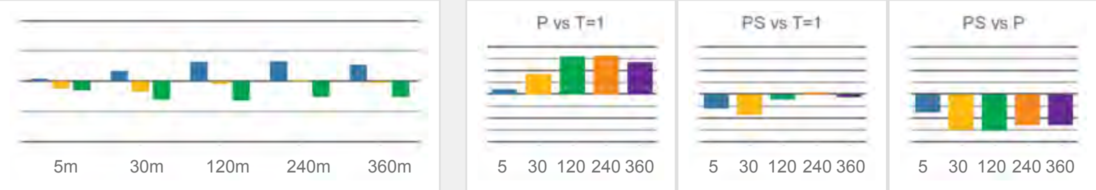

PgSg Time Course

log$_2$ (Fold Change)

Legend: P vs T=1 | PS vs T=1 | PS vs P

Locus		5m	30m	120m	240m	360m
**PGN_1632**	P vs T=1	-0.392	-0.189	0.912	0.955	0.694
	PS vs T=1	-0.699	-0.489	0.289	0.442	0.343
	PS vs P	-0.351	-0.323	-0.470	-0.370	-0.285

hypothetical protein

hypothetical proteins

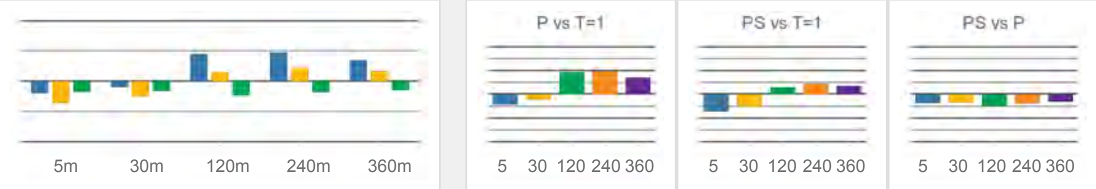

Locus		5m	30m	120m	240m	360m
**PGN_1633**	P vs T=1	-1.502	-0.665	0.556	1.206	0.711
	PS vs T=1	-1.432	-0.985	0.057	0.720	0.867
	PS vs P	-0.310	-0.413	-0.353	-0.223	0.220

formiminotransferase-cyclodeaminase

central intermediary metabolism

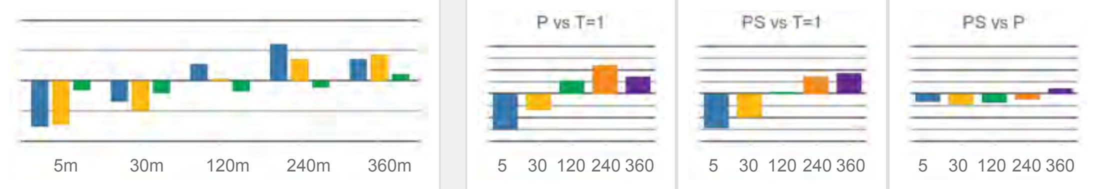

Locus		5m	30m	120m	240m	360m
**PGN_1634**	P vs T=1	-1.081	-0.722	0.494	1.069	0.873
	PS vs T=1	-0.926	-0.701	-0.163	0.516	0.772
	PS vs P	0.047	-0.051	-0.591	-0.436	-0.053

imidazolonepropionase

energy metabolism

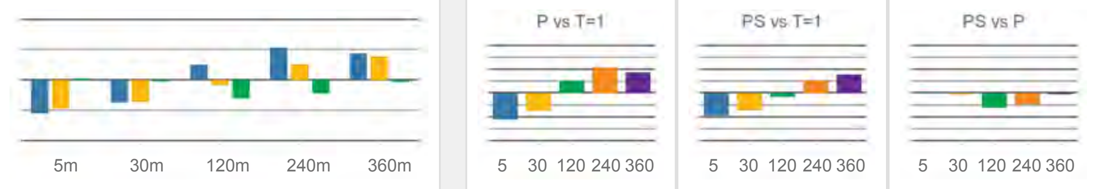

Locus		5m	30m	120m	240m	360m
**PGN_1635**	P vs T=1	-1.647	-1.128	-0.317	-0.035	-0.209
	PS vs T=1	-1.476	-1.178	-0.606	-0.309	-0.103
	PS vs P	0.065	-0.095	-0.262	-0.227	0.113

conserved hypothetical protein with nucleoside recognition domain

hypothetical proteins-Conserved

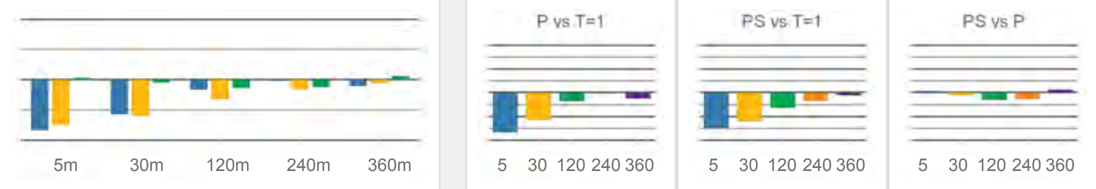

Locus		5m	30m	120m	240m	360m
**PGN_1636**	P vs T=1	-0.809	-0.247	0.582	0.703	0.864
	PS vs T=1	-0.850	-0.570	-0.255	0.157	-0.180
	PS vs P	-0.214	-0.315	-0.629	-0.334	-0.842

conserved hypothetical protein

hypothetical proteins-Conserved

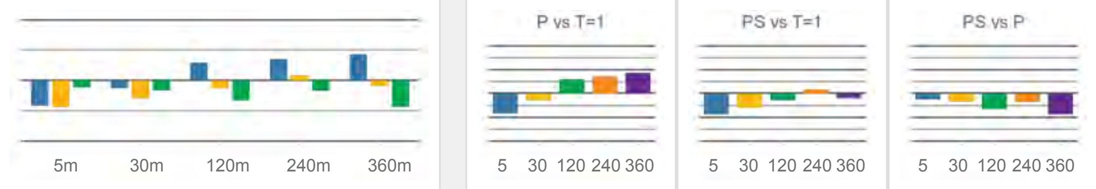

Locus		5m	30m	120m	240m	360m
**PGN_1637**	P vs T=1	-0.866	-0.033	0.849	0.578	0.647
	PS vs T=1	-0.890	-0.284	0.086	0.521	0.434
	PS vs P	-0.231	-0.229	-0.553	0.032	-0.128

putative methenyltetrahydrofolate cyclohydrolase

hypothetical proteins-Conserved

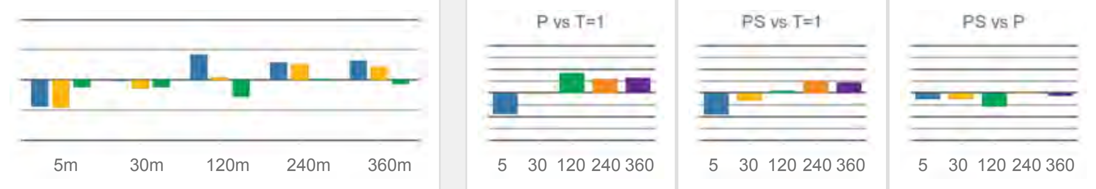

Locus		5m	30m	120m	240m	360m
**PGN_1638**	P vs T=1	-0.678	-0.236	0.502	0.643	0.549
	PS vs T=1	-0.585	-0.494	-0.362	0.224	0.308
	PS vs P	-0.032	-0.269	-0.732	-0.286	-0.171

histidine ammonia-lyase

energy metabolism

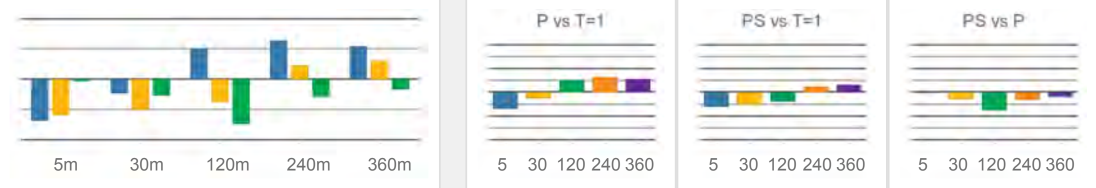

PgSg Time Course

Hackett Laboratory UW

log2 (Fold Change)

P vs T=1 | PS vs T=1 | PS vs P

Locus		5m	30m	120m	240m	360m
**PGN_1639**	P vs T=1	2.464	2.188	2.409	2.580	1.929
	PS vs T=1	3.780	4.058	3.949	3.707	3.679
	PS vs P	1.291	1.808	1.510	1.116	1.699
	conserved hypothetical protein					
	hypothetical proteins-Conserved					
**PGN_1640**	P vs T=1	-0.455	-0.428	-0.387	-0.748	-1.050
	PS vs T=1	-0.007	-0.140	-0.619	-1.018	-0.865
	PS vs P	0.444	0.287	-0.218	-0.279	0.165
	serine/threonine transporter					
	transport and binding proteins					
**PGN_1641**	P vs T=1	-0.331	-0.247	-0.130	-0.007	-0.068
	PS vs T=1	0.033	-0.213	-0.521	-0.255	-0.133
	PS vs P	0.342	0.026	-0.384	-0.227	-0.061
	arginine/ornithine transport system ATPase					
	transport and binding proteins					
**PGN_1642**	P vs T=1	-0.665	-0.253	0.175	0.276	-0.009
	PS vs T=1	-0.598	-0.454	-0.469	-0.300	-0.231
	PS vs P	0.027	-0.202	-0.615	-0.539	-0.221
	conserved hypothetical protein					
	hypothetical proteins-Conserved					
**PGN_1643**	P vs T=1	1.509	1.772	2.092	1.661	0.765
	PS vs T=1	1.470	1.970	2.260	2.030	1.850
	PS vs P	-0.040	0.212	0.208	0.373	1.044
	conserved hypothetical protein					
	hypothetical proteins-Conserved					
**PGN_1644**	P vs T=1	0.325	0.267	0.260	0.382	-0.022
	PS vs T=1	0.125	-0.091	0.029	0.224	0.155
	PS vs P	-0.197	-0.359	-0.239	-0.156	0.156
	transposase in ISPg1					
**PGN_1645**	P vs T=1	-0.623	-0.392	0.098	0.325	0.855
	PS vs T=1	-0.410	-0.597	-0.198	0.019	0.184
	PS vs P	0.169	-0.220	-0.267	-0.257	-0.603
	putative dipeptidyl peptidase III					
	hypothetical proteins-Conserved					

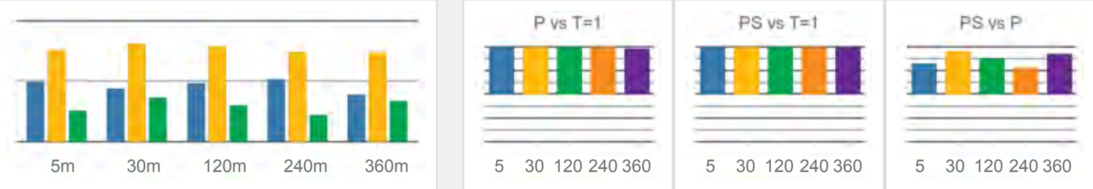

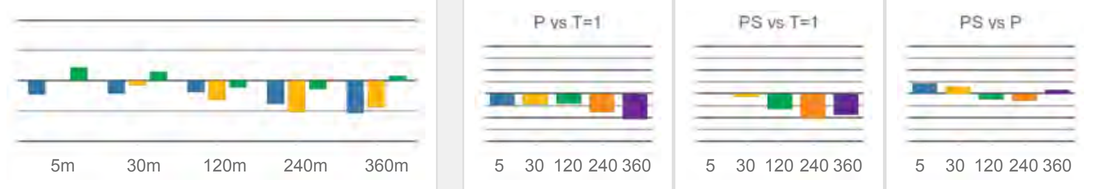

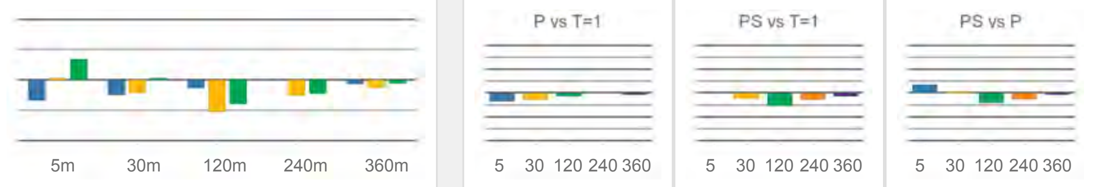

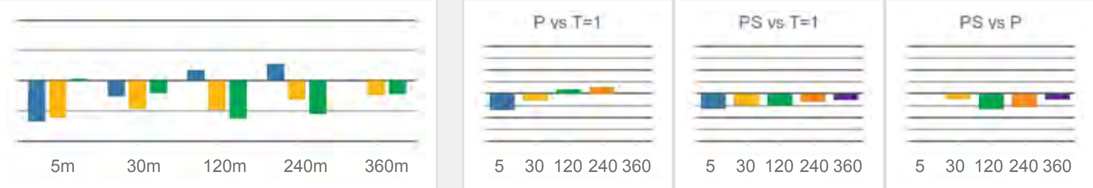

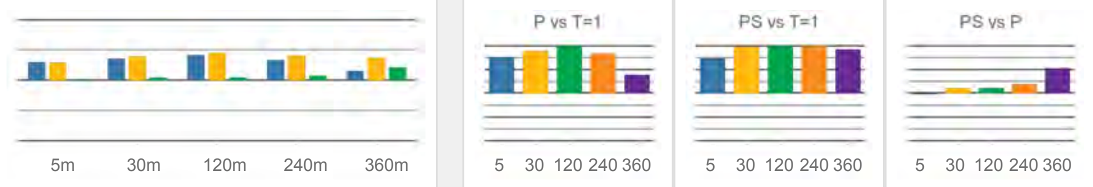

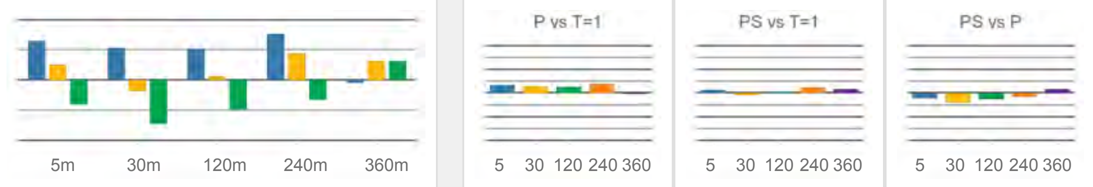

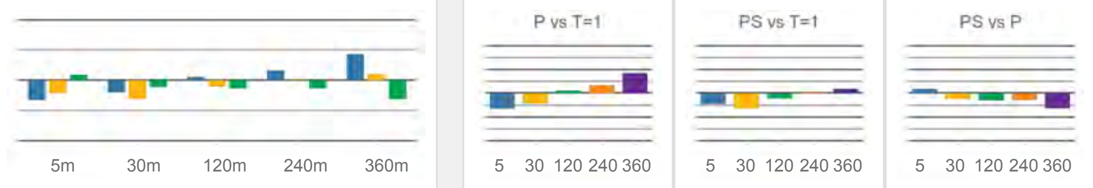

PgSg Time Course

Legend: P vs T=1 | PS vs T=1 | PS vs P

Locus		log$_2$ (Fold Change) 5m	30m	120m	240m	360m
**PGN_1646**	P vs T=1	-0.294	-0.039	0.228	0.153	-0.117
	PS vs T=1	-0.844	-0.891	-1.102	-0.810	-0.805
	PS vs P	-0.540	-0.820	-1.276	-0.906	-0.669

seryl-tRNA synthetase

protein synthesis

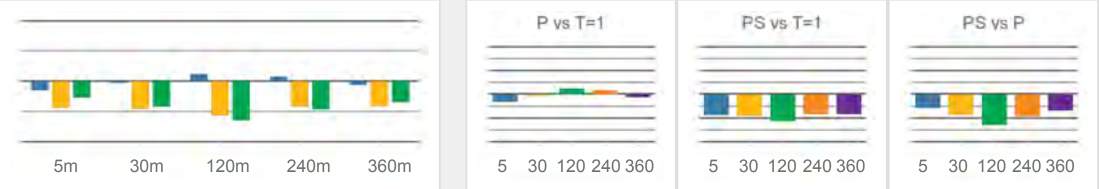

Locus		5m	30m	120m	240m	360m
**PGN_1647** *rpmA*	P vs T=1	0.019	0.344	1.081	1.221	0.465
	PS vs T=1	-1.302	-0.797	-0.526	-0.880	-0.938
	PS vs P	-1.290	-1.091	-1.522	-2.005	-1.372

putative 50S ribosomal protein L27

protein synthesis

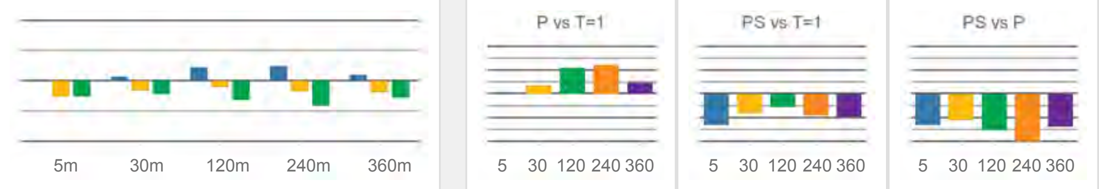

Locus		5m	30m	120m	240m	360m
**PGN_1648** *rplU*	P vs T=1	0.490	0.742	1.499	1.455	0.576
	PS vs T=1	-0.848	-0.362	0.031	-0.408	-0.263
	PS vs P	-1.262	-1.007	-1.300	-1.703	-0.805

putative 50S ribosomal protein L21

protein synthesis

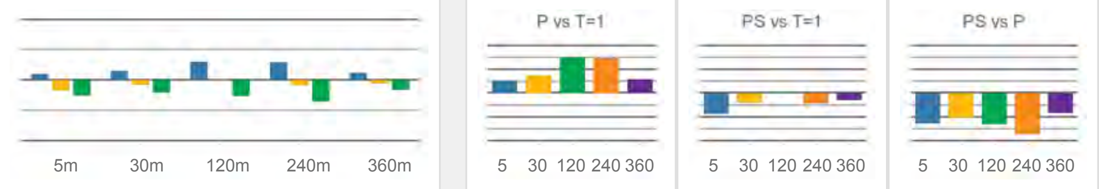

Locus		5m	30m	120m	240m	360m
**PGN_1649**	P vs T=1	3.082	3.317	4.069	3.988	3.362
	PS vs T=1	2.075	2.607	3.256	2.613	2.999
	PS vs P	-0.762	-0.458	-0.413	-0.983	-0.193

conserved hypothetical protein

hypothetical proteins-Conserved

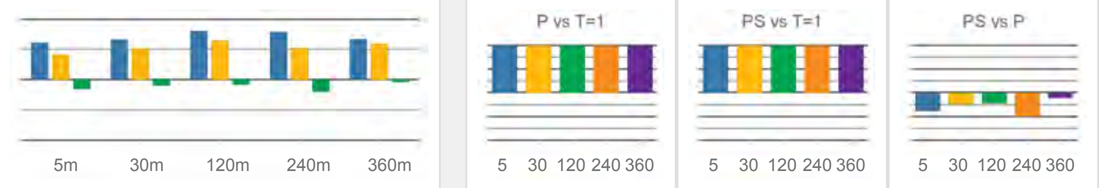

Locus		5m	30m	120m	240m	360m
**PGN_1650**	P vs T=1	-0.059	-0.466	-1.049	-0.976	-0.574
	PS vs T=1	-0.540	-0.570	-0.185	0.084	0.078
	PS vs P	-0.434	-0.104	0.734	0.920	0.632

conserved hypothetical protein

hypothetical proteins-Conserved

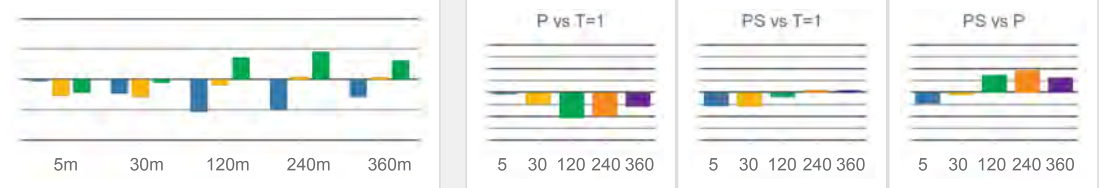

Locus		5m	30m	120m	240m	360m
**PGN_1651**	P vs T=1	0.498	0.669	0.577	0.132	-0.038
	PS vs T=1	0.369	0.560	0.842	0.825	0.466
	PS vs P	-0.126	-0.094	0.278	0.647	0.471

putative glycosyltransferase

cell envelope

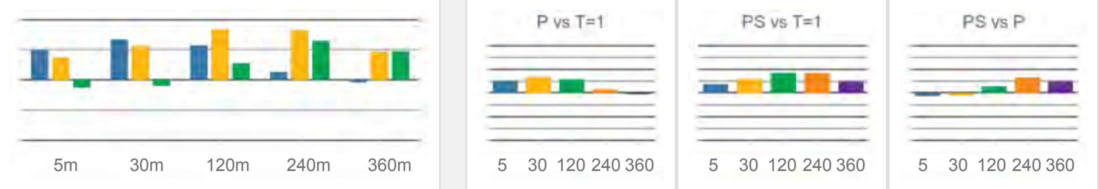

Locus		5m	30m	120m	240m	360m
**PGN_1652**	P vs T=1	-1.025	-0.651	-0.537	-0.719	-0.825
	PS vs T=1	-0.772	-0.533	-0.330	-0.223	-0.720
	PS vs P	0.205	0.112	0.211	0.465	0.078

probable nitroreductase

unknown function

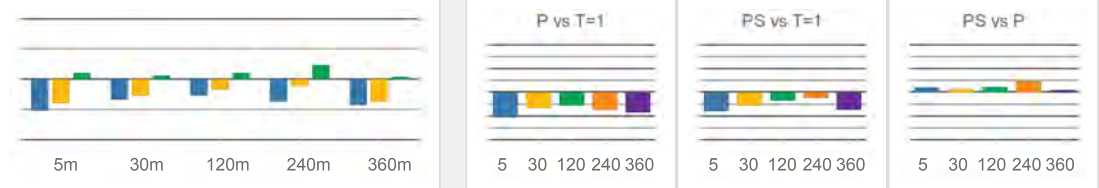

log2 (Fold Change)

Legend: P vs T=1 | PS vs T=1 | PS vs P

Locus		5m	30m	120m	240m	360m
**PGN_1653**	P vs T=1	0.612	0.684	0.528	0.382	0.828
ApbE	PS vs T=1	0.789	0.820	0.528	0.318	0.326
	PS vs P	0.180	0.148	0.004	-0.083	-0.465

putative thiamine biosynthesis lipoprotein ApbE

biosynthesis of cofactors, prosthetic groups, and carriers

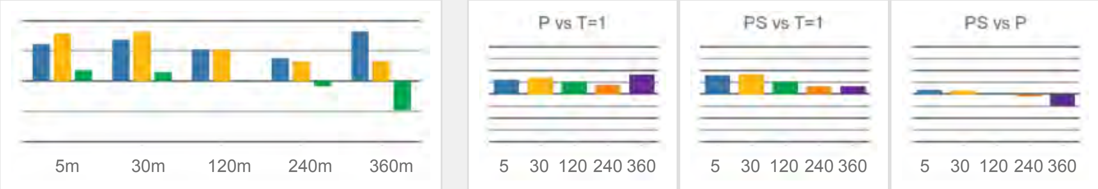

Locus		5m	30m	120m	240m	360m
**PGN_1654**	P vs T=1	-0.246	0.588	1.391	1.349	0.609
	PS vs T=1	-0.384	0.366	0.909	0.897	1.059
	PS vs P	-0.249	-0.199	-0.373	-0.366	0.430

putative electron transport complex RnfABCDGE type A subunit

energy metabolism

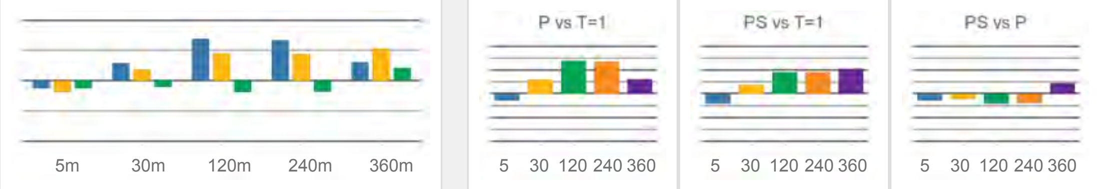

Locus		5m	30m	120m	240m	360m
**PGN_1655**	P vs T=1	0.299	1.133	1.724	1.406	0.538
	PS vs T=1	0.495	1.116	1.417	1.275	1.322
	PS vs P	0.100	0.009	-0.219	-0.095	0.728

putative electron transport complex RnfABCDGE type E subunit

energy metabolism

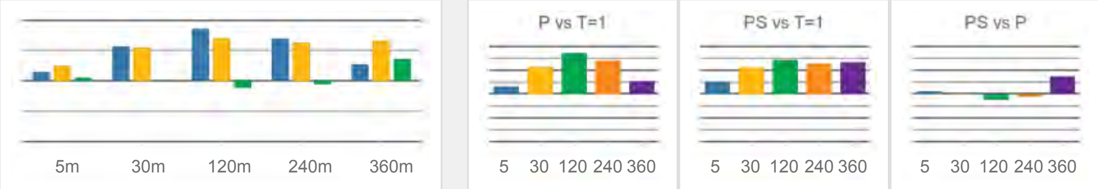

Locus		5m	30m	120m	240m	360m
**PGN_1656**	P vs T=1	1.145	1.987	2.554	1.831	0.768
	PS vs T=1	1.378	1.711	2.099	2.030	1.968
	PS vs P	0.159	-0.210	-0.325	0.208	1.105

putative electron transport complex RnfABCDGE type G subunit

hypothetical proteins-Conserved

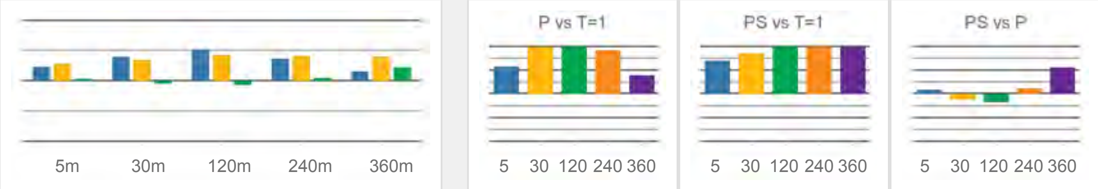

Locus		5m	30m	120m	240m	360m
**PGN_1657**	P vs T=1	0.894	1.044	0.596	0.288	-0.073
	PS vs T=1	1.154	0.930	0.461	0.443	0.463
	PS vs P	0.275	-0.069	-0.134	0.124	0.488

electron transport complex RnfABCDGE type D subunit

cell envelope

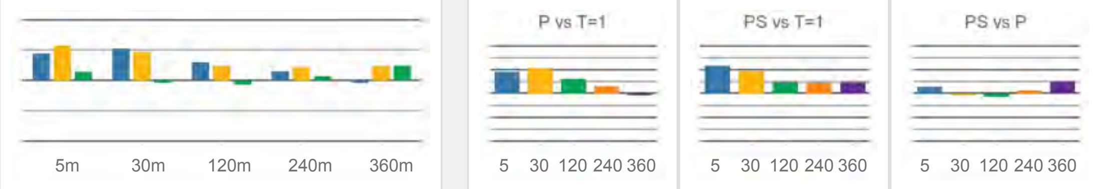

Locus		5m	30m	120m	240m	360m
**PGN_1658**	P vs T=1	0.749	0.921	0.815	0.283	-0.274
	PS vs T=1	0.798	0.541	0.209	0.174	0.122
	PS vs P	0.072	-0.326	-0.557	-0.124	0.344

electron transport complex RnfABCDGE type C subunit

energy metabolism

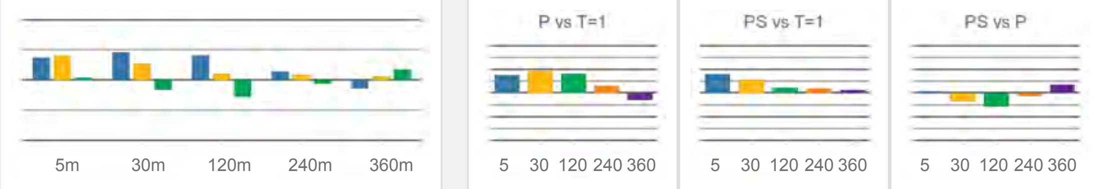

Locus		5m	30m	120m	240m	360m
**PGN_1659**	P vs T=1	0.348	0.220	0.248	0.091	-0.273
	PS vs T=1	0.373	0.231	0.245	0.402	0.225
	PS vs P	0.051	0.014	0.013	0.297	0.444

putative electron transport complex RnfABCDGE type B subunit

energy metabolism

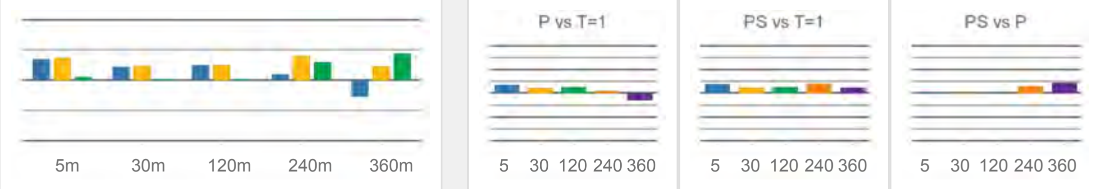

PgSg Time Course

log$_2$ (Fold Change)

Legend: P vs T=1 | PS vs T=1 | PS vs P

Locus		5m	30m	120m	240m	360m
**PGN_1660**	P vs T=1	-0.983	-1.771	-2.345	-2.184	-2.213
	PS vs T=1	-1.767	-1.693	-1.161	-1.045	-1.189
	PS vs P	-0.634	0.007	0.933	0.949	0.869
	conserved hypothetical protein					
	hypothetical proteins-Conserved					
**PGN_1661**	P vs T=1	-0.182	-0.012	0.111	0.553	1.074
	PS vs T=1	-0.546	-0.358	0.809	1.395	1.314
	PS vs P	-0.393	-0.365	0.637	0.827	0.265
	conserved hypothetical protein					
	unknown function					
**PGN_1662**	P vs T=1	0.034	-0.839	-0.186	0.063	1.270
	PS vs T=1	-0.242	-0.626	0.546	0.639	0.585
	PS vs P	-0.201	-0.152	0.612	0.478	-0.296
	partial transposase in ISPg3					
**PGN_1663**	P vs T=1	0.104	-0.574	-0.597	0.465	1.654
	PS vs T=1	-0.312	-1.597	-0.197	-0.658	-0.073
	PS vs P	-0.193	-0.875	-0.062	-0.521	-0.533
	hypothetical protein					
	hypothetical proteins					
**PGN_1664**	P vs T=1	-0.135	-0.876	-1.320	-0.470	0.283
	PS vs T=1	-0.611	-1.285	-1.545	-1.788	-0.710
	PS vs P	-0.255	-0.498	-0.712	-0.908	-0.436
	conserved hypothetical protein					
	hypothetical proteins-Conserved					
**PGN_1665**	P vs T=1	0.317	-1.155	-1.086	-0.029	1.265
	PS vs T=1	-0.194	-0.580	-1.108	-0.056	0.563
	PS vs P	-0.211	-0.140	-0.678	-0.043	-0.034
	hypothetical protein					
	hypothetical proteins					
**PGN_1666**	P vs T=1	-0.313	0.247	0.843	0.871	0.540
	PS vs T=1	-0.046	0.071	0.296	0.509	0.442
	PS vs P	0.231	-0.175	-0.520	-0.337	-0.095
	phosphoribosylformylglycinamidine synthase					
	purines, pyrimidines, nucleosides and nucleotides					

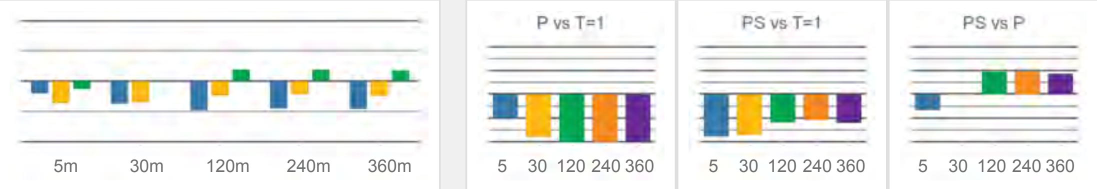

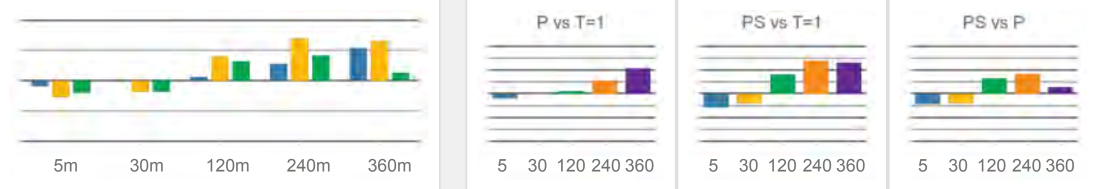

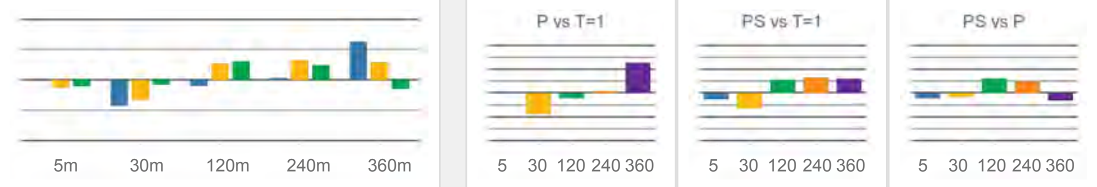

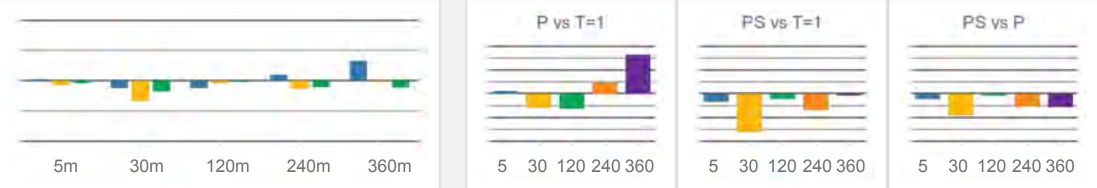

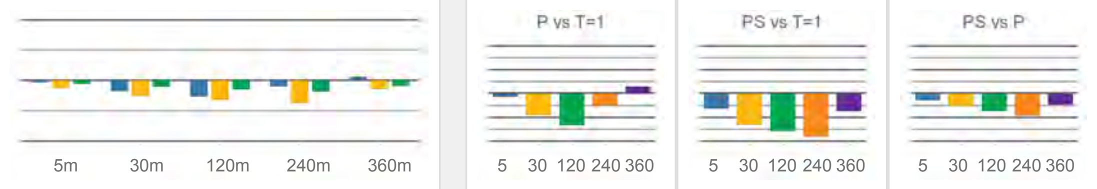

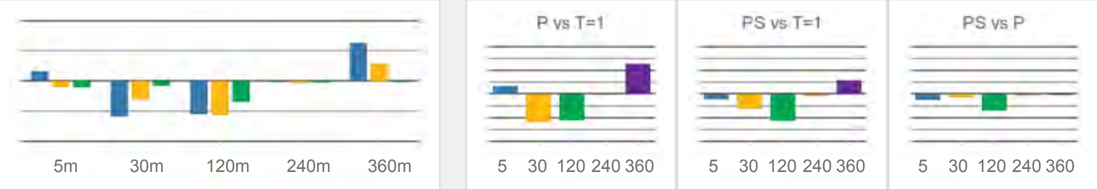

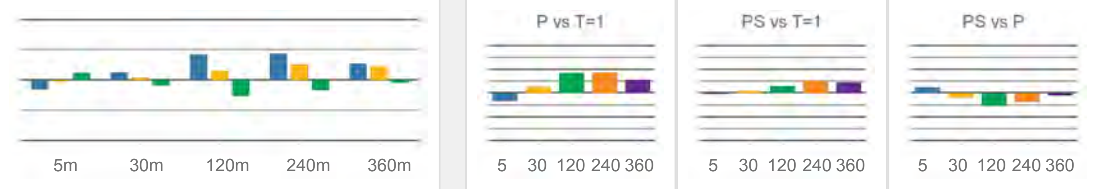

PgSg Time Course

log$_2$ (Fold Change)

Legend: P vs T=1 | PS vs T=1 | PS vs P

Locus		5m	30m	120m	240m	360m
**PGN_1667**	P vs T=1	-0.647	-0.311	0.140	0.081	0.048
	PS vs T=1	0.002	-0.137	-0.454	-0.274	-0.143
	PS vs P	0.572	0.147	-0.554	-0.332	-0.184
	putative DNA processing Smf-like protein					
	cellular processes					
**PGN_1668**	P vs T=1	-0.862	-0.889	-0.615	-0.023	-0.008
	PS vs T=1	-0.369	-0.382	-0.488	-0.476	-0.073
	PS vs P	0.450	0.451	0.088	-0.395	-0.046
	conserved hypothetical protein					
	cell envelope					
**PGN_1669**	P vs T=1	0.274	0.194	0.216	0.330	-0.023
	PS vs T=1	0.165	-0.056	0.073	0.161	0.185
	PS vs P	-0.110	-0.256	-0.152	-0.169	0.188
	transposase in ISPg1					
**PGN_1670**	P vs T=1	0.146	0.500	0.739	0.375	-0.514
	PS vs T=1	-0.052	0.335	0.506	0.062	-0.205
	PS vs P	-0.213	-0.151	-0.200	-0.307	0.266
	conserved hypothetical protein with predicted lysozyme domain					
	protein fate					
**PGN_1671**	P vs T=1	0.309	0.248	0.120	0.038	-0.317
	PS vs T=1	-0.021	-0.096	-0.191	-0.362	-0.452
	PS vs P	-0.318	-0.335	-0.309	-0.399	-0.150
	probable chromate transport protein					
	transport and binding proteins					
**PGN_1672**	P vs T=1	1.269	0.946	0.869	0.955	1.013
	PS vs T=1	1.011	1.039	0.867	0.878	0.985
	PS vs P	-0.209	0.086	-0.015	-0.075	-0.020
	probable chromate transport protein					
	transport and binding proteins					
**PGN_1673** *porN*	P vs T=1	-0.501	-0.521	-0.612	-0.848	-1.299
	PS vs T=1	0.610	0.765	0.359	-0.307	-0.588
	PS vs P	1.083	1.256	0.953	0.510	0.667
	Por secretion system protein porN/gldN					
	hypothetical proteins					

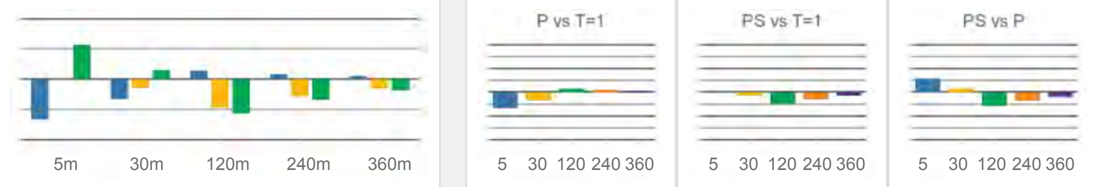

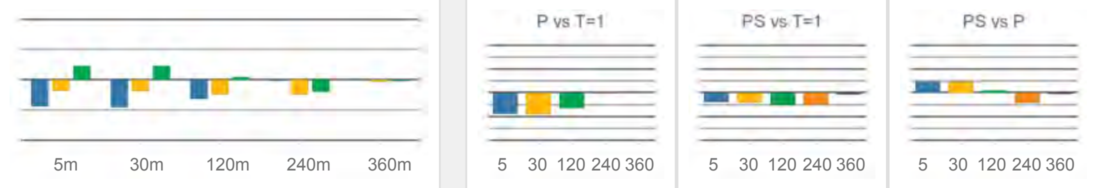

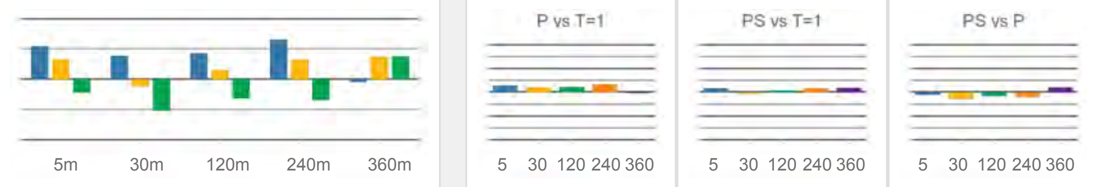

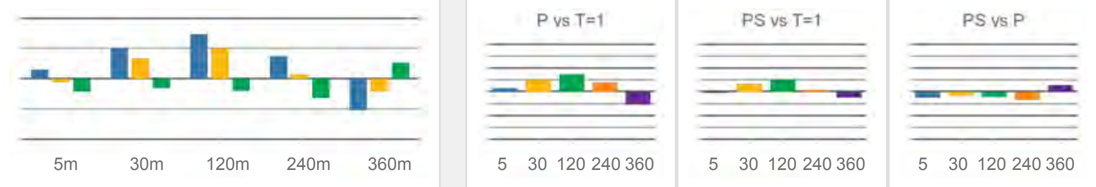

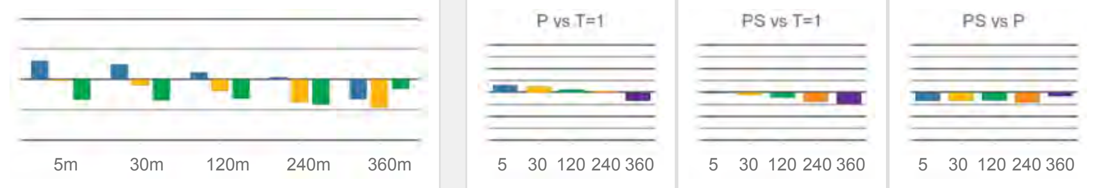

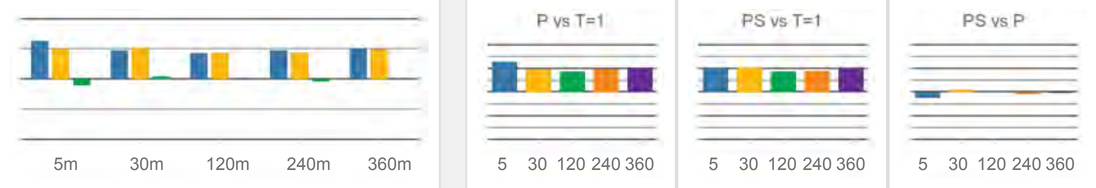

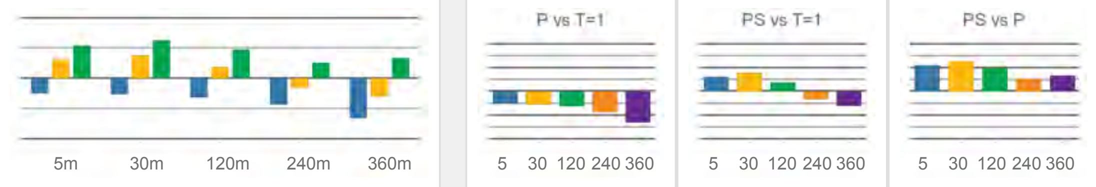

PgSg Time Course

log$_2$ (Fold Change)

Legend: P vs T=1 | PS vs T=1 | PS vs P

Locus		5m	30m	120m	240m	360m
**PGN_1674**	P vs T=1	0.146	0.044	-0.596	-0.840	-0.773
porM	PS vs T=1	1.318	1.182	0.379	-0.493	-0.780
	PS vs P	1.157	1.121	0.923	0.277	-0.038
	Por secretion system protein porM/gldM					
	hypothetical proteins					

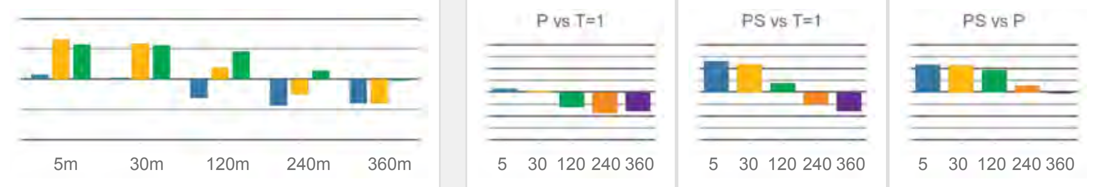

Locus		5m	30m	120m	240m	360m
**PGN_1675**	P vs T=1	0.489	0.445	-0.133	-0.297	-0.168
porL	PS vs T=1	1.366	1.100	0.083	-0.269	-0.072
	PS vs P	0.874	0.656	0.184	-0.021	0.080
	Por secretion system protein porL/gldL					
	hypothetical proteins					

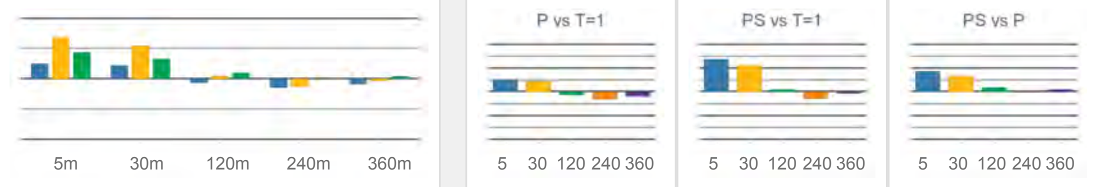

Locus		5m	30m	120m	240m	360m
**PGN_1676**	P vs T=1	-0.093	-0.652	-1.342	-1.289	-1.295
porK	PS vs T=1	0.523	0.095	-0.659	-0.803	-0.950
	PS vs P	0.624	0.739	0.643	0.450	0.323
	Por secretion system protein porK/gldK					
	cell envelope					

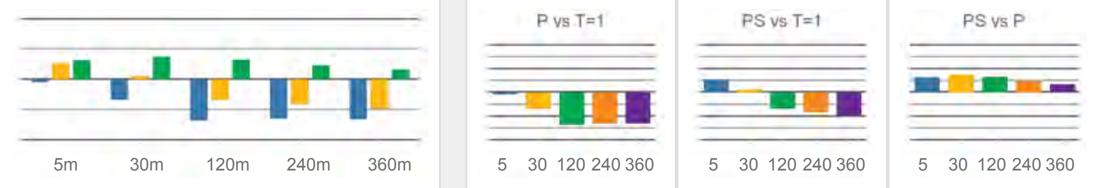

Locus		5m	30m	120m	240m	360m
**PGN_1677**	P vs T=1	-0.014	-0.341	-0.533	-0.383	-0.512
porP	PS vs T=1	0.483	0.084	-0.280	-0.427	-0.530
	PS vs P	0.506	0.404	0.219	-0.045	-0.033
	Por secretion system protein porP					
	hypothetical proteins					

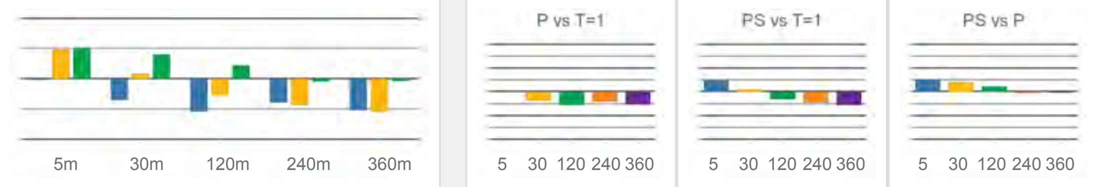

Locus		5m	30m	120m	240m	360m
**PGN_1678**	P vs T=1	0.054	-0.044	0.137	-0.179	-0.812
	PS vs T=1	-0.953	-0.786	-0.897	-1.138	-1.206
	PS vs P	-0.928	-0.678	-0.937	-0.882	-0.402
	conserved hypothetical protein					
	hypothetical proteins-Conserved					

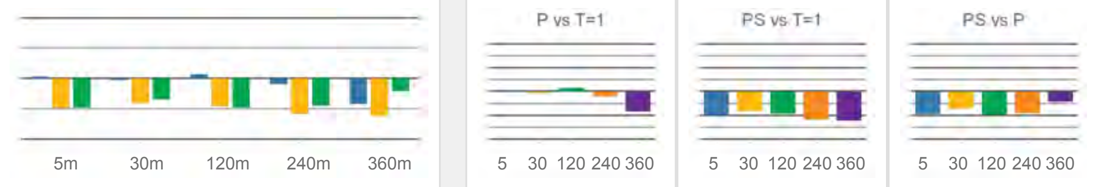

Locus		5m	30m	120m	240m	360m
**PGN_1679**	P vs T=1	0.651	0.484	1.630	2.664	4.102
	PS vs T=1	0.127	0.474	1.090	1.808	2.168
	PS vs P	-0.563	-0.246	-0.414	-0.367	-1.325
	conserved hypothetical protein					
	hypothetical proteins-Conserved					

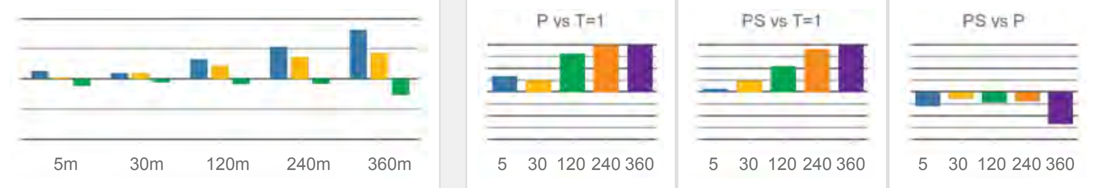

Locus		5m	30m	120m	240m	360m
**PGN_1680**	P vs T=1	-0.223	-0.086	0.680	1.641	3.075
	PS vs T=1	-0.503	-0.451	0.303	1.388	1.326
	PS vs P	-0.374	-0.452	-0.365	0.008	-1.434
	putative ABC transporter permease protein					
	transport and binding proteins					

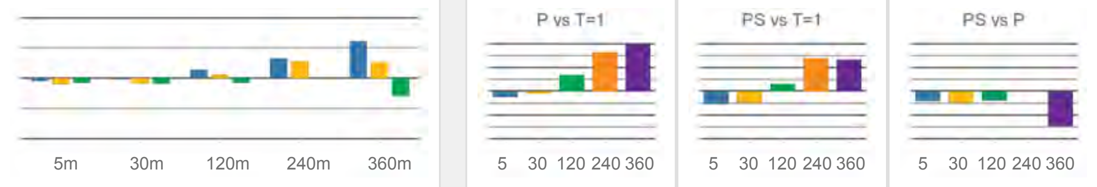

log$_2$ (Fold Change)

Locus		5m	30m	120m	240m	360m
**PGN_1681**	P vs T=1	-0.235	-0.174	0.588	1.141	2.399
	PS vs T=1	-0.814	-0.450	0.169	0.580	0.855
	PS vs P	-0.638	-0.375	-0.317	-0.270	-1.051
	putative ABC transporter ATP-binding protein					
	transport and binding proteins					
**PGN_1682**	P vs T=1	0.308	0.341	0.766	1.753	2.695
	PS vs T=1	-0.007	0.060	0.356	0.979	1.424
	PS vs P	-0.341	-0.321	-0.437	-0.512	-1.029
	probable ABC transporter permease protein					
	transport and binding proteins					
**PGN_1683**	P vs T=1	0.532	0.810	1.366	2.003	2.923
	PS vs T=1	0.829	0.866	1.331	1.450	1.682
	PS vs P	0.204	0.006	-0.019	-0.380	-1.028
	probable ABC transporter permease protein					
	transport and binding proteins					
**PGN_1684**	P vs T=1	-0.624	-0.098	0.583	0.366	0.802
	PS vs T=1	-0.689	-0.344	0.013	-0.743	0.494
	PS vs P	-0.354	-0.193	-0.106	-0.615	0.136
	hypothetical protein					
	hypothetical proteins					
**PGN_1685**	P vs T=1	-0.145	0.253	0.409	0.192	-0.083
	PS vs T=1	0.004	-0.157	-0.334	-0.204	-0.330
	PS vs P	0.139	-0.393	-0.717	-0.383	-0.250
	NADP-dependent malate dehydrogenase					
	energy metabolism					
**PGN_1686**	P vs T=1	-0.209	0.419	0.789	0.158	-0.102
	PS vs T=1	0.055	0.648	0.938	1.010	0.996
	PS vs P	0.091	0.231	0.270	0.642	0.872
	hypothetical protein					
	hypothetical proteins					
**PGN_1687**	P vs T=1	0.550	0.948	0.789	0.297	0.033
	PS vs T=1	1.496	2.315	2.389	1.773	1.358
	PS vs P	0.850	1.325	1.550	1.292	1.178
	conserved hypothetical protein					
	hypothetical proteins-Conserved					

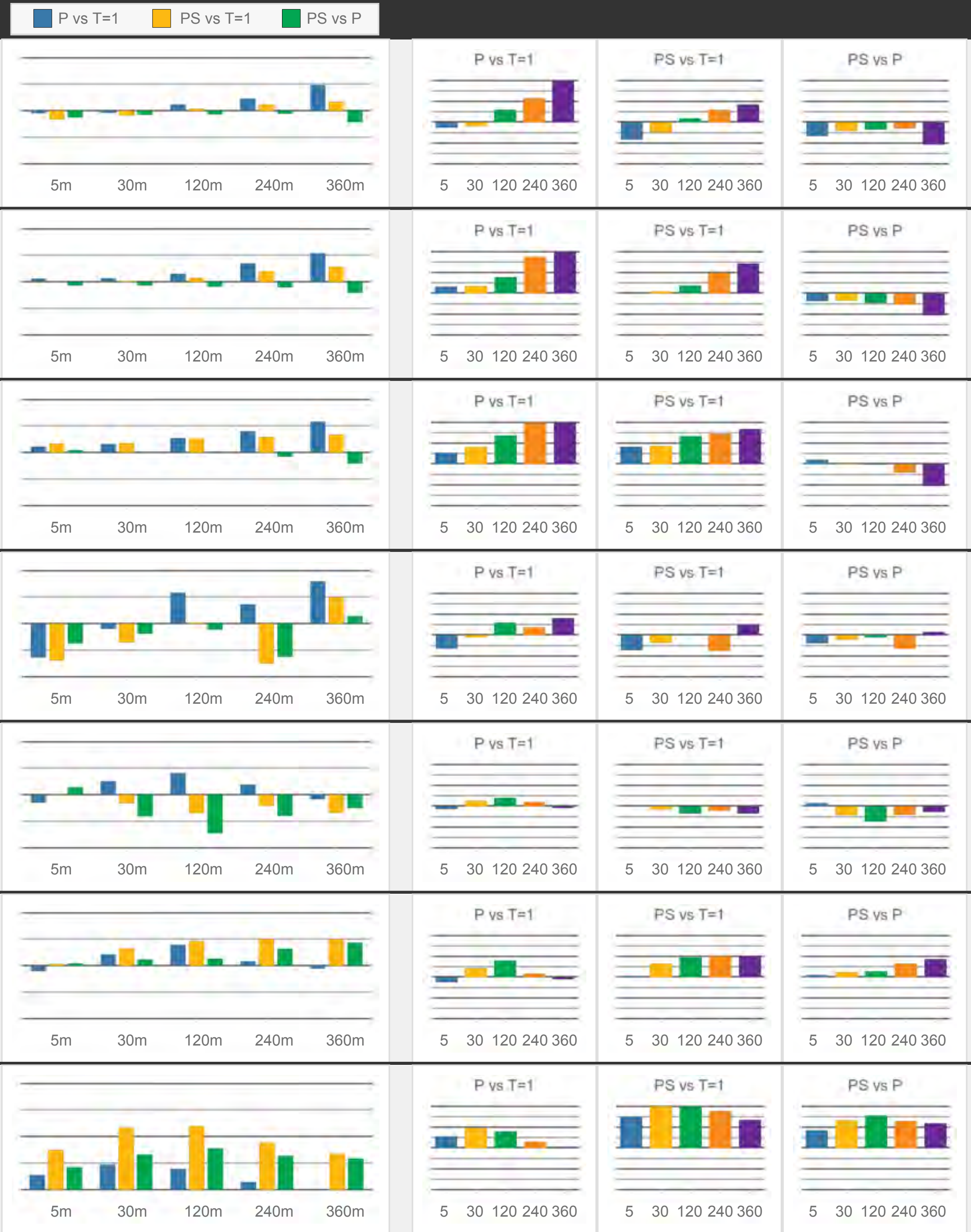

PgSg Time Course

log$_2$ (Fold Change)

Legend: P vs T=1 · PS vs T=1 · PS vs P

Locus		5m	30m	120m	240m	360m
**PGN_1688**	P vs T=1	0.327	0.717	0.506	0.001	-0.138
	PS vs T=1	1.175	1.709	1.718	1.247	0.822
	PS vs P	0.778	0.967	1.164	1.049	0.829

putative ribose 5-phosphate isomerase B

energy metabolism

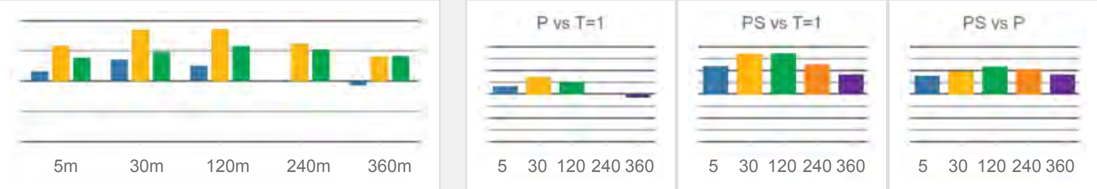

Locus		5m	30m	120m	240m	360m
**PGN_1689**	P vs T=1	0.158	0.541	0.978	1.023	0.740
	PS vs T=1	0.459	0.335	0.321	0.537	0.477
	PS vs P	0.278	-0.200	-0.628	-0.456	-0.256

transketolase

energy metabolism

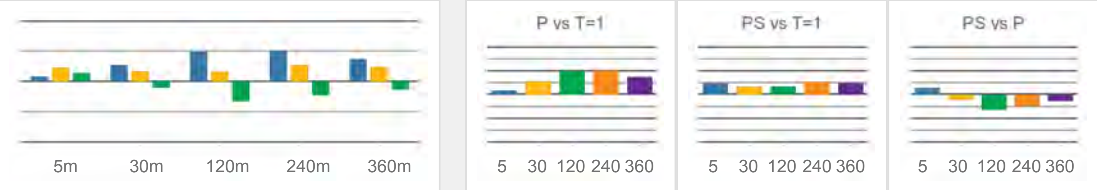

Locus		5m	30m	120m	240m	360m
**PGN_1690**	P vs T=1	-0.119	0.193	0.295	0.228	0.620
	PS vs T=1	-0.076	0.160	0.383	0.781	0.630
	PS vs P	0.017	-0.037	0.082	0.524	0.019

putative exported fucosidase

cell envelope

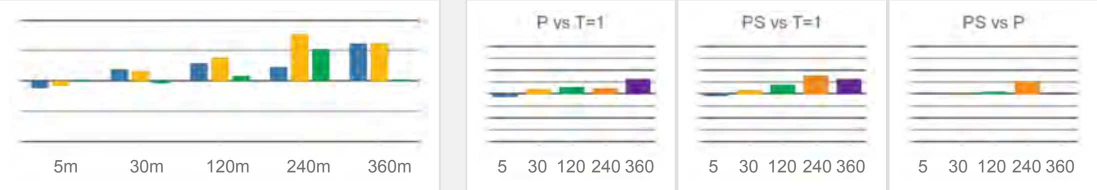

Locus		5m	30m	120m	240m	360m
**PGN_1691**	P vs T=1	-0.397	-0.641	-0.818	-1.040	-0.690
	PS vs T=1	-0.043	-0.569	-1.226	-1.088	-1.008
	PS vs P	0.362	0.064	-0.420	-0.109	-0.315

putative cysteine desulfurase

biosynthesis of cofactors, prosthetic groups, and carriers

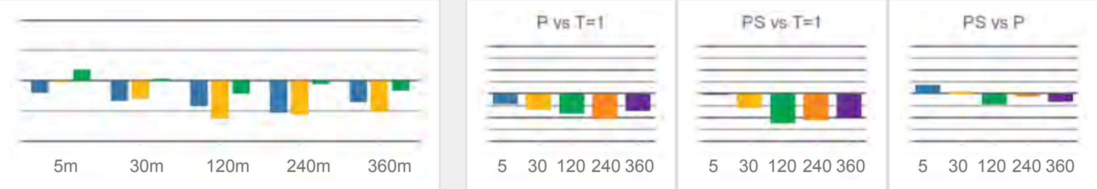

Locus		5m	30m	120m	240m	360m
**PGN_1692**	P vs T=1	-0.503	-0.829	-0.349	-0.612	0.792
	PS vs T=1	-0.406	-0.728	-0.948	-0.096	-0.650
	PS vs P	0.092	-0.056	-0.470	0.291	-0.898

conserved hypothetical protein

hypothetical proteins-Conserved

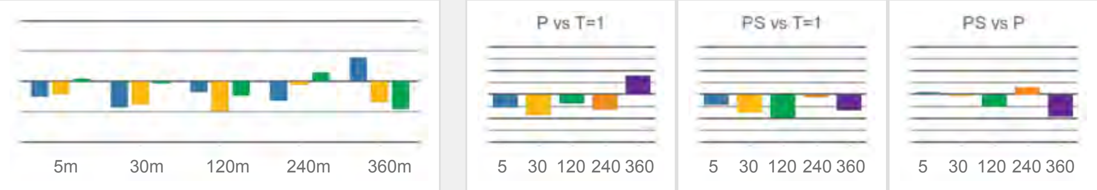

Locus		5m	30m	120m	240m	360m
**PGN_1693**	P vs T=1	-0.294	-0.155	0.130	0.290	0.264
	PS vs T=1	0.124	0.219	-0.608	-0.679	-0.783
	PS vs P	0.393	0.363	-0.687	-0.861	-0.977

selenide water dikinase

protein synthesis

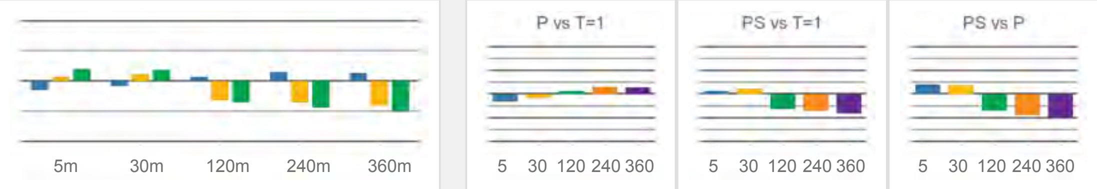

Locus		5m	30m	120m	240m	360m
**PGN_1694**	P vs T=1	0.065	0.527	1.052	0.983	0.443
	PS vs T=1	0.168	0.400	0.062	-0.233	-0.278
	PS vs P	0.086	-0.114	-0.943	-1.168	-0.712

putative alanyl dipeptidyl peptidase

hypothetical proteins-Conserved

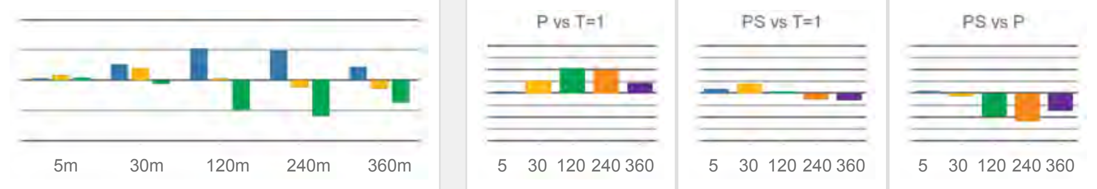

PgSg Time Course

Hackett Laboratory UW

log$_2$ (Fold Change)

Legend: P vs T=1 · PS vs T=1 · PS vs P

Locus		5m	30m	120m	240m	360m
**PGN_1695**	P vs T=1	0.425	0.995	1.149	0.589	-0.299
	PS vs T=1	-0.017	0.283	0.493	0.803	0.654
	PS vs P	-0.445	-0.656	-0.600	0.214	0.905

putative fructose-bisphosphate aldolase class I

energy metabolism

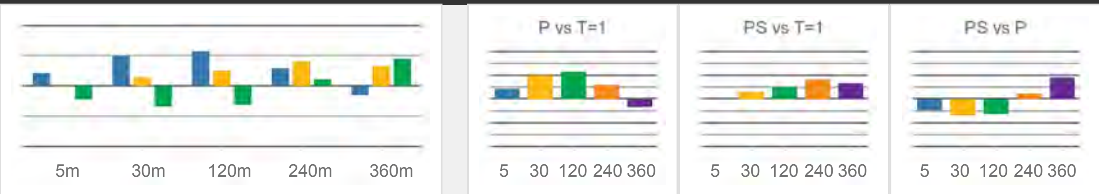

Locus		5m	30m	120m	240m	360m
**PGN_1696**	P vs T=1	0.317	0.274	0.541	0.404	1.278
	PS vs T=1	-0.473	-0.421	-0.080	0.697	0.827
	PS vs P	-0.708	-0.621	-0.494	0.261	-0.213

conserved hypothetical protein

hypothetical proteins-Conserved

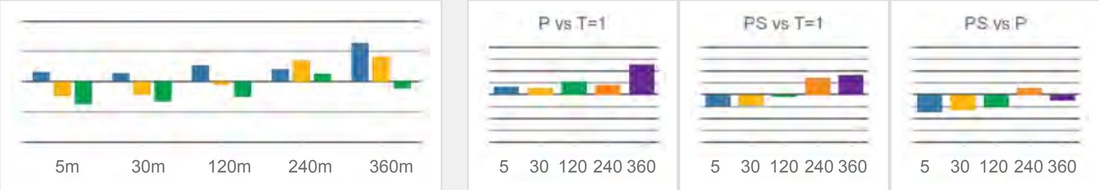

Locus		5m	30m	120m	240m	360m
**PGN_1697**	P vs T=1	-0.336	-0.305	-0.211	-0.408	-0.159
	PS vs T=1	-0.670	-0.157	0.531	0.709	0.387
	PS vs P	-0.343	0.134	0.731	1.054	0.537

conserved hypothetical protein

hypothetical proteins-Conserved

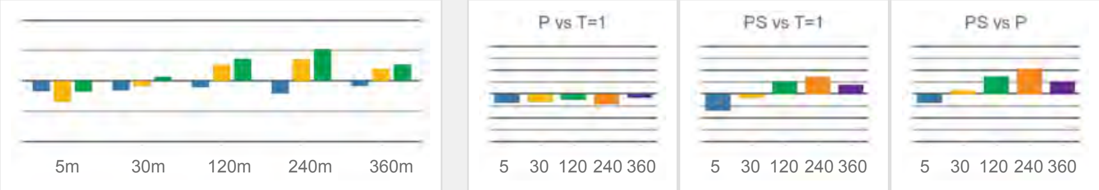

Locus		5m	30m	120m	240m	360m
**PGN_1698** *rpsO*	P vs T=1	-0.777	-0.621	-0.436	-0.582	-1.018
	PS vs T=1	-2.130	-1.538	-1.125	-1.516	-1.254
	PS vs P	-1.262	-0.806	-0.569	-0.828	-0.233

putative 30S ribosomal protein S15

protein synthesis

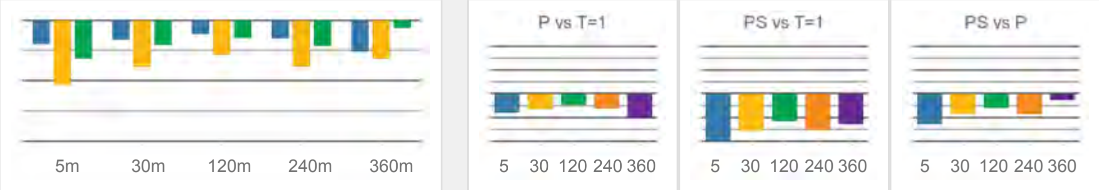

Locus		5m	30m	120m	240m	360m
**PGN_1699**	P vs T=1	0.104	-0.020	-0.413	-0.548	-0.578
	PS vs T=1	0.182	-0.287	-0.593	-0.504	-0.335
	PS vs P	0.092	-0.253	-0.191	0.013	0.223

probable zinc ABC transporter zinc-binding protein

cellular processes

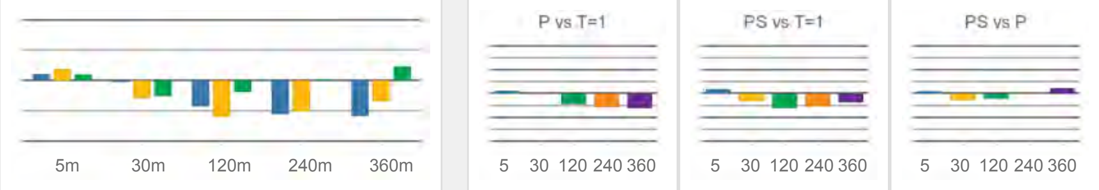

Locus		5m	30m	120m	240m	360m
**PGN_1700**	P vs T=1	-0.371	-0.152	-0.020	-0.095	-0.240
	PS vs T=1	-0.129	-0.240	-0.733	-1.001	-0.868
	PS vs P	0.221	-0.078	-0.672	-0.857	-0.617

probable metal uptake system ABC transporter ATP-binding protein

transport and binding proteins

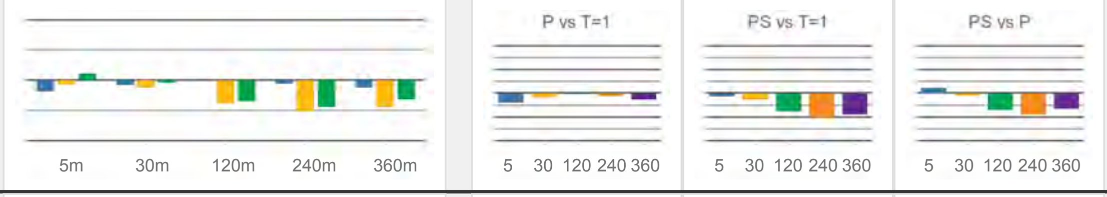

Locus		5m	30m	120m	240m	360m
**PGN_1701**	P vs T=1	-1.423	-0.949	-0.683	-0.939	-0.833
	PS vs T=1	-0.836	-0.809	-1.029	-1.231	-1.375
	PS vs P	0.508	0.128	-0.312	-0.310	-0.535

conserved hypothetical protein

hypothetical proteins-Conserved

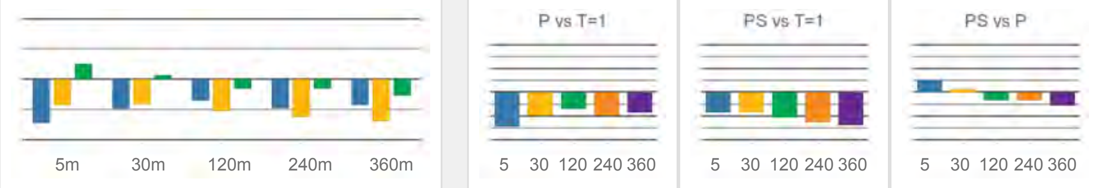

PgSg Time Course

log$_2$ (Fold Change)

Legend: P vs T=1, PS vs T=1, PS vs P

Locus		5m	30m	120m	240m	360m
**PGN_1702**	P vs T=1	-0.163	-0.376	-0.771	-1.151	-1.517
	PS vs T=1	-0.220	-0.795	-1.525	-1.726	-1.659
	PS vs P	-0.033	-0.400	-0.745	-0.581	-0.156

protein-export membrane protein SecD/SecF

protein fate

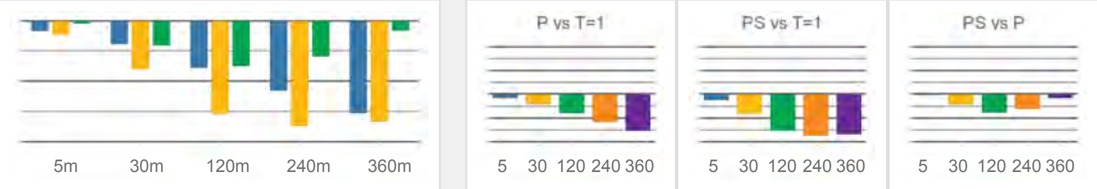

Locus		5m	30m	120m	240m	360m
**PGN_1703**	P vs T=1	0.263	0.409	0.284	-0.042	-0.419
	PS vs T=1	0.240	0.512	0.530	0.441	0.641
	PS vs P	-0.028	0.122	0.250	0.419	0.965

putative ribonuclease III

transcription

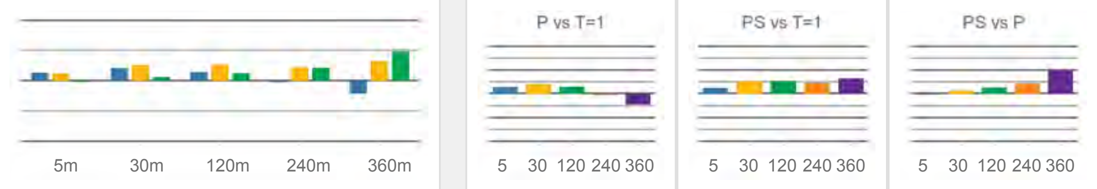

Locus		5m	30m	120m	240m	360m
**PGN_1704**	P vs T=1	1.786	2.385	2.468	2.056	1.377
	PS vs T=1	1.853	2.595	3.158	3.272	3.057
	PS vs P	0.014	0.243	0.718	1.172	1.593

beta-ketoacyl-acyl-carrier-protein synthase II

fatty acid and phospholipid metabolism

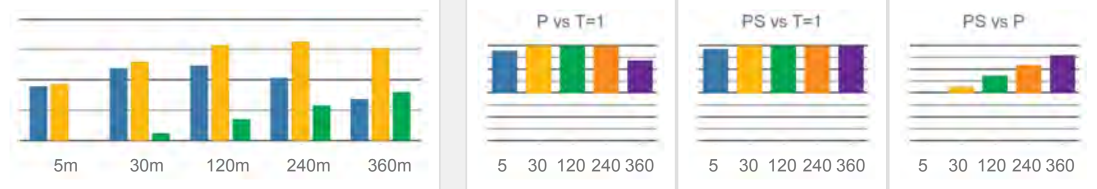

Locus		5m	30m	120m	240m	360m
**PGN_1705**	P vs T=1	1.971	2.703	2.964	2.486	1.645
	PS vs T=1	2.153	3.080	3.703	3.816	3.663
	PS vs P	0.126	0.393	0.769	1.300	1.944

putative acyl carrier protein

fatty acid and phospholipid metabolism

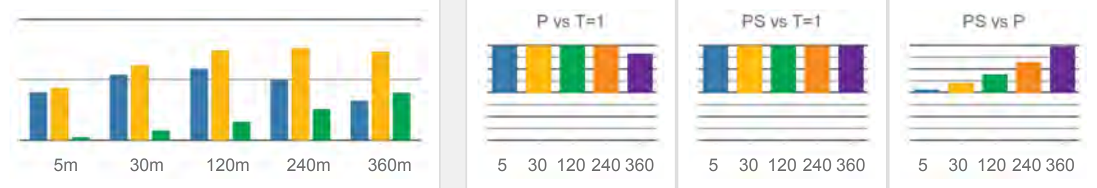

Locus		5m	30m	120m	240m	360m
**PGN_1706**	P vs T=1	-1.159	-1.787	-1.845	-1.426	-0.889
	PS vs T=1	-2.355	-1.996	-1.184	-0.708	-0.576
	PS vs P	-1.132	-0.270	0.520	0.678	0.359

probable phosphoribosylglycinamide formyltransferase

purines, pyrimidines, nucleosides and nucleotides

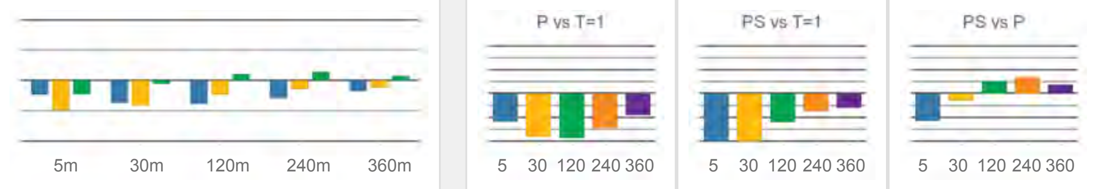

Locus		5m	30m	120m	240m	360m
**PGN_1707**	P vs T=1	-0.326	-0.645	-0.881	-0.368	0.566
	PS vs T=1	-0.951	-0.811	-0.338	0.080	0.127
	PS vs P	-0.593	-0.186	0.424	0.438	-0.347

conserved hypothetical protein

hypothetical proteins-Conserved

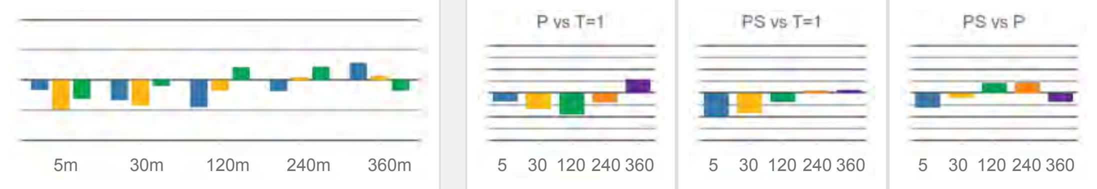

Locus		5m	30m	120m	240m	360m
**PGN_1708**	P vs T=1	-0.824	-0.745	-0.905	-0.668	0.370
	PS vs T=1	-0.472	-1.074	-1.270	-0.915	-0.566
	PS vs P	0.330	-0.326	-0.409	-0.256	-0.833

magnesium chelatase subunit ChlI

unknown function

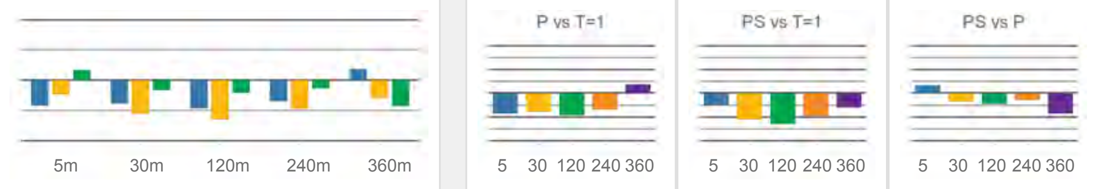

log$_2$ (Fold Change)

Legend: P vs T=1 | PS vs T=1 | PS vs P

Locus		5m	30m	120m	240m	360m
**PGN_1709**	P vs T=1	0.222	-0.059	-0.474	0.371	0.832
	PS vs T=1	0.230	-0.113	-0.079	0.085	0.038
	PS vs P	0.044	-0.088	0.135	-0.171	-0.627
	conserved hypothetical protein					
	hypothetical proteins-Conserved					
**PGN_1710**	P vs T=1	1.114	1.059	1.050	1.060	0.892
	PS vs T=1	1.450	1.446	1.888	2.202	1.860
	PS vs P	0.320	0.356	0.799	1.095	0.921
	conserved hypothetical protein					
	hypothetical proteins-Conserved					
**PGN_1711**	P vs T=1	-0.080	-0.529	-1.259	-1.229	-1.008
	PS vs T=1	0.065	-0.569	-0.782	-0.578	-0.568
	PS vs P	0.174	-0.034	0.411	0.584	0.420
	phenylalanyl-tRNA synthetase alpha subunit					
	protein synthesis					
**PGN_1712**	P vs T=1	-0.944	-1.041	-1.113	-1.246	-1.064
	PS vs T=1	-0.643	-1.209	-1.623	-1.247	-1.635
	PS vs P	0.300	-0.170	-0.509	-0.033	-0.564
	putative endonuclease III					
	DNA metabolism					
**PGN_1713**	P vs T=1	-1.269	-1.581	-1.691	-1.476	-0.744
	PS vs T=1	-0.623	-1.015	-1.290	-1.019	-0.860
	PS vs P	0.620	0.505	0.302	0.372	-0.099
	conserved hypothetical protein					
	hypothetical proteins-Conserved					
**PGN_1714**	P vs T=1	-0.384	-0.266	-0.050	0.071	0.664
	PS vs T=1	-0.042	-0.168	-0.072	0.258	0.373
	PS vs P	0.306	0.074	-0.032	0.178	-0.255
	transcription-repair coupling factor					
	DNA metabolism					
**PGN_1715** *grpE*	P vs T=1	2.296	2.738	2.865	2.773	2.552
	PS vs T=1	0.463	0.497	0.795	0.931	1.061
	PS vs P	-1.700	-2.039	-1.912	-1.697	-1.404
	putative chaperone protein GrpE					
	protein fate					

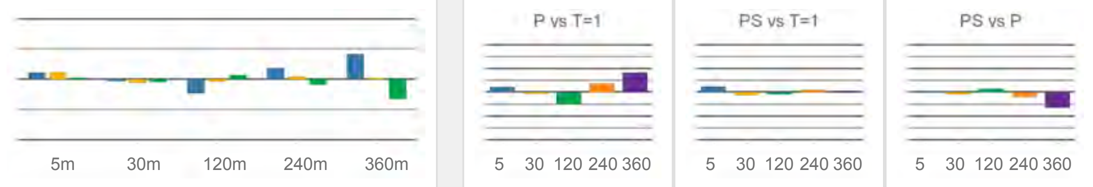

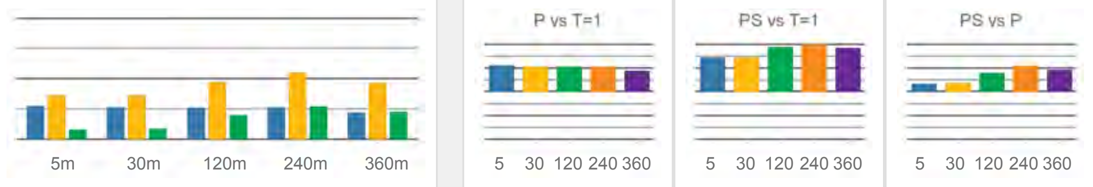

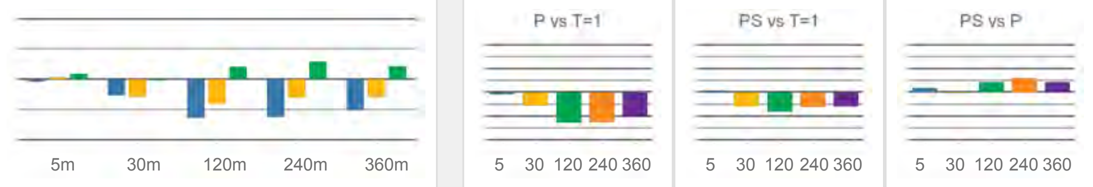

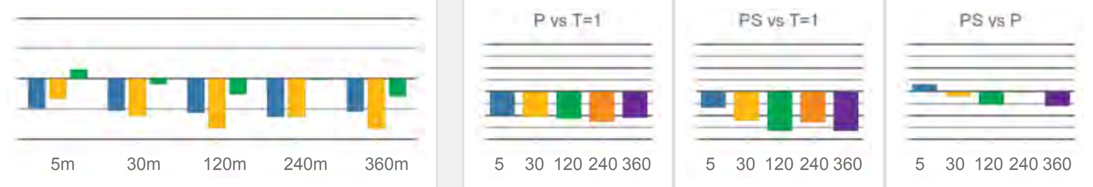

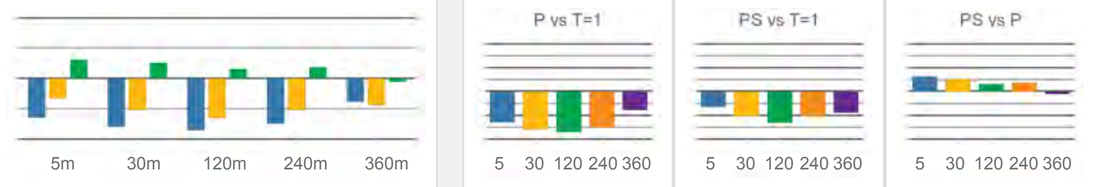

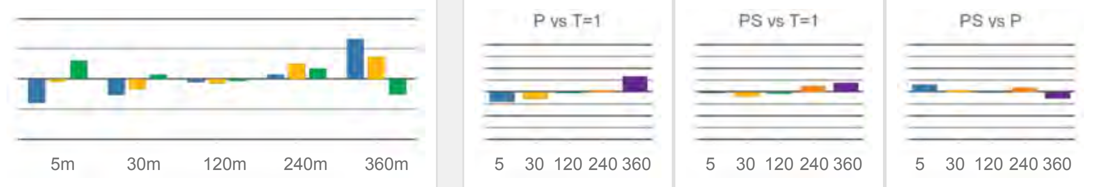

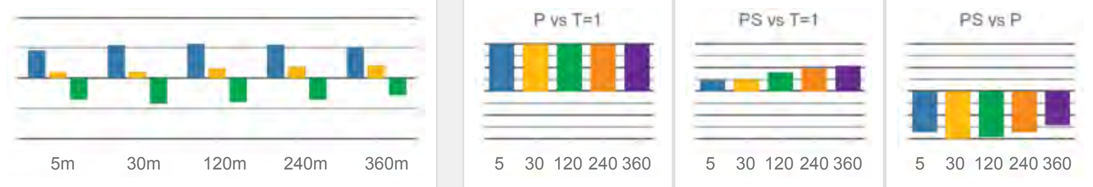

PgSg Time Course

log$_2$ (Fold Change)

Locus			5m	30m	120m	240m	360m
PGN_1716	*dnaJ*	P vs T=1	1.700	2.548	2.998	3.074	2.703
		PS vs T=1	-0.031	0.052	-0.030	0.280	0.423
		PS vs P	-1.697	-2.408	-2.943	-2.704	-2.229
		chaperone protein DnaJ					
		protein fate					
PGN_1717		P vs T=1	1.044	1.866	2.239	2.048	1.544
		PS vs T=1	-0.130	-0.050	-0.453	-0.488	-0.350
		PS vs P	-1.142	-1.803	-2.560	-2.390	-1.819
		conserved hypothetical protein					
		hypothetical proteins-Conserved					
PGN_1718		P vs T=1	0.463	0.914	1.578	1.848	1.635
		PS vs T=1	-0.325	-0.391	-0.546	-0.233	0.025
		PS vs P	-0.776	-1.246	-2.021	-1.942	-1.534
		probable UDP-2,3-diacylglucosamine hydrolase					
		hypothetical proteins-Conserved					
PGN_1719		P vs T=1	0.464	1.055	1.977	2.605	2.449
		PS vs T=1	-0.324	-0.311	0.382	0.843	1.127
		PS vs P	-0.805	-1.285	-1.452	-1.539	-1.194
		conserved hypothetical protein with appr-1-p processing enzyme domain					
		hypothetical proteins-Conserved					
PGN_1720		P vs T=1	-0.340	0.002	0.910	1.548	1.538
		PS vs T=1	-1.866	-1.566	-0.822	-0.590	-0.157
		PS vs P	-1.424	-1.347	-1.374	-1.483	-1.287
		hypothetical protein					
		hypothetical proteins					
PGN_1721	*BioF*	P vs T=1	-0.175	-0.378	-0.822	-0.792	-0.667
		PS vs T=1	0.335	0.355	0.421	0.136	-0.419
		PS vs P	0.502	0.704	1.166	0.859	0.221
		8-amino-7-oxononanoate synthase					
		biosynthesis of cofactors, prosthetic groups, and carriers					
PGN_1722		P vs T=1	-0.030	-0.374	-0.704	-0.931	-0.898
		PS vs T=1	0.086	0.079	-0.052	-0.601	-0.722
		PS vs P	0.144	0.435	0.607	0.239	0.130
		putative uridine kinase					
		purines, pyrimidines, nucleosides and nucleotides					

P vs T=1 PS vs T=1 PS vs P

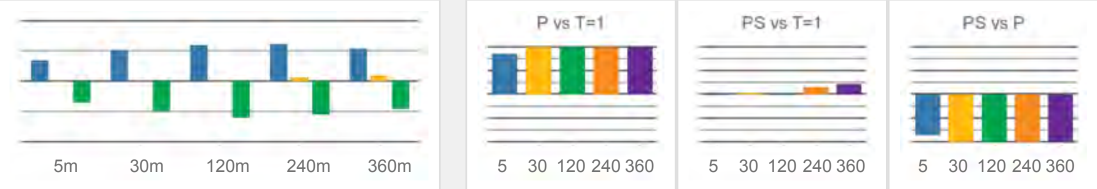

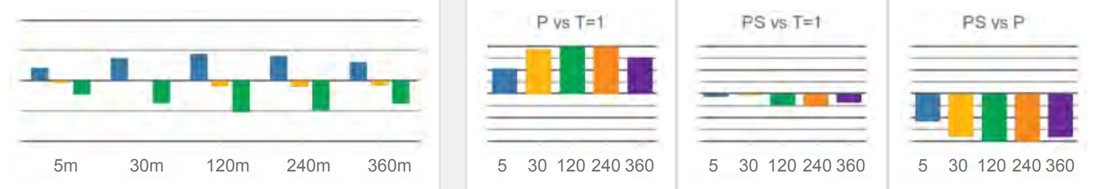

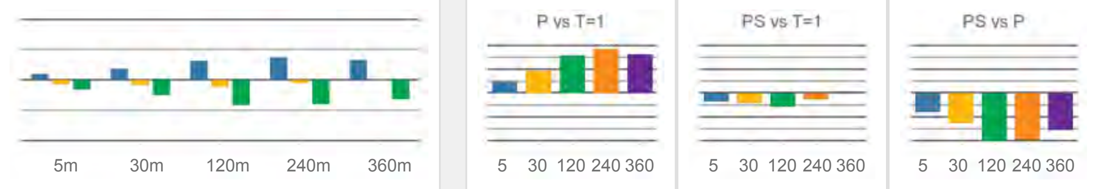

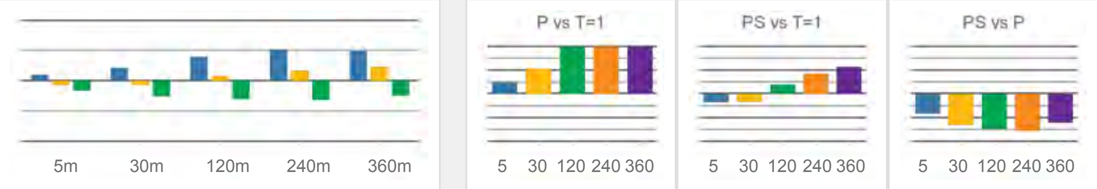

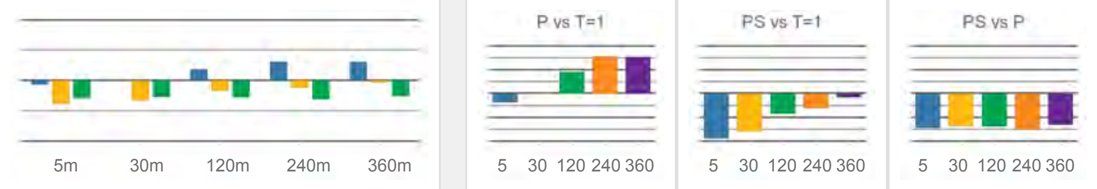

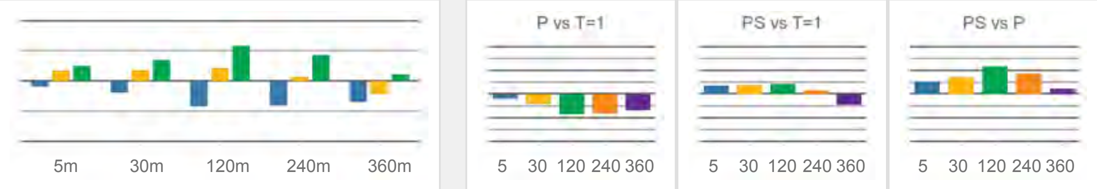

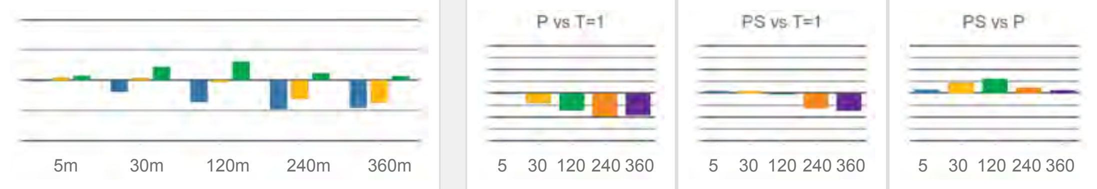

P vs T=1 | PS vs T=1 | PS vs P

log2 (Fold Change)

Locus		5m	30m	120m	240m	360m
**PGN_1723**	P vs T=1	-0.136	-0.812	-1.280	-0.904	-0.404
	PS vs T=1	-0.115	-0.796	-1.315	-1.356	-1.237
	PS vs P	0.054	0.010	-0.092	-0.440	-0.791

conserved hypothetical protein

hypothetical proteins-Conserved

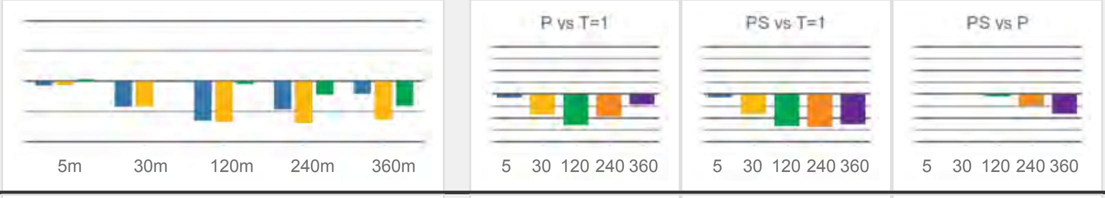

Locus		5m	30m	120m	240m	360m
**PGN_1724**	P vs T=1	-0.900	-1.752	-1.975	-1.734	-0.936
	PS vs T=1	-0.691	-1.613	-2.228	-2.572	-1.949
	PS vs P	0.271	0.075	-0.357	-0.845	-0.896

conserved hypothetical protein

cell envelope

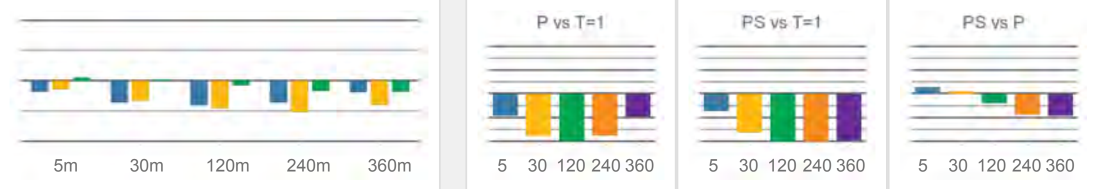

Locus		5m	30m	120m	240m	360m
**PGN_1725**	P vs T=1	-0.835	-1.743	-2.102	-1.468	-0.688
	PS vs T=1	-0.800	-1.584	-2.188	-2.264	-1.526
	PS vs P	0.064	0.088	-0.291	-0.814	-0.779

probable polysaccharide deacetylase

central intermediary metabolism

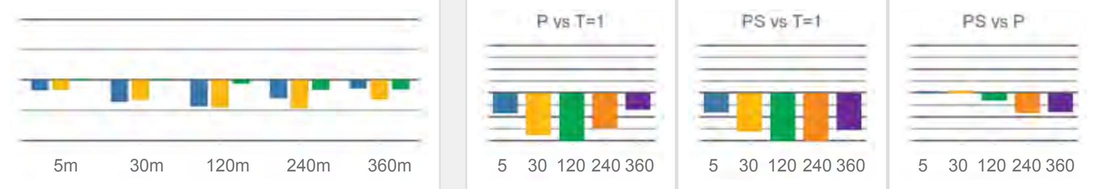

Locus		5m	30m	120m	240m	360m
**PGN_1726**	P vs T=1	0.351	-0.259	-0.197	0.541	1.706
	PS vs T=1	-0.029	0.131	0.416	0.479	0.744
	PS vs P	-0.345	0.266	0.438	-0.033	-0.798

transposase in ISPg3

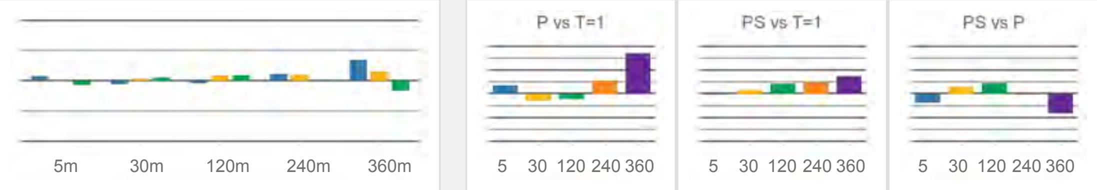

Locus		5m	30m	120m	240m	360m
**PGN_1727**	P vs T=1	0.398	0.298	0.305	0.346	-0.082
	PS vs T=1	0.157	-0.086	0.033	0.128	0.141
	PS vs P	-0.234	-0.384	-0.276	-0.218	0.200

transposase in ISPg1

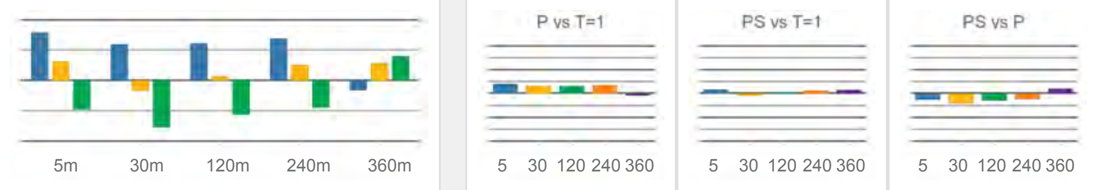

Locus		5m	30m	120m	240m	360m
**PGN_1728** *kgp*	P vs T=1	1.285	1.846	1.957	1.323	0.398
	PS vs T=1	1.593	1.859	1.294	0.578	0.187
	PS vs P	0.303	0.045	-0.621	-0.733	-0.229

lysine-specific cysteine proteinase Kgp

protein fate

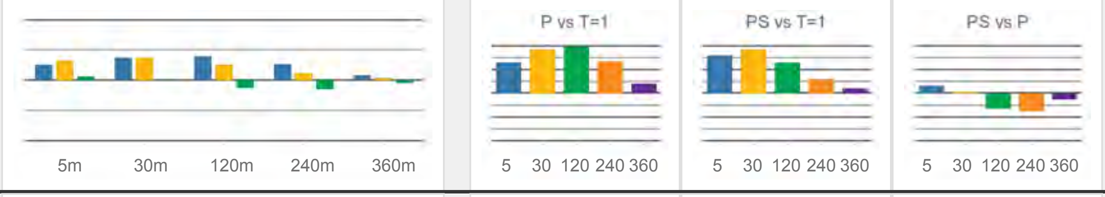

Locus		5m	30m	120m	240m	360m
**PGN_1729**	P vs T=1	0.395	-0.075	0.434	1.043	2.221
	PS vs T=1	-0.406	-0.218	1.342	1.022	0.845
	PS vs P	-0.765	-0.216	0.868	0.074	-1.203

probable acetyltransferase

unknown function

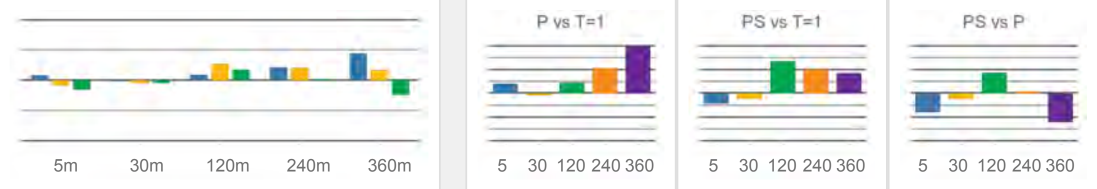

PgSg Time Course

log$_2$ (Fold Change)

Legend: P vs T=1 | PS vs T=1 | PS vs P

Locus		5m	30m	120m	240m	360m
PGN_1730	P vs T=1	-0.008	-0.114	0.228	0.560	0.966
	PS vs T=1	-1.171	0.436	1.752	1.254	0.822
	PS vs P	-1.155	0.509	1.496	0.707	-0.103

conserved hypothetical protein

hypothetical proteins-Conserved

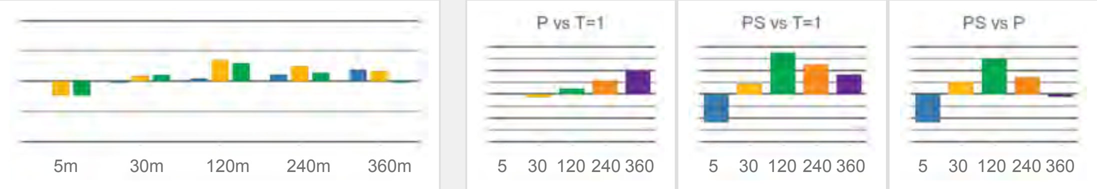

Locus		5m	30m	120m	240m	360m
PGN_1731	P vs T=1	-0.307	-0.686	-0.710	0.490	1.302
	PS vs T=1	-0.334	-0.468	-0.394	-0.917	-0.352
	PS vs P	-0.025	-0.053	-0.084	-0.603	-0.583

conserved hypothetical protein

hypothetical proteins-Conserved

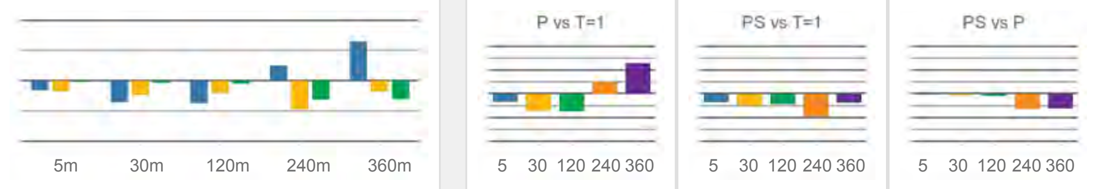

Locus		5m	30m	120m	240m	360m
PGN_1732	P vs T=1	-1.018	-0.105	1.149	1.775	1.767
	PS vs T=1	-0.935	-0.702	-0.499	-0.240	0.992
	PS vs P	-0.407	-0.596	-1.152	-1.145	-0.356

hypothetical protein

hypothetical proteins

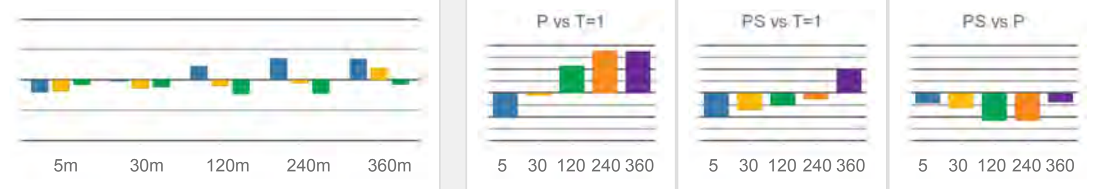

Locus		5m	30m	120m	240m	360m
PGN_1733	P vs T=1	0.862	1.604	2.134	1.730	0.910
hagA	PS vs T=1	1.123	1.409	1.243	1.112	0.815
	PS vs P	0.223	-0.173	-0.837	-0.595	-0.113

hemagglutinin protein HagA

cellular processes

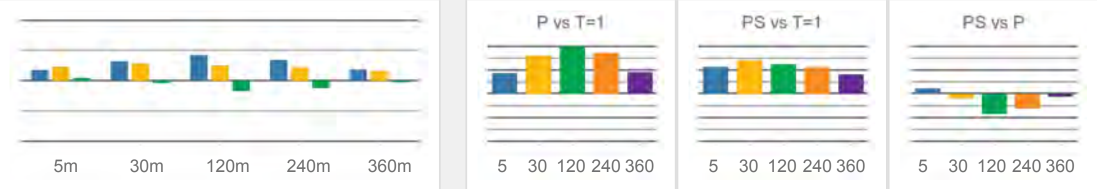

Locus		5m	30m	120m	240m	360m
PGN_1734	P vs T=1	0.638	0.261	-0.214	-0.004	0.737
	PS vs T=1	0.841	0.308	-0.955	-0.630	-0.510
	PS vs P	0.264	0.066	-0.778	-0.602	-1.129

nucleoside permease NupG

transport and binding proteins

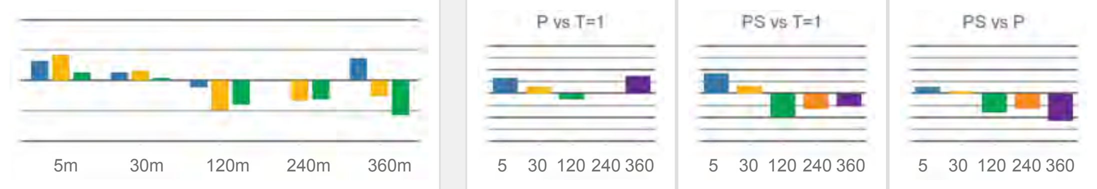

Locus		5m	30m	120m	240m	360m
PGN_1735	P vs T=1	1.268	0.853	0.195	-0.302	-0.829
	PS vs T=1	1.035	0.645	-0.074	-0.426	-0.587
	PS vs P	-0.205	-0.192	-0.266	-0.138	0.219

conserved hypothetical protein

hypothetical proteins-Conserved

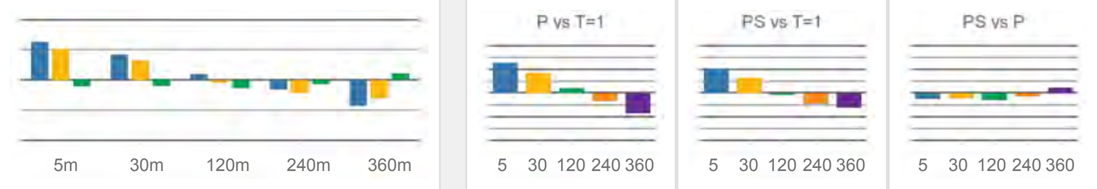

Locus		5m	30m	120m	240m	360m
PGN_1736	P vs T=1	0.315	-0.667	-1.883	-2.235	-2.327
	PS vs T=1	-0.600	-1.308	-1.970	-2.163	-2.291
	PS vs P	-0.763	-0.585	-0.149	-0.024	-0.031

putative glycogen synthase

unknown function

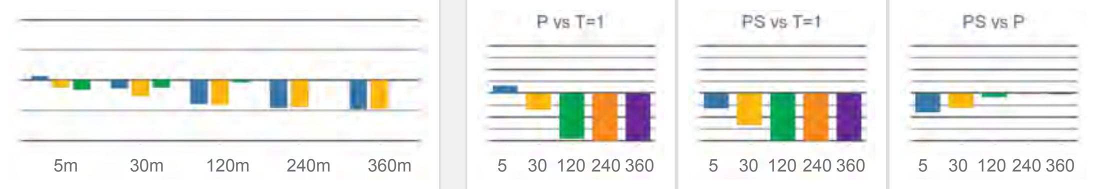

PgSg Time Course

log$_2$ (Fold Change)

Legend: P vs T=1 | PS vs T=1 | PS vs P

Locus		5m	30m	120m	240m	360m
**PGN_1737**	P vs T=1	-0.495	-0.168	-0.100	0.170	0.464
recQII	PS vs T=1	-0.191	-0.059	-0.149	-0.076	-0.073
	PS vs P	0.283	0.104	-0.057	-0.231	-0.516

ATP-dependent DNA helicase RecQ

DNA metabolism

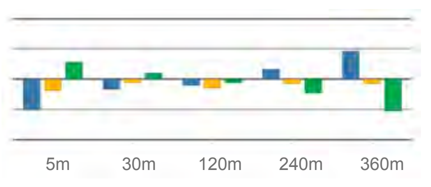

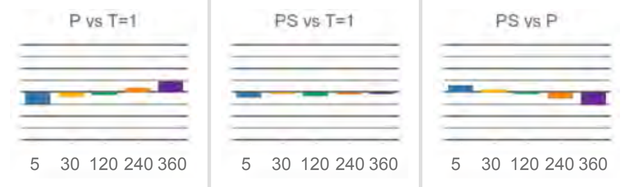

Locus		5m	30m	120m	240m	360m
**PGN_1738**	P vs T=1	0.543	0.707	0.485	0.610	1.181
	PS vs T=1	1.029	1.840	2.062	1.592	1.619
	PS vs P	0.414	1.071	1.466	0.883	0.444

putative long-chain-fatty-acid-CoA ligase

fatty acid and phospholipid metabolism

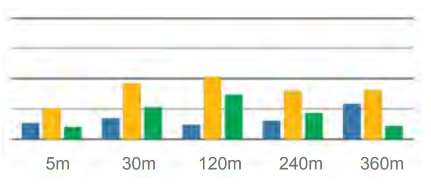

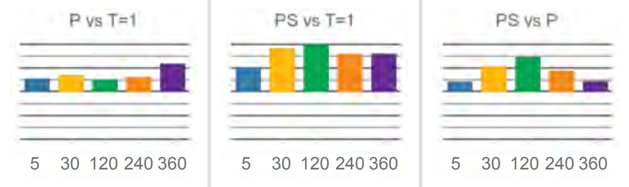

Locus		5m	30m	120m	240m	360m
**PGN_1739**	P vs T=1	-0.799	-0.503	-0.166	-0.423	-1.197
	PS vs T=1	-1.266	-0.897	-0.370	-0.282	-0.170
	PS vs P	-0.483	-0.381	-0.171	0.144	0.979

conserved hypothetical protein

hypothetical proteins-Conserved

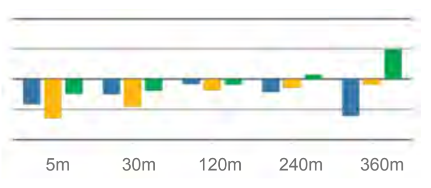

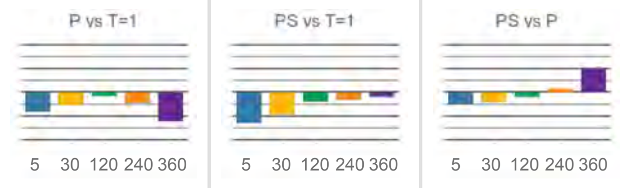

Locus		5m	30m	120m	240m	360m
**PGN_1740**	P vs T=1	4.145	4.160	4.313	4.132	3.236
	PS vs T=1	3.189	3.686	3.386	2.948	2.959
	PS vs P	-0.833	-0.371	-0.815	-1.087	-0.275

putative RNA polymerase ECF-type sigma factor

transcription

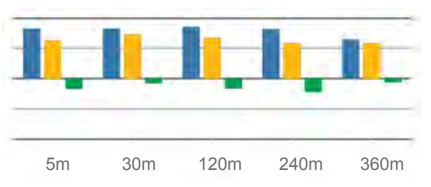

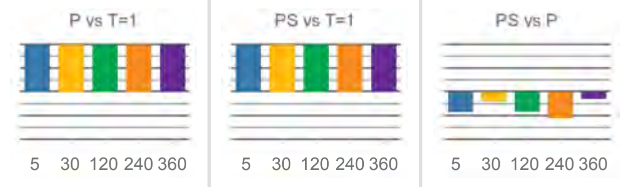

Locus		5m	30m	120m	240m	360m
**PGN_1741**	P vs T=1	0.237	0.229	0.180	0.348	0.585
	PS vs T=1	0.593	0.637	1.116	1.340	1.112
	PS vs P	0.322	0.368	0.865	0.933	0.515

conserved hypothetical protein

hypothetical proteins-Conserved

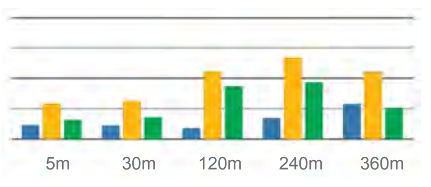

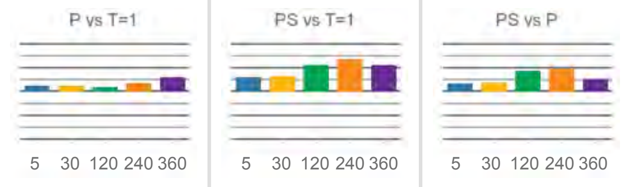

Locus		5m	30m	120m	240m	360m
**PGN_1742**	P vs T=1	0.162	0.145	0.163	0.037	0.315
	PS vs T=1	0.494	0.507	0.644	0.678	0.624
	PS vs P	0.276	0.293	0.408	0.479	0.281

conserved hypothetical protein

hypothetical proteins-Conserved

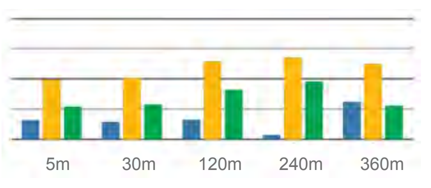

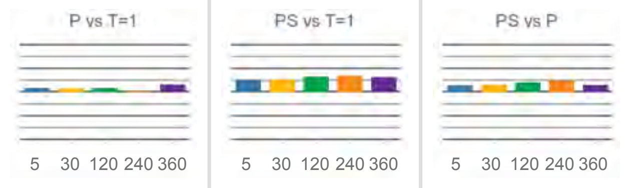

Locus		5m	30m	120m	240m	360m
**PGN_1743**	P vs T=1	1.880	1.976	2.011	1.906	1.300
	PS vs T=1	1.874	1.837	1.461	1.208	1.180
	PS vs P	0.017	-0.108	-0.514	-0.664	-0.129

phosphopyruvate hydratase

energy metabolism

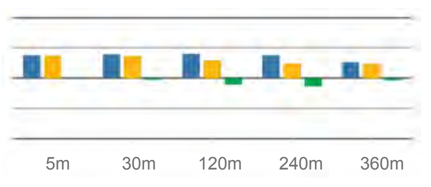

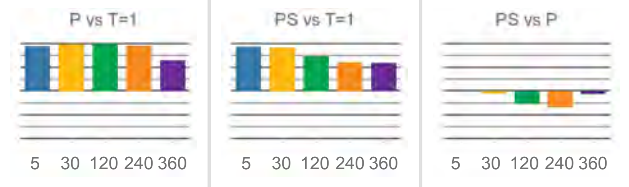

log2 (Fold Change)

Legend: P vs T=1 | PS vs T=1 | PS vs P

Locus		5m	30m	120m	240m	360m
**PGN_1744**	P vs T=1	-0.087	0.168	0.088	-0.272	-0.964
	PS vs T=1	-0.440	0.158	0.235	-0.072	-0.179
	PS vs P	-0.350	0.017	0.167	0.188	0.730

conserved hypothetical protein

hypothetical proteins-Conserved

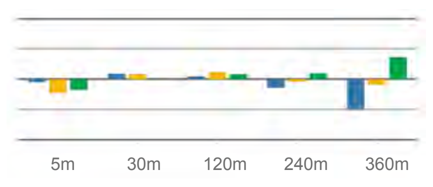

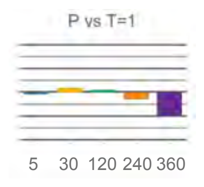

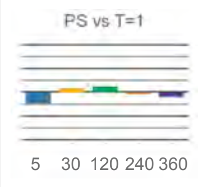

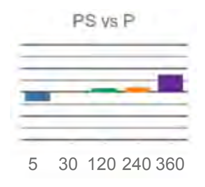

Locus		5m	30m	120m	240m	360m
**PGN_1745**	P vs T=1	-0.946	-1.755	-2.426	-2.329	-2.811
	PS vs T=1	-0.827	-1.311	-1.680	-2.018	-2.176
	PS vs P	0.155	0.409	0.652	0.244	0.534

putative NapC/NirT cytochrome c-type protein

energy metabolism

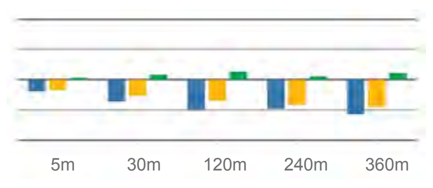

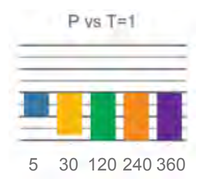

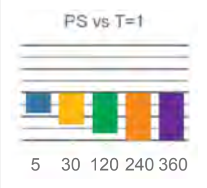

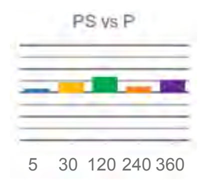

Locus		5m	30m	120m	240m	360m
**PGN_1746**	P vs T=1	0.361	0.446	0.682	0.611	0.441
	PS vs T=1	1.011	1.033	0.899	0.692	0.687
	PS vs P	0.614	0.555	0.212	0.067	0.225

cytochrome c nitrite reductase catalytic subunit NrfA

energy metabolism

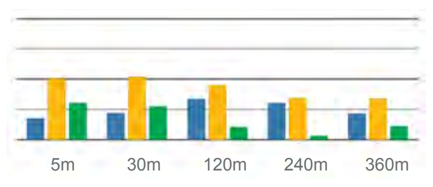

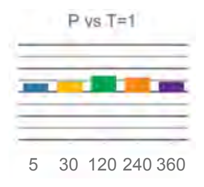

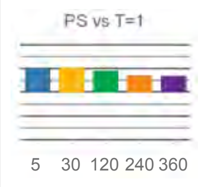

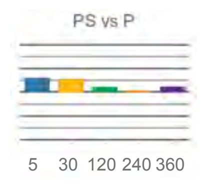

Locus		5m	30m	120m	240m	360m
**PGN_1747**	P vs T=1	0.147	-0.249	-0.538	-0.384	0.254
	PS vs T=1	0.387	0.075	-0.158	-0.384	-0.229
	PS vs P	0.253	0.300	0.316	-0.041	-0.448

conserved hypothetical protein

hypothetical proteins-Conserved

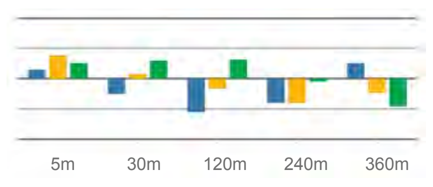

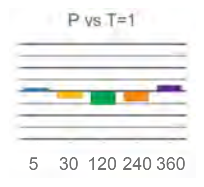

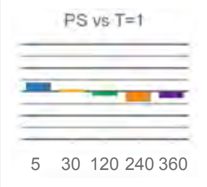

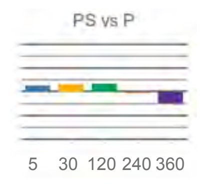

Locus		5m	30m	120m	240m	360m
**PGN_1748**	P vs T=1	-0.190	-0.744	-1.053	-0.744	0.249
	PS vs T=1	0.211	0.171	-0.493	-0.570	-0.580
	PS vs P	0.416	0.830	0.410	0.101	-0.732

putative cytochrome c biogenesis protein CcsA

energy metabolism

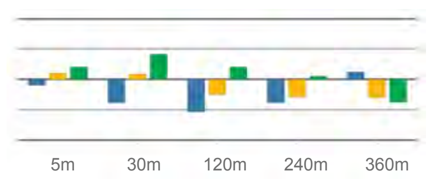

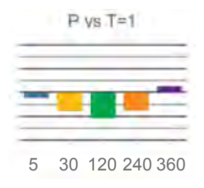

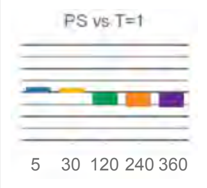

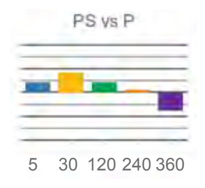

Locus		5m	30m	120m	240m	360m
**PGN_1749**	P vs T=1	0.004	0.362	0.424	0.225	-0.295
	PS vs T=1	0.433	0.517	0.634	0.895	0.709
	PS vs P	0.405	0.157	0.216	0.650	0.948

probable NADPH-quinone reductase

central intermediary metabolism

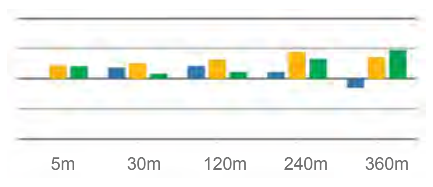

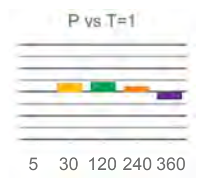

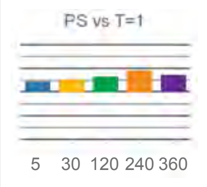

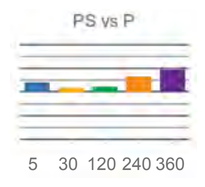

Locus		5m	30m	120m	240m	360m
**PGN_1750**	P vs T=1	-0.154	0.211	0.077	-0.331	-0.036
	PS vs T=1	0.472	0.181	-0.039	0.272	0.049
	PS vs P	0.587	-0.014	-0.110	0.515	0.077

putative 3-deoxy-D-manno-octulosonate cytidylyltransferase

cell envelope

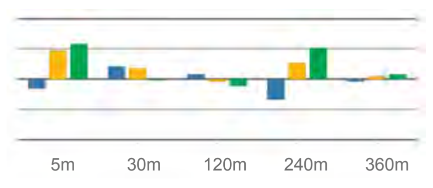

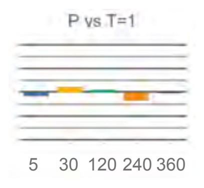

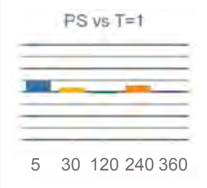

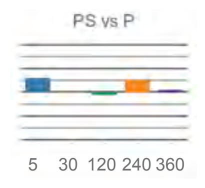

log₂ (Fold Change)

Legend: P vs T=1 · PS vs T=1 · PS vs P

Locus		5m	30m	120m	240m	360m
**PGN_1751**	P vs T=1	-0.793	-0.696	-1.121	-1.004	-0.818
	PS vs T=1	-0.019	-0.180	-0.515	-0.758	-0.939
	PS vs P	0.749	0.506	0.559	0.216	-0.127
	DNA primase					
	DNA metabolism					
**PGN_1752**	P vs T=1	0.330	0.737	1.085	0.764	-0.183
	PS vs T=1	0.386	0.880	1.528	2.058	2.015
	PS vs P	-0.009	0.142	0.485	1.260	2.063
	putative ferredoxin 4Fe-4S					
	energy metabolism					
**PGN_1753**	P vs T=1	0.511	0.826	0.993	0.618	0.137
	PS vs T=1	0.911	1.053	1.032	1.226	1.041
	PS vs P	0.368	0.230	0.059	0.587	0.863
	putative 2-oxoglutarate oxidoreductase alpha subunit					
	energy metabolism					
**PGN_1754**	P vs T=1	1.693	2.202	2.514	2.210	1.588
	PS vs T=1	2.061	2.542	2.727	2.413	2.116
	PS vs P	0.305	0.365	0.288	0.222	0.477
	hypothetical protein					
	hypothetical proteins					
**PGN_1755**	P vs T=1	1.582	2.179	2.377	1.823	0.934
	PS vs T=1	1.808	2.333	2.430	1.845	1.419
	PS vs P	0.195	0.210	0.136	0.034	0.424
	putative 2-oxoglutarate oxidoreductase beta subunit					
	energy metabolism					
**PGN_1756**	P vs T=1	0.936	1.827	2.600	2.349	1.294
	PS vs T=1	1.142	1.911	2.249	1.678	1.320
	PS vs P	0.178	0.096	-0.312	-0.643	0.019
	putative 2-oxoglutarate oxidoreductase gamma subunit					
	energy metabolism					
**PGN_1757**	P vs T=1	1.205	0.985	0.584	0.278	0.006
	PS vs T=1	1.331	0.663	0.209	0.124	-0.050
	PS vs P	0.146	-0.308	-0.373	-0.165	-0.070
	GTP pyrophosphokinase					
	cellular processes					

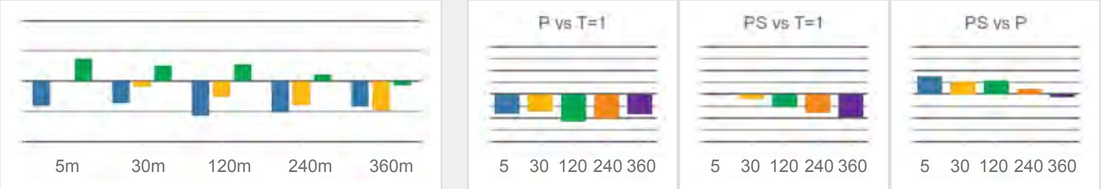

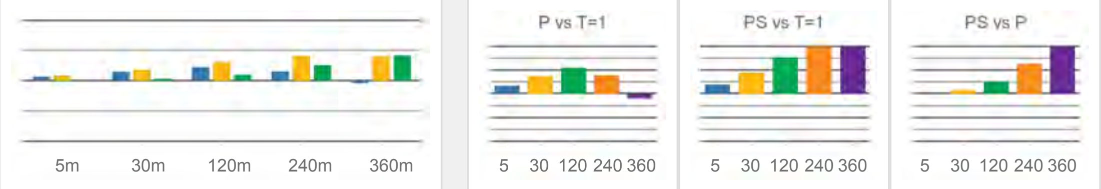

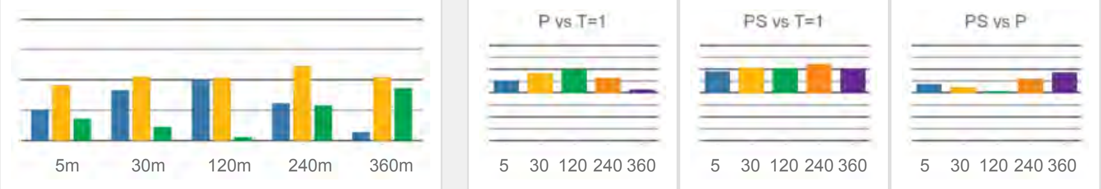

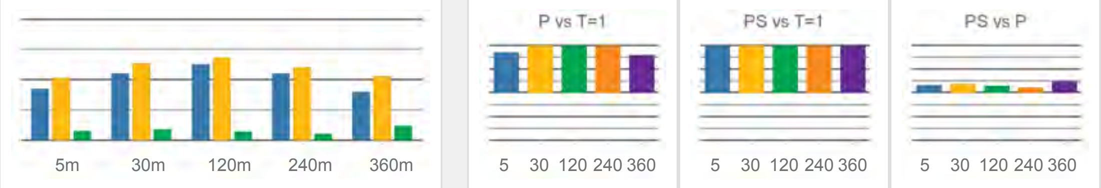

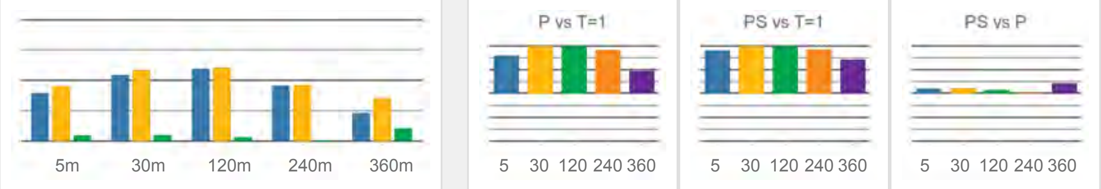

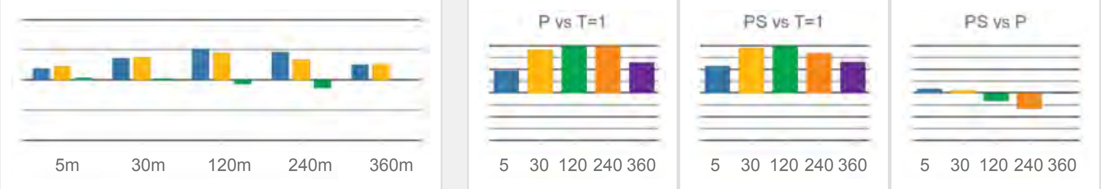

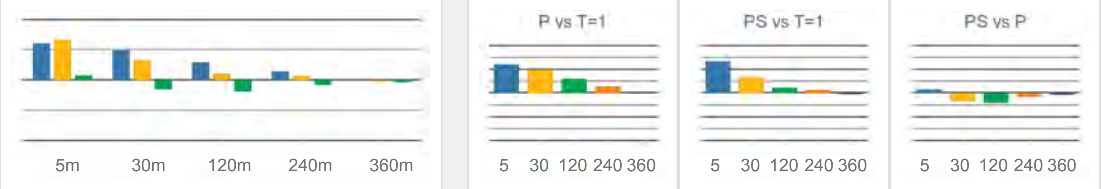

PgSg Time Course

log2 (Fold Change)

Legend: P vs T=1 | PS vs T=1 | PS vs P

Locus		5m	30m	120m	240m	360m
**PGN_1758**	P vs T=1	-0.525	0.066	0.505	0.173	-0.555
	PS vs T=1	-0.946	-0.240	-0.126	-0.714	-0.964
	PS vs P	-0.453	-0.231	-0.491	-0.797	-0.427
	putative v-type ATPase subunit K					
	transport and binding proteins					
**PGN_1759**	P vs T=1	0.863	1.253	1.105	0.488	-0.253
	PS vs T=1	0.855	1.001	0.334	-0.652	-0.756
	PS vs P	0.008	-0.204	-0.722	-1.120	-0.521
	putative v-type ATPase subunit I					
	transport and binding proteins					
**PGN_1760**	P vs T=1	1.023	1.142	0.987	0.464	0.039
	PS vs T=1	0.882	0.640	-0.279	-0.450	-0.204
	PS vs P	-0.095	-0.436	-1.194	-0.887	-0.257
	putative v-type ATPase subunit D					
	transport and binding proteins					
**PGN_1761**	P vs T=1	0.257	0.597	0.679	0.349	0.243
	PS vs T=1	0.381	0.011	-0.624	-0.542	-0.627
	PS vs P	0.132	-0.508	-1.204	-0.839	-0.835
	v-type ATPase subunit B					
	transport and binding proteins					
**PGN_1762**	P vs T=1	-0.088	-0.114	-0.650	-0.889	-0.653
	PS vs T=1	-0.744	-1.273	-1.799	-1.589	-1.249
	PS vs P	-0.574	-1.058	-1.113	-0.692	-0.565
	v-type ATPase subunit A					
	transport and binding proteins					
**PGN_1763**	P vs T=1	-0.425	-0.808	-1.181	-1.185	-0.531
	PS vs T=1	-1.652	-1.913	-1.944	-1.521	-1.170
	PS vs P	-1.114	-1.030	-0.759	-0.343	-0.559
	conserved hypothetical protein					
	hypothetical proteins-Conserved					
**PGN_1764**	P vs T=1	0.615	-0.025	-0.876	-0.728	-0.281
	PS vs T=1	-1.033	-0.966	-0.841	-0.651	-0.527
	PS vs P	-1.564	-0.895	-0.013	0.057	-0.219
	putative v-type ATPase subunit E					
	transport and binding proteins					

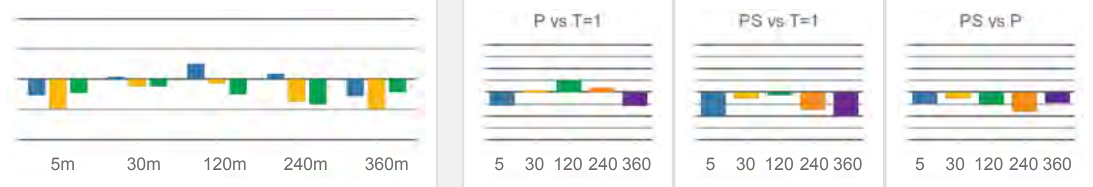

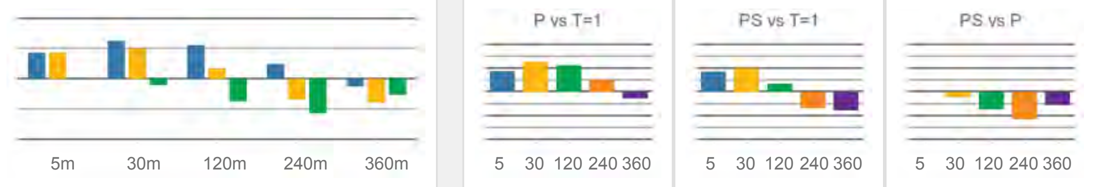

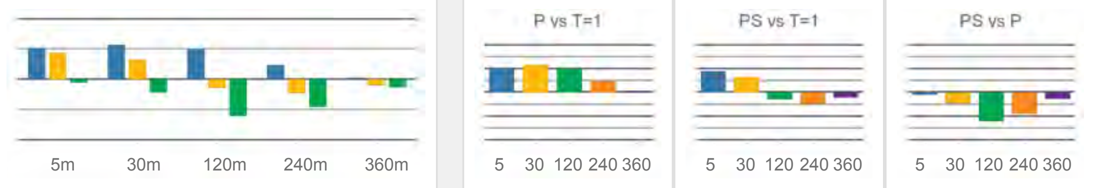

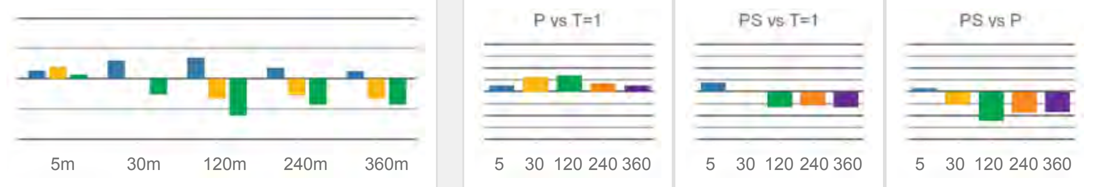

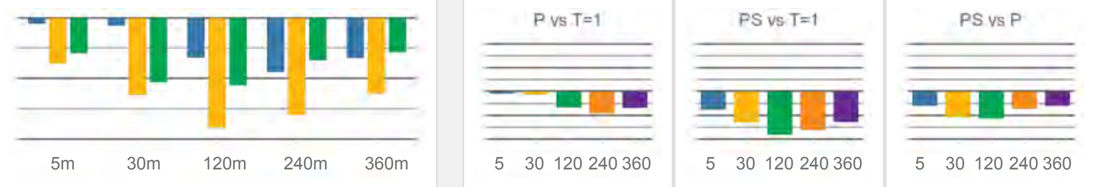

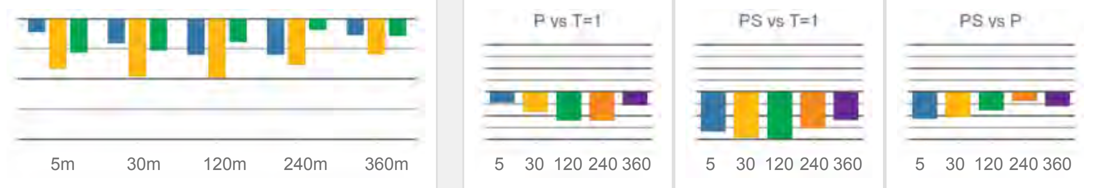

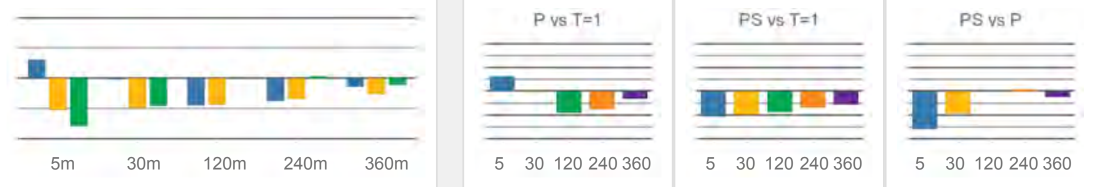

PgSg Time Course

log$_2$ (Fold Change)

Legend: P vs T=1 | PS vs T=1 | PS vs P

Locus		5m	30m	120m	240m	360m
**PGN_1765**	P vs T=1	0.402	0.384	1.550	2.595	3.992
	PS vs T=1	-0.246	0.056	2.121	3.082	2.155
	PS vs P	-0.660	-0.480	0.625	1.047	-0.605

hypothetical protein

hypothetical proteins

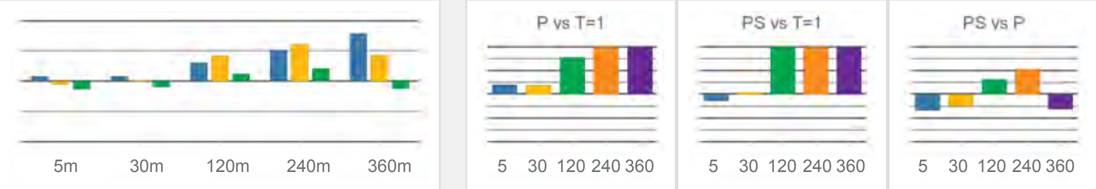

Locus		5m	30m	120m	240m	360m
**PGN_1766**	P vs T=1	-0.412	-0.190	-0.293	1.290	2.753
	PS vs T=1	-0.648	-0.233	0.720	0.987	1.264
	PS vs P	-0.457	-0.197	0.373	0.158	-0.632

conserved hypothetical protein

hypothetical proteins-Conserved

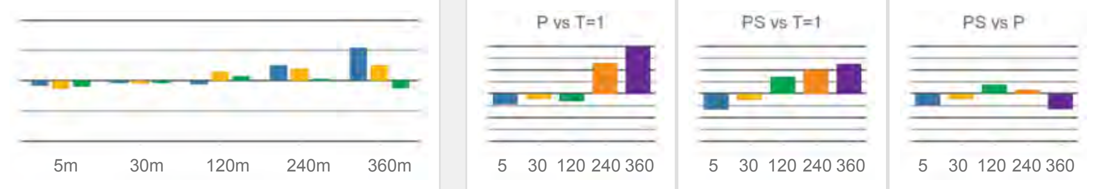

Locus		5m	30m	120m	240m	360m
**PGN_1767**	P vs T=1	-0.419	-0.217	-0.026	-0.387	-1.248
	PS vs T=1	-0.872	-0.469	-0.253	-0.606	-0.800
	PS vs P	-0.454	-0.237	-0.197	-0.217	0.400

immunoreactive 46 kDa antigen

cell envelope

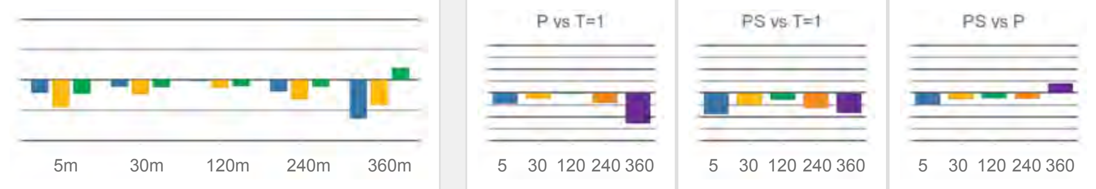

Locus		5m	30m	120m	240m	360m
**PGN_1768**	P vs T=1	-0.217	-0.841	-1.287	-1.366	-1.288
	PS vs T=1	-0.785	-0.941	-0.561	-0.453	-0.858
	PS vs P	-0.517	-0.109	0.680	0.860	0.402

putative DNA-binding response regulator/sensor histidine kinase

signal transduction

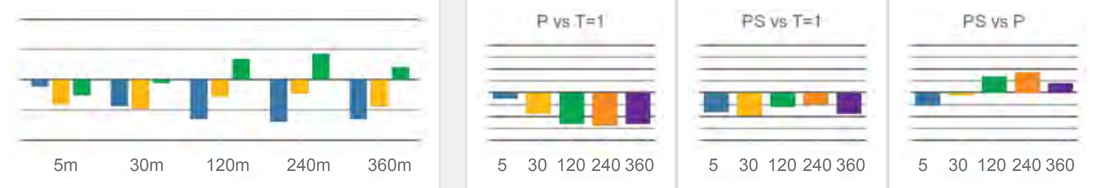

Locus		5m	30m	120m	240m	360m
**PGN_1769**	P vs T=1	1.018	0.813	0.430	-0.316	-1.447
	PS vs T=1	0.285	0.299	-0.202	-0.706	-1.144
	PS vs P	-0.460	-0.293	-0.459	-0.366	0.126

hypothetical protein

hypothetical proteins

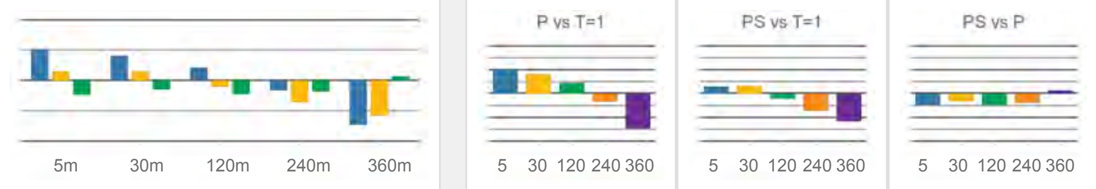

Locus		5m	30m	120m	240m	360m
**PGN_1770**	P vs T=1	-0.868	-0.760	-0.877	-1.392	-2.228
	PS vs T=1	-1.550	-1.220	-1.384	-1.708	-1.753
	PS vs P	-0.658	-0.426	-0.479	-0.322	0.431

conserved hypothetical protein

hypothetical proteins-Conserved

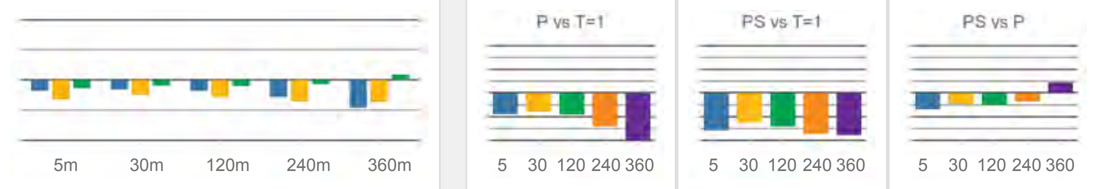

Locus		5m	30m	120m	240m	360m
**PGN_1771**	P vs T=1	-0.065	0.307	0.317	0.190	0.096
	PS vs T=1	0.613	0.793	0.240	0.117	-0.034
	PS vs P	0.649	0.483	-0.074	-0.078	-0.137

DNA polymerase I

DNA metabolism

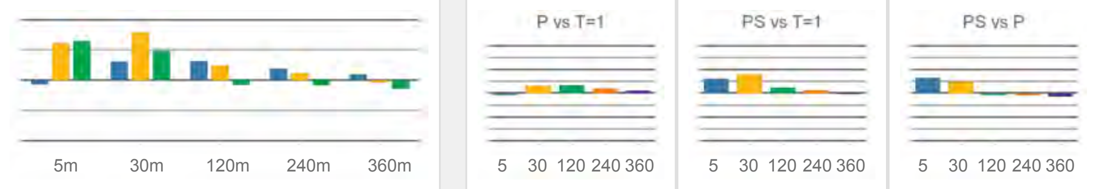

PgSg Time Course

Hackett Laboratory UW

log$_2$ (Fold Change)

Legend: P vs T=1 | PS vs T=1 | PS vs P

Locus		5m	30m	120m	240m	360m
**PGN_1772**	P vs T=1	0.188	0.696	0.710	0.253	0.101
	PS vs T=1	0.567	0.572	-0.096	-0.463	-0.685
	PS vs P	0.366	-0.106	-0.780	-0.714	-0.786

1,4-alpha-glucan branching enzyme

energy metabolism

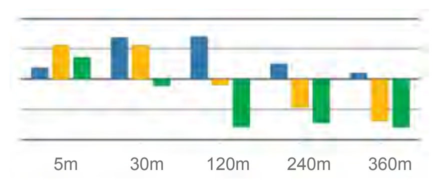

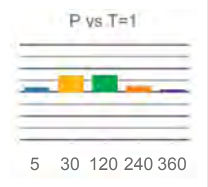

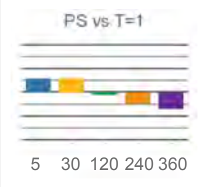

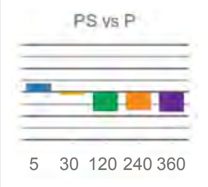

Locus		5m	30m	120m	240m	360m
**PGN_1773**	P vs T=1	-0.365	-0.312	-0.749	-1.088	-0.883
	PS vs T=1	-0.446	-0.778	-1.318	-1.105	-1.228
	PS vs P	-0.073	-0.450	-0.571	-0.043	-0.349

sodium/hydrogen antiporter

transport and binding proteins

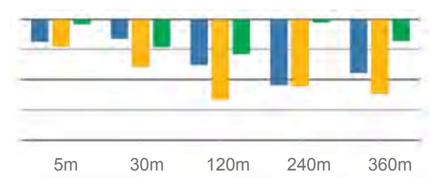

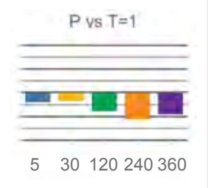

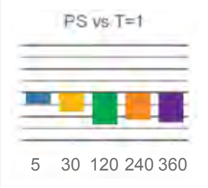

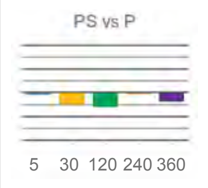

Locus		5m	30m	120m	240m	360m
**PGN_1774**	P vs T=1	-0.040	-0.130	-0.763	-0.234	-0.612
	PS vs T=1	-0.191	-0.049	0.396	1.013	0.827
	PS vs P	-0.146	0.072	0.885	1.173	1.259

conserved hypothetical protein

hypothetical proteins-Conserved

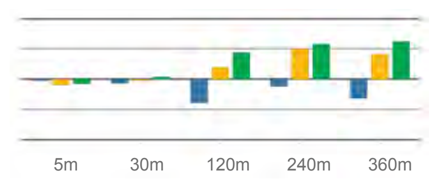

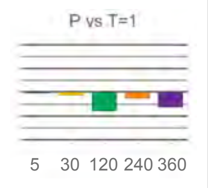

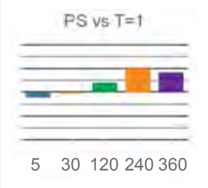

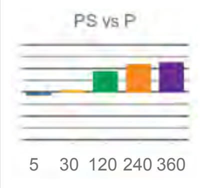

Locus		5m	30m	120m	240m	360m
**PGN_1775**	P vs T=1	-0.361	0.040	0.284	-0.048	-0.323
	PS vs T=1	-0.429	-0.252	-0.415	-0.637	-0.862
	PS vs P	-0.077	-0.277	-0.665	-0.573	-0.540

conserved hypothetical protein

hypothetical proteins-Conserved

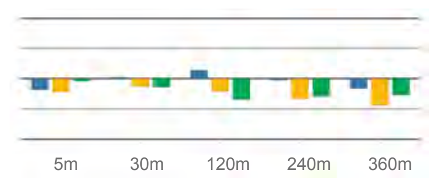

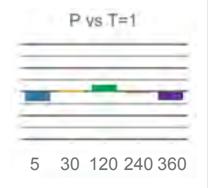

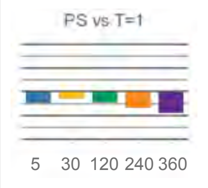

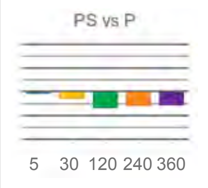

Locus		5m	30m	120m	240m	360m
**PGN_1776**	P vs T=1	0.021	0.577	0.912	0.753	0.788
	PS vs T=1	0.500	0.542	0.109	0.123	0.037
	PS vs P	0.445	-0.019	-0.754	-0.593	-0.719

peptidyl-dipeptidase

protein fate

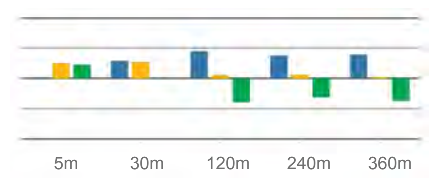

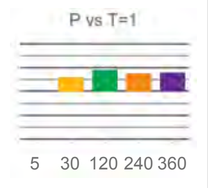

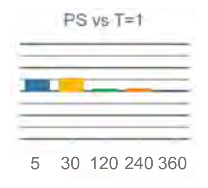

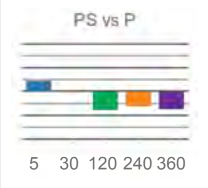

Locus		5m	30m	120m	240m	360m
**PGN_1777**	P vs T=1	-0.463	-0.845	-1.151	-1.543	-1.981
	PS vs T=1	-1.379	-1.567	-1.315	-1.189	-1.317
	PS vs P	-0.807	-0.664	-0.145	0.318	0.601

bleomycin hydrolase

protein fate

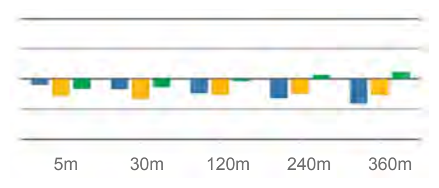

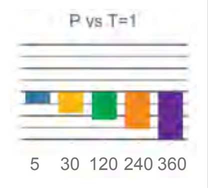

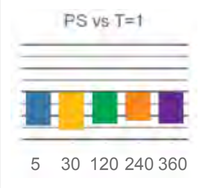

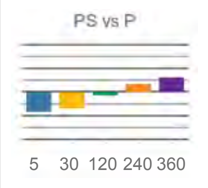

Locus		5m	30m	120m	240m	360m
**PGN_1778**	P vs T=1	0.151	-0.238	1.131	1.226	3.258
	PS vs T=1	-0.924	-0.154	1.272	0.918	2.205
	PS vs P	-0.946	-0.359	0.347	-0.175	-0.097

hypothetical protein

hypothetical proteins

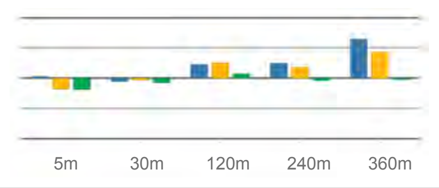

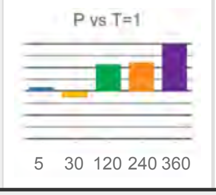

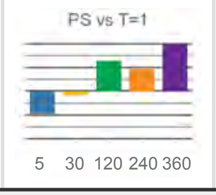

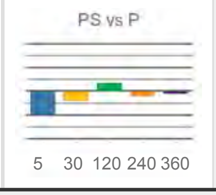

PgSg Time Course

log$_2$ (Fold Change)

Legend: P vs T=1 | PS vs T=1 | PS vs P

Locus		5m	30m	120m	240m	360m
**PGN_1779**	P vs T=1	-2.130	-2.087	-1.275	-0.609	0.234
	PS vs T=1	-1.906	-1.895	-1.483	-1.186	-1.162
	PS vs P	-0.331	-0.330	-0.215	-0.190	-0.496

conserved hypothetical protein

hypothetical proteins-Conserved

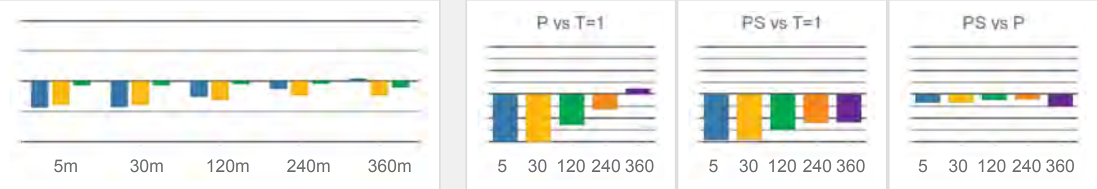

Locus		5m	30m	120m	240m	360m
**PGN_1780**	P vs T=1	-0.195	0.005	0.665	0.568	-0.135
	PS vs T=1	-0.163	0.423	0.439	-0.100	-0.036
	PS vs P	0.014	0.406	-0.184	-0.629	0.087

putative YjgF-like protein

transcription

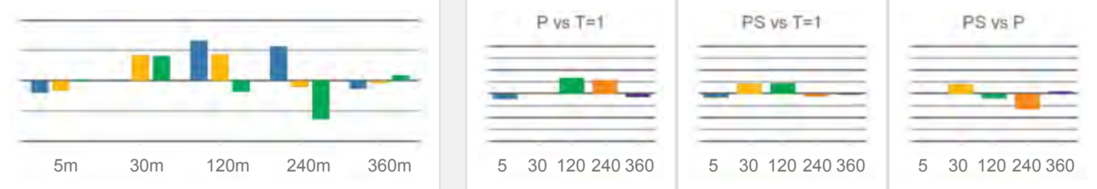

Locus		5m	30m	120m	240m	360m
**PGN_1781**	P vs T=1	-0.168	0.140	0.104	0.093	0.033
	PS vs T=1	0.619	0.947	0.537	-0.178	-0.300
	PS vs P	0.738	0.793	0.427	-0.263	-0.330

putative tRNA:rRNA methyltransferase

protein synthesis

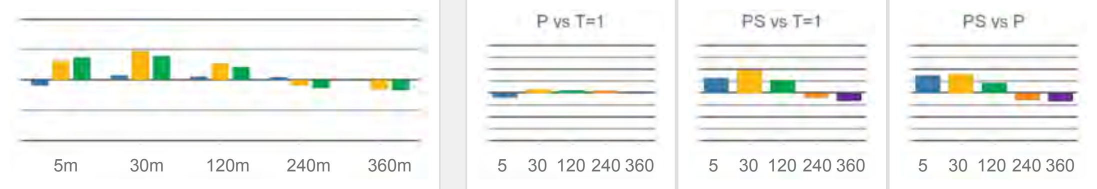

Locus		5m	30m	120m	240m	360m
**PGN_1782**	P vs T=1	-1.004	-0.710	-0.573	-0.841	-0.889
	PS vs T=1	-0.404	-0.495	-1.326	-1.581	-1.532
	PS vs P	0.588	0.215	-0.741	-0.738	-0.643

DNA repair protein RecN

DNA metabolism

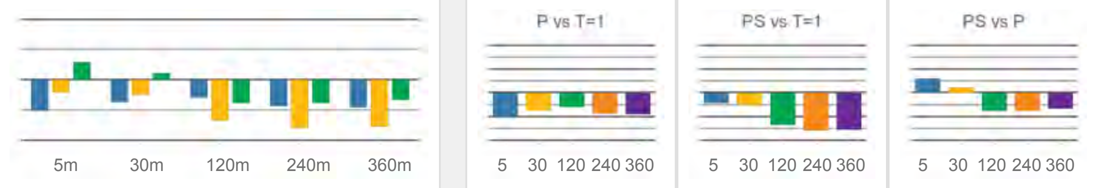

Locus		5m	30m	120m	240m	360m
**PGN_1783**	P vs T=1	-0.513	-0.971	-1.356	-1.221	-0.769
	PS vs T=1	0.307	-0.341	-1.506	-1.574	-1.513
	PS vs P	0.825	0.609	-0.214	-0.392	-0.722

conserved hypothetical protein

hypothetical proteins-Conserved

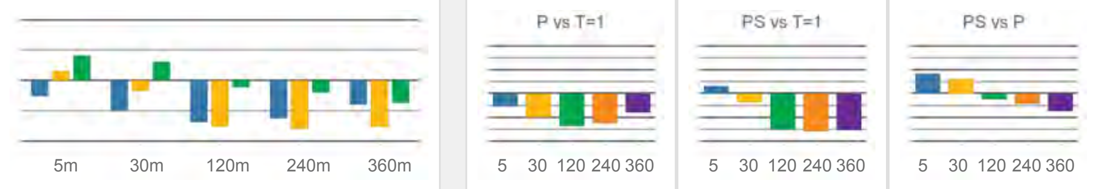

Locus		5m	30m	120m	240m	360m
**PGN_1784**	P vs T=1	0.244	0.161	-0.305	-0.680	-0.572
	PS vs T=1	0.872	0.333	-0.550	-0.776	-0.936
	PS vs P	0.636	0.182	-0.249	-0.137	-0.373

DNA/pantothenate metabolism flavoprotein

unknown function

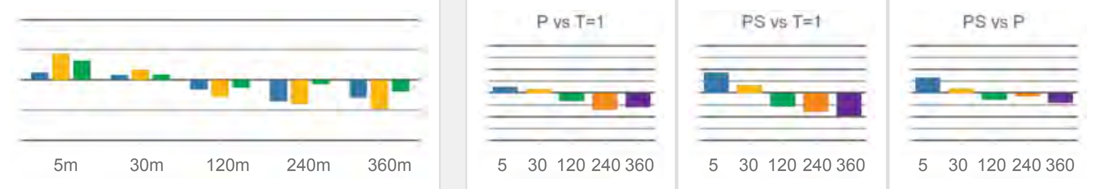

Locus		5m	30m	120m	240m	360m
**PGN_1785**	P vs T=1	-0.259	-0.122	-0.778	-1.465	-1.735
	PS vs T=1	-0.126	-0.574	-1.202	-1.260	-1.497
	PS vs P	0.146	-0.422	-0.419	0.136	0.182

putative DNA polymerase III epsilon chain

DNA metabolism

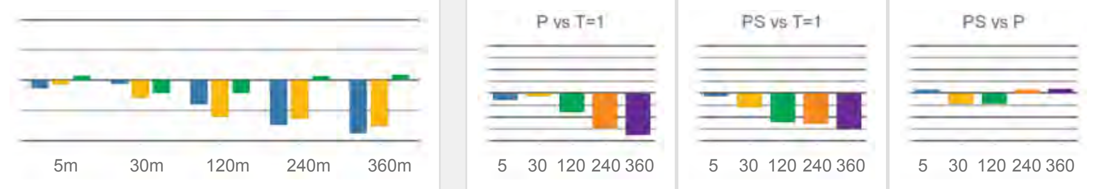

PgSg Time Course

log2 (Fold Change)

Legend: P vs T=1 | PS vs T=1 | PS vs P

Locus		5m	30m	120m	240m	360m
**PGN_1786**	P vs T=1	0.349	0.321	-0.293	-0.888	-1.330
	PS vs T=1	0.243	0.133	-0.227	-0.252	-0.443
	PS vs P	-0.092	-0.172	0.061	0.589	0.839
	putative DNA polymerase III beta chain					
	DNA metabolism					

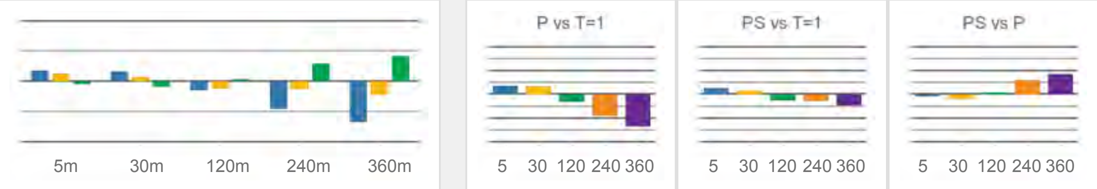

Locus		5m	30m	120m	240m	360m
**PGN_1787**	P vs T=1	-0.832	-1.283	-1.976	-1.419	-0.856
	PS vs T=1	-1.124	-1.344	-1.740	-1.817	-1.527
	PS vs P	-0.256	-0.070	0.020	-0.409	-0.604
	probable 5-formyltetrahydrofolate cyclo-ligase					
	central intermediary metabolism					

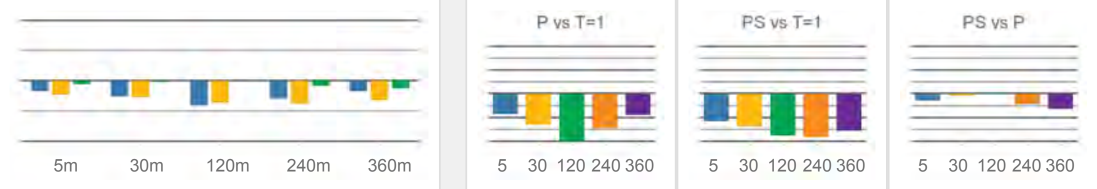

Locus		5m	30m	120m	240m	360m
**PGN_1788**	P vs T=1	0.431	-0.299	-0.781	-0.661	-0.160
	PS vs T=1	-0.001	-0.081	-0.494	-0.465	-0.636
	PS vs P	-0.378	0.213	0.235	0.165	-0.447
	carboxyl-terminal processing protease					
	protein fate					

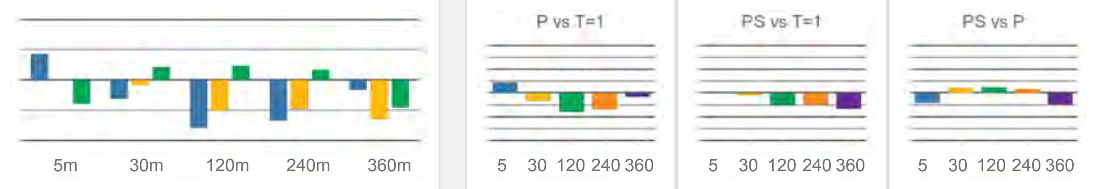

Locus		5m	30m	120m	240m	360m
**PGN_1789**	P vs T=1	-0.734	-1.496	-2.055	-1.616	-1.481
	PS vs T=1	-1.391	-1.449	-1.477	-1.257	-1.231
	PS vs P	-0.558	0.006	0.358	0.304	0.219
	putative deoxycytidylate deaminase					
	unknown function					

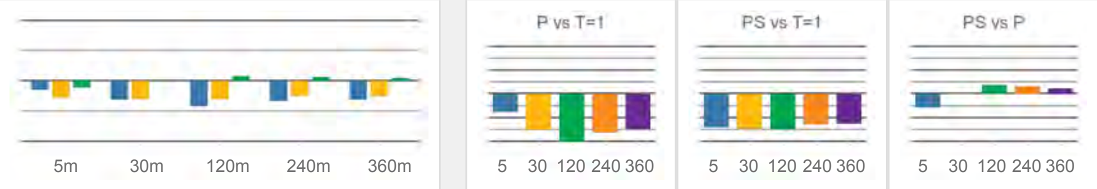

Locus		5m	30m	120m	240m	360m
**PGN_1790**	P vs T=1	-0.138	-0.283	-0.194	0.475	1.338
	PS vs T=1	-0.954	-0.384	0.301	0.039	-0.072
	PS vs P	-0.731	-0.136	0.382	-0.141	-0.878
	conserved hypothetical protein					
	hypothetical proteins-Conserved					

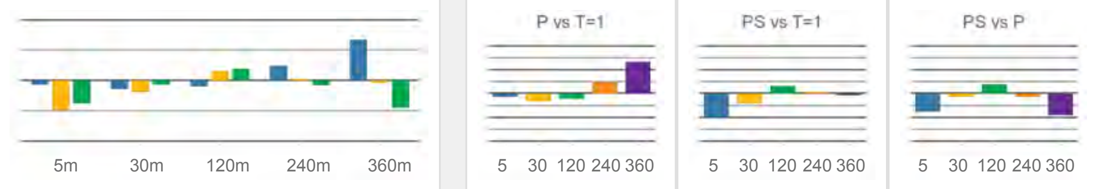

Locus		5m	30m	120m	240m	360m
**PGN_1791**	P vs T=1	0.557	0.982	1.615	1.930	1.592
	PS vs T=1	-0.338	0.196	0.711	0.742	0.754
	PS vs P	-0.887	-0.752	-0.836	-1.074	-0.785
	putative flavodoxin					
	energy metabolism					

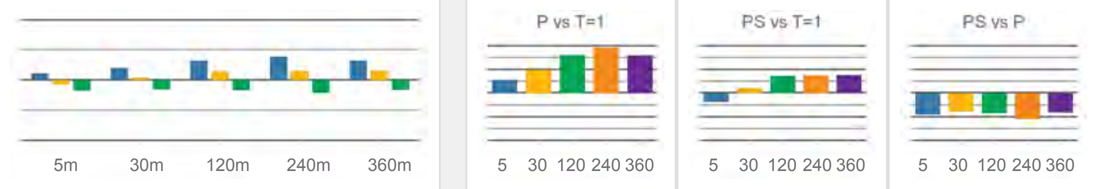

Locus		5m	30m	120m	240m	360m
**PGN_1792**	P vs T=1	-0.136	-0.012	-0.199	-0.314	-0.052
	PS vs T=1	-0.104	-0.327	-0.880	-0.973	-0.782
	PS vs P	0.036	-0.295	-0.670	-0.658	-0.704
	glycerate kinase					
	energy metabolism					

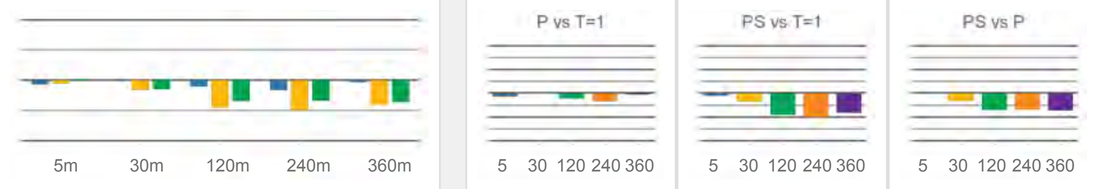

PgSg Time Course

Hackett Laboratory UW

log$_2$ (Fold Change)

Locus		5m	30m	120m	240m	360m
**PGN_1793**	P vs T=1	0.300	-0.261	-0.977	-1.456	-1.375
	PS vs T=1	0.106	-0.141	-1.155	-1.722	-1.853
	PS vs P	-0.148	0.136	-0.190	-0.324	-0.499
	conserved hypothetical protein					
	hypothetical proteins-Conserved					
**PGN_1794**	P vs T=1	0.086	-0.243	-0.444	-0.781	-0.742
	PS vs T=1	-0.044	-0.079	-0.813	-1.079	-0.815
	PS vs P	-0.065	0.157	-0.374	-0.416	-0.141
	conserved hypothetical protein					
	hypothetical proteins-Conserved					
**PGN_1795**	P vs T=1	0.063	0.237	0.474	0.125	-0.510
	PS vs T=1	0.232	0.458	0.113	-0.239	-0.378
	PS vs P	0.153	0.221	-0.318	-0.357	0.080
	conserved hypothetical protein					
	hypothetical proteins-Conserved					
**PGN_1796**	P vs T=1	-0.280	-1.103	-1.827	-2.141	-2.093
	PS vs T=1	-0.507	-0.933	-1.160	-1.243	-1.540
	PS vs P	-0.126	0.156	0.585	0.779	0.484
	conserved hypothetical protein					
	unknown function					
**PGN_1797**	P vs T=1	1.269	1.387	1.428	1.582	1.349
	PS vs T=1	0.146	1.413	3.107	3.299	3.044
	PS vs P	-1.087	0.027	1.604	1.657	1.613
	conserved hypothetical protein					
	hypothetical proteins-Conserved					
**PGN_1798**	P vs T=1	1.086	1.058	0.852	1.137	1.892
	PS vs T=1	0.523	0.384	0.184	0.340	0.631
	PS vs P	-0.511	-0.613	-0.673	-0.707	-1.088
	conserved hypothetical protein					
	central intermediary metabolism					
**PGN_1799**	P vs T=1	1.497	1.530	1.828	1.645	2.758
	PS vs T=1	0.868	0.421	0.771	1.663	0.986
	PS vs P	-0.496	-0.880	-0.784	0.061	-1.155
	hypothetical protein					
	hypothetical proteins					

P vs T=1 | PS vs T=1 | PS vs P

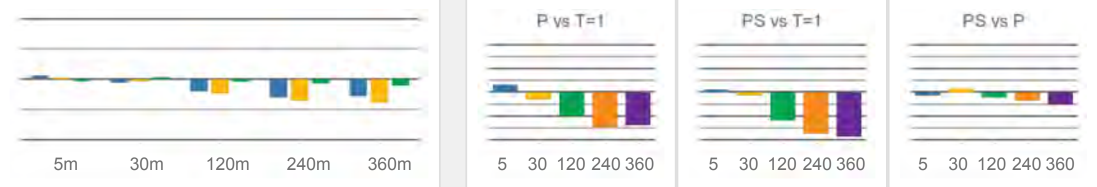

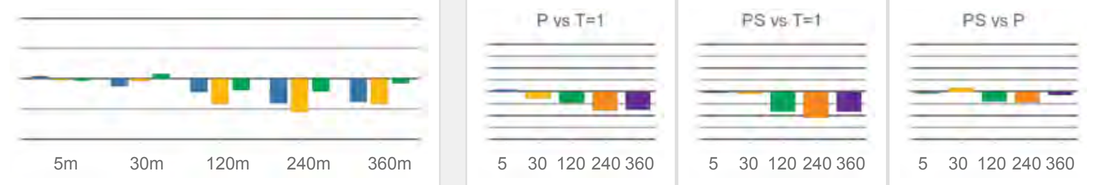

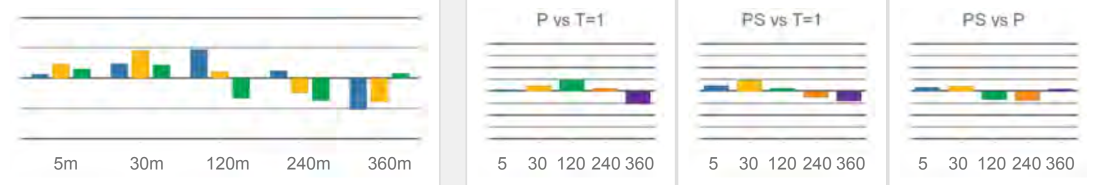

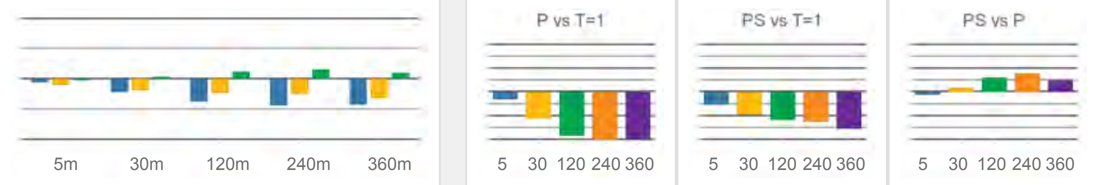

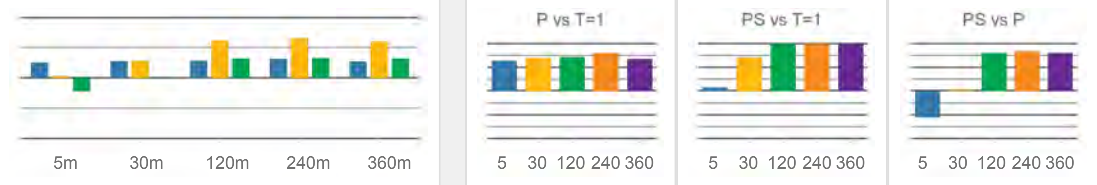

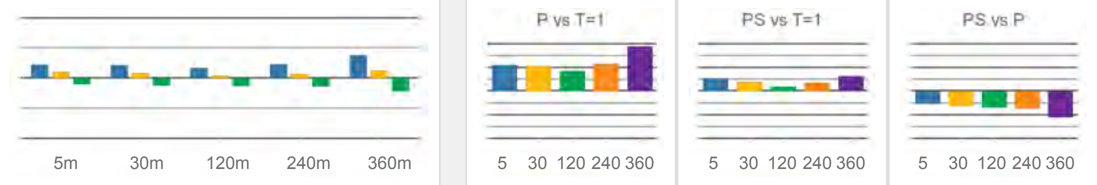

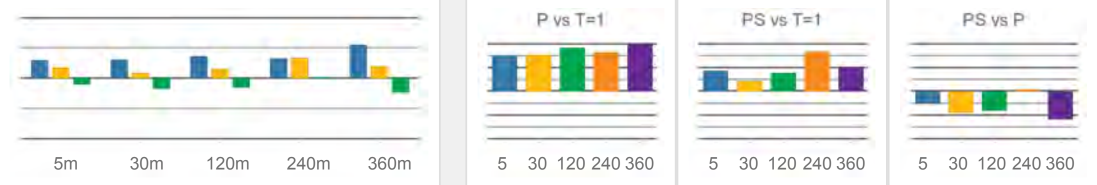

PgSg Time Course

log$_2$ (Fold Change)

Legend: P vs T=1 | PS vs T=1 | PS vs P

Locus		5m	30m	120m	240m	360m
**PGN_1800**	P vs T=1	0.369	0.464	0.439	0.300	-0.206
	PS vs T=1	0.372	-0.252	-0.626	-0.526	-0.541
	PS vs P	0.023	-0.679	-1.029	-0.796	-0.341
	urocanate hydratase					
	energy metabolism					
**PGN_1801**	P vs T=1	-0.317	-0.240	-0.548	-0.106	0.634
	PS vs T=1	-0.100	-0.488	-0.951	-0.612	-0.752
	PS vs P	0.204	-0.224	-0.490	-0.427	-1.185
	conserved hypothetical protein					
	hypothetical proteins-Conserved					
**PGN_1802**	P vs T=1	-0.005	-0.468	-0.606	-0.532	0.138
	PS vs T=1	0.939	0.195	-0.844	-0.780	-0.776
	PS vs P	0.951	0.622	-0.272	-0.269	-0.849
	hemolysin					
	cellular processes					
**PGN_1803**	P vs T=1	0.046	0.069	-0.181	-0.356	-0.161
	PS vs T=1	0.783	0.314	-0.258	-0.605	-0.535
	PS vs P	0.731	0.248	-0.084	-0.275	-0.369
	conserved hypothetical protein					
	hypothetical proteins-Conserved					
**PGN_1804**	P vs T=1	-0.208	-1.007	-1.936	-2.118	-2.069
	PS vs T=1	-0.379	-1.364	-1.798	-2.069	-1.973
	PS vs P	-0.104	-0.341	0.079	-0.030	0.053
	Na+/H+ antiporter					
	transport and binding proteins					
**PGN_1805**	P vs T=1	0.298	0.145	-0.822	-1.181	-1.003
	PS vs T=1	0.557	0.097	-0.152	-0.072	-0.613
	PS vs P	0.278	-0.029	0.616	1.005	0.349
	cysteinyl-tRNA synthetase					
	protein synthesis					
**PGN_1806**	P vs T=1	0.502	0.171	-1.001	-1.121	-0.977
	PS vs T=1	1.056	0.755	-0.243	-0.534	-0.758
	PS vs P	0.576	0.591	0.673	0.488	0.178
	conserved hypothetical protein					
	hypothetical proteins-Conserved					

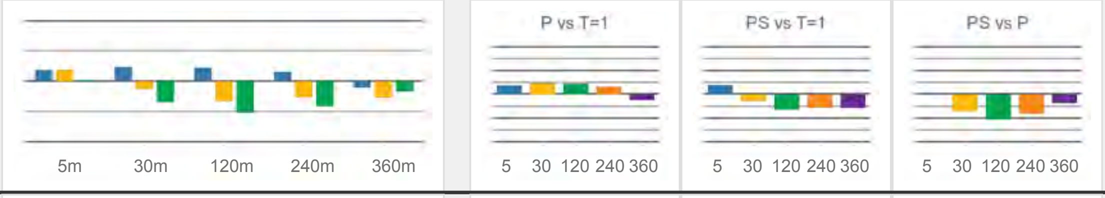

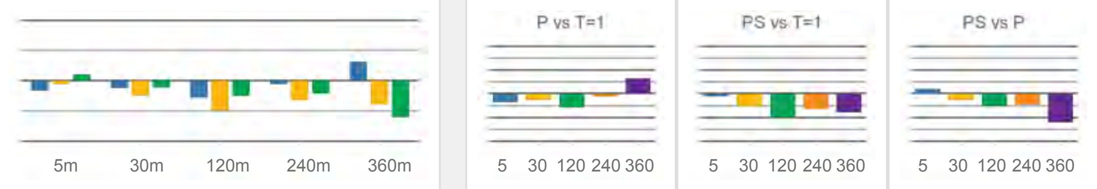

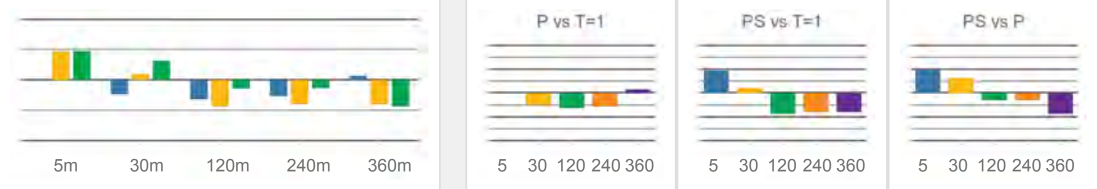

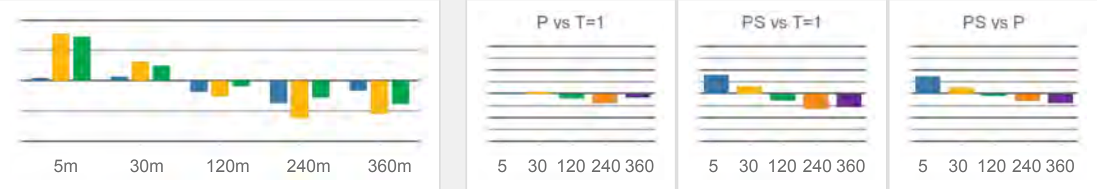

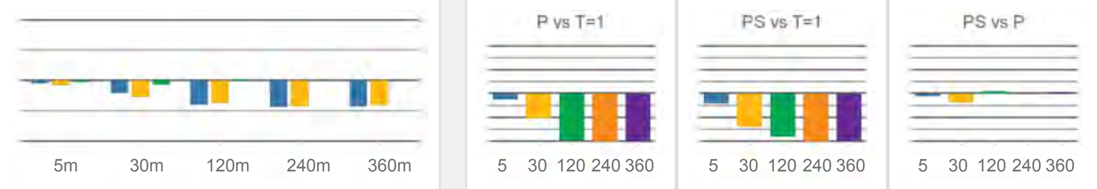

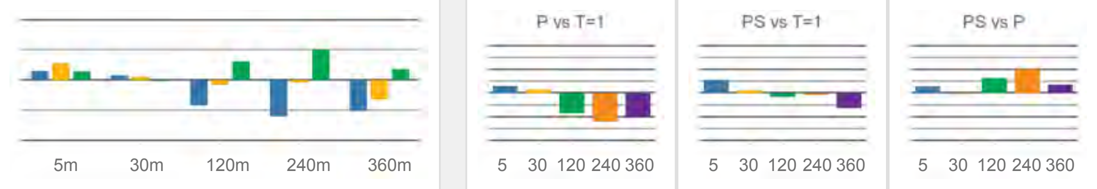

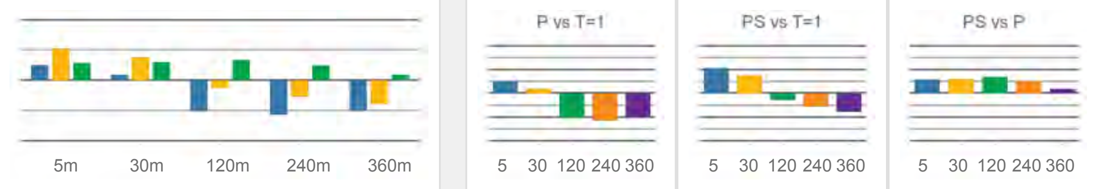

log$_2$ (Fold Change)

Legend: P vs T=1 | PS vs T=1 | PS vs P

PGN_1807

Locus	5m	30m	120m	240m	360m
P vs T=1	0.199	0.009	-0.977	-1.218	-0.829
PS vs T=1	0.941	0.623	-0.523	-1.002	-1.142
PS vs P	0.754	0.623	0.388	0.106	-0.326

putative glycosyltransferase

cell envelope

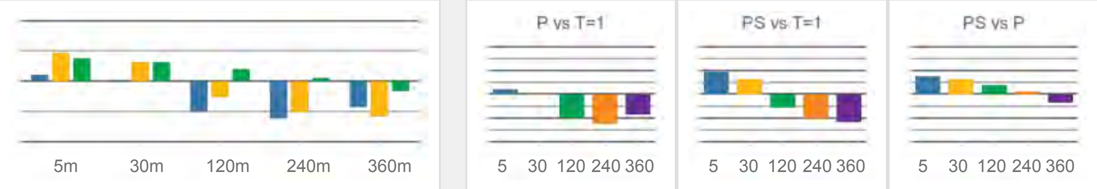

PGN_1808

Locus	5m	30m	120m	240m	360m
P vs T=1	-0.794	-0.968	-1.247	-1.629	-1.855
PS vs T=1	-0.546	-0.502	-1.401	-1.875	-1.946
PS vs P	0.266	0.464	-0.154	-0.288	-0.139

conserved hypothetical protein

hypothetical proteins-Conserved

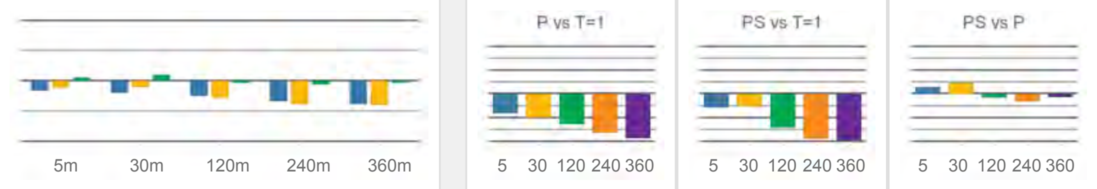

PGN_1809

Locus	5m	30m	120m	240m	360m
P vs T=1	-1.405	-1.251	-1.286	-1.251	-1.564
PS vs T=1	-1.181	-1.143	-1.822	-2.132	-2.442
PS vs P	0.194	0.099	-0.535	-0.839	-0.911

hypothetical protein

hypothetical proteins

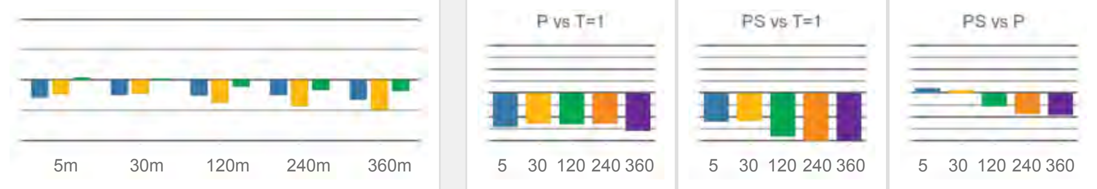

PGN_1810

Locus	5m	30m	120m	240m	360m
P vs T=1	-0.280	-0.097	-0.301	-0.932	-1.285
PS vs T=1	0.248	0.512	0.082	-0.485	-0.493
PS vs P	0.498	0.616	0.400	0.275	0.606

hypothetical protein

hypothetical proteins

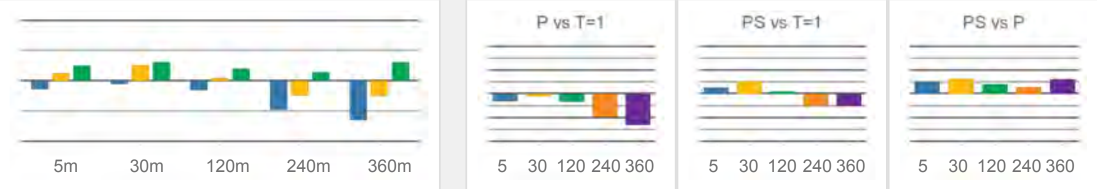

PGN_1811

Locus	5m	30m	120m	240m	360m
P vs T=1	0.787	0.850	0.211	-0.389	-0.855
PS vs T=1	0.899	0.664	-0.500	-1.242	-1.238
PS vs P	0.147	-0.137	-0.691	-0.864	-0.412

putative alpha-L-fucosidase

cell envelope

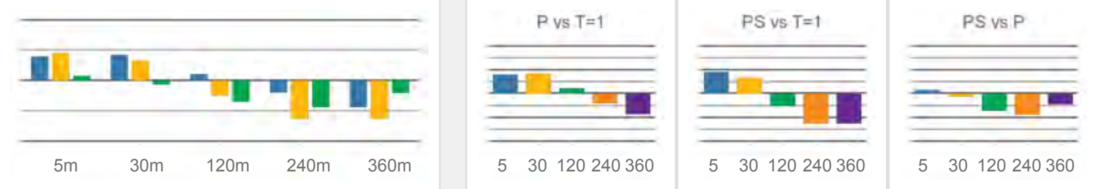

PGN_1812

Locus	5m	30m	120m	240m	360m
P vs T=1	0.806	0.743	0.107	-0.323	-0.692
PS vs T=1	0.800	0.137	-1.021	-1.238	-1.130
PS vs P	0.040	-0.552	-1.105	-0.916	-0.455

polyphosphate kinase

central intermediary metabolism

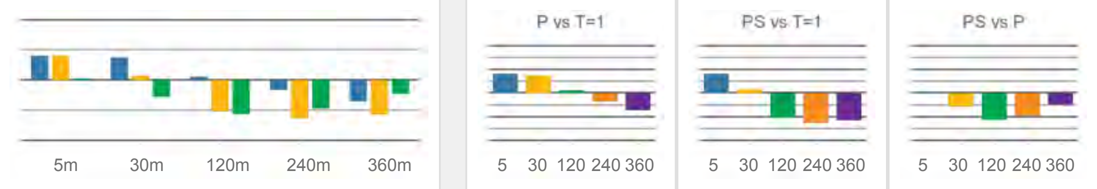

PGN_1813

Locus	5m	30m	120m	240m	360m
P vs T=1	0.180	-0.051	-0.279	-0.190	-0.321
PS vs T=1	0.185	0.002	0.215	0.469	0.318
PS vs P	0.010	0.046	0.468	0.637	0.617

GTP-binding protein

unknown function

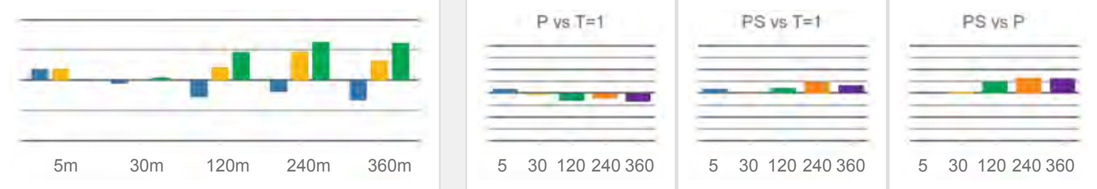

log2 (Fold Change)

Legend: P vs T=1 | PS vs T=1 | PS vs P

Locus		5m	30m	120m	240m	360m
PGN_1814	P vs T=1	-0.729	-0.473	-0.534	-0.723	-0.456
	PS vs T=1	-0.428	-0.405	-0.564	-0.566	-0.484
	PS vs P	0.247	0.069	-0.037	0.097	-0.026

conserved hypothetical protein

unknown function

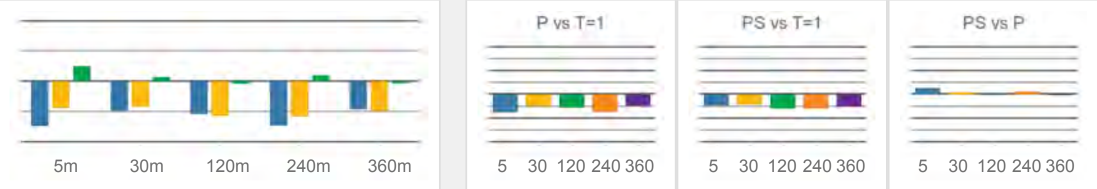

Locus		5m	30m	120m	240m	360m
PGN_1815	P vs T=1	-0.032	-0.111	-0.520	-0.536	0.344
	PS vs T=1	0.364	0.149	0.247	0.374	0.024
	PS vs P	0.389	0.247	0.628	0.714	-0.228

conserved hypothetical protein

hypothetical proteins-Conserved

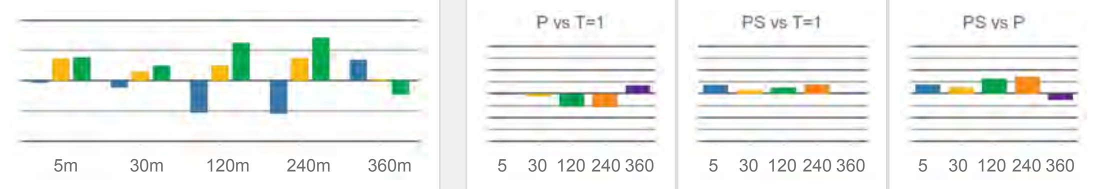

Locus		5m	30m	120m	240m	360m
PGN_1816	P vs T=1	0.501	0.221	0.081	-0.184	-0.850
	PS vs T=1	0.233	0.721	1.373	1.570	1.379
	PS vs P	-0.247	0.484	1.267	1.698	2.145

conserved hypothetical protein

hypothetical proteins-Conserved

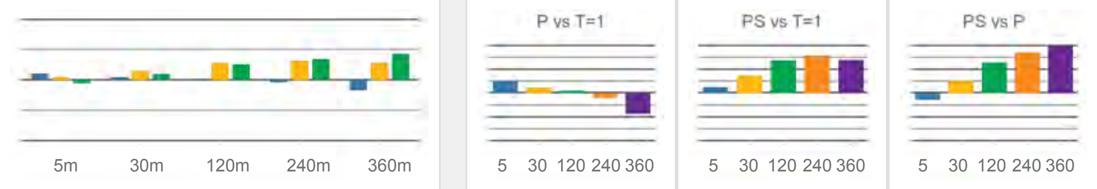

Locus		5m	30m	120m	240m	360m
PGN_1817	P vs T=1	-0.688	-0.626	-1.296	-1.270	-1.167
	PS vs T=1	-1.204	0.313	0.935	0.937	0.440
	PS vs P	-0.538	0.903	2.032	1.994	1.494

conserved hypothetical protein

hypothetical proteins-Conserved

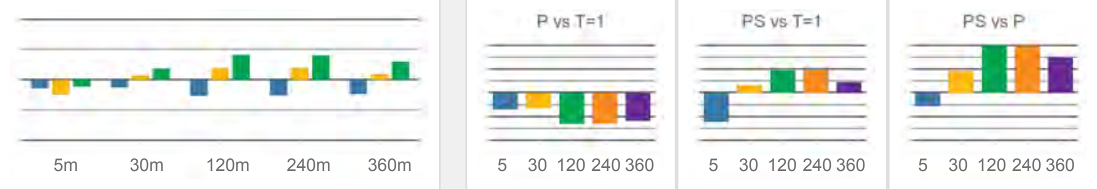

Locus		5m	30m	120m	240m	360m
PGN_1818	P vs T=1	-1.565	-2.551	-3.193	-2.939	-2.038
	PS vs T=1	-1.477	-1.563	-1.401	-1.929	-2.390
	PS vs P	0.134	0.870	1.605	0.887	-0.341

conserved hypothetical protein

hypothetical proteins-Conserved

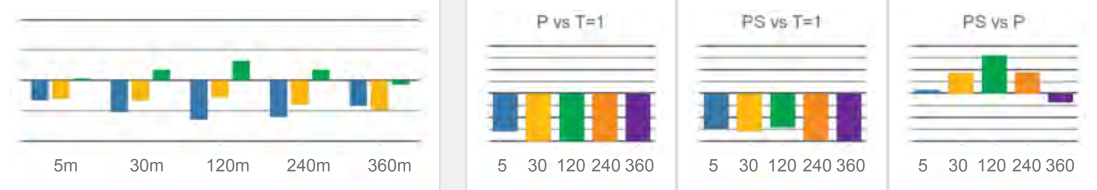

Locus		5m	30m	120m	240m	360m
PGN_1819	P vs T=1	-1.913	-2.639	-3.563	-3.943	-3.392
	PS vs T=1	-1.617	-2.110	-2.884	-3.932	-3.928
	PS vs P	0.324	0.516	0.582	-0.194	-0.578

hypothetical protein

hypothetical proteins

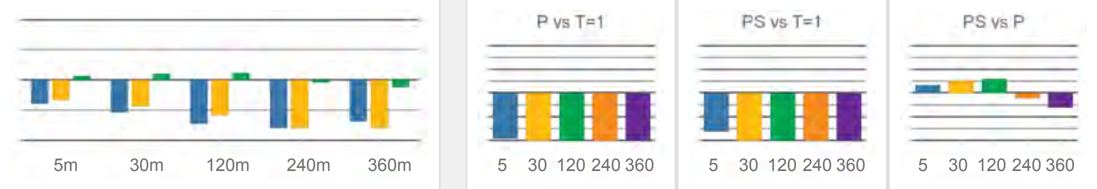

Locus		5m	30m	120m	240m	360m
PGN_1820	P vs T=1	-0.781	-1.539	-2.203	-2.715	-2.183
	PS vs T=1	-0.087	-0.322	-1.094	-1.489	-2.462
	PS vs P	0.747	1.142	0.963	0.877	-0.344

conserved hypothetical protein

hypothetical proteins-Conserved

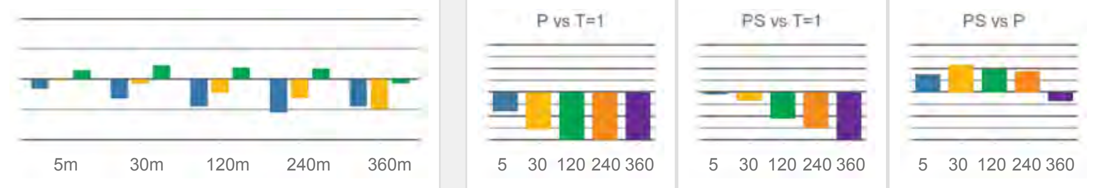

PgSg Time Course

log$_2$ (Fold Change)

Legend: P vs T=1 | PS vs T=1 | PS vs P

PGN_1821

Locus	5m	30m	120m	240m	360m
P vs T=1	-0.395	-0.979	-1.145	-0.829	-0.764
PS vs T=1	0.465	0.350	-0.258	-0.971	-1.067
PS vs P	0.854	1.256	0.822	-0.151	-0.307

conserved hypothetical protein

hypothetical proteins-Conserved

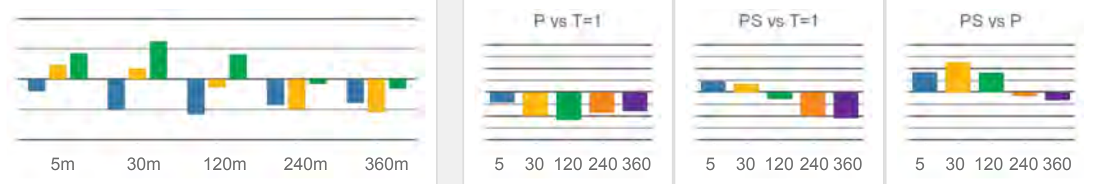

PGN_1822

Locus	5m	30m	120m	240m	360m
P vs T=1	-0.986	-1.709	-1.487	-1.396	-1.199
PS vs T=1	-0.162	-0.052	-0.421	-0.464	-0.616
PS vs P	0.669	0.998	0.702	0.588	0.402

hypothetical protein

hypothetical proteins

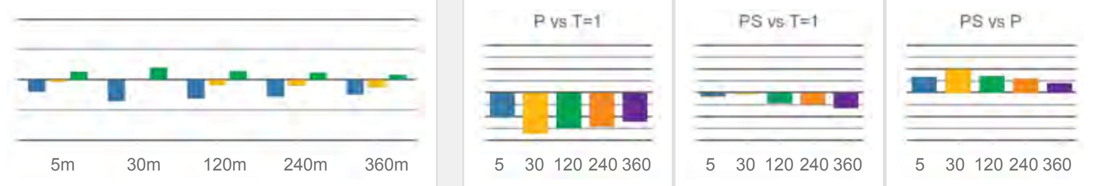

PGN_1823

Locus	5m	30m	120m	240m	360m
P vs T=1	-1.234	-2.358	-2.280	-1.564	0.137
PS vs T=1	-0.491	-0.316	-0.541	-0.634	-1.056
PS vs P	0.644	1.264	1.085	0.668	-0.755

conserved hypothetical protein

hypothetical proteins-Conserved

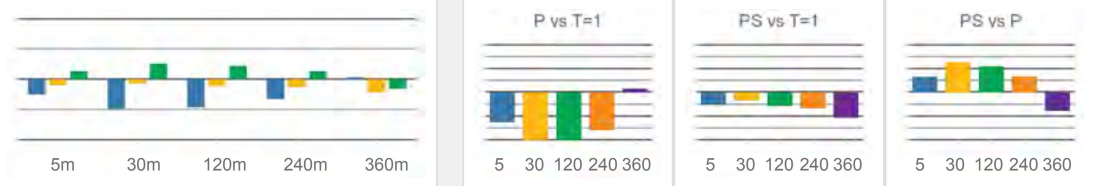

PGN_1824

Locus	5m	30m	120m	240m	360m
P vs T=1	-0.414	-0.957	-1.266	-0.545	0.361
PS vs T=1	0.174	-0.186	-0.358	-0.017	-0.271
PS vs P	0.554	0.524	0.409	0.415	-0.417

hypothetical protein

hypothetical proteins

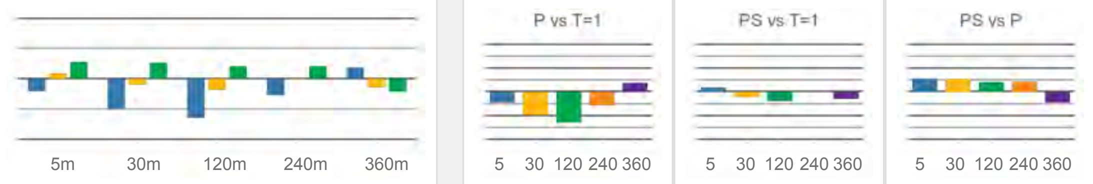

PGN_1825

Locus	5m	30m	120m	240m	360m
P vs T=1	-0.577	-1.282	-0.876	0.059	1.941
PS vs T=1	-0.442	-0.486	-0.128	0.025	0.271
PS vs P	0.098	0.506	0.491	0.010	-1.398

hypothetical protein

hypothetical proteins

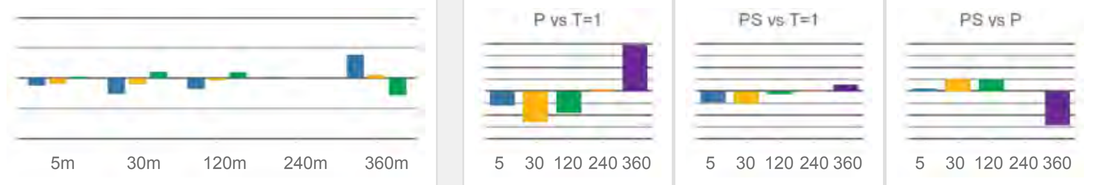

PGN_1826

Locus	5m	30m	120m	240m	360m
P vs T=1	-0.799	-1.918	-0.943	-0.253	1.924
PS vs T=1	-1.232	-0.423	1.178	1.349	1.302
PS vs P	-0.549	0.385	1.525	1.254	-0.002

conserved hypothetical protein

hypothetical proteins-Conserved

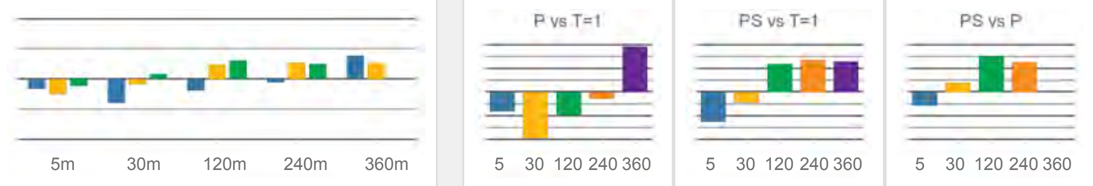

PGN_1827

Locus	5m	30m	120m	240m	360m
P vs T=1	0.055	0.227	0.715	1.134	1.049
PS vs T=1	-0.208	-0.170	0.133	0.957	1.059
PS vs P	-0.288	-0.412	-0.578	-0.146	0.018

S-adenosylmethionine synthase

central intermediary metabolism

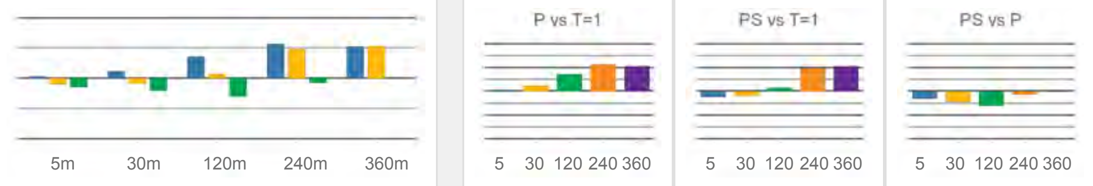

PgSg Time Course

log$_2$ (Fold Change)

Legend: P vs T=1 | PS vs T=1 | PS vs P

Locus		5m	30m	120m	240m	360m
**PGN_1828**	P vs T=1	-0.472	-0.366	0.259	0.524	0.486
	PS vs T=1	-0.529	-0.330	-0.142	-0.126	0.392
	PS vs P	-0.105	-0.012	-0.365	-0.568	-0.064

conserved hypothetical protein

hypothetical proteins-Conserved

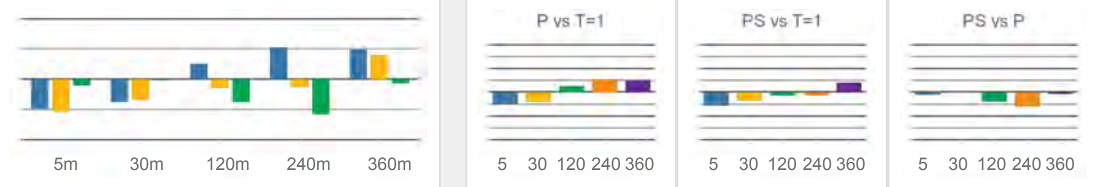

Locus		5m	30m	120m	240m	360m
**PGN_1829**	P vs T=1	-0.485	-0.595	0.175	0.669	0.906
	PS vs T=1	-0.622	-0.732	-0.094	0.138	0.557
	PS vs P	-0.190	-0.247	-0.234	-0.365	-0.236

probable nicotinamide mononucleotide transporter

transport and binding proteins

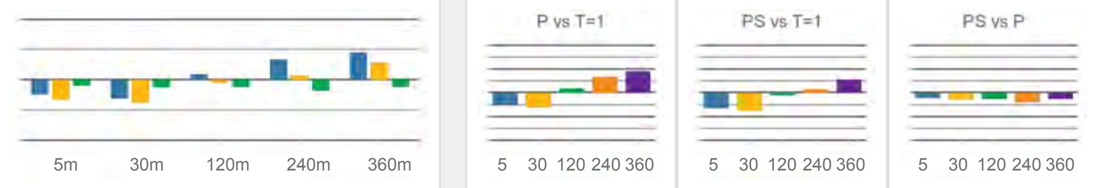

Locus		5m	30m	120m	240m	360m
**PGN_1830**	P vs T=1	-1.335	-0.911	-0.511	-0.355	-0.070
	PS vs T=1	-1.289	-1.013	-0.671	-0.483	-0.480
	PS vs P	0.007	-0.114	-0.156	-0.117	-0.393

putative TonB-dependent receptor

transport and binding proteins

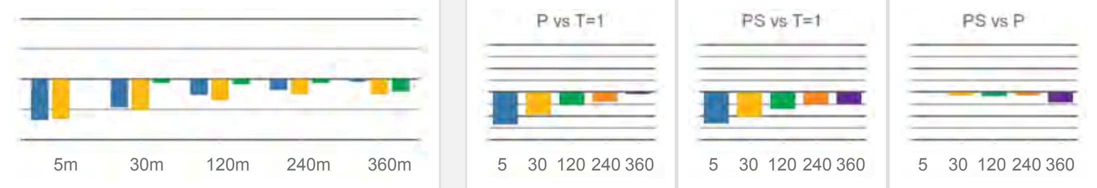

Locus		5m	30m	120m	240m	360m
**PGN_1831**	P vs T=1	-0.596	-0.233	0.320	0.204	0.320
	PS vs T=1	-0.230	-0.482	-0.355	-0.144	-0.172
	PS vs P	0.318	-0.254	-0.619	-0.315	-0.459

putative GTPase

hypothetical proteins-Conserved

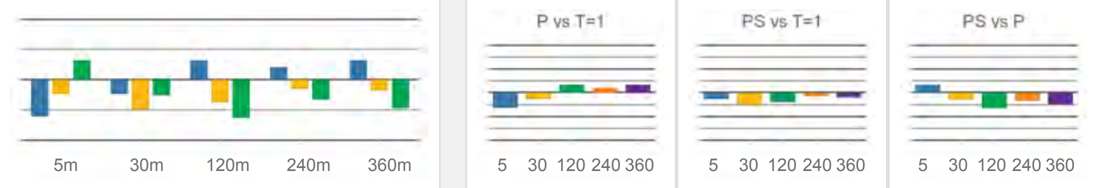

Locus		5m	30m	120m	240m	360m
**PGN_1832**	P vs T=1	1.160	1.833	2.199	1.749	1.107
	PS vs T=1	1.347	1.934	1.430	1.005	0.860
	PS vs P	0.175	0.114	-0.736	-0.726	-0.255

putative ribosome recycling factor

protein synthesis

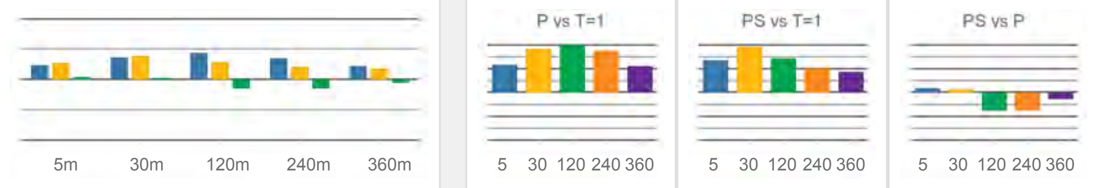

Locus		5m	30m	120m	240m	360m
**PGN_1833**	P vs T=1	-0.025	0.016	0.074	0.104	-0.092
	PS vs T=1	0.203	0.259	0.001	0.004	0.015
	PS vs P	0.213	0.231	-0.073	-0.094	0.092

putative uridylate kinase

purines, pyrimidines, nucleosides and nucleotides

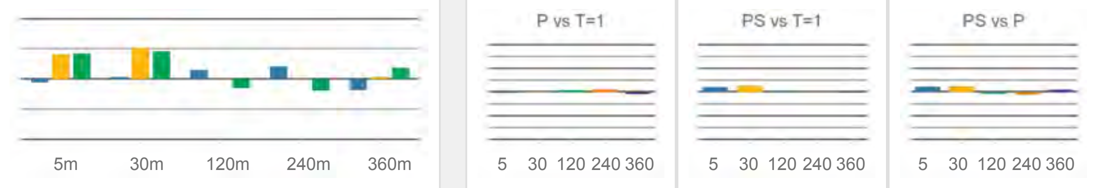

Locus		5m	30m	120m	240m	360m
**PGN_1834**	P vs T=1	-0.831	-0.618	-0.423	-0.481	-0.312
	PS vs T=1	-0.621	-0.773	-0.727	-0.612	-0.411
	PS vs P	0.196	-0.156	-0.294	-0.129	-0.091

conserved hypothetical protein

hypothetical proteins-Conserved

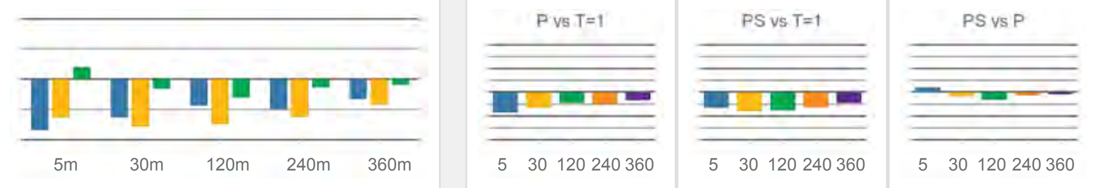

PgSg Time Course

P vs T=1 | PS vs T=1 | PS vs P

Locus		log2 (Fold Change) 5m	30m	120m	240m	360m
**PGN_1835**	P vs T=1	0.031	-0.441	0.570	0.670	2.921
	PS vs T=1	-0.484	-0.899	0.164	1.412	0.799
	PS vs P	-0.441	-0.637	-0.183	0.713	-1.146

conserved hypothetical protein

hypothetical proteins-Conserved

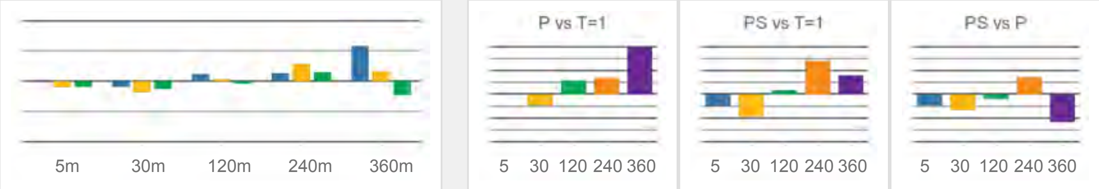

Locus		5m	30m	120m	240m	360m
**PGN_1836**	P vs T=1	0.329	-0.239	-0.060	0.577	1.731
	PS vs T=1	-0.082	0.104	0.490	0.574	0.791
	PS vs P	-0.384	0.228	0.418	0.019	-0.790

transposase in ISPg3

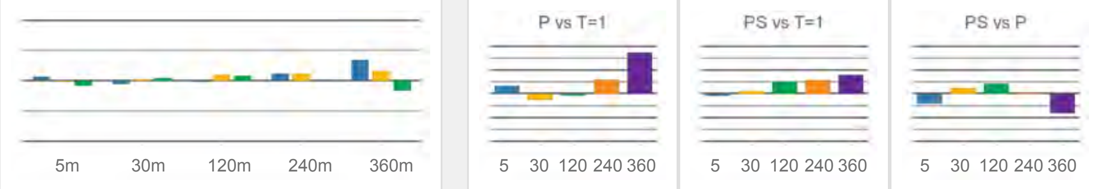

Locus		5m	30m	120m	240m	360m
**PGN_1837**	P vs T=1	-1.353	-2.184	-2.625	-2.777	-3.114
	PS vs T=1	-0.576	-0.597	-0.960	-1.214	-1.350
	PS vs P	0.742	1.254	1.323	1.208	1.406

conserved hypothetical protein

hypothetical proteins-Conserved

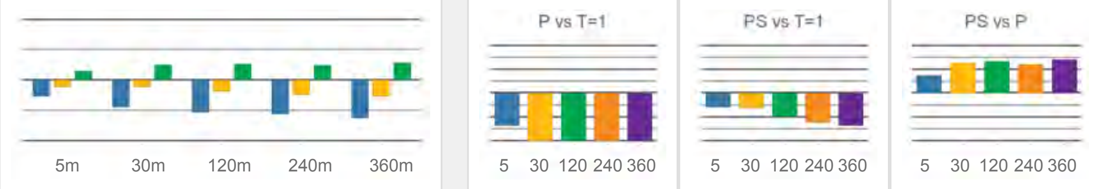

Locus		5m	30m	120m	240m	360m
**PGN_1838**	P vs T=1	0.523	0.467	0.538	0.650	0.335
	PS vs T=1	0.349	0.242	0.340	0.457	0.419
	PS vs P	-0.172	-0.231	-0.204	-0.187	0.062

partial transposase in ISPg1

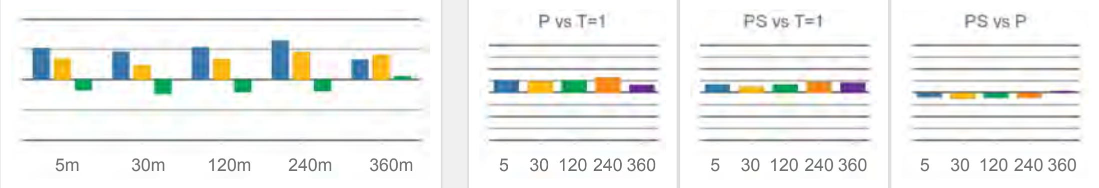

Locus		5m	30m	120m	240m	360m
**PGN_1839**	P vs T=1	-0.341	-0.554	-0.578	-0.448	-0.719
	PS vs T=1	-0.571	-0.921	-0.765	-0.442	-0.425
	PS vs P	-0.227	-0.380	-0.211	-0.007	0.259

partial transposase in ISPg1

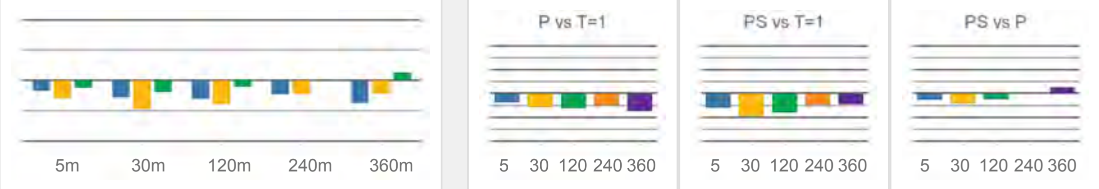

Locus		5m	30m	120m	240m	360m
**PGN_1840** *rplQ*	P vs T=1	0.142	0.523	0.748	0.373	-0.696
	PS vs T=1	0.169	0.880	1.172	0.735	0.572
	PS vs P	-0.000	0.374	0.472	0.368	1.184

50S ribosomal protein L17

protein synthesis

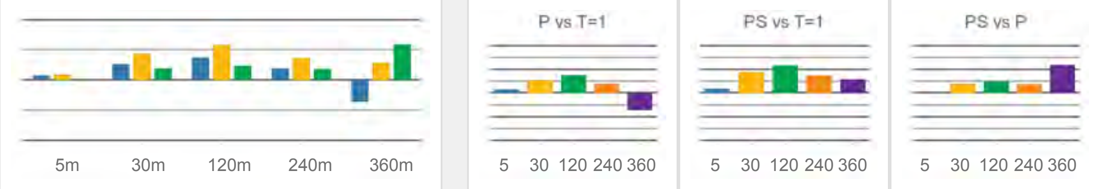

Locus		5m	30m	120m	240m	360m
**PGN_1841** *rpoA*	P vs T=1	0.137	0.390	0.318	-0.347	-1.289
	PS vs T=1	0.002	0.583	0.648	0.209	0.070
	PS vs P	-0.124	0.244	0.384	0.505	1.221

DNA-directed RNA polymerase alpha subunit

transcription

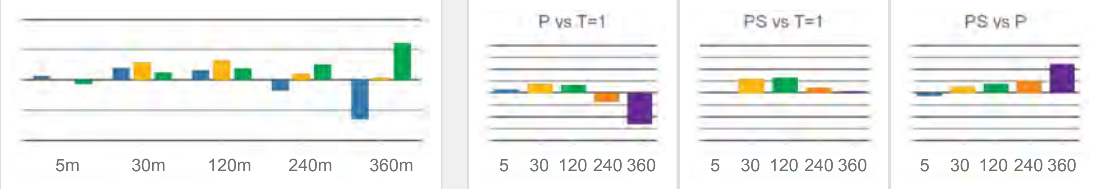

PgSg Time Course

log2 (Fold Change)

Legend: P vs T=1 | PS vs T=1 | PS vs P

Locus		5m	30m	120m	240m	360m
**PGN_1842**	P vs T=1	0.486	0.441	0.092	-0.678	-1.639
rpsD	PS vs T=1	0.272	0.675	0.597	-0.070	-0.324
	PS vs P	-0.159	0.279	0.529	0.544	1.191
	30S ribosomal protein S4					
	protein synthesis					

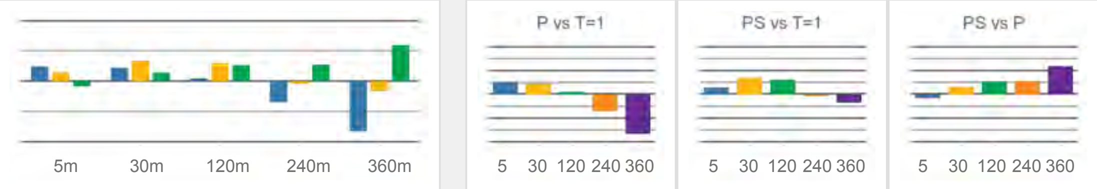

Locus		5m	30m	120m	240m	360m
**PGN_1843**	P vs T=1	0.403	0.427	0.091	-0.589	-1.437
rpsK	PS vs T=1	0.284	0.734	0.585	-0.134	-0.354
	PS vs P	-0.071	0.355	0.519	0.392	0.954
	30S ribosomal protein S11					
	protein synthesis					

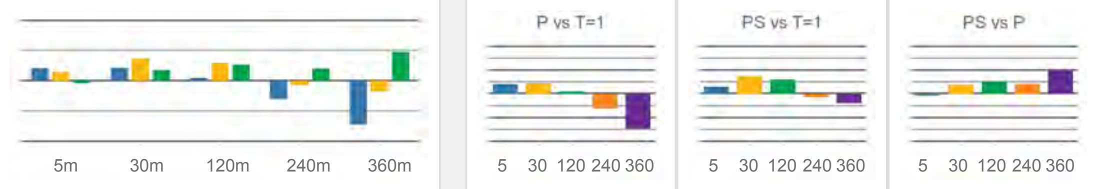

Locus		5m	30m	120m	240m	360m
**PGN_1844**	P vs T=1	0.443	0.487	0.204	-0.525	-1.432
rpsM	PS vs T=1	0.547	0.915	0.645	0.084	-0.266
	PS vs P	0.131	0.460	0.466	0.552	1.052
	30S ribosomal protein S13					
	protein synthesis					

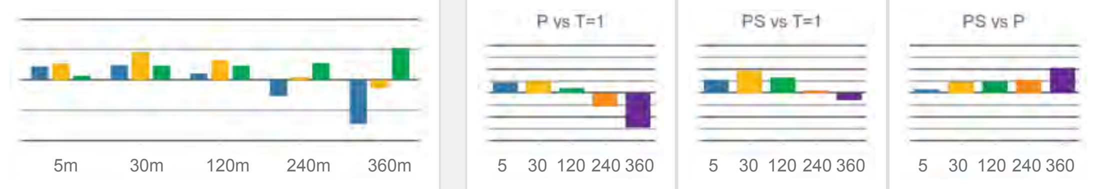

Locus		5m	30m	120m	240m	360m
**PGN_1845**	P vs T=1	0.634	0.632	0.492	-0.128	-1.188
rpmJ	PS vs T=1	0.730	1.142	1.066	0.617	0.403
	PS vs P	0.138	0.540	0.617	0.662	1.349
	50S ribosomal protein L36					
	protein synthesis					

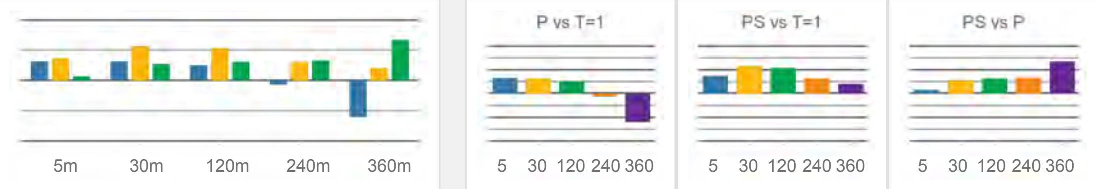

Locus		5m	30m	120m	240m	360m
**PGN_1846**	P vs T=1	0.870	0.906	0.615	-0.146	-1.179
	PS vs T=1	1.002	1.393	1.222	0.679	0.342
	PS vs P	0.183	0.540	0.646	0.718	1.291
	translation initiation factor IF-1					
	protein synthesis					

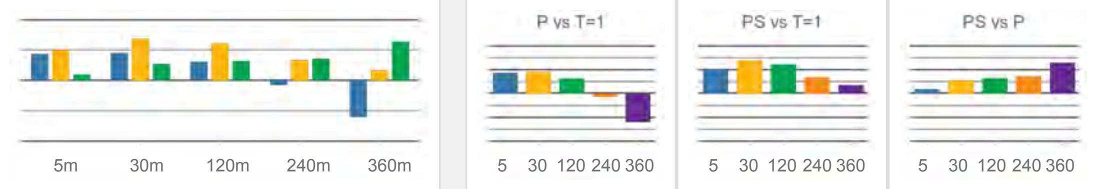

Locus		5m	30m	120m	240m	360m
**PGN_1847**	P vs T=1	0.142	0.179	-0.394	-1.376	-2.156
	PS vs T=1	-0.112	0.029	-0.497	-1.245	-1.493
	PS vs P	-0.198	-0.084	-0.071	0.069	0.570
	putative methionine aminopeptidase type I					
	protein fate					

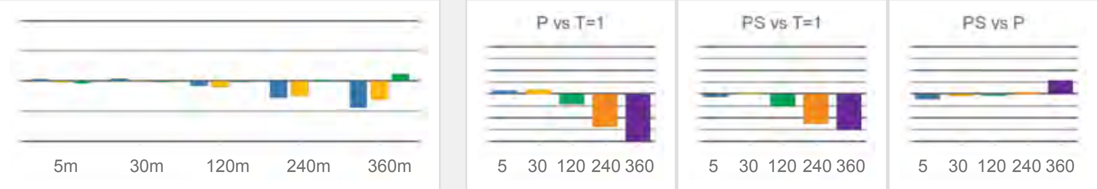

Locus		5m	30m	120m	240m	360m
**PGN_1848**	P vs T=1	0.439	0.732	0.506	-0.430	-1.551
	PS vs T=1	0.073	0.346	-0.169	-1.051	-1.380
	PS vs P	-0.303	-0.274	-0.574	-0.629	0.074
	preprotein translocase SecY subunit					
	protein fate					

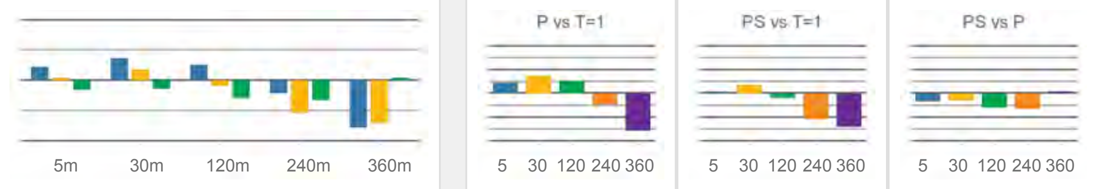

PgSg Time Course

log2 (Fold Change)

P vs T=1 | PS vs T=1 | PS vs P

Locus		5m	30m	120m	240m	360m
**PGN_1849**	P vs T=1	0.970	1.278	1.174	0.549	-0.566
rplO	PS vs T=1	0.792	1.198	0.926	0.164	0.085
	PS vs P	-0.119	0.048	-0.122	-0.369	0.495
	50S ribosomal protein L15					
	protein synthesis					

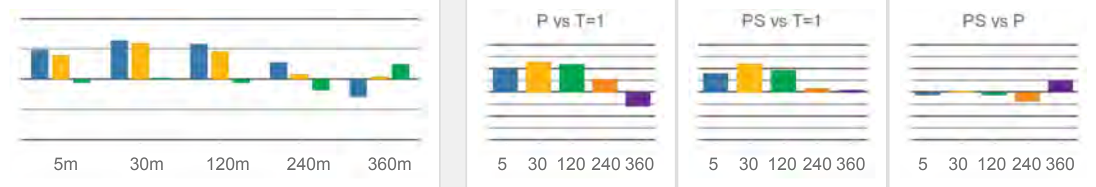

Locus		5m	30m	120m	240m	360m
**PGN_1850**	P vs T=1	1.339	1.751	1.924	1.436	0.480
rpmD	PS vs T=1	1.343	1.880	1.629	0.915	0.909
	PS vs P	0.021	0.238	-0.127	-0.449	0.322
	50S ribosomal protein L30					
	protein synthesis					

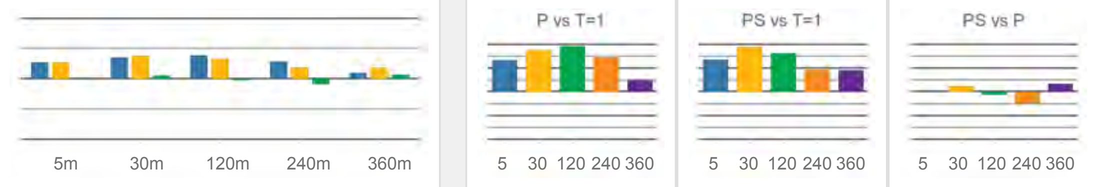

Locus		5m	30m	120m	240m	360m
**PGN_1851**	P vs T=1	1.446	1.761	1.954	1.391	0.320
rpsE	PS vs T=1	1.511	1.888	1.432	0.515	0.335
	PS vs P	0.086	0.186	-0.424	-0.832	-0.036
	30S ribosomal protein S5					
	protein synthesis					

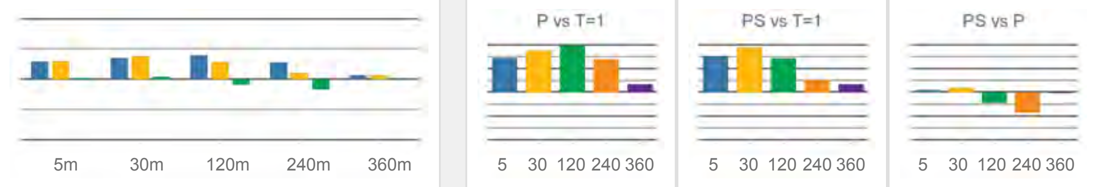

Locus		5m	30m	120m	240m	360m
**PGN_1852**	P vs T=1	1.326	1.688	1.876	1.334	0.207
rplR	PS vs T=1	1.365	1.825	1.416	0.867	0.746
	PS vs P	0.054	0.213	-0.330	-0.422	0.438
	50S ribosomal protein L18					
	protein synthesis					

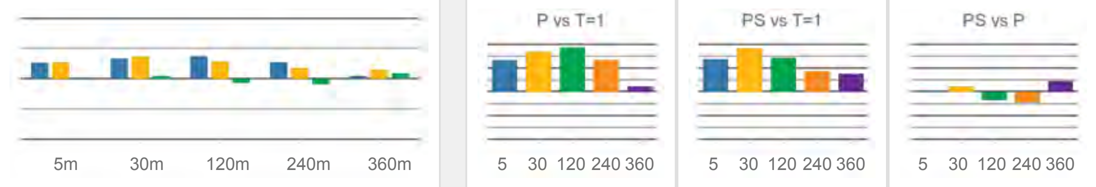

Locus		5m	30m	120m	240m	360m
**PGN_1853**	P vs T=1	1.532	1.887	2.074	1.609	0.404
rplF	PS vs T=1	1.603	2.050	1.768	1.330	1.208
	PS vs P	0.087	0.244	-0.171	-0.226	0.680
	50S ribosomal protein L6					
	protein synthesis					

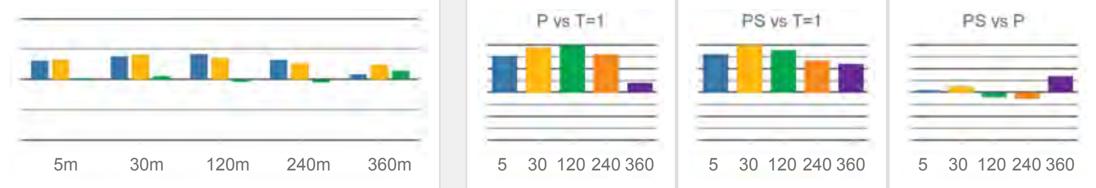

Locus		5m	30m	120m	240m	360m
**PGN_1854**	P vs T=1	1.825	2.091	1.814	0.794	-0.127
rpsH	PS vs T=1	1.765	2.138	1.664	0.754	0.404
	PS vs P	0.018	0.172	-0.037	-0.084	0.409
	30S ribosomal protein S8					
	protein synthesis					

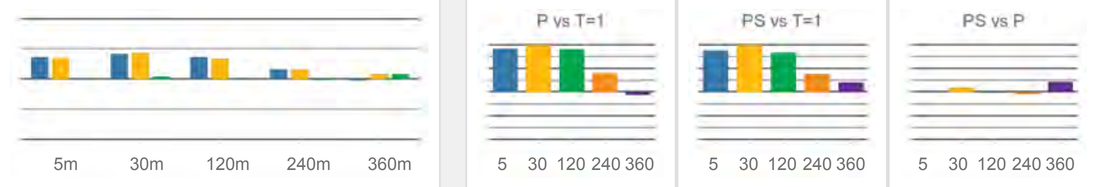

Locus		5m	30m	120m	240m	360m
**PGN_1855**	P vs T=1	0.355	0.292	-0.000	-0.947	-1.753
rpsN	PS vs T=1	0.066	0.157	-0.297	-0.767	-0.807
	PS vs P	-0.178	-0.033	-0.206	0.071	0.742
	30S ribosomal protein S14					
	protein synthesis					

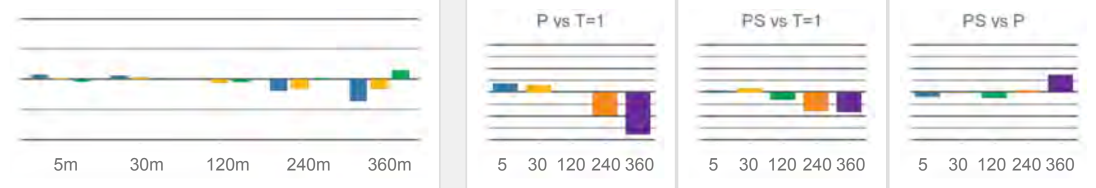

PgSg Time Course

log2 (Fold Change)

Legend: P vs T=1 | PS vs T=1 | PS vs P

Locus		5m	30m	120m	240m	360m
**PGN_1856**	P vs T=1	1.042	1.343	0.909	-0.198	-0.913
rplE	PS vs T=1	0.990	1.349	0.813	-0.073	-0.438
	PS vs P	0.004	0.117	-0.017	0.041	0.356
	50S ribosomal protein L5					
	protein synthesis					

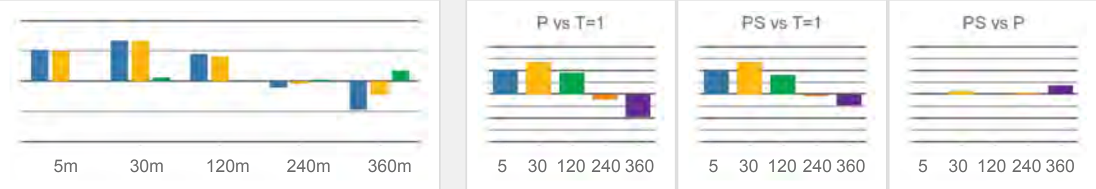

Locus		5m	30m	120m	240m	360m
**PGN_1857**	P vs T=1	1.540	1.804	1.157	0.156	-0.561
rplX	PS vs T=1	1.543	1.888	1.363	0.205	-0.072
	PS vs P	0.064	0.189	0.255	-0.029	0.377
	50S ribosomal protein L24					
	protein synthesis					

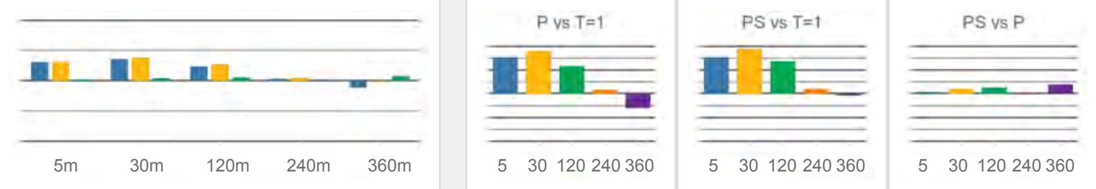

Locus		5m	30m	120m	240m	360m
**PGN_1858**	P vs T=1	1.391	1.676	0.923	0.363	-0.235
rplN	PS vs T=1	1.512	1.818	1.106	0.075	-0.100
	PS vs P	0.167	0.237	0.204	-0.316	0.067
	50S ribosomal protein L14					
	protein synthesis					

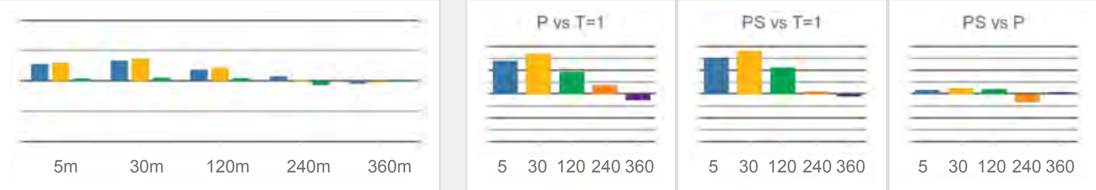

Locus		5m	30m	120m	240m	360m
**PGN_1859**	P vs T=1	1.938	2.343	2.413	2.200	1.489
rpsQ	PS vs T=1	2.130	2.526	2.220	1.714	1.761
	PS vs P	0.201	0.255	-0.106	-0.420	0.251
	30S ribosomal protein S17					
	protein synthesis					

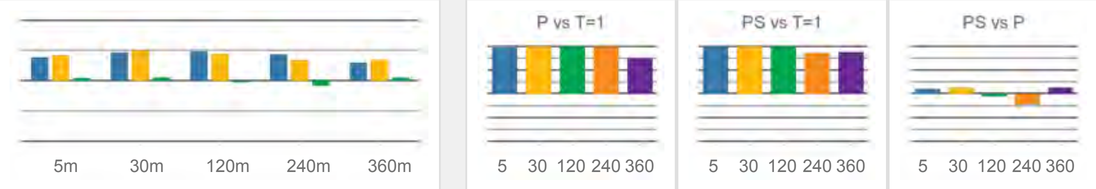

Locus		5m	30m	120m	240m	360m
**PGN_1860**	P vs T=1	1.756	2.217	2.116	1.557	0.690
rpmC	PS vs T=1	1.958	2.373	1.955	1.454	1.363
	PS vs P	0.210	0.255	-0.063	-0.097	0.563
	50S ribosomal protein L29					
	protein synthesis					

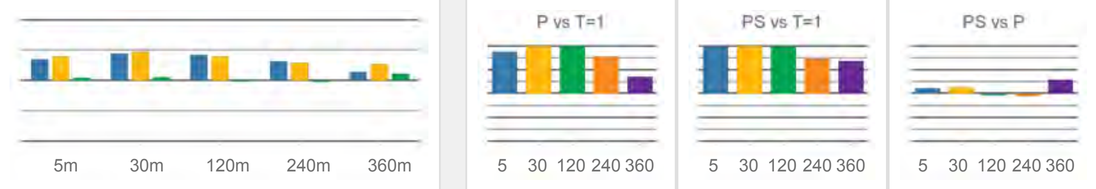

Locus		5m	30m	120m	240m	360m
**PGN_1861**	P vs T=1	1.474	2.159	2.166	1.471	0.529
rplP	PS vs T=1	1.709	2.156	1.856	1.314	1.190
	PS vs P	0.217	0.136	-0.161	-0.140	0.548
	50S ribosomal protein L16					
	protein synthesis					

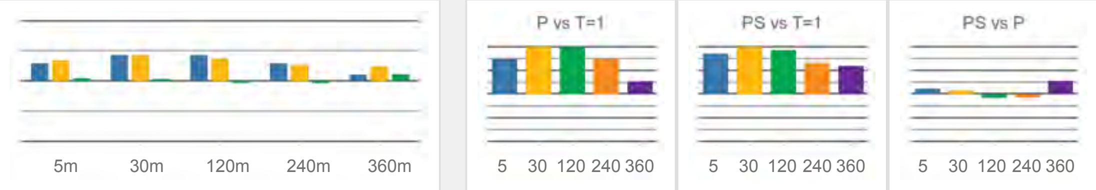

Locus		5m	30m	120m	240m	360m
**PGN_1862**	P vs T=1	1.341	1.979	2.017	1.380	0.479
rpsC	PS vs T=1	1.635	2.011	1.602	0.950	0.782
	PS vs P	0.280	0.154	-0.273	-0.393	0.226
	30S ribosomal protein S3					
	protein synthesis					

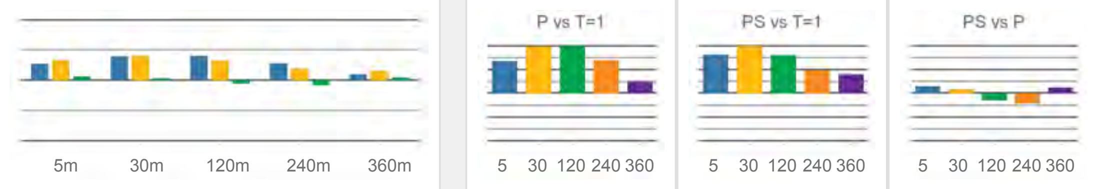

log₂ (Fold Change)

Locus		5m	30m	120m	240m	360m
**PGN_1863**	P vs T=1	1.510	2.041	1.999	1.445	0.477
rplV	PS vs T=1	1.662	1.964	1.391	0.973	0.743
	PS vs P	0.176	0.062	-0.462	-0.416	0.188
	50S ribosomal protein L22					
	protein synthesis					
**PGN_1864**	P vs T=1	1.442	2.181	1.951	1.301	0.612
rpsS	PS vs T=1	1.696	2.064	1.546	1.167	1.084
	PS vs P	0.244	0.074	-0.254	-0.129	0.369
	30S ribosomal protein S19					
	protein synthesis					
**PGN_1865**	P vs T=1	0.974	1.589	1.459	1.017	0.309
rplB	PS vs T=1	1.380	1.618	1.075	0.905	0.892
	PS vs P	0.369	0.150	-0.280	-0.096	0.497
	50S ribosomal protein L2					
	protein synthesis					
**PGN_1866**	P vs T=1	1.567	2.262	2.343	1.745	0.784
rplW	PS vs T=1	1.969	2.347	1.915	1.613	1.380
	PS vs P	0.371	0.180	-0.310	-0.105	0.508
	50S ribosomal protein L23					
	protein synthesis					
**PGN_1867**	P vs T=1	0.492	1.314	1.486	0.777	-0.314
rplD	PS vs T=1	0.616	0.948	0.590	0.172	0.210
	PS vs P	0.081	-0.225	-0.722	-0.560	0.412
	50S ribosomal protein L4					
	protein synthesis					
**PGN_1868**	P vs T=1	0.897	1.738	2.219	1.561	0.400
rplC	PS vs T=1	0.893	1.301	1.115	0.579	0.621
	PS vs P	-0.034	-0.322	-0.926	-0.899	0.164
	50S ribosomal protein L3					
	protein synthesis					
**PGN_1869**	P vs T=1	1.409	2.198	2.648	1.848	0.669
rpsJ	PS vs T=1	1.146	1.718	1.571	1.327	1.281
	PS vs P	-0.259	-0.254	-0.744	-0.408	0.458
	30S ribosomal protein S10					
	protein synthesis					

P vs T=1 · PS vs T=1 · PS vs P

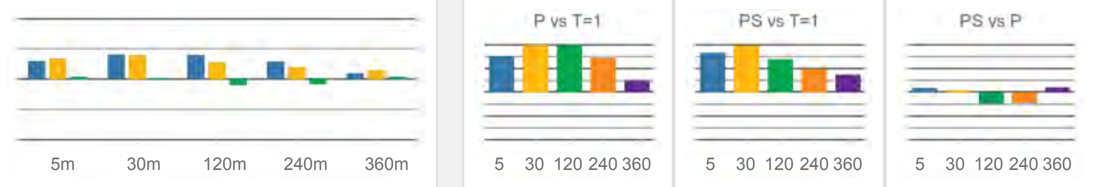

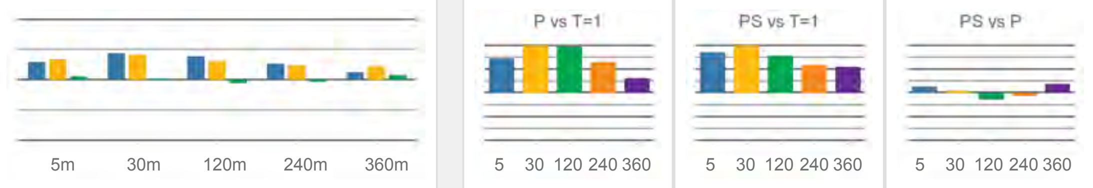

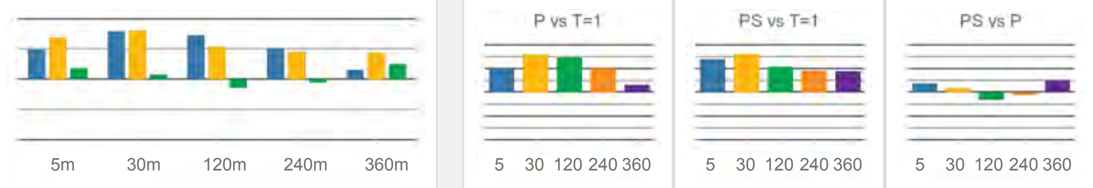

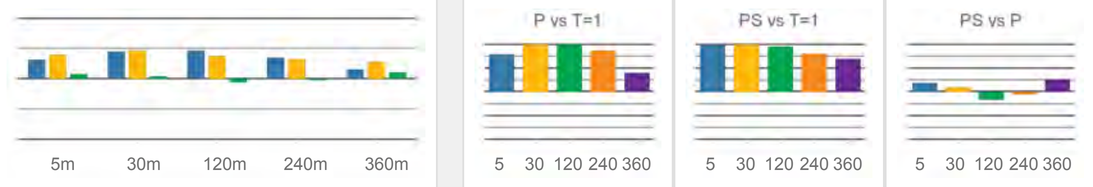

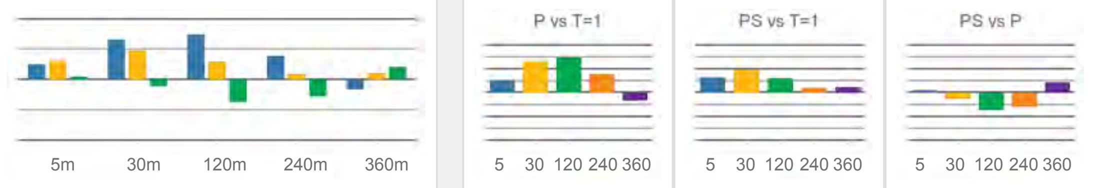

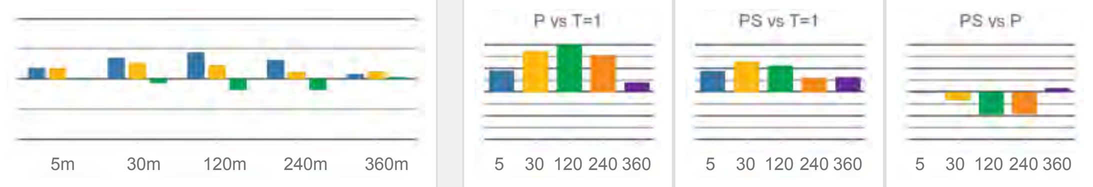

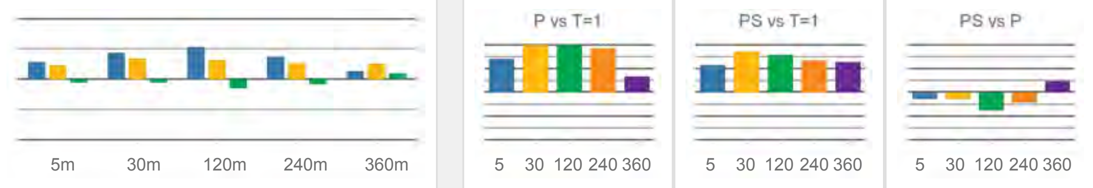

PgSg Time Course

log2 (Fold Change)

Legend: P vs T=1 | PS vs T=1 | PS vs P

Locus		5m	30m	120m	240m	360m
**PGN_1870**	P vs T=1	0.428	0.731	0.744	0.388	0.095
	PS vs T=1	0.052	-0.234	-0.690	-0.635	-0.287
	PS vs P	-0.321	-0.846	-1.321	-0.964	-0.368
	translation elongation factor G					
	protein synthesis					

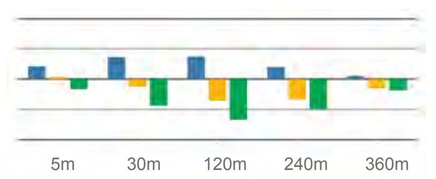

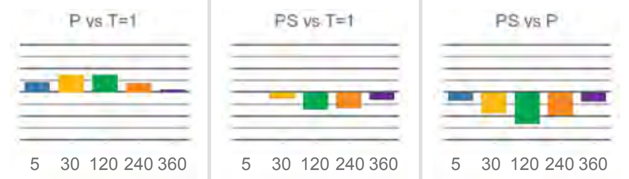

Locus		5m	30m	120m	240m	360m
**PGN_1871**	P vs T=1	0.377	0.390	0.461	0.321	-0.097
rpsG	PS vs T=1	-0.339	-0.381	-0.735	-0.754	-0.413
	PS vs P	-0.670	-0.720	-1.129	-1.005	-0.311
	30S ribosomal protein S7					
	protein synthesis					

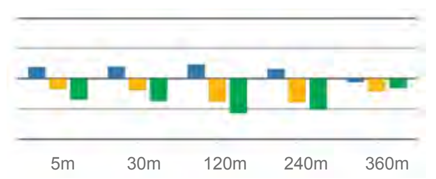

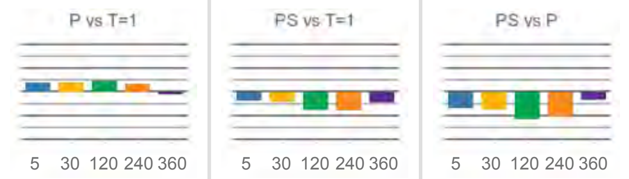

Locus		5m	30m	120m	240m	360m
**PGN_1872**	P vs T=1	0.529	0.378	-0.052	-0.270	-0.136
rpsL	PS vs T=1	-0.594	-0.972	-1.308	-1.050	-0.593
	PS vs P	-1.011	-1.236	-1.195	-0.747	-0.419
	30S ribosomal protein S12					
	protein synthesis					

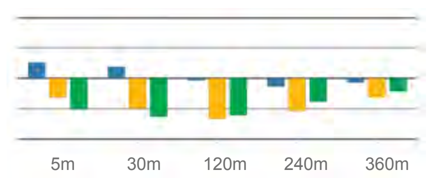

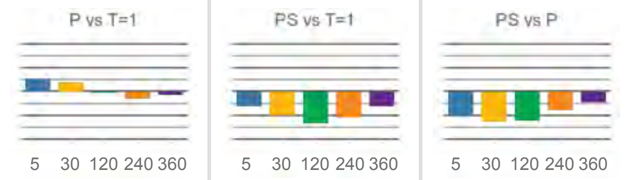

Locus		5m	30m	120m	240m	360m
**PGN_1873**	P vs T=1	0.002	-0.254	-0.127	0.111	0.414
	PS vs T=1	0.084	-0.309	0.145	0.535	0.311
	PS vs P	0.080	-0.081	0.245	0.417	-0.087
	conserved hypothetical protein					
	hypothetical proteins-Conserved					

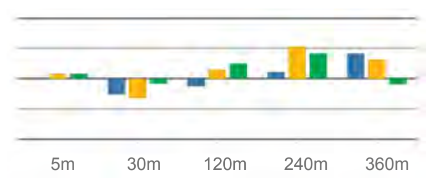

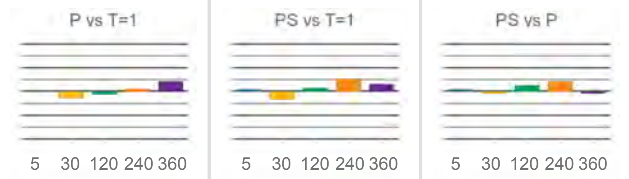

Locus		5m	30m	120m	240m	360m
**PGN_1874**	P vs T=1	-1.003	-0.705	-0.138	0.150	-0.209
	PS vs T=1	-0.183	-0.214	-0.149	0.056	0.153
	PS vs P	0.688	0.410	-0.007	-0.054	0.341
	putative 3-phosphoshikimate 1-carboxyvinyltransferase					
	amino acid biosynthesis					

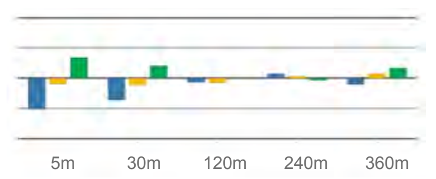

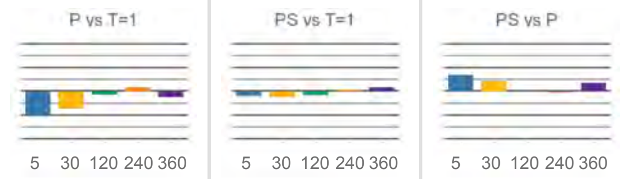

Locus		5m	30m	120m	240m	360m
**PGN_1875**	P vs T=1	0.292	0.738	1.032	0.925	0.814
	PS vs T=1	0.904	1.004	0.881	0.876	0.846
	PS vs P	0.545	0.256	-0.120	-0.041	0.026
	conserved hypothetical protein					
	hypothetical proteins-Conserved					

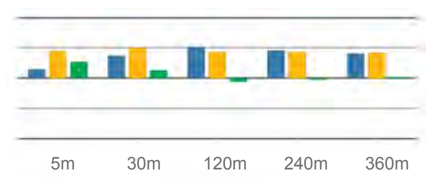

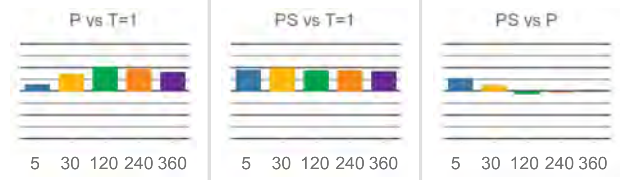

Locus		5m	30m	120m	240m	360m
**PGN_1876**	P vs T=1	-0.145	-0.228	0.319	0.700	1.567
	PS vs T=1	-0.127	-0.060	-0.244	-0.087	0.236
	PS vs P	-0.021	0.085	-0.522	-0.653	-1.123
	putative ABC transporter membrane protein					
	transport and binding proteins					

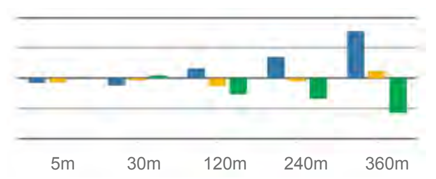

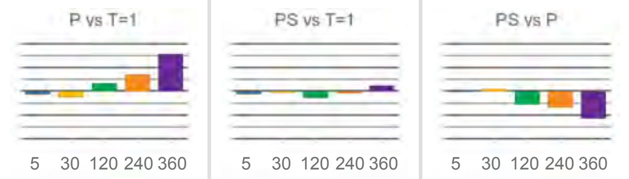

log₂ (Fold Change)

Legend: P vs T=1 | PS vs T=1 | PS vs P

Locus		5m	30m	120m	240m	360m
**PGN_1877**	P vs T=1	-0.542	-0.432	-0.512	-0.496	-0.350
porW	PS vs T=1	0.168	-0.073	-1.008	-1.306	-1.275
	PS vs P	0.699	0.357	-0.494	-0.801	-0.914
	Por secretion system protein porW/sprE					
	unknown function					
**PGN_1878**	P vs T=1	0.042	0.326	0.251	0.077	0.048
	PS vs T=1	0.712	0.564	-0.213	-0.748	-0.677
	PS vs P	0.638	0.253	-0.441	-0.811	-0.714
	conserved hypothetical protein					
	hypothetical proteins-Conserved					
**PGN_1879**	P vs T=1	0.940	1.776	2.230	2.071	1.782
	PS vs T=1	1.415	1.719	1.904	1.593	1.203
	PS vs P	0.378	-0.015	-0.213	-0.370	-0.523
	hypothetical protein					
	hypothetical proteins					
**PGN_1880**	P vs T=1	0.456	1.090	1.382	1.236	0.624
	PS vs T=1	1.024	1.126	0.940	0.890	0.705
	PS vs P	0.555	0.040	-0.432	-0.340	0.075
	malate dehydrogenase					
	energy metabolism					
**PGN_1881**	P vs T=1	-1.047	-0.705	-0.538	-0.278	0.511
	PS vs T=1	-1.324	-0.872	-0.426	-0.402	-0.054
	PS vs P	-0.406	-0.187	0.074	-0.080	-0.320
	conserved hypothetical protein					
	hypothetical proteins-Conserved					
**PGN_1882**	P vs T=1	-0.089	0.008	-0.168	-0.624	-1.115
	PS vs T=1	-0.717	-0.511	-0.390	-0.239	-0.240
	PS vs P	-0.604	-0.484	-0.205	0.355	0.824
	probable permease					
	cell envelope					
**PGN_1883**	P vs T=1	-0.493	-0.621	-1.079	-1.013	-1.024
	PS vs T=1	-0.253	-0.375	-0.663	-0.287	-0.508
	PS vs P	0.240	0.242	0.387	0.699	0.499
	glutaminyl-tRNA synthetase					
	protein synthesis					

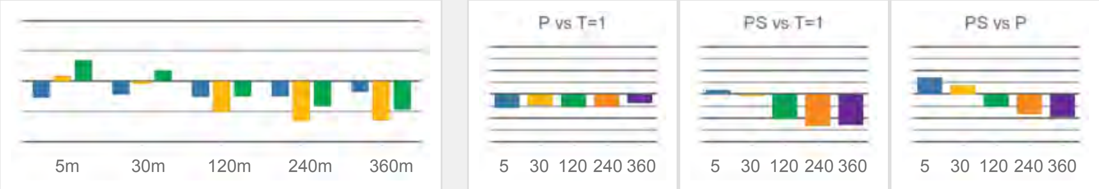

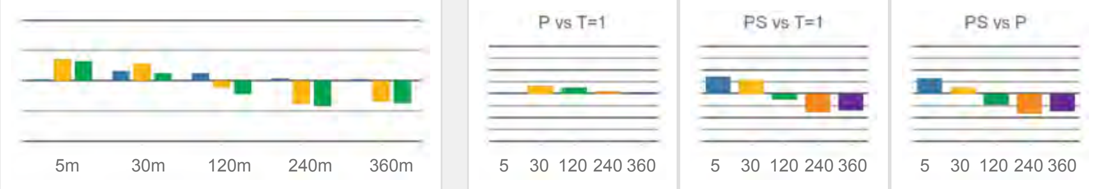

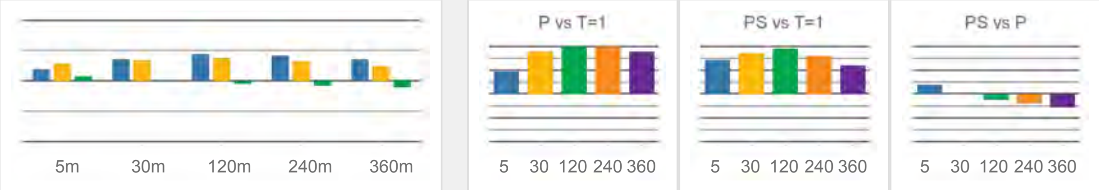

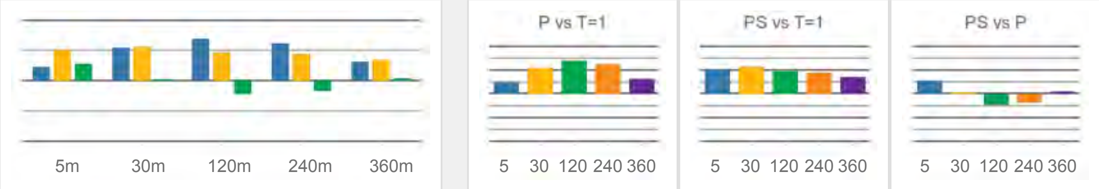

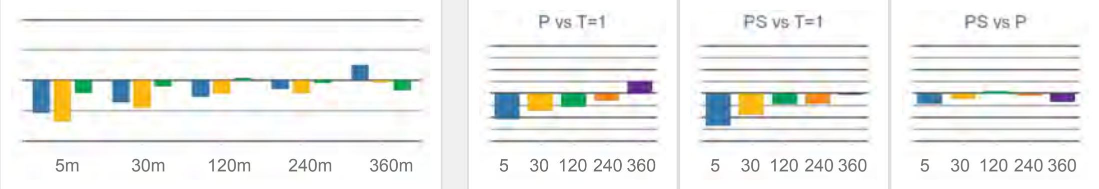

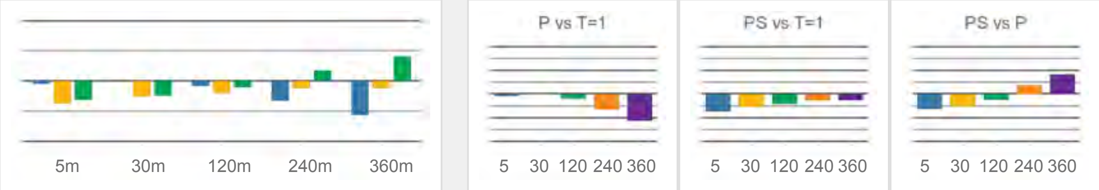

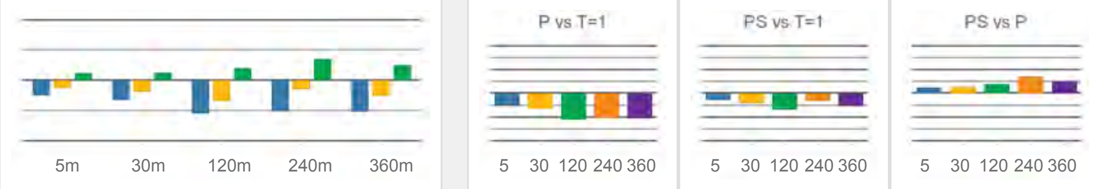

PgSg Time Course

log2 (Fold Change)

Legend: P vs T=1, PS vs T=1, PS vs P

Locus		5m	30m	120m	240m	360m
**PGN_1884**	P vs T=1	0.313	0.210	-0.231	-0.448	-0.061
	PS vs T=1	0.898	1.115	0.650	0.369	-0.017
	PS vs P	0.577	0.885	0.812	0.680	0.031

probable alkaline phosphatase

unknown function

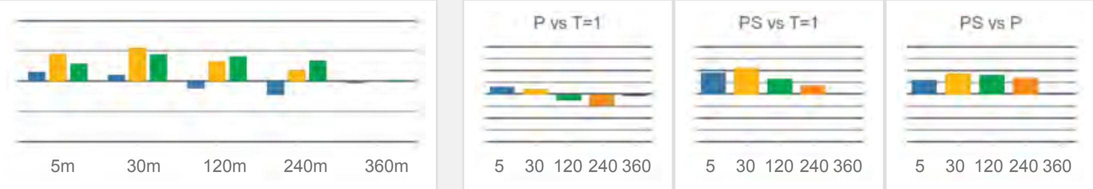

Locus		5m	30m	120m	240m	360m
**PGN_1885**	P vs T=1	-0.713	-0.201	-0.294	-0.864	-1.646
	PS vs T=1	-0.452	0.242	0.158	-0.580	-0.986
	PS vs P	0.236	0.458	0.470	0.264	0.606

conserved hypothetical protein

unknown function

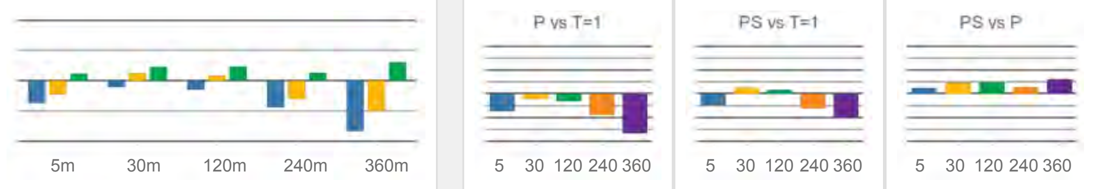

Locus		5m	30m	120m	240m	360m
**PGN_1886**	P vs T=1	-0.512	-0.426	-0.370	-0.180	0.172
	PS vs T=1	0.171	0.007	-0.255	-0.261	-0.197
	PS vs P	0.650	0.409	0.095	-0.075	-0.338

putative NAD dependent epimerase

unknown function

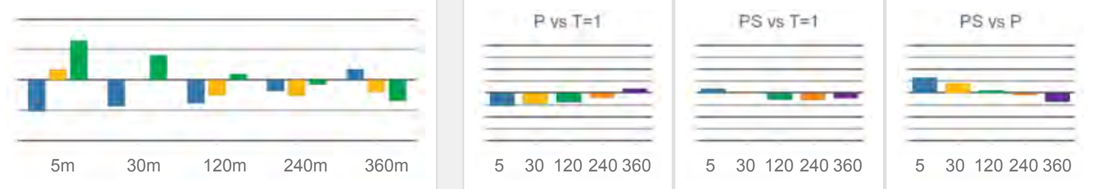

Locus		5m	30m	120m	240m	360m
**PGN_1887**	P vs T=1	-0.991	-0.795	-0.479	-0.566	-0.314
	PS vs T=1	-0.740	-0.397	-0.382	-0.270	-0.331
	PS vs P	0.040	0.245	0.099	0.171	0.011

hypothetical protein

hypothetical proteins

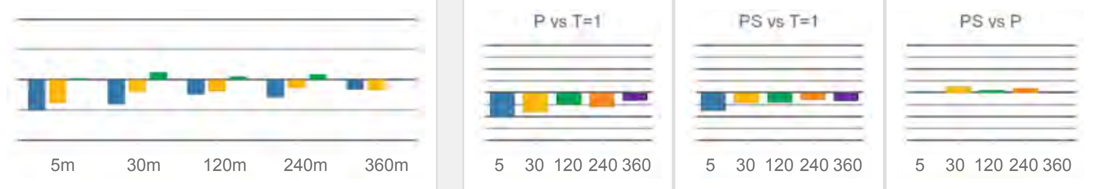

Locus		5m	30m	120m	240m	360m
**PGN_1888**	P vs T=1	0.459	0.671	0.881	1.036	0.932
	PS vs T=1	0.302	0.207	0.217	0.294	0.388
	PS vs P	-0.154	-0.445	-0.638	-0.702	-0.523

4-hydroxybutyrate CoA-transferase

energy metabolism

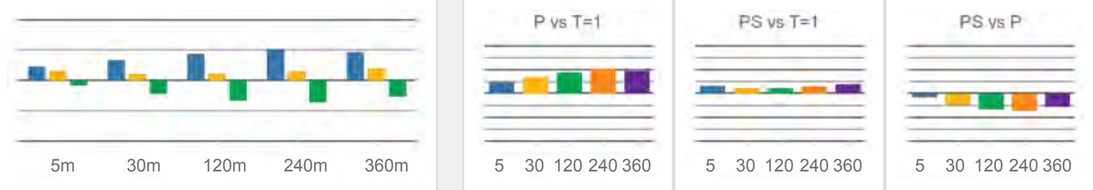

Locus		5m	30m	120m	240m	360m
**PGN_1889**	P vs T=1	-0.417	-0.529	-0.989	-1.618	-2.257
	PS vs T=1	-0.924	-0.461	-0.944	-1.740	-1.736
	PS vs P	-0.450	0.110	0.062	-0.176	0.423

conserved hypothetical protein

hypothetical proteins-Conserved

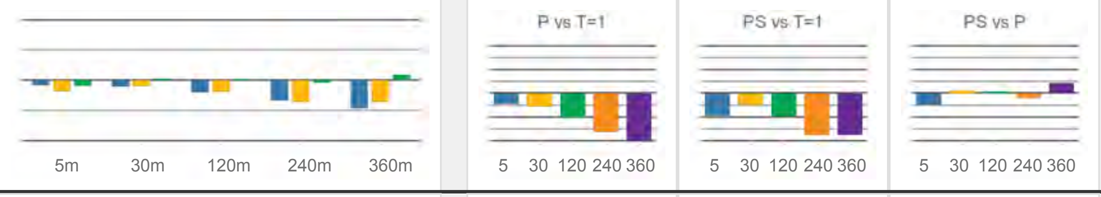

Locus		5m	30m	120m	240m	360m
**PGN_1890** *rpmG*	P vs T=1	0.731	0.634	0.087	-0.673	-1.464
	PS vs T=1	0.366	0.766	0.432	-0.518	-0.561
	PS vs P	-0.256	0.221	0.378	0.083	0.766

50S ribosomal protein L33

protein synthesis

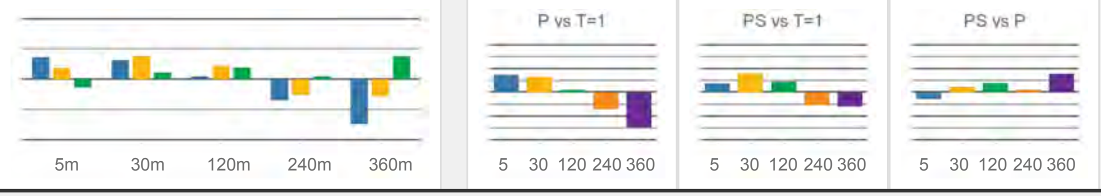

PgSg Time Course

Hackett Laboratory UW

log₂ (Fold Change) legend: P vs T=1 · PS vs T=1 · PS vs P

Locus		5m	30m	120m	240m	360m
**PGN_1891**	P vs T=1	0.368	0.346	-0.142	-0.862	-1.607
rpmB	PS vs T=1	0.124	0.507	0.283	-0.469	-0.574
	PS vs P	-0.183	0.219	0.443	0.325	0.919
	50S ribosomal protein L28					
	protein synthesis					
**PGN_1892**	P vs T=1	-1.064	-1.277	-2.029	-1.932	-1.297
	PS vs T=1	-1.164	-1.419	-1.913	-2.249	-1.925
	PS vs P	-0.082	-0.130	0.066	-0.353	-0.608
	conserved hypothetical protein					
	hypothetical proteins-Conserved					
**PGN_1893**	P vs T=1	-1.966	-1.317	-1.038	-0.997	-1.224
	PS vs T=1	-1.364	-1.185	-1.068	-0.821	-0.816
	PS vs P	0.394	0.097	-0.022	0.179	0.362
	hypothetical protein					
	hypothetical proteins					
**PGN_1894**	P vs T=1	-0.103	-0.436	0.631	1.757	2.351
	PS vs T=1	-0.816	-0.303	1.074	1.317	1.333
	PS vs P	-0.632	-0.310	0.363	0.332	0.142
	conserved hypothetical protein					
	hypothetical proteins-Conserved					
**PGN_1895**	P vs T=1	-0.432	-0.675	-0.883	-1.098	-0.690
	PS vs T=1	-0.235	-0.406	-0.168	0.358	0.063
	PS vs P	0.192	0.225	0.623	1.247	0.707
	conserved hypothetical protein					
	unknown function					
**PGN_1896**	P vs T=1	-0.964	-0.889	-0.656	-0.071	0.351
wbaP	PS vs T=1	-0.891	-1.050	-1.048	-0.463	-0.499
	PS vs P	0.041	-0.185	-0.410	-0.299	-0.765
	galactose-1-phosphate transferase					
	cell envelope					
**PGN_1897**	P vs T=1	-1.001	-0.947	-0.823	-0.650	-0.318
	PS vs T=1	-1.147	-1.523	-1.513	-1.483	-1.021
	PS vs P	-0.146	-0.565	-0.670	-0.784	-0.659
	putative transport related membrane protein					
	transport and binding proteins					

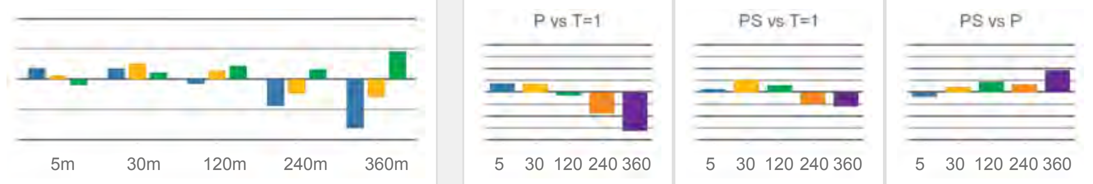

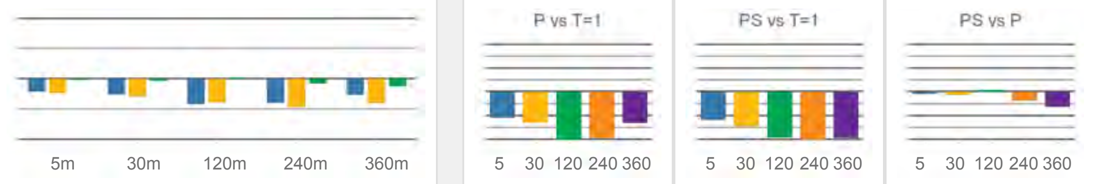

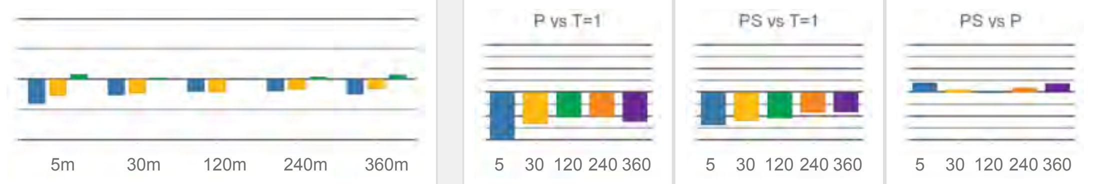

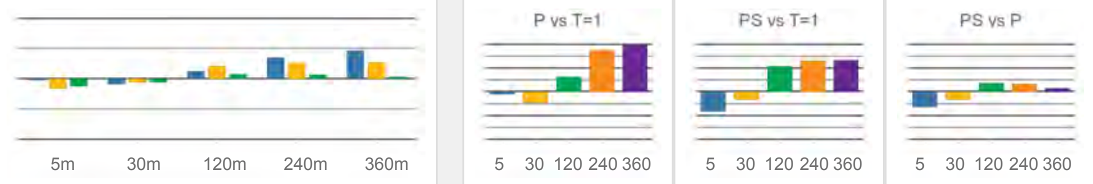

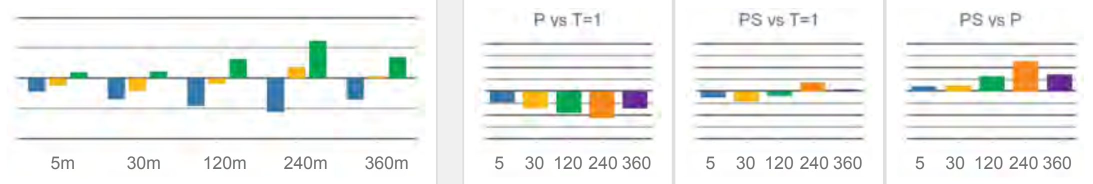

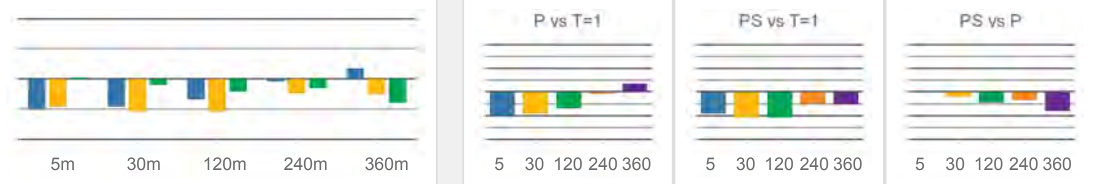

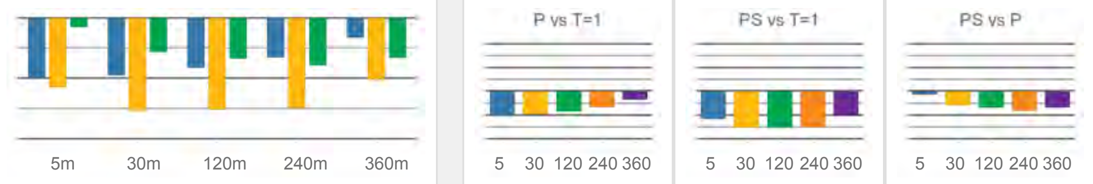

PgSg Time Course

Hackett Laboratory UW

log$_2$ (Fold Change)

Locus		5m	30m	120m	240m	360m
**PGN_1898**	P vs T=1	-0.269	-0.239	0.019	0.389	1.525
	PS vs T=1	-0.634	-0.658	-0.410	0.499	0.529
	PS vs P	-0.373	-0.426	-0.428	0.160	-0.842
	probable transport protein					
	transport and binding proteins					
**PGN_1899**	P vs T=1	-0.056	0.425	1.248	1.597	1.413
	PS vs T=1	0.375	0.498	1.108	1.863	1.766
	PS vs P	0.293	0.011	-0.102	0.335	0.369
	conserved hypothetical protein					
	hypothetical proteins-Conserved					
**PGN_1900**	P vs T=1	0.159	-0.050	0.065	-0.070	-0.209
	PS vs T=1	0.483	0.648	1.065	1.011	0.949
	PS vs P	0.296	0.617	0.936	0.945	1.042
	hypothetical protein					
	hypothetical proteins					
**PGN_1901**	P vs T=1	0.367	0.303	0.346	0.400	0.013
	PS vs T=1	0.204	0.022	0.134	0.212	0.300
	PS vs P	-0.161	-0.285	-0.217	-0.188	0.260
	transposase in ISPg1					
**PGN_1902**	P vs T=1	-1.391	-2.362	-3.020	-3.283	-3.165
	PS vs T=1	-0.797	-1.196	-1.288	-1.358	-1.732
	PS vs P	0.633	0.990	1.458	1.548	1.211
	conserved hypothetical protein					
	hypothetical proteins-Conserved					
**PGN_1903**	P vs T=1	-0.547	-1.600	-2.502	-3.000	-2.937
	PS vs T=1	-0.176	-0.491	-0.460	-0.569	-1.104
	PS vs P	0.474	0.984	1.790	2.035	1.597
	putative adenine-specific DNA methyltransferase					
	DNA metabolism					
**PGN_1904** *hagB*	P vs T=1	-1.273	-0.789	0.254	0.782	0.683
	PS vs T=1	0.422	0.684	1.226	1.740	1.705
	PS vs P	1.387	1.247	0.925	0.973	1.004
	hemagglutinin protein HagB					
	cellular processes					

P vs T=1 | PS vs T=1 | PS vs P

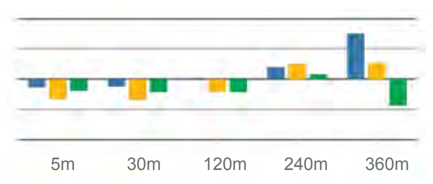
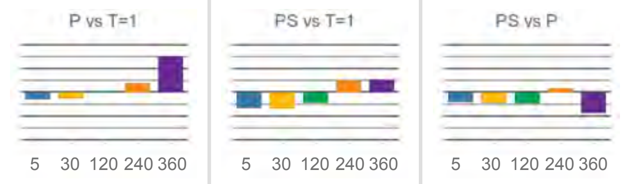
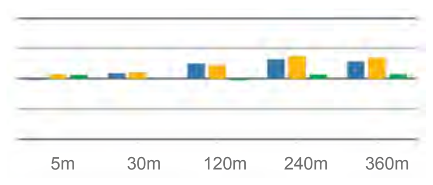
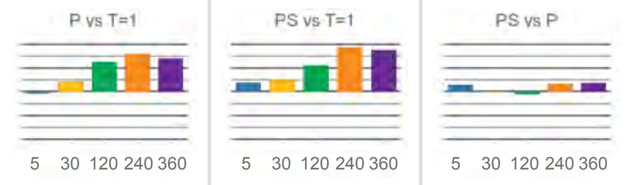
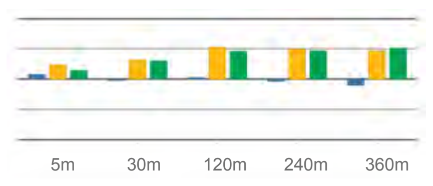
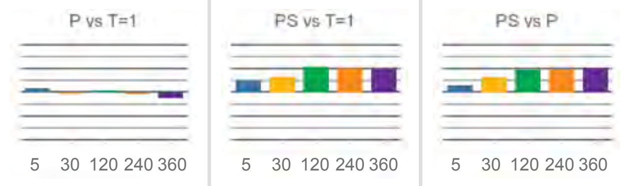
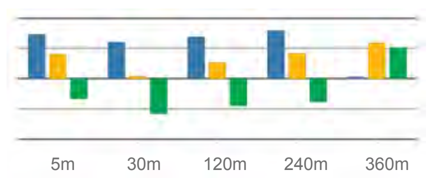
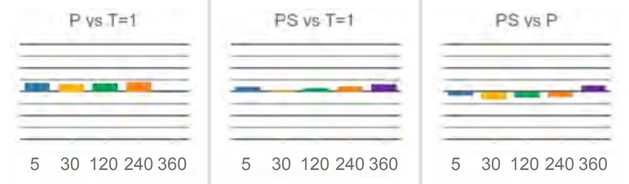
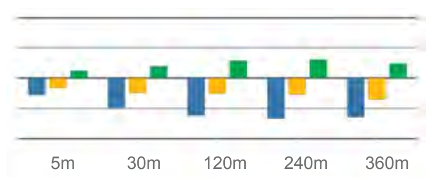
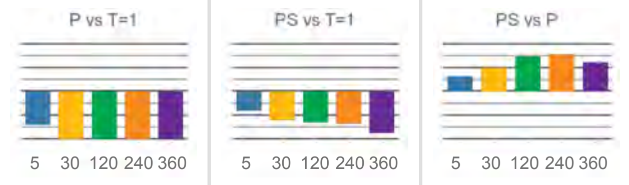
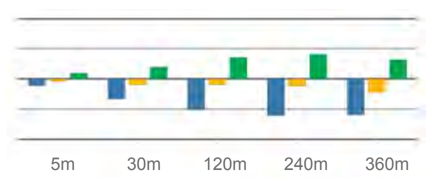
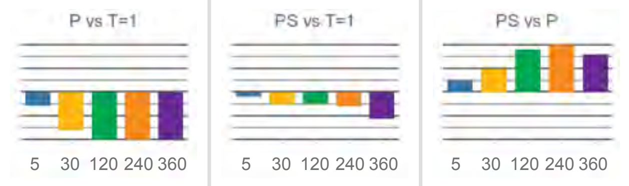
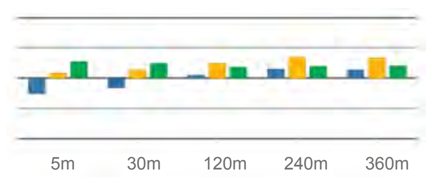
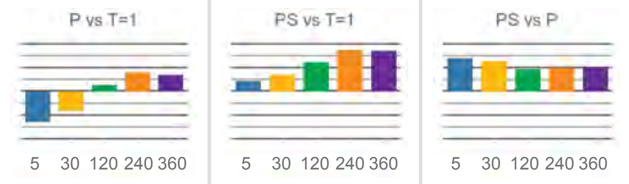

log$_2$ (Fold Change)

Locus		5m	30m	120m	240m	360m
**PGN_1905**	P vs T=1	-1.300	-1.230	-1.188	-1.786	-2.270
	PS vs T=1	-1.589	-1.141	-1.357	-1.942	-1.697
	PS vs P	-0.267	0.126	-0.064	-0.232	0.393

conserved hypothetical protein

hypothetical proteins-Conserved

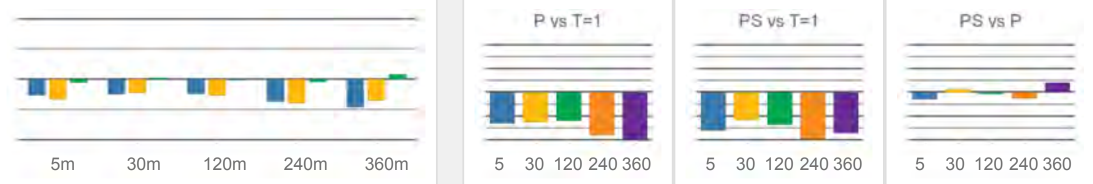

Locus		5m	30m	120m	240m	360m
**PGN_1906** *hagC*	P vs T=1	-1.275	-0.856	0.249	0.786	0.682
	PS vs T=1	0.435	0.644	1.258	1.756	1.727
	PS vs P	1.390	1.251	0.960	0.985	1.026

hemagglutinin protein HagC

cellular processes

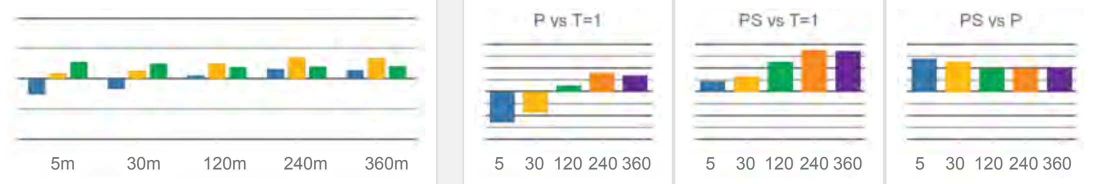

Locus		5m	30m	120m	240m	360m
**PGN_1907**	P vs T=1	-0.869	-0.808	0.332	1.239	1.926
	PS vs T=1	-1.025	-0.742	-0.220	0.270	0.950
	PS vs P	-0.277	-0.093	-0.486	-0.718	-0.770

conserved hypothetical protein

hypothetical proteins-Conserved

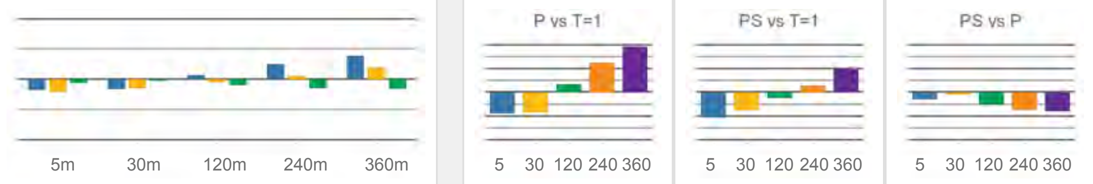

Locus		5m	30m	120m	240m	360m
**PGN_1908**	P vs T=1	0.404	0.843	1.339	1.944	3.089
	PS vs T=1	0.220	0.596	0.471	1.213	2.554
	PS vs P	-0.294	-0.129	-0.465	-0.062	0.430

hypothetical protein

hypothetical proteins

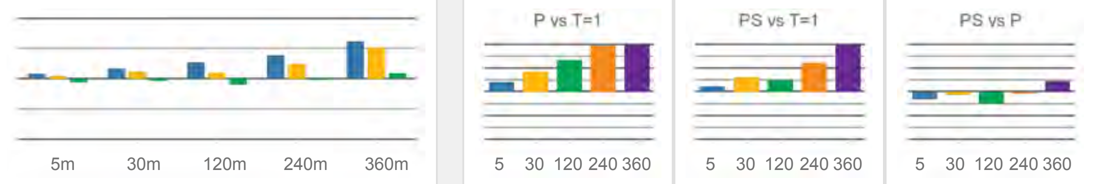

Locus		5m	30m	120m	240m	360m
**PGN_1909**	P vs T=1	-0.494	-0.961	0.155	0.163	2.263
	PS vs T=1	-0.069	-0.343	-0.191	0.374	0.836
	PS vs P	0.203	0.012	-0.150	0.128	-0.380

conserved hypothetical protein

hypothetical proteins-Conserved

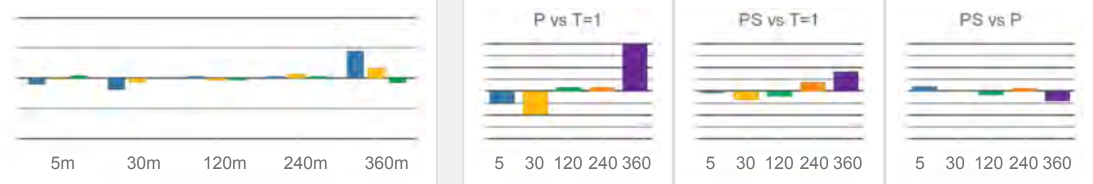

Locus		5m	30m	120m	240m	360m
**PGN_1910**	P vs T=1	0.900	1.396	1.993	2.187	1.872
	PS vs T=1	-1.084	-0.850	-0.845	0.034	-0.003
	PS vs P	-1.820	-1.979	-2.526	-1.817	-1.663

conserved hypothetical protein

hypothetical proteins-Conserved

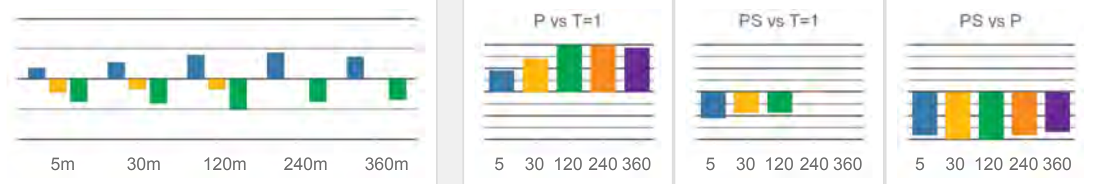

Locus		5m	30m	120m	240m	360m
**PGN_1911**	P vs T=1	0.288	-0.394	-0.012	0.624	1.534
	PS vs T=1	0.012	0.034	0.571	0.484	0.801
	PS vs P	-0.259	0.285	0.474	-0.104	-0.616

transposase in ISPg3

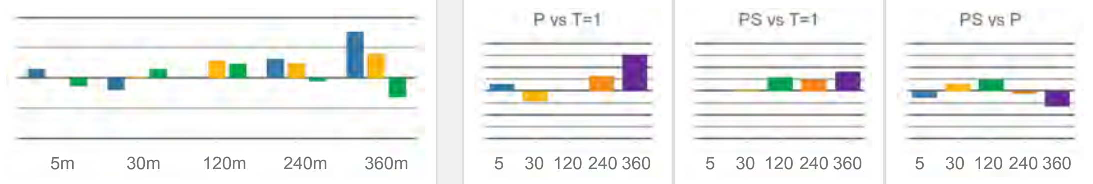

PgSg Time Course

Locus		log$_2$ (Fold Change) 5m	30m	120m	240m	360m
PGN_1912	P vs T=1	0.440	0.209	0.576	1.796	2.852
	PS vs T=1	-0.581	-0.487	0.422	1.061	1.003
	PS vs P	-0.917	-0.699	-0.230	-0.316	-1.380

partial transposase in ISPg6

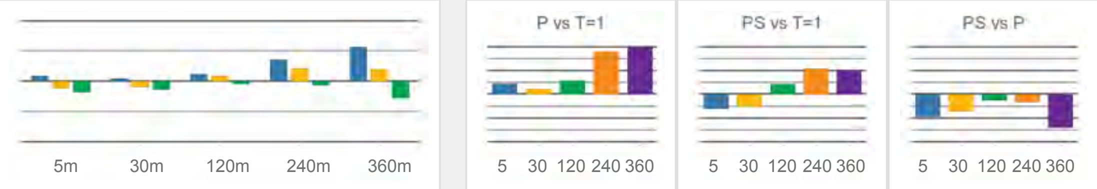

Locus		5m	30m	120m	240m	360m
PGN_1913	P vs T=1	0.338	-0.290	-0.599	0.680	1.514
	PS vs T=1	0.000	-0.065	0.271	0.478	0.682
	PS vs P	-0.312	0.147	0.593	-0.115	-0.723

transposase in ISPg3

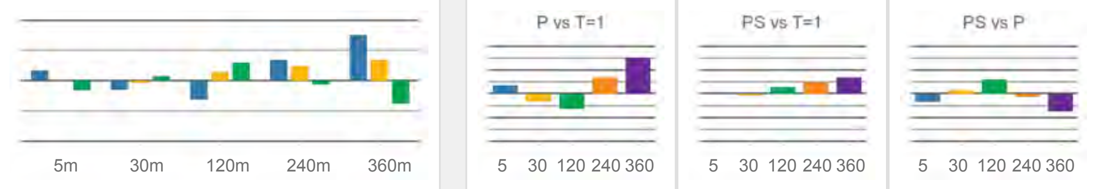

Locus		5m	30m	120m	240m	360m
PGN_1914	P vs T=1	-0.313	-0.726	-1.026	-1.547	-2.278
	PS vs T=1	-1.033	-1.175	-1.272	-1.374	-1.466
	PS vs P	-0.667	-0.431	-0.241	0.140	0.749

carboxyl-terminal processing protease

protein fate

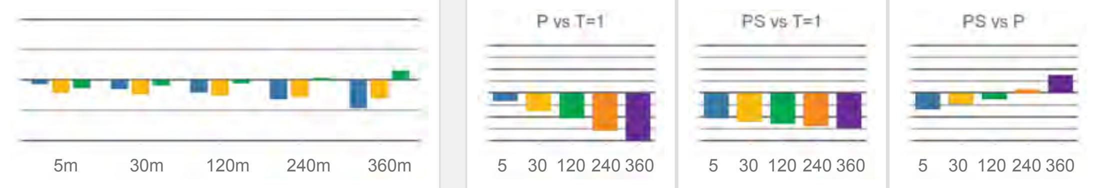

Locus		5m	30m	120m	240m	360m
PGN_1915	P vs T=1	0.408	0.194	0.520	1.598	2.650
	PS vs T=1	0.794	0.605	1.073	1.593	1.965
	PS vs P	0.285	0.187	0.268	0.156	-0.432

conserved hypothetical protein

hypothetical proteins-Conserved

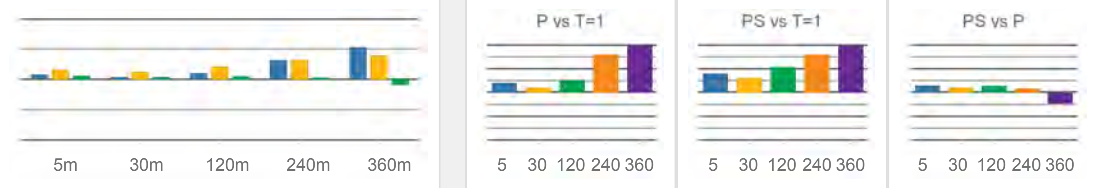

Locus		5m	30m	120m	240m	360m
PGN_1916	P vs T=1	0.267	0.033	0.164	0.949	2.085
	PS vs T=1	0.020	0.105	0.398	0.710	1.122
	PS vs P	-0.254	-0.001	0.112	-0.166	-0.804

putative ABC transporter ATP-binding protein

transport and binding proteins

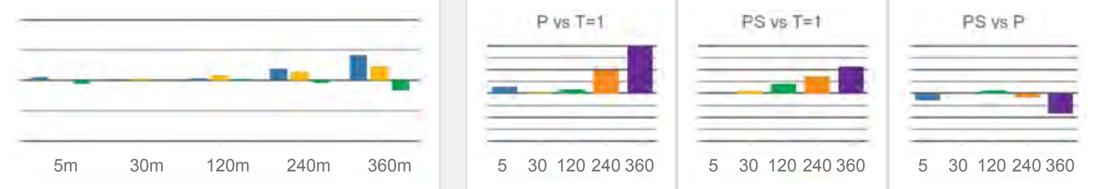

Locus		5m	30m	120m	240m	360m
PGN_1917	P vs T=1	0.706	0.540	0.651	1.465	2.590
	PS vs T=1	0.582	0.435	1.003	0.937	1.173
	PS vs P	-0.127	-0.163	0.231	-0.386	-1.160

putative ABC transporter ATP-binding protein

transport and binding proteins

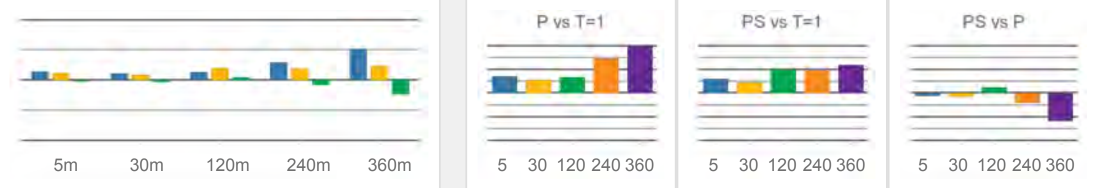

Locus		5m	30m	120m	240m	360m
PGN_1918	P vs T=1	0.491	0.631	1.257	1.970	2.319
	PS vs T=1	0.345	0.608	0.876	1.020	1.294
	PS vs P	-0.207	-0.083	-0.330	-0.686	-0.810

conserved hypothetical protein

hypothetical proteins-Conserved

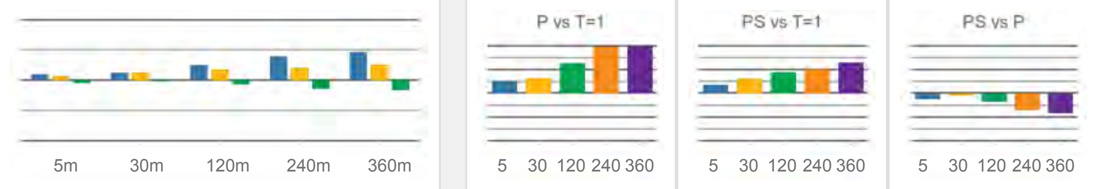

PgSg Time Course

log2 (Fold Change)

Legend: P vs T=1 | PS vs T=1 | PS vs P

Locus		5m	30m	120m	240m	360m
**PGN_1919**	P vs T=1	0.511	0.947	1.593	1.501	2.228
	PS vs T=1	0.304	0.483	0.565	0.879	1.525
	PS vs P	-0.273	-0.414	-0.814	-0.479	-0.460
	conserved hypothetical protein					
	hypothetical proteins-Conserved					

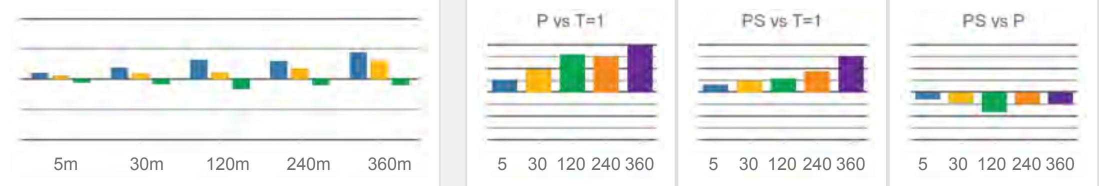

Locus		5m	30m	120m	240m	360m
**PGN_1920**	P vs T=1	0.491	0.446	0.716	1.455	2.633
	PS vs T=1	-0.142	0.037	0.472	1.058	1.202
	PS vs P	-0.617	-0.425	-0.277	-0.249	-1.171
	conserved hypothetical protein					
	cell envelope					

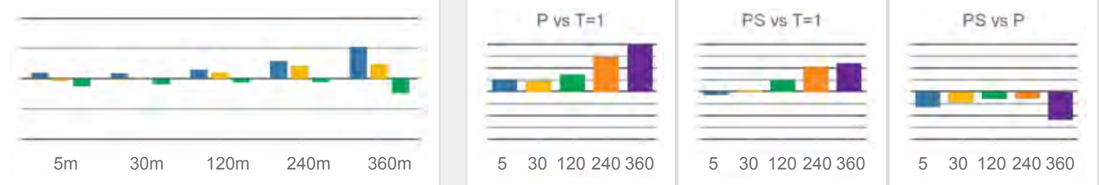

Locus		5m	30m	120m	240m	360m
**PGN_1921**	P vs T=1	0.087	-0.485	-0.179	0.755	2.062
	PS vs T=1	-0.250	-0.785	0.132	0.390	0.826
	PS vs P	-0.289	-0.441	0.123	-0.150	-0.886
	probable transcriptional regulator					
	regulatory functions					

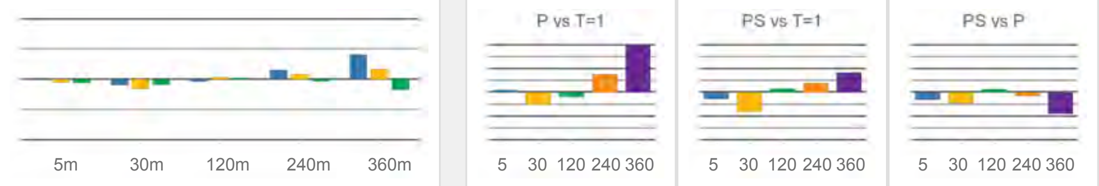

Locus		5m	30m	120m	240m	360m
**PGN_1922**	P vs T=1	0.157	-0.687	-0.630	0.045	1.133
	PS vs T=1	-0.323	-0.447	-0.129	0.056	0.281
	PS vs P	-0.433	0.138	0.352	0.020	-0.738
	transposase in ISPg3					

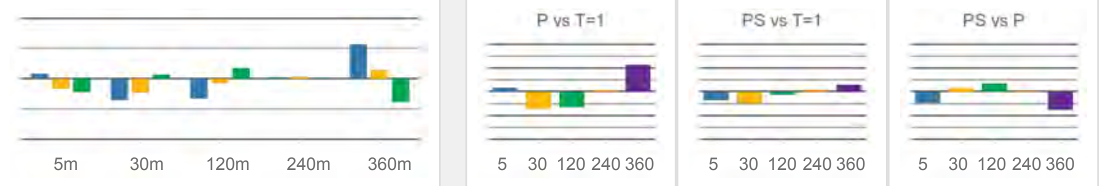

Locus		5m	30m	120m	240m	360m
**PGN_1923**	P vs T=1	0.545	-0.336	-0.421	-0.749	0.440
	PS vs T=1	-0.443	-0.579	-0.175	0.405	0.192
	PS vs P	-0.668	-0.344	0.023	0.499	0.004
	hypothetical protein					
	hypothetical proteins					

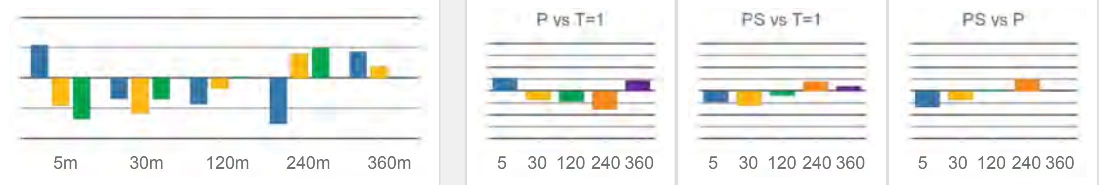

Locus		5m	30m	120m	240m	360m
**PGN_1924**	P vs T=1	0.735	0.440	0.683	0.265	1.109
	PS vs T=1	-0.016	-0.052	-0.102	-0.093	0.321
	PS vs P	-0.642	-0.460	-0.657	-0.453	-0.590
	conserved hypothetical protein					
	hypothetical proteins-Conserved					

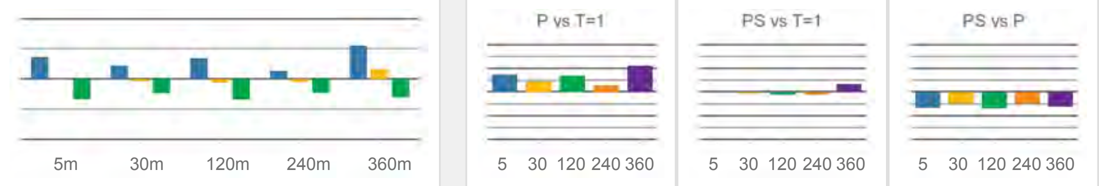

Locus		5m	30m	120m	240m	360m
**PGN_1925**	P vs T=1	1.937	1.929	1.796	1.714	1.802
	PS vs T=1	2.123	1.862	1.673	1.530	1.499
	PS vs P	0.193	-0.058	-0.124	-0.191	-0.292
	conserved hypothetical protein					
	hypothetical proteins-Conserved					

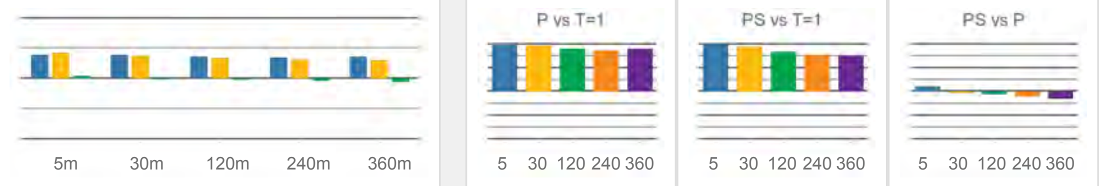

PgSg Time Course

log₂ (Fold Change)

Legend: P vs T=1 · PS vs T=1 · PS vs P

Locus		5m	30m	120m	240m	360m
**PGN_1926**	P vs T=1	0.400	0.367	0.444	0.430	0.082
	PS vs T=1	0.225	0.035	0.137	0.290	0.277
	PS vs P	-0.173	-0.333	-0.306	-0.141	0.171
	transposase in ISPg1					

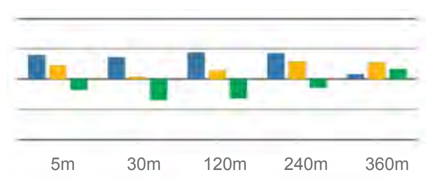

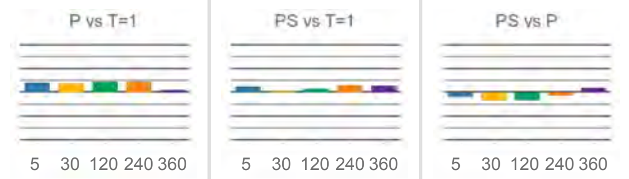

Locus		5m	30m	120m	240m	360m
**PGN_1927**	P vs T=1	-0.251	-0.162	-0.012	0.292	0.705
	PS vs T=1	0.826	0.935	0.088	-0.378	-0.056
	PS vs P	1.030	1.054	0.077	-0.653	-0.729
	conserved hypothetical protein					
	hypothetical proteins-Conserved					

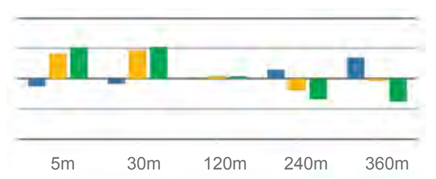

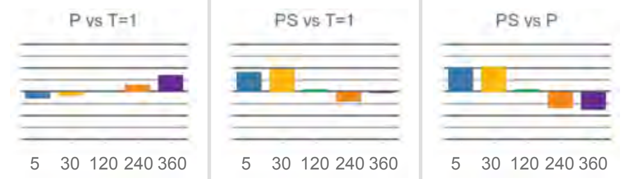

Locus		5m	30m	120m	240m	360m
**PGN_1928**	P vs T=1	0.734	0.866	0.568	0.164	0.087
	PS vs T=1	1.487	1.669	0.679	0.008	-0.184
	PS vs P	0.744	0.804	0.110	-0.200	-0.293
	conserved hypothetical protein					
	hypothetical proteins-Conserved					

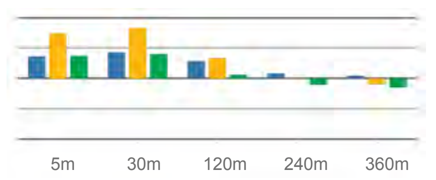

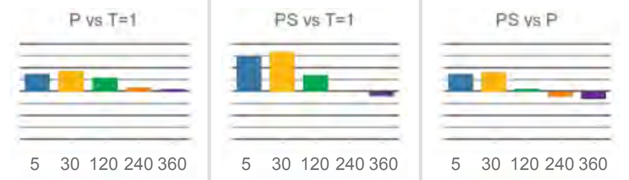

Locus		5m	30m	120m	240m	360m
**PGN_1929**	P vs T=1	1.090	1.251	1.142	0.611	0.139
	PS vs T=1	1.860	2.254	1.326	0.150	-0.029
	PS vs P	0.758	1.004	0.206	-0.483	-0.215
	conserved hypothetical protein					
	hypothetical proteins-Conserved					

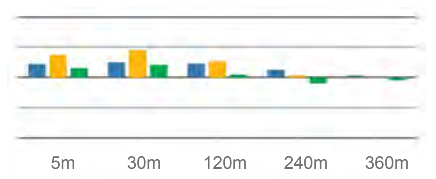

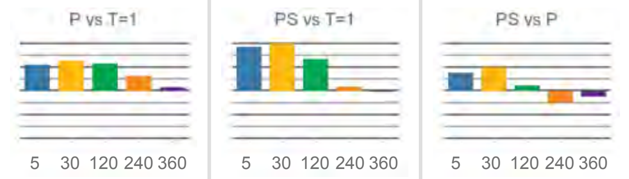

Locus		5m	30m	120m	240m	360m
**PGN_1930**	P vs T=1	0.652	0.827	0.308	-0.727	-1.249
	PS vs T=1	1.212	1.537	0.134	-1.224	-1.372
	PS vs P	0.579	0.757	-0.131	-0.589	-0.231
	conserved hypothetical protein					
	hypothetical proteins-Conserved					

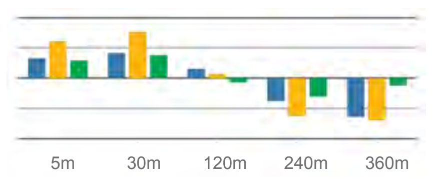

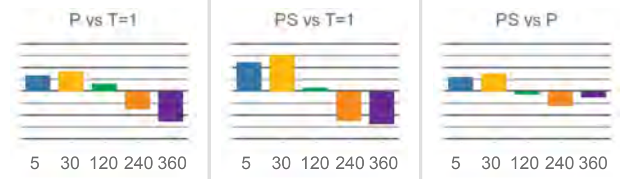

Locus		5m	30m	120m	240m	360m
**PGN_1931**	P vs T=1	0.324	0.278	-0.498	-1.595	-1.744
	PS vs T=1	0.848	1.048	-0.258	-1.587	-2.069
	PS vs P	0.561	0.811	0.253	-0.116	-0.395
	conserved hypothetical protein					
	hypothetical proteins-Conserved					

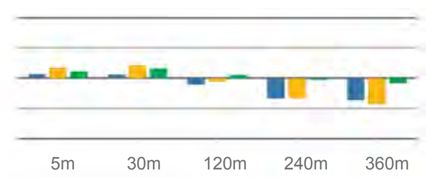

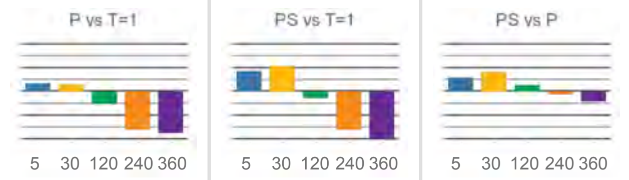

Locus		5m	30m	120m	240m	360m
**PGN_1932**	P vs T=1	-0.355	-0.887	-1.799	-2.341	-2.008
	PS vs T=1	0.235	0.241	-0.965	-1.895	-2.127
	PS vs P	0.659	1.108	0.738	0.272	-0.172
	conserved hypothetical protein					
	hypothetical proteins-Conserved					

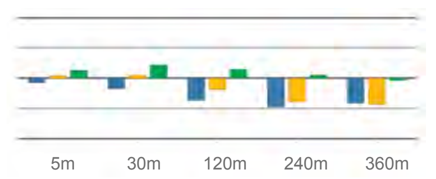

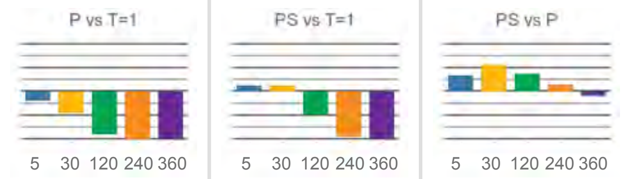

log2 (Fold Change)

Locus		5m	30m	120m	240m	360m
**PGN_1933**	P vs T=1	-0.324	-0.806	-1.663	-2.388	-2.732
	PS vs T=1	0.049	0.095	-0.685	-1.507	-1.962
	PS vs P	0.446	0.894	0.898	0.685	0.604

conserved hypothetical protein

hypothetical proteins-Conserved

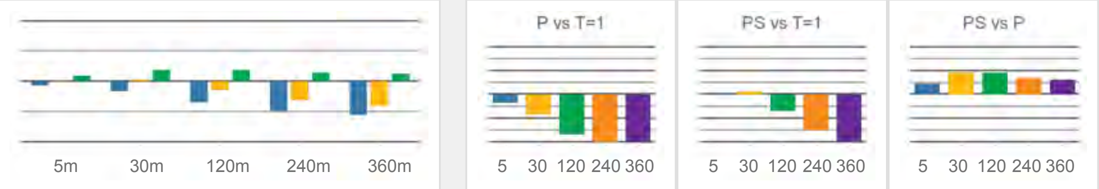

Locus		5m	30m	120m	240m	360m
**PGN_1934**	P vs T=1	-0.883	-1.005	-1.170	-1.667	-2.415
	PS vs T=1	-0.821	-0.668	-1.102	-1.271	-1.538
	PS vs P	0.079	0.331	0.078	0.296	0.659

conserved hypothetical protein

hypothetical proteins-Conserved

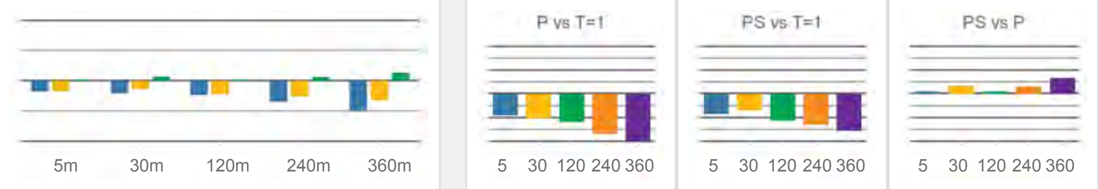

Locus		5m	30m	120m	240m	360m
**PGN_1935**	P vs T=1	-0.972	-1.564	-2.196	-2.574	-2.207
	PS vs T=1	-0.977	-1.205	-1.053	-1.451	-1.825
	PS vs P	0.057	0.328	1.041	0.948	0.325

hypothetical protein

hypothetical proteins

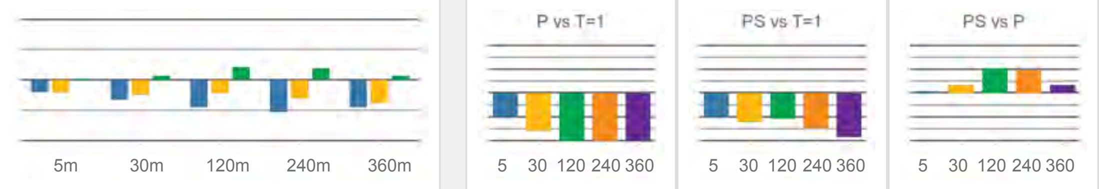

Locus		5m	30m	120m	240m	360m
**PGN_1936**	P vs T=1	1.085	1.682	1.975	2.777	2.839
	PS vs T=1	-0.605	0.013	0.022	0.445	1.030
	PS vs P	-1.424	-1.232	-1.506	-1.475	-1.234

conserved hypothetical protein

hypothetical proteins-Conserved

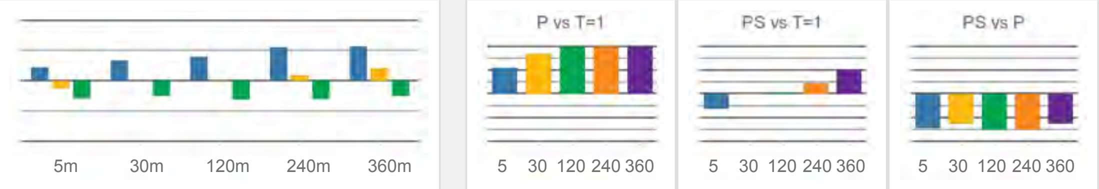

Locus		5m	30m	120m	240m	360m
**PGN_1937**	P vs T=1	-0.463	0.108	0.532	0.582	0.388
	PS vs T=1	-0.324	-0.295	-0.451	-0.207	-0.142
	PS vs P	0.088	-0.383	-0.928	-0.731	-0.509

glucose-inhibited division protein A

unknown function

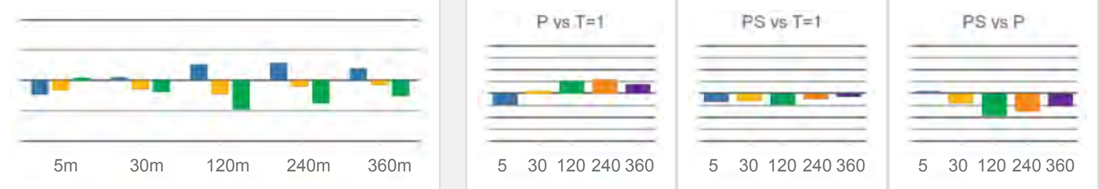

Locus		5m	30m	120m	240m	360m
**PGN_1938**	P vs T=1	0.217	0.331	0.050	0.205	0.572
uvrC	PS vs T=1	0.847	0.949	0.499	0.035	-0.242
	PS vs P	0.616	0.612	0.421	-0.175	-0.789

excinuclease ABC C subunit

DNA metabolism

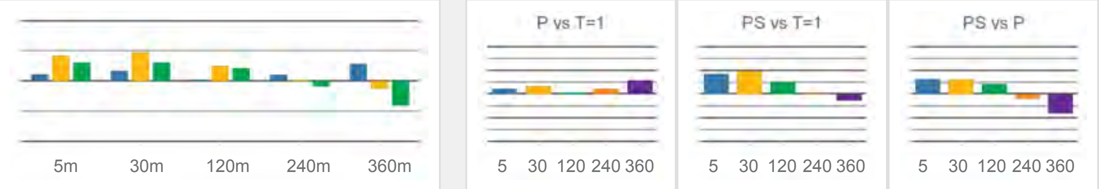

Locus		5m	30m	120m	240m	360m
**PGN_1939**	P vs T=1	0.191	0.539	0.341	-0.123	-0.486
	PS vs T=1	0.861	1.337	1.007	0.159	-0.312
	PS vs P	0.627	0.800	0.668	0.211	0.093

putative D-tyrosyl-tRNA deacylase

hypothetical proteins-Conserved

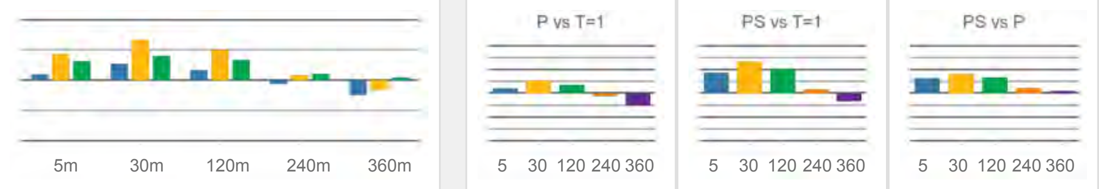

log2 (Fold Change)

P vs T=1 | PS vs T=1 | PS vs P

Locus		5m	30m	120m	240m	360m
**PGN_1940**	P vs T=1	0.158	0.513	0.240	-0.132	-0.286
	PS vs T=1	0.962	1.662	1.348	0.389	-0.160
	PS vs P	0.745	1.134	1.074	0.405	0.047

conserved hypothetical protein

hypothetical proteins-Conserved

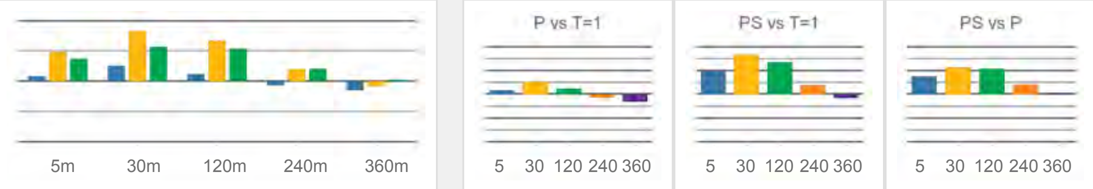

Locus		5m	30m	120m	240m	360m
**PGN_1941**	P vs T=1	1.379	1.559	1.491	1.048	0.719
	PS vs T=1	2.147	2.692	2.501	1.843	1.360
	PS vs P	0.744	1.120	1.007	0.753	0.601

putative deoxyribose-phosphate aldolase

purines, pyrimidines, nucleosides and nucleotides

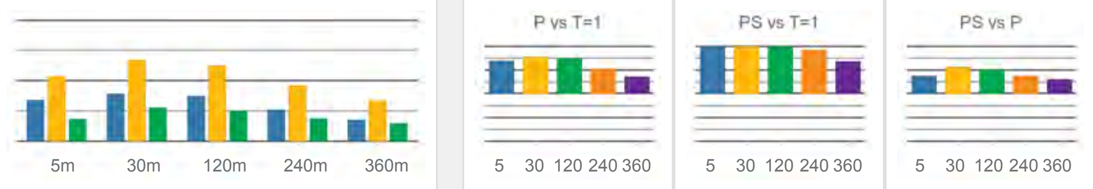

Locus		5m	30m	120m	240m	360m
**PGN_1942**	P vs T=1	1.837	2.028	2.404	2.369	1.935
	PS vs T=1	2.399	2.956	2.980	2.736	2.487
	PS vs P	0.533	0.903	0.591	0.380	0.531

hypothetical protein

hypothetical proteins

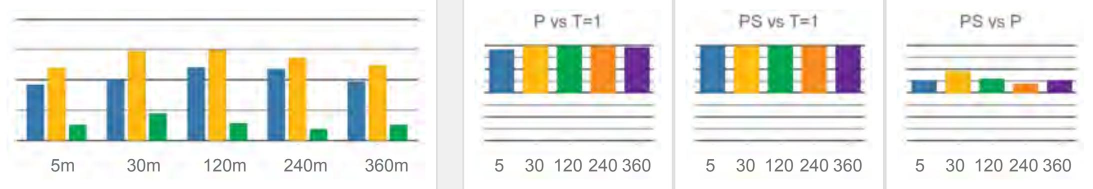

Locus		5m	30m	120m	240m	360m
**PGN_1943**	P vs T=1	-0.092	-0.720	-1.314	-1.341	-1.300
	PS vs T=1	-0.759	-1.145	-1.037	-0.909	-0.999
	PS vs P	-0.615	-0.412	0.248	0.402	0.286

putative polyprenyl synthetase

central intermediary metabolism

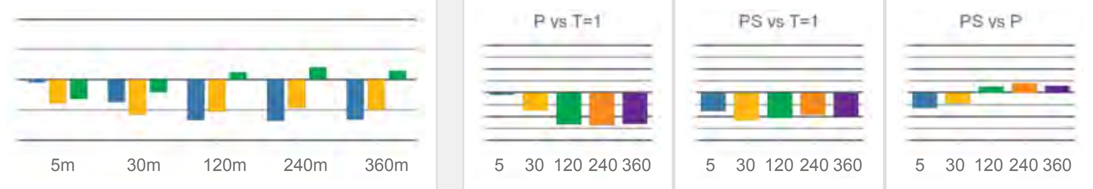

Locus		5m	30m	120m	240m	360m
**PGN_1944**	P vs T=1	-1.164	-1.825	-1.609	-1.011	-0.167
	PS vs T=1	-1.342	-1.718	-2.082	-1.864	-1.342
	PS vs P	-0.146	0.023	-0.524	-0.723	-1.021

conserved hypothetical protein

hypothetical proteins-Conserved

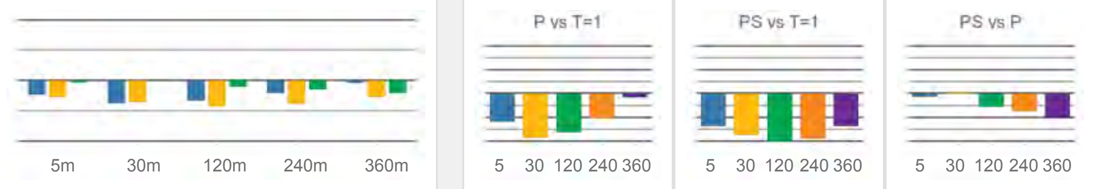

Locus		5m	30m	120m	240m	360m
**PGN_1945**	P vs T=1	-1.003	-1.324	-1.402	-1.083	-0.540
	PS vs T=1	-1.045	-1.584	-1.973	-1.353	-1.481
	PS vs P	-0.025	-0.281	-0.621	-0.235	-0.841

probable signal peptidase-related protein

protein fate

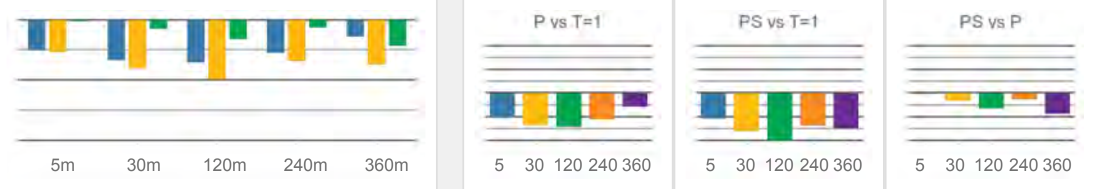

Locus		5m	30m	120m	240m	360m
**PGN_1946**	P vs T=1	-0.396	-0.310	-0.076	0.025	0.136
	PS vs T=1	-0.074	-0.188	-0.550	-0.445	-0.420
	PS vs P	0.304	0.110	-0.463	-0.448	-0.537

signal peptidase I

protein fate

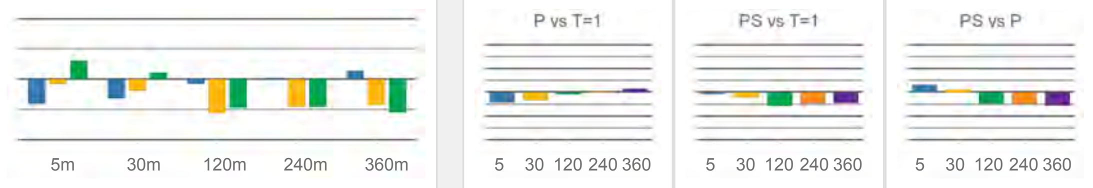

log$_2$ (Fold Change)

Legend: P vs T=1 | PS vs T=1 | PS vs P

Locus		5m	30m	120m	240m	360m
**PGN_1947** *dapB*	P vs T=1	-0.712	-0.503	-0.529	-0.811	-0.677
	PS vs T=1	-0.628	-0.660	-1.221	-1.228	-1.095
	PS vs P	0.077	-0.144	-0.666	-0.430	-0.411

putative dihydrodipicolinate reductase

amino acid biosynthesis

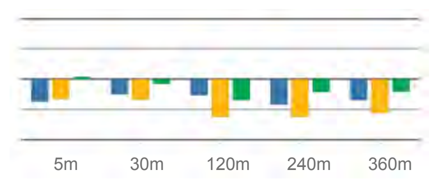

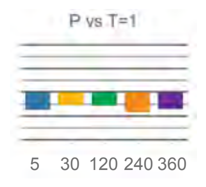

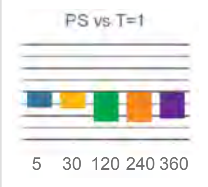

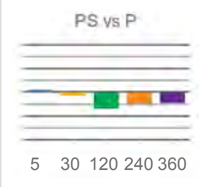

Locus		5m	30m	120m	240m	360m
**PGN_1948**	P vs T=1	-0.003	-0.033	0.229	0.118	0.217
	PS vs T=1	0.422	0.138	0.011	0.187	0.143
	PS vs P	0.405	0.147	-0.214	0.053	-0.076

deoxyguanosinetriphosphate triphosphohydrolase

purines, pyrimidines, nucleosides and nucleotides

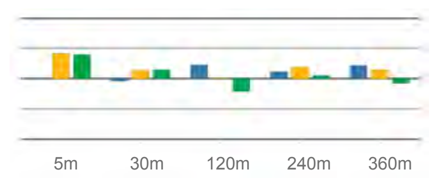

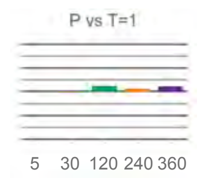

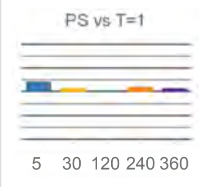

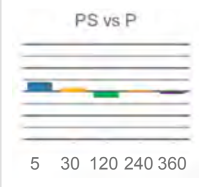

Locus		5m	30m	120m	240m	360m
**PGN_1949**	P vs T=1	0.032	-0.382	-0.077	0.512	1.321
	PS vs T=1	-0.510	0.346	1.097	1.756	1.654
	PS vs P	-0.553	0.544	1.002	1.205	0.432

conserved hypothetical protein

hypothetical proteins-Conserved

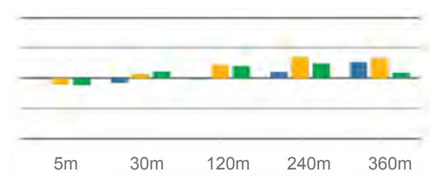

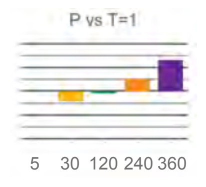

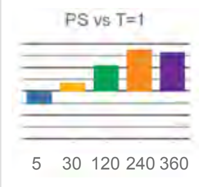

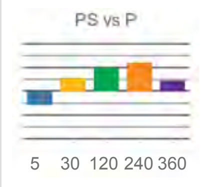

Locus		5m	30m	120m	240m	360m
**PGN_1950**	P vs T=1	-0.103	-1.054	-0.925	0.315	2.302
	PS vs T=1	-0.303	-0.495	0.864	1.431	0.984
	PS vs P	-0.201	-0.174	0.792	0.912	-0.406

hypothetical protein

hypothetical proteins

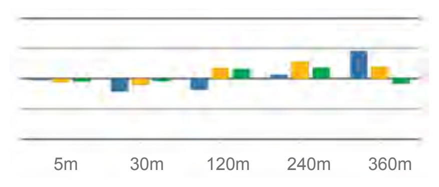

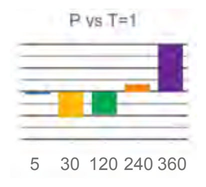

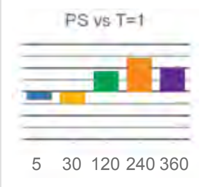

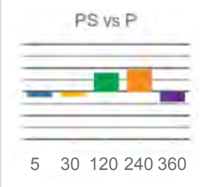

Locus		5m	30m	120m	240m	360m
**PGN_1951**	P vs T=1	0.504	-0.358	-0.814	-0.052	-0.388
	PS vs T=1	-0.682	0.031	0.717	0.969	0.708
	PS vs P	-1.064	0.316	1.288	1.015	1.008

conserved hypothetical protein

hypothetical proteins-Conserved

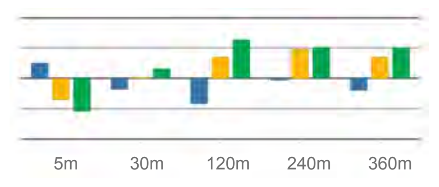

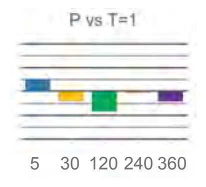

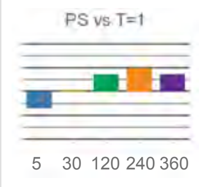

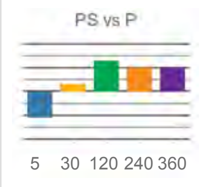

Locus		5m	30m	120m	240m	360m
**PGN_1952**	P vs T=1	0.536	0.595	0.768	1.332	1.717
	PS vs T=1	0.243	0.395	1.614	0.222	1.476
	PS vs P	-0.268	-0.171	0.793	-0.577	0.182

hypothetical protein

hypothetical proteins

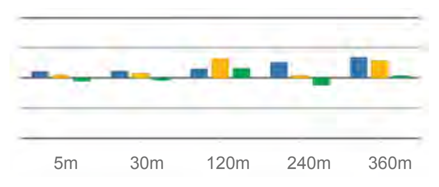

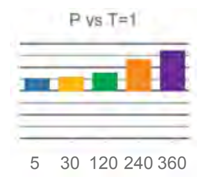

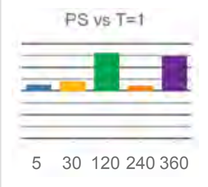

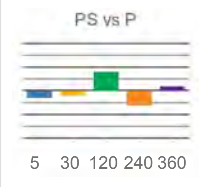

Locus		5m	30m	120m	240m	360m
**PGN_1953**	P vs T=1	0.577	0.817	0.871	1.412	2.112
	PS vs T=1	0.396	0.673	1.219	1.126	1.228
	PS vs P	-0.193	-0.138	0.332	-0.234	-0.815

TonB-dependent outer membrane receptor

transport and binding proteins

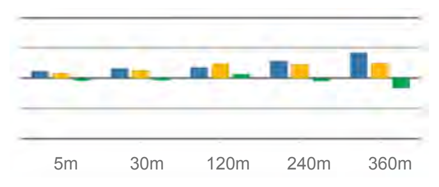

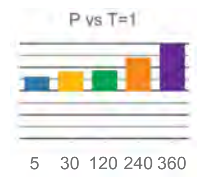

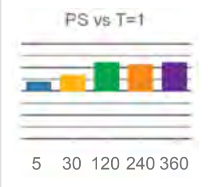

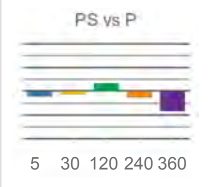

PgSg Time Course

log$_2$ (Fold Change)

Legend: P vs T=1, PS vs T=1, PS vs P

Locus		5m	30m	120m	240m	360m
**PGN_1954**	P vs T=1	-0.225	-0.686	-1.258	-1.515	-0.790
	PS vs T=1	-0.434	-0.469	-0.003	-0.004	-0.273
	PS vs P	-0.179	0.196	1.143	1.290	0.497
	probable DNA repair protein RecO					
	DNA metabolism					
**PGN_1955**	P vs T=1	0.025	-0.026	-0.394	-0.835	-1.427
	PS vs T=1	-0.026	0.019	-0.041	-0.337	-0.564
	PS vs P	-0.043	0.053	0.350	0.479	0.835
	phosphomannomutase					
	energy metabolism					
**PGN_1956**	P vs T=1	0.617	1.218	1.639	1.183	0.673
	PS vs T=1	1.073	1.245	1.915	1.616	1.526
	PS vs P	0.358	0.041	0.351	0.403	0.761
	hypothetical protein					
	hypothetical proteins					
**PGN_1957**	P vs T=1	0.322	0.228	0.235	0.395	0.023
	PS vs T=1	0.159	-0.014	0.149	0.240	0.134
	PS vs P	-0.162	-0.248	-0.097	-0.152	0.092
	transposase in ISPg1					
**PGN_1959**	P vs T=1	-0.308	-0.648	-1.376	-2.139	-1.628
	PS vs T=1	-0.054	0.193	-0.798	-1.873	-2.122
	PS vs P	0.322	0.841	0.504	-0.060	-0.544
	CRISPR-associated protein Cas2					
	hypothetical proteins-Conserved					
**PGN_1960**	P vs T=1	0.153	-0.229	-1.305	-1.998	-1.437
	PS vs T=1	0.543	0.751	-0.779	-2.316	-2.455
	PS vs P	0.451	0.999	0.470	-0.479	-1.024
	CRISPR-associated protein Cas1					
	hypothetical proteins-Conserved					
**PGN_1961**	P vs T=1	0.804	0.581	-0.115	-0.815	-1.018
	PS vs T=1	1.510	1.554	-0.068	-1.451	-1.260
	PS vs P	0.722	0.982	0.046	-0.708	-0.291
	CRISPR-associated protein Cas4					
	hypothetical proteins-Conserved					

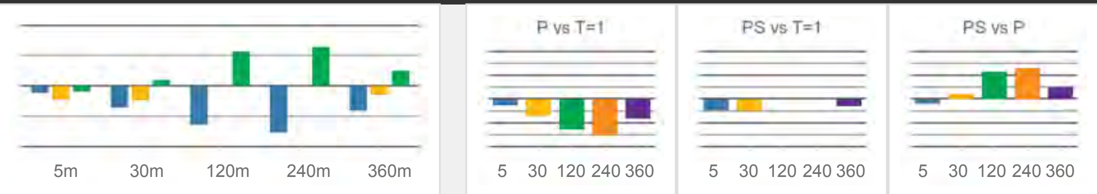

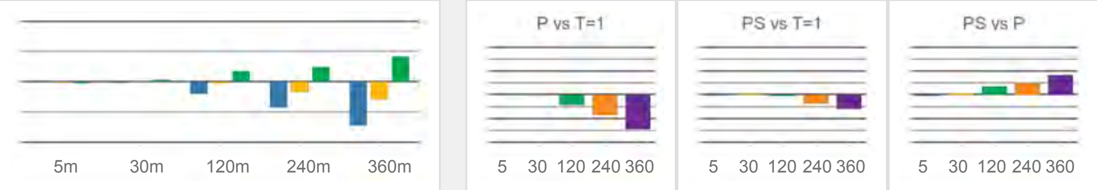

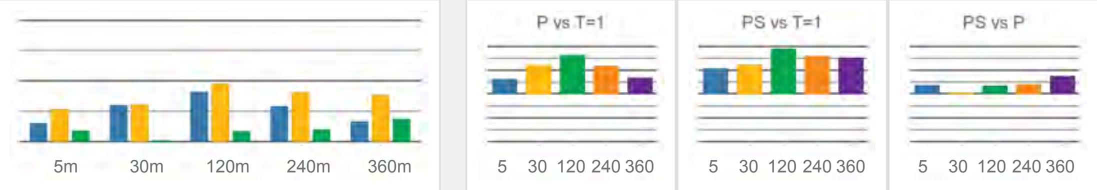

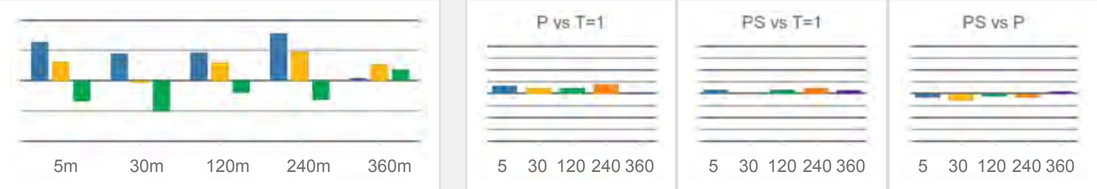

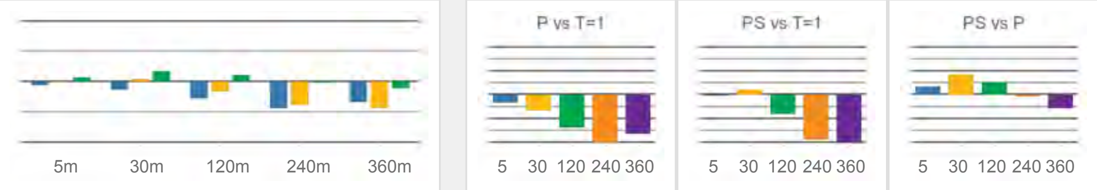

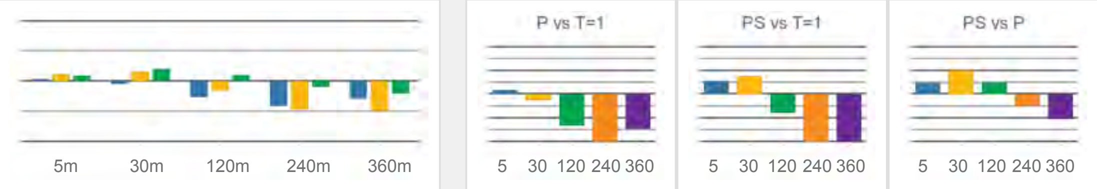

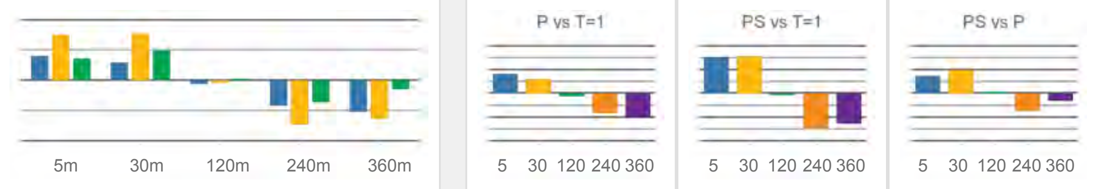

PgSg Time Course

log$_2$ (Fold Change)

P vs T=1 | PS vs T=1 | PS vs P

Locus		5m	30m	120m	240m	360m
**PGN_1962**	P vs T=1	0.512	0.837	0.673	-0.151	-1.081
	PS vs T=1	0.714	1.322	0.873	-0.322	-0.840
	PS vs P	0.201	0.502	0.222	-0.189	0.195
	conserved hypothetical protein					
	hypothetical proteins-Conserved					
**PGN_1963**	P vs T=1	0.718	0.576	-0.187	-1.217	-1.723
	PS vs T=1	1.018	1.231	0.310	-0.934	-1.567
	PS vs P	0.353	0.696	0.500	0.177	0.058
	conserved hypothetical protein					
	hypothetical proteins-Conserved					
**PGN_1964**	P vs T=1	0.630	0.191	-1.362	-2.243	-2.430
	PS vs T=1	0.572	-0.089	-1.486	-2.006	-2.268
	PS vs P	0.043	-0.202	-0.159	0.123	0.085
	probable CRISPR-associated helicase Cas3 core					
	unknown function					
**PGN_1965**	P vs T=1	-0.032	-0.292	-1.296	-2.161	-2.847
	PS vs T=1	-0.037	-0.471	-1.097	-1.448	-1.730
	PS vs P	0.067	-0.121	0.168	0.569	0.949
	hypothetical protein					
	hypothetical proteins					
**PGN_1966**	P vs T=1	-0.407	-0.947	-2.071	-2.823	-3.340
	PS vs T=1	-0.418	-0.776	-1.224	-1.352	-1.684
	PS vs P	0.057	0.193	0.780	1.314	1.510
	conserved hypothetical protein					
	hypothetical proteins-Conserved					
**PGN_1967**	P vs T=1	-0.357	-0.582	-0.907	-0.954	-0.608
	PS vs T=1	-0.173	-0.908	-1.794	-1.692	-1.608
	PS vs P	0.201	-0.313	-0.883	-0.731	-0.973
	putative sulfatase					
	hypothetical proteins-Conserved					
**PGN_1968**	P vs T=1	0.507	0.324	-0.351	-0.526	-0.352
	PS vs T=1	0.591	-0.031	-0.711	-0.603	-0.729
	PS vs P	0.095	-0.344	-0.367	-0.094	-0.378
	conserved hypothetical protein					
	hypothetical proteins-Conserved					

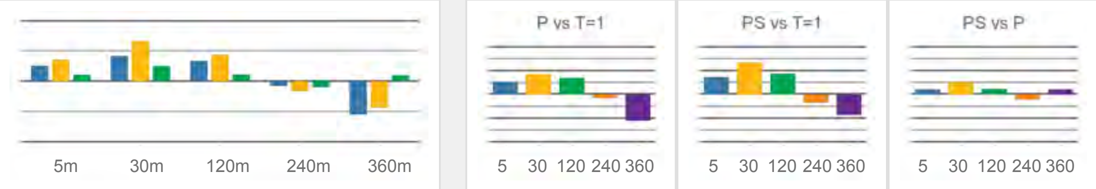

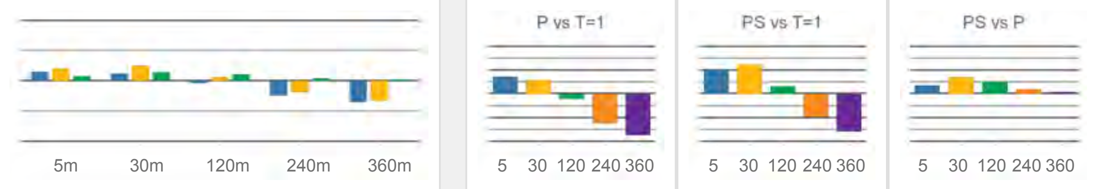

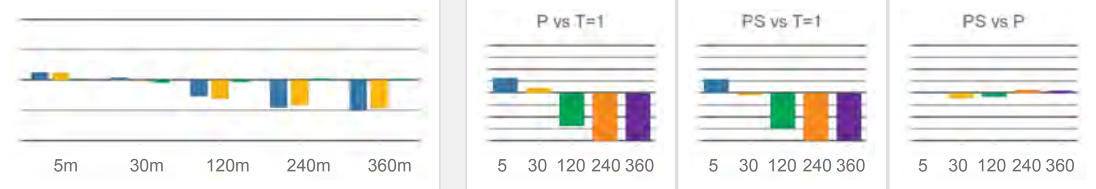

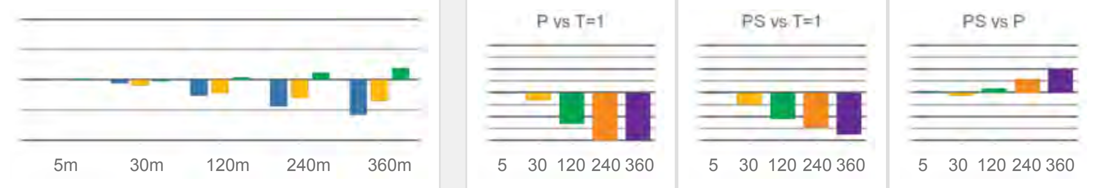

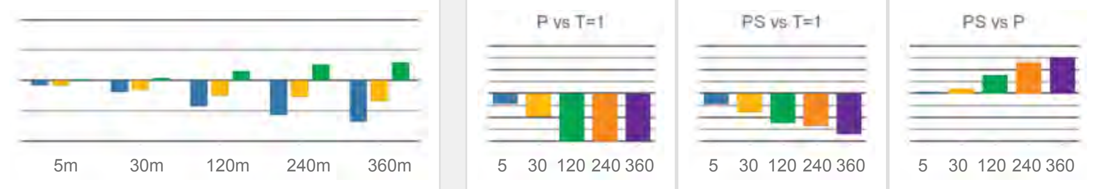

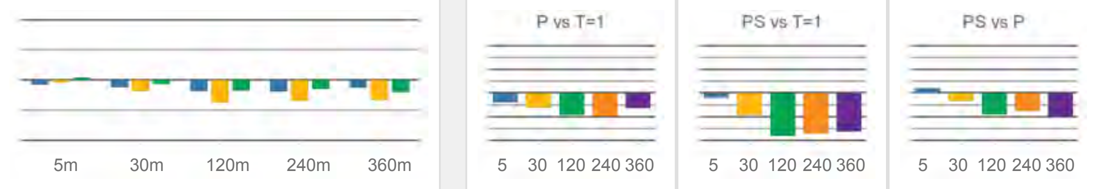

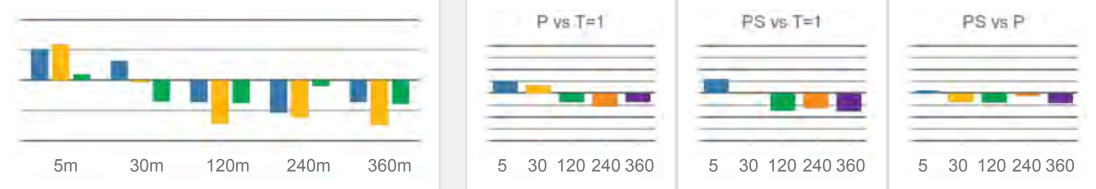

PgSg Time Course

Hackett Laboratory UW

log$_2$ (Fold Change)

Legend: P vs T=1 | PS vs T=1 | PS vs P

Locus		5m	30m	120m	240m	360m
**PGN_1969**	P vs T=1	-0.100	-0.407	-0.588	0.031	0.095
	PS vs T=1	0.068	-0.362	-0.360	-0.021	0.090
	PS vs P	0.163	0.013	0.158	-0.045	-0.006

putative methionyl-tRNA formyltransferase

protein synthesis

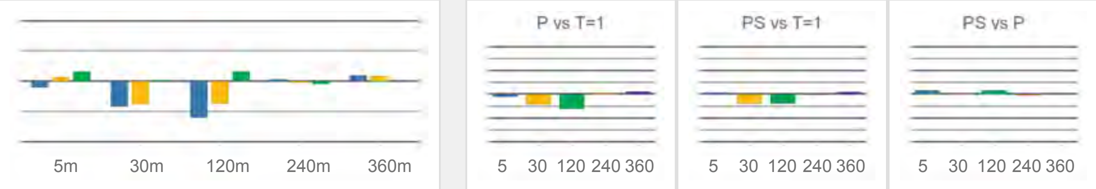

Locus		5m	30m	120m	240m	360m
**PGN_1970** *rgpA*	P vs T=1	0.557	0.905	0.942	0.429	-0.347
	PS vs T=1	1.035	1.101	0.501	-0.041	-0.287
	PS vs P	0.469	0.216	-0.410	-0.466	0.034

arginine-specific cysteine proteinase RgpA

protein fate

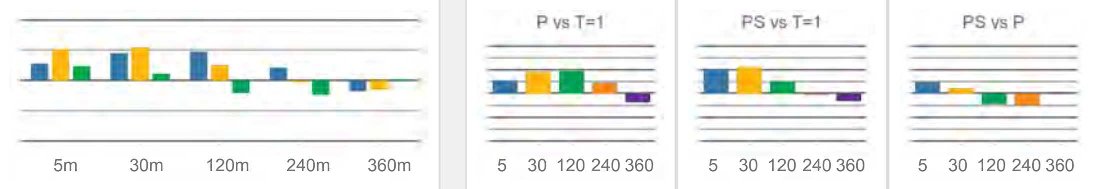

Locus		5m	30m	120m	240m	360m
**PGN_1971**	P vs T=1	-0.369	-0.294	0.519	0.886	3.229
	PS vs T=1	-0.690	-0.545	0.451	0.271	1.191
	PS vs P	-0.477	-0.380	0.082	-0.254	-0.573

hypothetical protein

hypothetical proteins

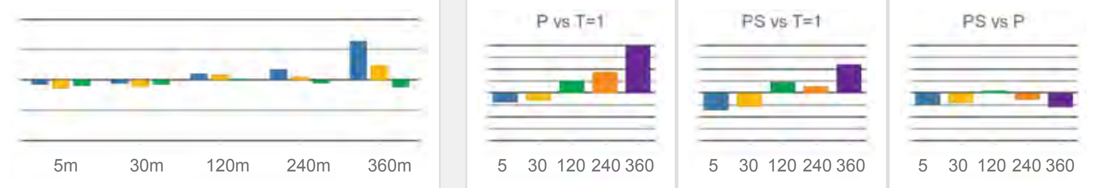

Locus		5m	30m	120m	240m	360m
**PGN_1972**	P vs T=1	0.047	-0.105	0.539	0.914	3.035
	PS vs T=1	-0.371	0.366	-0.036	0.512	0.581
	PS vs P	-0.389	0.181	-0.326	-0.071	-0.853

conserved hypothetical protein

hypothetical proteins-Conserved

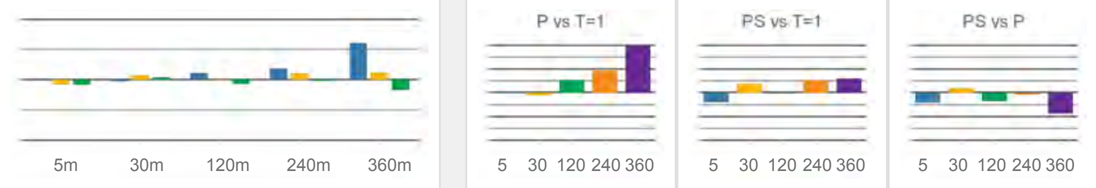

Locus		5m	30m	120m	240m	360m
**PGN_1973**	P vs T=1	-0.200	-0.523	-0.725	-0.780	-0.523
	PS vs T=1	-0.032	-0.460	-0.705	-0.514	-0.511
	PS vs P	0.205	0.043	-0.033	0.182	0.014

probable phosphoglycerate mutase

unknown function

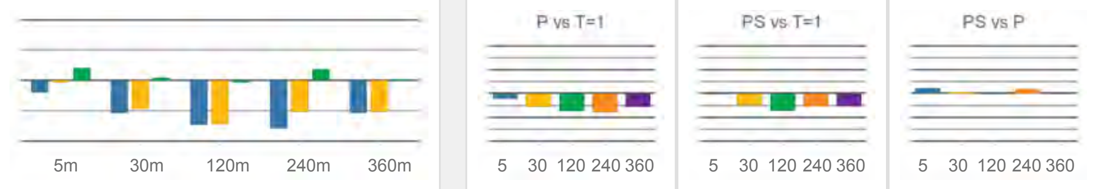

Locus		5m	30m	120m	240m	360m
**PGN_1974**	P vs T=1	-0.033	0.211	0.447	0.484	0.514
	PS vs T=1	-0.440	-0.326	-0.292	0.286	0.707
	PS vs P	-0.413	-0.506	-0.692	-0.160	0.214

conserved hypothetical protein

hypothetical proteins-Conserved

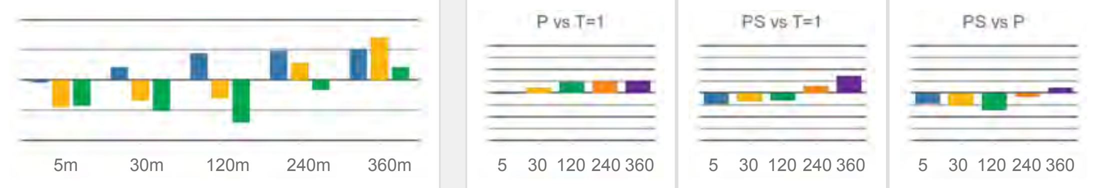

Locus		5m	30m	120m	240m	360m
**PGN_1975**	P vs T=1	-0.450	-0.339	-0.306	0.255	0.181
	PS vs T=1	-0.639	-0.644	-0.500	-0.225	0.168
	PS vs P	-0.203	-0.304	-0.219	-0.397	0.013

putative regulatory protein

DNA metabolism

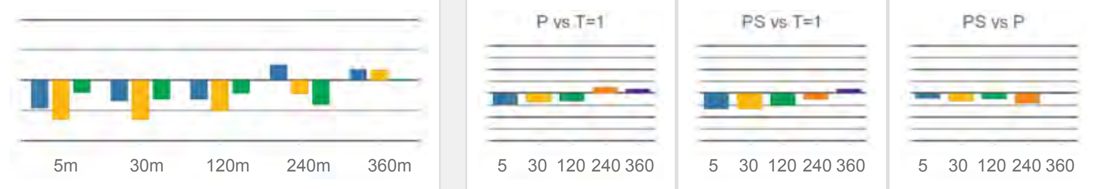

PgSg Time Course

log$_2$ (Fold Change)

Legend: P vs T=1 | PS vs T=1 | PS vs P

Locus		5m	30m	120m	240m	360m
**PGN_1976**	P vs T=1	0.255	0.599	1.175	1.527	1.342
	PS vs T=1	-0.004	-0.002	0.121	0.479	0.875
	PS vs P	-0.277	-0.571	-0.978	-0.937	-0.415

conserved hypothetical protein

hypothetical proteins-Conserved

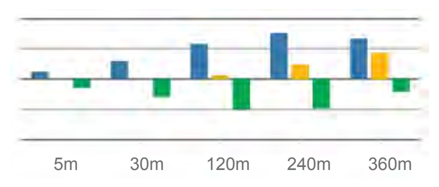

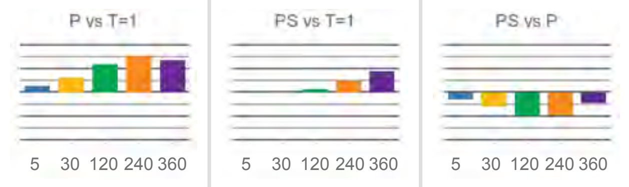

Locus		5m	30m	120m	240m	360m
**PGN_1977**	P vs T=1	0.434	0.010	-0.806	-1.081	-1.287
	PS vs T=1	-0.227	-0.731	-1.022	-0.773	-1.035
	PS vs P	-0.593	-0.697	-0.241	0.247	0.197

conserved hypothetical protein

hypothetical proteins-Conserved

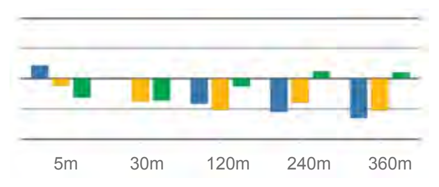

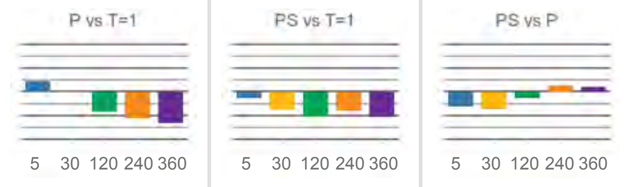

Locus		5m	30m	120m	240m	360m
**PGN_1978**	P vs T=1	-0.375	-0.857	-1.161	-1.590	-2.289
	PS vs T=1	-1.170	-1.929	-2.273	-2.307	-2.324
	PS vs P	-0.734	-1.041	-1.094	-0.726	-0.084

conserved hypothetical protein

hypothetical proteins-Conserved

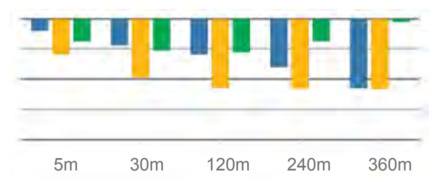

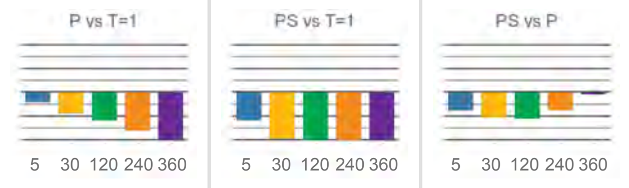

Locus		5m	30m	120m	240m	360m
**PGN_1979**	P vs T=1	-0.039	-0.207	0.148	0.481	0.548
	PS vs T=1	-0.329	-0.443	-0.292	-0.037	0.172
	PS vs P	-0.290	-0.252	-0.438	-0.492	-0.361

primosomal protein N

DNA metabolism

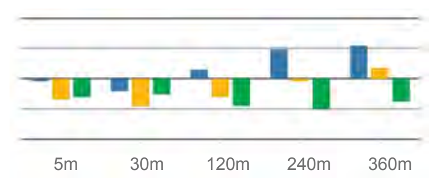

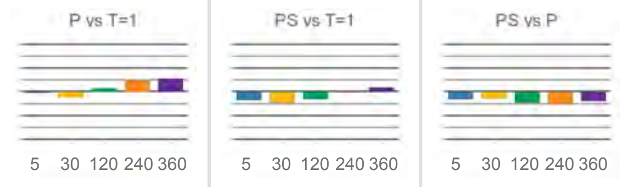

Locus		5m	30m	120m	240m	360m
**PGN_1980**	P vs T=1	1.120	1.202	1.434	1.619	1.318
	PS vs T=1	1.009	0.623	0.091	0.324	0.616
	PS vs P	-0.077	-0.529	-1.269	-1.193	-0.660

putative NADPH-dependent glutamate synthase

central intermediary metabolism

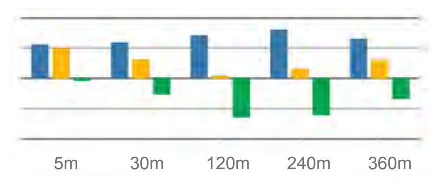

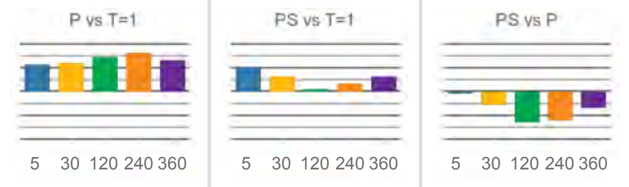

Locus		5m	30m	120m	240m	360m
**PGN_1981**	P vs T=1	0.309	-0.099	-0.053	0.030	0.082
	PS vs T=1	-0.048	-0.427	-0.862	-0.675	-0.773
	PS vs P	-0.283	-0.312	-0.769	-0.639	-0.802

conserved hypothetical protein

unknown function

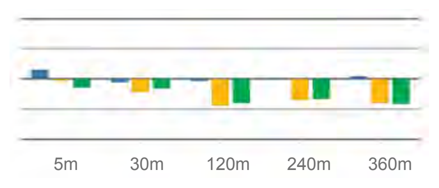

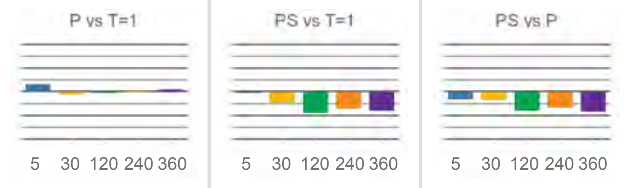

Locus		5m	30m	120m	240m	360m
**PGN_1982**	P vs T=1	-0.319	-0.707	-0.986	-0.575	0.222
	PS vs T=1	-0.109	-0.413	0.245	0.646	0.179
	PS vs P	0.198	0.205	1.011	1.095	0.007

putative tRNA guanine 1-methyltransferase

protein synthesis

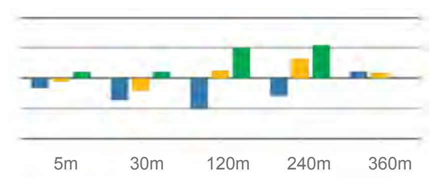

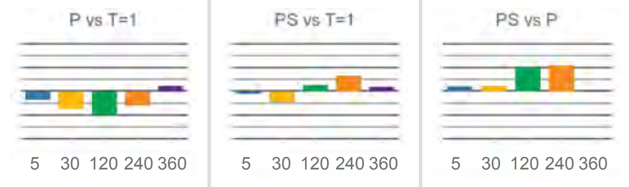

log$_2$ (Fold Change)

Legend: P vs T=1 | PS vs T=1 | PS vs P

Locus		5m	30m	120m	240m	360m
**PGN_1983**	P vs T=1	-0.957	-0.901	-0.658	0.184	0.901
	PS vs T=1	-0.758	-1.022	-0.950	-0.736	-0.239
	PS vs P	0.138	-0.174	-0.347	-0.732	-0.962

putative ion transporter

transport and binding proteins

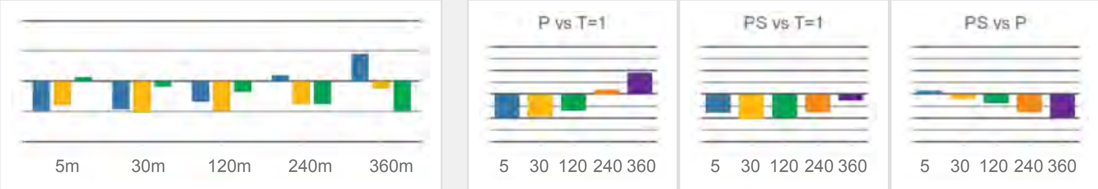

Locus		5m	30m	120m	240m	360m
**PGN_1984**	P vs T=1	0.089	0.775	2.046	2.587	2.611
	PS vs T=1	0.318	0.558	1.091	1.288	1.491
	PS vs P	0.084	-0.248	-0.816	-1.071	-0.977

hypothetical protein

hypothetical proteins

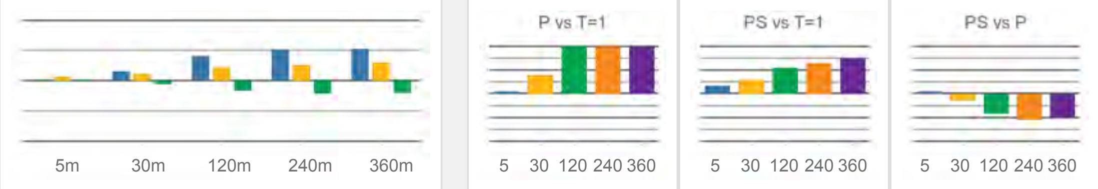

Locus		5m	30m	120m	240m	360m
**PGN_1985**	P vs T=1	0.276	0.880	2.072	2.798	2.909
	PS vs T=1	0.150	0.342	0.874	1.264	1.674
	PS vs P	-0.211	-0.540	-1.075	-1.312	-1.099

probable N-acetylmuramoyl-L-alanine amidase

cell envelope

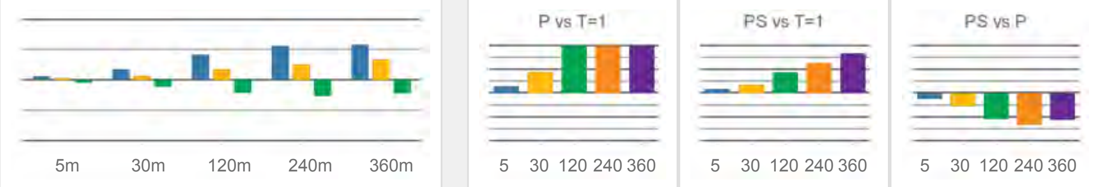

Locus		5m	30m	120m	240m	360m
**PGN_1986**	P vs T=1	0.498	1.103	2.210	2.984	3.082
	PS vs T=1	0.229	0.645	0.866	1.121	1.553
	PS vs P	-0.313	-0.455	-1.273	-1.736	-1.446

DNA-binding protein, histone-like family

DNA metabolism

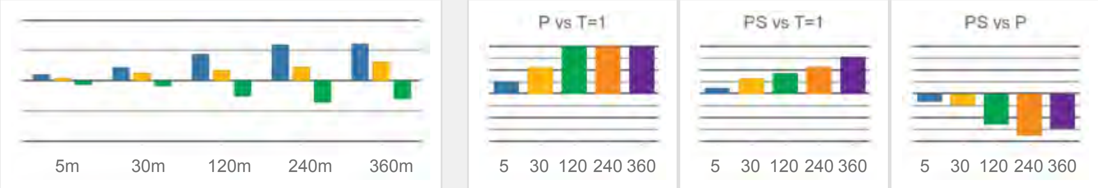

Locus		5m	30m	120m	240m	360m
**PGN_1987**	P vs T=1	0.654	0.571	0.326	0.085	-0.008
	PS vs T=1	1.197	1.371	1.631	1.377	1.060
	PS vs P	0.528	0.773	1.253	1.189	1.001

conserved hypothetical protein

hypothetical proteins-Conserved

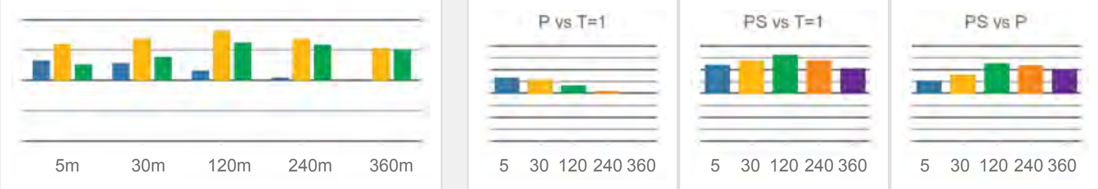

Locus		5m	30m	120m	240m	360m
**PGN_1988**	P vs T=1	-0.188	-0.807	-1.254	-1.068	-0.754
	PS vs T=1	-0.068	0.071	0.630	0.871	0.624
	PS vs P	0.126	0.787	1.727	1.804	1.321

conserved hypothetical protein

energy metabolism

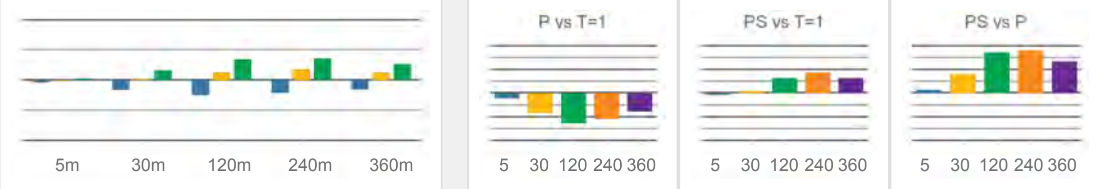

Locus		5m	30m	120m	240m	360m
**PGN_1989**	P vs T=1	-0.331	-0.386	0.000	0.255	0.502
	PS vs T=1	-0.009	0.141	0.117	0.549	0.356
	PS vs P	0.263	0.442	0.091	0.301	-0.116

conserved hypothetical protein with NIF3 domain

hypothetical proteins-Conserved

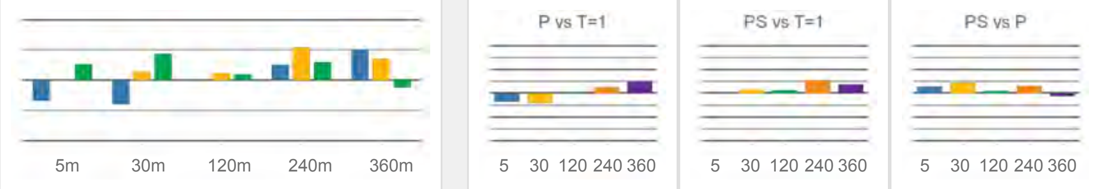

PgSg Time Course

log$_2$ (Fold Change)

Legend: P vs T=1 | PS vs T=1 | PS vs P

Locus		5m	30m	120m	240m	360m
**PGN_1990**	P vs T=1	-0.544	-0.825	-1.259	-1.502	-1.865
	PS vs T=1	-1.827	-1.843	-2.103	-1.841	-1.893
	PS vs P	-1.216	-0.970	-0.830	-0.346	-0.058
	conserved hypothetical protein					
	hypothetical proteins-Conserved					
**PGN_1991**	P vs T=1	-0.136	0.575	1.125	1.141	0.763
	PS vs T=1	-0.327	-0.042	-0.097	-0.018	-0.008
	PS vs P	-0.292	-0.520	-1.036	-0.978	-0.707
	putative cell-cycle protein					
	hypothetical proteins-Conserved					
**PGN_1992**	P vs T=1	1.119	1.147	1.202	1.217	1.002
	PS vs T=1	1.112	0.703	0.205	0.193	0.073
	PS vs P	0.003	-0.430	-0.977	-0.997	-0.917
	putative helicase					
	DNA metabolism					
**PGN_1993**	P vs T=1	2.350	2.283	1.673	0.901	0.036
	PS vs T=1	2.004	2.121	1.563	0.715	0.046
	PS vs P	-0.279	-0.100	-0.082	-0.216	-0.065
	conserved hypothetical protein					
	hypothetical proteins-Conserved					
**PGN_1994**	P vs T=1	1.210	1.249	0.716	0.205	-0.670
	PS vs T=1	0.819	0.833	0.158	-0.514	-0.914
	PS vs P	-0.337	-0.353	-0.529	-0.718	-0.297
	conserved hypothetical protein					
	hypothetical proteins-Conserved					
**PGN_1995**	P vs T=1	0.048	0.155	0.577	0.594	-0.142
	PS vs T=1	-0.380	-0.165	-0.257	-0.650	-0.669
	PS vs P	-0.414	-0.300	-0.767	-1.169	-0.534
	conserved hypothetical protein					
	hypothetical proteins-Conserved					
**PGN_1996** *dapA*	P vs T=1	-0.558	-1.022	-1.739	-1.771	-1.676
	PS vs T=1	-1.257	-1.123	-0.767	-0.604	-0.881
	PS vs P	-0.644	-0.101	0.872	1.056	0.736
	putative dihydrodipicolinate synthase					
	amino acid biosynthesis					

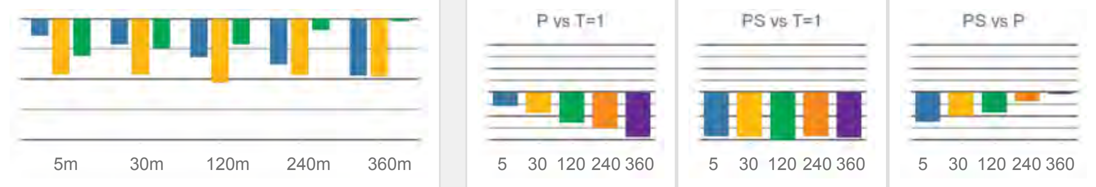

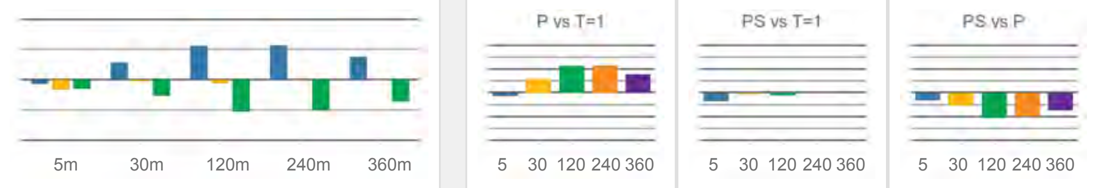

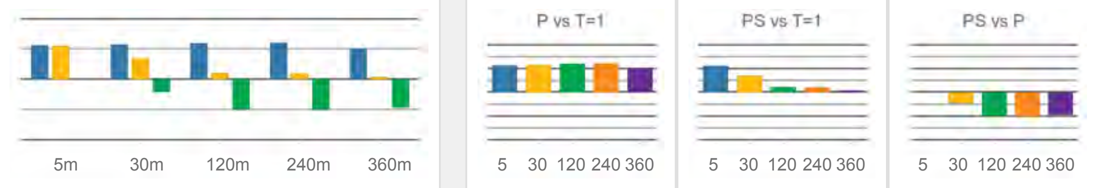

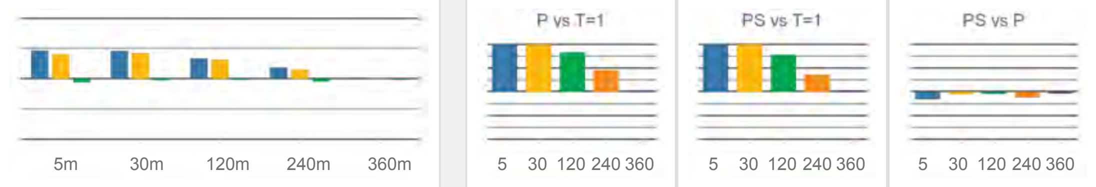

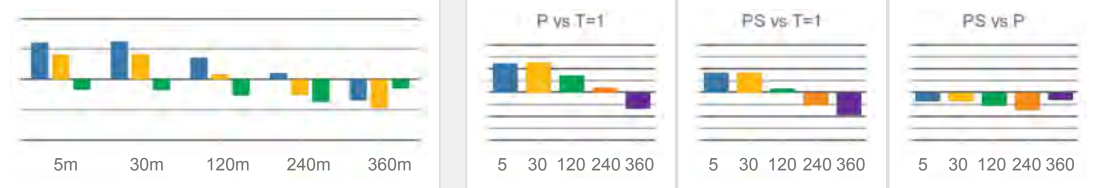

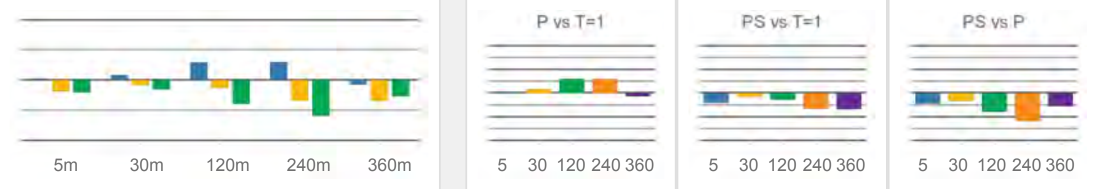

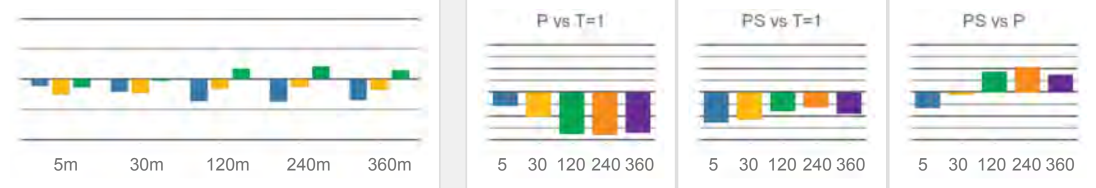

PgSg Time Course

log₂ (Fold Change)

Legend: P vs T=1 | PS vs T=1 | PS vs P

Locus		5m	30m	120m	240m	360m
**PGN_1997**	P vs T=1	-0.405	-0.834	-0.811	-0.679	-0.948
BioD	PS vs T=1	-0.897	-0.982	-0.396	-0.144	-0.480
	PS vs P	-0.458	-0.177	0.379	0.518	0.419

putative dethiobiotin synthase

biosynthesis of cofactors, prosthetic groups, and carriers

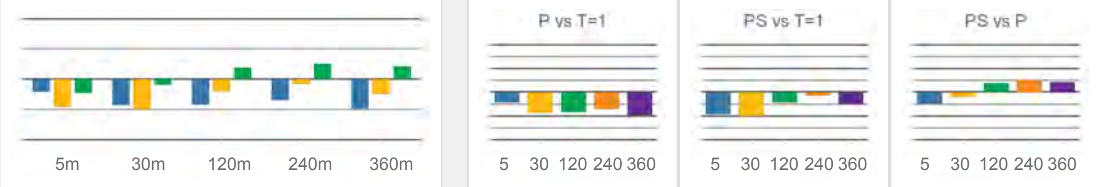

Locus		5m	30m	120m	240m	360m
**PGN_1998**	P vs T=1	0.645	1.022	1.444	1.374	0.471
	PS vs T=1	0.582	1.003	0.994	0.589	0.429
	PS vs P	-0.077	0.000	-0.396	-0.734	-0.060

immunoreactive 23 kDa antigen

cell envelope

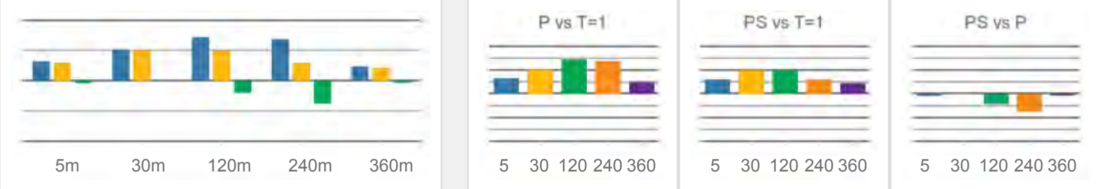

Locus		5m	30m	120m	240m	360m
**PGN_1999**	P vs T=1	0.295	-0.080	-0.699	-1.246	-1.844
	PS vs T=1	0.267	0.054	-0.535	-0.772	-0.882
	PS vs P	0.005	0.145	0.150	0.423	0.898

putative dihydroorotate dehydrogenase

purines, pyrimidines, nucleosides and nucleotides

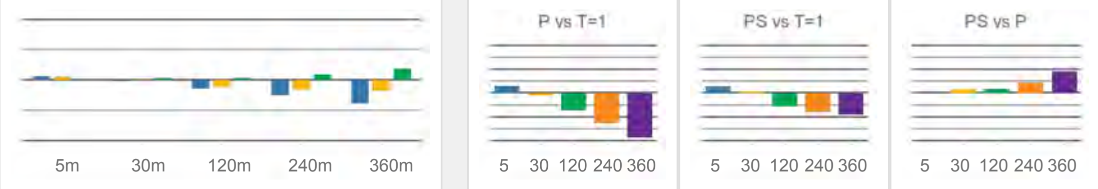

Locus		5m	30m	120m	240m	360m
**PGN_2000**	P vs T=1	1.363	1.079	1.177	2.069	2.576
	PS vs T=1	2.170	2.273	1.925	1.874	1.647
	PS vs P	0.766	1.039	0.551	0.018	-0.672

hypothetical protein

hypothetical proteins

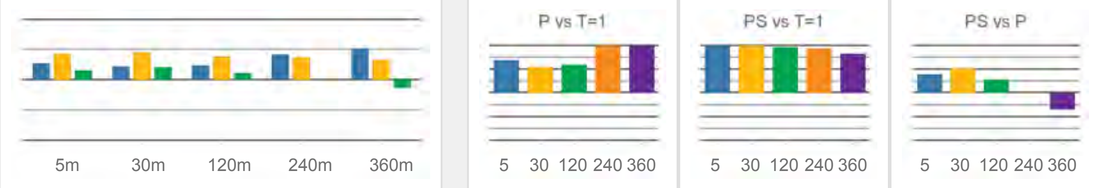

Locus		5m	30m	120m	240m	360m
**PGN_2001**	P vs T=1	-0.156	-0.396	-0.900	-1.096	-0.490
porY	PS vs T=1	0.463	0.325	0.549	0.663	0.398
	PS vs P	0.607	0.689	1.354	1.591	0.861

Por secretion system protein porY/putative sensor histidine kinase

hypothetical proteins

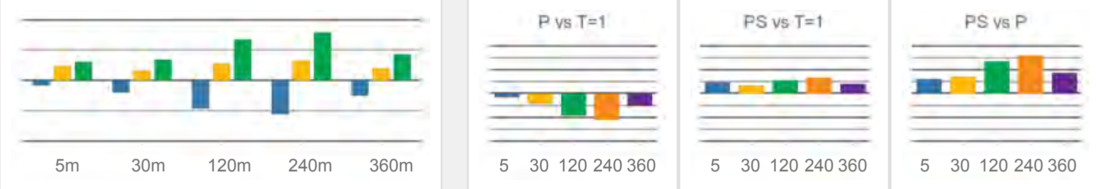

Locus		5m	30m	120m	240m	360m
**PGN_2002**	P vs T=1	1.042	-0.171	-0.599	-0.311	0.143
	PS vs T=1	0.870	0.435	0.234	0.251	-0.227
	PS vs P	0.029	0.446	0.473	0.389	-0.247

conserved hypothetical protein

hypothetical proteins-Conserved

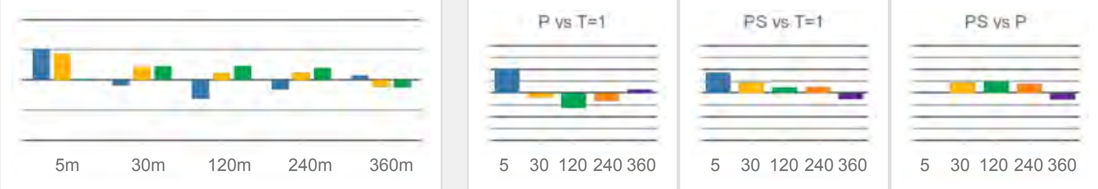

Locus		5m	30m	120m	240m	360m
**PGN_2003**	P vs T=1	0.194	-0.311	-1.021	-1.137	-1.055
	PS vs T=1	0.145	-0.503	-1.052	-1.135	-1.252
	PS vs P	-0.010	-0.176	-0.055	-0.027	-0.207

single-stranded-DNA-specific exonuclease

DNA metabolism

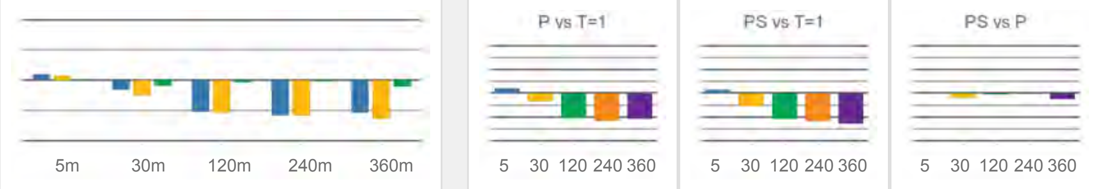

PgSg Time Course

log2 (Fold Change)

P vs T=1 | PS vs T=1 | PS vs P

PGN_2004

Locus	5m	30m	120m	240m	360m
P vs T=1	0.569	0.124	-0.231	-0.305	-0.173
PS vs T=1	0.669	0.112	-0.669	-0.827	-0.748
PS vs P	0.156	0.003	-0.442	-0.521	-0.555

conserved hypothetical protein

hypothetical proteins-Conserved

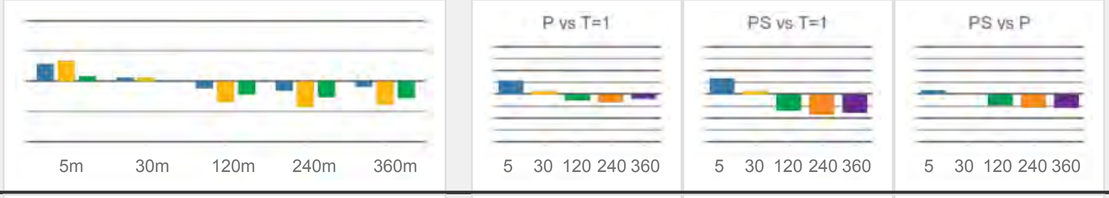

PGN_2005

Locus	5m	30m	120m	240m	360m
P vs T=1	0.036	-0.713	-1.840	-1.964	-1.935
PS vs T=1	-0.063	-0.305	-0.074	-0.202	-0.623
PS vs P	-0.032	0.383	1.611	1.601	1.213

conserved hypothetical protein

hypothetical proteins-Conserved

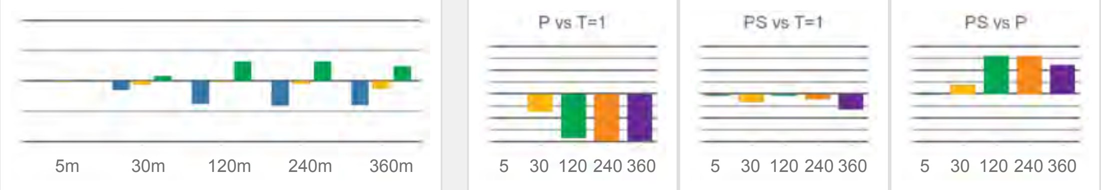

PGN_2006

Locus	5m	30m	120m	240m	360m
P vs T=1	-0.422	-0.955	-1.921	-2.092	-2.193
PS vs T=1	0.002	-0.618	-0.956	-1.202	-1.525
PS vs P	0.447	0.330	0.887	0.792	0.601

nicotinate phosphoribosyltransferase

biosynthesis of cofactors, prosthetic groups, and carriers

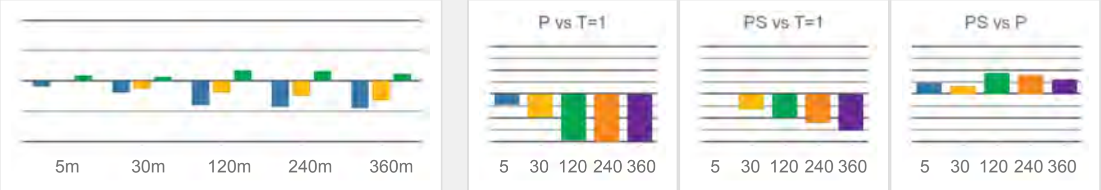

PGN_2007

Locus	5m	30m	120m	240m	360m
P vs T=1	0.712	0.425	-0.116	-0.404	-0.499
PS vs T=1	1.477	1.200	0.142	-0.424	-0.756
PS vs P	0.777	0.773	0.233	-0.078	-0.290

probable nicotinamide-nucleotide adenylyltransferase

unknown function

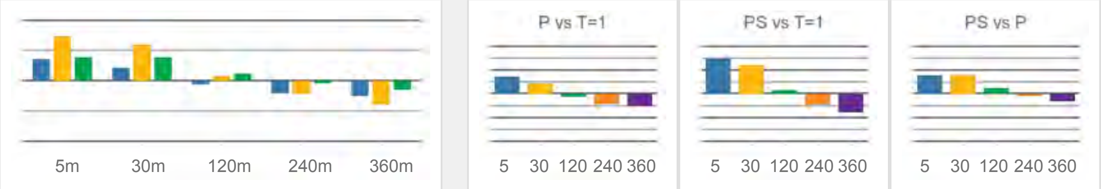

PGN_2008

Locus	5m	30m	120m	240m	360m
P vs T=1	-0.875	-0.732	-0.814	-1.237	-1.024
PS vs T=1	-0.286	-0.241	-0.717	-1.067	-1.352
PS vs P	0.554	0.474	0.088	0.049	-0.357

conserved hypothetical protein

hypothetical proteins-Conserved

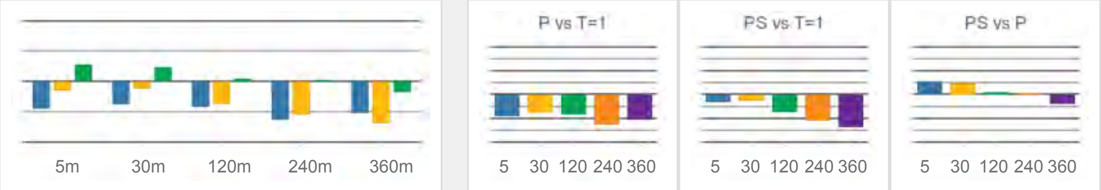

PGN_2009

Locus	5m	30m	120m	240m	360m
P vs T=1	-0.006	-0.396	-0.661	-0.663	0.067
PS vs T=1	0.992	1.076	0.395	-0.020	0.019
PS vs P	0.956	1.314	0.800	0.340	-0.001

conserved hypothetical protein

hypothetical proteins-Conserved

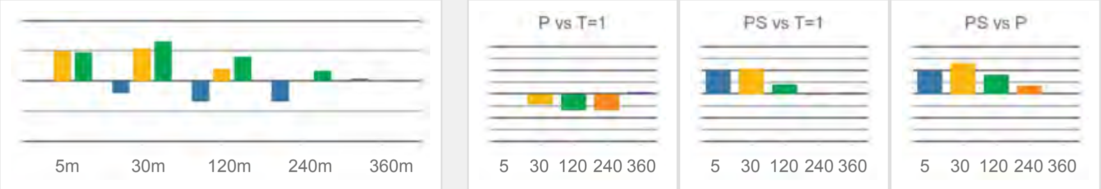

PGN_2010

Locus	5m	30m	120m	240m	360m
P vs T=1	-0.178	-0.247	-0.425	-0.339	0.302
PS vs T=1	0.255	0.200	-0.545	-0.668	-0.533
PS vs P	0.428	0.437	-0.141	-0.336	-0.784

putative secreted protein

unknown function

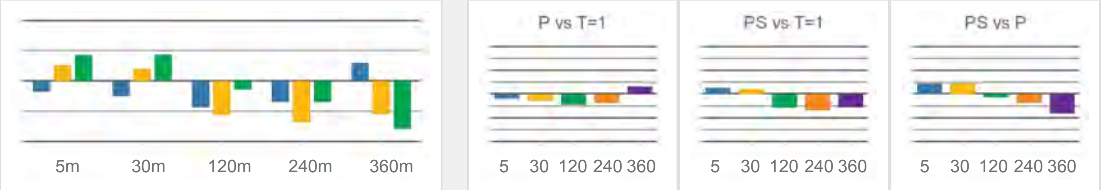

log2 (Fold Change)

Legend: P vs T=1 | PS vs T=1 | PS vs P

Locus		5m	30m	120m	240m	360m
**PGN_2011**	P vs T=1	-0.247	-0.306	-0.108	0.174	0.700
	PS vs T=1	-0.260	-0.267	-0.458	-0.594	-0.239
	PS vs P	-0.019	0.024	-0.346	-0.719	-0.876

putative helicase

DNA metabolism

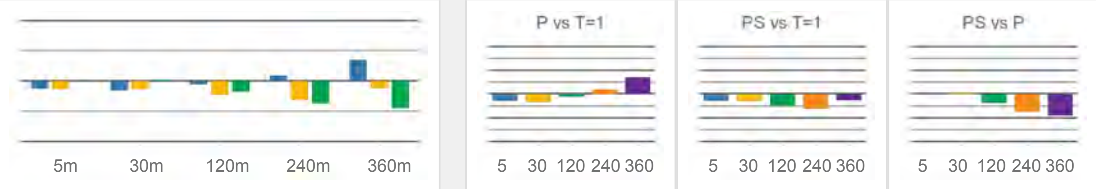

Locus		5m	30m	120m	240m	360m
**PGN_2012**	P vs T=1	-0.073	1.094	2.201	2.276	1.725
	PS vs T=1	-0.247	0.729	1.383	1.330	1.359
	PS vs P	-0.274	-0.345	-0.726	-0.859	-0.342

outer membrane efflux protein

transport and binding proteins

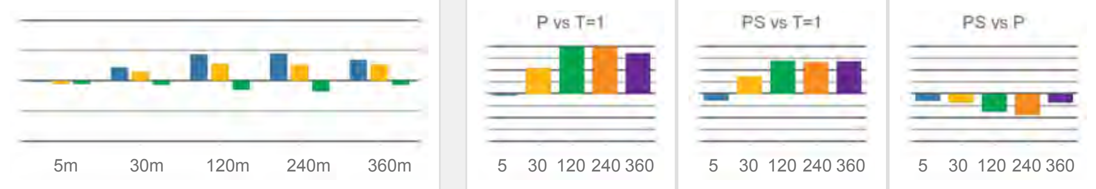

Locus		5m	30m	120m	240m	360m
**PGN_2013**	P vs T=1	0.755	1.557	2.153	1.892	1.653
	PS vs T=1	0.567	1.181	1.064	0.638	0.640
	PS vs P	-0.219	-0.331	-1.001	-1.188	-0.977

cation efflux system protein

transport and binding proteins

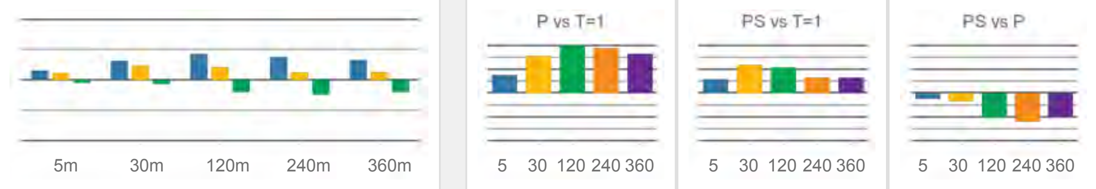

Locus		5m	30m	120m	240m	360m
**PGN_2014**	P vs T=1	1.159	1.955	2.934	2.804	2.256
	PS vs T=1	1.426	1.683	1.653	1.252	1.154
	PS vs P	0.201	-0.222	-1.115	-1.402	-1.038

cation efflux system protein

transport and binding proteins

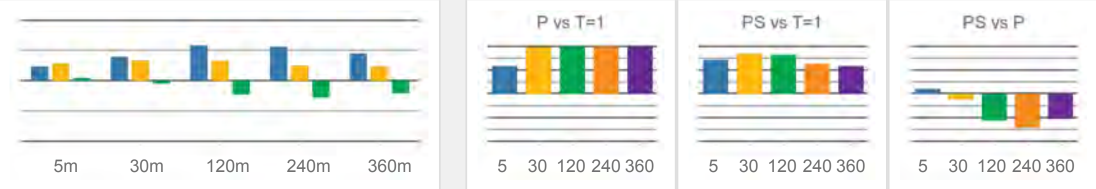

Locus		5m	30m	120m	240m	360m
**PGN_2015**	P vs T=1	2.479	2.842	3.308	2.627	1.993
	PS vs T=1	2.682	2.920	2.726	1.419	1.481
	PS vs P	0.245	0.187	-0.334	-1.030	-0.474

conserved hypothetical protein

hypothetical proteins-Conserved

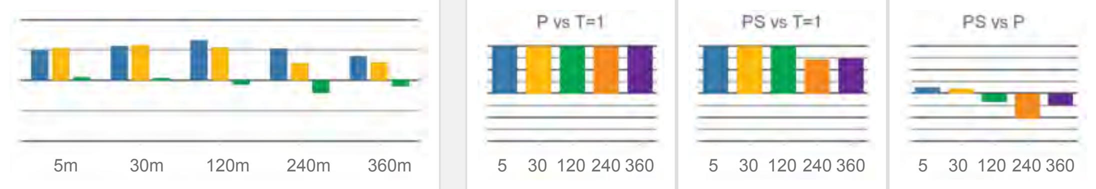

Locus		5m	30m	120m	240m	360m
**PGN_2016**	P vs T=1	0.378	0.280	-0.136	-0.218	-0.812
	PS vs T=1	-0.144	0.060	0.312	-0.092	-0.222
	PS vs P	-0.485	-0.195	0.428	0.108	0.528

hypothetical protein

hypothetical proteins

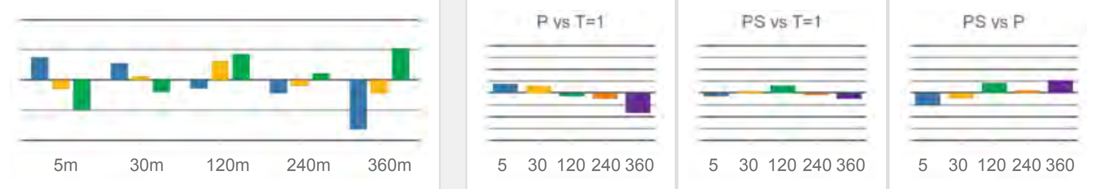

Locus		5m	30m	120m	240m	360m
**PGN_2017**	P vs T=1	-0.455	-0.238	-0.148	0.098	0.055
	PS vs T=1	-0.030	-0.207	-0.628	-0.584	-0.414
	PS vs P	0.401	0.031	-0.472	-0.644	-0.452

conserved hypothetical protein

hypothetical proteins-Conserved

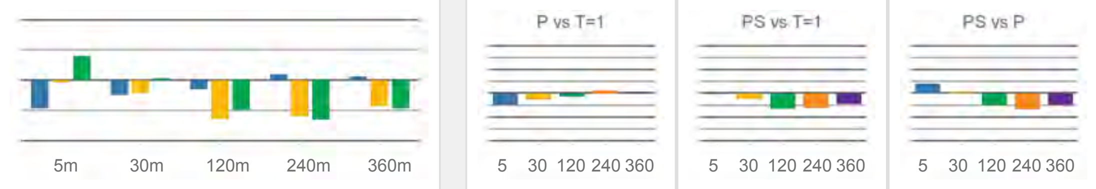

PgSg Time Course

log$_2$ (Fold Change)

Legend: P vs T=1, PS vs T=1, PS vs P

Locus		5m	30m	120m	240m	360m
**PGN_2018**	P vs T=1	-0.542	-0.018	0.160	0.153	-0.206
	PS vs T=1	-0.207	-0.216	-0.480	-0.334	-0.128
	PS vs P	0.298	-0.181	-0.609	-0.452	0.070

putative UDP-N-acetylglucosamine acyltransferase

cell envelope

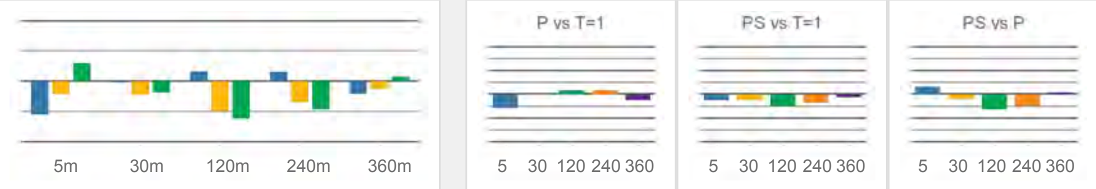

Locus		5m	30m	120m	240m	360m
**PGN_2019**	P vs T=1	0.463	0.679	0.876	1.039	1.049
	PS vs T=1	0.703	0.786	0.558	0.756	0.912
	PS vs P	0.219	0.107	-0.302	-0.251	-0.117

UDP-3-O-[3-hydroxymyristoyl] N-acetylglucosamine deacetylase

cell envelope

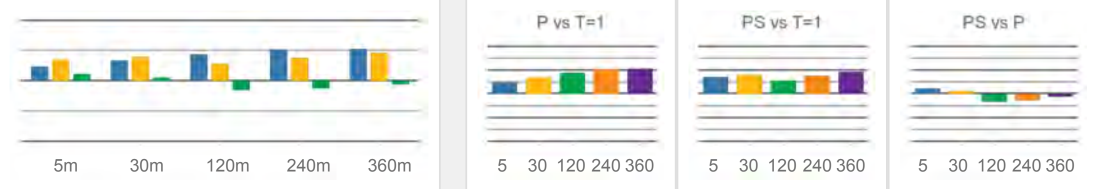

Locus		5m	30m	120m	240m	360m
**PGN_2020**	P vs T=1	0.277	0.917	1.234	1.169	0.703
	PS vs T=1	0.050	0.397	0.137	0.036	0.122
	PS vs P	-0.240	-0.480	-1.042	-1.082	-0.569

UDP-3-O-[3-hydroxymyristoyl] glucosamine N-acyltransferase

cell envelope

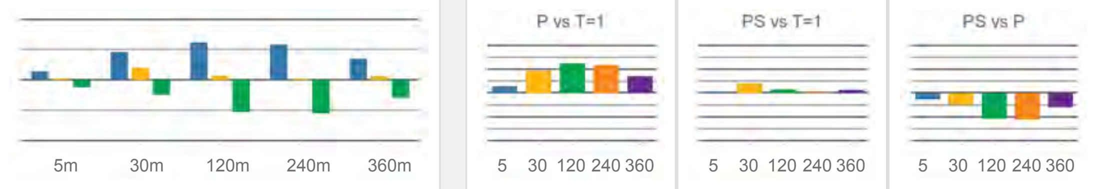

Locus		5m	30m	120m	240m	360m
**PGN_2021**	P vs T=1	-0.147	0.592	0.625	0.174	-0.602
	PS vs T=1	-0.246	0.109	-0.328	-0.783	-1.082
	PS vs P	-0.110	-0.435	-0.898	-0.924	-0.502

orotidine 5'-phosphate decarboxylase

purines, pyrimidines, nucleosides and nucleotides

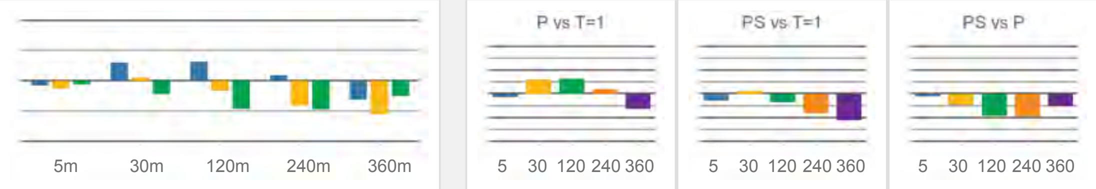

Locus		5m	30m	120m	240m	360m
**PGN_2022**	P vs T=1	-0.110	0.340	0.726	0.662	0.397
	PS vs T=1	0.100	0.191	-0.456	-0.605	-0.659
	PS vs P	0.185	-0.131	-1.124	-1.206	-1.030

peptide chain release factor 1

protein synthesis

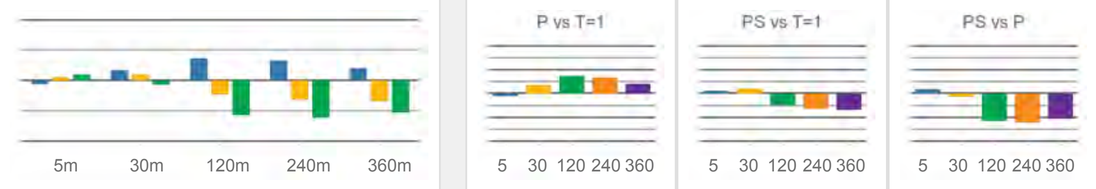

Locus		5m	30m	120m	240m	360m
**PGN_2023**	P vs T=1	-0.610	-0.467	-0.679	-0.567	-0.751
	PS vs T=1	-0.552	-0.790	-1.397	-1.476	-1.099
	PS vs P	0.058	-0.301	-0.712	-0.874	-0.349

putative phosphoribosylformylglycinamidine cyclo-ligase

purines, pyrimidines, nucleosides and nucleotides

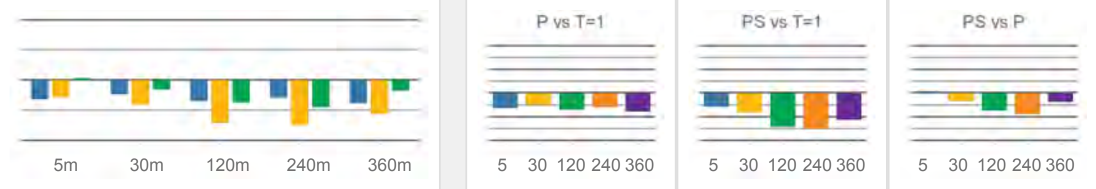

Locus		5m	30m	120m	240m	360m
**PGN_2024**	P vs T=1	-0.501	-1.204	-1.788	-1.818	-1.865
	PS vs T=1	-0.927	-1.524	-1.860	-1.910	-1.999
	PS vs P	-0.359	-0.309	-0.107	-0.121	-0.158

putative hemagglutinin

cell envelope

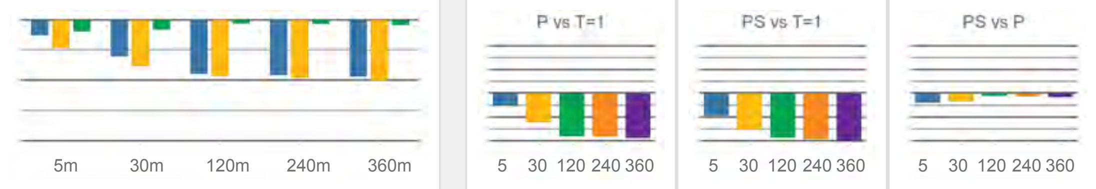

PgSg Time Course

Hackett Laboratory UW

log$_2$ (Fold Change)

Legend: P vs T=1 | PS vs T=1 | PS vs P

Locus		5m	30m	120m	240m	360m
**PGN_2025**	P vs T=1	-1.827	-2.227	-2.158	-2.038	-1.371
	PS vs T=1	-1.638	-1.544	-0.654	-0.741	-0.889
	PS vs P	0.132	0.525	1.342	1.126	0.482
	conserved hypothetical protein					
	hypothetical proteins-Conserved					
**PGN_2026**	P vs T=1	-1.404	-1.838	-1.759	-1.566	-0.972
	PS vs T=1	-1.578	-1.086	-0.107	0.077	-0.061
	PS vs P	-0.219	0.580	1.481	1.486	0.892
	putative abortive infection protein					
	cellular processes					
**PGN_2027**	P vs T=1	0.331	-0.361	-0.574	0.465	3.124
	PS vs T=1	-0.138	0.233	0.051	0.809	1.010
	PS vs P	-0.298	0.185	-0.072	0.261	-0.875
	conserved hypothetical protein					
	hypothetical proteins-Conserved					
**PGN_2028**	P vs T=1	-0.422	-0.449	-0.400	-0.252	0.470
	PS vs T=1	-0.504	-0.406	-0.100	0.053	-0.072
	PS vs P	-0.091	0.029	0.278	0.288	-0.509
	conserved hypothetical protein					
	hypothetical proteins-Conserved					
**PGN_2029**	P vs T=1	-0.457	-0.161	-0.092	-0.213	0.085
	PS vs T=1	-0.749	-0.303	0.269	0.007	0.074
	PS vs P	-0.304	-0.135	0.367	0.200	0.004
	conserved hypothetical protein					
	hypothetical proteins-Conserved					
**PGN_2030**	P vs T=1	-0.526	0.054	0.869	0.794	0.752
	PS vs T=1	-0.447	0.017	0.267	0.652	0.685
	PS vs P	0.008	-0.051	-0.539	-0.100	-0.046
	conserved hypothetical protein					
	hypothetical proteins-Conserved					
**PGN_2031**	P vs T=1	-1.614	-1.104	-0.502	-0.126	-0.209
	PS vs T=1	-0.996	-0.845	-0.701	-0.726	-0.737
	PS vs P	0.492	0.204	-0.186	-0.533	-0.503
	L-serine dehydratase					
	energy metabolism					

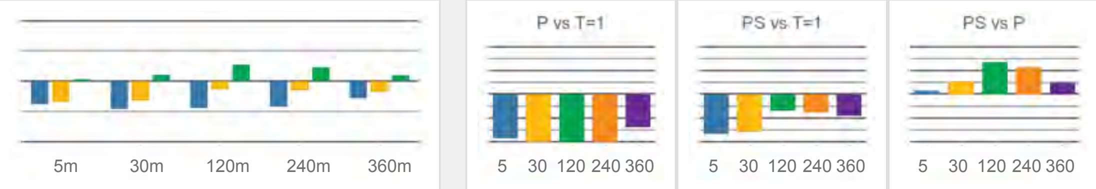

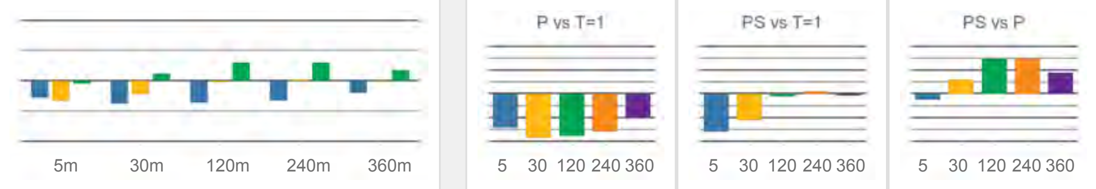

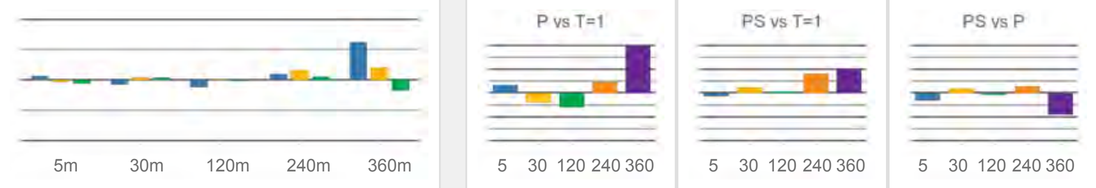

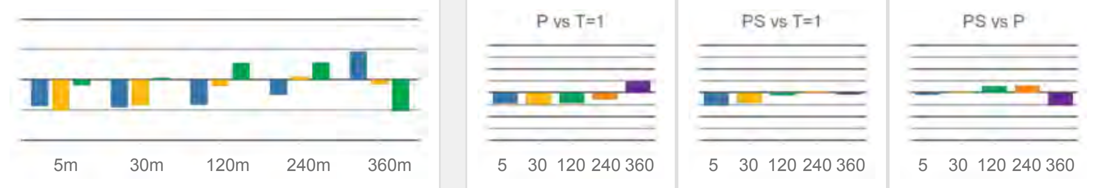

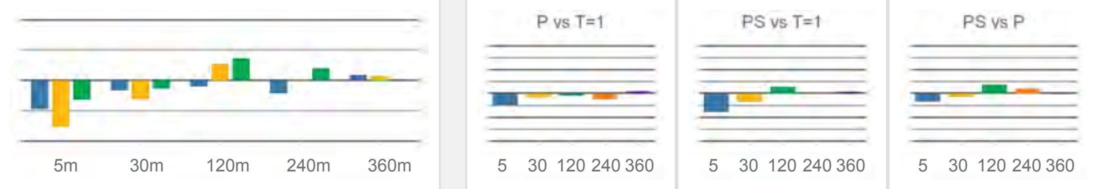

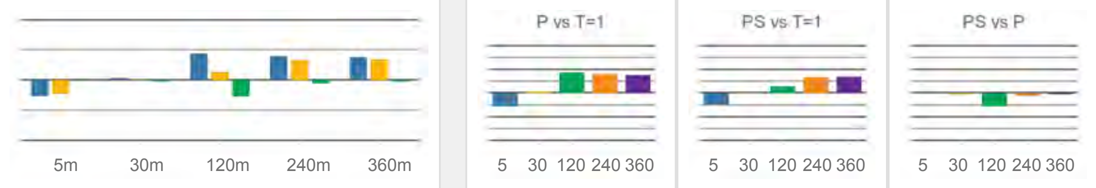

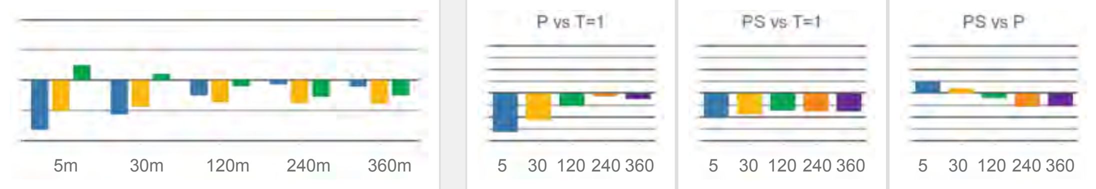

PgSg Time Course

log$_2$ (Fold Change)

Legend: P vs T=1 | PS vs T=1 | PS vs P

Locus		5m	30m	120m	240m	360m
**PGN_2032**	P vs T=1	-0.646	-0.626	-0.612	-0.542	-0.262
	PS vs T=1	-0.321	-0.796	-1.308	-1.167	-0.913
	PS vs P	0.321	-0.166	-0.683	-0.598	-0.617
	putative alpha-galactosidase					
	central intermediary metabolism					
**PGN_2033**	P vs T=1	0.370	0.745	1.172	1.043	0.885
	PS vs T=1	0.172	0.021	0.008	-0.016	0.176
	PS vs P	-0.196	-0.677	-1.082	-0.987	-0.671
	ATP-dependent RNA helicase					
	transcription					
**PGN_2034**	P vs T=1	0.190	0.153	-0.164	-0.041	0.254
	PS vs T=1	-0.553	-0.479	-0.347	-0.001	-0.013
	PS vs P	-0.705	-0.592	-0.195	0.047	-0.232
	probable sugar isomerase					
	unknown function					
**PGN_2035**	P vs T=1	-0.723	-1.638	-1.960	-1.353	-0.273
	PS vs T=1	-0.809	-1.409	-1.513	-1.198	-1.084
	PS vs P	-0.049	0.155	0.240	0.114	-0.719
	putative peptidase					
	protein fate					
**PGN_2036**	P vs T=1	-0.508	-0.592	-1.478	-0.054	1.592
	PS vs T=1	-0.587	-0.113	0.137	-0.045	0.765
	PS vs P	-0.157	0.303	0.555	0.045	-0.160
	hypothetical protein					
	hypothetical proteins					
**PGN_2037**	P vs T=1	3.047	3.404	2.797	2.105	1.121
dps	PS vs T=1	1.829	2.457	1.713	0.769	0.362
	PS vs P	-1.138	-0.839	-1.024	-1.312	-0.780
	DNA-binding protein from starved cells Dps					
	cellular processes					
**PGN_2038**	P vs T=1	-0.724	-0.851	-0.843	-0.840	-0.656
	PS vs T=1	-0.254	-0.486	-0.944	-1.365	-1.168
	PS vs P	0.466	0.352	-0.104	-0.520	-0.499
	conserved hypothetical protein					
	transport and binding proteins					

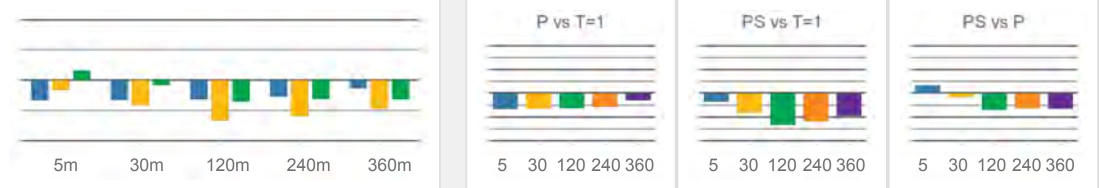

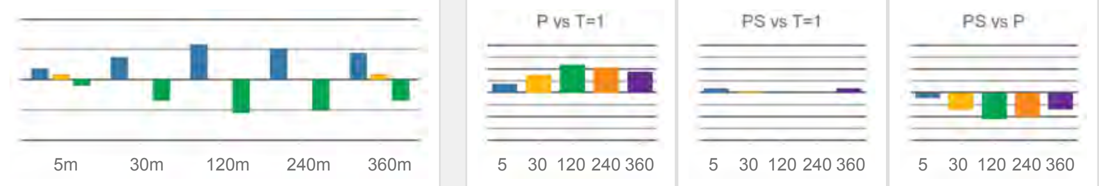

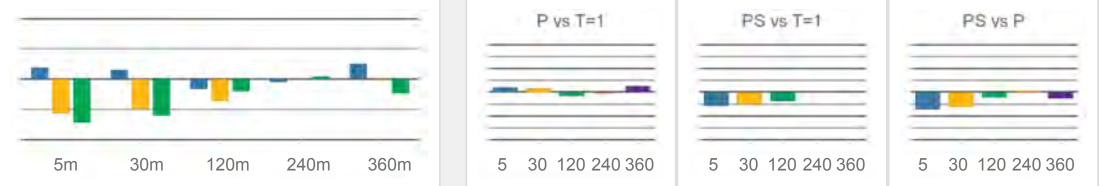

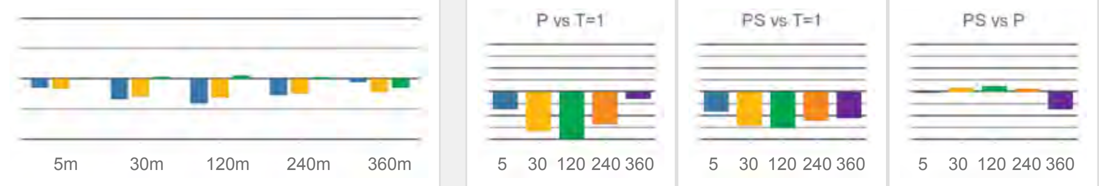

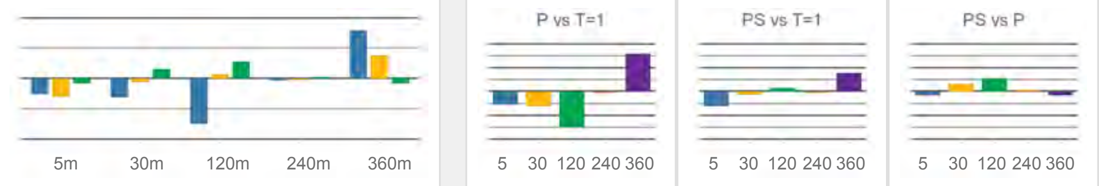

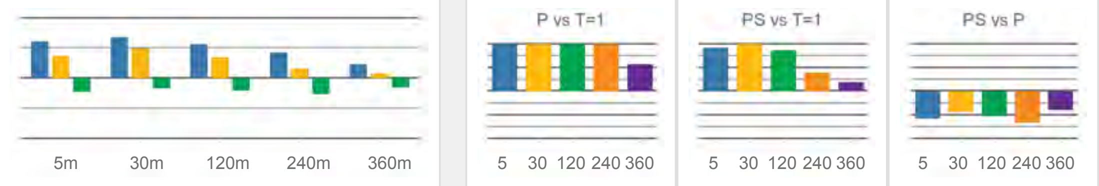

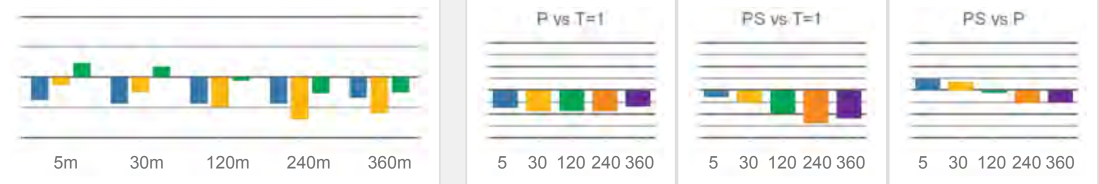

log$_2$ (Fold Change)

Locus		5m	30m	120m	240m	360m
**PGN_2039**	P vs T=1	-1.208	-1.638	-1.684	-1.311	-0.569
	PS vs T=1	-1.324	-2.029	-2.227	-2.034	-1.471
	PS vs P	-0.089	-0.415	-0.591	-0.681	-0.805

conserved hypothetical protein

transport and binding proteins

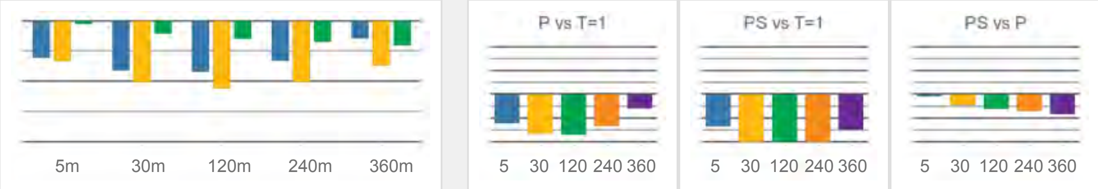

Locus		5m	30m	120m	240m	360m
**PGN_2040**	P vs T=1	-0.939	-0.794	-0.526	-0.482	-0.420
	PS vs T=1	-0.973	-0.983	-1.339	-0.899	-0.911
	PS vs P	-0.053	-0.191	-0.780	-0.380	-0.466

conserved hypothetical protein

transport and binding proteins

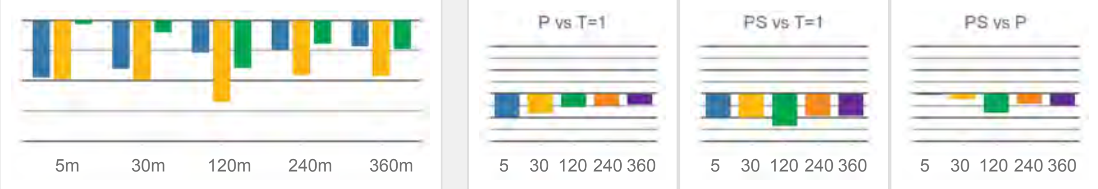

Locus		5m	30m	120m	240m	360m
**PGN_2041**	P vs T=1	-0.002	0.071	0.298	0.388	0.312
	PS vs T=1	-0.448	-0.724	-0.890	-0.408	-0.506
	PS vs P	-0.430	-0.769	-1.147	-0.746	-0.790

putative alkaline protease AprF

transport and binding proteins

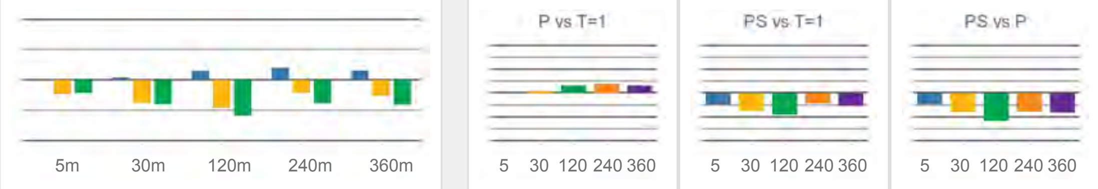

Locus		5m	30m	120m	240m	360m
**PGN_2042**	P vs T=1	-0.247	0.056	0.013	-0.373	-0.310
	PS vs T=1	0.163	-0.072	-0.848	-0.996	-0.997
	PS vs P	0.403	-0.100	-0.823	-0.618	-0.674

DNA mismatch repair protein MutS

DNA metabolism

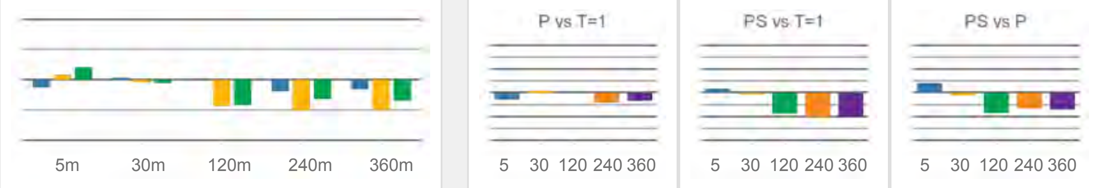

Locus		5m	30m	120m	240m	360m
**PGN_2043**	P vs T=1	-0.619	-0.234	-0.468	-1.122	-1.851
	PS vs T=1	-0.565	-0.511	-1.150	-1.536	-1.683
	PS vs P	0.055	-0.248	-0.654	-0.428	0.116

conserved hypothetical protein

hypothetical proteins-Conserved

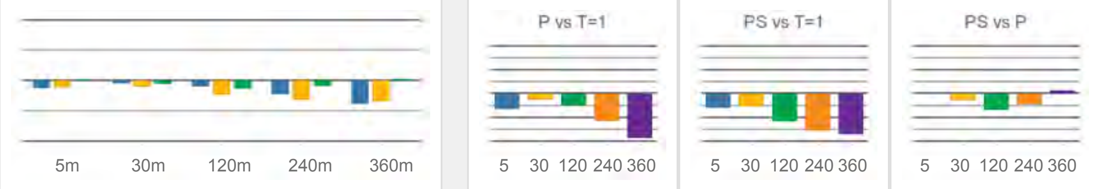

Locus		5m	30m	120m	240m	360m
**PGN_2044**	P vs T=1	-0.122	0.392	-0.615	-1.409	-2.473
	PS vs T=1	0.313	-0.074	-0.842	-0.975	-1.245
	PS vs P	0.450	-0.318	-0.222	0.101	0.440

conserved hypothetical protein

hypothetical proteins-Conserved

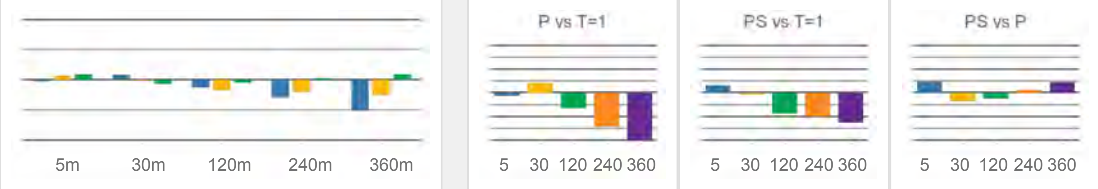

Locus		5m	30m	120m	240m	360m
**PGN_2045**	P vs T=1	-0.027	0.247	-0.460	-0.768	-0.814
	PS vs T=1	0.068	-0.313	-1.044	-0.966	-1.077
	PS vs P	0.104	-0.531	-0.583	-0.215	-0.272

phenylalanyl-tRNA synthetase beta subunit

protein synthesis

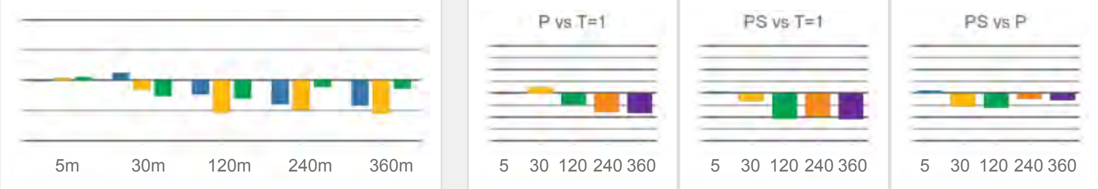

PgSg Time Course

log$_2$ (Fold Change)

Legend: P vs T=1 | PS vs T=1 | PS vs P

Locus		5m	30m	120m	240m	360m
**PGN_2046**	P vs T=1	-2.228	-1.550	-1.910	-1.976	-2.222
	PS vs T=1	-2.345	-2.221	-2.248	-2.185	-1.838
	PS vs P	-0.294	-0.483	-0.341	-0.229	0.254

conserved hypothetical protein

hypothetical proteins-Conserved

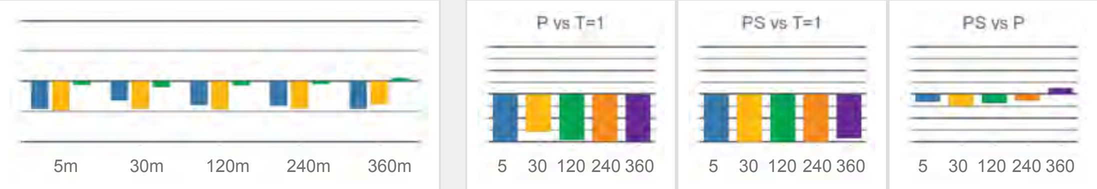

Locus		5m	30m	120m	240m	360m
**PGN_2047**	P vs T=1	1.798	2.235	3.017	3.099	2.121
	PS vs T=1	2.274	2.721	2.798	3.027	2.990
	PS vs P	0.388	0.481	-0.057	0.085	0.835

conserved hypothetical protein

hypothetical proteins-Conserved

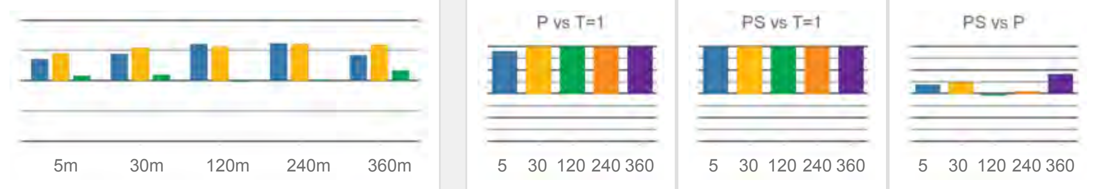

Locus		5m	30m	120m	240m	360m
**PGN_2048**	P vs T=1	-1.726	-1.016	-0.508	-0.832	-1.440
	PS vs T=1	-1.610	-1.165	-1.056	-0.832	-0.712
	PS vs P	-0.188	-0.153	-0.402	-0.005	0.548

conserved hypothetical protein

hypothetical proteins-Conserved

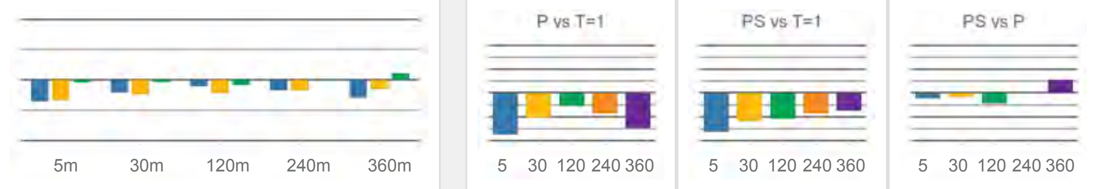

Locus		5m	30m	120m	240m	360m
**PGN_2049**	P vs T=1	-1.000	-0.761	-1.236	-1.724	-2.516
	PS vs T=1	-1.325	-1.285	-1.969	-2.873	-2.793
	PS vs P	-0.235	-0.347	-0.635	-1.113	-0.379

conserved hypothetical protein

hypothetical proteins-Conserved

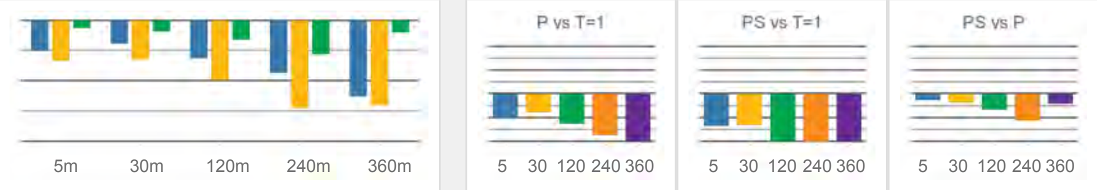

Locus		5m	30m	120m	240m	360m
**PGN_2050**	P vs T=1	-0.592	-0.662	-0.804	-0.273	0.159
	PS vs T=1	0.517	0.092	-0.560	-0.613	-0.355
	PS vs P	1.084	0.728	0.205	-0.321	-0.486

ATP-dependent helicase

unknown function

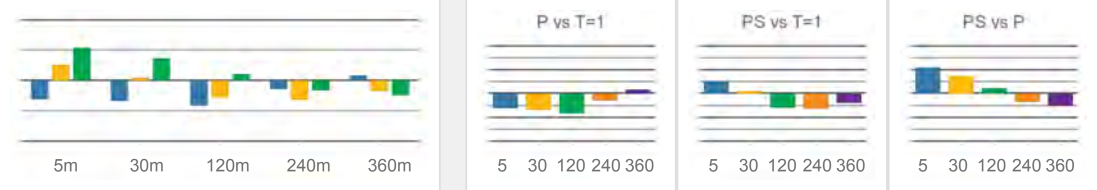

Locus		5m	30m	120m	240m	360m
**PGN_2051**	P vs T=1	-1.089	-1.154	-0.996	-0.554	-0.205
	PS vs T=1	-0.137	-0.662	-1.296	-1.538	-1.201
	PS vs P	0.914	0.445	-0.318	-0.892	-0.917

conserved hypothetical protein

hypothetical proteins-Conserved

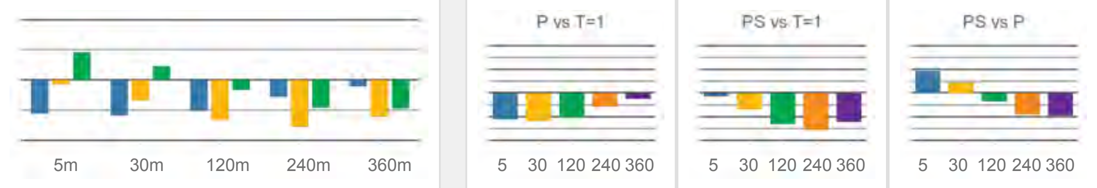

Locus		5m	30m	120m	240m	360m
**PGN_2052**	P vs T=1	-1.553	-2.034	-2.221	-1.846	-1.133
	PS vs T=1	-0.874	-1.414	-2.388	-2.557	-2.200
	PS vs P	0.682	0.578	-0.237	-0.686	-0.993

conserved hypothetical protein

hypothetical proteins-Conserved

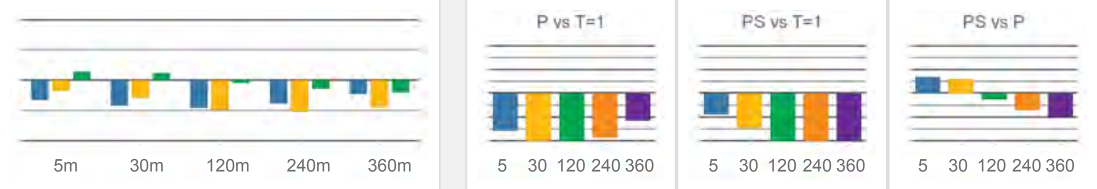

log$_2$ (Fold Change)

Legend: P vs T=1 | PS vs T=1 | PS vs P

Locus		5m	30m	120m	240m	360m
**PGN_2053**	P vs T=1	-1.033	-1.093	-1.386	-1.608	-1.592
	PS vs T=1	-0.614	-0.711	-1.591	-2.063	-1.894
	PS vs P	0.415	0.379	-0.217	-0.489	-0.322

conserved hypothetical protein

unknown function

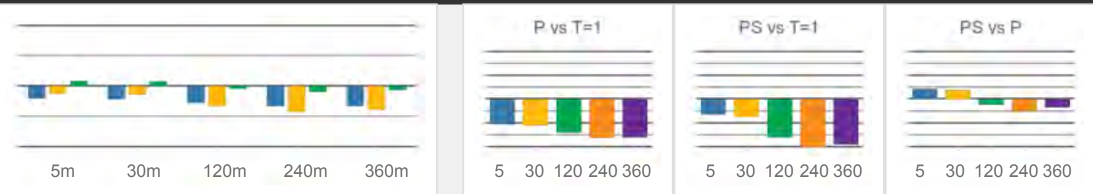

Locus		5m	30m	120m	240m	360m
**PGN_2054**	P vs T=1	0.226	0.605	0.621	0.190	-0.343
	PS vs T=1	0.706	0.836	0.054	-0.458	-0.455
	PS vs P	0.463	0.257	-0.517	-0.634	-0.149

putative glycerol-3-phosphate cytidylyltransferase

cell envelope

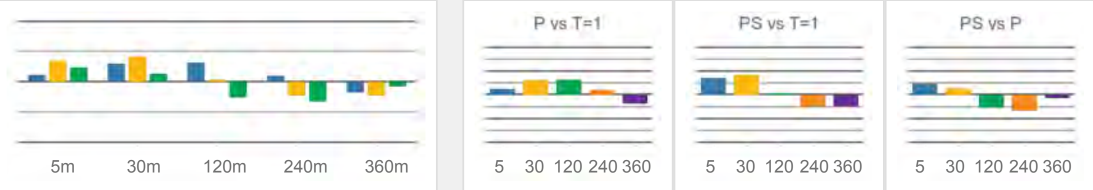

Locus		5m	30m	120m	240m	360m
**PGN_2055**	P vs T=1	-0.825	-0.565	-0.710	-1.071	-1.321
PdxA	PS vs T=1	-0.281	-0.434	-1.084	-1.180	-1.319
	PS vs P	0.524	0.138	-0.365	-0.136	-0.031

putative 4-hydroxythreonine-4-phosphate dehydrogenase

biosynthesis of cofactors, prosthetic groups, and carriers

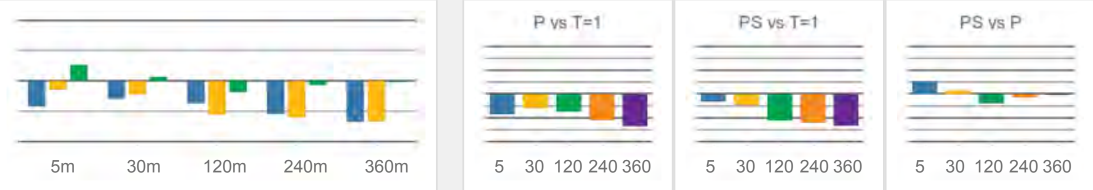

Locus		5m	30m	120m	240m	360m
**PGN_2056**	P vs T=1	-0.621	-0.778	-1.135	-1.239	-1.217
	PS vs T=1	-0.412	-0.784	-1.345	-1.453	-1.485
	PS vs P	0.216	-0.001	-0.217	-0.227	-0.274

conserved hypothetical protein

hypothetical proteins-Conserved

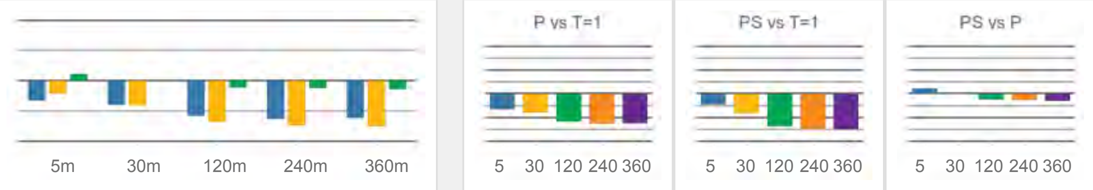

Locus		5m	30m	120m	240m	360m
**PGN_2057**	P vs T=1	-0.655	-0.457	-0.333	-0.276	0.352
	PS vs T=1	-0.442	-0.501	-0.503	-0.162	-0.384
	PS vs P	0.176	-0.060	-0.184	0.090	-0.683

conserved hypothetical protein

hypothetical proteins-Conserved

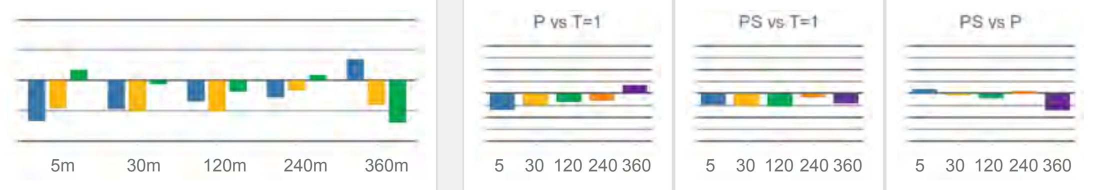

Locus		5m	30m	120m	240m	360m
**PGN_2058**	P vs T=1	0.551	0.572	1.073	1.991	2.162
	PS vs T=1	0.317	0.517	1.279	1.660	1.529
	PS vs P	-0.281	-0.139	0.159	-0.052	-0.434

conserved hypothetical protein

hypothetical proteins-Conserved

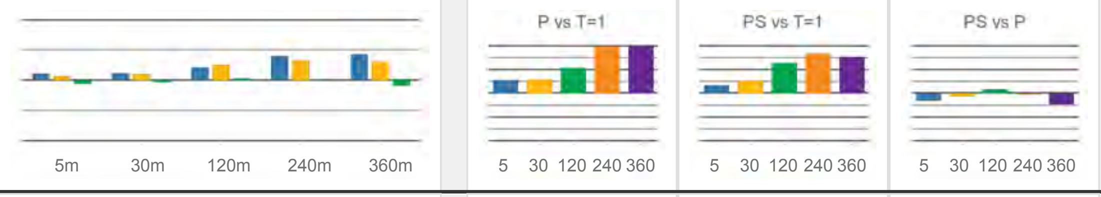

Locus		5m	30m	120m	240m	360m
**PGN_2059**	P vs T=1	-0.002	0.517	0.691	1.216	0.828
	PS vs T=1	-0.042	0.228	0.445	0.624	0.569
	PS vs P	-0.113	-0.242	-0.203	-0.363	-0.196

conserved hypothetical protein

hypothetical proteins-Conserved

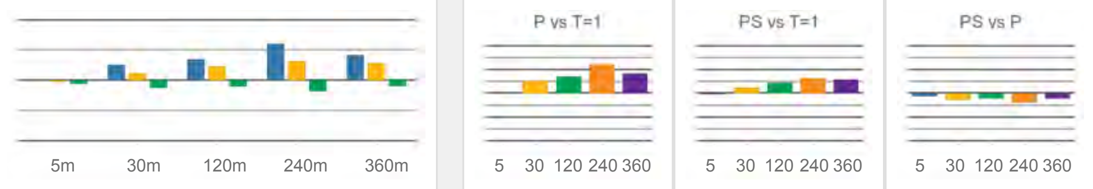

log2 (Fold Change)

P vs T=1 | PS vs T=1 | PS vs P

Locus		5m	30m	120m	240m	360m
**PGN_2060**	P vs T=1	0.199	0.744	0.724	0.312	-0.150
	PS vs T=1	0.569	0.683	0.586	0.844	0.667
	PS vs P	0.353	-0.049	-0.125	0.518	0.790
	histidyl-tRNA synthetase					
	protein synthesis					

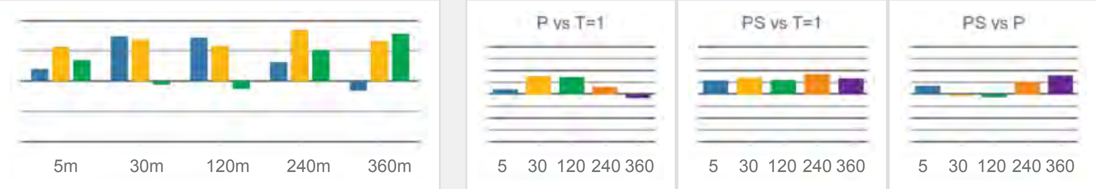

Locus		5m	30m	120m	240m	360m
**PGN_2061**	P vs T=1	1.006	1.119	0.594	0.243	0.400
	PS vs T=1	1.362	1.530	1.272	1.315	0.838
	PS vs P	0.356	0.421	0.646	0.958	0.406
	probable dihydrofolate reductase					
	biosynthesis of cofactors, prosthetic groups, and carriers					

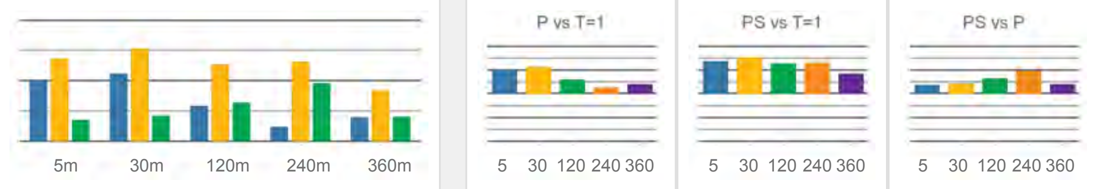

Locus		5m	30m	120m	240m	360m
**PGN_2062**	P vs T=1	0.625	0.230	-0.248	-0.401	-0.793
	PS vs T=1	0.433	-0.015	-0.478	-0.439	-0.598
	PS vs P	-0.125	-0.217	-0.242	-0.059	0.147
	thymidylate synthase					
	purines, pyrimidines, nucleosides and nucleotides					

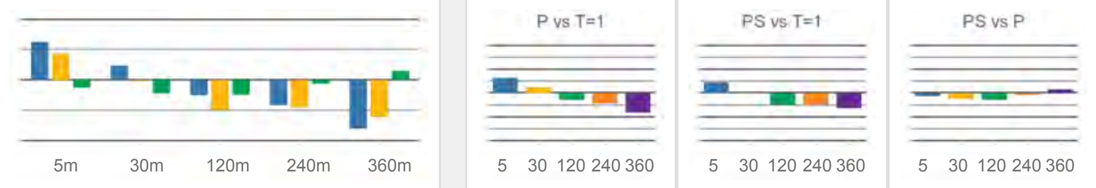

Locus		5m	30m	120m	240m	360m
**PGN_2063**	P vs T=1	0.517	0.376	1.217	2.330	4.083
	PS vs T=1	-0.548	0.421	1.166	0.759	1.989
	PS vs P	-0.646	-0.100	0.144	-0.252	-0.182
	hypothetical protein					
	hypothetical proteins					

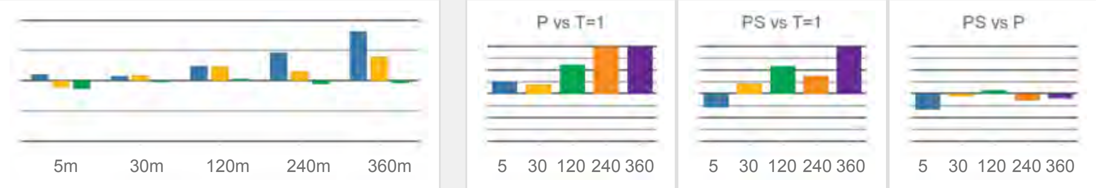

Locus		5m	30m	120m	240m	360m
**PGN_2064**	P vs T=1	2.292	2.295	1.748	1.347	0.812
	PS vs T=1	1.549	1.956	1.981	1.790	1.538
	PS vs P	-0.668	-0.267	0.245	0.412	0.668
	putative peptidase M48 family					
	hypothetical proteins-Conserved					

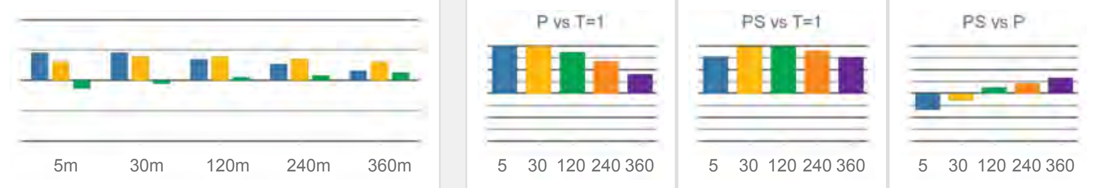

Locus		5m	30m	120m	240m	360m
**PGN_2065**	P vs T=1	0.248	0.417	0.579	0.269	-0.586
	PS vs T=1	-0.264	0.130	-0.314	-0.830	-1.024
	PS vs P	-0.494	-0.257	-0.843	-1.071	-0.468
	putative Lys- and Rgp- gingipain domain protein					
	hypothetical proteins					

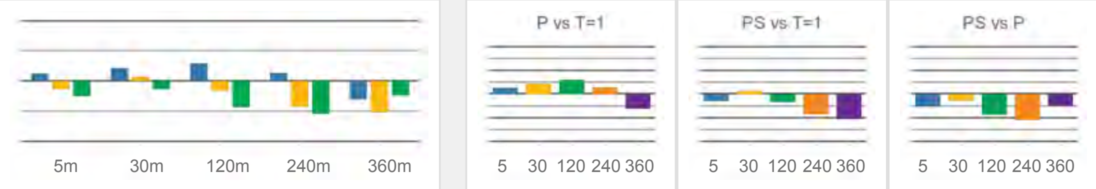

Locus		5m	30m	120m	240m	360m
**PGN_2066**	P vs T=1	-0.147	-0.101	0.143	0.740	0.952
	PS vs T=1	-0.100	-0.267	-0.454	0.130	0.335
	PS vs P	0.038	-0.173	-0.596	-0.561	-0.584
	putative ABC transporter ATP-binding protein					
	transport and binding proteins					

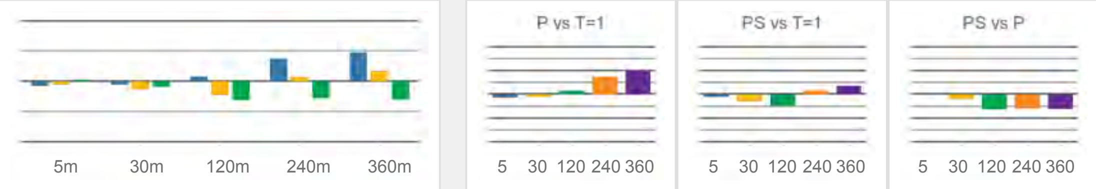

PgSg Time Course

log$_2$ (Fold Change)

Legend: P vs T=1 | PS vs T=1 | PS vs P

Locus		5m	30m	120m	240m	360m
**PGN_2067**	P vs T=1	-0.027	0.136	0.253	0.611	0.673
	PS vs T=1	-0.241	-0.380	-0.348	0.153	0.551
	PS vs P	-0.216	-0.505	-0.593	-0.423	-0.104

TPR domain protein

unknown function

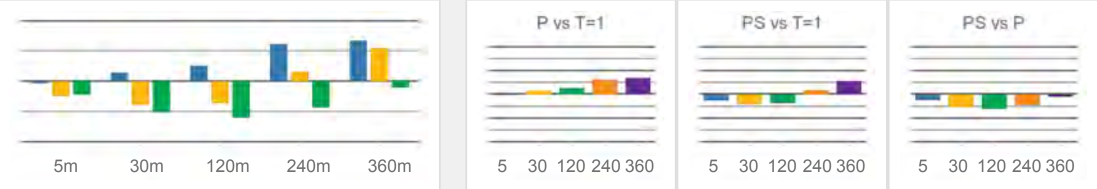

Locus		5m	30m	120m	240m	360m
**PGN_2068**	P vs T=1	0.848	1.185	1.498	1.897	2.104
	PS vs T=1	0.425	0.922	1.174	1.615	1.960
	PS vs P	-0.440	-0.249	-0.300	-0.211	-0.089

putative peptide deformylase

protein fate

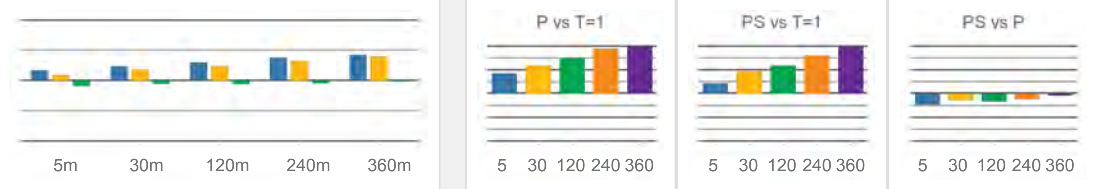

Locus		5m	30m	120m	240m	360m
**PGN_2069**	P vs T=1	-0.308	-0.448	-0.093	0.179	0.572
	PS vs T=1	-0.439	-0.403	-0.092	0.144	0.201
	PS vs P	-0.144	-0.004	-0.003	0.007	-0.303

probable endonuclease

DNA metabolism

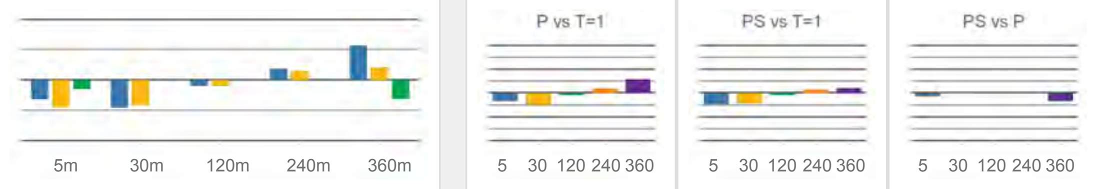

Locus		5m	30m	120m	240m	360m
**PGN_2070**	P vs T=1	0.572	1.209	1.854	1.608	1.093
	PS vs T=1	0.691	0.935	0.777	0.829	0.781
	PS vs P	0.094	-0.263	-1.039	-0.757	-0.313

conserved hypothetical protein

hypothetical proteins-Conserved

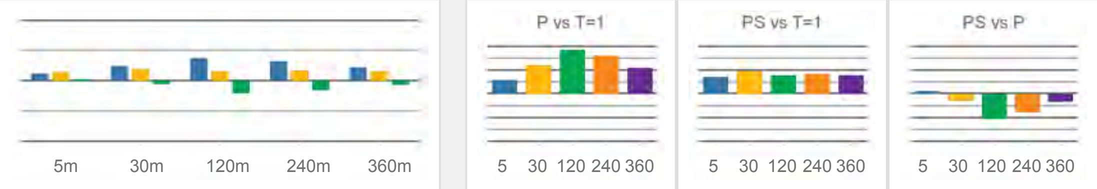

Locus		5m	30m	120m	240m	360m
**PGN_2071**	P vs T=1	0.067	0.735	1.794	1.849	1.275
	PS vs T=1	0.206	0.979	1.499	1.614	1.655
	PS vs P	0.075	0.222	-0.252	-0.197	0.375

probable ketopantoate reductase ApbA/PanE

biosynthesis of cofactors, prosthetic groups, and carriers

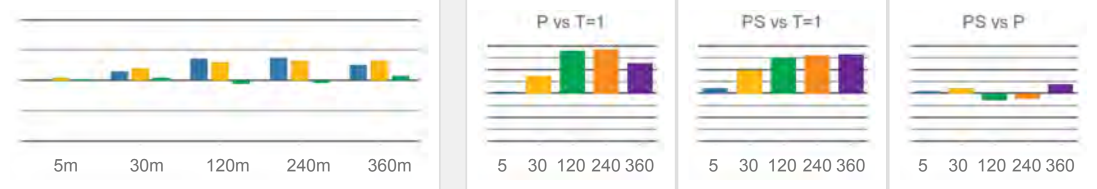

Locus		5m	30m	120m	240m	360m
**PGN_2072**	P vs T=1	0.839	0.856	0.740	0.584	0.576
	PS vs T=1	0.637	0.530	0.420	0.797	0.674
	PS vs P	-0.180	-0.299	-0.306	0.206	0.096

ABC transporter ATP-binding protein

transport and binding proteins

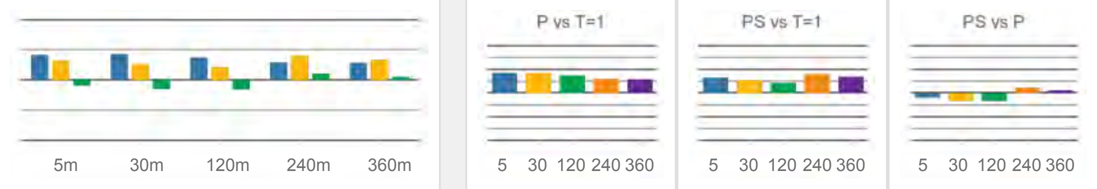

Locus		5m	30m	120m	240m	360m
**PGN_2073**	P vs T=1	-0.304	0.092	0.491	0.524	-0.062
	PS vs T=1	-0.228	-0.215	-0.098	0.199	0.247
	PS vs P	0.051	-0.302	-0.566	-0.304	0.296

conserved hypothetical protein

hypothetical proteins-Conserved

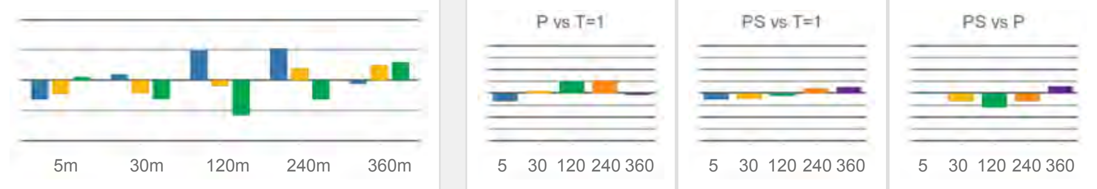

log2 (Fold Change)

Legend: P vs T=1 | PS vs T=1 | PS vs P

Locus		5m	30m	120m	240m	360m
**PGN_2074**	P vs T=1	-0.862	-0.593	-0.577	-0.527	-0.891
	PS vs T=1	-1.228	-1.195	-1.444	-1.496	-1.583
	PS vs P	-0.371	-0.552	-0.816	-0.882	-0.697

conserved hypothetical protein

hypothetical proteins-Conserved

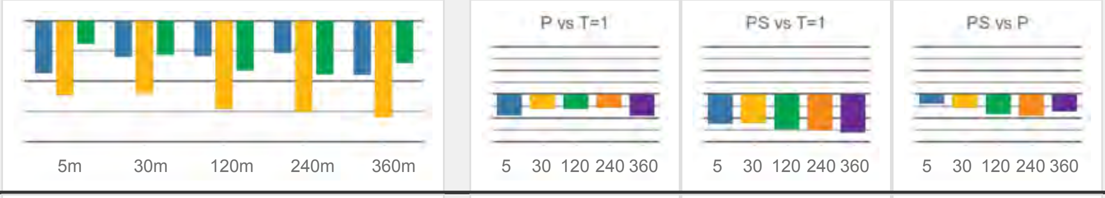

Locus		5m	30m	120m	240m	360m
**PGN_2075** *uvrAII*	P vs T=1	0.300	0.404	0.797	0.794	0.531
	PS vs T=1	0.341	-0.037	-0.627	-0.514	-0.415
	PS vs P	0.044	-0.431	-1.392	-1.268	-0.931

excinuclease ABC A subunit

DNA metabolism

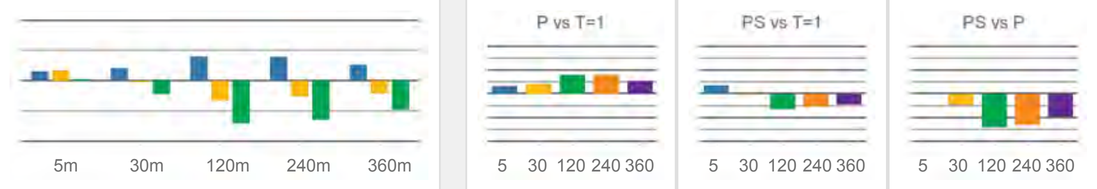

Locus		5m	30m	120m	240m	360m
**PGN_2076**	P vs T=1	2.432	2.450	2.476	1.986	1.502
	PS vs T=1	1.168	1.424	1.497	1.064	0.909
	PS vs P	-1.109	-0.872	-0.806	-0.797	-0.557

conserved hypothetical protein

hypothetical proteins-Conserved

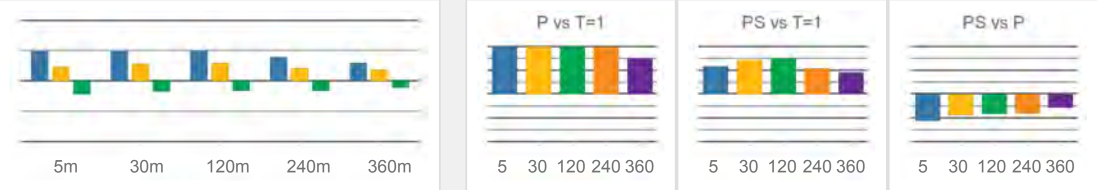

Locus		5m	30m	120m	240m	360m
**PGN_2077**	P vs T=1	2.174	2.752	3.360	3.190	2.318
	PS vs T=1	1.463	2.193	2.592	2.358	2.350
	PS vs P	-0.657	-0.387	-0.506	-0.625	0.047

conserved hypothetical protein

unknown function

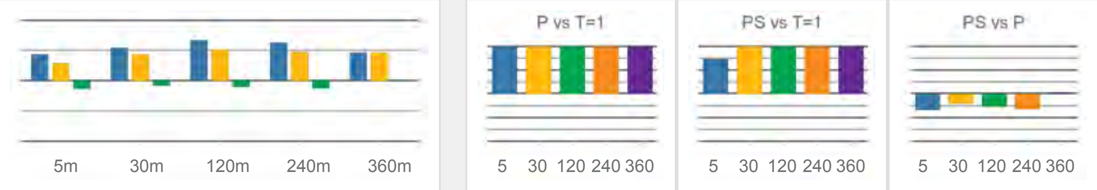

Locus		5m	30m	120m	240m	360m
**PGN_2078**	P vs T=1	0.057	0.349	0.373	0.282	0.323
	PS vs T=1	0.745	0.717	0.393	0.366	0.413
	PS vs P	0.659	0.362	0.018	0.069	0.085

conserved hypothetical protein

hypothetical proteins-Conserved

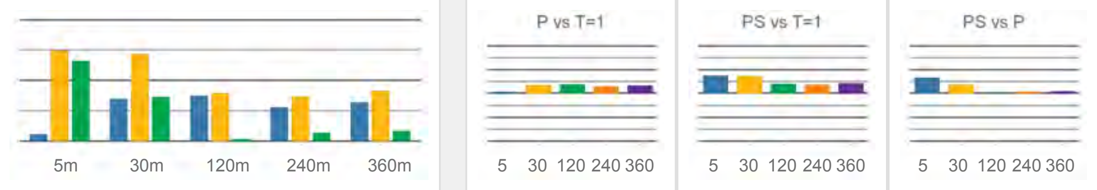

Locus		5m	30m	120m	240m	360m
**PGN_2079**	P vs T=1	0.780	1.157	0.971	0.503	-0.106
	PS vs T=1	1.395	1.438	1.237	1.122	0.694
	PS vs P	0.595	0.293	0.271	0.593	0.757

mannose-1-phosphate guanylyltransferase

cell envelope

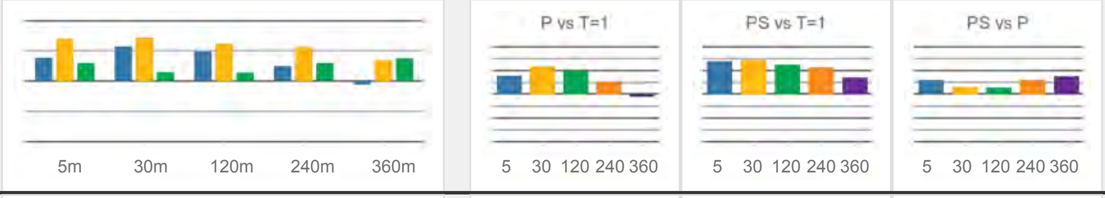

Locus		5m	30m	120m	240m	360m
**PGN_2080**	P vs T=1	0.742	0.876	0.417	0.003	0.143
	PS vs T=1	0.532	0.234	-0.346	-0.207	-0.304
	PS vs P	-0.178	-0.590	-0.736	-0.221	-0.434

conserved hypothetical protein

hypothetical proteins-Conserved

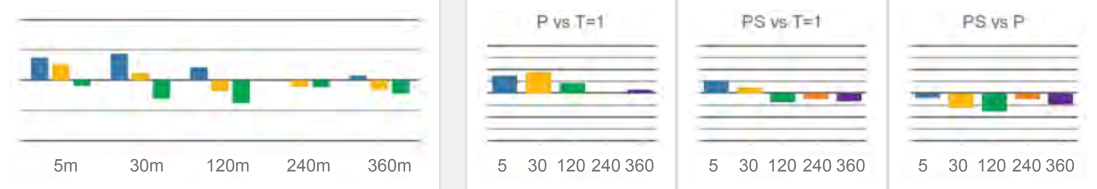

PgSg Time Course

log$_2$ (Fold Change)

Legend: P vs T=1 | PS vs T=1 | PS vs P

Locus		5m	30m	120m	240m	360m
**PGN_2081**	P vs T=1	-0.230	-0.029	0.037	-0.201	-0.194
Dxs	PS vs T=1	-0.044	-0.297	-0.441	-0.069	-0.213
	PS vs P	0.172	-0.259	-0.462	0.116	-0.026

1-deoxy-D-xylulose 5-phosphate synthase

biosynthesis of cofactors, prosthetic groups, and carriers

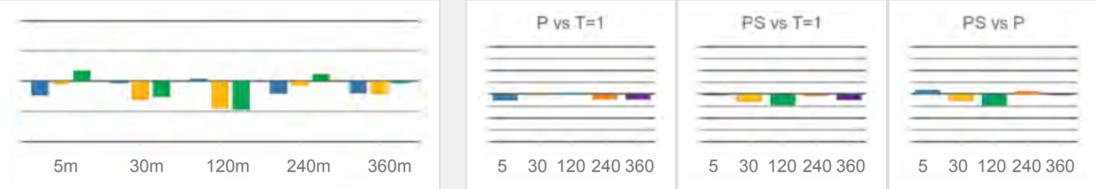

Locus		5m	30m	120m	240m	360m
**PGN_2082**	P vs T=1	-0.412	-0.212	-0.256	-0.121	0.276
	PS vs T=1	-0.285	-0.516	-0.982	-0.575	-0.368
	PS vs P	0.116	-0.293	-0.722	-0.436	-0.609

putative potassium uptake protein TrkA

transport and binding proteins

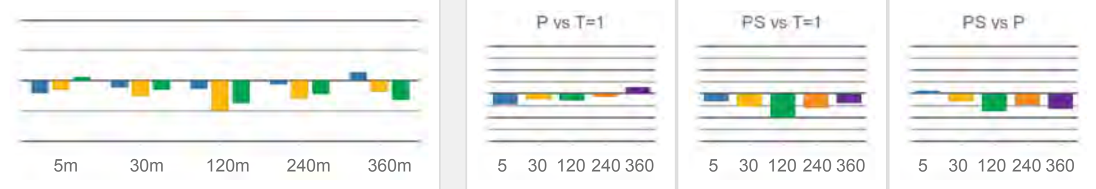

Locus		5m	30m	120m	240m	360m
**PGN_2083**	P vs T=1	-0.815	-0.786	-0.790	-0.736	-0.338
	PS vs T=1	0.084	0.240	-0.453	-0.733	-0.796
	PS vs P	0.868	0.995	0.308	-0.026	-0.449

potassium uptake protein TrkH

transport and binding proteins

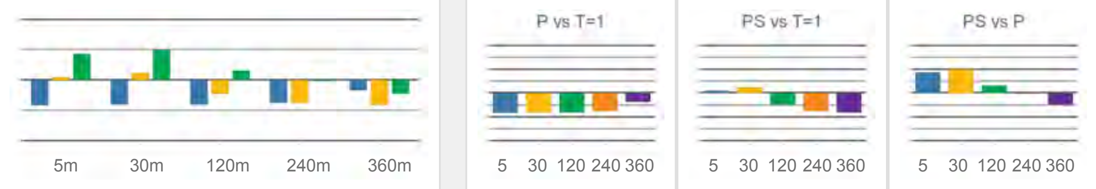

Locus		5m	30m	120m	240m	360m
**PGN_2084**	P vs T=1	-0.117	-0.335	0.526	1.409	2.040
	PS vs T=1	0.010	0.388	0.217	0.068	1.199
	PS vs P	-0.018	0.337	-0.270	-0.700	-0.329

conserved hypothetical protein

hypothetical proteins-Conserved

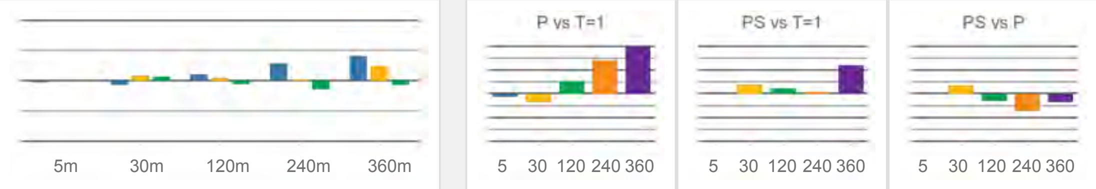

Locus		5m	30m	120m	240m	360m
**PGN_2085**	P vs T=1	-0.897	-1.002	-0.845	-0.667	0.012
	PS vs T=1	-0.727	-0.857	-1.048	-0.523	-0.518
	PS vs P	0.154	0.113	-0.220	0.141	-0.469

putative Fe-S oxidoreductases

hypothetical proteins-Conserved

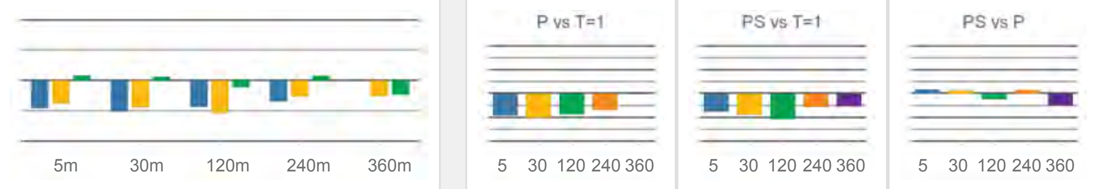

Locus		5m	30m	120m	240m	360m
**PGN_2086**	P vs T=1	-0.465	-0.128	0.491	0.577	0.791
	PS vs T=1	-0.204	-0.499	-0.534	-0.050	0.260
	PS vs P	0.194	-0.377	-0.937	-0.537	-0.456

probable acetyltransferase

unknown function

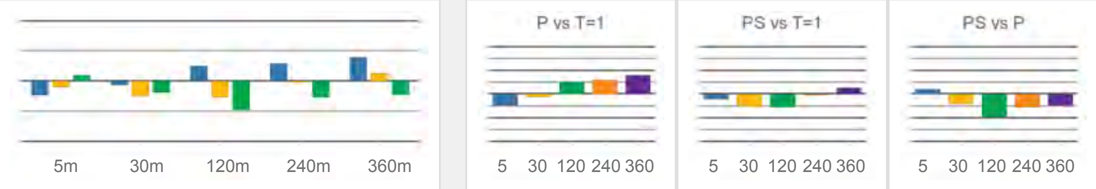

Locus		5m	30m	120m	240m	360m
**PGN_2087**	P vs T=1	-0.577	-0.346	0.119	0.337	0.681
	PS vs T=1	-0.161	-0.239	-0.334	-0.130	0.156
	PS vs P	0.362	0.078	-0.432	-0.420	-0.474

glycosyltransferase

unknown function

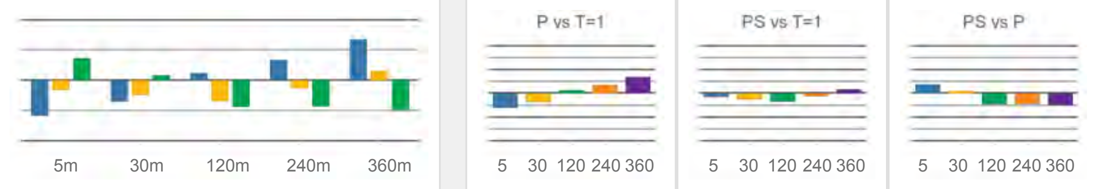

log$_2$ (Fold Change)

Legend: P vs T=1 | PS vs T=1 | PS vs P

Locus		5m	30m	120m	240m	360m
**PGN_2088**	P vs T=1	0.059	0.240	0.389	0.479	0.246
	PS vs T=1	-0.201	0.237	0.528	0.551	0.768
	PS vs P	-0.274	-0.008	0.139	0.079	0.505

conserved hypothetical protein

hypothetical proteins-Conserved

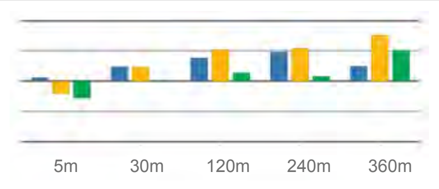

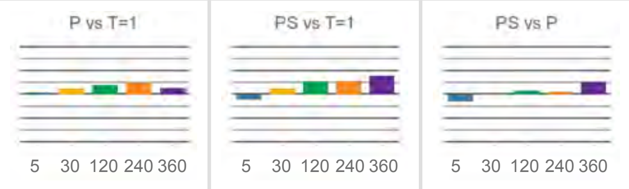

Locus		5m	30m	120m	240m	360m
**PGN_2089**	P vs T=1	-0.237	0.207	0.723	0.850	0.465
	PS vs T=1	-0.186	0.197	0.202	0.446	0.271
	PS vs P	-0.009	-0.020	-0.472	-0.331	-0.194

conserved hypothetical protein

hypothetical proteins-Conserved

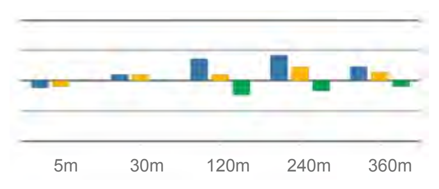

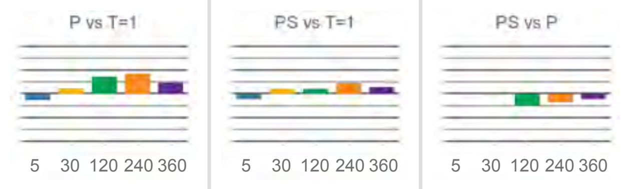

Locus		5m	30m	120m	240m	360m
**PGN_2090**	P vs T=1	0.112	0.571	1.417	1.850	2.495
	PS vs T=1	0.203	0.765	1.387	2.118	2.514
	PS vs P	0.001	0.140	-0.018	0.308	0.077

conserved hypothetical protein

hypothetical proteins-Conserved

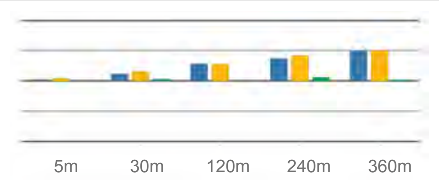

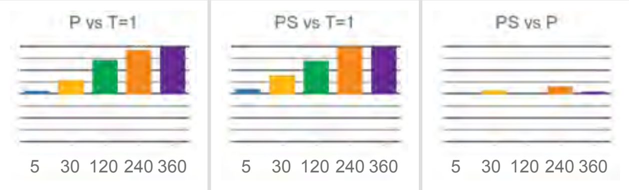

Locus		5m	30m	120m	240m	360m
**PGN_2091**	P vs T=1	0.485	1.055	1.772	1.884	1.563
	PS vs T=1	0.023	0.046	0.409	0.403	0.586
	PS vs P	-0.479	-0.937	-1.212	-1.270	-0.877

conserved hypothetical protein

hypothetical proteins-Conserved

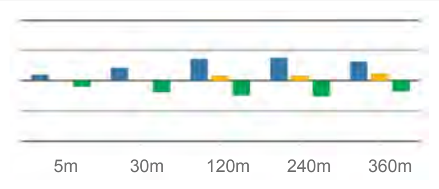

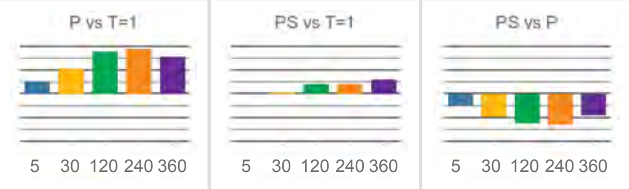

Supplement: Table S3 — The third of three sequential files making up the master list. [file Table3.PDF]
