# Supplementary material for: Insights into Dynamic Polymicrobial Synergy Revealed by Time-Coursed RNA-Seq
Source: Front Microbiol. 2017 Feb 28;8:261. doi: 10.3389/fmicb.2017.00261 (PMC5329018; doi:10.3389/fmicb.2017.00261)
Supplement: Table S4 — A table of differentially expressed genes annotated for the initiation, elongation or termination steps of protein translation. [file Table4.pdf]

		Pg versus time 1 minute Log2 (fold change)				
Gene	Gene Name/ Function	5	30	120	240	360
PGN_0313	peptide chain release factor 3	-0.25	-0.43	-0.76	-0.55	-0.15
PGN_0350	probable translation initation factor SUI1	1.14	1.35	1.64	1.48	1.21
PGN_0355	translation initiation factor IF-2	0.22	0.60	0.47	-0.02	-0.39
PGN_0616	probable elongation factor P	0.65	0.88	0.90	0.82	0.30
PGN_0963	translation initiation factor IF-3	-0.37	-0.57	-0.76	-1.20	-1.99
PGN_1014	elongation factor G	0.30	0.80	1.38	1.40	0.81
PGN_1244	putative peptide chain release factor RF-2	0.64	0.66	0.52	0.22	-0.14
PGN_1405	elongation factor P	0.70	0.94	0.96	0.75	0.29
PGN_1578	translation elongation factor Tu	1.47	1.87	2.11	1.83	1.09
PGN_1587	putative translation elongation factor Ts	0.27	0.76	1.27	1.13	0.60
PGN_1846	translation initiation factor IF-1	0.87	0.91	0.62	-0.13	-1.19
PGN_1870	translation elongation factor G	0.42	0.73	0.74	0.39	0.10
PGN_2022	peptide chain release factor 1	-0.11	0.34	0.73	0.66	0.40

		PgSg versus time 1 minute Log2 (fold change)				
Gene	Gene Name/ Function	5	30	120	240	360
PGN_0313	peptide chain release factor 3	-0.52	-0.82	-1.14	-1.05	-0.90
PGN_0350	probable translation initation factor SUI1	0.85	1.13	1.46	1.56	1.45
PGN_0355	translation initiation factor IF-2	-0.24	-0.42	-1.04	-1.14	-1.05
PGN_0616	probable elongation factor P	0.50	0.56	0.74	0.91	0.91
PGN_0963	translation initiation factor IF-3	-0.83	-0.69	-0.86	-1.15	-1.42
PGN_1014	elongation factor G	0.84	0.80	0.82	0.98	0.94

PGN_1244	putative peptide chain release factor RF-2	-0.07	-0.10	-0.27	-0.41	-0.38
PGN_1405	elongation factor P	0.52	0.56	0.74	1.08	1.08
PGN_1578	translation elongation factor Tu	0.96	1.24	1.05	0.71	0.67
PGN_1587	putative translation elongation factor Ts	-0.07	0.28	0.43	0.31	0.20
PGN_1846	translation initiation factor IF-1	1.00	1.39	1.22	0.68	0.34
PGN_1870	translation elongation factor G	0.05	-0.23	-0.69	-0.64	-0.29
PGN_2022	peptide chain release factor 1	0.10	0.19	-0.46	-0.61	-0.66

		PgSg versus Pg Log2 (fold change)				
Gene	Gene Name/ Function	5	30	120	240	360
PGN_0313	peptide chain release factor 3	-0.22	0.43	1.06	0.48	-0.82
PGN_0350	probable translation initation factor SUI1	-0.28	-0.19	-0.12	0.09	0.21
PGN_0355	translation initiation factor IF-2	-0.45	-0.98	-1.48	-1.10	-0.66
PGN_0616	probable elongation factor P	-0.15	-0.29	-0.14	0.11	0.58
PGN_0963	translation initiation factor IF-3	-0.57	-0.14	0.41	0.82	0.89
PGN_1014	elongation factor G	0.49	-0.01	-0.52	-0.40	0.13
PGN_1244	putative peptide chain release factor RF-2	-0.67	-0.71	-0.76	-0.61	-0.25
PGN_1405	elongation factor P	-0.15	-0.35	-0.20	0.33	0.76
PGN_1578	translation elongation factor Tu	-0.49	-0.58	-0.99	-1.08	-0.42
PGN_1587	putative translation elongation factor Ts	-0.34	-0.45	-0.78	-0.78	-0.40
PGN_1846	translation initiation factor IF-1	0.18	0.54	0.65	0.72	1.29
PGN_1870	translation elongation factor G	-0.32	-0.85	-1.32	-0.96	-0.37
PGN_2022	peptide chain release factor 1	0.19	-0.13	-1.12	-1.21	-1.03

Table S4 (above) Differential expression of genes associated with translation initiation, elongation, and termination. Results are expressed as log_2 fold change for the comparisons at the times indicated in minutes. Higher mRNA levels are represented by red font, lower mRNA levels are represented by green font, statistical thresholds are given in the main text.
