# Supplementary material for: Insights into Dynamic Polymicrobial Synergy Revealed by Time-Coursed RNA-Seq
Source: Front Microbiol. 2017 Feb 28;8:261. doi: 10.3389/fmicb.2017.00261 (PMC5329018; doi:10.3389/fmicb.2017.00261)
Supplement: Figure S1 — Representative qRT-PCR data for fimA, mfa1, clpB, and clpP. [file Image1.PDF]

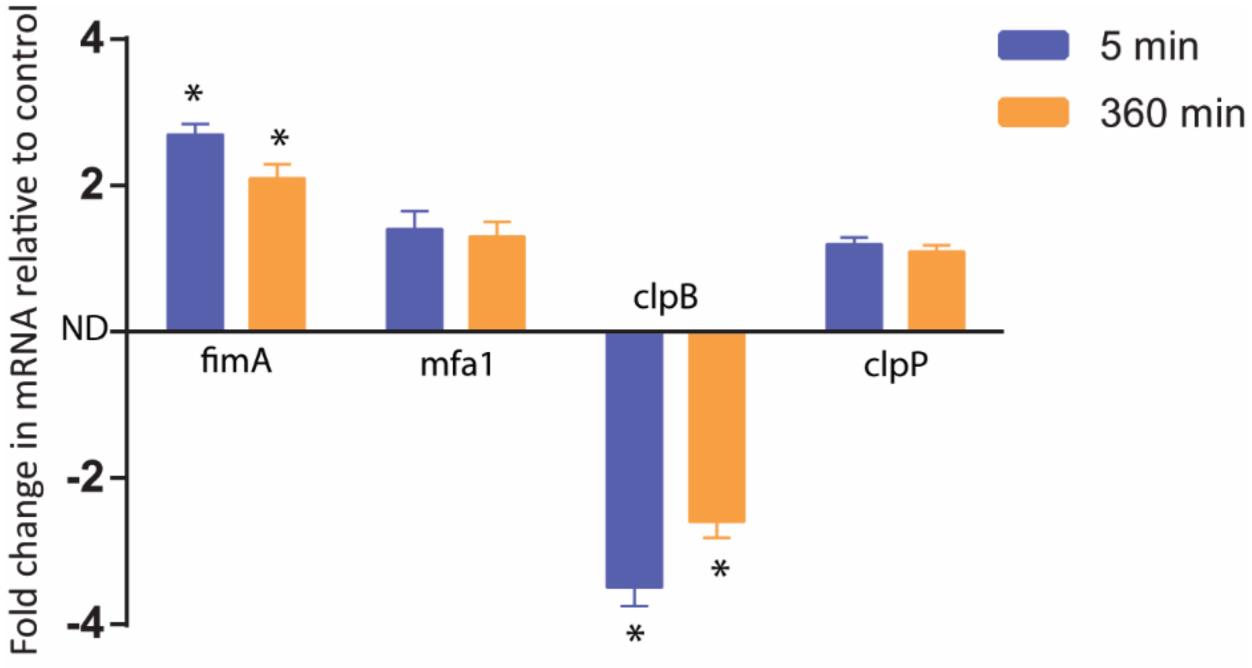

Figure S1. qRT-PCR confirmation of selected differentially expressed genes (*fimA, clpB*) along with unregulated controls (*mfa1*, *clpP*). mRNA levels in PgSg communities were normalized to 16S and expressed relative to the Pg alone condition at the same time point. *: differential expression of *fimA* and *clpB* was significant at $P < 0.005$, n=3 using ANOVA with Tukey post test. One representative experiment of three is shown. See the main text for methodological details.
