# Supplementary material for: Insights into Dynamic Polymicrobial Synergy Revealed by Time-Coursed RNA-Seq
Source: Front Microbiol. 2017 Feb 28;8:261. doi: 10.3389/fmicb.2017.00261 (PMC5329018; doi:10.3389/fmicb.2017.00261)
Supplement: Figure S2 — RNA-Seq reads across the fim gene loci. [file Image2.PDF]

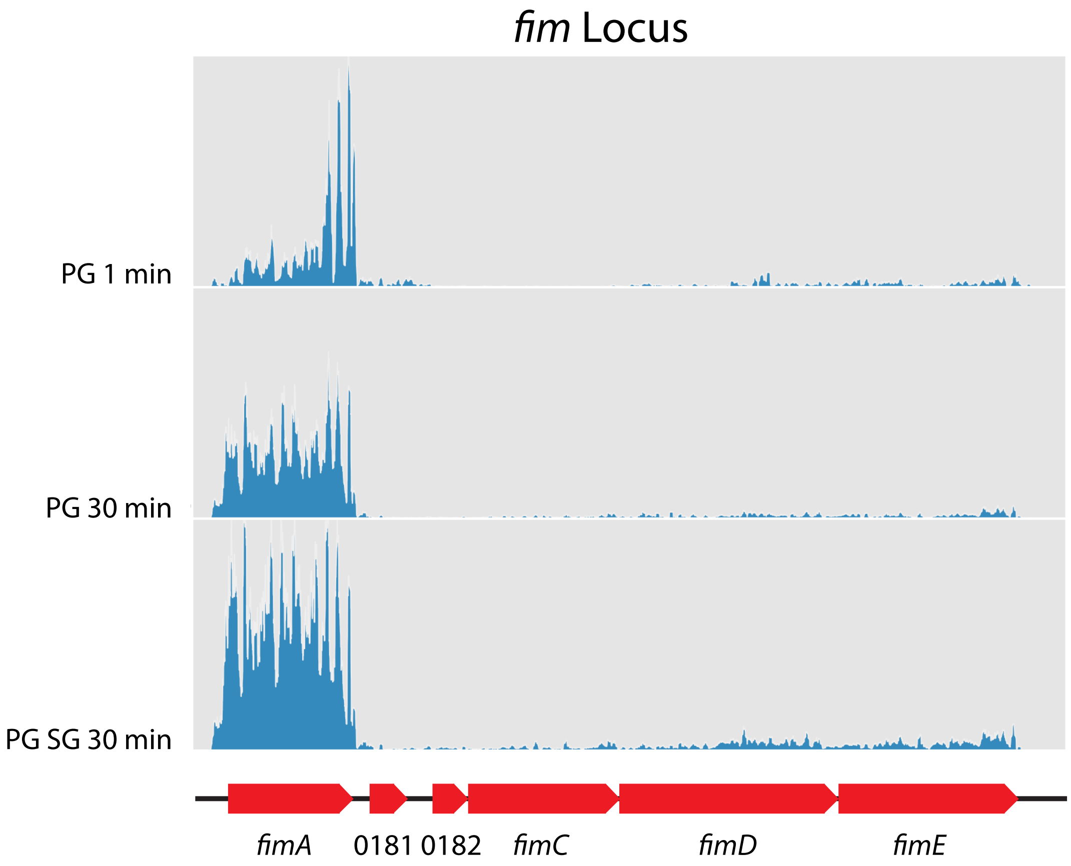

Figure S2 | Pileup of reads for *fim* Locus. The figure shows non-normalized reads for a representative replicate of the Pg time 1 minute (reference state) and the Pg and PgSg samples at 30 minutes. The transcriptional locus, covering genes PGN_0180 – 0185, is shown with ORFs in red and direction indicated by the arrows. The number of aligned reads for each nucleotide of the sequence is indicated by the blue curve. FimA is the structural component of the fimbriae and its transcript would be expected to be present at significantly higher levels than the minor components. The figure is provided to illustrate the general transcriptional structure of the *fim* locus. As the reads are not normalized no direct comparison of the read levels can be made between samples.
